# Loss of *Serpina1* in Mice Leads to Altered Gene Expression in Inflammatory and Metabolic Pathways

**DOI:** 10.3390/ijms231810425

**Published:** 2022-09-09

**Authors:** Sri Harsha Meghadri, Beatriz Martinez-Delgado, Lena Ostermann, Gema Gomez-Mariano, Sara Perez-Luz, Srinu Tumpara, Sabine Wrenger, David S. DeLuca, Ulrich A. Maus, Tobias Welte, Sabina Janciauskiene

**Affiliations:** 1 Department of Respiratory Medicine, Platform Bioinformatics, Member of the German Center for Lung Research (DZL), Biomedical Research in Endstage and Obstructive Lung Disease (BREATH), Hannover Medical School, 30625 Hannover, Germany; 2 Functional Genomics Unit, Rare Diseases Research Institute, Carlos III Health Institute, 28220 Majadahonda, Spain; 3 Division of Experimental Pneumology, Member of the German Center for Lung Research (DZL), Biomedical Research in Endstage and Obstructive Lung Disease (BREATH), Hannover Medical School, 30625 Hannover, Germany; 4 Department of Respiratory Medicine, Molecular Pneumology, Member of German Center for Lung Research (DZL), Biomedical Research School in Endstage and Obstructive Lung Disease (BREATH), Hannover Medical School, 30625 Hannover, Germany

**Keywords:** liver, SERPINA, mice, gene knockout, RNA sequencing, transcriptomics, metabolism, single-cell, alpha1-antitrypsin, protein misfolding, data analysis

## Abstract

The *SERPINA1* gene encodes alpha1-antitrypsin (AAT), an acute phase glycoprotein and serine protease inhibitor that is mainly (80–90%) produced in the liver. Point mutations in the *SERPINA1* gene can lead to the misfolding, intracellular accumulation, and deficiency of circulating AAT protein, increasing the risk of developing chronic liver diseases or chronic obstructive pulmonary disease. Currently, siRNA technology can knock down the *SERPINA1* gene and limit defective AAT production. How this latter affects other liver genes is unknown. Livers were taken from age- and sex-matched C57BL/6 wild-type (WT) and *Serpina1* knockout mice (KO) aged from 8 to 14 weeks, all lacking the five serpin A1a-e paralogues. Total RNA was isolated and RNA sequencing, and transcriptome analysis was performed. The knockout of the *Serpina1* gene in mice changed inflammatory, lipid metabolism, and cholesterol metabolism-related gene expression in the liver. Independent single-cell sequencing data of WT mice verified the involvement of *Serpina1* in cholesterol metabolism. Our results from mice livers suggested that designing therapeutic strategies for the knockout of the *SERPINA1* gene in humans must account for potential perturbations of key metabolic pathways and consequent mitigation of side effects.

## 1. Introduction

Defective human *SERPINA1* genes are associated with liver, lung, and skin diseases due to mutations leading to low levels or polymerization of the alpha1-antitrypsin (AAT) protein and, in very rare cases, hemorrhagic disease due to the loss of the function of AAT (Pittsburgh mutation) [1]. Most of the available studies related to AAT and liver diseases focus on inherited misfolding of AAT protein leading to its polymerization, intracellular accumulation, and liver cell damage to different degrees. Therefore, pharmaceutical companies explore interventions using siRNA technology to knock down mutant *SERPINA1* gene and to stop the production of misfolded AAT protein [2]. It is not currently clear whether such knockouts or knockdowns affect liver function at the transcriptional levels. To illuminate this, we compared liver transcriptome profiles of strain-, age- and sex-matched wild-type (WT) and *Sepina1* knockout (KO) mice. We investigated the major changes in the expression of specific liver genes and pathways in KO mice compared to WT mice. Furthermore, we assessed whether these differences could help us predict how liver cells without the *Serpina1* gene react to inflammatory stimuli (infection, trauma, etc.) and what can be the expected putative systemic consequences.

The human *SERPINA1* (serpin family member 1) is a 12.2 kb inducible gene located on chromosome 14q32.1 [3]. The *SERPINA1* gene is mainly expressed in cells originating from two different embryonal layers: hepatocytes from the endoderm and macrophages- from the mesoderm [4]. It encodes AAT, one of the most abundant circulating acute-phase proteins having anti-protease and immunomodulatory activities [5]. During inflammation, infections, and late pregnancy, serum levels of AAT can increase 4-6-fold above baseline levels, which are about 1–2 g/L in humans [6,7,8].

Murine AAT is encoded by a cluster of up to six individual *Serpina1*-related genes located on chromosome 12, depending on the mouse strain. In C57BL/6 mice, five *Serpina1* paralogues, *Serpina1a* through *Serpina1e*, are present and expressed [9]. Recently, AAT-KO mice lacking all five *Serpina1* paralogues were described to spontaneously develop emphysema with age and to exert an impaired lung antibacterial immunity but retain normal body weight, behavior, lifespan, and gender distribution [10,11].

Besides its anti-protease activity, AAT regulates cell proliferation and wound healing. Some studies demonstrated that AAT inhibits senescence-associated gene expression and aging-related pathways in both Drosophila and human cells [12]. A characteristic of senescence is an elevated expression of inflammatory cytokines and chemokines, growth factors, and proteases, which is termed senescence-associated secretory phenotype [12,13]. Different studies reported that treatment with AAT significantly decreased the expression of IL-6 and IL-8, two major senescence-associated factors [12,14,15]. In other experimental models, AAT was similarly found to inhibit the expression of IL-8, IL-6, TNF-α, and IL-1β and to suppress the activation of nuclear factor (NF)-κB, a factor regulating the expression of inflammatory cytokines [14,16].

Given that these prior studies have established anti-inflammatory and immunomodulatory roles of AAT, the transcriptome study of a *Serpina1* knockout model presented here aimed at cataloging the transcriptional changes, which occurred in the absence of Serpina1, and interpreting these changes at the level of genes and pathways.

## 2. Results

### 2.1. Transcriptome Quality Control and Differential Gene Expression

**Figure 1:**
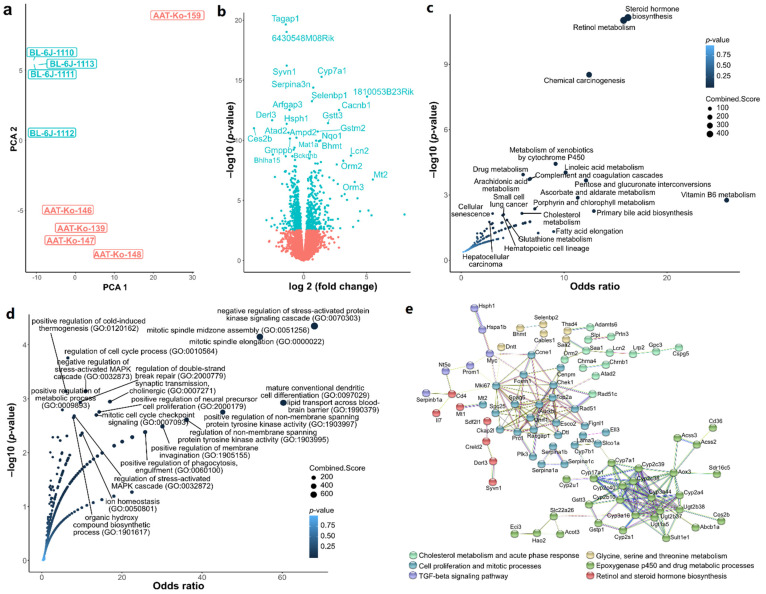
Differential gene expression (DEGs) and pathway analysis of *Serpina1* KO vs. WT. (**a**) Principal component analysis of samples selected for RNA-Seq data analysis and DEG exploration with WT labeled blue and KO labeled red. (**b**) Volcano plot of DEGs where x-axis represents the log_2_ fold _change and y-axis represents the significance level wherein *p*-values have been −log_10_ transformed. Significantly expressed genes are labeled blue (*p*-value ≤ 0.05), and non-significant genes are labeled in red. (**c**) A plot showing the KEGG pathways that were identified from DEGs between KO vs. WT mice using EnrichR. X-axis: odds ratio, Y-axis negative Log_10_ *p*-value. (**d**) Gene ontology analysis using EnrichR: x-axis: odds ratio, y-axis negative Log_10_ *p*-value. (**e**) Protein-protein interaction networks as generated with String-Db: Differentially expressed genes were subjected to k-means clustering (n = 6) with high confidence interaction settings.

The initial 10 samples (five *Serpina1* KO and five WT) were clustered using PCA to identify sample similarities and differences (Figure S1). The clustering showed an outlier (WT BL-6J-1109), which was removed to reduce a potentially detrimental source of variance amongst the differentially expressed genes (DEGs) (Figure 1a). Using DESeq2, 801 DEGs were found between the *Serpina1* KO and WT mice livers. Among the latter, 438 were upregulated and 363 downregulated in the KO relative to WT mice (Figure 1b). Top downregulated genes in KO livers apart from *Serpina1* included (by Log_2_Fc values) the following genes: *Ces2b* (carboxylic ester hydrolase 2B), *Ugt2b38* (UDP glucuronosyltransferase two family, polypeptide B38), *Mup2* (major urinary protein 2), *Bglap3* (bone gamma-carboxyglutamate protein 3), and *Derl3* (derlin-3). Among the top upregulated genes were *Sult1e1* (sulfotransferase family 1E, member 1), *Slc22a26* (Solute carrier family 22, member 26), *Rgs16* (Regulator of G-protein signaling 16), *Slc22a27* (solute carrier family 22, member 27), *Mt1* (Metallothionein-1), and *Orm3* (Alpha-1-acid glycoprotein 3). The full list of the DEGs is presented in Supplementary Table S1.

### 2.2. Hypothesis-Free Pathway Screening

The DEGs were further analyzed using EnrichR [17] to identify the gene terms (gene ontology, GO) [18] and pathways (KEGG) [19]. We observed changes associated with steroid hormone biosynthesis, retinol metabolism, and several other metabolic pathways associated with the KO phenotype in KEGG enrichment (Figure 1b). The GO analysis revealed that cholesterol metabolism, cytokine responses, and stress responses were associated with KO phenotype (Figure 1c).

To mine potential interactions based on protein-protein networks, we queried stringDB [20] using the DEGs. K-means clustering revealed six clusters (Figure 1d) linked with loss of *Serpina1*. The main interactions within the clusters involved the epoxygenase p450 (cytochrome p450 related genes) (Figure 1b,d), cell cycle-associated genes (Figure 1c,d), cholesterol metabolism, and acute-phase response pathways (Figure 1b,d).

### 2.3. Hypothesis-Driven Pathway Analysis

In parallel to the hypothesis-free pathway analysis reported above, we conducted a targeted analysis of specific gene sets of interest. The former approach, although objective, suffered from the burden of multiple hypothesis corrections from hundreds of irrelevant gene sets. Based on prior literature and knowledge of *SERPINA1*, there were specific bioprocesses that were the object of our inquiry here. Human *SERPINA1* plays an essential role in the modulation of acute-phase responses, inflammatory processes, and the lower respiratory tract protection from proteolytic damage. To examine these processes, we defined gene sets for these pathways of interest and calculated the overrepresentation of differentially expressed genes.

The *p*-values for the over-representation were as follows: (a) acute-phase response 0.0247, (b) complement pathway 0.1881, (c) cholesterol metabolism 0.0028, (d) bile acid metabolism 3.194e-07, and (e) steroid hormone biosynthesis 0.1421 (data not represented in plots).

**Figure 2:**
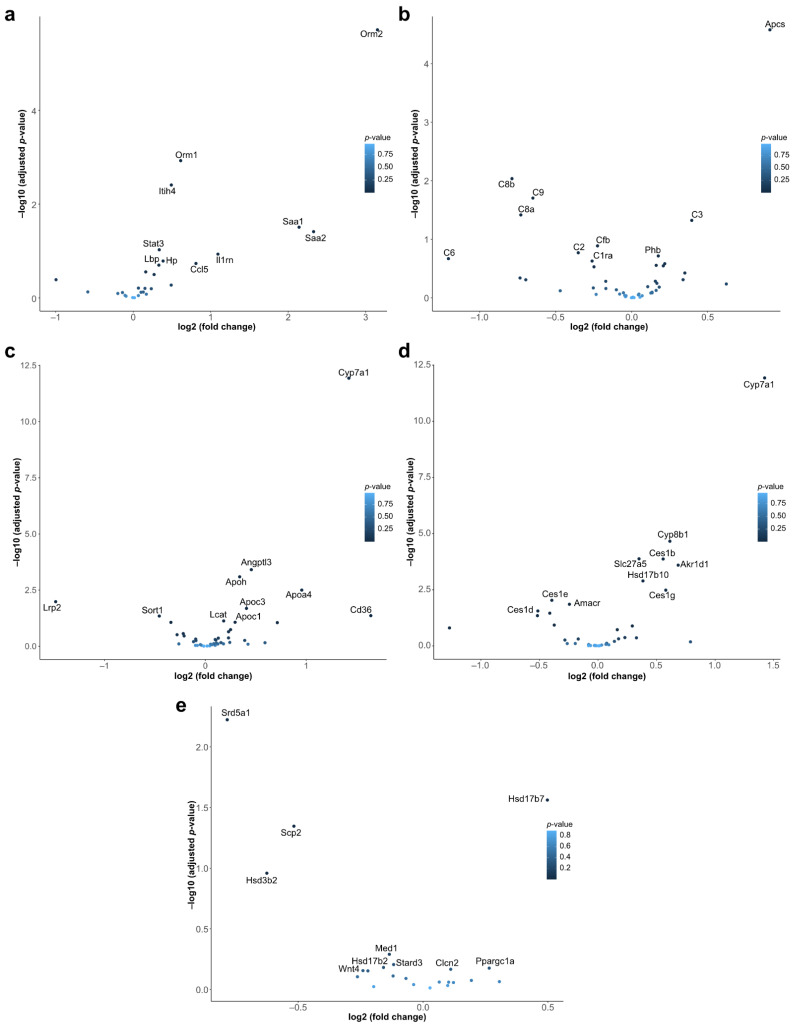
Differential expression of gene sets in *Serpina1* KO vs. WT mice. Volcano plots depict the fold-change (x-axis) and significance (y-axis) for genes associated with: (**a**) acute phase response (n = 38); (**b**) complement pathway (n = 187); (**c**) cholesterol metabolism (n = 47); (**d**) bile acid metabolic processes (n = 44); and (**e**) steroid hormone biosynthesis (n = 30). The ten most highly significant DEGs in each pathway are annotated.

We observed that genes associated with an acute-phase response, such as *Orm2* (Alpha-1-acid glycoprotein 2), *Saa1* (Serum amyloid A1), and *Saa2* (Serum amyloid A2), were upregulated (Figure 2a). This was likely due to pro-inflammatory mice phenotype because of the loss of Serpina1 gene. Since the pro-inflammatory state was generally reflected by upregulated interleukins, we investigated the expression of interleukins. The transcriptional levels of *Il6*, *Il7*, *Il15,* and *Il18* were only slightly upregulated (Figure S2), with no significant changes in transcription factors controlling the acute phase proteins. We also analyzed the complement pathway gene set (Figure 2b). Although, in general, this pathway remained not altered significantly in *Serpina1* KO mice, specific genes were affected. For example, *Apcs* and *C3* were slightly upregulated, whereas *C4a* and *C8a* were downregulated (Figure 2b). Hence, the parallel upregulation of pro-inflammatory interleukins and acute-phase proteins, and alterations in some of the complement pathway genes suggested a pro-inflammatory phenotype in KO mice.

As illustrated in Figure 2c, the expression of several cytochrome P450 proteins was disturbed in the KO vs. WT mice livers. Specifically, KO mice livers upregulated the *Cyp7a1* (Cytochrome P450 Family 7 Subfamily A Member 1) gene that has an important role in cholesterol and bile acid metabolism and is associated with hypercholesterolemia. Several genes from bile acid metabolic processes were concomitantly upregulated in the livers of the KO vs. WT mice (Figure 2d). Among these genes were *Cyp8b1* (sterol 12-alpha-hydrolase), *Ces1g* (carboxylesterase 1G, the major liver enzyme for the hydrolysis or transesterification of various xenobiotics), and *Akr1d1* (aldo-keto reductase family 1 member D1, crucial for bile acid biosynthesis and important for steroid metabolism). Likewise, several genes of steroid hormone biosynthesis, like, *Hsd17b7* (hydroxysteroid (17-beta) dehydrogenase 7), *Scp1* (Synaptonemal complex protein 1), and *Hsd3b2* (Hydroxy-Delta-5-Steroid Dehydrogenase, 3 Beta-Steroid Delta Isomerase 2) were significantly higher expressed in the livers of the *Serpina1* KO vs. WT mice (Figure 2e). Genes related to linoleic acid and retinol metabolism (Figure S3) and vitamin D signaling were also altered in *Serpina1* KO mice. Regarding glycolysis, most genes of canonical and anaerobic glycolysis were largely unaffected, with the exception of *Pfkl* and *Pfkp* gene members of the phosphofructokinase A family (Figures S4 and S5). It is noteworthy that both the hypothesis-driven and EnrichR results showed a predisposition towards altered metabolic processes in the KO mice (Figure 1b).

### 2.4. Single-Cell Sequencing Data Analysis

**Figure 3:**
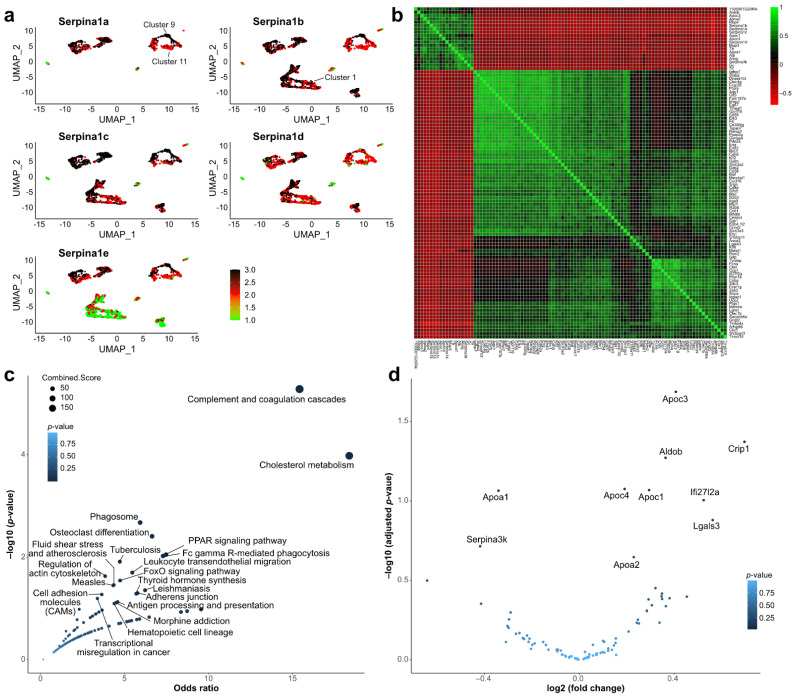
scSEQ data analysis of *Serpina1* gene expression. (**a**): Global expression of *Serpina1* isoforms: *Serpina1* was broadly expressed in the wild-type mice, with two among thirteen clusters that showed a lower expression. (**b**): Correlation matrix of *Serpina1*: Top 100 genes with *Serpina1* highly correlated are shown in this plot with highly correlated genes in green and anti-correlated genes in red. (**c**): Pathway enrichment of correlated genes. (**d**): Expression changes in the KO dataset of the correlated genes (n = 100). The ten most highly significant DEGs are annotated.

To complement and potentially validate our results, we performed a co-expression analysis of data from *tabula muris*, a compendium of single-cell transcriptome data from *Mus musculus* [21]. Among the three liver samples, we observed a global expression of four isoforms of *Serpina1* (Figure 3a). We identified the top 100 correlated genes from this single-cell dataset and clustered them (Figure 3b). As expected, *Serpina1a* correlated with each and with other known *Serpina1*-related genes like *Alb* (Figure 3b). To summarize this set of *Serpina1*-associated genes, we performed an EnrichR analysis. We observed that complement and cholesterol pathways were enriched with *Serpina1*-associated genes (Figure 3c).

We further examined whether these *Serpina1*-associated genes were also differentially expressed in the KO experiment (Figure 3d). For example, *Apoc3* was positively correlated in the WT mice and was downregulated in the KO mice. However, it must be noted that the fold-change values of most of these genes were relatively low. Overall, the analysis of the *Serpina1* gene in single cells, along with pathways of the correlated genes, confirmed its roles in cholesterol metabolism and provided further indication of a role in the complement system.

## 3. Discussion

Our study examined the loss of the *Serpina1* gene and its role in cellular pathways in the liver, the primary organ that produces the bulk of AAT. We found 801 DEGs, of which 55% were upregulated and 45% were downregulated in the KO relative to WT mice livers. By focusing on the transcript level, we characterized the consequences of loss of *Serpina1* and which specific cellular processes were disrupted. Our data revealed changes in a broad range of cellular processes, including inflammation and acute phase response, metabolic processes, cellular differentiation, and molecular transport across membranes, to name a few.

Inflammatory processes are crucial to the survival of an organism through their roles in harmful stimuli, like pathogens, damaged cell response, and wound healing. Loss of *Serpina1* in mice altered key inflammatory transcriptional signatures. Our results indicated a pro-inflammatory phenotype, likely via the alteration of acute-phase response and complement pathway mechanisms, among others. We showed that the expression of genes that modulate these responses, such as *Il6*, *Il7*, *Apcs*, *C3*, *C4a,* and *C8a*, were upregulated in KO mice. In addition to these transcripts, we showed a significant upregulation of *Saa1* and *Saa2*, which are key transcripts of the acute-phase response and are implicated in the interaction between various cell types such as macrophages, hepatocytes, and myeloid-derived suppressor cells [22,23]. This complex interaction likely facilitates the binding of SAA1/2 to receptors, such as SR-B1, that participate in the cholesterol efflux [24,25]. Similarly, we show an upregulation of *Orm2* coding for a key protein that has varied functions like transporter or modulator of the activity of the immune system depending on its glycosylation. Through the interaction with the farnesoid X receptor, orosomucoids also play a role in bile acid homeostasis [26].

A crucial physiological role of the liver is the metabolic control of biomolecules such as cholesterol and bile acid and its function as a biosynthesis hub for steroids and complement factors. Our analysis of KEGG enrichment showed that in *Serpina1* KO mice, these pathways were enriched, indicating that alteration of *Serpina1* expression can lead to changes in homeostatic conditions required for healthy liver function. Transcripts, such as *St1e1*, *Cyp7a*, *Cyp8b1*, *Slc2a1*, and *Derl3*, were shown in our data to be differentially expressed. *St1e1*, for example, coded for estrogen sulfotransferase, a key enzyme in estrogen homeostasis [27]. Gene ontology annotations related this gene to sulfotransferase and flavanol 3-sulfotransferase activities. Several of the cytochrome p450 enzymes played roles in key enzymatic and catalytic reactions, e.g., *Cyp7a1* metabolized cholesterol to 7-alpha-hydroxycholesterol, the first step in bile acid synthesis. Dysregulation of bile acid homeostasis caused a wide spectrum of diseases usually associated with hepatic injury [28]. The transport of biomolecules is also a crucial step in which the liver participates. The DEG *Derl3* is involved in the negative regulation of retrograde transport and has a regulatory role in controlling the regulation of *Slc2a1* [29]. Similarly, the retinol metabolism and its associated genes, such as retinol dehydrogenases *Rdh16*, *Rdh11*, and short-chain dehydrogenase/reductase family 16C member 5 (*Sdr16c5*), are differentially expressed in the KO mice (Figure S2). Since retinol dehydrogenases control key enzymatic steps involved in retinol metabolism and other steroid metabolic processes [30], it seems likely that perturbation of *Serpina1* expression in the liver alters immune regulatory and metabolic pathways which are involved in the homeostasis of the organism.

The analysis of *Serpina1*-correlated genes in the tabula muris scSEQ dataset provided an independent method to identify genes that could be co-regulated and functionally linked to *Serpina1*. KEGG enrichment analysis of these genes supports the hypothesis that *Serpina1* expression affects immune responses and metabolism-associated pathways and primarily provides independent validation of the implicated role of *Serpina1* in cholesterol metabolism. The single-cell dataset showed that the various *Serpina* genes in mice were generally co-expressed. Cluster 9 (Figure 3a) was one of the clusters expressing the highest levels of *Serpina* genes and was also associated with *Mup3*, *Cyp2f2*, and *Scd1*. Conversely, clusters tending for lower expression of *Serpina* genes, such as 1 and 11, showed higher levels of Cytochrome P450 genes. This may imply that liver cells specializing in xenobiotic metabolism express less *Serpina*, which is consistent with the pathway enrichment findings from the RNA-seq data from the knockout mice. Ultimately, further scRNA-seq studies specifically involving the *Serpina* knock-out mice would go much further to elucidate the role of these genes in the liver.

In conclusion, circulating AAT is not a single-function protein as an anti-protease but expresses broader anti-inflammatory effects. *SERPINA1* regulation affects the modulation of various inflammation-related substances like chemokines, oxygen species, complement factors, lipoproteins, and others. A recent small cohort phase 2 trial using fazrisiran, an RNA interference against *SERPINA1* “Z” mutation that caused degradation of AAT messenger RNA and abolished protein synthesis in hepatocytes, improved liver enzymes, and reduced fibrosis [31]. Our data suggested that knockdown therapies might be prone to side effects. Human studies should be carefully monitored to observe any such adverse long-term effects that could be due to alterations of key genes and pathways discussed above.

At first glance, *Serpina1a* to *e* knockout represents a similar situation as in AAT-Null patients. However, each of the mouse *Serpina1* paralogues displayed specific functions. Amino acid polymorphisms in the reactive center loop determined AAT’s substrate specificity: *Serpina1a* and *Serpina1b* products AAT 1 and AAT 2 displayed anti-elastase activity, while *Serpina1c* and *Serpina1d* products AAT 3 and AAT 4 had anti-chymotryptic activity. The product of *Serpina1e*, AAT 5, lacks inhibitory activity against trypsin, chymotrypsin, and pancreatic as well as against neutrophilic elastase [32], suggesting that the results from the mouse AAT KO model may not reflect the situation of AAT-Null patients. Ultimately, the differences between the *Serpina* gene system in mice and humans create a limitation of these model-based studies. Further CRISPR-knock out or gene silencing of *SERPINA1* in human-derived organoid models would be warranted.

## 4. Materials and Methods

### 4.1. Animal Model

WT mice (C57BL/6) were purchased from Janvier Labs (Janvier Labs, Le Genest-Saint-Isle, France). *Serpina1*-KO mice lacking all 5 serpin A1 a-e paralogues expressed in mice were used as previously described by [10]. As described earlier, *Serpina1*-KO mice have normal behavior, lifespan, and gender distribution. Only at the advanced age of more than 35 weeks do they spontaneously develop lung emphysema [11]. Livers from 10 male age-matched healthy mice (KO = 5 and WT = 5) aged from 10 to 12 weeks were isolated and frozen in nitrogen.

### 4.2. Total RNA Isolation from the Mice Livers

The isolation of the total RNA was performed using RNeasy Minikit (Qiagen, Hilden, Germany) as described in [33]. Livers were shortly collected in an RLT buffer supplemented with 1% mercaptoethanol. After an initial homogenization step using T10 basic ultra-turrax (Ika, Staufen im Breisgau, Germany), RNA isolation was continued with RNeasy Minikit according to the instructions of the manufacturer.

### 4.3. Transcriptome Analysis (RNA-seq)

Agilent 2100 Bioanalyzer based on an Agilent RNA 6000 Nano Kit was used to assess the quality of total RNA. RNASeq libraries were prepared by using 200 ng RNA of each sample. TruSeq Stranded mRNA (Illumina, San Diego, CA, USA) was used for library preparation according to the manufacturer’s protocol. After reverse transcription and adapter ligation, the cDNA products were purified with Ampure XP beads (Beckman Coulter, Brea, CA, USA), and enriched with 15 cycles of PCR to create the cDNA library. Sequencing was performed on a NovaSeq 6000 sequencer using 200 cycles in paired-end mode. RNA-Seq data was quality control analyzed with fastQC v0.11.8 and low-quality 30 ends were removed using Trimmomatic v0.38.

### 4.4. Data Preprocessing

The fastq files generated through high-throughput sequencing (HTS) were subjected to fastqc to assess the overall read quality. Lower quality reads were removed using trimmomatic. The processed files were aligned with STAR with hg19 as the reference, the output bam files were then subjected to HT-Seq to count the number of transcripts per gene.

### 4.5. Gene Expression Analysis

The principal component analysis (PCA) was performed to characterize the variation among the samples and to assess putative outliers. Differential expression analysis was performed using DEseq2 [34]. Expression values were normalized with rlog using only genes with average values greater than 3.4.

### 4.6. Gene Ontology and Pathway Analysis

For performing GO term analysis, we used genes meeting the cut-off criteria (log_2_FC +/− 1.2) post-normalization and log_2_FC calculations in the complete dataset. In total 221 genes were analyzed using EnrichR to obtain the GO terms (GO biological process 2021) and KEGG pathways (KEGG 2019 mouse). For mining the known interactions among differentially expressed genes, we used the STRING database with a full STRING network based on evidence through all available resources with a high confidence setting. The network was clustered into six clusters using k-means clustering algorithm.

### 4.7. Single-Cell Sequencing (scSEQ) Data Analysis

We utilized the publicly available tabula muris dataset for liver (10 × sequencing) [21]. The selected data consisted of three liver samples subjected to single-cell sequencing using the 10× platform. The data we processed with a Seurat and used a SCTransform to normalize variances in the scSEQ data [35,36]. The top 100 Serpina1-correlated genes were determined using Pearson’s r. This set of genes was then used to obtain KEGG pathways and volcano plots for differential expression.

## Data Availability

Merged counts matrix for the study is available at 10.5281/zenodo.6974384 under restricted access.

## Supplementary Materials

The following supporting information can be downloaded at: https://www.mdpi.com/article/10.3390/ijms231810425/s1.
