# Supplementary material for: Loss of Serpina1 in Mice Leads to Altered Gene Expression in Inflammatory and Metabolic Pathways
Source: Int J Mol Sci. 2022 Sep 9;23(18):10425. doi: 10.3390/ijms231810425 (PMC9499171; doi:10.3390/ijms231810425)
Supplement: Supplementary file 1 [file ijms-23-10425-s001.zip › Full Supplementary Table S1.pdf]

Supplementary Table S1: *Serpina1* KO vs. wildtype all DEGs

Gene Name	baseMean	log2FoldChange	lfcSE	stat	*p*-value	adjusted *p*-value
Il15ra	403,5279214	-0,197396698	0,14296773	-1,380708067	0,16736874	0,506462808
Syt8	0,093953095	-0,517475177	3,352475198	-0,154356154	0,877328924	NA
Farsb	915,5641788	-0,070558063	0,105673773	-0,66769702	0,504327012	0,804997854
Trpv2	28,63898358	0,215417664	0,361435778	0,596005369	0,55117164	0,830401734
Calm1	5114,88163	-0,129221721	0,111341374	-1,160590324	0,245808542	0,605051632
Cgref1	18,76801947	-0,502904213	0,363662856	-1,382885836	0,166699874	0,50558317
Gpr137b-ps	18,92225329	0,605553812	0,377015279	1,606178439	0,108234709	0,40832289
Mrpl48	547,7895752	0,04888088	0,119061503	0,410551513	0,681401424	0,89166825
Vmn2r29	38,67614352	-0,115452072	0,324432727	-0,355858278	0,721946703	0,909644412
Cyp2d13	3779,897853	-0,160109597	0,172759263	-0,926778653	0,354041471	0,704551502
Trappc13	617,005355	-0,073073681	0,101002357	-0,723484915	0,469382019	0,784101987
Cdcp1	129,3227149	0,571497282	0,27967802	2,043411501	0,04101172	0,238839464
Gt(ROSA)26Sor	60,8805198	-0,268707278	0,388300245	-0,692009036	0,488931654	0,796400974
Abhd1	77,94505132	0,101407312	0,183003743	0,554126981	0,579491946	0,84530168
Pisd-ps2	8,260518421	-0,390684582	0,529277931	-0,738146368	0,46042551	0,780953195
Pisd-ps3	3490,559009	0,185796905	0,170839507	1,087552337	0,276792782	0,637663478
Pisd-ps1	130,285067	-0,093389597	0,292296755	-0,319502681	0,749345359	0,919838327
Mx2	138,7549897	0,074735367	0,186779566	0,400126033	0,689063691	0,894327388
Oas1b	40,76701816	0,174817724	0,240443544	0,727063496	0,467187059	0,782586618
Mcm7	361,1614928	-0,160629633	0,153350128	-1,047469834	0,294882938	0,655827892
Adamts7	280,9160148	-0,359356725	0,283430993	-1,267880837	0,204840508	0,557844548
D030028A08Rik	21,24878088	-0,290736793	0,390095914	-0,745295664	0,456093002	0,778008437
Slc13a2os	0,329241348	-1,910044376	3,172950079	-0,601977443	0,547189152	NA
Mtch2	4561,587862	0,131654067	0,080725845	1,63087876	0,102915904	0,399190747
Gnas	11106,56192	-0,120624679	0,119356759	-1,010622947	0,312196929	0,671711748
Gm614	0,300682528	-0,59004097	3,301569414	-0,1787153	0,858161255	NA
Snhg10	5,828136979	-0,397185525	0,676132436	-0,587437465	0,556909938	0,833822555
Snhg1	153,5846565	0,352595458	0,183991402	1,916369212	0,055318117	0,284475895
Gm12070	0,67741889	0,050889357	1,827258569	0,027850113	0,977781697	NA
Gm1821	3,42290943	-0,938441715	0,960460633	-0,977074626	0,328532208	NA
Dio3os	12,47765177	0,797442217	0,534447724	1,492086467	0,135676479	0,458266143
Myl6	4551,245172	-0,036832999	0,103708035	-0,355160514	0,722469344	0,909644412
Snord87	1,016791976	-0,318263469	1,70121757	-0,187079815	0,851598054	NA
Dpys	6326,821221	0,163055019	0,094933772	1,717565987	0,085875809	0,363485758
Ankrd33b	776,6995585	-0,256791658	0,105706471	-2,429289864	0,015128432	0,13432477
Tmem212	0,093303375	-0,517475177	3,352475198	-0,154356154	0,877328924	NA
Stk26	7,074060394	1,659551314	0,717372565	2,313374383	0,020702064	0,160630236
Grb14	1508,070536	0,124915298	0,117169551	1,066107173	0,286375198	0,648131311
Ubb	811,5495041	-0,535536855	0,13083671	-4,093169676	4,26E-05	0,002350713
Twist1	2,7203227	1,478377933	1,115143599	1,325728754	0,18492956	NA
Glmn	81,28263858	0,453384472	0,194483345	2,331225182	0,019741489	0,156724445
Usp33	976,0792753	-0,30540398	0,111448671	-2,740310653	0,006138114	0,078078443
Eif5a	8957,949792	-0,000586781	0,099063191	-0,005923301	0,995273917	0,998860221
Hmga1	2,652456568	1,01484633	1,04361365	0,972434895	0,330834239	NA
Dio1	2459,05826	-0,6025647	0,477455611	-1,262032921	0,206936941	0,559462075
Ggps1	215,0018108	-0,032746047	0,124552485	-0,26290962	0,792620242	0,936597818
Sirt3	1717,636312	-0,004074008	0,076354452	-0,053356519	0,957447848	0,98781716
Dkc1	405,6233153	0,093130506	0,110344195	0,84400005	0,398669378	0,739635153
Dnajc17	87,50967539	0,062429978	0,194073166	0,32168269	0,747693093	0,919586841
Adam23	156,8887598	0,006831595	0,324473512	0,021054401	0,98320226	0,995413661
Cldnd1	381,0660145	-0,060163307	0,099901104	-0,602228647	0,547021949	0,828308578
Pan2	843,4546252	-0,012706109	0,104565285	-0,121513646	0,903284207	0,973489035
Taz	681,7058354	0,093422267	0,115839039	0,806483448	0,419964148	0,75408072
Reep3	2286,55785	-0,041488849	0,130708214	-0,317415775	0,750928139	0,920556312
Reep2	1,937462507	-0,603729929	1,369941911	-0,440697466	0,659432031	NA
Ptrhd1	148,9694882	0,220645323	0,136954262	1,611087671	0,107160615	0,406189471
Scarb1	4281,967834	-0,037630482	0,107915526	-0,348703131	0,727312193	0,910689961
Gm5523	0,477834548	1,266973284	2,170191723	0,583807076	0,559350117	NA
Dach1	2,321252681	-0,087616298	1,209730201	-0,072426313	0,942262645	NA
1600012H06Rik	686,9799191	0,066368671	0,10437094	0,635892239	0,524846662	0,816659969
Prr5l	27,12686181	0,261229216	0,365388393	0,714935726	0,47464876	0,788662599
Cdh8	0,086476712	0,780932884	3,352475198	0,232942181	0,815806304	NA
Irx3	9,724557296	0,783986587	0,516992271	1,516437732	0,129408704	0,446986398

Sez6l	0,113662148	0,780932884	3,352475198	0,232942181	0,815806304	NA
Nat8f7	26,12436347	-0,925559778	1,181837249	-0,783153331	0,433537077	0,763329011
Slc10a7	207,3855761	-0,263876848	0,133019674	-1,983743006	0,047284497	0,259242489
Csmd2	0,12663974	0,780932884	3,352475198	0,232942181	0,815806304	NA
4930477N07Rik	0,086476712	0,780932884	3,352475198	0,232942181	0,815806304	NA
Ankle2	613,4092892	0,02663441	0,085367183	0,311998235	0,755041865	0,922462845
Tgfa	403,4368794	-0,38872336	0,134517371	-2,889763283	0,00385532	0,05784711
Gm2083	69,14384851	-1,199427371	0,737518938	-1,626300438	0,103885759	0,401053626
Ccdc9	397,8248112	0,217582421	0,120116412	1,81142958	0,070074381	0,32382454
Slc4a4	1346,717117	0,48522107	0,225747889	2,149393606	0,031603212	0,206478957
Mtl5	7,134305646	0,409869094	0,558563048	0,733791996	0,46307552	0,781575132
Lcn2	784,4901943	3,758258103	0,625663937	6,006831912	1,89E-09	8,43E-07
Nme1	2031,14807	-0,129602147	0,143390864	-0,903838247	0,366081178	0,714472885
Ganab	5229,2548	-0,237105261	0,091596402	-2,588587057	0,009637058	0,102236232
Rara	536,2844738	-0,031504429	0,145261376	-0,216880977	0,828301103	0,94762353
Smarcad1	283,1747463	-0,03404489	0,143316935	-0,237549668	0,812230393	0,943005481
Rnf208	3,447163775	1,676629725	0,957335565	1,751350087	0,079885625	NA
Ubash3a	2,29716591	0,264514209	1,125531701	0,235012669	0,8141989	NA
Ep300	692,1106578	-0,044887957	0,148686983	-0,301895674	0,762731594	0,925557106
Apol10b	6,918703594	0,619532691	0,613301658	1,010159817	0,312418727	0,672017932
Fan1	119,6547397	-0,10139045	0,176355527	-0,574920738	0,565344904	0,837554967
Sh2d4b	0,945949102	1,6000011	1,715763794	0,932529935	0,351062724	NA
Zfp354a	105,7528585	-0,110254801	0,207597639	-0,53109853	0,595350503	0,852897651
Zfp459	3,361201098	1,98182774	1,1787517	1,681293643	0,092705892	NA
Ano5	2,291035489	-1,708570087	1,032754443	-1,654381735	0,098049975	NA
Slc25a48	139,4893685	-0,097972556	0,205825224	-0,47599878	0,634075247	0,867989994
Frs2	378,6850144	-0,059388826	0,138510172	-0,428768695	0,668091563	0,88634674
Prpf39	384,8564284	0,181569239	0,118470325	1,532613664	0,125371073	0,440843206
Fam179b	255,6103116	-0,163689126	0,173769305	-0,941991026	0,346197235	0,698497639
Tmem26	32,84960448	0,476031523	0,321316787	1,481502186	0,138472803	0,462146673
Mettl24	1,22160035	-0,339984111	1,24609605	-0,27283941	0,784976663	NA
Slc7a15	1,879327587	1,227822342	1,334835108	0,919830722	0,357661226	NA
Catsper4	0,087021394	-0,517475177	3,352475198	-0,154356154	0,877328924	NA
Gtf3c4	482,232216	0,035558463	0,128993416	0,275661073	0,782808397	0,93249269
Adra2c	0,645266574	2,752362233	2,243171795	1,226995739	0,219824193	NA
Ncald	382,1759032	0,173145018	0,198023612	0,874365518	0,381919229	0,726456076
Ccdc78	2,492324212	0,211833011	1,088582047	0,194595356	0,845709739	NA
A4galt	5,008344521	0,359181279	0,658867031	0,545149874	0,585650443	NA
Pym1	298,9096756	0,002258908	0,130538128	0,017304582	0,986193631	0,996004319
Slfn14	0,093953095	-0,517475177	3,352475198	-0,154356154	0,877328924	NA
Asph	1025,099547	-0,21771849	0,119579106	-1,820706792	0,068651439	0,320580855
Spag9	1838,016099	0,225361089	0,10985722	2,051399885	0,04022802	0,236119007
Usp40	809,2881787	0,043953421	0,117752655	0,373269039	0,708948217	0,903348143
St6gal1	2253,863128	0,309456783	0,166834188	1,854876312	0,063613891	0,306733002
Ldlr	4199,105282	-0,219025687	0,112504607	-1,946815269	0,051556886	0,273331789
Cyb5r2	1,102067009	-1,374644858	1,61386651	-0,851771103	0,394341148	NA
Smpd5	9,2045885	0,916919812	0,770518517	1,190003605	0,234044975	0,591363094
Gm5741	1,252040867	0,326441938	1,604150306	0,203498349	0,838745536	NA
Ypel2	772,7816419	-0,081093373	0,203699166	-0,398103608	0,690553815	0,894644908
Hif3a	1,582727554	2,47085178	1,720183641	1,436388373	0,15089186	NA
Stk16	1731,919924	-0,245672174	0,076388285	-3,216097494	0,001299467	0,028479625
Dnttip2	568,3410008	0,053078895	0,141294435	0,37566161	0,707168482	0,902542762
1700001G17Rik	2,393310356	0,278206633	1,107739171	0,25114814	0,801699578	NA
Uevld	227,5729533	-0,01219881	0,153186037	-0,079633956	0,936528388	0,982191945
Als2	2254,532911	-0,244028352	0,130565825	-1,869006317	0,061621936	0,300885192
Etnppl	5535,622674	-0,148201489	0,437650563	-0,338629722	0,734888689	0,913858791
Knop1	842,3048005	-0,103499398	0,103494013	-1,000052032	0,317285328	0,67555145
Pqlc1	1918,78902	-0,091960063	0,077521224	-1,186256592	0,235520983	0,592971756
Rchy1	624,9428428	0,057359606	0,089124778	0,643587652	0,51984286	0,813876985
Acin1	1389,820084	0,06324154	0,096305743	0,656674653	0,511390136	0,809564799
Kctd17	100,0327352	-0,346773423	0,164121693	-2,112904251	0,034608964	0,217104886
Pip5k1a	272,523816	-0,079309523	0,132591271	-0,598150412	0,549739575	0,82961684
Kpna1	878,3818995	-0,390783902	0,14846703	-2,632125803	0,008485244	0,095327078
Upp2	4087,935795	0,519842847	0,260074442	1,998823272	0,045627479	0,253214514
Ly6c1	3,953723718	1,644840935	0,947002539	1,736891791	0,082406273	NA
Egfl8	15,3544675	-0,251411911	0,412798703	-0,609042396	0,542496338	0,826611597

Supplementary Table S1: *Serpina1* KO vs. wildtype all DEGs

Dner	0,086476712	0,780932884	3,352475198	0,232942181	0,815806304	NA
Txnrd1	3508,4737	-0,001834975	0,107013095	-0,0171472	0,986319185	0,996004319
Tcn2	2677,453558	0,40242574	0,173643735	2,317536765	0,02047451	0,159698791
Pgf	0,407849306	-1,152707391	2,507822049	-0,459644811	0,645771189	NA
Kcnj15	6,419193272	-0,422097396	0,882352269	-0,478377412	0,632381607	0,867306747
Sun3	4,781411813	2,520908824	0,955982099	2,636983293	0,008364695	NA
Arhgef1	1001,184927	-0,165931633	0,11549596	-1,436687767	0,150806732	0,483253884
Mir3569	0,093303375	-0,517475177	3,352475198	-0,154356154	0,877328924	NA
Scrg1	0,093953095	-0,517475177	3,352475198	-0,154356154	0,877328924	NA
Stmn3	0,227324296	1,389394708	3,349408814	0,414817893	0,67827522	NA
Sptbn1	1714,308418	-0,122053482	0,108238446	-1,127635199	0,259474015	0,620981526
Mir7036	2,006100576	2,001576072	1,207827714	1,657170182	0,097485073	NA
Tars2	680,7618342	-0,189540625	0,097186321	-1,95028089	0,051142649	0,272835625
1810011H11Rik	25,57503111	0,24198922	0,334822982	0,722737783	0,469840998	0,784378659
Fmnl3	73,01688334	-0,150156432	0,270215237	-0,555691952	0,578421459	0,84530168
Rbp4	70931,08784	-0,009744052	0,105066024	-0,092742178	0,926108389	0,978534437
Mir6938	0,494174502	1,428175777	2,441007209	0,585076427	0,558496328	NA
Dcun1d2	355,5146917	-0,311538881	0,124954533	-2,493217916	0,012659113	0,121248489
Akap2	0,142634598	-0,517475177	3,352475198	-0,154356154	0,877328924	NA
Cenpm	58,07807093	1,684720943	0,414194607	4,06746229	4,75E-05	0,002562099
Tmpo	924,2258941	-0,045525004	0,154380392	-0,294888512	0,768079048	0,927592484
Vav1	77,72346302	0,318724325	0,306043399	1,041435055	0,29767368	0,658220216
Zfp68	403,5983074	0,25166466	0,100796114	2,496769476	0,01253304	0,120716293
Treml4	142,8852569	0,187390639	0,253351733	0,739646171	0,459514718	0,780591139
Hsd11b2	0,295994437	0,617282395	2,762021179	0,223489378	0,823154649	NA
Poln	0,984213457	1,685213427	1,56846264	1,074436448	0,282627114	NA
Zpbp	0,187906191	-1,241948083	3,339949325	-0,371846385	0,710007226	NA
Postn	316,1002807	-0,174661095	0,276531881	-0,631612869	0,527639875	0,818474757
Isg15	237,7224372	0,16404829	0,190647323	0,860480427	0,389524268	0,73337178
Snrpa	843,4240473	-0,156595331	0,115255234	-1,358683034	0,174247051	0,517013028
Nap1l1	1423,28767	-0,246578363	0,118440027	-2,08188371	0,037353093	0,226546639
Elane	1,946801078	0,045187357	1,49683221	0,030188659	0,975916593	NA
Cfhr1	3410,113814	-0,072571215	0,108665232	-0,667842085	0,504234399	0,804997854
Cadm1	789,6026293	0,163438396	0,113700993	1,437440356	0,150592909	0,483083894
Apom	7878,896987	-0,171479536	0,08210656	-2,088499819	0,036752772	0,224873137
Nup210	409,0737849	-0,198355713	0,190989865	-1,038566697	0,299006301	0,659904651
Rpp25	1,58566563	2,492597022	1,729356055	1,441344028	0,149487515	NA
Elmo1	61,41758013	0,63263396	0,250563049	2,524849383	0,011574789	0,115136419
Cbx3	205,6870024	0,11224116	0,11403205	0,984294851	0,324970561	0,682335948
Cd72	75,73003993	0,37643985	0,268363804	1,402722143	0,160699712	0,497355545
Lrrfip2	917,3286127	-0,273257782	0,134317404	-2,034418285	0,04190944	0,242024752
Idh3g	1642,442344	0,075343814	0,090021567	0,836952932	0,402619025	0,742634347
Id3	1428,961092	-0,247182143	0,522777849	-0,472824439	0,636338437	0,869590981
Prom1	49,55920848	1,34330629	0,732976147	1,832673949	0,066851074	0,317011065
Ptbp1	2227,544098	0,05478873	0,084674872	0,647048285	0,517600693	0,813424627
Ntn1	224,8564237	-0,007900057	0,166917553	-0,047329096	0,962250939	0,988526411
Nthl1	136,1933458	-0,266794817	0,164281241	-1,624012674	0,104373104	0,401673966
Nsg2	1,580292932	0,554459315	1,277640077	0,433971449	0,664309174	NA
Ceacam20	0,888300442	3,209780574	1,837968135	1,746374441	0,080745908	NA
Wfdc15b	4,199420542	0,380688916	0,820242438	0,464117557	0,642563524	NA
Zfp868	119,9638005	-0,036832886	0,141781564	-0,259786142	0,795028741	0,937443926
Iigp1	8742,866949	0,243555545	0,16003595	1,521880204	0,1280391	0,445305601
Gjc1	7,913126337	-0,170012626	0,558269609	-0,304534983	0,760720345	0,925140571
Snord89	4,410089594	0,566107192	0,794147149	0,71284924	0,475939052	NA
Rbbp8	157,1269243	0,046547542	0,22599213	0,205969748	0,836814542	0,951141594
Amer2	8,347465391	-0,928594628	0,631019392	-1,47157859	0,141134711	0,466809953
Slc17a3	2234,785602	-0,551895602	0,145312846	-3,797982206	0,000145879	0,005945895
Pcdhb12	1,438192586	0,337604765	1,358106066	0,248584977	0,803681831	NA
Pcdhga10	1,823994819	-1,81375867	1,194214864	-1,518787552	0,12881598	NA
Sh3glb1	1448,436614	0,035862127	0,085204441	0,420895041	0,673831727	0,888626355
Sfmbt1	538,9134015	-0,35707053	0,114533695	-3,117602463	0,001823286	0,036058439
Art2b	7,46318506	0,538314848	0,523350641	1,028593082	0,30367093	0,66325429
Zbp1	251,5647962	0,359063261	0,216612075	1,657632709	0,097391624	0,388433358
Hpgds	22,16771858	0,787984009	0,422480946	1,865135022	0,062162481	0,302640278
Dll4	75,01632814	-0,187941685	0,400910048	-0,468787664	0,639221418	0,871049783
Unc93b1	909,9918752	0,079823976	0,117166395	0,681287296	0,49568972	0,80006558

Samd4	103,5921327	0,431316291	0,207850532	2,075127195	0,037974748	0,228686669
Barhl1	4,467858672	-0,391190891	0,844590729	-0,463172135	0,643240988	NA
Rdh11	2515,848031	0,856155023	0,196426538	4,358652512	1,31E-05	0,001041395
Ramp2	131,9592129	-0,210373892	0,244814667	-0,859318989	0,390164551	0,733990715
Ndufa1	1958,886226	-0,038481899	0,139316625	-0,276219001	0,782379864	0,932480735
Stk19	169,101575	-0,264614798	0,139588375	-1,89567934	0,058002459	0,290969934
Ppt2	888,5200666	-0,102344051	0,095510875	-1,071543436	0,283925151	0,645214235
Irgm2	1510,962082	0,297572127	0,135472619	2,196548125	0,028052735	0,192787383
Rbm14	335,7857279	-0,223860688	0,149012916	-1,502290495	0,133022102	0,453898031
Ncoa6	726,3839398	0,044234544	0,148182003	0,298514953	0,765310165	0,926709042
Usp14	1053,420231	0,019524711	0,118208735	0,165171477	0,868809015	0,961775826
Ncapg	8,857982325	-1,487467485	0,671802053	-2,21414549	0,026818772	0,18930315
Wnk1	1831,787328	-0,218225137	0,114613064	-1,904016256	0,056908067	0,288295377
Vsir	140,9660432	-0,244242161	0,206470078	-1,182942165	0,236832069	0,59395959
H13	3486,519796	-0,337387465	0,116924989	-2,885503492	0,003907881	0,0583195
Ephb6	18,96546258	-0,154438043	0,439129535	-0,351691314	0,725069775	0,910252319
Chpt1	3184,226632	0,051163728	0,174402768	0,293365346	0,76924291	0,927740953
Smn1	164,8247415	0,091158371	0,147992055	0,615968007	0,537915635	0,82460384
Per1	138,3699759	0,978537264	0,29712423	3,293360703	0,000989974	0,023507922
Ifng	1,051810614	1,091352872	1,508010811	0,723703613	0,469247715	NA
Hus1	138,8544182	0,081016679	0,153387276	0,528183832	0,59737175	0,853549036
Lrrc47	423,0511603	-0,102050148	0,102357423	-0,996998024	0,318765469	0,676547953
Synj2	79,63833076	-0,342196781	0,217223842	-1,575318704	0,115182899	0,420158301
Ajap1	1,765118922	0,483700614	1,154963253	0,418801735	0,675361037	NA
Zc3h12c	103,560219	-0,085916142	0,159569988	-0,53842294	0,590285093	0,849647008
Gprasp2	5,172637339	0,889113621	0,671720252	1,323636766	0,185623709	NA
Klhdc7b	5,980001178	0,392172096	0,706535689	0,555063392	0,578851301	0,84530168
Klk8	4,406459293	0,440713393	0,792161194	0,556343073	0,577976347	NA
Tcf20	599,5126674	0,22922469	0,109589007	2,091675952	0,036467513	0,2237085
Nit1	2690,315045	0,118857327	0,076783561	1,547952772	0,121633668	0,433672149
Dnah7b	2,029280614	0,262879388	0,995693384	0,264016405	0,791767278	NA
Hagh	7630,753901	-0,170701268	0,11406445	-1,496533469	0,134514693	0,456327747
Srp19	461,3554638	-0,323144796	0,148528283	-2,175644856	0,029581826	0,198235307
Desi2	731,5237461	-0,155026554	0,1347391	-1,150568425	0,249909829	0,60896459
Txnl4a	1552,827355	-0,041474829	0,099070822	-0,418638184	0,675480579	0,889164486
Mien1	1214,513422	0,025985621	0,107435397	0,241872061	0,808879299	0,94241441
Cyb5b	17323,57157	0,054958616	0,107037621	0,513451399	0,607635599	0,857689206
Coprs	57,91789565	0,220871454	0,263880509	0,837013142	0,402585181	0,742634347
2410004B18Rik	93,85381154	0,00017677	0,177487877	0,000995956	0,999205342	0,999803624
Polr2e	732,6412674	0,052655615	0,127599455	0,412663319	0,679853303	0,891462871
Tti2	277,1200606	-0,017930528	0,120056652	-0,14935056	0,881277021	0,965734294
Rbm47	1365,14803	-0,449592093	0,12228393	-3,67662449	0,000236341	0,008516541
Sardh	24372,23797	0,229435067	0,100184472	2,290126034	0,022014012	0,165937181
Dnajc28	314,6954993	0,119451945	0,144834289	0,824749067	0,409514069	0,746907036
Ano1	54,1815309	0,67342681	0,316220989	2,129608197	0,033203974	0,21273931
Commd1	866,7035791	0,026566419	0,07020806	0,378395569	0,70513676	0,90193988
Casc3	562,223803	0,052904768	0,093116501	0,568156741	0,569928542	0,841119289
Prpf8	5251,398194	-0,063807922	0,116243892	-0,548914195	0,583064336	0,846950064
Socs7	264,8842198	-0,468795293	0,14008348	-3,346542307	0,000818262	0,0206014
Sugct	773,2534553	0,170449127	0,124002561	1,374561349	0,169267478	0,508724295
Bspry	4,070118621	1,678540355	0,847308659	1,981025848	0,047588375	NA
Ssu2	0,180429807	0,059593471	3,352475198	0,017775962	0,985817581	NA
Fam221b	0,093303375	-0,517475177	3,352475198	-0,154356154	0,877328924	NA
Tlr5	176,966026	-0,173117911	0,225190085	-0,768763469	0,442033739	0,769003232
Dand5	96,37650858	-0,052064284	0,186433693	-0,279264349	0,780041964	0,931606698
Brip1	39,40363866	-0,113020361	0,307522511	-0,367518984	0,713231934	0,905933635
Agap1	256,379881	-0,365167552	0,150290764	-2,429740468	0,015109638	0,13432477
Crip3	6,813696664	-0,486436338	0,558107629	-0,871581596	0,383436675	0,728045008
Ric8b	303,6570277	-0,28692893	0,144687046	-1,983100342	0,047356222	0,259363104
Gpatch11	290,663047	-0,021646603	0,115421221	-0,187544394	0,85123382	0,955747343
Retnlg	2,320365576	0,633994795	1,089886211	0,581707327	0,560763835	NA
Ppp1r9a	152,499814	-0,440541225	0,23496334	-1,874936005	0,060801528	0,298734152
Phactr3	0,48444531	-1,523020831	2,363589044	-0,644367867	0,519336916	NA
Nrg2	0,180974489	-1,204797453	3,342691436	-0,360427361	0,718527568	NA
Clk3	982,0089349	-0,156941997	0,115215451	-1,362161036	0,173147069	0,51532004
Serpina3f	47,48165491	0,539119045	0,343913505	1,567600683	0,116974353	0,424373183

Cdk19	162,5051876	0,012003369	0,140947639	0,085161901	0,932132679	0,980925833
R3hdm2	1970,371506	0,031654349	0,135060748	0,234371193	0,81469682	0,944076707
Sugp2	294,1720644	-0,280205569	0,149496808	-1,874324757	0,060885677	0,298973723
Pdss2	240,7517863	-0,132029703	0,117798278	-1,120811824	0,26236797	0,622562722
Arhgap39	81,90492573	-0,291081528	0,171176122	-1,700479742	0,089040724	0,369687145
Ddx51	247,0791515	0,055814874	0,139807621	0,399226262	0,689726494	0,894436573
Tmbim1	1230,334242	-0,050681732	0,081393425	-0,622675998	0,533497458	0,821247926
Rufy3	1369,417188	-0,167011379	0,114516126	-1,458409264	0,144727769	0,472725517
N6amt1	151,736613	0,410608544	0,141989832	2,891816534	0,003830215	0,057613111
9130024F11Rik	0,12663974	0,780932884	3,352475198	0,232942181	0,815806304	NA
Nup107	116,4557811	0,192197871	0,192176638	1,000110487	0,317257042	0,67555145
Atp5j	4946,722525	0,103995082	0,100768298	1,032021814	0,302061898	0,662128551
Caprin1	6475,874118	-0,024598859	0,099032143	-0,248392675	0,803830601	0,940361036
Keap1	1489,894859	0,062344576	0,083560379	0,746102119	0,455605729	0,77770595
Reck	143,1562715	0,061587369	0,151492631	0,406537063	0,68434803	0,892349329
Rpl13	3753,212987	-0,024716989	0,13036265	-0,18960177	0,8496212	0,954743122
Gas2l3	52,47866713	-0,318407792	0,259376372	-1,227589813	0,219600988	0,574490867
Lhb	0,086476712	0,780932884	3,352475198	0,232942181	0,815806304	NA
Pex5	3035,99263	-0,03526111	0,101941753	-0,34589468	0,729421867	0,911594858
Man2b2	1248,727655	0,186422553	0,110637055	1,684992005	0,091990114	0,376435823
Man2a1	3525,894943	0,112055992	0,098545754	1,137096088	0,255498119	0,615895123
Man1a	3697,518862	0,005598216	0,110205569	0,050797943	0,95948653	0,987937108
Mak	1,430805325	0,348540817	1,403505485	0,248335914	0,803874514	NA
Mfap2	3,424431024	0,560449864	0,886547178	0,632171505	0,527274815	NA
Smad6	143,0016069	-0,019785781	0,235031402	-0,084183564	0,932910486	0,981257162
Smad5	535,7506892	0,030567144	0,109599429	0,278898757	0,780322522	0,931606698
Smad4	559,8088674	0,025888846	0,113596373	0,227902049	0,81972239	0,946198293
Smad1	374,6109456	-0,05797361	0,097550536	-0,594293096	0,552316095	0,830773388
Tep1	354,7774563	-0,090049167	0,146212841	-0,61587728	0,537975518	0,82460384
Prim2	78,42395455	-0,18814018	0,180814315	-1,040515956	0,298100255	0,658727654
Aldh1a2	6,598122052	0,72280262	0,770874627	0,937639656	0,348429625	0,700474534
Mecp2	330,9752307	-0,008345739	0,140018181	-0,059604679	0,952470492	0,986410877
Ankfy1	756,0300789	-0,056641187	0,12576842	-0,450360969	0,652450183	0,877662367
Numbl	18,52164096	-0,092651242	0,345600785	-0,268087475	0,788631986	0,935202374
Slc22a3	69,42733382	0,648177008	0,352460243	1,839007437	0,065914092	0,314155258
Msh3	309,3910393	0,159002476	0,145104062	1,095782391	0,273174016	0,634872238
Mdm1	27,83266265	0,149182951	0,349151146	0,427273268	0,669180301	0,886730269
Pax8	1,489308181	1,178147965	1,282454678	0,918666356	0,358270113	NA
Oxt	0,087021394	-0,517475177	3,352475198	-0,154356154	0,877328924	NA
Coro1a	110,9222528	0,65971464	0,21444001	3,076453123	0,002094792	0,039203296
Dnajc10	559,6481429	-0,199529026	0,1381998	-1,443772174	0,148803072	0,479794094
0610009O20Rik	924,8333012	0,101530018	0,115385669	0,879918784	0,378903308	0,724790145
Ormdl2	1080,424175	-0,016940533	0,103334533	-0,163938738	0,869779371	0,961775826
Alg14	354,0357275	0,054159959	0,099333904	0,54523135	0,585594412	0,847482024
Msmo1	6645,370361	0,92539382	0,160723837	5,757663815	8,53E-09	3,07E-06
Drap1	1376,724545	0,0086399	0,097787032	0,088354251	0,929595121	0,980107033
Popdc3	1,237836178	-0,315032296	1,24770406	-0,252489598	0,800662654	NA
Mrps23	617,526039	-0,140295567	0,104652205	-1,340588731	0,180054018	0,52303526
Atp6v1g1	1302,676572	0,052355187	0,125282122	0,417898307	0,676021471	0,88927955
Ing3	186,8171243	0,058028418	0,136094871	0,426382106	0,669829438	0,887039558
Nenf	235,3088474	-0,050812239	0,142848129	-0,355708116	0,722059166	0,909644412
Aspn	1,528316547	0,113823831	1,301544611	0,087452885	0,930311534	NA
Armt1	369,4766305	-0,271369208	0,14299914	-1,897698187	0,057735852	0,290395259
Hdac9	22,66267705	0,051760338	0,459311467	0,112691151	0,910275417	0,9747592
Ern1	893,5048711	-0,178592514	0,201886044	-0,884620403	0,376361398	0,722097522
Scyl1	1147,141149	-0,174607477	0,091310358	-1,912241731	0,055845196	0,285438885
Tsc22d4	302,7285607	0,435364958	0,186094533	2,339482795	0,019310461	0,155145164
Slco3a1	94,37179867	0,171538188	0,230423825	0,744446403	0,456606455	0,778260481
Rbm45	286,2519186	-0,149673603	0,113123271	-1,323101792	0,185801529	0,532805747
Las1l	1019,478533	-0,147484364	0,076065807	-1,938904877	0,052512922	0,276416937
Ttc27	250,8618934	-0,109644507	0,11290558	-0,971116811	0,331490113	0,688274004
Dnm1l	1295,310128	-0,225805165	0,103676934	-2,17796917	0,029408332	0,197567835
2900011O08Rik	2,60628076	1,629312856	1,064292359	1,530888428	0,12579697	NA
Zfp566	12,55783205	-0,008686045	0,436682237	-0,019890996	0,984130328	0,995554769
Snora41	2,047009783	0,303600114	1,202481431	0,252478006	0,800671612	NA
Synpo	177,1531018	-0,33420472	0,167918118	-1,990283859	0,046559675	0,256471486

Fxyd5	130,4015671	0,159861365	0,180266788	0,886804314	0,375184264	0,721201232
Fyttd1	513,5067723	0,039843709	0,117980729	0,337713704	0,735578947	0,914229638
Svip	371,8603781	0,102996476	0,127166755	0,80993241	0,417979023	0,752384753
Gmppb	462,5951536	-1,092263521	0,167551893	-6,518956601	7,08E-11	4,30E-08
Ctcf	782,5022165	0,055101708	0,090879878	0,60631362	0,544306516	0,827477215
Tmc6	164,4698873	0,239867154	0,128977072	1,859765843	0,062918662	0,304547281
Npm2	1,367629496	-0,162031619	1,388512288	-0,116694407	0,907102222	NA
Bbs9	114,7924566	-0,029720515	0,171927368	-0,172866689	0,862756216	0,960942085
Car5b	82,99384779	0,265586306	0,196399799	1,352273818	0,176287737	0,519345693
Bre	672,5690916	0,045033955	0,096404187	0,467136926	0,640401917	0,871049783
Zdhhc23	1,889024189	-1,122014629	1,004031729	-1,117509135	0,263776696	NA
Btla	14,82683287	0,856436081	0,535219019	1,600160029	0,109563087	0,409719974
Tsc22d1	1422,361074	-0,593134919	0,480550312	-1,234282664	0,217097594	0,570772415
Hdac4	163,3974435	-0,190086124	0,161678324	-1,17570568	0,239712508	0,597294217
Dsg1c	206,7823137	0,768031339	0,470785053	1,631384292	0,102809257	0,399087387
Pkig	284,0882761	-0,277693471	0,152546913	-1,820380789	0,068701036	0,320580855
Fam195b	454,9417553	0,123932234	0,125016856	0,991324192	0,321527304	0,678746017
Dntt	74,2658436	1,383491547	0,45226804	3,059007987	0,002220712	0,040895094
Atp1a3	6,100331428	0,076886657	0,755038336	0,101831461	0,918890453	0,976907366
Stx6	255,9051426	0,08885198	0,142402195	0,623950914	0,532659822	0,820654481
D830030K20Rik	0,209517726	0,059593471	3,352475198	0,017775962	0,985817581	NA
Cers2	9136,404746	0,117033342	0,080891041	1,446802269	0,147952311	0,478118068
Stk40	975,2097567	-0,064339599	0,103231144	-0,623257643	0,533115228	0,820918853
Nus1	1208,381709	-0,275017131	0,095159404	-2,890067808	0,003851587	0,05784711
Acot12	2246,444234	0,044303011	0,134621066	0,329094194	0,742084493	0,916899135
Dcaf17	284,1632771	-0,10555914	0,118758287	-0,888857043	0,374079914	0,720823634
Brcc3	511,105977	-0,199148095	0,121527628	-1,638706342	0,101274433	0,395541305
Ano8	104,4677184	0,464128168	0,187475431	2,475674626	0,013298471	0,124326755
Trpc4ap	2616,560252	-0,128793676	0,092643444	-1,390208203	0,164465663	0,502924549
Cd300lf	23,05694676	0,457773335	0,351461083	1,302486555	0,192750111	0,541552905
Gsn	543,1746152	0,119418872	0,4492899	0,265794694	0,79039733	0,935843529
Mtr	99,24828513	-0,342645132	0,225126813	-1,522009427	0,128006719	0,445305601
Rnf217	301,7131382	0,303759259	0,174759496	1,738155962	0,082183336	0,354079608
Eng	1416,12286	-0,141071898	0,211526272	-0,666923766	0,50482083	0,805075709
Pclo	32,15729174	0,872573187	0,379423435	2,29973456	0,021463263	0,163367723
Sult2b1	1,647134808	0,109037438	1,166747566	0,09345418	0,925542749	NA
Gyg	134,9685118	0,545128886	0,166682148	3,270469537	0,001073691	0,025007275
Frg1	509,1880856	0,113888366	0,092834533	1,226788805	0,219901979	0,574490867
Fpr1	89,05650406	0,053239427	0,261497747	0,203594211	0,838670618	0,951141594
Flt3l	181,7638918	0,113705074	0,141829857	0,801700548	0,422726199	0,755522295
Fcer2a	20,81251253	0,307835645	0,406889526	0,756558291	0,449314538	0,773349269
Tnfrsf19	126,3537893	1,149312023	0,221576045	5,18698681	2,14E-07	3,86E-05
Stom	1462,48843	-0,079428503	0,096802886	-0,820517922	0,411920917	0,749099865
Snora68	5,971064835	0,430177713	0,679260377	0,633303115	0,526535719	0,817757439
Snora69	0,575562839	0,534485897	2,420632929	0,220804192	0,8252449	NA
Sfxn3	39,84884888	0,261838121	0,240581404	1,088355611	0,276438149	0,637501364
Tle6	59,42481715	0,010783454	0,20899627	0,051596394	0,958850293	0,987896001
Ogfr	917,0692025	-0,078532514	0,073079691	-1,074614749	0,282547246	0,644163391
Nxf2	0,44777597	-1,530984821	2,09129203	-0,732076056	0,464122148	NA
Chrdl1	3,783520463	1,446017474	0,849370009	1,702458832	0,08866939	NA
Plekha2	99,2120588	0,176393175	0,264847322	0,666018344	0,505399375	0,805643837
Plekha3	395,032917	-0,046392592	0,115640653	-0,401178918	0,688288401	0,893892329
Trem2	18,20662158	0,05431543	0,535723122	0,10138713	0,919243152	0,976974458
Il23a	2,911753505	-0,343608403	0,86965845	-0,395107301	0,692763701	NA
Ctns	419,5910585	0,041579179	0,110513188	0,376237262	0,706740516	0,902297674
Inpp5e	253,0388313	0,002000173	0,153524108	0,013028396	0,989605138	0,996712773
Tfec	36,91422392	0,283265493	0,296471323	0,955456638	0,339346824	0,694113783
Slc19a1	115,8561859	0,20134519	0,174191713	1,155882712	0,247729135	0,607129386
Msr1	236,5190662	0,122244772	0,222475745	0,549474602	0,582679789	0,846891345
Ren2	0,285269197	-1,521344816	3,321239936	-0,458065315	0,646905517	NA
Tsta3	623,7468296	-0,192577375	0,093196315	-2,066362562	0,038794258	0,230917381
Mup1	1934,652676	-1,745596241	1,278937771	-1,364879732	0,172290854	0,513629257
Ndst3	0,360209894	-0,130834948	2,660913913	-0,049169177	0,960784475	NA
Akap12	71,0750745	-0,210241525	0,306650124	-0,685607172	0,492960857	0,798745371
Glis2	36,94864346	0,494169289	0,282668558	1,74822871	0,080424432	0,350731846
Sp6	1,451480658	0,242329745	1,188768673	0,20384937	0,838471211	NA

Tfap4	104,7203155	0,307627065	0,156023137	1,971675936	0,048646612	0,264148764
Nxph3	1,073569232	0,407924151	1,628794545	0,25044543	0,8022429	NA
Klb	573,184954	0,578469444	0,165310834	3,499283318	0,000466511	0,013952528
Sf3b1	2486,445129	0,056622658	0,06622425	0,855013963	0,392543418	0,734985963
Tlr9	7,757340351	0,607761007	0,534641077	1,136764519	0,25563674	0,616118185
Ift122	101,1306899	-0,433322509	0,154903291	-2,797374449	0,005151979	0,070015871
Tnxb	235,4292154	-0,298314713	0,289090676	-1,031907072	0,302115653	0,662128551
Dscam	0,207615244	0,059593471	3,352475198	0,017775962	0,985817581	NA
Cacnb1	48,14485669	2,807211446	0,384862254	7,294067982	3,01E-13	3,09E-10
Trim17	0,229655992	-1,252384348	3,339190293	-0,375056298	0,707618598	NA
Kcnmb1	1,018048271	-1,521687826	1,54774855	-0,983162172	0,325527629	NA
Mrpl34	885,1331044	-0,017315281	0,165158249	-0,104840547	0,916502335	0,976559041
Tm2d1	319,1923492	0,01602841	0,110392592	0,145194617	0,884557216	0,967174729
Klrk1	30,59144375	0,386216265	0,33572485	1,150395226	0,249981126	0,60896459
1110032A03Rik	885,352207	-0,074834597	0,096932652	-0,772026712	0,440098607	0,767402932
Il1rn	58,28950171	1,093171184	0,434956414	2,513289032	0,011961129	0,116636279
Id4	28,48198407	-0,181057887	0,340475961	-0,531778766	0,594879231	0,852405191
F13b	5366,422332	0,02691521	0,10659458	0,252500734	0,800654047	0,939490312
Clec4g	1032,145997	-0,296800859	0,233344904	-1,27194061	0,203394221	0,555983883
Nln	630,9140274	-0,147439139	0,105237957	-1,401007226	0,161211912	0,497950692
Mff	614,4145391	0,014258995	0,0972941	0,146555596	0,883482805	0,966395158
Nudt16	194,1470279	0,306983892	0,170488716	1,800611198	0,071764183	0,328566905
Cd247	8,180858617	0,899731126	0,523917643	1,717314044	0,085921807	0,363485758
Clca3a2	1,733451413	3,388998734	1,578093089	2,147527771	0,031751289	NA
Ank1	2,022071412	-0,908136532	1,255911474	-0,723089605	0,469624835	NA
Slmap	1289,609491	-0,060166507	0,117709056	-0,511145948	0,609248864	0,857753956
Pard6g	13,58104064	-0,394479353	0,407636866	-0,967722466	0,333182995	0,68995097
Glrx	668,2644176	0,397561539	0,166070465	2,393932832	0,016668801	0,14321671
Lbh	191,436168	-0,010083729	0,241333806	-0,041783327	0,966671426	0,990593599
6030458C11Rik	390,5325979	-0,069817328	0,116045372	-0,601638192	0,547415	0,828528375
Clec9a	43,26278071	0,155086826	0,386079978	0,401696113	0,687907685	0,893697803
Serpina3i	1,47860458	0,353417732	1,360977574	0,259679321	0,795111145	NA
Fam198a	332,9825222	-0,337690969	0,277131388	-1,218522998	0,223025291	0,57807298
Pfdn4	122,6223079	0,053357029	0,149671056	0,356495304	0,721469669	0,909525085
Kcnma1	4,195310035	0,436459641	0,748990691	0,582730395	0,560074809	NA
Zwint	1496,162565	0,007235948	0,116869715	0,061914657	0,950630796	0,985972877
Carhsp1	2106,725928	0,061229845	0,107734073	0,568342429	0,569802474	0,841119289
Tax1bp1	3231,185332	0,003976547	0,077367474	0,051398182	0,959008234	0,987896001
Aggf1	657,589021	-0,110680881	0,093452321	-1,184356682	0,2362719	0,593521051
Cox6b1	5390,47882	0,054511999	0,107315967	0,507957953	0,611482831	0,858645358
Aagab	847,9203746	0,13496811	0,097195586	1,388623858	0,164947159	0,503350506
Mpi	333,196225	-0,269751909	0,153990548	-1,751743288	0,07981796	0,349746007
Krtcap2	1160,467957	-0,260393717	0,113304941	-2,298167368	0,021552265	0,163772191
Arrdc2	227,830893	0,38885267	0,2037035	1,908915011	0,056273056	0,286814521
Polr2d	107,5647864	0,157130221	0,197664135	0,794935414	0,426651073	0,758848669
Eif1b	445,2953494	0,142301947	0,160656954	0,885750312	0,37575209	0,721655743
Cdan1	168,0662324	0,063648089	0,12343468	0,515641869	0,606104562	0,856569383
Zfp580	18,59364884	0,546146669	0,384957892	1,418717944	0,155981264	0,491124238
Ngdn	483,340084	-0,353540194	0,121069873	-2,92013352	0,003498814	0,055030177
Dlgap1	37,31331719	-0,074447632	0,324842004	-0,229181053	0,818728199	0,945934875
Secisbp2l	1258,389525	0,010497048	0,121690282	0,08626036	0,931259449	0,980584977
Sncaip	12,56624043	-0,243360227	0,463580536	-0,524957818	0,599612515	0,85430922
Gpsm1	29,65064968	-0,0984849	0,264733452	-0,37201532	0,709881443	0,903762023
Lama3	184,1712665	-1,421905868	0,573904839	-2,477598676	0,013226983	0,123918384
Macf1	1485,364919	-0,135924733	0,080615427	-1,686088364	0,091778781	0,376031419
Rad51ap1	5,782274749	-1,177228596	0,68457659	-1,719644835	0,085497021	0,362514962
Ubox5	154,336031	0,104528777	0,135009191	0,774234526	0,438792114	0,766497562
Ammecr1l	1288,100999	-0,010385091	0,086831743	-0,119600164	0,90479989	0,973666426
Rhobtb1	1558,273837	-0,254005422	0,088737783	-2,862426942	0,004204102	0,060959475
Gabrb3	18,28628016	-0,357685128	0,359770311	-0,994204127	0,3201235	0,677494232
1110017D15Rik	0,74928523	1,196135257	2,347712887	0,509489582	0,610409099	NA
Mfsd4b5	5,791545094	-0,496735072	0,580423392	-0,855815047	0,392100091	0,734686211
Aldh3a1	0,612123332	-0,668617678	2,334418034	-0,286417286	0,774558537	NA
Exosc1	282,5415453	-0,01385884	0,114188602	-0,121367982	0,903399576	0,973489035
Etv2	0,219943115	0,059593471	3,352475198	0,017775962	0,985817581	NA
Fzd6	107,3810869	0,256748773	0,283557367	0,905456188	0,36522376	0,713632922

Supplementary Table S1: *Serpina1* KO vs. wildtype all DEGs

Agt	17210,47498	0,234285116	0,100421035	2,333028282	0,019646661	0,156064296
Folr1	1,600568779	-0,078106034	1,490861516	-0,052389865	0,958218049	NA
Anxa6	3876,471256	0,048897712	0,089421841	0,546820684	0,584501931	0,847068435
Ager	8,467599218	0,258431825	0,529788947	0,487801465	0,625690474	0,864942192
Speg	22,10749176	-0,089362832	0,33694167	-0,265217514	0,790841904	0,935974275
Map2k3os	26,52561063	-0,522237667	0,307478465	-1,698452822	0,08942233	0,370643577
Polg2	145,5477898	0,138984825	0,171383109	0,810959876	0,417388713	0,752195715
Airn	11,3146477	0,119776755	0,457928254	0,261562273	0,793658933	0,936746676
Hgd	19429,79105	0,213488703	0,123200456	1,73285644	0,083121188	0,356855223
Dctd	3,301143413	-0,310322894	0,88685811	-0,349912675	0,726404234	NA
Ppargc1a	206,1325464	0,264879792	0,259121105	1,022223921	0,306674931	0,666006686
Serp2	1,113840089	-2,983896808	1,483621305	-2,01122537	0,044301657	NA
Asb7	197,6388008	-0,200163633	0,148303879	-1,349685753	0,177116804	0,520079413
Naaladl2	129,7740782	-0,242227113	0,257686594	-0,940006653	0,347214148	0,69918752
Gm6787	5,223460188	0,273098885	0,656142726	0,41621872	0,677249963	NA
Gm10319	1711,914012	-0,005388859	0,17808107	-0,030260707	0,975859134	0,993395322
6330549D23Rik	14,00689571	-0,956412331	0,433633685	-2,205576652	0,027413655	0,190881853
Maff	42,93052274	-1,078016631	0,332442489	-3,242716163	0,001183961	0,026877772
Tex35	0,200683542	0,059593471	3,352475198	0,017775962	0,985817581	NA
Rab5c	2294,730052	-0,049944776	0,07516192	-0,664495753	0,506373066	0,806213623
Etohd2	29,24142402	0,117250081	0,338423574	0,346459555	0,728997375	0,911594858
Cdk3-ps	3,455770869	-0,467427054	0,860370912	-0,543285515	0,586933236	NA
H2-K2	259,9936623	0,088294059	0,261712008	0,337371067	0,735837193	0,914312906
Adarb1	207,4844058	-0,489739569	0,138324807	-3,540504266	0,000399363	0,012562556
Gm4759	0,884073309	0,201412704	1,491171828	0,135070084	0,892556463	NA
Atp10d	57,67160335	0,278898088	0,248694564	1,121448268	0,2620971	0,622562722
Mir6392	2,511517638	0,893866014	1,243919278	0,718588441	0,472394537	NA
Trpm3	0,200683542	0,059593471	3,352475198	0,017775962	0,985817581	NA
Triobp	424,1609382	-0,104832357	0,133697323	-0,784102142	0,432980179	0,763032854
Slc35g2	4,759399964	-0,495944628	0,733727108	-0,675925181	0,499088149	NA
Nav3	0,533058279	-0,253249591	2,424555742	-0,104451957	0,916810693	NA
Ercc6	270,9773246	-0,197388149	0,136194184	-1,449314086	0,147249889	0,47747598
Crebl2	704,9957323	-0,237136797	0,11686862	-2,029088697	0,042449257	0,243203202
Trp53inp2	4502,853325	-0,629961591	0,136085321	-4,629166376	3,67E-06	0,000371841
Zfp786	7,373524645	0,14204739	0,642136676	0,221210524	0,824928515	0,947537943
Spdye4b	0,443168919	2,196464572	3,313880474	0,662807421	0,5074539	NA
Adgrg2	14,08834801	-0,118716051	0,520069396	-0,228269635	0,819436629	0,946091978
Necab1	1384,53005	-0,037385916	0,150548791	-0,248330894	0,803878398	0,940361036
Car12	0,281209566	-1,718135424	3,30990564	-0,519088944	0,603698718	NA
Erc1	769,8980623	-0,404965729	0,120295577	-3,366422436	0,0007615	0,019767947
Sdk1	1,64506318	1,429354306	1,402734192	1,018977304	0,308213741	NA
Vps37b	445,7690068	-0,162555759	0,114239944	-1,422932766	0,154755647	0,489743509
Tigar	267,3914535	-0,269957361	0,14892622	-1,812691958	0,069879345	0,323505282
9130023H24Rik	87,38160393	0,37704921	0,216148396	1,744399758	0,081089407	0,352089732
Arhgap18	366,0730053	0,35283794	0,14967697	2,357329521	0,018406905	0,150600925
Adamts3	2,242146897	-1,08458618	1,072174647	-1,011576037	0,311740808	NA
Fam185a	197,4441171	-0,040723974	0,1362023	-0,298996229	0,764942923	0,926399306
Slc5a6	139,9449957	0,039416529	0,162333021	0,242812758	0,808150452	0,942196162
Fhad1	65,70207619	0,572720188	0,208759692	2,743442385	0,006079872	0,07769098
Hnrnpul1	1458,493729	-0,066506569	0,109732604	-0,606078477	0,544462642	0,827477215
Hap1	34,60486664	0,106490147	0,272616752	0,390622168	0,696076537	0,897381603
Rad54l	4,6518983	-0,610533878	0,897449269	-0,680299042	0,496315131	NA
Pla2g5	0,462068663	1,251763544	2,495664123	0,501575325	0,61596628	NA
Nfia	795,4689195	0,023991809	0,103656943	0,231453949	0,816962158	0,944998904
Mpl	0,172953423	1,307385949	3,350719078	0,39018071	0,696402925	NA
Pianp	14,67023379	-0,196638449	0,403014776	-0,487918708	0,625607423	0,864942192
1110065P20Rik	378,0342125	-0,328892322	0,172922341	-1,901965475	0,057175668	0,289000033
Wdr90	122,5427079	-0,230216629	0,169890642	-1,35508717	0,175389786	0,517727103
Casz1	8,745254288	0,073542971	0,650698567	0,113021566	0,910013458	0,9747592
Wee1	88,23241932	-0,787615738	0,23377057	-3,3691826	0,000753915	0,019647339
Ltbr	1851,820556	0,12334654	0,084246916	1,464107484	0,143164583	0,470269006
Pex5l	0,739768161	1,177281897	1,776958776	0,662526285	0,507633995	NA
Unc13b	248,3327293	-0,41660531	0,274575295	-1,517271644	0,129198114	0,446717571
Ubxn8	668,7232761	0,163733376	0,12256976	1,335838272	0,18160214	0,525961659
Rbm46os	0,475875753	1,260339921	2,4786841	0,508471378	0,611122806	NA
Tmem64	981,2575114	-0,263418329	0,16675225	-1,5796988	0,114175861	0,41832777

Wdr47	49,01078854	-0,27653283	0,253279761	-1,091807844	0,27491757	0,636648709
Lgals8	3397,439879	-0,04107665	0,103107902	-0,39838508	0,690346354	0,894644908
C1rl	4174,819929	-0,001773322	0,110189469	-0,016093386	0,98715989	0,996231603
Slc25a29	37,31749987	0,456560808	0,277106469	1,647600687	0,099434639	0,392688228
Lcmt2	214,9961058	0,232728038	0,160356129	1,451319881	0,146690808	0,476487281
9830107B12Rik	0,113662148	0,780932884	3,352475198	0,232942181	0,815806304	NA
Eme1	5,652693163	0,357355653	0,818506104	0,436594977	0,662405106	0,883360984
Sema3b	16,28939829	0,589815008	0,553838729	1,064958042	0,286894918	0,64832626
Mpv17	938,4770611	-0,21626765	0,075550283	-2,862565709	0,004202261	0,060959475
Preb	2661,727351	-0,388135303	0,107061652	-3,625343866	0,000288577	0,009742396
Ccdc130	134,2207813	0,281615605	0,134122982	2,099681952	0,035756828	0,221106863
Ate1	530,3111892	-0,15284215	0,104771814	-1,458809815	0,144617461	0,472725517
Gpatch4	348,6128576	-0,022255114	0,174087058	-0,127838992	0,898276392	0,971699969
Med27	152,9448906	-0,206741569	0,145064563	-1,425169352	0,154108253	0,489157561
Ppp1r18	99,80126693	-0,050889364	0,229024001	-0,22220101	0,824157408	0,947472731
Hps3	159,2400955	-0,051373998	0,142552731	-0,360385929	0,718558547	0,90815359
9930012K11Rik	9,778965957	-0,279433009	0,49473258	-0,564816266	0,572198742	0,841794408
Zfp607b	11,45805441	-0,040248849	0,485969698	-0,082821725	0,933993294	0,981801883
Amigo2	110,7136315	0,542276293	0,258217411	2,100076413	0,03572212	0,220994456
Egfl7	415,9255569	0,073557459	0,223021636	0,329822076	0,741534406	0,916735109
Gm24148	0,696448399	2,857899309	2,6329458	1,085437957	0,277727729	NA
Apol7c	4,619070057	0,066660187	0,772419879	0,086300455	0,931227577	NA
Trmt10a	113,7530844	-0,02680927	0,179676509	-0,149208545	0,881389078	0,965734294
Ets1	317,4906233	0,028565075	0,227845561	0,12537034	0,900230368	0,972376014
Taf6l	83,95961941	0,018812831	0,166499273	0,11299047	0,91003811	0,9747592
Psrc1	3,035327956	0,239530813	1,158741529	0,206716344	0,836231392	NA
Pias2	689,8254648	-0,195065673	0,113097324	-1,724759405	0,08457084	0,360024318
Cdv3	1863,882227	-0,05675516	0,089573434	-0,633615983	0,526331468	0,817627711
Pifo	0,12663974	0,780932884	3,352475198	0,232942181	0,815806304	NA
Jak3	234,0916865	0,287952128	0,172206131	1,672136328	0,094497443	0,382250018
Rpgrip1l	66,77905169	-0,225047141	0,179124433	-1,25637322	0,208980686	0,561671273
Phka2	558,3769157	0,212325699	0,133503841	1,590408915	0,111742662	0,413687165
Nat1	47,44677068	0,257157334	0,323166479	0,7957426	0,426181661	0,758368511
Afap1l2	45,82805867	-0,019053788	0,258666194	-0,073661685	0,941279587	0,983128178
Sep-07	1181,639636	-0,000741732	0,141027384	-0,005259486	0,995803557	0,998991922
Fam160a1	174,2480682	-0,112344901	0,147300834	-0,762690186	0,445648185	0,771326444
D930015E06Rik	648,1332629	-0,080694272	0,176282765	-0,457754744	0,647128653	0,876230372
4932438A13Rik	1610,839507	-0,132457857	0,115791168	-1,143937485	0,252649564	0,612043137
Ap5z1	284,1280018	-0,082441849	0,149485376	-0,551504441	0,581287923	0,845803031
Otub2	69,41005901	0,042281176	0,190878266	0,22150859	0,824696449	0,947537943
Smox	67,79521814	0,117712073	0,195022234	0,603582837	0,546121026	0,828069865
Zfp513	291,5590143	0,019322415	0,129620657	0,149068949	0,881499228	0,965734294
Cacna1g	3,040040937	-0,165280599	0,802537972	-0,205947387	0,836832009	NA
Snord64	0,583799806	0,396879134	1,907665652	0,208044389	0,835194309	NA
Ptgr2	1755,680093	0,002873406	0,079714129	0,036046379	0,971245378	0,992121884
H2afz	773,4948546	-0,314106114	0,101060044	-3,108113771	0,001882856	0,036692532
Usp43	3,953702786	0,243955402	0,820945923	0,29716379	0,766341465	NA
Ahsa2	1126,312433	-0,563580151	0,145527378	-3,87267439	0,000107648	0,004797136
Cdkn2aip	278,6237062	0,131370149	0,126494839	1,038541569	0,299017993	0,659904651
Lgalsl	841,6046212	0,192335084	0,118529071	1,622682791	0,104657232	0,402447734
Ugt1a5	1066,974701	1,688810281	0,408969736	4,129426044	3,64E-05	0,002123102
Evl	109,3652652	0,472622997	0,24518402	1,927625612	0,053901711	0,280284703
Evi5	3143,738797	-0,024942398	0,106739351	-0,233675749	0,815236716	0,944199918
Dsg1a	0,172953423	1,307385949	3,350719078	0,39018071	0,696402925	NA
Ccl20	0,113662148	0,780932884	3,352475198	0,232942181	0,815806304	NA
Fnbp1	271,1134079	-0,127818976	0,124270993	-1,028550372	0,303691009	0,66325429
Copb2	3590,775641	-0,197201134	0,095047536	-2,074763252	0,038008482	0,22878676
Sgsh	199,3662124	0,062433407	0,154156228	0,405000875	0,685476868	0,893161227
Socs6	207,6522071	-0,033217933	0,136323445	-0,24366999	0,807486417	0,942032857
Ppm1l	181,7758725	0,024081572	0,14706197	0,163751184	0,869927022	0,961775826
Lrrc4c	28,94092902	0,353589779	0,302376995	1,169367327	0,242255668	0,600476091
Harbi1	339,1751893	-0,022021203	0,102415646	-0,21501796	0,829753316	0,948328541
Zfp385b	358,4104672	0,109698352	0,154637165	0,709391896	0,478081315	0,790436446
Zfp438	49,28416996	0,122597162	0,234186793	0,523501604	0,600625235	0,854422974
Cadm2	0,180429807	0,059593471	3,352475198	0,017775962	0,985817581	NA
Mief1	559,279899	-0,147946337	0,092611545	-1,597493456	0,110155755	0,410672697

Supplementary Table S1: *Serpina1* KO vs. wildtype all DEGs

Tnpo1	736,7950886	-0,206843011	0,147051122	-1,406606134	0,159544213	0,495312554
Aldh8a1	9120,952603	0,123676154	0,064016715	1,931935342	0,053367487	0,278808102
Aebp2	432,175156	-0,062612227	0,127757538	-0,490086359	0,62407279	0,863867171
Plscr4	44,57618383	-0,241319706	0,250366511	-0,963865757	0,335113234	0,691456742
Sik2	175,4440145	0,023650483	0,17353293	0,136288158	0,891593485	0,969104575
Rnf214	570,5502103	-0,148914795	0,124824338	-1,192994866	0,232871384	0,589632107
Gmpr2	352,8263109	-0,088161712	0,130596527	-0,675069345	0,499631709	0,802860694
Zfp592	801,6751668	0,01265565	0,100369778	0,126090248	0,899660487	0,972141416
Slc25a40	82,33016357	-0,035875508	0,163667547	-0,219197445	0,826496246	0,947537943
Prrg4	5,855373911	0,195767634	0,69336311	0,282345038	0,777678954	0,93069465
Dpy19l3	225,8904008	-1,388123183	0,479082389	-2,89746235	0,003761949	0,057281885
Zer1	648,4519864	0,044200915	0,109341676	0,404245813	0,686031968	0,893161227
Slc6a1	0,888396769	0,73303446	1,594124379	0,459835173	0,645634534	NA
Coq4	193,6209819	-0,109917872	0,124707747	-0,881403719	0,378099346	0,723903192
Radil	0,206965523	0,059593471	3,352475198	0,017775962	0,985817581	NA
C130074G19Rik	5615,688179	0,047127004	0,106720197	0,441594052	0,658782989	0,881654058
Yod1	76,73595647	0,07062648	0,231233806	0,305433194	0,760036244	0,924925298
Grsf1	2208,006774	-0,016505916	0,075846946	-0,217621362	0,827724139	0,947580409
Ttc16	3,344418308	0,032282421	0,791022595	0,040810998	0,967446572	NA
Spin4	6,516755954	-0,004987914	0,79185904	-0,006298992	0,994974165	0,998634355
Zfp14	35,34113943	0,051145161	0,255068071	0,200515732	0,841077256	0,951786586
Pcid2	273,3320594	-0,022300432	0,137370127	-0,162338297	0,871039458	0,961834188
Ablim1	1290,04569	-0,291986457	0,100293007	-2,911334144	0,003598889	0,05588101
Cnot6l	847,2727981	0,043127725	0,097566751	0,442033011	0,658465318	0,881448167
Pigv	198,9661888	0,033459747	0,179592803	0,186308953	0,852202487	0,95595696
Cep120	317,2487001	0,096436363	0,124291173	0,775890687	0,437813532	0,766215379
Enpp5	653,9633832	-0,12506471	0,150442495	-0,831312392	0,405797176	0,745391028
Camta1	76,22828654	-0,048647698	0,224416342	-0,216774312	0,828384232	0,94762353
Gprc5d	0,722919456	-1,292784801	1,713873857	-0,754305689	0,450665687	NA
Gpm6b	12,9202953	0,026275807	0,457981962	0,057373017	0,954248057	0,986616875
Ahnak	379,7657191	-0,119674306	0,196780445	-0,608161581	0,543080313	0,826741141
Gfap	5,283588156	-0,191685743	0,629911768	-0,304305702	0,760895002	NA
Gem	41,68929106	0,489482022	0,24223363	2,020702173	0,043310605	0,24545124
Il18bp	392,9669169	0,593896263	0,188886005	3,14420469	0,001665389	0,033991735
Gpd2	553,2203755	-0,12266654	0,274651418	-0,446626276	0,65514492	0,879380767
Ifngr1	496,4497655	0,150105672	0,134847022	1,113155264	0,265641752	0,626006345
Vil1	7,730112821	0,377304758	0,610040766	0,618491057	0,536251691	0,823283057
Gusb	792,7780186	-0,168028091	0,119720611	-1,403501781	0,160467261	0,497178481
Magi1	811,408745	0,089469911	0,100304062	0,891986919	0,372399944	0,719447489
Adamts10	182,5500589	-0,031416642	0,213464604	-0,147174947	0,882993934	0,966395158
Car2	211,9544883	0,15003641	0,254092499	0,590479491	0,554869231	0,832552947
Bin1	333,6617956	-0,176371336	0,10103046	-1,745724375	0,080858857	0,351670169
Mir6929	0,142634598	-0,517475177	3,352475198	-0,154356154	0,877328924	NA
Ptprc	195,5748408	0,24792584	0,258683109	0,958415263	0,337853402	0,693194655
Mir7037	0,093303375	-0,517475177	3,352475198	-0,154356154	0,877328924	NA
Lct	14,1106549	5,359252862	3,18897969	1,680554091	0,09284956	0,378677779
Nfyc	491,3394214	-0,069922643	0,100895073	-0,69302336	0,488294892	0,796400974
Atp6v1b2	1023,05758	0,137784711	0,112762049	1,221906772	0,221742884	0,576410775
Atp6v1e1	1735,934372	0,064418283	0,09069262	0,710292447	0,477522803	0,790092068
Gzmm	3,466840601	-0,164436647	0,774767	-0,212240128	0,831919702	NA
Trim24	409,3294265	0,789490714	0,29772912	2,651708084	0,008008575	0,09174519
Hip1r	668,5565989	-0,381546929	0,185422387	-2,057717704	0,03961724	0,234044578
Alg1	438,2429504	-0,14883055	0,103908551	-1,43232244	0,152051564	0,485381413
Lgi3	3,722635438	0,113289706	0,792782045	0,142901453	0,886368011	NA
Fcamr	2,211028875	-0,538938784	1,081101327	-0,49850904	0,618125299	NA
Eif4a1	5585,229601	-0,119055261	0,141735243	-0,839983469	0,400917655	0,741644961
Dennd6a	423,8220024	-0,143480514	0,131843292	-1,08826556	0,27647789	0,637501364
Pcbp3	29,97495822	-0,443152498	0,314923622	-1,407174526	0,159375643	0,495312554
Tssc4	342,6513856	-0,353103594	0,130724569	-2,701126473	0,006910505	0,084138787
Clec2i	8,027994992	0,749061161	0,512223737	1,462371044	0,14363956	0,470584709
Gm12709	0,086476712	0,780932884	3,352475198	0,232942181	0,815806304	NA
Zfp811	2,181197282	-0,325471221	0,999359053	-0,325679965	0,744666504	NA
Camkk2	323,3126103	-0,29352847	0,13840124	-2,120851451	0,033934305	0,215416775
Ado	335,6127873	-0,009419479	0,135051418	-0,069747352	0,944394752	0,984069637
Gm5617	277,7616249	0,238233475	0,14383078	1,656345572	0,097651857	0,389224129
1700123J17Rik	0,401002453	2,064678166	3,036583623	0,679934565	0,496545894	NA

Supplementary Table S1: *Serpina1* KO vs. wildtype all DEGs

Tmem132b	0,227324296	1,389394708	3,349408814	0,414817893	0,67827522	NA
Ddx19b	412,7120527	-0,292543859	0,165751117	-1,764958593	0,077570725	0,345105832
Dcun1d4	599,8530979	-0,174440917	0,17365606	-1,004519604	0,315128227	0,673856248
Mocs1	2587,158237	-0,007642145	0,126219048	-0,060546688	0,951720233	0,986015793
Rasgef1a	0,086476712	0,780932884	3,352475198	0,232942181	0,815806304	NA
Ankrd2	9,291889448	0,287116911	0,571606795	0,502297931	0,615457964	0,859955845
Dnm2	1589,104294	-0,224500719	0,09820459	-2,286051181	0,022251265	0,167215939
Raet1d	54,09698338	0,850958949	0,493095438	1,725748981	0,084392582	0,359726093
Lpar2	24,64634252	-0,151840896	0,324496413	-0,467927811	0,639836214	0,871049783
Ube2j1	949,5267805	-0,208105647	0,119719581	-1,738275768	0,082162234	0,354079608
B3galnt1	17,97068289	0,83814285	0,36108716	2,321164923	0,020277945	0,159093803
B3galt2	0,113662148	0,780932884	3,352475198	0,232942181	0,815806304	NA
Rfc2	454,1221503	-0,180564285	0,111465866	-1,619906541	0,105252354	0,403011718
Tbx20	43,92142831	-0,037780978	0,279318292	-0,135261381	0,892405218	0,969104575
Gfra4	0,122496332	0,780932884	3,352475198	0,232942181	0,815806304	NA
Fgf21	22,78387623	0,067668429	0,439628297	0,153921914	0,877671305	0,965046146
Rnf14	1366,251995	0,171194256	0,108750861	1,574187586	0,115444089	0,420651954
Fxyd1	6142,320514	0,115557589	0,082825308	1,395196605	0,16295656	0,49993802
Plcd1	12,13939711	0,422055992	0,443484617	0,951681244	0,341258665	0,695152687
Jph1	28,98345954	-0,171977071	0,290761282	-0,591471703	0,554204409	0,832018326
Sap30	76,24674533	0,192589181	0,193547905	0,995046579	0,31971361	0,677390791
Donson	50,91208397	-0,206479958	0,249992325	-0,825945188	0,408835186	0,746898413
Scaf11	1357,335079	0,000216087	0,09915162	0,002179359	0,998261124	0,999803624
Dbndd1	0,295445946	1,389394708	3,349408814	0,414817893	0,67827522	NA
Klhl35	0,261385121	1,389394708	3,349408814	0,414817893	0,67827522	NA
Zfp661	107,3761502	-0,10759096	0,165029021	-0,651951755	0,514432296	0,811881167
Mfsd8	291,6969632	-0,113385347	0,12302868	-0,921617189	0,356728289	0,707120636
Atxn7l1	299,6883449	-0,388098749	0,205028598	-1,892900569	0,058371094	0,291803151
Thumpd2	53,7563288	0,348791808	0,216423356	1,611618146	0,10704506	0,405981674
Msantd3	6,713053092	0,207003048	0,625400167	0,330992953	0,740649809	0,916181159
Dhx36	853,9366543	-0,058336625	0,10546039	-0,553161483	0,580152839	0,845309347
Tmem163	4,907370688	0,228680948	0,766722775	0,29825767	0,765506509	NA
Egln3	462,1609817	-0,179949618	0,151305422	-1,189313749	0,234316227	0,591595413
Cenpn	16,77980262	-0,409801512	0,365747987	-1,120447756	0,262523004	0,622562722
2610020H08Rik	48,72198919	0,019582829	0,242962623	0,080600173	0,935759928	0,982114499
Zfp157	123,41587	0,090220632	0,17832585	0,505931318	0,612904866	0,859082108
Rubie	0,259430135	1,627812584	3,345388605	0,486584005	0,626553161	NA
Tpd52l2	747,3286083	0,055059516	0,081866199	0,67255493	0,501230483	0,803641654
Ssh2	474,7789231	-0,488515429	0,11313172	-4,318111944	1,57E-05	0,001175349
Large2	14,10085142	-0,232126194	0,395215981	-0,587340101	0,556975314	0,833822555
Aurka	25,9223046	-0,453648469	0,384362594	-1,180261755	0,237896124	0,595140959
Pcsk6	2417,363875	-0,085894167	0,173382583	-0,495402507	0,620316025	0,862327643
Rad51c	33,93428066	-1,452508508	0,421692624	-3,444472167	0,000572176	0,016172124
Nfatc2	7,808376745	0,332612463	0,686514847	0,484494202	0,628035178	0,865852703
Depdc5	348,3711895	-0,087414914	0,144555519	-0,60471516	0,545368274	0,827491596
Slc25a19	292,2988841	-0,022192964	0,106690798	-0,208011981	0,835219613	0,951141594
Irf5	470,0825157	0,107815793	0,161599676	0,667178276	0,504658266	0,805075709
Snord72	1,438576687	0,298662839	1,386069787	0,215474604	0,82939731	NA
Brwd3	125,1570436	-0,242859791	0,153240299	-1,584829798	0,11300501	0,41624842
Kng1	68781,05526	0,432503932	0,093278868	4,636676483	3,54E-06	0,000361326
Fbxo9	1350,917367	-0,027334434	0,105927992	-0,258047317	0,796370389	0,937785232
Slc25a25	3774,431765	-0,748615098	0,250171002	-2,99241356	0,00276781	0,046839059
Ska1	2,249963148	-1,053638259	0,988605449	-1,065782371	0,286522032	NA
Cpsf4l	96,21141981	1,392860593	0,45032503	3,093011716	0,001981363	0,038104827
Zfyve27	340,9631742	-0,021943778	0,117659253	-0,186502781	0,852050498	0,95595696
Pqlc3	5,890468669	0,335186616	0,612907773	0,546879369	0,584461611	0,847068435
Dis3l	547,7932471	-0,018908589	0,12323155	-0,15343951	0,878051689	0,965046146
Gpr174	4,108357363	0,584282091	0,774292094	0,754601649	0,450488034	NA
Nr1i3	2593,89719	0,452478371	0,220142752	2,055386183	0,039841719	0,234955421
Bcl11a	4,763185194	0,414198973	0,688195705	0,601862188	0,547265876	NA
Anp32e	575,0466218	-0,396516778	0,123605503	-3,207921715	0,001336979	0,028782722
Sep-06	42,4630926	0,424739938	0,355128279	1,19601835	0,231689398	0,58867957
Trip10	286,4856804	-0,274639663	0,167123944	-1,643329236	0,100314832	0,393294268
Nmt2	479,0228329	-0,294832157	0,091101985	-3,236286846	0,001210956	0,027346748
Stip1	2731,450823	-0,544651388	0,156220177	-3,486434323	0,000489506	0,014414537
Bloc1s1	1381,997538	0,123059259	0,098028428	1,255342574	0,209354426	0,561873081

Supplementary Table S1: *Serpina1* KO vs. wildtype all DEGs

Mixl1	0,12663974	0,780932884	3,352475198	0,232942181	0,815806304	NA
Slamf1	1,355288384	0,185939047	1,34974954	0,137758185	0,890431532	NA
Azi2	923,539379	0,048764272	0,090033386	0,541624325	0,588077331	0,848706754
Dbf4	67,45400739	-0,290227356	0,223934772	-1,296035242	0,194963343	0,544089091
Rps11	3725,114223	-0,012405539	0,117179313	-0,105867998	0,915687085	0,976559041
Podxl	124,1998848	-0,256358497	0,255721424	-1,002491274	0,316106379	0,67419376
Prim1	88,31030756	-0,219359142	0,208814623	-1,050497032	0,293489655	0,654598464
Dapk2	250,8625635	-0,896449441	0,23872196	-3,755203089	0,000173201	0,006830454
Nr6a1	256,2352216	-0,262936744	0,195524075	-1,344779381	0,178696491	0,521301605
Sh3bp1	52,3779729	0,552893949	0,224537105	2,462372309	0,013802131	0,1275245
Col1a1	242,6172505	-0,443734508	0,558077581	-0,795112584	0,426548015	0,758848669
Ppt1	803,1980219	0,165569606	0,084629677	1,956401253	0,050417905	0,269938714
Pigm	209,7928888	-0,18163366	0,129148914	-1,406389375	0,159608534	0,495312554
Ube2t	11,39536853	-0,080673504	0,449964823	-0,179288469	0,857711201	0,958260148
Mov10	624,3812403	0,27334753	0,105359456	2,594428066	0,009474844	0,101415126
Lrrc28	508,169066	-0,133992986	0,144658379	-0,92627186	0,354304716	0,704551502
Pnpla2	1345,618527	0,254398889	0,099128558	2,566353171	0,010277413	0,106602332
1600029I14Rik	0,416483249	-0,030920724	2,232468219	-0,013850466	0,988949281	NA
Pex16	1660,946908	0,278150363	0,127463809	2,182190898	0,029095447	0,196651683
4930523C07Rik	202,824221	0,045734882	0,218985456	0,208848947	0,834566159	0,950858008
Fam172a	210,5954245	0,246590028	0,129875668	1,898662247	0,057608898	0,290084129
Rabggtb	1097,514159	0,02080625	0,109094408	0,190717843	0,848746659	0,954095425
Myeov2	597,9305448	0,124461869	0,145355875	0,856256197	0,391856084	0,734537856
1810008I18Rik	2563,765413	0,524603813	0,190136179	2,759095177	0,005796165	0,075086174
Rab26os	29,37936219	-0,215843612	0,317970194	-0,678817121	0,497253744	0,801227589
4732491K20Rik	29,58920652	0,307226866	0,313240386	0,980802219	0,326690284	0,684457358
1700048M11Rik	0,12663974	0,780932884	3,352475198	0,232942181	0,815806304	NA
1700097N02Rik	0,689058872	-2,144899282	2,183090023	-0,982506108	0,325850575	NA
Gm16551	363,7051917	-0,731603347	0,308191421	-2,373860196	0,017603214	0,146719059
Gm15941	0,093953095	-0,517475177	3,352475198	-0,154356154	0,877328924	NA
Gm10433	26,06859402	0,422130588	0,296352457	1,424420746	0,154324712	0,489157561
8430429K09Rik	18,34241651	0,478824925	0,363043003	1,318920681	0,187195631	0,533703441
5430437J10Rik	0,380568667	1,000985803	2,588151393	0,386757052	0,698936075	NA
Med9os	0,274277864	-1,699264605	2,746395376	-0,61872541	0,536097268	NA
Sp3os	16,93017349	1,277142774	0,410085216	3,114335077	0,0018436	0,036299097
1700101I11Rik	4,747040507	0,552778505	0,890533017	0,620727693	0,534778806	NA
Gm15545	30,34772745	0,347105815	0,289823352	1,197646129	0,23105481	0,587927627
Toporsos	122,1423942	0,339449097	0,201486609	1,684722862	0,092042053	0,376533113
AI847159	0,316132704	-0,672311707	2,716600932	-0,24748269	0,804534686	NA
A830082K12Rik	4,577890388	0,4021424	0,655622171	0,61337523	0,539628261	NA
St18	0,172953423	1,307385949	3,350719078	0,39018071	0,696402925	NA
4833417C18Rik	7,729425008	0,14528597	0,523265908	0,277652276	0,781279299	0,932196631
4932702P03Rik	1,634854341	-0,109494234	2,032985919	-0,053858825	0,957047642	NA
2810468N07Rik	1,895295488	0,728053152	1,12473656	0,647309937	0,517431372	NA
Smc2os	0,614731052	-0,854346909	2,062172029	-0,414294684	0,678658307	NA
Ube2j2	38,84595956	0,149628874	0,342906756	0,436354406	0,662579614	0,883417459
Klrb1a	0,379222292	-2,088965346	3,059126193	-0,682863411	0,494693156	NA
Ntf3	11,74774038	-0,075191847	0,495346606	-0,151796432	0,879347497	0,965347075
Ociad1	2710,09881	0,020545633	0,077884215	0,263797134	0,791936243	0,936507892
Crem	562,4772786	-0,064273593	0,179715985	-0,357639821	0,720612874	0,909027506
Gpr35	16,62829002	0,670548779	0,406655288	1,64893658	0,099160628	0,392602307
Ankrd26	105,7902745	-0,245510847	0,178133657	-1,378239521	0,168129349	0,507127649
Myef2	170,2809754	-0,162514517	0,141681292	-1,147042878	0,2513639	0,610832604
Pfkfb2	437,0137234	-0,302923484	0,136926495	-2,212307301	0,026945439	0,189659578
Nlrp1b	15,8501612	0,837637738	0,366838733	2,283395025	0,02240711	0,168103622
Cysltr2	3,100716502	0,423890091	0,895939613	0,473123506	0,636125067	NA
Dcdc2b	20,44969544	-0,277531915	0,345787812	-0,802607568	0,422201593	0,755522295
Sun1	343,5872696	0,204056644	0,130348982	1,565464045	0,117474142	0,425267208
Eps8	42,1997691	-0,153538612	0,266226568	-0,576721598	0,56412754	0,837355044
4930451G09Rik	14,11405155	0,094105815	0,466638778	0,201667369	0,840176774	0,951657825
Lama2	49,88226347	-0,418181157	0,274141321	-1,525421838	0,127153915	0,44395944
Dusp15	0,086476712	0,780932884	3,352475198	0,232942181	0,815806304	NA
Zyx	1033,907682	0,023451519	0,129827867	0,18063548	0,856653705	0,9578959
Prnp	909,123228	0,073272319	0,16697315	0,438826957	0,660786926	0,882666274
Dmc1	0,427463156	2,206280922	2,965530913	0,743975021	0,456891587	NA
Disp1	78,93891808	0,254468153	0,243025861	1,047082613	0,295061478	0,656024762

Mkks	739,8876207	0,104399903	0,103677924	1,006963666	0,313952235	0,672632601
Fbxo41	0,327323282	0,664081931	2,690749744	0,246801819	0,805061603	NA
Rnaseh1	154,0306373	0,132492836	0,148220512	0,893890018	0,371380739	0,71913887
Angpt1	16,3781275	0,569485265	0,469372443	1,213290796	0,225018661	0,580187941
Zfp322a	360,493585	-0,305535485	0,146832364	-2,080845643	0,037448037	0,226843138
Hist1h2ad	0,313801008	1,795331527	3,226882632	0,55636716	0,577959884	NA
Shq1	89,55182285	0,1734805	0,174346381	0,995033562	0,319719941	0,677390791
Ube2q2	501,1807708	-0,179208458	0,127526149	-1,405268327	0,159941503	0,495885425
Shprh	295,4111456	-0,041423158	0,150218684	-0,27575237	0,782738269	0,93249269
Zfp12	206,8558483	-0,149297071	0,118613458	-1,25868577	0,208143853	0,56023257
Tm6sf1	40,91090887	0,107427744	0,266951113	0,402424786	0,687371432	0,893386271
Aldh16a1	1456,007356	0,187774401	0,068914048	2,724762311	0,006434779	0,080173872
Cth	13821,16279	0,640512398	0,229310377	2,793211565	0,005218755	0,070190676
Tbc1d12	196,3302514	0,017219489	0,119297494	0,144340747	0,885231404	0,967437149
Lrrc3b	0,187906191	-1,241948083	3,339949325	-0,371846385	0,710007226	NA
Enox2	316,8175367	0,189047069	0,131890609	1,433362613	0,151754237	0,48501133
3830406C13Rik	386,0829834	0,310617094	0,134996161	2,300932791	0,02139543	0,163367723
Ido2	2650,892129	0,031247365	0,130183162	0,240026165	0,810309973	0,942417935
Oit1	0,738614865	2,947927908	3,21283393	0,917547552	0,358855787	NA
Osgin2	40,13415112	-0,078049377	0,238161938	-0,327715577	0,743126725	0,917092327
Clptm1l	2160,197593	-0,188339403	0,074799057	-2,517938201	0,011804403	0,116136542
Vps25	2022,762009	-0,008682901	0,076606596	-0,113344034	0,909757808	0,9747592
Lmbrd1	1319,880529	-0,078398475	0,091770746	-0,854286117	0,392946477	0,735241631
Capns2	0,748129716	0,377934924	1,627943365	0,232154836	0,816417755	NA
Mir6939	0,086476712	0,780932884	3,352475198	0,232942181	0,815806304	NA
Vipas39	317,3859354	0,218435219	0,095890997	2,277953365	0,022729356	0,16947505
Dtx2	120,4057441	-0,178030205	0,158383372	-1,124046057	0,260993487	0,621964694
Sipa1	307,8914026	-0,308767382	0,187721889	-1,644812882	0,100008405	0,392993389
Iglon5	9,292149761	-0,512216672	0,496361268	-1,031943274	0,302098692	0,662128551
Rap2b	60,31749782	0,26690345	0,219701244	1,214847243	0,224424359	0,579668007
Atox1	2306,188553	0,249408115	0,122371881	2,038116218	0,041538312	0,240737274
Numb	936,7390881	-0,164978877	0,094998762	-1,73664239	0,082450312	0,354886743
Nudc	1524,184234	-0,254608064	0,093473761	-2,7238453	0,006452672	0,080321952
Neurod6	0,12663974	0,780932884	3,352475198	0,232942181	0,815806304	NA
Enpp2	1604,277538	-0,303500925	0,13847958	-2,191665549	0,028403665	0,194135275
Mapkapk2	1394,917643	0,082024725	0,076436478	1,073109677	0,28322191	0,644666109
Lig3	680,7267252	-0,130859831	0,116175458	-1,126398236	0,259996993	0,621695547
Olfr56	0,899323898	1,598934457	1,989819535	0,803557523	0,421652563	NA
Piga	123,1481867	-0,672817413	0,217410605	-3,094685348	0,001970218	0,038038569
Atf2	603,1686029	0,03368522	0,131591263	0,255983714	0,797963413	0,938325224
Asgr1	11350,73146	0,279992131	0,096425939	2,903701371	0,003687797	0,05655415
Lhx9	0,786856153	0,657808295	2,268715205	0,289947497	0,771856404	NA
Arsa	1202,153877	-0,099952959	0,175656044	-0,569026589	0,569338096	0,840859231
Arsb	916,5946181	-0,190260178	0,107292111	-1,773291414	0,076180431	0,34176382
Nfyb	798,2072628	-0,190722193	0,101334547	-1,882104354	0,059821848	0,2959149
Ccng1	729,5563646	-0,095835665	0,120311575	-0,796562297	0,425705282	0,758328303
Ccna2	28,69545485	-1,116163606	0,556497138	-2,005695142	0,044888784	0,251200566
Lhx2	81,64075167	0,236997721	0,204728138	1,157621632	0,247018478	0,606485359
Eif2d	820,7253704	0,081377044	0,103137469	0,789015323	0,430103051	0,761019227
Rnf148	0,469488733	0,01358032	2,949191015	0,004604761	0,996325945	NA
Actc1	1,399733448	0,379129192	1,612007277	0,235190745	0,814060689	NA
Zfp946	357,3528444	0,182962304	0,167035153	1,09535209	0,273362415	0,634872238
Hdac6	490,3078509	0,003586769	0,097075153	0,036948373	0,97052617	0,992007633
Large1	137,6618009	0,104820664	0,167408593	0,626136698	0,531225284	0,819884984
Ap3b1	1200,374884	0,005380148	0,101322406	0,053099292	0,957652796	0,98781716
Hdac3	1000,717247	0,184429829	0,095608048	1,929019914	0,05372839	0,280036978
Tnfrsf11a	14,53813415	0,067366495	0,454376666	0,148261343	0,882136522	0,96610823
Mpg	208,2968892	0,124405475	0,111828304	1,112468581	0,265936732	0,626044756
Runx1t1	2,58355929	-0,356146615	0,950518492	-0,374686677	0,707893502	NA
Mpeg1	1499,094852	0,653447458	0,182974227	3,57125409	0,000355276	0,011445024
Ceacam1	4823,298685	0,042406026	0,096063734	0,441436367	0,658897119	0,881654058
Steap2	218,7770349	0,648506589	0,222317907	2,917023636	0,00353389	0,055321516
Slc9a5	11,14044057	-0,543222653	0,439091145	-1,237152375	0,216030518	0,569610871
Cys1	121,3900877	-0,883129046	0,290873233	-3,036130339	0,002396358	0,042715871
Rnf44	1211,520725	-0,107233162	0,09196356	-1,166039698	0,243598395	0,602221548
Zfp444	383,4733229	0,005812617	0,170633367	0,034064949	0,972825359	0,992650147

Fam98c	231,8337142	-0,158826914	0,169595762	-0,936502851	0,349014346	0,700954068
Wdfy4	65,77594888	0,154106392	0,235892999	0,653289386	0,513569734	0,81119019
Arhgef40	219,7659984	-0,155280958	0,135169299	-1,148788666	0,250643134	0,609910458
Lrrc18	8,796160259	1,24569609	0,70470705	1,767679336	0,077114526	0,343533524
Ythdf3	1282,072915	-0,109941717	0,152481427	-0,721017108	0,470898989	0,785164453
Cdk5r1	11,34422308	-0,887973397	0,432950213	-2,050982699	0,040268632	0,236119007
Acvrl1	67,25044917	0,001143426	0,294364135	0,003884394	0,99690071	0,999481197
Zfp82	7,674536969	-0,135706704	0,489833219	-0,277046755	0,781744205	0,932196631
Fen1	108,742228	-0,636164491	0,19512419	-3,260305617	0,001112922	0,025697163
Ncaph2	1061,297839	-0,042305801	0,083284221	-0,50796899	0,61147509	0,858645358
Nrep	470,5146476	-0,317459901	0,279113824	-1,137385086	0,255377339	0,615826054
Eef2k	351,597756	-0,577842947	0,144385168	-4,002093532	6,28E-05	0,003191504
Fsbp	3,229518015	-0,301029158	0,829362467	-0,36296453	0,716631381	NA
Tmem54	1,601073626	-0,247846381	1,334234041	-0,1857593	0,852633523	NA
Foxj3	244,0358428	0,016586233	0,114663982	0,144650769	0,884986611	0,967327774
Slc25a14	52,16005262	0,044849027	0,265648553	0,168828426	0,865931599	0,961775826
Akt1s1	1030,720992	-0,070773241	0,107727683	-0,656964293	0,511203876	0,80946164
Nsmce1	249,085184	0,1821502	0,147508485	1,234845576	0,216887982	0,570555917
Ikbkap	271,672811	0,016099962	0,109817021	0,146607165	0,883442098	0,966395158
Prkrip1	139,1235906	-0,178155141	0,175496681	-1,015148202	0,310035187	0,669180355
AI846148	64,04506974	0,004542117	0,180234904	0,025201095	0,979894563	0,994664228
Ap2b1	1356,450808	-0,134818488	0,098660531	-1,36648857	0,171785666	0,512405749
Rnf149	1800,046673	0,195493517	0,082624142	2,366058043	0,017978626	0,1487353
Adcy7	91,99565961	0,314419414	0,289357537	1,086612145	0,277208256	0,638192109
Espnl	4,195568487	-1,229327488	0,710676272	-1,729799539	0,083666097	NA
Prok1	19,2868139	-0,450205996	0,619208238	-0,727067193	0,467184795	0,782586618
Map6	0,73102918	-0,424136863	1,618262083	-0,26209405	0,793248934	NA
Rab11fip1	242,0761904	-0,071547078	0,12081089	-0,592223744	0,553700772	0,831920164
Clasp2	185,3482837	0,223172172	0,190900251	1,169051225	0,242382994	0,600633595
Cps1	107196,9122	0,277411826	0,15257854	1,818157568	0,069040053	0,321433335
Fancm	113,1126046	0,012046531	0,204346448	0,058951508	0,952990732	0,986479135
Pld4	243,5795527	0,185003729	0,227504439	0,813187338	0,416110658	0,751551608
Zfp869	454,4570405	0,074574224	0,115944957	0,643186436	0,520103133	0,814008287
Hepacam2	4,250025224	0,412370592	0,77795379	0,530070806	0,59606284	NA
Dcaf4	76,78573727	0,944575072	0,308235325	3,064460805	0,002180627	0,040433854
Arfip1	426,3199325	-0,305428562	0,138521602	-2,204916468	0,027459957	0,191005287
Tmem161a	383,9325055	-0,250218658	0,099372745	-2,517980735	0,011802978	0,116136542
Tpm3	1771,649748	-0,01218079	0,118478096	-0,102810481	0,918113386	0,976907366
Tm6sf2	986,8739304	0,468155047	0,187667462	2,494598911	0,012609958	0,1211952
Ints10	214,6752864	-0,324689752	0,131956634	-2,46057922	0,013871294	0,128069983
Ocel1	137,7425203	0,02695834	0,164076985	0,164302995	0,869492624	0,961775826
Mär-01	41,62028807	0,619057815	0,317454664	1,950066843	0,051168153	0,272862797
Ddx60	111,1113309	0,723503023	0,298378464	2,424782984	0,01531754	0,135596096
Chtop	1175,373695	-0,034360739	0,075704089	-0,453882204	0,649913613	0,87680086
Vasn	174,2098909	-0,299510511	0,185332284	-1,616073055	0,106078519	0,404035248
Ugp2	7167,336952	0,256679335	0,117296445	2,188295933	0,028648053	0,19520684
Ch25h	1,134799463	1,192186253	1,459695674	0,816736169	0,414079217	NA
Ano7	0,18725647	-1,238536202	3,340198538	-0,370797181	0,710788601	NA
Eif4ebp3	698,6030843	0,070984573	0,269641303	0,263255564	0,792353606	0,936597818
Cyb561a3	490,8633529	-0,277016657	0,105329629	-2,629997468	0,00853855	0,09560459
Synrg	404,9823337	-0,100997112	0,127394515	-0,792790112	0,427900118	0,75995385
Copg1	2610,18339	-0,406870543	0,138081312	-2,946601083	0,003212874	0,051750495
Lims1	363,9627951	0,218038865	0,142515333	1,529932679	0,126033393	0,441736801
Rnase4	20080,89714	0,707119728	0,141663045	4,991560976	5,99E-07	8,66E-05
Tmem30b	835,1849375	0,128350305	0,119184614	1,076903303	0,281523468	0,642699555
Utp15	183,4852209	0,123048109	0,160478291	0,766758594	0,443225059	0,769910424
Ric3	9,384159568	0,045611157	0,459644479	0,099231382	0,920954559	0,977341679
Zfp365	0,296539119	-0,589942906	3,316285543	-0,177892675	0,858807264	NA
Fgfr1op	343,281318	-0,079315839	0,135943538	-0,583446922	0,559592478	0,835327361
Jund	1263,849738	-0,134877419	0,18937472	-0,712225044	0,476325431	0,789577814
Gnmt	50902,93028	0,554344435	0,148573882	3,731102849	0,000190643	0,007282032
Slc43a3	538,7121158	-0,018479223	0,15670561	-0,117923173	0,906128532	0,974110031
Fabp7	72,1521816	0,534461285	0,3060533	1,746301331	0,080758605	0,351452404
Habp4	2114,973541	-0,113155289	0,08969288	-1,261586081	0,207097767	0,559656044
Clec1b	157,7772217	0,285390679	0,201709151	1,414862332	0,157108864	0,492826562
Rabgef1	327,4142638	-0,16500499	0,121669141	-1,356177815	0,175042599	0,517287416

Litaf	2586,089036	0,141414325	0,105013428	1,34663088	0,178099143	0,520240034
Tub	0,313801008	1,795331527	3,226882632	0,55636716	0,577959884	NA
Miox	3,98352392	0,507489933	0,837603812	0,605883027	0,544592431	NA
Hacl1	13407,94474	0,604500007	0,139520616	4,332693076	1,47E-05	0,001131727
Son	2601,703421	-0,011568013	0,102699177	-0,112639788	0,91031614	0,9747592
Sort1	677,4588006	-0,456693517	0,151914118	-3,006261198	0,002644817	0,045273443
Pdgfc	162,994063	0,694851399	0,227052644	3,060309656	0,002211083	0,040828678
Plag1	8,410087347	-0,702471869	0,573578413	-1,224718108	0,220681449	0,574949018
Arl10	48,84639789	-0,537004507	0,250375844	-2,144793594	0,031969354	0,207676525
Trpv4	10,79204184	0,768968309	0,502876772	1,529138652	0,126230075	0,441789741
Mlycd	2008,739048	-0,055604814	0,06938079	-0,801443944	0,422874684	0,755522295
Dnajb12	1140,883591	-0,016403709	0,108591199	-0,151059283	0,879928951	0,96546599
Stat2	1931,829003	-0,054295772	0,088242553	-0,615301463	0,538355652	0,824902774
Pex3	686,762828	0,053348216	0,111023908	0,480511061	0,63086404	0,866751905
C1qtnf1	326,1761061	-0,165148081	0,220065869	-0,750448409	0,452984685	0,775627787
Rgs17	1,570612323	0,197160885	1,189840448	0,165703633	0,868390187	NA
Mir126b	0,711969847	-1,278767606	1,98213178	-0,645147623	0,518831525	NA
Mir219c	0,142634598	-0,517475177	3,352475198	-0,154356154	0,877328924	NA
Lama1	113,3063521	-0,000341595	0,164827742	-0,002072439	0,998346434	0,999803624
Hpn	10297,70636	0,13775445	0,059029926	2,333637515	0,01961471	0,156064296
Fstl1	157,7736704	-0,184521391	0,205755467	-0,896799458	0,369825937	0,717858445
Tmem214	1622,749464	-0,346182198	0,094875794	-3,648793682	0,000263475	0,009196845
Ugt3a2	7974,188687	-0,012850238	0,168367494	-0,076322561	0,939162478	0,982918677
Zfp410	354,0951639	0,047384829	0,098988594	0,478689782	0,632159336	0,867306747
Tbc1d10b	224,3148969	-0,036092373	0,124291797	-0,29038419	0,771522338	0,928273961
Mib1	307,5981549	-0,283466677	0,204455963	-1,386443674	0,165611468	0,503882503
Zfp639	361,4055782	0,062606647	0,101713721	0,615518203	0,538212552	0,824872591
Eps15l1	427,1595335	0,006122202	0,109151893	0,056088826	0,955271045	0,987017436
Fxn	343,581855	-0,081352825	0,139645108	-0,582568386	0,560183893	0,835463907
Eps15	889,1731274	0,093360538	0,128371729	0,727267124	0,467062333	0,782586618
Hnrnpll	478,1135666	0,028956071	0,102954095	0,281252247	0,778516934	0,930864224
Fmo4	235,4100287	0,99567523	0,215663542	4,616799013	3,90E-06	0,000388963
Eml3	614,0820898	-0,245484527	0,094048364	-2,610194543	0,009049075	0,098887698
Etfa	7410,85732	0,069106879	0,063751281	1,084007687	0,278361391	0,63864998
Dlat	1312,907915	0,273966902	0,095734647	2,861732005	0,00421333	0,060961049
Msh5	22,46041526	0,155346972	0,421124252	0,36888631	0,712212468	0,905478699
Tug1	887,7757475	0,159326769	0,102865632	1,548882414	0,121409989	0,433611562
Teddm2	0,576005791	-0,551868716	2,355690536	-0,234270465	0,814775014	NA
Lrrc23	0,330540789	-1,915238087	3,169662278	-0,604240426	0,545683807	NA
Ubtf	1267,385705	-0,028824206	0,109372629	-0,263541312	0,792133386	0,936507892
Amotl1	123,9029425	-0,2047155	0,27248413	-0,751293295	0,452476163	0,774936437
Aldh7a1	14149,59407	0,181113678	0,094935563	1,907753779	0,056423047	0,287141119
Kcnc1	0,093953095	-0,517475177	3,352475198	-0,154356154	0,877328924	NA
Eid2b	77,81809981	0,024445298	0,201607365	0,121252007	0,903491432	0,973489035
Zfp703	142,7756614	-0,204299975	0,194357413	-1,051156074	0,293186914	0,654344293
Rbm43	111,7339432	0,293253703	0,190692929	1,537832077	0,124089678	0,437950082
Oxr1	1549,721967	-0,089849795	0,12597986	-0,713207609	0,475717298	0,789452019
Efcab12	3,372074662	-0,278418336	0,780549456	-0,356695317	0,721319911	NA
Zdhhc24	88,42311663	0,156480467	0,193508208	0,808650284	0,418716333	0,753407625
Dnajb3	0,462068663	1,251763544	2,495664123	0,501575325	0,61596628	NA
Lmtk3	2,535886574	-0,079465463	0,887948154	-0,089493359	0,928689831	NA
Serf2	2437,376955	-0,092821634	0,104361712	-0,889422308	0,373776161	0,720823634
Tubgcp4	714,2988997	-0,436540931	0,093582744	-4,664758839	3,09E-06	0,000325255
Slc39a13	261,7373425	-0,012399365	0,12687476	-0,097729172	0,922147351	0,977341679
Tmem222	460,1720983	0,126869498	0,091157015	1,391768896	0,163992392	0,502041284
Lnx1	7,379727572	1,926291171	0,687962675	2,799993723	0,00511036	0,069899835
Cbfb	234,8618275	-0,002920485	0,211565353	-0,013804174	0,988986212	0,996575327
Hsd3b3	8180,714095	-0,119990385	0,158289951	-0,758041708	0,448426015	0,77297141
Siva1	108,118315	0,210420614	0,177833006	1,183248367	0,236710729	0,59395378
Ang	4333,497118	0,620370319	0,117616705	5,274508558	1,33E-07	2,78E-05
Etfbkmt	1340,550936	-0,762030376	0,282098156	-2,701295131	0,006907002	0,084138787
Il11ra1	868,8767224	0,037040505	0,153552877	0,241223125	0,809382189	0,94241441
Rnf112	0,420081975	1,124593069	2,207967604	0,509334044	0,610518099	NA
Guca2b	0,227324296	1,389394708	3,349408814	0,414817893	0,67827522	NA
Mbp	21,92411277	0,995296504	0,342369061	2,907086582	0,003648122	0,056318411
C1qb	1542,440256	0,160599607	0,194692918	0,824886743	0,409435894	0,746907036

Supplementary Table S1: *Serpina1* KO vs. wildtype all DEGs

Lgals9	17852,6606	0,220870326	0,09520591	2,319922438	0,020345074	0,159251235
Rnd2	234,9764493	0,471040324	0,171938371	2,739588159	0,006151621	0,078115555
Oit3	474,2632036	-0,310055838	0,407294438	-0,761257235	0,446503438	0,771877354
Ldb2	66,31258949	-0,045785993	0,347057941	-0,131926077	0,895042762	0,970433882
Msh6	355,44599	-0,005607179	0,120268524	-0,046622167	0,962814364	0,988622522
Cyth1	817,955015	0,031881754	0,116328021	0,274067711	0,784032587	0,933246486
Ldb1	640,5141422	0,131039489	0,136331451	0,961183118	0,336460103	0,69270916
Mapk1	2042,287791	-0,019181146	0,097698635	-0,196329725	0,844352097	0,953068489
Rtn2	11,59966329	-0,233013827	0,405173141	-0,575096923	0,565225749	0,83747956
Cdkl3	11,64645169	0,471780215	0,421089545	1,120379787	0,262551955	0,622562722
Snord57	0,259974817	0,586045239	3,35071904	0,174901337	0,86115716	NA
Gm15455	1,376190639	-1,756347677	1,321910736	-1,328643175	0,183965721	NA
Atp7a	112,0210479	-0,098935791	0,192212656	-0,514720485	0,606748357	0,856921485
Hspb1	582,53201	-0,622652937	0,194449001	-3,202140071	0,001364107	0,029178784
Hspa1l	1,493885929	-2,058767023	1,347077257	-1,528321418	0,126432755	NA
Hoxd9	0,285269197	-1,521344816	3,321239936	-0,458065315	0,646905517	NA
Hoxc4	0,093303375	-0,517475177	3,352475198	-0,154356154	0,877328924	NA
Hmmr	9,236685973	-1,445482285	0,947964052	-1,524828165	0,127301963	0,444244308
Slc25a10	4773,086404	0,040579917	0,154966153	0,261863099	0,79342699	0,936746676
Xkr4	0,240301888	1,389394708	3,349408814	0,414817893	0,67827522	NA
Xkr9	544,9323862	-0,250752931	0,198499156	-1,263244323	0,206501388	0,558880735
Cyp2c50	19261,24098	0,726872241	0,198218289	3,667029146	0,000245385	0,008748133
Mta3	467,4519003	0,083513303	0,149646699	0,558069795	0,576796725	0,844877333
Eif3e	1852,992252	0,133952597	0,065865761	2,033721225	0,041979711	0,242221301
Gata5	4,539959367	-0,133769502	0,762016257	-0,175546782	0,860650015	NA
Pdgfa	93,19370728	-0,162728152	0,191600858	-0,849308053	0,395709901	0,737426273
Il1rap	4352,458442	-0,197014475	0,112066327	-1,758016702	0,078744667	0,348127467
Il1r1	545,9566789	-0,415359399	0,143225114	-2,900045856	0,003731081	0,0569513
Lilra6	0,945833633	0,633097654	1,467449334	0,431427266	0,666157728	NA
Cidea	0,51540925	-0,60320412	2,100743361	-0,287138416	0,774006339	NA
Chkb	9,264910482	0,975118161	0,538972786	1,809215949	0,070417461	0,324561524
Fkbp1a	1401,393309	-0,126115258	0,09477067	-1,330741445	0,183274101	0,529313341
Gpr12	27,13778789	-0,115937046	0,400828335	-0,289243639	0,772394939	0,928273961
Tfdp2	399,3601005	0,159698989	0,12332939	1,294898061	0,195355397	0,544332286
Ahdc1	338,2665064	-0,092882121	0,190031445	-0,488772377	0,625002856	0,864383696
Spata1	17,39096262	0,411868161	0,39025608	1,05537923	0,291251918	0,653114648
Ss18	831,8129717	-0,249941177	0,112864926	-2,214515923	0,026793309	0,18930315
Smim1	145,8384841	0,35097063	0,214943755	1,632848696	0,102500822	0,398751316
Spsb3	449,4629579	-0,128785011	0,139770715	-0,921401959	0,356840606	0,707120636
Pde9a	1014,498425	-0,250153721	0,129373419	-1,933578964	0,053164916	0,278511661
Tmem192	286,227472	0,088624617	0,106056982	0,835632082	0,403361915	0,743286759
Irak1bp1	21,02666725	0,268089833	0,37084337	0,722919309	0,46972946	0,784373852
Gne	2525,169069	-0,035150346	0,082244607	-0,42738785	0,669096855	0,886730269
Srsf7	632,6581403	0,075360587	0,176807628	0,426229272	0,66994079	0,887039558
Rida	16459,81766	-0,057330378	0,105339791	-0,544242378	0,586274693	0,8480747
Xkr5	0,592854496	2,649630537	1,990554112	1,331101989	0,183155455	NA
Dct	105,4681021	1,368216174	0,696645643	1,96400593	0,049529404	0,26689182
Hadhb	2730,502938	-0,06216585	0,079175705	-0,785163205	0,432357886	0,762604031
Cacna1f	1,693438857	0,207939283	1,079025833	0,192710199	0,847185936	NA
Zap70	188,6547179	0,69501747	0,314700135	2,20850706	0,027208946	0,190567596
Cd1d1	5104,428814	0,247317972	0,110819828	2,231712295	0,025633984	0,183556901
Itga2	2,91937469	0,782727078	0,958276066	0,816807501	0,414038445	NA
Nfatc1	285,5772025	-0,095871403	0,117679093	-0,814685093	0,415252588	0,751551608
Sufu	112,2102944	-0,235255217	0,171555955	-1,37130313	0,170280481	0,510344993
Prpf40a	1038,184013	-0,071801709	0,135654468	-0,529298523	0,59659838	0,853131217
Skap2	465,8234409	0,121729481	0,117109554	1,039449619	0,298595678	0,659386458
Mtmr1	651,7783025	0,006238057	0,101901715	0,061216404	0,951186866	0,985972877
Neu2	76,57061495	-0,469774497	0,191589015	-2,45199077	0,014206831	0,129557379
Slit1	4,937622511	-0,845191122	0,709628379	-1,19103343	0,233640461	NA
Ccnc	367,0801591	0,21340107	0,219004145	0,974415667	0,329850194	0,6866113
Rbp3	0,180974489	-1,204797453	3,342691436	-0,360427361	0,718527568	NA
Nt5c	461,2464008	0,064713303	0,102137359	0,633590921	0,526347827	0,817627711
Slc46a3	1184,411829	-0,117436642	0,14018012	-0,837755323	0,402168136	0,742519791
Rgs10	50,69116683	-0,041671561	0,316852107	-0,131517388	0,895366033	0,970578812
Atf7ip	410,8018924	-0,123401255	0,208655525	-0,591411393	0,554244809	0,832018326
Gnpnat1	1481,319563	0,048902667	0,125057428	0,391041679	0,695766428	0,897068317

Zdhhc9	2703,120652	0,115359616	0,093452127	1,234424717	0,217044684	0,57074555
Nr1h5	64,26458268	0,34815355	0,276382207	1,259681487	0,207784287	0,560178344
Zfp871	1058,322472	-0,260521541	0,139332584	-1,869781877	0,061514114	0,300578286
Paox	645,0033042	0,442823321	0,165304972	2,678826393	0,007388069	0,087408046
Fam20a	1053,074262	-0,117957697	0,078063262	-1,511052618	0,130775045	0,44968032
Pygb	180,1730256	-0,091952123	0,268935234	-0,341911776	0,732417287	0,912649428
Aifm2	655,4132661	0,157768555	0,150315859	1,049580243	0,293911145	0,655154967
Atoh8	381,9508737	0,209290831	0,203223486	1,02985553	0,303077829	0,662824716
Lrrc56	107,1838494	-0,091793251	0,180335142	-0,509014769	0,610741872	0,858645358
Tmem121	0,113662148	0,780932884	3,352475198	0,232942181	0,815806304	NA
Tgm4	0,98097456	1,585214672	1,708046041	0,928086617	0,353362641	NA
Slc9a9	99,08461699	-0,164671969	0,257081385	-0,640544118	0,521818917	0,815071515
Mir122	5,449054883	0,825149406	0,741292817	1,113122084	0,265656	0,626006345
Zfp866	255,2494528	-0,144233081	0,119326115	-1,208730213	0,22676651	0,581916106
Sybu	17,55520765	1,347681632	0,775120224	1,738674323	0,082092065	0,354079608
Galm	3747,453796	-0,097329032	0,084084124	-1,157519718	0,247060088	0,606485359
B4galnt4	0,869229359	1,428922562	2,307251487	0,61931808	0,535706836	NA
Opcml	0,113662148	0,780932884	3,352475198	0,232942181	0,815806304	NA
Ikbkg	1731,496304	-0,177979268	0,12543411	-1,41890645	0,155926292	0,491066806
Tmem205	7303,714228	0,128225187	0,115981279	1,10556797	0,26891356	0,6293952
Cpsf4	394,565319	0,352710041	0,13513687	2,610020788	0,009053672	0,098887698
Gen1	18,07792282	0,041571758	0,415560755	0,100037738	0,920314365	0,977341679
Myo1g	47,74401133	0,376842217	0,298887523	1,260816153	0,207375093	0,559854123
Dclk3	406,4422369	-0,224257102	0,204359014	-1,097368292	0,272480429	0,634510789
Nptx2	0,093953095	-0,517475177	3,352475198	-0,154356154	0,877328924	NA
Itgb3	91,07529083	0,326607972	0,231142707	1,413014395	0,157651492	0,493680148
Yme1l1	2036,915942	-0,097210926	0,106457103	-0,91314645	0,361165523	0,710630864
Tjp3	1340,078074	-0,320336902	0,130219747	-2,459971764	0,013894795	0,12819842
Abca7	211,4911496	-0,15303426	0,146921272	-1,041607235	0,297593813	0,658152471
Prmt5	423,8392001	-0,086048069	0,133492463	-0,644591221	0,519192125	0,813725618
Col5a1	241,1911246	-0,132648698	0,166632738	-0,796054244	0,426000507	0,758368511
Fam181b	1,300153038	2,149975677	1,494577879	1,438516993	0,150287419	NA
Mtg2	424,8375856	-0,05146709	0,10643203	-0,483567687	0,628692711	0,865922214
Srd5a3	257,4906342	0,017957036	0,141405253	0,12698988	0,898948408	0,971783074
Ccl24	73,51520943	0,20567237	0,210360276	0,977714871	0,328215365	0,685487182
Tdp2	607,6626318	0,091493889	0,103869399	0,880855095	0,378396255	0,724234722
Slurp1	0,20013886	1,337107854	3,350237755	0,399108348	0,689813372	NA
Phrf1	515,3250185	-0,023568505	0,122149569	-0,192947919	0,846999756	0,953824301
BC107364	0,086476712	0,780932884	3,352475198	0,232942181	0,815806304	NA
A630076J17Rik	0,093953095	-0,517475177	3,352475198	-0,154356154	0,877328924	NA
Rad54b	14,82258093	-0,524326584	0,38726794	-1,353911671	0,175764559	0,518576695
Tceanc	34,44683605	0,22088155	0,297017055	0,743666219	0,457078431	0,77843077
4930563E22Rik	3,561912153	0,938190634	0,86101509	1,089633207	0,275874749	NA
Kdf1	142,8797424	0,478540257	0,208914097	2,290607787	0,021986108	0,165876004
Mon2	1321,546121	-0,133856367	0,096446782	-1,387878009	0,165174195	0,503431976
Zfp790	213,3395	0,061623907	0,142354707	0,432889844	0,665094799	0,884653503
Ccdc125	171,2314653	-0,090269266	0,156558396	-0,576585273	0,56421965	0,837355044
Tmem11	632,6692432	0,080987233	0,122399676	0,661662148	0,508187768	0,807274415
Plscr3	205,0262738	0,074725269	0,145958945	0,511960875	0,608678392	0,857753956
Zfp574	336,9516444	0,189002052	0,122447832	1,543531224	0,122701938	0,43508389
App	1455,732795	-0,041784681	0,187322572	-0,22306271	0,8234867	0,947466482
Frem1	9,713562551	-0,263272114	0,476784649	-0,552182446	0,58082336	0,845763837
Mup21	4942,743354	-1,736396656	0,939296613	-1,848613772	0,064513603	0,30935522
Pla2g4c	1,320579357	-0,946215875	1,278883312	-0,73987663	0,459374856	NA
Olfr78	0,172953423	1,307385949	3,350719078	0,39018071	0,696402925	NA
Eif5a2	5,554386089	1,362505401	0,647828835	2,103187336	0,035449397	0,220038873
Aph1b	134,2352449	-0,254816238	0,282316419	-0,902590925	0,366743045	0,715138239
Mtmr9	417,6403941	-0,142579093	0,177552079	-0,803026882	0,421959197	0,755480449
Baiap2l2	0,520185999	1,487180202	2,384076407	0,623797206	0,532760775	NA
Skint3	1,504923599	-0,402158782	1,204600671	-0,333852364	0,738490967	NA
Ccdc157	381,3801267	-0,234145558	0,182590355	-1,28235447	0,199718328	0,549955578
Vps37d	10,57030697	0,696760308	0,469787795	1,483138377	0,13803765	0,461457253
9930104L06Rik	104,3088779	0,087364545	0,158241092	0,552097714	0,580881408	0,845763837
Vmn2r57	9,202326353	-0,260738566	0,519538689	-0,501865543	0,615762105	0,860021268
Lhfpl4	0,372290591	-2,063763532	3,073186103	-0,671538743	0,501877387	NA
Aak1	397,9172538	-0,369760418	0,098537098	-3,75249958	0,00017508	0,006871753

Zscan20	74,65497166	-0,119098451	0,176643079	-0,674232199	0,500163702	0,803121392
Colgalt2	20,21171071	-0,42250757	0,449849965	-0,939218857	0,347618389	0,699685372
Klhl38	0,539084619	1,544523896	2,081135117	0,74215455	0,457993702	NA
Nup153	539,2230451	0,082463279	0,127777871	0,645364324	0,518691117	0,813725618
Sgsm1	166,5897178	0,421087314	0,227507518	1,850872082	0,064187958	0,308457517
Ggt1	2,666041306	1,432412059	0,976582786	1,46675948	0,142441496	NA
Awat2	22,01369431	0,112666251	0,361406227	0,311744079	0,755235026	0,922462845
Apol10a	0,086476712	0,780932884	3,352475198	0,232942181	0,815806304	NA
Ppp1r3b	8346,155569	0,236419706	0,187684011	1,259668869	0,20778884	0,560178344
Rgma	7,761809148	0,724936639	0,706120476	1,026647242	0,3045866	0,664033955
Zfp507	360,2193329	-0,171572192	0,119167491	-1,439756688	0,149936249	0,482038697
BC048671	1,599173011	1,086118567	1,171160838	0,927386344	0,353725979	NA
Ttll13	21,49672386	-0,366374339	0,345135905	-1,061536438	0,288446182	0,649625922
Tmem130	1,526876612	4,020641742	1,445252922	2,781964098	0,005403102	NA
Apoe	477341,8155	0,052279777	0,079108687	0,660860124	0,50870202	0,80781294
Slc35d1	1794,392164	-0,080483416	0,101465966	-0,793206031	0,427657794	0,759882649
Impad1	497,135787	0,032286856	0,186749053	0,172888994	0,862738683	0,960942085
Nrp2	232,3906788	0,672412489	0,289821289	2,320093506	0,02033582	0,159251235
Nkain3	0,122496332	0,780932884	3,352475198	0,232942181	0,815806304	NA
Rbmx2	29,02383126	-0,247318159	0,316950388	-0,780305586	0,435211025	0,764966631
Slc28a2	17,35612703	1,106890501	0,411859288	2,687545316	0,007197933	0,08600517
Cops7b	202,3561721	-0,085648669	0,132776217	-0,645060316	0,518888099	0,813725618
Chd6	345,2530376	-0,100756831	0,122522156	-0,822356009	0,410874308	0,748353623
Gabpb2	656,2687049	-0,042740191	0,11369927	-0,375905591	0,706987084	0,90248356
Ticam1	424,1694494	-0,063032129	0,104162951	-0,605130021	0,545092608	0,827491596
Olfr433	0,463855677	-0,107586204	2,186279157	-0,049209729	0,960752158	NA
Fcgr2b	611,6667721	-0,298744575	0,269149289	-1,109958627	0,267016855	0,627372291
Bsg	9896,416297	0,153279997	0,081798343	1,873876549	0,060947442	0,298973723
Zfp874b	153,1177757	-0,165087257	0,120982849	-1,364550914	0,172394242	0,513762509
Xpot	1825,379892	0,149810586	0,099245634	1,509492962	0,131172853	0,450462334
Trim9	0,093303375	-0,517475177	3,352475198	-0,154356154	0,877328924	NA
Mfhas1	701,5419922	-0,638054689	0,132621489	-4,811095785	1,50E-06	0,000182432
Nlrc3	3,477660797	0,911547481	0,864628873	1,054264448	0,291761861	NA
Ttll5	249,6967972	-0,052868025	0,152328251	-0,347066445	0,728541402	0,911204977
Prdm2	832,7977671	-0,098570877	0,180508477	-0,546073395	0,585015487	0,847353417
Gucy2c	21,61908971	1,564211035	0,822567007	1,901621414	0,057220666	0,289000033
Zfp980	0,093303375	-0,517475177	3,352475198	-0,154356154	0,877328924	NA
Rwdd2a	1,057174439	-0,568466185	1,449666492	-0,392135838	0,694957849	NA
Ccnd1	687,2845137	-0,45796921	0,247519417	-1,850235492	0,064279616	0,308676071
Sypl2	0,920032807	0,216853631	1,92382129	0,112720257	0,910252341	NA
Grik5	168,157375	0,325588998	0,174038787	1,870784117	0,06137501	0,300117962
Dync1i2	1117,901206	-0,049817833	0,071193786	-0,699749734	0,484083611	0,79420977
Osbpl1a	2253,336949	0,267719857	0,106091382	2,52348355	0,01161985	0,115306358
Uspl1	399,9265286	0,081557705	0,13821739	0,590068333	0,555144838	0,832592702
Rtn4	1843,135899	0,597900578	0,222236331	2,690381784	0,007137032	0,08549729
Gprin2	0,320627671	0,66756312	3,24176613	0,205925749	0,836848911	NA
Marveld1	780,0208334	-0,504025204	0,108689749	-4,637283731	3,53E-06	0,000361326
Rnf182	0,42925017	1,003845674	2,50550516	0,400656	0,68867341	NA
Slc17a9	254,8400768	-1,054081046	0,229903006	-4,584894581	4,54E-06	0,000433743
Slfn5	210,2320331	-0,090482511	0,180256338	-0,501965764	0,615691604	0,860018481
Mprip	2418,075469	-0,150586607	0,122891477	-1,225362497	0,220438671	0,574490867
Fam107a	1,660262876	0,601556632	1,318314196	0,456307483	0,648168884	NA
Foxn3	222,3074011	-0,576033408	0,231094002	-2,492636775	0,012679848	0,121256719
Zfp300	3,282922686	1,514757637	0,845446578	1,791665704	0,073186531	NA
Gprin3	116,138651	0,102745407	0,325818455	0,315345571	0,752499288	0,92117599
Epb41l1	79,92193797	0,627384528	0,322471328	1,945551352	0,051708656	0,273779412
Tspan18	42,71980721	-0,199160605	0,360075842	-0,553107378	0,580189885	0,845309347
Fsd1	0,314545931	-0,664815037	3,254120508	-0,204299452	0,838119498	NA
Rexo4	521,7544095	-0,244054635	0,129225526	-1,888594636	0,058946166	0,293501414
Fez1	2,426765352	1,367192693	1,140093771	1,199193197	0,230452832	NA
Mpv17l2	347,0642599	0,132911569	0,141269626	0,940836131	0,346788842	0,698541514
P2ry6	41,28778605	0,014440727	0,2731644	0,052864602	0,957839788	0,987896001
Pyroxd1	257,4118928	0,228755683	0,107380364	2,130330678	0,033144322	0,212521078
Helz2	3129,589045	-0,253338453	0,107361657	-2,359673467	0,018291027	0,149935011
Gm5069	3,761374588	-0,791457091	0,843895761	-0,937861201	0,348315745	NA
Zfp251	36,21295909	-0,170540007	0,333131225	-0,511930417	0,60869971	0,857753956

Slc7a6os	381,1547106	0,192151667	0,12960492	1,482595465	0,138181923	0,461607728
Mamdc4	24,94913892	-0,296595981	0,404665493	-0,732941125	0,463594339	0,781663619
Spn	17,74567448	0,822157715	0,400131014	2,054721295	0,039905932	0,235069225
Bend7	204,382686	-0,093637916	0,157362075	-0,595047543	0,551811692	0,830638744
Phactr2	109,9394023	0,063523443	0,247726366	0,256425847	0,797622032	0,938325224
Retn	2,34824	4,643995325	1,92132773	2,417076094	0,015645746	NA
Gm14295	20,12528275	0,002009199	0,31667396	0,006344692	0,994937702	0,998634355
Rapgef6	398,932392	-0,045633401	0,129499672	-0,352382366	0,724551525	0,910252319
Jdp2	29,55986623	0,397293666	0,34151377	1,163331323	0,244695102	0,603538467
Cryab	94,19371047	0,035643415	0,21298247	0,167353749	0,867091715	0,961775826
Mafb	480,807064	0,259028161	0,215232586	1,203480223	0,22879054	0,584502337
C87436	318,9309514	-0,149198008	0,097681915	-1,527386195	0,126665007	0,442946503
Spata7	40,86625778	0,555072199	0,274801408	2,019903036	0,043393446	0,245531991
Cln3	360,2751533	-0,019743315	0,092332291	-0,213828929	0,830680465	0,9488355
Dbnl	730,1545031	-0,178091805	0,075407174	-2,361735584	0,018189611	0,149739474
Sh3rf2	2,051496757	1,309243721	1,096473208	1,194049897	0,232458451	NA
Wac	1297,13458	-0,010009966	0,102934185	-0,097246275	0,92253082	0,977360689
Miga2	1215,956001	-0,009536647	0,177704422	-0,053665784	0,957201444	0,98781716
Hand2	105,2887315	0,424252314	0,162311613	2,613813677	0,008953785	0,09827845
Tnfrsf8	0,086476712	0,780932884	3,352475198	0,232942181	0,815806304	NA
Epor	33,40243489	0,331856916	0,334987283	0,990655266	0,321853942	0,679006683
Uba1	4967,07721	-0,128655415	0,086232136	-1,491966007	0,135708057	0,458266143
Ubr1	410,9846385	0,023795467	0,136854619	0,173874053	0,861964445	0,960620429
Sumo1	584,0504854	0,132453577	0,11365995	1,165349602	0,243877509	0,60244002
Hoxb4	25,52100758	0,059729176	0,349046131	0,171121153	0,864128504	0,961272625
Hoxb7	3,790317421	0,666780078	1,023473254	0,651487546	0,514731814	NA
Casp3	717,7760302	0,090682809	0,130280885	0,696056138	0,486393674	0,794938512
Hoxb3	11,20376902	0,160611391	0,480165314	0,334491865	0,738008429	0,915168786
Tpsb2	0,340986444	1,884176617	3,333184127	0,565278288	0,571884494	NA
Loxl1	60,67419931	0,066133845	0,368973321	0,179237471	0,857751242	0,958260148
Zbtb7a	898,1268736	-0,270380256	0,119663951	-2,259496317	0,023852529	0,174922909
Kcnab2	14,14923313	0,198642307	0,405001117	0,49047348	0,623798892	0,863666879
Lmnb2	209,4436045	-0,480647154	0,183358615	-2,62135027	0,008758223	0,097249732
Lmnb1	125,6859609	0,144515831	0,196322278	0,736115289	0,461660539	0,781516914
Nfe2l1	2839,854199	0,020840398	0,098587275	0,211390339	0,832582695	0,950100347
Hoxa5	5,739447786	0,015230492	0,605089314	0,02517065	0,979918847	0,994664228
Zrsr2	206,5475787	0,050556234	0,116497407	0,433968745	0,664311138	0,884226961
Hoxa3	10,0606515	-0,203532179	0,431207437	-0,472005263	0,636923032	0,86976752
Ly86	176,6293461	0,404505454	0,242075452	1,670989151	0,094723821	0,382702557
Tubb4a	14,13916709	-0,004864813	0,456374529	-0,010659695	0,991494955	0,997293361
Hoxa1	2,813130114	-0,450219385	0,907512042	-0,496102931	0,619821791	NA
Tubb2a	4817,147297	-0,010709077	0,193448428	-0,055358819	0,955852603	0,987389387
Tuba1c	1593,721494	-0,030583258	0,148334949	-0,20617702	0,836652638	0,951141594
Hnrnpa1	72,82789976	0,336961594	0,21249564	1,585734154	0,112799628	0,41594735
Aprt	775,5448199	0,475508609	0,114598917	4,14932901	3,33E-05	0,001990138
Foxa2	953,3316555	-0,255327507	0,233995195	-1,091165598	0,275200022	0,636861537
1700056E22Rik	16,76593839	0,487159027	0,386439471	1,260634753	0,207440472	0,559917558
Stfa2l1	0,61862582	1,725793506	2,056708033	0,839104763	0,401410524	NA
Srrm3	0,12663974	0,780932884	3,352475198	0,232942181	0,815806304	NA
Rtn1	30,62337939	0,494975939	0,374788036	1,320682338	0,186607306	0,533163472
Pcsk5	358,5650528	0,202701046	0,158984134	1,27497657	0,202317535	0,554031776
Mir1247	0,113662148	0,780932884	3,352475198	0,232942181	0,815806304	NA
Psmd4	2623,188964	0,002027365	0,098199042	0,020645466	0,983528472	0,995431227
Mrps33	251,0129608	-0,021646696	0,125861314	-0,171988478	0,863446587	0,961272625
Rcn1	143,6461779	0,016869426	0,219253022	0,076940451	0,938670918	0,982918677
Fam188b	65,25764679	-0,483190571	0,208538804	-2,317029548	0,020502122	0,159820917
Zfp729b	175,1184704	-0,051178269	0,189928423	-0,26946082	0,787575088	0,934424153
Cops4	823,7937362	0,006314892	0,087501021	0,072169355	0,942467133	0,983364013
Slc7a11	0,502739454	-1,673660788	2,057340172	-0,813507076	0,415927391	NA
Chml	57,57598157	0,062854084	0,216277401	0,290617898	0,77134357	0,928273961
Snrpa1	397,4882397	0,095982063	0,127515309	0,752710113	0,451624132	0,774656859
Pex14	1641,48544	0,001956676	0,101333577	0,019309257	0,984594399	0,995554769
Vps29	792,5616598	-0,047483408	0,093615299	-0,507218456	0,612001547	0,858888222
1110037F02Rik	536,8023364	0,006287998	0,107041768	0,058743404	0,953156487	0,986479135
Zbtb20	121,5063161	-0,17269217	0,165868423	-1,041139517	0,297810801	0,65841452
Ikbke	782,763526	0,037106963	0,139802328	0,265424504	0,790682462	0,935868422

Mrpl54	547,9079622	0,133884577	0,156334972	0,856395567	0,391779016	0,734532481
Sostdc1	0,113662148	0,780932884	3,352475198	0,232942181	0,815806304	NA
Hnrnpk	5094,164982	0,080953612	0,089077239	0,908802438	0,363454419	0,712361223
Commd5	258,9933116	0,169549117	0,141983033	1,194150557	0,23241908	0,589440547
Ms4a4d	117,47826	0,006504552	0,173098198	0,037577236	0,970024758	0,991981655
Ppp3r1	710,7189018	-0,074617683	0,156764622	-0,475985473	0,634084727	0,867989994
Rap1b	1065,103504	0,006264507	0,135147944	0,046352959	0,96302893	0,988725638
Rab21	644,3228804	0,025869943	0,09495374	0,27244786	0,785277677	0,933556547
Luzp1	362,0593014	0,032626144	0,093538113	0,348800539	0,727239059	0,910689961
Sost	0,37991922	2,003317719	3,326247913	0,602275528	0,546990748	NA
Nudt7	16087,24805	-0,405876325	0,357666147	-1,134791003	0,256462897	0,616948518
Tsnaxip1	0,173498105	0,059593471	3,352475198	0,017775962	0,985817581	NA
Cyp4f18	12,24118738	0,578112958	0,523771336	1,103750661	0,269701313	0,63024591
Cyp4f16	59,40028102	0,320667144	0,40803411	0,78588318	0,43193593	0,7622164
Hspb2	4,919564817	0,096820895	0,700015963	0,13831241	0,889993518	NA
Derl3	124,7132768	-2,509200309	0,357222318	-7,024198056	2,15E-12	2,06E-09
Dusp19	116,3509987	-0,07609596	0,185583693	-0,410035812	0,681779677	0,89172987
Nts	0,207615244	0,059593471	3,352475198	0,017775962	0,985817581	NA
Lap3	9519,391411	-0,032286701	0,081614731	-0,395598937	0,692400921	0,895338108
Mtap	632,748566	-0,02465603	0,095922227	-0,257041883	0,797146441	0,938299386
Ubxn6	1037,189237	-0,072743261	0,103704207	-0,701449474	0,483022556	0,79399946
Morf4l1	2926,382167	0,019984795	0,081696708	0,244621789	0,806749291	0,941758461
Trim39	275,3732122	-0,244740234	0,158588211	-1,543243547	0,122771695	0,435126199
Cpne8	71,39532897	0,623912897	0,231012059	2,700780637	0,006917695	0,084138787
Plekhf1	213,6481181	0,176398062	0,153908952	1,146119572	0,25174568	0,611258264
Fabp4	185,7690239	0,023976318	0,327495775	0,073211076	0,941638152	0,983128178
Ky	0,086476712	0,780932884	3,352475198	0,232942181	0,815806304	NA
Chmp3	1603,984646	-0,132503789	0,064270938	-2,061643916	0,03924165	0,232686021
Cinp	314,6364283	-0,023043098	0,103592065	-0,222440761	0,823970784	0,947472731
P2rx4	1517,729312	-0,073514851	0,184912332	-0,397565971	0,690950149	0,894804328
Sar1b	3513,32153	0,064451701	0,123466684	0,522016947	0,601658531	0,854462419
Dus2	265,1208929	-0,029006306	0,130149131	-0,222869764	0,823636869	0,947472731
Rab6a	1434,141484	-0,139128038	0,104242896	-1,334652458	0,18199012	0,526628987
Abhd12	818,5328817	0,119055948	0,082009166	1,451739532	0,146574043	0,476487281
Bves	0,122496332	0,780932884	3,352475198	0,232942181	0,815806304	NA
Rnmtl1	180,153816	0,097239039	0,185234558	0,52495085	0,599617359	0,85430922
Stk35	293,9105265	-0,271599688	0,114626416	-2,369433658	0,017815351	0,147933805
Rbfox1	0,386069875	2,077175647	3,034738263	0,68446616	0,493680851	NA
Smim5	2,274694097	0,304586789	1,205927124	0,25257479	0,800596814	NA
Cox14	518,1769808	0,233508895	0,128448254	1,817921907	0,069076069	0,321433335
Aamdc	376,710106	0,239754309	0,144051673	1,664363233	0,096039863	0,385586553
Wfdc21	9871,164858	0,685045528	0,134155914	5,106338666	3,28E-07	5,36E-05
Nkx6-2	0,430110561	-0,033969686	2,218801737	-0,015309924	0,987784925	NA
Usp24	2047,507378	-0,129531324	0,119471503	-1,084202686	0,278274941	0,638593277
Zfp7	56,11739179	-0,375742965	0,208339801	-1,803510237	0,071308119	0,327375771
Ctdsp2	1009,419606	0,332796491	0,192171364	1,731769415	0,083314626	0,357211681
Zfp810	162,6169479	-0,223788951	0,192151726	-1,164647103	0,244161869	0,602840659
Cul4a	2535,499083	0,090164401	0,137061042	0,6578412	0,510640177	0,808952308
Vopp1	73,83608944	-0,091985642	0,177305893	-0,518796303	0,603902796	0,855545543
Gimap7	9,028047357	0,147723358	0,534260121	0,276500813	0,782163435	0,932305899
Aimp2	269,6252099	0,003680807	0,128729092	0,028593437	0,977188847	0,994219002
Tmem119	22,60268986	-0,236216818	0,413729363	-0,570945258	0,568036751	0,839867654
Emc1	634,8590313	-0,197453259	0,097410145	-2,027029725	0,042659373	0,243203202
Ctnnd2	4,568526882	1,886563997	0,843730598	2,235979117	0,025353139	NA
Gja5	12,23682039	-0,540891918	0,484622396	-1,116110033	0,264375038	0,625289954
Gja4	65,19058129	0,105921216	0,233016989	0,45456435	0,649422687	0,87669042
Mir30c-1	0,142634598	-0,517475177	3,352475198	-0,154356154	0,877328924	NA
P3h1	446,5957182	-0,604058419	0,106761897	-5,657996315	1,53E-08	4,99E-06
Cd320	78,26841983	0,162242036	0,217735975	0,745131971	0,456191944	0,778008437
Arl6ip1	3205,358726	-0,126127891	0,109827405	-1,148419121	0,250795584	0,610059346
Nsmf	3087,172743	-0,101636389	0,115102507	-0,88300761	0,37723216	0,723145504
Pdlim4	19,75317216	-0,124266449	0,439942557	-0,282460625	0,777590335	0,930671906
Capg	57,65788258	0,578978794	0,238883644	2,423685367	0,01536391	0,135846636
Chmp6	392,8695467	0,099699837	0,086205785	1,156533026	0,247463199	0,607034037
Sf3b2	2888,752917	-0,066152223	0,083745121	-0,789923307	0,429572559	0,760857915
Ppp6r3	1536,251195	-0,026028833	0,107153785	-0,242910997	0,808074346	0,942196162

Lmbr1l	284,239888	-0,149258882	0,152949362	-0,975871227	0,329128282	0,68631973
Rnase6	12,23346333	0,286006242	0,39067711	0,73207832	0,464120766	0,781663619
Themis3	0,122496332	0,780932884	3,352475198	0,232942181	0,815806304	NA
Tmem33	1724,108928	-0,322863253	0,120721399	-2,674449229	0,007485211	0,087934785
Rnf34	222,324099	0,130316228	0,117116281	1,11270805	0,265833837	0,626006345
Atp13a2	496,7646045	-0,241719833	0,109608617	-2,205299553	0,027433081	0,19091768
Gemin5	305,1047631	0,018752169	0,136229144	0,137651668	0,890515719	0,968877905
Gm5512	2,00521835	-0,385705949	0,983789536	-0,392061447	0,695012813	NA
Rab11fip3	456,5984844	-0,278616267	0,098418748	-2,830926768	0,004641335	0,065335593
Pcdh15	1,899333287	-0,691986497	1,113101279	-0,621674335	0,534156031	NA
Dnlz	486,1436729	-0,011515278	0,106158472	-0,108472526	0,913620873	0,976232707
Aven	391,0914401	-0,162704976	0,138580672	-1,174081304	0,240362461	0,598065465
Ubr5	2053,721738	-0,215545506	0,115923495	-1,859377226	0,062973688	0,304703304
Gpr19	91,2690426	0,151318856	0,154941166	0,976621381	0,32875663	0,686006046
Rpap2	246,2157304	0,114836263	0,105587361	1,087594782	0,276774036	0,637663478
Hltf	294,0369007	-0,240168161	0,171818191	-1,397804037	0,162171922	0,498749809
Gm15908	0,622007848	0,63771241	2,068144465	0,308350031	0,757816	NA
Gm12596	0,520766531	1,495047622	2,421133206	0,617499119	0,536905562	NA
LOC100862268	0,087021394	-0,517475177	3,352475198	-0,154356154	0,877328924	NA
1700124L16Rik	0,514380507	-1,665589868	2,896954197	-0,574945185	0,565328369	NA
1010001N08Rik	37,57002575	0,219844606	0,293197756	0,74981681	0,453365043	0,77576312
Pla2g12a	177,2170395	0,181274827	0,256488902	0,70675505	0,479718713	0,791837124
A630066F11Rik	20,46138092	0,664615534	0,352616004	1,88481387	0,059454972	0,294826972
Map3k20	267,213627	0,13404476	0,11904082	1,126040291	0,260148465	0,621724139
Cnot1	2134,246857	-0,146933209	0,132536624	-1,108623447	0,267592659	0,627952652
Eif5	5151,017007	-0,233842765	0,114120431	-2,049087631	0,040453547	0,236580694
Slc20a1	499,5665468	-0,031980093	0,188025459	-0,170083845	0,864944198	0,961775826
Csnk1e	151,2346547	0,029332697	0,15493659	0,189320655	0,849841508	0,954750514
Glyr1	2226,461995	-0,136592216	0,100531071	-1,35870647	0,174239621	0,517013028
Phf19	10,209712	-0,897714894	0,513099347	-1,749592742	0,080188613	0,350251568
Zbtb48	279,9346862	0,222916631	0,127613986	1,746804071	0,08067133	0,351186914
Trpm2	111,4566659	0,435119177	0,255368193	1,703889477	0,088401737	0,368519746
Tubg1	1314,690432	-0,278176025	0,118313157	-2,351184184	0,018713768	0,15208776
Snx21	198,3261761	0,178871938	0,19552876	0,914811397	0,360290651	0,710222016
Ttll3	27,08926565	-0,529775137	0,348062719	-1,522068031	0,127992035	0,445305601
Krba1	260,5934452	0,060711783	0,17288151	0,351175685	0,72545655	0,910252319
Gpsm3	75,25870081	0,267026924	0,267251267	0,999160554	0,317716921	0,675759967
Med10	301,0942939	0,144781257	0,116253694	1,245390599	0,212988207	0,566429151
Nfxl1	262,6999949	-0,636022744	0,134024807	-4,745559865	2,08E-06	0,000237592
Entpd5	7032,160984	0,219086677	0,129721339	1,688902374	0,09123814	0,374850245
Fam173a	568,533459	-0,011898583	0,10211016	-0,116526927	0,907234947	0,974750784
Adgrf4	0,493581368	2,385382152	2,875757806	0,829479502	0,406833131	NA
Nprl3	319,5755107	-0,122562423	0,139040846	-0,881485019	0,378055359	0,723903192
Hdgf	4889,421175	-0,037621298	0,072717928	-0,517359329	0,604905345	0,856127415
Ispd	54,27092158	0,400172368	0,249443107	1,60426308	0,108656076	0,409049888
Enpep	2230,997328	0,20798093	0,133232876	1,561033109	0,118515939	0,427533619
Galnt6	27,92948348	0,633961357	0,384847367	1,647305949	0,099495176	0,392688228
Bzrap1	6,889966705	0,878465647	0,606306065	1,44888151	0,147370675	0,477508134
4930539E08Rik	5,106219483	1,108179992	0,822293293	1,347669987	0,177764549	NA
Rnf43	447,2412581	0,120979027	0,209238002	0,578188597	0,563136797	0,83722511
Dcaf15	137,67817	0,351475482	0,173180736	2,029529899	0,042404347	0,243203202
Wdr37	251,8503158	-0,197481313	0,168262738	-1,173648523	0,240535837	0,59816966
Thsd4	24,53226016	1,24926562	0,375354327	3,328230232	0,000873996	0,02151833
Tbc1d16	448,3217062	-0,460899577	0,136665979	-3,3724529	0,000745018	0,019453417
Ptk2b	1009,699115	-0,220395632	0,126637299	-1,740369018	0,081794242	0,353542587
Cfap69	5,988942245	0,241898512	0,622848355	0,38837465	0,697738801	0,898398347
Nufip1	193,6818053	0,311447028	0,142969033	2,178422981	0,02937456	0,197530872
Zfp354b	54,32468798	0,335221216	0,224814725	1,491099904	0,135935268	0,458687176
Pdk4	603,4843489	-0,067640979	0,229275414	-0,295020639	0,767978113	0,927586634
Ptpn6	655,0048517	0,031281288	0,09303843	0,336218996	0,736705729	0,914722813
H2-Ke6	1833,867999	-0,068435571	0,104453296	-0,655178663	0,51235273	0,810227859
Cdca3	49,7460209	-0,611454061	0,243044108	-2,515815195	0,011875744	0,116483357
Chek2	50,36154123	0,124477025	0,266164745	0,467669093	0,640021247	0,871049783
P3h3	64,29083047	-0,070753612	0,255443511	-0,276983401	0,781792852	0,932196631
Smarcd3	33,38214498	0,423684708	0,265187482	1,59767989	0,110114236	0,410672697
Mir6917	0,093953095	-0,517475177	3,352475198	-0,154356154	0,877328924	NA

Vwa9	274,4583298	-0,030945774	0,124496319	-0,248567779	0,803695135	0,940361036
Shroom3	243,64815	-0,312232288	0,16232206	-1,923535767	0,054412799	0,282612553
Soga1	102,8439787	-0,3169245	0,192328862	-1,647826	0,099388382	0,392688228
Eya2	1,764321465	0,781713385	1,180349199	0,662272984	0,507796288	NA
Neat1	9830,998563	1,123428551	0,431243428	2,605091414	0,009184975	0,100011543
Cldn34c1	1,954506848	-0,199313451	1,025077249	-0,194437493	0,845833335	NA
Mkl1	192,3499913	-0,244647557	0,16709758	-1,464099942	0,143166643	0,470269006
Kcnq3	0,122496332	0,780932884	3,352475198	0,232942181	0,815806304	NA
Gm5420	0,361948979	-2,086101604	3,030153327	-0,68844754	0,491170995	NA
Plin5	1157,377475	-0,011080104	0,113786423	-0,097376324	0,922427546	0,977360689
Tmem102	269,625445	0,183868912	0,195100054	0,942433939	0,345970518	0,698434374
Nlrp12	689,3385828	0,094807994	0,264519018	0,358416551	0,720031607	0,908788588
Srgap2	440,6559559	0,060075147	0,169719093	0,353968115	0,723362779	0,910181193
Arap1	1974,409531	0,060158741	0,147110694	0,4089352	0,682587216	0,891903322
Dsel	17,2294109	0,042373416	0,445656337	0,095080924	0,92425055	0,977951927
Tmem164	198,8963708	-0,290452221	0,172631459	-1,682498796	0,092472158	0,37789723
Nmt1	1852,572541	-0,019667488	0,087272882	-0,225356237	0,821702154	0,946779723
Dcn	4257,37635	0,214905762	0,160840145	1,336145034	0,181501873	0,525785165
F2rl2	2,51043584	1,009859753	0,936515199	1,078316458	0,280892555	NA
Shfm1	1472,339816	0,071083518	0,12559015	0,565995967	0,571396522	0,841381253
Trim45	44,6508381	-0,431515329	0,286999698	-1,503539316	0,132700026	0,453146016
Pias3	189,5941906	-0,057035534	0,137343289	-0,415277182	0,677939003	0,890573502
Snord100	1,328302915	-0,604337846	1,35903951	-0,444680115	0,656550941	NA
Cd99l2	706,7577031	-0,012183907	0,083092653	-0,146630371	0,88342378	0,966395158
Hspa14	434,8786455	-0,133193465	0,09888622	-1,346936567	0,178000663	0,520240034
Lrrc14b	25,44830495	0,470310651	0,30562641	1,538841656	0,123842958	0,437310222
Acmsd	1238,192798	0,158621542	0,531469544	0,298458386	0,765353333	0,926709042
Snx12	232,9367563	0,18099003	0,185730782	0,974475142	0,329820676	0,6866113
Setd2	1045,327399	0,020422738	0,077683353	0,262897227	0,792629793	0,936597818
L3mbtl1	0,676923404	-0,140679951	1,823451204	-0,077150379	0,938503916	NA
Pfkfb3	167,05914	-0,638003235	0,351405399	-1,815576074	0,069435427	0,322320214
Rdh13	182,7645188	-0,15727401	0,168401038	-0,933925418	0,350342367	0,701997467
Traf2	285,3131049	0,02579855	0,115159192	0,224025104	0,822737769	0,947062273
Fgf13	0,394090941	-0,073152886	2,256596235	-0,032417357	0,974139221	NA
Inpp5d	102,2827696	0,258854212	0,23752052	1,089818309	0,275793187	0,637060499
Rnh1	1804,877342	0,042667377	0,094627246	0,450899487	0,652061993	0,877662367
Eva1c	11,20569659	-0,025417732	0,511767906	-0,049666522	0,960388135	0,98838005
Yif1b	896,7043459	-0,130659591	0,099202307	-1,317102346	0,187804321	0,534203397
Mir99a	0,087021394	-0,517475177	3,352475198	-0,154356154	0,877328924	NA
Adgrg5	5,006622591	0,345466808	0,735400182	0,469767096	0,638521425	NA
Zfp667	78,67615242	-0,209736649	0,234598664	-0,894023204	0,371309476	0,719112906
Dctn1	666,2410742	-0,116388283	0,077970462	-1,492722763	0,135509772	0,458151054
Slc38a3	28334,80552	0,492962929	0,151542483	3,252968535	0,001142061	0,026144211
Tspear	4,548653653	-0,250854864	0,663741785	-0,377940442	0,705474838	NA
Lsp1	102,4643831	0,254745394	0,249604763	1,020595084	0,307446319	0,666599067
Csrp3	534,0984425	0,78193185	0,245246414	3,188351822	0,001430863	0,030315706
Coch	0,881848241	-0,809104427	1,546050514	-0,523336347	0,600740212	NA
Ddr1	62,69054251	0,738422944	0,285423671	2,587111791	0,009678419	0,102528351
Zfp955b	152,0337952	0,169452587	0,175071026	0,967907662	0,333090487	0,68995097
Gm17359	0,213116452	1,337107854	3,350237755	0,399108348	0,689813372	NA
Tap1	606,6032055	0,298441411	0,129110132	2,311525871	0,020803826	0,161045945
Glo1	7631,543343	-0,285722652	0,119982578	-2,381367833	0,017248479	0,145577599
Gm20300	539,7998575	-0,098155858	0,148568921	-0,66067558	0,508820388	0,807845752
Rpgr	45,97021221	0,150655181	0,217134721	0,693832749	0,487787102	0,796339696
Mkrn3	5,857019467	-0,146505153	0,633313546	-0,231331154	0,817057546	0,944998904
2410089E03Rik	101,9533265	-0,087486776	0,182652087	-0,478980436	0,631952547	0,867306747
Kdm4a	768,3437665	-0,118651498	0,101470466	-1,169320521	0,242274518	0,600476091
Fip1l1	670,5959652	-0,088175252	0,095518199	-0,923125157	0,355941986	0,706022019
Ireb2	1940,518005	0,135984286	0,119065214	1,142099202	0,253412787	0,612785071
Kcnk5	753,2518072	-0,158296541	0,213563775	-0,741214379	0,458563466	0,779631911
Rnf130	2129,815114	0,148955545	0,072883059	2,043760875	0,040977176	0,238807265
Wsb2	1225,53629	-0,147985252	0,083907243	-1,763676724	0,077786422	0,345490591
Snx30	186,8911843	-0,034150686	0,159599962	-0,213976782	0,830565163	0,948801646
A830082N09Rik	6,778105609	-0,517469468	0,617896971	-0,837468855	0,402329077	0,742560142
Dact3	12,35807402	0,514902414	0,487907459	1,055328022	0,291275329	0,653114648
Tex264	1476,563772	0,088119618	0,099783675	0,883106563	0,377178699	0,723145504

Supplementary Table S1: *Serpina1* KO vs. wildtype all DEGs

Mroh2a	34,66102015	0,078740497	0,287743618	0,27364811	0,784355058	0,933246486
Ier5l	13,3478551	-0,284563658	0,579905874	-0,490706632	0,623633955	0,863617397
Psen2	7404,394443	0,300374807	0,117598847	2,554232575	0,010642214	0,109274782
Slc10a2	812,4651612	-0,760932435	0,237147955	-3,208682255	0,001333448	0,028782722
Gm19345	0,662391383	2,837885497	1,903085468	1,491202337	0,13590838	NA
Rtel1	255,5559125	0,074285187	0,134785003	0,551138367	0,581538826	0,845891913
Dtx1	17,86407483	0,58261933	0,461749329	1,261765406	0,207033214	0,559609186
Ereg	6,310536929	0,502295407	0,863913044	0,581418941	0,560958135	0,835965813
Pcdh20	1,011054898	-1,688988958	2,546720592	-0,663201516	0,507201501	NA
Ank2	79,01313975	-0,2889108	0,320313989	-0,90196123	0,367077463	0,715477272
Pkn2	935,5898539	-0,014797444	0,106916521	-0,138401847	0,889922838	0,968877905
Sccpdh	47,92258646	-0,205402753	0,241476665	-0,850611188	0,394985373	0,736890797
Pdap1	1466,950619	-0,122547662	0,08943108	-1,370302828	0,170592391	0,510601034
Tbc1d10c	20,07805002	1,002004131	0,366906606	2,730951461	0,006315177	0,079349252
Mapk1ip1l	1057,259196	-0,198077616	0,092728921	-2,13609318	0,03267182	0,210319319
Ddx31	146,3495852	-0,16974673	0,169552055	-1,001148173	0,316755178	0,674637563
Lonrf2	0,12663974	0,780932884	3,352475198	0,232942181	0,815806304	NA
Fli1	47,94454187	-0,052556044	0,254262051	-0,206700306	0,836243918	0,951141594
Mst1	4556,938333	0,088298291	0,108571825	0,813270768	0,416062833	0,751551608
Foxj1	15,77616291	0,990223022	0,546208463	1,812903111	0,069846766	0,323505282
Grip1	1,621660056	1,910146437	1,504817879	1,269353896	0,204314872	NA
Slc5a5	0,578711432	-0,894736102	2,086597865	-0,428801408	0,668067754	NA
Ddx50	1264,37507	-0,414237264	0,108020776	-3,834792529	0,00012567	0,005295032
Pag1	177,9415371	-0,163204118	0,14925934	-1,093426507	0,274206581	0,635693562
Pdxdc1	2136,865934	-0,019640108	0,073936843	-0,265633571	0,790521428	0,935843529
A230050P20Rik	1738,767401	0,140614619	0,147946712	0,950440983	0,341888228	0,695250579
Prrx1	5,074757763	0,020567775	0,677401517	0,030362753	0,97577775	NA
Dclre1c	143,7329091	-0,010984881	0,179360312	-0,061244769	0,951164276	0,985972877
Bricd5	1,371562828	-0,920131354	1,268521981	-0,725357044	0,468233019	NA
Glp2r	2,085640705	0,741669626	0,965886625	0,767864061	0,442567951	NA
Syt15	2,55969399	-0,320892777	0,869689488	-0,368973963	0,712147132	NA
Trim12c	274,9113299	0,489027299	0,166261007	2,941322855	0,003268137	0,052325419
5730435O14Rik	22,33037397	0,279992728	0,359695066	0,778416928	0,436323263	0,76575019
Snx10	417,8203936	0,612242613	0,125302493	4,886116777	1,03E-06	0,000132204
Nmi	533,8624302	0,437330444	0,133249023	3,282053666	0,00103054	0,02438458
Camk1d	2925,227806	0,914266075	0,167209302	5,467794342	4,56E-08	1,19E-05
Gspt1	3080,239365	-0,213332822	0,087914303	-2,42659972	0,015241061	0,135117869
Pgap1	235,4988713	-0,016443534	0,118048903	-0,139294255	0,88921763	0,968705223
Cfap70	0,48943796	2,371477124	2,403094546	0,986843039	0,323719592	NA
1700013F07Rik	0,453963022	1,234906816	2,502925914	0,493385285	0,621740377	NA
Itga3	226,333449	0,319962927	0,137014646	2,335246168	0,019530565	0,155697148
Senp2	565,0802353	-0,278971865	0,109789595	-2,540968156	0,011054599	0,112131207
Morn5	0,823737586	2,212481502	1,919816851	1,152444047	0,24913865	NA
9030612E09Rik	0,299593163	1,742274127	3,264336744	0,533729901	0,593528419	NA
Carf	66,17386416	0,0780475	0,189055393	0,412828741	0,679732092	0,891462871
Fus	1450,220434	0,472597819	0,103184781	4,580111656	4,65E-06	0,000440634
Itpr2	995,3281613	-0,40069737	0,117645821	-3,405963476	0,00065931	0,017770797
Adcy8	0,876048434	1,465844399	1,707744533	0,858351101	0,39069862	NA
Cacnb3	19,28094064	0,35627484	0,375690592	0,948319835	0,342966656	0,695663554
Txndc15	2142,514405	-0,107678647	0,089410748	-1,20431436	0,228468098	0,584236803
Tsr2	331,6537268	-0,084886415	0,112119102	-0,7571093	0,448984383	0,773315152
Tmem127	1257,171266	-0,05876913	0,074735546	-0,786361143	0,431655941	0,7622164
Qrich1	1040,50601	-0,061852839	0,092722656	-0,667073633	0,504725101	0,805075709
Snord17	1,989433889	-0,431994509	1,052812397	-0,410324299	0,68156807	NA
Stx16	533,2872893	-0,271586727	0,114651585	-2,36880046	0,017845879	0,148003444
Hnrnph2	1096,664715	-0,159297464	0,102305892	-1,557070283	0,1194538	0,429872906
Crispld2	15,29054391	0,384277321	0,482203176	0,796919929	0,425497536	0,758328303
Ncam1	4,604885768	0,422182846	0,843578966	0,500466302	0,616746778	NA
Ptprz1	0,287160254	0,617200912	2,785784868	0,221553688	0,824661338	NA
Dixdc1	462,5089128	0,033896592	0,192381265	0,176194869	0,860140851	0,959267413
Dll1	99,49527765	0,658647956	0,294526371	2,236295359	0,02533243	0,181982407
Dlg4	26,6143782	0,149167955	0,321991975	0,463266063	0,643173668	0,873573989
Plekha8	597,3248335	-0,430460887	0,112920377	-3,812074476	0,000137805	0,005721489
Gm17455	0,569412184	0,429513908	2,111294284	0,203436305	0,838794026	NA
Bcas1	0,746860848	2,983046125	2,635602489	1,131827025	0,257707163	NA
Rab11fip2	152,8073153	0,095197586	0,182795586	0,520787116	0,602515079	0,854608409

Hdac5	1150,365227	-0,091722733	0,109604548	-0,836851524	0,402676031	0,742637034
Gpr162	14,56813514	-0,659483804	0,488799765	-1,349190102	0,177275913	0,520079413
Ung	89,03054324	-0,896209421	0,25811992	-3,472066089	0,000516469	0,014959977
Aip	499,7025561	-0,162335184	0,087366049	-1,858103758	0,063154281	0,305356089
Cenpx	578,863403	0,056470314	0,118852251	0,475130371	0,634694052	0,868410828
Park2	31,40577514	0,531953407	0,252523034	2,106553999	0,035156258	0,219020003
Cacna1s	1,066670225	1,831887841	1,656898828	1,105612371	0,268894333	NA
Lnpk	151,2807856	0,184534177	0,159484272	1,157068188	0,247244502	0,606720219
Afg3l2	1729,275063	0,032128486	0,080509275	0,399065655	0,689844828	0,894436573
Tra2a	578,0739655	-0,402150366	0,144015133	-2,792417419	0,005231582	0,070292482
Hnrnpa3	513,0988153	-0,02741038	0,135979528	-0,201577253	0,840247229	0,951657825
1190005I06Rik	4,434445924	-0,534843036	0,761366178	-0,702478061	0,482381079	NA
Nostrin	504,836504	0,048648442	0,131981388	0,368600777	0,712425316	0,905518486
Fam58b	159,751226	0,04072376	0,138053347	0,294985677	0,768004821	0,927586634
Adipor2	15701,10552	0,102684929	0,160378443	0,640266403	0,521999419	0,815258205
Ddx39	364,6344439	-0,17839191	0,149654128	-1,192027991	0,23325027	0,590144372
Tmem140	713,2362665	0,072688525	0,198880948	0,365487623	0,714747444	0,906371815
Atp5c1	7754,241447	0,117518254	0,063129699	1,86153673	0,062668419	0,303886141
Tmtc3	203,5472115	-0,225967771	0,23517865	-0,960834542	0,336635367	0,69270916
Cadps2	277,0023233	-0,443929808	0,153857654	-2,885328075	0,00391006	0,0583195
Zfp119b	60,99897074	-0,230634627	0,214723505	-1,07410051	0,282777636	0,644358994
BC037034	331,9895147	-0,108092505	0,104150361	-1,0378505	0,299339662	0,659904651
E130304I02Rik	0,213661134	0,059593471	3,352475198	0,017775962	0,985817581	NA
Ces2a	11849,2765	-0,577497191	0,242212158	-2,38426178	0,017113424	0,144899552
Cyp3a13	4910,151641	0,369827528	0,126316511	2,927784539	0,003413865	0,053947949
Tmem39a	237,2902609	-0,410887631	0,159075905	-2,582965853	0,009795502	0,103285357
Myof	60,88103585	0,454363324	0,231948012	1,958901567	0,050124315	0,269013237
Megf8	282,982409	-0,181181564	0,187125085	-0,968237708	0,332925667	0,68995097
Tmem196	0,227324296	1,389394708	3,349408814	0,414817893	0,67827522	NA
Gan	20,05357129	-0,190510598	0,363078846	-0,524708614	0,599785768	0,854399141
Plcb1	365,5574365	-0,162234751	0,148729221	-1,090806168	0,275358182	0,636882421
Susd5	0,093953095	-0,517475177	3,352475198	-0,154356154	0,877328924	NA
A230065H16Rik	0,207615244	0,059593471	3,352475198	0,017775962	0,985817581	NA
Gsg1l	4,410454004	-0,2825728	0,719735591	-0,392606401	0,694610211	NA
Fam71f2	1,566249398	1,811846226	1,514048937	1,196689342	0,231427663	NA
Pabpc4l	3,327950367	0,421392421	0,984199096	0,4281577	0,66853631	NA
Ly96	111,0749937	0,152983198	0,160562265	0,952796711	0,340693081	0,694847567
F830016B08Rik	470,8181731	-0,387884613	0,222202954	-1,745632112	0,080874899	0,351670169
Serpina3j	0,330540789	-1,915238087	3,169662278	-0,604240426	0,545683807	NA
Ssu72	1104,780202	-0,098576486	0,088978062	-1,107874042	0,267916217	0,628270814
Trip13	14,24455284	0,093833059	0,437361154	0,214543651	0,830123131	0,94862092
Ndufaf1	799,4596542	0,110349075	0,089300203	1,235709116	0,216566708	0,569937071
Ppp1r35	146,2999309	0,040753414	0,173305693	0,235153347	0,814089715	0,944076707
Wdr53	213,4449592	0,002009362	0,148860588	0,01349828	0,989230258	0,996575327
Kctd5	109,9211745	-0,100175787	0,148601004	-0,674125909	0,500231269	0,803121392
Sectm1b	2,235194034	0,324487118	1,367694764	0,237251122	0,812461978	NA
Haghl	146,2033521	0,215930675	0,180724247	1,19480744	0,232162273	0,58906385
Qars	1313,280705	-0,249138122	0,086933506	-2,865846932	0,004158953	0,060766165
Tmem132a	23,10529904	0,557490941	0,323982904	1,720741848	0,085297679	0,362012428
Cisd1	3189,310616	0,354360812	0,109062778	3,249145282	0,001157523	0,026407732
Mir6913	0,087021394	-0,517475177	3,352475198	-0,154356154	0,877328924	NA
0610007P14Rik	2210,513483	0,562053816	0,12039012	4,668604177	3,03E-06	0,000322112
Necab3	1,412600117	-1,495992838	1,359138397	-1,100692057	0,271030705	NA
Taf1a	134,8713808	0,130869553	0,190240615	0,687915951	0,491505711	0,797760449
Chst11	4,865691519	-0,241437042	0,665762319	-0,362647503	0,71686822	NA
1700123O20Rik	643,8060486	0,09781661	0,097670316	1,001497831	0,316586188	0,674637563
Tmeff1	2,250836433	0,418500988	1,169667692	0,357794775	0,720496901	NA
Zyg11a	78,42808833	-0,478514035	0,292915811	-1,633623097	0,102338015	0,398531
Otof	0,122496332	0,780932884	3,352475198	0,232942181	0,815806304	NA
Nrf1	233,3526495	-0,011391988	0,135972424	-0,083781608	0,933230072	0,981308229
Mthfsd	304,5359905	-0,160531075	0,149509487	-1,073718319	0,282948948	0,644404577
Ssr4	2153,263955	-0,293093129	0,128184969	-2,286485936	0,022225846	0,167118861
Ces1e	4556,783962	-0,394276088	0,108237274	-3,64270156	0,000269792	0,009295988
Slc26a7	0,086476712	0,780932884	3,352475198	0,232942181	0,815806304	NA
Rasa2	52,25067599	-0,340197383	0,206641052	-1,646320422	0,099697807	0,392993389
Gpx2	2,50868533	2,151925577	1,151681131	1,868508148	0,061691275	NA

Ccser1	0,615914029	-0,025421717	2,358096283	-0,01078061	0,991398484	NA
Pon2	1707,130706	-0,161332347	0,099009122	-1,629469515	0,103213663	0,399497239
Nxph4	0,595042918	2,703769806	2,282393832	1,184620186	0,236167652	NA
Ccdc63	0,093303375	-0,517475177	3,352475198	-0,154356154	0,877328924	NA
Fam65b	96,27285835	-0,227347184	0,312457767	-0,727609322	0,466852772	0,782586618
Dusp1	578,0251049	-0,965413607	0,18838331	-5,124730039	2,98E-07	4,98E-05
Orm3	191,2022565	4,057770062	0,791769674	5,124937458	2,98E-07	4,98E-05
Ephx1	12495,84525	-0,426203817	0,153023788	-2,785212828	0,00534926	0,070973656
Vps45	244,2329675	0,057234552	0,149712814	0,382295613	0,702242108	0,900467556
4930507D05Rik	0,541834103	-1,012497205	2,384981597	-0,424530406	0,671179036	NA
Timd2	2581,509689	0,15218824	0,127349956	1,195039604	0,232071557	0,58906385
Zfp212	163,6626898	-0,121844665	0,152034658	-0,801426902	0,422884546	0,755522295
Grm1	0,259430135	1,627812584	3,345388605	0,486584005	0,626553161	NA
Smcr8	682,7135468	-0,174580943	0,096111826	-1,816435604	0,069303578	0,321819913
Scarna17	1,871946877	0,133940242	1,167748631	0,11469955	0,908683271	NA
Nf2	706,8262373	-0,405555146	0,105053815	-3,860451383	0,000113178	0,004964719
Mybpc1	0,66908672	1,974958207	1,996568613	0,989176227	0,322576928	NA
2810474O19Rik	528,2689648	-0,553367943	0,238661042	-2,31863541	0,020414813	0,159605638
Col1a2	363,2876232	-0,464611805	0,291253723	-1,595213276	0,110664551	0,411311191
Fhod3	34,04861354	0,055211379	0,260480384	0,211959832	0,832138372	0,9499007
Psmf1	806,3090412	0,010907937	0,092745227	0,117611838	0,906375224	0,974296886
Strada	81,484373	0,225853926	0,179107939	1,260993381	0,207311232	0,559794761
Ngf	85,64801956	-0,133046672	0,226207133	-0,588163024	0,556422873	0,833822555
Naca	4293,578931	0,051386101	0,102018533	0,503693783	0,614476578	0,859778545
Nrros	111,7205506	0,378520816	0,197867013	1,913006168	0,055747263	0,285429939
Myh11	41,91112893	0,233145421	0,433529089	0,537784954	0,59072552	0,850006402
Mc5r	0,087021394	-0,517475177	3,352475198	-0,154356154	0,877328924	NA
Muc1	38,11634735	0,995404223	0,397558926	2,503790401	0,01228708	0,119119632
Tmprss2	154,6430205	0,848102272	0,210011098	4,038368819	5,38E-05	0,002844172
Rars	979,4886673	-0,140315665	0,118195707	-1,187146883	0,235169688	0,592708165
Mrpl55	457,3992318	-0,138516463	0,108202092	-1,280164375	0,200487332	0,551390896
Riok2	528,0382202	-0,116898177	0,136787924	-0,854594278	0,392775796	0,735128044
Armc10	213,4523244	0,071522469	0,128312144	0,557409979	0,577247349	0,84496439
Gatad1	900,3254767	-0,060499397	0,087981962	-0,687634096	0,491683231	0,797760449
Mrpl53	610,8331274	-0,030425338	0,115325802	-0,263820736	0,791918055	0,936507892
Pak1ip1	928,568691	-0,155331776	0,117556962	-1,321331999	0,186390689	0,532805747
Utp11l	537,1283703	-0,009760558	0,105846376	-0,09221438	0,926527713	0,978802766
Eif2s2	1963,32686	-0,084628832	0,087612922	-0,965940073	0,334074169	0,690638919
Glod4	1290,880548	-0,114376399	0,086271006	-1,325780291	0,184912483	0,531519026
2010109I03Rik	0,501057478	0,373904481	2,15129781	0,173804147	0,862019385	NA
Ccdc77	74,8486416	0,15912588	0,226411831	0,702816097	0,482170364	0,793477208
Mrpl45	700,9602063	-0,031726042	0,086494119	-0,366799994	0,713768212	0,90610393
Pfdn1	718,4729396	0,117833227	0,118389321	0,99530284	0,319588997	0,677329731
Dnajb4	761,8838932	-0,171674563	0,139292889	-1,232471836	0,217772881	0,571609443
Uqcrb	5143,979714	0,087454171	0,090199933	0,969559162	0,332266277	0,689440922
Upf3a	306,8486021	-0,042586413	0,109505949	-0,388895886	0,697353165	0,898223509
Luc7l	634,2201041	-0,023312879	0,12829055	-0,181719378	0,855802958	0,957825847
Nudcd2	573,2438803	-0,138615071	0,110243618	-1,257352348	0,208626076	0,561078659
Cdk12	439,8550616	0,142159689	0,120997801	1,174894817	0,240036799	0,597588822
H2afj	635,183136	0,109804324	0,181553436	0,604804439	0,545308944	0,827491596
2610002M06Rik	526,6500697	-0,074158695	0,128690996	-0,57625395	0,564443544	0,837355044
Thap4	321,5314047	0,090635995	0,10136952	0,894114866	0,371260436	0,719112906
Gpatch1	269,7066704	-0,088414701	0,129442489	-0,683042343	0,494580087	0,799811441
Ccdc43	280,6213554	-0,06681607	0,101657229	-0,657268258	0,511008443	0,809427989
Zfp579	164,747365	0,238908894	0,189730504	1,259201284	0,207957638	0,560182483
Ctdnep1	856,2932785	-0,117315276	0,078706525	-1,49054065	0,136082136	0,458903704
Use1	857,6695235	0,094697838	0,122212682	0,774860973	0,438421816	0,766278109
Zmat5	145,9350355	0,174775096	0,146706701	1,191323192	0,233526733	0,590620297
Abhd5	961,8123015	-0,349162104	0,136434332	-2,559195324	0,010491478	0,1081423
Cdt1	59,99721463	-0,127769235	0,235919658	-0,541579434	0,588108263	0,848706754
Dram2	1348,063391	0,059143149	0,122319536	0,483513518	0,628731164	0,865922214
Ormdl3	3021,82807	-0,050334517	0,129099307	-0,389889909	0,696617956	0,897560279
Slc25a39	12079,16937	0,025960497	0,065908222	0,393888592	0,693663293	0,895984052
Ccdc91	609,81886	-0,127468014	0,081186735	-1,570059623	0,116401241	0,422986733
Arl8b	1475,39396	-0,119666587	0,091550504	-1,307110093	0,191175324	0,540266029
Stx17	647,2755601	0,209891039	0,106344728	1,973685419	0,048417524	0,26355614

Uqcrc2	7145,605497	0,015851776	0,062128202	0,255146219	0,798610168	0,938733973
Lipt2	90,80744221	-0,001861408	0,210290014	-0,008851622	0,99293752	0,998016217
Ccdc47	2470,846622	-0,113603605	0,082845618	-1,371268723	0,170291202	0,510344993
Rbm25	1437,601098	-0,131080506	0,10643	-1,23161239	0,218093911	0,572041886
Napa	1851,449523	0,048418762	0,081771914	0,592119717	0,553770425	0,831931319
Sf3a1	677,2361697	-0,181544056	0,114007338	-1,592389224	0,111297276	0,412992267
Rrp8	273,6204285	-0,073373982	0,208422198	-0,352044947	0,724804555	0,910252319
Mettl6	256,0868358	0,231061296	0,126256514	1,830094066	0,067235874	0,317560326
Med28	402,4130905	0,121126062	0,126674218	0,956201382	0,338970502	0,694071514
2610301B20Rik	96,97103702	-0,185306147	0,199004821	-0,931164111	0,351768679	0,702880198
Map4k5	361,8252061	0,062183363	0,13350549	0,46577383	0,641377399	0,871931508
F12	13375,55352	-0,048766023	0,071061007	-0,686255726	0,492551863	0,798560012
Ugt1a6b	1286,835768	-0,208482857	0,151304476	-1,377902768	0,16823331	0,507127649
Dennd4b	153,5788566	0,260292164	0,152118669	1,71111256	0,087060336	0,366110999
Pigs	600,7691287	-0,124757022	0,09852197	-1,266286305	0,205410598	0,558251976
Nol9	403,8822449	0,050192853	0,199735113	0,251297092	0,801584423	0,939799232
Plec	1331,426417	-0,11614422	0,093914751	-1,236698369	0,216199084	0,569642404
Gadd45gip1	1038,683956	-0,137215659	0,113925687	-1,204431265	0,228422934	0,584236803
Kng2	7480,394599	0,389174357	0,098987454	3,931552344	8,44E-05	0,003972999
Ccdc155	0,209517726	0,059593471	3,352475198	0,017775962	0,985817581	NA
Trim56	113,1724356	-0,41647483	0,288507472	-1,443549544	0,148865727	0,479794094
Ccdc84	242,5041045	-0,000567289	0,152411768	-0,003722082	0,997030215	0,999481197
Prmt8	0,093953095	-0,517475177	3,352475198	-0,154356154	0,877328924	NA
N4bp2l2	737,9451604	0,22333301	0,074346185	3,003960574	0,002664899	0,045497379
Xkr8	230,1619093	-0,090122891	0,114486502	-0,787192282	0,431169314	0,762070672
Gpr176	1,144500637	-3,009026713	1,711403784	-1,758221374	0,07870985	NA
Fam178b	1,242011657	0,749387418	1,349917535	0,555135701	0,578801845	NA
BC055324	10,90483235	-0,253974357	0,459607151	-0,55259009	0,580544129	0,845549021
4933407G14Rik	0,093953095	-0,517475177	3,352475198	-0,154356154	0,877328924	NA
Zfp143	203,5921333	-0,041366336	0,148066792	-0,279376187	0,779956144	0,931606698
Cyp2d10	15583,72614	-0,085158667	0,13494247	-0,631073875	0,527992222	0,81852028
Rab7	4877,472504	-0,072021056	0,06354245	-1,133432158	0,257032816	0,617800607
Kif20a	28,41043279	-0,308173865	0,364933387	-0,844466074	0,398409013	0,739635153
Pold2	399,4203452	-0,022266916	0,112122252	-0,198594976	0,842579584	0,952289345
Cyp2c40	1477,030145	2,331776899	0,972611781	2,397438468	0,016510154	0,142154471
Cyp2c39	105,7194985	2,301505817	0,651945286	3,53021314	0,000415225	0,012865677
Pola2	221,5138598	-0,161418079	0,11436077	-1,411481224	0,158102768	0,493849511
Serpinb9	260,9095077	0,550704897	0,136957248	4,020998547	5,80E-05	0,003038274
Pola1	82,51071553	-0,129044395	0,234634576	-0,549980303	0,582332883	0,846770537
Cyp2c38	1413,913705	1,485989759	0,277907337	5,347069184	8,94E-08	2,10E-05
Endou	10,08415167	0,208114956	0,486357778	0,427905063	0,668720241	0,886564944
Fdps	11450,27418	0,799464025	0,166372936	4,805252851	1,55E-06	0,000185241
Wbp1	859,3099981	-0,166398762	0,120388242	-1,382184504	0,166915057	0,505901813
Plekhg2	166,6070916	-0,015889295	0,148960039	-0,106668174	0,915052232	0,976451566
Pex10	109,4263043	0,243926797	0,161103949	1,514095702	0,130001569	0,448279636
Kntc1	8,879533571	-2,419260181	0,729171116	-3,31782229	0,000907222	0,021941826
Cabyr	33,48572225	-0,620178736	0,622908886	-0,995617096	0,319436225	0,677329731
Tnnt2	5,148268866	2,1811246	0,938748156	2,323439558	0,020155551	NA
Znhit6	156,8692045	0,002925714	0,150315573	0,019463809	0,984471108	0,995554769
Slc39a2	19,59485133	0,229105293	0,413330963	0,554290177	0,579380272	0,84530168
Iffo1	129,3830713	0,2145858	0,146906907	1,460692381	0,144099881	0,471596403
Ptpn4	60,76332392	0,089076865	0,261234237	0,340984652	0,733115136	0,913081447
Glrx3	126,8419008	0,253733984	0,188194311	1,348255334	0,177576273	0,52020157
Ppp2r3c	190,2759549	0,127330871	0,135304809	0,941066856	0,346670599	0,698497639
Chst12	32,43693186	-0,082410198	0,374104101	-0,220286808	0,825647793	0,947537943
Lrp10	1124,636007	0,038936092	0,104018806	0,374317818	0,708167877	0,902784051
Psmd14	1377,704196	0,102421289	0,110827148	0,924153438	0,355406434	0,705273655
Psmd12	2009,732022	0,086345681	0,081824679	1,055252313	0,291309945	0,653114648
Yae1d1	261,8643453	-0,240817649	0,137600004	-1,750128206	0,080096194	0,350251568
Smarca2	2217,809915	-0,161140286	0,075696715	-2,128761938	0,033273961	0,213023735
Xab2	513,0864358	0,00533885	0,089995449	0,059323554	0,952694401	0,986424351
Cep19	131,0830254	0,057920438	0,155874765	0,371583163	0,710203227	0,903999518
Rbm18	281,1941162	-0,071774865	0,137098769	-0,523526694	0,60060778	0,854422974
Mtdh	1406,282881	-0,092938525	0,122991537	-0,755649755	0,449859216	0,773622955
Polr3k	428,9448335	-0,219138972	0,100960886	-2,17053338	0,029966463	0,199910998
Slc25a37	1008,593532	-0,516677993	0,115334558	-4,47981944	7,47E-06	0,000644353

Supplementary Table S1: *Serpina1* KO vs. wildtype all DEGs

Atat1	481,8035533	-0,429438427	0,148497414	-2,891891624	0,0038293	0,057613111
Sdr42e1	3777,363533	0,567257721	0,121468724	4,669989965	3,01E-06	0,000322112
Traf3ip1	196,7912294	-0,05326215	0,122778484	-0,433806866	0,664428695	0,884295394
3110079O15Rik	0,359084026	-0,720209661	2,627628721	-0,274091106	0,784014609	NA
Bmper	22,74949271	1,977083111	0,793600311	2,491283186	0,012728263	0,121459059
3110082I17Rik	99,92901517	-0,486133226	0,332436724	-1,462333104	0,143649951	0,470584709
Zbtb49	40,22353077	-0,220751666	0,256604554	-0,860279612	0,389634929	0,733459499
Tiam2	279,4927884	-1,057862636	0,239774612	-4,41190427	1,02E-05	0,000840405
Isca2	641,0303791	0,287767176	0,094028488	3,060425432	0,002210228	0,040828678
Rhbdd3	295,1051795	0,115975768	0,117155704	0,989928475	0,32220908	0,679434258
Tmtc2	75,61576406	0,731762951	0,212124223	3,449690655	0,000561229	0,015896343
Gemin4	190,346111	0,049747288	0,182471434	0,272630554	0,785137222	0,933556547
Gm1661	0,173498105	0,059593471	3,352475198	0,017775962	0,985817581	NA
Ube2u	144,6796917	0,834080849	0,236220302	3,530944815	0,000414078	0,012865677
Pum1	1003,61816	-0,062460368	0,114876715	-0,543716518	0,586636564	0,848304107
Pus10	363,1587079	0,052693286	0,1398931	0,37666823	0,706420176	0,902122257
Zfp101	139,8892896	-0,060168531	0,180280238	-0,333750007	0,738568211	0,915438384
Xpc	469,6307358	-0,099162976	0,087450467	-1,133933069	0,256822624	0,61741803
Stim1	685,5449488	-0,174583488	0,109237383	-1,598202776	0,109997856	0,41054197
Sult2a2	787,0668829	9,227123807	2,35841132	3,912431954	NA	NA
Tsc22d3	2281,519117	0,608371436	0,427999904	1,421428908	0,155192107	0,490024394
Klhl7	265,1610711	0,001758949	0,121048112	0,014530993	0,988406353	0,996575327
Vrk2	133,171408	0,131306881	0,149714661	0,877047575	0,380460798	0,725866642
Rps20	3708,792821	0,117508886	0,14447585	0,813346221	0,416019584	0,751551608
Rims1	0,487843795	0,427018354	2,473856998	0,172612384	0,862956117	NA
Cmc1	344,0278304	0,369942832	0,132389589	2,794349866	0,005200418	0,07004174
Pef1	911,2660418	0,167540812	0,082223699	2,037621933	0,041587757	0,240791131
Ccdc80	209,2820812	-0,115450857	0,232227039	-0,49714649	0,619085752	0,861782322
Rnmt	255,5543487	-0,12320999	0,120287636	-1,024294715	0,305696093	0,664636701
Ppa1	5283,164049	0,025600383	0,123290045	0,207643553	0,835507295	0,951141594
Fam45a	525,8452072	0,049698532	0,148413751	0,334864742	0,73772712	0,91507318
Tmem86a	166,4939821	0,352047248	0,188169361	1,870906323	0,061358067	0,300117962
Ufm1	1305,45094	-0,240919595	0,107405914	-2,243075688	0,024891934	0,180368708
Tmco4	312,4573765	0,266394776	0,125940212	2,115247965	0,034408819	0,216957051
Cep83	217,7770636	0,017511309	0,124558337	0,140587208	0,888196054	0,96836734
Dync2h1	76,01158048	-0,378794174	0,208353261	-1,818038138	0,069058304	0,321433335
Il7	12,5358587	1,347320459	0,618810551	2,177274543	0,029460089	0,19767486
Mmab	1305,587503	0,328960944	0,120665974	2,726211313	0,006406597	0,079971795
Mllt3	100,3767219	-0,005654844	0,27099294	-0,020867125	0,983351651	0,995413661
Trub1	350,3110646	-0,167746871	0,108539269	-1,545494752	0,122226637	0,434047185
Dtx3	110,9361382	0,406738673	0,199381214	2,040004999	0,041349828	0,240072963
Atg5	1179,798556	0,229836513	0,129010151	1,781538211	0,074824568	0,338112818
Tnfrsf25	5,74462614	-0,064533647	0,622735546	-0,103629298	0,917463537	0,976907366
Hes7	0,6160777	2,702630981	2,293713422	1,178277528	0,238685983	NA
Cdo1	41035,17972	0,617057712	0,153622631	4,016711001	5,90E-05	0,003069981
Sptbn4	1,630295261	0,824976295	1,273430635	0,647837638	0,517089969	NA
Timeless	58,46066503	-0,19018119	0,264817709	-0,718158882	0,472659328	0,787019872
Mrps2	1345,158437	-0,024569665	0,083288297	-0,29499541	0,767997386	0,927586634
Gm7325	0,087021394	-0,517475177	3,352475198	-0,154356154	0,877328924	NA
Jarid2	260,1093913	-0,007043942	0,121370181	-0,058036845	0,95371928	0,986479135
Esf1	309,0138392	-0,01622115	0,116958251	-0,138691799	0,889693699	0,968877905
Arrdc4	307,818168	0,755039949	0,314124787	2,403630591	0,016233168	0,140466809
Acvr1	354,9193167	-0,076969143	0,150343299	-0,511955928	0,608681855	0,857753956
Echdc1	3202,312139	0,249990507	0,108721676	2,299362156	0,021484383	0,163381522
Tmem229a	18,5808644	0,235003785	0,339069208	0,693085008	0,488256205	0,796400974
Tnks	308,4483033	-0,288195339	0,202612716	-1,42239512	0,154911579	0,489743509
Chsy1	80,07668366	-0,132239519	0,219795101	-0,601649076	0,547407754	0,828528375
Slc4a11	4,684397453	1,832673904	0,85057407	2,154631758	0,031190662	NA
Gm14305	45,25281225	-0,350029531	0,257812042	-1,357692714	0,17456121	0,517225036
Aktip	346,0742661	-0,100546431	0,107231793	-0,937655039	0,348421716	0,700474534
Lta	0,172953423	1,307385949	3,350719078	0,39018071	0,696402925	NA
Axin1	589,1539995	-0,079500012	0,09067523	-0,876755555	0,380619424	0,725866642
Sphk1	5,220809361	1,883682237	0,721078898	2,612310863	0,008993244	NA
Mir7674	0,213116452	1,337107854	3,350237755	0,399108348	0,689813372	NA
Ppp2r5d	889,7829824	-0,251633878	0,096026504	-2,620462775	0,008781052	0,097260878
Tex261	874,6090337	0,018193639	0,088998631	0,204426057	0,83802057	0,951141594

E130317F20Rik	13,7836957	0,467769647	0,430568976	1,086398866	0,277302563	0,638192109
Trim15	0,61108505	-0,939471274	1,994596012	-0,471008298	0,637634809	NA
Zscan10	0,407199585	-1,150083117	2,508709016	-0,458436235	0,646639064	NA
Zfp692	163,6265846	-0,268566148	0,164784054	-1,629806658	0,103142366	0,399438862
Psap	11080,91347	0,017933342	0,090559854	0,198027509	0,843023541	0,952533952
Prtn3	42,70181354	1,288804747	0,586700081	2,196701157	0,028041796	0,192787383
Tle4	221,7242618	-0,032417327	0,168289464	-0,19262838	0,847250017	0,953824301
Unc119	226,2636849	-0,37184128	0,188531727	-1,972300825	0,048575276	0,264079785
Tom1	299,4436482	-0,003236419	0,120682437	-0,026817644	0,978605181	0,994524718
Tnfrsf12a	145,1406231	-0,255863748	0,245264597	-1,043215168	0,29684865	0,657874966
Tfap2a	0,675100266	-0,501258191	1,76111645	-0,284625239	0,775931273	NA
Tia1	128,6956322	-0,051133137	0,197514603	-0,258882819	0,795725655	0,937598879
Rangap1	1336,173298	-0,523774152	0,141839071	-3,692735355	0,000221855	0,008076805
Ranbp1	732,5229814	-0,064388775	0,109695743	-0,586976063	0,557219783	0,833822555
Ranbp2	1889,682628	-0,313892022	0,132996247	-2,360156982	0,018267203	0,14991666
Sox6	311,1896044	-0,017474391	0,191436436	-0,09128038	0,927269807	0,978888597
Tnfrsf1b	608,6407703	0,058294095	0,120530606	0,483645584	0,628637418	0,865922214
Arxes1	0,113662148	0,780932884	3,352475198	0,232942181	0,815806304	NA
Traf5	112,7134629	-0,352599684	0,198120885	-1,779719915	0,07512181	0,33883383
Traf3	284,4343914	-0,333741029	0,115023892	-2,901493104	0,00371389	0,056808912
Hsp90b1	31450,65203	-0,480399407	0,167164895	-2,873805581	0,004055585	0,059581451
Rfwd2	856,6149183	-0,100755809	0,077289356	-1,303618171	0,192363802	0,541552905
Btk	32,5425132	0,359687854	0,35757593	1,005906226	0,314460681	0,673320811
Bop1	715,7227009	-0,250393024	0,116704453	-2,145531015	0,031910414	0,207596268
Ltc4s	10,41106132	-0,632590993	0,616975403	-1,025309908	0,305216982	0,664351325
Pitpnm3	4,623685299	1,099370125	0,789738924	1,392067799	0,163901868	NA
Eci2	3091,098307	-0,122437712	0,179882256	-0,680654751	0,496089974	0,800192636
Tulp4	286,7542369	-0,425430004	0,171423618	-2,481746734	0,013074016	0,123262711
A830018L16Rik	0,313801008	1,795331527	3,226882632	0,55636716	0,577959884	NA
Hsd3b1	0,982891507	1,650328164	2,27787894	0,724502139	0,468757514	NA
Duox2	0,586187815	-1,810483038	2,009117345	-0,901133546	0,367517319	NA
Fam26e	6,308302066	0,293512283	0,605081297	0,485079087	0,627620246	0,865852703
Clcf1	17,423612	0,872115816	0,418613683	2,083342834	0,037219985	0,226281937
Tyw1	423,4109109	-0,197573558	0,117438098	-1,682363391	0,092498396	0,37789723
Tiparp	173,4635099	0,04246679	0,239900248	0,177018534	0,859493831	0,959066274
AI467606	10,61061617	0,641496517	0,478593478	1,340378727	0,180122248	0,52303526
Prkd2	223,3661668	0,312281665	0,125895733	2,480478553	0,013120616	0,123354086
Abtb2	796,3785266	0,130441365	0,126613301	1,030234298	0,302900033	0,662675256
Garnl3	3,574875875	0,375639237	0,750124879	0,500768935	0,616533751	NA
Ldlrad3	212,8177065	0,218956615	0,208140092	1,051967513	0,292814455	0,654074594
AI182371	12981,33932	-0,132694681	0,075659374	-1,753843229	0,079457374	0,349223642
Ifitm10	9,97847768	0,309556149	0,494824338	0,625587961	0,531585237	0,819884984
Nyap1	3,085005166	0,592984312	0,894608603	0,662842175	0,507431639	NA
Tmem201	410,8795028	0,017658281	0,116549961	0,151508249	0,879574804	0,965358801
Ppip5k1	119,4337191	0,048299881	0,172526129	0,279956906	0,779510568	0,931533405
Mir340	0,086476712	0,780932884	3,352475198	0,232942181	0,815806304	NA
Mir339	0,829728724	-0,670804045	1,888299074	-0,35524248	0,722407943	NA
Kcnab1	10,42903977	1,47721961	0,6387907	2,31252523	0,020748757	0,160805871
Cyp4a14	5354,31496	1,14341214	0,794943057	1,438357289	0,150332705	0,482544521
Agxt2	4054,176322	0,270730049	0,086811962	3,118580004	0,001817248	0,035992279
Pde1c	4,453923889	0,117627827	0,664669007	0,176972036	0,859530355	NA
Hmgb3	173,5217828	-0,041435986	0,136938161	-0,302589031	0,762203075	0,925512526
Rnf145	435,7013531	-0,060333949	0,209743763	-0,287655511	0,773610449	0,928621142
Eif4enif1	676,5921231	-0,019608066	0,104479881	-0,187673124	0,8511329	0,955719447
Prkci	456,2034736	-0,057529074	0,138524629	-0,415298523	0,677923383	0,890573502
Chpf2	484,9522671	0,133574665	0,135124776	0,988528296	0,322893984	0,680341949
Ube3c	971,8070496	-0,023059131	0,130383835	-0,176855753	0,859621695	0,959128896
Zc3h14	1358,3565	-0,064090606	0,111292438	-0,575875654	0,564699233	0,837355044
Il17re	7,338533945	0,356535613	0,566197662	0,62970167	0,52888979	0,818868823
Dnajc1	848,0703919	-0,117883352	0,110794616	-1,063980872	0,287337365	0,648668225
Ddx55	131,296046	0,016553827	0,169551921	0,097632791	0,922223885	0,977345313
Abcf2	996,4501964	-0,223566217	0,098698247	-2,2651488	0,02350355	0,173329312
Arrb2	432,6088488	-0,124078255	0,191179831	-0,649013312	0,516329772	0,813339545
Pkd1l3	0,82300329	0,52840345	1,754392013	0,301188928	0,763270432	NA
Agap3	570,0640705	-0,243047141	0,091125667	-2,667164463	0,007649423	0,089158792
Eif4g3	1502,819964	-0,235990751	0,158014411	-1,493476127	0,135312599	0,458120833

Supplementary Table S1: *Serpina1* KO vs. wildtype all DEGs

Ascc1	352,2996319	0,218274485	0,12063671	1,809353758	0,070396062	0,324561524
Dmtn	8,499434748	0,503904461	0,629924436	0,799944298	0,423743071	0,756445602
Itgal	241,6543905	-0,0262631	0,225170636	-0,116636432	0,907148166	0,974735881
Tmem248	1691,108029	-0,336568017	0,09159238	-3,674629006	0,000238195	0,008560303
Hp1bp3	2197,387247	-0,07983462	0,072119793	-1,106972401	0,268305861	0,628633203
Pigx	270,9680834	0,228905067	0,105857323	2,16239237	0,030587945	0,202340544
Snord53	1,938815647	0,065271093	1,174254191	0,055585148	0,955672296	NA
Bclaf1	962,6753783	0,378981488	0,108843077	3,481907152	0,000497856	0,014628221
Gm20736	0,087021394	-0,517475177	3,352475198	-0,154356154	0,877328924	NA
Dock10	97,45183161	0,02674201	0,230990756	0,115770909	0,907834107	0,9747592
Brpf1	301,929599	-0,04460672	0,102850351	-0,433705084	0,664502615	0,884305739
Mtif2	862,2966168	-0,08665942	0,104578733	-0,82865242	0,407301117	0,746679655
Mbnl1	1338,773249	0,001995183	0,122234985	0,016322517	0,986977094	0,996190907
Urgcp	560,1204583	-0,043174162	0,105940895	-0,40753065	0,683618287	0,892293506
Pigw	85,80687962	-0,009343145	0,195180128	-0,047869348	0,961820368	0,988526411
Zfp382	1,804564311	0,098748519	1,172817999	0,08419765	0,932899287	NA
Eif4a2	5139,102947	0,111025554	0,081066759	1,369557087	0,170825203	0,510601034
Zfp182	128,0246759	0,101788167	0,132331398	0,769191351	0,441779724	0,768930234
Tpm1	901,9340781	0,054607273	0,09070124	0,602056516	0,547136519	0,828388235
Elmsan1	301,215345	-0,123340819	0,142921255	-0,86299843	0,38813833	0,731971786
Apoa4	5523,982754	0,956089934	0,238693047	4,005520662	6,19E-05	0,003157586
Trp63	0,244992664	1,389394708	3,349408814	0,414817893	0,67827522	NA
Sgk1	540,6116361	0,155589011	0,546596428	0,284650619	0,775911827	0,929745022
Fkbp8	6169,797076	-0,032933871	0,071469739	-0,460808617	0,644935919	0,874634642
Masp2	8103,790705	-0,054347082	0,086697894	-0,626855852	0,530753728	0,819832073
Slc43a2	217,0003773	0,105969215	0,273569192	0,387357998	0,698491194	0,898800587
Daxx	296,7723376	0,08456764	0,100721297	0,839620242	0,401121346	0,741816472
Cep350	684,6771143	-0,300237957	0,102308649	-2,934629271	0,003339464	0,053085965
Rapgef1	956,6929496	-0,185867325	0,076964986	-2,414959518	0,015736959	0,137714114
Def8	1098,875929	-0,097812158	0,102299714	-0,956133246	0,33900492	0,694071514
Msi2	383,250791	0,082088694	0,128493319	0,638855735	0,522916786	0,815322684
Slc8b1	949,0390208	0,355444822	0,24093371	1,475280577	0,140137126	0,465234266
Adgrl4	156,1659757	-0,103955124	0,352125047	-0,295222182	0,767824158	0,927586634
Baiap2	971,9438633	-0,002390937	0,214741813	-0,011134006	0,991116532	0,99719344
Csnk1d	1526,113414	0,044718601	0,076556923	0,584122242	0,559138072	0,83506102
Myo9b	668,7008104	-0,244316014	0,093907453	-2,601667973	0,009277162	0,10013789
H2-M5	0,12663974	0,780932884	3,352475198	0,232942181	0,815806304	NA
Slco1a1	6877,708989	-1,397303503	0,577110552	-2,421205953	0,01546911	0,136415921
Sdad1	250,3262525	-0,133280743	0,155465025	-0,857303713	0,391277057	0,734518115
Cpe	20,58061716	1,612539998	0,579384892	2,783193038	0,005382677	0,071248523
Sec24a	1870,926536	-0,141186566	0,165949374	-0,85078095	0,394891046	0,73681764
Arid2	487,7026785	0,086263532	0,126548217	0,681665332	0,495450594	0,80006558
Acad11	7864,52799	-0,182469917	0,146231702	-1,247813676	0,212099299	0,56507683
Snip1	264,3573684	0,048475143	0,175719607	0,275866446	0,782650647	0,93249269
Agbl5	162,9927495	-0,058352618	0,149864169	-0,389370043	0,697002429	0,897958158
Osbpl9	3124,92936	0,082797982	0,079341906	1,043559278	0,296689342	0,657874966
1700028K03Rik	0,651227615	0,062757093	1,834448695	0,034210329	0,97270943	NA
Fam187b	109,4123658	0,174842488	0,195959894	0,892236084	0,372266405	0,719447489
Tspan9	986,3994672	-0,073455551	0,090398041	-0,812579014	0,416459468	0,751673636
Ston2	349,1941785	-0,319613425	0,218956344	-1,459713016	0,144368963	0,472355765
Ttyh3	198,4511457	0,146297341	0,182399986	0,802068816	0,422513153	0,755522295
Adhfe1	3035,640827	0,073538045	0,067343244	1,091988449	0,274838179	0,636575123
Whsc1	382,015821	-0,575729422	0,153772774	-3,744027032	0,000181094	0,007058448
Srrm2	4567,126219	0,082253442	0,128247043	0,64136716	0,521284164	0,814845193
Rnf139	1196,492637	0,012940072	0,106882669	0,121068008	0,903637168	0,973489035
Tasp1	179,9979367	-0,241443933	0,158274961	-1,525471446	0,12714155	0,44395944
Metap1	886,0930792	-0,204666249	0,114986514	-1,779915253	0,075089831	0,338803899
Dnali1	4,368156678	0,52441862	0,808409067	0,648704525	0,516529379	NA
Myh10	449,4732645	-0,153889346	0,18597739	-0,827462658	0,407974879	0,746892025
Mmd2	460,1881039	1,051083928	0,791595343	1,327804587	0,184242671	0,530663359
Lysmd4	203,1328512	-0,051058369	0,145353783	-0,351269629	0,725386077	0,910252319
Kif27	2,035869266	0,10274591	1,142705298	0,089914618	0,928355065	NA
Tmem65	110,6230515	-0,237210255	0,217572585	-1,090258019	0,275599502	0,637014134
Ralgps1	231,0242176	0,509363866	0,226045773	2,253366034	0,024236079	0,176862524
Hspa12a	30,60662518	-0,088659971	0,362723594	-0,244428465	0,806898999	0,941758461
Prox2	42,22958958	0,382529274	0,264477786	1,446356915	0,14807712	0,47829017

Supplementary Table S1: *Serpina1* KO vs. wildtype all DEGs

Ankrd9	76,96687154	0,007452255	0,22156988	0,03363388	0,973169105	0,992710425
Fbxl22	11,81300781	-0,883820793	0,603935049	-1,463436829	0,143347887	0,470500538
Plch2	0,902636228	-1,705013367	1,526547543	-1,116908134	0,264033606	NA
Hopx	692,3374807	-0,37460885	0,216094817	-1,733539263	0,082999865	0,356552758
Hcar1	1,699135349	0,127672605	1,338796844	0,095363688	0,924025957	NA
Golim4	2021,631892	0,103103962	0,142260641	0,724753956	0,468602987	0,78348591
Rnf38	203,8227893	-0,008513481	0,11089079	-0,076773563	0,938803683	0,982918677
Tyms-ps	0,12663974	0,780932884	3,352475198	0,232942181	0,815806304	NA
Cyld	475,5966863	-0,030022149	0,100189621	-0,299653284	0,764441636	0,925959974
Cecr2	411,6365715	-0,432934714	0,219020277	-1,97668782	0,048076928	0,262394191
Fis1	1190,106758	0,080642547	0,092068275	0,875899404	0,381084723	0,726157592
Mapre2	454,9745748	-0,373886819	0,134811324	-2,773408101	0,005547251	0,073122991
Aen	247,5556142	-0,053416164	0,139916963	-0,381770462	0,70263163	0,900494767
Gabrq	0,26680148	-0,62623849	2,8346967	-0,220919046	0,825155467	NA
Ntng1	0,172953423	1,307385949	3,350719078	0,39018071	0,696402925	NA
Med16	402,3912598	0,132924767	0,115843087	1,147455322	0,251193488	0,610767125
Prap1	0,093953095	-0,517475177	3,352475198	-0,154356154	0,877328924	NA
Uox	48303,08212	0,150608215	0,102136555	1,474577005	0,140326301	0,465404335
Klk1	2,301755008	-0,613691223	1,013019632	-0,605803879	0,544644994	NA
Mmp7	3,984023217	2,885966109	1,235150337	2,336530236	0,019463624	NA
Col4a2	584,8718214	-0,472458524	0,187916913	-2,514188405	0,011930668	0,116510842
Umps	951,503379	0,31971966	0,115180498	2,77581418	0,005506367	0,072741719
Hpca	1,809628011	0,1961618	1,688238836	0,116193157	0,90749946	NA
Cdk4	842,2322981	-0,031640631	0,105364114	-0,300297985	0,76394987	0,925867457
Vps13b	975,5031802	-0,322947544	0,13715175	-2,354673148	0,018539004	0,151496302
Cenpt	37,03581911	-0,160250957	0,313328417	-0,511447249	0,609037917	0,857753956
Lypd6	2,414681717	0,324027127	1,144683752	0,283071308	0,777122175	NA
Tmem251	144,0810805	-0,055441427	0,172630305	-0,321156978	0,748091433	0,919838327
Gnal	16,84596578	-0,112421141	0,47687961	-0,235743233	0,81363192	0,943942377
Mrs2	167,5507565	0,108518071	0,138905072	0,781239083	0,434661891	0,764403422
Glt28d2	111,7852411	0,017025315	0,150032997	0,113477136	0,909652289	0,9747592
Atp8b5	20,35387606	-0,048471747	0,342495518	-0,141525201	0,887455053	0,968356501
Ubn2	362,8668803	-0,388186419	0,117207505	-3,311958727	0,000926452	0,022196664
Iqcb1	121,500746	0,373567177	0,151346423	2,468292073	0,01357595	0,126214793
Tnrc6b	670,5763039	-0,201975936	0,117387263	-1,720594984	0,085324344	0,362012428
Sh2b3	453,3783885	0,110227362	0,107755077	1,02294356	0,306334532	0,665483647
Gpr173	1,559918774	0,116612037	1,199612733	0,097208068	0,922561161	NA
Dlec1	11,80344987	0,535049262	0,433554287	1,234099806	0,217165716	0,570839255
Ccdc66	137,0438197	0,45569649	0,14120925	3,227100854	0,001250514	0,02795672
Arhgap4	192,8617998	0,055230759	0,202721479	0,272446508	0,785278716	0,933556547
Eef1d	1851,00476	-0,045183225	0,103206702	-0,437793517	0,661535971	0,882818366
Cask	374,6917939	-0,300136226	0,115283654	-2,603458653	0,00922884	0,100023317
Smtn	179,3332345	-0,426349138	0,233563705	-1,825408351	0,06793943	0,319375503
Ift74	97,95814613	0,077943715	0,206761378	0,376974249	0,706192742	0,902122257
Tmem211	0,122496332	0,780932884	3,352475198	0,232942181	0,815806304	NA
Gnb4	34,05513335	-0,182626153	0,329669911	-0,553966699	0,579601636	0,84530168
Gnb3	0,334329723	-0,658036583	3,191185965	-0,206204399	0,836631252	NA
Nagpa	345,185791	-0,148901983	0,107546269	-1,384538807	0,166193534	0,504505304
Abcc2	7158,175941	-0,184985127	0,150812667	-1,226588795	0,219977183	0,574490867
Grap	74,96693457	0,097487584	0,294032515	0,331553755	0,740226244	0,916128178
Cyp2u1	315,2657935	-1,449329917	0,526905217	-2,75064636	0,005947781	0,076604904
Gm8580	0,249136072	1,389394708	3,349408814	0,414817893	0,67827522	NA
Lair1	67,14938017	0,248327067	0,28729524	0,864361927	0,387389101	0,731704562
Commd8	365,310999	-0,037847969	0,103227865	-0,366644886	0,713883922	0,90610393
Krr1	476,4478113	-0,109868186	0,135485739	-0,810920666	0,417411232	0,752195715
E2f7	6,536084549	0,154659894	0,607079343	0,254760594	0,798908012	0,938745583
Cnbp	5979,99259	0,113285126	0,072058009	1,572137889	0,115918576	0,42199407
Rgs7	5,51303552	0,096320186	0,808561215	0,119125409	0,905176	0,973769127
Prdx2	3111,735659	0,075241599	0,098503119	0,763849911	0,44495669	0,770752398
H2-Aa	728,1190038	0,710325612	0,243637911	2,915497048	0,003551225	0,055462994
H2-DMb1	88,91846154	0,512196343	0,254125911	2,015521911	0,043849993	0,24693789
Ezr	173,0231285	0,457301637	0,165438727	2,76417527	0,005706688	0,074261434
Serpinb6a	265,329509	0,247683737	0,195806691	1,264940107	0,205892799	0,558736902
Hsh2d	2,064370957	0,56873299	1,165515623	0,487966852	0,62557332	NA
Sgsm2	256,1221561	-0,055749387	0,201339425	-0,276892552	0,781862612	0,93219667
Trit1	288,0452819	0,156028728	0,184868114	0,844000216	0,398669285	0,739635153

Mir1249	0,544582018	2,490749746	2,363897854	1,053662172	0,292037614	NA
Cltc	4214,126103	-0,141165889	0,100910374	-1,398923452	0,161835939	0,498455421
1700025G04Rik	70,76591987	0,377712601	0,239148661	1,579405046	0,114243181	0,418443037
Ttc23	1191,930844	0,169517014	0,150998344	1,122641547	0,261589762	0,622343615
Ubxn11	4,227867814	-0,541240989	0,783709889	-0,690613959	0,489808173	NA
Slc6a2	0,113662148	0,780932884	3,352475198	0,232942181	0,815806304	NA
Mtcp1	34,67010974	0,016272039	0,319547149	0,050922185	0,959387528	0,987937108
Mrpl17	1009,487161	-0,081400501	0,088835852	-0,916302362	0,359508329	0,708993488
Sra1	1068,288306	0,077785531	0,110298176	0,705229533	0,480667405	0,792555814
Rsph1	3,028363061	0,352205551	0,827187205	0,425786991	0,670263067	NA
Tbrg1	1093,93753	-0,100184012	0,097318796	-1,029441556	0,30327223	0,663017899
Arhgap21	808,5622197	-0,061161065	0,115243206	-0,530712979	0,595617691	0,853006203
Ugt2b36	16696,04183	-0,03472952	0,127094838	-0,273256734	0,784655871	0,933246486
Rnps1	441,4194021	0,015766422	0,12358918	0,127571216	0,898488312	0,971757159
Rnase10	2,270864765	0,458314096	1,208844573	0,379134015	0,704588348	NA
Slc38a10	2361,449818	-0,355245946	0,101889865	-3,486568041	0,000489261	0,014414537
Cyba	167,6276963	0,208306415	0,202192039	1,030240436	0,302897152	0,662675256
Mir3057	2,778829471	-1,137545947	0,967902942	-1,175268612	0,239887268	NA
Fam189b	20,50246715	0,20539993	0,350447725	0,586107187	0,557803487	0,834302421
Ndufaf6	258,353931	0,177232543	0,118273007	1,498503738	0,134002422	0,455962937
Asxl3	0,553643307	-1,878143524	2,345612912	-0,800704802	0,423302562	NA
Zfp651	13,84016303	0,042495831	0,401731836	0,105781586	0,915755647	0,976559041
Bcas3	437,6700226	-0,093266241	0,103363532	-0,902312827	0,366890713	0,715321853
Dnajb11	3534,001905	-0,593533617	0,160611924	-3,695451763	0,000219496	0,008039573
Morc4	59,00262214	0,78590736	0,305054736	2,576283096	0,009986884	0,104553371
Tle1	1060,883556	-0,105557919	0,157873106	-0,668625084	0,503734668	0,804997854
Gcdh	14008,95316	0,209786201	0,104683485	2,004004751	0,045069551	0,251505172
Paqr7	1126,208309	-0,782593438	0,215739563	-3,627491532	0,000286188	0,009710785
Fn1	33782,64439	0,129068395	0,157254277	0,820762382	0,41178163	0,749099865
Zfp952	116,119918	-0,07825658	0,154069176	-0,50793145	0,611501418	0,858645358
Focad	319,7529082	-0,202366147	0,11138906	-1,816750657	0,069255301	0,321819299
Psme2b	27,52087866	-0,051659018	0,287185462	-0,179880339	0,857246511	0,958260148
Casp8	645,6201633	-7,96E-05	0,138964834	-0,000573071	0,999542755	0,999875741
Spin1	1157,711429	-0,115371053	0,144983214	-0,795754553	0,426174712	0,758368511
Cntnap1	37,96333724	-0,052903116	0,308946961	-0,171236888	0,864037503	0,961272625
Slc9a1	272,1163716	-0,140752117	0,132872854	-1,059299262	0,289463511	0,650939895
Matn4	0,453963022	1,234906816	2,502925914	0,493385285	0,621740377	NA
Madcam1	0,093953095	-0,517475177	3,352475198	-0,154356154	0,877328924	NA
Lyz1	0,821965806	2,142620822	1,707528048	1,254808566	0,209548262	NA
Mre11a	156,0496399	0,024507336	0,131511727	0,186350955	0,852169551	0,95595696
S100a11	148,9264618	0,152929095	0,20762902	0,736549713	0,461396225	0,781516914
Gpc3	25,06764401	1,590975049	0,450539897	3,5312634	0,00041358	0,012865677
Hsd17b10	3641,027845	0,383524948	0,089274479	4,296020018	1,74E-05	0,001277341
Loxl3	48,02434369	0,183408658	0,232162648	0,790000715	0,429527351	0,760857915
Lifr	7332,686448	-0,76285837	0,211388924	-3,608790637	0,000307628	0,010230536
Cog1	453,5598653	0,082563798	0,110356832	0,748153027	0,45436786	0,776572096
Ksr1	31,32712924	0,023958773	0,29876823	0,080191835	0,936084684	0,982191945
Kcnh2	0,902541026	-0,278054525	1,544915854	-0,179980369	0,85716798	NA
Gtpbp1	453,834064	0,025550743	0,102832203	0,248470244	0,80377059	0,940361036
Pigt	1377,332004	-0,17676794	0,078458314	-2,253017309	0,024258058	0,176926335
Mtmr4	1469,250706	-0,45329158	0,115921373	-3,910336519	9,22E-05	0,00426363
Ifrd1	276,5897241	-0,03433443	0,154500915	-0,222228006	0,824136394	0,947472731
Hsd17b6	3071,020481	-0,782426392	0,436020627	-1,794471049	0,072738016	0,331436445
Chrna4	863,1665742	1,543340119	0,597289835	2,583904877	0,009768873	0,103159611
Tmem116	3,628863467	0,082180592	0,898120526	0,091502855	0,927093038	NA
Gtf3c3	329,2696508	0,165847678	0,113036957	1,467198718	0,142322006	0,469336287
C330018D20Rik	270,2733282	0,279793367	0,146223568	1,913462858	0,055688824	0,285312203
Ankrd55	7,888130766	0,5784414	0,527352941	1,096877167	0,272695091	0,63465219
Naip5	58,24384484	0,326551116	0,323942001	1,008054267	0,313428411	0,672227864
Scn9a	0,113662148	0,780932884	3,352475198	0,232942181	0,815806304	NA
Recql	160,8190081	-0,237518538	0,130166323	-1,824731095	0,068041618	0,319375503
Scn10a	0,361384107	0,663535372	2,626667335	0,252614925	0,800565796	NA
Stat1	1630,340279	0,298768559	0,10779228	2,771706451	0,00557633	0,073282047
Gm5176	0,533707999	-0,246596291	2,124736657	-0,116059696	0,907605231	NA
Hmgn3	19,9373171	0,45132372	0,450737431	1,001300733	0,316681439	0,674637563
Hist1h4a	0,219943115	0,059593471	3,352475198	0,017775962	0,985817581	NA

Supplementary Table S1: *Serpina1* KO vs. wildtype all DEGs

Hist1h2bn	0,087021394	-0,517475177	3,352475198	-0,154356154	0,877328924	NA
Cxxc1	941,7582763	-0,194183731	0,11521674	-1,685377752	0,091915713	0,376361767
Wdr33	859,5403285	0,013424293	0,080984673	0,165763379	0,868343167	0,961775826
Hist1h2ac	1,481469155	0,268669069	1,436284404	0,187058405	0,851614841	NA
Mettl23	367,272256	-0,098252676	0,09049251	-1,085754788	0,277587494	0,638252527
Slc38a1	35,79931972	0,202319356	0,442777163	0,456932681	0,647719433	0,87637316
Slc45a3	1138,186137	-0,035992753	0,211428314	-0,170236201	0,864824383	0,961775826
Sptan1	2206,089581	0,082694292	0,087524746	0,944810417	0,344755673	0,697599302
Sorbs2	1968,495931	-0,275827417	0,157637699	-1,749755409	0,080160528	0,350251568
Lpin3	132,6413144	0,457902226	0,162976323	2,809624236	0,004959937	0,068501447
Trp53inp1	1227,22285	-0,048642146	0,163579558	-0,297360787	0,766191078	0,926944289
Itsn2	517,6612265	0,044418882	0,109267296	0,406515797	0,684363653	0,892349329
Nat8f6	35,60225559	-1,707727523	0,903994281	-1,889091069	0,058879627	0,293376491
Spint2	1837,762549	0,057449636	0,127391902	0,450967723	0,652012812	0,877662367
Gm5483	0,18725647	-1,238536202	3,340198538	-0,370797181	0,710788601	NA
Gm14124	0,086476712	0,780932884	3,352475198	0,232942181	0,815806304	NA
Alg12	295,4802764	-0,614717023	0,14182447	-4,334350925	1,46E-05	0,001129725
Lmo2	89,30372824	0,218079542	0,21997421	0,991386861	0,321496713	0,678746017
Thap2	271,161495	-0,200532479	0,146930123	-1,364815298	0,172311109	0,513629257
Lmo4	349,0749282	0,033224522	0,220332297	0,150792792	0,880139172	0,96546599
Ccnt1	113,969615	-0,320253508	0,235561617	-1,359531794	0,173978135	0,516900754
Hk2	37,43521398	-0,319286903	0,283868096	-1,124772058	0,260685638	0,621964694
Atp1b1	1427,947275	0,151255589	0,159181337	0,950209315	0,342005906	0,695250579
Ppib	8250,046221	-0,266190513	0,10997396	-2,420486753	0,015499744	0,136494096
Pou2af1	15,87768606	1,140174467	0,50493193	2,258075594	0,023940947	0,175378915
Zdhhc6	1869,973497	-0,006536668	0,071199128	-0,091808257	0,926850383	0,978888597
Pole4	399,8813286	-0,032047772	0,121159859	-0,264508164	0,791388375	0,936283441
1700010I14Rik	32,49819003	0,165889167	0,440534487	0,376563406	0,706498087	0,902122257
Rnf141	225,5987994	-0,047092282	0,19804818	-0,237781946	0,812050222	0,942960081
Ift172	374,9674825	-0,235613638	0,151882594	-1,551287949	0,120832693	0,432506634
Gpr179	0,173498105	0,059593471	3,352475198	0,017775962	0,985817581	NA
Kcnk2	2,263410029	-1,468871385	1,051059086	-1,39751552	0,162258603	NA
Ace	21,34505053	0,555637146	0,585718607	0,948641787	0,342802831	0,695589013
Slc25a44	2261,362505	0,046320432	0,16716916	0,277087183	0,781713163	0,932196631
Rcn2	163,3771887	0,133323157	0,187687285	0,710347303	0,477488793	0,790092068
Ano4	0,528242915	0,445659557	2,469965579	0,180431485	0,856813839	NA
2410002F23Rik	173,6395378	0,067554379	0,224363849	0,301092977	0,763343596	0,925598865
Mrps18b	715,4666281	-0,127235053	0,120968165	-1,051806093	0,292888523	0,654130749
Samd8	733,3635654	0,152575035	0,112579984	1,35525899	0,175335056	0,517679851
Etaa1	137,9815944	-0,278383396	0,14100002	-1,974350049	0,048341954	0,263251966
Cuta	955,0350824	-0,060258805	0,123185599	-0,489170856	0,624720741	0,864316628
4930548H24Rik	0,087021394	-0,517475177	3,352475198	-0,154356154	0,877328924	NA
Trmt112	517,9693823	-0,073379187	0,097022211	-0,756313285	0,449461385	0,773349269
Ctdp1	230,3505692	-0,013354951	0,144037855	-0,09271834	0,926127326	0,978534437
Crebzf	513,3837556	0,06466697	0,138083237	0,468318758	0,639556656	0,871049783
Nectin3	964,3007684	0,060433404	0,15213314	0,397240231	0,691190318	0,894879271
Anapc5	1666,693463	-0,046124792	0,070016838	-0,658767132	0,510045317	0,808610978
Cxcl12	8865,989085	-0,30725878	0,107364878	-2,861818382	0,004212182	0,060961049
Dennd5a	850,9829518	-0,066528618	0,099728441	-0,667097745	0,5047097	0,805075709
Arhgap23	304,0697014	-0,519337883	0,126168999	-4,116208314	3,85E-05	0,002219463
Smoc1	2285,584464	0,330319349	0,164970009	2,0022994	0,045252541	0,251865618
Pik3cg	20,99682232	-0,028361001	0,349329539	-0,081186955	0,935293274	0,982050665
Ppfia4	29,83813647	0,182814044	0,312093513	0,585766881	0,558032182	0,834302421
Olfml2b	8,83574443	0,550782302	0,543272222	1,013823787	0,310666843	0,669972995
Fsd1l	9,429032195	-0,275084	0,471726384	-0,583143129	0,559796952	0,835352768
Efcab5	0,652955474	-2,091271382	1,929036908	-1,084101281	0,278319895	NA
Zfp944	232,724079	-0,029141874	0,161192475	-0,180789295	0,856532966	0,9578959
Slc35e2	1040,982011	0,137271386	0,145690211	0,942214201	0,346082985	0,698487835
Gm5126	0,244992664	1,389394708	3,349408814	0,414817893	0,67827522	NA
1700008J07Rik	46,68940447	-0,139339419	0,239996666	-0,580588979	0,561517502	0,836472635
Snora3	4,174814468	0,050988273	0,728454945	0,069995095	0,944197564	NA
Snora21	1,283157174	2,943529524	1,659513346	1,773730553	0,07610773	NA
Cntn2	6,963519498	0,202843384	0,590743852	0,343369438	0,731320548	0,912061199
Tacc1	698,8780991	-0,182551082	0,121132104	-1,507041285	0,131800075	0,451803899
Gm17769	0,810134546	1,337459811	1,739241862	0,768990121	0,441899175	NA
Wbscr25	8,295686413	0,148479717	1,783019349	0,083274316	0,933633422	0,981500765

Supplementary Table S1: *Serpina1* KO vs. wildtype all DEGs

Zmat3	146,3440974	-0,225502012	0,190056385	-1,186500584	0,235424671	0,592840917
Acss2	14034,2408	1,328671576	0,240288018	5,529495765	3,21E-08	9,79E-06
Bank1	63,76773928	0,077225563	0,268362414	0,287765941	0,77352591	0,928621142
Cadps	0,113662148	0,780932884	3,352475198	0,232942181	0,815806304	NA
Nolc1	751,3162691	-0,449093951	0,255996885	-1,754294593	0,079380042	0,349223642
Ermard	138,9142827	0,325756372	0,127525547	2,554440101	0,010635873	0,109274782
Rbm20	65,29857238	-0,052457713	0,300690029	-0,174457774	0,861505714	0,960349361
Ubap2l	1502,749344	-0,005804525	0,092557196	-0,062712844	0,94999517	0,985972877
Majin	0,180324769	-1,201310802	3,342952034	-0,359356278	0,719328578	NA
Tnnt3	4,884834056	4,202964178	3,22109636	1,304824106	NA	NA
Gtf2f1	695,4234151	-0,377859547	0,124165345	-3,043196535	0,002340794	0,042289291
Snora75	0,440404624	0,238114245	2,561725724	0,092950718	0,925942713	NA
Gigyf2	863,0895959	-0,289322341	0,111562359	-2,593368799	0,009504079	0,101415126
Rcan1	543,0173251	-0,512013862	0,186701963	-2,742412852	0,006098963	0,077728349
1810010H24Rik	65,46229462	0,9427587	0,222627461	4,234691878	2,29E-05	0,001532624
Satb1	11,73161524	0,089779304	0,461835179	0,194396851	0,845865156	0,953584926
Rnls	5,682204361	-0,355108546	0,634976282	-0,559246945	0,575993198	0,84407027
Gpr52	0,299593163	1,742274127	3,264336744	0,533729901	0,593528419	NA
LOC100504703	29,02262522	0,238645182	0,326362279	0,731227831	0,464640003	0,781663619
Zfp560	85,93617625	0,133773606	0,190015392	0,70401458	0,481423694	0,793276609
Rc3h1	413,6732859	0,002564739	0,124711638	0,020565354	0,983592378	0,995431227
Pecam1	289,0727004	-0,079715137	0,27551617	-0,289330157	0,772328737	0,928273961
E030030I06Rik	65,33247368	0,242466758	0,225876198	1,073449791	0,283069355	0,64447449
Llgl2	958,7632156	-0,107348624	0,113826613	-0,943088973	0,345635395	0,698427993
Car13	9,315566014	0,465080779	0,490069879	0,949009108	0,34261598	0,695589013
Porcn	120,2216212	-0,034372894	0,207931797	-0,1653085	0,868701169	0,961775826
Wbscr27	481,4416235	0,445851224	0,19029551	2,342941372	0,019132389	0,154084887
Grpel1	2909,753178	-0,024453002	0,069352659	-0,352589241	0,724396404	0,910252319
Sltm	719,2703046	-0,200181299	0,118009493	-1,696315212	0,089826201	0,371791481
Elob	2048,490821	0,03225462	0,111227272	0,289988415	0,7718251	0,928273961
BC005537	3006,934215	0,0277486	0,094449567	0,293792768	0,768916262	0,927598042
Cptp	483,0325894	-0,011148921	0,112455516	-0,09914072	0,921026542	0,977341679
Serpinb7	0,680742636	2,866720508	2,194139401	1,306535267	0,191370594	NA
Ldhd	4576,856681	0,138212338	0,104907089	1,317473761	0,187679871	0,534076669
Rflna	1,312024834	0,603988083	1,389742674	0,434604257	0,663849704	NA
Mtfr2	3,292747728	-0,568250702	0,885578991	-0,64167139	0,521086569	NA
Chst13	131,4523048	0,110027303	0,203840076	0,539772674	0,589353814	0,849403961
Zswim1	119,7708274	-0,586400167	0,220489567	-2,65953703	0,007824813	0,090493012
Cog7	585,2422418	-0,220610939	0,136538169	-1,615745545	0,10614934	0,404074749
Vcan	3,508972067	-0,540602349	0,8480079	-0,637496831	0,523801273	NA
Trub2	229,4513271	0,046034749	0,125926956	0,365567074	0,714688148	0,906371815
Nxpe3	10,96823775	-0,134667522	0,468377471	-0,287519213	0,773714793	0,928621142
Dclk2	14,05784609	0,186930981	0,424731977	0,440115159	0,659853704	0,882334883
Tnik	41,74411356	0,357665312	0,357741374	0,999787385	0,317413412	0,675716545
Klhl13	163,8543269	0,326710912	0,245849371	1,328906846	0,183878705	0,530143284
Pdzrn4	0,087021394	-0,517475177	3,352475198	-0,154356154	0,877328924	NA
Rbl2	900,9542258	0,022263631	0,117255631	0,189872594	0,849408968	0,9546657
Pdlim2	34,23797389	0,351485156	0,335353139	1,048104567	0,294590432	0,655663613
Cox4i1	8527,127846	0,033842001	0,109192952	0,30992844	0,756615375	0,923140802
Cacna1e	1,446789883	0,242919044	1,132711547	0,214457992	0,830189923	NA
Cacna1c	4,876370644	0,542312737	0,772174181	0,702319179	0,482480135	NA
Stmn2	0,919328537	-0,336723141	1,510678552	-0,222895295	0,823616999	NA
Rbm4b	184,4485109	0,261678241	0,156155393	1,675755383	0,093786117	0,380194927
Mob4	407,4662553	-0,105429025	0,139985217	-0,753143994	0,45136339	0,774518953
Rwdd3	133,70414	0,214690494	0,202897582	1,058122485	0,289999605	0,651597431
Ntpcr	187,2243035	0,015146199	0,14863085	0,10190481	0,918832232	0,976907366
Hvcn1	23,10728348	0,419790908	0,362067383	1,159427575	0,246281942	0,605518092
Mterf4	419,9192428	0,114409633	0,104372674	1,096164621	0,273006739	0,634872238
Hs6st2	0,086476712	0,780932884	3,352475198	0,232942181	0,815806304	NA
Naglu	773,8533293	-0,041877779	0,085009987	-0,492621868	0,622279793	0,86313492
Akr1c12	2107,58778	-0,173860051	0,113646232	-1,52983559	0,12605743	0,441736801
Odc1	2023,202556	-0,836841361	0,159366199	-5,251059294	1,51E-07	3,02E-05
Abcc5	85,13943939	0,032700246	0,270361162	0,120950235	0,903730452	0,973489035
Taf4b	49,17310528	0,550343347	0,279503176	1,9690057	0,048952437	0,264881063
Smim10l1	1794,755607	0,01235622	0,108753564	0,113616693	0,909541654	0,9747592
Fam212b	1,996817712	-1,072727111	1,062539919	-1,009587585	0,31269292	NA

Supplementary Table S1: *Serpina1* KO vs. wildtype all DEGs

Aifm1	3111,58871	0,143662657	0,105422275	1,362735312	0,172965944	0,515007063
Fnip2	551,2461678	-0,005798141	0,238061406	-0,024355651	0,980568923	0,994739255
Nlrx1	242,8280185	0,051272198	0,121198094	0,423044593	0,672262726	0,888049388
Oscar	1,099838587	0,911752392	1,443610776	0,631577713	0,527662854	NA
Tfpt	194,742767	-0,127375649	0,138280002	-0,921142954	0,356975797	0,707128379
Esrp1	4,306742885	0,686484871	0,763585964	0,899027619	0,36863795	NA
Arhgef9	111,1782457	0,587439965	0,208342754	2,819584336	0,004808589	0,066976019
Pdp1	28,96275822	-0,284009549	0,366998641	-0,773870847	0,439007171	0,766699787
Sntg1	5,535929759	-1,036008956	0,630664561	-1,642725817	0,100439675	0,393294268
H2-Q4	1039,88224	0,637449289	0,15026676	4,242117742	2,21E-05	0,001502624
Apol11b	1,911929	0,898083229	1,196264188	0,750739876	0,45280922	NA
Pum2	1162,723277	0,00641899	0,080163593	0,080073634	0,936178693	0,982191945
Fcrla	8,867469804	0,5894224	0,565786788	1,041774769	0,297516115	0,658152471
Blzf1	238,0061597	-0,076258087	0,140799424	-0,541607949	0,588088614	0,848706754
Tor1aip2	4116,942362	0,153796882	0,086374218	1,780587842	0,074979807	0,33842169
Nrde2	245,9586279	-0,084629065	0,110015172	-0,769249035	0,441745486	0,768930234
Pla2g4a	30,03854501	0,134458802	0,326105246	0,412317201	0,680106942	0,891524003
Tmem40	0,179780087	0,059593471	3,352475198	0,017775962	0,985817581	NA
Phf14	476,9030161	0,166262159	0,106980404	1,554136577	0,120151842	0,431224155
Mir6942	1,36767436	-0,59340245	1,416563564	-0,418902805	0,675287167	NA
Hmgxb4	200,7605442	0,116471453	0,195230657	0,596583829	0,550785271	0,830245606
Wrn	253,7668017	0,095328825	0,126469924	0,75376676	0,450989287	0,774175112
Dnajb2	1860,214568	-0,348953385	0,102192927	-3,414652987	0,000638633	0,017353429
Rbfox2	521,7883267	0,05482697	0,126106093	0,434768602	0,663730398	0,883893982
Unc45b	40,88077821	0,131136166	0,26813596	0,489065942	0,624795012	0,864329938
Gpr107	373,3465114	-0,082297684	0,108495154	-0,75853788	0,448129045	0,77297141
Gskip	154,9830684	0,455348838	0,148061428	3,075404883	0,00210217	0,039251266
Tgm1	368,6853555	0,219475989	0,206317927	1,063775657	0,287430341	0,648768569
Itgb1bp1	264,8790913	-0,085180192	0,108889234	-0,782264594	0,43405909	0,763946284
Actb	12426,15503	-0,284009364	0,13923645	-2,039763039	0,041373933	0,240072963
Hmgcr	3313,879401	0,38868673	0,265643845	1,463187414	0,143416104	0,470500538
Nefm	0,180324769	-1,201310802	3,342952034	-0,359356278	0,719328578	NA
Slc6a9	724,3103664	-0,444755441	0,181723901	-2,447424027	0,014388146	0,130498723
Glud1	27144,5072	-0,01133592	0,115899515	-0,097808177	0,922084614	0,977341679
Itgb6	2,17089555	1,298097621	1,033362287	1,256188305	0,209047706	NA
Cables1	40,18162388	1,870146483	0,48108383	3,887360924	0,00010134	0,004577077
Pdgfrb	298,6496776	0,078817002	0,218134306	0,361323274	0,717857797	0,907864998
Etl4	290,8883201	0,316231352	0,132943753	2,378685307	0,017374502	0,145962115
Repin1	1467,094678	0,063939959	0,078776104	0,81166693	0,416982775	0,752195715
Nbr1	4894,793387	-0,064483273	0,09502479	-0,678594219	0,497395007	0,801288247
Gimap1	50,81720893	0,187447192	0,243685783	0,769216774	0,441764634	0,768930234
Irgm1	1927,780397	0,153608264	0,102636566	1,496623182	0,134491335	0,456327747
Cfl2	1136,947918	0,067641076	0,11053198	0,61195933	0,540564666	0,826407188
Cfi	19570,05363	0,007884913	0,115323409	0,06837218	0,945489367	0,984440501
Col13a1	86,87138176	-0,308704564	0,460467486	-0,670415552	0,502592923	0,80422495
Gm12610	0,421351117	-1,125417522	2,481765817	-0,453474503	0,650207099	NA
Med22	533,1403996	-0,118502607	0,125749818	-0,942368022	0,346004253	0,698434374
Tecpr2	360,1346122	-0,26143051	0,179644569	-1,455265309	0,145595827	0,474401806
Rps29	3751,860899	0,032648136	0,156138093	0,209097825	0,834371873	0,95084595
Atg4d	632,2233143	-0,220585556	0,119309024	-1,848858952	0,064478182	0,309296311
Gpr156	0,207615244	0,059593471	3,352475198	0,017775962	0,985817581	NA
Mtrr	166,85026	-0,243576356	0,19081636	-1,276496187	0,201780174	0,553126746
Col23a1	4,585021365	2,153374941	0,794518497	2,710289249	0,006722456	NA
Ago3	61,0558739	-0,621129276	0,217507826	-2,855664039	0,004294693	0,061604884
Ostc	1735,790951	-0,40364923	0,094202846	-4,284894215	1,83E-05	0,001321191
Zfpl1	397,8246351	-0,164466296	0,101550741	-1,619547974	0,105329413	0,403011718
Scd3	0,876793357	0,013797604	1,546382146	0,008922506	0,992880964	NA
Snx5	1203,791549	0,180052044	0,095245123	1,890406963	0,058703553	0,293022089
Prdm16	13,47072644	-0,268166278	0,439528266	-0,61012294	0,541780372	0,826550472
Zfp709	46,32383445	0,093640839	0,227688663	0,411267023	0,680876747	0,89166825
Actr8	571,6975855	0,005202597	0,09091638	0,057223981	0,954366775	0,986616875
Rragd	37,84389308	0,39562497	0,365213163	1,083271388	0,278687981	0,639179897
4933434E20Rik	558,6718555	0,150082529	0,104152704	1,440985423	0,149588801	0,481660087
Dcp2	252,8026299	-0,057828933	0,121953248	-0,47418936	0,63536488	0,868882271
Gtf3c2	1138,434118	0,005282843	0,096956956	0,054486478	0,956547581	0,987707257
Med26	125,9100745	0,11840188	0,17837198	0,663791927	0,506823493	0,806249795

5730508B09Rik	60,3147754	-0,063192713	0,206934413	-0,305375564	0,760080131	0,924925298
Sugp1	440,2243596	-0,1217788	0,086953332	-1,400507573	0,161361377	0,497950692
Ankrd24	136,6609189	-0,084520395	0,149771639	-0,564328438	0,572530631	0,841794408
Cic	829,1422131	-0,363809651	0,115067924	-3,161694755	0,001568539	0,032460986
Zfp654	296,0416976	-0,190312405	0,152991541	-1,243940705	0,213521387	0,566976917
Osbpl3	43,50543262	0,382981569	0,390949841	0,979618171	0,327274632	0,684930268
Cdc40	551,586254	0,170500075	0,124013039	1,374856027	0,169176084	0,508707843
Psd3	1093,646449	-0,043032675	0,152938713	-0,281372024	0,778425073	0,930864224
Usp15	1027,363515	-0,112307827	0,08059446	-1,393493125	0,163470723	0,500903069
Dram1	23,32036567	0,48491769	0,318247487	1,523712553	0,127580532	0,444752056
Syde1	83,03536886	0,258073588	0,307652482	0,838847734	0,40155476	0,742100471
Cnot2	864,7765041	-0,013411589	0,104263964	-0,12863111	0,897649549	0,971242863
Arhgef3	600,1384661	-0,156194788	0,171455673	-0,910992239	0,362299463	0,711550958
Pnpt1	861,7782153	-0,02102776	0,105545984	-0,199228423	0,842084067	0,952074658
Armcx3	169,2653936	-0,614428922	0,172899948	-3,553667477	0,000379899	0,012087778
Ankrd61	0,66772892	1,030754335	2,00310075	0,514579377	0,60684698	NA
Tmem25	1020,075682	0,164290499	0,1710202	0,960649671	0,336728345	0,69270916
Galnt14	3,504216681	0,507136841	0,790547638	0,641500672	0,521197445	NA
Dpyd	11408,16483	0,354583195	0,144198288	2,458997267	0,013932568	0,128458275
Tecpr1	199,8417208	-0,026165788	0,144373574	-0,181236685	0,856181801	0,9578959
Mir3064	64,36174114	0,220051656	0,242737731	0,906540799	0,36464968	0,713511989
Camsap1	229,642923	-0,189469528	0,149198083	-1,269919321	0,20411337	0,557198625
Apbb1	15,86130325	0,080437982	0,401997204	0,200095874	0,8414056	0,951912623
Fam214b	328,2346247	-0,233898827	0,150245054	-1,55678221	0,119522202	0,429902963
Bcl7c	314,8297809	6,39E-05	0,135689744	0,000470733	0,99962441	0,999875741
Bcl7b	341,0882848	-0,21033084	0,12353361	-1,70262036	0,088639137	0,369089997
Kcnj5	5,629511327	-1,043199698	0,839592434	-1,242507263	0,214049463	0,567220469
Cd2ap	965,270769	0,183832755	0,104189892	1,76440104	0,077664483	0,345293141
Lipg	131,8451533	0,097538507	0,283895662	0,343571673	0,731168431	0,912061199
Atf4	3476,173877	-0,041947181	0,13216365	-0,317388185	0,750949071	0,920556312
Jup	1168,542363	0,04702614	0,130264965	0,361003746	0,718096647	0,907909407
Chrnb2	47,45862765	-0,130389296	0,249069656	-0,523505345	0,600622632	0,854422974
Cd24a	93,98272789	0,856435211	0,325158619	2,633899772	0,00844104	0,094990122
Cd19	30,01429558	0,68396058	0,411055537	1,663912827	0,096129851	0,385586553
AA413626	0,76301043	0,41195604	2,278685408	0,180786711	0,856534994	NA
Myo1c	916,8684504	-0,204205707	0,087847534	-2,32454683	0,020096205	0,158411651
Kmt2c	597,336117	-0,457597533	0,17174574	-2,664389425	0,007712822	0,089429935
F8	222,9891056	-0,198798104	0,295618318	-0,67248236	0,501276666	0,803641654
Trappc12	601,4285265	0,089183855	0,121374533	0,734782271	0,462472106	0,781516914
Grhl1	22,0436741	0,362035354	0,406764768	0,890036165	0,373446467	0,720823634
Gm21119	0,274277864	-1,699264605	2,746395376	-0,61872541	0,536097268	NA
C1ql4	10,11439639	-1,62025539	0,618223287	-2,620825557	0,008771713	0,097260878
Ankrd37	17,69171069	-0,598886368	0,46899424	-1,276958899	0,201616758	0,553080595
Sytl2	21,59843465	-0,007902299	0,381451094	-0,020716415	0,983471874	0,995431227
Fas	494,4772672	0,232148777	0,142843701	1,62519436	0,104121152	0,401498609
Zbtb24	302,0733342	-0,050165117	0,155362394	-0,322890986	0,746777803	0,919391514
Yes1	494,0113233	0,123161086	0,133125584	0,925149641	0,354888074	0,705239841
Ap2m1	2933,949248	-0,005204966	0,063318151	-0,082203382	0,934484984	0,98189048
Xrcc5	560,7340377	0,310645169	0,142521249	2,179641079	0,029284077	0,19731337
Ak4	1236,544851	-0,224381174	0,110693798	-2,027043771	0,042657936	0,243203202
Ctnna1	3304,650116	0,029347456	0,062814983	0,467204712	0,640353423	0,871049783
Psmb1	3449,563294	0,168260973	0,079427009	2,118435204	0,034138227	0,215993354
Clec10a	10,28908341	1,025483945	0,546291651	1,877173014	0,060494385	0,29803066
Ap1m2	6,781462541	0,78897442	0,605257127	1,303535943	0,192391854	0,541552905
Rps15a-ps6	7,729504059	0,184304552	0,550545838	0,334766952	0,737800892	0,91507318
Cebpa	5781,476692	0,088312619	0,141900117	0,622357616	0,533706744	0,82137064
Rgs6	0,550704411	0,497266989	2,126906625	0,233798223	0,815141629	NA
Hnf4a	13086,8566	-0,068305074	0,090257817	-0,756777374	0,449183251	0,773349269
Elk4	206,3445168	-0,216700525	0,23278161	-0,930917716	0,351896129	0,702880198
Elk1	153,5079744	-0,50740256	0,153968965	-3,295485947	0,000982516	0,023455819
Foxm1	16,30346646	-1,203983386	0,554445316	-2,171509709	0,029892664	0,199665228
Areg	0,306964509	-0,626838302	2,723438812	-0,230164268	0,817964126	NA
Gnai2	2064,26701	-0,019164176	0,095157247	-0,201394816	0,840389868	0,951657825
Elf1	595,5083493	0,210574566	0,141827309	1,484725107	0,137616658	0,460640235
Cfd	17,21016052	0,988804299	1,383033234	0,71495339	NA	NA
Sh3pxd2a	997,8439056	-0,028132912	0,223513878	-0,12586651	0,899837593	0,972184322

Supplementary Table S1: *Serpina1* KO vs. wildtype all DEGs

Acvr2b	152,8306238	0,324697593	0,185588277	1,749558746	0,080194484	0,350251568
Cd160	4,356144407	0,866300872	0,938162188	0,923402033	0,355797732	NA
Zpbp2	4,37081989	0,634713322	0,76053656	0,83455991	0,403965543	NA
Atl3	2363,014946	0,065933926	0,108671243	0,60672837	0,544031191	0,827477215
Arhgap19	77,56169739	0,12488127	0,298516674	0,418339346	0,675699026	0,889164486
Snora5c	2,058210534	-1,634003782	1,073340847	-1,522353115	0,127920626	NA
Cdyl	68,8930206	-0,182564817	0,200903261	-0,908720032	0,363497928	0,712361223
Ublcp1	587,4983663	-0,089410526	0,104181929	-0,858215307	0,390773585	0,734518115
Stx1b	54,37732934	-0,118250637	0,326893462	-0,3617406	0,717545884	0,907650451
Gtf3a	309,3800749	0,054537812	0,123892382	0,440203114	0,659790005	0,882334883
Uqcrfs1	4383,831944	0,087951789	0,092542232	0,950396233	0,341910957	0,695250579
Snrnp40	213,310232	-0,075153127	0,116179735	-0,646869498	0,517716408	0,813424627
Metap1d	765,285554	0,375352667	0,141444657	2,653706941	0,007961294	0,091595986
Asf1b	19,43916805	-1,121201254	0,382760514	-2,929250052	0,00339781	0,053800673
Mab21l2	21,96032502	0,001073702	0,373805011	0,00287236	0,997708192	0,999577399
Arhgap33	16,11215587	-0,011813259	0,481296014	-0,024544685	0,980418141	0,994739255
Agtpbp1	283,3082758	-0,504778666	0,163692179	-3,083706678	0,002044391	0,038697159
Terf2	243,2092068	0,177376529	0,124338921	1,426556768	0,153707693	0,488800701
Phldb1	117,0993128	-0,109357415	0,265570851	-0,411782446	0,680498889	0,891658301
Pilra	134,0049029	0,072536814	0,220116646	0,329538067	0,741749026	0,916899135
AF529169	0,113662148	0,780932884	3,352475198	0,232942181	0,815806304	NA
Kcna5	0,122496332	0,780932884	3,352475198	0,232942181	0,815806304	NA
Clec2e	0,670276518	0,764074527	1,833747907	0,416673701	0,676917093	NA
Nckap1l	175,7755988	0,336821857	0,263411222	1,278692132	0,201005491	0,55202186
Pank2	593,8039415	0,031109833	0,090785459	0,342674179	0,73184359	0,912276055
Tef	863,4664748	-0,363203147	0,163568496	-2,22049573	0,026385135	0,187330256
Dusp7	103,285032	-0,205187429	0,16939981	-1,211261272	0,225795286	0,581069718
Eif4g1	11090,66559	-0,227166308	0,111951462	-2,029149989	0,042443016	0,243203202
Nphp4	2,426003347	0,415882296	1,076106858	0,386469329	0,699149113	NA
Wasf2	422,6363848	-0,110481316	0,168199967	-0,656845052	0,511280553	0,809487175
Pde5a	18,46345055	0,025771356	0,338131417	0,076216982	0,939246473	0,982918677
Phc3	423,6165417	-0,063728713	0,11008665	-0,578895927	0,562659398	0,83722511
Pxylp1	189,7923936	-0,177274133	0,176692691	-1,003290697	0,315720623	0,67419376
Grwd1	204,8980705	-0,023691675	0,207549531	-0,114149497	0,909119286	0,9747592
Srsf10	599,3132263	-0,076470075	0,159193553	-0,480359118	0,630972059	0,866751905
Fam84a	52,68178563	0,01309288	0,313235528	0,041798836	0,966659063	0,990593599
Dpp8	1016,081527	-0,200522501	0,114885743	-1,745408061	0,080913863	0,351670169
Apoc2	189,047311	0,110066098	0,125172439	0,879315757	0,379230095	0,725074694
Cryl1	521,9047339	0,010726567	0,1788727	0,059967601	0,952181439	0,986187919
Lonrf3	351,9386029	-0,139041751	0,177373405	-0,78389289	0,433102962	0,763032854
Fryl	418,0933884	-0,322566125	0,12100675	-2,665687045	0,007683118	0,089240315
Oprl1	0,093303375	-0,517475177	3,352475198	-0,154356154	0,877328924	NA
Bub1b	20,81724737	-0,243903092	0,381436779	-0,639432551	0,522541579	0,815322684
Bub1	4,628769864	-0,965813683	0,787552642	-1,226348096	0,220067709	NA
Pnliprp2	0,093953095	-0,517475177	3,352475198	-0,154356154	0,877328924	NA
Cdh11	5,688886693	1,467818768	0,702154638	2,090449436	0,036577446	0,224108101
Btg3	0,454707671	-1,551721951	2,086851925	-0,743570702	0,457136233	NA
Pak1	81,27099102	0,676599077	0,227741377	2,970909749	0,00296919	0,048705649
Marco	320,5215182	0,425940114	0,605899355	0,702988228	0,482063086	0,793477208
Clcn2	1005,319142	0,110780983	0,112206302	0,987297337	0,323496896	0,680937786
Myl3	0,438629036	-0,108084369	2,215478345	-0,048786019	0,961089824	NA
Mapkapk5	751,5130473	-0,008208257	0,095455976	-0,085989972	0,931474389	0,980584977
Brca2	70,88476256	0,295889227	0,21302277	1,389002818	0,164831893	0,50334664
Fry	34,75087122	0,126564435	0,329784004	0,383779787	0,70114167	0,899833253
Fam171b	19,3978309	0,117394191	0,449148579	0,261370507	0,793806799	0,936746676
Gpr68	2,109066594	0,745349431	1,011211628	0,737085502	0,461070355	NA
Fam78a	30,57135043	0,655046092	0,323268848	2,026319874	0,042732016	0,243203202
Slitrk6	0,113662148	0,780932884	3,352475198	0,232942181	0,815806304	NA
Unc80	0,208973043	1,337107854	3,350237755	0,399108348	0,689813372	NA
F8a	139,9320794	0,010825332	0,13816086	0,0783531	0,93754718	0,982447738
Bnipl	0,890345455	-1,763026357	1,647341742	-1,070225025	0,284518033	NA
Kcnj1	0,086476712	0,780932884	3,352475198	0,232942181	0,815806304	NA
Picalm	2739,023875	-0,041829183	0,103682303	-0,403436093	0,686627439	0,893161227
Fbxw15	0,896506219	0,845462201	1,450903784	0,582714175	0,560085729	NA
Uchl5	487,8867688	0,052772992	0,113435088	0,465226349	0,641769372	0,872109649
Dpep2	1,370273496	0,620858194	1,258555501	0,493310143	0,621793463	NA

Ncor1	2145,887663	-0,158169628	0,120078624	-1,317217189	0,187765834	0,534203397
Nwd2	7,659094	0,216132627	0,537111866	0,402397788	0,687391298	0,893386271
Glt1d1	405,2105203	0,267170626	0,205197948	1,302014122	0,192911558	0,541594346
C030006K11Rik	541,5620792	0,023774087	0,09617753	0,247189617	0,804761481	0,94070615
Cbx1	410,9356822	-0,037549052	0,104868209	-0,35805944	0,720298832	0,908788588
Capn1	544,6712692	-0,031046585	0,130688014	-0,237562607	0,812220355	0,943005481
Zbed6	358,2735991	-0,200560405	0,163174729	-1,229114373	0,219028927	0,57359407
Zfp638	724,3407321	-0,074318828	0,107702924	-0,690035381	0,490171938	0,797285888
Zfp934	3,927204414	0,467025503	0,76584674	0,609815879	0,541983781	NA
Pqbp1	1055,459352	-0,105240796	0,115827234	-0,908601485	0,363560523	0,712361223
Cpeb1	57,66006553	0,57132274	0,256228511	2,229739144	0,025764766	0,184053149
Ncmap	11,04387837	2,210271915	0,740972535	2,982933658	0,002854999	0,047625442
Gimap4	217,0837673	-0,160308012	0,235621197	-0,680363287	0,496274461	0,800192636
Parp3	911,5486244	0,159376518	0,112822035	1,412636447	0,157762647	0,493680148
Ctu1	109,0876001	0,07629165	0,220664533	0,345735897	0,729541204	0,911594858
Apbb3	216,2027943	0,011835203	0,210965037	0,056100307	0,955261899	0,987017436
Soat2	995,3608416	0,15539073	0,16061559	0,967469778	0,333309242	0,689998647
Igflr1	50,29096607	0,193981852	0,285541073	0,679348332	0,49691718	0,80083943
Mypop	88,74286514	-0,442263715	0,212664777	-2,079628422	0,037559628	0,227107493
Ube2m	1024,794818	-0,069292123	0,113807933	-0,60885143	0,54262292	0,826611597
Usp11	31,21442625	-0,031321171	0,31146047	-0,100562269	0,91989795	0,977341679
Cald1	6544,715002	-0,16082058	0,136578371	-1,177496696	0,238997315	0,596444859
Mrps35	684,5565303	0,054748888	0,096475804	0,567488277	0,570382489	0,841196193
Mob1a	273,4302357	-0,06569572	0,203959188	-0,322102284	0,747375208	0,91954512
Mat2a	1290,887143	0,074240646	0,107849688	0,688371449	0,491218898	0,797760449
Eva1a	1615,545669	-0,01483212	0,139996573	-0,105946309	0,915624951	0,976559041
Krcc1	1287,156838	0,126962973	0,087930791	1,443896626	0,148768056	0,479794094
Hibadh	9007,082557	0,197531191	0,078001254	2,532410453	0,01132813	0,113783449
Sds	3083,447161	0,236474718	0,456468967	0,518052123	0,604421903	0,855714966
Eif4b	5494,101069	0,12581723	0,093534492	1,345142608	0,178579186	0,521158073
Zfp932	127,0642537	0,047239739	0,182135573	0,259365802	0,795353013	0,937578206
Parm1	3,209205201	0,398576722	0,939451389	0,424265403	0,671372268	NA
Slc2a9	876,4142398	0,233415743	0,133036129	1,754528973	0,07933991	0,349223642
9430015G10Rik	91,9587653	-0,383587554	0,195198117	-1,965119134	0,049400451	0,266538487
Rrp9	155,0932563	0,027386476	0,168251699	0,162770874	0,870698842	0,961775826
Fam76a	285,3178722	0,057899809	0,126893223	0,456287635	0,648183155	0,876446263
Gnl2	690,074762	0,158365795	0,110294778	1,435841283	0,15104751	0,483793521
Cyp26b1	255,3108241	-0,426561631	0,981519644	-0,434593066	0,663857829	0,883975629
Fam109a	184,0490777	0,253036317	0,156272912	1,619194994	0,105405315	0,403011718
Snora15	0,854452134	-0,435821314	1,70850824	-0,255088799	0,798654515	NA
Nim1k	79,38115761	0,195967137	0,210906364	0,929166539	0,352802785	0,703763121
G2e3	79,05101903	-0,059874444	0,20830547	-0,287435774	0,773778673	0,928621142
Ror1	87,19387688	-0,233208552	0,272295224	-0,856454801	0,391746263	0,734532481
Zfp770	168,4713692	-0,03516243	0,168367059	-0,20884388	0,834570115	0,950858008
Sestd1	67,62745084	0,285201122	0,196127672	1,454160545	0,145901801	0,475167156
Fstl4	0,575301521	-1,012195801	2,056709696	-0,492143253	0,622618077	NA
Hmga2-ps1	5,411950226	0,177858447	0,700290317	0,253978161	0,79951243	0,93892144
Ss18l1	117,2996673	-0,074477743	0,151455682	-0,491746116	0,622898833	0,863474234
Phtf1os	8,838432796	-0,48113969	0,646271584	-0,744485294	0,456582935	0,778260481
Zfp473	3,247626877	-0,906415175	0,754463021	-1,201404375	0,22959438	NA
Agbl2	1,248905064	0,443035046	1,526943341	0,290145046	0,771705275	NA
Zfp324	120,0244449	-0,204553308	0,263834679	-0,775308649	0,4381573	0,766215379
Clec16a	527,8437481	-0,337191756	0,128201788	-2,630164227	0,008534363	0,09560459
Fbxl5	519,0005163	0,370357732	0,156363834	2,368563888	0,017857296	0,148006317
Napepld	37,57702202	-0,07477069	0,253272566	-0,295218274	0,767827143	0,927586634
D630039A03Rik	471,2612893	0,151634379	0,195125648	0,77711147	0,437093007	0,76575019
Dgkb	0,208973043	1,337107854	3,350237755	0,399108348	0,689813372	NA
Slx4	93,8826375	-0,102543031	0,191486522	-0,535510439	0,592296941	0,85107672
Lrrc8d	1076,717405	-0,080196605	0,125422942	-0,639409379	0,522556649	0,815322684
Entpd3	0,582541814	-1,850680781	2,257838198	-0,819669356	0,412404624	NA
Elmod2	334,2460079	-0,088554575	0,147989597	-0,598383782	0,549583884	0,82961684
Slc35f1	2,715358111	-2,648591262	1,106183184	-2,394351406	0,016649788	NA
Fbxl21	34,24849942	0,044013186	0,298046169	0,147672376	0,882601333	0,966166219
Scfd2	255,5993142	-0,689280621	0,179361911	-3,842959846	0,000121559	0,005208741
Ubxn10	1,663025997	1,917994245	1,366725173	1,403350346	0,160512392	NA
8030462N17Rik	129,7841366	-0,127554284	0,166540694	-0,765904601	0,443733068	0,770142004

Pde12	247,4486158	-0,284296319	0,175709599	-1,617989687	0,10566482	0,403240888
Sec22c	254,0357711	-0,023948553	0,167049521	-0,143361998	0,886004294	0,96772371
Cggbp1	956,2026849	-0,125858108	0,119395073	-1,054131503	0,291822715	0,653716132
Lpp	1050,169495	0,044240751	0,132186323	0,334684782	0,737862883	0,91507318
B3gntl1	63,41941687	-0,070544656	0,194367046	-0,362945556	0,716645555	0,907405448
Creb3l2	269,9737305	-0,766471482	0,191674824	-3,998811448	6,37E-05	0,003211656
Jmjd4	194,5415447	-0,100972845	0,150348673	-0,671591194	0,501843986	0,803893049
Cd226	4,892346165	0,242372018	0,712030618	0,3403955	0,733558707	NA
Itih4	55860,64365	0,491442908	0,124760078	3,939103887	8,18E-05	0,003918075
Aar2	429,5327903	-0,085069772	0,084858216	-1,002493049	0,316105522	0,67419376
Nfkb2	177,400776	0,065983758	0,162097894	0,407061166	0,683963065	0,892293506
1700088E04Rik	7,189781251	0,478280074	0,539472787	0,886569417	0,375310766	0,721323983
Zmynd19	189,8679358	-0,162223609	0,19772373	-0,820455943	0,411956235	0,749099865
Defb8	0,122496332	0,780932884	3,352475198	0,232942181	0,815806304	NA
Tmem120a	1054,042278	0,244025382	0,137975872	1,768609096	0,076959132	0,342991041
Fmnl2	184,5630595	0,161904994	0,220056646	0,735742353	0,461887509	0,781516914
Akr1b10	40,9273273	-0,167954362	0,274068944	-0,612817927	0,539996736	0,826407188
Limd2	273,0739857	0,77574168	0,190613772	4,06970426	4,71E-05	0,002547842
Smim7	964,9081077	0,158569656	0,084887237	1,868003504	0,061761582	0,301302009
Trak2	467,3051476	0,027066156	0,10625955	0,254717401	0,798941375	0,938745583
Cdc42se1	443,319625	-0,050688023	0,146990565	-0,344838613	0,730215703	0,911763681
Fam175a	137,5827063	0,242211639	0,132787281	1,824057528	0,068143376	0,319428046
Sbf2	700,5118591	0,180940689	0,142996273	1,265352481	0,205745001	0,558725355
Ptger2	20,73960735	0,38985107	0,394952723	0,987082876	0,323602012	0,680937786
Slc4a1ap	301,784557	-0,175698536	0,114736672	-1,531319783	0,125690379	0,441385519
Dync1i1	0,122496332	0,780932884	3,352475198	0,232942181	0,815806304	NA
Cpt1c	8,526386229	-0,378246866	0,590330074	-0,640737925	0,52169297	0,814970006
Ipo9	681,4826109	-0,089343483	0,09360973	-0,954425176	0,339868468	0,694330921
Ush1c	1,198655364	0,784193271	1,455538915	0,538764895	0,590049089	NA
Ldlrad2	0,244992664	1,389394708	3,349408814	0,414817893	0,67827522	NA
Tspan2	28,98589967	-0,113219526	0,388690022	-0,29128488	0,770833451	0,927992136
Seh1l	994,8755955	0,060280183	0,11405506	0,528518271	0,597139669	0,853484526
Lrrc49	5,843795331	0,878231857	0,688359631	1,27583289	0,202014598	0,553542358
Sesn1	410,3158808	0,305302315	0,171989904	1,77511765	0,075878463	0,340773122
Eme2	199,0363237	-0,21174865	0,200954455	-1,053714634	0,292013587	0,653924564
Taok3	1173,619731	0,060756525	0,185009128	0,328397448	0,742611172	0,917092327
Hnrnpr	583,9013888	0,0662094	0,12578652	0,526363237	0,598635853	0,854045336
Mdfi	11,78546391	0,079586273	0,440021786	0,180868937	0,856470452	0,9578959
Gpbp1	1096,067371	0,128124675	0,086036992	1,489181249	0,136439647	0,459349518
Phf6	199,8414453	-0,091510954	0,169120796	-0,541098174	0,588439917	0,848843045
Zcchc8	462,9300712	0,062603491	0,108448507	0,577264664	0,56376068	0,837355044
Nfix	1914,148963	-0,263874108	0,160605732	-1,642993093	0,100384362	0,393294268
Pcolce	225,8733101	-0,02995842	0,243970842	-0,122795085	0,902269367	0,973248278
Snord1b	0,285269197	-1,521344816	3,321239936	-0,458065315	0,646905517	NA
Pdzk1ip1	199,6752053	1,081954187	0,240368615	4,501229032	6,76E-06	0,000590348
Bdnf	38,20101672	-0,707575688	0,358068292	-1,976091444	0,048144421	0,262604149
Ralgps2	385,8274875	0,120118159	0,156250908	0,768751749	0,442040698	0,769003232
Fut7	3,689671625	0,282253439	0,724192807	0,389749023	0,696722142	NA
Cldn12	2131,92826	-0,084507647	0,10659374	-0,792801219	0,427893646	0,75995385
Men1	724,8340703	-0,088790607	0,09787373	-0,907195503	0,364303421	0,713048566
Olfr1393	2,135455664	0,540343127	1,132667473	0,477053627	0,633323935	NA
Olfr1392	0,279910125	-1,713254196	3,310169948	-0,517572881	0,604756307	NA
Tspan8	30,04978669	1,425258756	0,322294582	4,422223758	9,77E-06	0,000808037
Ralgds	57,70897135	-0,25057393	0,229545431	-1,091609313	0,275004861	0,636740558
Dmwd	446,6606818	-0,046940912	0,134588226	-0,348774284	0,727258771	0,910689961
Tle3	450,7610755	-0,323861609	0,141000658	-2,296880117	0,02162561	0,16389613
Rfx2	24,31808047	0,073293157	0,312363721	0,234640428	0,814487828	0,944076707
Rfx1	193,1323987	-0,170564543	0,129775075	-1,314308949	0,188742257	0,536004306
Ctla2a	49,43960076	0,204732626	0,261805312	0,782003328	0,434212618	0,7641159
Capn8	59,60773321	-1,855293084	0,853050023	-2,174893655	0,029638086	0,198245973
Prph	4,12566012	2,158634857	1,227821291	1,758101829	0,078730185	NA
Ythdc2	164,8108389	-0,146550303	0,161731406	-0,906133863	0,364865004	0,713569421
Sephs2	21487,29854	0,170998412	0,094489711	1,809704045	0,070341695	0,324561524
3425401B19Rik	0,093953095	-0,517475177	3,352475198	-0,154356154	0,877328924	NA
Zfp994	117,6246587	0,114487815	0,204085831	0,560978754	0,574812021	0,843448788
Gimap8	140,3414756	0,431630652	0,298297291	1,446981469	0,147902114	0,478071412

Supplementary Table S1: *Serpina1* KO vs. wildtype all DEGs

Usp30	396,2308771	-0,02400305	0,103921522	-0,230972851	0,817335894	0,945157301
Mir146	0,12663974	0,780932884	3,352475198	0,232942181	0,815806304	NA
Havcr1	0,122496332	0,780932884	3,352475198	0,232942181	0,815806304	NA
Ovgp1	14,69465667	-0,12974218	0,382346659	-0,339331278	0,734360183	0,913675851
U2af2	1378,428732	-0,022728243	0,108718218	-0,209056433	0,834404185	0,95084595
Lrit2	124,0796795	0,057968011	0,179916567	0,32219385	0,747305843	0,91954512
Nacc1	792,6245989	-0,372374593	0,120336268	-3,094450235	0,00197178	0,038038569
Zcrb1	553,085675	0,044720702	0,104685883	0,427189428	0,669241361	0,886730269
Ccdc109b	2,2481545	0,800941353	1,050799651	0,762220802	0,445928233	NA
Dlgap4	399,1705067	-0,074429298	0,128605832	-0,578739677	0,56276484	0,83722511
Cxx1c	24,335747	0,002755151	0,342289835	0,008049175	0,993577757	0,99822248
Mblac2	278,4655154	0,00131902	0,186267976	0,007081305	0,994349983	0,998457633
Fam110a	104,1379117	-0,291381997	0,221894488	-1,313155634	0,18913051	0,536686321
Sord	29520,08479	-0,047384207	0,086265254	-0,549284962	0,582809906	0,846891345
Itpka	25,1959075	-0,105875222	0,317436722	-0,333531739	0,738732935	0,915522746
Arhgap1	381,9572508	-0,073234288	0,107033529	-0,684218197	0,493837393	0,799287181
Cacnb4	3,746645023	0,154861575	0,861906995	0,179673185	0,857409146	NA
Dennd1a	617,9975664	0,287078972	0,139742686	2,054339884	0,039942807	0,235069225
Rabgap1	524,1720288	-0,013877695	0,10531102	-0,13177818	0,895159746	0,970433882
Fam129b	232,386058	1,104359449	0,2351419	4,696565981	2,65E-06	0,000289923
Tubb4b	4124,216807	-0,244665693	0,172237022	-1,420517437	0,155457096	0,490513551
Ubqln1	4219,024663	-0,094183238	0,098536628	-0,955819573	0,339163398	0,694071514
9530068E07Rik	3398,782389	-0,149449099	0,073340085	-2,037754659	0,041574475	0,240791131
Unc5cl	2,314630946	0,676337135	0,99731947	0,67815495	0,497673453	NA
Unc5a	31,16466915	0,075866504	0,304814532	0,248893986	0,803442788	0,940233423
Olfr1371	0,12663974	0,780932884	3,352475198	0,232942181	0,815806304	NA
Fra10ac1	331,3623286	0,049471866	0,12786092	0,386919368	0,698815902	0,898938178
Prickle2	30,2620256	-0,022183906	0,304627662	-0,072823018	0,941946954	0,983128178
Slc25a2	4,326871218	-0,645068484	0,728687293	-0,885247335	0,376023248	NA
Ldah	1391,773462	-0,368153085	0,107035528	-3,439540994	0,000582702	0,016393311
Chrne	4,024472063	2,219413174	0,850042702	2,610943154	0,00902929	NA
Jade1	503,1461772	-0,007090609	0,18960619	-0,037396505	0,97016886	0,991983746
Ap4b1	386,321281	0,136436375	0,121190508	1,125800838	0,260249829	0,621855222
Pnpla5	23,66076424	1,963507782	1,243517551	1,578994828	0,114337243	0,418558216
Apol7a	3502,306628	0,208023965	0,143253261	1,452141216	0,146462344	0,476296539
Clta	2159,485796	-0,083425079	0,089383185	-0,933341983	0,350643425	0,702050628
Pira2	8,897634889	0,528959769	0,699834687	0,755835312	0,449747942	0,773622955
Cxadr	3040,574772	-0,389713334	0,106827062	-3,648076878	0,000264211	0,009198519
Cx3cr1	19,60466601	0,449316709	0,414198522	1,084785881	0,2780165	0,638507547
Cux1	615,3319017	-0,219232157	0,138510337	-1,582785536	0,113470355	0,41709793
Zfp933	120,6128282	-0,075058661	0,193377844	-0,388145091	0,697908665	0,898426898
Rundc3b	8,006328123	0,529716459	0,516249328	1,026086486	0,30485082	0,664100532
Dlgap3	1,270477196	0,831749074	1,582535608	0,525580005	0,599180052	NA
C2cd4c	2,493450474	-1,429936363	0,98273257	-1,455061537	0,145652226	NA
Ttc8	119,5254591	0,262026741	0,15068478	1,738906486	0,082051213	0,354079608
Rsl24d1	328,3276009	0,103309871	0,116494706	0,88682031	0,375175651	0,721201232
Dcaf13	454,1839544	0,023374792	0,096795549	0,241486224	0,809178292	0,94241441
Aars2	537,5277019	-0,163566343	0,094975728	-1,722190981	0,085034928	0,3613579
Them6	77,1849038	0,412350509	0,191467933	2,153627	0,031269435	0,205324695
Ska3	2,045944905	-2,273430169	1,276804817	-1,780562024	0,074984028	NA
Plekhg6	330,8571689	0,058309072	0,106392694	0,54805523	0,58365398	0,847033224
Papd7	452,1564053	-0,40616614	0,170250135	-2,38570231	0,017046543	0,144603579
Dscc1	1,754647819	-0,694755006	1,100180049	-0,631492097	0,527718815	NA
Gpt	6388,73515	0,291078039	0,10760962	2,704944415	0,006831582	0,083591067
Eid2	8,427635426	0,03035644	0,493639361	0,061495178	0,950964855	0,985972877
Orai3	538,9871189	-0,109345002	0,092119986	-1,186984568	0,235233708	0,592708165
Rgl1	339,7049711	-0,092694974	0,229111321	-0,404584871	0,685782681	0,893161227
Sh3bgr	1,137284321	-1,660120522	1,507990147	-1,100882871	0,270947638	NA
Crtam	0,20013886	1,337107854	3,350237755	0,399108348	0,689813372	NA
Hmgn2	355,5250226	0,102276296	0,151923407	0,6732096	0,500813955	0,803541005
Fbxl3	748,2954951	1,56E-06	0,129916017	1,20E-05	0,999990432	0,999996918
Fbxl8	59,24984661	0,048074272	0,203845232	0,235837118	0,813559065	0,943942377
Atxn10	1634,177941	0,043147627	0,098731132	0,437021499	0,662095757	0,883036529
Oat	16877,54203	1,318855301	0,264271055	4,990540108	6,02E-07	8,66E-05
Dbp	50,17863004	-0,4632517	0,311785729	-1,48580149	0,137331636	0,460262382
St6galnac6	1767,986373	0,17769768	0,099943912	1,777974026	0,075408121	0,339490966

Slc7a8	147,6444886	0,314061648	0,289215184	1,085909959	0,27751883	0,638252527
Phgdh	5,575496723	-0,233517527	0,898527274	-0,259889192	0,794949248	0,937443926
Chn2	868,9350553	-0,16853775	0,139757105	-1,205933317	0,227843203	0,583531758
Nt5c3	442,6070308	0,25130438	0,10056361	2,498959417	0,012455857	0,120146001
4931414P19Rik	111,0155223	-0,422323824	0,198539929	-2,127148056	0,033407783	0,213595723
Clec4a2	27,77375916	0,35772564	0,364679846	0,980930654	0,32662694	0,684457358
Ces2e	4444,785389	-0,099047825	0,250708988	-0,395070897	0,692790566	0,895476852
Rab3gap2	550,2744925	-0,169850357	0,142369975	-1,193020905	0,232861186	0,589632107
Adss	987,9396614	0,027240741	0,085232518	0,319605026	0,749267764	0,919838327
Arhgap6	148,3650791	0,327631881	0,34491767	0,949884304	0,342171043	0,695250579
Kit	55,255036	-0,063401909	0,395434571	-0,160334765	0,872617375	0,962860819
Tnrc18	624,7296568	-0,355582104	0,145175677	-2,449322856	0,014312509	0,130165943
Golph3l	179,7663026	-0,196614896	0,158002538	-1,244378089	0,213360443	0,566967951
Zfp740	726,8880286	-0,134554085	0,099738482	-1,349068919	0,17731483	0,520079413
Acyp2	21,85615874	-0,229533966	0,341654783	-0,671830099	0,501691865	0,803893049
Spata9	0,516816246	-0,704770893	2,113982034	-0,333385469	0,73884333	NA
Nhej1	72,48514833	-0,414008808	0,219977649	-1,882049429	0,059829305	0,2959149
Lrba	647,2937533	0,049304896	0,116383783	0,423640606	0,671827937	0,887904019
Gstt4	0,086476712	0,780932884	3,352475198	0,232942181	0,815806304	NA
Rsph9	11,09121917	-0,404225025	0,591441549	-0,683457267	0,494317944	0,799621608
Selenof	3663,292069	-0,058936435	0,093209592	-0,63230011	0,527190791	0,818299511
Smurf1	513,0192189	0,036362722	0,150830157	0,2410839	0,809490091	0,94241441
Brsk1	6,570743508	-0,951934059	0,647610727	-1,469917066	0,141584217	0,46748318
Israa	0,220592835	0,059593471	3,352475198	0,017775962	0,985817581	NA
Frs3	56,8749956	-0,223424169	0,239605583	-0,932466455	0,351095515	0,702558889
Slf1	47,6058703	0,600599913	0,229567534	2,616223216	0,008890841	0,097932422
Mgll	3098,009384	-0,481988689	0,237570152	-2,02882679	0,042475936	0,243203202
Vegfa	3444,117304	-0,200401774	0,113973472	-1,758319455	0,078693169	0,348014879
Olfml3	105,6483101	0,089711402	0,26428744	0,33944633	0,734273522	0,913675851
Mmadhc	1452,312504	-0,023116985	0,117869243	-0,196123982	0,844513126	0,953132002
Magi3	772,1440151	-0,074437903	0,122822786	-0,606059388	0,544475318	0,827477215
Golga2	1036,488379	-0,14329237	0,103176838	-1,388803662	0,164892461	0,50334664
Spg21	638,0848359	0,277852532	0,086439061	3,214432545	0,001307026	0,028598418
Commd7	596,9176035	0,06940386	0,090689366	0,765292152	0,444097596	0,770157058
Tm9sf4	1495,960621	-0,180801418	0,103882877	-1,740435225	0,081782625	0,353542587
Trmt12	101,1976782	0,122450365	0,239451646	0,511378253	0,60908622	0,857753956
Relb	45,45792302	0,542073052	0,269137963	2,014108478	0,043998146	0,247168435
Psmb6	3403,1521	0,022663637	0,098367165	0,230398393	0,817782209	0,945428084
P2rx3	32,87885007	0,516201811	0,291201127	1,772664194	0,076284368	0,342000574
Osbpl6	1,123265888	-1,709910133	1,59179732	-1,074200912	0,282732644	NA
Atp1a4	1,138198668	-0,317322346	1,434477569	-0,221211089	0,824928075	NA
Dok3	62,40175502	0,20387717	0,249775804	0,816240669	0,414362501	0,75133762
Pign	235,6464859	0,140758641	0,120183919	1,171193638	0,241520952	0,599385841
Ttpa	8393,040646	0,451576233	0,189729051	2,380111175	0,017307416	0,145675893
Aatk	64,34405631	-0,511184628	0,301587567	-1,694979114	0,090079383	0,372378253
Zfp329	127,2717912	-0,45548839	0,190382488	-2,392491001	0,016734438	0,143473504
Romo1	1123,257533	0,057826504	0,11908142	0,485604757	0,627247423	0,865852703
Polr3d	203,4951332	0,123071517	0,140419694	0,876454814	0,380782831	0,725866642
Caly	0,953389362	-0,267419748	1,637199689	-0,163339726	0,870250957	NA
Mrps18a	681,0608552	-0,078187847	0,098850513	-0,790970576	0,428961162	0,760782936
Dpm3	439,6502083	-0,05812373	0,111150465	-0,522928352	0,601024114	0,854422974
Uckl1	391,3806097	0,018951537	0,105555556	0,179540878	0,857513023	0,958260148
Boll	0,086476712	0,780932884	3,352475198	0,232942181	0,815806304	NA
Chst15	159,8919584	-0,098502591	0,186720327	-0,527540803	0,597818089	0,853597141
Mboat7	802,3668865	-0,156693934	0,073432423	-2,133852162	0,032854884	0,211070135
Bcl9	158,4437184	-0,088444891	0,185838023	-0,475924625	0,634128078	0,867989994
Spns3	2,643218617	1,071043187	0,872609424	1,227402728	0,219671262	NA
Tcaf1	61,14329057	0,132853408	0,230335974	0,576780977	0,564087422	0,837355044
Brinp3	0,087021394	-0,517475177	3,352475198	-0,154356154	0,877328924	NA
Prepl	559,4974154	-0,400037007	0,116687168	-3,428286198	0,000607405	0,016882316
Crb2	2,946277662	2,419859566	1,053188013	2,29765202	0,021581602	NA
Ptpn5	0,147722973	0,780932884	3,352475198	0,232942181	0,815806304	NA
Sec14l1	294,5600623	-0,350075896	0,125710971	-2,784768055	0,005356603	0,070973656
Lrriq1	0,113662148	0,780932884	3,352475198	0,232942181	0,815806304	NA
Pou2f2	18,49852894	0,480103257	0,422165065	1,137240612	0,255437714	0,615860558
Omp	2,754425283	0,045299934	0,977800205	0,046328415	0,963048491	NA

Supplementary Table S1: *Serpina1* KO vs. wildtype all DEGs

Perm1	1,966726785	-0,3398471	1,057817983	-0,321271812	0,748004416	NA
Arhgef10l	636,2072529	0,177777087	0,149334475	1,19046246	0,233864676	0,591139508
Ggn	4,289630848	-0,918972308	0,743699239	-1,235677354	0,216578518	NA
Champ1	232,5333584	0,198536988	0,133368953	1,488629724	0,1365849	0,459349518
Gpr135	16,83288003	0,361959918	0,364214638	0,993809366	0,320315686	0,677612453
Mthfd1l	31,81222952	-0,346013311	0,566493516	-0,61079836	0,541333079	0,826407188
Cebpzos	325,6162574	0,231716928	0,102823959	2,253530507	0,024225719	0,176862524
Lrwd1	206,01425	-0,151856785	0,132909637	-1,142556622	0,253222725	0,612785071
Cbr1	2550,815316	0,304226615	0,098378395	3,092412865	0,001985365	0,038104827
Cbl	51,07521556	-0,315863583	0,314319865	-1,004911295	0,314939565	0,673776132
Dag1	2723,372641	0,083782387	0,144919816	0,578129269	0,563176848	0,83722511
Zbtb7b	2095,68896	-0,179181053	0,098614512	-1,816984639	0,069219466	0,321794167
Gm6566	17,08156324	0,169269128	0,397934049	0,425369805	0,670567114	0,887361539
Kif13a	363,9130378	-0,127999968	0,162669694	-0,786870406	0,431357733	0,7622164
Scyl2	678,385562	-0,066610002	0,148554553	-0,44838748	0,653873576	0,878394288
Atp2a2	5377,203759	-0,689766857	0,11748842	-5,870934839	4,33E-09	1,70E-06
Kif11	24,11334432	-0,866965967	0,370925226	-2,337306565	0,019423249	0,155304678
Adam17	493,8733725	-0,127533304	0,09698205	-1,315019677	0,18850329	0,535621781
Ifnar2	2018,435728	0,034772275	0,09425413	0,36892044	0,712187028	0,905478699
Lyst	554,8619084	-0,731048862	0,208993173	-3,497955713	0,000468839	0,013963718
Gm40893	3,844353466	-0,121990657	0,895827411	-0,136176517	0,891681739	NA
Gm26839	1,856527507	1,497975374	1,12440634	1,332236685	0,182782425	NA
Nefh	2,872417984	0,391046949	0,903575659	0,432777206	0,665176634	NA
Neu1	1036,587862	0,104203534	0,103422108	1,007555695	0,313667808	0,672348265
Cldn3	2884,543241	0,174826037	0,106319461	1,644346538	0,100104642	0,392993389
Sep-02	1654,814241	-0,023140128	0,102855886	-0,224976218	0,821997775	0,946779723
Mmp24	4,273387811	1,050772132	1,189637108	0,883271147	0,377089789	NA
Entpd2	74,77884592	0,340227636	0,242989978	1,400171474	0,161461976	0,497950692
Ache	7,6349254	-0,170317738	0,591035644	-0,288168302	0,773217911	0,928621142
Mknk2	2216,782464	0,211252354	0,171861454	1,229201479	0,218996274	0,57359407
Gemin8	30,68183508	0,292918271	0,253873456	1,153796366	0,248583665	0,60747894
Kcnab3	17,27094598	-0,496764134	0,452727054	-1,097270707	0,272523072	0,634510789
Adam19	34,60065036	-0,267482381	0,280358476	-0,954072746	0,340046821	0,694376958
Asic1	0,82300329	0,52840345	1,754392013	0,301188928	0,763270432	NA
Abl2	183,2103503	0,099899402	0,126047771	0,792551917	0,428038933	0,75995385
Adipoq	1,098561176	2,712272072	1,918485486	1,413756889	0,157433296	NA
Colq	0,919312157	0,9365459	1,663132734	0,56312156	0,573352111	NA
Snord69	1,134849654	-0,705875888	1,552983601	-0,454528874	0,649448215	NA
Lhx1	0,113662148	0,780932884	3,352475198	0,232942181	0,815806304	NA
Lor	6,815369684	-0,227837065	0,567755505	-0,40129433	0,688203438	0,89386882
Igf2	8,823995959	0,842321699	0,568844211	1,480759903	0,138670565	0,462662037
Hao1	6373,916459	-0,666112033	0,156322512	-4,261139517	2,03E-05	0,001401589
Pglyrp1	2,920789353	0,349860561	1,065395324	0,328385674	0,742620074	NA
H2-Q2	15,55332162	0,728119784	0,443286016	1,642550763	0,100475915	0,393294268
F930015N05Rik	2,909571257	0,544732146	0,934041563	0,583199043	0,559759315	NA
Gm14326	94,51337835	0,120859635	0,207326281	0,582944112	0,559930924	0,835459433
Prr3	132,6671462	-0,122789023	0,147994125	-0,829688496	0,406714927	0,745871812
Vasp	400,8166309	-0,134662286	0,108239684	-1,24411197	0,213458356	0,566976917
Raly	1322,310188	0,058339117	0,08782735	0,664247716	0,506531778	0,806233823
Ppih	174,1364196	-0,10755911	0,157062517	-0,684817182	0,493459291	0,799062169
Lsm2	238,6104664	0,076428394	0,108261825	0,705958861	0,480213721	0,79208634
Banp	159,7202033	-0,56131534	0,247012865	-2,272413386	0,023061554	0,171378497
Gm14812	0,087021394	-0,517475177	3,352475198	-0,154356154	0,877328924	NA
Eif2ak4	276,412575	0,216960369	0,135577312	1,600270472	0,109538594	0,409719974
Mir6945	0,219943115	0,059593471	3,352475198	0,017775962	0,985817581	NA
Klf10	225,4876033	-0,040725624	0,289301612	-0,140772198	0,888049907	0,96836734
Orc3	557,647283	0,058402608	0,100321761	0,58215294	0,560463668	0,835469208
Prss30	2,606067376	0,441264335	1,037468727	0,425327842	0,6705977	NA
Hnf4g	8,098955109	0,60590879	0,61398494	0,986846337	0,323717975	0,680937786
Syn2	1,619823616	-0,970715718	1,296684105	-0,748613879	0,454089964	NA
Irf4	7,028342019	0,3814543	0,624408774	0,610904773	0,541262625	0,826407188
Sp1	979,5861837	0,029289879	0,120457154	0,24315599	0,807884559	0,942196162
Sod2	4525,564987	0,009512164	0,083178865	0,114357938	0,908954056	0,9747592
Kdm5c	1542,197747	-0,116161675	0,130917848	-0,887286776	0,374924523	0,720996253
Mapk12	55,99817016	-0,055913341	0,309473622	-0,180672398	0,856624725	0,9578959
Sh3gl1	500,2277812	-0,078355087	0,110963024	-0,706136909	0,480103001	0,79208634

Srsf3	962,4618608	-0,144141338	0,102071039	-1,41216685	0,157900839	0,493680148
Sult1d1	2143,196842	1,109730237	0,192877263	5,753556537	8,74E-09	3,07E-06
Ror2	1,705242586	2,589307376	1,317189842	1,965781464	0,04932386	NA
Sema4b	125,6321641	0,272244578	0,192014369	1,4178344	0,156239118	0,491473123
Taf5l	302,4485049	-0,009700585	0,118764996	-0,081678825	0,934902117	0,981974299
Lgals6	1,042056005	0,067543225	1,68777665	0,040019054	0,968077935	NA
Rnf103	2318,849245	0,431538551	0,160050512	2,696264736	0,007012191	0,084455842
Tlx1	0,20013886	1,337107854	3,350237755	0,399108348	0,689813372	NA
Tlr1	31,08851538	0,570067721	0,326656519	1,74515948	0,080957112	0,351743787
Tnfrsf26	15,73866221	0,682042928	0,517947271	1,316819233	0,187899224	0,534246008
Zfp446	76,77994275	-0,094718409	0,19614207	-0,482907157	0,62916166	0,866097574
Zfp384	699,5579642	-0,135716149	0,122885897	-1,104407846	0,269416259	0,629991376
Zfp747	77,18858887	-0,054016994	0,2308673	-0,23397421	0,815005	0,944076707
Rttn	34,5200304	0,172089204	0,265553561	0,648039529	0,516959384	0,813424627
Clspn	8,178092686	-1,224966025	0,644462095	-1,900757289	0,057333811	0,289352857
Ddi2	953,5191644	-0,15022625	0,173244146	-0,867136082	0,385867453	0,730226156
Lamp2	13980,56499	0,129005484	0,105118767	1,227235507	0,219734088	0,574490867
Zscan18	3,255867059	-0,438151117	0,861142409	-0,508802159	0,610890906	NA
Pate2	0,122496332	0,780932884	3,352475198	0,232942181	0,815806304	NA
Ceacam16	0,393546259	1,035669797	2,561579599	0,404309043	0,685985476	NA
Lamtor4	983,4562987	-0,032922279	0,112487235	-0,292675689	0,769770054	0,927934035
Drc7	0,12663974	0,780932884	3,352475198	0,232942181	0,815806304	NA
Parp4	291,097784	0,160342674	0,174218798	0,920352318	0,357388678	0,707149983
Kcnh3	0,944076443	0,296999693	1,908096054	0,155652381	0,876307034	NA
Prpf3	312,4820177	0,010247833	0,126835496	0,080796251	0,93560399	0,982081594
Megf11	2,231544394	-1,208284879	1,451482388	-0,832448873	0,405155625	NA
6430550D23Rik	27,48045534	0,068188283	0,293868875	0,232036423	0,816509724	0,944998904
Mgat4e	0,087021394	-0,517475177	3,352475198	-0,154356154	0,877328924	NA
Hbs1l	1323,070074	-0,00841174	0,084256038	-0,099835461	0,920474955	0,977341679
Hnrnpc	2751,89493	-0,031639752	0,100647212	-0,314362924	0,753245412	0,921533083
Abat	10963,90258	0,246735961	0,11295501	2,184373766	0,028934794	0,196360033
Itpr1	1169,760912	-0,136847982	0,147403289	-0,928391649	0,353204449	0,704249109
Snrpc	348,8225757	-0,087261848	0,117514453	-0,742562686	0,457746487	0,778822589
Cnn1	2,285639748	3,831582389	2,000362356	1,915444158	0,055435884	NA
Mettl1	240,2801143	-0,289249089	0,151320319	-1,911501983	0,055940102	0,285771198
Cd8b1	6,168486808	1,422135811	0,785718739	1,809980773	0,070298769	0,324561524
Myl4	10,38463601	1,095419967	0,462729209	2,367302403	0,017918285	0,1484198
Cd8a	6,136054474	0,557348528	0,671759968	0,829684046	0,406717444	0,745871812
Cd83	43,50613292	1,01838671	0,380736447	2,674781252	0,007477802	0,087925013
Myh4	0,093953095	-0,517475177	3,352475198	-0,154356154	0,877328924	NA
Cd7	14,20773298	0,085675834	0,389132107	0,220171589	0,825737522	0,947537943
Sec61g	814,899774	-0,274012646	0,162723829	-1,683912235	0,092198631	0,377058275
Sec22b	1279,660495	-0,36767927	0,125032178	-2,940677151	0,003274957	0,052350793
Myd88	370,5873652	-0,022708648	0,100999574	-0,224839051	0,822104485	0,946779723
Cd44	172,7145626	0,347228596	0,224313497	1,547961229	0,121631631	0,433672149
Clk1	1143,982507	0,323851122	0,121021559	2,67597877	0,007451136	0,087765854
Trpm7	963,6206079	-0,012780183	0,132138315	-0,096718222	0,922950169	0,977495112
Prorsd1	343,3932462	0,041190543	0,103837254	0,396683665	0,691600747	0,895323946
Pgm3	398,1009521	-0,524934558	0,167346001	-3,136821637	0,0017079	0,034595324
Kirrel3	2,929178441	0,622030441	0,814078357	0,764091608	0,444812653	NA
Rab17	470,8636799	0,316465854	0,141889415	2,230369714	0,025722908	0,183996556
Nectin2	1302,638185	-0,137075937	0,14710337	-0,931834101	0,351422268	0,702791968
Cdkn1c	37,2727725	0,752356379	0,359826868	2,090884381	0,03653843	0,223971694
Cdc45	36,36278662	-0,692274271	0,277425782	-2,495349444	0,012583314	0,121026135
Aoc1	2,375011505	1,643145238	1,206241728	1,362202285	0,173134055	NA
Mpp7	100,9971088	0,041788158	0,253914116	0,164575955	0,869277758	0,961775826
Slk	760,4660905	-0,078462336	0,09774518	-0,802723333	0,422134664	0,755522295
G630025P09Rik	9,312743359	0,328156397	0,511755928	0,641236142	0,521369271	0,814845193
Slc7a9	0,206965523	0,059593471	3,352475198	0,017775962	0,985817581	NA
Spp1	1566,269374	0,778964507	0,23357423	3,334976237	0,000853067	0,021158908
Cep250	276,9897389	-0,237648019	0,141290917	-1,681976622	0,092573373	0,37789723
Notch3	18,94650521	0,17283677	0,388122886	0,445314553	0,656092452	0,879592859
Agps	261,9228334	-0,081164258	0,115816477	-0,700800614	0,483427455	0,794038919
Spout1	339,105212	-0,348645542	0,124631616	-2,797408509	0,005151435	0,070015871
Dpp9	1619,055289	-0,279083343	0,092018215	-3,032914119	0,002422045	0,043001756
Mir7042	0,172953423	1,307385949	3,350719078	0,39018071	0,696402925	NA

Igf2bp1	0,113662148	0,780932884	3,352475198	0,232942181	0,815806304	NA
Ap1s2	62,63753018	-0,046252879	0,274822229	-0,168301082	0,866346422	0,961775826
Kpna3	911,4861516	-0,040199318	0,132468546	-0,303463117	0,761536951	0,92534041
Samd11	4,800198912	0,946058311	2,661655185	0,355439847	0,722260102	NA
Psmg4	111,6487961	0,261733366	0,166737462	1,569733421	0,116477143	0,423147532
Trim3	249,4597248	-0,201867829	0,152724341	-1,321779011	0,18624175	0,532805747
C77080	1777,382421	0,323103778	0,176064191	1,835147608	0,066483822	0,316036222
Slc10a5	1103,20935	-0,232347008	0,15673711	-1,482399466	0,138234036	0,461666457
Tmem53	421,90248	0,341791135	0,153295111	2,229628417	0,025772122	0,184053149
Mir219a-1	0,122496332	0,780932884	3,352475198	0,232942181	0,815806304	NA
Kiz	353,2833343	0,114338408	0,115885359	0,986651026	0,323813746	0,680937786
Chd2	842,9247045	0,006435417	0,136545253	0,047130287	0,962409388	0,988526411
Cd209b	5,054227061	-0,537250792	0,729002358	-0,736967153	0,461142325	NA
Setd6	439,3256191	0,06408412	0,123591519	0,518515512	0,60409864	0,855575199
Plekhs1	5,011188757	1,921493714	0,805620426	2,385110471	0,017073993	NA
Itpripl1	141,8321943	0,449419383	0,188889359	2,379273167	0,017346816	0,145855081
Dut	224,4137203	-0,089497993	0,11912167	-0,7513158	0,452462623	0,774936437
Mir30b	0,122496332	0,780932884	3,352475198	0,232942181	0,815806304	NA
Mfsd2b	10,73757185	-0,136494323	0,614749876	-0,222032291	0,824288746	0,947473126
Arfgef1	1715,435923	-0,13911379	0,128255275	-1,084663305	0,278070806	0,638507547
Magt1	2049,205515	-0,34395783	0,143991405	-2,388738621	0,016906325	0,14387057
Gas2l1	475,2764288	-0,317761428	0,113454059	-2,800793822	0,005097708	0,069898722
Pkib	16,63126707	1,012105514	0,395718418	2,557640654	0,010538493	0,10854323
Usp34	1084,393021	-0,233265454	0,112852501	-2,066994096	0,03873471	0,230871307
Apol9a	1872,783463	0,318079282	0,103122044	3,084493589	0,00203899	0,038665614
Hps5	361,2940908	-0,044010838	0,102235938	-0,430483045	0,666844298	0,885394917
Fam183b	0,695249958	-2,970774407	2,12563357	-1,397594792	0,162234783	NA
Exoc7	837,3646409	-0,031971398	0,085216801	-0,375177169	0,707528709	0,902731692
Spast	663,4169655	-0,108629534	0,118662258	-0,915451438	0,359954685	0,709765172
Akt1	1430,981908	-0,026728252	0,083805238	-0,318932957	0,749777352	0,920166276
Mia2	7850,442906	-0,003315936	0,120136784	-0,027601336	0,977980116	0,994418631
Gp6	0,320676396	1,743349119	3,194451911	0,545742797	0,585242749	NA
Angel2	469,9763834	-0,007790781	0,090639952	-0,085953059	0,931503732	0,980584977
Milr1	12,54980725	0,802817341	0,524449677	1,530780504	0,12582365	0,441505609
Arhgef2	93,45634868	0,386189766	0,247821653	1,558337464	0,119153272	0,429289976
Nono	2538,454527	-0,055824848	0,078524821	-0,710919772	0,477133953	0,789944745
Nuggc	0,434562287	-1,460519177	2,986529069	-0,489035648	0,62481646	NA
Slc4a2	754,0420539	-0,184488193	0,069203711	-2,665871394	0,007678906	0,089240315
Aqp9	8899,784065	0,204359407	0,110624965	1,847317256	0,064701173	0,309828757
Ifitm7	0,113662148	0,780932884	3,352475198	0,232942181	0,815806304	NA
Fer1l5	0,501802401	-1,56454058	2,384681684	-0,656079422	0,511773024	NA
Pomgnt2	83,71095478	-0,082114471	0,179948572	-0,456321881	0,648158532	0,876446263
Mir6946	0,285269197	-1,521344816	3,321239936	-0,458065315	0,646905517	NA
Ly6a	252,7856211	1,029410205	0,303855521	3,387827877	0,000704485	0,018650009
Ndufs8	1840,506946	0,044016011	0,070130197	0,627632786	0,530244526	0,819425454
Thoc6	140,9879411	-0,12977065	0,182756336	-0,710074695	0,477657818	0,790092068
Snx18	508,6416507	0,108875921	0,106138365	1,025792337	0,304989479	0,664202396
Steap3	925,0976639	0,022450857	0,10540625	0,2129936	0,831331954	0,949272028
Lad1	265,8230501	0,601357461	0,187386041	3,209190281	0,001331094	0,028782722
Helb	249,6290638	-0,187374883	0,138441013	-1,353463674	0,175907547	0,518682841
Slc8a3	2,30789453	0,488384746	1,125477401	0,433935631	0,664335185	NA
Glra3	0,401022643	-0,105719668	2,585000526	-0,040897349	0,967377731	NA
Cd34	42,54793591	0,339400325	0,282902081	1,199709536	0,230252168	0,586778733
Rtkn	277,8741362	-0,317465015	0,176233017	-1,80139352	0,071640877	0,328339692
Higd1b	0,113662148	0,780932884	3,352475198	0,232942181	0,815806304	NA
Rdh1	4,183375254	-0,141720126	0,718588952	-0,197220018	0,843655366	NA
Adcy4	102,38558	0,041003654	0,212154022	0,193273047	0,846745134	0,953824301
Apoa5	23100,34404	-0,002639487	0,106776944	-0,024719637	0,980278592	0,994739255
Fbxw7	223,0214226	-0,283946718	0,146194775	-1,942249434	0,052106911	0,275125315
Nelfb	622,658084	-0,105818077	0,082541986	-1,281990926	0,199845829	0,550139839
Hist1h4j	1,428675517	0,317605976	1,1877969	0,267390811	0,789168274	NA
Abcg5	1196,023068	0,179715566	0,245236077	0,732826787	0,463664081	0,781663619
Mfsd12	106,110612	-0,197273401	0,185785949	-1,06183165	0,288312119	0,649625922
Tmtc4	87,13391016	-0,071763746	0,218020296	-0,329160848	0,742034114	0,916899135
Tdrd3	232,1574103	0,045004507	0,128190954	0,351073973	0,725532853	0,910252319
Gpm6a	33,74425767	1,051240714	0,481130217	2,184940121	0,028893237	0,196177597

Supplementary Table S1: *Serpina1* KO vs. wildtype all DEGs

Catsper3	2,877643211	1,167433287	0,948950604	1,230236096	0,218608707	NA
Racgap1	19,78442624	-1,266075185	0,377182254	-3,356666895	0,000788881	0,020253442
Artn	0,79773776	2,187341907	1,972591981	1,108866876	0,267487615	NA
Bbs12	11,31762085	0,37000066	0,494248209	0,748613052	0,454090463	0,77650747
Mtif3	679,6237343	0,080540674	0,088124322	0,913943747	0,360746405	0,710581841
Btbd8	10,82101999	-0,6812853	0,492952661	-1,382050154	0,166956302	0,505901813
Rnf6	826,3607746	-0,060121892	0,099191833	-0,606117355	0,544436827	0,827477215
Xcr1	7,873987079	0,568869299	0,545661986	1,042530567	0,297165761	0,658144463
Tssk4	2,942004137	-0,454184228	0,877101689	-0,517823913	0,604581133	NA
Cited1	0,340986444	1,884176617	3,333184127	0,565278288	0,571884494	NA
Asap1	461,9028367	0,125719529	0,172170104	0,730205342	0,465264678	0,781663619
Spc25	71,85538832	-1,33397146	0,264508186	-5,043214271	4,58E-07	6,95E-05
Atp9a	1216,799988	-0,161613092	0,125491896	-1,287836883	0,19780275	0,547499994
Mup8	66,02659402	-1,959871552	1,086047959	-1,804590244	0,071138826	0,326823011
Mup7	63378,49556	-1,658849766	1,13835429	-1,457235046	0,14505151	0,473320389
Mup13	21,77235586	-1,597552699	0,671244909	-2,379984829	0,017313351	0,145675893
Tomm5	360,5252401	0,192199293	0,135248096	1,421086869	0,155291507	0,490222469
Plcxd2	1978,79227	-0,558932424	0,272500918	-2,051121247	0,040255141	0,236119007
Fmo5	24109,7067	-0,132544821	0,10630121	-1,246879704	0,21244161	0,565312874
Palm	107,6966464	-0,302241236	0,208686152	-1,44830518	0,14753172	0,47753781
Olfr20	8,96916657	0,068619339	0,467769362	0,146694813	0,883372913	0,966395158
Ifit1bl2	1,748070169	2,010075866	1,36571545	1,471811618	0,141071755	NA
Akap14	0,213116452	1,337107854	3,350237755	0,399108348	0,689813372	NA
I830077J02Rik	9,39257344	0,554233455	0,518864621	1,068165823	0,285445718	0,646939988
Elovl1	1576,195937	-0,187616719	0,09905336	-1,894097471	0,058212073	0,291583817
Dnaaf3	6,296403395	0,243907146	0,684901827	0,356119865	0,721750801	0,909525085
Hmcn1	36,88463508	-0,327028388	0,24193659	-1,351711156	0,176467735	0,519614321
Pkp2	923,1887899	-0,077330479	0,082106279	-0,941833928	0,346277672	0,698497639
Plxdc2	16,54355241	0,705219698	0,545928327	1,29178074	0,196433087	0,545402687
C1qtnf4	5,578697554	1,815620741	0,79997566	2,269594978	0,023232169	0,172167884
Map1lc3b	2096,85498	0,111614245	0,080428648	1,387742399	0,1652155	0,503431976
Retsat	7439,047408	-0,461062457	0,193040462	-2,388423917	0,016920811	0,143902245
Mapk13	5,995371736	1,087761846	0,71874636	1,513415449	0,130174163	0,448469369
Ccdc142	87,84239284	-0,215386632	0,185573586	-1,160653498	0,24578284	0,605051632
Wdr18	1224,08927	-0,140048585	0,086769568	-1,614028844	0,10652117	0,405028874
Tssk6	0,411908937	-0,918926577	3,055071653	-0,300787242	0,763576737	NA
Gpr21	0,533707999	-0,246596291	2,124736657	-0,116059696	0,907605231	NA
Cyp2r1	460,1504361	0,265266723	0,104643999	2,53494444	0,011246516	0,113475227
Cog3	431,1577717	-0,353763821	0,103575546	-3,4155149	0,000636615	0,017333833
Arhgap32	228,6621023	0,049647349	0,169187004	0,293446589	0,769180819	0,927740953
Cyp3a44	77,40648607	6,338338057	1,99621706	3,175174776	NA	NA
Rnf150	27,27636657	-0,013558287	0,373468171	-0,036303729	0,971040177	0,992115867
Fam166b	1,003700879	1,519282762	1,701328429	0,892997928	0,371858282	NA
Mycbpap	3,007771389	0,266630337	0,92477823	0,288318138	0,773103225	NA
Gm11992	19,34544812	0,978292738	0,401717431	2,435275799	0,014880443	0,133246243
Wdr93	18,67248797	-0,038625309	0,355354316	-0,108695203	0,913444247	0,976232707
Lrif1	199,8995019	-0,003337007	0,193240488	-0,017268673	0,986222277	0,996004319
Gm11437	194,555295	0,247895653	0,196578033	1,261054702	0,207289139	0,559794761
Cldn10	0,992098592	0,17637321	1,421457367	0,124079142	0,901252613	NA
Kpnb1	3353,355836	-0,131015024	0,110694845	-1,183569331	0,236583587	0,593857674
Impact	197,4902018	-0,14072033	0,213683975	-0,658544143	0,510188542	0,808610978
Lrp1	13099,33599	0,017255548	0,137931334	0,125102448	0,900442445	0,972376014
Pml	570,8880136	0,039927999	0,154906997	0,25775465	0,796596266	0,937923049
Igbp1	1375,587301	0,074042119	0,085772011	0,863243365	0,388003676	0,731971786
Eci1	2483,21829	-0,009065834	0,140071686	-0,064722818	0,948394695	0,985571619
Rapsn	0,270219305	1,389394708	3,349408814	0,414817893	0,67827522	NA
Gpr83	0,086476712	0,780932884	3,352475198	0,232942181	0,815806304	NA
Cnot11	469,2356266	0,084411434	0,094441384	0,893797091	0,371430466	0,71913887
Utp14a	348,9370228	0,105531855	0,140307171	0,752148694	0,451961644	0,774656859
Sgk3	259,8709172	0,038919137	0,194187503	0,200420399	0,841151807	0,951786586
Zc3h10	189,4750268	-0,000526342	0,17513223	-0,003005396	0,997602044	0,999545959
Csnk1g2	1703,265166	-0,007626046	0,083239995	-0,091615163	0,927003804	0,978888597
Mir25	1,998708246	-0,428295857	1,052477187	-0,406940752	0,684051504	NA
Ssx2ip	184,1475785	-0,21115774	0,147975784	-1,426974975	0,153587108	0,488738507
Mtfr1l	1656,400953	-0,024298997	0,069474968	-0,349751823	0,726524958	0,910393262
Serinc2	232,035993	-0,249864506	0,471271467	-0,530192307	0,595978604	0,853075785

Slc4a9	13,50890666	1,636252261	0,755267868	2,166452899	0,030276595	0,201176841
Dido1	922,3522985	0,057095674	0,10730132	0,532105975	0,594652599	0,852263143
Gm7030	0,213661134	0,059593471	3,352475198	0,017775962	0,985817581	NA
Zfp516	125,3103493	-0,1036615	0,293281779	-0,353453598	0,72374841	0,910252319
Rspo2	0,142634598	-0,517475177	3,352475198	-0,154356154	0,877328924	NA
Sacs	1,968534584	-1,56160999	1,257235213	-1,242098514	0,214200217	NA
Oasl1	539,4328763	0,130896455	0,206394674	0,634204615	0,525947296	0,817603419
Dusp22	311,0111458	0,070725686	0,109206917	0,647630095	0,517224228	0,813424627
Snord42a	0,401567325	-1,129138825	2,515782745	-0,448822072	0,653560015	NA
Snord4a	1,298706037	-2,084965303	1,501752447	-1,388354856	0,165029016	NA
Cnpy4	160,5320204	-0,365101364	0,13972567	-2,612987028	0,008975471	0,098435661
Snord42b	2,658140196	0,382469233	1,07154818	0,356931438	0,721143134	NA
2810006K23Rik	219,8607365	0,081244793	0,145833769	0,557105489	0,577455358	0,84496439
Mlx	987,9518711	-0,113338823	0,120417511	-0,941215465	0,346594453	0,698497639
Lrrc9	1,180384877	1,930217736	1,544115186	1,250047765	0,2112821	NA
1500009L16Rik	0,12663974	0,780932884	3,352475198	0,232942181	0,815806304	NA
Ptprr	0,086476712	0,780932884	3,352475198	0,232942181	0,815806304	NA
Mttp	7660,246172	-0,212459146	0,14361481	-1,479367948	0,139042002	0,463554245
H2afy	1709,106692	-0,102272359	0,075806757	-1,349119297	0,177298651	0,520079413
Gaa	2793,458869	-0,016125434	0,078031014	-0,20665416	0,83627996	0,951141594
Cyp4a10	6792,901614	1,004164318	0,436623991	2,299837709	0,021457416	0,163367723
Ccl6	178,4580432	0,485823351	0,329799961	1,473084924	0,140728138	0,466258507
Rpl37a	3027,04356	0,082857775	0,136453485	0,607223591	0,543702538	0,827311545
Afdn	1300,222061	-0,229520047	0,122962113	-1,866591597	0,061958642	0,301977792
Enah	18,8248292	0,269276011	0,38756207	0,694794542	0,487184069	0,795646692
Scn7a	3,314974919	0,614878601	0,831773749	0,739237805	0,459762609	NA
En2	1,044915036	2,609049666	1,535308979	1,699364559	0,089250515	NA
Egln2	1379,278255	0,012548547	0,072388269	0,173350565	0,86237588	0,960918748
Zmynd15	91,44794372	-0,170760098	0,238470016	-0,716065278	0,473951043	0,787826089
Egln1	640,0952841	-0,133748254	0,096525698	-1,385623282	0,165861965	0,504089882
Slc24a3	9,041317356	0,170463933	0,527876409	0,322923946	0,746752841	0,919391514
Krt25	0,113662148	0,780932884	3,352475198	0,232942181	0,815806304	NA
Ttyh2	1587,275074	-0,162439075	0,096653307	-1,680636505	0,092833541	0,378677779
Cpsf1	1140,687662	-0,163573296	0,115919059	-1,411099242	0,158215353	0,493856377
Foxp1	385,5089011	-0,083777536	0,151278805	-0,553795594	0,579718744	0,84530168
Pi15	3,395858694	0,078822091	1,158905801	0,068014235	0,945774303	NA
Pigf	110,8448726	0,061055257	0,210420584	0,290158196	0,771695216	0,928273961
Pcsk7	675,9746318	-0,087778916	0,09628607	-0,911647091	0,361954524	0,71109038
Pde8a	850,8738648	-0,230092167	0,121294199	-1,896975857	0,057831125	0,290515036
Prkcz	506,8229658	0,011248701	0,151401525	0,074297144	0,940773949	0,983128178
Oas1g	31,43239801	0,69870037	0,453354711	1,541178138	0,123273437	0,43588421
Dnm1	8,553252565	0,390755166	0,601566719	0,649562474	0,51597488	0,813164632
Hipk1	1242,848181	-0,14962511	0,124961226	-1,197372292	0,231161478	0,588028724
Mdm4	759,4959897	0,069212902	0,100986001	0,685371248	0,493109683	0,798745371
D030018L15Rik	0,122496332	0,780932884	3,352475198	0,232942181	0,815806304	NA
Esr1	727,5089329	0,050128107	0,24663626	0,203247111	0,838941892	0,951218231
4930556N13Rik	0,180324769	-1,201310802	3,342952034	-0,359356278	0,719328578	NA
4930554I06Rik	0,086476712	0,780932884	3,352475198	0,232942181	0,815806304	NA
Tmem183a	2350,562259	-0,008043945	0,077370063	-0,103967147	0,91719542	0,976907366
Srpk1	432,1331233	-0,170122179	0,10530686	-1,615489988	0,106204627	0,404170127
Hnrnpu	4743,03687	0,112870616	0,082861642	1,36215761	0,17314815	0,51532004
Phf20l1	527,8972644	-0,120974763	0,159046182	-0,760626637	0,446880105	0,772195705
Sclt1	114,9968667	0,061122349	0,168485939	0,362774186	0,716773577	0,907438063
Mir374b	0,361948979	-2,086101604	3,030153327	-0,68844754	0,491170995	NA
Efr3b	14,8231727	0,411428023	0,540288709	0,761496615	0,4463605	0,771778779
Zc3h7b	893,635339	0,087102914	0,116273806	0,749118976	0,453785499	0,776283856
Mical3	426,0734032	-0,439976874	0,138719883	-3,171692951	0,001515532	0,031608644
Pip5k1c	834,4372662	0,003057572	0,115732123	0,026419391	0,978922828	0,994593468
Ick	506,0068798	-0,410539992	0,132509627	-3,098189921	0,001947066	0,037725111
Adam15	72,03463043	0,246542512	0,228564909	1,078654258	0,280741884	0,641688878
Cntln	24,13163147	0,01194096	0,392056908	0,030457211	0,975702418	0,993311424
Bcl2	29,55176355	-0,060026321	0,281187608	-0,213474277	0,830957051	0,948982362
Sfmbt2	2,684447013	1,588824045	0,966262169	1,64429913	0,100114429	NA
Fam109b	3,890826084	-0,031217771	0,683132297	-0,045697987	0,963550968	NA
Mia3	4920,399912	-0,302606907	0,098222337	-3,080835969	0,002064203	0,038918301
Myo1d	460,4367474	0,358308265	0,150535524	2,380223988	0,017302117	0,145675893

Supplementary Table S1: *Serpina1* KO vs. wildtype all DEGs

Htatip2	1177,894245	0,511429344	0,150679552	3,394152282	0,000688414	0,01833348
Ccdc151	56,95799384	-0,278027371	0,269636919	-1,031117595	0,302485678	0,662312842
Fgd6	1030,428129	-0,214162869	0,135357253	-1,582204602	0,11360287	0,417177269
Cox6c	4945,297971	0,096578408	0,098038	0,985111987	0,324569066	0,681829643
Car7	37,61004953	0,162759198	0,269762839	0,603341804	0,546281328	0,828173482
Chrac1	302,91314	0,232145335	0,115165456	2,015754927	0,043825609	0,246904579
Snora20	1,206228722	-0,535986726	1,546369127	-0,346609821	0,728884467	NA
Tmem9	363,7099923	0,388030948	0,141948973	2,733594617	0,006264712	0,079012204
Efcc1	6,922140212	-0,773971259	0,568913514	-1,360437465	0,17369153	0,516247679
Folh1	0,087021394	-0,517475177	3,352475198	-0,154356154	0,877328924	NA
Zfp217	303,9646784	-0,713503552	0,175317455	-4,069780466	4,71E-05	0,002547842
Efl1	291,0966069	0,008985415	0,117222964	0,076652346	0,938900115	0,982918677
Gng12	1163,799781	0,014076476	0,088246237	0,159513611	0,873264235	0,963097638
Bfar	1183,175734	-0,164036649	0,097732142	-1,678430924	0,093263005	0,380045721
Impdh1	78,5462695	-0,030415928	0,237406069	-0,128117735	0,898055801	0,971603788
Impdh2	1007,253104	-0,117927538	0,10923839	-1,079542989	0,280345741	0,6416138
Epb41l4a	10,58357337	1,670821051	0,520249413	3,211577007	0,001320086	0,028696305
Epb41l2	304,7358095	-0,004072138	0,2131549	-0,019104127	0,984758039	0,995554769
Nr4a2	5,902345157	0,035941457	0,634802405	0,056618338	0,954849227	0,986886688
Bcap31	3013,498004	-0,100674793	0,08774965	-1,147295668	0,251259444	0,610767125
Cd52	186,7491616	0,469422467	0,255640929	1,836257089	0,066319643	0,315525732
Lamc3	42,8516257	0,740132042	0,294361954	2,514360404	0,01192485	0,116510842
Aadat	3068,875173	-0,462729368	0,096866167	-4,776996782	1,78E-06	0,000208665
Insrr	0,086476712	0,780932884	3,352475198	0,232942181	0,815806304	NA
Dnajc18	216,1137853	-0,331783061	0,144929546	-2,289271368	0,02206359	0,166085663
Dapl1	0,810478987	1,406683651	1,921998211	0,731886035	0,46423813	NA
Gpr84	1,795693282	0,489448124	1,262974754	0,387535952	0,698359474	NA
Arpc2	3173,184262	-0,052933667	0,071289563	-0,742516356	0,457774546	0,778822589
Pelp1	347,6856977	0,042911841	0,098567865	0,435353255	0,663306035	0,88377495
Mfsd2a	293,4858025	2,205725741	0,753063898	2,929002101	0,003400521	0,053800673
Spatc1l	2,890719592	-0,607503262	1,195864724	-0,50800333	0,611451008	NA
Styxl1	2,478121101	0,657533405	0,908027641	0,724133688	0,468983664	NA
Mgrn1	1299,349816	-0,113742256	0,106223845	-1,070778937	0,28426884	0,645517011
Tspyl2	327,3677637	-0,755629149	0,179657059	-4,205953013	2,60E-05	0,001682953
Atg2b	798,9346965	-0,113168726	0,116389034	-0,972331518	0,330885649	0,687536562
Tbc1d22a	372,9697344	0,023503419	0,103680523	0,226690782	0,820664195	0,94671323
Cstf3	347,2792484	-0,399916837	0,170326059	-2,347948628	0,018877125	0,152857832
Osbpl2	722,7761976	-0,173721995	0,087754804	-1,979629461	0,04774518	0,261149793
Madd	542,9280767	-0,198554197	0,135791435	-1,462199708	0,143686492	0,4705891
Bcas3os1	0,113662148	0,780932884	3,352475198	0,232942181	0,815806304	NA
Sh3bgrl3	219,4010518	0,321634674	0,184760149	1,740822771	0,081714649	0,353542587
Apobec1	568,0652494	-0,292222275	0,158808509	-1,840092049	0,065754724	0,313731232
Ssfa2	1030,936503	-0,099099601	0,135376491	-0,73202962	0,464150489	0,781663619
Mrpl41	561,7577434	0,028456914	0,091834769	0,309870805	0,756659205	0,923140802
Emp1	35,07761315	0,423396642	0,506646974	0,835683749	0,40333284	0,743286759
Arid3a	32,77255323	-0,52379529	0,399660776	-1,310599693	0,189993043	0,538025204
Dennd2d	283,2534313	0,30013965	0,166536385	1,802246698	0,071506601	0,327836675
Epc1	344,5996311	0,130371894	0,100718597	1,294417295	0,19552132	0,544454182
Mir106b	0,443168919	2,196464572	3,313880474	0,662807421	0,5074539	NA
Gm10639	1,39368604	1,654600726	1,530834993	1,080848513	0,279764505	NA
Mup10	36,3012053	-1,188196307	0,593669048	-2,001445604	0,04534439	0,252166869
Tppp2	0,320627671	0,66756312	3,24176613	0,205925749	0,836848911	NA
Dedd	665,986916	0,05894783	0,10942506	0,53870503	0,590090402	0,84953676
Epb41	2490,148566	-0,10232998	0,098201614	-1,042039695	0,297393275	0,658144463
Arpp19	1482,425697	0,129918601	0,08594122	1,511714654	0,130606469	0,449440897
Lncppara	8,115046714	0,973469236	0,592250284	1,643678801	0,100242567	0,393234413
Ghitm	6530,416568	-0,066079284	0,075262647	-0,877982465	0,379953235	0,725449129
Wfdc6a	0,173498105	0,059593471	3,352475198	0,017775962	0,985817581	NA
Tirap	625,7019116	-0,140208852	0,162145088	-0,8647123	0,387196717	0,731547895
Frmd4a	131,8528188	0,188126242	0,227242772	0,82786458	0,407747196	0,746679655
Dcun1d1	770,8580663	0,140580105	0,132961287	1,057301027	0,290374225	0,652000842
Folr2	286,5436248	-0,01564523	0,253408286	-0,061739219	0,950770508	0,985972877
Gstp3	534,6807626	0,345605402	0,182591203	1,892782329	0,058386822	0,291803151
Gadd45b	39,81264282	1,090028883	0,393065117	2,773150901	0,005551637	0,073122991
Fnta	702,8730808	0,06612125	0,092650352	0,713664319	0,475434775	0,789379968
Cxcl1	126,8218568	-0,058730786	0,623348193	-0,094218267	0,924935774	0,978203178

Edar	0,086476712	0,780932884	3,352475198	0,232942181	0,815806304	NA
Dusp2	7,49206467	0,800121975	0,606722271	1,318761505	0,187248857	0,533703441
Polr2c	508,7084075	0,0164856	0,113749394	0,14492912	0,884766835	0,967324813
Grb10	98,84003319	-0,655089693	0,197261687	-3,320917018	0,000897222	0,021888617
Eif2ak3	473,6167123	-0,695614385	0,130103435	-5,346625802	8,96E-08	2,10E-05
Polr1a	435,2702169	-0,049529447	0,138356704	-0,357983717	0,720355499	0,908788588
Dhrs7	866,521867	0,504483351	0,17678733	2,853617125	0,004322459	0,061870402
Mir142b	0,093953095	-0,517475177	3,352475198	-0,154356154	0,877328924	NA
Dopey1	316,1128145	0,032013406	0,165818366	0,193063091	0,846909558	0,953824301
Chd9	564,9408107	-0,418594802	0,162117928	-2,582038927	0,009821851	0,103392378
2210416O15Rik	0,207615244	0,059593471	3,352475198	0,017775962	0,985817581	NA
Ddost	3097,471801	-0,332498359	0,095628289	-3,476987432	0,000507082	0,014801689
Sdf2	749,0679646	-0,130402282	0,107466117	-1,213426951	0,224966627	0,580187941
Entpd1	97,83913404	0,11780386	0,274986457	0,428398769	0,668360821	0,886438999
Fgd2	80,20373753	0,295070056	0,199742141	1,477254899	0,139607323	0,464512269
Acsm5	3151,531045	-0,00262577	0,133988751	-0,019596941	0,984364904	0,995554769
Adck2	225,2066086	-0,041761044	0,124971266	-0,334165165	0,738254929	0,915389551
Pced1a	514,4357104	-0,103667898	0,092176722	-1,124664619	0,26073118	0,621964694
Zfp947	24,17478224	0,471611675	0,347837448	1,355839282	0,17515031	0,517378056
Gdpgp1	112,97033	-0,195624381	0,14713328	-1,329572618	0,183659125	0,529829877
Orai2	19,86686182	0,376943453	0,383393381	0,983176735	0,325520463	0,682862556
Hlf	872,2705318	-0,12275588	0,177618968	-0,691119206	0,489490632	0,796616325
Tada2a	148,7183165	-0,001729003	0,148498254	-0,011643257	0,990710235	0,99719344
Coq3	487,8963108	0,353712313	0,18580084	1,90371751	0,056946985	0,288295377
Gcn1l1	923,967532	-0,381119592	0,114304285	-3,334254646	0,000855283	0,021174594
Rhbdf2	269,3895829	0,179881345	0,154802788	1,16200327	0,245234137	0,604561161
Mppe1	200,9811591	-0,016960339	0,154839001	-0,109535316	0,912777912	0,97607806
Scamp1	1074,904528	0,192383238	0,083655284	2,299714123	0,021464421	0,163367723
Ep400	808,9545931	-0,224974288	0,096415789	-2,333376	0,01962842	0,156064296
Dhrs4	3191,522782	-0,273340718	0,085056124	-3,213651227	0,001310588	0,028629487
Dhx58	372,9197678	0,205220867	0,191250558	1,073047152	0,283249961	0,644666109
Tmx4	429,3908045	-0,264404044	0,154873994	-1,707220408	0,087781093	0,367306865
Rabep2	270,896681	-0,274662126	0,145525638	-1,887379636	0,059109282	0,293856809
Lsm6	812,7972041	-0,173154676	0,169216763	-1,023271412	0,306179537	0,665363171
4930503B20Rik	0,113662148	0,780932884	3,352475198	0,232942181	0,815806304	NA
Troap	4,734663184	-1,844663962	0,868303654	-2,124445698	0,033632892	NA
Clvs1	1,198044533	-0,19231418	1,427165358	-0,134752556	0,892807518	NA
Mylk	1622,607059	-0,115957506	0,152174616	-0,762002949	0,446058244	0,771555527
Galnt2	883,1313252	-0,242150652	0,116917397	-2,07112592	0,03834703	0,22978998
Pth2r	0,527012662	0,436660893	2,136661737	0,204365944	0,838067541	NA
Nol6	598,8890479	-0,212881543	0,094501656	-2,252675258	0,024279632	0,176987133
Fgd4	416,5595335	-0,194467867	0,148943319	-1,305650151	0,191671557	0,541059343
Cog8	518,8154595	-0,001695073	0,115806074	-0,014637172	0,988321643	0,996575327
Rhbdl3	3,190051886	0,529304968	0,986458896	0,536570728	0,591564168	NA
Atxn7	456,8587728	-0,252923934	0,105310787	-2,401690674	0,016319502	0,141031297
Mavs	2638,478601	-0,261351436	0,100494823	-2,600645774	0,009304848	0,100238928
Mgat5	51,65006474	0,124029322	0,334993164	0,370244338	0,711200443	0,904924215
Brwd1	483,7723106	0,012429353	0,115218826	0,107876059	0,914094009	0,976392941
Adam9	2028,330432	-0,203350051	0,112771832	-1,803198963	0,071356973	0,327487599
E2f3	159,4258838	-0,293748549	0,129347656	-2,271000177	0,023146967	0,17191767
Rora	1405,765162	-0,509330953	0,124047085	-4,105948584	4,03E-05	0,002280995
Ddx19a	483,8187449	-0,076160236	0,09569244	-0,795885612	0,426098525	0,758368511
Thpo	617,8623956	0,041242757	0,130674302	0,315614903	0,752294824	0,921094377
Zfp189	142,3510007	0,223404612	0,147622254	1,51335321	0,130189964	0,448469369
Per3	96,20893895	-0,755015349	0,253595583	-2,977241711	0,002908546	0,048005373
Cdk5	515,5911104	-0,141470968	0,099653866	-1,419623482	0,155717326	0,490700453
Cntrl	431,8037822	0,283024961	0,138922784	2,037282524	0,041621738	0,240883557
Cox6b2	5,63496503	-0,579900367	0,632519631	-0,916810071	0,359242175	0,708603477
Rnf13	3539,400873	0,177028422	0,12745378	1,388961722	0,16484439	0,50334664
Chgb	0,269274339	0,059593471	3,352475198	0,017775962	0,985817581	NA
Chad	0,12663974	0,780932884	3,352475198	0,232942181	0,815806304	NA
Cenpa	72,32070941	-0,002182917	0,23528959	-0,009277574	0,992597673	0,997972347
Cd63	113,8006999	0,257739953	0,370662291	0,695349808	0,486836107	0,795175555
Ift52	627,239363	0,10954227	0,092714399	1,181502245	0,237403262	0,594575536
B9d2	95,63133214	-0,190503032	0,161933182	-1,176429868	0,239423142	0,596781687
Mars	1794,678414	-0,048501488	0,11400436	-0,425435384	0,670519317	0,887361539

Zfp354c	14,56235662	-0,658036973	0,382534664	-1,720202206	0,085395692	0,362200193
Map3k6	16,50676247	-0,583480971	0,383989093	-1,519524855	0,128630437	0,446085683
Usp21	432,2731076	-0,046359418	0,087749168	-0,528317465	0,597279012	0,853549036
Usp25	752,8382021	-0,026314408	0,120191993	-0,218936451	0,826699551	0,947537943
Snai3	2,399243613	0,656871408	1,093024568	0,600966737	0,547862141	NA
Eif3g	1603,359695	-0,196123569	0,103899501	-1,887627628	0,059075958	0,293856809
Fbxl12	119,1813213	0,413384601	0,195662437	2,112743804	0,034622702	0,217104886
Kdm2b	149,745289	-0,104478311	0,165939883	-0,629615433	0,528946224	0,818868823
B130006D01Rik	0,554066772	0,67418655	2,100753167	0,320926114	0,748266384	NA
Acan	0,093953095	-0,517475177	3,352475198	-0,154356154	0,877328924	NA
Dnajb7	0,350249842	-0,720602587	2,645726487	-0,272364733	0,785341587	NA
Ell	684,7330553	0,061488652	0,0860182	0,714833042	0,474712215	0,788670014
Gdi2	6169,35946	0,076367436	0,071629695	1,06614214	0,286359393	0,648131311
Fdxr	484,0173152	0,068332101	0,171972357	0,397343517	0,691114161	0,894867333
Natd1	164,2766813	-0,250504411	0,13379257	-1,87233425	0,061160375	0,299726193
Stau2	106,6112589	0,448610461	0,166138074	2,700226679	0,006929225	0,084138787
Cfap74	5,09767622	0,303292064	0,69505576	0,436356449	0,662578132	NA
Slc25a13	3827,02022	-0,946747008	0,189428693	-4,997907099	5,80E-07	8,51E-05
Phldb3	263,516711	-0,342618248	0,113318573	-3,023495956	0,002498724	0,043553373
Hnrnph3	377,9341901	0,011036938	0,122181439	0,090332362	0,928023104	0,979144572
Scnn1a	885,0020165	0,118585276	0,147594879	0,803451157	0,421714017	0,755243764
Vcam1	220,1844709	0,319746348	0,346441226	0,922945435	0,356035642	0,706103025
Hist1h1a	0,486849634	0,329886297	2,157167305	0,152925689	0,878456877	NA
Xpr1	587,1686265	0,069335171	0,13960775	0,496642708	0,619441031	0,861846105
Tcf3	388,7920841	0,020283575	0,103239633	0,196470816	0,844241673	0,953068489
Map2k5	526,5985672	0,032033843	0,109281619	0,293131113	0,769421936	0,927873161
Tcf21	108,3041533	0,187299351	0,253342914	0,739311586	0,459717815	0,78083693
Tcf12	434,0631701	0,103422649	0,144911939	0,713693086	0,475416983	0,789379968
Nfib	1619,140141	0,115718914	0,088389148	1,309198214	0,190467217	0,538804246
Susd2	63,99607685	0,037681309	0,263177688	0,143178207	0,886149441	0,967728466
Thbd	51,51428849	-0,116898396	0,287267042	-0,40693285	0,684057308	0,892297995
Rps15	6256,196172	0,033647905	0,128630247	0,26158626	0,793640438	0,936746676
Araf	1528,978344	0,013035573	0,09796268	0,133066727	0,894140609	0,970273198
Creb1	363,444248	-0,227898515	0,143955542	-1,583117341	0,113394722	0,417049253
Lck	20,481713	0,342019101	0,376163727	0,90922935	0,363229075	0,712338199
Bcl2l1	799,6915709	-0,200588399	0,110040043	-1,822867324	0,068323488	0,319740014
Aqr	675,2399785	-0,143126555	0,116662262	-1,226845365	0,219880717	0,574490867
Mir8103	0,093303375	-0,517475177	3,352475198	-0,154356154	0,877328924	NA
Ankrd35	1,432316288	-0,544868778	1,179722767	-0,461861713	0,644180492	NA
Tbc1d24	389,8564713	-0,174175424	0,141269555	-1,232929661	0,217602008	0,57153659
Nfasc	13,79096601	0,334671263	0,426371056	0,784929601	0,432494846	0,762604031
Cfap57	0,234744367	0,059593471	3,352475198	0,017775962	0,985817581	NA
Nars	3369,204307	-0,166127014	0,095624925	-1,737277332	0,082338231	0,354518456
Atraid	1751,37517	0,138211723	0,082474875	1,675803983	0,093776594	0,380194927
Zfp943	298,5869922	-0,002717314	0,127514114	-0,021309909	0,982998439	0,995413661
Adcyap1r1	40,16391451	0,090433918	0,474028982	0,190777192	0,848700159	0,954095425
Apobec3	48,52029278	0,543858944	0,283066008	1,921314917	0,054692018	0,283347063
Mir8102	0,307069547	0,617382779	2,733615373	0,225848444	0,821319297	NA
Ptpn2	333,020389	0,010687627	0,11656795	0,091685807	0,926947674	0,978888597
Ttyh1	26,9685174	0,057603157	0,58591627	0,09831295	0,921683795	0,977341679
Fam89a	251,9164673	-0,021197339	0,228444567	-0,092789859	0,926070507	0,978534437
B230217C12Rik	6,050422229	0,178657417	0,626519632	0,285158529	0,775522691	0,92961202
Rbfox3	17,08551677	0,312095702	0,384867002	0,810918318	0,41741258	0,752195715
Adgrf2	0,603604061	-0,70950489	2,058169001	-0,344726254	0,730300179	NA
Dffa	370,4586876	-0,003283302	0,101316159	-0,032406503	0,974147877	0,993072906
Ccne1	13,71395338	-1,20732462	0,564967408	-2,136981006	0,032599537	0,210237918
Hspg2	486,8532721	-0,278316715	0,181960618	-1,529543694	0,126129716	0,441736801
Adgrb2	1,094532794	0,636458303	1,807728795	0,35207621	0,724781109	NA
Chrm4	0,180974489	-1,204797453	3,342691436	-0,360427361	0,718527568	NA
Cit	8,780487664	-0,463353924	0,539388229	-0,859036031	0,390320637	0,734026811
Chrm1	7,187681029	1,582613035	0,974345662	1,624282938	0,104315437	0,401673966
Socs3	230,8992275	-1,168054434	0,489327372	-2,3870613	0,016983659	0,144212847
Chl1	0,240301888	1,389394708	3,349408814	0,414817893	0,67827522	NA
Socs2	497,5179976	-0,182115654	0,751465821	-0,242347221	0,808511127	0,942326905
Proser3	81,86223494	-0,416862405	0,172331293	-2,418959422	0,015564976	0,136720216
Rptor	817,4311499	-0,109746152	0,120481153	-0,910898917	0,362348637	0,711550958

Cacnb2	7,676673652	-0,159700146	0,695044002	-0,229769835	0,818270626	0,945665629
Pigyl	438,5692588	-0,028367113	0,132707439	-0,213756763	0,830736744	0,9488355
Lrrc32	301,4488696	-0,494399682	0,17382269	-2,84427586	0,004451249	0,063307181
Asap2	491,8942551	0,514526154	0,173820838	2,960094782	0,003075444	0,050079917
Epha7	43,25088246	0,171703966	0,290297173	0,591476534	0,554201173	0,832018326
Epha2	444,0583685	0,424681273	0,21974322	1,932625146	0,053282392	0,278635557
Cxcl2	3,24610123	0,505014835	0,830923527	0,607775348	0,543336482	NA
Epha3	9,946596872	0,147100715	0,56619051	0,259807808	0,795012028	0,937443926
Ccl25	74,9957629	-0,385266072	0,192197635	-2,004530766	0,045013235	0,251475944
Ccl22	3,111354707	0,546656997	1,105209217	0,494618565	0,620869395	NA
Epas1	3312,488271	-0,168071253	0,09293579	-1,808466396	0,070533942	0,324865285
Gng4	0,20013886	1,337107854	3,350237755	0,399108348	0,689813372	NA
Timp3	397,7573686	-0,302193187	0,219723637	-1,375333081	0,169028205	0,508491916
Top2a	41,05051846	-1,597592168	0,480283383	-3,326353203	0,000879904	0,021620484
Tlr6	11,25092036	-0,233937514	0,453132103	-0,516267799	0,605667383	0,856421285
Tbpl1	192,7360843	-0,004442576	0,119690353	-0,037117245	0,970391522	0,992007633
Eif4g2	10898,07689	-0,040399422	0,106642409	-0,378830735	0,704813562	0,90177553
Spag1	26,58950948	-0,306145267	0,357150954	-0,857187315	0,391341372	0,734518115
Dsc2	1322,644877	0,191300625	0,124720425	1,533835585	0,125070106	0,440132206
Uba2	651,1229193	-0,100343131	0,107354161	-0,934692515	0,349946785	0,701835969
Timm44	1142,887763	0,01266442	0,079353028	0,15959592	0,873199393	0,963097638
Tmem182	0,113662148	0,780932884	3,352475198	0,232942181	0,815806304	NA
Kcns3	1,097771563	-0,026052586	1,516752748	-0,017176554	0,986295766	NA
Sv2b	0,113662148	0,780932884	3,352475198	0,232942181	0,815806304	NA
Ino80e	187,8008101	-0,212990733	0,132296962	-1,609944253	0,107410027	0,406788853
Nipa1	22,33213115	0,309599999	0,361150234	0,857260967	0,391300675	0,734518115
Syne4	80,48982319	0,33818257	0,395852326	0,854314975	0,392930492	0,735241631
Fam13a	565,9866869	0,33937239	0,443383641	0,765414775	0,444024598	0,770157058
Katnal1	138,5593781	-0,55818281	0,155255023	-3,595264101	0,000324063	0,010644702
Usp1	403,5522642	0,088008355	0,144069513	0,610874247	0,541282835	0,826407188
Nadk	4782,351557	-0,110262814	0,10133196	-1,088134623	0,276535681	0,637524663
Gak	1793,587727	-0,249262287	0,085449886	-2,917058167	0,003533499	0,055321516
2700038G22Rik	5,990227978	0,283701544	0,633684183	0,447701791	0,654368429	0,878857794
Zfp207	1720,421116	0,263067125	0,086376696	3,045579862	0,002322321	0,042012326
Khdrbs1	656,1686301	0,051344349	0,087433505	0,587238825	0,557043321	0,833822555
Gm15987	0,963063207	0,741434471	1,919721093	0,386219891	0,699333824	NA
Gm20257	31,66109101	0,087778648	0,284534865	0,308498743	0,757702856	0,923804952
A730043L09Rik	0,338296039	0,667319034	3,192071415	0,20905517	0,83440517	NA
4930480G23Rik	112,8522422	0,673455311	0,195823872	3,439086892	0,00058368	0,016393311
9430037G07Rik	25,849556	-0,086257811	0,336351893	-0,256451094	0,797602539	0,938325224
Kcnd3os	0,086476712	0,780932884	3,352475198	0,232942181	0,815806304	NA
0610040F04Rik	5,657577226	-0,366047719	0,654076342	-0,559640665	0,575724562	0,843861602
Gm15133	2,664012429	0,713284903	1,005468854	0,709405269	0,478073019	NA
Scarletltr	0,797048106	0,480525553	1,755600664	0,273710055	0,78430745	NA
4930429F24Rik	2,728381218	-1,338226727	1,041120411	-1,285371714	0,198662421	NA
1700110K17Rik	0,451958187	1,213504365	2,501740245	0,485064094	0,627630881	NA
Krt4	3,436111784	-0,157569254	0,767065014	-0,205418382	0,83724526	NA
Snhg5	100,2280844	-0,113844138	0,209568379	-0,543231469	0,586970442	0,848346794
4933433G15Rik	1,170507457	1,853647078	1,512924229	1,225208139	0,220496809	NA
C130083M11Rik	0,093303375	-0,517475177	3,352475198	-0,154356154	0,877328924	NA
4933422A05Rik	0,447126249	-1,517310793	2,416716129	-0,627839892	0,53010883	NA
Plet1os	24,44057071	-0,39007389	0,317784035	-1,227481081	0,219641828	0,574490867
1110036E04Rik	0,122496332	0,780932884	3,352475198	0,232942181	0,815806304	NA
AW046200	2,170712566	-0,288218426	1,046479928	-0,275417061	0,782995838	NA
1110046J04Rik	4,293672728	-0,519923933	0,673204547	-0,772311975	0,439929675	NA
B930018H19Rik	0,113662148	0,780932884	3,352475198	0,232942181	0,815806304	NA
B930025P03Rik	23,63641651	0,982260319	0,433537771	2,265685676	0,023470635	0,173262795
6030466F02Rik	1,063764216	-1,283046495	1,494329405	-0,858610217	0,390555599	NA
2310034O05Rik	5,547372128	3,177902856	1,063743161	2,98747195	0,002812951	0,047303576
LOC100503496	19,13116372	0,749110836	0,339556281	2,206146309	0,027373757	0,190807622
BC039966	0,087021394	-0,517475177	3,352475198	-0,154356154	0,877328924	NA
A630077J23Rik	0,340300874	0,667300037	3,188296412	0,209296737	0,834216598	NA
Mir7048	0,142634598	-0,517475177	3,352475198	-0,154356154	0,877328924	NA
Pcdh19	0,142634598	-0,517475177	3,352475198	-0,154356154	0,877328924	NA
Arhgap44	25,67016388	-0,057235711	0,280112962	-0,20433082	0,838094987	0,951141594
Bicd1	2,276948916	0,738451031	1,149155411	0,642603275	0,520481557	NA

Itsn1	851,2311228	-0,107355146	0,129870638	-0,826631395	0,408446017	0,746898413
Get4	461,692117	0,046194724	0,095852339	0,481936324	0,629851181	0,866228449
Sapcd2	1,593659455	-0,699557498	1,238316593	-0,564926209	0,572123956	NA
Mybl1	8,656934259	-0,536376284	0,514933431	-1,041641992	0,297577692	0,658152471
Adgre5	416,0353815	0,024496633	0,231191929	0,105957994	0,91561568	0,976559041
Nek3	44,96467399	-0,491001956	0,278040308	-1,765938042	0,077406244	0,344603422
Pcx	12314,24404	0,18276929	0,112529226	1,624193968	0,104334418	0,401673966
Col6a3	166,482457	-0,150188083	0,23778921	-0,631601757	0,527647138	0,818474757
Dpp6	0,172953423	1,307385949	3,350719078	0,39018071	0,696402925	NA
Mcm3	121,9349949	-0,375976178	0,299928824	-1,253551338	0,210005128	0,562470273
Cacul1	1279,554285	-0,093817358	0,144074752	-0,651171399	0,514935851	0,812100671
Zbtb25	41,79153659	0,046443548	0,309249122	0,150181665	0,880621291	0,96546599
Depdc1a	1,319152967	0,03412589	1,289170365	0,026471203	0,978881502	NA
Jagn1	572,4671079	-0,239479571	0,127992735	-1,871040353	0,061339488	0,300117962
3110009E18Rik	16,27828441	0,030208914	0,38875393	0,077707032	0,9380611	0,982832198
Ttc4	580,3295053	0,087582862	0,120033001	0,729656526	0,465600161	0,781663619
Senp8	68,50690974	-0,074921653	0,204348307	-0,366637012	0,713889797	0,90610393
Gas1	438,3117178	0,219436574	0,260722331	0,841648557	0,399984701	0,740788928
Ankrd10	144,5357642	0,045974493	0,235091999	0,195559582	0,844954898	0,953185557
1700001P01Rik	0,180974489	-1,204797453	3,342691436	-0,360427361	0,718527568	NA
Pdia5	1899,471368	-0,345440001	0,121632551	-2,840029237	0,00451094	0,063952021
Mms19	595,6607273	0,133368662	0,105723568	1,261484686	0,207134274	0,559656044
Apol8	0,12663974	0,780932884	3,352475198	0,232942181	0,815806304	NA
Tysnd1	1328,919407	-0,248418419	0,126566829	-1,962745069	0,049675803	0,267192299
Adpgk	412,9329618	-0,138236602	0,119647691	-1,155363723	0,247941511	0,607129663
Slc39a5	18,36672941	1,199944415	0,696583363	1,722614233	0,084958309	0,361147103
Fam214a	1386,136362	-0,250668235	0,248227613	-1,009832194	0,312575693	0,672052821
Cmtm4	911,3387049	-0,388223767	0,098576418	-3,938302632	8,21E-05	0,003918075
Tcf4	247,734362	0,000836817	0,170225267	0,004915936	0,996077666	0,998991922
Adgrl2	1028,401866	0,045631059	0,10939864	0,417108102	0,676599339	0,889512888
Nt5c3b	64,28370376	0,200841668	0,211123142	0,951301056	0,341451571	0,695250579
Gba	676,6448044	-0,092203369	0,079732568	-1,156407876	0,247514362	0,607048156
Mark2	443,668133	-0,058528283	0,103322139	-0,56646411	0,571078324	0,8413217
Tcf19	67,84892518	-0,443344893	0,230699573	-1,921741286	0,054638319	0,283233692
6430548M08Rik	215,5051748	-1,359184784	0,149457882	-9,094099073	9,54E-20	3,19E-16
Dhx57	318,9902412	-0,190669587	0,13843805	-1,377291768	0,168422059	0,507353427
Slc10a1	17026,71529	0,491165784	0,184962531	2,655488012	0,007919375	0,091192181
Rbmx	424,6517016	0,265100639	0,160414149	1,652601344	0,098412024	0,390695308
Psmd10	275,2935422	0,004701942	0,124966942	0,037625485	0,969986289	0,991981655
Syde2	91,02702336	-0,602871884	0,273039882	-2,207999356	0,027244318	0,190567596
Ccdc152	2,11141275	-0,347856258	1,08736818	-0,319906601	0,749039134	NA
Rusc2	438,3019923	-0,177973381	0,158618185	-1,122023812	0,261852316	0,622562722
Shh	4,11481557	-1,176957917	0,823239541	-1,429666408	0,152812786	NA
F2r	638,2663007	-0,648557454	0,295249313	-2,196643399	0,028045924	0,192787383
Shd	1,003453287	-0,669000227	1,489904038	-0,44902236	0,653415527	NA
Jchain	27,072099	0,826727716	0,327870387	2,521507731	0,011685311	0,115462616
Vnn1	518,2661985	0,198640874	0,551972699	0,359874454	0,718941022	0,908375628
Cirh1a	438,0418884	-0,062171671	0,148849117	-0,417682499	0,67617927	0,889369368
Tesk1	537,0774206	-0,279016791	0,126042826	-2,213666576	0,026851724	0,189333814
Gpc6	54,98131608	0,033628769	0,319840057	0,105142456	0,916262771	0,976559041
Mta2	1387,388367	0,047922872	0,065985006	0,726269112	0,467673811	0,782859415
Parg	347,0147744	-0,22122276	0,148033896	-1,494406123	0,135069502	0,457497891
Vrk3	641,4391963	0,038986986	0,078901538	0,494122	0,621220022	0,862654966
Wdr44	85,92550599	-0,252851624	0,207011458	-1,221437818	0,221920296	0,576647704
Acot7	514,6484841	-0,001618826	0,091248895	-0,017740776	0,985845652	0,995929003
Dhx30	582,2443848	-0,121602719	0,117543546	-1,034533356	0,300886891	0,661404508
Mir6955	1,437069855	-0,064842526	1,395927625	-0,046451209	0,962950622	NA
Pradc1	157,4047432	0,154105715	0,143928518	1,070710077	0,28429981	0,645517011
Tatdn3	113,6325273	0,176440566	0,16964585	1,040052359	0,298315576	0,658985613
Pls3	2060,266157	0,149789494	0,132080003	1,134081546	0,256760344	0,617379323
Dek	841,629525	-0,011915411	0,121135623	-0,098364222	0,921643083	0,977341679
1110008F13Rik	2002,492935	0,044841727	0,084770052	0,528980766	0,596818792	0,853263869
Psmd9	892,626742	0,130959312	0,085545928	1,530865526	0,125802631	0,441505609
Lgals2	2,625942425	3,296755288	1,374676781	2,398203952	0,016475689	NA
Kctd20	531,7759145	-0,005453538	0,109931134	-0,049608679	0,96043423	0,98838005
Rab5a	865,5679544	-0,077416308	0,118431275	-0,653681291	0,513317159	0,811058031

Podxl2	3,614237033	-0,22906989	0,814687658	-0,281175108	0,778576096	NA
Relt	8,300683213	0,700109776	0,537863126	1,301650441	0,193035908	0,541594346
Hist1h4d	2,974311454	-1,47683469	0,872343697	-1,692950491	0,090464891	NA
Xylt1	0,834943398	2,160156783	1,707095419	1,265398969	0,205728345	NA
Gapt	2,357780847	-0,110411959	1,091820566	-0,101126469	0,919450066	NA
Spink10	0,396879234	-0,105762241	2,592188938	-0,04080036	0,967455052	NA
Rtn4rl1	411,7897123	-0,304885592	0,189567176	-1,608324812	0,107764059	0,407667716
Stac3	9,846506697	0,410150622	0,487934764	0,840584955	0,40058049	0,741431617
Rft1	364,8935873	0,13111028	0,128251233	1,022292547	0,30664246	0,666006686
Isg20l2	390,7487596	0,12258269	0,106882283	1,146894377	0,251425277	0,610832604
Cdh16	0,417942278	2,113820908	3,008057355	0,702719616	0,482230501	NA
Zbtb10	67,04348688	0,070862059	0,188366607	0,376192255	0,706773972	0,902297674
D630003M21Rik	11,88036209	0,051852978	0,49816501	0,104087957	0,917099548	0,976907366
6820408C15Rik	0,547446175	0,471183429	2,126006176	0,221628438	0,824603142	NA
F830045P16Rik	0,093953095	-0,517475177	3,352475198	-0,154356154	0,877328924	NA
Lmo1	0,66099746	0,198205355	1,99814117	0,099194871	0,920983547	NA
Gab1	277,0506819	-0,232821849	0,145272705	-1,602653778	0,109011114	0,40910239
Ctf1	94,4091798	0,157433155	0,173834492	0,905649693	0,365121297	0,713569421
Fam213a	2158,05168	-0,000748665	0,112081636	-0,006679641	0,994670457	0,99855443
Nek1	305,0374906	-0,130048977	0,164159149	-0,792212789	0,428236613	0,760002028
Cdnf	14,00314819	0,563543604	0,434894618	1,295816461	0,195038726	0,544130368
1700052K11Rik	65,87755804	0,015747097	0,271749774	0,057947048	0,953790807	0,986479135
Zbtb16	167,933321	0,256151228	0,300203736	0,853257961	0,393516267	0,735998738
Colgalt1	1710,76441	0,029915086	0,071218844	0,420044536	0,674452919	0,888627551
Liph	1,585362112	1,03029591	1,210976071	0,850797909	0,394881623	NA
Fam149a	833,7957959	-0,211861208	0,09463993	-2,238602764	0,025181774	0,181772069
P2rx2	1,294595516	1,497176623	1,346733452	1,111709686	0,266262994	NA
Lrfn4	5,380571275	0,157490659	0,691666583	0,227697365	0,819881521	0,946300272
Smc3	625,6411325	-0,073377826	0,109194519	-0,671991842	0,50158889	0,803893049
Itgb2	181,5715319	0,276033749	0,218467346	1,263501181	0,206409122	0,558880735
Csk	1538,272165	-0,09598584	0,078583269	-1,221453895	0,221914212	0,576647704
Csf3r	68,57036216	0,894067703	0,33129347	2,69871816	0,00696071	0,084198069
Csf2rb2	34,24829445	0,483228783	0,318191904	1,518670892	0,128845357	0,446309134
Csf2rb	183,4960749	-0,445843001	0,160974169	-2,76965555	0,00561156	0,073470343
Csf1	256,9337565	0,223623368	0,152707325	1,464391889	0,143086903	0,470269006
Gstm2	2714,065957	1,115125145	0,166037882	6,716088715	1,87E-11	1,31E-08
Cry1	688,2855664	-1,103091287	0,228415063	-4,829328129	1,37E-06	0,000171166
Crp	10020,46301	0,16120294	0,083402799	1,932824101	0,05325787	0,278635557
Fut1	0,925944007	-0,265384389	1,543822113	-0,171900886	0,86351545	NA
Lcn5	0,340300874	0,667300037	3,188296412	0,209296737	0,834216598	NA
Fosb	5,238330634	-0,921772998	0,99262209	-0,928624305	0,353083822	NA
Crcp	573,6159206	0,216279312	0,108455129	1,994182421	0,046132123	0,255211169
Snord104	91,91061021	0,195826455	0,197595561	0,991046831	0,321662713	0,678817492
Dlc1	1307,041667	-0,262281428	0,118208685	-2,218799981	0,026500335	0,187749329
Plk3	1159,613851	-1,533182784	0,409663636	-3,742540588	0,000182169	0,007075561
Gpi1	5759,930824	0,045158226	0,110514796	0,408617015	0,682820742	0,891903322
Cmklr1	69,88795741	0,210591376	0,297774696	0,707217163	0,479431534	0,791837124
Gpr65	44,27394922	0,303099063	0,340915595	0,889073622	0,373963514	0,720823634
Gpam	5336,093711	0,522125361	0,27288277	1,913368735	0,055700864	0,285312203
Hcls1	153,907812	-0,001001211	0,210225563	-0,004762556	0,996200044	0,999039711
Secisbp2	412,2854902	-0,218161766	0,113993717	-1,913805184	0,055645053	0,285308756
Nup210l	2,228569382	-0,09482582	0,956770909	-0,099110266	0,921050721	NA
Ilf3	424,3814986	-0,080385181	0,109409562	-0,73471806	0,462511219	0,781516914
Dnmt1	290,2482266	-0,229507488	0,098695161	-2,325417832	0,020049629	0,158137751
Zfp821	53,79143584	-0,21558589	0,22014872	-0,979273875	0,327444675	0,685071653
Ghrl	0,180429807	0,059593471	3,352475198	0,017775962	0,985817581	NA
Abcb1a	97,060769	1,2929766	0,336759184	3,839469455	0,0001233	0,005266466
Trnt1	330,4823829	0,193723683	0,140388456	1,379911776	0,16761381	0,506611452
Slc29a1	5758,438103	0,207881913	0,13197859	1,57511845	0,115229106	0,420212198
Serpinb12	0,086476712	0,780932884	3,352475198	0,232942181	0,815806304	NA
Grin2a	0,093303375	-0,517475177	3,352475198	-0,154356154	0,877328924	NA
Slc22a6	0,270219305	1,389394708	3,349408814	0,414817893	0,67827522	NA
Polr3g	336,5386958	-0,045797883	0,145037936	-0,315764854	0,752180997	0,921039361
Btaf1	618,0822891	0,173421813	0,126040348	1,375922991	0,168845476	0,508170908
Zfp467	608,6024934	-0,110992163	0,117456824	-0,944961384	0,344678591	0,697587612
Homer2	692,7634849	-0,300300518	0,129980553	-2,3103496	0,020868807	0,161328775

Rpl36	3187,153559	0,106964995	0,144945606	0,737966457	0,460534833	0,780953195
Atp5l	960,0703462	0,173580078	0,154301781	1,12493892	0,260614918	0,621964694
LOC105246056	5,790227452	-0,800822882	0,590014823	-1,357292818	0,174688188	0,517225036
Aox3	17234,07399	-1,217182498	0,356116315	-3,417935225	0,000630981	0,017215486
Nat8	466,5622345	-0,974683449	0,800153435	-1,218120684	0,223178115	0,578230275
Arid5b	736,2615718	-0,832288453	0,452960755	-1,837440538	0,066144887	0,314918445
Sct	0,361299258	-2,093941636	2,509883782	-0,834278324	0,404124163	NA
Wdr31	2,877846565	-0,24182699	0,890737523	-0,271490741	0,78601362	NA
Slc29a3	123,6744589	-0,143296634	0,199579389	-0,717993152	0,472761509	0,787091982
Sep-14	0,180429807	0,059593471	3,352475198	0,017775962	0,985817581	NA
Xpo6	890,7310274	0,1123148	0,097084153	1,156880873	0,247321034	0,606796643
Tbc1d30	215,3014231	0,482523074	0,24075232	2,004230218	0,045045405	0,251505172
Ttc21a	0,939571046	-0,734140466	1,641270988	-0,447299971	0,654658488	NA
Pacsin3	1324,776382	-0,043424195	0,085421027	-0,508354871	0,611204495	0,858645358
Esyt2	612,249819	0,070963066	0,107237457	0,661737682	0,50813935	0,807274415
Pex26	464,2418674	0,049523902	0,123603804	0,40066649	0,688665685	0,894208581
Nfam1	113,9305394	0,163090522	0,216425061	0,75356579	0,451109993	0,774282898
Armc9	121,0933301	0,03122221	0,16818045	0,185647084	0,852721527	0,95621826
Pop5	727,9468608	0,057511526	0,106331179	0,540871707	0,588596014	0,848952434
Nphp3	220,5690232	-0,25310749	0,13295078	-1,903768373	0,056940357	0,288295377
Mlf1	3,12651083	0,68295318	0,95985607	0,711516238	0,476764392	NA
Fkbp3	590,0909959	-0,094459816	0,097559912	-0,968223677	0,332932672	0,68995097
Slc39a1	1328,488393	-0,052804392	0,073795395	-0,715551314	0,474268445	0,788130102
Mfi2	0,929160519	-0,345941533	1,639696286	-0,210979031	0,832903634	NA
Fbxw2	879,3826208	-0,022519502	0,070322405	-0,320232242	0,748792283	0,919838327
Dnajc12	181,8283582	0,017426276	0,192291975	0,090624043	0,927791327	0,978977289
Clic4	1890,760907	-0,13787399	0,118804451	-1,160511998	0,245840412	0,605051632
Pcdhgb7	1,0598153	1,1309502	1,456861472	0,776292202	0,437576476	NA
Scmh1	254,248953	0,317607368	0,125920699	2,522280864	0,011659657	0,115379686
Gtse1	13,26230486	-0,779249983	0,453713303	-1,717494238	0,085888906	0,363485758
Ddx41	737,1770366	-0,173037704	0,107889183	-1,603846639	0,108747861	0,409049888
Lpcat2	54,12016706	0,219469801	0,252844329	0,868003651	0,385392334	0,729677128
Parp12	944,7624555	0,383106987	0,140389115	2,728893804	0,006354716	0,079547008
Zxdc	243,2624277	0,071106487	0,126008695	0,564298258	0,572551167	0,841794408
Slc13a4	5,83558568	-0,019840814	0,803727439	-0,024685998	0,980305424	0,994739255
Kdm3a	439,9745115	-0,029486561	0,157515371	-0,18719799	0,851505401	0,95589686
Whrn	29,3448856	-0,681341019	0,371717203	-1,832955305	0,066809218	0,316952604
Mpp6	1621,627328	-0,166245282	0,108686918	-1,529579498	0,126120847	0,441736801
Slc26a1	5362,249442	0,169078248	0,120668812	1,401176037	0,161161438	0,497950692
Pomc	2,959478234	-0,266994693	0,901994975	-0,296004635	0,767226542	NA
Plpp1	260,5521663	0,009251245	0,297524495	0,031094061	0,975194526	0,993185764
Pde7a	116,1722495	-0,222444116	0,146837541	-1,514899495	0,129797858	0,447902843
Pde6d	162,8718923	0,065473695	0,133206238	0,491521237	0,623057836	0,863474234
Pcp4	1,368034787	2,223437784	1,619056468	1,373292302	0,169661495	NA
Pde1b	27,3048214	0,465812507	0,336079817	1,386017496	0,165741561	0,504048892
Pi4k2b	713,3418506	-0,379151866	0,10768642	-3,52088838	0,000430104	0,01312798
Mterf3	374,314015	-0,110678132	0,1181319	-0,936903008	0,348808452	0,700915372
Mir5104	0,142634598	-0,517475177	3,352475198	-0,154356154	0,877328924	NA
Fyb	242,7816663	0,120022286	0,242680915	0,494568292	0,620904889	0,862608071
Acy1	868,1791229	0,37670848	0,112953717	3,335069362	0,000852781	0,021158908
Lrrn3	16,26794866	0,65615799	0,387856454	1,691754729	0,090692749	0,373642945
Ankib1	445,0903756	-0,280565083	0,140290693	-1,999883797	0,045512813	0,2528931
Paf1	940,7443608	-0,074752813	0,0771356	-0,969109109	0,332490753	0,689692612
Robo1	313,4700593	0,713409787	0,332051004	2,148494592	0,031674486	0,206641847
Trappc3	737,4881343	0,04146363	0,079460889	0,521811809	0,601801366	0,854462419
Gbp9	96,59523879	0,354378987	0,222434924	1,593180518	0,1111197	0,412655354
Peli3	77,26075004	-0,478296271	0,213705572	-2,238108567	0,025213976	0,181772069
Otop2	0,147722973	0,780932884	3,352475198	0,232942181	0,815806304	NA
Zc3h12d	95,33992558	0,61276685	0,250592763	2,445269537	0,014474392	0,13080163
Klhl25	336,0400448	0,120125456	0,230370498	0,521444617	0,602057077	0,854563604
Cul5	455,5651722	0,147917592	0,114375823	1,293259263	0,195921405	0,54489515
Gstm4	2015,518225	0,282693786	0,168071497	1,681985296	0,092571691	0,37789723
Plekhb1	580,5723208	-0,100806584	0,236280818	-0,426638884	0,669642372	0,887039558
Col4a5	44,758718	-0,114612696	0,392543397	-0,291974585	0,770306056	0,927934035
Mefv	11,37894732	0,473617552	0,661217563	0,716280962	0,47381788	0,787824815
Egflam	15,22624031	0,987792935	0,460516616	2,144966982	0,031955487	0,207676525

Supplementary Table S1: *Serpina1* KO vs. wildtype all DEGs

Cnga1	0,087021394	-0,517475177	3,352475198	-0,154356154	0,877328924	NA
Tfr2	9617,896002	0,025084372	0,06986603	0,359035316	0,71956867	0,908631658
Patj	268,1964588	0,274899982	0,110418951	2,489608706	0,01278838	0,121731107
Il2rg	89,21694162	0,42560843	0,307382333	1,38462229	0,166167992	0,504505304
Ass1	48,14712208	0,296528012	0,325978384	0,909655444	0,363004249	0,712106207
Asgr2	5077,561084	0,092759455	0,090504726	1,024912821	0,305404324	0,664560005
Fxyd2	2,582230026	0,018009558	0,90419839	0,019917707	0,98410902	NA
Atp1b3	775,363418	0,023170552	0,117630429	0,196977532	0,84384512	0,953068489
Flot1	508,7142263	0,260366874	0,128010261	2,033953148	0,041956319	0,242190862
Bmp6	93,90946542	0,098697734	0,332505706	0,296830196	0,76659615	0,927141662
Hist1h1b	1,027032084	-2,095766915	1,444997757	-1,450359978	0,146958161	NA
Ap1s1	617,0835118	0,047552589	0,101597139	0,468050478	0,639748493	0,871049783
Ap1g2	197,2310029	-0,080635822	0,16195058	-0,497903878	0,618551794	0,8615773
Epm2a	28,61212709	0,516657564	0,347159775	1,488241441	0,136687232	0,459349518
Nptn	951,770088	-0,101975842	0,113388322	-0,899350482	0,368466006	0,716892516
Rarg	179,9148422	0,044008939	0,209250279	0,210317231	0,833420087	0,950519804
Sfrp2	0,836402822	-2,421250882	2,120280766	-1,141948237	0,253475536	NA
Ephb4	539,6756906	-0,25105992	0,152594851	-1,645271239	0,099913888	0,392993389
Ephb3	9,07182504	0,208960869	0,523505206	0,399157194	0,689777382	0,894436573
Gp1bb	0,605881664	1,800495087	2,800943509	0,642817351	0,520342623	NA
Tcea2	27,39960658	0,420175539	0,293267492	1,432738203	0,151932667	0,485352203
Got2	9833,255228	0,024559508	0,077198465	0,318134668	0,750382787	0,920556312
Tbx2	60,24261656	0,00832318	0,3077293	0,027047084	0,97842218	0,994429459
C1rb	1,205110801	0,364356825	1,602432668	0,227377307	0,820130364	NA
Vps26a	1057,094371	-0,013901129	0,091694614	-0,151602458	0,879500494	0,965356495
Phf8	328,5291932	-0,051983246	0,141999847	-0,366079591	0,714305686	0,906371815
Tmed4	1197,418342	-0,15857633	0,092674917	-1,71110301	0,087062099	0,366110999
Eaf2	10,66213343	0,498795033	0,500268186	0,997055274	0,318737681	0,676547953
Ildr1	5,454120338	1,348468617	0,978746325	1,377750887	0,168280214	0,507154685
Eif3b	2648,786986	-0,20658914	0,106010413	-1,94876271	0,051323769	0,27322396
Gbp8	86,68301589	0,21862322	0,281194567	0,777480241	0,436875488	0,76575019
Gm10584	1,61521179	1,879687847	1,400940831	1,341732503	0,179682744	NA
AA986860	26,65073734	0,038059762	0,328694492	0,115790689	0,90781843	0,9747592
Wdr25	95,54158387	-0,079038984	0,185889837	-0,425192605	0,670696275	0,887361539
Frat2	48,04899707	-0,408498423	0,272926222	-1,496735708	0,134462041	0,456327747
Igsf1	8,376537191	-0,12815992	0,556635167	-0,23024043	0,817904947	0,945488218
Tmem60	170,6562585	0,18902152	0,172867772	1,093445686	0,274198164	0,635693562
Ccdc73	0,220592835	0,059593471	3,352475198	0,017775962	0,985817581	NA
Rhno1	122,2728439	0,071387704	0,156069082	0,457410931	0,647375709	0,876230372
Tuba4a	6701,168664	-0,207568187	0,138725243	-1,496253908	0,134587501	0,456327747
Ksr2	2,487286157	-0,562423887	1,000809547	-0,561968947	0,574137177	NA
Ube2h	1766,573312	0,064210248	0,102160834	0,628521177	0,529662579	0,819097846
Rasgrp1	36,25583547	0,106757073	0,360412628	0,296207913	0,767071306	0,927464619
Kmt5a	962,6022249	-0,346857833	0,164977483	-2,102455594	0,035513386	0,220116801
Rab20	196,9269068	-0,04828085	0,13353804	-0,361551287	0,717687373	0,907735335
Rab19	8,182689145	1,233449617	0,517527156	2,383352452	0,01715576	0,144978103
Atp6v0a4	2,908306015	3,455305889	1,244771928	2,775854605	0,005505682	NA
Kcnn3	0,489630809	-0,74100367	2,454718941	-0,301869048	0,762751892	NA
A530064D06Rik	4,073566105	1,078582754	0,797357022	1,352697378	0,176152328	NA
Dlk2	0,147722973	0,780932884	3,352475198	0,232942181	0,815806304	NA
Gng2	52,05706841	0,036821608	0,27796598	0,132468037	0,894614103	0,970433882
Tubgcp2	285,7431487	0,219315959	0,161142233	1,361008558	0,173510985	0,515940471
Nin	55,73291121	0,07467263	0,267005673	0,279666828	0,779733131	0,931568564
Tmem185b	182,4892801	-0,079473851	0,142903161	-0,556137808	0,578116651	0,845288649
Daam1	1462,494667	-0,231952821	0,124177869	-1,867907879	0,061774913	0,301302009
Exosc10	711,8589429	-0,071315695	0,091468512	-0,779674819	0,435582304	0,765317365
N4bp2	277,8163494	-0,318049393	0,221549938	-1,435565251	0,151126088	0,483843582
Mink1	1492,435172	-0,193292804	0,089943435	-2,149048494	0,031630556	0,206478957
Heatr5b	360,8036015	-0,177637324	0,150343466	-1,181543362	0,237386938	0,594575536
Eps8l1	22,09009372	0,162877761	0,64890292	0,251004821	0,801810382	0,939799232
Kif13b	567,8205564	-0,624689802	0,179583688	-3,478544234	0,000504145	0,014780521
Agmat	3833,36271	0,267746204	0,083431905	3,209158476	0,001331241	0,028782722
Wdr75	287,0052105	0,180279614	0,188449071	0,956648993	0,338744451	0,694071514
Glipr1	23,7603139	0,906309471	0,441181821	2,054276555	0,039948933	0,235069225
Rec114	22,10315121	-0,061955756	0,683190307	-0,090685942	0,927742141	0,978977289
Thoc3	572,7718168	-0,002753082	0,089849214	-0,030641136	0,975555736	0,993304169

Fbrsl1	516,1259845	0,013945926	0,136095656	0,102471499	0,918382434	0,976907366
Slx4ip	54,19885745	-0,483371696	0,239660888	-2,016898545	0,043706102	0,246335109
Trmt11	172,8717875	0,145842034	0,1410654	1,033861127	0,30120109	0,661643276
Zbtb8a	56,97313364	-0,270022461	0,212725661	-1,269345972	0,204317697	0,557220166
Tex19.1	0,18660675	-1,235117516	3,340448777	-0,369745983	0,711571767	NA
Zmiz2	847,2538427	-0,222853232	0,092957405	-2,397369346	0,016513269	0,142154471
Marveld3	29,94278215	0,150710787	0,302432405	0,498328831	0,61825229	0,861339607
Lonp1	3912,11277	-0,110199865	0,063365442	-1,739116185	0,082014328	0,35403602
Vgll3	4,462377435	-0,317188748	0,709650295	-0,446964864	0,654900429	NA
1700094D03Rik	71,94699772	-0,021638507	0,182680336	-0,118450114	0,90571102	0,974052822
Elp5	525,2443506	-0,124531232	0,09636827	-1,292243093	0,196272972	0,545184577
Cyp4f14	7916,397546	0,016956076	0,093823343	0,180723423	0,856584673	0,9578959
Ect2l	1,029264543	-0,065207606	1,454107683	-0,044843726	0,964231872	NA
Mettl21b	1,251544905	-1,609199156	1,58864404	-1,01293878	0,311089404	NA
Rpl22	2260,077873	0,120589484	0,109375524	1,102527148	0,270232563	0,631046136
Zfp219	512,9079191	-0,149834354	0,137535746	-1,089421173	0,275968199	0,637060499
Zmym4	242,2787636	-0,00015413	0,112399307	-0,001371275	0,998905881	0,999803624
Ssr1	3141,00903	-0,355264479	0,112975495	-3,14461539	0,001663053	0,033991735
Myl12a	1679,317315	0,05228308	0,088601489	0,590092561	0,555128596	0,832592702
Rps10	2701,468093	0,011344757	0,124343223	0,091237436	0,927303929	0,978888597
Fam69a	270,0757271	0,372802789	0,188724553	1,975380434	0,048224991	0,262820096
Herc4	1287,031085	0,033055979	0,105106952	0,314498503	0,753142453	0,921533083
Gatm	158,4581476	-0,41507609	0,271884356	-1,526664118	0,126844554	0,443226567
Kif2c	8,450200332	-0,519067294	0,507392526	-1,023009341	0,306303429	0,665483647
Dst	1758,646644	-0,170385183	0,097040269	-1,755819364	0,079119257	0,348953931
Ppp2r1b	413,1112028	0,079164951	0,117376497	0,674453172	0,500023248	0,803112736
Sigmar1	6436,683076	0,417288496	0,108401152	3,849483955	0,000118367	0,005137817
Ccpg1	1472,540264	-0,151012523	0,115059369	-1,312474809	0,18935998	0,536693982
Cep57	251,8512827	0,231904745	0,136958658	1,693246332	0,090408589	0,373047045
P2rx7	61,13016678	0,030671527	0,270128233	0,11354432	0,909599028	0,9747592
Emc8	446,8608941	-0,048882664	0,098306742	-0,497246307	0,61901537	0,861782322
Batf	9,411376784	0,020162334	0,464148452	0,043439409	0,965351263	0,989924013
Aass	7416,882353	0,425260797	0,14312983	2,971154212	0,002966828	0,048705649
Galnt1	1017,738217	-0,182840807	0,115989412	-1,576357734	0,114943383	0,419857401
Slc25a46	885,5987315	0,049353276	0,116198491	0,424732502	0,671031687	0,887361539
Dnah8	15,28964947	0,062631367	0,51367666	0,12192761	0,902956349	0,973489035
Gbp3	174,687609	0,746036522	0,244743955	3,048232682	0,002301916	0,041756196
Tmod3	492,122248	0,007809165	0,120588874	0,064758585	0,948366216	0,985571619
Neu3	91,71313322	0,441485846	0,31362535	1,407685464	0,159224228	0,495312554
Pgap2	629,9902283	0,075895108	0,094593612	0,802328047	0,422363223	0,755522295
Pcdh7	18,24094714	0,158930828	0,417985412	0,380230563	0,703774274	0,900790644
Ugt1a6a	1353,219147	-0,304658889	0,139093427	-2,190318375	0,028501155	0,194503289
Sp4	155,3443295	-0,031211077	0,205450794	-0,151915093	0,879253904	0,965347075
Hspa1b	148,6169829	-1,325563896	0,509465093	-2,601873839	0,009271595	0,10013789
Hspd1	12350,26201	-0,031847365	0,123329021	-0,25823091	0,796228704	0,937785232
Axl	492,7123026	0,167529399	0,200340815	0,836222011	0,403030018	0,742879955
Tal2	0,113662148	0,780932884	3,352475198	0,232942181	0,815806304	NA
Tnfaip2	1201,131171	0,012012582	0,13335502	0,090079714	0,928223869	0,979201839
Rps6ka1	368,0890894	-0,289568104	0,254421982	-1,138141059	0,255061586	0,615589901
Sparcl1	149,6282717	0,045014191	0,250429918	0,179747656	0,857350678	0,958260148
Dnajc2	489,4474843	0,059234454	0,11587379	0,511198036	0,609212394	0,857753956
Rab11b	1130,505121	0,057241042	0,07535323	0,759636211	0,447472068	0,772766561
Rxrg	171,5316636	0,322222861	0,207578351	1,552295117	0,120591626	0,432066609
Rps6	4062,861592	-0,039968526	0,119913881	-0,333310255	0,738900099	0,91559509
Aqp5	0,179780087	0,059593471	3,352475198	0,017775962	0,985817581	NA
Lpar1	16,5763154	-0,195490081	0,518305924	-0,377171226	0,706046362	0,902122257
Gpaa1	529,1205031	-0,23426658	0,126068643	-1,858246227	0,063134056	0,305356089
Eef1a1	106355,6156	-0,083737399	0,094730823	-0,883950921	0,376722707	0,722583339
Got1	5380,430231	0,853720016	0,404063675	2,112835348	0,034614863	0,217104886
Il6st	2223,301225	-0,136868532	0,130055735	-1,052383668	0,292623559	0,654074594
Thrsp	14338,27021	0,587661132	0,398375618	1,475143321	0,140174016	0,465234266
Rps5	5252,674627	0,011895753	0,131870091	0,090208118	0,928121834	0,979171464
Gtf2i	2251,231594	-0,137735621	0,081220208	-1,695829459	0,089918182	0,371941885
Paxbp1	408,1044346	-0,197063786	0,134021071	-1,470394057	0,14145506	0,467194607
Pex2	1149,675519	-0,028907288	0,110246143	-0,262206798	0,793162013	0,936746676
Rrm2	123,6889134	-0,051957506	0,318616217	-0,163072382	0,870461444	0,961775826

Supplementary Table S1: *Serpina1* KO vs. wildtype all DEGs

Syngr2	1650,608684	0,039200722	0,097010913	0,404085693	0,686149706	0,893161227
Rrm1	277,6572369	-0,075577792	0,177950802	-0,424711724	0,671046836	0,887361539
Fbp1	24787,07029	0,244851888	0,120807451	2,026794577	0,042683425	0,243203202
Abcb10	1082,349254	-0,44898573	0,134084348	-3,348532004	0,000812409	0,020557413
Rnf5	1715,86657	-0,247313675	0,083605511	-2,958102543	0,003095391	0,050282244
Exosc9	367,919453	0,028762218	0,130852632	0,219806186	0,826022102	0,947537943
Shoc2	663,4928348	-0,190375666	0,133707083	-1,423826336	0,15449675	0,489429924
Dgke	33,37418221	-0,452402477	0,252920709	-1,788712671	0,073661101	0,334275378
Itgax	26,45198722	0,308305683	0,395268233	0,779991048	0,435396145	0,765139999
Lmna	991,9418995	-0,135127114	0,101839254	-1,32686669	0,184552785	0,530941722
Rabep1	1396,060541	-0,186459357	0,104675041	-1,781316301	0,074860793	0,338112818
Mfn2	2416,46706	0,131932454	0,123372815	1,069380269	0,284898354	0,646107905
Cops2	1434,87138	-0,054661091	0,107555512	-0,508212823	0,611304099	0,858645358
Tead2	20,67117766	-0,180560751	0,360068432	-0,50146232	0,61604579	0,860327605
P4hb	46829,43537	-0,080280933	0,10319168	-0,777978736	0,436581549	0,76575019
Nfkbil1	147,9386279	-0,23778218	0,162491499	-1,463351503	0,143371222	0,470500538
Pcna	505,5572005	-0,015804875	0,122474331	-0,129046426	0,897320913	0,971044462
Pck1	22878,08717	0,043712631	0,243873458	0,179243085	0,857746834	0,958260148
Thbs4	0,18725647	-1,238536202	3,340198538	-0,370797181	0,710788601	NA
Eif2ak2	414,3898013	0,10231111	0,153644568	0,665894739	0,505478383	0,805643837
Cox6a2	1,381998053	0,25732457	1,300889855	0,197806578	0,8431964	NA
Cox5b	4724,433434	0,007951989	0,0902886	0,088073016	0,929818643	0,980265392
Nsg1	16,48725818	0,299901896	0,40738063	0,736171222	0,461626504	0,781516914
Dcaf6	799,3418315	0,294900298	0,121251678	2,432133736	0,015010164	0,133890644
Casp14	0,087021394	-0,517475177	3,352475198	-0,154356154	0,877328924	NA
Muc13	0,274362713	1,389394708	3,349408814	0,414817893	0,67827522	NA
Lasp1	4333,63206	-0,273532216	0,079851151	-3,425526271	0,00061361	0,017019391
Dnmt3aos	0,086476712	0,780932884	3,352475198	0,232942181	0,815806304	NA
Cep55	4,30202657	-0,988046552	0,923988385	-1,069327892	0,284921947	NA
Rbms3	75,82838435	0,516824956	0,248269119	2,081712613	0,037368728	0,226546639
Ffar2	0,985996662	0,696162775	1,559169566	0,446495872	0,655239094	NA
Usp19	1620,029146	-0,191708146	0,084005328	-2,282095073	0,022483728	0,168400998
Tsku	1283,72863	-1,016420649	0,399368264	-2,545071153	0,010925547	0,111159545
Csrp2bp	490,8738266	-0,071109382	0,101910837	-0,697760747	0,485326831	0,79430833
Hnrnpf	1465,402262	-0,09814945	0,114194348	-0,859494822	0,390067575	0,733963887
Med23	259,4073705	-0,104302131	0,134117435	-0,777692561	0,436750279	0,76575019
Set	1857,074632	-0,178490244	0,123695372	-1,442982391	0,149025432	0,480193058
Scn3b	0,895597899	0,875775641	1,885924545	0,464374698	0,642379314	NA
Jmjd1c	992,9384174	-0,184886471	0,154714547	-1,195016722	0,232080497	0,58906385
Aig1	327,246028	0,496387088	0,152235403	3,260654733	0,001111553	0,025697163
Eya3	528,55998	0,008089905	0,078694634	0,102801231	0,918120728	0,976907366
Zic5	0,086476712	0,780932884	3,352475198	0,232942181	0,815806304	NA
Zfand6	1755,408891	0,00027876	0,134544673	0,002071879	0,998346881	0,999803624
Nipa2	524,0642161	-0,040192442	0,139370626	-0,288385317	0,773051806	0,928591792
Dnaja3	2779,220243	-0,075362114	0,080870549	-0,931885772	0,351395561	0,702791968
Slco1a6	0,086476712	0,780932884	3,352475198	0,232942181	0,815806304	NA
Mccc1	1515,093024	0,127845621	0,109810537	1,164238193	0,244327496	0,602993225
Tenm4	2,853334326	1,276989811	1,225071896	1,042379484	0,297235774	NA
Sars2	290,5444222	0,039687233	0,109669295	0,361880992	0,717440965	0,907650451
Anxa9	36,83934549	0,421411257	0,299379778	1,4076143	0,159245311	0,495312554
Isyna1	393,7246967	1,43569591	0,895825714	1,602650925	NA	NA
Fahd1	1456,680177	0,008011923	0,120470841	0,06650508	0,946975713	0,98514655
Plbd2	1941,997918	-0,168322222	0,106152383	-1,585665976	0,112815101	0,41594735
Cyp2d40	1581,28007	-0,274434429	0,285543874	-0,961093739	0,336505037	0,69270916
Rgs19	45,79012304	0,188725617	0,277598326	0,679851424	0,496598542	0,800461286
Immp2l	486,3445216	0,319331329	0,142765104	2,236760388	0,025302005	0,181959388
Ssbp3	1505,73436	0,039513507	0,08112767	0,487053395	0,626220492	0,865266164
Tdrd12	0,287704936	-0,589751774	3,345560051	-0,176278938	0,860074808	NA
1600014C10Rik	3139,336845	-0,02724909	0,079770168	-0,341594995	0,732655704	0,912764338
1600014C23Rik	0,20013886	1,337107854	3,350237755	0,399108348	0,689813372	NA
Slc35a5	322,857705	-0,007913191	0,131743715	-0,060065038	0,952103835	0,986187919
Pinx1	112,3230562	-0,161630368	0,250368827	-0,645569057	0,518558482	0,813725618
Mrgbp	118,2758326	-0,199909269	0,165953921	-1,204607082	0,228355021	0,584236803
Cnksr1	5,978663624	0,841898686	0,68648409	1,226392132	0,220051146	0,574490867
Mapre1	753,1697358	-0,075363537	0,099383735	-0,758308559	0,448266285	0,77297141
Adck1	385,2707189	0,133864687	0,119060587	1,1243409	0,260868434	0,621964694

Eif2ak1	1394,14261	0,004063261	0,083109469	0,048890465	0,961006587	0,988470616
Btrc	375,0330948	-0,067598159	0,120786331	-0,55965074	0,575717688	0,843861602
P4ha1	300,6814902	-0,656747963	0,217323425	-3,021984235	0,002511236	0,043600932
Zbtb14	164,4230934	-0,01452743	0,145794576	-0,099643141	0,920627642	0,977341679
Ikbkb	1256,645716	-0,100400438	0,099039525	-1,013741107	0,310706303	0,669972995
Adcy9	691,5249497	0,011961543	0,180164529	0,066392329	0,947065477	0,985157551
Pcgf2	172,6618536	-0,463994714	0,250893393	-1,84937	0,064404404	0,309053295
C2cd5	154,3179963	-0,130658703	0,142374655	-0,917710412	0,358770495	0,708060636
Wdr82	1037,798439	0,032593418	0,108811419	0,299540415	0,76452774	0,925980373
Csprs	1,418582247	-2,615663183	1,369732346	-1,909616277	0,056182638	NA
Lima1	1507,74467	-0,097628851	0,147063104	-0,663856862	0,506781928	0,806249795
Sap30l	360,1895655	0,044568294	0,099973401	0,445801521	0,655740623	0,879592859
Cped1	946,3537355	-0,245196131	0,123110223	-1,991679685	0,046406214	0,256037813
Serpini1	5,915187649	-0,077728834	0,701590193	-0,110789511	0,911783263	0,975612405
Gabpb1	216,9950542	0,093502509	0,114780033	0,814623475	0,415287869	0,751551608
Gab2	54,91204373	0,205641279	0,243410758	0,844832334	0,398204459	0,739593694
Idh1	12157,32985	0,148162049	0,102738405	1,442129151	0,149265943	0,480852142
Hyal2	976,8630521	0,066319538	0,094389333	0,702616869	0,482294547	0,793477208
Serpina1d	36726,78572	-2,771899239	0,090336145	-30,68427632	9,23E-207	1,23E-202
Pea15a	501,7416573	-0,105535089	0,163499774	-0,64547544	0,518619129	0,813725618
Tekt4	0,279910125	-1,713254196	3,310169948	-0,517572881	0,604756307	NA
Serpina1a	24564,22893	-7,677876464	1,101299547	-6,971651343	3,13E-12	2,79E-09
Flad1	1095,023633	-0,136540375	0,169557227	-0,805276058	0,420660395	0,75436738
Tmcc3	342,2601199	-0,284332922	0,124889745	-2,276671495	0,02280585	0,169855937
Cobll1	930,8037342	-0,22330565	0,137805406	-1,620441874	0,105137391	0,402976428
Wdr59	294,0596129	-0,084219787	0,142426904	-0,59131937	0,554306454	0,832018326
Supt20	265,301116	-0,135948411	0,134078807	-1,013944068	0,310609442	0,669972995
Clstn1	89,84164166	0,133454363	0,24656424	0,541255954	0,588331175	0,848843045
Prokr1	1,238620917	-0,472007073	1,291153547	-0,365570055	0,714685923	NA
Huwe1	3798,873232	-0,131575592	0,104973383	-1,25341862	0,210053398	0,562470273
Mark3	846,9418788	-0,140060768	0,094770491	-1,477894297	0,139436073	0,464404798
Klf13	468,3534699	0,148701075	0,142354347	1,044584013	0,296215272	0,657387444
Slc15a2	73,43018137	0,59298659	0,821528955	0,721808509	0,470412217	0,784744314
Midn	902,8040597	0,068168672	0,18094588	0,376735144	0,706370443	0,902122257
F9	5827,216024	0,148710578	0,091838653	1,61925914	0,105391518	0,403011718
Zfp24	399,7654826	0,018360273	0,145693466	0,126019878	0,89971619	0,972141416
Stard10	19297,60467	0,098883321	0,070346544	1,40565997	0,159825119	0,49581126
Fads3	113,1944824	0,311665477	0,182187539	1,710684924	0,087139292	0,366110999
Mlst8	227,4358234	-0,093651473	0,143599153	-0,652172877	0,514289655	0,811764185
Tpx2	20,19639121	-0,836922475	0,668436161	-1,252060442	0,210547841	0,562962817
Ddx6	1845,694625	-0,116415718	0,114697823	-1,014977569	0,31011652	0,669240962
Ccdc86	214,1698709	0,264749337	0,193530588	1,367997381	0,171312896	0,511550248
Zfp839	203,7874529	-0,049478843	0,125157895	-0,395331379	0,692598344	0,895338108
Puf60	1479,83631	0,066764057	0,091032098	0,733412264	0,463307021	0,781663619
Ttc19	256,1691821	-0,11024556	0,177213825	-0,622104741	0,533872998	0,821423422
Hpf1	157,8072349	-0,021963036	0,170166221	-0,12906813	0,897303739	0,971044462
Trmo	60,7736653	0,251443128	0,195557588	1,285775359	0,198521472	0,548694141
Pcf11	498,7541878	0,03725217	0,125359005	0,297163892	0,766341387	0,926944289
Gk	1922,676986	0,14342149	0,14772262	0,970883747	0,331606173	0,688274004
Adra1b	2444,777291	0,177145058	0,12822637	1,38150256	0,167124491	0,506067344
Bglap	0,093953095	-0,517475177	3,352475198	-0,154356154	0,877328924	NA
Cp	32755,74948	0,105292107	0,086243884	1,220864625	0,22213728	0,576987235
Cox8a	5802,242925	0,032316358	0,134270329	0,240681306	0,809802128	0,94241441
Cox5a	3866,691999	0,059558463	0,125074528	0,476183795	0,633943443	0,867989994
Gbas	302,9513567	0,009414356	0,13939368	0,0675379	0,946153491	0,984653942
Abca2	1730,741153	-0,178054512	0,129815426	-1,371597489	0,170188776	0,510344993
Shroom2	400,2941949	-0,042890024	0,141634201	-0,302822507	0,76202513	0,92545349
Fam83a	16,22719935	0,578052118	0,44990544	1,284830248	0,19885161	0,548812381
Npr2	887,2892842	0,019952197	0,265965557	0,075017974	0,940200411	0,983128178
Ubtd2	62,63443678	-0,098235187	0,249592598	-0,393582132	0,693889575	0,896031076
Rab6b	25,85368216	0,30915558	0,317845659	0,972659437	0,330722592	0,687518322
Klf8	0,68821902	1,939083024	2,272761341	0,853183741	0,393557418	NA
Sh2d7	0,368315808	0,66341466	2,61309566	0,25388074	0,799587694	NA
Ifi209	54,85178802	0,570317591	0,30379314	1,87732215	0,060473954	0,29803066
Maml2	31,55526994	-0,137936996	0,412056984	-0,334752234	0,737811995	0,91507318
Cfap100	5,365718646	-0,301350908	0,732188451	-0,411575609	0,680650512	NA

Phf21a	391,2756518	-0,179184348	0,16443356	-1,089706673	0,275842376	0,637060499
Cul9	146,2773993	0,484189826	0,182287104	2,656193533	0,007902825	0,091080054
Snapc5	367,0778437	0,209740904	0,100532692	2,086295518	0,036951862	0,225266506
B3gnt5	4,445569342	0,681853036	0,728075332	0,936514404	0,3490084	NA
Irf1	1020,510488	0,156896229	0,171056428	0,917219133	0,359027823	0,708359351
Llgl1	209,3699951	-0,109311635	0,149236202	-0,732473981	0,463879318	0,781663619
Cnot4	331,8982297	-0,033016278	0,112233683	-0,294174418	0,768624628	0,927598042
Gdap10	9,102442249	-0,373820752	0,534391326	-0,699526234	0,484223224	0,79420977
Cd200r4	2,523663674	1,785778652	1,075561908	1,660321584	0,096849776	NA
Npc1l1	0,416367781	-1,314797021	2,181370552	-0,602738962	0,546682358	NA
Gtf3c1	1327,014624	-0,23147624	0,088603586	-2,612492898	0,008988456	0,098497269
Sgsm3	210,5065562	-0,217052699	0,134804937	-1,610124261	0,107370732	0,406762331
Lbx2	3,670410265	0,062985933	0,8550229	0,073665784	0,941276325	NA
Sep-03	2,722837046	0,476245763	1,038358386	0,45865259	0,646483665	NA
Cln8	591,7462535	-0,056741258	0,139353591	-0,407174706	0,683879678	0,892293506
Insl3	2,430722382	0,451711036	1,007263031	0,448453901	0,653825649	NA
Timm10	353,1116616	0,01216531	0,154403004	0,078789335	0,937200187	0,982315115
Timm8b	852,205356	0,05706052	0,123449309	0,462218223	0,643924836	0,873795284
Spdef	1,003521755	2,559690512	1,657166924	1,544618394	0,122438594	NA
Zfp292	305,63105	0,155358927	0,222880059	0,697051712	0,485770432	0,79430833
Mrps12	934,3701312	-0,219008475	0,081795871	-2,677500367	0,007417377	0,087576343
Mzt2	279,1409981	0,24943061	0,144134399	1,730541854	0,08353351	0,357477432
Lrch3	214,3134873	0,120225111	0,126779804	0,948298602	0,342977462	0,695663554
Pnma2	0,12663974	0,780932884	3,352475198	0,232942181	0,815806304	NA
A830010M20Rik	85,75826731	0,105261847	0,24201419	0,434940807	0,663605394	0,883893982
Mir3473a	0,086476712	0,780932884	3,352475198	0,232942181	0,815806304	NA
Eqtn	0,695723665	2,884950839	2,148561217	1,342736161	0,179357421	NA
Pbx3	54,77121934	-0,639221288	0,241707782	-2,644603675	0,008178665	0,09289768
Ppp1r3f	9,408822611	-0,406910436	0,517466569	-0,786351159	0,431661789	0,7622164
Mbd5	143,4947235	0,017171445	0,150329534	0,114225358	0,909059151	0,9747592
Rbm41	87,22332635	-0,210032293	0,180888942	-1,161111844	0,245596419	0,605051632
Kmt2b	608,9561472	-0,087873066	0,092610157	-0,948849118	0,342697356	0,695589013
Kdelr2	2483,905699	-0,387908388	0,108186242	-3,585561161	0,000336354	0,01102137
Zfp319	112,9921465	-0,079806829	0,190831715	-0,418205273	0,675797041	0,889164486
Adcy3	24,95184195	-0,019196813	0,329658707	-0,058232386	0,953563524	0,986479135
Calml4	13,3501648	-0,205231945	0,567359229	-0,361731924	0,717552369	0,907650451
Yipf2	664,5598115	-0,240281267	0,111794591	-2,149310298	0,031609811	0,206478957
Tymp	1058,256856	-0,015056962	0,186505589	-0,080731963	0,935655117	0,982081594
Prokr2	0,093953095	-0,517475177	3,352475198	-0,154356154	0,877328924	NA
Ptbp3	1391,722975	0,164265487	0,165026479	0,995388668	0,319547268	0,677329731
Gm2027	0,093953095	-0,517475177	3,352475198	-0,154356154	0,877328924	NA
Gria4	0,969736903	-0,662420356	1,603545496	-0,413097325	0,679535309	NA
Bub3	688,5750124	-0,101544914	0,110581747	-0,918279158	0,358472736	0,707901565
Wdr12	346,92074	-0,078280305	0,172000471	-0,455116807	0,649025208	0,876446263
Shf	178,7812087	0,214838954	0,128969609	1,665810692	0,095751126	0,385586553
Nutf2	43,72559181	-0,365916729	0,251824612	-1,453061821	0,14620659	0,476043815
Cnot8	1100,062579	-0,139457976	0,073434816	-1,899071643	0,057555057	0,289947338
Prdm9	130,505442	-0,1367538	0,153880106	-0,888703575	0,374162409	0,720878692
Klhdc8a	66,87632279	0,296956609	0,212482429	1,397558421	0,162245712	0,498749809
Slc39a14	2708,968018	0,140458673	0,108788885	1,291112346	0,196664724	0,545819118
Slc30a6	329,4518538	0,022356449	0,093377779	0,23941937	0,810780412	0,942631822
Ly6g5b	4,942928283	0,052976841	0,736619579	0,071918861	0,94266648	NA
Csnka2ip	0,12663974	0,780932884	3,352475198	0,232942181	0,815806304	NA
Bphl	3276,755742	0,303800608	0,107904447	2,815459574	0,004870752	0,067618992
Apopt1	470,7851216	0,120622038	0,116064542	1,039266912	0,298680619	0,659465103
Srsf6	2011,043388	0,054981068	0,108526675	0,506613398	0,612426106	0,858888222
Mlh1	419,0753574	-0,0114235	0,131584935	-0,086814652	0,930818841	0,980584977
Nsmce2	216,3949646	0,106134466	0,119968947	0,884682819	0,376327725	0,722097522
Trap1	5361,536473	-0,101165381	0,062501616	-1,618604237	0,105532443	0,403046295
Syf2	898,6714802	0,01066548	0,099760846	0,106910482	0,914859998	0,976451566
Zwilch	20,81231845	-0,398162702	0,412753488	-0,964650121	0,334720086	0,691301202
Snrpg	207,8808768	0,097254711	0,198081572	0,490983133	0,623438377	0,863525452
Them4	465,2832568	-0,36742363	0,140234907	-2,620058285	0,008791474	0,097295708
Them7	525,5774954	0,446901423	0,172054431	2,597442105	0,009392095	0,100691997
Slc7a13	0,093953095	-0,517475177	3,352475198	-0,154356154	0,877328924	NA
Cbx5	619,2158565	-0,086982291	0,126083037	-0,689881	0,490269025	0,797285888

Supplementary Table S1: *Serpina1* KO vs. wildtype all DEGs

Kctd9	246,6627077	0,106862339	0,133322025	0,801535518	0,422821691	0,755522295
Six3	0,093953095	-0,517475177	3,352475198	-0,154356154	0,877328924	NA
Six2	0,147722973	0,780932884	3,352475198	0,232942181	0,815806304	NA
Sin3a	690,0574489	0,023331181	0,099319911	0,234909404	0,81427905	0,944076707
St3gal5	1381,295229	0,092240619	0,213638596	0,431760087	0,665915791	0,885041079
St8sia1	1,864552307	-0,827752105	1,022727281	-0,809357607	0,418309479	NA
St6galnac4	46,82228564	0,730194657	0,345509289	2,113386474	0,034567703	0,217104886
Coro1c	599,3412099	-0,10938873	0,109057633	-1,003035979	0,315843502	0,67419376
Shcbp1	10,05674979	-1,637366902	0,612496116	-2,673269035	0,007511598	0,088167301
Mir1938	0,443168919	2,196464572	3,313880474	0,662807421	0,5074539	NA
Usp6nl	449,9505853	0,275222729	0,192732094	1,428006738	0,153289919	0,488120445
Fbn1	64,28925609	-0,102251533	0,303831593	-0,336540161	0,736463572	0,914722813
Pemt	7715,690784	0,1262827	0,064852192	1,947238715	0,051506122	0,273331789
Clmn	2225,730115	0,177621742	0,13470133	1,318633913	0,18729153	0,533703441
Col6a5	4,255331443	0,505282194	1,028666091	0,491201371	0,62328403	NA
Evpl	10,74672653	0,566508228	0,531130933	1,066607484	0,28614912	0,64794415
Amz2	419,5482973	0,188528005	0,090137924	2,091550337	0,036478759	0,2237085
2810013P06Rik	163,4241475	-0,018827626	0,17899684	-0,105184126	0,916229707	0,976559041
Mira	0,294636637	-0,629863441	2,754870789	-0,228636292	0,819151615	NA
Ebf3	1,285344472	3,772151906	1,681544107	2,243266705	0,024879621	NA
Klra7	1,862646091	0,717013506	1,202768015	0,59613616	0,551084269	NA
Cldn18	0,552720398	-1,795161334	2,033496011	-0,882795601	0,377346717	NA
Tead3	254,4960135	-0,233310871	0,122857678	-1,899033705	0,057560045	0,289947338
Rasa4	47,93086516	0,467201923	0,322528295	1,448561043	0,147460207	0,47753781
Gatad2a	1185,449447	-0,231487029	0,095928936	-2,413109523	0,015817066	0,137992723
Ece2	156,069822	-0,003113778	0,161250971	-0,019310134	0,9845937	0,995554769
Dus3l	378,648899	-0,106121367	0,122231362	-0,86820081	0,385284411	0,729677128
Rrp36	374,13177	-0,02528344	0,127298093	-0,198616017	0,842563124	0,952289345
Slc22a7	986,032305	0,143364677	0,399475338	0,358882423	0,719683049	0,908631658
Cbs	14906,6273	0,027969334	0,126893038	0,220416615	0,825546707	0,947537943
Map3k7cl	0,207615244	0,059593471	3,352475198	0,017775962	0,985817581	NA
Cyyr1	14,70846561	-0,073282923	0,511049997	-0,143396777	0,885976828	0,96772371
Slc7a4	27,93748654	0,711673781	0,31436125	2,263872478	0,023581959	0,173678048
Senp1	155,2126842	-0,132936145	0,154960619	-0,857870506	0,390963972	0,734518115
Schip1	0,259974817	0,586045239	3,35071904	0,174901337	0,86115716	NA
Ctc1	194,5304613	0,044421476	0,172428237	0,257622979	0,796697894	0,937923049
Zfp318	603,0527992	-0,104762944	0,116437627	-0,899734449	0,368261586	0,716740303
Hacd3	3809,146771	0,007837765	0,097949716	0,080018252	0,936222739	0,982191945
Tesc	9,769253448	0,895877177	0,530380745	1,689120854	0,091196272	0,374798348
Snrpf	332,9018464	-0,065139599	0,176435182	-0,369198469	0,711979797	0,905478699
Ndufa11	1327,647022	-0,011561591	0,106445088	-0,108615543	0,913507432	0,976232707
Draxin	0,215799707	0,059593471	3,352475198	0,017775962	0,985817581	NA
Polr3gl	227,7075731	0,325662507	0,161083001	2,021706234	0,04320671	0,245174236
Krt28	0,259974817	0,586045239	3,35071904	0,174901337	0,86115716	NA
2010003K11Rik	208,804286	0,722090387	0,253324431	2,850456956	0,004365646	0,062222089
Eif1ad	530,1104271	-0,085885521	0,106604066	-0,80564958	0,420444929	0,75436738
Maf1	404,5233935	0,060344653	0,132587501	0,455130782	0,649015154	0,876446263
Srpx2	54,88882467	-0,127103152	0,257102752	-0,49436714	0,621046916	0,862626101
Glod5	1,043356995	2,622594966	1,930825794	1,358276326	0,174376019	NA
Aimp1	531,6024072	-0,029391349	0,116010701	-0,253350326	0,79999751	0,939161109
Apoc4	13614,2271	0,191267812	0,070826512	2,700511527	0,006923294	0,084138787
Plcb4	24,60892414	0,216688763	0,450165942	0,481353082	0,630265577	0,866281784
Rnasel	51,92877009	0,00357215	0,292359663	0,01221834	0,990251417	0,997113143
Ik	2053,024187	-0,201068259	0,093901013	-2,141278907	0,032251553	0,208496621
1110008P14Rik	433,0990147	0,072568271	0,10190955	0,712085087	0,476412088	0,789577814
1700001O22Rik	0,679711697	-0,170846358	1,823129679	-0,09371048	0,925339145	NA
Sypl	1065,790111	0,004414431	0,122717772	0,03597222	0,97130451	0,992121884
Celf1	2042,35482	-0,142410604	0,09445605	-1,50769172	0,131633446	0,451580071
Fdxacb1	100,937581	0,041570058	0,18365001	0,22635478	0,820925495	0,946779723
Trat1	0,875820927	-0,78390984	1,950298406	-0,401943537	0,687725581	NA
F730035M05Rik	1,118606757	1,575861739	1,941600333	0,811630341	0,417003776	NA
C77370	0,369958895	0,666831354	3,099090077	0,215170046	0,829634744	NA
Hsd11b1	15349,05538	0,084360529	0,115418668	0,730908879	0,464834812	0,781663619
Nemp2	13,6384543	0,10943455	0,471830026	0,231936384	0,816587425	0,944998904
Ezh2	103,1681419	0,243884546	0,148667984	1,640464475	0,100908629	0,394458322
Rhbdd1	532,4934585	0,104938728	0,121956017	0,860463721	0,389533474	0,73337178

9330136K24Rik	1,909318379	-0,575885276	1,121198315	-0,513633733	0,607508091	NA
Cks2	526,4888924	0,167004713	0,096024628	1,739186261	0,082002005	0,35403602
Nfx1	982,642191	0,088404468	0,121260448	0,72904619	0,465973408	0,781831056
Uba7	310,4775711	0,375914675	0,193791526	1,939789021	0,052405335	0,276322766
Ehhadh	8005,475555	0,277536764	0,162396677	1,709005188	0,087449989	0,366609877
Actr3	1705,624316	0,036408776	0,080916662	0,449954004	0,652743606	0,877662367
Pi16	5,162628218	0,695126887	0,775241355	0,896658676	0,369901078	NA
Crot	14877,90618	-0,133720904	0,096935878	-1,379477926	0,167747448	0,506611452
Srgap1	21,86882899	-0,406807207	0,365287943	-1,113661742	0,265424328	0,626006345
Ocstamp	29,12915871	0,690276727	0,295327937	2,33732282	0,019422405	0,155304678
Rtf1	618,0088635	-0,069292051	0,096594699	-0,717348383	0,47315916	0,787502227
Hyal6	0,093303375	-0,517475177	3,352475198	-0,154356154	0,877328924	NA
Abcb8	733,2724949	0,161670661	0,103926429	1,555626051	0,11979704	0,430528664
Cyp4a32	746,6627384	-0,118347305	0,187513844	-0,631139028	0,527949624	0,81852028
Cyp3a57	0,327323282	0,664081931	2,690749744	0,246801819	0,805061603	NA
Bahcc1	221,1963095	-0,184399494	0,144200316	-1,278773159	0,200976949	0,55202186
Paqr3	85,76796195	-0,554247532	0,193575478	-2,863211488	0,004193705	0,060944211
Usp49	65,35876157	-0,061736073	0,211501989	-0,291893579	0,770367993	0,927934035
Dnajc6	1,229912511	0,739124639	1,839301823	0,401850653	0,687793941	NA
Inf2	1188,323678	-0,057144162	0,113737172	-0,502422919	0,61537006	0,859955845
Exoc8	191,4743723	0,164410484	0,179964836	0,913570047	0,360942811	0,710581841
Nsfl1c	1468,568852	0,053662556	0,084438657	0,63552119	0,525088551	0,816743321
Zfp273	62,31278088	-0,142208491	0,226294275	-0,628422841	0,529726978	0,819097846
Bhlhb9	42,41799051	0,213636976	0,272632682	0,783607361	0,433270535	0,763060702
Enkd1	14,94338833	0,01806008	0,387014056	0,046665179	0,962780083	0,988622522
Helz	189,3751525	-0,051657782	0,179646997	-0,287551606	0,773689994	0,928621142
D830013O20Rik	1,338605583	1,325530518	1,436189433	0,922949638	0,356033451	NA
Wrnip1	1349,883377	-0,13117169	0,119420439	-1,098402343	0,27202884	0,633801597
Rnf121	379,2525438	-0,246271935	0,113086344	-2,177733639	0,029425873	0,197586387
Sv2c	0,613384336	-0,041692855	2,038569482	-0,020452016	0,98368279	NA
Nipal3	294,8850451	0,10505164	0,167142833	0,628514178	0,529667162	0,819097846
Ramp1	96,53824052	0,306309052	0,210017898	1,45849023	0,144705466	0,472725517
Gulo	9205,705703	0,218655419	0,120516149	1,814324639	0,06962776	0,322876701
Nmnat2	5,280071381	-0,466835115	0,808946511	-0,577090214	0,563878515	NA
Tmem229b	48,32879121	0,167088099	0,300471951	0,556085513	0,578152398	0,845288649
Zbtb1	183,1917741	0,224387949	0,161127589	1,392610357	0,163737649	0,501491096
Slc26a11	143,7926639	-0,271977193	0,176131909	-1,544167629	0,122547729	0,434777077
Klhl8	72,54991864	-0,386539713	0,211555145	-1,827134541	0,067679543	0,31878896
Dcaf12l1	61,56785119	0,026874319	0,264092998	0,101760817	0,918946528	0,976907366
Slitrk4	0,227324296	1,389394708	3,349408814	0,414817893	0,67827522	NA
Prss35	0,113662148	0,780932884	3,352475198	0,232942181	0,815806304	NA
Opa3	842,5082932	-0,27530054	0,137177711	-2,006889739	0,044761405	0,25069762
Rpl23a	3666,137187	0,026365107	0,111455409	0,236552962	0,813003615	0,943739284
Mettl25	57,88035484	0,113152252	0,226926453	0,498629625	0,618040331	0,861339607
Mbnl2	1053,235088	-0,408384021	0,171318581	-2,38376957	0,017136329	0,144905489
Cercam	7,446817688	0,053957888	0,527629907	0,102264651	0,918546612	0,976907366
Zranb1	781,5081869	-0,047527269	0,104715962	-0,453868423	0,649923532	0,87680086
Tmem176b	6140,870516	0,148889002	0,129383176	1,150760143	0,249830926	0,60896459
Fam71d	1,195718164	-0,261694049	1,407936342	-0,185870654	0,852546196	NA
Nxpe2	500,0869625	0,129195925	0,548159179	0,235690527	0,813672822	0,943942377
Psma6	2527,900774	0,102390473	0,07958041	1,286629121	0,198223588	0,54821083
Batf2	13,28014964	-0,259282294	0,424526007	-0,610757149	0,541360366	0,826407188
Spata17	5,666331442	-0,259677119	0,622458297	-0,417179947	0,676546791	0,889512888
Atg7	1057,999904	0,059179635	0,081124409	0,729492334	0,465700555	0,781663619
Armcx1	35,91918415	0,048892152	0,313160487	0,156124908	0,875934565	0,96416635
Snx7	592,2441466	0,057893026	0,108246544	0,53482563	0,592770438	0,851332066
Dnmbp	790,8089307	-0,050297715	0,180682476	-0,278376279	0,780723526	0,93182516
Coro7	362,9106282	0,332045173	0,208656008	1,591352083	0,111530362	0,413513675
Phf23	492,3037909	0,138403319	0,144238873	0,959542427	0,337285557	0,69270916
Ttll9	0,274382903	-0,66487896	3,339949097	-0,199068591	0,84220909	NA
4933427D14Rik	69,73732632	-0,073701486	0,186866743	-0,394406649	0,693280835	0,895850714
Hsf2bp	11,80749526	0,432748144	0,524588795	0,824928302	0,409412297	0,746907036
Skida1	14,00242354	-0,450396108	0,570882121	-0,788947651	0,430142603	0,761019227
Anks3	308,6498075	-0,161513977	0,131640959	-1,226927995	0,219849655	0,574490867
Capsl	6,068968198	0,444327543	0,729309898	0,609243813	0,542362844	0,826611597
Dynlrb2	0,25327948	1,389394708	3,349408814	0,414817893	0,67827522	NA

Supplementary Table S1: *Serpina1* KO vs. wildtype all DEGs

Shisa9	2,030177129	0,588829153	0,982247183	0,59947146	0,548858536	NA
Zfp131	317,0948866	-0,109466281	0,193432591	-0,56591436	0,571451999	0,841381253
Eif2b2	427,3829355	-0,0727818	0,111562612	-0,652385229	0,514152691	0,811764185
Pofut2	406,9107848	-0,096560792	0,143548511	-0,672670107	0,501157189	0,803641654
Snora7a	3,248749433	0,56794375	0,800649618	0,709353676	0,478105027	NA
Cd164l2	1,261545639	2,067631756	1,853466759	1,115548334	0,264615518	NA
Ptcd2	706,2641356	-0,01926761	0,095827445	-0,201065678	0,840647219	0,951708907
Ubap2	571,323228	-0,147165476	0,108438973	-1,357127158	0,174740811	0,517225036
Hint2	1381,78381	0,135765437	0,138853825	0,977757993	0,328194032	0,685487182
Pygo2	332,3906727	-0,175101947	0,120843506	-1,448997577	0,147338259	0,477508134
Ptges3l	16,04949346	0,202487945	0,382225757	0,529760074	0,596278291	0,853075785
Rasl11a	12,00240017	0,644935187	0,446323015	1,444996484	0,148458873	0,479060264
Cd177	1,162704658	0,291421206	1,579221301	0,184534749	0,853593969	NA
Ubac2	717,052071	-0,03693485	0,085609577	-0,431433626	0,666153104	0,885111436
Zswim7	144,0459959	0,327278357	0,172740825	1,894620776	0,05814266	0,291422114
Lman1	5906,027317	-0,25149718	0,126036388	-1,995433101	0,045995664	0,254729092
Gpx8	25,64599268	0,088093857	0,350131557	0,251602164	0,801348586	0,939799232
Gm4787	7,314784455	0,269636426	0,549316227	0,490858293	0,623526677	0,863558287
Bend6	3,481061746	0,554633592	0,788888866	0,703056686	0,482020424	NA
Epb41l5	1216,311352	-0,062404047	0,118993007	-0,524434573	0,599976315	0,854422974
Pik3cd	114,8879322	0,512492789	0,192187185	2,666633521	0,007661517	0,089207724
Begain	0,086476712	0,780932884	3,352475198	0,232942181	0,815806304	NA
Kcnj16	25,15480352	-0,010696906	0,304857077	-0,035088265	0,972009359	0,992423486
Ptprs	109,3937343	0,265159415	0,195164289	1,358647198	0,174258411	0,517013028
Elmod3	454,5922638	-0,179941038	0,171248378	-1,050760539	0,293368584	0,65443761
Patz1	62,46812712	0,360609768	0,200571175	1,797914218	0,072190603	0,329841479
Zfp687	333,1838954	-0,178181128	0,177897324	-1,00159532	0,316539082	0,674637563
Cnep1r1	374,9718856	0,129293363	0,116315967	1,111570204	0,26632299	0,626512766
Lrrc15	0,631720726	-0,582237906	2,365555144	-0,246131614	0,805580351	NA
Plb1	2,171990567	-1,091071073	1,190462818	-0,916509996	0,359399467	NA
Lrrc71	4,668737557	-0,093983816	0,831236642	-0,113065055	0,90997898	NA
Rnase13	10,13232192	-0,849369805	0,484683835	-1,752420328	0,079701559	0,349577792
Nfu1	803,5558178	0,036740853	0,083745114	0,438722342	0,660862737	0,882666274
Camk2a	1,104466178	1,275063922	1,83291761	0,695647156	0,486649827	NA
Oaz3	1,361918211	-1,375768991	1,305835405	-1,053554671	0,292086852	NA
Trim69	2,524467364	-0,964954125	0,917993604	-1,051155609	0,293187127	NA
Ankrd6	6,937000184	0,373017852	0,587252789	0,635191282	0,525303668	0,816792829
Smc1b	9,551621989	0,773665998	0,544056927	1,422031333	0,155017155	0,489743509
Eri3	574,0974438	0,003119709	0,080937644	0,038544598	0,969253474	0,991724161
Tex40	0,213661134	0,059593471	3,352475198	0,017775962	0,985817581	NA
H2-Eb2	2,802631696	1,131090079	1,258651567	0,898652263	0,368837912	NA
Col22a1	0,44842569	-1,521507338	2,415445747	-0,629907477	0,52875512	NA
Ypel5	981,0235239	0,04582752	0,113765015	0,402826121	0,687076144	0,893359362
Lrrc27	13,76901597	-0,279552429	0,386104594	-0,724032901	0,469045537	0,783960411
Ms4a6d	42,73117776	0,43330776	0,316735849	1,368041415	0,171299113	0,511550248
Pmvk	3536,609003	0,611792912	0,196737178	3,109696494	0,001872797	0,036658008
Mif4gd	1168,82478	0,075389277	0,069307156	1,087756022	0,276702829	0,637663478
Tmem52	0,42317886	0,005915703	2,557141943	0,002313404	0,998154172	NA
Plxnb1	1285,982899	-0,057779664	0,165840797	-0,348404407	0,727536494	0,910785759
1110008L16Rik	322,3157825	0,151839301	0,106219172	1,429490536	0,152863293	0,487274527
Rnf113a2	189,50913	-0,162103715	0,181296183	-0,894137496	0,37124833	0,719112906
St7l	544,5913518	-0,086452272	0,128442318	-0,673082467	0,500894827	0,803541005
Sulf1	24,10391092	1,303577248	0,479222707	2,720190901	0,006524424	0,080838759
Zfp976	98,06634311	-0,305200551	0,157706763	-1,935240727	0,052960762	0,277986819
Ypel4	0,883524818	0,719415062	1,566158111	0,459350213	0,645982694	NA
Tcirg1	1126,512414	0,000450253	0,131711481	0,003418482	0,997272451	0,999481197
Tfe3	700,8292663	-0,212043629	0,109128555	-1,94306273	0,052008579	0,274994558
Clip4	9,648665785	0,312116134	0,595792888	0,523866834	0,600371164	0,854422974
Kbtbd12	13,71987533	0,218848034	0,416939707	0,524891322	0,599658742	0,85430922
Slc35c2	544,0750208	-0,031603208	0,113893006	-0,277481551	0,78141037	0,932196631
Ralbp1	669,3301211	-0,197086876	0,081062953	-2,431281721	0,015045511	0,134095623
Dcaf11	7288,922291	-0,041219146	0,075413639	-0,546574158	0,584671327	0,847160046
Slc25a12	409,7844171	0,059691168	0,108889261	0,548182324	0,583566718	0,846998529
P2ry10	4,816288151	0,32685289	0,770774815	0,424057564	0,671523833	NA
Slc38a5	1,036992931	-1,498448371	1,592328565	-0,941042197	0,346683235	NA
Smndc1	373,4782173	0,263793292	0,117932465	2,236816578	0,025298331	0,181959388

Ccdc134	110,3569387	-0,448038169	0,155386398	-2,883380886	0,003934314	0,058572211
Zdhhc17	218,5494168	0,128796908	0,120283193	1,070780587	0,284268098	0,645517011
Fastkd2	367,032837	-0,01934507	0,096996574	-0,19944076	0,841917979	0,952074658
Asxl2	456,5149818	-0,126819263	0,134326797	-0,944109927	0,345113477	0,698150529
0610010K14Rik	421,1822915	0,185431521	0,096758122	1,916443993	0,055308606	0,284475895
Gal3st1	33,55842751	0,900188105	0,301072632	2,989936681	0,002790353	0,0470419
Rapgef3	64,01498217	-0,10631269	0,301510694	-0,352600066	0,724388288	0,910252319
Rapgef4	2524,967445	-0,144249784	0,130028911	-1,109367009	0,267271889	0,627508918
Dlg3	636,9416098	0,113735765	0,103621498	1,097607801	0,272375785	0,634499367
Nrp	3,152644075	1,553927989	1,179660311	1,317267331	0,187749032	NA
Kcnip2	0,648496631	-1,948772858	2,294309939	-0,849393896	0,395662149	NA
Setmar	80,5635983	0,458443728	0,193891315	2,364436638	0,018057516	0,149019093
Ndufv3	2401,661144	-0,001920159	0,128234905	-0,014973759	0,988053116	0,996575327
Strn4	760,3779959	-0,075879619	0,075084999	-1,010582944	0,312216082	0,671711748
Plin1	1,457249152	3,143828478	2,462047215	1,276916404	0,201631762	NA
Rian	11,22930063	-0,189772035	0,465825815	-0,407388403	0,683722743	0,892293506
Afg3l1	1541,819543	-0,128748981	0,070469461	-1,827018088	0,06769705	0,31878896
Paqr5	0,520080961	0,46559698	2,141715557	0,217394405	0,827900991	NA
Hdac7	157,2767582	0,14479597	0,198719556	0,728644795	0,46621897	0,781944725
Rxrb	665,5044829	-0,143657865	0,090310614	-1,590708544	0,111675183	0,413687165
Cd68	327,560268	0,107183253	0,202992356	0,5280162	0,597488092	0,853549036
Ap5s1	503,0017777	0,097168191	0,137079629	0,708844868	0,478420751	0,790899842
Kcnip3	10,94737254	0,048638856	0,463689309	0,104895357	0,916458843	0,976559041
Iqsec2	211,6684807	-0,117164224	0,146058455	-0,80217351	0,422452597	0,755522295
Dmd	507,6854883	0,319237987	0,17393063	1,835432819	0,066441585	0,315993435
Mrpl44	600,4296986	-0,016356725	0,101080081	-0,16181947	0,87144802	0,962126401
Acsl3	1367,390377	0,11039164	0,192435628	0,573654893	0,566201358	0,838041311
Hsbp1l1	54,78018937	0,302963681	0,276768847	1,094645167	0,273672119	0,635293489
C2cd4d	18,87187238	-0,359695396	0,43581692	-0,825336005	0,409180856	0,746907036
Tiam1	24,11998555	-0,541594732	0,385712948	-1,404139361	0,160277355	0,496811489
Poli	141,9637111	-0,24913001	0,161176012	-1,545701546	0,122176664	0,434047185
Capza1	334,1589523	-0,046259263	0,113527026	-0,407473569	0,683660203	0,892293506
Wnt7a	0,567080488	2,603253552	2,281559452	1,140997465	0,253870978	NA
Wnt5a	32,72905126	-0,202045152	0,442743702	-0,456347885	0,648139835	0,876446263
Wnt4	67,71042531	-0,240708566	0,255675889	-0,941459777	0,346469293	0,698497639
Wnt2b	3,086804424	1,732487024	0,977585872	1,772209557	0,076359778	NA
Wnt11	0,686883455	0,271343097	1,767937631	0,153480017	0,878019748	NA
Wnt10a	0,45481271	-0,619323583	2,983187513	-0,207604644	0,835537678	NA
Mbd2	1219,410878	-0,104739368	0,073628824	-1,422532134	0,15487183	0,489743509
Trappc5	644,9394971	0,014495861	0,117974264	0,122873079	0,902207604	0,973248278
Dimt1	73,85541049	0,061979749	0,183746281	0,337311581	0,73588203	0,914312906
Snx31	0,695470465	-1,175235017	1,972964474	-0,59566963	0,551395951	NA
Uba5	895,0301699	-0,477146199	0,110162134	-4,3313086	1,48E-05	0,00113236
Ppm1m	374,9939742	-0,468223711	0,187351627	-2,49917077	0,012448431	0,120146001
Alg5	919,0578354	0,124436693	0,070354749	1,768703523	0,076943364	0,342991041
Nemf	649,8455603	-0,342662782	0,12672401	-2,704008349	0,006850857	0,083591067
Mier1	579,3357644	0,062650198	0,146165636	0,428624672	0,668196388	0,886397847
Ccdc57	79,07251585	-0,017600656	0,227752631	-0,077279704	0,938401037	0,982879468
Noxred1	8,874245973	-0,496231974	0,546949042	-0,907272773	0,364262568	0,713048566
Xrcc3	83,10514745	0,016646913	0,193570131	0,085999389	0,931466903	0,980584977
Poc1b	147,7783753	-0,228204926	0,141422745	-1,613636658	0,106606261	0,405237162
B4galnt1	2689,028874	-0,119001625	0,088193485	-1,349324431	0,177232782	0,520079413
Spata24	31,41817777	0,819790445	0,270105046	3,035080076	0,002404718	0,042750903
Prkg1	18,85066911	0,076831201	0,371413941	0,206861381	0,836118117	0,951141594
Cdk18	986,6386719	-0,284910888	0,143254027	-1,988850813	0,046717672	0,257129914
Ppp1cc	601,9948743	0,328892889	0,095027746	3,461019564	0,000538134	0,015405374
Sfrp1	10,39861637	1,181682138	0,470006332	2,514183444	0,011930836	0,116510842
Rax	0,086476712	0,780932884	3,352475198	0,232942181	0,815806304	NA
Znrf1	843,6351743	-0,082938568	0,13274888	-0,624777915	0,532116828	0,820420928
Kcnip4	0,142634598	-0,517475177	3,352475198	-0,154356154	0,877328924	NA
Triqk	2,078055802	0,383442766	1,154676776	0,33207801	0,739830354	NA
Bag1	1979,040404	-0,055130892	0,099744195	-0,552722817	0,580453227	0,845508737
Camk2b	111,2062458	-0,018023439	0,427604643	-0,042149775	0,966379301	0,990533227
Prkag3	5,536036812	2,858489674	1,357403503	2,10585111	0,035217288	0,219189906
Dnmt3a	346,8207415	0,05059671	0,117470676	0,430717789	0,666673582	0,885344107
Mlf2	3979,48485	-0,146575373	0,080037599	-1,831331452	0,067051086	0,317311846

Txnrd3	205,3372906	0,134760217	0,175168511	0,769317595	0,441704795	0,768930234
Mär-02	1106,26721	0,007481414	0,089247524	0,083827689	0,933193433	0,981308229
Xbp1	5545,19381	-0,357828742	0,176573629	-2,026512934	0,042712249	0,243203202
Runx2	3,777614487	0,536107823	0,743728515	0,720838064	0,471009153	NA
Cyp4a31	146,9383373	0,015803171	0,261575352	0,060415368	0,951824819	0,986047734
Ncor2	989,60451	-0,445179639	0,144440843	-3,08208974	0,002055529	0,03881407
4933408J17Rik	0,87797138	0,741042888	1,5685826	0,472428349	0,636621073	NA
1810034E14Rik	207,1081852	-1,194886941	0,577899662	-2,067637376	0,038674135	0,230715978
4931431C16Rik	0,523498785	2,446616326	2,377939611	1,028880765	0,303535708	NA
2010315B03Rik	407,4060434	-0,089749454	0,101099211	-0,887736446	0,374682537	0,720996253
Cdh23	0,449274932	2,274020357	2,120299593	1,072499549	0,283495717	NA
Nol8	300,3602678	-0,099758916	0,196699075	-0,507165151	0,612038945	0,858888222
Slc6a4	6,135331519	0,26935062	0,576471471	0,467240156	0,640328066	0,871049783
Serpina1b	43560,11467	-5,433134138	0,216039364	-25,14881566	1,46E-139	9,73E-136
Ufd1l	826,0929613	-0,110588007	0,081997186	-1,348680512	0,177439608	0,520144503
Uck1	1102,507619	0,207527787	0,224039877	0,92629843	0,354290911	0,704551502
Ugt8a	3,198245888	0,444622225	0,861578522	0,516055372	0,605815736	NA
Ugcg	393,1542762	0,003627257	0,099296246	0,036529647	0,970860039	0,992007633
Fto	937,5449363	-0,009621187	0,091456339	-0,105199781	0,916217284	0,976559041
Zim1	1,303353592	-1,111650753	1,262200787	-0,88072418	0,378467126	NA
Ikzf2	2,50555729	-1,118965092	0,917572705	-1,219483846	0,222660603	NA
Zfx	274,9143862	0,190189284	0,107887039	1,762855727	0,077924826	0,345649679
Map3k3	279,9206843	-0,090865502	0,133942615	-0,678391279	0,497523637	0,801288247
Nubp2	757,7934957	-0,299971414	0,129113263	-2,323319913	0,020161973	0,158742883
Zfp97	56,46304118	0,269417927	0,221926753	1,213994812	0,224749705	0,579829951
Zfp90	50,24190764	0,096436535	0,253347792	0,38064881	0,703463858	0,900719679
Zfp9	45,98908593	-1,057635749	0,379463089	-2,787190057	0,005316729	0,070973656
Zfp41	57,86680405	-0,270837548	0,283421391	-0,955600236	0,339274242	0,694071514
Zfp54	87,03828977	0,008731906	0,185348655	0,047110705	0,962424995	0,988526411
Zscan21	414,1973516	0,192827085	0,113219027	1,70313322	0,08854314	0,368994153
Zfp36	1906,989207	-0,010212779	0,207349601	-0,049253914	0,960716946	0,988470616
Zfp35	274,4120899	0,099687104	0,136419032	0,730741908	0,464936813	0,781663619
Methig1	1,01982824	0,982819932	1,658809578	0,592485084	0,553525806	NA
Bcat1	0,213116452	1,337107854	3,350237755	0,399108348	0,689813372	NA
Slc30a4	288,0474533	-0,190314042	0,138085191	-1,37823644	0,1681303	0,507127649
Bpnt1	1815,53932	-0,198668834	0,095408683	-2,082293005	0,037315714	0,226546639
Ikzf3	13,04469887	0,865501511	0,392980398	2,202403774	0,0276368	0,191563289
Hist1h3a	0,435106969	-2,349954371	3,269276053	-0,718799616	0,472264395	NA
Hebp1	4818,732604	0,280291903	0,095956426	2,921033173	0,003488727	0,054936147
Emg1	960,3725025	0,135876066	0,0973168	1,396224149	0,162647007	0,499638749
Zfp260	647,174722	-0,217197849	0,107397416	-2,022374992	0,043137627	0,2449902
Cadm3	138,255893	-0,283153102	0,206446723	-1,37155532	0,170201911	0,510344993
Plscr1	332,3836455	0,269044167	0,146928039	1,831128829	0,067081316	0,317342576
Scnn1b	0,489630809	-0,74100367	2,454718941	-0,301869048	0,762751892	NA
Tcea1	857,1364039	-0,02102571	0,129097482	-0,162866927	0,870623212	0,961775826
Tcap	0,093303375	-0,517475177	3,352475198	-0,154356154	0,877328924	NA
Tbxas1	60,96502575	0,299428621	0,254405317	1,176974694	0,239205607	0,596519262
Tbx3	589,5843844	1,015173032	0,189791609	5,348882599	8,85E-08	2,10E-05
Tbx1	0,95379981	2,517280363	2,124188135	1,185055279	0,235995591	NA
Szt2	517,8745167	-0,355765668	0,152305109	-2,335874808	0,019497767	0,155528431
Fam105a	57,61892257	0,452865257	0,284969641	1,589170185	0,112021976	0,414165319
Cd300lb	26,92114452	0,575279692	0,343244568	1,676005233	0,093737168	0,380194927
Ippk	210,3740568	-0,311065327	0,15893748	-1,957155269	0,050329216	0,269788007
Scarna13	1,448252995	1,560903013	1,317150756	1,185060256	0,235993624	NA
Mir3073a	0,49288444	-1,484825691	2,430043457	-0,611028452	0,541180745	NA
Fam131b	0,113662148	0,780932884	3,352475198	0,232942181	0,815806304	NA
Gria1	0,549959488	2,568387935	2,329356324	1,102617023	0,270193515	NA
Atp11a	300,7339152	0,88605245	0,278479587	3,181750085	0,001463881	0,030917254
Thsd1	80,46521387	0,287053569	0,203543974	1,410277905	0,15845764	0,494380441
Gpcpd1	830,7336828	0,684814737	0,208945077	3,27748682	0,001047356	0,024651593
Rhoc	277,3812386	-0,48737268	0,225778521	-2,158631733	0,030878749	0,203860738
Rhob	357,4354788	0,124344821	0,176132616	0,705972715	0,480205106	0,79208634
Ovol3	16,71861433	-0,306075064	0,416380097	-0,735085722	0,462287289	0,781516914
Sass6	26,56010571	0,301186063	0,300817547	1,001225048	0,31671802	0,674637563
St3gal3	1982,842519	-0,207508193	0,082144059	-2,526149741	0,011532032	0,115044027
Klrb1	0,293986917	-0,62664923	2,757017991	-0,227292398	0,820196384	NA

Smim24	16,6087831	0,277282785	0,355043348	0,780982902	0,434812551	0,764552565
Serac1	137,7218855	-0,215258747	0,155971767	-1,380113539	0,167551689	0,506611452
Nav2	947,3623318	-0,139697099	0,180098504	-0,775670514	0,437943554	0,766215379
9030624J02Rik	670,6027799	0,058366942	0,085795417	0,680303726	0,496312166	0,800192636
Taf3	140,9843663	0,025229308	0,164965093	0,152937251	0,87844776	0,965046146
Fam110c	14,03772884	0,782128746	0,55194301	1,417046201	0,15646942	0,491966057
Afmid	5147,885828	0,00615058	0,118216192	0,052028237	0,958506194	0,987896001
Arhgap42	1447,423419	-0,170441217	0,126293505	-1,349564385	0,177155755	0,520079413
Nipbl	911,3394321	-0,034193108	0,092087057	-0,371312859	0,710404522	0,904169655
Mob2	441,8967019	0,08507161	0,098285863	0,865552868	0,386735412	0,73103259
Tmem219	602,7114134	0,031277725	0,137640359	0,227242392	0,820235266	0,946381744
Fkbp6	0,293986917	-0,62664923	2,757017991	-0,227292398	0,820196384	NA
Rpl37rt	62,80084508	0,074697752	0,234899529	0,317998729	0,750485901	0,920556312
Acsm2	59,96439274	-1,178625067	0,351506488	-3,353067743	0,000799211	0,020415847
Aloxe3	0,96321099	3,346964051	1,811358004	1,847765071	0,064636336	NA
Cd3d	9,328513391	0,462659184	0,574468997	0,805368412	0,420607114	0,75436738
Cd2	8,908950356	0,625449476	0,499352047	1,252522101	0,21037968	0,562962817
C9	16536,07265	-0,649922686	0,193021714	-3,36709623	0,000759642	0,019758075
Atxn1	407,9993751	-0,245722937	0,232494227	-1,056899088	0,290557645	0,652193612
Mir320	0,235937973	-1,303449117	3,335546383	-0,390775294	0,695963339	NA
Mir1967	0,172953423	1,307385949	3,350719078	0,39018071	0,696402925	NA
Serping1	23979,27905	0,007329632	0,068234115	0,107418876	0,914456681	0,976392941
Mbl1	4932,852105	-0,170742029	0,127101555	-1,343351219	0,179158274	0,522135977
Ctla4	0,894844136	-1,171283039	1,714183976	-0,683288991	0,494424249	NA
Mir30a	0,086476712	0,780932884	3,352475198	0,232942181	0,815806304	NA
Mir1894	4,056534402	0,109739042	0,698824903	0,157033675	0,875218309	NA
Mir1898	1,508558769	-0,629302722	1,343887608	-0,468270351	0,639591268	NA
Mir1896	0,543637052	1,324206804	2,407177065	0,550107769	0,582245459	NA
Mirlet7i	0,087021394	-0,517475177	3,352475198	-0,154356154	0,877328924	NA
Mir365-2	1,159164565	0,751268753	1,410240388	0,532723896	0,594224722	NA
Ckap4	59,55464109	-0,311523643	0,308699167	-1,009149606	0,312902891	0,672219008
Mbl2	9791,970762	0,165682486	0,132656567	1,248958043	0,21168042	0,564073259
Mir1192	0,208973043	1,337107854	3,350237755	0,399108348	0,689813372	NA
Mir1191	0,285269197	-1,521344816	3,321239936	-0,458065315	0,646905517	NA
Pdpr	184,7218407	-0,454407681	0,264894289	-1,715430267	0,08626637	0,364119818
Tmem263	152,6009035	-0,291300718	0,150242378	-1,938871857	0,052516944	0,276416937
B930059L03Rik	7,389615523	-0,377897064	0,563768527	-0,670305358	0,502663152	0,80422495
Kmt5b	548,9867298	0,227420489	0,128430386	1,770768554	0,076599197	0,342478335
Phyhipl	0,20013886	1,337107854	3,350237755	0,399108348	0,689813372	NA
Ppp1r16b	41,30965511	-0,197986946	0,280137762	-0,706748512	0,479722776	0,791837124
Slc44a1	705,9913465	0,001370877	0,129431743	0,010591508	0,991549357	0,997293361
Ctbp1	946,21788	0,024765042	0,086827311	0,285221797	0,775474222	0,92961202
BC068281	127,5069682	-0,164269174	0,146116276	-1,124235972	0,260912933	0,621964694
Trim36	3,170351407	0,898393138	0,878912554	1,022164417	0,306703089	NA
Itch	1191,019733	-0,082881633	0,128482367	-0,645081776	0,518874193	0,813725618
Amfr	5648,194384	0,00602398	0,075991943	0,079271303	0,936816831	0,982210769
Slc35a1	481,7287018	-0,062540108	0,136746153	-0,45734455	0,647423413	0,876230372
Tlr2	51,82043842	0,442454433	0,234844136	1,88403441	0,059560321	0,295130442
Cltb	1553,625018	-0,128864916	0,091849642	-1,402998564	0,160617267	0,497288616
Trp53bp1	217,1523158	-0,026937262	0,144255408	-0,186733115	0,851869891	0,95595696
Asb14	0,086476712	0,780932884	3,352475198	0,232942181	0,815806304	NA
Apc2	8,616379308	0,118858279	0,467718497	0,254123537	0,79940012	0,93892144
Dis3l2	389,4027203	-0,151247889	0,11057003	-1,367892266	0,171345801	0,511550248
Slc44a2	197,525692	0,158983873	0,181336032	0,876736251	0,380629912	0,725866642
Lsm14b	513,6149759	-0,007179182	0,108377388	-0,066242431	0,947184815	0,985157551
Lrrc8a	552,9572692	0,064452389	0,110969834	0,580809997	0,561368517	0,836472635
Mcmdc2	2,862919904	-0,861243487	1,02563805	-0,839714836	0,401068294	NA
Mb21d2	451,712652	0,241984798	0,139967853	1,728859827	0,083834185	0,358305379
Zfp874a	493,9891989	-0,018418004	0,08949674	-0,205795253	0,836950849	0,951141594
Fhod1	180,8467269	-0,065944724	0,15807051	-0,417185494	0,676542734	0,889512888
Tusc5	7,097232682	0,480512795	0,620329625	0,774608814	0,438570848	0,766338212
Vwa3a	0,172953423	1,307385949	3,350719078	0,39018071	0,696402925	NA
Hist1h2bb	1,088719282	-2,216181646	1,467492598	-1,510182504	0,130996862	NA
Hist1h2ah	0,259430135	1,627812584	3,345388605	0,486584005	0,626553161	NA
Tmc3	3,2554274	1,301251943	1,082775993	1,201773914	0,229451135	NA
Ocrl	172,5649652	0,248085215	0,208040435	1,192485558	0,23307091	0,589913858

Mup12	715,7110302	-1,614092808	1,061062036	-1,521204936	0,128208417	0,445315232
Mtpn	2197,471797	-0,122541438	0,134083451	-0,913919187	0,360759311	0,710581841
Npm3	294,5509344	0,166621414	0,135853555	1,226478128	0,220018801	0,574490867
Rbm4	18,94165044	-0,383881125	0,333511907	-1,151026745	0,249721233	0,60896459
Mst1r	8,108789372	0,927035987	0,517789415	1,790372611	0,07339403	0,333666539
Irf2	1144,349252	0,109354607	0,159278404	0,686562674	0,492358357	0,798476599
Naga	751,7448598	0,137340595	0,085716597	1,60226375	0,109097298	0,40910239
Npc1	2451,540995	-0,015095986	0,19787742	-0,076289586	0,939188711	0,982918677
Fam83b	1,068081653	1,839770239	1,51423185	1,214985828	0,224371496	NA
Proca1	91,0515513	-0,550245485	0,344936238	-1,59520927	0,110665447	0,411311191
Mir7051	3,411070458	0,525252065	0,806682607	0,651126057	0,514965117	NA
Numa1	1355,776233	-0,151931408	0,099849825	-1,521599139	0,128109554	0,445315232
Cenpo	89,82632651	-0,042837097	0,170385216	-0,251413228	0,801494641	0,939799232
Smek2	902,5684892	0,093070074	0,142108568	0,654922331	0,512517762	0,81029446
Angel1	110,2256791	0,189953226	0,172870281	1,098819446	0,271846828	0,633708849
Zfp622	476,4244193	0,154118976	0,123523802	1,247686471	0,212145898	0,565088365
BC024978	239,5937624	-0,640685042	0,166678395	-3,843839764	0,000121124	0,005206779
Pzp	133372,0397	-0,356317199	0,134809876	-2,643109025	0,008214858	0,093150496
Egr3	2,429743777	0,081469249	1,135724295	0,071733298	0,942814157	NA
Ctbp2	35,6731575	-0,060379918	0,270597683	-0,223135384	0,82343014	0,947466482
Sbf1	900,3100408	-0,305402223	0,074006439	-4,126697965	3,68E-05	0,002129833
Fgfrl1	554,7726492	0,152042811	0,116965148	1,299898415	0,193635788	0,542139655
Xrra1	3,442577108	1,446891991	0,802608133	1,802737765	0,071429408	NA
Mir7005	0,093303375	-0,517475177	3,352475198	-0,154356154	0,877328924	NA
Zc4h2	49,76511759	0,115317346	0,24413107	0,472358335	0,636671039	0,86976752
Kcnh4	0,113662148	0,780932884	3,352475198	0,232942181	0,815806304	NA
Bcap29	221,2245438	0,410422862	0,150335977	2,730037543	0,006332711	0,079420279
Nip7	265,1064525	0,240339728	0,210803725	1,140111392	0,254239897	0,614302038
Tsen34	565,3911526	-0,049639817	0,094835201	-0,523432407	0,600673377	0,854422974
Dyx1c1	9,960778613	0,4157471	0,540472666	0,769228726	0,44175754	0,768930234
Telo2	135,1385249	0,181253037	0,17063873	1,062203387	0,288143362	0,649500693
Rasl12	2,285393549	1,250032165	1,280977858	0,975842133	0,329142702	NA
Ccdc69	34,31147706	0,624456988	0,417203498	1,496768341	0,134453547	0,456327747
Dcdc2a	93,26259683	0,809145995	0,258526889	3,129833032	0,001749057	0,034900211
Rab11fip5	32,12864292	0,397397633	0,36216088	1,097295855	0,272512082	0,634510789
Umodl1	0,520185999	1,487180202	2,384076407	0,623797206	0,532760775	NA
R3hcc1l	362,4702759	0,122618398	0,136531622	0,89809523	0,369134782	0,717291119
Zmym6	231,3731022	-0,104420825	0,155522771	-0,67141824	0,501954129	0,803956481
Vit	0,51448455	1,494857447	2,428757534	0,615482372	0,538236207	NA
Shisa8	0,601056464	-0,084933002	1,708784556	-0,049703751	0,960358467	NA
Anapc2	953,8219025	0,038867597	0,104011667	0,373684977	0,708638704	0,903161152
Srd5a1	3260,99586	-0,784326273	0,206609271	-3,796181411	0,000146942	0,005971026
Dync1h1	2194,516632	-0,09217778	0,081351253	-1,133083712	0,257179101	0,617941661
Bcdin3d	70,19466399	0,030272379	0,183129948	0,165305453	0,868703567	0,961775826
Fbxo34	420,5479856	0,245619539	0,110880912	2,215165215	0,026748726	0,18930315
Kcp	194,0977157	-0,675480734	0,218192971	-3,095795119	0,001962859	0,037976073
Serpinb8	69,65483515	0,698611077	0,32234888	2,167251447	0,030215686	0,20087196
Marcksl1-ps4	290,3195541	0,11484279	0,15128136	0,759133776	0,447772536	0,772918303
Sgce	170,3862262	-0,017375984	0,198300501	-0,087624511	0,930175121	0,980359737
1700066M21Rik	653,2697192	-0,573381091	0,209019255	-2,74319747	0,006084408	0,07769098
Aldh3b3	7,208267937	3,811583343	1,368619295	2,784984369	0,00535303	0,070973656
Krt80	4,326881279	-0,809679073	0,794630438	-1,018937905	0,308232447	NA
Zfp763	58,26781661	0,100077963	0,214237615	0,467135348	0,640403045	0,871049783
Spef2	2,737322104	0,586021875	0,991163597	0,591246366	0,55435536	NA
Zfp974	57,60012788	-0,146605509	0,296680564	-0,494152723	0,621198326	0,862654966
Tjap1	176,0577743	-0,048104084	0,157456246	-0,305507629	0,759979561	0,924925298
Smap1	718,6805415	-0,058461414	0,084526264	-0,691636083	0,489165896	0,796547973
Tepp	0,406549865	-1,138993395	3,029939865	-0,375912872	0,706981671	NA
Nuak2	265,7710689	-0,239457152	0,147366115	-1,624913242	0,104181046	0,401613728
Ccdc30	92,87654656	-0,387402146	0,2293522	-1,689114583	0,091197474	0,374798348
Col20a1	68,45192194	0,086842688	0,35893274	0,241946967	0,808821257	0,94241441
Lrrc69	0,781181988	-1,63543757	1,945035962	-0,840826392	0,4004452	NA
Clhc1	0,788113689	-1,650283994	1,944110583	-0,848863232	0,3959574	NA
Rhobtb3	126,0817871	0,15014594	0,169938296	0,883532102	0,376948846	0,722809684
Nrbp2	1312,410351	0,074822948	0,159530366	0,469020097	0,639055271	0,871049783
Gm10336	117,2557902	0,011203008	0,160468155	0,069814525	0,944341286	0,984069637

Tspan14	760,8409008	0,044667866	0,131805386	0,338892573	0,73469066	0,913766809
Ccdc112	5,680641767	0,424081052	0,606881203	0,698787587	0,484684787	0,79420977
Guk1	856,1355272	-0,260778529	0,110796411	-2,353673072	0,018588952	0,151643025
Rin3	623,4135457	-0,213948544	0,130684881	-1,637133092	0,101602663	0,396591535
Pdcd6ip	2794,361217	-0,037658143	0,073728361	-0,510768753	0,609512992	0,857753956
Grk6	425,1490605	-0,111258399	0,087131969	-1,276895264	0,201639226	0,553080595
Txk	5,035150235	-0,371125657	0,846228253	-0,438564484	0,660977136	NA
Fhl2	5,343338475	-0,149951366	0,660860069	-0,226903353	0,820498895	NA
Erbin	1512,820302	-0,11003237	0,12484072	-0,881382054	0,378111067	0,723903192
Lyrm1	67,01487136	0,153438938	0,187000077	0,820528744	0,41191475	0,749099865
Eid3	1,795851071	-1,504126241	1,284177628	-1,171275848	0,241487916	NA
Chmp1b	662,2625755	0,05752389	0,116076743	0,495567745	0,620199414	0,862315769
Cyp39a1	1023,037898	0,327993137	0,21270128	1,542036495	0,123064725	0,435597647
G0s2	1418,990163	0,368569878	0,462789748	0,79640891	0,425794401	0,758357009
Ercc1	103,8274631	0,079756136	0,157923413	0,505030475	0,613537431	0,859428114
Fasn	21900,97046	0,547248387	0,252023761	2,171415845	0,029899752	0,199665228
Hes1	200,6761207	0,260503148	0,475048583	0,548371594	0,583436777	0,846950064
Mybpc3	1,46802969	1,225978739	1,821268854	0,673145393	0,500854798	NA
Nipsnap1	6759,487084	0,158663276	0,06862352	2,312083035	0,020773108	0,160901321
Msh2	227,0712716	0,168003484	0,141660557	1,185958091	0,23563885	0,593010215
Tmbim6	29961,46134	0,101258728	0,083911871	1,206727095	0,227537261	0,582859866
Ddx52	419,9028541	0,038302724	0,112709776	0,339834972	0,73398081	0,913675851
Rita1	96,22999365	-0,111308112	0,176090708	-0,632106673	0,527317175	0,818305666
Efhd1	10,28414523	0,051674091	0,461976745	0,111854312	0,910938923	0,974815504
Hist3h2a	58,13603043	0,045807872	0,225049526	0,203545741	0,838708498	0,951141594
Hist2h3b	0,643042356	-0,27115136	2,027178875	-0,133757984	0,893593952	NA
4931428F04Rik	49,93617661	-0,398491625	0,268086373	-1,486429988	0,137165423	0,459935926
Smchd1	279,1779839	-0,062444949	0,143625016	-0,434777665	0,663723819	0,883893982
Lrguk	0,213116452	1,337107854	3,350237755	0,399108348	0,689813372	NA
Tldc1	216,9553205	-0,058184091	0,173560499	-0,335238093	0,737445489	0,915064854
Sema3d	40,31979916	-0,116028493	0,296104049	-0,39185041	0,695168746	0,8969032
Nabp1	369,8550376	0,105894203	0,206966765	0,511648346	0,608897143	0,857753956
Hist1h4c	4,360381638	0,596808787	0,885901938	0,673673644	0,500518821	NA
Hist1h2bj	0,688727578	-0,477035944	1,757088193	-0,271492316	0,786012408	NA
Hist1h3i	0,605896868	-0,87568378	2,3533399	-0,372102551	0,709816497	NA
Hist1h2bh	1,529382582	0,494704593	1,201393978	0,411775489	0,680503989	NA
Hist1h2bg	3,280463339	-0,070146644	1,027351237	-0,068279126	0,945563441	NA
Dcaf5	996,4762022	-0,097405953	0,113624385	-0,857262753	0,391299688	0,734518115
Hist1h3e	1,178492399	-0,365599276	1,309408812	-0,279209421	0,780084114	NA
Hist1h2be	12,43602016	-0,000674813	0,411695371	-0,001639108	0,998692181	0,999803624
Cdk13	710,3390169	0,045729004	0,108478409	0,421549364	0,673353973	0,888626355
Trmt61a	101,6699354	-0,034792081	0,288298065	-0,120680937	0,903943758	0,973561919
E130308A19Rik	81,27745915	-0,046817393	0,186961905	-0,250411402	0,802269213	0,939799232
Taf9	440,8950581	0,131869759	0,105211702	1,253375398	0,210069121	0,562470273
Fkbp10	30,61246524	0,181990196	0,37150721	0,489869889	0,624225972	0,863900312
Musk	0,240301888	1,389394708	3,349408814	0,414817893	0,67827522	NA
Slc35f5	1074,859846	0,20666129	0,089792774	2,301535864	0,021361361	0,163367723
Cox18	223,0554463	-0,20401213	0,165976149	-1,229165341	0,219009821	0,57359407
Itpkb	93,08809498	-0,141102688	0,347076797	-0,406546012	0,684341456	0,892349329
Tmprss9	4,638176517	-0,038177942	0,750937919	-0,050840344	0,959452742	NA
Sstr1	0,25327948	1,389394708	3,349408814	0,414817893	0,67827522	NA
Tob1	2532,917443	-0,029530099	0,200007693	-0,147644815	0,882623086	0,966166219
Hey1	11,44625719	-0,635304229	0,615117183	-1,032818212	0,301688979	0,661990308
Tpd52l1	165,092314	0,242475856	0,134082567	1,808407024	0,070543176	0,324865285
Fhl4	0,206965523	0,059593471	3,352475198	0,017775962	0,985817581	NA
Smpd2	599,7186945	0,071304767	0,110792094	0,643590752	0,519840849	0,813876985
Fhl3	13,48776929	-0,025857124	0,384249504	-0,067292538	0,946348817	0,984653942
Fhl1	141,0124327	0,245994287	0,209619004	1,173530466	0,240583147	0,59816966
Smarcc1	647,0042156	-0,220590859	0,115268833	-1,913707745	0,055657509	0,285308756
Fh1	4476,813012	0,048133779	0,164549185	0,292519096	0,769889762	0,927934035
Asb10	0,552720398	-1,795161334	2,033496011	-0,882795601	0,377346717	NA
Fgf12	10,05879971	0,174047549	0,479119519	0,363265411	0,716406629	0,907403148
Slc1a1	0,688162707	1,938775387	1,797112981	1,078827769	0,280664513	NA
Slc4a3	12,95760029	0,684732044	0,627824599	1,090642268	0,275430323	0,636882421
Slc17a1	328,6619418	0,21958584	0,14257874	1,54010226	0,123535429	0,436454849
Fgf11	16,92027079	-0,024826111	0,399818374	-0,062093472	0,950488395	0,985972877

Fgf1	2781,609099	-0,233673755	0,1731839	-1,349281059	0,177246707	0,520079413
Slc16a2	4128,450224	-0,08229374	0,126786552	-0,649073099	0,516291129	0,813339545
Pla2g6	1126,039979	-0,616403181	0,136545667	-4,514263942	6,35E-06	0,000566285
Lef1	4,788255787	-0,560869515	0,797157632	-0,70358671	0,48169019	NA
Ebf2	0,113662148	0,780932884	3,352475198	0,232942181	0,815806304	NA
Nek4	326,2701181	0,050448021	0,123301957	0,409142098	0,682435384	0,891903322
Fam92a	318,9183497	0,075436228	0,108013259	0,69839785	0,48492842	0,79420977
Tpm2	133,3476094	0,171006705	0,535373395	0,319415769	0,749411254	0,919838327
Gml2	0,086476712	0,780932884	3,352475198	0,232942181	0,815806304	NA
Hbegf	20,7669034	0,338572419	0,350030131	0,967266498	0,333410827	0,690102081
Tpi1	12142,86237	0,115859792	0,082810697	1,399092105	0,161785365	0,498455421
Xrcc1	390,1393466	-0,199906797	0,121361197	-1,647205208	0,099515874	0,392688228
Htt	554,3964884	-0,193493014	0,132904324	-1,455882004	0,145425241	0,474192695
Slc1a3	6,071437852	0,452669622	0,600516624	0,753800318	0,450969133	0,774175112
Tubgcp5	91,14872822	-0,148744445	0,177788108	-0,836638888	0,40279558	0,742671411
Prr11	7,027405547	-1,11207122	0,65566129	-1,696106263	0,089865758	0,371840085
Rab39	11,89971317	0,108178975	0,439103949	0,246363019	0,805401231	0,94096033
Plppr4	0,086476712	0,780932884	3,352475198	0,232942181	0,815806304	NA
Cep295	133,5721533	-0,044107062	0,168517071	-0,261736461	0,793524629	0,936746676
Samd9l	472,0261901	0,68738275	0,180482662	3,80858052	0,000139767	0,005767107
Rps17	4598,29548	0,039613831	0,132172208	0,299713772	0,764395493	0,925959974
Abcd4	272,7786612	0,301101816	0,162314375	1,855053287	0,063588617	0,306733002
Pard3b	106,139452	-0,123144245	0,304540308	-0,40436107	0,685947223	0,893161227
Ttc5	184,1365961	0,181898926	0,148404296	1,225698522	0,220312147	0,574490867
Mcc	636,7667119	-0,0958514	0,203028831	-0,472107333	0,636850179	0,86976752
Got1l1	0,392896538	1,035637931	2,562728481	0,404115355	0,686127894	NA
Immt	3008,018666	-0,002791082	0,088983317	-0,031366346	0,97497738	0,993185764
Vps33a	755,5684138	-0,181877487	0,087828956	-2,070814635	0,038376122	0,22986128
Ptprb	839,2782338	-0,303585911	0,265414477	-1,143818207	0,252699037	0,612043137
Irak4	267,0975192	-0,120848247	0,10521655	-1,148566907	0,250734609	0,610022019
Gpank1	180,1220478	0,258410157	0,156585486	1,650281674	0,098885339	0,392051629
Sucnr1	1173,001338	-0,223380558	0,189149609	-1,180972876	0,237613499	0,59478675
Tas1r1	1,641011015	-0,186868796	1,588365481	-0,117648487	0,906346185	NA
Parp6	334,956548	-0,238763689	0,104669025	-2,281130348	0,022540735	0,168540055
A430078I02Rik	0,579905834	-1,793734642	2,011159983	-0,891890579	0,372451585	NA
Ctnnbip1	332,7187894	-0,273864859	0,132065554	-2,073703933	0,038106816	0,229172298
Tbc1d7	254,1046909	0,063709255	0,164624986	0,386996263	0,698758974	0,898938178
Pgm1	119,5374403	0,117535084	0,179074328	0,656348038	0,511600216	0,809801478
Cpeb4	1277,817077	-0,309401599	0,225433495	-1,37247395	0,169915943	0,50990039
Chst1	3,573976008	-1,009380952	0,951611249	-1,060707251	0,288822964	NA
Jam2	149,5967314	-0,026878787	0,312263996	-0,086077125	0,931405108	0,980584977
Ap3m2	68,63335531	-0,18450467	0,181102365	-1,018786639	0,30830427	0,667160859
Avl9	438,717473	-0,099795198	0,197729878	-0,504704696	0,613766262	0,859658581
Wdr76	61,55564829	-0,306003594	0,203395087	-1,504478786	0,132458131	0,453014264
Saal1	98,95675442	0,121859392	0,166192117	0,73324412	0,46340955	0,781663619
Elp3	563,0363419	-0,135078473	0,115603759	-1,168460903	0,242620901	0,600901069
Polr3h	197,0329561	0,018796441	0,136391267	0,13781264	0,890388495	0,968877905
Tomm20l	0,086476712	0,780932884	3,352475198	0,232942181	0,815806304	NA
9130019O22Rik	41,01673479	0,343017351	0,226123715	1,516945494	0,129280446	0,446717571
Dlst	4910,269732	0,024383179	0,097206325	0,250839426	0,801938259	0,939799232
Fndc8	0,395579793	-0,099662659	2,594198898	-0,038417509	0,969354801	NA
Fasl	3,417643527	1,641058727	0,990835341	1,656237579	0,097673716	NA
Kcnmb3	0,147722973	0,780932884	3,352475198	0,232942181	0,815806304	NA
Kansl2	337,3038832	0,041876759	0,108859584	0,384686011	0,700470061	0,89948941
Sipa1l1	922,9429531	-0,180329066	0,121452849	-1,48476605	0,137605808	0,460640235
Asb2	78,73995225	0,535650776	0,354135876	1,51255722	0,130392168	0,448869806
Ktn1	417,575353	0,142999906	0,141315225	1,011921439	0,311575617	0,670999483
Rbm3	494,1896524	0,498208064	0,156995804	3,173384592	0,001506728	0,031474133
Uimc1	518,2318916	-0,044114997	0,095627007	-0,461323616	0,644566444	0,874269004
Neurl4	518,1231294	-0,191104943	0,097713264	-1,955772797	0,050491925	0,270223739
Serpina3a	0,343089167	0,663808922	2,658186063	0,24972252	0,802801942	NA
Rbm39	2089,488766	0,203619477	0,07452904	2,732082363	0,006293541	0,079300984
Gss	1276,985945	-0,56915895	0,203539827	-2,796302613	0,005169098	0,070015871
Scml2	0,829775404	-0,708878117	1,935909879	-0,366173098	0,714235915	NA
E2f1	90,94656789	-0,470406952	0,199257017	-2,360804942	0,018235319	0,14991666
Dpf2	560,8293939	0,045772069	0,082162941	0,557088979	0,577466637	0,84496439

Anapc15	255,9105287	-0,055807257	0,148518336	-0,375760049	0,707095291	0,902535511
Pfkp	80,01350528	0,496570643	0,276281522	1,797335699	0,072282342	0,330147807
Polr2a	1451,841642	-0,112231003	0,116148229	-0,966273909	0,333907138	0,690487939
Elf2	587,680323	0,158843791	0,102478642	1,550018498	0,121137076	0,433132274
Bfsp1	2,113725232	2,325491418	1,163744719	1,99828311	0,045685976	NA
Mapk7	75,19390105	-0,179747737	0,261306986	-0,687879569	0,491528623	0,797760449
Mlh3	297,7348732	-0,142732012	0,135939706	-1,049965583	0,293733937	0,654983618
Otud5	991,2709337	-0,082576744	0,087493028	-0,943809421	0,345267046	0,69817409
Wnk2	11,95340399	0,40381353	0,468742376	0,861482875	0,388972149	0,732830983
Gal3st2	0,44138895	-2,373938194	2,896039699	-0,819718803	0,412376429	NA
Nmral1	184,430065	-0,223482772	0,201474649	-1,109235194	0,267328734	0,627508918
Tbc1d9b	1536,472858	-0,055964705	0,109056899	-0,513169784	0,60783256	0,857753956
Cnot6	842,9848798	-0,087521401	0,136534884	-0,641018609	0,521510593	0,814920768
Tlr7	39,04794031	0,006182194	0,369400865	0,016735732	0,986647441	0,996105587
Usp37	143,5227548	0,02544115	0,169224709	0,150339455	0,880496807	0,96546599
Tmx2	1174,053109	-0,01045027	0,11428446	-0,09144087	0,927142288	0,978888597
Nckap1	2625,731585	0,014579703	0,088042695	0,1655981	0,868473243	0,961775826
Gm16063	6,876945273	1,3659443	0,781600346	1,747624994	0,080528984	0,350795696
Pde10a	10,48990176	0,425815467	0,593219035	0,717804793	0,472877658	0,787187325
Dgkq	515,4259292	0,064981224	0,136330316	0,476645442	0,633614619	0,867989994
Fam160a2	431,3299227	-0,200954265	0,097469079	-2,06172323	0,039234094	0,232686021
Naa60	2637,907691	-0,267141621	0,088648396	-3,013496375	0,002582561	0,044607573
Zfp451	224,2252151	-0,018153378	0,136522143	-0,132970209	0,894216941	0,970277273
Kcnip1	0,244992664	1,389394708	3,349408814	0,414817893	0,67827522	NA
1700020G17Rik	0,086476712	0,780932884	3,352475198	0,232942181	0,815806304	NA
Med8	1145,531861	-0,01111579	0,079786672	-0,139318881	0,88919817	0,968705223
Slu7	842,6645452	-0,088524383	0,07878432	-1,123629463	0,261170252	0,622164129
Zfp335	410,4279638	-0,150513601	0,130468922	-1,15363566	0,248649572	0,60747894
Kcnc3	192,040571	-0,545611783	0,191086014	-2,855320335	0,004299344	0,061605499
Tmem14a	126,8147468	0,023782889	0,201324828	0,118131924	0,905963129	0,974075992
Dpp10	0,093953095	-0,517475177	3,352475198	-0,154356154	0,877328924	NA
Ubl7	933,3438517	0,06053483	0,099502293	0,608376234	0,54293797	0,826618577
Clic3	16,2025623	0,592978019	0,485080114	1,222433164	0,221543864	0,576229557
Mcemp1	2,056041365	-0,34679515	1,06315414	-0,32619461	0,744277119	NA
Kmt2e	1028,928427	0,027418896	0,160139662	0,171218645	0,864051847	0,961272625
Espn	14,55175117	0,672590853	0,556736095	1,208096365	0,227010198	0,582290739
Brat1	263,7586458	-0,087029687	0,145684163	-0,597386054	0,550249663	0,829906108
Mir343	0,554066772	0,67418655	2,100753167	0,320926114	0,748266384	NA
Cct7	2392,848191	-0,183561218	0,113212329	-1,621388943	0,104934251	0,402692427
Cct5	3538,40049	-0,144960991	0,083743123	-1,731019653	0,083448259	0,357455872
Slc26a2	55,44509439	-0,543777214	0,318918752	-1,705065036	0,088182296	0,368178985
Dld	3307,532799	-0,019450451	0,095186039	-0,204341424	0,838086701	0,951141594
Angpt4	2,86541485	-1,333048274	0,939915128	-1,418264516	0,156113552	NA
Gsr	1675,858753	0,001869068	0,133850064	0,013963893	0,988858788	0,996575327
Trh	0,113662148	0,780932884	3,352475198	0,232942181	0,815806304	NA
Dynlt1b	6,755227175	0,541949348	0,687358121	0,788452674	0,430431971	0,761221675
Tcp10b	4,499205966	-0,129630663	0,666170687	-0,194590764	0,845713334	NA
Tectb	1,559991467	-0,675158144	1,340931973	-0,503499177	0,614613359	NA
Aebp1	54,54626267	0,283912488	0,341529485	0,831297153	0,405805783	0,745391028
Abhd17c	1350,002991	-0,002722213	0,106531767	-0,025553061	0,979613826	0,994664228
Pnpla3	296,627882	1,114414514	0,844349387	1,319849972	0,186885113	0,533290732
Slc19a2	3113,718657	0,063072464	0,133672948	0,471841649	0,63703982	0,869838153
Hkdc1	9,621898786	1,250559584	0,607435492	2,058752907	0,039517915	0,233561011
Rnpep	962,6356206	-0,034082864	0,130210266	-0,261752511	0,793512254	0,936746676
Ntng2	17,70467	-0,245632554	0,426447461	-0,575997224	0,564617058	0,837355044
Pkdcc	544,8150384	-0,084780751	0,167662794	-0,505662283	0,61309375	0,859251205
Diexf	189,6008398	0,057949481	0,153630635	0,377200039	0,706024951	0,902122257
Fam20b	501,1936486	-0,096042771	0,0885254	-1,084917678	0,277958118	0,638491765
Chtf8	971,2025946	-0,093789795	0,077691184	-1,207212844	0,227350185	0,582853433
Chtf18	7,349623688	-0,601210584	0,792747668	-0,758388335	0,448218539	0,77297141
Casd1	659,4614524	-0,193766942	0,117973081	-1,642467422	0,100493172	0,393294268
Ndufb8	2903,912396	0,139457635	0,096185756	1,449878241	0,147092476	0,477300804
Slc25a26	154,958401	0,169229231	0,130003496	1,301728308	0,193009279	0,541594346
9430083A17Rik	8,645939496	-0,100309427	0,651042713	-0,154075033	0,877550574	0,965038548
Tpcn1	1279,942933	-0,118273004	0,121324724	-0,974846677	0,32963632	0,6866113
Ifi208	39,80030202	-0,178123874	0,320227144	-0,556242271	0,578045245	0,845288649

Supplementary Table S1: *Serpina1* KO vs. wildtype all DEGs

Trappc3l	0,12663974	0,780932884	3,352475198	0,232942181	0,815806304	NA
1700065D16Rik	0,286615571	1,706362568	3,314438116	0,514827101	0,606673845	NA
Cdc7	6,319948839	-0,727068448	0,566162885	-1,284203658	0,199070705	0,54896375
Armc3	2,117423834	0,518977031	1,08117488	0,480012106	0,631218785	NA
Wasf3	0,333605263	0,663964464	2,676598865	0,248062746	0,804085859	NA
Angptl6	298,3477429	-0,11202942	0,164310896	-0,681813698	0,495356762	0,80006558
Mall	0,273083462	-0,658048199	3,340448562	-0,196993963	0,843832262	NA
Prc1	19,9388484	-1,469661343	0,573511128	-2,562568137	0,01039012	0,107428857
Prmt6	143,8621874	0,26001652	0,191827482	1,355470638	0,175267657	0,517595164
Prkd3	3620,335732	0,109844509	0,110530339	0,993795097	0,320322633	0,677612453
Abcg3	91,86094561	0,167683944	0,302795188	0,553786687	0,579724841	0,84530168
Metrnl	68,43258749	0,086809345	0,185891575	0,466989134	0,640507652	0,871103439
Prpsap2	287,3185209	0,073273408	0,098284405	0,745524256	0,455954853	0,77799081
Susd4	360,3908873	-0,234051315	0,505960338	-0,462588266	0,643659521	0,873795284
Pycr1	5,064372363	-1,447741729	0,677269158	-2,137616502	0,032547881	NA
Depdc7	1023,940979	-0,194878788	0,140486028	-1,387175587	0,165388228	0,503661781
Tmem63a	404,1270488	-0,045985779	0,122121156	-0,376558666	0,70650161	0,902122257
Chrna2	101,995492	-0,796278618	0,288991906	-2,755366505	0,005862642	0,075727212
Cdc27	560,400304	-0,186476797	0,107930884	-1,727742698	0,084034365	0,358827341
Slc25a38	468,6281176	-0,243358132	0,107389878	-2,266117972	0,023444161	0,173219033
Sgms1	225,1768086	0,006611556	0,176211214	0,037520631	0,970069891	0,991981655
Bloc1s4	189,1441582	-0,019669016	0,200964788	-0,097872947	0,922033182	0,977341679
Tor1aip1	1163,989996	0,106703313	0,097416999	1,095325397	0,273374105	0,634872238
Tmem143	518,1773827	-0,108628308	0,088198309	-1,231637083	0,218084682	0,572041886
Mtss1	2141,386287	-0,228291644	0,128608535	-1,775089372	0,075883132	0,340773122
Prdm15	92,70175121	-0,157495281	0,177470732	-0,887443688	0,374840071	0,720996253
Cyp4f15	3147,27613	0,161904845	0,097296755	1,6640313	0,096106174	0,385586553
Kdm4c	209,887604	-0,025693084	0,145057786	-0,177123092	0,859411704	0,95905468
Ttc30a2	2,297640477	-0,907501788	1,112061914	-0,816053294	0,414469656	NA
Slc16a3	6,846902862	0,178677193	0,706437361	0,252927156	0,800324504	0,939320605
Camk2d	145,3419782	0,299719845	0,145896534	2,054331491	0,039943619	0,235069225
Pi4kb	879,6387896	0,00918805	0,089063005	0,10316349	0,917833216	0,976907366
Cept1	1622,741507	0,126850113	0,120614802	1,051696068	0,292939016	0,654134241
Osbpl10	3,352144905	-0,111806292	0,754846156	-0,148117985	0,882249656	NA
Adam32	9,809781597	-0,55643936	0,558715973	-0,995925276	0,319286453	0,677223638
Rp2	240,9402521	-0,026389256	0,17975147	-0,146809679	0,883282245	0,966395158
Efna5	92,5900011	0,140461213	0,191342109	0,734084167	0,462897443	0,781516914
Cflar	1663,591181	-0,504049195	0,116165783	-4,339050486	1,43E-05	0,001112267
Dtna	5,702958821	0,891304215	0,60804984	1,46584072	0,142691685	0,470094907
Rcan2	158,5816454	0,642525574	0,391189129	1,642493431	0,100487786	0,393294268
Fndc3a	1710,823708	-0,532729627	0,126843114	-4,199909714	2,67E-05	0,001708043
Yipf6	660,0418936	-0,21298193	0,131178061	-1,623609381	0,104459202	0,401874841
Rps24	7731,2305	0,021346758	0,119500413	0,178633341	0,858225613	0,958691362
Acsl4	1754,434818	-0,028424088	0,210439152	-0,135070341	0,89255626	0,969104575
Rnf138	223,2476514	-0,040088814	0,156178525	-0,256685829	0,797421312	0,938325224
Brinp2	0,12663974	0,780932884	3,352475198	0,232942181	0,815806304	NA
Zfp81	42,15730534	-0,118548887	0,266192532	-0,445350161	0,656066723	0,879592859
Tenm3	374,611888	0,800667251	0,258991752	3,0914778	0,001991629	0,038104827
Mir6960	0,093953095	-0,517475177	3,352475198	-0,154356154	0,877328924	NA
Tuba8	176,8197861	0,505009703	0,60564168	0,833842385	0,404369807	0,744376512
Dock8	1213,886573	0,02855819	0,119694819	0,238591694	0,811422206	0,942886004
Col16a1	83,03860627	-0,059457637	0,264648782	-0,224666203	0,822238959	0,946779723
Ints8	512,7607491	-0,294534854	0,133840633	-2,200638531	0,027761624	0,191905453
Sec16b	2489,507993	-0,0188302	0,104444835	-0,180288476	0,856926102	0,958117567
Ccdc138	72,96059643	0,158635184	0,211061637	0,751605957	0,45228806	0,774915567
Ehmt2	1363,941314	0,001255811	0,089620381	0,014012559	0,988819961	0,996575327
Fut10	16,90508963	-0,040041649	0,422819455	-0,094701528	0,924551904	0,977951927
Cbll1	198,0373736	0,036461171	0,143637893	0,253840894	0,799618479	0,938963499
Unk	225,4324351	0,101551518	0,134395316	0,755617985	0,44987827	0,773622955
Mknk1	426,3741029	-0,240061254	0,107779603	-2,227334742	0,02592491	0,184749534
Lamb3	65,15480042	0,016456555	0,244916927	0,067192394	0,94642854	0,984653942
Stard6	2,393085075	0,570155252	0,964151622	0,591354346	0,554283023	NA
Papss1	504,0679255	-0,533414686	0,119276634	-4,472080312	7,75E-06	0,000663842
Mfng	21,93664408	0,427046726	0,395008557	1,081107531	0,279649284	0,640395902
Mme	776,0420612	-0,931282111	0,243001727	-3,832409441	0,000126894	0,005318025
Myo1b	5117,762671	-0,009115332	0,124153675	-0,073419752	0,9414721	0,983128178

Lilra5	114,3032877	0,318619914	0,221211706	1,440339298	0,149771428	0,481899934
Prmt9	282,366421	0,239930276	0,138904422	1,727304808	0,084112936	0,359037626
Palb2	22,8129461	-0,076122516	0,360494017	-0,211161664	0,832761124	0,950100347
Klhl42	250,5458742	0,148608206	0,136433035	1,089239168	0,276048431	0,637060499
Gtf2ird1	565,7384597	-0,446143142	0,117836959	-3,786105363	0,000153027	0,00614359
Ppp2r5c	1119,951187	-0,167253551	0,089260439	-1,873770209	0,060962104	0,298973723
Plcb3	675,0555259	-0,185497103	0,11259432	-1,647481891	0,099459035	0,392688228
Ace2	13,38045817	0,055544709	0,412670786	0,134598114	0,892929633	0,969431238
Spaca9	1,924751248	-1,17919629	1,071876461	-1,10012332	0,271278395	NA
Nkd1	135,2886424	0,198867262	0,214388946	0,927600352	0,353614915	0,704458903
Mettl18	78,04519186	0,246324249	0,207523344	1,186971278	0,23523895	0,592708165
Cdc16	495,4102486	0,123567355	0,099869976	1,237282313	0,215982292	0,569610871
Pck2	103,6531018	0,005965365	0,224662039	0,026552617	0,978816566	0,994593468
Cacna1d	27,2807547	-0,232236808	0,481523602	-0,482295795	0,629595834	0,866228449
Mss51	11,62261646	0,227115395	0,413509994	0,549237984	0,58284214	0,846891345
Hs2st1	346,4544604	-0,140299586	0,130390728	-1,07599358	0,281930128	0,643414796
Hcst	6,960298639	1,126712839	0,60402727	1,865334389	0,062134548	0,302614487
Hax1	1317,157896	-0,213987777	0,120116394	-1,781503499	0,074830234	0,338112818
Gpr34	1,918724388	0,076405539	1,228508852	0,062193723	0,950408561	NA
Pigq	914,8306375	-0,136839287	0,079511239	-1,721005596	0,085249808	0,362012428
Ggt5	48,47875066	-0,005660392	0,255293456	-0,022172099	0,982310674	0,995338444
Ecsit	1227,247479	-0,113412297	0,102225712	-1,109430241	0,267244623	0,627508918
G3bp2	1748,977975	0,048958197	0,11945653	0,409841106	0,68192251	0,89172987
Ms4a2	0,093953095	-0,517475177	3,352475198	-0,154356154	0,877328924	NA
Mapk14	1931,689832	-0,16866047	0,09890993	-1,705192489	0,08815853	0,368178985
Fxr2	988,9574298	-0,233768832	0,095678601	-2,44327183	0,01455477	0,131287083
Rad1	118,5409635	0,15701624	0,155686905	1,008538512	0,313196009	0,672219008
Mxd3	4,698941674	-1,070561118	0,795456041	-1,345845733	0,178352273	NA
Banf1	923,1569586	-0,004387816	0,127683641	-0,034364744	0,972586297	0,992650147
Gpr157	87,42071339	0,188150236	0,246298889	0,763910211	0,444920752	0,770752398
Sh3pxd2b	35,76056081	0,368467037	0,267908357	1,375347304	0,169023798	0,508491916
Tarm1	0,093303375	-0,517475177	3,352475198	-0,154356154	0,877328924	NA
Sema3a	3,996231324	0,148855858	0,849110686	0,175307955	0,860837661	NA
Gm960	7,868233485	-0,867034936	0,519842694	-1,66787943	0,095339664	0,384609525
Lama5	81,83190297	0,299835683	0,306687674	0,97765808	0,328243461	0,685487182
Gpr75	0,501106203	1,428452334	2,432118318	0,587328472	0,556983123	NA
Uggt2	44,64467798	0,288167239	0,22878519	1,259553725	0,207830398	0,560178344
Bcr	994,158183	-0,245283921	0,087614461	-2,799582606	0,005116872	0,069899835
Bbs7	112,7434069	0,111698324	0,164975231	0,677061176	0,498367141	0,801990802
Ddx42	896,6952353	-0,029904975	0,080263786	-0,372583663	0,709458334	0,90343073
Sik3	1109,853228	-0,078870987	0,139339804	-0,566033432	0,571371054	0,841381253
Efhc1	3,513646088	-0,383647921	0,841431354	-0,455946786	0,648428245	NA
Cenpu	5,621144444	-0,823963428	0,730309375	-1,128238875	0,25921905	0,620723532
Sulf2	322,3463609	0,550027723	0,195077826	2,819529686	0,004809408	0,066976019
Faim2	0,541669986	1,563478027	2,878325361	0,543190165	0,586998878	NA
Ddx43	0,540439734	1,561989932	2,083596081	0,749660621	0,45345913	NA
Sorbs3	973,6577126	0,50369929	0,270689912	1,860798156	0,062772686	0,304171091
Ndor1	371,0517244	-0,134594009	0,126008216	-1,068136769	0,285458822	0,646939988
Slc16a10	1936,023307	-0,093556193	0,09691117	-0,965380909	0,33435406	0,690984608
Zfp655	754,2547048	0,046097258	0,172184043	0,26772085	0,7889142	0,935202374
Hspa12b	19,99080147	-0,679084397	0,361646008	-1,877759967	0,060414009	0,29803066
Ank3	616,6552377	0,454517094	0,157724653	2,881712445	0,003955205	0,058659427
Slc25a32	199,2672167	0,20447731	0,173620858	1,177723188	0,23890698	0,596330734
Twnk	805,5836749	-0,218920918	0,102211875	-2,14183449	0,032206803	0,208308055
Tmprss7	1,119226701	-0,05199301	1,363117267	-0,038142727	0,969573885	NA
Stxbp5l	8,192109924	0,121803747	0,869020768	0,140162067	0,888531942	0,96836734
4930525G20Rik	5,512727727	0,565183385	0,628093566	0,899839476	0,368205683	0,716735844
Grm8	32,12405422	-0,389553387	0,886655778	-0,439351321	0,660406993	0,882607568
Rfx3	51,89873458	0,15823552	0,218297428	0,724862044	0,468536668	0,78348591
Nploc4	1926,142221	-0,20422019	0,085399184	-2,391359952	0,016786087	0,143473504
Hmgcs2	57304,85473	-0,070472207	0,195557673	-0,360365338	0,718573944	0,90815359
Orm1	28300,89604	0,612168399	0,141877785	4,31475864	1,60E-05	0,001186702
Hmgb2	103,7024014	-0,10663435	0,184614859	-0,577604371	0,563531255	0,837355044
Knl1	4,904358119	-1,8293197	0,760829728	-2,404374636	0,016200162	NA
Lin37	231,2903517	-0,289748609	0,120782628	-2,398926193	0,016443229	0,141917059
Crlf2	166,3340615	-0,260552527	0,236103532	-1,103552006	0,26978752	0,630337183

Supplementary Table S1: *Serpina1* KO vs. wildtype all DEGs

Morn1	51,96502382	-0,454230939	0,208374318	-2,179879674	0,029266382	0,19731337
Gpn2	234,1989511	-0,037890745	0,133433729	-0,283966768	0,776435848	0,930009104
Ppp1r8	285,5040649	0,059076475	0,117274668	0,50374455	0,614440898	0,859778545
Cwc22	327,5403844	-0,019579376	0,174579012	-0,112151945	0,910702931	0,974815504
Thg1l	70,97536029	0,309425899	0,228594431	1,353602085	0,175863361	0,518666949
Gm4737	115,15913	0,090383312	0,252975008	0,357281586	0,720881014	0,909194176
Slc9a8	727,5933468	0,085603124	0,129653293	0,660246427	0,509095703	0,807845752
Hoxd8	0,113662148	0,780932884	3,352475198	0,232942181	0,815806304	NA
Senp7	123,384651	-0,130713312	0,171492185	-0,762211479	0,445933796	0,771440077
Mex3d	49,48240058	-0,019947537	0,313276625	-0,063673876	0,949229906	0,985650844
Taf5	101,3945699	0,23198686	0,158360306	1,46493061	0,142939851	0,470144068
Sytl5	105,851647	2,205464145	0,910682347	2,421771052	0,015445078	0,136293892
Arid5a	35,41935894	-0,137188993	0,304439299	-0,450628395	0,652257398	0,877662367
Xaf1	157,3394384	0,46971716	0,212867824	2,206614189	0,027341025	0,190773571
Sgk2	1567,369923	-0,10132827	0,138345625	-0,732428439	0,463907106	0,781663619
Gtf2h1	696,1322219	-0,171030561	0,080705114	-2,119203522	0,034073271	0,215686483
Aifm3	133,4150302	-0,053361774	0,169484915	-0,314846748	0,752878016	0,921533083
Tsga10	26,67472257	-0,459900779	0,287101928	-1,601872833	0,109183733	0,40910239
Sytl4	61,96564731	0,115093	0,243701874	0,472269655	0,636734327	0,86976752
Suv39h1	138,8064634	0,001108223	0,202227935	0,005480068	0,99562756	0,998990157
Pld1	300,619583	0,288502855	0,125814461	2,293081828	0,021843294	0,165171381
Atp11c	4320,345662	0,005136444	0,172469448	0,029781764	0,976241102	0,993495054
Btf3l4	397,2854857	-0,004570343	0,105168275	-0,043457428	0,9653369	0,989924013
Lrfn2	0,086476712	0,780932884	3,352475198	0,232942181	0,815806304	NA
Glipr2	42,73522622	0,096164585	0,24296125	0,395802149	0,692250991	0,895338108
Lca5	8,1794357	-0,956436961	0,509583133	-1,876900744	0,060531699	0,298065665
Eef2kmt	199,0629407	-0,186963671	0,118517955	-1,577513467	0,114677426	0,419229562
Hsph1	1907,501388	-1,350996724	0,195030526	-6,927103941	4,30E-12	3,38E-09
Atp6ap2	851,1360686	0,081698176	0,128154485	0,637497597	0,523800775	0,81568929
Mipep	898,4206639	0,248614388	0,140022574	1,775530769	0,07581029	0,340674878
Trpc5os	0,227324296	1,389394708	3,349408814	0,414817893	0,67827522	NA
Orm2	1820,464979	3,15121766	0,539103341	5,845294251	5,06E-09	1,93E-06
Mrpl20	1076,960677	-0,008793344	0,136276878	-0,064525579	0,94855174	0,985635646
Oxld1	229,1436083	0,448857284	0,149249193	3,007435246	0,002634622	0,045156754
Vps72	438,6740013	0,14660853	0,094444022	1,552332548	0,120582674	0,432066609
Gsta3	59586,81928	0,127886418	0,104325299	1,225842811	0,220257833	0,574490867
Tac1	0,179780087	0,059593471	3,352475198	0,017775962	0,985817581	NA
Actg2	2,190274455	2,395115569	1,281708106	1,86869035	0,061665907	NA
Abcg1	138,9167122	0,180467197	0,200338442	0,900811625	0,367688486	0,716004058
Smim8	230,7523303	0,412046437	0,133791351	3,079768877	0,002071613	0,038941909
Emb	8,927537646	0,379518089	0,502920761	0,754628002	0,450472217	0,773983173
Pdpn	11,35870502	1,967455146	0,543531463	3,619763123	0,000294873	0,009929861
Mir684-1	3,434770374	-0,026676192	0,991379117	-0,026908164	0,978532982	NA
Lrrc19	0,987706021	2,491821401	1,663541635	1,497901434	0,134158861	NA
Fancb	28,14805107	0,098830505	0,291388346	0,339171097	0,734480842	0,913675851
Smo	204,0723514	-0,056858922	0,145077185	-0,391921873	0,695115941	0,8969032
Lrrc58	1182,798193	-0,25257495	0,182699882	-1,382458203	0,166831056	0,50586627
Senp5	622,9813024	-0,192625695	0,130902802	-1,47151697	0,141151362	0,466809953
Msrb3	310,4535565	-0,165592075	0,116191028	-1,425170929	0,154107798	0,489157561
Tmem91	0,705351172	-0,353880096	1,81817105	-0,194635205	0,84567854	NA
Fndc7	2,403975445	0,972229399	0,996693996	0,975454254	0,329334983	NA
Cep95	145,9914901	-0,080242649	0,157361476	-0,509925626	0,610103568	0,85830523
Tmem154	5,313883836	0,010945304	0,816869867	0,013399079	0,989309402	NA
Dennd5b	1233,94079	-0,023141995	0,130592304	-0,177207957	0,859345046	0,95905468
Simc1	237,658018	-0,407880153	0,251514651	-1,621695405	0,104868583	0,402692427
Zmat4	0,259974817	0,586045239	3,35071904	0,174901337	0,86115716	NA
B430306N03Rik	61,79519675	0,244593331	0,281410259	0,869169914	0,384754201	0,729303688
Fbxo48	0,8590431	-0,687914996	1,574540045	-0,436899016	0,662184586	NA
Ptpn7	9,218327035	1,012012598	0,683677302	1,480248934	0,138806826	0,463001111
Topbp1	286,8782956	-0,157963413	0,150298468	-1,05099816	0,293259435	0,654344293
Map10	16,62279168	-0,360184611	0,372434408	-0,967108846	0,333489624	0,690158326
Mgat1	1487,734908	-0,084241973	0,081129751	-1,038361045	0,299101998	0,659904651
Fem1a	685,5526119	-0,098595051	0,111672281	-0,882896366	0,377292267	0,723157036
Fam234a	1406,509044	-0,12781318	0,069428723	-1,840926558	0,065632322	0,313259019
Cyp2a4	930,6025461	2,803207587	1,633167984	1,716423304	0,086084594	0,363775092
Fga	145742,6663	0,096133832	0,12914117	0,744408864	0,456629158	0,778260481

Sox12	130,611858	-0,488010366	0,303951649	-1,605552616	0,108372244	0,408552414
Snai1	25,09482811	0,23692031	0,332155723	0,71328083	0,475671997	0,789452019
Pirb	136,1546095	0,331850199	0,298713237	1,11093235	0,266597469	0,626827571
Urb1	103,6132242	-0,004680663	0,223844516	-0,020910333	0,983317184	0,995413661
Cxcr6	17,92053409	0,574985604	0,463775751	1,239792298	0,215052228	0,568551815
Kank3	102,7305737	0,22939674	0,192743518	1,190165784	0,233981239	0,59132234
Epsti1	72,37969175	0,161044492	0,212793746	0,75681027	0,449163539	0,773349269
Rab30	107,2437369	0,150277686	0,272631383	0,551211987	0,581488364	0,845891913
Cant1	610,9696311	-0,109613259	0,094541487	-1,159419665	0,246285165	0,605518092
Zdhhc20	237,4190445	0,171342869	0,188738397	0,907832596	0,363966674	0,712843608
Trappc8	1244,78526	-0,154089209	0,114311503	-1,347976402	0,177665973	0,52020802
Gm9776	15,94270956	0,213914505	0,380030299	0,562888028	0,573511133	0,842372044
Tead1	203,7015031	-0,19624758	0,190911455	-1,027950788	0,303972977	0,663347176
Snora47	0,847511966	2,30846597	1,884248046	1,225139108	0,220522812	NA
Lum	202,3358518	-0,280652534	0,286459395	-0,979728852	0,32721998	0,684930268
Slc39a7	2189,670374	-0,203268813	0,092072756	-2,207697704	0,027265353	0,190567596
Guca2a	0,113662148	0,780932884	3,352475198	0,232942181	0,815806304	NA
Thumpd3	425,5434885	-0,053603107	0,157004857	-0,341410498	0,732794573	0,912852278
Serpina1e	170596,6025	-6,001166507	0,810128453	-7,4076728	1,29E-13	1,56E-10
Serpina1c	36363,74467	-7,515214679	0,972315849	-7,72919076	1,08E-14	1,81E-11
Plxna2	838,5576468	-0,847215649	0,215866224	-3,924725387	8,68E-05	0,004058788
Gsta2	1343,354982	0,954064509	0,242825102	3,929019288	8,53E-05	0,00400099
Gsta1	63,21795578	0,253517166	0,472490006	0,536555615	0,59157461	0,850578037
Meis3	26,9703264	0,599361825	0,286132734	2,09469856	0,036197797	0,222803109
Grn	5545,863371	0,244470615	0,115121495	2,123587914	0,033704617	0,214383309
Gldn	10,93406477	1,213507978	0,69115069	1,755779162	0,079126124	0,348953931
Tagln2	503,2011063	-0,031126499	0,199417122	-0,156087396	0,875964133	0,96416635
Camk2g	260,1312749	-0,007403128	0,133531622	-0,055441013	0,955787122	0,987389387
Rnf24	124,4968027	-0,106250913	0,168441709	-0,630787435	0,52817952	0,81852028
Obsl1	18,9041167	0,109543066	0,460476665	0,23789059	0,811965954	0,942944131
Mfsd7b	78,01321521	0,092153556	0,179958002	0,512083683	0,608592444	0,857753956
B3gnt9	17,16425267	0,64308295	0,423580233	1,518208121	0,128961941	0,446309134
Hadha	7816,686197	-0,077947724	0,098347464	-0,792574828	0,428025579	0,75995385
Slc9b2	3,450405364	0,249431831	0,934278051	0,266978155	0,78948598	NA
Ints3	880,7152313	-0,304502372	0,084278375	-3,613054623	0,000302611	0,010095144
Wdr92	265,1835405	-0,239876587	0,129799856	-1,848049712	0,064595152	0,309635203
Ttll1	93,85773703	-0,029125599	0,188957051	-0,154138725	0,877500355	0,965038548
Gins2	15,49138786	-0,132862211	0,51185388	-0,259570584	0,795195029	0,937556185
Vcpip1	788,1636673	-0,084437995	0,143564779	-0,588152575	0,556429886	0,833822555
Plekha7	158,5191325	-0,255432194	0,167700734	-1,523142964	0,127722942	0,444900472
Hs3st3a1	16,72063295	-0,022137969	0,346695298	-0,063854252	0,94908628	0,985650844
Vps39	472,1051277	0,020354586	0,104266674	0,195216607	0,845223378	0,953247119
Sirt5	329,5634523	0,257581557	0,143788259	1,791394928	0,073229942	0,333223655
Zfp277	291,3263467	0,204328097	0,108235248	1,887814744	0,059050825	0,293856809
Acad12	121,7823714	0,012937756	0,175264355	0,073818525	0,941154787	0,983128178
Slc7a6	16,60557051	1,965365995	0,558138386	3,521287989	0,000429456	0,01312798
Rpf1	611,0855188	-0,100815057	0,139092339	-0,724806682	0,468570636	0,78348591
Anapc10	105,7297586	0,040835462	0,206048351	0,198183881	0,842901199	0,952476217
Dcaf12	706,0033973	-0,141178598	0,087023487	-1,622304537	0,104738158	0,402484746
2310057M21Rik	39,89332436	-0,165453539	0,284097086	-0,582383796	0,560308194	0,835469208
Toe1	173,6727001	-0,13180128	0,144292739	-0,913429751	0,361016565	0,710581841
Rpa1	674,2764583	0,025780652	0,128227242	0,201054408	0,840656031	0,951708907
Pomgnt1	466,7624082	-0,382369378	0,114175387	-3,348965015	0,00081114	0,020557413
Dnaaf1	0,373092283	2,037329247	3,059752286	0,665847774	0,505508405	NA
Zdhhc21	244,1790529	-0,044330128	0,135350957	-0,327519871	0,743274716	0,917190298
Cuedc2	379,525419	0,036377833	0,142152176	0,255907679	0,798022125	0,938325224
Rnf152	438,9803716	-0,143647474	0,249379041	-0,576020637	0,564601233	0,837355044
Rab4a	434,7091858	-0,149466753	0,177910147	-0,840124946	0,400838334	0,741644961
Crat	1745,838417	-0,128905323	0,1209502	-1,06577189	0,286526771	0,648131311
Il3ra	41,32241412	-0,259090626	0,400123973	-0,647525875	0,517291654	0,813424627
Il18	318,7049322	0,305600566	0,11624693	2,628891504	0,008566369	0,095835801
Cpox	2610,09338	-0,113022877	0,187920674	-0,60143929	0,547547436	0,828552821
Pnn	875,2423261	-0,126266877	0,137256101	-0,919936355	0,357606019	0,707149983
Grb2	1231,630161	0,002601703	0,074972725	0,03470199	0,972317374	0,992650147
Igfbp1	2404,41189	0,660395753	0,858240543	0,769476295	0,441610612	0,768930234
Icam5	1,260078748	0,978675337	1,583962569	0,617865192	0,536664205	NA

Fst	186,6220123	-1,708307877	0,337885445	-5,055878858	4,28E-07	6,66E-05
Eno3	103,7229655	0,234088051	0,225484207	1,038157192	0,299196879	0,659904651
Cox7c	2634,317173	0,093157473	0,125308115	0,7434273	0,457223021	0,778577833
Cdk8	688,9928127	-0,159800912	0,110234054	-1,449651043	0,147155854	0,477390589
Ugt2b34	13052,18026	-0,291168242	0,15155295	-1,921231106	0,054702578	0,283347063
Trpt1	83,91143334	0,221092879	0,176713182	1,251139707	0,210883511	0,563522218
Tmem17	27,06024645	0,309123019	0,27026154	1,143792116	0,25270986	0,612043137
Pcmtd2	736,4911507	0,091019008	0,151549361	0,600589847	0,548113202	0,828815941
Erlin2	736,4667557	-0,094100899	0,105297519	-0,893666817	0,371500185	0,719169703
Fzd2	2,652829269	1,051204007	1,009911164	1,040887599	0,297927718	NA
Tsc2	1093,539507	-0,181497864	0,095435791	-1,901779855	0,057199941	0,289000033
Cds2	2171,540264	0,163670767	0,102350398	1,599121946	0,109793509	0,410122779
Meaf6	167,3866918	-0,325541151	0,149307715	-2,18033711	0,029232482	0,197278674
Nek7	1136,999674	0,11482761	0,107054088	1,072613035	0,283444774	0,644784493
Tpte	0,241026348	0,059593471	3,352475198	0,017775962	0,985817581	NA
Ccr6	0,962640538	-0,744339097	1,601975619	-0,464638218	0,642190559	NA
Igll1	0,086476712	0,780932884	3,352475198	0,232942181	0,815806304	NA
Chchd7	344,2701491	-0,045611507	0,120542503	-0,378385268	0,70514441	0,90193988
Clec4n	66,11075584	0,393541551	0,2922482	1,346600428	0,178108956	0,520240034
Cyp2c29	33414,74939	0,267674059	0,093218055	2,871482977	0,004085508	0,059955164
Rab23	257,1794875	0,425914831	0,243084943	1,752123456	0,079752582	0,349577792
Casp4	28,30481864	0,049377783	0,292080932	0,16905514	0,86575327	0,961775826
Ophn1	50,32030416	0,140691971	0,234651097	0,599579428	0,548786561	0,82900876
Nxf7	2,060416363	0,676924658	1,357005817	0,498836961	0,617894247	NA
Papln	44,2129689	0,119714405	0,290194344	0,412531835	0,679949652	0,891462871
Card14	4,315804873	0,783868517	0,866788502	0,904336543	0,365816975	NA
Idh3b	4651,345754	0,006294776	0,062781862	0,100264246	0,920134542	0,977341679
Cyp4f13	1681,904205	-0,240485688	0,113317326	-2,122232283	0,033818237	0,214781952
Pabpc4	718,16082	-0,246672446	0,115692452	-2,1321395	0,03299538	0,211870912
Usp48	851,6632029	-0,297432718	0,116969908	-2,542813978	0,010996375	0,11179509
Gmppa	972,3676795	-0,458842259	0,119604077	-3,836342962	0,00012488	0,005295032
Peg10	46,4941651	-0,937027384	0,49153358	-1,906334423	0,056606831	0,287419948
Nobox	0,456530809	-1,313010104	2,134867087	-0,615031311	0,538534043	NA
Kirrel	42,10825531	0,199339576	0,272882757	0,730495318	0,465087474	0,781663619
Lzts2	247,899391	0,090611564	0,197853079	0,457973991	0,646971128	0,876230372
Eml4	406,2619392	-0,045861582	0,116328189	-0,394243067	0,693401593	0,89592016
9030624G23Rik	0,340986444	1,884176617	3,333184127	0,565278288	0,571884494	NA
4930513O06Rik	4,95497954	1,971919507	0,923857991	2,134440061	0,032806775	NA
Mctp1	0,907581329	0,828147574	1,658352798	0,49937961	0,617511981	NA
Nxnl2	0,93925802	-1,360479046	1,842960703	-0,73820296	0,460391125	NA
Efcab11	1,383415074	-1,311997659	1,368174308	-0,95894043	0,337588756	NA
Zc2hc1b	0,122496332	0,780932884	3,352475198	0,232942181	0,815806304	NA
Rbm6	665,5679968	-0,216299287	0,090728307	-2,384033112	0,017124062	0,144899552
Uhrf1bp1l	1114,008353	0,301934935	0,110897664	2,722644692	0,006476167	0,080389854
Fam122b	50,50685074	0,219292843	0,228315611	0,960481164	0,336813107	0,69270916
Arhgap10	73,83509201	0,418988756	0,272005633	1,540367938	0,123470692	0,436341444
Foxd2	0,640543484	0,96868348	2,057726341	0,470754279	0,637816218	NA
Gm867	5,879490721	-0,114567178	1,276251516	-0,089768496	0,928471184	0,979385455
Adarb2	2,59687889	-0,71788234	0,950875835	-0,754969591	0,450267228	NA
Atg9a	1631,187359	-0,116151569	0,071493599	-1,6246429	0,104238671	0,401673966
P2ry14	38,51884312	-0,103093809	0,327741931	-0,314557886	0,753097359	0,921533083
Rap1gds1	639,4757801	-0,134230556	0,086834203	-1,545825853	0,122146631	0,434047185
Zfp27	142,5409326	-0,280723655	0,178571574	-1,572051188	0,11593868	0,42199407
Fgfr4	1067,991253	-0,015545888	0,138955024	-0,111877122	0,910920837	0,974815504
Odf2	222,0751635	0,003429894	0,114243446	0,030022672	0,976048972	0,993495054
Slc25a22	3523,813137	-0,043901211	0,130659108	-0,335998089	0,736872307	0,914722813
Plxdc1	15,26857222	-0,311866565	0,482009447	-0,647013388	0,517623279	0,813424627
Ccnt2	339,9271203	-0,099259518	0,136012875	-0,729780312	0,46552448	0,781663619
Cdh26	1,529651837	0,471980635	1,373683873	0,34358752	0,731156511	NA
Fbxo33	315,8272162	-0,193509505	0,158686102	-1,219448345	0,22267407	0,577371924
Fiz1	405,6452239	-0,081426324	0,106089496	-0,767524844	0,442769528	0,769450906
D430042O09Rik	405,1032669	-0,445023655	0,265755942	-1,674557686	0,094021047	0,380783818
Myo5c	5,264299683	0,394230025	0,709997929	0,555255176	0,578720134	NA
Cyp2c44	10292,52995	-0,294022685	0,154249349	-1,906151873	0,056630504	0,287430983
Mrm2	161,6771058	-0,080636302	0,166335338	-0,484781547	0,627831314	0,865852703
Nudt12	964,0429241	-0,038151945	0,149629039	-0,254976873	0,798740961	0,938733973

2310035C23Rik	410,1744367	0,178991577	0,127914716	1,399304032	0,161721831	0,49843611
Fggy	2564,292808	0,183677791	0,07157234	2,566323699	0,010278286	0,106602332
Prr29	0,142634598	-0,517475177	3,352475198	-0,154356154	0,877328924	NA
Rn45s	96355,72418	-0,392915648	0,351227949	-1,118691292	0,263271862	0,623393822
Zc3h12a	79,66574234	0,34463771	0,199118622	1,730816067	0,083484575	0,357477432
Mark4	231,3025132	-0,189086235	0,16614779	-1,138060491	0,255095224	0,615589901
3110070M22Rik	12,45656347	0,346962982	0,525662365	0,660049122	0,509222307	0,807950751
Baiap2l1	980,3762639	-0,328294648	0,081816414	-4,012576847	6,01E-05	0,003100138
Rwdd1	500,7513873	-0,009359378	0,100797379	-0,09285339	0,926020035	0,978534437
Lman2	6013,310745	-0,169337802	0,099300722	-1,705302832	0,08813796	0,368178985
Nepn	0,260519499	-0,589751774	3,345560051	-0,176278938	0,860074808	NA
Ppdpf	1630,398788	0,072633114	0,105312755	0,689689626	0,490389391	0,797285888
Mrpl16	974,7726377	0,002509037	0,092829573	0,027028423	0,978437064	0,994429459
Prelid1	3415,224623	0,048133084	0,056753746	0,848104101	0,396379998	0,737761616
Lamtor1	1528,20649	0,071549251	0,073453465	0,974075902	0,330018853	0,686792356
Mrpl51	1607,381629	0,082862698	0,109726237	0,755176705	0,450142964	0,773715774
Zmat2	1014,053729	-0,170024049	0,10679039	-1,592128734	0,111355782	0,413073099
Psmg3	284,1016919	0,08054848	0,193050157	0,417241206	0,676501988	0,889512888
Polr2l	408,562217	0,054282974	0,101799161	0,533235964	0,593870249	0,851766384
Rsrc1	372,5513612	0,032951962	0,096941915	0,339914489	0,733920925	0,913675851
Ccdc59	273,5222958	0,075674414	0,10071837	0,751346692	0,452444035	0,774936437
Fam136a	1324,271245	0,073674126	0,099849069	0,73785491	0,460602622	0,780953195
Acot11	184,2997775	0,36009211	0,561514952	0,641286771	0,521336383	0,814845193
Rpl36al	2628,942303	-0,145722249	0,112764765	-1,292267563	0,196264501	0,545184577
Dda1	362,8385138	-0,174914484	0,124332771	-1,406825269	0,159479208	0,495312554
Exoc2	243,918079	0,099494755	0,119356442	0,833593504	0,404510087	0,744376512
Rps21	1889,093225	0,029258005	0,155826094	0,187760629	0,8510643	0,955719447
Rpl15	2445,813987	-0,012207124	0,095833517	-0,127378446	0,898640875	0,971764932
Ctrb1	0,355017277	-2,060768074	3,080293056	-0,669016888	0,503484705	NA
Fbxo25	152,9143929	0,007265973	0,15219316	0,047741784	0,961922033	0,988526411
Mis18a	51,39376372	0,337060389	0,278418424	1,210625303	0,226039042	0,581158101
Pnkd	5289,036714	-0,119504707	0,097955608	-1,219988409	0,222469269	0,577161019
Mrps25	383,3865559	0,105966128	0,134406099	0,788402676	0,430461206	0,761221675
3632451O06Rik	1,849772116	0,273451911	1,209968314	0,225999233	0,821202017	NA
Ribc1	38,49398723	-0,072257674	0,237732876	-0,303944811	0,761169937	0,92534041
Srsf9	485,8997243	0,013087078	0,124040391	0,105506582	0,915973847	0,976559041
2610528J11Rik	260,6607197	0,291558598	0,181952493	1,602388583	0,109069708	0,40910239
Nradd	16,88296367	0,477201356	0,375184498	1,271911176	0,20340468	0,555983883
Rbm22	468,366411	0,263537123	0,113616848	2,319525021	0,020366586	0,159321761
Mgst3	532,5812794	0,63626954	0,172096991	3,697156688	0,000218028	0,008029789
Borcs7	154,6091083	0,440189666	0,152725012	2,882236907	0,003948627	0,058659427
Gfpt1	653,9101306	-0,895698699	0,149599921	-5,987293945	2,13E-09	8,95E-07
Ankra2	227,5156821	0,236915938	0,109821339	2,157285093	0,030983458	0,204100361
Capzb	2501,648514	0,016990242	0,060586627	0,28042891	0,779148459	0,931369434
Pira6	1,069428369	1,548763422	1,68374964	0,919829994	0,357661606	NA
4930562C15Rik	0,142634598	-0,517475177	3,352475198	-0,154356154	0,877328924	NA
Mnd1-ps	0,174042788	-1,166820872	3,345560292	-0,348766954	0,727264274	NA
BC051019	0,122496332	0,780932884	3,352475198	0,232942181	0,815806304	NA
2700049A03Rik	89,15556848	-0,001364368	0,19922907	-0,006848236	0,994535941	0,998494367
Mn1	319,9805279	-0,151223758	0,148747638	-1,016646448	0,30932165	0,668280727
Dnajc5	1181,456674	-0,116729275	0,080972102	-1,441598675	0,149415622	0,48121837
Treh	45,31776373	0,754572351	0,378884778	1,991561536	0,046419187	0,256037813
Umod	0,12663974	0,780932884	3,352475198	0,232942181	0,815806304	NA
Apool	613,9928116	0,000672644	0,094390135	0,007126206	0,994314158	0,998457633
Fam170b	0,504967481	1,466558707	2,398555497	0,611434136	0,54091221	NA
Card6	357,429243	-0,071019462	0,121748181	-0,583330785	0,559670642	0,835350766
Tex11	0,328697462	-0,630293653	2,671069774	-0,235970493	0,813455567	NA
Elf3	38,79304049	-0,081671259	0,264711544	-0,308529267	0,757679634	0,923804952
Cog5	190,9922485	0,087315928	0,186817688	0,467385767	0,640223904	0,871049783
Map2k7	912,6886778	0,088665294	0,106839915	0,829889225	0,406601418	0,745871812
1700024P16Rik	283,5435213	0,003697656	0,148775993	0,024853847	0,980171541	0,994739255
Man2c1os	18,89310622	0,237713448	0,344946411	0,689131529	0,4907405	0,797476473
Lncbate1	28,20509705	2,627539247	0,458891798	5,725836148	1,03E-08	3,53E-06
Abhd6	897,8637936	-0,469812912	0,183548574	-2,559610793	0,010478945	0,108096461
Acbd5	5264,331113	-0,137140784	0,132842652	-1,032355059	0,301905817	0,662128551
Zfp712	17,25204161	0,319199076	0,441879541	0,722366724	0,47006904	0,784465484

Fam129c	10,1735569	0,147374564	0,511972353	0,28785649	0,773456594	0,928621142
Arsg	560,056976	-0,152774088	0,105570545	-1,44712796	0,147861088	0,478054387
Dmkn	2,750111995	3,389955391	1,406393942	2,410388221	0,015935554	NA
Trim66	1,06479784	1,122992209	1,597468715	0,702982286	0,482066789	NA
Cuedc1	46,89610592	0,047733771	0,248831884	0,19183141	0,847874266	0,953880403
Traip	7,145235451	-0,236641764	0,506220786	-0,467467497	0,640165442	0,871049783
Zw10	341,7962314	-0,340440365	0,103950076	-3,275037177	0,001056481	0,024779106
Pnck	1,532253039	1,263226323	1,168373289	1,081183844	0,279615343	NA
Vat1	640,4091708	0,239598682	0,11587656	2,067706194	0,038667659	0,230715978
Gnpda1	459,6252072	0,175079308	0,274627386	0,637515836	0,523788898	0,81568929
Mir7059	0,213116452	1,337107854	3,350237755	0,399108348	0,689813372	NA
6230400D17Rik	3,264785644	0,792917084	0,899848557	0,881167257	0,378227299	NA
Spag8	6,947174528	-0,115835175	0,588910741	-0,196693942	0,844067051	0,953068489
Rbms1	334,0266001	0,248641233	0,174720736	1,423077988	0,154713548	0,489743509
Mpzl1	209,2255769	0,297114106	0,195843763	1,517097618	0,12924204	0,446717571
Ceacam2	2,658470768	0,628948446	0,918496952	0,684758338	0,493496429	NA
Gucy1b3	121,8570035	-0,132160405	0,241872798	-0,546404583	0,584787861	0,847207294
Pnp2	6,434976007	0,676914164	0,843311788	0,802685524	0,422156522	0,755522295
Naip2	143,3088407	0,563208337	0,229436993	2,454740753	0,014098622	0,12927605
Ehbp1l1	202,5980559	0,189495376	0,189834996	0,998210975	0,318177066	0,675759967
Mir6236	10276,03082	0,200527334	0,238325074	0,841402588	0,400122436	0,740788928
Arvcf	300,5820769	0,049259535	0,128304795	0,38392591	0,701033362	0,899833253
Plin2	10803,66074	-0,234156443	0,173468725	-1,349848186	0,177064685	0,520079413
Osm	0,656850242	0,036024628	2,024992363	0,017790007	0,985806377	NA
Mtf2	150,8352918	0,163635401	0,179789518	0,910149841	0,362743493	0,711918625
Esyt1	461,9529002	-0,215939273	0,130637247	-1,652968644	0,098337245	0,390573569
Cldn1	3305,145235	-0,159487886	0,117208578	-1,360718542	0,173602653	0,516098257
Tnfrsf1a	1934,654456	0,333055814	0,081499643	4,086592324	4,38E-05	0,00239809
Mok	3,123541906	-1,315825076	1,156946559	-1,137325718	0,255402147	NA
Tnc	22,77320536	-0,227742189	0,503016913	-0,452752548	0,650726936	0,876972622
Tjp2	818,3530695	-0,225228628	0,097162147	-2,318069679	0,020445534	0,159672894
Tmem165	232,5898056	-0,038693299	0,170107742	-0,227463482	0,820063362	0,946381744
Rbm15	187,7492204	0,015358303	0,214984628	0,071439075	0,943048312	0,983437952
Heatr9	0,206965523	0,059593471	3,352475198	0,017775962	0,985817581	NA
Gm12253	0,273628144	-1,689229531	3,311482904	-0,510112714	0,609972499	NA
Gm12185	5,102022112	0,326293926	0,799851476	0,407943144	0,683315417	NA
Zzef1	896,0185704	-0,323143392	0,115197458	-2,805126075	0,00502969	0,069321576
Rpl32l	1,445376043	0,168723741	1,214348899	0,138941733	0,889496192	NA
Cdh18	3,157397289	0,242654633	0,808541379	0,300114056	0,764090158	NA
Spata22	138,9643303	-0,089795591	0,155145773	-0,57878207	0,562736231	0,83722511
Ccnjl	10,66384866	0,352664424	0,584676535	0,603178686	0,546389825	0,828173482
Cabp2	3,161191594	-1,029529919	0,856563147	-1,201931138	0,229390209	NA
Pphln1	386,8152896	-0,138079595	0,118435811	-1,165860171	0,243670984	0,602261755
Celf2	174,5127876	0,018497083	0,180484925	0,102485472	0,918371344	0,976907366
Ipo8	1112,915982	-0,247286879	0,108404543	-2,281148676	0,022539651	0,168540055
Hsf5	3,892843258	-0,181479295	0,828770721	-0,218974066	0,82667025	NA
Polr3a	356,6234677	-0,042810272	0,099062714	-0,432153233	0,665630047	0,884945574
Pla2g16	238,6412084	0,445887376	0,181832274	2,452190511	0,014198946	0,129557379
Dppa3	1,161776942	-1,414542873	1,601498813	-0,883261893	0,377094788	NA
Gbgt1	6,136808841	-0,296968042	0,542710085	-0,547194625	0,584245028	0,847068435
Arap3	270,8306983	-0,075803963	0,233773847	-0,324261948	0,745739726	0,918959756
Cyp2c68	5710,14979	0,740851746	0,221559463	3,343805485	0,000826377	0,020766595
Sorbs1	1134,779509	-0,235061704	0,138197489	-1,700911533	0,088959601	0,369687145
Anks6	7,670454228	0,997787409	0,639335174	1,56066403	0,118603043	0,427632243
Bach2	154,9374115	0,442639679	0,580454473	0,762574327	0,4457173	0,771326444
Tec	564,7757194	0,163694771	0,198655858	0,8240118	0,409932853	0,747360196
Cep170	159,6462444	-0,094657391	0,161285217	-0,586894401	0,55727463	0,833822555
Cyp26a1	759,5284496	0,082543963	0,278856129	0,29600914	0,767223101	0,927501905
Ces1c	80356,67381	0,164456085	0,074231985	2,215434302	0,026730269	0,189278055
Mir3068	0,20013886	1,337107854	3,350237755	0,399108348	0,689813372	NA
4931406H21Rik	0,896754531	-0,12118514	1,543728989	-0,078501564	0,937429086	NA
Myo15	1,54409453	-0,285429448	1,316096493	-0,216875776	0,828305156	NA
Ly6d	24,50485563	1,259605583	0,48378992	2,603620972	0,009224471	0,100023317
Mdk	13,57807998	0,25442667	0,414819958	0,613342405	0,53964996	0,826407188
Bckdk	329,3786322	-0,043615786	0,112865202	-0,386441394	0,699169799	0,898942204
Sep-04	299,9840693	0,135755071	0,164670885	0,824402394	0,409710955	0,747057523

Supplementary Table S1: *Serpina1* KO vs. wildtype all DEGs

Rev3l	391,3272359	-0,279748053	0,159327847	-1,755801377	0,079122329	0,348953931
Ccnd2	134,3104506	-0,29009908	0,208689217	-1,390100954	0,164498224	0,502924549
Cited2	510,8538518	-0,02692318	0,185079804	-0,145467951	0,884341418	0,967085557
Msc	4,2318502	0,259481686	0,749003856	0,346435714	0,729015289	NA
Mrvi1	38,98652966	-0,099285127	0,241800517	-0,410607589	0,681360298	0,89166825
Cst7	0,700415565	2,033883025	1,941322851	1,047678918	0,294786564	NA
Rasgrp2	284,0648807	0,607410945	0,163214736	3,721544765	0,000198008	0,007482101
Meis2	104,7980937	-0,115031511	0,236272846	-0,486858788	0,626358406	0,865266164
Cbfa2t3	54,31570628	-0,248675876	0,269953947	-0,921178886	0,35695704	0,707128379
Mpo	0,48947453	-1,55859538	2,895569783	-0,538268975	0,590391367	NA
Ahr	720,1073945	0,468208651	0,196394805	2,384017498	0,017124788	0,144899552
Hdgfrp3	13,23045861	0,089052386	0,485504234	0,18342247	0,854466546	0,957135493
Sdcbp	1151,916357	0,086944006	0,099310192	0,875479192	0,381313227	0,72637014
Wwp2	545,3676114	0,321546769	0,113550206	2,831758563	0,004629279	0,065283575
Haus2	252,3407834	0,130757121	0,144377332	0,905662402	0,365114569	0,713569421
Surf2	312,6839308	0,216017128	0,106121632	2,035561685	0,041794388	0,241464641
Surf1	1043,391593	0,052554154	0,089486196	0,587287828	0,557010415	0,833822555
Supt5	1735,410277	-0,225298874	0,092164554	-2,444528456	0,014504164	0,130929214
Nat8f4	189,8882983	0,120055945	0,147190929	0,815647717	0,41470165	0,751551608
Cyfip1	983,6333516	0,088630868	0,121334922	0,73046462	0,465106232	0,781663619
Tmem184a	294,559088	0,559790386	0,150002754	3,731867384	0,000190066	0,007282032
Sema4a	2205,095861	0,302967563	0,139085197	2,178287618	0,02938463	0,197530872
Hsd17b13	11915,8372	-0,191154075	0,129522068	-1,475841744	0,139986382	0,46507901
Txlna	766,830198	-0,614500043	0,132438776	-4,639880094	3,49E-06	0,000361286
Cnot7	465,0900072	0,106889155	0,111887371	0,955328148	0,339411777	0,694134533
Myh14	503,9567944	0,332027979	0,171828158	1,932325783	0,053319308	0,278665297
Lgr4	1127,633926	0,001409326	0,111223722	0,012671095	0,989890199	0,996824501
Dhx29	409,9912171	-0,32073098	0,125193583	-2,561880354	0,010410718	0,107558643
Ifitm1	208,9218709	0,214756372	0,212941861	1,008521156	0,313204337	0,672219008
Atp6v0a1	1217,155356	-0,022654536	0,108294765	-0,20919327	0,834297366	0,95084595
Sdccag3	971,8169978	-0,134055806	0,107971815	-1,24158149	0,214391015	0,56766844
Tmem184b	697,3007737	-0,083592759	0,113657173	-0,735481596	0,462046244	0,781516914
Gas7	245,0146129	0,757014052	0,275166824	2,751109456	0,005939379	0,076590047
Vps13d	1846,201975	-0,019492374	0,135491771	-0,143863893	0,885607948	0,967611365
Zmynd8	921,0013702	-0,169464471	0,135876566	-1,247194242	0,212326284	0,565238523
Tbx10	0,122496332	0,780932884	3,352475198	0,232942181	0,815806304	NA
Vldlr	93,66096838	1,622599859	0,755961491	2,146405444	0,031840645	0,207243227
Rad21l	0,086476712	0,780932884	3,352475198	0,232942181	0,815806304	NA
Fam118b	127,2146211	-0,024818512	0,175000867	-0,141819368	0,887222685	0,968356501
Klc3	6,103088202	1,174538798	0,633296955	1,854641475	0,063647441	0,306733002
Ncf1	100,4282905	0,070840933	0,229491069	0,308687101	0,757559557	0,923804952
Agbl3	83,64025419	0,017302282	0,210434696	0,082221623	0,934470479	0,98189048
Snord88c	0,906931882	1,488825976	2,002757555	0,743388021	0,457246795	NA
Dhx15	1598,847245	-0,064680606	0,092943077	-0,695916342	0,486481222	0,79498441
Top2b	1094,953189	0,026464169	0,08255141	0,320578031	0,748530188	0,919838327
Klra9	1,594371171	-0,396942683	1,282223417	-0,309573728	0,756885137	NA
Hsp90aa1	5522,779302	-0,858642543	0,218997032	-3,920795347	8,83E-05	0,004111186
Zswim4	248,3340554	-0,264258847	0,151802619	-1,740805583	0,081717663	0,353542587
Rbbp5	559,5988948	-0,013114309	0,103919009	-0,1261974	0,89957567	0,972141416
Lrtm2	10,18544332	2,850461267	0,940230576	3,031661957	0,002432114	0,043123253
Nhlrc3	149,173893	0,000674174	0,198124021	0,003402787	0,997284974	0,999481197
Fam161b	14,2716928	0,122377241	0,497814029	0,245829233	0,805814426	0,941208637
Syne3	75,75451831	-0,445575713	0,248107045	-1,795901089	0,072510249	0,330736787
Fbxo45	213,5194142	-0,095984968	0,129389952	-0,741827057	0,458192125	0,779234832
Nav1	93,34408265	-0,19196763	0,236135186	-0,812956482	0,416243009	0,751673636
9930111J21Rik2	0,880920111	0,815507584	1,705158711	0,47825905	0,632465839	NA
Pskh1	758,0784794	-0,061515045	0,09781389	-0,628898866	0,529415269	0,818994761
Mrps27	1061,076138	-0,063969364	0,109788642	-0,58265922	0,560122731	0,835463907
Zfp775	60,18783238	-0,210347115	0,189046878	-1,112671719	0,265849446	0,626006345
Sspo	28,15272408	-0,279067443	0,332882372	-0,838336502	0,401841741	0,742183948
Apex1	802,764129	-0,093947392	0,165128089	-0,568936472	0,569399253	0,840859231
Apbb2	179,6628062	0,018894648	0,161578466	0,116937908	0,906909258	0,974557501
Blmh	921,5136711	0,120782117	0,098673315	1,224060594	0,22092937	0,575078806
Plppr1	0,895693101	-0,394387753	1,484338417	-0,265699351	0,790470764	NA
Faap24	57,53285837	0,467892533	0,198799204	2,353593601	0,018592926	0,151643025
Pja1	1472,419415	-0,11540829	0,088905945	-1,298094176	0,194254975	0,543304344

Supplementary Table S1: *Serpina1* KO vs. wildtype all DEGs

Tek	189,1251206	-0,170776585	0,30380128	-0,562132541	0,574025719	0,84275089
Mlxip	181,7604324	0,036198062	0,185675142	0,194953735	0,845429167	0,953318365
Plcb2	44,27429066	0,063610246	0,281567965	0,225914359	0,821268029	0,946779723
Sfxn5	734,7225897	-0,043853641	0,18809533	-0,233145823	0,815648173	0,944267443
B3galnt2	701,3856923	-0,407277304	0,098079096	-4,152539313	3,29E-05	0,001984851
Tmem108	10,96117139	1,000780895	0,437123834	2,289467689	0,022052193	0,166085663
Kat5	417,935969	0,101182468	0,091999357	1,09981713	0,271411807	0,633246849
Uvrag	657,3064122	-0,30828258	0,114457848	-2,693415842	0,0070724	0,084951404
Mogat2	7,775937065	1,23666588	0,627320972	1,97134471	0,048684461	0,264148764
Ing1	259,5677247	-0,251424903	0,106865802	-2,352716199	0,018636852	0,151739388
Clcn4	1352,813729	-0,22552626	0,106221375	-2,123172099	0,033739433	0,214383309
Dcst1	0,808700243	2,201738976	1,890130989	1,164860525	0,244075455	NA
Atl1	32,2409121	-0,131212724	0,292479833	-0,448621442	0,653704763	0,878329546
Poldip3	789,9442627	0,093712889	0,1053828	0,88926171	0,373862445	0,720823634
Cdc42se2	302,2005496	-0,178943492	0,11090592	-1,613471063	0,106642205	0,405258567
Khk	10610,08826	0,368839874	0,204311228	1,805284404	0,071030189	0,326548348
Ube2a	606,1854015	0,024257825	0,103646209	0,234044495	0,814950436	0,944076707
2410016O06Rik	137,913805	0,491628418	0,227201636	2,163841892	0,030476486	0,20212719
Fbxl2	1,562611863	0,166025478	1,286236184	0,129078532	0,897295509	NA
Suds3	1837,53442	-0,062662774	0,074041419	-0,846320535	0,397373955	0,738461552
Rab3gap1	784,8997713	-0,021662545	0,086474632	-0,250507516	0,802194893	0,939799232
Timm29	1065,398291	-0,083753818	0,090390212	-0,926580606	0,354144328	0,704551502
Mfsd11	276,9653166	-0,24011807	0,111624325	-2,151126739	0,031466198	0,206110532
Fam83g	74,57562226	0,207533901	0,226725284	0,915354023	0,360005807	0,709765172
Psmd11	2856,926856	0,105508117	0,087984443	1,199167859	0,230462682	0,586979539
Rgmb	41,01144781	0,066440109	0,278445672	0,238610672	0,811407489	0,942886004
Crlf3	157,5819801	0,11313087	0,129513172	0,873508603	0,382385919	0,726783025
Brap	2672,766941	-0,135653086	0,094935526	-1,428896974	0,153033847	0,487585678
Calca	0,608437645	0,811173984	2,082145446	0,389585648	0,696842966	NA
Gm3219	259,0543218	-0,077167801	0,163590671	-0,471712722	0,637131855	0,869874976
Tcerg1	409,1235932	0,082977132	0,152203038	0,54517395	0,585633886	0,847482024
Lrrc40	249,9903853	0,118606867	0,153387318	0,773250804	0,439373964	0,766839495
Kynu	5035,96754	0,255514683	0,087912962	2,906450611	0,003655546	0,056367929
Psd2	0,213661134	0,059593471	3,352475198	0,017775962	0,985817581	NA
Olfm1	56,71730892	0,323249017	0,318574182	1,014674246	0,310261135	0,669444982
Nacc2	600,1994377	-0,123472883	0,106552294	-1,158800793	0,246537394	0,605683673
Rnf146	482,7463775	0,088656659	0,083706219	1,059140653	0,289535728	0,650992794
Alkbh1	108,6780785	0,153458087	0,250888935	0,611657451	0,540764419	0,826407188
Tmed7	1994,804991	-0,06489889	0,178975504	-0,362613256	0,716893807	0,907504337
Tmem186	323,0004053	0,08198368	0,123090387	0,666044536	0,505382634	0,805643837
Klhl28	51,448783	-0,231726895	0,257931373	-0,898405233	0,368969548	0,717178523
Sorcs3	0,907486126	2,437303396	1,857400142	1,312212345	0,189448498	NA
Atp5h	4799,736226	0,172581794	0,093715621	1,841547794	0,065541323	0,312991971
Dusp3	828,6863194	-0,187806616	0,07571211	-2,480536009	0,013118501	0,123354086
Tm9sf1	1299,747065	-0,230216627	0,069454742	-3,314627897	0,000917652	0,022104663
Gorasp1	630,4708927	-0,112410967	0,135773039	-0,827932908	0,407708497	0,746679655
Stx11	9,300172542	0,049359757	0,513453986	0,096132776	0,923415121	0,977755168
Snx11	177,0080877	-0,087766044	0,171587626	-0,511494017	0,609005177	0,857753956
Echs1	5740,90476	0,222640569	0,066755052	3,335186798	0,000852421	0,021158908
Mef2a	591,1249232	-0,054477538	0,099154394	-0,549421321	0,582716346	0,846891345
Ctnnd1	2750,642449	0,083360536	0,103589942	0,804716503	0,420983294	0,754607148
Cipc	688,8395611	-0,163383684	0,115450054	-1,415189324	0,157012994	0,492826562
Galk2	330,0893643	0,145130205	0,125191253	1,15926793	0,246346989	0,605518092
Mgea5	1013,753979	-0,183043034	0,08981173	-2,038074922	0,041542441	0,240737274
Etv5	91,26865301	0,062522379	0,184703775	0,338500817	0,734985812	0,913858791
Pank1	4756,753647	0,37059716	0,160765889	2,30519772	0,021155503	0,16249252
Wdr54	9,734230265	0,095858356	0,440067086	0,217826689	0,827564149	0,947580409
Commd4	366,8878034	-0,046506029	0,123478685	-0,376632035	0,706447078	0,902122257
Ggt6	834,0115321	0,238547257	0,1352354	1,763940924	0,077741926	0,345490591
1810055G02Rik	410,2164151	0,200905164	0,22528267	0,891791472	0,372504714	0,719447489
Cyb561d1	142,1264638	0,264904316	0,163118955	1,623994682	0,104376944	0,401673966
Tnfrsf13c	3,728753703	0,714960029	0,846142982	0,844963611	0,398131157	NA
Fam83d	4,49640985	-0,928137365	0,695485096	-1,33451798	0,182034158	NA
Klhl6	51,83682832	0,191099533	0,232841864	0,82072669	0,411801964	0,749099865
Actl9	1,368027437	-1,222742853	1,457184358	-0,839113353	0,401405703	NA
Tcf23	1,807518765	-0,577156257	1,228902427	-0,469651817	0,638603798	NA

Trim32	168,4392293	0,084944971	0,120824311	0,70304536	0,482027482	0,793477208
Loxl4	401,9353787	0,078760374	0,417932202	0,188452513	0,850521938	0,955320139
Sfxn2	1281,931389	0,175596091	0,107615637	1,631696811	0,102743372	0,398985659
Fancg	63,66947023	-0,509463178	0,210595357	-2,419156749	0,015556535	0,136720216
Necab2	2,91202694	-1,069502033	0,903213889	-1,184107161	0,236370646	NA
S1pr5	162,496391	-0,209340969	0,269748883	-0,776058704	0,437714325	0,766215379
Rnf8	36,49611997	-0,140496992	0,242009623	-0,580542997	0,5615485	0,836472635
Rheb	878,6183488	0,028202058	0,094723929	0,29772897	0,765910031	0,926898815
Ddx10	335,9633502	0,019929731	0,12097342	0,164744714	0,869144921	0,961775826
Prdm8	0,113662148	0,780932884	3,352475198	0,232942181	0,815806304	NA
Dynlt1a	20,58545308	0,618819112	0,329194681	1,879796813	0,060135775	0,296991199
Dynlt1f	2,228535369	-0,678108787	1,145544653	-0,591953168	0,553881949	NA
D2hgdh	657,3156252	-0,22742548	0,078827774	-2,88509327	0,003912977	0,0583195
Sgcd	0,656785137	0,921446593	1,846869217	0,498923576	0,617833225	NA
Acvr2a	487,3903041	0,038079105	0,129547707	0,293938858	0,768804624	0,927598042
Rpph1	41,66481996	-0,107246809	0,360047857	-0,297868206	0,765803755	0,926898815
Cep152	74,09918524	0,143059476	0,264399167	0,541073854	0,588456679	0,848843045
Rasgrp4	10,37411411	-0,612723316	0,49568064	-1,236125171	0,21641204	0,569754344
Acp2	1659,190372	0,033684891	0,096253349	0,349960717	0,726368179	0,910393262
Pfn1	3735,50411	-0,113236659	0,09910922	-1,142544144	0,253227908	0,612785071
Bckdhb	1737,719653	0,497745841	0,081237737	6,127027406	8,95E-10	4,28E-07
Cd300ld3	0,510436344	-0,55396608	1,901650028	-0,291308112	0,770815684	NA
Rundc3a	40,46687227	0,059823357	0,266744597	0,224272049	0,822545624	0,946967955
AY358078	0,394221993	-1,149575818	2,531438941	-0,454119513	0,64974281	NA
Mfsd14b	677,3217206	-0,00182272	0,127445654	-0,014301943	0,98858909	0,996575327
Ube2e2	357,7675588	0,388954265	0,222463031	1,748399554	0,080394865	0,350731846
Myrfl	0,213661134	0,059593471	3,352475198	0,017775962	0,985817581	NA
Bscl2	939,3626247	-0,026323601	0,095416837	-0,275880039	0,782640206	0,93249269
Pabpc1	6216,159644	-0,060615641	0,066423898	-0,912557718	0,361475201	0,710984546
Gnb1	1816,688326	-0,12000333	0,06604419	-1,817015691	0,069214711	0,321794167
Mär-05	2035,070213	0,123097982	0,078596012	1,566211548	0,1172991	0,424748556
Polr2f	492,9539721	0,077554462	0,109134346	0,710632951	0,477311718	0,790043378
Spef1	30,68299476	-0,062480638	0,277411044	-0,225227653	0,821802178	0,946779723
4931423N10Rik	0,302276418	0,61733957	2,745727361	0,224836442	0,822106515	NA
Abl1	618,3665007	-0,113233638	0,120297485	-0,941280181	0,346561296	0,698497639
Spo11	0,827055305	2,290706752	1,853472546	1,235900018	0,216495731	NA
Rusc1	176,4987106	-0,39427293	0,156091016	-2,525916871	0,011539678	0,115044027
Slc39a8	651,3984063	0,05763227	0,097177698	0,59306066	0,553140557	0,831358753
Snord47	2,763669609	-0,167473245	0,956555583	-0,17507947	0,861017189	NA
Spata31d1b	0,12663974	0,780932884	3,352475198	0,232942181	0,815806304	NA
Abhd4	1204,106834	0,141690051	0,097346114	1,455528578	0,145522985	0,474395704
Oard1	560,5739046	-0,127442767	0,113821496	-1,119672226	0,262853465	0,622953609
Exoc3l4	39,97134731	0,289355297	0,287605704	1,006083305	0,314375499	0,673320811
Ms4a7	19,04802126	0,505797459	0,442101743	1,144074789	0,252592622	0,612043137
Pitpnm2	814,3225144	-0,454746844	0,155409579	-2,926118488	0,003432202	0,05417368
Nup88	820,8512019	-0,084179029	0,082701546	-1,017865243	0,308742001	0,667567818
Stard7	2653,219883	0,189753622	0,076937085	2,466347959	0,013649866	0,126725738
Wnt9a	15,97076874	-0,116794071	0,426480863	-0,273855362	0,784195776	0,933246486
Rabl2	80,41080241	-0,013762405	0,174005326	-0,079091858	0,93695956	0,982210769
Gtf2f2	211,8675953	-0,061220672	0,184403061	-0,331993796	0,739893944	0,916128178
Ddah1	3829,914688	0,233910476	0,150106687	1,558294845	0,11916337	0,429289976
Ppp6r2	512,0858976	-0,043326925	0,134394222	-0,32238681	0,747159675	0,91954512
Slc25a33	379,8924455	-0,59898333	0,205389344	-2,916331096	0,003541744	0,055379625
Mus81	233,1605719	0,126306486	0,130628527	0,966913504	0,333587275	0,690201449
Ddb2	118,3666354	0,059753117	0,159482713	0,374668304	0,707907168	0,902731692
Drosha	315,2210778	-0,093160924	0,144470107	-0,644845679	0,519027197	0,813725618
Mir7061	0,209517726	0,059593471	3,352475198	0,017775962	0,985817581	NA
Mir680-2	0,300268898	-0,658045551	3,302424069	-0,199261372	0,842058294	NA
Sf3a2	390,780617	0,03711887	0,125745269	0,295190985	0,767847988	0,927586634
Gpc1	106,4532635	-0,224224412	0,331603694	-0,67618189	0,498925169	0,802566549
Ryk	896,4068912	0,040521783	0,095060262	0,426274686	0,669907701	0,887039558
Mlkl	135,5160556	0,636248924	0,21368208	2,97754928	0,002905629	0,048005373
Tnfrsf9	0,57441571	-0,982780737	1,817324398	-0,540784429	0,588656176	NA
Ak5	2,316159664	0,940237023	1,041794606	0,902516693	0,366782458	NA
Fam46c	76,20166777	0,399087627	0,315692795	1,264164511	0,206170986	0,558880735
Pogk	147,9606672	-0,189032129	0,185123447	-1,021113922	0,307200468	0,66646434

Supplementary Table S1: *Serpina1* KO vs. wildtype all DEGs

Zfp949	157,5601363	-0,027989696	0,203344577	-0,137646633	0,890519698	0,968877905
Gm20604	1,090453695	-0,154492706	1,452951769	-0,106330237	0,915320342	NA
AK010878	67,21769739	-0,26756553	0,239013959	-1,119455662	0,262945796	0,622953609
Moap1	1,266766108	-1,101144242	1,38329957	-0,796027315	0,426016159	NA
Fam102b	30,49204501	-0,217309221	0,377673841	-0,575388596	0,565028515	0,83747956
Tyr	0,113662148	0,780932884	3,352475198	0,232942181	0,815806304	NA
Limch1	11,51118192	0,669063058	0,469877303	1,423910145	0,154472485	0,489429924
Rfc1	575,5638122	-0,032315729	0,121318159	-0,266371739	0,789952928	0,935501878
Sox5	338,6203714	-0,137350791	0,205637523	-0,667926697	0,504180385	0,804997854
Sucla2	3536,632577	-0,009375339	0,071543738	-0,13104346	0,895740928	0,970827764
Pcbp1	3721,963704	-0,092170084	0,105498737	-0,873660541	0,382303146	0,726783025
Mirlet7a-1	0,173498105	0,059593471	3,352475198	0,017775962	0,985817581	NA
Csmd1	0,64550001	2,819307148	2,689604207	1,048223802	0,294535507	NA
Ces1d	25344,1101	-0,513581748	0,159023883	-3,229588779	0,001239684	0,027761028
Pip4k2c	555,826012	-0,001257008	0,105999156	-0,01185866	0,99053838	0,99719344
Gopc	821,0957344	-0,143429241	0,131190448	-1,093290281	0,274266369	0,635693562
Steap4	2331,165155	0,125812154	0,168471591	0,746785576	0,455193004	0,777281421
Cdk9	760,4113063	0,070731353	0,105645413	0,669516554	0,503166024	0,804644327
Trim33	408,8779403	-0,070784095	0,110123974	-0,642767346	0,520375074	0,814243255
Trim11	379,986656	0,031179717	0,138793505	0,22464824	0,822252934	0,946779723
Wrb	70,5863502	0,25180403	0,220698556	1,140940994	0,253894479	0,613689258
Mrpl3	490,5007833	0,190233436	0,085569178	2,22315371	0,026205437	0,186426396
Mrpl1	722,688601	-0,103256255	0,088370371	-1,168448816	0,242625774	0,600901069
Scgb3a1	1,35367089	0,680336467	1,464464459	0,464563317	0,642244207	NA
Gpha2	0,093303375	-0,517475177	3,352475198	-0,154356154	0,877328924	NA
Smarca5	823,7397821	0,001740692	0,129434693	0,013448416	0,98927004	0,996575327
Tchp	54,86102487	-0,059331289	0,221730412	-0,267583001	0,789020317	0,935225873
Col4a6	3,108792618	0,067942393	0,860107119	0,078992944	0,937038236	NA
Tfpi	117,4887361	-0,146565665	0,234170598	-0,625892686	0,531385332	0,819884984
Ppp1r3a	0,300137846	0,617320176	2,751219827	0,224380535	0,822461216	NA
Pofut1	395,0989961	-0,229484645	0,115230766	-1,991522341	0,046423492	0,256037813
Il1rl2	2,84853814	-0,733293819	0,928329598	-0,789906754	0,429582227	NA
Zfp358	252,8534367	-0,022807532	0,135891521	-0,167836317	0,866712051	0,961775826
Strc	0,266906519	0,586045239	3,35071904	0,174901337	0,86115716	NA
Tmprss3	0,172953423	1,307385949	3,350719078	0,39018071	0,696402925	NA
Eif3h	2805,509504	0,047328744	0,091085115	0,519610081	0,603335371	0,855263553
Mrps6	258,1403095	-0,262649821	0,166690389	-1,575674653	0,115100802	0,420121786
Tshz2	316,1537266	0,222173809	0,13630704	1,629951097	0,103111833	0,399438862
Gjc2	14,95818551	-0,55350451	0,371894681	-1,488336722	0,136662115	0,459349518
Usp3	694,4191006	-0,075755382	0,09310375	-0,813666282	0,415836156	0,751551608
Tmem45b	1,407016328	0,764846077	1,405247343	0,544278614	0,586249761	NA
Med17	308,2898482	0,154002834	0,120643102	1,276515864	0,201773222	0,553126746
Trerf1	15,00525344	0,039406852	0,359654066	0,109568766	0,912751382	0,97607806
Nae1	557,3603103	0,065638377	0,092196452	0,711940381	0,476501695	0,789577814
Ces1f	14538,0895	-0,411870629	0,131566523	-3,130512374	0,001745016	0,034871637
Mbip	89,91434144	-0,032791568	0,215094405	-0,152451982	0,878830461	0,965334355
Cd300ld	224,1412327	0,175512585	0,234886033	0,747224441	0,454928093	0,77694605
Tnrc6a	1060,385117	-0,055239803	0,129563108	-0,426354417	0,669849611	0,887039558
Blvrb	2915,549933	-0,21751588	0,160257839	-1,357286987	0,17469004	0,517225036
Plekha5	593,7603144	-0,068013427	0,185146196	-0,367349849	0,713358075	0,905933635
Gm5801	0,722138683	-0,437660347	1,618253396	-0,270452296	0,786812312	NA
Card9	11,99184012	0,360240909	0,490828547	0,733944493	0,462982569	0,781516914
Uvssa	259,7705659	0,097414468	0,168869808	0,576861366	0,564033111	0,837355044
Zfp280d	217,8120181	0,109921936	0,11720697	0,93784471	0,34832422	0,700473301
Scgb1c1	0,782680674	-0,799418327	1,7113561	-0,467125648	0,640409985	NA
Ebf4	8,033964224	0,557857246	0,554803342	1,00550448	0,314653993	0,673488511
Arfrp1	358,4711583	0,085592072	0,108032787	0,792278667	0,428198208	0,760002028
Mical1	36,44287004	0,4417657	0,274000534	1,612280431	0,10690093	0,405895634
Spag7	795,7517018	0,04478773	0,087682607	0,510793776	0,609495468	0,857753956
Ckap5	362,6015133	-0,092047603	0,13720282	-0,670887105	0,502292452	0,804113015
5430427O19Rik	9,999733369	0,302244866	0,44420561	0,680416588	0,496240721	0,800192636
Pgam5	368,5407308	-0,251977691	0,131515804	-1,915949896	0,055371474	0,284497014
Sesn3	142,7454751	-0,038383861	0,25598686	-0,149944655	0,88080828	0,965523606
Iffo2	369,9843932	0,17847063	0,181718511	0,982126858	0,326037355	0,683635102
Cyp1a1	13,60429645	0,180185076	0,490127687	0,367628846	0,713150003	0,905933635
Ccdc15	18,38354344	0,389842137	0,388177179	1,00428917	0,315239252	0,673985857

Fam184a	25,87646449	-1,67486076	0,543706272	-3,080451425	0,002066871	0,038918301
Frrs1	380,479256	-0,286838737	0,152172011	-1,884963831	0,059434721	0,294826972
Zkscan5	346,5458734	-0,04459725	0,14218906	-0,313647547	0,753788743	0,921825989
Taf9b	14,25817548	0,504753841	0,463329388	1,089406056	0,275974862	0,637060499
Hoxb6	1,660270835	-1,174933651	1,238975399	-0,948310719	0,342971296	NA
Hoxb5	10,67981162	-0,000537601	0,533184795	-0,001008283	0,999195507	0,999803624
Nudt1	467,8977914	0,11490727	0,133420513	0,86124141	0,389105097	0,73293034
Hsd17b4	10965,63423	-0,115261659	0,136965685	-0,841536757	0,400047302	0,740788928
Hsp90ab1	31258,05975	-0,357630378	0,117549468	-3,042381937	0,002347139	0,04234669
Hspa4	3417,307433	-0,159979388	0,084692432	-1,888945495	0,058899133	0,293376491
Nfic	3592,735104	-0,398429842	0,127149645	-3,133550572	0,001727052	0,034651586
Limk2	198,0748874	0,338236193	0,160002766	2,113939664	0,034520421	0,216973911
Neo1	444,0899608	-0,246035336	0,117301313	-2,097464459	0,03595248	0,221906144
Zhx1	991,510359	-0,048426942	0,104705579	-0,462505844	0,643718612	0,873795284
Taf12	395,3111829	-0,060307809	0,129505279	-0,465678386	0,641445726	0,87193573
Taf13	305,3590167	-0,264693572	0,129998866	-2,036122155	0,041738091	0,241243637
Stag2	1196,796297	-0,088086915	0,114315268	-0,77056125	0,440967037	0,768480766
Serpinb1a	119,222666	2,038521121	0,385369902	5,28977772	1,22E-07	2,64E-05
Cox7b	5127,32605	0,189963557	0,084264257	2,254378826	0,024172346	0,176590217
Eid1	202,6406941	0,34479423	0,292287791	1,179639521	0,238143617	0,595289658
Cul7	173,1745204	-0,242146875	0,207315005	-1,168014225	0,242801027	0,601014405
Ska2	29,5316205	-0,083467	0,333369907	-0,250373529	0,802298499	0,939799232
Wbscr22	680,5923926	-0,033087215	0,09570983	-0,345703414	0,729565618	0,911594858
Usp38	475,0926911	0,109090535	0,104032301	1,048621764	0,294352235	0,655361522
C2cd2l	897,9009416	-0,53717752	0,108450351	-4,95321145	7,30E-07	0,000101133
Aldoa	2375,792351	0,008760559	0,157394225	0,055659975	0,955612686	0,987294126
Serpinb2	0,43629734	2,237934017	2,448793276	0,913892585	0,36077329	NA
Abi2	42,28980211	0,42257541	0,465837711	0,9071301	0,364338002	0,713048566
Tnfaip8l2	46,7883622	0,656441151	0,300329806	2,185734274	0,028835052	0,195911301
4930404N11Rik	1,365033105	1,521886276	1,625763054	0,936105832	0,349218701	NA
Scn2b	1,202460328	0,847844826	1,562898791	0,542482233	0,587486343	NA
6720483E21Rik	0,213116452	1,337107854	3,350237755	0,399108348	0,689813372	NA
Hist1h2ak	0,093953095	-0,517475177	3,352475198	-0,154356154	0,877328924	NA
Hist1h2ai	0,180974489	-1,204797453	3,342691436	-0,360427361	0,718527568	NA
Cldnd2	0,889272471	-1,808867487	1,846106362	-0,979828424	0,327170819	NA
Runx1	11,83748858	0,076362333	0,412006508	0,18534254	0,852960374	0,956316741
Usp20	172,0796312	-0,079895908	0,1305643	-0,611927673	0,540585612	0,826407188
Zfp963	108,9128597	-0,22111391	0,193780178	-1,141055356	0,253846888	0,61368518
Msrb2	304,4890458	0,155494847	0,130555297	1,191026725	0,233643094	0,590802822
Pcolce2	41,65312865	-0,082984932	0,345038458	-0,240509224	0,809935513	0,942417935
Chmp5	1157,111513	-0,098303617	0,085093529	-1,155241986	0,247991346	0,607129663
BC053393	0,113662148	0,780932884	3,352475198	0,232942181	0,815806304	NA
Ggnbp1	263,0231378	0,05099861	0,208256032	0,244884194	0,806546101	0,941639579
Sall2	118,1195548	-0,408345411	0,172368544	-2,369025123	0,017835042	0,148003444
Cdc42ep1	744,5180127	0,065579848	0,165815746	0,395498311	0,692475168	0,895338108
Clec4b1	4,693306727	0,044213695	0,810817037	0,054529805	0,956513063	NA
1810046K07Rik	78,80877341	1,725272165	0,438673796	3,932927339	8,39E-05	0,00396963
Slc39a11	624,5509904	-0,065765021	0,109092674	-0,602836271	0,546617615	0,828173482
Tmem147	1812,995887	0,025760671	0,133465613	0,193013543	0,846948362	0,953824301
Med6	267,1026876	0,101252756	0,137008079	0,73902763	0,45989022	0,780953195
Ppp1r12a	407,750904	-0,110052486	0,11572332	-0,950996619	0,341606092	0,695250579
Anxa13	31,08212946	0,859552552	0,412852905	2,081982572	0,037344062	0,226546639
Synj2bp	1647,947244	-0,115652557	0,095413907	-1,212114254	0,225468648	0,580946341
1600002K03Rik	74,47410827	0,244472892	0,194668195	1,25584404	0,209172521	0,561671273
Xrcc6bp1	99,02726271	0,037915689	0,201250753	0,188400235	0,850562915	0,955320139
Mrpl12	2565,365041	0,03989483	0,091493014	0,436042366	0,662805992	0,883542125
Tm2d2	1305,905434	0,07136979	0,065497426	1,089657937	0,275863852	0,637060499
Slc35c1	536,4489123	-0,201561928	0,109121006	-1,847141406	0,064726649	0,309828757
Mgme1	239,2008897	-0,142789351	0,131764107	-1,083674107	0,27850932	0,638879736
Hspa13	932,4671779	-0,75676492	0,166136779	-4,555071576	5,24E-06	0,000479524
Rbsn	289,0384907	0,017891933	0,116961885	0,152972335	0,878420092	0,965046146
Acbd7	0,25327948	1,389394708	3,349408814	0,414817893	0,67827522	NA
Tardbp	2319,418252	-0,055472017	0,087939805	-0,630795319	0,528174365	0,81852028
Rab7b	26,90850746	0,406476675	0,438515457	0,926938078	0,353958685	0,704551502
Ppp4r4	165,6359925	0,193304559	0,213247322	0,906480591	0,364681534	0,713511989
Cyp2j9	225,9782644	-0,075893194	0,235896187	-0,321722853	0,747662664	0,919586841

Supplementary Table S1: *Serpina1* KO vs. wildtype all DEGs

Bcor	374,5716958	0,322638329	0,223995001	1,440381827	0,149759402	0,481899934
Plppr5	2,477047463	0,307613358	0,949947304	0,323821497	0,74607318	NA
Glrx5	1611,635128	0,08568645	0,142493772	0,601334704	0,547617079	0,828552821
Inpp4a	261,9411242	0,016634045	0,131377407	0,126612674	0,899246965	0,972027222
Klhdc8b	202,5537645	-0,085977505	0,22944364	-0,374721675	0,707867471	0,902731692
Gm5464	0,551957172	0,549626793	2,128277016	0,258249649	0,796214243	NA
Pak3	1,49090347	1,723600803	1,596790912	1,079415464	0,28040256	NA
Iba57	280,5574734	-0,226951812	0,1698196	-1,33642885	0,181409142	0,525744378
Dpf3	34,39817021	0,339720377	0,286937038	1,183954427	0,236431103	0,593697862
Zdhhc8	191,9584158	0,051752374	0,132536889	0,390475244	0,696185158	0,897385953
Sbsn	12,04712575	0,878028599	0,577422289	1,52060046	0,128360131	0,445494962
Sh3bgrl2	807,5826262	-0,388720045	0,122745202	-3,16688586	0,001540808	0,031936527
Nox1	0,172953423	1,307385949	3,350719078	0,39018071	0,696402925	NA
Hinfp	92,2664224	-0,125750547	0,179594937	-0,700189823	0,483808766	0,794055997
D130040H23Rik	3,418847262	0,460421447	0,781127933	0,589431548	0,555571819	NA
Zfp677	12,03634341	-0,180927668	0,429909887	-0,420850214	0,673864462	0,888626355
Lcor	62,33175678	-0,517565344	0,335755832	-1,541493237	0,123196788	0,43588421
Lcorl	184,4935381	-0,549542217	0,213573485	-2,573082595	0,010079715	0,105195714
Cep72	20,75178382	0,153857774	0,319482286	0,48158468	0,630101012	0,866281784
Fcf1	508,5039277	0,081292566	0,126204501	0,644133652	0,51948877	0,813866972
Sf3a3	475,2802333	-0,106492372	0,114490456	-0,930141916	0,352297611	0,703280089
Mir6937	0,249136072	1,389394708	3,349408814	0,414817893	0,67827522	NA
Gm1123	0,236587694	-1,308576888	3,335186821	-0,392354899	0,694796005	NA
Fam161a	47,92811343	0,148498544	0,261385443	0,568120941	0,569952849	0,841119289
4930415F15Rik	0,093953095	-0,517475177	3,352475198	-0,154356154	0,877328924	NA
Exoc6b	169,7948189	-0,132733909	0,140050103	-0,947760165	0,343251564	0,695819585
Fbxl13	1,29494284	-0,119913732	1,323968435	-0,090571443	0,927833123	NA
Samd5	2,687532464	-0,444395708	0,988511204	-0,449560618	0,65302729	NA
Kndc1	0,113662148	0,780932884	3,352475198	0,232942181	0,815806304	NA
Sycp2	0,267995883	-1,667347305	3,312696416	-0,503320285	0,614739107	NA
Svopl	0,142634598	-0,517475177	3,352475198	-0,154356154	0,877328924	NA
Tcp11l1	51,13866086	-0,159012053	0,254067258	-0,625865977	0,531402852	0,819884984
Mbtps1	1677,282015	-0,189399796	0,093480152	-2,026096352	0,042754912	0,243209687
Ifi213	9,487726915	0,137487666	0,533505133	0,25770636	0,796633537	0,937923049
Orc4	356,5610449	-0,149556261	0,14142102	-1,057524975	0,290272063	0,651880935
Add3	303,9044366	0,492563704	0,194029101	2,538607365	0,011129465	0,112719557
Dnase1l1	116,1976456	0,258311567	0,148860677	1,735257237	0,082695252	0,355711979
Polr3e	322,9357249	-0,016630556	0,134741468	-0,123425668	0,901770032	0,973023693
Abcc4	58,49037046	0,184854089	0,332924118	0,55524391	0,578727838	0,84530168
Gins1	69,63202867	0,416142963	0,23806635	1,748012532	0,080461857	0,350731846
Col11a2	24,9254659	-0,193097161	0,374489671	-0,51562747	0,606114621	0,856569383
Pxn	1109,03944	-0,006332475	0,119205525	-0,053122329	0,957634441	0,98781716
Pts	661,9497521	0,153457833	0,076829603	1,997378979	0,04578403	0,253872539
Klf6	223,2520006	0,03714486	0,203400468	0,182619342	0,855096707	0,957445239
Ptpro	13,56572989	0,405959388	0,414963418	0,978301632	0,327925163	0,685433319
Ptprn2	0,307614229	-0,630030301	2,72139296	-0,231510227	0,816918442	NA
Ptpru	15,29486363	-0,6290299	0,517835948	-1,214728145	0,224469794	0,579668007
Map3k4	642,9904363	-0,011587289	0,110734009	-0,104640739	0,916660887	0,976559041
Ptprf	4398,663377	-0,215233311	0,144007546	-1,494597449	0,135019532	0,457444533
Gnb2	2442,402597	-0,009463497	0,090272323	-0,104832766	0,91650851	0,976559041
Pthlh	0,206965523	0,059593471	3,352475198	0,017775962	0,985817581	NA
2410012E07Rik	0,12663974	0,780932884	3,352475198	0,232942181	0,815806304	NA
Gys1	83,08889535	-0,209337218	0,180866392	-1,157413577	0,24710343	0,606485359
Gm9199	0,093953095	-0,517475177	3,352475198	-0,154356154	0,877328924	NA
Polq	3,844817472	-1,153305162	0,735039716	-1,569037886	0,116639112	NA
Prrc2b	1341,003234	-0,203588966	0,092130871	-2,209780107	0,027120427	0,190527057
Plp1	27,72289879	-0,361836132	0,364612612	-0,992385123	0,321009693	0,678403192
Apoo	152,4084496	0,065389897	0,148843783	0,439318963	0,660430435	0,882607568
Eda	30,41094608	0,351599518	0,284220634	1,237065419	0,216062797	0,569610871
Lyplal1	411,3839594	0,278850853	0,120198912	2,319911624	0,020345659	0,159251235
Cnst	258,0072857	0,195893095	0,226333038	0,865508176	0,38675993	0,73103259
Aph1a	956,8045833	0,090112292	0,086014317	1,047642944	0,294803144	0,655777576
Thrap3	1233,670192	0,108844178	0,106301634	1,023918199	0,305873913	0,66482226
Plekha4	18,88277604	0,416211831	0,604367073	0,688673903	0,491028502	0,797734846
Ubxn1	3513,918418	-0,007995504	0,117094274	-0,068282618	0,94556066	0,984440501
Ehd2	86,27997593	0,048759282	0,197443352	0,246953272	0,804944387	0,940755443

Ipo13	346,7797151	-0,097169587	0,101835458	-0,95418225	0,339991398	0,694369844
Pax6	0,12663974	0,780932884	3,352475198	0,232942181	0,815806304	NA
Snord49b	0,481112335	-0,557238121	2,131855383	-0,261386455	0,793794502	NA
Nxt1	164,5479823	0,001130373	0,161782835	0,006986974	0,994425246	0,998458214
Ccr8	0,086476712	0,780932884	3,352475198	0,232942181	0,815806304	NA
Pou2f1	198,0236191	0,465317475	0,19601072	2,373938915	0,017599462	0,146719059
Cep135	101,3710866	0,38272464	0,189433346	2,020365723	0,043345466	0,245531991
Irgc1	0,213661134	0,059593471	3,352475198	0,017775962	0,985817581	NA
Snora28	25,47295194	-0,190889759	0,435435602	-0,438388037	0,661105017	0,882666274
Arid1b	676,4960982	-0,026698844	0,163976646	-0,162821014	0,870659362	0,961775826
Mir1839	1,869304782	0,021311481	0,996026408	0,021396502	0,982929364	NA
Mir1964	0,093953095	-0,517475177	3,352475198	-0,154356154	0,877328924	NA
Ost4	1425,622377	-0,181392337	0,093474524	-1,940553731	0,052312431	0,275992458
Spopl	99,15066384	-0,002718126	0,172498195	-0,015757418	0,987427919	0,99629614
Mcf2l	6,765423796	0,599357586	0,694844371	0,86257817	0,388369437	0,732211395
Oaf	5618,902336	0,029558464	0,115168452	0,256654177	0,797445748	0,938325224
Nrg1	6,387051645	0,303685706	0,617026324	0,492176256	0,622594748	0,863340855
Vkorc1	3532,704362	-0,088421069	0,08427889	-1,04914847	0,294109791	0,65521643
Gm13498	0,22911131	0,059593471	3,352475198	0,017775962	0,985817581	NA
2510009E07Rik	213,7852005	-0,173463863	0,156093134	-1,111284388	0,266445957	0,626660951
Tmf1	383,7140683	-0,091791371	0,132564831	-0,692426265	0,488669676	0,796400974
Pdlim7	147,3051657	-0,180920742	0,211657121	-0,85478221	0,392671728	0,735036172
Fkbp15	776,2401662	0,048200122	0,087029926	0,553833884	0,579692537	0,84530168
Rnf220	484,4986605	0,199543707	0,118364464	1,685841341	0,091826363	0,376111104
Mier3	225,1913516	0,002167414	0,197224823	0,010989558	0,991231777	0,99719344
Chn1	2,445174864	1,099789126	0,970791727	1,132878552	0,257265258	NA
Zfp608	67,69892024	0,341665995	0,396152457	0,862460876	0,388433954	0,73222977
Basp1	39,32280617	0,140373385	0,307924659	0,455869255	0,648483999	0,876446263
Pfkm	506,0586681	-0,121242285	0,112470914	-1,077987905	0,281039154	0,641764538
Akap17b	142,9691485	-0,700209684	0,180458488	-3,880170403	0,000104383	0,004682887
Setdb1	649,0716583	0,042305449	0,098162473	0,430973747	0,666487459	0,885184864
Mest	59,15443252	-0,082362717	0,401185113	-0,205298538	0,837338886	0,951141594
B4galt2	5,345609381	0,692139716	0,677282037	1,021937212	0,306810619	NA
Pdlim5	1149,572344	-0,10403751	0,104901138	-0,991767225	0,321311089	0,678746017
Kat2b	1658,081944	-0,031086639	0,124675252	-0,249340891	0,803097103	0,940189734
Kcne3	29,01972741	0,541368277	0,368517831	1,469042285	0,141821321	0,467919357
Cacybp	911,9633245	-0,459370152	0,150482877	-3,052640681	0,002268374	0,041521802
Dennd4c	489,6713305	-0,150342967	0,151284401	-0,993777061	0,320331416	0,677612453
Calu	2821,356511	-0,186615649	0,090440382	-2,063410672	0,039073627	0,232270039
Sbno2	563,1969591	-0,063850657	0,130506329	-0,489253339	0,624662351	0,864316628
Cyp2d12	5,96874378	0,529676105	0,61374094	0,863028798	0,388121634	0,731971786
Pde4a	152,5351243	-0,543539227	0,213325357	-2,547935395	0,010836254	0,110439837
Nup54	486,5004016	0,108644947	0,0973476	1,116051623	0,264400038	0,625289954
6430573F11Rik	147,5920895	0,528854223	0,19452571	2,718685475	0,00655419	0,081132374
Atp2c1	858,0783386	-0,270546815	0,116140735	-2,329473941	0,019833972	0,157271869
Auts2	7,133803145	0,560120522	0,670784525	0,835023023	0,403704746	0,743815979
Cc2d1b	349,4387277	-0,019291522	0,119580216	-0,161327041	0,871835827	0,962328069
Sep-09	2457,692758	-0,281396326	0,148642336	-1,893110222	0,058343213	0,291803151
4930524C18Rik	0,122496332	0,780932884	3,352475198	0,232942181	0,815806304	NA
Mir101b	0,421351117	-1,125417522	2,481765817	-0,453474503	0,650207099	NA
Mir17hg	80,14719634	-0,087362603	0,321072795	-0,272095938	0,785548253	0,933683966
Serpine3	0,290357571	0,059593159	3,288255867	0,01812303	0,985540706	NA
Stim2	481,1014037	-0,391349441	0,10990532	-3,560787074	0,000369745	0,011854001
Dnajb6	671,345482	-0,109216107	0,104083023	-1,049317212	0,294032147	0,65521643
Creb3l4	4,490736979	-0,040727889	0,677668621	-0,060100008	0,952075984	NA
Mir6386	0,093953095	-0,517475177	3,352475198	-0,154356154	0,877328924	NA
Tnfrsf23	12,5061887	0,5897424	0,429254325	1,373876433	0,169480047	0,509049371
Def6	6,74092321	0,968548666	0,545342891	1,776036107	0,075726966	0,340529367
Pin4	300,7455982	-0,168256956	0,132641932	-1,26850501	0,204617663	0,557589387
Slc35f2	6,558221901	0,320757019	0,605166204	0,530031282	0,596090242	0,853075785
Slc25a35	54,42480851	0,150458201	0,249666829	0,602635927	0,546750914	0,828173482
Tecr	6587,714755	0,12902727	0,092387605	1,396586377	0,162537989	0,499455681
Prdm5	2,813864937	0,684712793	0,854838872	0,800984625	0,423140547	NA
Mir3473e	0,576343886	0,3908496	2,109519946	0,185278931	0,853010263	NA
Mmp28	5,954963748	0,223308313	0,628190078	0,355478892	0,722230855	0,909644412
Ttc38	1886,565376	0,100099952	0,120055487	0,833780731	0,404404556	0,744376512

Naa20	321,82829	0,080469763	0,135159201	0,595370218	0,551596029	0,830638744
Zfp991	1,546542733	0,148393467	1,867584372	0,079457437	0,936668785	NA
Wfikkn1	5,785214224	-0,221814008	0,646043107	-0,343342426	0,731340867	0,912061199
Gltscr1l	352,3748099	-0,245539825	0,140180617	-1,75159612	0,07984328	0,349746007
Mipol1	44,6584134	0,393462555	0,23617254	1,665996204	0,095714171	0,385586553
Col7a1	15,4775335	-0,064904434	0,410820125	-0,157987473	0,87446667	0,963600184
Rem1	8,367670241	0,371989483	0,500025714	0,743940707	0,456912347	0,77843077
Rac1	3103,759089	0,041525922	0,079990912	0,519133002	0,603667996	0,855462946
Fancc	92,2938943	0,469723853	0,174564843	2,690827346	0,007127507	0,085467158
Erbb4	34,42632594	-0,297956509	0,371424113	-0,802200228	0,422437144	0,755522295
Gapdh	26597,80226	0,112558858	0,117769352	0,955756789	0,339195124	0,694071514
Ptgis	29,53395591	0,235414093	0,357480185	0,65853746	0,510192834	0,808610978
Ephx2	18636,05332	-0,323449007	0,150779603	-2,145177464	0,031938661	0,207676525
Epha8	0,093303375	-0,517475177	3,352475198	-0,154356154	0,877328924	NA
Stat5b	1614,080298	0,066053086	0,161927245	0,407918297	0,683333659	0,892293506
Stau1	2040,461587	0,089422125	0,086148596	1,037998638	0,299270689	0,659904651
Strn	165,6023407	-0,301586646	0,18832824	-1,601388331	0,109290936	0,409171591
Stat5a	406,1017253	-0,29506314	0,146790867	-2,010091952	0,044421458	0,249002293
Thap11	363,1988288	0,165036241	0,117614254	1,40319932	0,16055741	0,497218443
Cd82	2057,284442	0,162541359	0,119158537	1,364076491	0,172543493	0,514092703
Col4a4	5,801978855	0,403418314	0,607135036	0,664462253	0,5063945	0,806213623
Nrcam	0,530795117	0,495673827	2,137151084	0,231932047	0,816590793	NA
C1ra	8147,066526	-0,260519035	0,126844672	-2,05384294	0,039990895	0,235109182
Ifi30	578,6537415	0,023755465	0,150387879	0,157961302	0,874487293	0,963600184
Ssbp2	32,81832771	-0,183216555	0,286484046	-0,639534934	0,522474995	0,815322684
Gpr155	828,3334248	-0,208115813	0,105659258	-1,969688378	0,048874096	0,264749107
Cbfa2t2	243,8883544	0,018731367	0,1417508	0,132142935	0,894871236	0,970433882
Ddhd1	524,8831768	-0,387258339	0,126056759	-3,072094988	0,002125621	0,039633784
Fbln2	30,3971211	0,46764887	0,278901769	1,676751181	0,093591148	0,380194927
Atp8b4	65,86760373	0,075072661	0,303909002	0,247023487	0,804890047	0,940755443
Enkur	2,270968203	-0,06603658	0,945598642	-0,069835739	0,944324401	NA
Dym	761,2745418	-0,061650713	0,118171495	-0,521705452	0,601875428	0,854462419
Dlg5	130,6975084	0,012661429	0,195387338	0,064801688	0,948331897	0,985571619
Cage1	12,34357811	-0,129933489	0,41554073	-0,312685328	0,754519743	0,922298112
Nudt4	3601,420045	-0,016824126	0,125238041	-0,134337182	0,893135953	0,969576496
D630041G03Rik	0,627708644	-0,853717203	2,055374222	-0,415358524	0,677879464	NA
Tsen15	161,765835	-0,151585928	0,18594714	-0,815209787	0,414952237	0,751551608
Fopnl	388,210922	0,15585113	0,117743273	1,323652094	0,185618615	0,532518298
Ikbip	72,24115745	0,0083486	0,253857907	0,032886901	0,973764778	0,992775209
Cyc1	5350,526763	-0,001535825	0,094443945	-0,016261762	0,987025563	0,996190907
Hspbp1	440,4868321	-0,110156301	0,10758466	-1,023903414	0,305880898	0,66482226
Sharpin	783,3223268	-0,024733051	0,08641729	-0,28620489	0,774721199	0,929351416
Tmem42	150,4925196	-0,073014229	0,180558507	-0,404379887	0,685933388	0,893161227
Aurkaip1	1418,329475	0,02939099	0,114698516	0,256245598	0,797761202	0,938325224
Akr7a5	2850,395566	0,021426501	0,103874291	0,206273383	0,836577369	0,951141594
Chchd3	2463,939931	0,057644617	0,081096305	0,710816815	0,477197759	0,789952556
Tmem167	1071,059357	-0,221505313	0,123072866	-1,799789993	0,071893805	0,32893507
Txndc12	832,0430271	-0,205196957	0,079246238	-2,589358965	0,00961548	0,10216528
Sdhaf2	657,6391458	-0,045911117	0,102854387	-0,446370042	0,655329969	0,879460797
Gtpbp8	168,2219681	0,09401336	0,143578321	0,654787987	0,512604266	0,810335395
Gng11	141,5272909	0,209024754	0,220243551	0,949061859	0,342589152	0,695589013
Hsd17b14	0,347232576	0,667197765	3,168193746	0,210592476	0,833205284	NA
Haao	15470,91294	0,251951069	0,092365489	2,727761986	0,00637656	0,079745772
Them5	4,465419791	-0,650607845	1,038054653	-0,626756832	0,530818644	NA
Sdhc	3839,912934	0,104983289	0,07521574	1,395762232	0,162786107	0,499921685
Emc6	1512,043309	-0,090932798	0,134586745	-0,675644529	0,499266363	0,802825596
Tomm7	1122,867882	0,054438739	0,10051529	0,541596598	0,588096436	0,848706754
Ndufb5	2330,410943	0,03045478	0,11072214	0,275055922	0,783273275	0,93296333
Med21	420,8511149	-0,203521726	0,127977537	-1,590292576	0,111768871	0,413687165
Dtd1	257,5169602	0,071360165	0,130131356	0,548370254	0,583437697	0,846950064
Atp5d	7116,124869	0,046586053	0,093964226	0,49578499	0,620046116	0,862258786
Elp4	415,4095976	0,0272261	0,108991654	0,249799864	0,802742125	0,940105661
Ift46	603,3258819	-0,001840581	0,100117686	-0,018384174	0,985332377	0,995899882
Xpnpep2	0,12663974	0,780932884	3,352475198	0,232942181	0,815806304	NA
Gadd45a	98,8264489	-0,793945009	0,205575991	-3,862051233	0,000112439	0,004961045
Glrp1	0,88001475	2,366629728	1,662524558	1,42351565	0,154586729	NA

Glul	20501,52892	0,545099953	0,39784669	1,370125643	0,170647684	0,510601034
Gli3	7,836607253	-0,099830256	0,478519082	-0,208623356	0,834742277	0,950942904
Lag3	58,09939851	-0,347512641	0,299401979	-1,160689193	0,245768318	0,605051632
Edn1	10,43072834	-0,453965959	0,458168441	-0,990827649	0,321769746	0,678936195
Pip5k1b	0,452607907	1,213498532	2,500792722	0,485245547	0,627502177	NA
Anpep	3815,616292	-0,18169099	0,094735326	-1,917880039	0,055126224	0,284116184
Lamc2	21,61288572	0,818753701	0,428649268	1,910078382	0,056123122	0,286457769
Lamb2	119,1565426	0,194720099	0,18502302	1,052410121	0,292611427	0,654074594
Lhx6	169,2976904	-0,195172312	0,316148006	-0,617344752	0,537007354	0,823970082
Fam107b	1306,067782	-0,057909001	0,145110935	-0,399067109	0,689843756	0,894436573
Ccdc28b	89,95424694	-0,4131522	0,209773706	-1,96951376	0,048894124	0,264749107
Pomp	2636,120028	0,184977931	0,099289767	1,863011038	0,062460713	0,303271436
Naxd	1519,023062	0,067407326	0,068373005	0,985876313	0,324193812	0,681362532
Snx9	872,5245109	0,113954329	0,130011635	0,876493312	0,38076191	0,725866642
Pycrl	667,8007435	-0,209618472	0,13435749	-1,560154723	0,118723322	0,427935319
Pithd1	597,5449606	-0,146065322	0,093251774	-1,566354343	0,117265685	0,424742603
Fndc10	8,72266814	-0,088711135	0,505565454	-0,175469139	0,860711018	0,959574131
Fam193a	465,3179979	0,156893519	0,109158227	1,437303655	0,150631731	0,483083894
Ankrd49	160,7229679	0,219687271	0,160418817	1,369460734	0,170855301	0,510601034
Dynll1	496,0590739	-0,412838449	0,212700451	-1,940938285	0,052265763	0,275855109
Actl6a	493,0595766	-0,067640717	0,113498031	-0,595963792	0,551199416	0,830401734
Zfp109	60,86125146	-0,275625952	0,238017895	-1,158005166	0,246861928	0,606226509
Tmem267	355,656845	-1,709354035	0,783822794	-2,180791433	0,029198847	0,197151201
Mrpl15	1640,488861	0,182281388	0,087362414	2,086496699	0,036933654	0,225266506
Zfp433	16,56390297	0,036015372	0,426187986	0,084505836	0,932654263	0,981243003
Bcat2	427,8834579	0,097201059	0,159744995	0,608476398	0,542871555	0,826611597
Oip5	7,343416515	-0,317971012	0,647479886	-0,491090178	0,623362668	0,863525452
Gsta4	2163,277924	0,013325561	0,146991986	0,090655018	0,927766714	0,978977289
Lcat	10550,00253	0,181751239	0,065780795	2,762983293	0,00572757	0,074413878
Bmyc	89,67898957	0,085953101	0,279035144	0,308036828	0,758054309	0,923924043
Gm5803	0,087021394	-0,517475177	3,352475198	-0,154356154	0,877328924	NA
Hoxb8	1,202217966	0,349104261	1,371756048	0,254494421	0,799113614	NA
Mx1	41,21759045	0,440522039	0,312798573	1,408324965	0,159034867	0,495312554
Cd22	46,66720995	0,525578317	0,345999768	1,519013499	0,128759098	0,446231076
Mir7013	0,617271829	0,835728938	1,853154144	0,450976483	0,652006499	NA
Gm14322	38,66621419	0,250288629	0,255171582	0,98086404	0,326659793	0,684457358
Ubp1	1421,939265	-0,113612638	0,069781659	-1,628116042	0,103500283	0,400374791
Nlk	319,4035961	-0,196442374	0,117985602	-1,664969042	0,095918932	0,385586553
Gp5	1,886305977	-0,249267219	1,176587735	-0,211856041	0,832219347	NA
Rps6ka2	15,50216432	0,3314632	0,487220758	0,6803142	0,496305535	0,800192636
Golga3	619,9581966	-0,481284151	0,112149035	-4,291469393	1,77E-05	0,001289634
Mycn	18,87647856	-0,264877768	0,459010956	-0,577061973	0,563897591	0,837355044
Hhex	3095,905963	-0,279009744	0,172469093	-1,617737644	0,10571915	0,403240888
Pax1	0,607243516	2,687961657	2,697457556	0,996479686	0,319017133	NA
Dnajc30	543,9121638	0,21781793	0,131627961	1,654799853	0,097965104	0,389790319
H2-Oa	7,315521043	0,218680907	0,580540761	0,376684846	0,706407826	0,902122257
H2-M2	2,184828145	-1,121725098	1,122859282	-0,998989914	0,317799577	NA
Hspa5	31486,9589	-0,809349355	0,190027022	-4,259127713	2,05E-05	0,00140701
Pafah2	3020,4416	0,057525011	0,096690487	0,594939714	0,551883769	0,830638744
Cbx2	219,8468808	0,173439523	0,236624923	0,732972339	0,463575301	0,781663619
Rad51	13,14611419	-1,477141817	0,547281228	-2,699054421	0,00695368	0,084198069
Snord123	0,18725647	-1,238536202	3,340198538	-0,370797181	0,710788601	NA
Rtkn2	0,459568995	2,337243478	2,900421903	0,805828792	0,420341574	NA
Htra3	26,40303772	0,141820713	0,376593926	0,376587893	0,706479886	0,902122257
Lrrc74b	0,147722973	0,780932884	3,352475198	0,232942181	0,815806304	NA
Smim20	480,15973	0,060372206	0,113125742	0,53367346	0,593567474	0,851620901
Tshr	0,227324296	1,389394708	3,349408814	0,414817893	0,67827522	NA
Exog	57,60120594	-0,605635434	0,210558895	-2,876323193	0,004023375	0,059273033
Fam175b	710,3278693	-0,03301884	0,092174934	-0,358219299	0,720179206	0,908788588
Pik3c2g	105,0380264	0,421428906	0,154302832	2,731180637	0,006310787	0,079349252
Samd15	0,782105693	-1,570104891	1,674595033	-0,93760274	0,348448603	NA
Mllt10	627,0908829	-0,135809273	0,115364582	-1,17721809	0,23910847	0,596519262
Mir3058	0,982725921	-1,891951508	1,493829869	-1,266510697	0,205330302	NA
P4ha2	154,2803552	-0,542317449	0,168240729	-3,223461117	0,001266515	0,028033175
Ptger4	11,0314745	0,357394572	0,468068633	0,76355164	0,445134478	0,770855289
Sep-08	115,7926075	-0,302665616	0,232595646	-1,301252284	0,193172114	0,541635485

Alg11	561,6328275	-0,238383893	0,106782134	-2,232432371	0,0255864	0,183511044
Cyp8b1	13306,18112	0,613741888	0,115004361	5,33668361	9,47E-08	2,18E-05
Pcdh9	1,270005801	-0,749873247	1,49021006	-0,503199695	0,614823879	NA
Fbxo36	137,2236504	0,314263632	0,142411493	2,206729428	0,027332968	0,190773571
Prr13	1186,443572	-0,029366023	0,10881973	-0,26985936	0,787268453	0,934401226
Dnajc15	600,0228418	0,151758343	0,077284048	1,963643816	0,049571412	0,266959327
Tmem57	1011,854351	-0,221413071	0,092247784	-2,400199358	0,016386145	0,141515744
Zan	0,420626657	-0,031019798	2,228411788	-0,013920137	0,988893696	NA
Ppil2	1095,598277	-0,065043093	0,074003907	-0,878914303	0,379447743	0,725074694
Pi4k2a	2105,361132	0,136992505	0,085935166	1,594137903	0,110905151	0,412087539
Tbc1d10a	181,5014112	0,250596759	0,16203078	1,546599721	0,121959797	0,433849653
Fbxw11	741,3066514	-0,147247428	0,095277935	-1,545451508	0,12223709	0,434047185
Rgs5	906,5166472	0,069473331	0,216995525	0,320160202	0,748846891	0,919838327
Vsig10l	26,8806877	-0,254781126	0,29083627	-0,87602941	0,381014046	0,726126412
Cd207	104,2553957	-0,209015147	0,298946756	-0,699171819	0,48444466	0,79420977
Csad	12291,91261	-0,553635946	0,257407505	-2,15081509	0,031490798	0,206170652
Mast3	585,5116329	0,110259491	0,193533106	0,569719017	0,56886829	0,840725201
Wdtc1	2355,318633	-0,032745081	0,143162857	-0,228726092	0,819081814	0,945948926
Tmem39b	97,51660251	0,167925606	0,180461097	0,930536324	0,352093466	0,703082532
Zfp341	92,18555573	-0,006976248	0,271949544	-0,025652728	0,979534329	0,994664228
Fez2	775,4252146	-0,278767923	0,106133857	-2,626569236	0,008625044	0,096170324
H2afy2	15,03941833	0,349013812	0,432169135	0,80758616	0,419328856	0,753719022
Smim4	113,8137783	0,312516677	0,182190015	1,715333725	0,086284059	0,364119818
Asb6	191,6243556	-0,038833499	0,157083355	-0,247215875	0,80474116	0,94070615
Ing4	673,8110343	-0,146409864	0,125447689	-1,167098936	0,243170417	0,601527104
Krt23	37,54346439	-0,236377765	0,326374613	-0,724252916	0,468910477	0,783901984
Syt13	0,20013886	1,337107854	3,350237755	0,399108348	0,689813372	NA
Znrd1as	266,7952295	0,311177216	0,131580115	2,36492586	0,018033681	0,149006352
Ift43	58,35667346	0,174050123	0,239727475	0,726033269	0,467818376	0,782859415
Tmem234	653,29961	-0,072788059	0,127040582	-0,572951236	0,566677712	0,838321824
Cog2	425,8510525	0,037469465	0,104327009	0,359154022	0,71947987	0,908631658
Gper1	0,093953095	-0,517475177	3,352475198	-0,154356154	0,877328924	NA
Rnf114	1577,480236	0,024335726	0,06705913	0,362899526	0,716679941	0,907405448
Ppfia3	0,240301888	1,389394708	3,349408814	0,414817893	0,67827522	NA
Myo9a	117,3835787	0,243451123	0,213603219	1,139735271	0,254396608	0,614569616
Fsd2	3,524367647	1,176134881	0,881089418	1,334864383	0,181920737	NA
Cnnm1	7,006493687	0,2671394	0,535401884	0,498951177	0,61781378	0,861267197
Eprs	2984,535579	-0,085961567	0,107506713	-0,79959255	0,423946905	0,756607419
A930017M01Rik	0,415230487	-0,057645711	3,039527376	-0,018965354	0,984868744	NA
Wwc2	285,8406717	0,031182946	0,142350275	0,219057855	0,82660498	0,947537943
Nol12	293,6400372	-0,106794281	0,177936505	-0,600181961	0,548384974	0,828815941
Icmt	808,22075	-0,278650524	0,093365578	-2,984510253	0,002840327	0,047572443
Rhou	2215,64913	-0,109125068	0,088589883	-1,231800556	0,218023596	0,572041886
Unc45a	361,6581236	-0,185265754	0,124125661	-1,49256611	0,1355508	0,458151054
Smc4	249,5200219	0,071136021	0,16920734	0,420407417	0,674187851	0,888627551
Sarnp	753,3905066	-0,088244357	0,104104619	-0,847650731	0,396632513	0,738006967
Stt3b	2571,78596	-0,193331407	0,126977579	-1,522563343	0,127867987	0,445173728
Rab22a	430,063413	-0,005085686	0,1068108	-0,047613972	0,962023897	0,988526411
Ndufc2	1473,870914	0,176146275	0,092372898	1,906904278	0,056532984	0,287361327
Tspan13	14,75473033	0,019580638	0,485421074	0,040337429	0,967824114	0,991231429
Rpl24	3013,013326	0,067794881	0,115853152	0,585179425	0,558427078	0,83440808
Cmtm3	153,8256614	0,034704185	0,22353796	0,155249625	0,876624527	0,964416829
Pilrb2	53,70179242	0,266644254	0,259094376	1,029139493	0,30341413	0,663017899
Ssr2	2197,778877	-0,284517383	0,084698901	-3,359162631	0,00078179	0,020138259
Tomm20	812,1321227	-0,036634675	0,108491675	-0,337672687	0,73560986	0,914229638
Pkp4	1966,248159	-0,253160714	0,078964154	-3,206020714	0,001345843	0,028866309
Sf3b6	704,5006409	-0,091535493	0,090371026	-1,012885401	0,311114902	0,670529604
Tmsb10	210,6640393	0,306252759	0,203416647	1,505544228	0,132184215	0,45288846
Rpl4	14895,418	0,05897093	0,10282475	0,573509102	0,566300038	0,838041311
Slc25a11	1537,911136	0,023185781	0,092270064	0,251281726	0,801596302	0,939799232
Ppp6c	722,0701784	-0,087327371	0,094931207	-0,919901613	0,357624176	0,707149983
2310033P09Rik	151,2706705	0,052502177	0,143780874	0,365154111	0,714996371	0,906371815
Cstf1	464,1432735	0,08442187	0,09342822	0,903601394	0,366206802	0,71461374
Echdc3	2350,155228	0,324351738	0,144246185	2,248598383	0,024538059	0,178190829
Derl1	2790,103504	-0,073717852	0,073776655	-0,999202963	0,317696381	0,675759967
Ndufa10	3712,780014	0,049024935	0,092975431	0,527289142	0,597992813	0,853664273

Sec13	1405,398391	-0,108672247	0,116003023	-0,936805306	0,348858715	0,700915372
Jkamp	392,3439477	-0,075972053	0,131917186	-0,575907163	0,564677934	0,837355044
Cyb5r4	807,2302913	0,024870598	0,112732216	0,220616596	0,825390978	0,947537943
Pcyt2	12829,04639	0,083051186	0,087436528	0,949845425	0,342190801	0,695250579
Gdpd3	9,212539484	0,117871081	0,535643656	0,22005503	0,825828297	0,947537943
Mrpl28	988,8895995	-0,053375602	0,110755451	-0,481923024	0,62986063	0,866228449
Acat1	10808,45672	-0,064745576	0,091194547	-0,70997201	0,477721494	0,790092068
Zgpat	376,3504804	-0,028561969	0,095749875	-0,298297715	0,765475948	0,926709042
Galnt7	54,95602156	0,083591446	0,211801555	0,394668709	0,693087399	0,895773921
Fcgr4	141,9948573	0,455810317	0,257419747	1,770689014	0,07661243	0,342478335
Bivm	341,757046	-0,130100941	0,098421933	-1,321869399	0,186211644	0,532805747
Dlgap5	5,835594432	-0,946541278	0,724406346	-1,306644099	0,191333612	0,54044772
Stxbp6	316,107378	0,063107866	0,139982937	0,450825417	0,652115381	0,877662367
Sesn2	114,545	-0,090685069	0,215928686	-0,419976942	0,674502298	0,888627551
Amhr2	6,630411916	-0,459021041	0,531302196	-0,863954723	0,387612763	0,731859161
Zfp119a	85,28570535	-0,06080004	0,195033875	-0,311740921	0,755237426	0,922462845
2210407C18Rik	0,906376817	-0,925174206	2,185560303	-0,423312139	0,672067539	NA
Thyn1	423,401448	0,169574784	0,130308604	1,301332215	0,193144765	0,541635485
Slamf7	12,9419091	0,784468165	0,439159126	1,786295942	0,074051354	0,335476973
Rab3il1	116,6936629	0,007799821	0,168521624	0,046283797	0,963084053	0,988725638
Src	68,91738291	0,197471421	0,229869046	0,859060515	0,39030713	0,734026811
Timm17b	754,7359754	-0,044835326	0,099454486	-0,450812505	0,652124688	0,877662367
Ap5m1	248,2597285	-0,100394198	0,171755838	-0,584516945	0,558872568	0,834811996
Tmem38a	62,07508914	-0,220866648	0,218799979	-1,00944547	0,312761041	0,672219008
Ndufv1	3792,041923	-0,038605485	0,094251912	-0,40959896	0,68210016	0,891746239
Zc3h11a	1641,287039	-0,091223026	0,082244366	-1,109170512	0,267356631	0,627508918
Spry1	118,1723427	-0,246885944	0,174960197	-1,41109777	0,158215787	0,493856377
Scamp3	142,111141	-0,366179646	0,173187711	-2,114351206	0,034485282	0,216957051
Rnf126	617,4323955	-0,338126006	0,132454941	-2,55276249	0,010687235	0,10955746
Cep85	818,8789344	0,439260709	0,132392218	3,317874081	0,000907054	0,021941826
Zfp78	35,03348171	-0,107021617	0,277806154	-0,385238467	0,700060746	0,899228386
Gstm3	3392,369873	0,562434674	0,267732164	2,100736297	0,035664121	0,220786205
Mroh7	8,630581805	-0,0631351	0,508887219	-0,124065013	0,901263799	0,972555955
Ctbs	246,3370147	0,224105267	0,128579712	1,74292868	0,081346074	0,352860369
Nme7	139,3879336	-0,129208124	0,160339583	-0,805840467	0,420334841	0,75436738
Cdk5rap2	128,2004534	0,172354324	0,180470857	0,955025794	0,339564653	0,694134533
Mir6371	0,331469375	1,851207654	3,180323419	0,582081571	0,560511738	NA
Prkcsh	3016,793341	-0,165074959	0,082284713	-2,006143719	0,044840917	0,251037778
Dok1	34,1930331	0,23158851	0,280443584	0,825793576	0,408921199	0,746898413
Gpx1	47270,82505	0,062651621	0,112104161	0,558869717	0,576250638	0,844262442
Patl1	488,2662149	-0,091809628	0,120352108	-0,762841875	0,445557704	0,771326444
Map4k2	393,571446	-0,149385926	0,126372585	-1,182107072	0,237163219	0,594531234
Dsc1	0,142634598	-0,517475177	3,352475198	-0,154356154	0,877328924	NA
Ppa2	1097,673132	0,06953011	0,086434562	0,804424852	0,421151653	0,754607148
Tfdp1	469,3994949	-0,290078454	0,13133082	-2,208761453	0,027191237	0,190567596
Chrdl2	4,829892318	0,149039284	0,717210939	0,207803976	0,835382028	NA
Mir191	0,086476712	0,780932884	3,352475198	0,232942181	0,815806304	NA
Rbpj	255,9544191	0,20497446	0,135402012	1,513821382	0,130071148	0,4482911
Il9r	0,942921131	1,580408831	1,88019365	0,840556413	0,400596485	NA
Sephs1	679,167157	-0,064734592	0,119353937	-0,542375007	0,587560193	0,848648685
Rnf169	720,9926772	-0,043408784	0,105735495	-0,410541267	0,681408938	0,89166825
Lhfp	265,1329508	0,104068784	0,157994594	0,658685727	0,5100976	0,808610978
Cdca2	11,46455393	-0,419983423	0,526057811	-0,798359826	0,42466171	0,757334234
B4gat1	457,4783316	0,241557647	0,099048524	2,438780886	0,014736901	0,132226596
2700081O15Rik	45,06180695	-0,047972584	0,324658102	-0,147763398	0,882529496	0,966166219
Gpd1l	719,7107957	-0,07718914	0,128883159	-0,598907884	0,549234314	0,829403992
Ankhd1	917,4207458	-0,105450197	0,114644726	-0,91979981	0,357677383	0,707149983
Ccdc116	0,922097765	0,294663635	1,647551208	0,178849455	0,858055912	NA
Lyrm2	315,6783992	0,234813257	0,121079473	1,939331668	0,052460965	0,276416937
Card11	15,45145627	1,225140699	0,404901019	3,025778254	0,002479941	0,043424918
Fzd9	0,980165251	0,322989255	1,491731164	0,216519748	0,828582634	NA
Hist1h4n	0,52429263	-1,657341448	2,034774382	-0,814508705	0,415353587	NA
Hist1h4i	22,81806308	-0,051397615	0,376768564	-0,13641694	0,891491682	0,969104575
Cebpb	829,4807413	0,171517232	0,24103966	0,711572661	0,476729441	0,789764052
Phtf2	143,868078	-0,121279983	0,129176786	-0,938868251	0,347798391	0,699942299
Rbm48	100,0366026	0,032873042	0,247025783	0,133075349	0,894133791	0,970273198

Mfsd13a	156,853399	0,283731234	0,145906208	1,944613855	0,05182147	0,274159572
Sash3	39,10565202	0,444251953	0,268714	1,653251979	0,098279592	0,390573569
Fbxl4	422,8702183	0,492426147	0,212142866	2,321200593	0,02027602	0,159093803
Znfx1	936,6299883	-0,062929188	0,129983498	-0,484132131	0,628292099	0,865852703
Timm22	284,7123246	0,007171588	0,129837388	0,055235151	0,955951125	0,987414865
Rph3al	69,06194211	0,027095165	0,197740286	0,137024002	0,891011823	0,968922813
Tlcd2	3399,596534	0,349226776	0,222815846	1,567333661	0,117036721	0,424373183
Tomm34	258,0194814	0,069288632	0,115840468	0,598138394	0,549747594	0,82961684
Sf1	2195,273011	-0,165504039	0,08325906	-1,987820179	0,046831581	0,2575448
Tspan5	9,666741685	0,427996923	0,601114563	0,71200558	0,476461321	0,789577814
Gjd3	0,113662148	0,780932884	3,352475198	0,232942181	0,815806304	NA
Cenpw	22,77458096	-0,247405323	0,363286699	-0,681019492	0,495859157	0,80006558
Gm572	0,093303375	-0,517475177	3,352475198	-0,154356154	0,877328924	NA
Zfp672	321,2736409	0,288042155	0,149135216	1,931416082	0,053431618	0,278939777
AI837181	433,1918781	0,116318487	0,091445376	1,271999662	0,203373239	0,555983883
Zfp995	34,58200536	0,44552383	0,286468154	1,555229871	0,119891333	0,430708406
Rreb1	913,0724477	-0,676418068	0,19384764	-3,489431543	0,000484049	0,014316928
Ces3b	12837,26287	-0,484661028	0,22206946	-2,182474926	0,0290745	0,196651683
Krt14	0,635245829	-1,233208494	2,351809622	-0,524365783	0,600024151	NA
Crlf1	0,259974817	0,586045239	3,35071904	0,174901337	0,86115716	NA
Uso1	2697,434365	-0,456915068	0,12521793	-3,648958813	0,000263305	0,009196845
Ppie	293,2424557	0,138273239	0,12553262	1,101492502	0,270682366	0,631986125
Slc2a8	367,90205	-0,091990536	0,125771786	-0,731408365	0,464529757	0,781663619
Hps1	186,6033413	0,147670489	0,141528139	1,043400206	0,296762978	0,657874966
Bcam	99,62362279	0,450664994	0,238526867	1,889367847	0,058842556	0,293376491
Tspan6	7,961237798	0,017525881	0,588805564	0,029765142	0,976254359	0,993495054
Smad9	173,2279144	-0,160870565	0,224270146	-0,717307087	0,473184635	0,787502227
Tspan7	376,1269093	-0,042633136	0,304238927	-0,140130445	0,888556927	0,96836734
Hes6	2841,729879	-0,458750627	0,185428511	-2,474002642	0,013360871	0,124735672
Ebag9	469,9191285	0,114002408	0,106560531	1,069837081	0,284692648	0,645970131
Oas2	109,5455044	0,106898331	0,285372412	0,374592382	0,70796364	0,902731692
Oas3	43,38306011	0,21009652	0,325793407	0,644876525	0,519007205	0,813725618
Dars	1758,351018	0,056635637	0,10272935	0,551309212	0,581421724	0,845891913
Akr1d1	5715,882961	0,684340989	0,144714157	4,72891528	2,26E-06	0,000253587
Ahcyl1	2701,472641	0,067183114	0,0881982	0,761728856	0,446221849	0,771638844
Mpp4	34,60163919	-1,044865477	0,415018039	-2,517638704	0,011814444	0,116137723
Chst10	0,782949109	1,275072265	1,963472331	0,649396605	0,516082058	NA
Eif3l	2126,275654	-0,078796227	0,064552825	-1,220647218	0,22221962	0,577088987
Abcc10	240,089603	-0,139063377	0,195253916	-0,712218123	0,476329716	0,789577814
Nek9	1202,384549	-0,160626055	0,072036051	-2,22980094	0,025760661	0,184053149
Mgl2	2,98039788	0,656291663	0,889526241	0,737799103	0,460636539	NA
Spsb4	160,2311953	-0,733335877	0,22744095	-3,224291309	0,001262849	0,028033175
Tifa	389,4130266	0,521281081	0,178557952	2,919394376	0,003507122	0,055096025
Pitrm1	1094,878455	0,079018637	0,101946391	0,775099896	0,438280633	0,766215379
Lpcat3	3804,015966	0,281586725	0,111780094	2,519113327	0,011765078	0,116079212
Mir1949	0,556205619	1,581259037	2,074312959	0,76230495	0,44587802	NA
Pex1	851,8512548	-0,112250543	0,147512448	-0,760956408	0,446683105	0,77203703
Fam57a	35,91774881	-0,217094103	0,253281374	-0,857126205	0,39137514	0,734518115
Cpne3	1045,980178	-0,08372836	0,136058517	-0,61538492	0,538300548	0,824902774
Ago1	632,9856684	-0,039383331	0,125698226	-0,313316525	0,754040197	0,921880511
Trim26	1008,005587	0,043843753	0,111945571	0,391652416	0,695315054	0,897005399
Swi5	2551,657625	0,147631486	0,090475683	1,631725574	0,10273731	0,398985659
Pld6	19,8924296	0,402134998	0,336508359	1,195022317	0,232078311	0,58906385
Lrrn1	3,453540604	2,21771234	0,914090585	2,426140665	0,015260354	NA
Lrrfip1	886,5265716	0,021489581	0,132374794	0,162338918	0,871038969	0,961834188
Phox2a	0,086476712	0,780932884	3,352475198	0,232942181	0,815806304	NA
Mboat1	14,34369947	0,563057723	0,385259241	1,461503484	0,14387732	0,470983323
Lrrc45	252,529384	-0,165008936	0,12810541	-1,288071569	0,197721051	0,547387188
BC030867	3,799684207	-1,033473956	0,750440287	-1,377156816	0,16846377	NA
Aldh1l2	13,95978015	-0,422420521	0,481018063	-0,878180163	0,379845955	0,72534789
Lrrc20	176,9050995	0,171425067	0,191571416	0,894836354	0,370874573	0,718999735
Zbtb8b	2,463968916	0,31841918	0,99787073	0,319098627	0,749651725	NA
Gbp5	36,99026717	0,238896513	0,296853283	0,804762915	0,420956506	0,754607148
Nsl1	8,732860463	-0,805204379	0,572382723	-1,406758706	0,159498951	0,495312554
Sowaha	0,630144383	0,024928792	1,838840297	0,013556801	0,989183569	NA
Abcc3	5616,154756	0,002859762	0,091437227	0,031275689	0,975049678	0,993185764

Supplementary Table S1: *Serpina1* KO vs. wildtype all DEGs

C1qtnf3	3,468859993	-2,673367318	0,955032415	-2,799242492	0,005122265	NA
Prcc	523,1244003	-0,00503488	0,082958845	-0,060691302	0,951605059	0,985972877
Ankrd17	1194,396138	-0,035391426	0,12540664	-0,282213337	0,777779932	0,93073218
Erp44	2485,306549	-0,220080184	0,092049817	-2,390881275	0,016807987	0,143473504
Atp11b	1032,107441	0,021004505	0,118075616	0,177890287	0,858809139	0,958796604
Asb5	2,052043382	1,671396799	1,208245021	1,383326038	0,166564917	NA
Mfap4	42,50590786	0,62230776	0,512515806	1,214221597	0,224663116	0,579757618
Calr3	71,21075941	-0,110765096	0,196225879	-0,564477513	0,5724292	0,841794408
Fam20c	1402,668635	-0,144896758	0,120424301	-1,203218598	0,22889174	0,584649154
Tmem59	5110,855577	-0,038350249	0,063621928	-0,602783499	0,546652726	0,828173482
Clasp1	376,1232053	-0,316387037	0,096963222	-3,262959209	0,001102554	0,025552025
Cyp2d26	19878,53112	0,115275026	0,14148034	0,814777701	0,415199567	0,751551608
Ndfip2	691,1087393	0,088821435	0,096064823	0,924598948	0,355174561	0,705239841
BC004004	2839,37612	0,156088007	0,090189427	1,730668583	0,083510892	0,357477432
Sfi1	270,6745301	0,00970069	0,176676078	0,054906644	0,956212839	0,987532595
Car15	14,42377283	-0,381620312	0,465441466	-0,819910428	0,412267172	0,749265881
Mynn	196,5379728	0,17836299	0,13792603	1,293178596	0,195949297	0,54489515
Mnd1	106,7064554	0,080147876	0,182921712	0,438153982	0,661274665	0,882666274
Unc5b	99,83583623	-0,202898947	0,183741136	-1,104265225	0,269478103	0,629991376
Mtfr1	1770,452424	-0,054632898	0,097190587	-0,562121282	0,574033389	0,84275089
Hspb7	0,937354818	1,594004048	1,718906399	0,927336153	0,353752029	NA
Bcar3	1617,61419	-0,316032187	0,104337512	-3,028941194	0,002454124	0,043283892
Erdr1	220,7898243	-0,039415493	0,833099811	-0,04731185	0,962264684	0,988526411
Gpr137	517,4099463	-0,222082519	0,126645216	-1,753580018	0,079502497	0,349223642
Myc	373,8849198	-1,809448894	0,649711922	-2,785001831	0,005352742	0,070973656
Mir1191b	0,179780087	0,059593471	3,352475198	0,017775962	0,985817581	NA
Shisa6	0,670592227	0,894521322	1,710126214	0,523073276	0,600923262	NA
Mir8094	4,417186927	0,404949933	0,663665727	0,610171531	0,541748186	NA
Eif2b3	255,3636862	-0,025696651	0,181677087	-0,141441343	0,887521296	0,968356501
Pdik1l	211,6875657	-0,163586013	0,169917465	-0,962738078	0,335678983	0,692189898
Ccnf	505,8704247	-0,071728762	0,185506164	-0,386665114	0,699004146	0,898942204
Chic2	216,4203033	0,023550904	0,154732188	0,152204298	0,879025804	0,965347075
Kctd18	102,4645693	-0,046481095	0,175842952	-0,264333	0,791523334	0,936283441
Bag6	2019,938409	-0,103145355	0,093340861	-1,10503968	0,269142396	0,62982053
Gm10863	0,274382903	-0,66487896	3,339949097	-0,199068591	0,84220909	NA
Ddx39b	1246,608161	-0,109498827	0,142648885	-0,767610817	0,442718434	0,769450906
Irak2	591,2643169	-0,463922403	0,119948612	-3,867676305	0,000109877	0,004864075
Ano2	1,323697161	0,667595051	1,442571117	0,462781379	0,64352108	NA
Smim22	103,8241517	0,664372342	0,250102187	2,656403566	0,007897904	0,091080054
Aqp8	5622,594649	0,829491895	0,243810228	3,402203019	0,00066845	0,017837331
Npat	137,8419693	0,150647374	0,155880542	0,966428345	0,333829886	0,690434986
Man2a2	952,0726815	-0,280577431	0,101414739	-2,766633656	0,005663836	0,073801001
Hirip3	118,6180399	-0,519999748	0,203547423	-2,554685976	0,010628364	0,109274782
Exoc4	252,4475585	0,02166373	0,130339894	0,166209512	0,867992074	0,961775826
Tpd52	290,2267706	-0,257809833	0,124136195	-2,076830474	0,037817209	0,228252036
Lcn6	0,12663974	0,780932884	3,352475198	0,232942181	0,815806304	NA
Fam163a	1,949106423	1,600481681	1,11895626	1,430334445	0,152621053	NA
Tm2d3	517,2460993	-0,185255362	0,12200865	-1,51837892	0,128918903	0,446309134
Gm561	167,5724797	0,236823286	0,170358504	1,390146552	0,16448438	0,502924549
Wwp1	1956,171269	0,054360383	0,131231881	0,414231529	0,678704553	0,890709117
Mir93	0,596043625	-1,842827151	1,957141908	-0,941590972	0,346402094	NA
Mir27a	0,12663974	0,780932884	3,352475198	0,232942181	0,815806304	NA
D730005E14Rik	1,321440819	0,034532518	1,321422107	0,026132844	0,97915138	NA
Sbk2	0,093953095	-0,517475177	3,352475198	-0,154356154	0,877328924	NA
Fam46a	301,9900721	0,331910442	0,221219828	1,500364796	0,133519934	0,455016057
AF357359	0,510521193	2,408136785	2,087733944	1,153469192	0,248717856	NA
Hint1	4227,063356	0,065050315	0,119702372	0,543433795	0,586831162	0,848346794
Egr1	1097,407641	-0,197668402	0,880037396	-0,224613639	0,822279854	0,946779723
Fgl2	62,80158749	0,622683603	0,264613791	2,353178952	0,018613674	0,151643025
Plxna3	18,45432045	0,398602692	0,395377815	1,008156444	0,313379364	0,672227864
Egfr	9216,220373	0,264273634	0,467290627	0,56554448	0,571703479	0,841407954
Gjb4	0,086476712	0,780932884	3,352475198	0,232942181	0,815806304	NA
Ly6e	9079,868042	0,594942635	0,159990685	3,718607955	0,000200324	0,007483408
Magohb	52,17977076	-0,025108412	0,264654998	-0,094872238	0,924416308	0,977951927
Sri	830,4486649	-0,071592105	0,085610891	-0,83624997	0,403014292	0,742879955
Nkapl	7,875326953	0,406072264	0,6234243	0,651357774	0,514815562	0,812039132

Supplementary Table S1: *Serpina1* KO vs. wildtype all DEGs

Ndufs1	3484,746211	0,045941007	0,076460471	0,600846504	0,547942227	0,828763393
Ggta1	67,15144139	0,143729396	0,340850955	0,421678139	0,673259963	0,888626355
Pdcd11	532,0808144	-0,109959452	0,133301384	-0,824893547	0,40943203	0,746907036
Zfp472	55,22895354	0,083637552	0,198380131	0,421602463	0,673315208	0,888626355
Muc20	0,113662148	0,780932884	3,352475198	0,232942181	0,815806304	NA
Fanci	6,011982561	-0,305460419	0,989888726	-0,308580561	0,757640609	0,923804952
Zfp92	0,49288444	-1,484825691	2,430043457	-0,611028452	0,541180745	NA
Trpc6	0,147722973	0,780932884	3,352475198	0,232942181	0,815806304	NA
Gtf2a2	484,349256	0,117793985	0,101900042	1,15597583	0,247691044	0,607129386
Lrmp	55,140882	0,482008139	0,254424297	1,894505143	0,058157992	0,291422114
Invs	418,5326842	-0,464614249	0,179878057	-2,582940116	0,009796233	0,103285357
Deaf1	299,6327275	-0,153719496	0,097836449	-1,571188419	0,116138889	0,422607733
Cyth3	114,9476424	-0,05605585	0,233214679	-0,240361585	0,810049956	0,942417935
Mvk	784,1221175	0,546316442	0,129675026	4,212965745	2,52E-05	0,001643665
Myh15	0,122496332	0,780932884	3,352475198	0,232942181	0,815806304	NA
Golga5	644,1824684	-0,162576524	0,096183869	-1,690268082	0,090976679	0,374323747
Tcp1	2648,112736	-0,130429066	0,11167368	-1,167948127	0,242827689	0,601014405
Ppp2cb	656,1384578	0,104223199	0,107283012	0,971479057	0,331309777	0,688097004
Lekr1	4,512375854	0,124626181	0,651048399	0,191423834	0,848193549	NA
Tsc1	681,8404933	0,024116956	0,165878204	0,14538954	0,884403323	0,967085557
Sntb2	48,1605985	-0,429215531	0,242283519	-1,771542413	0,076470546	0,342478335
Mtx1	886,5256547	0,037261824	0,087730042	0,424732777	0,671031487	0,887361539
Tbrg3	0,634392829	0,868042419	1,849370291	0,469371885	0,638803841	NA
Dsp	1562,145771	-0,162130968	0,114755832	-1,412834239	0,157704469	0,493680148
Trim12a	197,1627956	0,733978931	0,202412951	3,626146094	0,000287683	0,009736783
Paqr4	40,03603185	-0,53807894	0,32106991	-1,67589339	0,093759077	0,380194927
Vta1	333,0778018	0,108765146	0,103925208	1,046571354	0,29529732	0,656439951
Rnu12	0,122496332	0,780932884	3,352475198	0,232942181	0,815806304	NA
Il5ra	0,650567465	-0,286039997	1,822564712	-0,156943671	0,875289242	NA
Ajuba	240,2858946	0,261826889	0,223418562	1,171911977	0,241232398	0,598879543
Lcp2	137,3955702	0,461410048	0,225559602	2,045623607	0,040793417	0,238151612
Lgals4	32,04204344	-0,162169427	0,346276553	-0,468323442	0,639553307	0,871049783
Arhgap5	1022,646387	0,009250273	0,173692234	0,05325669	0,957527388	0,98781716
Arg2	2,382667274	0,314687457	0,949080405	0,331570914	0,740213285	NA
Lgals3	119,1735501	0,563268129	0,230835943	2,440123159	0,014682255	0,131994726
Car14	1964,694495	0,141727371	0,158476747	0,894310197	0,371155945	0,719112906
Brd4	1070,695965	-0,016029089	0,072218405	-0,221952968	0,824350497	0,947473126
Capn5	101,9457514	-0,174948116	0,210742218	-0,8301522	0,406452737	0,745871812
Arhgap25	57,51306372	0,352174343	0,234801711	1,499879798	0,133645542	0,455328045
Foxp2	51,43496432	-0,114494703	0,226989119	-0,50440613	0,613976011	0,859682163
Srd5a2	31,54801469	-0,030486402	0,295541441	-0,103154408	0,917840424	0,976907366
Gmnn	127,1926699	-0,009557796	0,176274569	-0,054221074	0,956759031	0,987715172
Dnajc4	248,3790128	0,23715031	0,120031455	1,975734698	0,048184833	0,262717386
Zfp386	292,7876873	0,053233464	0,119078619	0,447044691	0,654842792	0,879151766
Sult3a1	287,1317165	10,22700645	2,938353488	3,480522849	NA	NA
Sult5a1	423,0374321	-0,302124092	0,314988232	-0,959159935	0,337478181	0,692850411
Ncoa4	1259,276202	-0,188912959	0,117110669	-1,61311485	0,106719558	0,405365909
Ttk	4,657972372	-1,295560238	1,019080498	-1,271303142	0,203620825	NA
Ldoc1l	10,70883598	0,160778363	0,566689102	0,28371529	0,776628576	0,930009104
Zfp746	411,4112604	0,204495513	0,110376238	1,852713201	0,063923477	0,307628856
Chm	326,9579203	0,132102905	0,202821494	0,651325967	0,51483609	0,812039132
Smarcal1	358,6117906	-0,011733683	0,110520234	-0,106167732	0,915449272	0,976559041
Atg4c	346,0134831	-0,16781686	0,135634757	-1,237270324	0,215986741	0,569610871
Dgcr2	1744,138809	0,046286129	0,090472564	0,511604034	0,608928162	0,857753956
Psmd7	2137,252876	0,082344209	0,086381035	0,953267219	0,340454696	0,694720147
Nras	414,2200303	0,054321394	0,103353384	0,525588929	0,599173851	0,85430922
Gm13271	0,122496332	0,780932884	3,352475198	0,232942181	0,815806304	NA
Klhl4	6,238266107	-0,258954988	0,668587311	-0,387316636	0,698521811	0,898800587
Hexdc	109,7688834	0,11348164	0,177749566	0,638435541	0,523190201	0,815322684
Pgam2	0,087021394	-0,517475177	3,352475198	-0,154356154	0,877328924	NA
Mia	3,971605924	-0,022534222	0,734740853	-0,030669619	0,975533021	NA
Cpxm2	5,380092927	0,602793349	0,919997098	0,655212228	0,512331122	0,810227859
Cxcl13	50,90100331	0,452490646	0,44495942	1,016925646	0,309188802	0,66810168
Zmiz1	1077,79705	-0,289998049	0,15039648	-1,928223639	0,053827315	0,280141127
Mir423	0,187906191	-1,241948083	3,339949325	-0,371846385	0,710007226	NA
Slc7a2	6969,081041	0,08420315	0,35844074	0,234915123	0,814274612	0,944076707

Supplementary Table S1: *Serpina1* KO vs. wildtype all DEGs

Catip	1,092338273	0,150355163	1,549292979	0,097047598	0,922688595	NA
Rnpc3	308,357759	0,169582133	0,115758789	1,464961179	0,14293151	0,470144068
Tmem35b	130,9809353	0,30085456	0,189884288	1,584409971	0,113100454	0,416310565
Tomm40	891,1873175	-0,075226176	0,106960174	-0,703310155	0,481862483	0,793477208
Aph1c	48,91292309	0,185830753	0,241503963	0,769472892	0,441612631	0,768930234
Whsc1l1	1256,195733	-0,058553704	0,140726809	-0,416080666	0,677350977	0,890086324
Sertad1	133,717108	-0,070703846	0,244595025	-0,289064938	0,772531684	0,928273961
Slc1a4	198,5746353	0,257376332	0,252585442	1,018967404	0,308218442	0,667160859
Mgst1	83080,64259	0,091814313	0,132687275	0,691960198	0,488962325	0,796400974
Papolb	0,411908937	-0,918926577	3,055071653	-0,300787242	0,763576737	NA
Herpud2	679,1607156	0,129964582	0,116776021	1,112938947	0,265734651	0,626006345
Limk1	64,62418542	-0,341689114	0,199999804	-1,708447241	0,087553388	0,366889174
Golga7	1148,661454	0,021165709	0,086638948	0,244297856	0,807000144	0,941758461
Lamtor3	309,647487	0,052550644	0,109826498	0,478487841	0,632303027	0,867306747
Isg20	87,97995449	-0,180720856	0,182531597	-0,990079849	0,322135092	0,679385399
Runx3	7,429170025	0,668276695	0,677109286	0,986955442	0,323664483	0,680937786
Atp5j2	3661,452546	0,200892477	0,12327096	1,629682097	0,103168703	0,399438862
Angptl4	998,9692157	0,422819082	0,640370525	0,660272553	0,50907894	0,807845752
Snx1	553,1193085	0,120033107	0,098571503	1,217726256	0,223328016	0,578394783
Rbck1	1093,752922	-0,095685415	0,081419384	-1,175216636	0,239908056	0,597490836
Dusp14	30,13278368	0,091337547	0,464586939	0,196599471	0,844140985	0,953068489
Copz1	4375,152567	-0,030890008	0,093426073	-0,330635842	0,74091957	0,916370654
B4galt3	250,9371452	-0,094617188	0,111045383	-0,852058723	0,3941815	0,736368577
Mpp5	344,1609903	-0,101026767	0,122845065	-0,822391743	0,410853977	0,748353623
Extl1	162,9514809	1,375451013	0,673435893	2,042437934	0,04110811	0,239153318
As3mt	1370,348431	0,493231165	0,130989776	3,76541727	0,000166271	0,006576566
Dclk1	1,027325217	1,756994873	1,844222771	0,952702082	0,340741038	NA
Mir7069	0,12663974	0,780932884	3,352475198	0,232942181	0,815806304	NA
Shtn1	1291,502892	-0,221451863	0,093887888	-2,358684033	0,018339864	0,150144298
Gripap1	613,4973927	-0,32911785	0,124512032	-2,643261406	0,008211161	0,093150496
Sptssb	2,12421064	1,26257818	1,084249656	1,164471829	0,244232853	NA
Tor1b	792,1681529	-0,323253908	0,102268399	-3,160838651	0,001573156	0,03250622
Edaradd	2,767601103	0,680128036	0,970940471	0,700483764	0,483625241	NA
Ehd4	206,2062843	-0,101693747	0,184280282	-0,551842802	0,58105606	0,845794369
Wwtr1	324,9636028	0,227471706	0,197851848	1,149707263	0,250264462	0,609210778
Rassf6	482,0881125	-0,091585919	0,143443034	-0,63848286	0,523159408	0,815322684
Ppp1r14c	0,086476712	0,780932884	3,352475198	0,232942181	0,815806304	NA
Dnah5	9,34506931	0,978518955	0,55827226	1,752762989	0,079642699	0,349439855
Cd79b	35,75126883	0,921534348	0,325535058	2,830829819	0,004642742	0,065335593
Cx3cl1	19,63301862	0,610908568	0,382760701	1,596058755	0,110475676	0,411062987
Lpin1	1291,656528	0,437831879	0,40933962	1,069605427	0,284796951	0,64598752
Card10	273,8811207	0,331747257	0,20409383	1,62546441	0,104063641	0,401498609
Brd3	838,4225782	-0,016729459	0,103159572	-0,162170689	0,871171441	0,961900479
Nadk2	4011,339243	-0,205613554	0,151288685	-1,35908085	0,17412097	0,517013028
Serinc3	4681,861231	-0,010934531	0,090939237	-0,120239969	0,904293057	0,973666426
Creb3	773,2574989	-0,142943178	0,089767944	-1,59236329	0,1113031	0,412992267
Map2k6	117,5004087	-0,405078125	0,30177007	-1,342340292	0,179485685	0,522135977
Ube2i	784,9676696	-0,007483532	0,075210553	-0,099501092	0,920740421	0,977341679
Cul1	1639,096189	0,15112749	0,073634608	2,052397566	0,040131041	0,235725785
Nr2c2	488,411553	-0,226284552	0,117073263	-1,932845688	0,05325521	0,278635557
Atp6v0d1	1806,043537	-0,134215549	0,082905922	-1,618889771	0,105470982	0,403046295
Txn1	5130,919889	-0,052955807	0,104648845	-0,506033359	0,612833232	0,85907177
Tulp3	29,23700597	0,044834724	0,302023216	0,148447939	0,881989269	0,96610403
Tuft1	154,0398393	0,128210321	0,31738379	0,40395989	0,686242214	0,893161227
Tubb5	2100,865008	-0,553354047	0,114670073	-4,825618694	1,40E-06	0,000171184
Tuba1b	336,5839069	-0,460082707	0,163103885	-2,820795512	0,004790473	0,066921454
Tuba1a	123,1949	-0,227689591	0,268131002	-0,849172941	0,395785068	0,737426273
Usf1	581,0783594	0,119152696	0,139850669	0,851999468	0,394214387	0,736368577
Trim7	262,3828954	-0,059577933	0,254545791	-0,234055858	0,814941615	0,944076707
Clec2h	260,6703676	-1,060002635	0,637438782	-1,662908917	0,096330669	0,385838157
Mrpl43	910,9840153	-0,006949433	0,10102255	-0,068790906	0,945156057	0,984402565
Mrpl36	582,6990258	-0,01558266	0,136045902	-0,114539724	0,908809959	0,9747592
Mrpl27	784,5256249	0,188207986	0,086539105	2,174831667	0,029642733	0,198245973
Fzd7	648,9417608	-0,900742351	0,248189306	-3,629255292	0,00028424	0,009669224
Atp2b4	112,1263111	-0,20682851	0,261820085	-0,789964259	0,429548642	0,760857915
Asxl1	450,9756986	0,084777362	0,10453367	0,811005312	0,41736262	0,752195715

Supplementary Table S1: *Serpina1* KO vs. wildtype all DEGs

Rpusd3	438,7450318	-0,140463405	0,10837437	-1,296094318	0,194942992	0,544089091
Selenoh	88,72623896	-0,016802679	0,223963342	-0,075024239	0,940195426	0,983128178
Plpp6	408,9046219	0,306147379	0,107331095	2,852364261	0,004339534	0,061982086
Csnk2b	2067,442508	-0,055438646	0,086151282	-0,64350344	0,519897483	0,813876985
Abcb1b	26,91636676	-1,180537361	0,366703423	-3,219324629	0,001284929	0,028346897
Taf15	1389,056485	-0,026716173	0,084319951	-0,316842847	0,75136285	0,920662578
Nbas	714,1866659	-0,360213342	0,183398431	-1,96410264	0,04951819	0,26689182
Oxsm	969,8357904	-0,02270187	0,100603486	-0,225656895	0,821468286	0,946779723
Osgin1	10968,84337	-0,645887838	0,453662535	-1,423718708	0,154527916	0,489429924
Rasip1	104,9257631	-0,167055044	0,278321467	-0,600223352	0,548357392	0,828815941
Phospho2	494,336271	-0,021969246	0,131382834	-0,167215501	0,867200488	0,961775826
1700003E16Rik	10,67574448	0,552072248	0,473052695	1,167041756	0,243193507	0,601527104
Zbtb43	561,9222333	-0,037465301	0,10548192	-0,355182206	0,722453094	0,909644412
Smg9	239,6250574	-0,036241279	0,104942195	-0,345345162	0,729834898	0,911594858
Prkcdbp	22,14640518	0,280027394	0,399325589	0,701250814	0,483146504	0,79399946
Ybx1	4991,206954	-0,074600941	0,076200143	-0,979013141	0,327573487	0,685077831
Gm12504	0,683713943	-2,213042255	1,670252836	-1,324974403	0,185179641	NA
E330033B04Rik	11,75048942	0,323752755	0,427389831	0,757511601	0,448743419	0,772999712
Dusp11	1049,504715	-0,164881026	0,087744538	-1,879103009	0,060230429	0,297348822
Nedd4	5852,575274	-0,116683023	0,117829994	-0,990265881	0,322044179	0,679300825
Ndufs6	2187,24512	-0,059407025	0,124240469	-0,478161627	0,632535172	0,867408217
Cfap53	0,589436672	0,778069469	1,859750479	0,418373044	0,675674392	NA
Stat6	1498,786107	-0,214534624	0,073677851	-2,911792653	0,003593611	0,055863936
Ghr	12991,68223	-0,106826796	0,094643913	-1,128723367	0,259014549	0,620344949
Klhl33	0,603063984	0,800819849	1,855472926	0,431598779	0,666033046	NA
Zfp280c	112,7818088	0,147508673	0,182565342	0,807977414	0,419103585	0,753719022
Rcbtb2	1019,545838	0,012612947	0,100731787	0,125213177	0,900354786	0,972376014
Fig4	405,5440732	0,041380905	0,151463269	0,273207526	0,784693695	0,933246486
Tbl3	503,4631154	-0,036487619	0,117296209	-0,311072446	0,755745548	0,922554343
Nr1d1	316,9128261	0,78940285	0,755686485	1,044616869	0,29620008	0,657387444
Ubl4a	399,0299303	-0,008133017	0,115960407	-0,070136151	0,944085293	0,983908567
Pccb	4447,835873	-0,035480675	0,07960747	-0,445695302	0,655817358	0,879592859
Prmt7	316,3138219	0,146568549	0,112843428	1,298866504	0,193989747	0,542789645
Ccdc25	1119,165945	-0,305988893	0,106704327	-2,867633407	0,004135544	0,060556505
Ythdf2	574,4740281	0,079441414	0,141301457	0,562212282	0,573971395	0,84275089
Tmem51	221,350195	0,443064963	0,186524451	2,375372029	0,01753127	0,146484721
Bag2	99,13701986	-0,239100634	0,242398254	-0,986395857	0,323938897	0,681035945
Prkag2	869,6689834	0,069355589	0,12767103	0,543236698	0,586966843	0,848346794
D230025D16Rik	1055,692585	0,041477564	0,200560471	0,206808271	0,836159597	0,951141594
Tnpo2	955,18979	-0,24293958	0,095964051	-2,531568607	0,01135536	0,113885033
Ginm1	805,9760415	0,024285214	0,130159955	0,18657977	0,851990129	0,95595696
Ube4a	1917,088552	-0,208020598	0,095921232	-2,168660609	0,03010846	0,200458168
BC016579	2,274482259	-0,480296182	1,049197136	-0,457774965	0,647114124	NA
Slc10a3	118,1545253	-0,144551681	0,193233151	-0,748068745	0,454418693	0,776572096
Slc44a3	236,7738996	0,277493538	0,133704286	2,075427402	0,037946941	0,228686669
Pqlc2	267,8675742	0,156844255	0,169386615	0,925954244	0,354469759	0,704662593
Rho	0,396774196	-1,13642979	2,184762982	-0,520161592	0,602950953	NA
Fam193b	1102,013182	-0,188927175	0,16374186	-1,153811094	0,248577625	0,60747894
Lactb2	6885,792735	-0,032369859	0,131882379	-0,245444912	0,806111957	0,941297123
Mrgprf	6,994391247	0,652107684	0,7749762	0,841455111	0,400093023	0,740788928
C1s1	14967,44219	-0,100938561	0,11606967	-0,8696377	0,384498428	0,729232443
Serpinh1	433,8772351	-0,493838195	0,21341377	-2,31399406	0,020668048	0,160578725
Arl14	0,633446739	0,525433419	1,89896331	0,276694877	0,782014406	NA
Sox15	0,652746767	1,870464138	1,981755774	0,943841902	0,345250445	NA
Fosl1	0,552993586	-1,86922309	2,821993063	-0,662376926	0,507729688	NA
Fos	32,39705339	-0,584983189	0,415766094	-1,40700071	0,159427178	0,495312554
Snora44	3,285388351	0,293990608	0,81103016	0,362490351	0,716985632	NA
Mir1291	2,117566766	0,824356254	1,008453572	0,817445916	0,413673645	NA
Snora2b	3,420761149	-0,214838695	0,845010535	-0,254243806	0,79930721	NA
Snora34	1,799467419	-1,336062172	1,093053962	-1,222320415	0,221586482	NA
Cep131	101,7968868	0,003458955	0,18096057	0,01911441	0,984749836	0,995554769
Lst1	53,48869514	0,347300397	0,266357599	1,303887696	0,192271876	0,541552905
Osmr	61,27714862	0,597878802	0,394462906	1,515678137	0,129600759	0,447477413
Ptpn18	47,97845203	0,454165807	0,278851349	1,628702205	0,103376076	0,40001006
Tspo	1057,18816	0,094884508	0,167810995	0,565424858	0,57178482	0,841407954
Man2b1	2662,548599	-0,039473308	0,100636937	-0,392234797	0,694884736	0,896796412

Supplementary Table S1: *Serpina1* KO vs. wildtype all DEGs

Mog	0,375775812	1,987694774	3,082915622	0,644745111	0,519092378	NA
Apln	0,79813085	-0,995006621	1,719425345	-0,578685561	0,56280136	NA
Atp6v0c	0,398931345	-0,92194011	3,084711936	-0,298873972	0,765036207	NA
Lox	6,354886692	0,384847012	0,567378622	0,678289588	0,497588099	0,801288247
Atp8a1	81,6466572	0,465262584	0,228978166	2,031908077	0,042162963	0,242539058
Nbea	23,07961868	0,015755842	0,368313359	0,042778363	0,965878212	0,990247379
Ssmem1	1,166814182	3,663279816	1,675960367	2,185779501	0,028831741	NA
Htatsf1	647,6700457	-0,10131437	0,092182267	-1,099065732	0,271739396	0,633595764
3300002I08Rik	4,280938523	0,227665175	0,721266853	0,315646246	0,752271031	NA
Sec62	3509,067933	-0,133407326	0,113975735	-1,170488842	0,241804302	0,599755419
Ptcd3	892,4996375	-0,097048832	0,099490776	-0,975455574	0,329334328	0,686456976
Exoc1	271,3330814	0,064867604	0,140335802	0,462231329	0,643915439	0,873795284
Brd9	208,6207281	-0,113583502	0,155575222	-0,730087354	0,465336791	0,781663619
Ints2	111,7891815	-0,52591977	0,187592831	-2,803517417	0,00505485	0,069596594
Afap1	27,4770367	0,501814845	0,347144165	1,445551721	0,148302976	0,478788333
Enho	728,5198437	-0,229025079	0,229256925	-0,998988706	0,317800162	0,675759967
Wdr77	725,307619	0,001564475	0,136965931	0,011422367	0,990886468	0,99719344
Dhx34	203,4540668	-0,050084026	0,147638146	-0,339234997	0,734432707	0,913675851
Mier2	242,0941318	-0,148856561	0,10884934	-1,367546748	0,171453995	0,511694109
Snx20	14,768347	0,091278587	0,412837915	0,221100301	0,825014336	0,947537943
Rufy2	115,2988342	0,137409425	0,211459514	0,649814343	0,515812153	0,813164632
Polr3b	378,064428	-0,028694964	0,161404629	-0,177782783	0,858893569	0,958796604
Leng9	90,72812956	-0,677689043	0,289548339	-2,340503989	0,019257733	0,154814569
Leng1	305,9060749	-0,161184374	0,127078451	-1,268384782	0,204660574	0,557592665
Vps50	220,2853886	-0,150858759	0,123025096	-1,226243783	0,22010695	0,574490867
Caskin1	3,917280839	0,998668251	0,902730143	1,106275512	0,268607288	NA
Zfp511	145,0160217	-0,13396923	0,159156139	-0,841747172	0,399929488	0,740788928
Nup37	112,3529888	0,289340343	0,161613569	1,790322096	0,073402146	0,333666539
Gemin7	398,0784093	0,080939831	0,135096411	0,599126436	0,549088574	0,829277581
Klhl30	0,267451201	-0,629501011	2,83229786	-0,222258054	0,824113003	NA
2310068J16Rik	8,749738879	0,404464261	0,554330603	0,729644474	0,46560753	0,781663619
Zfp551	25,51139049	-0,717769934	0,329733656	-2,176817325	0,0294942	0,197747221
Akap11	640,3337726	0,218547681	0,1753758	1,246167832	0,212702786	0,565782639
Wars	583,4637929	-0,377764064	0,126070656	-2,996447212	0,002731455	0,046341149
Gm10033	126,9309344	-0,480524021	0,19681767	-2,441467892	0,014627689	0,131688602
Elovl3	10626,77466	-2,019693815	0,945026782	-2,137181562	0,032583227	0,210234152
Rnf181	2858,759176	-0,038439895	0,104992099	-0,366121789	0,7142742	0,906371815
6720489N17Rik	6,705828174	0,702537813	0,583309638	1,20439946	0,228435221	0,584236803
Chd5	0,113662148	0,780932884	3,352475198	0,232942181	0,815806304	NA
Slc12a5	31,27652292	-0,196330937	0,320269013	-0,613018834	0,539863887	0,826407188
Pmm2	1318,293185	-0,003445243	0,107674177	-0,031996931	0,974474498	0,993185764
Xpo1	669,9709876	-0,008968858	0,12789543	-0,070126489	0,944092983	0,983908567
Stk17b	95,64640793	0,298229186	0,29936025	0,996221732	0,319142423	0,677025555
Trim65	87,06532543	-0,226852439	0,189328382	-1,198195624	0,230840869	0,587687111
Vstm4	44,79393638	-0,188059109	0,328170434	-0,573053176	0,56660869	0,838312481
Ccdc62	43,93261036	0,469176168	0,26601038	1,763751353	0,077773851	0,345490591
Bace1	514,5466117	-0,309599296	0,11089103	-2,791923708	0,005239571	0,070329139
Rhpn1	0,894022931	0,20160869	2,21073735	0,091195225	0,927337469	NA
Jph2	2,56131554	-1,199756276	1,155437535	-1,038356674	0,299104033	NA
Zhx2	495,3553658	-0,376018307	0,110490047	-3,403187144	0,000666046	0,017837331
Lzts1	1,544606473	1,132160847	1,434008731	0,789507638	0,429815367	NA
Rrp12	203,4331508	-0,498876473	0,265966293	-1,87571315	0,060694679	0,298538325
Phkb	564,7175767	-0,158849731	0,119812249	-1,325822129	0,184898621	0,531519026
Fignl2	205,342747	-0,121016796	0,191680814	-0,631345377	0,527814723	0,81852028
Eppk1	160,213304	-0,097881789	0,197866876	-0,494685067	0,620822444	0,862608071
Cdc42bpg	358,290184	-0,052914359	0,095893172	-0,551805284	0,581081767	0,845794369
Mir6975	0,087021394	-0,517475177	3,352475198	-0,154356154	0,877328924	NA
Bcl2l14	5,017318001	0,496488833	0,728821294	0,681221634	0,495731261	NA
Iqcd	5,322256677	-0,204200302	0,699984653	-0,291721114	0,770499865	NA
Pih1d1	700,2907999	0,026329145	0,087082339	0,302347705	0,762387015	0,925557106
Kars	1421,957132	0,015659264	0,073088835	0,214249737	0,830352312	0,948720627
Map3k8	47,45033193	-0,258268843	0,311804707	-0,82830322	0,4074988	0,746679655
Nucb1	4913,922539	-0,231209893	0,089998032	-2,569054998	0,010197627	0,106013192
Snx33	442,6222313	0,162959416	0,128293509	1,270207802	0,204010619	0,557184468
Zcchc11	432,2887465	0,300470834	0,199249834	1,508010458	0,13155185	0,451415988
Atp6v0d2	235,5747221	3,018565795	0,657001511	4,594457919	4,34E-06	0,000423393

Smc5	507,4927119	-0,333109454	0,140838363	-2,365189764	0,018020835	0,148992292
Gm12060	0,784274838	0,460434067	2,273179366	0,202550698	0,839486228	NA
Kbtbd8	37,363224	-0,278416427	0,442799095	-0,62876467	0,529503133	0,819035911
Rbak	87,63885341	0,214248204	0,161067479	1,330176681	0,183460065	0,529507257
Capn10	494,4062461	-0,005424593	0,0879715	-0,061663068	0,950831152	0,985972877
Sycp3	1,103647984	1,133506215	1,863884065	0,608142017	0,543093287	NA
Sycp1	0,627640905	0,846944621	1,849648581	0,457894883	0,647027963	NA
Vamp7	584,5714882	-0,238274122	0,205743061	-1,158114987	0,246817115	0,606226509
Surf4	6587,799845	-0,21195059	0,091534305	-2,315531768	0,020583848	0,160271092
Abcc9	498,4706645	0,120608817	0,160246813	0,752644096	0,451663812	0,774656859
Tdg	1,135482378	1,996182415	1,692632818	1,179335763	0,238264503	NA
Dnah2	4,40628536	1,633569404	0,817067038	1,999308903	0,045574941	NA
Elk3	180,9255342	-0,267396557	0,272678978	-0,980627693	0,326776373	0,684530449
Arhgap8	2,285445312	1,030896627	1,119201273	0,921100299	0,356998064	NA
Cdpf1	135,0132944	0,150800886	0,149214091	1,010634352	0,312191468	0,671711748
Zfp809	626,7709058	-0,479260466	0,274302546	-1,747196564	0,080603248	0,351004826
Ccdc159	159,7172266	-0,130994764	0,223828492	-0,585246151	0,558382217	0,83440808
Egf	10,50956525	0,236122672	0,451460361	0,523019722	0,600960529	0,854422974
Rps4x	7379,513287	-0,071982522	0,099443266	-0,723855172	0,469154654	0,784016071
Tspan32	5,9358143	0,477126615	0,58445104	0,816367126	0,414290193	0,751308409
Tmem117	1,060040464	0,294864592	1,489522274	0,197959169	0,84307701	NA
Abi3bp	110,0528712	-0,086846641	0,261916021	-0,331582011	0,740204905	0,916128178
Mir328	0,241026348	0,059593471	3,352475198	0,017775962	0,985817581	NA
Obscn	2,390622052	1,22686996	1,070800515	1,145750252	0,251898504	NA
Dnaja1	3687,178231	-0,573865791	0,150884591	-3,803342591	0,000142757	0,005836436
Pex7	1653,818883	0,219102246	0,078329264	2,797195262	0,005154837	0,070015871
Hist1h3c	0,180974489	-1,204797453	3,342691436	-0,360427361	0,718527568	NA
Hist4h4	0,315588022	0,58642274	3,256277324	0,180089925	0,857081972	NA
Gm5643	0,656854051	0,189213248	1,99945065	0,094632617	0,924606641	NA
Corin	3,500306379	1,281045404	1,280453187	1,000462505	0,317086734	NA
AU041133	57,44404476	-0,119214644	0,207225049	-0,575290702	0,565094709	0,83747956
Gm3604	5,275892706	0,2912055	0,771886284	0,377264769	0,705976852	NA
1700023F06Rik	0,453965707	2,27926563	2,926455669	0,778848507	0,43606896	NA
Mettl3	332,8889639	-0,094013761	0,117518812	-0,799989039	0,423717148	0,756445602
Noc3l	178,504866	0,125981121	0,188331726	0,668932014	0,503538847	0,804978322
Xrcc2	35,08780656	-0,216036028	0,274365594	-0,787402038	0,431046554	0,761954434
Park7	4183,399043	0,140214209	0,107667745	1,302286112	0,192818597	0,541552905
Ptges3	1922,183019	-0,183504168	0,155898714	-1,177073007	0,239166368	0,596519262
Zfyve28	1,302157599	1,557452065	1,638147965	0,950739554	0,341736604	NA
Zmynd12	4,217331888	0,71825436	0,698775907	1,027875107	0,304008581	NA
Arntl2	17,85180504	-0,043306284	0,347724159	-0,124542064	0,900886097	0,972526743
Fcrlb	0,653416038	0,222184224	2,000601859	0,111058691	0,911569806	NA
Prmt1	372,5736204	-0,085149751	0,121982787	-0,698047264	0,485147637	0,79430833
Ggcx	1795,439884	-0,225689494	0,145932039	-1,54653835	0,121974606	0,433849653
Gm9079	6,475116305	-0,392925279	0,563086733	-0,697805962	0,48529855	0,79430833
Josd2	610,5792141	-0,079638634	0,171435204	-0,464540727	0,642260388	0,872599504
Brd2	2824,460657	-0,059931199	0,150162164	-0,399109852	0,689812264	0,894436573
Akr1c13	2842,940079	-0,04642519	0,084135598	-0,551790094	0,581092175	0,845794369
Nr1h3	2506,441033	-0,108217816	0,076379729	-1,416839482	0,156529863	0,492040382
Agfg1	459,2876339	-0,088150911	0,12183565	-0,723523131	0,469358548	0,784101987
Mea1	1192,40612	0,008431884	0,07845518	0,107473897	0,914413033	0,976392941
9430038I01Rik	153,9020343	0,575494956	0,189203508	3,041671705	0,002352683	0,04238952
Atf7ip2	0,316677386	-1,85847818	3,205847439	-0,579715103	0,562106756	NA
Lvrn	1,227128048	1,138185835	1,484245544	0,766844704	0,443173854	NA
Hgsnat	976,0559301	0,102832539	0,091018534	1,12979779	0,25856144	0,619629549
Tmem200a	0,935448557	2,503480165	1,926469016	1,299517482	0,193766398	NA
Rgs7bp	6,495821536	-0,490504403	0,58478899	-0,838771609	0,401597485	0,742100471
Tbcd	603,206028	0,061241481	0,087662468	0,698605477	0,48479862	0,79420977
Cstf2t	508,3281153	0,101767204	0,110092397	0,924379945	0,355288532	0,705249056
Crebrf	531,1511306	-0,175708518	0,235383478	-0,746477706	0,455378894	0,77741801
Zkscan1	876,9478244	-0,162620076	0,151283681	-1,074934689	0,28240397	0,644056409
Sec14l5	0,856627627	1,21981754	1,761295448	0,692568383	0,488580458	NA
Parp8	28,94226474	0,546133402	0,313503261	1,742034198	0,08150246	0,353309466
E130309D02Rik	359,1448371	0,059748199	0,107537593	0,5556029	0,578482349	0,84530168
Cetn3	485,4969997	-0,063010989	0,09717482	-0,648429187	0,516707397	0,813424627
Has3	1,306203115	1,278588862	1,612547337	0,792900048	0,427836059	NA

Supplementary Table S1: *Serpina1* KO vs. wildtype all DEGs

Gpc4	939,8174057	-0,029842692	0,096736078	-0,308495994	0,757704948	0,923804952
Grid1	20,19807935	0,819811046	0,706284728	1,160737326	0,245748738	0,605051632
Cela2a	18,1982094	-0,051538841	0,388250483	-0,132746366	0,894393972	0,970390603
Stx2	306,1921069	-0,148314313	0,137440961	-1,079112886	0,280537408	0,641688878
Ptp4a1	264,7473937	-0,048991328	0,19309775	-0,253712578	0,799717616	0,938997437
Adh5	9947,871004	0,080303554	0,094187831	0,852589478	0,393887	0,736368577
Esrra	603,6902604	-0,291002368	0,162020456	-1,796084123	0,072481139	0,330736787
Igfbp7	1917,374623	-0,078167107	0,16881809	-0,463025654	0,643345978	0,873719259
Rpl34	38,8053809	-0,254275959	0,26253975	-0,96852366	0,332782909	0,68995097
Amh	1,504625437	0,055518983	1,306772555	0,042485575	0,966111611	NA
Clock	1208,728901	-0,21695193	0,127856841	-1,696834749	0,089727907	0,371499656
Gm4262	1,573370139	0,334985936	1,238976432	0,27037313	0,786873209	NA
Exo5	98,60598852	0,148797291	0,194614593	0,764574171	0,444525154	0,770550383
Haus8	84,51108605	-0,167103926	0,191304219	-0,873498382	0,382391488	0,726783025
H2-Q10	62478,86005	0,169297338	0,088465319	1,913714223	0,055656681	0,285308756
Jag1	99,66498227	-0,328860304	0,211465978	-1,555145215	0,119911488	0,430708406
Mtx3	283,0105953	0,052736809	0,156730019	0,336481868	0,736507523	0,914722813
Taldo1	2980,522355	-0,023255942	0,114048321	-0,203913054	0,838421444	0,951141594
Wwox	146,6486913	-0,107463513	0,162973734	-0,659391609	0,509644329	0,808332547
Cbln3	30,43669323	0,381418213	0,308612687	1,235912289	0,216491169	0,56985045
Pnpla6	575,5524685	-0,489669594	0,113342173	-4,320277097	1,56E-05	0,001170414
Barx2	0,249136072	1,389394708	3,349408814	0,414817893	0,67827522	NA
Acrbp	71,38537311	-0,326254064	0,243116031	-1,341968536	0,179606198	0,522217324
Skp2	56,9503704	-0,381494446	0,253629839	-1,504138657	0,132545669	0,453057798
Ccl8	0,172953423	1,307385949	3,350719078	0,39018071	0,696402925	NA
Gpr132	8,538415658	0,440894875	0,645157361	0,683391219	0,494359667	0,799621608
Dbn1	52,13218198	-0,247205687	0,27810117	-0,8889056	0,374053815	0,720823634
Arhgap17	335,4752271	-0,121752589	0,102678201	-1,18576862	0,235713687	0,593010215
Pgghg	849,9580983	-0,046259943	0,10918824	-0,423671483	0,671805415	0,887904019
Mfsd6l	1,655293779	0,653141363	1,132675098	0,57663611	0,5641853	NA
Lyn	691,4192669	0,068766577	0,082417873	0,834364854	0,404075416	0,744293778
Eftud2	682,4225441	-0,088312001	0,097784908	-0,903125061	0,366459525	0,714711689
Ptpn11	1483,958029	-0,222258367	0,089882902	-2,472754677	0,013407614	0,124997488
Canx	8802,33121	-0,347210095	0,124871735	-2,780533928	0,005426959	0,071763613
Rsph3b	157,2588434	-0,191588597	0,136587858	-1,402676633	0,160713288	0,497355545
Slc35a2	284,5132523	-0,230911157	0,110931469	-2,081565841	0,037382144	0,226546639
Pth1r	292,7557909	0,142276508	0,160158157	0,888350058	0,374352481	0,720933206
Slc25a53	8,188590244	-0,01101438	0,517905788	-0,021267149	0,983032549	0,995413661
Tmsb15b1	2,287632088	1,527354272	1,016903792	1,501965362	0,133106055	NA
Nemp1	97,39440684	0,298325259	0,18754753	1,590664824	0,111685027	0,413687165
Mfap1b	33,09092676	-0,044614772	0,262530638	-0,169941203	0,865056377	0,961775826
9430020K01Rik	176,744876	-0,011202125	0,170987336	-0,065514355	0,947764477	0,985430339
Foxl1	0,234199685	1,337107854	3,350237755	0,399108348	0,689813372	NA
Hmg20b	580,8360503	-0,268054318	0,131184159	-2,043343651	0,041018431	0,238839464
Rpgrip1	17,09007548	2,03948313	0,668493688	3,050863706	0,002281841	0,041527555
Zfp287	7,851861473	0,279184975	0,578576467	0,482537731	0,629424002	0,866228449
Mcf2	0,573907151	1,628930814	2,354170454	0,6919341	0,488978715	NA
Cstf2	366,9060529	-0,149655817	0,157395667	-0,950825526	0,341692953	0,695250579
Cyp2a22	314,6902844	4,932925271	1,323280623	3,727799822	NA	NA
Shc1	1410,715231	-0,119645269	0,095350179	-1,254798582	0,209551888	0,561873081
Washc3	499,7265112	-0,040387306	0,088816733	-0,454726322	0,649306141	0,876648536
Plcxd1	27,59125824	1,459836225	0,633425774	2,304668179	0,021185164	0,16249252
Dgkh	4,542906274	-0,539836635	0,751274129	-0,71856146	0,472411167	NA
Sh2b1	1059,1964	-0,019937053	0,074412145	-0,2679274	0,788755203	0,935202374
Crhr2	4,17306449	0,607612743	0,733164257	0,828753907	0,407243675	NA
Sema4d	21,17175937	0,559099689	0,333221336	1,677862817	0,093373883	0,380085036
Pbdc1	275,9177339	-0,043528411	0,117916644	-0,369145608	0,712019195	0,905478699
Cysltr1	13,55640114	0,477725109	0,558498709	0,855373702	0,392344297	0,734887345
Aoah	35,77079036	0,278089322	0,401308947	0,6929557	0,488337353	0,796400974
S100a9	20,43125798	-0,409324632	0,424151566	-0,965043313	0,334523118	0,691222347
Klrc1	4,394106114	0,370924788	0,837351027	0,442974065	0,657784494	NA
Anxa8	5,422909139	3,243805982	1,016634272	3,190730504	0,001419136	0,030114964
Phyhd1	1736,995145	0,162126114	0,107429802	1,509135371	0,131264192	0,45054454
Sec23b	1661,972329	-0,617433946	0,13454578	-4,589024994	4,45E-06	0,000428309
D630023F18Rik	0,509671155	-1,693159142	2,053994323	-0,824325132	0,409754842	NA
Inpp5a	333,683968	-0,108404623	0,111052027	-0,976160681	0,328984845	0,686253454

Supplementary Table S1: *Serpina1* KO vs. wildtype all DEGs

Scnm1	321,3460955	-0,020835522	0,117324856	-0,177588303	0,859046311	0,958887045
Jag2	2,410850864	1,322850823	1,143886116	1,156453255	0,247495809	NA
Rb1cc1	925,0127991	-0,01897424	0,124818046	-0,152015195	0,879174951	0,965347075
Mir194-2	0,173498105	0,059593471	3,352475198	0,017775962	0,985817581	NA
Slc22a15	210,2272057	0,091243828	0,22027539	0,414226156	0,678708488	0,890709117
Ect2	37,97577907	0,182241517	0,529250152	0,344339092	0,730591289	0,911941257
Nkain4	7,583795324	1,638446143	0,683959821	2,395529814	0,016596364	0,142777861
Il18r1	7,790821502	1,279405296	0,630984598	2,027633163	0,042597702	0,243203202
Pln	0,207615244	0,059593471	3,352475198	0,017775962	0,985817581	NA
Bean1	0,180429807	0,059593471	3,352475198	0,017775962	0,985817581	NA
Cd300lg	266,9490832	-0,238625197	0,273596256	-0,872180055	0,383110159	0,727569276
Samhd1	519,0140949	0,142774898	0,151415928	0,942931828	0,345715774	0,698434374
Rbl1	65,82850696	-0,429835651	0,253743915	-1,693974222	0,090270182	0,372821149
Syce3	0,841453555	-0,679723206	1,887806193	-0,360059846	0,71880238	NA
Mtmr11	135,4863788	0,681083727	0,239753166	2,840770517	0,004500468	0,063871298
Rftn1	37,70458149	-0,263723226	0,267622825	-0,985428751	0,324413513	0,681609973
Me3	3,999497353	0,728749392	0,808849001	0,900970875	0,367603806	NA
Rars2	379,665183	0,073494523	0,09140869	0,80402118	0,421384743	0,754856308
Rnpepl1	1550,939883	-0,047741039	0,083138935	-0,574232032	0,565810796	0,837781736
Anapc13	1107,65851	0,10837263	0,110980258	0,976503679	0,328814926	0,686006046
Kank1	1366,753817	-0,224966818	0,142214019	-1,581889182	0,113674871	0,417177269
Vps37c	371,5360629	-0,339402907	0,118718014	-2,858899815	0,00425113	0,061176921
Mustn1	107,2472052	0,838200852	0,215252555	3,894034394	9,86E-05	0,004498493
Inpp5f	580,0678604	-0,28463152	0,140375969	-2,027637086	0,042597301	0,243203202
Arel1	1077,360762	-0,062339992	0,11021937	-0,565599244	0,571666242	0,841407954
Pcdh11x	0,383015831	-0,038427383	2,618382674	-0,014676	0,988290667	NA
Aicda	0,12663974	0,780932884	3,352475198	0,232942181	0,815806304	NA
1110002J07Rik	23,02138811	0,361539744	0,358975221	1,007144012	0,313865574	0,672554713
Mmp19	2084,272173	0,020329831	0,141070789	0,14411085	0,885412937	0,967477365
Ifngr2	372,1178719	0,253138642	0,114821323	2,20463094	0,027480003	0,191005731
Ifit2	98,13715908	0,302796461	0,235033077	1,288314248	0,197636596	0,54738008
Mep1a	0,340986444	1,884176617	3,333184127	0,565278288	0,571884494	NA
Meox2	0,573231143	2,646858416	2,704953146	0,978522833	0,327815804	NA
Map2k1	1052,746271	-0,216167415	0,094968107	-2,276210626	0,022833406	0,169966486
Nrxn1	81,0160047	-0,12298501	0,255279284	-0,481766511	0,629971822	0,866235628
Mrpl21	556,2770212	0,024692673	0,132474451	0,186395737	0,852134435	0,95595696
Glg1	421,518719	-0,317639518	0,186674292	-1,701570767	0,088835861	0,36952291
Gm15760	7,454281675	0,311519177	0,520680224	0,598292701	0,549644646	0,82961684
Coq10a	507,8403981	0,067644796	0,137738345	0,49111085	0,623348048	0,863525452
Grk3	126,7051337	-0,136988378	0,278771214	-0,49140073	0,623143048	0,863474234
Spns2	904,6109531	0,152847493	0,166183982	0,919748645	0,357704125	0,707149983
Ttc28	34,97462097	0,045857246	0,267998472	0,171110104	0,864137191	0,961272625
Crisp3	0,086476712	0,780932884	3,352475198	0,232942181	0,815806304	NA
Tnnc1	37,4217818	-0,066339636	0,403421995	-0,164442288	0,869382976	0,961775826
Mad2l2	605,9517815	0,031236718	0,117065316	0,266831534	0,789598874	0,935361858
Cd93	96,2004729	-0,288495296	0,274186595	-1,052185995	0,292714224	0,654074594
Upp1	33,02627988	0,351691924	0,353307499	0,995427283	0,319528495	0,677329731
Hsd17b7	1557,293996	0,49797716	0,153930565	3,235076536	0,001216101	0,027416624
Snord88a	0,558573165	0,367025282	1,918842605	0,191274303	0,848310692	NA
9430065F17Rik	9,373193231	-0,092224726	0,505906894	-0,182295847	0,855350558	0,95747983
9530020I12Rik	1,080513833	-1,432261148	1,460307765	-0,980794037	0,326694319	NA
Hormad2	1,865009334	0,252385509	1,339377513	0,188434931	0,850535719	NA
Nfkbiz	176,5490178	0,172412923	0,17933912	0,961379333	0,336361472	0,69270916
Kcnrg	0,63503244	-2,125599619	1,859326116	-1,143209682	0,252951543	NA
Negr1	0,389399041	0,666668286	3,069715667	0,217175908	0,828071259	NA
Tom1l2	626,5587744	-0,073020699	0,12861006	-0,567768176	0,570192392	0,841130547
Fndc1	8,277322907	1,766143991	0,993386286	1,77790253	0,075419865	0,339490966
Zfp30	55,50996891	-0,170790128	0,227665465	-0,750180218	0,453146171	0,775628467
Atp9b	679,6106756	-0,059721813	0,091941722	-0,649561616	0,515975434	0,813164632
Dazap1	958,111946	0,03379021	0,088730332	0,380819155	0,703337445	0,900719679
Cops5	1195,162903	0,12145598	0,077505326	1,567066248	0,117099208	0,424392729
Pcdhgb4	1,786510093	-0,217950497	1,212062582	-0,179817858	0,857295564	NA
Ihh	75,84077547	-0,456670629	0,331113944	-1,379194799	0,167834701	0,506611452
Sh3yl1	210,9264839	0,228339473	0,172641883	1,322619224	0,185962038	0,532805747
Ccdc106	3,744600876	-0,48196943	0,802226591	-0,600789647	0,547980101	NA
Pkp3	19,39280398	1,005704613	0,443566069	2,267316379	0,023370906	0,172812852

Rtn4ip1	635,5860559	-0,201818867	0,122592991	-1,64625127	0,099712038	0,392993389
Tm9sf2	3770,711534	-0,065331761	0,097481347	-0,670197568	0,502731854	0,804238621
Plpp3	3068,468593	0,400567926	0,193573665	2,069330696	0,038515065	0,230486976
Psmd5	917,4332491	-0,055589532	0,077035587	-0,721608474	0,470535228	0,784805121
Itpr3	45,50745675	0,351113594	0,287850728	1,219776641	0,222549559	0,577161019
Arhgef5	722,5027539	-0,003273076	0,080796825	-0,040509957	0,967686569	0,991231429
Itga9	301,3592557	0,071274377	0,251028485	0,283929438	0,776464456	0,930009104
Snord2	3,708867579	-0,651600142	0,83807257	-0,777498471	0,436864736	NA
Snora30	0,355017277	-2,060768074	3,080293056	-0,669016888	0,503484705	NA
Nol11	330,0049072	0,073615879	0,138978568	0,529692312	0,59632528	0,853075785
Zfp931	56,24172511	0,212127448	0,240485709	0,882079225	0,377733966	0,723899855
Pgbd1	14,89272372	-0,017537842	0,372150429	-0,047125679	0,962413061	0,988526411
Klhl32	0,706076427	-1,059185903	1,960188321	-0,540349053	0,588956336	NA
A430005L14Rik	743,6268298	0,037358234	0,111612377	0,334714077	0,737840782	0,91507318
Suz12	535,6741104	-0,018535878	0,123487884	-0,15010281	0,880683503	0,96546599
Uxt	435,2105941	-0,005187458	0,141315251	-0,036708409	0,970717504	0,992007633
Epb41l4aos	132,175476	0,475430952	0,183539943	2,59034052	0,009588104	0,102056813
Uchl3	534,6302495	0,082863802	0,105035294	0,788913886	0,430162339	0,761019227
Ddx20	231,4002317	0,170683084	0,1595186	1,069988603	0,284624439	0,645924991
Cetn2	333,4134122	0,184830933	0,122821885	1,504869693	0,132357582	0,452967915
Rfx5	284,8716015	0,025441044	0,125412212	0,20285938	0,839244944	0,951362994
Mrpl39	1224,80901	-0,113638334	0,068331976	-1,66303304	0,096305822	0,385838157
Slc7a10	0,279910125	-1,713254196	3,310169948	-0,517572881	0,604756307	NA
Clpp	1231,10541	-0,069707539	0,117968019	-0,590902005	0,554586083	0,832221499
Cxcl14	50,9585148	0,570687396	0,306192032	1,863821839	0,062346728	0,303095783
Prodh2	4326,324034	0,129093673	0,09784019	1,319433998	0,187024062	0,533459501
Polm	115,4605488	0,040955466	0,170163835	0,240682554	0,809801162	0,94241441
Gmeb1	198,9653297	0,187243821	0,138587867	1,351083792	0,176668593	0,519981998
Vezt	437,9979902	-0,152063533	0,138928123	-1,094548243	0,273714601	0,635293489
Micu1	737,0707962	0,00432602	0,112483263	0,038459231	0,969321536	0,991724161
Fbxw10	1,484630281	1,211859459	1,493852097	0,81123122	0,4172329	NA
Myo18a	1668,71769	-0,079038031	0,08558533	-0,923499754	0,355746829	0,705740033
Fabp2	1322,536224	-0,606497335	0,140071559	-4,329910658	1,49E-05	0,001133098
Gad2	0,12663974	0,780932884	3,352475198	0,232942181	0,815806304	NA
Nos2	1,278105171	2,178574935	1,649898221	1,320429895	0,186691528	NA
Trappc2	76,54098692	0,161942203	0,205649775	0,787465987	0,431009132	0,761954434
Cmtr1	40,86095532	-0,561311721	0,296592363	-1,892535991	0,058419603	0,291857875
Itm2a	16,83911848	0,168994745	0,431632716	0,391524411	0,695409649	0,897040871
Sms	93,36384877	-0,365147301	0,225622702	-1,618397871	0,105576881	0,403046295
Ttll10	3,113037002	1,133811616	0,979128283	1,15798066	0,246871929	NA
Pik3cb	312,6473787	0,179090747	0,128392911	1,394864752	0,163056628	0,49993802
Fundc1	191,8771945	-0,151671752	0,138312324	-1,09658885	0,272821164	0,63465219
Ccdc175	0,269274339	0,059593471	3,352475198	0,017775962	0,985817581	NA
Ift57	25,52811262	0,613023409	0,359625346	1,704616807	0,088265915	0,368413054
Fam43a	98,12279945	0,169490098	0,321282823	0,527541737	0,597817441	0,853597141
Mir1195	0,208973043	1,337107854	3,350237755	0,399108348	0,689813372	NA
Ppfibp2	1001,754876	0,16688702	0,101117397	1,650428366	0,098855353	0,392049012
Ttn	1,265050205	0,108099732	1,538884032	0,070245535	0,943998232	NA
Trpc1	6,712457161	0,340961819	0,721743818	0,47241391	0,636631378	0,86976752
Il12rb2	3,004310291	1,263603015	0,897402498	1,408067192	0,159111175	NA
Il12rb1	15,44370517	-0,571521579	0,575696856	-0,992747438	0,320833051	0,678351583
Il12a	0,439754904	0,241146527	2,563116173	0,094083339	0,925042955	NA
Il10rb	246,7578724	0,081848032	0,1906685	0,42926877	0,667727642	0,886039786
Rbbp4	2231,761753	-0,414650094	0,143238855	-2,89481575	0,003793811	0,057439938
Plxna1	224,9889416	-0,232876358	0,106468639	-2,187276554	0,028722341	0,195483304
Igfals	3170,511942	-0,101751173	0,13329259	-0,763367065	0,445244516	0,770945983
Ube2w	461,516873	0,07084778	0,131046496	0,540630859	0,588762044	0,849008711
Isoc2b	365,8031235	0,28219208	0,142271823	1,983471312	0,047314808	0,259242489
Mtpap	407,2892936	0,1251363	0,110414356	1,133333605	0,257074185	0,617800607
2810417H13Rik	7,877305619	-1,837886085	0,611736406	-3,00437585	0,002661263	0,045496715
Pla2g15	416,5627807	0,178121036	0,150297828	1,185120491	0,235969811	0,593259146
0610012G03Rik	585,0159845	0,04466228	0,129753926	0,344207546	0,730690208	0,911941257
Mir6240	0,920272981	0,781744302	1,56785998	0,498605942	0,618057019	NA
Mospd1	203,5832341	0,302670095	0,154803051	1,955194639	0,050560101	0,270337998
Aldh1l1	42667,93295	0,597676848	0,118200515	5,056465697	4,27E-07	6,66E-05
Rarb	134,1322298	-0,072649199	0,206896078	-0,351138598	0,725484372	0,910252319

Supplementary Table S1: *Serpina1* KO vs. wildtype all DEGs

Pitpnm1	129,1953872	-0,143903717	0,21013208	-0,684825073	0,493454312	0,799062169
Rrh	0,113662148	0,780932884	3,352475198	0,232942181	0,815806304	NA
Tbx15	0,584748855	2,638328875	2,271879867	1,161297705	0,245520853	NA
Gng5	1125,129287	0,017079182	0,108127789	0,157953679	0,874493299	0,963600184
Supt6	1302,991871	-0,258135516	0,099538118	-2,593333306	0,00950506	0,101415126
Uba3	900,7257129	-0,195391931	0,110276487	-1,771836741	0,076421662	0,34238646
Papss2	6685,829878	0,637530136	0,148988486	4,279056404	1,88E-05	0,001333939
Clec12b	0,883641282	0,169825446	1,739455136	0,097631403	0,922224987	NA
Rnf157	26,10136756	-0,510677807	0,402929217	-1,267413197	0,205007583	0,558049056
Rspo3	100,1871177	0,065045577	0,21537607	0,302009301	0,762644972	0,925557106
Ints4	601,2559438	-0,026276452	0,108188339	-0,242876937	0,808100732	0,942196162
2010107G23Rik	34,38321421	0,851032573	0,286037827	2,975244852	0,002927548	0,048140696
Coa7	412,6092204	0,154984684	0,106246041	1,458733737	0,144638407	0,472725517
Mir1947	0,093953095	-0,517475177	3,352475198	-0,154356154	0,877328924	NA
Gramd1a	282,3967767	0,000132963	0,110033698	0,001208381	0,999035851	0,999803624
Cwf19l2	281,760566	0,044632564	0,178489413	0,250057206	0,802543109	0,940003402
Stk11ip	150,8433762	0,031637527	0,165900084	0,190702299	0,848758837	0,954095425
Pigo	314,6950296	0,061519348	0,121681605	0,505576404	0,61315405	0,859251205
Clec7a	120,3508547	0,413407749	0,235855772	1,75279895	0,079636523	0,349439855
Impa1	1711,035904	-0,118121488	0,089969606	-1,312904364	0,189215176	0,536686321
Dnajb1	500,1834103	-0,51800513	0,196353793	-2,638121338	0,008336675	0,094132604
Npff	10,72414961	0,698770824	0,661928146	1,055659633	0,291123746	0,653114648
Rassf5	288,9638543	-0,142748125	0,211628344	-0,674522717	0,499979048	0,803112736
Mycbp	310,9752866	-0,01853011	0,13717085	-0,135087811	0,892542447	0,969104575
Cyb5r1	151,2773141	0,053803584	0,162549752	0,330997636	0,740646271	0,916181159
Rhpn2	412,9594309	0,083432919	0,185346536	0,450145554	0,652605492	0,877662367
Itfg1	1667,646171	-0,067386636	0,074157676	-0,908694011	0,363511666	0,712361223
Tube1	32,37944955	0,105444583	0,291194225	0,362110833	0,717269208	0,907650451
Vwa8	3870,456974	-0,105019116	0,14505304	-0,724004933	0,469062707	0,783960411
Adamts16	2,860469411	1,255711094	0,918263725	1,367484155	0,1714736	NA
Ulk3	198,4947919	-0,157375048	0,137452221	-1,144943652	0,2522325	0,612043137
Rnf7	624,6748743	-0,180398894	0,121349978	-1,486600138	0,137120451	0,45990048
Reln	893,6432897	-0,1861019	0,12841502	-1,449222214	0,147275536	0,47747598
Zfyve19	487,1725872	0,051280347	0,108889367	0,470939898	0,637683655	0,870184015
Tmem38b	1307,399573	0,2272771	0,116797709	1,945903746	0,051666303	0,273663551
Tcp11x2	0,371745909	-0,928546775	3,151455697	-0,294640593	0,768268449	NA
Foxp4	632,2032393	-0,384291201	0,124229255	-3,093403416	0,00197875	0,038104827
Xpo5	393,1247692	-0,060538973	0,106358894	-0,569195209	0,569223671	0,840790976
Cybrd1	3,932468554	1,057352559	0,849110039	1,245247978	0,213040612	NA
Camkmt	30,94053877	0,274928926	0,303753311	0,905105939	0,365409266	0,713689942
Hrct1	5,74374027	0,456501122	0,666993657	0,684415988	0,493712523	0,7992036
1190007I07Rik	223,222668	0,005107696	0,127419098	0,040085795	0,968024727	0,991231429
4933411K16Rik	53,52957954	0,315290757	0,348718834	0,904140317	0,365921003	0,714426527
Urad	943,3356229	0,284647377	0,143826253	1,979105841	0,047804091	0,261174047
Med24	574,6907644	-0,209759578	0,122970589	-1,705770294	0,088050856	0,367974959
Spr	1678,729852	0,052085673	0,097692306	0,533160442	0,593922522	0,851766384
Spta1	0,122496332	0,780932884	3,352475198	0,232942181	0,815806304	NA
Serpina3k	314225,0771	-0,418987667	0,190772892	-2,196264171	0,028073041	0,192787383
Slc2a5	92,10081688	0,379873054	0,277156374	1,370609117	0,170496839	0,510601034
Stmn1	91,4467148	-0,651282202	0,2588493	-2,516067084	0,011867259	0,116483357
Mllt11	31,25478638	0,373530729	0,278207628	1,342632952	0,179390855	0,522135977
Mtch1	1649,623481	0,152314205	0,087055965	1,749612508	0,0801852	0,350251568
Prx	4,82930761	-0,762622001	0,782254858	-0,974902224	0,329608763	NA
Ppp2ca	3059,384061	-0,021068102	0,085490294	-0,246438526	0,805342786	0,94096033
Tbc1d1	156,1005604	0,018129297	0,21118362	0,085846131	0,931588735	0,980584977
Pkp1	2,991385723	-0,013689217	0,848318971	-0,016136875	0,987125196	NA
Plxnb3	4,770693197	1,352491845	0,763112234	1,772336736	0,076338677	NA
Plce1	264,9721146	0,076434336	0,140827797	0,54275035	0,587301703	0,848572815
Elf4	55,82928038	-0,011120535	0,251020532	-0,044301298	0,964664237	0,989761795
Cyhr1	1425,2889	-0,06213579	0,070014001	-0,887476627	0,374822345	0,720996253
Isy1	376,5944365	-0,246605364	0,088153519	-2,797453427	0,005150719	0,070015871
Slc35b4	641,1078815	-0,327211958	0,136727412	-2,393170118	0,016703494	0,143422618
Stk4	520,6867104	-0,008828044	0,106881004	-0,082596943	0,934172032	0,981835369
Atl2	2144,027234	0,044574362	0,105894983	0,420929881	0,673806285	0,888626355
Ank	590,397483	0,29566685	0,13917586	2,124411877	0,033635718	0,214233402
Cd86	76,06631102	0,219887278	0,236126052	0,931228368	0,351735446	0,702880198

Supplementary Table S1: *Serpina1* KO vs. wildtype all DEGs

Dolpp1	404,343853	-0,109908126	0,104410695	-1,052651989	0,292500521	0,654074594
Retnla	0,388024861	2,013582874	2,565692911	0,784810553	0,432564652	NA
BC031361	24,39563035	0,104335074	0,340582481	0,306343044	0,759343469	0,924882144
Cep112	4,129043464	0,330589286	0,684904305	0,482679527	0,629323302	NA
Det1	151,9353706	0,335945264	0,141972735	2,366266052	0,017968527	0,1487353
Zfp773	5,021514183	-0,769955997	0,859541706	-0,895775029	0,370372929	NA
Adtrp	2771,238306	-0,018406018	0,147719955	-0,124600759	0,900839628	0,972526743
Pkdrej	0,627602016	2,761373889	2,660103617	1,038070048	0,299237445	NA
Cenpb	1565,783691	0,21174955	0,098361676	2,152764763	0,031337171	0,205567535
Itgb5	1936,710032	0,209941691	0,093534991	2,244525688	0,024798598	0,179789836
Mapk8ip3	460,8043238	-0,226263392	0,17216009	-1,314261578	0,188758192	0,536004306
Fam110b	7,94919402	0,040750925	0,527388002	0,077269344	0,938409278	0,982879468
Fam124b	0,241676068	0,059593471	3,352475198	0,017775962	0,985817581	NA
Zbtb37	87,45694805	0,066247106	0,154315037	0,429297802	0,667706516	0,886039786
Fem1c	244,0972957	-0,130195605	0,145124982	-0,897127445	0,369650915	0,717858445
Colec10	533,7770864	0,134502632	0,216155169	0,622250362	0,533777255	0,82137064
Erich5	1,317683062	2,049011822	1,52829382	1,340718516	0,180011861	NA
Tmem27	0,634592796	-0,689551399	2,333373707	-0,295516915	0,767599033	NA
BC002163	23,09737338	-0,161753495	0,318169025	-0,508388567	0,611180869	0,858645358
Fam131c	76,41640794	0,258982967	0,369107446	0,701646553	0,482899612	0,79399946
Sirpa	563,7558276	0,089654782	0,240628551	0,372585803	0,709456741	0,90343073
Golm1	88,02434269	-0,177594088	0,254032348	-0,69910029	0,484489358	0,79420977
Yap1	1451,504709	-0,19804444	0,093139853	-2,126312567	0,033477242	0,213835285
Phf11b	29,31919552	-0,152860827	0,317601487	-0,48129758	0,630305017	0,866281784
Eomes	1,80303834	0,230636075	1,230161951	0,187484318	0,851280918	NA
Hdc	28,95287386	0,029884593	0,430445431	0,069427136	0,94464963	0,984181803
Hdac2	672,3673911	0,013288006	0,08818082	0,150690437	0,880219917	0,96546599
Hdac1	469,2762235	-0,007135519	0,105765817	-0,067465264	0,946211314	0,984653942
Hcn3	9,660507192	1,1873789	0,918302231	1,293015372	0,196005743	0,54489515
Hcn2	12,58209805	-0,89273078	0,408446187	-2,18567539	0,028839363	0,195911301
Fgfr3	667,6729798	0,158324232	0,138836935	1,140361042	0,254135916	0,614161798
Mmrn1	5,015946368	1,433275893	0,806580469	1,776978178	0,07557183	NA
Gls	76,8666451	0,056877782	0,285553475	0,199184345	0,842118545	0,952074658
Dyrk1b	268,3469265	0,34855767	0,201832398	1,726965903	0,084173787	0,359182688
Aftph	980,7527418	-0,051452167	0,086036419	-0,598027755	0,549821414	0,829634592
Clu	33979,25687	-0,082332763	0,094571944	-0,870583378	0,383981675	0,728459063
Mecom	16,52810074	-1,010435234	0,425298925	-2,375823625	0,01750983	0,146397073
Slc37a4	2625,68151	0,305688327	0,190257132	1,606711524	0,108117664	0,408238928
G6pdx	289,6769307	1,18348119	0,325610029	3,634658287	0,000278349	0,009541672
Lgals7	0,31434569	0,667660234	3,262174889	0,20466721	0,837832141	NA
Etv6	493,27946	-0,228716496	0,14461753	-1,581526776	0,113757642	0,41723619
1700102P08Rik	3,381823714	0,099715321	0,762573485	0,13076159	0,895963909	NA
Ugt2b37	73,6019181	-1,490036668	0,47937448	-3,10829368	0,00188171	0,036692532
Scn4a	1,30644762	1,530666749	1,45411633	1,052643944	0,292504209	NA
Bmp2k	120,0788295	-0,065210176	0,183682716	-0,355015307	0,722578124	0,909644412
Caskin2	327,2470978	-0,173059552	0,132346816	-1,307621578	0,191001695	0,540080722
Timp4	0,093303375	-0,517475177	3,352475198	-0,154356154	0,877328924	NA
Baalc	0,093953095	-0,517475177	3,352475198	-0,154356154	0,877328924	NA
Mvp	935,7461296	0,063024051	0,110117996	0,572331981	0,567097087	0,838772055
Nme5	2,008405286	1,253522094	1,189723471	1,053624749	0,292054753	NA
Clk2	86,65420843	0,141003574	0,185429515	0,760416019	0,447005951	0,772195705
Tars	5228,479117	0,004290182	0,109843161	0,039057342	0,968844671	0,991555135
Aco2	5415,185614	-0,009135795	0,070214193	-0,130113223	0,896476849	0,970995624
Emid1	5,081279035	-0,278417092	0,676777522	-0,411386434	0,6807892	NA
Sertad3	127,2444182	-0,07014079	0,193151326	-0,363139056	0,716501011	0,907405448
Fam208a	523,1278487	-0,023881526	0,115499377	-0,206767576	0,836191379	0,951141594
Ccdc146	0,67805223	1,916629121	1,976372307	0,96977129	0,332160507	NA
Sp110	55,15336174	0,164578171	0,257790349	0,638418667	0,523201183	0,815322684
Map7d1	1188,787485	-0,189471796	0,107695606	-1,759327072	0,078521971	0,347468495
Zfp791	29,96591819	0,247056659	0,314599862	0,785304411	0,43227511	0,762604031
Wdfy1	159,7732653	-0,192610805	0,165519417	-1,163674982	0,244555752	0,603445154
Grk4	27,38487463	-0,143024594	0,273621465	-0,522709702	0,601176286	0,854422974
Pitpnb	1997,223447	-0,032374571	0,077384434	-0,418360248	0,675683746	0,889164486
Tacc2	574,276878	-0,084577187	0,189234035	-0,446944901	0,654914843	0,87916021
Il4	0,281859286	-1,720570404	3,309774099	-0,519845268	0,603171425	NA
Ammecr1	17,97792784	0,328251099	0,372430579	0,881375263	0,378114742	0,723903192

Supplementary Table S1: *Serpina1* KO vs. wildtype all DEGs

Gdf2	414,6363644	-0,042197969	0,165378098	-0,255160565	0,798599088	0,938733973
Slco1b2	19264,17808	0,167064593	0,204906526	0,815320997	0,414888593	0,751551608
Vav3	16,8432539	0,390069035	0,434932091	0,896850434	0,369798732	0,717858445
Il10ra	93,02319485	0,111508364	0,212427506	0,524924318	0,599635803	0,85430922
Borcs5	252,0085789	-0,181283412	0,128354663	-1,412363266	0,157843027	0,493680148
Tada2b	548,2267346	-0,098265087	0,150676166	-0,652160788	0,514297453	0,811764185
Ppfibp1	619,9676745	0,263079181	0,142990467	1,839837207	0,065792141	0,313797765
Cd163	242,5199631	-0,092792159	0,282896184	-0,328007815	0,742905753	0,917092327
Slco1c1	1,545602878	-0,209884692	1,376612332	-0,152464632	0,878820484	NA
Fam78b	2,200789466	-0,866938739	1,043630563	-0,830695047	0,406145925	NA
Mroh1	671,2204371	-0,291968553	0,115768944	-2,521993751	0,011669178	0,115388493
Hid1	41,78404452	-0,074327368	0,305310576	-0,243448391	0,80765806	0,942032857
Dhrs9	146,8569873	-0,159308908	0,214136089	-0,743961044	0,456900043	0,77843077
Actbl2	0,306419827	0,617376943	2,735241395	0,22571205	0,821425385	NA
Gpr150	0,122496332	0,780932884	3,352475198	0,232942181	0,815806304	NA
Fam26f	35,85607893	1,003550095	0,384436144	2,61044678	0,009042404	0,098887698
Zmat1	122,0808138	0,157289365	0,166853862	0,942677399	0,345845937	0,698434374
Rassf2	33,25306842	0,63168001	0,293491381	2,152294926	0,031374133	0,205618422
Etnk2	5308,368096	-0,047259892	0,095055974	-0,49717961	0,619062399	0,861782322
A630033H20Rik	8,442951529	0,939077958	0,616401972	1,523483054	0,127637897	0,444836039
Aldh4a1	10371,00237	0,15657404	0,111655218	1,402299346	0,160825876	0,497588783
Gpc5	0,086476712	0,780932884	3,352475198	0,232942181	0,815806304	NA
Grik4	2,975123477	-0,430525394	0,876240857	-0,491332253	0,623191472	NA
Ndufa5	1476,007755	0,104857816	0,131635924	0,796574467	0,425698211	0,758328303
Rbm15b	185,6022595	-0,301049364	0,196525221	-1,531861219	0,125556685	0,441147786
Zfp710	371,6300799	-0,296324794	0,106348119	-2,786366104	0,005330263	0,070973656
Fam228b	22,1964149	0,318906721	0,407872516	0,781878427	0,434286024	0,764144493
Ccdc40	2,790294949	-0,281771525	0,818890035	-0,344089575	0,730778922	NA
Kctd12b	39,08503907	0,021225627	0,286408104	0,074109728	0,940923075	0,983128178
Ccdc88c	30,44412314	0,182000431	0,30424561	0,598202325	0,54970494	0,82961684
Smpd3	88,13412748	0,923031012	0,272347735	3,389163539	0,000701062	0,018596222
Ecm1	3878,002284	-0,085517178	0,118350894	-0,72257315	0,469942169	0,784449601
Ivd	4582,117354	-0,035205434	0,092063511	-0,382403775	0,702161891	0,900451062
Efemp2	84,05383205	-0,172985969	0,229164698	-0,754854349	0,450336379	0,773849236
Mlxipl	9968,248859	0,012893846	0,162296045	0,079446461	0,936677515	0,982210769
Cyp2d22	6201,975642	0,193987507	0,106405185	1,82310201	0,068287942	0,319740014
Cacna1h	1,097189761	0,677599549	1,324460647	0,511604139	0,608928088	NA
Asic5	82,79256218	-0,011846621	0,229407339	-0,051640113	0,958815456	0,987896001
Mapk8ip2	0,087021394	-0,517475177	3,352475198	-0,154356154	0,877328924	NA
Xlr4b	5,803841083	-1,437846517	0,904114346	-1,590337022	0,111758858	0,413687165
Gmfg	121,0451691	0,335709007	0,183460903	1,829866751	0,067269866	0,317560326
Pappa	1,423571111	0,284767966	1,482806192	0,192046653	0,847705662	NA
Nova1	0,772634583	2,142343507	1,726384702	1,240942129	0,214627128	NA
Mapk11	24,70298595	-0,130041387	0,411270547	-0,316194261	0,751855064	0,920893298
Sall1	622,7163675	0,793555184	0,255336194	3,107883659	0,001884322	0,036692532
Prg4	1172,051329	0,595700965	0,111948043	5,321227152	1,03E-07	2,34E-05
Sh3kbp1	50,08979092	0,244264989	0,252263911	0,968291454	0,332898831	0,68995097
Extl2	206,4626766	0,111467255	0,123084137	0,905618361	0,365137887	0,713569421
Mvd	1253,37285	0,358756446	0,207812937	1,726343176	0,084285692	0,359537594
Cope	2598,732862	-0,170354595	0,103557557	-1,645023311	0,099965004	0,392993389
Lipa	7928,801802	0,084029832	0,107206631	0,783811883	0,4331505	0,763032854
Mir126a	1,407780802	-0,834534689	1,186116497	-0,70358577	0,481690776	NA
Gpld1	6861,688894	0,209580868	0,082062927	2,553904376	0,010652251	0,10929389
Top1	1193,957754	-0,36595131	0,118600646	-3,085576026	0,002031583	0,038665614
Mir5129	0,093953095	-0,517475177	3,352475198	-0,154356154	0,877328924	NA
Mir5128	0,18660675	-1,235117516	3,340448777	-0,369745983	0,711571767	NA
Dnaja2	2563,165966	-0,141545307	0,088714573	-1,595513585	0,110597434	0,411311191
B4galt4	42,75326497	-0,269890696	0,258193147	-1,045305416	0,295881835	0,656921786
Slc52a3	12,36170767	0,243601102	0,537737619	0,453011085	0,650540759	0,876831587
Camsap3	466,9176603	-0,036667305	0,102107612	-0,359104518	0,719516902	0,908631658
Hddc2	22,82374683	-0,491144291	0,314421879	-1,56205507	0,118275016	0,427187007
Tmem253	0,31434569	0,667660234	3,262174889	0,20466721	0,837832141	NA
Zfp872	16,62043272	-0,884387398	0,553690794	-1,597258628	0,110208069	0,410753184
Cdkl1	4,764684573	1,800226045	1,067516451	1,686368434	0,091724857	NA
Parp11	129,0741662	0,460807326	0,178030082	2,588367777	0,009643196	0,102236232
Tcerg1l	0,086476712	0,780932884	3,352475198	0,232942181	0,815806304	NA

Supplementary Table S1: *Serpina1* KO vs. wildtype all DEGs

Arhgap27	48,82143057	0,119784291	0,293073445	0,408717656	0,682746875	0,891903322
2610318N02Rik	0,173498105	0,059593471	3,352475198	0,017775962	0,985817581	NA
Arpc3	1969,235357	0,08183883	0,074715253	1,09534301	0,273366391	0,634872238
Kctd2	442,6500559	-0,070550735	0,1272953	-0,55422891	0,579422195	0,84530168
Tmem45a2	0,244992664	1,389394708	3,349408814	0,414817893	0,67827522	NA
Fam25c	728,0350142	-0,116563255	0,192470512	-0,605616173	0,54476966	0,827491596
Nbeal2	369,836748	-0,428246498	0,136742618	-3,131770511	0,001737556	0,034774537
Tefm	225,3076401	0,009002696	0,136548897	0,0659302	0,947433397	0,985164724
Coa4	126,7912205	0,024405133	0,185781952	0,131364389	0,895487058	0,970631302
Tespa1	1,887595406	0,101112329	1,101726277	0,091776271	0,926875797	NA
Gm10791	0,728525702	0,307346381	1,758787301	0,174749033	0,861276838	NA
Irf9	826,4793631	0,104958288	0,108866887	0,964097444	0,334997074	0,691456742
Higd1a	1272,494671	-0,03555843	0,132688042	-0,267985191	0,788710718	0,935202374
Mär-07	326,9894018	0,120911571	0,169688449	0,712550395	0,476124017	0,789577814
Trip4	1395,201393	-0,114944264	0,133096647	-0,863615023	0,387799409	0,731859161
Acpp	254,1426806	2,096576785	0,467414986	4,485471899	7,28E-06	0,000631579
Vapb	1829,97141	-0,095733511	0,066140496	-1,447426558	0,147777492	0,477899684
Dnajc7	1521,771426	-0,014909963	0,093550102	-0,159379438	0,873369938	0,963134761
Dbil5	7,122704019	-0,460462279	0,540214557	-0,852369254	0,394009179	0,736368577
Hs1bp3	360,5015456	0,15266638	0,129316964	1,180559571	0,237777732	0,594996111
Pnma1	0,962413107	0,468789008	1,712301187	0,273777191	0,784255853	NA
Capn7	807,2698164	-0,105483174	0,092831469	-1,136286818	0,255836546	0,616366745
Eif2b1	546,4239296	-0,075717239	0,103610583	-0,730786734	0,464909428	0,781663619
Myom1	13,49826991	0,319600732	0,510618196	0,625909406	0,531374365	0,819884984
Prdm14	1,400701689	0,645113634	1,601717573	0,402763661	0,687122096	NA
Gm10789	0,209517726	0,059593471	3,352475198	0,017775962	0,985817581	NA
4933428G20Rik	0,537122014	1,461108722	2,893238596	0,505008029	0,613553196	NA
Acadl	5052,488858	0,101012898	0,102918196	0,981487261	0,326352513	0,684178572
Sox17	40,48988378	-0,458192247	0,364353058	-1,257550159	0,208554488	0,560998984
Rad9b	30,43054789	0,445322998	0,299204594	1,48835615	0,136656994	0,459349518
Msto1	200,1854187	0,248993666	0,154539273	1,611199925	0,107136154	0,406189471
Taok1	858,9572097	-0,036377143	0,11004198	-0,330575143	0,740965425	0,916370654
Wrap53	190,1871573	-0,010257209	0,128629655	-0,07974218	0,936442311	0,982191945
Sde2	264,4113531	0,027450475	0,133440295	0,205713539	0,837014683	0,951141594
Tspan10	0,735801845	0,354711831	1,629115252	0,217732803	0,827637303	NA
Rnf144b	1394,404185	0,051222369	0,14776503	0,346647436	0,728856204	0,911513433
Xlr4a	11,66967891	-1,824555643	1,648023473	-1,10711751	0,268243126	0,628596381
Vps4a	522,0339564	-0,094411281	0,089955945	-1,049527978	0,293935185	0,655154967
Zcchc14	701,4827618	-0,022557318	0,110707272	-0,203756424	0,838543847	0,951141594
Pask	14,95567948	-0,253386409	0,424870544	-0,596384975	0,550918076	0,830351413
Rfxap	214,0885022	-0,162372512	0,129042468	-1,2582874	0,208287836	0,560507263
Wdr5	778,4177019	-0,022205599	0,073667906	-0,301428403	0,763087839	0,925576084
Pcdhga3	2,905360611	-0,504924111	1,037645996	-0,486605367	0,626538019	NA
Ptges2	453,2852589	-0,015549454	0,103309666	-0,150513057	0,88035985	0,96546599
Serpinc1	64277,25728	0,080297958	0,059616194	1,346915194	0,178007547	0,520240034
Socs4	115,4900676	-0,233582196	0,160810021	-1,452535069	0,146352885	0,476248605
Btnl2	0,901289238	-1,437261872	1,790831035	-0,802566989	0,422225055	NA
Lnx2	637,5823522	-0,006666101	0,113999729	-0,058474707	0,953370509	0,986479135
Setd7	191,1619456	-0,34909057	0,200286039	-1,742960073	0,081340589	0,352860369
Txlng	379,819061	-0,408576978	0,141281326	-2,891939004	0,003828723	0,057613111
Nudt6	262,5179594	0,228442137	0,11408519	2,002382063	0,045243656	0,251865618
Tmem220	460,9891054	0,033876772	0,111777538	0,303073165	0,761834104	0,925400703
Lzts3	768,3252317	0,052347321	0,124999962	0,418778698	0,675377874	0,889164486
2210011C24Rik	5,15750825	1,563509777	0,815088695	1,918208149	0,055084623	NA
Bglap3	13,28118162	-2,509348149	0,694525262	-3,613040856	0,000302627	0,010095144
Hikeshi	349,4370646	-0,312714206	0,111561182	-2,803073603	0,005061812	0,06962074
Chil3	4,219968173	1,364362175	0,750563705	1,817783308	0,069097259	NA
Tmem218	152,545703	0,543348338	0,230348544	2,358809518	0,018333664	0,150144298
Cox16	189,7277074	0,116317633	0,131452176	0,884866553	0,376228609	0,722097522
Gmpr	20,13084591	-0,638727419	0,478930617	-1,333653344	0,182317492	0,527224185
Kif1b	3448,663482	0,103106449	0,120197753	0,85780679	0,39099916	0,734518115
Cib1	1371,424751	-0,099529879	0,103280359	-0,963686415	0,335203167	0,691456742
Ctdspl2	170,2051851	0,136515809	0,185882505	0,73441989	0,462692868	0,781516914
Tlcd1	927,6626717	0,447668465	0,148258514	3,0195127	0,002531817	0,043901243
Syngr4	0,989715111	-1,120152245	2,101102498	-0,533125941	0,593946403	NA
1600002D24Rik	0,086476712	0,780932884	3,352475198	0,232942181	0,815806304	NA

Supplementary Table S1: *Serpina1* KO vs. wildtype all DEGs

Taf1b	123,433673	0,65924204	0,189851915	3,47240131	0,000515825	0,014959977
Tmem37	1555,371304	-0,210703617	0,126152819	-1,670225203	0,094874815	0,383176023
Plin4	69,64153074	-0,479550873	0,233273694	-2,055743466	0,03980725	0,234944905
Rpp30	123,0900322	0,084294333	0,165002069	0,510868339	0,609443252	0,857753956
Tubd1	92,30627238	-0,160079778	0,216038833	-0,74097687	0,458707465	0,779631911
Ptp4a3	161,9584121	0,135869478	0,196286978	0,692198124	0,488812917	0,796400974
Mir704	0,269274339	0,059593471	3,352475198	0,017775962	0,985817581	NA
Icam1	325,0283275	-0,034657143	0,151451832	-0,228832776	0,818998891	0,945934875
Smad2	495,1127196	-0,028954795	0,099863918	-0,289942507	0,771860222	0,928273961
Bcl2l11	108,4285201	0,034292945	0,239346401	0,143277464	0,886071054	0,96772371
Mxd4	479,5950301	-0,32866044	0,121886619	-2,69644398	0,007008418	0,084455842
Mad1l1	251,0166181	-0,265433671	0,117970673	-2,249997093	0,02444913	0,17783483
Mxd1	102,5769776	-0,294690946	0,170887363	-1,724474773	0,084622169	0,360024318
Bex2	2,24879792	-0,161453056	1,087249184	-0,148496829	0,881950688	NA
M6pr	2693,72307	0,036511291	0,074705381	0,488737092	0,62502784	0,864383696
Nr1i2	1750,42761	-0,340388886	0,104475484	-3,258074282	0,00112171	0,025810923
Cnp	393,6131434	0,08633879	0,166425464	0,518783532	0,603911703	0,855545543
Pik3c2a	705,7440898	-0,312064849	0,159956562	-1,950934965	0,051064783	0,272528974
Ppp2r3d	39,89531197	0,261208865	0,260332809	1,003365141	0,315684717	0,67419376
Ppara	3863,247723	-0,125498837	0,172743951	-0,726502068	0,46753104	0,782859415
Mir6976	0,142634598	-0,517475177	3,352475198	-0,154356154	0,877328924	NA
Khsrp	1054,336943	-0,246358934	0,087066985	-2,829533319	0,004661595	0,065463086
Wnt6	0,594265924	2,661063949	2,708283389	0,982564808	0,325821672	NA
Igfbp4	26370,92779	-0,012954422	0,093368079	-0,138745729	0,889651081	0,968877905
Vipr2	0,533602961	-0,986753547	2,354642883	-0,419067178	0,675167037	NA
Vamp3	205,7601311	-0,044345862	0,151665989	-0,292391607	0,769987224	0,927934035
Cldn20	2,719026296	-1,034012903	0,942554328	-1,097032683	0,272627105	NA
Mrpl11	792,3406199	0,076515204	0,104364732	0,733151923	0,463465775	0,781663619
Ndufa12	1296,844647	0,109880047	0,090064069	1,220020903	0,222456951	0,577161019
Tmem230	330,1527712	0,109534861	0,120139967	0,911727075	0,361912407	0,71109038
Zfp398	56,26774732	-0,362568842	0,238303021	-1,521461372	0,128144098	0,445315232
Ccdc169	2,452086199	-1,348139476	0,975429634	-1,382098133	0,166941572	NA
Flii	1325,76332	-0,089296822	0,096616396	-0,924240864	0,355360924	0,705273655
Asb3	167,4038574	0,091818568	0,147094253	0,624215875	0,532485823	0,820611292
Sapcd1	1,806901038	1,042009868	1,103550536	0,944233938	0,345050116	NA
1110059E24Rik	346,003612	0,060661027	0,144055615	0,421094497	0,67368608	0,888626355
Acyp1	139,088296	-0,036356818	0,181644093	-0,200154145	0,841360028	0,951912623
Icam4	13,4213788	0,068148372	0,493792447	0,138010155	0,890232392	0,968877905
Gcnt2	494,0470438	0,009261579	0,137536072	0,067339273	0,946311613	0,984653942
Cir1	237,0446318	0,1167929	0,164975985	0,707938797	0,478983265	0,791242713
Cep70	72,10216907	0,483673728	0,199106908	2,429216203	0,015131506	0,13432477
Kcnq5	2,101621825	0,004175599	1,087317311	0,003840276	0,99693591	NA
Klf9	1596,704191	-0,01228629	0,238410904	-0,051534094	0,958899935	0,987896001
Med7	301,4378329	0,012470747	0,19081951	0,065353627	0,947892445	0,985486748
Rpl3l	1,065477013	-1,598309162	1,434255958	-1,114382097	0,265115301	NA
Ndufb4	389,675626	0,385066487	0,16999859	2,265115776	0,023505576	0,173329312
Cd302	5827,397621	0,369237402	0,090933179	4,060535512	4,90E-05	0,002628715
1110059G10Rik	209,3197411	0,094115904	0,133804009	0,703386279	0,481815055	0,793477208
Cisd2	709,1047405	0,007238622	0,111851227	0,064716515	0,948399713	0,985571619
Klc1	316,9281336	-0,194968131	0,157481411	-1,238039013	0,215701595	0,569610871
Cdc42ep4	939,3696039	0,383705754	0,114216268	3,359466752	0,00078093	0,020138259
Prkacb	964,9781793	0,01182314	0,14190776	0,083315666	0,933600544	0,981500765
Naaa	90,15199993	0,427770678	0,161711426	2,645271826	0,008162532	0,092872243
Tbl1xr1	583,8539479	0,262012044	0,113686369	2,304691814	0,02118384	0,16249252
Trim23	199,2122858	0,050156107	0,185463553	0,270436463	0,786824491	0,934196858
Uck2	139,8102146	-0,141624645	0,16871959	-0,839408423	0,401240159	0,741933566
Ngrn	443,3600201	-0,107083846	0,107589387	-0,995301202	0,319589793	0,677329731
Snx27	665,6681683	-0,299965006	0,101753079	-2,947969833	0,003198683	0,051584065
Rcc1l	376,7033357	-0,04069554	0,113500894	-0,358548188	0,719933113	0,908788588
Hnrnpa0	755,1191476	0,240420993	0,118619737	2,026821153	0,042680706	0,243203202
Cnrip1	18,65240116	0,284567388	0,351132941	0,81042635	0,417695179	0,752229411
Ifi27l2a	151,3317158	0,525192872	0,201220512	2,610036454	0,009053257	0,098887698
Bicd2	446,5752537	-0,349877977	0,103538621	-3,3792026	0,000726964	0,019131464
Tspan4	895,290104	-0,117265611	0,192570198	-0,608949945	0,542557618	0,826611597
Hsd17b11	3518,533008	-0,271277114	0,099023246	-2,739529599	0,006152717	0,078115555
Rtn3	2391,341838	-0,024913208	0,060654689	-0,410738364	0,681264393	0,89166825

Mpv17l	658,9913838	0,033119062	0,120458401	0,274941907	0,78336087	0,932984541
Uqcr11	4146,130585	0,024937332	0,110907136	0,224848758	0,822096934	0,946779723
Reg4	1,015484864	2,532213818	1,695123508	1,493822608	0,135221991	NA
Lppos	298,8341392	0,53699882	0,184276253	2,914096699	0,003567194	0,055582536
Zfp644	292,935175	0,117291179	0,142237278	0,824616308	0,40958946	0,746937865
Tnfsf13b	7,748054442	0,382977737	0,564460819	0,678484182	0,49746475	0,801288247
Pcdhga11	0,804307793	-1,660105021	1,538500848	-1,079040693	0,280569587	NA
Prickle3	97,65460908	-0,189680464	0,21414394	-0,88576153	0,375746044	0,721655743
Plppr2	201,4520556	-0,136300796	0,161393102	-0,844526772	0,39837511	0,739635153
Zcchc5	1,140987365	1,93511131	1,827617134	1,058816573	0,289683324	NA
Mms22l	36,62617122	0,087739892	0,296624036	0,295794952	0,767386678	0,927586634
Aup1	4270,812266	0,091706949	0,06819634	1,344748846	0,178706355	0,521301605
Xiap	2136,815126	-0,33242725	0,134877278	-2,464664593	0,013714156	0,126969911
Clasrp	257,588562	0,087949518	0,160755074	0,547102593	0,584308251	0,847068435
4930445N18Rik	0,142634598	-0,517475177	3,352475198	-0,154356154	0,877328924	NA
Eif4e	959,2826351	-0,122772916	0,108196609	-1,134720545	0,256492426	0,616948518
Kat6a	595,9127215	-0,127474401	0,165937702	-0,768206382	0,442364583	0,769135977
Cdipt	1106,070536	-0,166359208	0,088597676	-1,877692678	0,060423219	0,29803066
Ggct	219,1304058	0,347769379	0,166572484	2,087796088	0,036816233	0,224952566
Cmtm8	1217,156933	0,222445732	0,095121333	2,338547262	0,019358877	0,155304678
Sugt1	596,8508794	-0,101348622	0,098192447	-1,032142742	0,302005253	0,662128551
Pex11g	957,0189137	0,169442614	0,112818656	1,501902431	0,133122309	0,454008201
A930018P22Rik	3,583342936	1,660663886	0,847275564	1,960004461	0,049995269	NA
Fam195a	606,6606411	-0,077421177	0,120690205	-0,64148683	0,521206435	0,814845193
Abhd18	404,1435177	-0,14853148	0,122777147	-1,209764876	0,226369128	0,581402791
Efcab2	61,53012958	0,565739736	0,236570635	2,391419946	0,016783344	0,143473504
Fam166a	2,803031002	0,607571245	1,310783182	0,46351773	0,642993309	NA
Nudt21	142,7199782	0,208406608	0,143470976	1,452604661	0,146333551	0,476248605
Fam98b	317,9971764	0,025150368	0,113107982	0,222357145	0,82403587	0,947472731
3830403N18Rik	0,661033583	1,884973485	1,979739172	0,952132237	0,341029922	NA
Tmbim4	1237,981809	0,055858287	0,108631127	0,514201485	0,607111129	0,857252713
Stx18	639,2074267	-0,329733419	0,11515207	-2,863460643	0,004190409	0,060944211
Ovol2	1,079524352	1,85495947	1,490626134	1,244416307	0,213346384	NA
LOC106740	17,39820441	0,326791283	0,368270173	0,887368313	0,374880637	0,720996253
Irf8	136,5251561	0,489951496	0,224377443	2,183604065	0,028991355	0,19654433
Rbbp7	1586,030385	-0,052077213	0,094145132	-0,553158853	0,58015464	0,845309347
Nfkb1	652,800095	-0,011844299	0,110141769	-0,107536847	0,914363095	0,976392941
Ptprv	2,206381528	0,741744008	1,120239893	0,662129614	0,507888159	NA
Rsbn1l	197,9941466	0,100404814	0,159274538	0,630388353	0,528440529	0,818528726
Cpn1	8807,749893	0,026850497	0,073503577	0,365295112	0,714891127	0,906371815
Prss23	121,3715711	0,069205898	0,328937575	0,210392195	0,833361584	0,950519804
Slc35e3	301,87345	-0,392095733	0,215497268	-1,819492825	0,068836276	0,320987855
Nubpl	195,2110986	0,16937643	0,135512814	1,249892345	0,211338876	0,564073259
Klhl24	836,1046677	0,028658825	0,201831578	0,14199376	0,887084934	0,968354576
Polr2k	214,5659583	0,054448955	0,180454851	0,301731734	0,762856575	0,925557106
Arhgef17	91,44525527	-0,008686472	0,237393822	-0,036590978	0,970811137	0,992007633
Nubp1	526,8236763	-0,132063503	0,137504381	-0,960431238	0,336838223	0,69270916
Thbs2	47,15878211	0,009667428	0,262302689	0,036855999	0,970599823	0,992007633
Gspt2	12,54018879	-0,041598731	0,484070141	-0,085935338	0,93151782	0,980584977
Rhod	637,9045417	0,065528572	0,136986821	0,478356762	0,632396303	0,867306747
Ankrd27	691,8743986	0,026558349	0,092707681	0,286474089	0,774515037	0,929237326
Mthfr	649,3245227	-0,568352401	0,17347459	-3,276286167	0,001051819	0,024713132
Btf3	2623,292	0,043631592	0,074216879	0,587893111	0,556604041	0,833822555
Gm10509	57,94654132	0,089205269	0,215995529	0,412995906	0,679609613	0,891454172
Arsi	1,364637153	0,188213388	1,213435105	0,155107914	0,876736243	NA
Itga6	63,54445684	-0,396729218	0,321911848	-1,232415707	0,217793836	0,571609443
Csnk2a2	365,5536282	-0,101256434	0,110447824	-0,916780708	0,359257564	0,708603477
Zfp953	83,71706398	0,104586542	0,191152389	0,547136987	0,584284623	0,847068435
Dbi	24220,46661	0,209449065	0,130728355	1,602170128	0,109117994	0,40910239
Cd1d2	2,558458137	0,026833319	0,906888743	0,029588325	0,976395377	NA
Homer3	45,17116089	-0,089985094	0,273577007	-0,328920531	0,742215756	0,916899135
Clpb	1103,791882	-0,152151615	0,093928562	-1,619865273	0,105261221	0,403011718
Fdft1	5901,133389	0,687151271	0,148912326	4,614468719	3,94E-06	0,000390278
Gstm1	60813,58855	-0,06005125	0,137857077	-0,435605126	0,663123251	0,883701629
Six1	0,087021394	-0,517475177	3,352475198	-0,154356154	0,877328924	NA
Alas2	2746,666919	-1,067492218	0,480860062	-2,219964396	0,026421185	0,187387171

Fcgrt	2879,498117	-0,101057062	0,119992089	-0,842197705	0,399677298	0,740682811
Vps4b	1228,389525	0,065689825	0,100827445	0,651507379	0,514719015	0,812039132
C2cd3	274,5780994	-0,269660158	0,107740942	-2,502856878	0,012319535	0,119329441
C330006A16Rik	1661,281864	-0,037381416	0,08227483	-0,454348144	0,64957827	0,87669042
Dctn2	2841,254673	0,03785371	0,072706098	0,520640101	0,602617509	0,854608409
Exosc8	235,1897015	0,541743369	0,155992594	3,472878778	0,000514908	0,014959977
Arhgef12	2556,213568	-0,108971406	0,122465237	-0,88981501	0,373565226	0,720823634
Furin	4338,517756	0,0109211	0,084453783	0,129314518	0,897108784	0,971044462
Dmpk	557,9155767	0,180321448	0,207037327	0,870961054	0,383775419	0,72829995
Fau	3563,110409	0,123605167	0,095593092	1,293034517	0,195999121	0,54489515
Maml3	71,83768309	-0,373586678	0,292020395	-1,279317077	0,200785422	0,55187095
AA467197	0,086476712	0,780932884	3,352475198	0,232942181	0,815806304	NA
Sgpp2	6,117578075	-0,028958474	0,78754176	-0,036770715	0,970667824	0,992007633
Gnptab	294,7137046	0,170535016	0,150905412	1,130078859	0,258442997	0,619531008
1810053B23Rik	37,01654086	5,038046699	0,66001341	7,633248994	2,29E-14	3,40E-11
D16Ertd519e	0,093303375	-0,517475177	3,352475198	-0,154356154	0,877328924	NA
Platr17	1,573622746	-0,383375689	1,197980661	-0,320018262	0,748954487	NA
9230112J17Rik	0,336157466	0,663936047	2,673207616	0,24836681	0,803850611	NA
1700034P13Rik	18,82141331	0,008002684	0,329137107	0,024314136	0,980602038	0,994739255
4933417E11Rik	0,12663974	0,780932884	3,352475198	0,232942181	0,815806304	NA
A430035B10Rik	21,65963362	-0,221768904	0,356890589	-0,621391852	0,534341832	0,821767588
Gm10007	0,220592835	0,059593471	3,352475198	0,017775962	0,985817581	NA
C920021L13Rik	11,64556094	0,559649974	0,460161371	1,216203725	0,223907325	0,5790477
Gm13003	0,093303375	-0,517475177	3,352475198	-0,154356154	0,877328924	NA
AV051173	8,789910189	0,25533939	0,7650571	0,333752069	0,738566655	0,915438384
Tab3	199,9559737	-0,116140166	0,156886051	-0,740283569	0,459127949	0,780132378
1810037I17Rik	441,699662	0,023241085	0,100678389	0,230844825	0,817435356	0,945190562
Lsm5	71,46189205	0,197087236	0,205914685	0,957130552	0,338501365	0,694071514
Chmp4c	20,36795927	1,115072866	0,414909524	2,687508486	0,007198727	0,08600517
Abhd15	705,6458536	-0,087325097	0,16731461	-0,521921529	0,601724968	0,854462419
Lsm1	325,9344175	0,0755216	0,098132305	0,769589579	0,441543388	0,768930234
Oma1	482,3423068	-0,094540326	0,097780718	-0,966860619	0,333613716	0,690201449
Rtca	208,8674119	0,0716126	0,126186188	0,567515362	0,570364092	0,841196193
Ergic3	2170,807773	0,021337113	0,068502049	0,31148138	0,755434696	0,922462845
Ccdc90b	204,134863	0,234178817	0,164698776	1,421861308	0,155066517	0,489743509
Exosc3	318,8369225	0,100580486	0,103915453	0,967906922	0,333090857	0,68995097
Chpf	64,11229967	-0,518595079	0,283379508	-1,83003733	0,067244357	0,317560326
Cmss1	84,52927842	-0,029550026	0,183184035	-0,161313326	0,871846628	0,962328069
Adprm	325,8832046	0,468024991	0,1317213	3,553145852	0,000380653	0,012087778
Psenen	717,4280587	-0,021094246	0,104745742	-0,201385236	0,840397359	0,951657825
Abhd14a	372,4367809	-0,156963718	0,157843923	-0,994423578	0,320016694	0,677494232
Adh6a	1,021082467	-1,864540659	1,707262585	-1,092122955	0,274779061	NA
Ndufb2	1002,427893	0,04738515	0,117242649	0,404163076	0,686092804	0,893161227
Rnaset2b	73,54368853	-0,340139007	0,330123293	-1,030339313	0,30285075	0,662675256
Leprotl1	492,9207355	0,230703616	0,102496102	2,250852571	0,024394877	0,177536802
Entpd6	250,5130963	-0,080928336	0,118591593	-0,682412087	0,494978415	0,80006558
Tns2	1126,124253	-0,115315785	0,181443392	-0,635546899	0,525071789	0,816743321
Mtmr14	529,3448512	-0,200816568	0,098093539	-2,047194657	0,040638976	0,237457372
Fam212a	9,343834838	-0,286910209	0,52959616	-0,541752813	0,587988802	0,848706754
Sympk	1782,858155	-0,185603866	0,083802591	-2,214774807	0,026775525	0,18930315
Rpl39l	0,256296747	0,059593471	3,352475198	0,017775962	0,985817581	NA
Fam135a	572,6322846	-0,219097262	0,145038398	-1,510615567	0,130886425	0,449709746
Denr	989,4028912	0,057767942	0,095472852	0,605071916	0,545131213	0,827491596
B230118H07Rik	301,4894935	0,127589554	0,133833678	0,953344156	0,340415726	0,694720147
Bcas2	583,1472386	0,055567441	0,074716757	0,743707876	0,457053224	0,77843077
Rfc3	135,1179046	0,237965794	0,161188052	1,476324031	0,139856926	0,464879971
Snora78	2,533838771	-0,570096781	0,875533896	-0,651141873	0,514954908	NA
Slc8a1	31,71951421	0,429177624	0,345335007	1,242786325	0,213946583	0,567220469
Rgs3	210,2453752	-1,043970646	0,26546549	-3,932603991	8,40E-05	0,00396963
Wdr86	5,776642909	1,150444361	0,779110606	1,476612374	0,139779573	0,464854007
Tbc1d9	39,22215401	0,199346579	0,357856422	0,557057431	0,577488191	0,84496439
Atxn7l3	561,3564969	-0,123198078	0,101633034	-1,212185384	0,225441425	0,580946341
Fbxo47	7,68913297	-0,472847239	0,624109068	-0,757635586	0,448669171	0,77297141
Mettl8	280,2822421	0,081296558	0,128935896	0,630519196	0,528354948	0,81852028
Zfp426	207,3968174	0,035670291	0,118182018	0,301825028	0,762785451	0,925557106
Arhgef7	689,1587866	-0,052947282	0,129680457	-0,408290377	0,683060503	0,892129335

Kpna2	759,2709445	-0,091926555	0,143109627	-0,642350604	0,520645564	0,814475842
Pura	142,97623	-0,051741529	0,150578141	-0,343619125	0,73113274	0,912061199
Rasa3	201,7801279	0,377135816	0,225502161	1,672426617	0,094440227	0,3821342
Dtnbp1	804,5136792	0,051964282	0,083579321	0,621736107	0,534115405	0,821607278
Aptx	249,8459477	0,012681744	0,112193329	0,113034742	0,910003012	0,9747592
Mrps15	840,1383302	0,002688034	0,113115058	0,023763717	0,981041081	0,994882668
Rtfdc1	1667,848801	-0,101472542	0,096970726	-1,046424488	0,295365092	0,656481448
Asf1a	167,8061383	0,195404123	0,175037536	1,116355536	0,264269978	0,625203564
Nudt2	278,2783579	0,342815655	0,112764795	3,040094711	0,002365038	0,042463022
Alkbh7	249,5938884	0,121969751	0,118591425	1,028487103	0,303720754	0,66325429
Tsfm	339,7627387	-0,113081102	0,149227683	-0,757775634	0,448585312	0,77297141
Grtp1	424,1604042	-0,194065844	0,106356397	-1,824674864	0,068050109	0,319375503
Cdkal1	241,4822188	-0,051954685	0,123719617	-0,419938938	0,674530062	0,888627551
Gca	175,2291548	0,193661218	0,174890506	1,107328367	0,268151984	0,628492966
Rabepk	238,0154645	-0,059739157	0,141031015	-0,423588794	0,671865729	0,887904019
Plpp7	17,58274417	0,876591085	0,445079711	1,969514819	0,048894003	0,264749107
Farp2	488,8986869	-0,093137317	0,087616473	-1,063011482	0,287776744	0,649221616
Gm17801	3,296489598	-0,15334405	0,844910758	-0,181491416	0,85598187	NA
Rps16	4059,875579	0,081133783	0,127063781	0,638528009	0,523130027	0,815322684
Ptger1	3,728027775	1,314072097	0,823722306	1,595285313	0,110648448	NA
Psmb10	734,6106383	0,123646868	0,120581309	1,025423167	0,305163562	0,664343211
Rgs9	12,80384075	0,221209081	0,447270781	0,494575301	0,620899941	0,862608071
Rgs16	308,9230519	4,479710346	1,136632773	3,941211668	8,11E-05	0,003898701
Serpinb6b	86,73787975	0,147494708	0,209885198	0,702739924	0,482217842	0,793477208
Rsl1d1	1079,950575	0,079444105	0,119009708	0,667543065	0,504425311	0,804997854
Spop	2955,075474	-0,136127322	0,08064066	-1,688073002	0,091397216	0,375273152
Rps13	2286,980808	0,049948784	0,109097868	0,457834651	0,647071239	0,876230372
Fam122a	220,7646118	-0,047431864	0,13107377	-0,361871519	0,717448044	0,907650451
Ankrd13d	9,923586482	0,687528364	0,568113464	1,21019551	0,22620388	0,581226154
Ankrd13a	572,4206747	-0,085789801	0,106328814	-0,806834926	0,419761594	0,753867914
Ccdc163	94,23583732	-0,026297544	0,20296201	-0,129568801	0,896907589	0,971044462
Zfp414	141,5559873	8,47E-05	0,168679489	0,000501852	0,99959958	0,999875741
Tmem129	631,8151882	-0,115021959	0,092233033	-1,247079873	0,212368212	0,565238523
Cacna2d1	15,95899858	0,455972237	0,446603231	1,020978366	0,307264689	0,66646434
Kif19a	10,21633058	0,783940215	0,513834162	1,52566776	0,127092627	0,44395944
Klrb1c	8,7233748	0,393564367	0,505807213	0,778091646	0,436514987	0,76575019
Cpeb2	727,878116	0,014895568	0,202401047	0,073594323	0,941333189	0,983128178
1700092M07Rik	1,582872798	-0,413654779	1,117056838	-0,370307727	0,711153217	NA
Lsm10	308,9095375	0,019407717	0,160717466	0,120756741	0,903883715	0,973561919
Rnf20	607,2632616	-0,170564149	0,093304678	-1,828034277	0,067544407	0,318406623
Cmip	1079,869542	-0,253719339	0,128451066	-1,975221751	0,048242989	0,262820096
Tmem170b	411,671017	0,006874608	0,095504334	0,071982154	0,94261611	0,983437952
Senp3	789,8572234	-0,121710221	0,086507082	-1,406939389	0,159445362	0,495312554
Kif9	23,2425632	-0,565610214	0,328613575	-1,721201609	0,085214246	0,362004847
Lyrm5	1116,919874	0,465859084	0,11201926	4,158740973	3,20E-05	0,001944621
Fam151b	79,44322839	0,128847695	0,184714147	0,697551852	0,485457502	0,79430833
Noxa1	2,457778105	1,290261164	1,083781151	1,190518181	0,233842788	NA
Sult6b1	0,12663974	0,780932884	3,352475198	0,232942181	0,815806304	NA
Zfp605	73,52559297	-0,64993099	0,237330201	-2,73850941	0,006171839	0,078283986
C530008M17Rik	0,187906191	-1,241948083	3,339949325	-0,371846385	0,710007226	NA
Apol6	0,547951967	1,563538293	2,868930064	0,544990034	0,585760372	NA
Cbwd1	139,2647133	-0,227146205	0,162854038	-1,394783997	0,163080986	0,49993802
Serpinb1b	0,810458721	-0,952287299	1,555685269	-0,612133648	0,540449337	NA
Sntg2	135,928762	0,325114586	0,230654476	1,40953079	0,158678276	0,494953305
Serpinb1c	2,361745188	0,734108789	1,046948525	0,701188999	0,483185074	NA
Gga3	237,4394175	0,107142218	0,131552004	0,81444763	0,415388562	0,751551608
Gse1	500,1753368	-0,531374771	0,205575446	-2,584816329	0,009743088	0,103046417
4930590J08Rik	1,714857542	1,937677173	1,215106611	1,594656104	0,110789159	NA
4430402I18Rik	56,4739831	0,400141672	0,252079055	1,587365804	0,11242982	0,415213883
B4galnt3	37,35220022	-0,202946868	0,2513667	-0,807373722	0,419451202	0,753719022
Map4k3	656,4454294	-0,020183182	0,148258174	-0,136135378	0,891714261	0,969104575
Spg20	899,4440768	-0,058247304	0,132954523	-0,438099451	0,661314192	0,882666274
Zfp383	61,22874789	-0,034505552	0,231487377	-0,149060188	0,88150614	0,965734294
Tdrd7	827,4939515	-0,103256013	0,101291714	-1,019392491	0,30801667	0,667059844
Ints7	448,4234306	-0,049712719	0,144431479	-0,344195873	0,730698986	0,911941257
Smarca4	1487,267462	-0,237573885	0,076829913	-3,09220556	0,001986752	0,038104827

Supplementary Table S1: *Serpina1* KO vs. wildtype all DEGs

Kctd21	274,5574369	-0,113936131	0,12577874	-0,905845701	0,365017528	0,713569421
Pds5a	757,8120452	-0,290352989	0,143924322	-2,017400426	0,043653743	0,246143774
Kif5a	41,99361346	0,285947681	0,26114532	1,094975319	0,273527449	0,635079623
Rcsd1	105,6158256	0,019514991	0,265486656	0,073506487	0,941403082	0,983128178
Macc1	2,741954257	1,603829111	1,153990705	1,389811117	0,164586241	NA
Txndc11	746,3398458	-0,367642747	0,147020398	-2,50062408	0,01239747	0,119842206
Trmt5	187,3080573	0,157657338	0,148359804	1,062668818	0,287932166	0,649353093
Tgds	575,594567	0,143408844	0,087930314	1,630937484	0,102903511	0,399190747
Rab1b	2623,729726	0,031948775	0,083483528	0,382695556	0,70194551	0,900259931
Tgfbr2	674,3836293	0,133251372	0,142619895	0,934311244	0,350143366	0,701844955
Idh3a	893,5531229	0,143573155	0,128270235	1,119302186	0,263011243	0,622997398
Mettl5	236,5611834	0,121078381	0,12714768	0,952265751	0,340962223	0,694866457
Ranbp17	3,007413031	-0,060574683	0,93855328	-0,064540484	0,948539873	NA
Golga1	467,0067069	-0,161444471	0,106121832	-1,521312515	0,128181431	0,445315232
Klhdc9	1,226080728	0,41852035	1,351589409	0,30965051	0,756826741	NA
Mief2	409,1019529	-0,408483498	0,1577672	-2,589153493	0,00962122	0,10216528
Larp4	1076,122965	-0,077104463	0,185819744	-0,414942255	0,678184176	0,89063303
1110004E09Rik	306,6648853	-0,238227294	0,139271938	-1,710518989	0,087169945	0,366124724
Fam134c	2236,162079	-0,02604825	0,091497528	-0,284688024	0,775883167	0,929745022
Ddx59	126,1157863	0,226431271	0,1798693	1,258865578	0,208078889	0,56023257
Mrpl19	370,2899291	-0,074476743	0,129814349	-0,573717346	0,566159088	0,838041311
Atad1	1318,4042	0,038504436	0,085428304	0,450722232	0,652189757	0,877662367
Far1	41,96354203	-0,163142956	0,309741879	-0,526706161	0,598397657	0,853968646
Comtd1	107,2688413	-0,16939835	0,263979948	-0,641709159	0,521062041	0,814845193
Trabd	2195,028828	-0,424500632	0,088355958	-4,804437003	1,55E-06	0,000185241
Mir7079	1,464532015	0,68126856	1,425022816	0,478075545	0,632596437	NA
Tmem240	0,528006824	0,542058679	2,443894458	0,221801182	0,824468659	NA
Sh3bp2	544,9850913	0,067021739	0,146680232	0,456924138	0,647725573	0,87637316
Isoc2a	2780,905013	0,028697397	0,097996847	0,292840002	0,769644451	0,927934035
Gm8817	0,086476712	0,780932884	3,352475198	0,232942181	0,815806304	NA
Sult2a3	5,34212096	5,830349135	3,233121275	1,803319034	NA	NA
Zfp185	4,627076018	0,435988134	0,744385727	0,585701899	0,558075858	NA
1700021F05Rik	475,1729215	-0,078374831	0,133180826	-0,588484343	0,55620724	0,833802026
Cdca5	7,192388764	-1,070309355	0,583610942	-1,833943262	0,066662417	0,316480771
Wdr13	694,7074718	-0,327576217	0,116430804	-2,813484114	0,004900781	0,067894858
Slc38a4	28256,11016	0,112610852	0,138874854	0,810880078	0,417434542	0,752195715
Mettl26	3263,967541	-0,186550612	0,132007439	-1,413182572	0,157602049	0,493669587
Ndufb10	3758,789671	0,022662696	0,094043295	0,240981516	0,809569443	0,94241441
Acbd6	594,9160717	0,146708662	0,126389263	1,160768394	0,245736099	0,605051632
Clcn3	1171,541584	-0,243486901	0,102752014	-2,369655764	0,017804654	0,147933805
Agpat4	15,38594478	-0,210919372	0,393479333	-0,536036721	0,591933172	0,85082836
Shisa5	788,7316221	0,260753486	0,113340454	2,300621519	0,021413034	0,163367723
Rgs8	0,113662148	0,780932884	3,352475198	0,232942181	0,815806304	NA
Dalrd3	970,0656924	-0,201200478	0,093227108	-2,158175689	0,030914175	0,203993881
Sfr1	967,7959545	-0,128343234	0,101384529	-1,265905514	0,205546912	0,558300827
Krtcap3	23,69198822	-0,142350487	0,335093427	-0,424808353	0,670976388	0,887361539
Tbc1d22bos	0,346582855	0,667207307	3,170053761	0,210471922	0,833299364	NA
Atpaf2	508,5736157	-0,239454514	0,081635146	-2,933228233	0,003354572	0,053262794
Mfap3	331,2895378	-0,186147423	0,110320071	-1,687339582	0,091538074	0,37573611
Gm6402	1,463123309	-0,433503059	1,321561094	-0,328023473	0,742893915	NA
Shank1	2,520768489	0,009784665	0,865942458	0,01129944	0,990984543	NA
Spcs2	2844,888822	-0,204174362	0,106174282	-1,923011458	0,054478611	0,28262497
Snrnp27	354,5861552	0,06264247	0,116644817	0,537036033	0,591242725	0,850384506
Mrps28	485,9445012	0,083919432	0,142811921	0,58762204	0,556786014	0,833822555
Rpl7l1	1677,700599	-0,159423831	0,100770689	-1,582045655	0,113639149	0,417177269
Mrnip	167,7673759	0,2324308	0,186036829	1,249380572	0,211525911	0,564073259
Iscu	2093,805585	-0,155948068	0,11452466	-1,361698592	0,173293026	0,515636426
Mri1	415,6936958	0,076932818	0,147962361	0,51994857	0,603099421	0,855110421
Rer1	2998,067458	-0,007386258	0,076572023	-0,096461578	0,923153988	0,977556092
Cfap157	0,122496332	0,780932884	3,352475198	0,232942181	0,815806304	NA
Tmem261	490,5773617	0,057391406	0,118852825	0,482877934	0,62918241	0,866097574
Fank1	0,47973703	1,303829658	2,481254228	0,525472015	0,599255103	NA
Sdhd	5608,894145	0,047751579	0,081940271	0,582760806	0,560054333	0,835463907
Mrpl57	508,6832482	-0,036388529	0,105291554	-0,345597795	0,729645003	0,911594858
Prpf38b	799,3628147	-0,022702472	0,102113578	-0,22232569	0,824060355	0,947472731
Chordc1	948,8269461	-0,765749771	0,181019102	-4,230215276	2,33E-05	0,001552851

Ndufb7	1882,110122	0,058684294	0,10026027	0,585319528	0,558332887	0,83440808
Tnfrsf22	21,77688704	0,186480018	0,463386637	0,402428563	0,687368652	0,893386271
Bzw2	224,9794657	0,130801751	0,131007722	0,998427794	0,318071962	0,675759967
Tmem107	35,92347321	0,059601834	0,285122147	0,209039651	0,834417285	0,95084595
Mrpl42	1626,469516	0,065029409	0,092095389	0,706109281	0,48012018	0,79208634
Atg12	786,8120486	-0,011379588	0,077513928	-0,146807003	0,883284357	0,966395158
Proz	8718,62408	0,042828135	0,0952723	0,44953397	0,653046508	0,877889274
Mettl21a	179,9377186	0,111587361	0,127600121	0,874508274	0,381841516	0,726456076
Psmc6	2306,062661	0,110898354	0,081463783	1,361320936	0,17341229	0,51587648
Ttl	129,8000599	-0,078447653	0,140625028	-0,557849863	0,57694691	0,844928702
Mrpl24	1542,809312	-0,030389822	0,100656061	-0,30191746	0,762714986	0,925557106
Mmachc	464,2768362	0,022307706	0,102955547	0,21667318	0,82846305	0,94762353
Eif4e3	123,3398576	0,765278216	0,257648837	2,970237414	0,002975697	0,048752562
Cog6	666,7041651	-0,252187854	0,128402112	-1,964047563	0,049524576	0,26689182
Lonp2	9741,472701	-0,092792081	0,152459143	-0,608635722	0,542765921	0,826611597
Fcmr	17,57343172	0,813436409	0,430589888	1,88912102	0,058875615	0,293376491
Acadsb	3284,113613	-0,188903814	0,103043514	-1,833243135	0,066766422	0,316862014
Appbp2	299,536307	0,07425859	0,146302262	0,507569667	0,611755167	0,858821257
Bzw1	2854,608902	-0,066337006	0,110030082	-0,602898821	0,546576	0,828173482
Pcyox1	4590,920272	-0,045674327	0,13123662	-0,348030351	0,72781739	0,910785759
Tmem246	6,156413302	0,102758281	0,615129165	0,167051551	0,867329487	0,961775826
Srfbp1	141,6053343	0,29918913	0,154783779	1,932948861	0,053242498	0,278635557
Crnkl1	373,7238666	0,028185573	0,154182028	0,182807123	0,85494936	0,957431562
Swt1	310,1552262	-0,156426773	0,123370572	-1,267942356	0,204818537	0,557844548
Ncbp3	330,9739596	-0,072014013	0,11313571	-0,636527692	0,524432537	0,816387819
Hypm	0,274382903	-0,66487896	3,339949097	-0,199068591	0,84220909	NA
Tbc1d23	224,0732608	0,132152661	0,142886661	0,924877522	0,35502962	0,705239841
Fam96a	947,4924202	0,044394925	0,104407523	0,425208105	0,670684976	0,887361539
Cdk2ap2	1503,371617	-0,404569936	0,108218138	-3,738466979	0,000185146	0,007153798
Serbp1	3787,425556	0,074182538	0,066153099	1,121376613	0,262127587	0,622562722
Slc11a1	213,9771348	0,040180063	0,220145183	0,182516202	0,855177641	0,957445239
A530088E08Rik	0,259974817	0,586045239	3,35071904	0,174901337	0,86115716	NA
Ppp1ca	3693,679404	0,27550483	0,120654388	2,283421556	0,022405549	0,168103622
Cenpq	76,02402339	0,474401177	0,220217582	2,154238419	0,031221479	0,205110545
Smarcd1	292,0531935	0,166949769	0,153522935	1,08745816	0,27683438	0,637663478
Zfp275	231,2314424	0,001967869	0,178440639	0,011028144	0,991200993	0,99719344
Aldh1a3	10,94303104	0,728358496	0,633109852	1,150445682	0,249960354	0,60896459
Ubqln4	1207,696805	-0,394980264	0,076865411	-5,138595622	2,77E-07	4,74E-05
Aff4	1443,137161	-0,190142999	0,129316527	-1,470368891	0,141461872	0,467194607
Slc12a9	207,6245507	-0,000856012	0,173577417	-0,004931587	0,996065179	0,998991922
Srrt	1275,5822	-0,172803536	0,079514155	-2,173242441	0,029762075	0,198944591
Sytl3	2,338453234	1,930795874	1,121725922	1,721272404	0,085201405	NA
Sytl1	123,1882995	0,540265881	0,197566789	2,734598686	0,006245637	0,078920531
Gm4461	0,122496332	0,780932884	3,352475198	0,232942181	0,815806304	NA
Homez	146,448682	0,009287523	0,194461411	0,047760236	0,961907327	0,988526411
Krit1	367,0857208	-0,184940474	0,114085573	-1,621068019	0,105003052	0,402692427
Fgf9	1,493363155	3,997709775	1,589751696	2,514675584	0,011914196	NA
Epb41l3	58,61936676	0,301129446	0,265036627	1,136180495	0,255881032	0,616366745
Hist2h4	1,558771645	-0,518957582	1,169064932	-0,443908262	0,657108911	NA
Gdf6	20,72824754	0,236164812	0,441600465	0,534792943	0,592793044	0,851332066
Ulk2	1642,748044	-0,348444796	0,09510946	-3,663618694	0,000248677	0,008818979
Arhgap12	686,568604	-0,053031232	0,11552069	-0,459062629	0,646189192	0,875808808
Tceal5	0,875239126	0,187694129	1,648082874	0,113886341	0,909327893	NA
Saxo2	6,985848922	0,821522709	0,615203137	1,335368206	0,181755865	0,526292866
Mob3b	1042,167755	0,111419052	0,071123115	1,566565963	0,117216179	0,424678346
Hapln4	393,8419658	-0,511807781	0,171838645	-2,978420727	0,00289738	0,048005373
Tmem150b	4,901978913	0,96633537	0,703958334	1,372716713	0,169840431	NA
Mir34a	0,093953095	-0,517475177	3,352475198	-0,154356154	0,877328924	NA
Vps33b	188,7825568	0,014440441	0,11984681	0,120490826	0,904094347	0,973645668
Lsg1	534,5867371	-0,163826195	0,100785366	-1,625495858	0,104056946	0,401498609
Tmem198b	122,4933783	-0,07803662	0,166835624	-0,467745547	0,639966565	0,871049783
Flrt1	5,989629271	0,431560988	0,629296755	0,685782955	0,492849987	0,798745371
Sall4	0,732500484	-1,523392865	1,736426116	-0,877315108	0,380315509	NA
AA388235	5,850643327	0,258089435	0,615957177	0,419005484	0,675212124	0,889164486
Dip2a	77,22562982	-0,165903224	0,180531746	-0,918969811	0,358111363	0,707401735
Uhrf1bp1	518,4609446	-0,131041883	0,08672268	-1,51104513	0,130776953	0,44968032

Zzz3	548,0636507	-0,032477334	0,104427611	-0,311003325	0,755798094	0,922554343
Ambra1	654,2111588	-0,11483055	0,104523095	-1,098614135	0,271936409	0,633801597
Map9	18,52193827	-0,066650366	0,368363215	-0,180936541	0,856417386	0,9578959
Mup6	28,29599574	-1,623877958	0,954096314	-1,702006321	0,088754183	0,369300805
Tmem28	1,135464418	0,271535136	1,63020118	0,166565415	0,867712008	NA
Ibtk	1433,80055	-0,216664925	0,1319874	-1,641557639	0,100681713	0,393916833
Gstk1	3008,11336	0,383352837	0,106945786	3,584552972	0,000337656	0,011036977
Rab27b	25,2671326	0,529234209	0,390243856	1,356162821	0,175047368	0,517287416
0610040J01Rik	807,6894169	-0,399533938	0,123569114	-3,233283185	0,001223762	0,027542883
Keg1	6093,212366	-0,738527797	0,416786391	-1,77195756	0,076401602	0,34238646
Pwp2	334,5301933	-0,112769968	0,20774979	-0,542816281	0,587256302	0,848572815
Abcg8	1414,727062	0,242300743	0,241803431	1,002056679	0,316316219	0,67445479
Tmem98	261,6424514	0,765520185	0,27457152	2,788053851	0,005302573	0,070973656
Snrnp35	132,457262	-0,195918359	0,185353679	-1,056997414	0,290512768	0,652193612
Zfp60	180,0903325	-0,052395568	0,113771598	-0,460532929	0,645133741	0,874664897
Ttc30a1	33,8595683	-0,172218612	0,259366923	-0,663996048	0,50669284	0,806233823
Gpsm2	494,4636899	-0,025177544	0,124297219	-0,202559191	0,839479589	0,951377482
Rap2a	701,2436411	-0,72021956	0,150030779	-4,800478712	1,58E-06	0,000187269
Ttpal	357,9891265	-0,300617885	0,134130163	-2,241239992	0,025010534	0,180984139
Ms4a4c	11,21261088	0,788897325	0,530190671	1,48795022	0,136764022	0,459396536
Zmym2	590,3173071	-0,018894499	0,089656529	-0,210743143	0,833087707	0,950383954
Pcgf5	118,1789458	-0,030248704	0,155870786	-0,194062692	0,846126794	0,95378323
Rpl7a	10858,29713	-0,045527156	0,093019465	-0,489436871	0,624532438	0,864234982
Mga	512,1509462	-0,249094384	0,110138471	-2,261647385	0,023719198	0,174393148
Acsm3	2731,178093	0,034527898	0,103448494	0,333768974	0,738553897	0,915438384
Zeb2	217,0347533	-0,092773359	0,212098291	-0,437407385	0,66181593	0,882945528
Rhog	421,2547422	0,122302339	0,113836661	1,074366892	0,282658275	0,644306647
Rala	709,8867008	-0,109387974	0,095057477	-1,150756128	0,249832578	0,60896459
Smpd4	391,9329029	-0,074173425	0,112645737	-0,658466327	0,510238528	0,808610978
Rnf170	400,9474855	-0,059614765	0,130074508	-0,458312437	0,64672799	0,876230372
Lcn12	7,261026351	0,421587001	0,572494963	0,736402987	0,461485488	0,781516914
Ccdc93	341,4291767	-0,031694098	0,104689555	-0,302743647	0,762085232	0,92545349
Zfp955a	147,2643391	0,213794161	0,152597592	1,401032336	0,161204404	0,497950692
C330007P06Rik	597,3663651	-0,195544235	0,111324595	-1,756523207	0,078999112	0,348906222
Mir6984	0,093953095	-0,517475177	3,352475198	-0,154356154	0,877328924	NA
Dapk3	473,5441387	-0,344877104	0,13298177	-2,593416401	0,009502764	0,101415126
Ccdc85c	91,54941041	-0,418204442	0,233028582	-1,794648705	0,072709688	0,331420327
Tnfrsf11b	84,7391967	0,0347845	0,242178328	0,143631761	0,885791259	0,96772371
Npr1	259,2175667	-0,17163478	0,235489615	-0,728842245	0,466098167	0,781929771
S100a10	3371,942118	0,450509749	0,219759648	2,050011241	0,040363334	0,236409184
Efnb2	41,56766299	-0,172094479	0,407886883	-0,421917168	0,673085478	0,888626355
Efna3	0,984383399	-0,265927337	1,5107874	-0,176019033	0,86027899	NA
Dcbld1	82,67469	0,339763288	0,262411701	1,294771869	0,195398939	0,544340158
Inpp5k	321,9257687	0,260123817	0,128783341	2,019856095	0,043398317	0,245531991
Dad1	1952,543863	-0,125727981	0,09829437	-1,279096469	0,200863087	0,551932278
Casp12	35,4249372	0,447410927	0,335511619	1,333518428	0,182361732	0,527224185
Snord71	0,26680148	-0,62623849	2,8346967	-0,220919046	0,825155467	NA
Usp8	1420,05987	-0,038639421	0,100301504	-0,385232719	0,700065004	0,899228386
Bnip3	2417,307091	0,275197692	0,132313771	2,079887003	0,037535898	0,227066708
Bmx	2,142873443	-0,50768579	1,032023218	-0,491932528	0,622767043	NA
Mafk	229,3558503	0,420530036	0,199176491	2,111343736	0,034742777	0,217553251
Mafg	594,5068731	-0,097732105	0,133053637	-0,734531631	0,462624789	0,781516914
Pitpna	1417,39435	-0,140843962	0,070061778	-2,010282431	0,044401305	0,249002293
Eda2r	7,455730344	-0,725968615	0,554385735	-1,309500892	0,190364736	0,538735426
Fut2	3,280961928	0,648350258	0,873186326	0,742510777	0,457777924	NA
Bhlhe41	11,50801109	-0,063605593	0,553621606	-0,114890012	0,908532302	0,9747592
Adra1a	93,89403582	0,187946316	0,303657193	0,618942414	0,535954296	0,823122647
Wnt5b	227,1626339	0,037565376	0,154304691	0,243449345	0,807657321	0,942032857
Pacsin1	4,040260283	0,919995342	0,710134426	1,295522804	0,19513994	NA
P2ry1	103,7667865	-0,165716827	0,199968754	-0,828713602	0,407266487	0,746679655
Tpgs1	323,8217265	-0,192968314	0,154378925	-1,249965392	0,21131219	0,564073259
Slco4a1	4,564540691	0,743871838	0,650281116	1,143923482	0,252655372	NA
Pom121	353,3098743	-0,163191731	0,121591996	-1,342125605	0,179555273	0,522182824
Csrnp2	45,61354376	-0,189022893	0,261964818	-0,721558317	0,470566074	0,784805121
Pxt1	0,142634598	-0,517475177	3,352475198	-0,154356154	0,877328924	NA
Mir8114	0,113662148	0,780932884	3,352475198	0,232942181	0,815806304	NA

Supplementary Table S1: *Serpina1* KO vs. wildtype all DEGs

H2-L	0,839103187	0,300603595	1,744044641	0,172360035	0,86315449	NA
Clp1	330,4282666	-0,058774608	0,152143509	-0,38631032	0,699266859	0,89898054
Cdc123	1005,951243	-0,121852589	0,0917503	-1,328089269	0,184148618	0,530578205
Mfap3l	9,218971896	-0,410356472	0,496840293	-0,825932352	0,408842467	0,746898413
Tbc1d25	138,1329154	-0,369169896	0,149751501	-2,465216672	0,013693042	0,126862287
Irf2bp2	1245,526668	-0,162482434	0,251709223	-0,64551641	0,518592587	0,813725618
Cav1	56,89435761	0,496873572	0,261803009	1,897890993	0,057710443	0,290395259
Fam124a	188,774288	0,458517535	0,238402252	1,923293637	0,054443183	0,28262497
Esrrg	57,80817842	1,406619271	0,736466551	1,909956765	0,05613878	0,286457769
Cdc42	3500,142626	0,00054075	0,070765043	0,007641478	0,993903042	0,998278497
Clcn5	36,62307761	-0,665274689	0,308366127	-2,157418183	0,030973096	0,204100361
Ciita	26,82288687	0,957595287	0,364010785	2,630678339	0,008521465	0,095573374
Wdr55	381,578744	0,015627329	0,113086729	0,138188885	0,890091139	0,968877905
Abca14	7,804348069	-0,38853487	0,602886279	-0,644457975	0,519278501	0,813765593
Isca1	948,3000077	-0,022513807	0,093056511	-0,241936935	0,80882903	0,94241441
Ube2f	1035,162596	-0,299061987	0,116684152	-2,563004318	0,010377076	0,107377033
Taf11	286,2827515	0,153773646	0,107941278	1,424604635	0,154271519	0,489157561
Coq9	2564,863549	0,022010209	0,069653985	0,315993531	0,752007418	0,920946649
Adk	13643,53003	0,390877336	0,085656975	4,5632867	5,04E-06	0,00046431
Tsks	0,49424224	-0,295568948	2,50811436	-0,117845084	0,906190407	NA
Map3k12	30,6829635	0,537824734	0,333624904	1,612064109	0,10694799	0,405959022
Renbp	78,51487084	-0,035696629	0,298532172	-0,11957381	0,904820767	0,973666426
Ces3a	50002,74956	-0,656631391	0,232875002	-2,819673156	0,004807259	0,066976019
Id2	3789,27874	-0,095375522	0,149579578	-0,637623951	0,5237185	0,81568929
Tnnc2	0,350249842	-0,720602587	2,645726487	-0,272364733	0,785341587	NA
Gm6034	2,001808439	-0,443523134	1,017437315	-0,435921827	0,662893448	NA
Acox3	842,507163	-0,083007598	0,107395728	-0,772913408	0,439573629	0,766945637
Fam81a	66,28467054	0,125884724	0,276803801	0,454779608	0,649267802	0,876648536
Atad5	44,44149526	-0,165200816	0,23698658	-0,697089329	0,485746892	0,79430833
Rab36	22,72909357	1,690461185	0,447978502	3,773531943	0,000160953	0,006442441
Asb1	124,5495537	-0,255412678	0,144861305	-1,76315323	0,07787465	0,345649679
Ilvbl	549,9887831	0,039363294	0,08636746	0,455765334	0,648558735	0,876446263
Gipr	0,537122014	1,461108722	2,893238596	0,505008029	0,613553196	NA
Trabd2b	8,095086829	0,716863668	0,596494155	1,201794958	0,229442979	0,585386105
1700037H04Rik	83,70970254	0,350841723	0,229328457	1,529865623	0,126049994	0,441736801
Fbxo32	72,18654539	-0,043680256	0,407413606	-0,107213543	0,914619571	0,976392941
Mansc1	21,46664226	0,622081425	0,366577782	1,69699708	0,089697213	0,37148762
Atg3	2580,938585	0,151570811	0,076048459	1,993081954	0,046252474	0,255554034
Fam114a2	963,85743	-0,087935297	0,073232996	-1,20076062	0,229844072	0,585962127
Nudt13	265,4814132	-0,11391904	0,116372081	-0,978920703	0,327619162	0,685077831
Pop1	155,7765488	-0,075786213	0,182914219	-0,414326526	0,67863499	0,890709117
4930563D23Rik	0,087021394	-0,517475177	3,352475198	-0,154356154	0,877328924	NA
Slc2a1	340,4486889	-0,304106985	0,117285922	-2,59286861	0,009517912	0,10147127
Cttnbp2nl	99,72817802	-0,01629319	0,224915898	-0,072441254	0,942250755	0,983291729
Gpr25	3,648910301	-0,726758102	0,81799914	-0,888458271	0,374294293	NA
Med15	750,93641	-0,121530017	0,091799846	-1,323858616	0,185550005	0,532435718
Fyco1	1329,431743	-0,047497299	0,152216517	-0,312037744	0,755011839	0,922462845
Arhgap28	10,40512434	0,279933655	0,469456759	0,596292736	0,550979683	0,830351413
Dhx32	842,8043494	0,038269578	0,118771507	0,322211775	0,747292265	0,91954512
Pnisr	920,8964824	0,170869661	0,235461318	0,72568039	0,468034726	0,783100692
Pxdn	216,5681775	-0,092840429	0,20360328	-0,455986901	0,648399398	0,876446263
Ccnl2	1515,27688	0,005864044	0,123014728	0,047669451	0,961979681	0,988526411
Dedd2	355,2688182	0,065191712	0,138985626	0,469053626	0,639031305	0,871049783
Tbkbp1	199,6830966	0,164499468	0,114760408	1,43341655	0,151738832	0,48501133
Nrap	3,395417002	1,11117809	0,813840171	1,365351735	0,172142526	NA
Synm	8,59009458	-1,305634244	0,576616801	-2,264301422	0,023555582	0,173602303
Pcdhgc5	0,087021394	-0,517475177	3,352475198	-0,154356154	0,877328924	NA
Pcdhgc4	0,401022643	-0,105719668	2,585000526	-0,040897349	0,967377731	NA
Pcdhgc3	65,33934426	-0,36422831	0,209415841	-1,739258638	0,081989279	0,35403602
Pcdhgb6	4,93828581	0,341479157	0,711565771	0,479898234	0,631299757	NA
Ncf4	46,62849381	0,587367661	0,340457292	1,725231548	0,084485752	0,359824792
Ncoa3	620,5461651	-0,121427682	0,106196986	-1,143419286	0,252864549	0,612195971
Nxph1	0,093953095	-0,517475177	3,352475198	-0,154356154	0,877328924	NA
Ntsr2	0,122496332	0,780932884	3,352475198	0,232942181	0,815806304	NA
Ptch1	528,1772302	-0,574016891	0,154392742	-3,717900737	0,000200885	0,007483408
Bex1	4,130819457	1,023040311	0,725367562	1,41037505	0,158428969	NA

Supplementary Table S1: *Serpina1* KO vs. wildtype all DEGs

Rad51b	41,25023389	1,171826235	0,590318905	1,985073197	0,047136328	0,258794897
Rad50	251,0479847	0,019311318	0,136439939	0,141537132	0,887445628	0,968356501
S1pr4	5,507441668	-0,836020715	0,63995578	-1,306372631	0,191425869	0,540562071
Ptprj	1274,583274	0,09456984	0,136492727	0,692856259	0,488399762	0,796400974
Spink1	2,593114579	-1,364598632	1,02610108	-1,329887141	0,183555459	NA
Serpine2	120,3340554	-0,787959814	0,585464613	-1,345870948	0,178344139	0,520813194
Soat1	57,83437856	0,044247594	0,249617313	0,177261717	0,85930282	0,95905468
Rep15	3,131363907	0,00333578	0,871185305	0,003829013	0,996944897	NA
Mthfd2	55,93342075	-0,918129152	0,246332724	-3,727191161	0,000193626	0,007374874
Map7	893,6823412	-0,238362504	0,098007777	-2,432077439	0,015012497	0,133890644
Map1b	6,327048233	0,292141528	0,593822774	0,491967538	0,622742292	0,863366349
Map4	1046,047561	-0,128484558	0,10634135	-1,208227633	0,226959716	0,582272968
Map2	19,71021954	0,102389155	0,44199588	0,231651831	0,816808447	0,944998904
Map2k3	1448,101397	-0,021742033	0,099523637	-0,218460995	0,827069946	0,947537943
Msi1	2,091034258	-0,775490911	1,048520617	-0,739604829	0,459539811	NA
Slc43a1	176,2820248	0,639745634	0,257744177	2,482095396	0,01306123	0,123229067
Kcnk13	12,15350531	0,092252813	0,442850801	0,208315787	0,834982406	0,951084586
Rarres1	961,1444039	-0,657750749	0,584946061	-1,124463933	0,260816263	0,621964694
Stt3a	3224,523175	-0,316793768	0,109110203	-2,903429384	0,003691002	0,05655415
Pdk1	1973,688796	0,494415354	0,22849456	2,163794858	0,030480097	0,20212719
Tlk1	1256,399275	-0,051445498	0,105713838	-0,486648669	0,626507328	0,865266164
Megf9	250,7203005	-0,227343577	0,261466919	-0,869492698	0,3845777	0,729241793
Gtdc1	136,6674862	0,561845512	0,16227372	3,462332108	0,000535516	0,01539637
Hrh1	1,922115201	0,210400012	1,112735995	0,189083496	0,850027377	NA
Cav2	47,45879551	-0,047405969	0,327455944	-0,144770525	0,884892055	0,967327774
Cacna1a	11,75440379	0,321260149	0,422759027	0,759913162	0,447306494	0,772615053
Plcxd3	0,227324296	1,389394708	3,349408814	0,414817893	0,67827522	NA
Map3k9	5,142921344	0,920367759	0,653240068	1,40892729	0,158856669	NA
Slc9a7	11,30843217	-0,934975519	0,505492435	-1,849633059	0,064366455	0,308982094
Gk5	1,741501259	-0,303985832	1,067859709	-0,284668323	0,775898262	NA
Pbrm1	746,5509746	-0,005349893	0,136763598	-0,039117816	0,968796456	0,991555135
Tmem173	59,54750246	0,490840074	0,386525584	1,269877326	0,204128331	0,557198625
Il1b	61,8629579	0,01539261	0,369091052	0,041704099	0,966734586	0,990593599
Pde4dip	1778,635781	-0,39978863	0,104908651	-3,810826161	0,000138503	0,005732658
Pear1	133,3460561	0,067894441	0,211628284	0,320819317	0,74834732	0,919838327
Mcat	481,8251364	-0,121628774	0,092120903	-1,32031677	0,186729279	0,533163472
Fech	3890,004848	-0,057283969	0,081621752	-0,701822348	0,48278996	0,793999136
Prkcd	296,8610531	-0,106960343	0,123180131	-0,868324642	0,385216636	0,729677128
Zfp819	0,172953423	1,307385949	3,350719078	0,39018071	0,696402925	NA
Spata2l	564,7022256	0,878040141	0,176678187	4,969714471	6,71E-07	9,54E-05
Pyroxd2	870,7594482	0,009163036	0,134055824	0,068352392	0,945505119	0,984440501
Gdf10	175,416308	-0,16824085	0,216437866	-0,777317078	0,436971721	0,76575019
Lgi2	4,835465203	-0,533965104	0,738574231	-0,722967418	0,469699902	NA
Setd5	816,2845927	-0,211239809	0,09542163	-2,213751842	0,026845855	0,189333814
Ccdc13	0,340986444	1,884176617	3,333184127	0,565278288	0,571884494	NA
Ddo	609,3144892	-0,135188207	0,174315707	-0,775536576	0,438022661	0,766215379
Cstad	0,918218983	2,407442002	1,561330962	1,541916519	0,123093881	NA
Fam228a	4,934814815	-0,338731916	0,675129269	-0,501728974	0,61585818	NA
Klc4	1395,885467	-0,061664148	0,072408984	-0,851609069	0,394431105	0,73637054
Slamf8	17,7311312	0,624378226	0,446636256	1,397956879	0,162126016	0,498749809
Naa38	529,0837474	-0,039668254	0,128655722	-0,308328722	0,757832213	0,923813154
F13a1	5,798908706	0,355617094	0,786892484	0,4519259	0,651322365	0,877509695
Aldob	107531,073	0,364159491	0,124237861	2,931147459	0,003377124	0,053557266
Tmc5	0,710459736	-0,706775584	1,762321035	-0,401048146	0,688384677	NA
Pop7	121,4128622	0,194324875	0,146747036	1,324216698	0,185431087	0,532322998
Ankrd42	22,32409862	-0,001327019	0,346811088	-0,003826345	0,996947026	0,999481197
Ampd2	913,2648352	-0,576719695	0,088175199	-6,540611194	6,13E-11	3,90E-08
Stpg4	0,086476712	0,780932884	3,352475198	0,232942181	0,815806304	NA
Galnt15	9,275186652	0,147436633	0,550753976	0,267699624	0,788930539	0,935202374
Csgalnact2	200,4535199	-0,1352829	0,159728091	-0,846957473	0,397018826	0,738461552
Usp50	1,326373841	2,227066724	1,706080368	1,305370348	0,19176677	NA
Gm17830	1,102794883	2,709833348	1,779024063	1,523213432	0,127705317	NA
Zglp1	2,943585201	-0,736552114	0,956216523	-0,770277543	0,441135274	NA
Cyp3a59	1405,589576	0,287026311	0,209237988	1,371769596	0,170135175	0,510344993
Ciz1	612,2977554	0,049758777	0,09398265	0,529446416	0,596495807	0,853075785
Slc25a5	11300,69007	0,182136607	0,079113236	2,302226729	0,021322389	0,163264046

Supplementary Table S1: *Serpina1* KO vs. wildtype all DEGs

Gngt2	167,2351668	0,252060671	0,211184374	1,193557395	0,232651148	0,589477023
2210013O21Rik	39,11474535	0,474040282	0,408802253	1,159583341	0,246218487	0,605518092
Gck	3340,763308	-0,01314863	0,173091272	-0,075963565	0,939448085	0,982975773
Syce2	17,78634092	-0,071403549	0,417344704	-0,171090104	0,864152917	0,961272625
Pcdhb22	3,231630975	0,742026532	1,081072238	0,686380157	0,492473414	NA
Ctu2	243,5118853	0,138400385	0,115528053	1,197980764	0,230924506	0,587707923
Mapkapk3	117,9459057	0,421600018	0,203820414	2,068487693	0,038594187	0,230668679
Gorab	87,35535959	0,074956131	0,194514055	0,385350721	0,699977588	0,899228386
Pdcd4	2884,479352	0,213540727	0,114521625	1,864632355	0,062232954	0,302652735
Ubr2	1758,716637	-0,206181784	0,149007257	-1,383702966	0,166449425	0,505052737
Bcl3	765,028525	-0,178065555	0,165176287	-1,078033404	0,281018849	0,641764538
Rnf123	1604,944257	-0,064163539	0,111300211	-0,576490721	0,56428354	0,837355044
Ccl27a	112,0386494	-0,112827741	0,165555931	-0,681508301	0,495549916	0,80006558
Pbx1	264,1085636	0,457914794	0,197218452	2,321865874	0,020240159	0,159043099
Dpysl3	31,26927308	-0,327226369	0,351118886	-0,931953198	0,351360713	0,702791968
Lrrc7	0,227324296	1,389394708	3,349408814	0,414817893	0,67827522	NA
Hnf1b	609,230975	-0,035270019	0,119404706	-0,295382149	0,767701968	0,927586634
Flnb	1612,983706	-0,193558274	0,096985522	-1,995744007	0,045961794	0,254647007
Gtpbp10	309,6620126	0,096222688	0,122771946	0,78375143	0,433185979	0,763032854
Hectd1	3311,314438	-0,424740272	0,126822316	-3,349097267	0,000810753	0,020557413
Fev	1,775242437	-0,674602884	1,397984773	-0,482553814	0,62941258	NA
Dock3	0,77948171	0,436139128	1,9421274	0,224567723	0,822315577	NA
Wipf1	102,3716781	0,304548867	0,219780745	1,385694034	0,165840351	0,504089882
Cpz	16,27299824	0,446945378	0,447722855	0,998263487	0,318151608	0,675759967
Cd109	0,794499987	1,241249207	1,981846404	0,626309488	0,531111964	NA
Klrb1f	2,019284712	-0,174771778	1,088682098	-0,160535182	0,87245951	NA
Actr5	161,5280752	0,032283633	0,157426493	0,205071157	0,837516531	0,951141594
Mir6988	0,093953095	-0,517475177	3,352475198	-0,154356154	0,877328924	NA
Glrb	0,093953095	-0,517475177	3,352475198	-0,154356154	0,877328924	NA
Itk	12,90221358	0,950165664	0,448261765	2,119666985	0,034034139	0,215686483
1700008O03Rik	1,591438546	-1,560030905	1,116118694	-1,397728497	0,162194613	NA
Eci3	41,27798814	6,092808913	1,860458107	3,27489713	NA	NA
Nabp2	858,647178	-0,010172542	0,072633271	-0,140053479	0,888617737	0,96836734
Ttc39b	609,6919592	0,277003656	0,140050736	1,977880762	0,04794216	0,261821378
Pfdn5	1738,403641	0,014462918	0,11123523	0,130021018	0,896549798	0,970995969
Alkbh3	449,5481052	0,215410982	0,107878006	1,996801665	0,045846733	0,25411483
Snrpd2	539,7336684	0,042662357	0,10288789	0,414648967	0,678398896	0,890709117
Stoml1	92,63970456	0,397528394	0,168111277	2,364674165	0,01804594	0,149015548
1700007K13Rik	2,52670411	-0,741945414	0,910530081	-0,814849975	0,415158191	NA
Dph7	98,33037131	0,018453971	0,171940607	0,107327592	0,914529096	0,976392941
Tmem160	339,5659184	-0,049657592	0,1430496	-0,34713548	0,72848954	0,911204977
1700007K09Rik	0,093303375	-0,517475177	3,352475198	-0,154356154	0,877328924	NA
Epm2aip1	688,7791075	-0,052396668	0,142890405	-0,366691298	0,713849299	0,90610393
Rasl11b	97,27951891	0,501595762	0,17750337	2,825837959	0,004715711	0,06608877
Aspscr1	1934,311149	0,133795102	0,095021757	1,40804703	0,159117144	0,495312554
Gtpbp4	745,9676107	0,044778728	0,231757885	0,193213394	0,846791849	0,953824301
Phkg2	525,5757382	-0,169795868	0,117577755	-1,444115579	0,148706466	0,479743424
Gstm7	1944,877958	0,45144067	0,131858713	3,42366961	0,000617817	0,017059565
Srrm4	75,30019423	1,234015737	0,358603429	3,441171047	0,000579202	0,016336192
Fam57b	5,153025321	-0,079846319	0,679480406	-0,117510848	0,906455248	NA
Fam216a	63,9558372	-0,074578589	0,210087824	-0,354987678	0,722598823	0,909644412
Pink1	3909,352963	0,073542681	0,074730736	0,984102181	0,325065275	0,682335948
Ftsj1	465,3824518	-0,171712796	0,120721448	-1,422388479	0,154913506	0,489743509
Thrb	832,7169554	-0,100746091	0,118653189	-0,849080345	0,395836586	0,737426273
Zfp281	433,9326972	0,004279108	0,1348409	0,031734496	0,974683785	0,993185764
Fam168b	1318,648018	-0,063499087	0,09686669	-0,655530677	0,512126142	0,810227859
Nusap1	15,98270007	-0,328676714	0,539814706	-0,608869508	0,542610937	0,826611597
Hipk2	238,1410818	-0,125438202	0,29173369	-0,429975031	0,667213805	0,885709597
Tacr2	0,086476712	0,780932884	3,352475198	0,232942181	0,815806304	NA
Sorbs2os	0,093953095	-0,517475177	3,352475198	-0,154356154	0,877328924	NA
Cherp	748,849784	-0,096170399	0,088679551	-1,084470975	0,278156029	0,638507547
Mpzl2	801,6533467	0,195300085	0,142574033	1,36981525	0,17074458	0,510601034
Dvl3	589,7187819	-0,36701346	0,107231112	-3,422639693	0,000620162	0,017059565
Mir6924	0,487499354	0,314652485	2,462988776	0,127752302	0,898344998	NA
Fam65a	352,2846636	-0,043274628	0,14082935	-0,307284158	0,758627092	0,9243607
Smarca1	1,573085703	0,209805928	1,201969945	0,174551726	0,861431883	NA

Ttc21b	113,3537101	0,270648944	0,175512941	1,542045517	0,123062533	0,435597647
Derl2	2450,251763	-0,031012217	0,114772947	-0,270204937	0,787002595	0,934325343
Rhbdf1	119,6893003	0,188318821	0,190842202	0,986777658	0,32375165	0,680937786
Dhx35	151,0318388	-0,03122859	0,142385469	-0,219324278	0,826397451	0,947537943
Ralgapb	425,4354777	-0,036350519	0,117219569	-0,310106232	0,756480173	0,923140802
Zfp563	104,6438972	-0,045406326	0,188321052	-0,241111261	0,809468885	0,94241441
2410131K14Rik	163,0849217	0,375133577	0,12667104	2,961478628	0,003061657	0,049916217
Ms4a4a	1,405537764	1,506289783	1,311650363	1,148392762	0,25080646	NA
Rnf125	2969,140719	-0,139965474	0,252865014	-0,553518544	0,579908386	0,84530168
C030034I22Rik	20,47335622	0,154468265	0,327889123	0,471099083	0,63756998	0,870117708
Rhoh	13,52889003	0,339308475	0,547984551	0,619193506	0,53578889	0,823122647
Miga1	35,36824543	0,041148984	0,340077308	0,120998912	0,903691897	0,973489035
Aox1	2673,074582	-0,486401083	0,161355426	-3,014469954	0,002574287	0,044522178
Cfh	21548,24711	-0,04554491	0,133871318	-0,340214096	0,733695304	0,913633803
Cxcr4	14,94977063	0,576319032	0,503552826	1,144505607	0,252414014	0,612043137
Psmc4	2627,61886	0,044312936	0,100129059	0,442558198	0,658085327	0,881115947
Bmp1	3188,787838	0,214883487	0,082522588	2,603935376	0,009216013	0,100023317
Lipe	391,0122043	-0,147344483	0,167373039	-0,880335827	0,378677409	0,724565376
Zfp945	226,2712038	0,355383258	0,136992281	2,594184548	0,009481558	0,101415126
Kalrn	662,936218	-0,068935862	0,20156149	-0,342009091	0,732344051	0,912649428
Snord58b	1,10381337	-0,566922331	1,510973731	-0,3752033	0,707509276	NA
Il15	29,86452352	0,78524965	0,324478008	2,420039668	0,015518814	0,136494096
Scn5a	0,207615244	0,059593471	3,352475198	0,017775962	0,985817581	NA
Slit2	1,13400873	0,31512416	1,502612923	0,209717456	0,833888201	NA
Gid8	1351,308286	-0,109815208	0,092011902	-1,193489158	0,232677856	0,589477023
Nrg4	81,69935355	0,210991561	0,299358041	0,704813407	0,480926364	0,792841065
Trps1	10,07707889	-0,114330907	0,468334703	-0,244122218	0,807136166	0,941834983
Nedd4l	1607,350894	-0,300302212	0,110802956	-2,710236459	0,006723526	0,082540692
Tnk1	2,49931855	-0,436080596	0,978892447	-0,445483666	0,655970261	NA
Pcdhb9	0,995137295	-2,162804405	1,708080699	-1,266219099	0,205434651	NA
Pcdhb7	0,916126614	-0,364677306	1,48250846	-0,245986661	0,805692558	NA
Wasf1	33,01456212	-0,59473038	0,275848473	-2,156003882	0,031083362	0,204504659
Bco2	825,2043777	0,272421945	0,176230987	1,545823181	0,122147277	0,434047185
Fam98a	633,0578582	-0,270663016	0,123345648	-2,194345903	0,028210552	0,19328248
Rbm28	679,3408461	-0,260683086	0,103665712	-2,514650996	0,011915027	0,116510842
Slc12a6	289,4888252	-0,068101587	0,173229087	-0,393130209	0,694223313	0,896202343
Myoz3	0,517131955	-0,520448302	2,103272377	-0,247446935	0,804562355	NA
Fam198b	9,417634071	-0,132807963	0,6040559	-0,219860385	0,825979889	0,947537943
Sec22a	391,9126506	0,121376737	0,096838716	1,253390608	0,210063588	0,562470273
Med12l	97,29102232	-1,425906663	0,238182777	-5,986606917	2,14E-09	8,95E-07
Prmt2	19,55153764	-0,17890568	0,344862382	-0,518774123	0,603918265	0,855545543
Parvb	78,7810257	0,053122191	0,238186781	0,223027455	0,823514139	0,947466482
Pold3	228,1384214	0,055136156	0,12586962	0,438041807	0,661355978	0,882666274
Cd209d	0,624098492	-0,022421173	1,83997272	-0,012185601	0,990277538	NA
Cd209c	0,087021394	-0,517475177	3,352475198	-0,154356154	0,877328924	NA
Rrbp1	9343,899292	-0,34683058	0,127604487	-2,718012419	0,006567537	0,081222392
Rdh5	407,9436936	0,292791404	0,127281578	2,300343929	0,021428743	0,163367723
Sipa1l2	78,73605943	-0,196278238	0,215657279	-0,910139639	0,362748872	0,711918625
Ptgs1	227,862851	0,146151691	0,223749705	0,653192779	0,513632005	0,811192708
Gna13	663,5893137	0,112335334	0,088896623	1,263662567	0,206351166	0,558880735
Swap70	193,1489951	0,104454939	0,126302198	0,827023919	0,408223504	0,746892025
Zfp64	226,4407698	-0,060714927	0,129953014	-0,467206765	0,640351954	0,871049783
Gna12	1015,951992	-0,375049705	0,169842671	-2,20821836	0,027229055	0,190567596
Kmt2a	442,4253619	-0,174688406	0,151361993	-1,154110104	0,248455032	0,60747894
Otc	11923,73665	0,025103106	0,089154414	0,281568848	0,77827413	0,930864224
Tnfrsf4	8,27747069	-0,095568879	0,598774454	-0,159607475	0,87319029	0,963097638
Pklr	6717,683875	0,340094192	0,37046886	0,918010199	0,358613526	0,707901565
Gm1045	0,093303375	-0,517475177	3,352475198	-0,154356154	0,877328924	NA
Cyp2g1	43,19088159	2,712535388	0,536909798	5,052124953	4,37E-07	6,71E-05
Coro1b	1506,914827	0,091468504	0,082202243	1,112725162	0,265826486	0,626006345
Srsf2	2064,866014	0,064656982	0,112604686	0,574194412	0,565836251	0,837781736
Spry2	18,65035303	0,385859183	0,466481841	0,827168711	0,408141444	0,746892025
Acot1	427,9395759	-0,448195078	0,309482184	-1,448209626	0,147558433	0,47753781
Ints6	403,8688184	0,177947574	0,153235327	1,161269908	0,245532153	0,605051632
Notch1	403,0407036	-0,118972032	0,178847379	-0,665215407	0,505912728	0,80595177
Gm10190	0,886886329	2,321059346	1,963111814	1,182336803	0,237072088	NA

Supplementary Table S1: *Serpina1* KO vs. wildtype all DEGs

Scoc	155,3251622	0,003562182	0,151994863	0,023436197	0,981302332	0,994983586
Lepr	70,44035815	0,714738754	0,667337674	1,071030127	0,284155884	0,645517011
Apold1	28,5104113	0,022204904	0,326178554	0,068075916	0,945725202	0,984535137
Hnrnpm	1773,94423	-0,093101756	0,08489729	-1,0966399	0,272798838	0,63465219
4930579F01Rik	1,824514559	-0,45329237	1,271447022	-0,356516915	0,721453487	NA
Zfp804b	0,229655992	-1,252384348	3,339190293	-0,375056298	0,707618598	NA
Fam171a2	16,82710197	-0,164838318	0,577598275	-0,285385752	0,775348622	0,929570059
Glt8d1	209,8074793	-0,196904167	0,128457886	-1,532830505	0,125317622	0,44077119
Rad21	1105,034279	-0,046655235	0,113712867	-0,410289846	0,68159334	0,89166825
Cyp27b1	0,989628692	0,961218467	1,451679188	0,662142486	0,507879909	NA
Cdkn1a	242,4090836	0,150876198	0,521117133	0,289524539	0,772180004	0,928273961
Gata3	3,013986098	2,440271118	1,134434202	2,151090925	0,031469024	NA
Gata1	0,086476712	0,780932884	3,352475198	0,232942181	0,815806304	NA
Ppic	68,24711438	-0,1178103	0,336722652	-0,349873401	0,72643371	0,910393262
Ppia	10341,1926	-0,06391613	0,114769677	-0,556907818	0,577590413	0,845021474
Cyp2j5	18625,90142	-0,04564229	0,077824742	-0,586475313	0,557556146	0,834150416
Yipf4	632,7927897	0,033003787	0,121778515	0,271014858	0,786379605	0,934050588
Upk1a	1,462068195	0,754572527	1,355379538	0,556724154	0,577715911	NA
S100a16	493,8468353	0,018725452	0,09340793	0,200469622	0,841113314	0,951786586
Knstrn	15,6358028	-0,790525511	0,435708219	-1,814346106	0,069624457	0,322876701
Dopey2	1344,089556	-0,35902568	0,141839346	-2,531213584	0,011366861	0,113885033
Aspdh	2980,370486	-0,000159101	0,098757984	-0,001611023	0,99871459	0,999803624
Tmem100	7,785603217	0,19323803	0,6331074	0,305221563	0,76019741	0,924925298
Saraf	2905,736266	-0,091104037	0,078301874	-1,163497537	0,244627697	0,60351129
1810043G02Rik	128,6050194	-0,03042034	0,152119271	-0,199976899	0,841498648	0,951937335
Cd40	13,81940515	0,470708543	0,503529493	0,934818216	0,34988199	0,701811302
Mir484	0,51338535	0,408089182	2,14432395	0,190311348	0,849065162	NA
D630029K05Rik	0,206965523	0,059593471	3,352475198	0,017775962	0,985817581	NA
Pex11b	591,4061463	-0,052345316	0,109704499	-0,477148313	0,633256514	0,867685388
D730001G18Rik	0,113662148	0,780932884	3,352475198	0,232942181	0,815806304	NA
1700020I14Rik	249,7992633	0,022765433	0,169950851	0,133953041	0,893439708	0,969827497
2810002D19Rik	54,17145741	0,265754921	0,236315489	1,124576819	0,260768401	0,621964694
Chd3os	11,55700252	-0,349548304	0,426823943	-0,818951959	0,412813824	0,74954611
1810032O08Rik	35,46583162	0,354591263	0,34389827	1,031093477	0,302496986	0,662312842
1500011B03Rik	21,60801433	0,194432591	0,331208214	0,587040365	0,557176598	0,833822555
Zfp862-ps	53,36218882	-0,561639963	0,237729687	-2,362515053	0,018151404	0,14951702
Gm11961	0,180324769	-1,201310802	3,342952034	-0,359356278	0,719328578	NA
A430093F15Rik	0,086476712	0,780932884	3,352475198	0,232942181	0,815806304	NA
Cldn5	129,6053347	-0,349528987	0,184579151	-1,893653672	0,058270994	0,291660398
Hacd1	193,2808286	-0,337756335	0,126937483	-2,660808509	0,007795328	0,090230074
Ninj2	0,093953095	-0,517475177	3,352475198	-0,154356154	0,877328924	NA
Tmod4	6,725728545	0,494823768	0,564622336	0,876380079	0,380823444	0,725866642
Ddx25	2,531600685	-0,674114522	0,965030787	-0,698541986	0,48483831	NA
Ms4a6b	29,26495077	0,839121458	0,383553093	2,187758286	0,028687214	0,195374102
Cep57l1	120,3684475	0,188153962	0,170576449	1,10304771	0,270006447	0,630738457
Ift80	113,9952493	0,165921135	0,160537983	1,033531951	0,301355026	0,661872079
Art4	63,14511509	0,09044377	0,281283886	0,321539109	0,747801879	0,919636034
Sos1	850,9986876	-0,117329397	0,143143261	-0,819664132	0,412407602	0,749317374
Prpf31	384,0965292	-0,205448694	0,127869839	-1,606701747	0,10811981	0,408238928
Siah1a	99,18247836	0,467709534	0,188753426	2,477886336	0,013216324	0,123905355
Shmt1	5534,156777	0,261478459	0,111558032	2,343878369	0,019084394	0,153976622
Twist2	1,385014469	0,400338182	1,212586775	0,330152192	0,741284969	NA
Fgfr2	824,5642591	-0,361467667	0,105226194	-3,435149119	0,000592228	0,016563795
Slc16a1	1571,760256	0,011627867	0,140612471	0,082694422	0,934094521	0,981831091
Flt3	8,161648418	0,347201809	0,632180797	0,549212837	0,582859396	0,846891345
Lsm3	124,0545716	0,094967354	0,152612161	0,622279071	0,533758381	0,82137064
Mrpl18	1029,006562	-0,09734452	0,113287481	-0,859269879	0,390191638	0,733990715
Cnih4	289,3388847	-0,0853123	0,10677525	-0,798989464	0,424296521	0,756961229
Qtrtd1	110,0723339	0,061155281	0,168990531	0,361885843	0,717437339	0,907650451
Casp1	61,20937159	0,24484714	0,292386477	0,837409249	0,402362569	0,742560142
Camk4	1,86571609	-0,738870386	1,311210873	-0,563502334	0,573092872	NA
Rictor	352,3628634	0,141830851	0,152982071	0,927107667	0,353870636	0,704551502
Armcx5	52,78025842	0,246032986	0,230255432	1,068521964	0,285285128	0,646765623
Rfx7	428,7070611	-0,058421928	0,147678869	-0,395601138	0,692399298	0,895338108
Tmem231	8,320693282	0,490981223	0,58007983	0,846402854	0,397328047	0,738461552
Prosc	1649,652534	-0,052773816	0,096245064	-0,548327507	0,583467043	0,846950064

Supplementary Table S1: *Serpina1* KO vs. wildtype all DEGs

Frzb	47,54286512	0,164653146	0,243202025	0,677022102	0,498391931	0,801990802
S100a8	10,96554158	0,194905861	0,494648099	0,394029334	0,693559382	0,895984052
Lrpap1	1244,593011	-0,093117184	0,073335342	-1,269745002	0,204175477	0,557198625
Fezf1	0,093953095	-0,517475177	3,352475198	-0,154356154	0,877328924	NA
Wasl	1436,174575	0,080625094	0,107522476	0,749844094	0,453348609	0,77576312
Tm7sf2	3383,473466	0,297596202	0,089836363	3,312647483	0,000924174	0,022181832
Otud3	119,4108706	-0,327355608	0,154850948	-2,114004548	0,034514879	0,216973911
Larp1	3975,004498	-0,144035136	0,099484121	-1,44782036	0,147667297	0,477774467
Upf2	575,2761981	-0,185772931	0,097306008	-1,90916198	0,056241199	0,286761477
Rad17	316,6466428	-0,049225012	0,132159973	-0,372465362	0,709546397	0,90343073
Mroh6	37,26815209	-0,429200883	0,362709858	-1,183317394	0,236683382	0,59395378
Gm15816	0,206965523	0,059593471	3,352475198	0,017775962	0,985817581	NA
Fer	201,235937	0,166059123	0,122858933	1,351624334	0,176495522	0,519614321
Mtus1	4690,903897	0,091203043	0,102332963	0,891238179	0,372801407	0,719916511
Aqp1	1322,692875	-0,214327038	0,278832329	-0,768659213	0,442095644	0,769003232
Cnga2	0,227324296	1,389394708	3,349408814	0,414817893	0,67827522	NA
Npr3	43,37317561	-0,493462841	0,308293608	-1,600626249	0,109459725	0,40956257
Cad	492,8336619	-1,020917929	0,196561036	-5,193897779	2,06E-07	3,82E-05
Tsr3	358,2937987	0,072173183	0,113014597	0,638618238	0,523071314	0,815322684
Tufm	2367,839392	-0,100431759	0,084580707	-1,187407415	0,235066957	0,592624129
Bri3	428,4485719	0,370732401	0,152666028	2,428388331	0,015166095	0,134542482
Fbxo6	971,6217495	0,326704476	0,114073526	2,863981567	0,004183524	0,060944211
Emc10	1471,716866	-0,007475037	0,083492628	-0,089529311	0,92866126	0,979431397
Usp2	111,5395461	-1,114497026	0,261077104	-4,268842457	1,96E-05	0,001361075
Mir345	0,187906191	-1,241948083	3,339949325	-0,371846385	0,710007226	NA
Npepl1	1449,141769	-0,125677764	0,079111512	-1,588615377	0,112147254	0,414399294
Inca1	458,6362197	0,49210668	0,140825539	3,494442012	0,000475054	0,014082027
Siglech	6,071988201	-0,708523042	0,665790476	-1,064183204	0,287245714	0,648651236
Mir1934	0,173498105	0,059593471	3,352475198	0,017775962	0,985817581	NA
Dab2ip	293,9767518	0,101788612	0,19260554	0,528482265	0,597164653	0,853484526
Zbtb44	762,209494	0,172165397	0,084962276	2,026374599	0,042726412	0,243203202
U2surp	766,0735848	-0,094007898	0,122542984	-0,767142227	0,442996958	0,769646047
Edil3	0,274277864	-1,699264605	2,746395376	-0,61872541	0,536097268	NA
Mrpl13	879,6306218	0,113665289	0,093438168	1,216476012	0,223803643	0,5790477
2310036O22Rik	1387,16362	-0,162920778	0,148122591	-1,099904992	0,271373519	0,633246849
Triap1	373,373987	-0,060103806	0,088142735	-0,681891775	0,495307387	0,80006558
Fam96b	385,7907553	-0,152363622	0,125727427	-1,211856675	0,225567249	0,580946341
Zfyve21	961,0790107	-0,011616655	0,077389852	-0,150105669	0,880681248	0,96546599
Ints1	590,4838417	-0,184654836	0,103111995	-1,790818183	0,073322476	0,333531197
Slc41a3	22,90402481	0,634566939	0,324302297	1,956714291	0,050381069	0,269938714
Snx15	293,6866453	-0,211524275	0,122658444	-1,724498279	0,084617929	0,360024318
Ndufaf4	708,8597422	-0,179130245	0,175261681	-1,022073076	0,306746315	0,666053514
Bola1	353,2916724	0,131573625	0,122529024	1,073815986	0,282905164	0,644404577
Dmrtc1a	0,113662148	0,780932884	3,352475198	0,232942181	0,815806304	NA
9530077C05Rik	4,330183465	0,526733652	0,710536588	0,741318126	0,458500574	NA
Card19	208,6645932	-0,146438239	0,139202618	-1,051979051	0,292809161	0,654074594
Phf5a	542,1999539	-0,182143357	0,123839913	-1,470796872	0,141346059	0,467158334
Mob1b	311,1024241	-0,251242554	0,192811293	-1,303048953	0,192558049	0,541552905
Tmem126b	290,1345929	0,023898364	0,118205328	0,202176707	0,839778582	0,951440666
Mrpl14	954,973205	0,010653324	0,093784426	0,113593739	0,909559851	0,9747592
Ppp1r14a	58,07692435	-0,494619943	0,340604158	-1,452184102	0,146450422	0,476296539
Gpihbp1	276,5949829	-0,07486164	0,303800282	-0,246417282	0,805359229	0,94096033
Vstm5	3,25898272	0,305402998	0,817639287	0,373518009	0,708762945	NA
Echdc2	4846,618757	-0,189483173	0,165232866	-1,146764433	0,251478993	0,610832604
Gmip	113,46467	0,196115224	0,185939601	1,054725423	0,291550921	0,653216736
Mid1ip1	2161,679445	0,284114139	0,166168414	1,709796298	0,087303548	0,366455614
Unkl	233,8509983	-0,17777937	0,157862631	-1,126165001	0,260095685	0,621709138
Zfp1	123,1437332	-0,083646013	0,172443323	-0,485063796	0,627631093	0,865852703
Atp6v0a2	754,170336	-0,451913666	0,08740269	-5,170477782	2,33E-07	4,11E-05
Cel	0,174042788	-1,166820872	3,345560292	-0,348766954	0,727264274	NA
C8g	8012,367264	0,155507239	0,115416848	1,347353017	0,177866564	0,52021398
Mup14	17756,79015	-1,412396304	1,085192786	-1,301516489	0,193081724	0,541609226
Cox6a1	7263,433955	-0,011526048	0,085271843	-0,135168278	0,892478827	0,969104575
Emd	395,1960557	-0,126596107	0,093837419	-1,349100482	0,177304693	0,520079413
Ltbp3	65,29726814	0,054198785	0,220422326	0,245886095	0,805770408	0,941208637
Gnl1	486,8034536	-0,279245317	0,085547712	-3,264205561	0,001097715	0,025522349

Supplementary Table S1: *Serpina1* KO vs. wildtype all DEGs

Twf1	1408,764661	0,053961234	0,089204629	0,604915175	0,54523536	0,827491596
Ptch2	20,53914137	-0,408271249	0,401967341	-1,015682638	0,309780539	0,668839796
Dnmt3b	141,6883976	-0,052327337	0,168009637	-0,31145438	0,755455219	0,922462845
Gna11	1313,837161	-0,129897911	0,080111907	-1,621455746	0,104919934	0,402692427
Ripk1	792,5964289	0,015149099	0,116811431	0,129688499	0,896812882	0,971044462
Ptgir	5,442357659	0,278220813	0,720837064	0,385969072	0,699519574	0,899132505
Trdmt1	63,93851432	0,881308244	0,304348931	2,895716578	0,003782939	0,057405346
Ptgfr	2,59416441	0,894454222	1,080492952	0,827820506	0,40777216	NA
Ring1	409,0722851	0,148384852	0,148708036	0,997826723	0,31836339	0,676016543
Chadl	8,549617605	0,703673136	0,497477646	1,414481921	0,157220452	0,493047236
Casp2	112,8926378	0,03702657	0,185890531	0,199184808	0,842118183	0,952074658
Rpl19	6151,273639	0,120795412	0,129457688	0,933087976	0,350774547	0,70210533
Rab34	119,7506866	-0,279060217	0,2061149	-1,353906081	0,175766343	0,518576695
Tgm7	0,72021982	1,142840174	1,778604375	0,642548838	0,52051689	NA
Optc	1,021010071	1,780243787	1,68648286	1,055595541	0,291153039	NA
Ccdc58	715,5662537	-0,012843403	0,120186367	-0,106862396	0,914898146	0,976451566
Dynlt3	1493,651775	0,101994035	0,146175784	0,697749191	0,485334059	0,79430833
Rpl14	3592,715642	0,021897369	0,122184064	0,179216243	0,85776791	0,958260148
Hat1	211,6826986	-0,114610454	0,17103931	-0,670082533	0,502805179	0,804259684
Eif2s1	1125,024222	-0,044583181	0,102287576	-0,435861153	0,662937472	0,883542125
Gtf3c6	512,2856555	-0,209167675	0,130932539	-1,597522483	0,11014929	0,410672697
Zfp606	144,4657752	-0,126029456	0,139532622	-0,903225743	0,366406098	0,714711689
Qpctl	295,6369461	-0,342027997	0,139257015	-2,456091682	0,014045732	0,128967987
Polr2g	281,6741087	0,081813703	0,134012317	0,61049391	0,541534676	0,826407188
Slc46a1	1077,940378	0,045852766	0,080934115	0,566544348	0,571023794	0,8413217
Ubxn4	3625,676878	-0,28758603	0,116059603	-2,477916701	0,013215199	0,123905355
Pllp	337,5857752	-0,318617028	0,171259539	-1,860433756	0,062824183	0,304310325
Dgat2	9605,094841	0,053529119	0,122456379	0,437128061	0,662018478	0,883021554
Pnrc2	1402,025919	0,266399037	0,128066117	2,080167994	0,037510127	0,227013529
Psmd8	2918,676858	-0,073174663	0,060266303	-1,214188691	0,224675678	0,579757618
Ddx24	892,4693338	0,041306566	0,097180836	0,425048467	0,670801343	0,887361539
Gpatch8	497,3239361	-0,076106804	0,154509917	-0,492569061	0,622317114	0,86313492
Cacna2d2	0,093953095	-0,517475177	3,352475198	-0,154356154	0,877328924	NA
Sult2a5	42,03463602	8,449424748	3,226843381	2,618479966	NA	NA
Herc6	343,0203103	0,222040337	0,172276832	1,288857793	0,19744753	0,547135762
Hddc3	40,62003027	0,625051408	0,235267117	2,656773351	0,007889246	0,091080054
Nhp2	462,1705641	-0,055391392	0,179826119	-0,308027512	0,758061398	0,923924043
Stxbp4	81,97480702	-0,276214899	0,210029847	-1,315122122	0,188468863	0,535621781
Polb	400,8330445	-0,193901113	0,104498358	-1,855542205	0,063518838	0,306618538
5730409E04Rik	158,4855094	-0,151078559	0,154504966	-0,977823325	0,328161713	0,685487182
Ttc39a	30,51158498	0,35299993	0,412729206	0,855282167	0,392394957	0,734887345
Lrrc1	12,57983437	0,362262385	0,404017969	0,896649189	0,369906141	0,717858445
Patl2	1,0841927	-0,863881695	1,57954039	-0,546919662	0,584433927	NA
Rnf115	758,882547	0,116033684	0,09144489	1,268891942	0,204479607	0,557326781
Brix1	618,807369	0,072088275	0,148130013	0,486655426	0,626502538	0,865266164
Rab32	1069,871287	-0,089058302	0,131948318	-0,674948371	0,499708567	0,802860694
Tamm41	64,20870743	0,289392498	0,211275149	1,369742251	0,170767374	0,510601034
Nop9	479,7124252	0,019483306	0,160582008	0,121329318	0,903430199	0,973489035
1700093K21Rik	0,259974817	0,586045239	3,35071904	0,174901337	0,86115716	NA
Abhd13	414,1668904	-0,121212802	0,146032115	-0,830042093	0,406514985	0,745871812
Ppp2r2d	542,3806349	0,348426331	0,123301144	2,825815894	0,004716036	0,06608877
Poldip2	2507,268594	-0,013240164	0,084549878	-0,156595896	0,875563337	0,964125574
Prpf6	1359,400682	-0,053011339	0,163179283	-0,324865621	0,745282778	0,918622973
Rbm10	706,5462809	-0,40947085	0,097862374	-4,184149993	2,86E-05	0,001788167
Pla2g4b	0,885131258	-0,362136903	1,674220359	-0,216301815	0,828752494	NA
Trim41	677,7222913	-0,184219634	0,104068494	-1,7701768	0,076697693	0,342478335
Tmprss4	12,41241182	1,093303245	0,714989244	1,529118452	0,126235082	0,441789741
Mios	269,8299766	0,365478787	0,124428694	2,937254868	0,003311318	0,052764024
Sectm1a	26,82310048	0,240090187	0,336722244	0,713021463	0,475832476	0,789452019
Gps1	1152,148037	-0,179643191	0,091237779	-1,968956208	0,04895812	0,264881063
Acnat2	828,6755943	1,626932117	0,624600577	2,604756028	0,00919397	0,100011543
Tceanc2	318,7684823	0,129944436	0,096136438	1,351666839	0,176481918	0,519614321
Nudcd1	397,7566902	-0,23685932	0,124944721	-1,895712901	0,057998018	0,290969934
4921536K21Rik	0,26680148	-0,62623849	2,8346967	-0,220919046	0,825155467	NA
Xrn1	293,7557374	-0,049338893	0,163423805	-0,301907625	0,762722483	0,925557106
Mir1948	2,067762633	-0,441681719	1,237312999	-0,356968463	0,721115415	NA

Supplementary Table S1: *Serpina1* KO vs. wildtype all DEGs

Snora74a	0,339756192	1,876228608	3,160061454	0,593731684	0,552691588	NA
Cnpy3	1019,240324	-0,315381778	0,092115976	-3,423746818	0,000617641	0,017059565
Stard13	756,332386	-0,377234702	0,17443452	-2,162614962	0,030570807	0,202340544
Tbca	1131,924187	0,077498906	0,132441753	0,585154642	0,558443741	0,83440808
Tarbp2	200,7809111	0,113042451	0,18515901	0,61051553	0,541520359	0,826407188
Slc6a6	3293,597478	-0,039916321	0,093582046	-0,426538241	0,669715689	0,887039558
Gng7	20,63311228	0,895835418	0,38827357	2,307227397	0,021042147	0,162139745
Gng8	1,446708789	-0,002590638	1,377248439	-0,001881025	0,99849916	NA
H2-T22	54,90631833	0,295674728	0,274220447	1,078237347	0,280927849	0,641764538
Mcm3ap	543,411231	-0,322898989	0,119137919	-2,710295698	0,006722325	0,082540692
Taf10	1158,568918	-0,07254804	0,112921456	-0,642464618	0,520571555	0,814455368
Gngt1	51,74869334	0,429206633	0,303030388	1,416381496	0,156663839	0,492230049
Gnb5	15,40919737	-0,005214636	0,364969149	-0,014287881	0,988600308	0,996575327
Zfp51	126,1458669	-0,169852148	0,166412567	-1,020669002	0,307411285	0,666599067
Ogg1	100,5948844	-0,325071857	0,166980435	-1,946766145	0,051562778	0,273331789
Cdh4	9,196173165	-0,138911845	0,595297524	-0,233348602	0,815490721	0,944248697
Mgat2	1085,372824	-0,376908309	0,150181697	-2,509682053	0,012083991	0,117491545
Strip1	471,2805648	-0,10240822	0,107784099	-0,950123638	0,342049433	0,695250579
Vaultrc5	0,493026577	-0,58971359	2,468504166	-0,238895116	0,811186913	NA
Zfp937	53,16160634	-0,029170033	0,244585486	-0,11926314	0,905066885	0,973751746
Ppp2r3a	456,5231499	-0,078548041	0,127159787	-0,617711329	0,536765643	0,82388288
Mbd1	605,9966043	-0,258293208	0,178330436	-1,448396658	0,147506149	0,47753781
Pcdhb4	1,300546128	-0,022195258	1,269318691	-0,017485962	0,986048932	NA
Pcdhb8	0,338296039	0,667319034	3,192071415	0,20905517	0,83440517	NA
Pcdhb10	1,348293891	-0,310607718	1,312786859	-0,236601788	0,812965733	NA
Pcdhb11	1,176466336	-0,518393987	1,451721519	-0,357089139	0,721025075	NA
Ltbp2	25,32816046	0,622163257	0,355069573	1,752229153	0,079734413	0,349577792
Pcdhb19	2,248804869	-0,385780702	0,894476118	-0,431292345	0,666255816	NA
Mt3	0,086476712	0,780932884	3,352475198	0,232942181	0,815806304	NA
Mt1	1918,593263	4,275998625	0,949921581	4,50142276	6,75E-06	0,000590348
Plekha6	1232,867136	-0,030714303	0,142237533	-0,215936694	0,829037094	0,948028134
Cdc73	255,1057979	0,007814525	0,170327726	0,045879347	0,963406416	0,9888507
Zkscan3	738,3067074	-0,256452387	0,116805273	-2,195554868	0,02812382	0,193011984
2210408I21Rik	34,1263113	0,168785044	0,302370606	0,558205859	0,57670382	0,844833813
Rfx8	0,581263634	-0,872927916	2,081769574	-0,419320143	0,674982177	NA
Pigp	252,9498209	0,275701709	0,14822325	1,860043604	0,062879357	0,304467267
Mir7087	1,157891614	1,930965846	1,481626606	1,303274278	0,192481139	NA
Ptrh2	474,2844771	0,014309018	0,101487204	0,140993322	0,887875218	0,968360648
Gm684	4,705484607	1,477765411	0,875834942	1,687264733	0,091552459	NA
Prr22	4,027418156	0,000549115	0,761991543	0,000720632	0,999425019	NA
Epg5	690,3937801	0,015159692	0,15346672	0,098781624	0,921311659	0,977341679
5830473C10Rik	1382,313297	0,109048503	0,224965376	0,484734607	0,627864614	0,865852703
Mir7089	0,086476712	0,780932884	3,352475198	0,232942181	0,815806304	NA
Fcgr3	214,6848246	0,19455789	0,246570582	0,789055566	0,430079531	0,761019227
Klf12	491,6120106	-0,049748852	0,264032694	-0,188419286	0,850547982	0,955320139
Avil	3,672303437	0,36214911	0,917759542	0,394601302	0,693137152	NA
Cmc4	38,93647481	0,340622618	0,286295823	1,189757554	0,234141697	0,591392471
Mmp25	1,454857288	1,733500301	1,769544808	0,979630633	0,327268478	NA
Phyh	51528,243	-0,158991289	0,112373046	-1,414852537	0,157111737	0,492826562
Cdh9	0,389842494	-1,1017021	2,532750933	-0,434982408	0,663575197	NA
Klf4	25,12278657	0,656723817	0,337276485	1,94713787	0,051518208	0,273331789
Gm5820	0,897927948	0,210253752	1,74055465	0,120796984	0,903851839	NA
Klhl15	33,29855086	-0,042048894	0,257957354	-0,163007154	0,870512802	0,961775826
Phldb2	853,9284733	0,093369976	0,154953923	0,60256607	0,546797398	0,828173482
Atp13a3	2457,640584	-0,230430001	0,158082325	-1,457658228	0,144934771	0,473192126
Mitf	121,2344742	-0,75526764	0,173177515	-4,36123384	1,29E-05	0,001040506
Ddx3y	884,7998451	-0,481486358	0,931800128	-0,516727078	0,605346691	0,856421285
Vangl2	16,11643988	0,296527608	0,415652661	0,713402405	0,475596785	0,789452019
Hist2h2be	108,5946736	0,106602567	0,237917622	0,448065032	0,654106264	0,878604103
Impa2	67,80699379	-0,290804606	0,23444486	-1,240396592	0,214828741	0,56833832
Srgap3	187,6867371	0,502081448	0,276940034	1,812960877	0,069837856	0,323505282
Rpl31	3008,127072	0,09246438	0,138436678	0,667918229	0,504185791	0,804997854
Ripply3	30,09974392	-0,056217887	0,405224448	-0,138732712	0,889661368	0,968877905
Rogdi	587,4612819	0,080839717	0,183240858	0,44116644	0,659092507	0,881654058
Zmynd10	5,867336952	-0,117680259	0,573346148	-0,205251678	0,837375496	0,951141594
Dok4	30,46624381	-0,062664017	0,287333209	-0,218088322	0,827360296	0,947569588

Supplementary Table S1: *Serpina1* KO vs. wildtype all DEGs

Prdm10	104,4133543	-0,316770599	0,206260664	-1,535778045	0,124592826	0,439132771
Rxra	7582,706803	-0,023730571	0,10692941	-0,221927446	0,824370364	0,947473126
Prss53	187,6044485	-0,584940821	0,165752635	-3,528998624	0,000417135	0,012865677
Klhl26	148,3723791	0,198953198	0,273601418	0,727164351	0,467125282	0,782586618
Dmtf1	419,028448	0,195214091	0,108073306	1,806311832	0,070869645	0,326034508
Mau2	629,8242485	-0,028230815	0,09078694	-0,310956785	0,755833474	0,922554343
Hspa9	15614,58849	-0,05394688	0,08827603	-0,611115835	0,541122898	0,826407188
Eln	62,42633507	0,223699757	0,283451603	0,789199124	0,429995634	0,761019227
Car11	18,12067762	-0,44735	0,349760197	-1,279019176	0,200890303	0,551932278
A330093E20Rik	2,061576433	1,018620387	1,522192476	0,669179754	0,50338082	NA
Pik3r1	1360,222978	-0,330658399	0,140974362	-2,345521508	0,019000482	0,153536232
Mospd4	0,904067452	2,386902423	1,559850691	1,530212114	0,125964234	NA
Gfm2	1380,901027	-0,05038929	0,10648479	-0,473206456	0,636065892	0,869424933
Slc33a1	1832,731015	-0,267012523	0,12746254	-2,094831334	0,036185989	0,222803109
Sema5b	25,1812529	0,864415577	0,421680339	2,049930948	0,04037117	0,236409184
Cd4	68,03792704	-1,450247246	0,264360962	-5,485860063	4,11E-08	1,10E-05
Rpp21	241,4768141	-0,066272164	0,131216006	-0,505061582	0,613515584	0,859428114
Golt1b	81,29936637	-0,214025468	0,278932125	-0,767303041	0,442901361	0,769579971
Cwc27	231,6263351	0,186715823	0,134320848	1,390073288	0,164506624	0,502924549
Timm21	491,3943109	-0,058758581	0,088480874	-0,664082285	0,506637647	0,806233823
Rpl37	3341,712302	0,041108376	0,138272443	0,297299843	0,766237601	0,926944289
Zbtb8os	626,0258166	-0,175441672	0,125153901	-1,40180746	0,16097275	0,497812792
Sh3bp5l	376,9505834	-0,067463958	0,105267651	-0,640880241	0,521600494	0,814920768
Ulk1	1416,727909	0,309782736	0,148064652	2,092212641	0,036419498	0,223663557
Hnrnpab	3206,183229	0,00948175	0,122839343	0,07718822	0,938473813	0,982879468
Ddx4	1,145726866	2,800841593	1,595310952	1,755671262	0,079144557	NA
Plekhm3	61,88427188	0,541196755	0,251936157	2,148150399	0,03170181	0,206641847
Comt	27344,45552	-0,363967612	0,155676402	-2,337975483	0,01938852	0,155304678
Myl1	0,677368603	2,819293777	2,691240386	1,047581551	0,294831441	NA
Ttll2	0,770552323	2,145069681	1,758468427	1,219851121	0,222521318	NA
Zbtb3	6,581772414	0,143082538	0,686029325	0,208566212	0,834786889	0,950942904
Hic1	78,99178784	-0,326689159	0,241440259	-1,353084857	0,176028523	0,518925098
Slc25a21	258,7741069	0,590289188	0,203043414	2,907206776	0,003646721	0,056318411
Wisp1	29,38909982	0,123709242	0,331740232	0,372909975	0,709215447	0,90343073
Cyp3a41a	457,7006572	9,131727093	3,114226098	2,932262079	NA	NA
Pigc	302,8940247	0,095595061	0,145812181	0,655604082	0,512078898	0,810227859
Ttc14	519,0614682	-0,105936319	0,110924164	-0,955033733	0,339560638	0,694134533
Gm10536	2,127429315	1,730398578	1,126313734	1,53633799	0,124455507	NA
Mboat4	0,407765529	-0,919879351	3,064390271	-0,300183485	0,764037202	NA
Prpf18	632,9895589	0,061638991	0,110626652	0,557180299	0,577404249	0,84496439
Slc25a30	345,1154062	-1,459148857	0,576917601	-2,529215358	0,011431786	0,114223875
1810009A15Rik	4,780722581	-0,508720696	0,651882508	-0,780387095	0,435163062	NA
Dzip1	17,2530361	0,409577861	0,443080692	0,924386615	0,355285061	0,705249056
Gprasp1	159,8218276	0,25069968	0,142160743	1,763494437	0,077817135	0,345512213
Med29	303,2153639	-0,123766692	0,131884914	-0,938444648	0,34801595	0,700169336
Rrp15	273,6090722	-0,087717127	0,145426487	-0,603171597	0,546394541	0,828173482
Arpc4	717,9871173	-0,005036824	0,098669725	-0,051047313	0,959287819	0,987937108
Med18	30,92167941	0,203914403	0,282989796	0,720571576	0,471173147	0,785523607
Paics	5112,119395	-0,138325465	0,148079094	-0,934132298	0,350235653	0,70188884
L3hypdh	531,2582238	0,294480928	0,139121163	2,116722734	0,034283386	0,216604249
Rpp14	298,408608	0,144733444	0,132308734	1,093906955	0,273995788	0,635693562
Gid4	790,6320542	-0,348115258	0,131596395	-2,645325192	0,008161244	0,092872243
Mboat2	1,11036732	-0,256318277	1,580867696	-0,162137716	0,871197406	NA
Cmtm6	2802,941624	-0,410323809	0,112113189	-3,659906666	0,000252307	0,008899984
Tmem141	368,9075709	-0,15203448	0,185816442	-0,818197136	0,41324463	0,749920926
Spin2c	10,26309797	0,502020471	0,524837686	0,956525197	0,338806961	0,694071514
Smg7	777,8864441	-0,191505446	0,106838601	-1,792474306	0,073057021	0,332776599
2010106E10Rik	0,790125963	-3,196710583	1,804674169	-1,77135055	0,076502426	NA
Dnajc19	1144,53463	0,213113263	0,08693197	2,451494682	0,014226429	0,129647667
Mogat1	8,429697961	1,239451378	0,600340701	2,064579956	0,038962761	0,231714038
Coa3	1285,108443	-0,062347929	0,106410811	-0,585917245	0,557931128	0,834302421
Sla	62,24295043	0,365886118	0,252145456	1,45109146	0,146754394	0,476487281
Pknox2	14,3635434	0,097353873	0,406844677	0,239290026	0,810880699	0,94266644
Prr19	0,087021394	-0,517475177	3,352475198	-0,154356154	0,877328924	NA
Wdr70	153,5292324	0,101170349	0,133438819	0,758177793	0,448344554	0,77297141
Mir7019	1,356695251	-0,545153489	1,356820269	-0,401787548	0,687840387	NA

Supplementary Table S1: *Serpina1* KO vs. wildtype all DEGs

Mir7226	0,207615244	0,059593471	3,352475198	0,017775962	0,985817581	NA
Cyp3a16	42,99257886	7,255248758	3,204373323	2,264170877	NA	NA
Cyp3a11	166804,8955	-0,463871928	0,215992197	-2,147632809	0,031742937	0,206737412
Cyp2f2	43836,8675	-0,469019451	0,135128564	-3,470912715	0,000518693	0,014977108
Gpr161	3,100035686	-0,01460129	0,782587364	-0,018657712	0,985114163	NA
Ice2	265,5092534	0,102335731	0,113806998	0,899204202	0,368543903	0,716892516
Flcn	575,4030621	0,226450882	0,181586168	1,247071208	0,212371389	0,565238523
Carmn	19,18771799	-0,014460395	0,460391976	-0,031408877	0,974943462	0,993185764
Dynll2	4101,316525	-0,090332451	0,086661237	-1,04236282	0,297243497	0,658144463
Slc12a3	0,809845125	0,26086151	1,933666854	0,134905095	0,892686911	NA
Rab3d	288,9600835	-0,177621575	0,184622176	-0,962081474	0,336008679	0,69269083
Abcf3	1265,075406	-0,389967103	0,101702496	-3,834390697	0,000125876	0,005295032
Casp9	714,0030462	-0,156792512	0,082680759	-1,89636032	0,057912415	0,290736416
Acox1	51543,66889	-0,073389521	0,139678096	-0,525418967	0,599291971	0,85430922
Rnf19a	687,2972726	-0,014185815	0,143027775	-0,099182236	0,920993579	0,977341679
Znhit2	330,4162378	-0,097318172	0,131062497	-0,74253257	0,457764726	0,778822589
Abca8b	1743,393969	-0,000227457	0,17548526	-0,001296159	0,998965815	0,999803624
Srp9	1242,977608	-0,146878898	0,09241813	-1,58928663	0,111995696	0,414165319
Rab10	1980,919347	0,02503864	0,109036679	0,229635024	0,818375389	0,945704951
Asns	219,1777863	0,47869433	0,911881585	0,524952294	0,599616355	0,85430922
Foxc2	2,04456856	0,642496435	0,983955999	0,65297273	0,513773859	NA
Rpl8	7805,679675	-0,029037185	0,11411463	-0,254456282	0,799143074	0,938900058
Rps3	7148,186327	-0,040049989	0,109008689	-0,367401808	0,713319323	0,905933635
Mapk3	928,7044055	0,065966944	0,123496361	0,534161039	0,593230122	0,851502416
Etv3	289,9618392	-0,137951746	0,113432911	-1,216152743	0,223926742	0,5790477
St8sia3os	2,707981835	-0,764939976	0,849943337	-0,899989379	0,368125903	NA
Opn1sw	1,719799401	0,434265994	1,244448835	0,348962514	0,727117452	NA
Cdk1	37,23583825	-0,943045468	0,419959527	-2,245562745	0,024732029	0,179404502
Cdc25a	81,7719928	-0,299551609	0,25244629	-1,186595407	0,235387248	0,592840917
Cd9	618,5305067	0,414169509	0,157909435	2,622829405	0,008720292	0,096909052
Bmi1	381,5293019	-0,073787231	0,202211859	-0,36490061	0,715185599	0,906371815
Cxcr5	8,266523636	0,284935361	0,616610938	0,462099102	0,644010254	0,873822499
Blm	45,37142752	0,231371077	0,240043829	0,963870131	0,33511104	0,691456742
Fam3c	552,3015472	-0,240839949	0,109997593	-2,189501999	0,028560373	0,194708634
Rlbp1	1,02386661	0,37541307	1,518784346	0,247179971	0,804768946	NA
Nrip3	0,60783665	1,785771579	2,028899459	0,880167606	0,37876852	NA
Mettl9	1040,895538	-0,141268909	0,082241197	-1,717738966	0,085844239	0,363485758
Creld2	2161,780487	-1,49858684	0,272945795	-5,490419219	4,01E-08	1,10E-05
Gatsl2	42,59249	-0,198593048	0,314043175	-0,632374983	0,527141877	0,818299511
Lactb	1320,218407	-0,111800389	0,102700735	-1,08860359	0,276328733	0,637487287
Polh	109,169509	0,127984633	0,197691162	0,647396841	0,51737514	0,813424627
Zfp202	22,01389852	0,794174784	0,359001346	2,212177732	0,026954387	0,189659578
Cd96	4,429286156	1,222656557	0,913966993	1,33774695	0,180978941	NA
Erap1	1808,994442	-0,006893443	0,126680063	-0,054416162	0,956603603	0,987707257
Eri1	247,9625374	0,133047343	0,16919529	0,786353704	0,431660298	0,7622164
Fibin	6,615497243	0,402246525	0,607939755	0,66165524	0,508192197	0,807274415
Tmem221	6,184323329	0,782660017	0,589620649	1,327395875	0,184377764	0,530666592
Pnma5	0,432383558	2,249728513	3,310742426	0,679523872	0,496805987	NA
Zbtb42	106,7850757	-0,371766982	0,149359495	-2,489074976	0,012807595	0,121731107
Fam169a	0,474521762	0,280585928	2,48356908	0,112976897	0,910048871	NA
Lonrf1	138,6765374	-0,298326509	0,267944407	-1,113389574	0,265541151	0,626006345
Oxtr	0,227324296	1,389394708	3,349408814	0,414817893	0,67827522	NA
Sh3glb2	512,9971764	0,005226282	0,120748303	0,043282449	0,965476382	0,989924013
Tmem134	1009,185324	-0,050375513	0,104759601	-0,480867743	0,6306105	0,86654659
Henmt1	0,206965523	0,059593471	3,352475198	0,017775962	0,985817581	NA
Napb	44,03807442	-0,093400524	0,271584479	-0,343909654	0,730914231	0,912039608
Ppp4r1	500,5297174	-0,140593631	0,116818907	-1,20351778	0,228776015	0,584502337
Timm50	702,7125486	-0,063416412	0,098314243	-0,645037896	0,518902628	0,813725618
Snord91a	0,692768633	-0,465456989	1,756079845	-0,26505457	0,790967424	NA
Rad51d	329,3479907	-0,09906126	0,158110623	-0,626531333	0,530966492	0,819884984
Myh3	0,23615848	1,389394708	3,349408814	0,414817893	0,67827522	NA
Mak16	376,6520044	0,070971994	0,195936118	0,362220066	0,717187586	0,907650451
Btbd11	20,78342466	0,288350395	0,436596987	0,660449806	0,50896522	0,807845752
Mir6400	0,503504643	-0,517792012	2,113011746	-0,245049282	0,806418273	NA
Apoc1	106125,106	0,294745418	0,109538272	2,690798501	0,007128123	0,085467158
Trim13	54,84404035	0,326552112	0,242141102	1,348602567	0,177464656	0,520144503

Supplementary Table S1: *Serpina1* KO vs. wildtype all DEGs

Rerg	68,96290934	0,113170814	0,201455103	0,561766924	0,57427483	0,842938099
Arl14ep	212,7128626	0,154163535	0,127453899	1,209563111	0,22644658	0,581402791
Pamr1	90,79103611	0,038568323	0,233679724	0,165047796	0,868906362	0,961775826
Nudcd3	530,8011426	-0,120070431	0,098044769	-1,224649034	0,220707485	0,574949018
Olfm2	25,72422271	-0,238023503	0,385775411	-0,617000194	0,537234598	0,824035032
Dusp18	0,958266543	1,445328567	1,698135599	0,851126711	0,394698969	NA
Apol9b	3655,912035	0,319293112	0,098493475	3,241769172	0,001187902	0,026917053
Rnasek	1557,138864	-0,18057275	0,114276626	-1,580137227	0,114075444	0,418172362
Wdr24	394,0318134	-0,104139095	0,108522558	-0,959607811	0,337252637	0,69270916
Galnt18	27,41480523	-0,038883711	0,362435904	-0,107284379	0,914563377	0,976392941
Maoa	314,928693	0,210880312	0,141651444	1,488726875	0,136559305	0,459349518
Zfp940	23,21121415	-0,502619505	0,308972403	-1,626745624	0,103791135	0,400919872
Hmces	154,6690069	-0,087429944	0,196562288	-0,444795108	0,65646783	0,879906285
Tmem87a	197,7039343	0,156756315	0,15392449	1,018397495	0,308489092	0,667452771
Suox	4614,894489	0,095912501	0,136110647	0,704665676	0,481018317	0,792841065
Mamstr	4,054930205	-0,138472806	0,762024495	-0,181717001	0,855804824	NA
4930465K10Rik	0,087021394	-0,517475177	3,352475198	-0,154356154	0,877328924	NA
Fkbp2	1769,423771	-0,024817662	0,097955339	-0,253356909	0,799992423	0,939161109
Arfgap1	597,0609765	-0,055905261	0,09554777	-0,585102733	0,558478641	0,83440808
Zfp420	54,21329665	0,14282017	0,243261994	0,587104333	0,557133638	0,833822555
Zfp954	149,584838	0,067078615	0,149886259	0,447530116	0,654492348	0,878857794
Shisa7	4,736838984	-0,461075873	0,803343192	-0,573946326	0,566004123	NA
Leng8	1810,278658	0,175040395	0,224008141	0,781401934	0,434566134	0,764361477
Zc3hc1	248,5063414	0,041010019	0,131687815	0,311418474	0,755482511	0,922462845
Stk38l	635,2737781	0,221269542	0,131164767	1,68695867	0,091611299	0,375827372
Dera	1387,094625	0,333872736	0,116032481	2,87740754	0,004009574	0,059165556
Mmp8	0,698447573	-0,518394743	2,027025081	-0,255741652	0,798150331	NA
Ccdc174	219,9040798	0,118647808	0,134150362	0,884438966	0,376459296	0,722181709
Gpatch3	96,12236961	-0,275016498	0,224421331	-1,225447227	0,220406762	0,574490867
Fam221a	4,98758872	-0,108142505	0,747240153	-0,144722557	0,884929929	NA
Lnpep	180,5886864	-0,439773978	0,298872185	-1,471444983	0,141170816	0,466809953
Adap1	23,98849196	0,25627647	0,363060714	0,705877721	0,480264184	0,79208634
Mtmr10	634,0763544	-0,044660071	0,135277986	-0,330135538	0,741297552	0,916611817
Fbxw8	1067,82439	-0,084981083	0,097405141	-0,87244967	0,382963114	0,727455793
Sc5d	19032,80951	0,623365682	0,114283276	5,454566075	4,91E-08	1,26E-05
Chfr	463,3206043	-0,080966556	0,106183565	-0,762514957	0,44575272	0,771326444
Pcgf3	381,3027319	-0,16299148	0,148793316	-1,095422052	0,273331778	0,634872238
AU040320	683,9137867	-0,24556513	0,090388693	-2,716768247	0,006592275	0,081452981
AU018091	0,12663974	0,780932884	3,352475198	0,232942181	0,815806304	NA
Prrg3	13,34259616	0,366943517	0,405687134	0,90449878	0,365730981	0,714206468
Rd3	61,66820277	-0,456626054	0,256241657	-1,782013355	0,074747054	0,338055942
1700011H14Rik	15,79045004	0,685930973	0,480878151	1,426413265	0,153749087	0,488816063
Nos1	1,43805386	0,132725377	1,244709755	0,106631588	0,915081258	NA
Pgp	337,8586717	-0,42421338	0,177461288	-2,390455886	0,016827471	0,143473504
Gemin6	113,2672229	-0,188033395	0,159345454	-1,180036148	0,237985838	0,595254008
Ilf2	413,6731822	-0,043185834	0,135127311	-0,319593676	0,749276369	0,919838327
Cdc37l1	1097,686306	-0,134976234	0,108687203	-1,241877885	0,214281621	0,567643514
Dsn1	8,819010652	-0,076175158	0,710997208	-0,107138476	0,914679123	0,976392941
Fam220a	174,8070179	-0,008357618	0,13322339	-0,062733861	0,949978434	0,985972877
Lsm14a	657,8484976	-0,011882858	0,084260074	-0,141025959	0,887849435	0,968360648
Arpc5	1689,697806	0,044449369	0,094871791	0,468520396	0,639412489	0,871049783
Rnf219	28,95595155	0,158038057	0,285331737	0,553874796	0,579664535	0,84530168
Vps28	1409,759118	0,081656209	0,080966576	1,008517504	0,313206089	0,672219008
Clec14a	244,8105082	-0,064819058	0,317285167	-0,204292746	0,838124738	0,951141594
Pin1rt1	0,22911131	0,059593471	3,352475198	0,017775962	0,985817581	NA
Sys1	627,7569435	-0,013684287	0,108401073	-0,126237561	0,89954388	0,972141416
Psmd6	1524,58489	0,025758926	0,103428242	0,249051178	0,803321194	0,940228147
Mir6992	0,235937973	-1,303449117	3,335546383	-0,390775294	0,695963339	NA
Adamts14	22,10403112	-0,015136298	0,30404377	-0,049783285	0,960295087	0,98838005
Pcbp2	3757,568445	0,114445838	0,06911367	1,655907412	0,09774057	0,38924447
Dhrs7b	450,3635831	0,119189081	0,114227691	1,043434222	0,296747231	0,657874966
Catsperg1	7,304512858	1,295071683	0,633987844	2,042738983	0,041078284	0,239083837
9530053A07Rik	0,655607114	0,016546817	2,025326545	0,00816995	0,993481395	NA
Trappc9	353,8146061	-0,148055575	0,109816175	-1,348212814	0,177589944	0,52020157
Cutc	230,3566206	0,181373771	0,122149712	1,484848123	0,137584061	0,460640235
Cpd	676,2795509	0,02874134	0,203104266	0,141510273	0,887466845	0,968356501

Tnfsfm13	0,845512912	-1,076544472	1,684758774	-0,638990275	0,522829258	NA
Hamp	13886,36319	-0,647899616	0,425656847	-1,522117221	0,127979712	0,445305601
Mesdc1	96,87592423	-0,266918744	0,220511912	-1,210450453	0,226106092	0,581158101
Kifc5b	8,917495995	-0,875133252	0,57186167	-1,530323325	0,125936718	0,441736801
Acox2	6470,892749	-0,041783631	0,087757364	-0,476126784	0,633984057	0,867989994
Kcnn1	119,0256017	0,056541384	0,243602448	0,232105155	0,816456341	0,944998904
Kremen1	256,5149345	0,086510714	0,191652521	0,451393559	0,651705925	0,877662367
Myo7b	2,195124231	0,917933952	1,812222587	0,506523845	0,612488955	NA
Gprc5b	12,79872825	0,850407761	0,496837946	1,711640117	0,086963011	0,365945385
Pkhd1l1	14,15288056	1,479345571	0,608980091	2,429218281	0,015131419	0,13432477
Acot3	227,2432482	1,281393101	0,761739071	1,682194271	0,092531175	0,37789723
Mta1	808,3024703	0,018670822	0,086676837	0,215407282	0,829449793	0,948256737
Akr1c20	2729,119806	0,209833999	0,165605722	1,267069742	0,205130354	0,558190046
Baz2a	996,9826219	-0,160090955	0,126277074	-1,267775299	0,204878206	0,557844548
Prelp	1345,352206	-0,090912489	0,163364292	-0,556501594	0,577868005	0,845288649
Klf16	86,73356723	-0,186249062	0,221930142	-0,839223824	0,401343721	0,742022432
Muc4	0,601141313	2,663972526	1,987986548	1,340035489	0,180233807	NA
Als2cl	1051,066408	-0,091235371	0,110877082	-0,822851475	0,41059246	0,748154641
Spata25	1,27239581	0,548969242	1,625733758	0,337674751	0,735608304	NA
Tex30	86,20715071	0,082310089	0,19051449	0,432041097	0,665711544	0,884945574
Kxd1	965,5723083	0,164782142	0,103000794	1,599814292	0,109639787	0,409892147
Spry3	0,486199913	0,320550957	2,464466492	0,130069107	0,896511751	NA
Gns	3295,111549	0,083593515	0,107859998	0,775018695	0,438328613	0,766215379
Mir6995	0,122496332	0,780932884	3,352475198	0,232942181	0,815806304	NA
Rnf185	883,3877072	-0,013485856	0,081419947	-0,165633317	0,868445527	0,961775826
Mid1	37,23941128	-0,468889897	0,2816859	-1,664584195	0,095995741	0,385586553
Nkrf	95,43395679	-0,061068218	0,20285906	-0,301037666	0,763385772	0,925598865
Sorcs2	2,353353573	0,743047181	1,00692407	0,737937649	0,460552339	NA
Prrt1	4,063071496	-0,670013536	0,761640295	-0,87969812	0,379022868	NA
Rnf165	2,592961819	-0,148450281	1,051011134	-0,141245203	0,887676237	NA
Tmem95	0,607338445	-0,100006593	1,842801633	-0,054268778	0,956721024	NA
Dzip3	53,27045844	-0,277511758	0,254756204	-1,089322863	0,276011534	0,637060499
Cd40lg	2,542930306	-0,312207355	0,889496505	-0,350993347	0,72559334	NA
Pros1	1315,326027	0,039779698	0,136047411	0,292395846	0,769983984	0,927934035
Arpin	160,9382384	0,093181264	0,135743931	0,686448844	0,492430113	0,798476599
Raver1	351,1696232	-0,036971341	0,154820812	-0,238800846	0,811260014	0,942886004
Tmem70	969,9925661	-0,011856288	0,091039838	-0,130231866	0,896382984	0,970995624
Spdl1	8,19475506	0,185361657	0,5723654	0,323851961	0,746050115	0,919003408
Steap1	5,100353816	-0,719692561	0,719269924	-1,000587592	0,317026231	NA
Zbtb40	197,425417	-0,795145769	0,12846733	-6,189478422	6,04E-10	2,99E-07
D330050I16Rik	17,93226912	0,082597889	0,501263264	0,164779459	0,869117573	0,961775826
Bcl10	259,3391408	0,455483865	0,124326727	3,663603778	0,000248691	0,008818979
Lalba	0,18660675	-1,235117516	3,340448777	-0,369745983	0,711571767	NA
Anxa1	71,13513542	0,057088607	0,239891595	0,237976685	0,811899177	0,942944131
C4b	34651,71334	0,207697992	0,10831161	1,917596755	0,055162162	0,284116184
Mcpt4	0,113662148	0,780932884	3,352475198	0,232942181	0,815806304	NA
Dpy30	473,7335775	0,033538994	0,101906504	0,329115338	0,742068511	0,916899135
C3ar1	67,01139949	0,026068411	0,252890666	0,103081745	0,917898093	0,976907366
Mmp11	23,89472968	0,387153134	0,382598613	1,011904176	0,311583872	0,670999483
Klk1b27	0,122496332	0,780932884	3,352475198	0,232942181	0,815806304	NA
Inhbc	1970,861054	-0,736490897	0,185352505	-3,973460713	7,08E-05	0,003468881
Mfap5	0,921316718	0,400540085	2,142965005	0,186909298	0,851731749	NA
Ebi3	22,16302567	0,223915269	0,360520009	0,621089711	0,534540598	0,821883066
Abce1	2385,061822	-0,160830858	0,124646655	-1,290294214	0,196948525	0,546493323
Gja6	1,467070559	0,38434764	1,524189789	0,252165211	0,800913367	NA
Atp8b1	1060,276821	-0,373846418	0,149127084	-2,506898198	0,012179578	0,118248936
Anxa11	619,5112934	-0,059092233	0,144012711	-0,410326509	0,681566448	0,89166825
Anxa3	87,13007894	0,120293061	0,247068368	0,486881675	0,626342186	0,865266164
Ankrd1	13,64199691	0,526324641	0,462682572	1,137550177	0,255308361	0,615770789
Aldh1a1	33903,94197	0,244090881	0,189617053	1,287283382	0,197995534	0,54780388
Osbpl7	46,77193461	0,073088887	0,25282561	0,289088145	0,772513925	0,928273961
Ankrd44	64,3039675	0,520629206	0,27088625	1,921947704	0,054612338	0,283208825
Ptprq	0,88287918	1,482913646	1,622088694	0,9142001	0,360611712	NA
Nsmce4a	732,3879147	0,109335486	0,098980769	1,104613421	0,269327133	0,629944543
Stx5a	1373,355257	-0,473701092	0,096807584	-4,893222964	9,92E-07	0,000131305
Thoc5	504,7278965	-0,185334915	0,139391755	-1,329597396	0,183650957	0,529829877

Wnk4	45,45366691	-1,345614511	0,406166554	-3,312962379	0,000923134	0,022181832
Lipc	4429,392391	0,124489582	0,17508737	0,711014062	0,477075522	0,789944745
Lfng	63,73807838	0,303348964	0,282085299	1,075380265	0,282204514	0,643821185
Rps2	12797,13437	0,058469893	0,076322481	0,766090049	0,443622724	0,770142004
Gm10814	2,903375949	0,365801512	0,880332487	0,41552654	0,677756491	NA
Ncoa7	34,69459854	0,24338347	0,330066482	0,737377116	0,460893048	0,78124498
Ubap1l	1,179223213	-0,06081644	1,400050255	-0,043438755	0,965351785	NA
Cym	3,162512324	-0,19087878	1,070095022	-0,178375542	0,858428056	NA
Zfp964	16,11562243	0,351918355	0,450813988	0,780628739	0,435020884	0,764732964
Zfp691	161,8639807	0,337618273	0,163010395	2,071145665	0,038345186	0,22978998
Dbndd2	133,9929819	-0,13383986	0,164185587	-0,815174233	0,414972585	0,751551608
Med14	360,5903502	-0,142084796	0,171574335	-0,828123831	0,407600374	0,746679655
Vps51	434,0951503	0,054635561	0,104564912	0,522503769	0,601319623	0,854422974
Zfp568	310,575387	0,052027003	0,154426397	0,336904858	0,736188623	0,914608838
Cldn7	13,65537515	0,853346696	0,522768702	1,632359957	0,102603679	0,398868447
Zfp764	47,41533726	0,067830244	0,281849945	0,24066084	0,809817992	0,94241441
Trim30d	127,804997	0,330374651	0,20307138	1,626889274	0,103760616	0,400917827
Mir3473c	0,433059567	1,155701935	2,51910075	0,458775591	0,646395325	NA
Armcx6	10,47894065	-0,377332413	0,6971891	-0,541219611	0,588356222	0,848843045
C1qa	1530,424615	0,180999468	0,172811481	1,047381035	0,294923875	0,655827892
Mir6998	0,213661134	0,059593471	3,352475198	0,017775962	0,985817581	NA
Orc5	401,295498	0,072764366	0,126019713	0,57740463	0,563666147	0,837355044
Psg16	0,147722973	0,780932884	3,352475198	0,232942181	0,815806304	NA
Rspo1	11,49917085	1,173939208	0,689510951	1,702567895	0,088648962	0,369089997
Shkbp1	224,0802511	-0,019367744	0,167913407	-0,115343643	0,908172747	0,9747592
Atp6v1b1	0,467626184	2,318820502	2,90848053	0,797261827	0,425298985	NA
Plekhd1os	0,113662148	0,780932884	3,352475198	0,232942181	0,815806304	NA
B230216N24Rik	1,274095855	0,005391342	1,261897854	0,004272407	0,996591122	NA
Gm14327	4,60147145	-0,199207395	0,892492914	-0,223203335	0,823377256	NA
AI506816	7,795873406	0,080176039	0,673415087	0,119058869	0,905228717	0,973769127
Psmb7	3017,566749	0,133124426	0,080381287	1,656161907	0,097689035	0,389224129
Lat	10,85492281	-0,077878227	0,507709244	-0,153391392	0,878089633	0,965046146
Bcl6	1065,360782	-0,014757162	0,944753113	-0,015620125	0,98753745	0,99633146
Itih2	32465,61211	-0,165241008	0,1508654	-1,09528764	0,273390641	0,634872238
Cd47	734,5914834	0,081052944	0,084886024	0,954844392	0,339656394	0,694215921
Nap1l4	915,1286109	0,044900956	0,079535123	0,564542483	0,572384997	0,841794408
Il13ra2	5,120486367	-0,048384452	0,903886354	-0,053529353	0,957310144	NA
Gpatch2l	270,566553	-0,075139502	0,120222203	-0,625005205	0,531967642	0,820285514
Kif15	7,098753182	-0,917854411	0,64536299	-1,422229698	0,15495958	0,489743509
1700105P06Rik	0,122496332	0,780932884	3,352475198	0,232942181	0,815806304	NA
Samd3	2,28163934	0,381673207	1,331115361	0,286731878	0,774317627	NA
Wnt7b	2,441419416	-0,058928981	0,931066161	-0,063291937	0,949534036	NA
Hook2	460,7035193	0,010501941	0,106623963	0,098495125	0,921539143	0,977341679
Nqo2	3642,795214	0,115196935	0,111803189	1,030354647	0,302843555	0,662675256
Frem3	0,776043696	0,473064799	1,97829663	0,239127334	0,811006847	NA
Tnrc6c	385,8044089	-0,318401845	0,117756017	-2,703911474	0,006852854	0,083591067
Snord22	7,207820202	-0,143609107	0,555983788	-0,258297293	0,796177476	0,937785232
Lhx3	0,087021394	-0,517475177	3,352475198	-0,154356154	0,877328924	NA
Cystm1	163,4187377	-0,187434014	0,149261385	-1,255743501	0,209208982	0,561671273
Gm14085	1,148857911	0,616875198	1,837084843	0,33579026	0,737029035	NA
Gm2518	4,343906253	0,370010941	0,728165895	0,508140993	0,611354469	NA
Zfp992	4,032770872	-0,463219963	0,687357641	-0,673914038	0,500365967	NA
Fastkd5	238,6895159	-0,295525309	0,145357987	-2,033086148	0,04204382	0,242277514
Astn1	2,07469548	-0,554116631	0,975106414	-0,568262729	0,569856583	NA
5730408K05Rik	9,144132992	0,033541295	0,569048178	0,058942804	0,952997665	0,986479135
Cep78	59,34288063	0,235049356	0,197257014	1,19158934	0,233422307	0,590467894
Trmt1	593,6637621	-0,076957643	0,128713608	-0,597898263	0,549907819	0,829638487
Abr	94,70196459	0,265085478	0,21317195	1,243528889	0,213673002	0,567064616
Slain1	4,868541889	1,295043941	0,665813287	1,94505572	0,051768272	NA
Uqcr10	2401,506501	0,03248301	0,128294992	0,25319001	0,800121386	0,939224059
Cdkn2aipnl	309,9434073	0,068599472	0,142855579	0,480201559	0,631084078	0,866751905
Tti1	380,8481595	-0,24338962	0,118954814	-2,046067853	0,040749695	0,238000295
Nvl	435,8849307	-0,131225496	0,113915286	-1,151956865	0,249338799	0,608508653
Adamtsl1	22,71837178	0,93889369	0,343429477	2,733876247	0,006259356	0,079012204
Mcm6	147,0583848	-0,713621353	0,289198629	-2,467582075	0,013602903	0,126377494
Mcm5	75,28199534	-1,123359061	0,392863084	-2,859416186	0,004244216	0,06114323

Supplementary Table S1: *Serpina1* KO vs. wildtype all DEGs

Traf7	384,393889	-0,050076458	0,1124042	-0,445503443	0,655955972	0,879592859
Rundc1	273,3891643	0,08246946	0,103915902	0,793617319	0,427418247	0,759658939
Apoc3	38491,63125	0,407660733	0,121810855	3,346669985	0,000817885	0,0206014
Mturn	51,58823295	-0,080935945	0,305846986	-0,264628881	0,79129537	0,936263422
Llph	152,6784429	0,007282244	0,150106614	0,048513814	0,961306755	0,988526411
Rmdn1	513,3853279	0,175318813	0,109663363	1,598699959	0,109887287	0,410358418
Mrpl35	598,0935756	0,006428354	0,110142628	0,058363904	0,953458766	0,986479135
Zdhhc12	357,6722134	-0,166941743	0,122448381	-1,363364226	0,17276775	0,514646178
Col12a1	55,95645391	-0,066601161	0,265160982	-0,251172553	0,801680704	0,939799232
Trim30b	3,927119094	0,371048568	0,750302958	0,494531661	0,620930752	NA
Jph4	0,093953095	-0,517475177	3,352475198	-0,154356154	0,877328924	NA
Ogfod2	471,4876382	-0,167931815	0,147589647	-1,137829234	0,255191795	0,615600706
A530016L24Rik	2,698609119	4,082633931	1,619561155	2,520827274	0,011707931	NA
A530099J19Rik	3,063673806	0,088197323	0,887056184	0,099426986	0,920799257	NA
Ccdc50	1136,131832	-0,093155111	0,123814491	-0,752376485	0,451824684	0,774656859
Cdca7l	7,479341273	0,466541485	0,684517251	0,681562787	0,495515452	0,80006558
Wipi1	338,0409109	-0,173455642	0,195255731	-0,888351095	0,374351923	0,720933206
Wdr60	128,1132679	-0,158432177	0,153528353	-1,031940838	0,302099833	0,662128551
Psma7	2123,153916	0,18113062	0,093952028	1,927905383	0,053866896	0,280212659
Amph	0,835888365	3,143761256	2,072811266	1,516665462	0,129351169	NA
Sphk2	1396,478393	0,067035556	0,146307425	0,45818287	0,646821066	0,876230372
Acsl6	3,759306572	0,213573418	0,824389484	0,259068586	0,795582322	NA
Odf3b	227,1763076	-0,080373804	0,27355407	-0,293813228	0,768900627	0,927598042
Sstr3	0,482702416	-0,058142556	2,481362332	-0,023431707	0,981305913	NA
Sstr2	48,44397265	-0,327973618	0,519522941	-0,631297662	0,527845915	0,81852028
Ctgf	107,8988678	0,947837659	0,314896726	3,009995278	0,002612518	0,044950444
Olfr1442	1,071417865	-2,18957397	1,465179213	-1,494406931	0,135069291	NA
Olfr1443	0,539884942	-1,00632337	2,386285928	-0,421711145	0,673235869	NA
Plppr3	5,100506736	0,008081549	0,635822736	0,012710381	0,989858856	NA
Ran	2296,337346	-0,003117704	0,122525432	-0,025445364	0,979699728	0,994664228
Bche	2909,568773	0,482998311	0,245981715	1,963553717	0,049581869	0,266959327
Olfr1449	0,244992664	1,389394708	3,349408814	0,414817893	0,67827522	NA
Cyp2b13	743,9450738	10,68875071	3,156426547	3,386345461	NA	NA
Cyp2a5	9177,056308	0,904309265	0,193312727	4,67796032	2,90E-06	0,000314924
Rnf41	175,8916663	-0,179331368	0,161154349	-1,112792601	0,265797514	0,626006345
E030024N20Rik	0,402406457	-1,138884394	3,039517437	-0,374692502	0,707889169	NA
Mir6911	0,295445946	1,389394708	3,349408814	0,414817893	0,67827522	NA
Irak1	1801,601542	-0,28778457	0,105410241	-2,730138609	0,00633077	0,079420279
Calr4	0,472746174	-0,108833433	2,186423911	-0,049776913	0,960300165	NA
Hgs	1281,549911	-0,121082821	0,063820886	-1,897228762	0,057797752	0,290515036
Mettl10	107,2251222	0,453751849	0,160076951	2,834585777	0,004588514	0,064914117
Cand1	1225,124024	-0,151955556	0,092368208	-1,645106673	0,099947815	0,392993389
Ugt2a3	6943,276633	-0,03169708	0,112768489	-0,281081008	0,778648268	0,930937998
Fam219a	103,5055171	0,074929197	0,173684413	0,431410025	0,666170262	0,885111436
Ddhd2	347,9642114	-0,147992276	0,201304447	-0,735166451	0,462238128	0,781516914
Tmem106b	3449,552644	0,334760243	0,127905243	2,617251913	0,00886409	0,097781395
Man2c1	629,1048283	0,118584977	0,089728083	1,321603813	0,186300113	0,532805747
Jmjd8	1476,095452	-0,096775573	0,102876712	-0,940694656	0,346861358	0,698582329
Cyp2c55	416,0735661	-0,568353394	0,4348891	-1,306892709	0,191249153	0,540323314
Lypd6b	0,52964563	2,464443583	2,833613935	0,869717484	0,384454815	NA
Noxo1	19,17077383	0,21256103	0,357296227	0,594915407	0,551900018	0,830638744
Tmub2	666,6905624	0,124602444	0,072958427	1,707855401	0,087663176	0,367156644
Cdadc1	1212,456991	-0,043970081	0,086486621	-0,50840327	0,61117056	0,858645358
Anks4b	1528,722655	0,017669599	0,111032547	0,159138917	0,873559427	0,963216615
Naalad2	21,15678791	-0,561326148	0,368311099	-1,524054394	0,127495124	0,444570243
Chaf1b	36,04303604	-0,031955854	0,38955517	-0,082031653	0,934621543	0,981892281
Eif3k	1127,333295	-0,022770782	0,094418857	-0,241167735	0,809425116	0,94241441
Ppm1j	7,634106552	0,248423333	0,627113081	0,396138017	0,692003214	0,895338108
Sgol1	4,908921279	-1,335511448	0,696228464	-1,918208629	0,055084563	NA
Kcnmb2	0,307069547	0,617382779	2,733615373	0,225848444	0,821319297	NA
Shmt2	6738,09051	0,111894192	0,102448945	1,092194665	0,274747547	0,636475473
Slc41a2	1375,64316	0,059705025	0,240803721	0,247940626	0,804180346	0,940361036
Cep104	149,0308165	-0,310110487	0,150351745	-2,062566591	0,039153825	0,232539974
Sarm1	13,86060282	0,296074419	0,53354926	0,554914871	0,57895289	0,84530168
Vsnl1	0,122496332	0,780932884	3,352475198	0,232942181	0,815806304	NA
Ino80d	314,5551012	-0,107453217	0,126443884	-0,849809524	0,395430994	0,737426273

Supplementary Table S1: *Serpina1* KO vs. wildtype all DEGs

Tmem80	196,1076812	0,20035485	0,229915926	0,871426586	0,383521275	0,728102233
Rilpl2	173,3419227	0,162075115	0,122764076	1,320216144	0,186762863	0,533163472
Lysmd3	389,8574285	-0,360718538	0,177416575	-2,033172705	0,042035078	0,242277514
Bcl9l	798,8661473	-0,30148236	0,160253761	-1,881281026	0,059933701	0,296321614
Spdya	1,792712861	0,51595342	1,324633196	0,389506636	0,696901403	NA
Dhtkd1	620,979441	0,321277369	0,228569815	1,405598409	0,159843409	0,49581126
Ogdhl	1,507607925	0,384472124	1,190345803	0,32299196	0,746701331	NA
C030016D13Rik	13,26121189	0,52674282	0,469013752	1,123086088	0,261400934	0,622343615
Zfp62	417,9922066	0,019191623	0,141984376	0,13516715	0,89247972	0,969104575
Bdh1	4636,359833	-0,167179381	0,136787469	-1,22218345	0,221638261	0,576257504
Ermap	1,231506924	1,052558835	1,459014567	0,721417632	0,470652601	NA
1700018L02Rik	98,22611118	-0,460429827	0,320353398	-1,437255949	0,150645281	0,483083894
Aamp	3038,247733	-0,039094045	0,0778123	-0,502414713	0,615375832	0,859955845
Foxred2	8,865596017	0,246427845	0,470004557	0,524309481	0,600063303	0,854422974
Ccnyl1	89,00030845	-0,219370346	0,176544426	-1,242578713	0,214023118	0,567220469
Gnaz	1,812478749	0,707029545	1,183564434	0,597373091	0,550258315	NA
Slc1a5	95,75263648	0,657054049	0,232178219	2,82995559	0,004655447	0,065445494
Adgre1	311,2505771	0,075798447	0,261412974	0,289956713	0,771849354	0,928273961
Gnao1	5,658363977	-0,591848184	0,622402904	-0,950908455	0,341650849	0,695250579
Ptpn21	561,2520489	-0,316356596	0,100967555	-3,133250042	0,001728821	0,034651586
Rpl18	3562,074607	-0,038042055	0,10582674	-0,359474879	0,719239867	0,908608521
Phka1	181,9839249	-0,238698055	0,163757179	-1,457634143	0,144941413	0,473192126
Pcnx2	3,007665531	1,483544496	1,040881208	1,425277432	0,154077021	NA
Robo2	40,3713218	0,131520748	0,266300563	0,493880849	0,62139033	0,862654966
Eml1	43,63154493	-0,344838655	0,289804819	-1,189899659	0,234085833	0,591363094
Mmp23	27,59578974	0,324095317	0,381662966	0,849166268	0,39578878	0,737426273
Prkar2b	9,690913151	0,044990357	0,514180691	0,087499118	0,930274786	0,980359737
Srgn	133,9205304	0,034725981	0,25299487	0,137259626	0,890825582	0,968877905
Prep	1052,989231	-0,185245691	0,123751088	-1,496921712	0,13441363	0,456327747
Ppp5c	1160,854075	-0,150391584	0,067692668	-2,221682037	0,026304801	0,186859133
Ppm1b	2245,030214	0,152328911	0,096094262	1,585202979	0,112920224	0,416117064
Ppef2	0,667643221	0,083503804	2,023865179	0,041259569	0,967088965	NA
Lias	721,9311398	-0,054405466	0,104487667	-0,520687923	0,602584189	0,854608409
Stam	334,0004187	-0,199068749	0,113712871	-1,750626358	0,080010292	0,350133418
Prkra	374,9660444	0,018284832	0,117459014	0,15566989	0,876293232	0,964369791
Tpo	0,401567325	-1,129138825	2,515782745	-0,448822072	0,653560015	NA
Mdh1	17451,79495	0,15909561	0,088553878	1,796596755	0,07239966	0,330570715
Grem2	169,9143425	0,681263469	0,34371154	1,98207913	0,047470385	0,259775512
Mir221	0,467590061	0,3247882	2,178530305	0,149085922	0,881485834	NA
Mfsd14a	716,376825	0,087601249	0,121466851	0,7211947	0,470789732	0,785080196
Mndal	206,2375495	0,0979237	0,240474821	0,407209784	0,683853917	0,892293506
Serpinb9b	0,38246734	0,666763449	3,086759005	0,216007614	0,828981812	NA
Necap2	181,2920296	0,150946872	0,148275898	1,018013538	0,308671523	0,667523388
Eif3f	1646,200198	0,059053777	0,084406338	0,699636759	0,48415418	0,79420977
Atp6v1d	857,8057321	-0,30401034	0,087902392	-3,458499061	0,000543194	0,015452802
Nop10	1278,258414	-0,026328136	0,109610856	-0,240196427	0,810177983	0,942417935
Timmdc1	771,7116944	-0,020997312	0,088812994	-0,236421615	0,813105525	0,943775635
Pdhb	2844,268679	-0,009384586	0,119502542	-0,078530432	0,937406124	0,982376928
Scn1b	68,90158615	0,012898217	0,24921849	0,051754657	0,958724186	0,987896001
Ccdc34	27,98996267	0,034622181	0,33036636	0,104799353	0,916535023	0,976559041
Tspan31	5808,329479	0,002306606	0,08226587	0,028038426	0,977631503	0,994348571
Higd2a	1516,304286	-0,016510505	0,105130169	-0,157048208	0,875206855	0,964021167
Cyb5a	27059,12424	0,181999563	0,070931232	2,565859331	0,010292056	0,106618776
Slc16a9	60,1273117	0,032439584	0,281605235	0,115195244	0,908290368	0,9747592
Plbd1	1014,874462	0,36046518	0,179251556	2,010945895	0,044331172	0,248809167
Mrpl33	430,9442292	0,134113077	0,140612866	0,953775289	0,340197401	0,694472293
Wdr45b	671,1646988	0,004474008	0,153777909	0,029093957	0,976789655	0,993888492
Tmem223	436,9962261	0,033484394	0,143461074	0,23340404	0,815447676	0,944248697
Lztr1	775,1570702	-0,084364673	0,078147189	-1,079561202	0,280337626	0,6416138
Rsph3a	165,9263933	-0,212140146	0,127828022	-1,659574667	0,097000048	0,387479132
2700062C07Rik	189,6947795	0,147309375	0,132373884	1,112828078	0,265782274	0,626006345
Ypel3	779,9758885	0,119420915	0,178880388	0,667602058	0,504387644	0,804997854
Gkn3	0,25327948	1,389394708	3,349408814	0,414817893	0,67827522	NA
Ttc33	320,4266208	0,159741537	0,124964254	1,278297847	0,201144427	0,552289966
Agpat2	7763,070221	0,006056814	0,116189849	0,052128596	0,958426227	0,987896001
Tmed9	1936,143731	-0,420130422	0,150845685	-2,785166981	0,005350016	0,070973656

Supplementary Table S1: *Serpina1* KO vs. wildtype all DEGs

Tvp23b	456,449095	-0,210331783	0,100929419	-2,083949203	0,037164788	0,226049159
Saysd1	293,3996712	-0,007535185	0,156746344	-0,048072475	0,961658483	0,988526411
Tmem167b	841,4870811	-0,135857062	0,097826485	-1,388755422	0,164907135	0,50334664
Mettl16	340,6204554	-0,156982035	0,120446903	-1,303329772	0,192462201	0,541552905
Dhrs1	3698,140751	-0,207381902	0,129280044	-1,604129268	0,108685562	0,409049888
Atic	749,8674043	-0,174400053	0,106060018	-1,644352478	0,100103415	0,392993389
Ufl1	936,1620708	-0,296887653	0,16163818	-1,836742118	0,066247974	0,315296963
Ahi1	22,27285938	-0,424087777	0,334095572	-1,269360661	0,20431246	0,557220166
Calcoco1	790,9537591	0,364864524	0,180045048	2,026517966	0,042711734	0,243203202
Dhx40	918,0146681	-0,058912856	0,108365674	-0,543648679	0,586683255	0,848304107
Ccar1	925,5410261	-0,074935228	0,102088998	-0,734018644	0,462937375	0,781516914
Eepd1	689,4295167	0,550942367	0,185976019	2,962437691	0,003052136	0,049821738
2700060E02Rik	2027,321808	0,006057602	0,068387669	0,088577397	0,929417772	0,979997334
Ankzf1	515,3834227	0,021115529	0,150881162	0,139948079	0,888701014	0,968379155
Cwc25	280,6185899	-0,051234595	0,100261419	-0,511010069	0,609344006	0,857753956
Fgfr1op2	1094,203444	0,040864723	0,079957298	0,511081847	0,609293747	0,857753956
Ero1lb	2420,075981	-0,894235093	0,214528691	-4,168370619	3,07E-05	0,001890049
Slc35a4	311,5350314	0,162084139	0,141580072	1,144823108	0,252282441	0,612043137
Tprgl	978,5361731	0,154593247	0,077144079	2,003954786	0,045074904	0,251505172
Rpa3	125,1399123	0,268698466	0,150355267	1,787090476	0,073922866	0,335256454
Mrps11	313,5490388	-0,067867264	0,115015186	-0,590072202	0,555142245	0,832592702
Bambi	118,4183528	-0,099199047	0,18877147	-0,525498087	0,599236983	0,85430922
Fam32a	1285,27741	-0,118612181	0,078345304	-1,513966681	0,13003429	0,448279636
Mmd	2916,594402	0,043792495	0,140489973	0,311712605	0,755258948	0,922462845
Gpalpp1	440,1354781	0,11093908	0,128447248	0,863693703	0,387756174	0,731859161
Pdcl	468,3009044	-0,313253019	0,165491196	-1,892868177	0,058375402	0,291803151
Sumf2	225,3239021	0,176360996	0,113492865	1,553939059	0,120198954	0,431277459
Mtfp1	696,2193607	-0,029716666	0,114825857	-0,258797685	0,795791344	0,937598879
Entpd4	0,086476712	0,780932884	3,352475198	0,232942181	0,815806304	NA
Poc5	103,1660285	0,338716966	0,202678322	1,671204711	0,09468125	0,38267105
Decr1	4288,143185	-0,159982511	0,13344918	-1,198827235	0,23059513	0,587205008
Frmd8	374,2216834	-0,088622566	0,158895615	-0,557740795	0,577021397	0,844928702
Ergic1	2406,281584	-0,148133341	0,131531339	-1,126220885	0,260072035	0,621709138
Dynlrb1	1778,172522	-0,133699731	0,073261731	-1,824960023	0,068007062	0,319375503
Tctn2	34,22032633	-0,319312654	0,276246908	-1,155895849	0,247723761	0,607129386
Slc6a8	142,5074266	-0,278889237	0,217480814	-1,282362485	0,199715518	0,549955578
Klra2	64,70984149	0,159093376	0,314741709	0,50547281	0,613226791	0,859263072
Zfp57	13,12563214	-0,220682653	0,650207898	-0,339403218	0,734305995	0,913675851
Zfp983	205,7622682	0,247747113	0,141199672	1,754587034	0,079329971	0,349223642
Abhd11	872,0556349	0,004279308	0,098904839	0,043266923	0,965488758	0,989924013
Mir8091	0,995288293	1,022323433	1,844704243	0,55419368	0,579446303	NA
Efcab3	1,316788435	0,525954476	1,50892848	0,348561567	0,727418485	NA
Kansl1	606,2783683	-0,111605102	0,131085684	-0,851390473	0,394552482	0,736494294
D3Ertd751e	268,2128174	0,050394134	0,122710366	0,410675441	0,681310537	0,89166825
B230219D22Rik	853,0889514	-0,104095449	0,143079445	-0,727536013	0,466897662	0,782586618
Col14a1	1055,261525	-0,136873538	0,13153152	-1,040613975	0,298054743	0,658727654
Gmcl1	264,2593986	-0,184438213	0,125089889	-1,47444541	0,140361706	0,465404335
Lig1	72,18020351	-0,033360477	0,303149008	-0,110046465	0,912372525	0,975956816
Melk	3,947518192	-2,123176572	0,936821145	-2,266362777	0,023429181	NA
Trim27	465,7878236	-0,044646038	0,120810156	-0,36955534	0,711713832	0,905405102
2310039H08Rik	884,2692565	0,263817415	0,13295169	1,984310357	0,047221252	0,25915473
Fgf17	0,086476712	0,780932884	3,352475198	0,232942181	0,815806304	NA
Fap	4,236676161	-0,564762661	0,695619224	-0,81188478	0,416857749	NA
Ntrk2	116,9291735	2,441661457	1,139901949	2,141992528	0,032194083	0,208308055
Zfand2b	617,3160714	-0,029837236	0,106022526	-0,281423556	0,778385553	0,930864224
Ogdh	5894,183518	-0,178178823	0,083393127	-2,136612815	0,032629496	0,210239869
Plekhg5	129,4692875	0,104466667	0,180289066	0,579439835	0,562292432	0,837220482
BC037704	11,13374132	0,121538084	0,444249867	0,273580463	0,78440705	0,933246486
Grip2	2,083132193	-0,466654084	1,051217133	-0,443917882	0,657101955	NA
Gm10768	2773,043698	-0,049638971	0,079498922	-0,624398039	0,532366212	0,82055233
Coro2a	24,30972191	0,690976284	0,335037892	2,062382497	0,039171334	0,232540661
Slitrk2	0,113662148	0,780932884	3,352475198	0,232942181	0,815806304	NA
Nxt2	658,0799353	0,095186181	0,151086771	0,63001003	0,528688022	0,818817211
Pick1	376,5094132	-0,128427688	0,097174533	-1,321618788	0,186295124	0,532805747
Mir6910	0,482702416	-0,058142556	2,481362332	-0,023431707	0,981305913	NA
Apaf1	54,09028678	-0,0269003	0,269352995	-0,099870062	0,920447484	0,977341679

Supplementary Table S1: *Serpina1* KO vs. wildtype all DEGs

Bod1	520,1426089	0,115604572	0,103179174	1,120425445	0,262532507	0,622562722
Rfx4	120,8414269	0,800581996	0,680549558	1,17637575	0,239444758	0,596781687
Kyat1	4574,583357	-0,201778773	0,136733238	-1,475711214	0,140021434	0,465079889
2810021J22Rik	74,14340308	0,056051697	0,197632958	0,283615128	0,776705342	0,930009104
Aim1	466,5060902	0,682989213	0,179468286	3,805626211	0,000141446	0,005800582
Zfp759	80,26268504	-0,0225412	0,189590308	-0,118894265	0,905359126	0,973831053
Gm21992	0,259430135	1,627812584	3,345388605	0,486584005	0,626553161	NA
Dnajc8	700,6248216	0,120716673	0,095297538	1,266734438	0,205250262	0,558251976
Tmem125	196,2047505	-0,417740923	0,158485742	-2,635826519	0,008393263	0,094532042
AI314180	1929,981446	0,028031328	0,083601805	0,335295728	0,737402016	0,915064854
Poglut1	285,6324829	-0,073885012	0,122903454	-0,601163023	0,54773141	0,828572118
Fam149b	625,529722	-0,160287	0,088808967	-1,804851527	0,071097919	0,326747362
Slc22a22	0,086476712	0,780932884	3,352475198	0,232942181	0,815806304	NA
Wdr45	900,6713252	0,026619818	0,10543138	0,252484773	0,800666382	0,939490312
Slc16a13	37,59418705	-0,508853867	0,425178135	-1,196801587	0,2313839	0,588317108
Snapc4	227,8986402	0,122506077	0,154952574	0,790603689	0,429175293	0,760857915
Dnajc16	296,4096536	-0,167760148	0,126376613	-1,32746197	0,184355912	0,530666592
Tarsl2	315,2732966	-0,01224525	0,10852806	-0,112830271	0,910165119	0,9747592
Tshz3	15,34763697	-0,476775213	0,411263435	-1,159293952	0,246336386	0,605518092
Mbtps2	165,3331157	0,046685209	0,178423316	0,261654197	0,793588056	0,936746676
Dmrta2	0,584748855	2,638328875	2,271879867	1,161297705	0,245520853	NA
Cyp4a12b	2326,597354	-0,428473539	0,94081012	-0,45543041	0,648799621	0,876446263
Mab21l3	7,069466853	0,411920369	0,576703971	0,714266574	0,475062358	0,788957597
Tex10	159,9608991	0,15098142	0,145782478	1,035662325	0,300359704	0,66077158
Pced1b	51,94449797	1,250941946	0,301471556	4,149452651	3,33E-05	0,001990138
Cpsf7	726,4116283	0,07210269	0,08879644	0,811999782	0,416791757	0,75217184
Cpa2	0,142634598	-0,517475177	3,352475198	-0,154356154	0,877328924	NA
Foxred1	959,1301097	-0,090390078	0,105551404	-0,85636074	0,391798274	0,734532481
Slc36a4	49,39595461	0,329827701	0,323525603	1,019479442	0,307975408	0,667059844
Ntm	17,55030159	-0,201220522	0,401604875	-0,501041033	0,616342245	0,860471958
Nup133	262,4322712	0,040757925	0,109339842	0,372763708	0,709324316	0,90343073
Psip1	637,315174	-0,300317583	0,147904088	-2,03048872	0,042306887	0,243048341
Nhs	0,788113689	-1,650283994	1,944110583	-0,848863232	0,3959574	NA
Mospd2	952,5288945	0,322852693	0,144649163	2,231970692	0,0256169	0,183556901
2310034G01Rik	2,279565702	0,622275017	0,963535737	0,645824532	0,518392998	NA
Arhgef16	165,5985666	0,247637104	0,210006807	1,179186082	0,238324087	0,595542938
Tchh	5,695587886	-0,070895902	0,914139815	-0,077554769	0,938182223	0,982847752
Smim6	16,32386532	-0,621425516	0,38698996	-1,605792344	0,108319543	0,408497596
Phax	507,9470193	0,141666475	0,111860286	1,266459081	0,20534877	0,558251976
Map3k15	20,47882503	-0,400607079	0,321793676	-1,244919055	0,213161506	0,566777282
Npy1r	0,732492018	1,117806464	1,954706799	0,571853776	0,567421042	NA
Nppc	0,093303375	-0,517475177	3,352475198	-0,154356154	0,877328924	NA
Col6a1	235,4981985	0,091516009	0,240219648	0,380968043	0,703226962	0,900719679
Prkcb	64,92013897	0,196661619	0,278401453	0,706395808	0,479942027	0,79204357
Pole2	12,73481149	-0,594654652	0,439664976	-1,352517678	0,176209767	0,519230412
Slc1a2	2287,324056	0,645185845	0,238153153	2,70912158	0,006746161	0,082742597
C1qtnf6	29,62140934	-0,326850241	0,344736637	-0,948115767	0,343070522	0,695663554
Nob1	191,2981632	0,47965558	0,197173952	2,432651854	0,014988705	0,133857045
Alg13	113,5593281	-0,125889164	0,172555063	-0,729559375	0,465659562	0,781663619
Mrpl49	866,3147148	-0,050044274	0,098498848	-0,508069635	0,611404509	0,858645358
Slc39a9	1415,036496	-0,222358162	0,097996188	-2,269049097	0,02326534	0,172318191
Mrfap1	2371,900783	-0,040659945	0,102697592	-0,395919162	0,692164664	0,895338108
E2f2	34,48138301	-1,074569957	0,310415358	-3,461716467	0,000536742	0,015398514
Gramd3	1132,572997	-0,00851226	0,117931318	-0,072179806	0,942458816	0,983364013
Tmem35a	1,376391806	-0,517628171	1,277547069	-0,405173464	0,685350009	NA
Narfl	485,527676	-0,149392161	0,085197192	-1,753486917	0,079518463	0,349223642
Wdr48	683,738243	-0,116428816	0,084051541	-1,385207402	0,165989058	0,504228066
Larp6	0,667259251	1,889333717	1,976907464	0,955701646	0,339222991	NA
Mapk1ip1	201,4304088	0,550418786	0,161764534	3,402592474	0,000667498	0,017837331
Gstcd	83,81769847	-0,242831194	0,208499347	-1,164661654	0,244155977	0,602840659
Gpr89	524,6203331	-0,176260194	0,097864539	-1,801062938	0,07169296	0,328465795
Atp5s	116,3061822	0,267815463	0,167075207	1,602963529	0,108942706	0,40910239
Mpnd	1835,425948	0,129398864	0,090975256	1,422352296	0,154924004	0,489743509
Bbx	256,5303678	-0,236707376	0,155835133	-1,518960276	0,128772495	0,446231076
Rpl22l1	452,219973	-0,02835772	0,155056406	-0,182886478	0,854887093	0,957431562
Ccdc39	1,781843543	-0,154071074	1,235199657	-0,124733741	0,900734344	NA

Supplementary Table S1: *Serpina1* KO vs. wildtype all DEGs

Mfap1a	107,6308913	0,037936626	0,151961593	0,249646146	0,802861009	0,940128653
Klhl10	1,143233362	-0,058835433	1,276640914	-0,046086125	0,963241605	NA
4921524J17Rik	166,6118133	-0,054368496	0,167302265	-0,32497167	0,745202513	0,918622973
Ifrd2	852,0511851	-0,174027663	0,123546936	-1,408595545	0,158954797	0,495312554
Syap1	1081,648849	0,030766853	0,134434835	0,228860715	0,818977175	0,945934875
Rnf225	10,11343478	1,354425343	0,587111895	2,306928805	0,021058789	0,162174513
3110002H16Rik	500,4913574	-0,359184588	0,125680912	-2,857908815	0,004264429	0,061302316
Spryd4	803,3887163	0,172235388	0,086861677	1,982869713	0,047381985	0,259397933
Odf2l	169,0071718	0,240635583	0,153560696	1,567038894	0,117105601	0,424392729
Cdk5rap1	204,9283475	0,29410979	0,17394036	1,69086571	0,090862454	0,374146038
Zcchc18	1,332326042	-1,728350094	1,304611914	-1,324800176	0,185237436	NA
Rnaseh2b	151,7625328	0,039213654	0,19132557	0,204957727	0,837605153	0,951141594
Gapvd1	722,9745845	-0,121943048	0,11244468	-1,084471475	0,278155808	0,638507547
Rnaseh2c	605,9099265	-0,320769406	0,16580859	-1,93457652	0,053042284	0,278196269
Nkain1	2,54348492	0,264635691	0,980497565	0,269899386	0,787237659	NA
Rffl	505,7211109	-0,03321032	0,14396113	-0,230689492	0,817556037	0,945248349
Fam103a1	384,6040963	0,105430748	0,154786937	0,681134659	0,495786288	0,80006558
Zkscan6	106,1728726	0,243427587	0,205370586	1,185308918	0,235895329	0,593243914
Dr1	415,7010242	0,162281989	0,178242782	0,910454761	0,362582729	0,711906082
Snrpd3	1507,061192	-0,111609144	0,12054645	-0,925860064	0,354518708	0,704662593
Ift27	189,4727531	0,110150327	0,138740499	0,793930598	0,427235835	0,759435698
Fbxo5	19,57555097	-0,087447761	0,331731264	-0,263610248	0,792080261	0,936507892
Carmil1	462,4067084	-0,60845996	0,28774174	-2,114604433	0,034463676	0,216957051
Svop	0,086476712	0,780932884	3,352475198	0,232942181	0,815806304	NA
Atp8b3	0,122496332	0,780932884	3,352475198	0,232942181	0,815806304	NA
Stx4a	813,2586946	0,068874583	0,134705799	0,51129635	0,60914356	0,857753956
Col18a1	25883,95731	0,054055008	0,101364111	0,533275606	0,593842812	0,851766384
Nos1ap	36,55034581	0,366856792	0,388086296	0,945296948	0,344507296	0,697587612
Uhrf1	23,36080439	-1,58303562	0,566484234	-2,79449193	0,005198134	0,07004174
Mis12	463,6893361	-0,288864878	0,168655888	-1,712747071	0,086759081	0,365547481
Mcm8	44,95812149	-0,139540642	0,252569577	-0,552483969	0,580616815	0,845562767
Dph6	131,8084493	0,49356493	0,162364889	3,039850138	0,002366959	0,042463022
Golph3	1627,597829	-0,068802333	0,106724844	-0,644670262	0,519140892	0,813725618
Cdip1	1407,533793	0,36175992	0,128779563	2,809140758	0,004967392	0,068533609
Ubr7	364,2056285	0,038540803	0,105737318	0,36449575	0,715487847	0,90658298
Slc25a23	11150,66084	-0,251592631	0,093351463	-2,695111817	0,007036501	0,084672355
Necap1	954,0038744	-0,05512248	0,088081972	-0,625808875	0,53144031	0,819884984
Naa16	194,2296022	-0,07974852	0,178663513	-0,446361539	0,65533611	0,879460797
Cgnl1	1947,410604	-0,331872394	0,10927748	-3,036969676	0,002389695	0,042653981
Pigk	759,6972165	0,022285156	0,088848291	0,250822558	0,8019513	0,939799232
Ftsj3	1103,555784	-0,191099503	0,117179041	-1,630833473	0,102925462	0,399190747
Acot13	1605,477035	0,31631198	0,100684526	3,141614628	0,00168019	0,034137482
Ppp1r2	610,5307281	-0,17131299	0,135940006	-1,260210258	0,207593523	0,559991488
Fancl	113,6113106	0,011135037	0,174612124	0,063770126	0,949153265	0,985650844
Itpa	653,0875055	-0,223423504	0,122836183	-1,818873712	0,068930699	0,321204084
Kcng4	1,06116929	1,764440653	1,545190281	1,14189215	0,253498852	NA
Lrrc57	257,0747027	0,092487974	0,102818204	0,899529173	0,368370864	0,716848629
Anp32a	5127,486726	0,288836088	0,116493725	2,479413278	0,013159873	0,123636218
Tmigd1	0,720763378	1,051190368	2,022883417	0,519649506	0,603307886	NA
Rdm1	138,8542948	0,053654285	0,15886428	0,33773662	0,735561676	0,914229638
Dusp26	0,187906191	-1,241948083	3,339949325	-0,371846385	0,710007226	NA
Aste1	55,84955451	-0,254658122	0,247753443	-1,027869155	0,304011381	0,663347176
Rplp2	3282,892392	0,020338519	0,122654759	0,165819243	0,868299202	0,961775826
Kbtbd4	352,0225891	-0,164369894	0,11026535	-1,490675847	0,13604662	0,458903704
Selenoi	1662,116522	-0,182375417	0,087783218	-2,077565867	0,037749363	0,228038895
Fam111a	78,84172979	-0,049993698	0,236114905	-0,211734613	0,832314083	0,949906691
Fam210b	3206,438309	-0,094571746	0,086736413	-1,090334985	0,27556561	0,637014134
Khdc3	0,180324769	-1,201310802	3,342952034	-0,359356278	0,719328578	NA
Tctex1d1	0,234199685	1,337107854	3,350237755	0,399108348	0,689813372	NA
Acbd4	1307,794422	-0,187236096	0,138604086	-1,350869958	0,176737093	0,519981998
Ndufa6	4403,233629	0,157223067	0,116214824	1,352865847	0,176098492	0,519016918
Zfp830	207,7832961	-0,054036357	0,130313886	-0,414663078	0,678388565	0,890709117
Rbm8a	1055,153568	-0,115163669	0,108225893	-1,064104594	0,28728132	0,648651236
Nanp	4,267515945	0,085400463	0,794180866	0,107532763	0,914366335	NA
Pbld2	4619,097254	0,044334568	0,10500591	0,422210214	0,672871587	0,888566554
Luc7l3	1024,159524	0,056428282	0,115931714	0,486737241	0,62644455	0,865266164

Nsdhl	3474,716847	0,824448245	0,149783313	5,504272999	3,71E-08	1,08E-05
Dock7	391,5103128	0,639446564	0,197843469	3,232083257	0,001228912	0,02761232
Snx2	1050,253772	0,084948503	0,080644544	1,05336949	0,292171682	0,653967601
Gabarapl2	706,3119656	-0,268427657	0,11444872	-2,345396763	0,019006841	0,153536232
Lamtor5	632,8523749	0,075560641	0,105748296	0,714532946	0,474897691	0,788782114
Mrps24	898,2104787	0,110374769	0,126142539	0,875000372	0,381573703	0,72637014
Mastl	9,95008037	-0,241462858	0,524737601	-0,460159245	0,645401922	0,87491161
Pcdhgb5	2,137666464	0,0919892	1,100958325	0,083553753	0,933411238	NA
2610524H06Rik	6,454262255	0,620502181	0,604643633	1,026227926	0,304784161	0,664100532
Foxn2	267,69381	-0,153867077	0,184799348	-0,832616993	0,405060772	0,744879793
Plekhh1	7,621953795	0,026521013	0,548126862	0,048384808	0,961409567	0,988526411
Myo1e	1574,49584	-0,176152065	0,134017919	-1,314391887	0,188714359	0,536004306
Tanc2	131,8775545	-0,396916253	0,145642374	-2,725280017	0,006424697	0,080122928
Snca	11,65169681	0,410346517	0,654228506	0,627222007	0,530513719	0,819570954
Rab18	2023,941257	-0,062529406	0,134165134	-0,46606301	0,641170399	0,871881542
Pcdhgb2	3,524058955	0,207425755	0,813563379	0,254959553	0,798754339	NA
Capn15	546,6792996	-0,305604404	0,091274763	-3,348180745	0,000813439	0,020557413
Dmrt1	0,427903795	-2,257267924	3,274223637	-0,689405543	0,490568096	NA
Sart1	814,9520511	-0,164163852	0,088147546	-1,862375739	0,062550146	0,30342268
Hs6st1	2857,101784	0,183180014	0,099585622	1,839422298	0,065853097	0,313976481
Mapk6	730,5524773	0,066742186	0,124420235	0,536425493	0,591664516	0,850578037
Cdr2	17,24483933	-0,093288446	0,357936677	-0,260628351	0,794379124	0,937091195
Cdkn2c	119,1394718	0,098274628	0,166469414	0,590346451	0,554958404	0,832592702
Cdkn2b	6,789079853	0,752001582	0,596198651	1,261327213	0,207190981	0,559696145
St3gal1	3543,680883	0,163170663	0,109258238	1,493440373	0,135321952	0,458120833
Doc2a	1,337143672	1,524892509	2,099858332	0,72618828	0,467723356	NA
Tbc1d15	1013,198674	-0,007281365	0,117174082	-0,062141427	0,950450207	0,985972877
Pop4	318,3612784	0,249353096	0,12262026	2,033539116	0,041998086	0,242222783
Nat9	588,5630598	0,144487976	0,117888755	1,225629838	0,220338004	0,574490867
Plin3	1360,647471	0,004171413	0,092963634	0,044871451	0,964209773	0,989447379
Tmem14c	4075,923938	0,226805638	0,107667232	2,106542864	0,035157224	0,219020003
Mtus2	30,08229855	0,879629765	0,336231668	2,616141931	0,008892958	0,097932422
Nanos1	1,485435053	0,045710444	1,251582532	0,036522117	0,970866043	NA
Scyl3	575,6667191	-0,095626709	0,098256729	-0,973233176	0,330437426	0,687043861
BC046251	0,142634598	-0,517475177	3,352475198	-0,154356154	0,877328924	NA
Gm7102	0,122496332	0,780932884	3,352475198	0,232942181	0,815806304	NA
Lyrm9	138,7723745	0,065923551	0,144825078	0,455194305	0,648969457	0,876446263
Slc27a5	23963,53558	0,350161817	0,071656876	4,886646387	1,03E-06	0,000132204
Ntn4	74,51228441	-0,068738026	0,419482262	-0,163863964	0,869838236	0,961775826
Tro	1,39708412	0,655494219	1,570359181	0,417416746	0,676373608	NA
Dpt	97,43388993	-0,07588578	0,581193576	-0,130568855	0,896116382	0,97084116
Hao2	36,16187045	6,25624559	2,370773186	2,638905158	NA	NA
Arl6	62,75047675	0,093746777	0,245361677	0,382075872	0,702405088	0,900494767
Rybp	171,2719647	-0,247198145	0,210706924	-1,173184724	0,240721736	0,598180092
Eif4e2	1137,691483	-0,030426795	0,08464908	-0,359446259	0,719261274	0,908608521
Srpk3	3,432493398	0,291018973	0,750306159	0,387866966	0,698114486	NA
Fscn3	0,086476712	0,780932884	3,352475198	0,232942181	0,815806304	NA
Tmem63c	0,299593163	1,742274127	3,264336744	0,533729901	0,593528419	NA
Zfp72	44,47080216	0,028313331	0,227307192	0,124559769	0,90087208	0,972526743
Trnau1ap	327,9575814	0,257811996	0,10846066	2,377009287	0,017453649	0,1461101
Iqgap2	7785,729115	-0,035305795	0,138953449	-0,254083619	0,799430958	0,93892144
Eri2	111,1370963	0,239143948	0,141652064	1,688248949	0,091363451	0,375249761
Fbxo24	0,273083462	-0,658048199	3,340448562	-0,196993963	0,843832262	NA
Rprd1b	688,0034385	-0,070847504	0,089658069	-0,790196632	0,429412943	0,760857915
Fuz	234,0176135	-0,036789065	0,177184316	-0,207631612	0,83551662	0,951141594
Ppm1d	123,5160653	0,223110596	0,165804117	1,345627601	0,178422646	0,520853371
Ccs	4410,924295	0,05955676	0,134069784	0,444222088	0,656882024	0,880121846
Slc26a3	0,174042788	-1,166820872	3,345560292	-0,348766954	0,727264274	NA
Tagln3	6,121756506	-0,399583355	0,66914527	-0,597154868	0,550403988	0,830045225
Ranbp9	742,9649759	-0,138632259	0,146261302	-0,947839632	0,343211101	0,695819585
Clec4e	1,078939737	1,144553941	1,483108195	0,77172653	0,440276414	NA
Abcb11	21103,71707	0,229471752	0,146334399	1,568132674	0,116850173	0,424156926
Rbpms	945,3655265	-0,007316617	0,095665064	-0,076481599	0,939035953	0,982918677
Sepsecs	444,5974471	0,086611992	0,196358302	0,441091571	0,659146706	0,881654058
Lacc1	363,4143105	0,531853206	0,182611589	2,912483306	0,003585674	0,055805438
Prrt3	0,349600122	-0,716166738	2,64656705	-0,270602152	0,786697041	NA

Nckap5	11,15736863	0,199094563	0,614741154	0,323867308	0,746038495	0,919003408
Zfp180	352,5725611	-0,130308998	0,12001476	-1,085774767	0,277578653	0,638252527
Mir5098	0,086476712	0,780932884	3,352475198	0,232942181	0,815806304	NA
Aard	17,65956359	-0,206194392	0,537986135	-0,38327083	0,701518966	0,900058258
Mir3076	0,348085575	-2,032397377	3,287587523	-0,618203276	0,536441351	NA
Mir466n	0,643787279	-1,992088979	1,915137823	-1,04018048	0,298256059	NA
Mir3082	12,82685269	0,254925758	0,473848732	0,537989744	0,590584129	0,849985922
Tcf25	3273,202572	-0,121054797	0,090669795	-1,335117133	0,181838013	0,526302748
Rgs9bp	2,645567837	-1,086646552	1,301294706	-0,835050314	0,40368938	NA
Cyp3a25	17604,58925	0,148337136	0,141464642	1,048581003	0,294371003	0,655361522
Maged1	2835,653957	-0,418883875	0,100887485	-4,151990469	3,30E-05	0,001984851
Slc30a9	1111,39182	-0,191208585	0,098601743	-1,93920086	0,052476885	0,276416937
Kcne4	5,962374989	0,127943548	0,630537929	0,202911739	0,839204019	0,951362994
Rgr	0,314545931	-0,664815037	3,254120508	-0,204299452	0,838119498	NA
Cdon	45,46267183	0,072074996	0,278333932	0,258951524	0,795672643	0,937598879
Dgcr14	184,5913397	-0,002936788	0,14437799	-0,020340966	0,983771376	0,995537017
Skiv2l	1808,676555	-0,2029349	0,085324992	-2,378375853	0,017389091	0,145962115
Tnr	0,321277392	0,66755313	3,239687753	0,206054775	0,836748125	NA
Sec61b	1236,691713	-0,225842634	0,133262482	-1,694720303	0,090128492	0,372419313
Lpin2	4200,992136	-0,169289674	0,202960372	-0,834102106	0,404223448	0,744376512
Tmod1	6,562011637	1,254470651	0,732372126	1,712886941	0,086733341	0,365547481
Sppl2a	3600,511065	-0,105363573	0,111789785	-0,94251522	0,345928923	0,698434374
Mir6340	0,12663974	0,780932884	3,352475198	0,232942181	0,815806304	NA
Ubqln2	444,5233955	-0,337264597	0,14438347	-2,335894805	0,019496725	0,155528431
Nme6	509,8224325	-0,627806866	0,178420533	-3,518691804	0,00043368	0,013206994
Gas8	69,76329368	0,439711049	0,204300573	2,152275166	0,031375689	0,205618422
Nfat5	665,8534738	-0,056563231	0,133942003	-0,422296442	0,672808655	0,888566554
Golga4	1629,836484	-0,491285128	0,111688854	-4,398694324	1,09E-05	0,000883538
Akap7	139,3639567	0,403256741	0,169438591	2,379958073	0,017314608	0,145675893
Prpf19	2930,838425	0,042238133	0,085242424	0,495506008	0,620242982	0,862315769
Kdelr3	8,732403303	0,209513934	0,497314594	0,421290541	0,673542937	0,888626355
Pim2	118,2062231	0,028552743	0,177340486	0,161005217	0,872089292	0,962516448
Camk1	330,3483223	-0,112875775	0,098071595	-1,15095279	0,249751658	0,60896459
Pde1a	47,37518891	0,321447537	0,238667816	1,346840735	0,178031532	0,520240034
Vgll4	183,5537266	-0,299455487	0,202563026	-1,478332411	0,139318827	0,464245611
Ccz1	862,5858461	0,177028436	0,098462273	1,797931628	0,072187843	0,329841479
Ythdc1	669,8312477	-0,039753256	0,096329791	-0,412678736	0,679842006	0,891462871
Ablim2	60,46151369	0,196114523	0,209035112	0,938189383	0,348147093	0,70022243
A930004D18Rik	34,59741219	-0,491417961	0,362026743	-1,357407899	0,17465164	0,517225036
Gm17762	8,401910671	0,070000507	0,571836135	0,122413577	0,902571487	0,973338566
Il20rb	14,51927293	-0,76093527	0,396297767	-1,920109911	0,054844018	0,283546844
Gm5113	31,96292302	-0,518790724	0,285303798	-1,818380012	0,069006071	0,321433335
Tmem216	76,01505473	0,162054122	0,18165925	0,89207746	0,372351416	0,719447489
Fitm1	326,644235	-0,249637549	0,533524159	-0,467902989	0,639853966	0,871049783
Golt1a	340,5851836	0,296433437	0,110479484	2,683153713	0,007293146	0,0865959
Haus7	316,5683756	0,30486304	0,16338502	1,865917938	0,062052848	0,30232672
Btbd9	333,3292033	0,005827643	0,130573349	0,044631179	0,96440129	0,989567952
Slirp	443,2256123	0,037007992	0,130395003	0,283814496	0,776552545	0,930009104
Cd209f	15,5539284	-0,979157976	0,557395115	-1,756667664	0,078974472	0,348906222
Mettl7a1	6928,439063	-0,10449802	0,09055837	-1,153929994	0,248528871	0,60747894
Scrn1	0,827150508	0,645301954	1,7250377	0,374079914	0,708344862	NA
Lrrc51	58,69789531	0,147001871	0,234712453	0,626306228	0,531114102	0,819884984
4930426L09Rik	0,596777398	0,429548764	1,905334635	0,225445314	0,821632864	NA
Taf4	327,3829523	-0,095146302	0,098835998	-0,962668494	0,335713913	0,692189898
Gm3230	4,209983446	-0,040595888	0,663521665	-0,06118246	0,951213899	NA
Tff3	327,3909331	1,300592315	1,371173163	0,948525212	NA	NA
Gulp1	6,258669127	-1,012237197	0,743639917	-1,36119266	0,173452813	0,515882238
Cenpv	332,3873725	0,18653836	0,113337124	1,64587166	0,099790185	0,392993389
Prrc1	921,4503016	-0,193401342	0,112881699	-1,713309985	0,086655526	0,365341448
Trip11	289,3088617	-0,334101073	0,19189032	-1,741104361	0,081665287	0,353542587
Abracl	176,1953546	0,366933618	0,154173789	2,380000007	0,017312638	0,145675893
Ssbp1	720,5266082	0,10087609	0,096686581	1,04333082	0,296795102	0,657874966
Ppil6	11,44400429	0,881260222	0,481558017	1,830018794	0,067247129	0,317560326
Fut11	96,85112805	-0,19901561	0,20496262	-0,970984901	0,331555798	0,688274004
2610044O15Rik8	75,83126849	-0,076698609	0,169655926	-0,452083288	0,651208982	0,877445362
Cep68	279,5857668	0,037150666	0,12079269	0,307557234	0,758419266	0,924275949

Supplementary Table S1: *Serpina1* KO vs. wildtype all DEGs

Camk2n2	55,08432235	-0,370730317	0,414629796	-0,894123676	0,371255723	0,719112906
Fam217b	2,076427743	-2,124731308	1,072449868	-1,98119406	0,047569515	NA
Tshz1	323,912211	-0,17669354	0,117546546	-1,503179341	0,132792803	0,453347035
Klf3	718,5369497	-0,227693137	0,156390831	-1,455923818	0,145413681	0,474192695
Klc2	220,9496429	-0,235878828	0,14649806	-1,610115709	0,107372598	0,406762331
Kif5c	4,378620313	0,739820203	0,826558022	0,895061427	0,370754252	NA
Kif5b	775,3670482	-0,138870484	0,165888337	-0,837132293	0,40251821	0,742634347
Kif4	9,266295467	-1,434797042	0,51223067	-2,801076012	0,005093252	0,069898722
Kif3c	41,67779876	0,022220013	0,278647531	0,079742364	0,936442165	0,982191945
Kif3b	329,2676481	-0,120367581	0,12728158	-0,945679505	0,344312079	0,697519845
Kif3a	128,9395483	0,04581589	0,155886376	0,293905672	0,768829983	0,927598042
Kif2a	185,8513738	0,001855191	0,142739303	0,012997057	0,989630141	0,996712773
Kif1a	6,366352285	0,577730297	0,607792854	0,950538153	0,341838877	0,695250579
Naa25	400,9862944	0,052815014	0,147958795	0,356957584	0,72112356	0,909414289
Akap8	786,0221032	0,034076935	0,117584358	0,289808406	0,771962817	0,928273961
Sel1l3	484,2041311	-0,625505165	0,164309593	-3,806869428	0,000140737	0,005789271
Efcab14	384,8014857	-0,089458656	0,099604762	-0,898136337	0,369112869	0,717291119
Hscb	70,29575276	0,604632099	0,184843917	3,271041371	0,001071522	0,025000318
Dnajb13	5,995504038	0,235766265	0,682038532	0,345678805	0,729584114	0,911594858
Prpf38a	232,4652687	0,103763491	0,136607647	0,759573081	0,447509815	0,772766561
Ppp1cb	1959,889005	-0,089163394	0,162792114	-0,54771323	0,583888827	0,847068435
9330182L06Rik	11,23996193	0,561990291	0,472592152	1,189165518	0,234374541	0,591595413
Phf13	142,7978529	0,090173937	0,152899048	0,589761273	0,55535071	0,832808035
Plaa	1415,920031	-0,017831679	0,11088042	-0,160819008	0,872235954	0,96259885
Dnajc11	1381,215651	-0,173829867	0,102094362	-1,702639239	0,088635602	0,369089997
Slc22a29	24,32404323	3,545252302	1,186687398	2,987519971	NA	NA
Slc38a7	451,3348703	-0,155326248	0,096077005	-1,616684943	0,105946306	0,403646669
Atrip	138,8158036	0,051609852	0,174841294	0,29518114	0,767855508	0,927586634
Rbm34	238,8966089	0,091488362	0,161515915	0,566435587	0,571097709	0,8413217
Creb5	5,468654183	0,989026661	0,644096395	1,535525845	0,124654714	0,439132771
Gba2	242,7735767	0,013702957	0,130399757	0,105084221	0,91630898	0,976559041
Nod1	337,0082541	0,091497354	0,131446616	0,696079955	0,486378759	0,794938512
Itih5	650,6532198	-0,431611769	0,145039174	-2,975828925	0,002921978	0,048108283
Uba6	161,892558	-0,301295796	0,202149065	-1,490463469	0,136102415	0,458903704
2900026A02Rik	1820,718783	-0,252750213	0,117991961	-2,142096903	0,032185685	0,208308055
Slc25a24	31,68012272	0,209603697	0,33984678	0,616759403	0,537393433	0,824184101
Rsbn1	138,6781521	0,11005488	0,195992851	0,561524973	0,574439711	0,84308755
Pogz	385,3681229	-0,069812665	0,114567717	-0,609357215	0,542287691	0,826611597
1190002N15Rik	789,2602463	-0,43956588	0,203600344	-2,158964332	0,030852934	0,203790947
Rnf166	239,2665493	0,078001771	0,146152877	0,533699868	0,593549201	0,851620901
Nrxn3	2,109863276	0,499536353	1,365928569	0,365711915	0,714580054	NA
4930415O20Rik	0,358654812	1,937230844	3,118014193	0,621302767	0,534400434	NA
Lancl1	523,4073152	-0,026977937	0,147066957	-0,183439824	0,854452931	0,957135493
Kif17	0,681498711	-1,230025877	1,794891046	-0,685292781	0,493159186	NA
Ikzf1	51,04385365	0,43314142	0,237832426	1,821204225	0,068575818	0,320443941
Ugt2b5	20525,67641	-0,436183529	0,206816314	-2,109038307	0,034941275	0,218285004
Ugdh	5021,948919	0,12506166	0,182361563	0,68578958	0,492845808	0,798745371
Hoxc8	0,113662148	0,780932884	3,352475198	0,232942181	0,815806304	NA
Hoxc6	0,460894724	1,234792144	2,492970198	0,495309629	0,620381575	NA
D830031N03Rik	19,52596369	-1,166101102	0,4618177	-2,525024704	0,011569016	0,115136419
Strn3	590,2788794	-0,117257917	0,138060306	-0,849323901	0,395701085	0,737426273
Taok2	875,6738237	-0,176921613	0,114796803	-1,54117195	0,123274942	0,43588421
U2af1	1270,721145	-0,067504426	0,107840001	-0,625968339	0,531335708	0,819884984
Dxo	487,3451876	-0,343499464	0,122694738	-2,799626707	0,005116173	0,069899835
Atp6v1a	965,4710945	-0,147789852	0,120721278	-1,224223724	0,220867842	0,575078806
Atf3	39,57359201	-0,150296408	0,332968983	-0,451382609	0,651713815	0,877662367
Atp5a1	27195,11729	0,040630969	0,075638892	0,537170339	0,591149958	0,850384506
Gnat1	129,5312725	1,006682969	0,609822732	1,65077967	0,098783573	0,391880589
Maob	6064,604676	0,423587126	0,162446047	2,60755577	0,009119121	0,099447036
Vdac3	1453,13055	0,057673307	0,094447549	0,610638469	0,54143895	0,826407188
Hsf4	182,4428185	-0,230423537	0,146490546	-1,572958409	0,115728449	0,421458359
Myo7a	391,7784943	-0,162071944	0,166294764	-0,974606417	0,329755529	0,6866113
Chmp1a	1066,781713	0,092807909	0,073837228	1,256925698	0,208780542	0,561381148
Klhdc4	451,2498148	-0,095116553	0,117355937	-0,810496298	0,417654992	0,752229411
Cbr4	554,0358556	0,148849663	0,082316764	1,808254546	0,070566893	0,324865285
Reep6	21382,25809	-0,033398435	0,090610211	-0,368594612	0,712429912	0,905518486

Aff1	770,7665648	-0,106316826	0,137685911	-0,772169247	0,440014193	0,767402932
Tox	70,99697032	1,378903269	0,433582275	3,180257467	0,001471443	0,031027942
Ndrg4	2,535526073	-0,392610413	0,920909767	-0,426328862	0,66986823	NA
Dkk4	1,67014397	-3,532885811	1,460976933	-2,418166729	0,015598928	NA
Cngb1	1,104951234	1,16380478	1,629757218	0,714097024	0,475167186	NA
Polr1c	354,352617	-0,055355057	0,120184595	-0,460583632	0,645097357	0,874664897
Rpl28	5907,653723	-0,038904186	0,111487165	-0,348956634	0,727121866	0,910689961
Snora43	2,929581232	-1,463569266	0,857048101	-1,707686258	0,087694573	NA
Snora17	2,999045202	0,842001199	0,854367239	0,985526084	0,324365725	NA
Plcl1	11,54288354	-0,514059586	0,438243988	-1,172998606	0,240796365	0,59825434
Mfsd4a	0,962545609	0,925956935	1,510190962	0,613138973	0,539784453	NA
Ccdc162	131,2514307	0,329804585	0,256867354	1,283949012	0,199159797	0,549010742
Ccdc85b	146,8210331	-0,3035466	0,177047105	-1,714496259	0,086437624	0,364537725
Tmem123	1021,315357	0,068098779	0,128959441	0,528063545	0,597455232	0,853549036
Antxr2	940,3532477	0,327769963	0,18294912	1,791590811	0,073198536	0,333194154
Tslp	2,84228723	0,488918999	0,964143583	0,507101855	0,612083354	NA
Gfra2	20,21770413	0,200017676	0,421128689	0,474956186	0,634818203	0,868410828
Arhgdig	0,086476712	0,780932884	3,352475198	0,232942181	0,815806304	NA
Lta4h	966,5227419	0,078907908	0,112523275	0,701258545	0,483141679	0,79399946
Gdf9	6,490778499	-0,262858752	0,551644766	-0,476500038	0,63371818	0,867989994
Gcsam	2,840438948	0,84703947	0,833279563	1,016512954	0,309385182	NA
Eral1	368,603557	-0,104676547	0,10604091	-0,987133616	0,32357714	0,680937786
Ramp3	2,434761198	0,025225924	1,032748652	0,024426005	0,980512805	NA
Mad2l1	66,11890066	-0,406728827	0,262323372	-1,550486422	0,12102481	0,432962452
Ly6i	0,347232576	0,667197765	3,168193746	0,210592476	0,833205284	NA
Zfp276	423,1043731	0,248237017	0,130381197	1,903932642	0,056918957	0,288295377
Mir7237	0,219943115	0,059593471	3,352475198	0,017775962	0,985817581	NA
Ubap1	1413,940737	-0,055353611	0,10344418	-0,535106093	0,592576497	0,851332066
Hdx	0,093303375	-0,517475177	3,352475198	-0,154356154	0,877328924	NA
Zfp428	36,85144407	-0,522128432	0,29341656	-1,779478402	0,075161363	0,338897895
Adamts13	122,6852418	-0,2085066	0,186882703	-1,115708394	0,264546976	0,6255268
Rhot1	837,3552357	-0,052768696	0,084515427	-0,624367619	0,532386185	0,82055233
Foxc1	2,22940008	1,142668456	1,113695547	1,026015107	0,304884463	NA
Mageb3	0,274382903	-0,66487896	3,339949097	-0,199068591	0,84220909	NA
Grid2ip	0,174042788	-1,166820872	3,345560292	-0,348766954	0,727264274	NA
Serpinf2	21093,05246	0,112787014	0,080533611	1,400496176	0,161364788	0,497950692
Laptm4a	4357,93131	-0,001064215	0,060592904	-0,017563356	0,98598719	0,995969984
Dirc2	844,5883018	-0,050618417	0,161053246	-0,314296165	0,75329611	0,921533083
Spsb2	277,3311922	0,122625086	0,122472893	1,001242664	0,316709505	0,674637563
Mir6387	0,086476712	0,780932884	3,352475198	0,232942181	0,815806304	NA
Wdr7	598,5145687	-0,215742312	0,122974744	-1,754362767	0,079368367	0,349223642
Fbxo44	100,6790439	0,086219405	0,191798637	0,449530853	0,653048757	0,877889274
Ctr9	779,4013167	-0,157959159	0,08027919	-1,967622723	0,049111463	0,265388501
Col4a3	7,461748703	1,181858148	0,768665807	1,537544843	0,124159943	0,437955618
5930430L01Rik	44,79589896	0,176629809	0,282321048	0,625634576	0,531554654	0,819884984
9630001P10Rik	0,147722973	0,780932884	3,352475198	0,232942181	0,815806304	NA
AW112010	1852,01585	0,382583102	0,1412392	2,708760039	0,006753517	0,082756886
6430710C18Rik	2,050631104	0,302652037	1,037395053	0,291742317	0,770483651	NA
Szrd1	2220,209935	-0,181741938	0,091485265	-1,986570603	0,046970002	0,258093692
4921504A21Rik	0,600275691	0,828172684	2,340164705	0,353895041	0,723417544	NA
9330162012Rik	106,2294879	0,258837199	0,166835913	1,551447731	0,120794424	0,432485446
6030443J06Rik	11,79320244	0,153811943	0,482617662	0,31870351	0,749951352	0,920166276
D630024D03Rik	16,36366791	0,382671592	0,515721275	0,742012421	0,45807981	0,779143636
Stap2	952,4172174	0,154875563	0,138134516	1,121193804	0,262205376	0,622562722
D830032E09Rik	2,132298753	-0,543537542	0,927745351	-0,585869324	0,557963333	NA
Nfatc4	11,41585075	-0,097558922	0,507115799	-0,192379969	0,847444581	0,953880403
Zfp229	142,1920023	-0,00603153	0,201643155	-0,029911902	0,976137314	0,993495054
1700084E18Rik	7,195602857	-0,379264187	0,552428514	-0,686539846	0,492372746	0,798476599
Ube2l6	1727,625784	-0,032195496	0,164869425	-0,195278758	0,845174726	0,953247119
Mapk8ip1	39,44697975	0,014273149	0,249414047	0,057226723	0,954364591	0,986616875
Ywhaz	2020,306085	0,042650085	0,144763496	0,294619061	0,7682849	0,927598042
Ranbp3	714,9041834	-0,05381395	0,075962533	-0,708427534	0,478679799	0,79113243
Ppp2r2a	516,9335957	0,07270746	0,12142178	0,598800804	0,549305726	0,829418144
Zdhhc13	147,7219876	-0,15787248	0,230674615	-0,684394682	0,493725973	0,7992036
Gpx4	5492,432339	0,12993568	0,119046141	1,091473267	0,275064689	0,6367688
Crybg3	328,3217871	-0,245077905	0,19276859	-1,271358084	0,203601287	0,556407525

Supplementary Table S1: *Serpina1* KO vs. wildtype all DEGs

C2cd2	4211,27571	0,099066346	0,117355728	0,844154327	0,398583173	0,739635153
Glyctk	5897,551269	-0,254977538	0,066463731	-3,836341011	0,000124881	0,005295032
Mrpl40	587,9389185	0,039549257	0,105555313	0,374678028	0,707899935	0,902731692
Nat2	423,11825	0,165432638	0,117033206	1,413552987	0,157493193	0,493444223
Cdk7	383,1951591	-0,147057073	0,112145746	-1,311303179	0,189755355	0,537579856
Mas1	1,333062301	1,053649866	1,581431804	0,666263232	0,505242862	NA
Chrd	153,3783556	-0,369251424	0,177112956	-2,084835758	0,037084212	0,225867346
Zscan12	148,2621089	-0,122933717	0,135402861	-0,907910782	0,363925361	0,712843608
Vamp8	2538,249662	0,13862079	0,087193284	1,5898104	0,111877549	0,413860252
Abcc6	3341,147945	-0,037664399	0,109163134	-0,345028557	0,730072902	0,911670524
Apba3	843,5657487	-0,272627222	0,10232095	-2,664432097	0,007711843	0,089429935
Chst3	6,632725801	0,11113896	0,616752159	0,180200358	0,856995277	0,958117567
Txnl1	2737,951186	-0,100906566	0,107180021	-0,941468057	0,346465051	0,698497639
Zfp407	167,3428362	-0,121535454	0,16631073	-0,73077338	0,464917586	0,781663619
Zbtb5	277,2909856	-0,25561387	0,109841011	-2,327125976	0,019958561	0,157885211
Gata4	590,830346	-0,223575417	0,179237642	-1,247368657	0,212262354	0,565238523
Wfdc16	0,463855677	-0,107586204	2,186279157	-0,049209729	0,960752158	NA
Mir292b	7,00548851	-0,829781959	0,712046304	-1,165348313	0,24387803	0,60244002
Gm5294	0,692304991	2,837002898	2,260335926	1,255124455	0,209433584	NA
Pdzd7	2,042265673	3,632992705	1,330537163	2,730470675	0,006324396	NA
Tgfbr3l	77,42832936	-0,103283531	0,294918747	-0,350210122	0,726181011	0,910393262
Gm3336	4,950871067	0,457776681	0,681542445	0,671677435	0,50178907	NA
Kat6b	406,7671544	-0,158168699	0,185577074	-0,852307319	0,394043545	0,736368577
Galnt12	3,397140524	0,623047218	0,755817756	0,824335249	0,409749095	NA
Slco4c1	0,093303375	-0,517475177	3,352475198	-0,154356154	0,877328924	NA
Zfp707	775,3542197	-0,016088834	0,19581951	-0,08216155	0,934518249	0,98189048
Acp5	3328,213722	-0,26617528	0,119637646	-2,224845522	0,02609161	0,185739477
Bdp1	303,860556	-0,234458124	0,142868511	-1,641076275	0,100781583	0,394192212
Slc29a2	26,59151478	0,086059092	0,297361372	0,289409118	0,772268318	0,928273961
Degs1	2194,477334	0,193328522	0,120339819	1,606521636	0,108159345	0,408238928
Thra	455,174253	0,038420672	0,120930749	0,317708047	0,750706406	0,920556312
Kifap3	181,4565577	0,04972115	0,218153557	0,227918126	0,819709891	0,946198293
Tcta	534,3049863	0,097673142	0,119973993	0,814119288	0,415576617	0,751551608
Aldh6a1	9345,511357	0,05000075	0,124730102	0,400871554	0,688514694	0,894099363
Nkx2-3	0,087021394	-0,517475177	3,352475198	-0,154356154	0,877328924	NA
Cdkn2a	0,086476712	0,780932884	3,352475198	0,232942181	0,815806304	NA
Nnt	1222,368044	-0,172997129	0,093346951	-1,853270275	0,063843629	0,307355233
Rras	673,3965274	0,198303876	0,137095973	1,446460247	0,148048154	0,47829017
Mir8093	0,259974817	0,586045239	3,35071904	0,174901337	0,86115716	NA
Mir3061	3,067054963	0,205062624	0,8118053	0,252600746	0,800576753	NA
Mir3960	1,51240598	-0,695853375	1,207554755	-0,576249957	0,564446243	NA
Ccdc167	240,192654	-0,433537214	0,15759112	-2,751025658	0,005940899	0,076590047
Mocos	1945,792591	0,174103967	0,100433962	1,733516872	0,083003841	0,356552758
Cthrc1	0,207615244	0,059593471	3,352475198	0,017775962	0,985817581	NA
Dtwd1	154,6349748	-0,062053782	0,136957704	-0,453087194	0,650485956	0,876831587
Cdc42ep2	88,96708568	-0,191825069	0,250455885	-0,765903621	0,443733651	0,770142004
Snord14a	1,57094695	-1,025479281	1,418165381	-0,723102746	0,469616762	NA
Fat1	966,3258334	-0,216603723	0,118871496	-1,822167046	0,068429643	0,319984575
Ptp4a2	2905,733145	-0,056288789	0,088409168	-0,636684975	0,524330062	0,81632335
Pfkfb1	1146,981691	0,092534372	0,169178553	0,54696278	0,584404303	0,847068435
Gm1110	0,113662148	0,780932884	3,352475198	0,232942181	0,815806304	NA
Rc3h2	278,8882079	0,014462922	0,145940419	0,099101551	0,921057641	0,977341679
Zkscan8	214,2385703	0,020717925	0,140280644	0,147689121	0,882588118	0,966166219
Actr1b	1187,581699	-0,176775965	0,089901005	-1,96634025	0,04925932	0,266079941
Gprc5c	1502,073151	0,109726342	0,085111316	1,289209798	0,19732516	0,546971183
Lcp1	2687,287889	0,116600112	0,071947363	1,620630803	0,105096842	0,40293653
Col25a1	11,34262016	-0,329351046	0,572696158	-0,575088624	0,565231361	0,83747956
Senp6	1086,407798	-0,024584981	0,097211597	-0,252901727	0,800344155	0,939320605
Rhbdd2	292,0657125	-0,015845094	0,10206017	-0,155252476	0,87662228	0,964416829
Glyat	5487,568314	-0,143553113	0,128707175	-1,115346629	0,264701911	0,625782466
Scrn2	860,5081272	0,050362859	0,090804869	0,554627289	0,579149619	0,84530168
Gabrp	0,862356736	-1,085927643	1,607999302	-0,67532843	0,499467126	NA
Bud13	145,4398733	-0,141888043	0,133992466	-1,05892553	0,289633696	0,650994097
Nod2	7,976393809	0,865566472	0,58782575	1,472488186	0,140889095	0,466455253
Vash2	0,287160254	0,617200912	2,785784868	0,221553688	0,824661338	NA
Pcca	2303,302574	0,182145393	0,122949142	1,481469413	0,13848153	0,462146673

Ropn1l	295,6910692	0,698869917	0,144356183	4,841288436	1,29E-06	0,000162698
Ncapd2	93,04168078	-0,41808881	0,195680341	-2,136590771	0,032631291	0,210239869
Sgcz	0,48379559	-1,532734497	2,052695968	-0,746693383	0,455248665	NA
St8sia6	0,807822742	-0,957645984	1,967883119	-0,486637633	0,626515149	NA
Il17d	0,273083462	-0,658048199	3,340448562	-0,196993963	0,843832262	NA
Lctl	0,18660675	-1,235117516	3,340448777	-0,369745983	0,711571767	NA
Eml6	44,06061131	-0,739638221	0,300026047	-2,465246699	0,013691895	0,126862287
Zranb3	73,12864621	0,04466348	0,259891752	0,171854165	0,863552181	0,961272625
Dnajb9	1576,891518	-0,889883548	0,215585904	-4,127744582	3,66E-05	0,002129383
Spag6l	0,748129716	0,377934924	1,627943365	0,232154836	0,816417755	NA
Hist1h3f	0,833660338	0,297053861	1,744408656	0,170289146	0,864782746	NA
Gm16702	0,295445946	1,389394708	3,349408814	0,414817893	0,67827522	NA
4933440M02Rik	0,23615848	1,389394708	3,349408814	0,414817893	0,67827522	NA
Gm16907	2,74283515	0,70380085	1,220223871	0,576780104	0,564088012	NA
Gm10390	0,974824756	0,332076247	1,648046125	0,201496938	0,840310024	NA
I730030J21Rik	3,710688156	0,648376027	0,892790704	0,72623519	0,467694602	NA
Zfp652os	2,981977308	0,734602341	0,971718627	0,755982566	0,449659649	NA
C730002L08Rik	15,33774182	0,61656508	0,608121038	1,013885463	0,310637409	0,669972995
9530080O11Rik	11,3271289	0,641856282	0,428486271	1,497962304	0,134143045	0,456110978
Gm15350	0,443168919	2,196464572	3,313880474	0,662807421	0,5074539	NA
A530072M11Rik	22,5875709	0,152343777	0,351636829	0,433241812	0,664839106	0,884623407
0610031O16Rik	645,2170304	0,031917681	0,130020925	0,245481112	0,806083931	0,941297123
Gm16596	0,087021394	-0,517475177	3,352475198	-0,154356154	0,877328924	NA
Gm19710	4,972236136	-0,427879093	0,812490025	-0,526626887	0,598452717	NA
Gm16675	14,11258074	0,189021979	0,456555508	0,414017521	0,678861275	0,890822182
Mirt2	35,33939473	0,424812067	0,335305787	1,266939263	0,205177008	0,558203382
Gm10865	2,93345061	-0,166882195	0,879410838	-0,189765907	0,849492573	NA
Gm16853	0,415230487	-0,057645711	3,039527376	-0,018965354	0,984868744	NA
Mir140	0,093953095	-0,517475177	3,352475198	-0,154356154	0,877328924	NA
Tlr12	184,1780261	0,232085354	0,249603549	0,929815923	0,352466401	0,70347633
Scarna2	0,293986917	-0,62664923	2,757017991	-0,227292398	0,820196384	NA
Drp2	0,179780087	0,059593471	3,352475198	0,017775962	0,985817581	NA
Hdac8	135,4395103	0,242598989	0,1363634	1,779062338	0,075229542	0,33897666
Slc1a6	0,343089167	0,663808922	2,658186063	0,24972252	0,802801942	NA
Tnfsf9	1,846429982	0,822227817	1,468423086	0,559939315	0,575520831	NA
S1pr3	63,22150839	-0,348682565	0,215449723	-1,618394116	0,105577689	0,403046295
Ptprn	0,531224331	-1,676853381	2,031852758	-0,825282922	0,409210985	NA
Nos3	58,12121107	0,004855486	0,22733365	0,021358413	0,982959748	0,995413661
Dtwd2	39,73208244	0,235026946	0,267350585	0,879096434	0,379348991	0,725074694
Pde6c	9,265493021	1,620871881	0,660570667	2,453744864	0,014137725	0,129462863
Hmox2	1231,441398	0,1314273	0,109598679	1,199168652	0,230462374	0,586979539
Ctps2	251,721112	0,597083575	0,149246781	4,000646257	6,32E-05	0,003198925
Rnf25	414,5388882	-0,06769923	0,127516905	-0,53090396	0,595485334	0,852999403
BC025920	25,56668633	0,595087838	0,393445484	1,512503924	0,130405715	0,448869806
D930016D06Rik	167,3182604	-0,123888051	0,160763331	-0,770623812	0,440929943	0,768480766
1700034H15Rik	4,390167966	-0,438036856	0,715053093	-0,612593471	0,540145176	NA
4930581F22Rik	295,0231083	0,491774972	0,177441088	2,77148307	0,005580157	0,073282047
Ppp1r3fos	4,892629636	0,118911001	0,722686092	0,16454032	0,869305808	NA
D4Ertd617e	0,953722851	-0,199684738	1,541851869	-0,129509677	0,896954368	NA
Snhg12	104,2280502	0,16060335	0,226053736	0,710465365	0,477415601	0,790092068
5033406O09Rik	176,2928128	-0,41004215	0,15682963	-2,614570663	0,008933968	0,098141508
6530402F18Rik	14,75255887	-0,007999714	0,4431239	-0,018052997	0,985596575	0,995899882
Rmi1	173,6899385	0,112780825	0,143456376	0,78616809	0,431769018	0,7622164
G530011O06Rik	8,453387485	-0,525084922	0,712847833	-0,736601694	0,461364604	0,781516914
Mccc1os	3,900306476	0,402844023	0,817446447	0,492807846	0,622148366	NA
C530005A16Rik	16,38931742	0,220949222	0,3980207	0,555119928	0,578812633	0,84530168
4933407K13Rik	18,46017	0,169309224	0,370139411	0,457420146	0,647369087	0,876230372
Gm13483	3,797507994	-0,600618144	0,714367257	-0,84076942	0,400477122	NA
Plekhb2	424,6760447	-0,146728041	0,124653673	-1,177085584	0,239161348	0,596519262
Cdk6	437,854736	-0,079556819	0,154464993	-0,515047567	0,606519781	0,856870226
Fam184b	13,29593195	0,599290481	0,457604621	1,309625065	0,190322705	0,538730519
Impg1	0,086476712	0,780932884	3,352475198	0,232942181	0,815806304	NA
Peli1	401,8737584	-0,174350854	0,129162286	-1,349858847	0,177061264	0,520079413
1500015O10Rik	0,302276418	0,61733957	2,745727361	0,224836442	0,822106515	NA
Ipo4	859,0838242	-0,18041186	0,134633932	-1,340017758	0,180239572	0,523262288
Gtf2h2	277,7754327	0,008921177	0,106053445	0,084119636	0,932961313	0,981257162

Supplementary Table S1: *Serpina1* KO vs. wildtype all DEGs

Foxj2	256,0253714	0,025915695	0,117241082	0,221046193	0,825056465	0,947537943
Ptprt	10,52417883	-0,704602034	0,467996259	-1,505571938	0,132177097	0,45288846
Ndufa7	1926,679008	0,253111699	0,080759008	3,134160594	0,001723465	0,034651586
Crip2	2003,739301	0,090688558	0,1235628	0,733947099	0,46298098	0,781516914
Yaf2	313,6380514	-0,15425417	0,153159965	-1,0071442	0,313865483	0,672554713
Gucy1a3	159,2134875	0,048121185	0,204950175	0,234794556	0,814368193	0,944076707
Actn4	4718,153215	-0,05395816	0,077356352	-0,697527204	0,485472921	0,79430833
Rps19	2915,465145	-0,020424748	0,134339583	-0,152038194	0,879156812	0,965347075
Cd274	57,19533902	0,597596135	0,299669919	1,994181251	0,04613225	0,255211169
Fignl1	11,4992061	-1,410793651	0,533166249	-2,646067063	0,008143367	0,092812169
Mkrn2	319,5019564	0,085850252	0,105631669	0,81273214	0,41637165	0,751673636
Nfe2l2	2005,157149	0,03685944	0,111260917	0,331288296	0,740426731	0,916128178
Rad18	20,48973033	-0,057228356	0,442127688	-0,129438525	0,897010666	0,971044462
Selenos	2255,873936	-0,310018471	0,115797259	-2,677252235	0,007422873	0,087576343
Fam188a	868,493915	-0,205478161	0,170892369	-1,202383476	0,229214988	0,585109066
Txnip	1082,900738	0,09660115	0,23450035	0,411944589	0,680380038	0,891658301
Cenph	4,36701928	-1,137505751	0,817541784	-1,391373228	0,164112279	NA
Mrps34	1013,410197	0,083003973	0,128433742	0,646278558	0,518098972	0,813633872
Fastk	899,4268261	-0,078752284	0,098621753	-0,798528533	0,424563842	0,757304069
Pga5	0,087021394	-0,517475177	3,352475198	-0,154356154	0,877328924	NA
Pmaip1	15,73636833	0,390754314	0,397291568	0,983545451	0,325339057	0,682710621
Cars2	313,4672748	-0,331488142	0,149117666	-2,222997123	0,026215994	0,186426396
Nudt11	1,633626402	-0,466128099	1,200661197	-0,388226171	0,697848667	NA
H2-Eb1	617,8158464	0,645553493	0,24183853	2,669357491	0,007599652	0,088888663
Elp2	925,0270891	-0,063920703	0,089005129	-0,718168757	0,47265324	0,787019872
Gpr62	0,087021394	-0,517475177	3,352475198	-0,154356154	0,877328924	NA
Gfer	834,6787226	-0,033389695	0,105266515	-0,317191984	0,751097932	0,920556312
Rab2a	2507,415527	-0,041844347	0,076443943	-0,547386036	0,584113547	0,847068435
Ngly1	1010,784024	0,082550784	0,087557203	0,94282116	0,345772386	0,698434374
Ahcyl2	494,2061875	-0,210557057	0,180150501	-1,168784186	0,242490593	0,60078887
Lrit1	397,6756415	0,127558248	0,149901751	0,850945685	0,394799525	0,736750517
Pdcd1lg2	1,279635555	-0,582281529	1,400072434	-0,41589386	0,677487672	NA
Hyou1	7002,411624	-1,146946816	0,21167488	-5,41843613	6,01E-08	1,49E-05
Rfc5	197,0589107	0,12539509	0,144672243	0,866752929	0,386077398	0,730258734
Frmd6	108,3429464	0,117619224	0,159203718	0,738796967	0,460030294	0,780953195
Zbtb46	47,88726018	-0,112672994	0,2253167	-0,500064992	0,617029315	0,860830256
Slc37a3	574,2715272	0,002604017	0,115867647	0,022474062	0,982069802	0,995338444
Slc14a1	6,517836882	0,45724603	0,68035769	0,672067116	0,50154097	0,803893049
Cul4b	784,8869861	0,100145175	0,098917219	1,012413978	0,311340158	0,670895406
Cep89	138,2945658	0,029118529	0,142978848	0,203656204	0,838622169	0,951141594
Wdsub1	99,26346253	0,323809278	0,178727902	1,811744418	0,070025697	0,32382454
Pygo1	3,89771528	1,166437316	0,89638929	1,301261996	0,193168791	NA
Ndc1	133,2781856	-0,344489107	0,149930443	-2,297659504	0,021581176	0,163772191
Zfp719	86,46683827	0,031822196	0,220147582	0,144549378	0,885066668	0,967336191
Diaph2	339,3035624	0,128303854	0,157281704	0,815758293	0,414638391	0,751551608
Dennd2a	78,72073664	-0,108240282	0,262950302	-0,411637793	0,680604926	0,89166825
Tmc7	92,37886366	-0,692834374	0,184646571	-3,752219019	0,000175276	0,006871753
Tyw3	47,8503117	0,213457847	0,230049355	0,927878483	0,353470607	0,704458903
Fbxo7	441,4745236	-0,000813235	0,091905261	-0,008848622	0,992939913	0,998016217
Kctd11	52,41576906	-0,057135751	0,267727797	-0,213409856	0,831007293	0,948982362
Clic6	3,551337071	0,834826413	1,100512024	0,758580002	0,448103839	NA
Dcaf10	502,8954734	-0,337630037	0,144383059	-2,338432489	0,019364824	0,155304678
Zfp771	171,3248102	-0,089109147	0,21472719	-0,414987721	0,678150892	0,89063303
Lamp3	0,269274339	0,059593471	3,352475198	0,017775962	0,985817581	NA
Dab1	4,884948482	0,744677379	0,89699933	0,83018722	0,40643294	NA
Gm13582	0,087021394	-0,517475177	3,352475198	-0,154356154	0,877328924	NA
Armcx2	57,77431733	0,068286776	0,252453991	0,270491964	0,786781799	0,934196858
Alg9	388,5633692	0,043385069	0,118044864	0,367530343	0,713223463	0,905933635
Dnajc9	151,4015686	-0,134104263	0,13376131	-1,002563918	0,316071312	0,67419376
Hepacam	1,051073307	1,448240186	1,744059114	0,830384804	0,406321255	NA
Slc22a13	0,093303375	-0,517475177	3,352475198	-0,154356154	0,877328924	NA
Chmp7	907,8113014	-0,148078893	0,108175255	-1,368879532	0,171036935	0,510970454
Cmtm7	63,97849956	0,378328484	0,253089039	1,494843414	0,134955313	0,45734286
Trf	432072,9266	0,057594925	0,085735778	0,671772342	0,501728639	0,803893049
Mcoln3	0,208973043	1,337107854	3,350237755	0,399108348	0,689813372	NA
Mapre3	860,282148	-0,124503106	0,134369009	-0,926576053	0,354146693	0,704551502

Supplementary Table S1: *Serpina1* KO vs. wildtype all DEGs

Lrrc4	0,467166595	-2,447689718	2,857504183	-0,856583074	0,391675343	NA
Akr1c18	7,409095025	0,469022528	0,855375982	0,548323237	0,583469974	0,846950064
Dock9	381,1192207	-0,058010683	0,141009489	-0,411395596	0,680782483	0,89166825
Capn12	1,334490241	0,983010994	1,624778434	0,605012335	0,545170801	NA
Cntfr	47,480044	-0,2530989	0,312065178	-0,811044994	0,417339832	0,752195715
Cklf	13,18033369	0,095581505	0,467739234	0,204347847	0,838081682	0,951141594
Fuom	602,2128139	0,182305359	0,166668046	1,093823102	0,27403257	0,635693562
Kif21a	1294,121424	-0,083971032	0,104394864	-0,804359803	0,421189209	0,754607148
Alg6	144,9240062	-0,151017844	0,185199706	-0,81543242	0,414824833	0,751551608
Tmem241	109,2049978	0,269266449	0,216907923	1,241385957	0,214463204	0,56766844
Tns1	2066,229701	-0,323096396	0,140926491	-2,292659065	0,021867641	0,165262008
Tmem255a	1,513620607	0,801884712	1,580544658	0,507347077	0,611911313	NA
Kpna4	824,037847	-0,116098223	0,140408569	-0,826859953	0,408316444	0,746898413
Dgcr6	977,3675256	0,294904775	0,089537257	3,293654342	0,00098894	0,023507922
Slbp	259,9262072	-0,160552298	0,157780083	-1,017570119	0,308882294	0,66769994
Cstb	689,5071304	0,069909481	0,108083509	0,646809871	0,517755003	0,813424627
Hivep1	373,1083073	-0,265297538	0,156904489	-1,690821841	0,090870834	0,374146038
Zfhx4	417,2394276	-0,011411349	0,198303759	-0,057544796	0,954111223	0,986581557
Fcrls	0,347232576	0,667197765	3,168193746	0,210592476	0,833205284	NA
Trim2	927,8617448	-0,112825137	0,150845452	-0,747951863	0,454489193	0,776593306
Mdh1b	0,482702416	-0,058142556	2,481362332	-0,023431707	0,981305913	NA
Plvap	1207,52638	-0,009833675	0,131786974	-0,074617959	0,940518685	0,983128178
Smarcd2	1441,611339	-0,048546781	0,101685788	-0,477419527	0,633063412	0,867527474
Tas1r2	0,996330658	2,493469215	1,787190803	1,395189149	0,162958808	NA
Tas1r3	3,129891628	0,700195306	0,841233936	0,832343153	0,405215279	NA
Lamtor2	1232,1864	-0,019642266	0,08736629	-0,224826602	0,82211417	0,946779723
Slc2a2	4383,681107	0,290013412	0,268789767	1,078960015	0,280605553	0,641688878
Hspa8	25268,78087	-0,786025033	0,166508696	-4,720624512	2,35E-06	0,00025978
Akr1c21	3,245884896	-0,476038842	0,930318641	-0,511694403	0,608864904	NA
Mrap	2370,017009	0,462871359	0,157348119	2,941702522	0,003264133	0,052323978
Atf5	9115,043323	0,391824237	0,103425062	3,788484435	0,000151569	0,006103395
Snf8	772,4590205	0,085755509	0,110125098	0,77870995	0,436150593	0,76575019
Snapc3	65,36075667	-0,132324172	0,27708613	-0,477556103	0,632966181	0,867527474
Maged2	137,6885907	0,178237372	0,154929535	1,150441533	0,249962062	0,60896459
Rai14	718,561131	-0,27763095	0,119974891	-2,314075453	0,020663584	0,160578725
Srxn1	1252,164789	0,178453078	0,085076164	2,097568448	0,035943285	0,221906144
Wdr38	0,122496332	0,780932884	3,352475198	0,232942181	0,815806304	NA
Slco1a4	1452,48799	0,839663693	0,373363788	2,248915725	0,024517858	0,178140896
Emc9	537,84649	0,027938347	0,142160997	0,196526105	0,844198403	0,953068489
Siglece	108,2031161	0,139269595	0,210114826	0,662826118	0,507441924	0,806849558
Gtpbp3	277,7206687	-0,157223557	0,13400038	-1,173306803	0,240672795	0,59816966
Krt8	6291,707323	-0,104569052	0,129027617	-0,8104393	0,417687738	0,752229411
Cluap1	161,0740553	-0,086314083	0,129669789	-0,665645277	0,505637859	0,805801947
Nckipsd	117,2671174	-0,181514927	0,168267959	-1,078725433	0,280710144	0,641688878
Serp1	6863,405674	-0,49467071	0,154936273	-3,192736613	0,001409314	0,02995409
1700113H08Rik	0,086476712	0,780932884	3,352475198	0,232942181	0,815806304	NA
Trim34a	179,0823774	0,378100609	0,173826788	2,175157311	0,02961833	0,198245973
Cadm4	182,7661252	-0,168986688	0,327263546	-0,516362699	0,605601113	0,856421285
Zfp418	36,71241278	-0,149676687	0,251132374	-0,596007136	0,55117046	0,830401734
Cnot3	555,6070868	-0,111971605	0,093780397	-1,193976662	0,232487097	0,589440547
Zfp282	107,7656467	0,068439978	0,19366562	0,353392504	0,723794205	0,910252319
Tcaf2	12,37948879	0,348684683	0,441350844	0,790039688	0,429504591	0,760857915
Epop	74,65555586	-0,092820261	0,252729553	-0,367271101	0,713416808	0,905933635
Erfe	0,427322268	0,014665161	3,020716141	0,004854862	0,996126395	NA
Ltbp4	866,2412442	-0,304532646	0,214783272	-1,417860166	0,156231594	0,491473123
Atp13a5	0,395521434	-1,146297358	3,056659237	-0,375016405	0,707648266	NA
A730017L22Rik	139,9102204	-0,04029754	0,14532955	-0,277283869	0,781562145	0,932196631
Tmem169	9,229546955	-0,499121168	0,550149217	-0,907246893	0,364276251	0,713048566
E430018J23Rik	86,24557595	0,161331284	0,183524734	0,879071068	0,379362743	0,725074694
Dnah17	4,347358994	2,046757344	1,010893193	2,024701874	0,042897986	NA
Cblc	367,7853604	-0,041595919	0,179591579	-0,231613975	0,816837852	0,944998904
Ncstn	1252,064145	-0,091938886	0,06869968	-1,338272393	0,180807658	0,524412763
Mchr1	0,180324769	-1,201310802	3,342952034	-0,359356278	0,719328578	NA
Tnip2	117,668289	0,148432245	0,16512987	0,898881861	0,368715591	0,716901482
Mpst	2896,575446	0,139829847	0,144271065	0,96921615	0,332437355	0,689688857
Eif4a3	1093,10969	-0,071620351	0,09138378	-0,783731546	0,433197648	0,763032854

Supplementary Table S1: *Serpina1* KO vs. wildtype all DEGs

Ufsp2	1364,200018	-0,0914006	0,105281929	-0,868150892	0,385311734	0,729677128
B3gnt8	5,718104088	-0,268723356	0,598159853	-0,449250071	0,653251271	0,877997875
Rif1	259,7087075	-0,032071584	0,136858899	-0,234340508	0,81472064	0,944076707
Utp20	389,286398	-0,335984186	0,118422828	-2,837157252	0,004551719	0,064461789
Rhoj	77,96532377	-0,140160253	0,273939402	-0,511646927	0,608898136	0,857753956
Rce1	215,4595473	-0,090299703	0,134164261	-0,673053333	0,500913362	0,803541005
Cacfd1	587,3021203	-0,441300432	0,104220774	-4,234284746	2,29E-05	0,001532624
Ccdc183	1,604152583	-1,47381974	1,341156438	-1,098917097	0,271804228	NA
Pak2	708,7269183	-0,106362831	0,103830726	-1,024386863	0,305652584	0,664636701
Efnb3	3,577187221	-0,962056825	0,904352542	-1,063807288	0,287416009	NA
Epha4	7,746850609	0,853463835	0,713542289	1,196094258	0,231659778	0,58867957
Piwil4	0,173498105	0,059593471	3,352475198	0,017775962	0,985817581	NA
Eno1b	3,298793089	-0,255325044	0,868432591	-0,29400675	0,768752745	NA
Vamp5	211,4678149	-0,058673916	0,168210602	-0,348812236	0,727230277	0,910689961
Camta2	652,5169921	-0,241905047	0,119693	-2,021045899	0,043275014	0,245353546
Hephl1	0,180974489	-1,204797453	3,342691436	-0,360427361	0,718527568	NA
Bend4	13,37660893	0,300425099	0,559860593	0,536606974	0,591539126	0,850578037
Thsd7a	7,422367217	0,336476444	0,703920078	0,478003759	0,632647529	0,867473315
Polr2m	1489,204612	-0,045840277	0,077164073	-0,594062438	0,552470352	0,830818463
Tpbg	0,390492215	-1,104026806	2,531900821	-0,436046624	0,662802902	NA
Faap20	294,6505464	0,026895088	0,123551025	0,217684056	0,827675288	0,947580409
Cops7a	1426,625995	-0,07860875	0,114704284	-0,685316597	0,493144161	0,798745371
Zkscan7	161,9541572	-0,200179269	0,192840514	-1,038056081	0,299243947	0,659904651
Tgif1	453,8637251	-0,14028433	0,191718487	-0,731720412	0,464339235	0,781663619
Tank	410,2095683	0,002559508	0,142746161	0,017930483	0,985694311	0,995899882
Sell	23,21154692	0,726077566	0,382061408	1,900421114	0,057377878	0,289356792
Nagk	309,7117671	-0,028443403	0,119563244	-0,237894203	0,811963152	0,942944131
Vpreb1	4,217810147	0,041443245	0,697938513	0,059379507	0,952649835	NA
Gm9733	0,522764216	-0,538804184	2,100325045	-0,256533714	0,797538751	NA
R3hcc1	263,0186744	0,18585071	0,112117389	1,657643936	0,097389356	0,388433358
Igsf9b	0,618953805	-1,089780511	1,806230927	-0,603345062	0,546279161	NA
Mup2	64,47242093	-2,573705787	0,80968707	-3,178642567	0,001479664	0,031103195
Rubcn	452,4953877	-0,133702629	0,118050294	-1,132590393	0,257386305	0,618226166
St8sia4	11,21299947	0,456318083	0,552293274	0,826224226	0,408676908	0,746898413
Fn3krp	241,5778663	0,065254412	0,170211212	0,383373169	0,701443094	0,900047291
Gtf2h3	321,1691687	-0,205790504	0,123626058	-1,66462077	0,095988439	0,385586553
Gltscr1	174,5490075	-0,126237211	0,16436354	-0,768036581	0,442465453	0,769135977
Aldh18a1	46,60418887	0,06379587	0,29157779	0,218795369	0,826809454	0,947537943
Erc2	188,7216952	0,088937594	0,134360747	0,661931371	0,508015205	0,807274415
Txndc9	1101,371763	-0,033980539	0,078700565	-0,431769955	0,665908618	0,885041079
Tmem18	307,1838383	0,05001218	0,133848446	0,373647816	0,708666354	0,903161152
Gja10	9,076906292	0,075582544	0,481458383	0,156986661	0,875255361	0,964021167
Obp2a	16,84783718	-1,343029675	0,762816032	-1,760620672	0,078302627	0,34686144
Gnrh1	5,003983133	-0,05055067	0,739986675	-0,068312946	0,945536519	NA
Pcsk9	2212,372139	0,15432141	0,158759453	0,972045485	0,331027921	0,687725295
Kcng3	0,086476712	0,780932884	3,352475198	0,232942181	0,815806304	NA
Dcstamp	0,147722973	0,780932884	3,352475198	0,232942181	0,815806304	NA
Rbm12	171,9504898	0,10395224	0,134368804	0,773633735	0,439147416	0,766717272
Manf	3906,809074	-0,871978359	0,171403685	-5,087278952	3,63E-07	5,85E-05
Snrnp25	50,74927167	0,218748909	0,242219026	0,903103743	0,366470837	0,714711689
Trmt10c	431,7154887	0,125479338	0,181327391	0,692004322	0,488934615	0,796400974
Pagr1a	434,7660351	-0,288808257	0,13828219	-2,088542695	0,036748908	0,224873137
Glt8d2	1,282392233	1,003542166	1,412456085	0,71049442	0,47739759	NA
Tmem215	1,04427567	-0,582164411	1,450033118	-0,401483527	0,688064164	NA
Heatr5a	405,5527887	-0,489193905	0,140904956	-3,471800553	0,00051698	0,014959977
Ppm1e	2,845598723	0,393577403	0,872675338	0,451000946	0,651988867	NA
P4ha3	1,428025796	0,317452842	1,164040303	0,272716367	0,785071253	NA
Rinl	39,53645398	0,218844039	0,294603413	0,74284285	0,45757683	0,778822589
Gchfr	2078,099392	0,274825458	0,06710698	4,095333435	4,22E-05	0,002348272
Klri2	1,835003625	-0,974044845	1,358562125	-0,716967466	0,473394171	NA
Lrig3	199,8284618	-0,166491295	0,159144628	-1,046163464	0,295485568	0,656530922
Ctnna2	0,407104383	1,08374679	2,548423831	0,425261598	0,670645985	NA
Olfr1029	0,520185999	1,487180202	2,384076407	0,623797206	0,532760775	NA
Olfr1033	135,4062241	-0,053921604	0,206100146	-0,26162817	0,793608124	0,936746676
Olfr1034	29,70420017	0,139227386	0,292850806	0,475420874	0,634487019	0,868303507
Scpep1	1273,442542	0,316971122	0,111903963	2,832528124	0,00461815	0,065195409

Supplementary Table S1: *Serpina1* KO vs. wildtype all DEGs

Prelid2	19,55185486	1,860655039	0,649394725	2,865214277	0,004167272	0,060821237
Cetn4	3,842967164	1,478513002	0,9211267	1,60511361	0,108468805	NA
Deptor	3796,38755	-0,132279104	0,098203298	-1,346992479	0,177982655	0,520240034
Olfr1251	0,086476712	0,780932884	3,352475198	0,232942181	0,815806304	NA
Wdr91	605,3635799	-0,455525167	0,132816373	-3,429736537	0,000604168	0,016827326
Kdm3b	655,7813028	0,048686576	0,110105351	0,442181741	0,658357698	0,881392356
3110043O21Rik	230,4136855	-0,193851936	0,145912188	-1,328552048	0,183995801	0,530366508
Nacad	0,536296325	1,556811705	1,886008351	0,825453241	0,409114319	NA
Scaper	127,7105786	0,270674722	0,228821799	1,182906187	0,236846329	0,59395959
Pepd	990,977309	-0,148499537	0,100821093	-1,472901471	0,140777606	0,466258507
Peg3	133,2389311	-0,490157642	0,206654972	-2,371864743	0,017698568	0,147330112
Urod	1893,641825	-0,154929266	0,116906528	-1,325240497	0,185091397	0,531690351
Padi2	9,237547379	0,206715317	0,525163126	0,393621157	0,693860759	0,896031076
Lats2	300,07457	-0,247509237	0,147092416	-1,682678445	0,092437357	0,37789723
Amot	175,8015254	-0,014150897	0,181619161	-0,077915218	0,937895494	0,982735705
Npb	12,55254008	0,504967809	0,567481573	0,889840009	0,3735518	0,720823634
Hbp1	2137,316515	-0,011583852	0,08398348	-0,137930131	0,890295637	0,968877905
Clec4a3	65,66490011	0,429147828	0,26623464	1,611915821	0,106980259	0,405966245
Rbks	285,5803039	0,116353438	0,127349415	0,913655067	0,360898122	0,710581841
Hsd3b2	1160,229817	-0,625704435	0,245263805	-2,551148695	0,010736851	0,109908849
Pkhd1	223,3197849	-0,00253204	0,187935908	-0,013472892	0,989250512	0,996575327
Ago2	171,8415882	-0,474848963	0,223960372	-2,120236536	0,033986103	0,215643192
Adcy2	4,183866392	0,962434672	0,703899354	1,367290175	0,17153437	NA
Il10	1,414056238	1,357945245	1,399761013	0,970126495	0,331983444	NA
Il18rap	5,099651369	0,403580232	0,732636971	0,550859768	0,581729809	NA
Hal	12733,17186	0,214797047	0,1723114	1,246563185	0,212557708	0,565509253
Snord98	0,426232903	2,19919185	2,931845427	0,750104978	0,453191482	NA
Tst	6379,751722	0,153330557	0,093957076	1,631921338	0,102696058	0,398985659
Hmox1	544,6569393	-0,089968544	0,258383433	-0,348197804	0,727691637	0,910785759
Ttc3	485,7620471	-0,191139486	0,097776217	-1,954866848	0,050598788	0,270337998
Tctex1d2	30,9840391	0,850897483	0,281086878	3,027169003	0,002468559	0,043423902
Hmga2	1,682753149	-1,844832138	1,371509147	-1,345111072	0,178589368	NA
Psmd3	2839,143156	0,157393313	0,07783458	2,022151489	0,043160704	0,245017178
Ntn3	156,0843199	-0,235927657	0,180856712	-1,304500418	0,192063017	0,541552905
Rpl13a	7638,39335	0,041474737	0,156728656	0,264627656	0,791296313	0,936263422
0610039H22Rik	0,12663974	0,780932884	3,352475198	0,232942181	0,815806304	NA
Cd200	18,31291447	0,837902462	0,413470512	2,026510812	0,042712466	0,243203202
Sirt4	242,8867703	0,245858676	0,153880878	1,597720778	0,110105132	0,410672697
Slc37a2	48,90290651	-0,138086618	0,230261617	-0,599694467	0,548709877	0,828986591
Egfem1	11,75144667	0,257812001	0,447232692	0,576460544	0,564303932	0,837355044
Kifc2	237,5822767	0,064411379	0,199321805	0,3231527	0,746579601	0,919391514
Ccnk	484,3408877	0,012258875	0,088821143	0,138017533	0,890226561	0,968877905
Atp5f1	7920,835054	0,0403872	0,068034236	0,593630536	0,552759252	0,83087907
Orc1	0,949426855	-0,71935713	1,601674767	-0,449128091	0,653339258	NA
Ap4s1	195,6036672	0,125742988	0,161329984	0,779414865	0,43573537	0,765414075
Rhbg	280,6948337	0,875327127	0,255920793	3,420304833	0,00062551	0,01716174
Tusc1	34,9568619	0,116603393	0,286781296	0,406593437	0,684306618	0,892349329
Mir7020	0,093953095	-0,517475177	3,352475198	-0,154356154	0,877328924	NA
Art2a-ps	1,082469549	2,649387496	1,632098072	1,623301652	0,104524937	NA
Clec5a	2,270598294	1,275076856	1,08217822	1,178250341	0,238696819	NA
Net1	2235,07516	0,156208217	0,094798626	1,64778988	0,099395796	0,392688228
Cd46	8,693226319	-0,470167286	0,588993245	-0,79825582	0,42472205	0,757334234
Col15a1	395,7651056	-0,058766115	0,414493879	-0,141778004	0,887255359	0,968356501
Kbtbd6	0,864583394	-3,334470083	1,690483894	-1,972494441	0,04855319	NA
Cend1	0,980030116	0,332643075	1,490033927	0,223245303	0,823344595	NA
Amotl2	616,6611167	-0,320319955	0,112335434	-2,851459632	0,004351901	0,062092387
Mir7008	0,086476712	0,780932884	3,352475198	0,232942181	0,815806304	NA
Naa10	944,9026642	0,034893496	0,093185601	0,374451581	0,708068374	0,902743285
Ehd3	1461,455583	-0,130287966	0,26234706	-0,496624458	0,619453903	0,861846105
Skiv2l2	600,2645114	-0,223213222	0,084477365	-2,642284388	0,008234888	0,093298488
Fgfbp1	1,215039109	1,998356612	1,711713172	1,167459972	0,243024664	NA
Btg2	388,2009348	-0,452116062	0,291842245	-1,549179633	0,121338544	0,433611562
Btg1	1509,848691	0,238310509	0,202034659	1,179552607	0,238178202	0,595289658
Edn3	0,334254984	0,663952394	2,675156878	0,248191947	0,803985897	NA
Capns1	2888,84766	-0,047692939	0,091550591	-0,520946271	0,602404201	0,854608409
Id1	116,9540041	0,301182248	0,602285506	0,500065576	0,617028904	0,860830256

Supplementary Table S1: *Serpina1* KO vs. wildtype all DEGs

Man1a2	787,5344286	-0,063430021	0,114025533	-0,556279099	0,578020073	0,845288649
Icam2	59,21442363	-0,555956263	0,278799125	-1,994110498	0,04613998	0,255211169
Vdr	0,667210526	0,911289972	2,305002006	0,395353223	0,692582226	NA
Gpx3	147,9745427	0,327157445	0,301365341	1,085584176	0,277663004	0,638252527
Il1rl1	0,22911131	0,059593471	3,352475198	0,017775962	0,985817581	NA
Vcl	1324,565149	-0,366159481	0,149750902	-2,445123712	0,014480246	0,13080163
Ica1	39,91291562	-0,721252423	0,342131496	-2,108114664	0,035021072	0,218499774
Pld3	627,8107434	-0,037284971	0,095517762	-0,390345938	0,696280758	0,897385953
Ifit3	399,3045614	0,466521583	0,255791166	1,823837746	0,068176605	0,319471798
Jun	418,82188	-0,597109611	0,29109549	-2,051249957	0,040242611	0,236119007
Eif6	1132,306003	-0,057496784	0,082265976	-0,698913289	0,484606222	0,79420977
Ola1	1163,709062	-0,060027456	0,116099676	-0,517033795	0,605132568	0,856358347
Elavl3	0,180429807	0,059593471	3,352475198	0,017775962	0,985817581	NA
Maz	1392,18244	-0,165890348	0,096659983	-1,716225709	0,086120739	0,363775092
Wdyhv1	153,7626366	0,210564286	0,152813776	1,377914289	0,168229753	0,507127649
Tmcc2	54,64203223	-0,393392538	0,370614544	-1,061460064	0,288480873	0,649625922
Gtpbp2	676,0227247	-0,028732878	0,136735677	-0,210134464	0,833562728	0,950601391
Uqcc1	942,7335111	-0,208681273	0,078648753	-2,653332243	0,007970138	0,091618892
Camkk1	9,766844774	-0,233730698	0,497539955	-0,46977272	0,638517407	0,870699634
Rps3a1	6499,469613	-0,03392658	0,118559238	-0,286157207	0,774757718	0,929351416
Cacna2d3	0,227324296	1,389394708	3,349408814	0,414817893	0,67827522	NA
Terf2ip	374,6446077	0,055511211	0,103881502	0,534370509	0,593085219	0,851477265
Ptprcap	19,02844882	0,429409527	0,357726241	1,200385874	0,229989512	0,586221123
Pkd2l2	4,645163946	-1,169995905	0,872136089	-1,341529056	0,179748742	NA
Cldn8	5,254360927	0,121579327	0,774959689	0,156884711	0,87533571	NA
Fanca	14,85812547	-0,801972704	0,427931622	-1,874067405	0,060921135	0,298973723
Defb25	0,920903636	1,597615343	2,251437929	0,709597774	0,4779536	NA
E2f8	175,2348749	-1,163269822	0,503967318	-2,30822472	0,020986641	0,161926817
Tnk2	675,3541365	-0,463385833	0,12418029	-3,731557003	0,0001903	0,007282032
Hnrnpul2	2325,088494	-0,09722229	0,075797112	-1,282664843	0,199609522	0,549954396
Tbrg4	863,5361248	0,039716015	0,095037908	0,417896565	0,676022744	0,88927955
Kctd15	180,1720598	-0,138429314	0,186929443	-0,740543126	0,458970503	0,779963984
Wdr62	72,22492562	-0,612065359	0,188322309	-3,250094811	0,001153665	0,026364705
Galr3	0,65957343	0,889518276	2,033597033	0,437411277	0,661813107	NA
Wbp4	406,1392898	-0,080382883	0,104920532	-0,766131106	0,443598296	0,770142004
Fut8	126,6136018	-0,174427734	0,156509852	-1,114484055	0,265071581	0,626006345
Pcdh12	102,7147091	-0,256260248	0,292649211	-0,875656718	0,381216681	0,726305517
Ostf1	888,3510693	0,033218696	0,100834811	0,329436781	0,741825571	0,916899135
Klrg1	0,259974817	0,586045239	3,35071904	0,174901337	0,86115716	NA
Mbd3	1150,417054	-0,189706324	0,113207905	-1,675733902	0,093790326	0,380194927
Mum1l1	2,520928302	-0,33239573	0,955441592	-0,347897488	0,727917172	NA
Col6a6	5,96907285	-0,352230312	0,643585329	-0,54729388	0,584176847	0,847068435
Zbtb38	196,2429161	-0,45472037	0,213841779	-2,126433721	0,033467162	0,213835285
Arhgap20	0,361404296	-1,127497653	2,591173255	-0,435130168	0,663467947	NA
Mrgpre	27,02760114	-0,175215247	0,370536551	-0,472868996	0,636306645	0,869590981
Nlrp10	2,5844052	-2,076116583	0,975381923	-2,128516568	0,033294278	NA
Slfn8	52,70446583	0,275199573	0,227633302	1,208960074	0,226678185	0,581885686
Chchd6	70,58951965	0,11833706	0,183731371	0,644076507	0,519525824	0,813866972
Itgb7	36,03713087	0,104248898	0,269083838	0,387421626	0,698444096	0,898800587
Olfr1396	0,955153344	-0,882057687	1,69947332	-0,519018261	0,603748007	NA
Wdr78	17,30214184	-0,035779793	0,403933354	-0,088578457	0,929416929	0,979997334
Gpr180	553,286734	-0,001651715	0,097925335	-0,016867091	0,986542647	0,99608667
Slc15a3	145,6303901	0,437556031	0,276105603	1,584741584	0,11302506	0,41624842
Rilpl1	63,93312894	-0,312935339	0,233459669	-1,340425699	0,180106985	0,52303526
Dexi	590,8614417	-0,228765835	0,14056399	-1,627485358	0,103634056	0,400776307
Nectin1	2016,410973	-0,109954497	0,137079071	-0,802124611	0,42248088	0,755522295
Ptpre	37,5642529	-0,080028773	0,360090808	-0,222246088	0,824122318	0,947472731
Dnaja4	163,7123508	-0,376064379	0,232495682	-1,617511239	0,105767972	0,403312041
Bhmt2	6434,585467	0,206760144	0,10614939	1,947822266	0,051436234	0,273311211
Lgi1	0,227324296	1,389394708	3,349408814	0,414817893	0,67827522	NA
Htra1	53,37641282	-0,871169778	0,353794903	-2,462358194	0,013802674	0,1275245
Smpdl3a	988,3833959	-0,028180751	0,092894882	-0,30336172	0,761614215	0,92534041
Ensa	902,9472478	0,087572773	0,11453592	0,764587853	0,444517005	0,770550383
Alas1	4720,110112	-0,086801789	0,464870454	-0,186722534	0,851878187	0,95595696
Mrps31	840,5855966	-0,144430006	0,088365877	-1,634454511	0,10216345	0,398083115
C1qbp	2392,484585	0,105454505	0,098328235	1,072474301	0,283507051	0,644784493

Supplementary Table S1: *Serpina1* KO vs. wildtype all DEGs

Amacr	3614,902446	-0,24411776	0,069811974	-3,496789273	0,000470894	0,013989727
Btc	57,48682505	0,737046931	0,213113743	3,458467387	0,000543258	0,015452802
Bsn	0,701825869	2,899631146	1,931090638	1,501551035	0,133213097	NA
Dcun1d5	472,5955243	-0,013459189	0,089627213	-0,150168551	0,880631637	0,96546599
Ttll11	2,716359421	-1,270293144	0,884713226	-1,435824747	0,151052216	NA
Ide	3856,911812	0,620649376	0,166198217	3,734392519	0,000188169	0,007249651
Tex15	0,313801008	1,795331527	3,226882632	0,55636716	0,577959884	NA
Cenpl	132,3995683	0,300183765	0,190085937	1,579200278	0,114290126	0,418500328
Polr3f	243,0570205	0,059479165	0,124319907	0,478436371	0,632339652	0,867306747
Hyls1	27,70372268	0,579214908	0,318862226	1,816505252	0,069292903	0,321819913
Mir1956	0,538215188	-0,521352449	2,08877461	-0,249597274	0,802898807	NA
Upf3b	394,0214753	-0,155960125	0,111388791	-1,400142004	0,161470799	0,497950692
Cldn15	8,957771015	1,798193227	0,560533208	3,208004809	0,001336593	0,028782722
Wbp11	850,4535007	-0,042899434	0,074827304	-0,573312573	0,566433074	0,838145408
Myoc	1,35255214	1,03701618	1,719177467	0,603204847	0,546372424	NA
Cd200r1	14,14250922	0,144988088	0,474791876	0,30537188	0,760082936	0,924925298
Hist1h1d	3,85567176	-0,178006063	0,920281093	-0,193425753	0,846625548	NA
Wdr4	240,6423333	0,109718048	0,149314577	0,734811363	0,462454386	0,781516914
Rps14	3949,545307	-0,037839431	0,127031006	-0,297875554	0,765798146	0,926898815
Ripk3	36,20620723	0,420851953	0,310057752	1,357334077	0,174675084	0,517225036
Clip2	75,64847794	-0,236511527	0,178932949	-1,321788574	0,186238564	0,532805747
Rnft2	3,315434135	-0,062831564	0,811897927	-0,077388502	0,938314488	NA
Ero1l	388,6729595	-0,488683121	0,159524959	-3,063364662	0,002188632	0,040526064
Slc35b1	1148,67565	-0,455687388	0,143586911	-3,173599768	0,001505611	0,031474133
Acvr1c	1,336890654	1,604016333	1,337167604	1,199562663	0,230309234	NA
Cryba4	2,943003178	-0,169395053	0,813800976	-0,208152924	0,835109564	NA
Spem1	0,086476712	0,780932884	3,352475198	0,232942181	0,815806304	NA
Katnb1	74,54047307	-0,064901623	0,227399124	-0,285408413	0,775331263	0,929570059
1110034G24Rik	82,68764632	0,163889976	0,18965552	0,864145561	0,387507933	0,731825619
Tmem110	269,5530178	0,031169922	0,18596732	0,167609676	0,866890359	0,961775826
Armc1	789,0509045	-0,202020209	0,133159915	-1,517124791	0,12923518	0,446717571
Pomk	191,3354954	0,366548592	0,14447165	2,537166233	0,011175387	0,113013431
S100pbp	371,5192645	0,06602083	0,120973945	0,545744212	0,585241776	0,847482024
Ccser2	961,4745809	-0,237942945	0,113647627	-2,093690395	0,036287569	0,223252881
Mterf2	57,18548702	1,243926208	0,330014331	3,769309666	0,0001637	0,006532839
Paqr8	6,831929187	-0,685734484	0,526504948	-1,302427427	0,192770312	0,541552905
Scrn3	1254,837725	-0,238890836	0,101890994	-2,34457263	0,0190489	0,153783058
Rab3a	104,1968564	-0,461758821	0,203777601	-2,265994005	0,02345175	0,173219033
Klhl12	307,1491356	-0,114542828	0,123350633	-0,928595379	0,353098818	0,70414351
Adm	38,10030476	0,1761348	0,344827631	0,510790855	0,609497513	0,857753956
Kif7	13,57225623	-0,110678276	0,456223595	-0,242596564	0,808317944	0,942309259
Adh7	66,8994425	0,159176009	0,316704714	0,502600694	0,615245041	0,859955845
Brca1	46,12930057	-0,170164162	0,375135615	-0,453607056	0,650111674	0,876831587
Krt10	38,68548384	-0,059637816	0,294265916	-0,202666408	0,83939578	0,951377482
Adcy1	22,79517787	0,647321717	0,408776938	1,583557333	0,11329449	0,416795277
Rrp1	1768,971106	-0,051048608	0,093402516	-0,546544249	0,58469188	0,847160046
Nnat	2,048430491	1,078921313	1,077084889	1,001704994	0,316486094	NA
Ccl12	0,784578551	-1,382294371	1,584284294	-0,872503992	0,382933491	NA
Ccl11	0,434883226	1,149382856	2,19554927	0,523505836	0,600622291	NA
Ctsd	9838,313634	0,168344171	0,094795583	1,775865133	0,075755149	0,340541556
Plat	12,70889705	0,637569265	0,561202449	1,136077125	0,255924288	0,616366745
Pigr	41631,48335	0,364096055	0,148183479	2,457062412	0,014007835	0,128796935
Pcyt1a	1991,851253	0,017636791	0,104973637	0,168011622	0,866574136	0,961775826
Syk	122,3528576	0,275470171	0,237576096	1,159502893	0,246251258	0,605518092
Stxbp3	603,4299177	0,068224976	0,100178211	0,681036079	0,495848662	0,80006558
Phkg1	1,111911668	1,044294571	1,394500309	0,748866504	0,453937671	NA
Phf2	552,0692371	-0,263191771	0,110910014	-2,373020803	0,01764327	0,146961295
Galk1	914,0427345	0,011026674	0,14696118	0,075031203	0,940189885	0,983128178
Cks1b	128,4567066	-0,872504107	0,217190926	-4,017221725	5,89E-05	0,003069981
Esd	5048,281355	0,068525998	0,078689838	0,870836692	0,383843328	0,72829995
Ppp2r1a	1624,203267	0,083833761	0,073053791	1,147562088	0,251149388	0,610767125
Nek5	4,50735326	1,887884114	0,801279219	2,356087703	0,018468557	NA
Ltv1	463,852783	0,079543	0,113147813	0,703000769	0,48205527	0,793477208
Fam89b	279,1472439	0,213453017	0,129913058	1,64304513	0,100373596	0,393294268
Ntrk3	2,580182024	0,07260135	1,148139432	0,063233914	0,949580239	NA
Supv3l1	480,9872779	-0,043919552	0,108974752	-0,403025025	0,686929815	0,89330962

Supplementary Table S1: *Serpina1* KO vs. wildtype all DEGs

Gm15800	579,250868	-0,300627947	0,129762324	-2,316758344	0,020516899	0,159842907
Zfp599	7,363833637	-0,773791712	0,548328822	-1,411181907	0,158190984	0,493856377
Ushbp1	234,1574539	-0,092953569	0,230377057	-0,403484486	0,686591846	0,893161227
Arhgap11a	31,88464388	-0,55051883	0,32574881	-1,690010254	0,091025993	0,374323747
Atrnl1	603,8123839	-0,273846391	0,122891716	-2,22835517	0,02585684	0,184362717
Pkn1	414,3111586	0,122711774	0,148503933	0,826320029	0,408622575	0,746898413
Xylb	3873,407581	0,017588887	0,062865193	0,279787367	0,779640644	0,931541315
AW551984	1,685871734	1,997513659	1,616371309	1,235801234	0,216532456	NA
Ei24	5200,943693	0,152419094	0,097240254	1,567448533	0,117009887	0,424373183
4931406C07Rik	7332,122307	-0,233472791	0,152574507	-1,530221496	0,125961912	0,441736801
Sik1	626,6445014	-0,327142458	0,395739444	-0,826661237	0,408429098	0,746898413
Incenp	51,15508073	-0,44723553	0,261667866	-1,709172534	0,087418995	0,366594901
Nes	66,64949733	-0,872892999	0,300241034	-2,907307467	0,003645547	0,056318411
Hnrnpdl	957,1768942	0,243299397	0,104050329	2,338285704	0,019372432	0,155304678
Sh3gl2	0,997226899	2,560492566	1,721547979	1,487319899	0,136930341	NA
Cdk2	234,9573726	-0,011520275	0,136281587	-0,084532882	0,932632761	0,981243003
Sfrp4	0,379918946	1,000946436	2,589354372	0,386562167	0,699080371	NA
Vezf1	346,7171257	-0,060812324	0,126766262	-0,479720099	0,631426434	0,86707135
Comp	0,555424572	1,658832023	2,385279254	0,695445625	0,486776076	NA
Slc23a2	352,2391733	0,132431511	0,161788271	0,818548276	0,413044188	0,749760726
Bbc3	352,83006	-0,095573584	0,167844524	-0,569417345	0,569072948	0,840790976
Zfp87	114,4453359	-0,004330265	0,146821786	-0,029493342	0,976471129	0,993640015
Nup155	435,958562	-0,13541172	0,130593016	-1,036898629	0,299783103	0,659937478
Pdzd3	0,721566537	0,674153069	2,030003433	0,332094546	0,739817868	NA
Acbd3	436,9033413	-0,197029837	0,127458591	-1,545834105	0,122144638	0,434047185
Atp13a1	1077,797261	-0,145754139	0,09474905	-1,538317677	0,12397096	0,437646623
Dclre1b	92,60176439	0,018339553	0,193977262	0,094544859	0,92467635	0,97800618
Mfsd5	428,4471995	-0,068456078	0,099514628	-0,687899651	0,491515976	0,797760449
Zfp704	152,5681219	0,384729767	0,19542672	1,968665124	0,048991559	0,264954753
Xpnpep1	834,650141	-0,00012261	0,104235765	-0,001176277	0,999061467	0,999803624
Igsf8	227,0720088	0,103064371	0,159280506	0,647062053	0,517591784	0,813424627
Farp1	510,7769435	-0,235004111	0,14133398	-1,662757324	0,096361022	0,385838157
Smco4	143,5747591	0,094031948	0,176696333	0,532166949	0,594610372	0,852263143
Bmp5	219,6124216	0,027897775	0,20645768	0,135125878	0,89251235	0,969104575
Fam131a	11,5915605	-0,01961168	0,47889297	-0,040952116	0,96733407	0,991131912
Gpn1	329,3245474	-0,135673057	0,127090311	-1,067532655	0,285731376	0,647228527
Ppargc1b	55,56271633	-0,347975012	0,310656274	-1,120128714	0,262658916	0,622604087
Pilrb1	58,873161	0,195770678	0,266259335	0,735263151	0,462179245	0,781516914
Pygl	15945,36831	0,034078582	0,178418522	0,191003612	0,848522763	0,954095425
Ptpn14	35,36369857	0,334167665	0,264527937	1,263260387	0,206495617	0,558880735
Kcmf1	1402,327051	0,060982164	0,093179086	0,654461925	0,512814249	0,810518464
Plcg1	738,1468043	-0,151882906	0,138196065	-1,099039299	0,271750925	0,633595764
Ykt6	1375,624116	-0,24104206	0,100748213	-2,392519455	0,016733141	0,143473504
Pcbp4	279,5640753	0,053411205	0,258926072	0,206279749	0,836572396	0,951141594
Drg2	920,9021382	-0,088620958	0,096919742	-0,914374675	0,360520004	0,710464613
Rassf3	1568,792349	-0,323182393	0,141125263	-2,290039258	0,022019041	0,165937181
Abcg4	0,53897958	0,519359315	2,133903132	0,243384673	0,807707416	NA
4833439L19Rik	1637,890067	-0,26000138	0,104460931	-2,488982021	0,012810944	0,121731107
Spaca1	0,609240927	-0,059305579	1,843125989	-0,032176628	0,974331195	NA
Bambi-ps1	0,927185343	0,893180495	1,628523564	0,548460283	0,583375893	NA
Mast2	482,6356035	-0,258798433	0,137260008	-1,885461307	0,059367584	0,294723185
Rbm19	287,8028183	-0,104189254	0,115084668	-0,905326973	0,365292191	0,713662326
4931402G19Rik	0,093953095	-0,517475177	3,352475198	-0,154356154	0,877328924	NA
Dap3	879,9536735	0,259247647	0,088196255	2,939440546	0,003288053	0,052455832
Pglyrp2	3825,989848	-0,032955486	0,116853315	-0,282024396	0,777924804	0,930822224
Fhl5	0,087021394	-0,517475177	3,352475198	-0,154356154	0,877328924	NA
Pes1	772,3249033	-0,023069471	0,10213017	-0,225883019	0,821292405	0,946779723
Gmfb	632,0672392	-0,126824686	0,137446425	-0,92272088	0,356152682	0,706230378
Pole3	293,8213109	-0,299984591	0,103264409	-2,905014369	0,003672362	0,056484846
Zfp111	105,1595932	0,134118405	0,183442837	0,731118246	0,46470693	0,781663619
Sh2d2a	11,60415195	0,147551789	0,485018476	0,304218904	0,760961123	0,92534041
Adamdec1	54,13603254	0,467983592	0,282010405	1,659455053	0,097024131	0,387479132
Zfp112	19,89292886	0,187686861	0,334792315	0,560606839	0,575065589	0,84359368
Grpel2	848,1076023	0,089887189	0,127688762	0,703955361	0,481460573	0,793276609
Rtp4	390,4088245	0,375566236	0,160779751	2,335905079	0,019496189	0,155528431
Cmya5	0,207615244	0,059593471	3,352475198	0,017775962	0,985817581	NA

Supplementary Table S1: *Serpina1* KO vs. wildtype all DEGs

Sec61a2	105,5225272	-0,205449097	0,166553885	-1,233529302	0,217378351	0,571173581
Abhd8	159,6795934	-0,811669813	0,163523435	-4,963629912	6,92E-07	9,74E-05
Mllt1	573,8103857	-0,269492823	0,109102192	-2,470095411	0,013507702	0,1257552
Fn3k	340,4584938	0,079910072	0,153840582	0,519434281	0,603457931	0,855346594
Noc2l	954,5547646	0,010299205	0,094982778	0,108432336	0,913652752	0,976232707
Mrps30	581,3437455	0,078241309	0,107892054	0,725181385	0,468340761	0,783439394
Hgh1	94,72095645	0,095540149	0,217333117	0,439602351	0,660225138	0,882607568
Pbk	9,197417226	-1,613167171	0,627474135	-2,570890307	0,010143745	0,10561661
Slc22a17	21,84766586	0,593122831	0,329748517	1,798712657	0,072064146	0,329488907
C1galt1c1	562,0935626	-0,263651768	0,152470627	-1,729197111	0,083773823	0,358161894
Stard3	377,5886606	-0,117834203	0,10518291	-1,120278977	0,262594898	0,622562722
Ccnb1	5,79553989	-0,07095546	0,877146399	-0,08089352	0,935526633	0,982081594
Iars	1410,891411	-0,259123045	0,104445444	-2,480941575	0,013103585	0,123354086
Tomt	1,469018596	0,691241375	1,255882145	0,550403059	0,582042951	NA
Chka	2582,218514	0,461158944	0,228277077	2,020171931	0,043365557	0,245531991
Cyp4b1	252,0624356	-0,187810303	0,312279972	-0,601416419	0,547562665	0,828552821
Prkab1	678,5288335	0,173410738	0,134460731	1,28967571	0,197163276	0,546862208
Pcdh1	2018,157796	0,152736865	0,159356848	0,958458125	0,337831798	0,693194655
Lhfpl3	0,087021394	-0,517475177	3,352475198	-0,154356154	0,877328924	NA
Susd6	917,6923789	0,071287758	0,112874739	0,631565211	0,527671025	0,818474757
Pigh	131,3379228	0,349213155	0,154519597	2,25999266	0,023821706	0,174792751
Lrrc42	444,4963088	0,084705049	0,132073437	0,64134811	0,521296538	0,814845193
Slc45a2	1,277654568	2,079204854	1,494857105	1,390905423	0,164254108	NA
Sla2	5,899222644	1,7255611	1,040159782	1,658938492	0,097128187	0,38761395
Foxe1	0,087021394	-0,517475177	3,352475198	-0,154356154	0,877328924	NA
Adamtsl2	181,9087386	-0,142116966	0,283736619	-0,500876365	0,616458137	0,860513269
Trim35	65,29661081	0,05248331	0,320097764	0,163960253	0,869762434	0,961775826
Nol3	30,82222394	0,315476511	0,275604758	1,144670044	0,252345865	0,612043137
Mir3473g	0,519949634	0,58356965	2,899656513	0,201254751	0,840499382	NA
Ier5	175,280768	0,398638342	0,316646591	1,258937732	0,208052824	0,56023257
Nkx1-2	0,336157466	0,663936047	2,673207616	0,24836681	0,803850611	NA
Acod1	3,234726725	1,319911779	0,879753179	1,500320556	0,133531387	NA
Mybl2	7,729933754	0,313634646	0,519602025	0,603605512	0,546105947	0,828069865
Txndc5	5832,163659	-0,51457291	0,100674712	-5,111242929	3,20E-07	5,28E-05
Ormdl1	628,7383626	0,145013753	0,120457378	1,203859446	0,228643908	0,584390655
BC003331	956,3935696	-0,014352999	0,105681847	-0,135813289	0,891968886	0,969104575
Tapbpl	570,167196	0,085525381	0,105224409	0,812790323	0,416338284	0,751673636
Imp3	740,9226268	0,0340422	0,14194958	0,239818954	0,810470613	0,942435559
Btbd2	550,9658786	-0,009874953	0,121704926	-0,081138484	0,935331821	0,982050665
Idi1	1022,141667	0,720271889	0,239116467	3,012222031	0,002593428	0,044679824
Ubald1	371,6877353	0,123592019	0,143082059	0,863784181	0,387706459	0,731859161
Zbtb7c	57,32117213	-0,221769171	0,437941794	-0,506389601	0,612583173	0,858991445
Nsun2	2120,747195	-0,153977506	0,112164333	-1,372784928	0,169819218	0,509724545
Gphn	1851,632658	0,148846872	0,124413871	1,196384869	0,231546403	0,588618343
Gsg1	0,688887166	0,966252022	1,839868918	0,525174382	0,599461972	NA
Ids	276,6817272	-0,083342916	0,197029722	-0,422996667	0,672297693	0,888049388
Kif22	17,39256684	-0,646171177	0,39238397	-1,646782814	0,099602695	0,392914851
Sbk1	468,2805833	1,028276315	0,2858155	3,597692621	0,000321053	0,010571807
Tmem159	43,76213376	0,373372653	0,264520933	1,411505124	0,158095726	0,493849511
Thumpd1	487,7998148	0,098512816	0,126683834	0,777627367	0,436788722	0,76575019
Spon1	11,01217531	0,749540666	0,469619387	1,596059889	0,110475423	0,411062987
Hip1	116,5898231	0,145541444	0,247069977	0,589069727	0,5558145	0,833316592
Ranbp10	2073,759092	0,11016243	0,172738941	0,637739406	0,523643329	0,81568929
Pitpnc1	533,8752856	-0,184701607	0,182453746	-1,012320169	0,311384995	0,670895406
Cd3eap	126,7912018	0,184023877	0,227815996	0,807774168	0,419220598	0,753719022
Veph1	8,204077278	0,088594296	0,54336473	0,163047565	0,870480984	0,961775826
Mzf1	6,976470407	-0,403145158	0,521694963	-0,772760304	0,43966425	0,766945637
Lace1	333,4200135	-0,287786042	0,09624923	-2,990008775	0,002789694	0,0470419
L3mbtl2	484,1196868	-0,239674467	0,11898245	-2,014368236	0,043970887	0,247168435
Nup43	86,7433087	0,438799072	0,17912018	2,449746714	0,014295674	0,130101335
Ranbp6	304,9397853	-0,081917777	0,148515582	-0,551576983	0,58123821	0,845803031
Psca	0,142634598	-0,517475177	3,352475198	-0,154356154	0,877328924	NA
2210016L21Rik	221,4618755	0,105746154	0,136042317	0,77730339	0,436979796	0,76575019
4932416H05Rik	19,29180294	0,154051819	0,366562007	0,420261281	0,674294591	0,888627551
Stmnd1	0,520766531	1,495047622	2,421133206	0,617499119	0,536905562	NA
Tbc1d8	310,0369632	0,183025127	0,158654259	1,153609915	0,248660132	0,60747894

Supplementary Table S1: *Serpina1* KO vs. wildtype all DEGs

Arhgap31	129,9045646	-0,290349816	0,291949972	-0,994519076	0,319970223	0,677494232
Nap1l3	34,5445946	0,117427451	0,303655438	0,386712821	0,698968824	0,898942204
Zscan25	114,9228118	-0,037990363	0,233673883	-0,16257856	0,870850269	0,961784159
Snord37	1,39947492	-0,406885429	1,317138126	-0,308916294	0,757385201	NA
Pde2a	351,1039845	-0,056997764	0,288709663	-0,197422432	0,843496977	0,952988345
Osgepl1	464,2171737	0,153806327	0,092357446	1,665337592	0,095845423	0,385586553
Nox4	633,4335359	-0,599363218	0,584305062	-1,025771051	0,304999515	0,664202396
Icosl	22,11308941	0,992206887	0,421635324	2,353234729	0,018610882	0,151643025
Dkkl1	0,180324769	-1,201310802	3,342952034	-0,359356278	0,719328578	NA
Crim1	225,8147124	-0,156783761	0,170309999	-0,92057872	0,357270416	0,707149983
Ybx2	1,869654324	-0,22326074	1,405791737	-0,158814947	0,873814672	NA
Snx3	2251,586982	-0,162583211	0,064277667	-2,52938881	0,011426137	0,114223875
Srsf4	848,5670621	-0,050368477	0,08176274	-0,616032151	0,5378733	0,82460384
Mgat4a	24,45705445	0,770813121	0,334198851	2,306450541	0,021085471	0,162193129
Gpbar1	0,180324769	-1,201310802	3,342952034	-0,359356278	0,719328578	NA
C5ar2	30,57611073	0,105343922	0,300840542	0,350165311	0,726214638	0,910393262
Exph5	616,1603821	-0,31501496	0,178042401	-1,769325498	0,076839571	0,342765506
Hoxc5	0,453963022	1,234906816	2,502925914	0,493385285	0,621740377	NA
Mir7011	1,767457356	-0,050744528	1,212035924	-0,041867181	0,966604579	NA
Gm4841	14,79041931	0,090724746	0,542868292	0,167121101	0,867274764	0,961775826
Armc2	1,848737302	1,194713243	1,035968195	1,153233515	0,248814551	NA
Ednrb	174,0587862	-0,105583575	0,241424114	-0,437336493	0,661867333	0,882945528
Gtf2e2	226,5621385	-0,117625959	0,146869589	-0,800887101	0,423197008	0,755975521
Nme8	0,12663974	0,780932884	3,352475198	0,232942181	0,815806304	NA
Sgol2a	6,716774195	-1,81030998	0,623014654	-2,905726165	0,003664019	0,056433498
Mir17	0,093953095	-0,517475177	3,352475198	-0,154356154	0,877328924	NA
Kif26b	6,743948096	-0,136014389	0,668623037	-0,203424622	0,838803156	0,951141594
Neb	309,2923455	0,0188211	0,231422951	0,081327717	0,935181332	0,982050665
Clca3a1	113,152872	0,652593456	0,32500068	2,007975667	0,044645877	0,25015538
Blvra	281,8436889	0,281112893	0,141048743	1,993019485	0,046259313	0,255554034
Rab13	137,4432025	-0,285658725	0,146633945	-1,94810775	0,051402072	0,273238289
Nudt22	225,855537	-0,009986111	0,135123802	-0,073903422	0,941087233	0,983128178
Foxd3	0,227324296	1,389394708	3,349408814	0,414817893	0,67827522	NA
Smlr1	647,9812334	0,248301469	0,109827575	2,260829926	0,023769791	0,174507596
Cyfip2	48,58263225	0,458920223	0,246365956	1,862758275	0,062496283	0,303271436
Nsun4	457,9386131	-0,216110929	0,102878258	-2,100647254	0,035671943	0,220786205
Rpusd1	76,74435657	0,028615936	0,171317749	0,167034277	0,867343078	0,961775826
Acsl5	17354,59616	0,359804555	0,154647635	2,326608849	0,019986093	0,157934757
AU019990	0,648067417	0,36115699	2,105070593	0,171565263	0,863779319	NA
Xlr3a	149,785248	-1,173318984	0,64127061	-1,829678401	0,067298043	0,317581197
Mib2	508,9492491	-0,104220248	0,098097004	-1,062420289	0,288044927	0,64949783
Cycs	2247,1232	-0,088796451	0,142505215	-0,623110187	0,533212116	0,820973486
Lbp	2661,277558	0,330475811	0,152322105	2,169585377	0,03003827	0,200147949
Fgf7	0,22911131	0,059593471	3,352475198	0,017775962	0,985817581	NA
Abcb4	5094,162461	0,110683297	0,144017727	0,768539401	0,442166792	0,769003232
Acat2	2101,713327	0,6584474	0,173759968	3,789407931	0,000151007	0,006099122
Egr2	2,759067492	0,424938373	0,937734336	0,453154328	0,650437617	NA
Mir3968	1,786796576	1,552662578	1,839845513	0,843909212	0,39872014	NA
Stag1	574,5493108	-0,059383456	0,126702026	-0,468685926	0,639294148	0,871049783
Pla2g4d	0,113662148	0,780932884	3,352475198	0,232942181	0,815806304	NA
Ggh	319,8188371	-0,038027153	0,153481002	-0,247764563	0,804316574	0,940432769
Igf2r	890,7587768	-0,133651213	0,112964971	-1,183120856	0,236761253	0,59395959
Zic1	0,22911131	0,059593471	3,352475198	0,017775962	0,985817581	NA
Irx1	7,672344744	1,072975684	0,599577057	1,789554274	0,073525595	0,33388712
Rbm46	3,831817079	-0,160550672	0,747069427	-0,214907298	0,829839595	NA
Ccdc171	91,46145606	0,295370067	0,263131081	1,122520633	0,26164114	0,622343615
Sec31a	4080,207371	-0,388991541	0,097066747	-4,007464469	6,14E-05	0,003143717
1700019A02Rik	0,259974817	0,586045239	3,35071904	0,174901337	0,86115716	NA
Manbal	226,355177	0,156596146	0,120244941	1,302309644	0,192810556	0,541552905
Tom1l1	516,8475837	0,027681506	0,09913795	0,279222094	0,780074389	0,931606698
Ccdc124	808,8652098	-0,07710802	0,105807883	-0,728754963	0,466151565	0,781929771
Lzic	125,4689988	0,114901886	0,170766139	0,672861063	0,501035686	0,803640801
Kbtbd3	63,3212222	0,002672384	0,198154432	0,01348637	0,98923976	0,996575327
Pgm2l1	76,74828641	-0,611189853	0,218519675	-2,796955711	0,00515866	0,070015871
2200002J24Rik	0,640543484	0,96868348	2,057726341	0,470754279	0,637816218	NA
Ap4m1	144,2479563	0,073647935	0,152301347	0,483567191	0,628693064	0,865922214

Aspa	278,7049787	0,085200602	0,155492879	0,547938934	0,583733834	0,847057161
Rcan3	30,16464856	0,524593366	0,298944306	1,75481973	0,079290148	0,349223642
Eed	414,7368444	-0,006561814	0,107111466	-0,061261549	0,951150913	0,985972877
Fxyd6	9,016106748	0,468497182	0,495804127	0,944923925	0,344697716	0,697587612
Xpo7	1574,154896	-0,1360588	0,103113327	-1,319507417	0,186999532	0,533459501
Doc2g	40,72855558	-0,387961113	0,293865955	-1,320197546	0,186769071	0,533163472
Trappc4	416,2099899	0,021980688	0,109658183	0,200447313	0,84113076	0,951786586
Cenpk	20,49769238	-0,229151948	0,340277152	-0,673427372	0,50067544	0,803541005
Lrrc61	199,8545828	0,496067055	0,510992691	0,9707909	0,331652415	0,688274004
Atmin	192,3341278	-0,051530825	0,126984631	-0,405803635	0,684886888	0,892717658
Clec12a	139,2068527	0,391639753	0,267135627	1,466070843	0,142628988	0,470004176
Prdm4	208,2894371	0,13390182	0,141979541	0,943106447	0,345626458	0,698427993
Scai	80,09867907	-0,532589374	0,219661082	-2,42459597	0,015325432	0,135596096
BC049715	3,498070381	0,830065175	0,869044269	0,95514717	0,339503278	NA
Rps6kc1	278,3515947	-0,021300741	0,153716816	-0,138571312	0,889788914	0,968877905
Prr18	1,645978843	0,298065866	1,14900006	0,259413272	0,79531639	NA
Nceh1	532,6340667	-0,079418775	0,239022497	-0,332264854	0,739689276	0,916067243
Tmem72	0,79773776	2,187341907	1,972591981	1,108866876	0,267487615	NA
Agmo	1630,603863	-0,014213122	0,144943055	-0,098060042	0,921884616	0,977341679
Vmac	307,9661116	-0,064294733	0,12178019	-0,527957236	0,597529017	0,853549036
Fam168a	841,1791049	-0,160808506	0,120039076	-1,33963465	0,180364153	0,523510282
Zfp750	284,6910762	0,230167973	0,247196677	0,931112731	0,351795254	0,702880198
Zfp935	90,87144592	0,229945495	0,169545157	1,356249269	0,175019871	0,517287416
Irf2bp1	410,0970153	0,11551988	0,140972126	0,819451928	0,412528618	0,749435398
Rnf186	128,364304	-0,098212844	0,182134134	-0,539233597	0,589725681	0,84953676
Gcnt1	11,49051011	0,917104137	0,572304393	1,602476144	0,109050359	0,40910239
Ptpn3	913,4971231	-0,18958976	0,111019916	-1,70770946	0,087690266	0,367156644
Bmp3	0,494230815	1,421586776	2,402768743	0,591645276	0,554088149	NA
Sumo3	2431,201708	-0,040565298	0,060494689	-0,670559669	0,502501082	0,80422495
Rorc	2540,192753	-0,549848802	0,210869451	-2,607531812	0,00911976	0,099447036
Smarcc2	2662,597548	-0,122495321	0,098985127	-1,23751239	0,215896917	0,569610871
Irf7	1075,734818	0,248414385	0,107715964	2,30619842	0,021099548	0,162208082
Wdr61	1302,305521	0,08303403	0,069231469	1,199368313	0,230384763	0,586979539
Foxa1	545,1313027	-1,026172997	0,239914835	-4,277238619	1,89E-05	0,001333939
2810459M11Rik	1681,436331	-0,240831413	0,099804832	-2,413023574	0,015820796	0,137992723
Ctnnal1	95,48130219	0,101471836	0,19947238	0,508701185	0,610961691	0,858645358
Vti1b	1064,132946	0,037154245	0,098103767	0,378723938	0,704892876	0,901790704
Bnip2	908,9965006	0,002353303	0,086301508	0,027268385	0,978245673	0,994429459
Tpmt	1332,399674	0,087638402	0,149129643	0,587665873	0,556756586	0,833822555
Plrg1	670,565447	0,006007014	0,09823015	0,061152449	0,951237799	0,985972877
Cd38	207,9727146	0,040136323	0,240341396	0,166997128	0,867372309	0,961775826
Ywhab	2701,988512	-0,074212963	0,069884509	-1,061937247	0,288264174	0,649625922
Ndel1	689,0233447	-0,089942562	0,080775965	-1,113481741	0,265501586	0,626006345
Rab27a	41,99361058	0,157516144	0,269862323	0,583690761	0,559428384	0,835284102
Tubb3	0,147722973	0,780932884	3,352475198	0,232942181	0,815806304	NA
Jam3	18,15187425	-0,391026565	0,376769804	-1,037839446	0,299344809	0,659904651
Mcts2	114,6629648	0,180443586	0,162223951	1,112311624	0,266004188	0,626093308
Rnf128	1856,353904	0,128869475	0,135244696	0,95286158	0,340660209	0,694847567
Zfp120	220,1592225	0,011696248	0,158795929	0,07365584	0,941284238	0,983128178
Tmem176a	3483,248772	0,17069386	0,147761768	1,155196389	0,248010013	0,607129663
Pycard	25,60087787	0,506191506	0,307697835	1,645092839	0,099950667	0,392993389
Aldh3a2	6400,633795	-0,008143598	0,217208192	-0,037492131	0,970092614	0,991981655
Alox12	19,36703633	0,0466699	0,433173351	0,107739547	0,914202299	0,976392941
Cep97	86,16195224	-0,469084365	0,179169326	-2,618106424	0,008841922	0,097773086
Smpd1	1294,356119	-0,12514306	0,09131193	-1,370500655	0,170530671	0,510601034
Zcchc3	13,86129547	0,133068121	0,491128518	0,270943585	0,786434422	0,934050588
Gjb1	18850,46031	0,036246617	0,066472917	0,545283982	0,585558219	0,847482024
Dck	131,8048779	0,06544062	0,1551852	0,421693692	0,67324861	0,888626355
Dcc	0,087021394	-0,517475177	3,352475198	-0,154356154	0,877328924	NA
Itm2b	26884,73869	0,054784754	0,083714989	0,654419886	0,512841326	0,810518464
Itgae	16,71249964	-0,609120165	0,367073877	-1,659393935	0,097036438	0,387479132
Rdx	4460,805208	0,045220578	0,121896947	0,370973832	0,710657023	0,904386988
Il4i1	0,244992664	1,389394708	3,349408814	0,414817893	0,67827522	NA
Map3k7	904,1138045	-0,153552239	0,09472042	-1,621110201	0,104994007	0,402692427
Srr	3125,170298	0,054735212	0,108670683	0,503679659	0,614486504	0,859778545
Rit1	353,9120726	0,408819852	0,127088497	3,2168124	0,001296233	0,028455407

Supplementary Table S1: *Serpina1* KO vs. wildtype all DEGs

Htr1d	0,360104855	-1,122856221	2,59314561	-0,433009321	0,665007998	NA
Pus7	363,7290984	-0,473028208	0,141087692	-3,352724818	0,000800202	0,020415847
Tspyl3	10,17226349	0,363130173	0,577131686	0,629198122	0,529219358	0,818910651
Nccrp1	0,834894673	1,192518053	1,762625273	0,6765579	0,498686498	NA
Trpm1	0,513939868	2,477931508	2,785588719	0,889553971	0,373705431	NA
Cdc37	2070,572879	-0,103014803	0,073620657	-1,399264933	0,161733551	0,49843611
Mcrs1	174,7761416	-0,066996515	0,152899117	-0,438174637	0,661259693	0,882666274
Ddah2	51,47540621	-0,169489211	0,246028875	-0,68889967	0,490886407	0,797600921
Hprt	1426,300656	-0,085712888	0,122318289	-0,700736482	0,483467484	0,794038919
Rpl41	5735,989315	0,0335767	0,111568324	0,300951905	0,763451169	0,925598865
Panx1	13,93872562	0,007483725	0,422628057	0,017707592	0,985872124	0,995929003
Msln	40,76164964	1,985939809	0,617289387	3,217194158	0,00129451	0,028455407
Prdx4	2128,262438	-0,378872798	0,125223408	-3,025574877	0,00248161	0,043424918
Ift20	865,7980237	-0,110915003	0,080101277	-1,384684571	0,166148939	0,504505304
Rplp1	9255,454378	0,019572355	0,122401637	0,159902725	0,872957702	0,963097638
Dclre1a	257,5911342	0,106740079	0,148377959	0,719379612	0,471907056	0,786445858
Asah2	457,2735185	0,27725428	0,182525418	1,518989976	0,128765019	0,446231076
Ap3m1	1246,0173	0,098296703	0,135276899	0,726633326	0,467450607	0,782831914
Sh2b2	61,4682794	0,31654894	0,297879816	1,062673344	0,287930113	0,649353093
Tmem45a	20,82219685	0,354399643	0,537800069	0,658980287	0,509908428	0,808610978
Zbtb22	418,9426445	0,145150487	0,130823596	1,109513053	0,267208917	0,627508918
Adrm1	1806,828993	0,021445276	0,087538344	0,244981509	0,806470749	0,941633837
Dok5	0,588174075	-1,875712458	1,93254732	-0,970590701	0,331752138	NA
Dyrk1a	516,8258112	0,133762687	0,11049096	1,2106211	0,226040654	0,581158101
Npm1	3456,913023	0,014169727	0,099188232	0,142856939	0,886403168	0,967847431
Nde1	476,797239	-0,406023779	0,149671409	-2,712767792	0,006672384	0,082063577
Inppl1	862,6009003	-0,338215854	0,094747213	-3,569665473	0,000357437	0,011486972
Rgl2	319,0532318	0,07752264	0,16052651	0,482927339	0,629147329	0,866097574
Pfkl	1009,096054	-0,293962968	0,090565283	-3,245868152	0,001170931	0,026668093
Sparc	1575,579558	-0,210335466	0,20534093	-1,024323135	0,305682674	0,664636701
Sgf29	439,1168498	-0,234006889	0,133018961	-1,759199494	0,07854363	0,347468495
Ccdc68	41,8646267	0,526999275	0,287209765	1,834893307	0,0665215	0,316036222
Tmem106c	417,6778104	0,086276818	0,096100968	0,897772618	0,369306786	0,717520226
Gpr63	1,947215366	0,941808462	1,134798542	0,829934501	0,406575817	NA
Sspn	22,31363845	0,034247314	0,303864524	0,112705864	0,910263752	0,9747592
Mär-11	0,220592835	0,059593471	3,352475198	0,017775962	0,985817581	NA
Gin1	136,8831942	-0,218657453	0,182622658	-1,197318311	0,23118251	0,588028724
Rpl26	1,03406081	3,437634196	2,990456572	1,1495349	0,250335485	NA
Acss2os	4,76916957	1,043345091	1,283467871	0,812910953	0,416269114	NA
B3glct	48,16252713	-0,225114	0,254175481	-0,885663711	0,375798769	0,721655743
Tbc1d4	61,59552117	-0,098150436	0,190091941	-0,516331388	0,605622977	0,856421285
Mir19a	0,087021394	-0,517475177	3,352475198	-0,154356154	0,877328924	NA
Zdhhc18	630,151297	-0,107703461	0,086584754	-1,243907915	0,213533456	0,566976917
Pih1d2	15,18467172	-0,010476539	0,418061676	-0,025059792	0,980007272	0,994664228
Ccsap	3,498775545	-0,738592379	0,81938388	-0,901399694	0,367375844	NA
Atxn3	404,2090781	-0,049713547	0,118744285	-0,418660542	0,675464237	0,889164486
Cfl1	5152,245921	0,017135832	0,075090437	0,228202587	0,81948875	0,946091978
Igfbp5	321,8063866	1,282023616	0,269195237	4,76243054	1,91E-06	0,000220444
Tkt	4527,893935	0,107976304	0,132494975	0,81494641	0,415102986	0,751551608
Trim21	192,6637813	0,299780418	0,136467985	2,196708758	0,028041253	0,192787383
Ebp	8180,426152	0,281071536	0,096973395	2,898439675	0,003750244	0,057168778
Tnni3	2,984693668	-0,55594927	1,005582899	-0,552862693	0,580357435	NA
Cct3	2710,88794	-0,199771559	0,121535822	-1,643725737	0,100232867	0,393234413
H2-T10	57,90914336	0,423805313	0,251229417	1,686925511	0,091617676	0,375827372
Psme2	1713,4955	0,140000106	0,103022356	1,358929378	0,174168969	0,517013028
Tnni2	1,126714011	0,151873334	1,398971171	0,108560732	0,913550908	NA
Hspa1a	10,03514751	0,089869607	0,664012077	0,135343332	0,892340426	0,969104575
Hgf	122,7581836	-0,187850522	0,264033265	-0,71146536	0,476795909	0,789776299
Foxf1	51,61330597	-0,058171408	0,233418565	-0,249215001	0,803194476	0,940189734
Traf6	426,870431	-0,117309466	0,142204863	-0,824932872	0,409409703	0,746907036
Lcn4	0,209517726	0,059593471	3,352475198	0,017775962	0,985817581	NA
Hexb	337,2647378	0,936682703	0,265449553	3,528665593	0,000417661	0,012865677
Khnyn	1442,719923	-0,124427552	0,198249347	-0,627631583	0,530245314	0,819425454
Crisp2	1,1511008	3,620275278	1,543188697	2,345970577	0,018977606	NA
Prps2	314,5317241	-0,022476387	0,162392046	-0,138408179	0,889917833	0,968877905
Tlr8	53,88801456	0,111278727	0,305568008	0,364170085	0,715731004	0,906719207

Supplementary Table S1: *Serpina1* KO vs. wildtype all DEGs

Gad1	0,519375566	0,410674675	2,440336197	0,168286106	0,866358204	NA
Tsx	0,710660053	2,911033275	1,727296403	1,685311954	0,091928401	NA
Ap3s1	535,3116989	-0,210179817	0,186404996	-1,127543906	0,259512588	0,620981526
Ampd3	25,29510849	0,405961491	0,312929209	1,297294977	0,194529712	0,543617834
Akap3	0,381926467	2,064703897	3,042585971	0,678601662	0,497390289	NA
Heph	23,20105547	-0,065655318	0,388864369	-0,168838605	0,865923592	0,961775826
2900097C17Rik	1344,290706	-0,038112237	0,111224681	-0,342659889	0,731854341	0,912276055
Arhgef11	1057,567982	-0,172421108	0,100816787	-1,710242048	0,087221123	0,366224622
Chst2	54,31032205	-0,299905321	0,412573388	-0,726913878	0,467278715	0,782642088
Gp9	0,942941119	-1,362030913	1,671871462	-0,81467442	0,415258699	NA
Pabpn1	2016,050873	0,190797092	0,136211281	1,400743689	0,161290733	0,497950692
Zfp326	267,2182443	-0,25379521	0,11999419	-2,115062485	0,034424622	0,216957051
Tob2	744,8881699	0,634169602	0,163490333	3,878942497	0,000104912	0,004690844
Tbx21	7,325697959	0,528105032	0,626071867	0,843521422	0,39893689	0,739993697
Syt17	1,14946914	2,667547044	1,621132847	1,64548331	0,099870182	NA
Olr1	0,507283146	0,32361822	2,438083241	0,132734689	0,894403208	NA
Mutyh	9,125577001	0,059515097	0,501907309	0,118577864	0,905609803	0,974022322
Txlnb	0,488044036	-0,579898183	2,127636503	-0,272555101	0,785195229	NA
Tomm70a	2676,527226	0,054418738	0,093307503	0,58321931	0,559745673	0,835352768
Fam104a	363,2521845	0,210636314	0,10007316	2,104823244	0,035306697	0,219460476
Atp5o	4635,592097	0,035821569	0,102152922	0,350666118	0,725838847	0,910393262
Fam50a	628,6562835	-0,135121005	0,085321645	-1,583666202	0,1132697	0,416795277
Coro6	1,397592233	2,29099855	1,384587832	1,65464299	0,097996938	NA
Gldc	6903,425933	0,010031591	0,137664227	0,072869989	0,941909576	0,983128178
D6Wsu163e	217,1906456	-0,087458158	0,134808339	-0,648759256	0,516493996	0,813424627
Slc2a10	5,237880749	0,273765231	0,683773837	0,400373949	0,688881109	NA
Larp7	261,0653593	0,034126682	0,112333521	0,303797844	0,761281909	0,92534041
Ccdc22	394,2426163	-0,133916645	0,100181205	-1,336744195	0,181306151	0,525559828
Usp39	581,2617301	-0,378597087	0,131388349	-2,881511868	0,003957723	0,058659427
Praf2	147,4786396	-0,222101098	0,155784179	-1,425697397	0,153955708	0,489157561
Gfm1	2808,738238	0,008845499	0,103844473	0,085180254	0,932118088	0,980925833
D10Jhu81e	2605,961635	0,094297233	0,066150711	1,425490835	0,154015367	0,489157561
Dgkg	1,022115816	-0,574629119	1,60213019	-0,358665683	0,719845203	NA
Zcchc7	332,2773126	-0,259723765	0,105996714	-2,45030015	0,014273717	0,129990006
Ubfd1	900,678145	-0,480266376	0,131814356	-3,643505836	0,00026895	0,009293414
Mir290b	1,940694302	0,075948562	0,971916339	0,078143107	0,937714218	NA
Cct4	2266,246549	0,01475169	0,086234097	0,171065629	0,864172161	0,961272625
Klf1	59,77297767	0,59963921	0,226381804	2,648795974	0,008077908	0,092381142
Fabp5	1993,164545	-0,089398151	0,462690414	-0,193213752	0,846791569	0,953824301
Adsl	475,3840605	-0,059249952	0,098718768	-0,600189342	0,548380056	0,828815941
Uhmk1	100,3954633	-0,448634572	0,27042793	-1,658980169	0,097119788	0,38761395
Adora3	0,327323282	0,664081931	2,690749744	0,246801819	0,805061603	NA
Kcnn4	9,270502796	0,966011714	0,566192546	1,706154065	0,087979399	0,367864993
Bace2	116,9510152	-0,093417558	0,188436809	-0,495750053	0,620070768	0,862258786
Lmbr1	107,1484757	-0,092813983	0,147786092	-0,628029212	0,529984803	0,819306989
Mapk4	16,6589025	-0,875904547	0,447080904	-1,959163407	0,050093653	0,268956645
Suco	343,0650978	-0,32249743	0,12380926	-2,604792484	0,009192992	0,100011543
Naprt	2417,739868	0,111475306	0,114039314	0,977516454	0,328313536	0,685487182
Mär-06	2366,658849	0,021956463	0,113639512	0,193211523	0,846793315	0,953824301
Sec24c	2987,299021	-0,131539175	0,10100027	-1,302364593	0,19279178	0,541552905
Insm1	0,667279441	0,175895823	1,995209304	0,088159083	0,929750238	NA
Fmod	49,42437315	0,651127214	0,323288772	2,014073086	0,044001861	0,247168435
Map3k11	920,4410013	-0,045052227	0,093258108	-0,483091796	0,629030559	0,866097574
Ms4a8a	24,1612884	0,598699616	0,524059073	1,142427728	0,253276271	0,612785071
Crybb3	8,400534423	0,69090536	0,628112792	1,099970211	0,271345101	0,633246849
Tnfrsf13b	1,222133257	0,441412561	1,41675907	0,311565015	0,755371126	NA
Hipk3	1327,283135	0,181400813	0,147935792	1,226213143	0,220118477	0,574490867
2310001K24Rik	1,091002983	-0,334880954	1,390954628	-0,240756203	0,809744076	NA
Herc3	125,2244588	0,286155291	0,220669911	1,296757175	0,19471475	0,543831245
Phf3	1178,093	-0,067138106	0,086304821	-0,777918368	0,436617139	0,76575019
Syne1	1041,217561	0,141005308	0,132612822	1,063285627	0,287652441	0,649109738
Vamp1	86,21496223	-0,139410262	0,240329331	-0,5800801	0,561860603	0,836751075
Myo1a	0,467045378	1,341999811	2,460373296	0,545445609	0,585447079	NA
St7	186,8511153	-0,036857094	0,123793196	-0,29773118	0,765908344	0,926898815
Atxn7l2	47,59551798	-0,016648947	0,273913055	-0,060781867	0,951532932	0,985972877
Akr1b8	58,42249674	-0,142106044	0,303833043	-0,467710959	0,639991303	0,871049783

Supplementary Table S1: *Serpina1* KO vs. wildtype all DEGs

Trrap	764,9767345	-0,151064277	0,137416734	-1,099314998	0,271630693	0,633536415
Mrps10	720,9969246	-0,134931132	0,100138185	-1,347449347	0,177835557	0,52021398
Cog4	1428,319559	0,071149644	0,103397502	0,688117631	0,491378709	0,797760449
Slc12a8	15,12071253	0,902478657	0,53507512	1,686639173	0,091672755	0,375827372
Akr1c14	8468,752615	-0,165977962	0,149011918	-1,113856954	0,265340559	0,626006345
Armc6	113,0348943	-0,065373226	0,163296291	-0,400335037	0,688909765	0,894264943
Nlrp6	3732,26889	-0,125435244	0,118231779	-1,060926639	0,288723242	0,649821721
Dctn3	536,715029	0,023947152	0,129228399	0,185308745	0,85298688	0,956316741
Ppm1a	1992,63394	0,233612587	0,088992285	2,625088081	0,008662655	0,096351967
Ppl	410,3676407	0,38506283	0,210148385	1,832337806	0,066901108	0,317011065
Asprv1	1,290967295	1,355797489	1,437102374	0,94342443	0,345463852	NA
Ptprm	59,22573664	0,202160826	0,238069576	0,849166992	0,395788377	0,737426273
Orc6	102,4231089	-0,022056677	0,16890944	-0,130582857	0,896105305	0,97084116
Rbms2	415,643417	-0,020580393	0,111429484	-0,184694319	0,853468802	0,956611759
Fkbp7	65,26296802	0,192357446	0,222876313	0,863068143	0,388100002	0,731971786
Ap1g1	2051,305573	-0,214188933	0,085919069	-2,49291497	0,012669918	0,121248489
Ywhaq	870,1980423	-0,18421882	0,108552002	-1,697055941	0,089686085	0,37148762
Ywhah	860,0573015	-0,104460121	0,084416551	-1,237436501	0,215925075	0,569610871
Map3k19	0,520730681	0,462939543	2,140721747	0,216253954	0,828789799	NA
Siae	962,6500016	0,012651585	0,134684038	0,093935299	0,925160553	0,978216671
Caap1	79,39085179	-0,465234843	0,191158082	-2,4337702	0,014942478	0,133622737
Gpatch2	290,0315809	-0,103400508	0,12513736	-0,826296064	0,408636166	0,746898413
Trmt1l	549,7573705	-0,019041061	0,122639171	-0,155260841	0,876615686	0,964416829
Nupl2	107,1843082	0,066868235	0,173356792	0,385726074	0,69969955	0,899209374
Pdxk	1829,496575	0,018368035	0,098761712	0,185983361	0,85245781	0,956082925
Kdm4b	442,4011742	-0,453321464	0,129310911	-3,505670631	0,000455458	0,013744975
Arv1	35,6064732	-0,194142728	0,266063017	-0,729687012	0,465581522	0,781663619
9630028I04Rik	0,200683542	0,059593471	3,352475198	0,017775962	0,985817581	NA
Mrpl52	950,8405625	0,030481891	0,111050248	0,274487378	0,783710102	0,933234199
Pdcl3	543,9235563	-0,004255502	0,099980316	-0,042563395	0,966049575	0,990271183
Ppil1	389,0847015	-0,086031694	0,128537399	-0,669312549	0,503296123	0,804756115
Cmc2	206,5402307	-0,242360216	0,129113124	-1,877115268	0,060502297	0,29803066
Bdh2	385,599431	-0,431701403	0,162756778	-2,652432712	0,007991405	0,091627015
Pdgfrl	11,32027317	-0,865425705	0,544099386	-1,590565486	0,111707397	0,413687165
Mcts1	525,1172327	-0,037756168	0,109328933	-0,345344703	0,729835243	0,911594858
Hyi	895,5293209	0,188963541	0,151280352	1,249095062	0,211630307	0,564073259
Fgfbp3	11,78579362	1,536070802	0,537061035	2,860141961	0,004234514	0,061069276
Washc1	443,0426549	0,082838489	0,105495083	0,785235545	0,432315479	0,762604031
Cgrrf1	803,1318071	-0,035248806	0,115122569	-0,306185017	0,759463779	0,924882144
Snx6	419,1549298	0,145372631	0,117072731	1,241729223	0,214336484	0,567643514
Mthfs	242,6760557	0,440509241	0,123003935	3,581261379	0,000341939	0,011149725
Mrpl30	1098,447848	-0,005883616	0,107118458	-0,054926258	0,956197213	0,987532595
Mrps18c	531,2264697	0,012358502	0,106309387	0,116250334	0,907454147	0,9747592
Gar1	135,748554	0,31529251	0,256485513	1,229279995	0,218966845	0,57359407
Ino80	379,4895914	0,07229747	0,150234731	0,481230065	0,630352995	0,866281784
Dus1l	1749,977973	-0,100777274	0,123877665	-0,81352255	0,415918523	0,751551608
Fkbp9	738,676021	-0,223888458	0,073356278	-3,052069496	0,002272695	0,041521802
Cngb3	0,086476712	0,780932884	3,352475198	0,232942181	0,815806304	NA
Zfp831	3,149036909	0,602256011	0,818349605	0,735939759	0,461767359	NA
Xlr3b	5,287717334	-1,163222473	1,245941636	-0,933609119	NA	NA
Ccdc166	84,78034208	0,230430464	0,256697618	0,897672778	0,369360026	0,717520226
Greb1l	19,75744248	1,038988758	0,703514709	1,476854349	0,139714684	0,464753823
Fam117b	136,1887902	0,286307267	0,192694866	1,485806414	0,137330333	0,460262382
Matn2	29,89732105	-0,243673434	0,330584095	-0,737099689	0,461061728	0,781332773
Anxa4	1125,88083	-0,087866599	0,092269645	-0,952280673	0,340954657	0,694866457
Kcnu1	1,10052301	0,622047582	1,589770173	0,391281452	0,695589207	NA
Stag3	4,221583846	0,40094726	0,6773882	0,591901748	0,553916383	NA
Cr1l	819,8689412	0,047540015	0,096373361	0,493289996	0,621807696	0,862965544
Cyp2e1	121451,8031	0,371380742	0,179275148	2,071568461	0,038305704	0,229748297
Ctss	1087,340176	0,347262576	0,21244754	1,634580356	0,102137048	0,398083115
Myoz1	0,415938566	1,114580217	2,211336641	0,504030095	0,614240229	NA
Ckb	178,0419922	0,02654882	0,208956831	0,127054092	0,898897587	0,971783074
Gbp7	321,6552959	0,727556074	0,21658422	3,359229377	0,000781602	0,020138259
Prickle4	31,22081961	-0,333313616	0,278927318	-1,194983763	0,232093374	0,58906385
Gm12250	59,52083439	0,46459832	0,228868804	2,029976616	0,042358916	0,243150001
Zfp266	344,3772297	0,077871961	0,15210554	0,511960057	0,608678965	0,857753956

AU019823	228,5284028	-0,055218737	0,1220029	-0,452601837	0,650835476	0,877030489
Slc40a1	3112,331242	-0,006798253	0,147387587	-0,046125007	0,963210615	0,988725638
Clec4f	2507,664235	0,074233944	0,198010302	0,374899402	0,707735284	0,902731692
Alb	2300193,241	0,100199631	0,125840725	0,796241687	0,425891572	0,758357009
Gabre	0,853734675	0,763954775	1,940415422	0,393706815	0,693797509	NA
Ccni	2901,002465	0,211435209	0,104041478	2,032220353	0,042131353	0,242468387
Ctps	122,828083	-0,216175656	0,242309057	-0,892148476	0,372313355	0,719447489
Alyref	237,1940367	0,013927	0,12887664	0,108064582	0,913944463	0,976388327
Letm1	2282,391797	-0,117927448	0,089444603	-1,318441181	0,187356003	0,533723077
Csde1	3915,917357	0,011001576	0,063312959	0,173764997	0,862050154	0,960635868
Rgs4	22,31448223	0,163602035	0,407220717	0,40175273	0,687866014	0,893697803
Ptgdr	0,172953423	1,307385949	3,350719078	0,39018071	0,696402925	NA
Dnase1	4,448619425	0,502214405	0,877409625	0,572383059	0,56706249	NA
Rgs2	102,2829373	0,387344921	0,26258288	1,475133952	0,140176534	0,465234266
Rgn	28595,43376	0,282290471	0,130837974	2,157557643	0,030962241	0,204100361
Ptprg	672,6561623	-0,116964756	0,145240422	-0,805318206	0,420636078	0,75436738
Ptpn22	23,6539655	0,349829408	0,338725335	1,032781939	0,301705958	0,661990308
Gata6	171,5556367	0,015955159	0,184777904	0,086347765	0,931189969	0,980584977
Ipo11	383,1183598	-0,040911682	0,117159778	-0,349195627	0,726942449	0,910653449
Foxs1	18,24594539	0,000633326	0,42617068	0,001486086	0,998814275	0,999803624
Ptpra	925,8603891	0,208379205	0,088289873	2,360171078	0,018266509	0,14991666
Ism2	3,062109262	1,801174899	1,153572522	1,561388525	0,118432108	NA
Zfp658	31,13880116	-0,148609633	0,273866351	-0,542635605	0,587380719	0,848572815
Synj1	248,416102	-0,084301221	0,115121754	-0,732278812	0,463998409	0,781663619
Tbc1d17	840,3274809	0,112126616	0,102455396	1,094394447	0,273782018	0,635339664
Nkain2	3,675659292	1,37279728	0,865471743	1,586183824	0,112697616	NA
Zfyve26	806,1776474	0,181461555	0,143650876	1,263212309	0,20651289	0,558880735
B3gnt2	477,09556	-0,177288303	0,135851684	-1,305013653	0,191888199	0,541327987
Srsf5	3727,27078	0,273381836	0,153832663	1,777137769	0,075545575	0,339942375
Erh	213,1883824	-0,128576021	0,15454823	-0,831947549	0,405438551	0,745264401
Ptdss2	230,7071078	-0,077201214	0,122206892	-0,631725529	0,527566244	0,818474757
Atf6	1787,785785	-0,365379406	0,109535704	-3,335710555	0,000850817	0,021158908
Rprd2	449,6468706	-0,036549817	0,114985992	-0,317863217	0,750588695	0,920556312
Cd27	5,76480538	-0,778935866	0,674561927	-1,154728475	0,248201635	0,60717432
Evi5l	150,4512765	-0,06679893	0,127480692	-0,523992526	0,600283738	0,854422974
Mdn1	285,0871501	-0,514215295	0,150839152	-3,409030658	0,000651942	0,017643333
Fam206a	117,4939429	0,319403268	0,15944216	2,003254777	0,045149949	0,251804256
Sult4a1	6,863781305	1,642738714	0,857327039	1,916116768	0,055350234	0,284497014
Jakmip1	4,666981832	-0,999214685	0,846096201	-1,180970538	0,237614428	NA
Cox10	353,1387251	-0,054352685	0,11876475	-0,457649974	0,647203934	0,876230372
Dhx38	579,5329329	-0,159058986	0,086516458	-1,838482398	0,065991353	0,314411405
Commd10	241,2889522	0,507751225	0,128463319	3,952499658	7,73E-05	0,00374618
Zswim3	44,42485979	0,062424731	0,21276592	0,293396287	0,769219263	0,927740953
Cidec	18,17169176	-0,461015199	0,382037617	-1,206727239	0,227537205	0,582859866
Tnfrsf21	132,5791459	0,484784121	0,198433429	2,443056711	0,014563449	0,131287083
Zfp334	46,05520701	0,115987143	0,283694093	0,408845817	0,682652814	0,891903322
Dtd2	287,2554535	0,196851096	0,115228814	1,708349575	0,087571498	0,366889174
Ppp1r3d	3,833070223	-0,146746704	0,782380849	-0,187564284	0,851218226	NA
Anapc1	972,252345	-0,130200228	0,110869787	-1,174352642	0,240253806	0,59790639
Zfp26	173,3781548	-0,100074818	0,142576127	-0,701904449	0,482738754	0,793999136
Zpr1	1034,424545	-0,02081306	0,135674891	-0,153403921	0,878079753	0,965046146
Rbbp9	593,3992238	-0,106973871	0,096815151	-1,10492903	0,269190342	0,629822486
Cops3	1222,892066	0,111820189	0,102718899	1,088603855	0,276328616	0,637487287
Oprd1	0,086476712	0,780932884	3,352475198	0,232942181	0,815806304	NA
Slc27a4	872,0904784	-0,260878828	0,095021608	-2,745468465	0,006042457	0,077377024
Fam163b	0,086476712	0,780932884	3,352475198	0,232942181	0,815806304	NA
C1qtnf7	16,0092307	0,086101532	0,396532582	0,217136084	0,828102294	0,94762353
Orai1	264,6479962	-0,405977836	0,158230777	-2,565732428	0,010295822	0,106618776
Cs	4786,706033	0,225429146	0,130007	1,733976988	0,082922166	0,356458663
Slc38a11	0,496598635	1,200446359	2,438320699	0,49232505	0,622489574	NA
Rps4l	595,1016431	0,078327657	0,180915282	0,43295213	0,665049547	0,884653503
Gm6498	1,170497347	-0,392043916	1,465250348	-0,267561046	0,789037219	NA
Tmem139	6,238221889	0,629587559	0,6636721	0,948642498	0,342802469	0,695589013
Wdr3	388,2325231	0,072715255	0,10687107	0,680401671	0,496250163	0,800192636
Csrp1	280,8103644	0,011322254	0,217923144	0,051955263	0,95856434	0,987896001
Csnk2a1	1469,837168	0,043658394	0,07535258	0,579388181	0,562327277	0,837220482

Supplementary Table S1: *Serpina1* KO vs. wildtype all DEGs

Anln	17,46949204	-0,553468885	0,360612779	-1,534801085	0,124832696	0,439643919
Vps11	745,0025621	0,085024081	0,102448809	0,829917713	0,406585309	0,745871812
Macrod2	92,24865117	-0,149699673	0,202271568	-0,740092513	0,459243861	0,780230167
Vsig4	982,0165181	0,349550345	0,207426015	1,685180837	0,091953687	0,376401973
Zfp385c	1,63012104	0,623180114	1,504760009	0,414139205	0,678772162	NA
Exoc3l	68,85468162	0,0937701	0,256600627	0,365432077	0,7147889	0,906371815
Slc15a5	110,6772373	-0,250609974	0,365450495	-0,685756285	0,492866807	0,798745371
Hist1h1c	702,2443879	-0,044855051	0,205968486	-0,217776284	0,827603424	0,947580409
Zc3h3	50,8951428	-0,028591313	0,211628962	-0,135101134	0,892531914	0,969104575
Klhl23	23,91175454	0,115933971	0,33004585	0,351266258	0,725388606	0,910252319
Ubr3	2231,524882	0,040035312	0,125978034	0,317795977	0,750639702	0,920556312
Zfp518b	45,70392495	0,034660759	0,298887032	0,115966085	0,907679421	0,9747592
Atg13	1258,318421	-0,320928389	0,101702901	-3,155548028	0,001601969	0,033050497
Commd3	1705,052924	0,040697307	0,078867242	0,516022955	0,605838377	0,856477829
Vwa1	12,53475542	0,779441442	0,396546738	1,965572698	0,04934799	0,266451246
Glis1	1,123731503	2,74448606	1,804882205	1,520590127	0,128362726	NA
Abca9	84,33911002	-0,023801305	0,243156446	-0,097884739	0,922023819	0,977341679
Wdfy3	1114,476001	-0,150830703	0,141737811	-1,0641529	0,28725944	0,648651236
Ugt2b35	5272,198908	-0,333451636	0,227630179	-1,464883247	0,142952774	0,470144068
Tmprss11e	1,331152231	1,606854305	1,727878823	0,929957751	0,35239296	NA
Lrrd1	11,30581172	-0,234689558	0,430418811	-0,5452586	0,585575673	0,847482024
Ttc34	0,093303375	-0,517475177	3,352475198	-0,154356154	0,877328924	NA
Pramef8	156,3582246	-0,141586191	0,130464061	-1,085250534	0,277810708	0,638271605
Tstd2	408,6195338	-0,088693934	0,111175127	-0,79778577	0,424994821	0,757668457
Azin2	13,350253	0,113477999	0,410974629	0,276119231	0,78245649	0,93248893
Podn	91,84664921	0,186200491	0,181381629	1,026567533	0,304624149	0,664033955
Kank4	1,322803574	0,371947734	1,244219921	0,298940507	0,764985439	NA
Bnc2	0,763845315	3,002486331	2,572077969	1,167338769	0,243073588	NA
Tspan12	2371,004747	0,105732563	0,142694056	0,740973844	0,458709299	0,779631911
Zfp462	15,13423425	0,405741576	0,395191367	1,026696457	0,304563418	0,664033955
Manea	370,0475029	0,285092033	0,137037871	2,080388667	0,037489898	0,226993861
Wdr63	2,236359635	1,988495638	1,321985017	1,504174112	0,132536542	NA
Zfp697	16,15779762	-0,5030688	0,486333758	-1,034410611	0,300944246	0,661404508
Frem2	1,827260629	0,510044873	1,233584038	0,41346585	0,679265338	NA
Nlgn3	2,555232259	0,275305029	1,097210111	0,250913682	0,801880846	NA
Dzank1	11,86475778	-0,184422573	0,529766074	-0,34812077	0,727749487	0,910785759
Krt222	0,594360853	-0,126721585	2,04587745	-0,061939968	0,950610638	NA
Pak7	0,293986917	-0,62664923	2,757017991	-0,227292398	0,820196384	NA
Exd1	54,48197289	-0,055297179	0,216926534	-0,254912011	0,79879106	0,938733973
Olfml2a	3,669168924	0,274138988	0,75756379	0,36186918	0,717449792	NA
Spata13	665,2798175	0,353175163	0,167692337	2,106090055	0,035196531	0,219162746
Ankmy1	1,898276109	-0,137639021	1,156908228	-0,118971425	0,905297994	NA
Adamts4	13,85802394	0,1920203	0,455863658	0,421223093	0,673592183	0,888626355
Raver2	6,242863239	0,48035418	0,700883554	0,685355188	0,493119814	0,798745371
Fnbp1l	499,4601836	-0,399575921	0,114041029	-3,503790902	0,000458685	0,013811182
Ppp1r16a	533,1628825	-0,143068475	0,098589869	-1,451147839	0,146738698	0,476487281
Copb1	2453,492526	-0,122719645	0,113680864	-1,079510142	0,280360375	0,6416138
Ppp1r3e	78,27795357	-0,292685609	0,204545409	-1,430907739	0,152456659	0,486326192
Gm4952	3181,678475	0,04022434	0,175703633	0,228932887	0,818921079	0,945934875
Fam102a	571,8843659	-0,390735752	0,144778218	-2,698857301	0,0069578	0,084198069
Rpl32	4488,970268	0,09371858	0,12238934	0,765741363	0,44383021	0,770142004
Ppp1r15a	271,3362457	-0,296142121	0,202253015	-1,464216102	0,143134912	0,470269006
Sfxn1	2328,462114	-0,036146351	0,093606547	-0,386151955	0,699384133	0,899044854
Sdr9c7	2033,076842	-0,721589127	0,263770779	-2,73566742	0,006225391	0,078739122
Rpp25l	355,0258804	0,177501919	0,126026657	1,408447405	0,15899863	0,495312554
Grm6	0,122496332	0,780932884	3,352475198	0,232942181	0,815806304	NA
Arl8a	414,6094498	0,115100309	0,087171431	1,320390274	0,186704749	0,533163472
Lurap1l	716,8094543	0,022802484	0,139857803	0,163040483	0,87048656	0,961775826
Tspo2	0,681467096	1,918082624	1,795780992	1,068104982	0,285473159	NA
Cilp2	0,12663974	0,780932884	3,352475198	0,232942181	0,815806304	NA
Sppl3	433,4702931	0,047066381	0,097656801	0,481957022	0,629836478	0,866228449
Cby1	165,8052606	-0,211750226	0,136833679	-1,547500782	0,121742536	0,433673318
Selenom	41,98069849	0,228653647	0,294354975	0,776795593	0,437279377	0,765883401
Lmntd2	74,57440225	0,234105194	0,389852359	0,600497058	0,548175021	0,828815941
Bcl11b	5,429553701	-0,129378415	0,668834684	-0,193438555	0,846615523	0,953824301
Nmd3	626,7288918	0,183850274	0,133561942	1,37651692	0,168661653	0,507888305

Vmn1r53	1,267180534	-0,8963856	1,373092753	-0,652822323	0,513870831	NA
Alg2	353,7187651	-0,28388855	0,179464969	-1,581860525	0,113681414	0,417177269
Bin3	335,6697578	0,264472537	0,16903961	1,564559551	0,117686219	0,425792369
Smyd3	73,65987821	0,012255195	0,189428694	0,064695555	0,948416402	0,985571619
Tmem144	118,221123	0,254665071	0,156234633	1,630016759	0,103097955	0,399438862
2310061I04Rik	749,9956039	-0,072814587	0,117670197	-0,618802291	0,536046613	0,823157267
Ifitm6	9,502698561	-0,756363044	0,533960752	-1,416514305	0,156624979	0,492223634
Dnal4	202,0288653	0,132380323	0,123232163	1,074235173	0,282717292	0,644331312
Zfp316	100,7268827	0,038107871	0,22037763	0,172920777	0,8627137	0,960942085
Igdcc4	3,691749876	0,664355515	0,848683583	0,782807077	0,433740411	NA
Scamp4	636,2172473	-0,161481071	0,088479713	-1,825063238	0,067991487	0,319375503
Nedd9	128,6909072	0,067916871	0,217560261	0,312174982	0,754907544	0,922462845
Prrc2a	2972,010154	-0,25330634	0,081088843	-3,123812469	0,001785242	0,035539776
Ptbp2	171,4875191	-0,183800334	0,136411241	-1,347398735	0,177851848	0,52021398
Pfn2	54,26290436	-0,339251833	0,304330858	-1,114746744	0,264958964	0,626006345
Fads2	8485,86962	0,045757303	0,161375216	0,283546038	0,776758296	0,930009104
Cul3	1736,588736	0,176808354	0,075212351	2,350788823	0,018733662	0,152156946
Rassf1	139,4254583	0,184160984	0,146870409	1,253901217	0,209877913	0,562408863
Snd1	3629,268684	-0,108812174	0,132384955	-0,821937617	0,411112402	0,748591897
Cpb2	11311,48188	0,007577926	0,077375668	0,097936812	0,921982469	0,977341679
1110004F10Rik	847,8893383	0,02756247	0,09302297	0,296297466	0,767002921	0,927464619
Dstn	3965,583413	0,054590272	0,108915999	0,50121445	0,616220206	0,860428813
Dennd6b	169,6458689	0,103559079	0,167181522	0,61944094	0,535625918	0,822988013
C1qtnf2	25,66665805	-0,268466381	0,314313496	-0,854135711	0,393029799	0,735294625
Cnppd1	1233,097356	0,029343335	0,135432784	0,216663459	0,828470627	0,94762353
Nkpd1	0,236587694	-1,308576888	3,335186821	-0,392354899	0,694796005	NA
Tmprss6	11537,6031	-0,131141618	0,056322309	-2,328413388	0,019890163	0,157530566
4632415L05Rik	156,6842154	0,185413542	0,180139708	1,029276357	0,30334983	0,663017899
Dnaic2	0,487299113	1,421191079	2,411883993	0,589245206	0,555696796	NA
Ccdc24	3,279052922	1,047812486	1,002247081	1,045463246	0,295808919	NA
Prrc2c	959,5949173	-0,088371971	0,138380817	-0,638614318	0,523073865	0,815322684
Esco1	256,0791705	0,211290725	0,166276063	1,270722443	0,203827409	0,556841672
Ndrg2	32393,6726	0,159011636	0,111957955	1,420279919	0,155526205	0,490615817
Dcun1d3	207,7552943	-0,195252306	0,142488266	-1,370304456	0,170591883	0,510601034
Fbxo27	0,549738981	0,332189906	2,121940732	0,15655004	0,875599479	NA
Mir7014	0,332014057	0,667413114	3,210961268	0,207854614	0,835342488	NA
Rbx1	894,1790726	0,03364693	0,09594996	0,35067164	0,725834704	0,910393262
Nrgn	7,124795033	0,637307352	0,680714502	0,936232957	0,349153258	0,700954068
Smc1a	1524,342837	-0,118904811	0,081085617	-1,466410624	0,142536454	0,469815052
Smim15	390,3058725	-0,125990195	0,117604793	-1,071301538	0,284033869	0,645351596
Zfr2	7,21675555	0,603217723	0,650557421	0,9272321	0,35380604	0,704551502
C8a	23572,57341	-0,729555193	0,236181571	-3,088959009	0,002008591	0,038349124
Gbp10	245,9677048	0,320651082	0,25125141	1,276216052	0,201879156	0,553284631
Edem3	943,1814762	-0,435692108	0,150497535	-2,895011594	0,003791445	0,057439938
Abca3	2260,034747	0,311942282	0,181673753	1,717046501	0,085970676	0,363485758
Prlr	3942,178422	1,106642851	0,395304821	2,799467127	0,005118703	0,069899835
Creld1	1341,726691	-0,495590658	0,138558625	-3,57675792	0,000347882	0,011261102
Sec11c	717,2623091	-0,461799201	0,115197065	-4,008775746	6,10E-05	0,003138339
Ap3s2	677,3983498	-0,098976083	0,093835132	-1,054787064	0,291522722	0,653216736
Lama4	82,23332242	-0,140120399	0,297408929	-0,471137163	0,637542788	0,870117708
Ybx3	604,0494363	0,048601378	0,134850443	0,360409482	0,718540937	0,90815359
Snx16	70,63655153	-0,108031577	0,201721757	-0,535547471	0,592271341	0,85107672
4930429B21Rik	1,691199671	-1,08773146	1,366596977	-0,795941655	0,426065948	NA
Lin7a	685,5931153	-0,038553343	0,117297997	-0,328678613	0,742398621	0,916955577
Sult2a8	17319,07104	-0,282882682	0,219289228	-1,289998074	0,197051327	0,546665115
Ablim3	851,2851589	-0,158138747	0,178327531	-0,886788182	0,375192952	0,721201232
Qk	1023,592209	-0,073669465	0,15155459	-0,48609194	0,626901981	0,865542971
Tas2r138	0,76776403	2,148163232	1,931558125	1,112140093	0,266077921	NA
Cnpy2	1892,888329	0,041024485	0,084297014	0,486665938	0,626495087	0,865266164
Sec11a	1301,600194	-0,226100092	0,121470614	-1,861356296	0,062693878	0,303899367
Psd	12,96134461	0,717192658	0,417268188	1,71878106	0,085654246	0,362951382
Smu1	1534,025442	-0,090568807	0,106344789	-0,851652514	0,394406984	0,73637054
Pxmp4	2638,188417	-0,323455263	0,128496792	-2,517224424	0,011828346	0,116188947
Dact1	64,80608626	-0,78448274	0,241925031	-3,24266876	0,001184158	0,026877772
Carm1	779,5754647	-0,018035837	0,088579425	-0,203612035	0,838656688	0,951141594
Ubd	15,63550471	1,098487972	0,516712855	2,125915701	0,033510279	0,21394409

Rab8a	1994,885916	-0,043495943	0,070771891	-0,614593482	0,53882322	0,825430002
Baz1a	209,8638348	-0,217559163	0,194199854	-1,120284893	0,262592378	0,622562722
Cmas	1116,469069	-0,273360227	0,119850712	-2,280839404	0,022557952	0,168573089
Inha	2,894146657	0,833740564	0,881436274	0,945888646	0,344205385	NA
Spa17	16,29999048	0,275442445	0,354740549	0,776461687	0,437476434	0,766027825
Ski	830,0145558	0,050796119	0,113289986	0,44837254	0,653884356	0,878394288
Slc26a4	10,87663166	1,338122412	0,492823329	2,715217266	0,00662323	0,081759895
Abhd2	471,5387475	0,213919278	0,446098906	0,479533294	0,631559288	0,867137324
Mkrn1	527,1053688	0,335652029	0,129079506	2,600351043	0,009312844	0,100244293
Lrrc6	0,259430135	1,627812584	3,345388605	0,486584005	0,626553161	NA
Apbb1ip	63,47317275	0,403507761	0,246708119	1,635567413	0,101930155	0,397591507
Plagl2	133,1538873	-0,079964094	0,177483228	-0,450544512	0,652317866	0,877662367
Ncs1	4,925165656	0,364599104	0,692279564	0,526664548	0,598426559	NA
Parva	2015,996676	-0,056821217	0,08767443	-0,648093371	0,516924561	0,813424627
Acp6	866,264918	0,161063437	0,089478922	1,80001539	0,071858208	0,32888476
Tmeff2	5,537563451	-0,733541101	0,923455505	-0,794343742	0,426995346	0,759210105
Akap10	248,0694958	0,026231939	0,118648344	0,221089803	0,82502251	0,947537943
Gm6634	0,302926139	0,617345448	2,744069617	0,224974412	0,82199918	NA
Cpxm1	32,34514532	-0,197187012	0,464496874	-0,424517414	0,671188508	0,887461098
Pard6a	77,88700761	-0,218732647	0,219131434	-0,998180148	0,318192011	0,675759967
Abcb9	39,71470747	-0,270829339	0,258453821	-1,047882898	0,294692562	0,655749811
Jmy	417,9653421	0,041138171	0,10403264	0,395435228	0,692521715	0,895338108
Tnip1	284,6949011	-0,004759101	0,107842815	-0,044129975	0,964800799	0,989779119
Gcc2	560,2997576	-0,118031695	0,146031699	-0,808260784	0,418940473	0,753709485
Tmem256	1454,514178	0,116057635	0,104296975	1,112761275	0,265810971	0,626006345
Dab2	303,9101832	-0,220853427	0,236646398	-0,93326342	0,350683977	0,702050628
Ctsw	11,29100471	0,1640524	0,511071975	0,320996665	0,748212918	0,919838327
Ypel1	4,95468785	2,8154139	0,864872952	3,255291883	0,001132759	NA
Qtrt1	87,57584865	0,348063589	0,180455235	1,928808488	0,053754642	0,280050037
Scn3a	3,57918381	-0,520180296	0,916140889	-0,567795087	0,570174116	NA
Atp4a	96,63752518	-0,357219941	0,854534291	-0,418028796	NA	NA
Mef2c	35,67071972	0,138953875	0,341277184	0,407158407	0,683891648	0,892293506
Lyar	324,0718184	-0,118616487	0,120602627	-0,983531536	0,325345902	0,682710621
Kin	201,8101744	0,095817882	0,165595473	0,578626214	0,562841413	0,83722511
Atp6v1f	1179,168714	-0,052276336	0,104951209	-0,498101321	0,61841263	0,861473215
Zfp524	321,7269078	0,040796434	0,120166593	0,339498967	0,734233875	0,913675851
Gng10	88,87082391	0,155352082	0,199932779	0,777021572	0,437146043	0,76575019
Pdzk1	1982,673166	-0,256659575	0,124627707	-2,059410236	0,039454956	0,233292042
Rdh7	25911,00646	0,00245607	0,071580941	0,034311784	0,972628528	0,992650147
4930577N17Rik	6,093431478	0,419948697	0,793137647	0,5294777	0,59647411	0,853075785
5031434O11Rik	3,042540113	-0,023819357	0,871355876	-0,027335969	0,978191769	NA
Lilrb4a	123,7436538	0,424582687	0,231765142	1,831952308	0,066958526	0,317098313
Zfp729a	128,0101579	-0,181327424	0,242517001	-0,747689538	0,454647443	0,776665176
Tet3	388,7207964	-0,243381014	0,120140153	-2,025809085	0,042784353	0,243209687
Fam229b	9,386576186	0,195550537	0,528735139	0,369845925	0,711497294	0,905215771
Tmprss12	0,12663974	0,780932884	3,352475198	0,232942181	0,815806304	NA
4930451I11Rik	0,087021394	-0,517475177	3,352475198	-0,154356154	0,877328924	NA
6030498E09Rik	0,734936497	-0,294502828	2,276540322	-0,129364205	0,89706947	NA
Slc18b1	92,65646515	-0,311687206	0,170538643	-1,82766323	0,06760011	0,31855688
1700029I15Rik	17,34574057	0,398039886	0,414727682	0,959762039	0,337174992	0,69270916
Tepsin	256,1878893	0,020271385	0,133959989	0,15132418	0,879719996	0,965438896
Prkab2	59,880712	0,030650131	0,207270347	0,147875137	0,88244131	0,966166219
1700067K01Rik	10,67078078	1,441883104	0,516022724	2,794224048	0,005202442	0,07004174
Ttc17	762,1617487	-0,291245085	0,088827087	-3,278786863	0,001042543	0,024581593
Reep1	11,12417857	0,601440392	0,661354403	0,909407104	0,363135273	0,712258725
Arl5a	1878,050058	-0,266915055	0,171876499	-1,552946779	0,12043585	0,4317798
Mypn	8,684136522	-0,272213315	0,483129099	-0,563438046	0,573136636	0,84235265
Tmem59l	0,227324296	1,389394708	3,349408814	0,414817893	0,67827522	NA
Tonsl	44,15618415	-0,215894379	0,233147986	-0,925997187	0,354447442	0,704662593
Imp4	603,4475183	-0,211050117	0,092756413	-2,275315638	0,022887002	0,170270634
Fam189a1	7,326254722	-0,212192572	0,727639953	-0,291617538	0,770579065	0,927934035
Tyw5	210,4327958	0,195773557	0,122054378	1,603986355	0,10871706	0,409049888
Tfeb	190,618451	-0,118798903	0,179215594	-0,662882624	0,507405731	0,806849558
Dnajc13	828,9024988	-0,10027005	0,136314423	-0,735579169	0,461986844	0,781516914
Fam19a2	68,73936286	2,750709538	1,090211448	2,523097279	0,011632622	0,115306358
Fam167b	122,9949606	-0,058632635	0,236891199	-0,247508708	0,804514553	0,94058199

Supplementary Table S1: *Serpina1* KO vs. wildtype all DEGs

Pcbd1	6678,961478	0,214813294	0,097370812	2,206136414	0,02737445	0,190807622
Sgta	1953,510835	-0,021542526	0,083455574	-0,258131664	0,796305295	0,937785232
Tmem170	125,7140045	-0,118023551	0,146444577	-0,805926401	0,420285288	0,75436738
Cops6	1891,168974	0,154483873	0,105259436	1,46764869	0,142199675	0,469051925
Slc9a3r1	5434,249677	-0,205244105	0,082470508	-2,48869698	0,012821219	0,121737839
Plpp2	459,47854	0,200483438	0,184736176	1,085241899	0,277814531	0,638271605
Msl3	742,7459192	0,078924118	0,10697722	0,737765645	0,460656874	0,780953195
Cpt1a	4988,475739	0,139563456	0,112247254	1,243357428	0,213736151	0,567064616
Xcl1	5,729149333	1,687345825	0,725476705	2,325844254	0,020026861	0,158066552
Cnn2	151,969173	-0,170504751	0,216930575	-0,785987643	0,431874726	0,7622164
Cndp2	1181,459757	-0,13987044	0,11728801	-1,192538264	0,233050256	0,589913858
Cldn6	20,68931025	1,008186174	0,375643254	2,683892661	0,007277047	0,086554124
Eif3d	2019,580852	-0,087654309	0,080985873	-1,082340732	0,279101153	0,639578902
Prkag1	940,2720928	-0,069211385	0,088893202	-0,778590298	0,436221096	0,76575019
Stx3	26,23035589	0,13272263	0,363696685	0,364926697	0,715166126	0,906371815
Slc17a8	452,478214	-0,5204444	0,493991949	-1,053548343	0,29208975	0,653967601
Kif20b	17,25813122	-0,66923372	0,353153006	-1,895024846	0,05808911	0,291295316
Ppp4r2	488,4805603	0,064105805	0,132993496	0,482022104	0,629790244	0,866228449
Trim46	5,749069852	1,034812628	0,683137354	1,514794384	0,129824483	0,447902843
Plekhm1	492,7867222	0,00635989	0,118829206	0,053521272	0,957316582	0,98781716
Gpr183	3,374820859	-0,846764564	0,858083212	-0,986809382	0,323736094	NA
Igf2bp2	40,7430377	0,287765158	0,439281198	0,655081891	0,512415031	0,810227859
Dhrs13	98,82401613	-0,086843329	0,171955098	-0,505034918	0,613534311	0,859428114
Nr2f2	559,9493656	0,054682404	0,15612319	0,350251646	0,726149851	0,910393262
Scd4	0,781181988	-1,63543757	1,945035962	-0,840826392	0,4004452	NA
Plch1	0,817370234	0,271370005	1,614621629	0,168070339	0,866527943	NA
Rims3	5,085710616	-1,026744008	0,786731359	-1,305075737	0,19186706	NA
Ap1s3	26,56626732	-0,21110447	0,271257927	-0,778242583	0,436426017	0,76575019
Arid1a	981,1304154	0,043619497	0,122994525	0,354645839	0,722854931	0,909744709
Spata5	103,7235399	0,044011351	0,254241772	0,173108262	0,862566331	0,960942085
Des	103,0268238	0,131537641	0,215073925	0,611592694	0,540807273	0,826407188
Npepps	740,431435	-0,078263991	0,084459919	-0,926640616	0,354113159	0,704551502
Srprb	704,4479448	-0,701698855	0,139449908	-5,031906185	4,86E-07	7,21E-05
Efnb1	424,4654706	-0,143629747	0,108278085	-1,32648954	0,184677598	0,531186489
Cntd1	4,006561279	-0,361608653	0,876498456	-0,412560513	0,679928637	NA
Selplg	72,96490971	0,22030301	0,257005396	0,857192158	0,391338695	0,734518115
Lefty1	10,71704675	-0,536344962	0,473860267	-1,131863123	0,257691984	0,618708277
Rdh16	2045,939804	1,179679786	0,216646474	5,445183422	5,18E-08	1,31E-05
Gclc	10770,62619	-0,464236278	0,135633886	-3,422716048	0,000619988	0,017059565
Tnfsf10	103,4511303	0,337292249	0,280125892	1,204073806	0,228561053	0,584362731
Prox1	809,9313094	0,092290426	0,227446413	0,405767782	0,684913233	0,892717658
Faf2	1457,441048	-0,09652072	0,079973001	-1,206916331	0,227464368	0,582859866
Snapc1	128,5781307	-0,084902284	0,139250979	-0,609706911	0,542055976	0,826611597
Zdhhc2	18,97198509	-0,855599849	0,391762926	-2,1839735	0,028964195	0,196459831
Pcdhb20	2,825140237	-0,854720227	0,90904332	-0,940241469	0,347093715	NA
Pcdhb17	5,572661843	-0,335361215	0,692604691	-0,484202922	0,628241864	0,865852703
Pcdhb13	0,707354883	0,479490515	2,360256023	0,203151908	0,839016301	NA
Pcdhb3	0,36279822	1,946356901	2,599162948	0,748839892	0,453953713	NA
Arhgef28	3,436205205	1,125563177	0,904370668	1,244581692	0,213285553	NA
Azgp1	42281,70935	0,052273667	0,074960172	0,697352552	0,485582188	0,79430833
Igf1	14268,7353	0,094674161	0,089972983	1,052251002	0,292684405	0,654074594
Clcn1	0,387940012	-1,136012433	2,196518869	-0,51718765	0,605025172	NA
Casp8ap2	137,8042241	-0,32310277	0,149416287	-2,162433406	0,030584785	0,202340544
Gm6525	0,754431887	-0,923301789	1,908661515	-0,483743074	0,62856822	NA
Mrpl46	540,9157519	-0,024080256	0,092934377	-0,259110314	0,795550127	0,937598879
Ptpmt1	1298,926833	0,099329478	0,127354707	0,779943514	0,435424124	0,765139999
Nosip	484,4350319	0,052473153	0,10239305	0,512467916	0,608323569	0,857753956
Ap1b1	1465,394813	-0,136592897	0,083058153	-1,644545312	0,100063613	0,392993389
Tyms	165,1879562	-0,068292205	0,146605009	-0,465824503	0,641341125	0,871931508
Kcnd2	0,51886027	2,505843628	3,297128873	0,76000779	0,447249928	NA
Snord73a	0,897392529	-1,571101065	2,143485282	-0,732965641	0,463579386	NA
Rnu73b	3,15251702	-0,82544873	0,869416281	-0,949428655	0,342402642	NA
Trappc1	751,5194016	0,143358589	0,086140921	1,664233307	0,096065814	0,385586553
Nufip2	667,0004785	0,128362113	0,157700188	0,813962966	0,415666167	0,751551608
Fbxo10	27,29087866	-0,048132564	0,290756523	-0,165542508	0,868516995	0,961775826
Hpse2	0,086476712	0,780932884	3,352475198	0,232942181	0,815806304	NA

Supplementary Table S1: *Serpina1* KO vs. wildtype all DEGs

Zcchc2	937,3443228	-0,077657191	0,136982466	-0,56691337	0,57077304	0,8413217
Mkl2	609,9724544	-0,44912264	0,135118245	-3,323922976	0,000887607	0,021693636
Cdkl4	8,984073884	0,676500364	0,680400764	0,994267496	0,320092656	0,677494232
Alg10b	440,4212703	-0,244055781	0,212727076	-1,147271828	0,251269294	0,610767125
Lrch1	188,3046342	0,100275562	0,179120357	0,559822254	0,575600683	0,843861602
Vamp4	264,2760033	-0,069249579	0,141943417	-0,487867494	0,6256437	0,864942192
Pdcd7	168,9038081	0,047312971	0,136985581	0,345386504	0,729803822	0,911594858
Greb1	0,565620335	1,609893863	2,35875799	0,682517609	0,494911712	NA
Nfil3	1377,195976	-0,111160339	0,197649274	-0,56241208	0,57383529	0,84275089
Mtx2	1124,150771	0,320551644	0,08204584	3,9069823	9,35E-05	0,004308321
Scn2a	4,178664942	0,062904628	1,241249054	0,05067849	0,959581717	NA
Fgd3	16,24173852	0,513945751	0,50409912	1,019533126	0,307949935	0,667059844
Hunk	108,7640617	-0,294517315	0,278437342	-1,057750778	0,290169079	0,651777651
Klra1	0,093953095	-0,517475177	3,352475198	-0,154356154	0,877328924	NA
Rgs14	9,429297258	0,551786821	0,590603308	0,93427655	0,350161257	0,701844955
Myadm	849,875219	0,015646306	0,139556524	0,112114473	0,910732642	0,974815504
Nudt5	251,7742182	-0,259901145	0,132839828	-1,956500166	0,050406263	0,269938714
Rwdd2b	186,2657667	0,358075437	0,129298508	2,769370208	0,005616478	0,073470343
Nop58	578,2835312	0,242699646	0,218317042	1,11168438	0,266273878	0,626507476
Stx8	377,9342182	0,094918775	0,091483773	1,037547661	0,299480695	0,659904651
Erbb3	2232,61483	-0,014155904	0,249138585	-0,056819394	0,954689064	0,986797441
Art3	41,3535055	-0,32408833	0,317270901	-1,021487721	0,307023424	0,666222391
Cnmd	58,4438255	0,216144857	0,358827654	0,602363989	0,546931875	0,828265999
Pikfyve	452,7419395	-0,242317715	0,109762784	-2,207649123	0,027268742	0,190567596
Plxnc1	260,3599436	-0,099407593	0,461862091	-0,215232199	0,829586288	0,948328541
Hs3st3b1	1576,724261	-0,006350675	0,166592534	-0,038121004	0,969591205	0,991924152
Blcap	826,6206501	-0,043379185	0,10294048	-0,421400647	0,673462547	0,888626355
Ears2	381,1661719	-0,294945151	0,119486489	-2,468439343	0,013570365	0,126214793
Ube2g1	1013,32972	0,034729228	0,123404642	0,281425621	0,778383969	0,930864224
Cdkl2	143,1710681	-0,107220302	0,170431382	-0,629111263	0,529276217	0,818910651
Rragc	1001,951744	0,079686866	0,091138701	0,874347177	0,381929215	0,726456076
Tsnax	777,6530804	0,095157561	0,100561851	0,946259044	0,344016477	0,697158752
Rab25	6,804811428	1,271163765	0,727278981	1,747835146	0,080492578	0,350751392
Ccrl2	33,63145351	0,279851772	0,270942761	1,032881523	0,301659347	0,661990308
Pbx2	456,5700091	-0,174263503	0,11478028	-1,518235564	0,128955025	0,446309134
Cd164	8312,560337	-0,121642701	0,154984149	-0,784871886	0,432528687	0,762604031
Zranb2	882,9098304	-0,221399539	0,131972702	-1,677616174	0,093422053	0,380085036
Mir3108	0,086476712	0,780932884	3,352475198	0,232942181	0,815806304	NA
Rab37	8,170128992	0,210690463	0,562509372	0,374554582	0,707991756	0,902731692
Hcfc1	1213,674347	-0,196046814	0,122251948	-1,603629375	0,108795772	0,409049888
Tgfbrap1	1080,73393	-0,219389233	0,106083095	-2,06808854	0,038631698	0,230668679
Mug1	80131,36578	-0,347906418	0,187934794	-1,851208129	0,064139617	0,308336044
Spg7	1079,549967	0,001753831	0,090717383	0,019332914	0,984575527	0,995554769
Slc36a2	0,56831074	2,6031916	3,292540805	0,790633056	0,429158151	NA
1600015I10Rik	0,113662148	0,780932884	3,352475198	0,232942181	0,815806304	NA
Ldlrad1	0,213116452	1,337107854	3,350237755	0,399108348	0,689813372	NA
Fam13c	6,601371462	1,218239622	0,850519284	1,432348031	0,152044243	0,485381413
Acss3	353,6997783	1,364906649	0,54336232	2,511964113	0,01200613	0,116989758
Glyatl3	0,093953095	-0,517475177	3,352475198	-0,154356154	0,877328924	NA
Spsb1	32,16353025	-0,247402672	0,271256672	-0,91206115	0,361736528	0,71109038
Golgb1	1366,105174	-0,336035294	0,104824089	-3,205706794	0,001347312	0,028866309
Zfp558	20,52774298	0,364207741	0,312734619	1,164590419	0,244184824	0,602840659
4930452B06Rik	30,47789261	-0,121281069	0,300395936	-0,403737381	0,686405848	0,893161227
Shpk	1287,237522	0,196703263	0,125232558	1,570703864	0,116251451	0,422672193
Zfp623	299,2380387	0,130483688	0,153856737	0,848085632	0,396390283	0,737761616
Aacs	2665,494879	0,350347389	0,408783974	0,857047758	0,391418491	0,734518115
Mccc2	2015,640329	0,116879655	0,093449816	1,250721084	0,211036255	0,563817685
Ccdc150	0,840540802	-0,801038025	1,933644859	-0,414263261	0,678681317	NA
Polr3c	549,6425913	0,094631569	0,09419821	1,004600507	0,315089253	0,673856248
Tmem81	56,15042261	-0,052430149	0,239364181	-0,219039242	0,826619479	0,947537943
Tc2n	59,67078107	0,119001844	0,220767669	0,539036554	0,589861632	0,84953676
Prr15	5,45781129	1,682364188	0,944404226	1,781402647	0,074846696	0,338112818
Gle1	677,8093677	-0,009833218	0,080621189	-0,121968153	0,90292424	0,973489035
Ndufv2	2834,668665	0,1488194	0,081311498	1,83023807	0,067214347	0,317560326
Slc25a17	1448,68474	-0,199102179	0,114157678	-1,744098012	0,081142001	0,352203704
Rab33a	0,820932182	-1,462349019	1,58440576	-0,922963711	0,356026117	NA

Supplementary Table S1: *Serpina1* KO vs. wildtype all DEGs

Pygm	32,81323424	-0,143004319	0,322415375	-0,443540633	0,657374736	0,880359254
Psma3	2999,317647	0,126741009	0,086186439	1,47054467	0,141414297	0,467194607
Rpsa	11975,97581	-0,037416199	0,131444048	-0,284654954	0,775908505	0,929745022
Notch4	66,43094371	-0,065469125	0,260790723	-0,251040849	0,801782527	0,939799232
Stxbp1	52,77397741	-0,088389359	0,330948908	-0,267078564	0,789408672	0,935361858
Treml1	1,231914876	-0,66297887	1,279176439	-0,518285711	0,604258941	NA
B3galt4	5,75889214	-0,814618641	0,758754744	-1,073625763	0,282990446	0,644404577
Cdk11b	1105,888701	-0,217905389	0,103376369	-2,107883949	0,035041029	0,218499774
Grap2	11,65124239	1,022040589	0,464621023	2,19972954	0,02782609	0,192152372
Tfcp2	175,6881454	-0,374433171	0,151863	-2,465598399	0,01367846	0,126862287
Gipc1	298,5487481	0,296260683	0,114804715	2,580561986	0,009863965	0,103666169
Dfna5	30,08290721	0,51910304	0,310820358	1,670106306	0,094898333	0,383176023
Timm10b	478,2850324	-0,054672793	0,121477403	-0,450065543	0,652663181	0,877662367
Pdss1	68,95312504	0,451087747	0,178605179	2,525614034	0,011549629	0,115057371
Prss16	0,903762096	-1,199753414	1,493532703	-0,803299058	0,421801902	NA
Cyp1a2	13772,76424	0,075993466	0,194874824	0,389960407	0,696565825	0,897560279
Tma16	89,81300853	0,058719447	0,180252003	0,325763078	0,744603616	0,918321563
Slc22a27	181,4198799	4,336739195	1,24697805	3,477799144	0,000505549	0,014789232
Tab2	2336,156544	0,187058575	0,124326591	1,504574148	0,132433597	0,453014264
Bloc1s5	655,7593976	0,142651514	0,10564746	1,350259767	0,176932673	0,520079413
Havcr2	4,960506686	0,949923198	0,715705125	1,327254989	0,184424348	NA
Acot4	364,1684976	0,418967359	0,245107382	1,709321666	0,087391382	0,366594097
Elovl5	14842,05496	0,581655799	0,213617323	2,722886843	0,006471422	0,080389854
Acot2	63,57846582	0,662804234	0,321686478	2,060404396	0,039359897	0,233056618
Trpm8	0,858711284	1,273334074	2,012164161	0,632818186	0,526852379	NA
Ifi35	654,5322518	0,080241374	0,129539228	0,619436873	0,535628596	0,822988013
Cpsf3l	475,387657	-0,218232409	0,097911143	-2,228882248	0,025821741	0,184210699
Alpk1	50,11719	0,696082963	0,249924156	2,785176809	0,005349854	0,070973656
Cfap52	0,240301888	1,389394708	3,349408814	0,414817893	0,67827522	NA
Hist2h2bb	0,087021394	-0,517475177	3,352475198	-0,154356154	0,877328924	NA
Hist1h2bk	0,235937973	-1,303449117	3,335546383	-0,390775294	0,695963339	NA
Rbbp6	1081,990721	0,03906648	0,109988343	0,355187461	0,722449158	0,909644412
Crtc3	430,948226	-0,367335551	0,239412536	-1,534320455	0,124950836	0,439828259
Ccdc191	86,34044447	-0,160088739	0,208804337	-0,766692594	0,443264308	0,769910424
Hist2h2ac	0,496457473	-1,655806381	2,06046595	-0,803607738	0,421623553	NA
Hist1h2af	0,229655992	-1,252384348	3,339190293	-0,375056298	0,707618598	NA
St13	3126,243122	-0,180815705	0,103116281	-1,753512673	0,079514046	0,349223642
Wdpcp	64,05488636	0,322727067	0,20940432	1,541167187	0,123276101	0,43588421
BC089597	3638,778959	0,830168628	0,213820523	3,882548868	0,000103367	0,004652918
Slc5a8	0,474103974	1,13407257	2,455378234	0,461872861	0,644172497	NA
Ncdn	230,2921438	-0,262565796	0,133769995	-1,962815331	0,049667636	0,267192299
Utp14b	148,9258909	0,562926968	0,251399068	2,239176829	0,025144413	0,18170576
Uchl4	1,555396296	1,757842172	1,317017696	1,334714163	0,181969916	NA
Rab28	296,892667	0,03365493	0,107016864	0,314482487	0,753154616	0,921533083
Selenot	3311,244857	-0,052582362	0,128274991	-0,409919048	0,681865332	0,89172987
Ilk	1594,969793	-0,066754757	0,095867563	-0,696322658	0,486226786	0,794860101
Pcdhgb8	0,394221993	-1,149575818	2,531438941	-0,454119513	0,64974281	NA
Paip1	1061,049731	-0,075284794	0,100731499	-0,747380853	0,4548337	0,776884084
Psmc3	2883,478334	0,046086464	0,086171787	0,534820806	0,592773774	0,851332066
Atp6v1h	1186,39574	0,4710372	0,179740433	2,620652417	0,008776169	0,097260878
Fzd1	148,9430302	0,103322358	0,203784266	0,507018329	0,612141958	0,858888222
Capza2	1369,475717	0,010697781	0,102010785	0,104869116	0,916479666	0,976559041
Igfbp6	32,99558214	1,016864377	0,482311928	2,108312728	0,035003948	0,218499774
Car8	1933,580267	0,152200515	0,123113458	1,236262204	0,216361115	0,56973247
Ryr1	1,692206159	0,336956567	1,261597871	0,26708714	0,789402069	NA
Bdkrb2	3,50994726	1,483146319	1,130890622	1,311485205	0,189693889	NA
Alkbh8	73,63678062	0,095705106	0,245844813	0,389290729	0,697061094	0,897958158
Dctn4	527,549896	0,094194271	0,095013481	0,991377964	0,321501056	0,678746017
Il11	0,546590389	1,63869009	2,390888077	0,685389712	0,493098034	NA
Igsf10	30,41582833	-0,095572562	0,343833586	-0,277961683	0,781041773	0,932049225
Mpped2	5,4459698	1,337669928	0,831636603	1,6084789	0,107730334	0,407655486
Tmem255b	0,968777391	1,51576466	1,991968879	0,760937922	0,446694147	NA
Zfp58	105,4304127	0,123783255	0,162775892	0,760452017	0,446984441	0,772195705
Fibp	478,9662328	0,051423612	0,132510414	0,388072229	0,697962583	0,898426898
Fndc4	1057,886823	0,105634299	0,094262586	1,120638671	0,262441697	0,622562722
Cabp1	1,909419912	0,278965701	1,261272064	0,221178054	0,824953797	NA

Supplementary Table S1: *Serpina1* KO vs. wildtype all DEGs

Gm765	2,820832051	1,681789124	0,97442038	1,725937962	0,084358574	NA
Zfp939	48,03571302	0,144354779	0,216098321	0,668005094	0,504130341	0,804997854
Actn3	0,522770242	2,452569149	2,807361547	0,873620696	0,382324852	NA
Acr	0,604153349	-0,916885217	2,03146454	-0,451341975	0,651743097	NA
Abca1	3907,964418	-0,28058991	0,151101323	-1,856965276	0,063316094	0,30602779
Atp1b2	15,27522003	-0,249719799	0,408555997	-0,611225392	0,541050375	0,826407188
Chst4	6,102325922	1,522982601	0,798262606	1,907871659	0,056407806	0,287141119
Col4a1	828,8007519	-0,324834556	0,200308202	-1,62167376	0,10487322	0,402692427
Tnf	3,993015203	0,359364058	0,743281883	0,483482869	0,62875292	NA
Adh4	4098,629626	0,131797857	0,180916009	0,728503011	0,466305726	0,781992129
Lrrc73	1,999746369	-0,914297806	1,219562174	-0,749693476	0,453439338	NA
Sprtn	89,4153256	-0,033074106	0,199522119	-0,165766616	0,868340619	0,961775826
Ccdc6	524,8764212	0,034382891	0,096817967	0,355129239	0,722492773	0,909644412
Rad51ap2	0,087021394	-0,517475177	3,352475198	-0,154356154	0,877328924	NA
Atg16l2	369,4902663	0,133748803	0,280110649	0,477485607	0,633016367	0,867527474
Cmah	4064,1735	-0,522038891	0,156525516	-3,335167984	0,000852479	0,021158908
Hrg	25395,50388	0,487322531	0,092091617	5,291714361	1,21E-07	2,64E-05
Rem2	3,333522462	2,23712328	1,016243417	2,20136558	0,027710154	NA
Acsbg1	1,691933625	0,952745677	1,180422673	0,807122482	0,419595921	NA
Lpcat4	20,81491693	0,889581537	0,368440444	2,414451377	0,015758926	0,137790114
Nans	437,1651986	-0,614783264	0,13518689	-4,547654458	5,42E-06	0,00049002
Xlr	11,78826361	0,509575959	0,522982695	0,974364858	0,329875412	0,6866113
Ppp3ca	789,81215	0,175724498	0,13251497	1,326072801	0,184815584	0,531469036
Rad23a	1208,716141	0,335968021	0,102463757	3,278896165	0,00104214	0,024581593
Kyat3	5867,82036	-0,346496644	0,137276141	-2,52408496	0,011599989	0,115301303
Zfp799	108,7398435	-0,163269366	0,22389014	-0,729238748	0,465855633	0,781825754
Zfp708	109,8461476	-0,14136319	0,207283558	-0,681979757	0,495251751	0,80006558
Msmp	2,729467404	0,018444831	1,138278786	0,016204142	0,987071531	NA
Trpc3	5,444524684	-0,871658168	0,678903694	-1,283920202	0,199169878	0,549010742
Myrip	1,720463986	1,386246624	1,28897839	1,075461492	0,282168164	NA
Zdhhc4	415,3847086	0,066750602	0,090467129	0,737843709	0,460609429	0,780953195
Bbof1	17,65398374	-0,297473042	0,362217243	-0,821255883	0,411500532	0,748947472
Pfn4	15,27547256	0,637386735	0,393389172	1,620244737	0,105179715	0,403011718
Apol7b	0,353964036	1,923250882	3,12828742	0,614793535	0,538691079	NA
Exosc6	124,0414869	0,087799943	0,195539923	0,449012875	0,653422369	0,878038361
Plxnb2	4901,277594	-0,083207203	0,09921618	-0,838645499	0,401668271	0,74210933
Snx29	68,39838516	-0,592425411	0,230356847	-2,571772524	0,010117935	0,105429984
Batf3	16,79701807	0,503244291	0,409877428	1,227792157	0,219525001	0,574490867
Ppil3	262,7140218	0,085681341	0,110519194	0,775262092	0,438184804	0,766215379
Ehbp1	217,4257883	-0,044506335	0,152063241	-0,292683062	0,769764418	0,927934035
Mybpc2	0,259430135	1,627812584	3,345388605	0,486584005	0,626553161	NA
Atp6ap1	1846,78629	-0,19612514	0,106486396	-1,841785873	0,065506477	0,312991971
Tyk2	658,9501605	0,003604687	0,090539377	0,039813469	0,968241838	0,991231429
Cyp51	5962,337473	0,642857544	0,179580447	3,579774712	0,000343891	0,011186065
Gabarapl1	8087,418755	0,241470241	0,154669377	1,561202646	0,118475945	0,427504699
Arc	3,083285541	-0,635431351	0,824949572	-0,770266902	0,441141586	NA
Foxo4	607,4682572	-0,205703081	0,104255378	-1,973069248	0,048487673	0,263723233
Extl3	641,5282113	-0,120123641	0,095904775	-1,252530351	0,210376676	0,562962817
Prpf40b	337,8878669	-0,090206269	0,137054434	-0,658178412	0,510423495	0,808704872
St3gal6	46,88535137	0,563337284	0,433004934	1,300995067	0,193260143	0,541742344
Msgn1	0,113662148	0,780932884	3,352475198	0,232942181	0,815806304	NA
Arl2	87,73633971	-0,257763337	0,171282885	-1,504898384	0,132350204	0,452967915
Fmn2	11,54233551	0,99097484	0,523288659	1,893744157	0,058258977	0,291660398
Calcrl	144,1093048	-0,000626193	0,314076885	-0,001993758	0,998409212	0,999803624
Sfrp5	0,992528973	0,977557165	1,866790464	0,523656609	0,6005174	NA
Pde3a	15,14510167	0,141346891	0,438841995	0,322090621	0,747384043	0,91954512
Sfn	18,87202369	0,300204771	0,355010042	0,845623322	0,397762911	0,738978927
Akr1e1	1147,406516	0,065070255	0,090143944	0,721848328	0,470387733	0,784744314
Phc2	690,7432061	0,05328174	0,08730109	0,610321585	0,541648801	0,826444056
Gabbr1	66,38961087	0,278849464	0,298680872	0,933603356	0,350508533	0,702050628
Sult3a2	25,53727701	20,74503938	3,227433587	6,427719988	NA	NA
Cyp4f17	276,8735776	-0,170047178	0,192868289	-0,881675154	0,377952501	0,723903192
Prrt4	0,213661134	0,059593471	3,352475198	0,017775962	0,985817581	NA
Sirt2	1314,40131	-0,034187405	0,083651906	-0,408686499	0,682769743	0,891903322
Jade3	87,59111652	0,195649223	0,20543231	0,95237805	0,340905288	0,694866457
1700001C19Rik	326,5297194	0,231744571	0,173573599	1,33513721	0,181831443	0,526302748

Supplementary Table S1: *Serpina1* KO vs. wildtype all DEGs

Aplf	88,92363625	-0,056833863	0,177621978	-0,319970896	0,748990393	0,919838327
Tprkb	3241,345663	0,220947932	0,102149659	2,162982578	0,03054252	0,202340409
Cdc14a	29,02691064	0,215400988	0,338742315	0,6358845	0,524851706	0,816659969
C5ar1	22,45511782	-0,128174037	0,359903647	-0,356134311	0,721739983	0,909525085
Mfsd4b3	349,873748	-0,162907721	0,146604441	-1,111205906	0,26647973	0,626660951
Hk3	114,2750642	0,228142733	0,272002307	0,838752935	0,401607967	0,742100471
Terb1	0,536472294	1,461149929	2,894232476	0,504848847	0,613665004	NA
Ndrg3	505,5046867	-0,109970712	0,098460046	-1,11690698	0,2640341	0,624866681
A130010J15Rik	178,2114516	-0,200818267	0,141427162	-1,419941287	0,155624775	0,490622294
Gtf2h5	648,0509849	0,081878416	0,116874625	0,700566237	0,483573755	0,794055997
Rasgef1b	229,661084	0,245106264	0,251188597	0,975785792	0,329170627	0,68631973
Lingo1	2,248053418	-0,391279059	1,06586204	-0,367101035	0,713543655	NA
Tlr4	57,15568367	0,214457016	0,249709498	0,858826028	0,390436506	0,734141441
Stap1	21,9550507	1,286912857	0,410445995	3,135401177	0,001716192	0,034605997
Atpaf1	161,964317	0,0624612	0,16269396	0,383918374	0,701038948	0,899833253
Gars	1345,201141	0,078926919	0,084312374	0,936124983	0,349208842	0,700954068
Flrt3	2,508768536	0,745581462	1,033822579	0,72118899	0,470793245	NA
Reep4	250,674969	0,125957136	0,155441218	0,810320052	0,417756254	0,752229411
Atad3a	1491,1102	0,184449835	0,08049734	2,291378019	0,02194156	0,165726956
Caln1	99,6041849	-0,387407149	0,177756322	-2,179428241	0,02929987	0,19731337
Il17rc	578,7975793	-0,075882947	0,10449068	-0,726217372	0,467705524	0,782859415
Pdrg1	377,3930946	0,091319298	0,098230056	0,929647212	0,352553775	0,70347633
Tmem56	3432,335093	-0,286205986	0,163526044	-1,750216537	0,080080956	0,350251568
Slc2a12	2,171554488	2,937588108	1,437898228	2,042973592	0,041055052	NA
Slc51b	45,29848162	0,756894189	0,358250142	2,112753356	0,034621884	0,217104886
Aoc2	20,95537125	-0,235611195	0,339721724	-0,693541737	0,487969643	0,796400974
Tnfrsf14	52,35546442	-0,06739716	0,313529443	-0,214962777	0,82979634	0,948328541
Kazald1	23,87768181	0,423488981	0,312130695	1,356768133	0,174854896	0,517287416
Gbf1	1930,134615	-0,590996486	0,151902567	-3,890628705	1,00E-04	0,004546589
Afap1l1	65,789658	-0,177125676	0,285655936	-0,6200665	0,535214006	0,822553871
Nsun3	122,3014655	0,012283964	0,201942635	0,060828977	0,951495413	0,985972877
Upk1b	9,55646478	2,087962219	0,647930511	3,222509486	0,00127073	0,028079973
Hic2	45,13187451	0,096118647	0,276613636	0,347483401	0,728228186	0,910983683
Mal2	1713,229059	-0,335579974	0,189549726	-1,770406005	0,07665953	0,342478335
Lmf2	753,8363817	-0,13217178	0,075553512	-1,74937969	0,080225409	0,350272207
Rfesd	222,0612024	0,243659029	0,113819935	2,14074125	0,03229491	0,208676004
Slc25a15	7881,843575	0,086481769	0,130033949	0,665070699	0,506005275	0,805958988
Mir7664	1,863835919	0,6777174	1,026809183	0,660022729	0,509239244	NA
Mctp2	405,2059364	-0,127730867	0,183186879	-0,697270829	0,48563332	0,79430833
Zfand2a	233,2395777	-0,031382658	0,144721439	-0,216848717	0,828326244	0,94762353
Chdh	5660,472003	-0,277817624	0,074755977	-3,716326593	0,00020214	0,007506703
Nr1h4	3061,504097	0,175369493	0,122029925	1,43710235	0,150688914	0,483107934
Abi3	99,49852588	0,104290638	0,225500192	0,462485807	0,643732978	0,873795284
Snap23	704,3684463	-0,258765459	0,137246053	-1,885412751	0,059374134	0,294723185
Plek	66,31994815	0,236090239	0,26429046	0,893298374	0,371697409	0,719235192
Crtap	151,0350271	-0,096192519	0,266408068	-0,361072093	0,718045555	0,907909407
Fam83h	369,9320072	0,206690019	0,12804173	1,614239503	0,106475486	0,40497035
Hexim1	1420,853521	-0,115144941	0,164292285	-0,700854217	0,483393999	0,794038919
Slc25a45	822,0987665	-0,096866729	0,095809257	-1,011037257	0,311998598	0,671577331
Mthfd1	6907,241342	0,233830168	0,085075881	2,748489528	0,005987055	0,076888506
Snupn	163,276445	0,045052442	0,141964044	0,317351079	0,750977223	0,920556312
Dhx33	491,8581034	-0,354768619	0,128448408	-2,761954203	0,005745654	0,074576354
Cxcr1	0,334254984	0,663952394	2,675156878	0,248191947	0,803985897	NA
Trim50	0,734074982	0,928139985	1,825374462	0,508465525	0,61112691	NA
Hist1h3b	1,380663855	1,539565609	1,6950778	0,908256606	0,363742664	NA
Fcrl1	12,87453042	0,299937016	0,574164448	0,522388693	0,601399727	0,854422974
Agfg2	1043,796076	-0,199538056	0,11741184	-1,699471339	0,08923041	0,370192045
Pik3ip1	11,39900805	0,584414537	0,413306211	1,413998924	0,157362218	0,493178253
Prkaa2	1506,706019	-0,239010609	0,131288169	-1,820503784	0,06868232	0,320580855
Arrb1	215,8495762	-0,069915921	0,232016935	-0,301339731	0,763155447	0,925576084
Edrf1	431,1193459	-0,189681862	0,112231926	-1,690088268	0,091011069	0,374323747
Trim62	25,52309765	0,254937754	0,285304507	0,893563712	0,371555369	0,719172395
4930486L24Rik	6,099460227	-0,806334604	0,781780539	-1,031407874	0,30234959	0,6622779
R3hdm4	886,0479286	0,044001794	0,104725324	0,420163839	0,67436577	0,888627551
Anapc7	482,1107532	0,150922829	0,093763508	1,609611585	0,107482678	0,406948716
Cd2bp2	715,5151822	-0,196379226	0,077842857	-2,522764888	0,011643622	0,115306358

Zfp688	73,73246678	0,073898293	0,242084797	0,305257885	0,760169749	0,924925298
Gorasp2	3377,540081	-0,200087468	0,093144107	-2,148149518	0,03170188	0,206641847
Mars2	54,01354407	0,039501073	0,287348086	0,137467675	0,890661142	0,968877905
Zfp397	411,9393943	0,052089307	0,131705064	0,395499654	0,692474177	0,895338108
Prcp	175,4626956	-0,165925715	0,152831428	-1,085677972	0,27762149	0,638252527
Tdp1	109,8379534	0,036367662	0,17342263	0,209705403	0,833897608	0,950814415
Ppp1r21	543,534811	-0,029761428	0,091275247	-0,326062423	0,744377127	0,918211645
Robo4	224,3653178	-0,079699861	0,265707183	-0,29995373	0,76421245	0,925934044
Neil1	140,6163729	-0,080546365	0,156323366	-0,5152548	0,60637498	0,856756194
Tmem135	2798,135083	0,192159648	0,118091104	1,627215276	0,103691385	0,400882047
Ttc39c	6973,675568	-0,55360906	0,415178836	-1,333423125	0,182392988	0,527224185
Susd3	3,138447921	-0,300762691	0,747308652	-0,402461138	0,687344683	NA
Tmx1	671,3844067	-0,094177978	0,145439445	-0,647540825	0,517281981	0,813424627
Zfp248	15,06390273	-0,405124081	0,379612564	-1,067204091	0,285879685	0,647454771
Angptl1	1,757329758	0,931010814	1,250102154	0,744747788	0,456424204	NA
Zfp618	14,02367429	0,046563517	0,413623432	0,11257466	0,910367776	0,9747592
Npl	21,25359546	-0,512327623	0,489657323	-1,046298297	0,295423332	0,656501749
Zfp518a	210,9180458	-0,109797067	0,149877145	-0,732580459	0,463814353	0,781663619
Dis3	205,8583415	-0,232957888	0,163351753	-1,426111954	0,153836029	0,488976193
Ccdc12	555,9223456	0,065632894	0,103599576	0,633524737	0,526391032	0,817627711
Sox8	0,086476712	0,780932884	3,352475198	0,232942181	0,815806304	NA
Tagln	151,0943827	-0,369978645	0,335469208	-1,102869163	0,270083987	0,630809369
Nsun6	68,37228684	0,366015599	0,210565069	1,738254122	0,082166046	0,354079608
Mtm1	203,5358082	0,020908318	0,164091	0,127419044	0,898608744	0,971764932
Zfp600	0,122496332	0,780932884	3,352475198	0,232942181	0,815806304	NA
Prdm11	2,884081546	0,448713347	0,951794503	0,471439313	0,637327047	NA
Tnfaip6	2,789915508	-0,83028905	1,022409143	-0,812090791	0,416739537	NA
Scfd1	1334,034014	-0,286456298	0,138186909	-2,07296263	0,038175758	0,22938054
Khdrbs3	373,3510771	0,009237712	0,134639161	0,068610882	0,945299357	0,984440501
Erf	504,7024531	0,246050413	0,173808027	1,415644706	0,156879555	0,492560538
Nr4a1	48,89883961	-0,747313866	0,482515111	-1,548788522	0,121432566	0,433611562
Plp2	30,89646041	0,407100669	0,329990608	1,233673504	0,217324591	0,571144576
Acot9	91,53863714	0,233222362	0,166759798	1,398552674	0,161947167	0,498634655
Snrpe	336,3064813	0,036005779	0,144465973	0,249233632	0,803180065	0,940189734
Snrpd1	430,6971833	-0,066852293	0,122639003	-0,545114456	0,585674801	0,847482024
Snrpb	1269,851067	-0,012552255	0,076901444	-0,163225218	0,870341111	0,961775826
Foxf2	0,093303375	-0,517475177	3,352475198	-0,154356154	0,877328924	NA
Gtf2h4	296,5125732	0,048766866	0,101752511	0,479269411	0,631746981	0,867216899
Gypa	0,79133316	-0,523315534	1,960804434	-0,266888184	0,789555255	NA
Snn	38,37794627	0,606755402	0,273358556	2,219632011	0,026443757	0,187447823
Evc	80,04622556	0,866999008	0,476478512	1,819597286	0,068820355	0,320987855
Stk32c	5,10615245	0,971048388	0,678548979	1,431066023	0,152411294	NA
Zbtb32	7,589654196	-0,201784951	0,77600523	-0,260030401	0,794840323	0,937443926
Nrip2	3,406315171	2,263147782	1,015791006	2,227965958	0,025882785	NA
Pnkp	817,5330892	-0,090505487	0,141928109	-0,63768543	0,523678472	0,81568929
Sertad2	361,6364956	-0,201662126	0,106044691	-1,901671115	0,057214164	0,289000033
Snrpb2	301,7699729	0,05996285	0,123808468	0,48431946	0,628159167	0,865852703
Sptbn2	1834,519683	0,053509229	0,151611959	0,352935414	0,724136861	0,910252319
Sez6	0,180974489	-1,204797453	3,342691436	-0,360427361	0,718527568	NA
Smoc2	212,1795167	0,684512049	0,287842743	2,378076452	0,017403217	0,145962115
Snord65	0,731374616	-2,172758714	1,931474337	-1,124922383	0,260621926	NA
Dph3	396,1937346	0,149582559	0,114850112	1,302415438	0,192774408	0,541552905
Acads	4011,962279	-0,107925508	0,107306141	-1,005771957	0,31452528	0,673320811
Acadm	7761,116563	0,16830827	0,076557671	2,198450778	0,027916999	0,192481874
Tusc3	144,8285944	0,001527241	0,156630982	0,009750566	0,992220297	0,997742998
Serpina6	6039,195204	0,52501332	0,206009718	2,548488126	0,010819097	0,110412601
Cirbp	402,8933654	0,600487808	0,21690528	2,76843333	0,005632651	0,073493174
Xndc1	40,1642592	-0,394102902	0,274441734	-1,43601666	0,150997601	0,483749562
Aurkc	1,063853396	0,11334666	1,39587939	0,081200898	0,935282185	NA
Atp7b	520,2183558	-0,207657987	0,150452541	-1,380222535	0,167518138	0,506611452
Atm	265,6091034	-0,050079433	0,133424421	-0,37533933	0,70740812	0,902731692
Gm6602	0,087021394	-0,517475177	3,352475198	-0,154356154	0,877328924	NA
Ccno	10,24024274	0,000235838	0,447155712	0,000527419	0,999579181	0,999875741
Sdr39u1	225,5802296	0,238157562	0,140666275	1,693067954	0,090442533	0,373071961
Zkscan4	20,12068682	0,247381597	0,49202472	0,502782862	0,615116944	0,859955845
A930005H10Rik	171,1851197	0,097514371	0,132953214	0,733448769	0,463284763	0,781663619

Lrfn1	5,142945299	0,191714472	0,735930703	0,260506147	0,794473375	NA
Cd59a	1414,60798	0,217354372	0,109812712	1,979318857	0,047780118	0,261149793
Cd33	11,76098879	-0,027459468	0,430328377	-0,063810498	0,949121119	0,985650844
Pttg1	705,2296679	0,051757058	0,101697538	0,50893128	0,610800393	0,858645358
Pcp2	0,093953095	-0,517475177	3,352475198	-0,154356154	0,877328924	NA
Sema6b	94,50368906	-0,359519878	0,207039524	-1,736479448	0,082479095	0,354896371
Cd151	981,8521678	0,090113549	0,080420718	1,12052654	0,262489449	0,622562722
Kifc1	10,39336174	-0,752974836	0,549537546	-1,370197252	0,170625336	0,510601034
Vav2	1003,208691	-0,126840912	0,088390172	-1,435011486	0,151283825	0,484052596
Gsg2	11,20217283	-0,375703426	0,541394711	-0,693954741	0,487710592	0,796312031
Hr	51,81418043	-0,606927357	0,354509357	-1,712020697	0,086892854	0,365765293
Fign	107,3554958	0,182382514	0,20427403	0,892832601	0,371946825	0,719340958
Wbp1l	5489,181801	-0,019670112	0,072937393	-0,269684884	0,78740269	0,934424153
Mal	10,93260178	0,948053618	0,487941792	1,942964579	0,052020438	0,274994558
Syncrip	949,3363398	-0,029027786	0,148769971	-0,195118586	0,845300113	0,953253244
Flot2	1050,458132	0,097501004	0,149195125	0,653513335	0,513425395	0,811058031
Usp4	2021,535628	0,050124839	0,092146073	0,543971522	0,58646107	0,84825252
C2	3624,170115	-0,351466701	0,154371973	-2,276752024	0,022801038	0,169855937
Apoa2	165241,1207	0,229840409	0,110204352	2,085583787	0,037016341	0,22555673
Cbr2	1,017281113	-0,004540541	2,136700114	-0,002125025	0,998304477	NA
Fam13b	480,48419	-0,140663424	0,140266073	-1,002832835	0,315941523	0,67419376
Fendrr	62,95748606	0,772252516	0,296727222	2,602567139	0,00925287	0,100082212
Map1lc3a	675,8918688	-0,236950676	0,136809697	-1,731972816	0,083278403	0,357192041
4930479D17Rik	0,249136072	1,389394708	3,349408814	0,414817893	0,67827522	NA
Cluh	7388,712673	-0,049423669	0,070256591	-0,703473769	0,481760548	0,793477208
Myo18b	5,18273682	-0,132847771	0,669459449	-0,198440356	0,842700546	NA
Scin	0,256296747	0,059593471	3,352475198	0,017775962	0,985817581	NA
Mesp2	5,199404362	-0,041653624	0,655118599	-0,063581807	0,949303218	NA
Snhg8	282,1187478	-0,022148255	0,146088393	-0,151608587	0,87949566	0,965356495
Pard6b	186,4027767	-0,176208328	0,18001476	-0,97885489	0,327651684	0,685077831
Ush2a	1,149353868	-0,372215163	1,656511776	-0,224698169	0,822214089	NA
Trem1	0,603624251	-1,992430547	1,944324567	-1,024741744	0,305485061	NA
Adal	368,7853905	-0,074595809	0,105347977	-0,708089626	0,478889602	0,79118575
F10	12636,90736	0,109198821	0,068136619	1,602645122	0,109013026	0,40910239
Lrp5	2245,131788	-0,058464573	0,133353817	-0,438416947	0,661084063	0,882666274
H3f3b	4045,823269	0,248544422	0,072675954	3,419898974	0,000626444	0,01716174
Epn2	188,676097	-0,048682766	0,170227287	-0,285986852	0,774888192	0,929351416
Chp1	6257,184141	0,027548921	0,088141693	0,312552665	0,754620546	0,922336997
Rcbtb1	1534,074086	-0,080215094	0,128591186	-0,623799319	0,532759387	0,820654481
Rab9	1600,639577	0,180623798	0,086660061	2,0842796	0,037134742	0,225969214
Fam3b	1,215006743	0,044640924	1,290214292	0,03459962	0,972399005	NA
Pf4	14,21281692	0,16877397	0,414227173	0,407443018	0,683682637	0,892293506
Kcnd3	1,165456382	2,825427657	1,546981808	1,826412982	0,067788078	NA
Rxfp1	0,093303375	-0,517475177	3,352475198	-0,154356154	0,877328924	NA
Bex4	11,49737408	0,904592699	0,703898329	1,285118406	0,198750911	0,548812381
Fam53b	256,4077224	0,381391482	0,249365742	1,529446181	0,126153871	0,441736801
Nudt9	961,7263387	0,065825019	0,096313537	0,683445138	0,494325606	0,799621608
Kdelc2	228,9897371	-0,192852329	0,148655077	-1,297314105	0,194523133	0,543617834
Col8a2	9,163524932	0,524799133	0,710461389	0,738673686	0,460105168	0,780953195
Wiz	501,1954419	-0,017124033	0,108482094	-0,157851238	0,874574023	0,963600184
Fbxo3	2736,467154	-0,100335613	0,075977513	-1,320596176	0,186636049	0,533163472
C130026I21Rik	0,912068054	1,513041209	1,595178174	0,948509222	0,34287028	NA
Pelo	441,9535457	-0,234041437	0,173641466	-1,347843011	0,177708881	0,52020802
Usb1	252,1635569	0,01592581	0,141179528	0,112805378	0,910184855	0,9747592
Sf3b3	902,811087	0,07172651	0,079179447	0,905872832	0,365003165	0,713569421
Mlec	3490,349468	-0,734638579	0,129501076	-5,672837651	1,40E-08	4,69E-06
Fbxw17	22,62679638	-1,28E-05	0,460885602	-2,77E-05	0,999977902	0,999996918
Pisd	830,2007802	-0,063306522	0,104619132	-0,605114193	0,545103124	0,827491596
Lsamp	3,347440653	-0,310124293	0,789669326	-0,392726781	0,694521288	NA
Alkbh2	100,7237198	-0,138060681	0,217329707	-0,635259129	0,525259425	0,816792829
Rab11fip4	79,75476842	-0,484485023	0,331370458	-1,462064619	0,143723503	0,470595031
Dmrta1	3,953664156	1,147253132	1,186321419	0,967067705	0,333510189	NA
A2m	3,636041339	-1,897240576	1,075254378	-1,764457429	0,077654997	NA
Mir1929	0,142634598	-0,517475177	3,352475198	-0,154356154	0,877328924	NA
Cript	624,7460612	0,00011485	0,088330736	0,001300222	0,998962573	0,999803624
Adgre4	130,3951108	0,108176344	0,22991803	0,470499612	0,637998112	0,870273408

Supplementary Table S1: *Serpina1* KO vs. wildtype all DEGs

Cchcr1	72,96965396	0,264698341	0,18973211	1,395116205	0,1629808	0,49993802
Mettl22	210,6245343	0,062651211	0,175267136	0,357461258	0,720746525	0,909110321
Rps6kl1	27,25934451	0,337747063	0,403703901	0,836620756	0,402805775	0,742671411
Actr2	2051,540822	-0,037733575	0,085191948	-0,442924194	0,657820567	0,880849675
Cdk17	404,7415822	-0,313844917	0,102903753	-3,049887959	0,002289268	0,041583176
Rassf9	5,346914335	0,172473992	0,724668717	0,238003915	0,811878058	NA
Hdgfrp2	843,467883	-0,127771487	0,085879598	-1,48779791	0,136804197	0,459416054
Tceal1	24,3815333	0,146383137	0,345789999	0,423329586	0,672054811	0,887904019
Mmgt1	598,419381	-0,311407488	0,148652339	-2,094871091	0,036182453	0,222803109
Slc22a26	456,4855288	5,150791541	1,295209361	3,976802281	6,98E-05	0,00343309
Dync1li1	279,7858839	0,023200075	0,13159692	0,17629649	0,860061019	0,959267413
Acaa1b	22112,14939	-0,343966351	0,214105911	-1,606524308	0,108158758	0,408238928
Apeh	1496,861446	0,021263262	0,127742092	0,166454624	0,86779919	0,961775826
Msantd2	98,10009309	0,300412618	0,18220542	1,648757857	0,099197252	0,392602307
Klhl36	112,5188076	-0,139513849	0,152959547	-0,912096378	0,361717985	0,71109038
Rfwd3	425,8648832	-0,096181321	0,129454999	-0,742971083	0,457499188	0,778822589
Aars	7033,511182	0,069971873	0,13770861	0,508115456	0,611372376	0,858645358
Vac14	657,0577404	-0,195763169	0,146509571	-1,336180072	0,181490423	0,525785165
Cmtr2	68,93639425	0,196626382	0,213117729	0,922618607	0,356205995	0,706231344
Tat	45090,78848	-0,345068784	0,253144731	-1,363128448	0,172842033	0,514752759
Ces4a	98,8750855	-2,287800671	0,431162687	-5,306119335	1,12E-07	2,50E-05
Dmrt3	0,235937973	-1,303449117	3,335546383	-0,390775294	0,695963339	NA
Apmap	961,1712345	-0,047285237	0,077266958	-0,611972294	0,540556088	0,826407188
Ifih1	865,3294952	0,164669316	0,143940339	1,144010894	0,252619119	0,612043137
9130008F23Rik	6,375741613	1,708451925	0,708637014	2,410898516	0,015913276	0,138415473
Izumo4	228,7063508	0,804242804	0,19627496	4,097531365	4,18E-05	0,002335825
Zfp768	531,5285792	0,012498713	0,125068142	0,099935227	0,920395749	0,977341679
Zfp553	164,2499413	0,159355453	0,178439492	0,893050362	0,371830203	0,7192878
Lrrk1	312,5778368	-0,03544799	0,155908848	-0,227363557	0,820141056	0,946381744
Eif3c	3283,314905	-0,094411927	0,077622445	-1,21629675	0,223871899	0,5790477
Yy2	12,62407041	0,239155768	0,55486874	0,43101323	0,666458751	0,885184864
Atxn1l	458,090125	0,023851295	0,118583815	0,201134486	0,840593417	0,951708907
Coq10b	372,1274252	-0,825199197	0,199229361	-4,141955751	3,44E-05	0,002028064
Clip3	14,60244445	0,024555005	0,371562831	0,066085742	0,947309562	0,985164724
Gm5936	0,236587694	-1,308576888	3,335186821	-0,392354899	0,694796005	NA
Nup62cl	7,035567687	-1,061278135	0,637223109	-1,665473395	0,095818347	0,385586553
Ppp1r10	806,3907258	0,323816032	0,261182748	1,23980636	0,215047025	0,568551815
Dgki	0,295445946	1,389394708	3,349408814	0,414817893	0,67827522	NA
Ada	29,2672504	-0,224526051	0,37078686	-0,605539395	0,544820657	0,827491596
Aplnr	0,614719626	0,785209023	2,078880607	0,377707609	0,705647815	NA
Tmed2	1434,991352	-0,155453051	0,100403253	-1,548286999	0,121553213	0,433672149
Morf4l2	2452,862796	-0,452286554	0,128301735	-3,525178795	0,000423197	0,013006256
Il21r	12,12001849	0,597608724	0,522349903	1,144077409	0,252591535	0,612043137
Clip1	849,6846638	-0,262942874	0,09242129	-2,845046565	0,004440493	0,063221462
Gsk3b	664,4714841	-0,223575128	0,098010814	-2,281127144	0,022540924	0,168540055
Spen	767,8560166	-0,197286955	0,16260062	-1,213322284	0,225006626	0,580187941
Ces1g	7608,515632	0,578505179	0,14501707	3,989221245	6,63E-05	0,003282365
Serinc1	3901,224143	-0,044712509	0,139745835	-0,319955935	0,749001734	0,919838327
Guf1	369,8626256	0,088675419	0,119350104	0,742985683	0,457490348	0,778822589
Fzr1	328,00886	0,117406989	0,103860516	1,130429476	0,2582953	0,61943354
Herpud1	5691,031784	-0,461440881	0,173057993	-2,666394504	0,007666967	0,089207724
Htra2	350,9026027	-0,060537727	0,102398575	-0,591196967	0,554388454	0,832018326
Piwil2	3,150276989	1,975447399	1,105176739	1,787449309	0,073864898	NA
Sae1	563,592787	-0,068510117	0,099223762	-0,690460786	0,489904462	0,796974051
Zfp113	166,5550173	0,50595271	0,20884316	2,422644398	0,015408001	0,136056515
Fkbpl	103,1578641	-0,200611855	0,163362016	-1,228020194	0,219439388	0,574443934
Pdcd10	427,3262473	0,038737306	0,120045441	0,322688688	0,74693102	0,91949547
Tdo2	31486,50207	-0,017616319	0,146509363	-0,120240229	0,904292851	0,973666426
Tmem115	543,2466961	-0,046405712	0,134882635	-0,344045113	0,73081236	0,911997614
Ak3	9722,714731	0,117504763	0,083545936	1,406468927	0,159584925	0,495312554
Foxo3	250,0729607	-0,22487558	0,241892572	-0,929650619	0,35255201	0,70347633
Foxo1	673,7712599	0,099178557	0,143207075	0,692553472	0,488589818	0,796400974
B4galt6	41,16431197	0,851417522	0,292576923	2,910063831	0,003613549	0,056043553
Nub1	948,0040523	0,048532752	0,070603644	0,687397272	0,491832416	0,79787739
Ccnl1	528,7193143	0,208690444	0,125149904	1,667523803	0,095410295	0,384691382
Ttc41	66,62396257	-0,149791492	0,278495513	-0,537859624	0,590673965	0,850006402

Supplementary Table S1: *Serpina1* KO vs. wildtype all DEGs

Fxyd7	5,956541705	0,018223627	0,869690218	0,020954159	0,983282224	0,995413661
Nme4	53,44087942	0,236857892	0,230833349	1,026099098	0,304844875	0,664100532
Nme3	338,2655558	0,187867372	0,122664274	1,531557363	0,125631701	0,441295377
Ruvbl1	395,837771	0,054213316	0,127812866	0,424161648	0,671447928	0,887716313
Rrs1	453,0183439	-0,035817343	0,242066966	-0,147964603	0,882370703	0,966166219
Mmp14	1305,78665	0,111330657	0,16328748	0,681807672	0,495360572	0,80006558
Slc22a21	4,838944855	0,790868942	0,650184108	1,21637692	0,223841372	NA
Ltbp1	61,23236577	0,097245224	0,316394534	0,307354312	0,7585737	0,9243607
Cyb561d2	198,5944702	-0,031776385	0,131142579	-0,242304099	0,808544538	0,942326905
Stub1	1345,548024	-0,057777489	0,107019191	-0,539879699	0,589279999	0,849389143
Arl3	354,9848813	0,072721324	0,132544721	0,548655005	0,58324223	0,846950064
Iqca	0,462289093	-1,558024498	2,945502192	-0,528950378	0,596839873	NA
Asb8	481,078454	0,123924835	0,119499005	1,037036536	0,29971883	0,659904651
Kbtbd11	18,47138991	0,318478561	0,43122192	0,738549101	0,460180841	0,780953195
Mrpl9	1020,215447	-0,054260905	0,101638856	-0,533859859	0,593438496	0,85161864
Ccdc181	121,5283973	0,149790692	0,155766058	0,961638839	0,336231054	0,69270916
Arhgap24	317,6559217	0,181320392	0,180416502	1,005010019	0,314892026	0,673776132
Fam151a	0,680837839	0,934257771	2,062221655	0,453034604	0,650523824	NA
Nrd1	2106,466496	-0,140477253	0,098188014	-1,430696557	0,152517201	0,486403257
Ndufa4l2	51,80039162	0,170172721	0,269181818	0,632185053	0,527265963	0,818305666
Hs3st1	4,214299731	0,95970347	0,837891989	1,1453785	0,252052401	NA
Nr1h2	893,6857478	0,130598892	0,094170961	1,386827634	0,165494328	0,503755391
Gstt2	1139,837956	0,70128268	0,188627635	3,717815147	0,000200953	0,007483408
Gstm5	260,3325079	0,117884517	0,139201617	0,846861692	0,397072217	0,738461552
S1pr2	158,533001	0,142202663	0,154743895	0,91895492	0,358119152	0,707401735
Tapbp	1966,474658	0,241580321	0,102926773	2,347108676	0,018919736	0,15301751
Il1r2	1,373625537	-0,228104064	1,40081401	-0,162836795	0,870646936	NA
Tfpi2	1891,753649	0,091521993	0,092852953	0,985665934	0,324297071	0,681472422
Fem1b	376,2035088	-0,13618782	0,103240446	-1,319132426	0,187124844	0,533633116
Anapc16	672,6198389	0,292862735	0,112689583	2,598844792	0,009353806	0,100523333
Tpst2	851,2829025	-0,3000273	0,152037716	-1,973374153	0,04845295	0,263641632
Gsto1	3071,333313	0,185020905	0,08840404	2,092901016	0,036357992	0,223480455
Igtp	891,3883389	0,573421367	0,139582226	4,108125954	3,99E-05	0,002269832
Caps2	0,541565944	-1,663506702	2,854024186	-0,582863562	0,559985152	NA
Cldn34b2	0,086476712	0,780932884	3,352475198	0,232942181	0,815806304	NA
Trim80	18,02086994	1,002388736	0,47451978	2,11242772	0,034649779	0,217104886
Sep-01	31,40784704	0,373615569	0,298907554	1,249936861	0,211322613	0,564073259
Rfk	1019,883975	0,01803594	0,148296234	0,121621023	0,903199163	0,973489035
Sit1	0,443124003	1,189880456	2,510312651	0,473996917	0,635502106	NA
Ndufb11	3234,269894	0,002745398	0,112235053	0,024461144	0,980484777	0,994739255
Nfkbid	11,78840322	-0,417383375	0,457105041	-0,913101668	0,361189073	0,710630864
Dmxl2	631,4968386	0,022399548	0,197245576	0,113561725	0,90958523	0,9747592
Slc17a5	585,6320598	-0,177417416	0,130605901	-1,358418067	0,174331065	0,517113823
Mapkap1	988,7393245	0,077203231	0,079770369	0,967818393	0,333135076	0,68995097
Tmem258	878,3441159	-0,206440073	0,110700979	-1,864844159	0,062203252	0,302651918
Sirt6	72,87472282	-0,044656895	0,222222801	-0,200955502	0,840733368	0,951715868
Papd5	749,5353518	-0,213991689	0,132276327	-1,617762562	0,105713778	0,403240888
Rexo2	1995,608527	-0,138866184	0,103729309	-1,338736226	0,180656559	0,524131409
Pus3	131,2729393	0,03436431	0,150324588	0,228600724	0,819179262	0,945979749
Otoa	0,187906191	-1,241948083	3,339949325	-0,371846385	0,710007226	NA
Fktn	122,6339362	-0,280098447	0,21455694	-1,305473722	0,19173159	0,541080647
Coasy	3207,004943	0,03341973	0,073014246	0,457715196	0,647157069	0,876230372
Tnni1	0,093953095	-0,517475177	3,352475198	-0,154356154	0,877328924	NA
6330407A03Rik	0,295445946	1,389394708	3,349408814	0,414817893	0,67827522	NA
Prdx6	12421,35154	0,170077621	0,097711486	1,74061032	0,081751907	0,353542587
Reep5	400,1249811	-0,369963125	0,126599506	-2,922310959	0,003474445	0,054775767
Sertad4	7,455876289	-0,36607125	0,589728581	-0,620745309	0,534767213	0,822042413
Acp1	1156,041777	-0,046117871	0,086514266	-0,533066665	0,593987433	0,851766384
Rangrf	45,71400763	-0,208103042	0,254084136	-0,819032013	0,412768149	0,74954611
Cdc34	1686,034939	-0,15489798	0,139277324	-1,11215505	0,266071491	0,626141482
Tdh	0,26680148	-0,62623849	2,8346967	-0,220919046	0,825155467	NA
Lat2	13,60004237	0,301272971	0,440594321	0,683787685	0,494109244	0,799533586
Slc44a4	7,938934154	0,80788256	0,598533602	1,349769766	0,177089846	0,520079413
Akr1a1	10764,73241	0,010177908	0,077198823	0,131840204	0,895110686	0,970433882
Rnf32	8,184396064	0,233390419	0,479762848	0,486470388	0,626633695	0,865351294
Reps1	450,9468778	0,328458052	0,154691529	2,12330988	0,033727893	0,214383309

Supplementary Table S1: *Serpina1* KO vs. wildtype all DEGs

4930426D05Rik	0,493581368	2,385382152	2,875757806	0,829479502	0,406833131	NA
Phc1	63,08605567	-0,419866371	0,245418728	-1,710816345	0,087115021	0,366110999
Sec1	0,174042788	-1,166820872	3,345560292	-0,348766954	0,727264274	NA
Wfdc18	2,300184691	0,203583045	1,024571342	0,198700702	0,842496875	NA
Ewsr1	2346,234051	0,046213163	0,066309829	0,696927807	0,485847974	0,794337969
H2-Bl	151,6002392	0,615644315	0,870930996	0,706880704	0,479640617	0,791837124
Gabra3	45,55478671	0,372166543	0,302636884	1,229746151	0,218792179	0,573423375
Fkbp4	5820,169924	-0,431729987	0,11537541	-3,741958436	0,000182592	0,007075561
Inpp1	159,9075454	0,013264172	0,164919699	0,080428062	0,935896809	0,982181054
Zmym3	395,8525417	0,169165041	0,130850816	1,292808453	0,196077316	0,544980798
Aldh9a1	7311,77713	0,223420119	0,147129423	1,518527802	0,128881396	0,446309134
Cftr	5,885187557	1,793314692	0,777467362	2,306610901	0,021076521	0,162193129
Gltp	126,6461949	-0,003598011	0,25842876	-0,013922641	0,988891698	0,996575327
Fzd3	5,880863889	-0,201002851	0,920740171	-0,218305725	0,827190914	0,947537943
Syt9	27,26200481	0,224214428	0,271096671	0,827064483	0,408200514	0,746892025
Ackr2	63,10333129	0,522751674	0,247225568	2,114472539	0,034474928	0,216957051
Mir6904	0,339756192	1,876228608	3,160061454	0,593731684	0,552691588	NA
Trmt2a	380,9856518	-0,077119265	0,148079381	-0,520796784	0,602508343	0,854608409
Ecd	730,1993354	-0,131522667	0,090308432	-1,456371944	0,145289827	0,473982357
Acad10	637,3089484	0,127758455	0,129151012	0,989217608	0,322556685	0,67984555
Brox	549,8482948	0,020883528	0,110187509	0,189527178	0,849679656	0,954743122
Rnf215	276,1341381	-0,070886088	0,110204152	-0,643225202	0,520077983	0,814008287
Ddx54	1071,726628	-0,077739854	0,081625894	-0,952392074	0,340898177	0,694866457
Lrrc34	0,249136072	1,389394708	3,349408814	0,414817893	0,67827522	NA
Prss8	300,9189492	0,018806617	0,235447437	0,079876072	0,936335821	0,982191945
Cacng7	8,848307729	-0,259592395	0,667918832	-0,388658595	0,697528715	0,898300876
Man1c1	61,11316403	0,079465575	0,264782362	0,300116573	0,764088238	0,925867457
Tsen2	153,891738	0,16220288	0,13943967	1,16324774	0,244729002	0,603538467
Ptpdc1	39,83010516	0,473569148	0,279619286	1,69362119	0,090337289	0,372867926
Arl5c	9,82394598	0,754968922	0,572751503	1,318143939	0,187455468	0,533818545
4930528A17Rik	2,190796654	0,023184551	0,920205399	0,025194974	0,979899446	NA
Zfp942	307,9202581	0,052307301	0,148795041	0,351539275	0,725183813	0,910252319
Fjx1	1,50767171	-0,217215322	1,535769694	-0,141437432	0,887524385	NA
Mast1	22,83948133	-0,235931884	0,322784426	-0,730927098	0,464823683	0,781663619
Tbk1	672,9798901	0,232343465	0,093433266	2,486731696	0,012892259	0,121979199
Slc5a1	39,52682049	0,511613961	0,415027342	1,232723508	0,217678938	0,571609443
Zadh2	2298,577548	0,132837077	0,093115832	1,426578855	0,153701323	0,488800701
Fads1	15344,85389	0,155200425	0,127680242	1,215539874	0,224160251	0,579427376
Tmem82	374,6038021	0,071704853	0,128063174	0,55991782	0,575535494	0,843861602
Csnk1a1	1584,745259	0,113723412	0,10799477	1,05304555	0,292320116	0,654063202
Fam83f	102,024967	0,375074425	0,319039174	1,175637525	0,239739754	0,597294217
Arcn1	4023,392996	-0,247927238	0,122781382	-2,019257585	0,043460454	0,24557177
Haus1	121,472462	0,075960586	0,175349944	0,43319424	0,664873663	0,884623407
Zc3hav1l	6,745211755	-0,072074943	0,602409972	-0,119644339	0,904764894	0,973666426
Klhl11	19,4031977	-0,241492943	0,344621337	-0,700748669	0,483459878	0,794038919
Rufy1	544,3722802	-0,066937212	0,086205099	-0,776487848	0,437460993	0,766027825
Sned1	122,1417067	-0,231079296	0,240607229	-0,960400472	0,336853701	0,69270916
Zfp11	37,82208997	-0,401891	0,271761896	-1,478834987	0,139184422	0,463913374
Nek11	0,890456519	0,71187957	1,689150885	0,421442262	0,673432164	NA
Mob3a	148,7852379	0,285909535	0,178893876	1,598207506	0,109996804	0,41054197
Kat7	585,689984	-0,065693879	0,104295411	-0,629882737	0,528771308	0,81885134
Nags	2143,73247	0,249617886	0,113266777	2,203804977	0,027538062	0,191150756
Xylt2	463,9183721	-0,029991691	0,127078148	-0,23600982	0,81342505	0,943900659
Nlrp3	22,74887496	0,362477585	0,350478803	1,034235401	0,301026128	0,661475725
Pcdhb15	0,883649844	-1,769600557	1,701517069	-1,040013403	0,298333674	NA
Lrp4	319,4198767	-0,423209564	0,163431761	-2,589518473	0,009611027	0,10216528
Zkscan16	0,240301888	1,389394708	3,349408814	0,414817893	0,67827522	NA
Parp1	1153,706003	-0,203170877	0,095647311	-2,124167158	0,033656168	0,214261579
Anks1	379,976118	-0,180490483	0,111893102	-1,613061743	0,106731094	0,405365909
Platr9	0,147722973	0,780932884	3,352475198	0,232942181	0,815806304	NA
Dazl	0,601837237	-0,963048427	2,28796686	-0,420918871	0,673814325	NA
Pax5	2,946661259	-0,207772924	0,906726631	-0,229146159	0,818755318	NA
Casq2	2,652911728	1,021861876	1,160341542	0,880656116	0,378503976	NA
Alox5ap	52,05143109	0,306560252	0,252027749	1,216374997	0,223842104	0,5790477
Mir6935	0,733741821	-0,217711884	1,909954468	-0,113987997	0,909247309	NA
Ssb	1666,088872	-0,02428392	0,091633056	-0,265012668	0,790999703	0,936078165

Supplementary Table S1: *Serpina1* KO vs. wildtype all DEGs

Marveld2	291,247609	0,128016868	0,137148092	0,933420702	0,350602795	0,702050628
Cox8b	0,756242125	2,11185558	2,230664258	0,946738431	0,343772082	NA
Ccr10	0,113662148	0,780932884	3,352475198	0,232942181	0,815806304	NA
Mrps7	1551,324352	-0,018342766	0,108701456	-0,168744437	0,865997664	0,961775826
Btd	2181,375648	0,187779322	0,070951931	2,646570981	0,008131244	0,092812169
Lmo7	468,2166962	0,13086838	0,123342746	1,061013995	0,288683541	0,649821721
Mir148a	0,410630482	-2,238019211	2,443059499	-0,916072332	0,359628959	NA
Myom3	22,57653784	2,14912105	0,608880131	3,529629135	0,000416143	0,012865677
Adgrf5	492,9874077	-0,036196501	0,273629258	-0,132283007	0,894760447	0,970433882
Trappc10	551,2994907	-0,348455769	0,098272472	-3,545812614	0,000391404	0,012370416
Qser1	470,690045	-0,076291367	0,133993489	-0,569366226	0,569107632	0,840790976
Nme2	5470,007212	-0,109558838	0,145022288	-0,755462073	0,449971781	0,773622955
Ssc4d	78,78303895	-0,088239173	0,212595992	-0,415055676	0,678101146	0,89063303
Gm8369	0,812318505	0,295917917	1,778358796	0,166399446	0,86784261	NA
Cers3	0,995832975	0,44355481	1,630349165	0,272061237	0,785574934	NA
Tmem74b	0,093953095	-0,517475177	3,352475198	-0,154356154	0,877328924	NA
Kmo	6949,519864	0,027744998	0,113522966	0,24439987	0,806921142	0,941758461
Hdlbp	12864,03541	-0,246504597	0,118746677	-2,075886272	0,037904471	0,228675482
Exd2	281,1611027	0,039734279	0,116885443	0,339942065	0,733900158	0,913675851
Lrrc59	2319,601178	-0,318524459	0,140458537	-2,267747236	0,023344617	0,172713993
Arhgdia	2443,97655	-0,131257769	0,115612435	-1,135325707	0,256238875	0,616816726
Uap1	881,6262474	-0,45440057	0,102003235	-4,454766242	8,40E-06	0,000710627
Cops8	611,7152086	-0,072512974	0,085650768	-0,84661207	0,397211384	0,738461552
Ttc1	387,3996151	0,129626858	0,106477463	1,217411217	0,223447797	0,578565749
Tex19.2	0,142634598	-0,517475177	3,352475198	-0,154356154	0,877328924	NA
Rbpms2	1493,057441	-0,100247397	0,114733026	-0,8737449	0,382257193	0,726783025
Gm26688	0,086476712	0,780932884	3,352475198	0,232942181	0,815806304	NA
Gm3893	2,347485041	-0,034242955	1,0883584	-0,03146294	0,974900347	NA
Tekt5	0,627602016	2,761373889	2,660103617	1,038070048	0,299237445	NA
Sdf4	3073,359244	-0,003328025	0,061902267	-0,053762565	0,957124335	0,98781716
Shroom4	12,29906772	-0,012200556	0,399241844	-0,030559311	0,975620992	0,993304169
Cpt2	4017,27308	-0,218042394	0,119774191	-1,820445557	0,06869118	0,320580855
Psme1	2341,216085	0,109386841	0,117483395	0,931083418	0,351810415	0,702880198
Cry2	640,7476519	-0,305749832	0,179702927	-1,701418206	0,088864485	0,369526998
Padi4	1,907315269	0,742630634	1,132346735	0,655833245	0,511931423	NA
Stc2	1,298618226	-0,296063131	1,228600225	-0,24097597	0,809573741	NA
Nhp2l1	493,0756048	-0,167344378	0,133929911	-1,24949219	0,211485108	0,564073259
Padi3	0,093303375	-0,517475177	3,352475198	-0,154356154	0,877328924	NA
Ptgfrn	89,93572088	-0,059326323	0,222515875	-0,266616139	0,789764728	0,935361858
Pdgfb	33,95956404	0,355064347	0,397518698	0,893201623	0,371749209	0,719235192
Zc3hav1	1344,769353	-0,003136914	0,095043502	-0,033005032	0,973670575	0,992775209
Pde4d	148,485976	0,248599411	0,163567111	1,519861845	0,128545702	0,445907495
Dpysl2	57,72756574	0,317813273	0,324516271	0,979344647	0,327409717	0,685071653
Pde3b	1824,356116	-0,168884825	0,148282431	-1,138940225	0,254728087	0,615036986
Bcar1	885,3413332	-0,229676222	0,117445028	-1,955606168	0,050511566	0,270223739
Ncam2	49,12337984	0,099498624	0,449558623	0,221325136	0,824839279	0,947537943
Vbp1	238,2657212	0,142932155	0,170579284	0,837922118	0,402074445	0,742449346
Tnfrsf17	0,093953095	-0,517475177	3,352475198	-0,154356154	0,877328924	NA
Srms	45,64631771	-0,331862778	0,310234446	-1,069716087	0,284747123	0,645984098
Tnfsf12	142,8428864	-0,132096544	0,164379058	-0,803609323	0,421622637	0,755181275
Oaz2	494,2609958	0,052524382	0,109325735	0,480439317	0,630915043	0,866751905
Nr1d2	454,9061002	0,480511083	0,292235313	1,644260843	0,100122334	0,392993389
Spic	42,37441432	0,054424962	0,322367539	0,168828916	0,865931213	0,961775826
Tgfb1	157,4391106	-0,159652053	0,203841323	-0,783217308	0,433499513	0,763329011
Tk1	734,2700197	-0,361002395	0,155736243	-2,318037133	0,020447303	0,159672894
Degs2	2,446148836	1,730204032	1,031568784	1,677255126	0,093492603	NA
Lysmd2	11,6257186	-0,033384228	0,679189388	-0,049153047	0,960797329	0,988470616
Gphb5	0,093953095	-0,517475177	3,352475198	-0,154356154	0,877328924	NA
Trmt10b	105,2544142	-0,012131033	0,161986688	-0,074889076	0,940302968	0,983128178
Zfp715	235,3510735	0,105166143	0,1618908	0,649611605	0,515943135	0,813164632
Apitd1	5,389647148	-0,267143239	0,794870403	-0,336084018	0,73680751	0,914722813
Polr2i	603,2955333	-0,358651643	0,112509277	-3,187751725	0,001433836	0,030330621
Zfp169	59,17553682	0,315026821	0,239230091	1,316836105	0,187893568	0,534246008
Rps27	16,89304114	-0,075958688	0,39416425	-0,192708214	0,84718749	0,953824301
Smdt1	1516,942761	0,154096932	0,103930637	1,482690155	0,138156751	0,461607728
Mitd1	246,5515783	0,202606463	0,116980692	1,731964991	0,083279796	0,357192041

Supplementary Table S1: *Serpina1* KO vs. wildtype all DEGs

Rmrp	12,5330447	-0,461985952	0,547793914	-0,843357219	0,39902869	0,739993697
Adam5	0,113662148	0,780932884	3,352475198	0,232942181	0,815806304	NA
Barx1	0,642445966	1,001170347	2,340364431	0,427783953	0,668808421	NA
Syngap1	24,26381635	-0,009937416	0,321688317	-0,030891443	0,975356114	0,993185764
Bak1	320,1884119	0,242503454	0,132226095	1,834006018	0,066653101	0,316480771
Bad	377,8077414	-0,079197645	0,105856926	-0,748157415	0,454365214	0,776572096
Bach1	655,0345844	-0,405144665	0,157243341	-2,576545766	0,009979299	0,104553371
Baat	7623,204285	0,073676334	0,093089706	0,791455214	0,428678401	0,760685099
Hnrnpd	938,2395828	0,30539233	0,076425572	3,995944292	6,44E-05	0,003238559
Slc7a3	0,607243243	1,769179115	2,305625378	0,767331559	0,44288441	NA
Mr1	205,0199577	0,96983022	0,252082625	3,847271193	0,000119441	0,005167648
Slc7a1	24,95560197	1,056780476	0,362123517	2,91828734	0,003519599	0,055227128
Rasl2-9	16,86949333	0,131078153	0,432053133	0,303384337	0,761596981	0,92534041
Cep85l	184,0239319	-0,284826868	0,325543317	-0,874927706	0,381613243	0,72637014
Phf21b	9,376589452	0,092320276	0,555768296	0,166112887	0,868068113	0,961775826
Dennd3	121,2799543	-0,118260421	0,212220386	-0,557252879	0,577354666	0,84496439
Syt14	1,094811511	0,643957292	2,156629652	0,298594286	0,765249625	NA
Wsb1	575,6945535	0,004743762	0,202158481	0,023465558	0,981278912	0,994983586
Lmtk2	384,1036183	-0,294661429	0,116323373	-2,533123153	0,011305123	0,113783449
Pde4b	520,6304424	0,260561338	0,41643498	0,625695128	0,531514929	0,819884984
Agk	162,9303695	0,141096378	0,195077784	0,723282657	0,469506246	0,784211519
Purg	44,40067618	0,077817342	0,220854041	0,352347379	0,72457776	0,910252319
Edf1	4011,10743	-0,065301151	0,106170885	-0,615057045	0,538517049	0,825055515
Rcl1	2273,54968	0,071505799	0,118344561	0,604217031	0,545699359	0,827806052
Ldb3	1,812813355	-0,209399624	1,304104782	-0,160569631	0,872432375	NA
Trim37	338,3613786	-0,341973123	0,114651579	-2,982716203	0,002857028	0,047625442
Fbrs	584,0387555	-0,066563817	0,112120665	-0,593680179	0,552726042	0,83087907
Selenow	1991,121911	0,030901015	0,107087245	0,288559247	0,772918686	0,928572062
Selenop	147205,9627	0,016442527	0,101843582	0,161448829	0,871739911	0,962328069
Rac2	248,0059494	0,435114947	0,228202664	1,906704063	0,056558921	0,287361327
Spata2	231,7018805	0,075167123	0,151326275	0,496722218	0,619384952	0,861846105
Fam134a	3503,378738	-0,076652923	0,060048521	-1,276516425	0,201773024	0,553126746
Ly9	53,64692994	0,454920233	0,32218926	1,411965851	0,157960016	0,493749698
Nsf	457,1431041	-0,190087121	0,124686581	-1,524519472	0,127378996	0,444323237
Kcnq1	37,02765071	0,132205667	0,404025495	0,327221099	0,743500664	0,917384437
Ptgds	186,4987455	1,71603621	0,648383764	2,646636613	0,008129666	0,092812169
Slc50a1	267,0035643	0,03479953	0,13201166	0,263609517	0,792080825	0,936507892
Slc22a1	7702,153576	0,0029062	0,134268004	0,021644766	0,982731324	0,995413661
Rad23b	5729,744853	-0,04857576	0,11782048	-0,412286216	0,680129651	0,891524003
Mtf1	385,8137626	-0,140345785	0,108025013	-1,299197116	0,193876291	0,542585752
Kcnk4	0,632387994	0,830234442	1,84829874	0,449188448	0,65329572	NA
Lrp6	1629,258913	-0,067523477	0,115983607	-0,582181213	0,560444627	0,835469208
Snx24	333,7501065	-0,036287258	0,127952641	-0,283599135	0,7767176	0,930009104
Tax1bp3	132,8883208	-0,182519141	0,17213396	-1,060331969	0,2889936	0,650101875
N4bp1	476,6379881	-0,108540852	0,106671269	-1,01752658	0,308902994	0,66769994
Nedd8	1311,151443	-0,092099167	0,109685021	-0,839669507	0,401093716	0,741816472
Kcnj2	17,81842647	-1,261046452	0,458786383	-2,748657106	0,005983995	0,076888506
Syt4	0,300918618	-0,661123873	2,741506622	-0,241153484	0,809436161	NA
Sox18	115,16391	-0,119357364	0,359079699	-0,332397974	0,739588768	0,916067243
Snta1	454,3391165	0,062315533	0,124043751	0,502367372	0,615409126	0,859955845
Kcnb1	35,80355214	-0,260812385	0,425636518	-0,612758478	0,540036049	0,826407188
Kcna2	73,57885591	0,28277547	0,238615179	1,185069076	0,235990137	0,593259146
Jrk	24,88796281	0,572475173	0,290280053	1,972147816	0,048592735	0,264079785
Jak2	392,4162378	0,058195607	0,100652502	0,578183409	0,563140299	0,83722511
Ivl	0,093303375	-0,517475177	3,352475198	-0,154356154	0,877328924	NA
Myo5b	677,9512161	-0,083932991	0,155210058	-0,540770311	0,588665909	0,848961654
Amn1	109,1172235	0,082661565	0,155056382	0,533106496	0,593959863	0,851766384
Slain2	896,5520315	-0,16611852	0,120044573	-1,383806995	0,166417561	0,505052737
Chst7	17,35586353	-0,676965068	0,531956438	-1,272594933	0,203161818	0,55567332
Ptprd	2705,392197	0,062130013	0,139295952	0,446028846	0,655576409	0,879592859
Gm12718	47,47128006	-2,723129467	1,207699434	-2,254807272	0,024145429	0,176490014
Fbln1	22,7506851	0,883882394	0,41263758	2,142030773	0,032191006	0,208308055
Nr2f6	1938,899175	-0,081441008	0,143752537	-0,566536145	0,571029369	0,8413217
Dbt	2735,749359	0,008046776	0,109149772	0,073722331	0,94123133	0,983128178
Pcmt1	806,5852038	0,02223977	0,087484524	0,25421376	0,799330421	0,93892144
Mtnr1a	29,14907833	-0,897270291	0,360680243	-2,487716776	0,012856607	0,121814305

Supplementary Table S1: *Serpina1* KO vs. wildtype all DEGs

Rsg1	3,639093911	0,462252699	0,743852125	0,621430905	0,534316143	NA
Tsg101	894,8181856	-0,155738858	0,092802936	-1,678167365	0,093314431	0,380085036
Cdh13	41,15596778	-0,145491442	0,429753672	-0,33854613	0,734951671	0,913858791
Crbn	723,1380569	0,168383621	0,109052199	1,544064417	0,122572728	0,434777077
Cse1l	785,6673922	0,134289737	0,115170069	1,166012468	0,243609405	0,602221548
Fkbp11	369,8169723	-0,915404337	0,200177936	-4,572953215	4,81E-06	0,000449589
Plekhj1	356,1633346	-0,087317957	0,138803626	-0,629075478	0,529299643	0,818910651
Nol7	916,0032679	0,091862111	0,090679366	1,013043153	0,311039549	0,670475288
Ino80b	276,377813	-0,061877024	0,134018451	-0,461705262	0,644292697	0,874028317
Olfr267	0,087021394	-0,517475177	3,352475198	-0,154356154	0,877328924	NA
Blk	8,478789602	1,319864802	0,526331036	2,507670479	0,012152994	0,118076584
Bid	352,2354529	0,087729335	0,132636968	0,66142446	0,508340144	0,807413494
Bcl2l2	564,5021059	-0,116651154	0,112464706	-1,03722455	0,299631219	0,659904651
Mrto4	523,4165091	-0,02694385	0,137552853	-0,195879981	0,844704107	0,953132002
Fam3a	426,396578	-0,013491086	0,094811477	-0,142293802	0,886847942	0,968253992
Sema6d	313,6574761	-0,039008522	0,190575823	-0,204687675	0,83781615	0,951141594
Reps2	688,225171	-0,039379181	0,182163012	-0,216175507	0,828850945	0,947977439
Gapdhs	8,639142753	0,630283193	0,561089301	1,12332064	0,261301341	0,622343615
Vps54	1037,996749	-0,030092037	0,114759671	-0,262217874	0,793153474	0,936746676
Inafm1	92,98999848	0,242920666	0,168835464	1,438801187	0,15020686	0,482372211
Zfhx2	324,5183354	-0,443540089	0,221486676	-2,002558784	0,045224668	0,251865618
Gabbr2	546,6526829	-0,120160226	0,134621808	-0,892576231	0,372084153	0,719362695
Hdhd2	685,874148	-0,130711798	0,075337861	-1,735008088	0,082739373	0,355787286
Neurl2	54,43144059	0,61571797	0,348800714	1,765242861	0,077522958	0,345008131
Cd55	233,7888926	-0,252719099	0,254025616	-0,994856748	0,319805941	0,677465636
Ccm2	470,3920646	-0,194412053	0,12488214	-1,556764262	0,119526465	0,429902963
D17Wsu92e	5475,590347	-0,119209389	0,064240841	-1,855663588	0,063501524	0,306618538
Scube1	13,93032115	0,797858457	0,536580936	1,486930308	0,137033219	0,45983863
Gstz1	21722,61658	0,159232505	0,069503449	2,291001483	0,021963328	0,165797702
Alpl	513,4433642	-0,704577788	0,158803871	-4,436779675	9,13E-06	0,000765512
Otud4	689,2876776	-0,013071444	0,13991471	-0,093424374	0,925566427	0,978363356
Gbp4	116,8058735	0,357196819	0,244142028	1,463069764	0,14344829	0,470500538
Mroh8	0,95777267	-0,17865475	1,482132952	-0,12053895	0,904056227	NA
Anxa7	2356,23845	0,080503418	0,071314851	1,128845079	0,258963193	0,62033308
Ift81	82,87022347	-0,001081927	0,232370223	-0,004656048	0,996285024	0,999049992
Nck2	77,27598804	0,030000806	0,306246939	0,097962793	0,921961838	0,977341679
Ncl	4527,453998	-0,095968653	0,110701348	-0,866914945	0,385988614	0,730226156
Gm35135	0,093953095	-0,517475177	3,352475198	-0,154356154	0,877328924	NA
Cdkn2d	71,08124966	-0,039248384	0,214993878	-0,182555823	0,85514655	0,957445239
Zfp36l1	4950,1955	-0,534908445	0,139508086	-3,834246881	0,00012595	0,005295032
Bpgm	462,2593064	-0,110635396	0,112897179	-0,979965991	0,327102907	0,684930268
Bnc1	4,012712567	2,895948116	1,047232563	2,765334291	0,005686449	NA
Bmpr2	530,0526127	-0,128492401	0,142628691	-0,900887476	0,367648151	0,716004058
Gm5141	3,805428582	-0,038805974	0,732275041	-0,052993714	0,957736916	NA
Hrk	2,467221411	3,203842205	1,762404835	1,817880967	0,069082328	NA
Mcpt8	0,234199685	1,337107854	3,350237755	0,399108348	0,689813372	NA
H3f3a	1578,652848	-0,172490166	0,103578862	-1,665302774	0,095852365	0,385586553
Kcnd1	4,996738786	-0,029205045	0,663138009	-0,044040673	0,964871983	NA
Itih1	19089,75356	-0,11582826	0,096777088	-1,196856214	0,231362603	0,588317108
Lsm8	117,8388407	0,534466744	0,170444384	3,135725157	0,001714298	0,034605997
C630043F03Rik	85,7580379	-0,137187837	0,233153555	-0,588401224	0,556263017	0,833802026
Nup98	565,0272385	-0,166700958	0,134727831	-1,237316424	0,215969633	0,569610871
Shcbp1l	0,801496318	-2,360105714	2,178972191	-1,083127964	0,278751628	NA
Tradd	262,96622	0,161352966	0,122419284	1,318035533	0,187491753	0,533818545
Zfp597	126,5171582	0,011555328	0,166965744	0,069207778	0,944824232	0,984210313
Lrrcc1	60,78996435	0,649577176	0,284952345	2,279599338	0,022631462	0,169027944
Naa35	623,6959641	0,033021108	0,097571024	0,338431497	0,735038042	0,913858791
Parpbp	2,943702441	-0,42016307	0,837116781	-0,501916913	0,615725968	NA
Slc9b1	0,408298785	-0,027276467	2,571361419	-0,010607792	0,991536365	NA
Dnajc21	631,6798302	-0,276228762	0,09597087	-2,878256308	0,003998801	0,059165556
4930432K21Rik	1,918710693	0,986034655	1,234491863	0,798737265	0,424442774	NA
Lrrc48	6,187865261	0,189227873	0,613522884	0,308428387	0,757756384	0,923804952
P4htm	4,288176682	0,261736436	0,702586714	0,372532573	0,709496365	NA
Gpr146	2024,829643	0,14360097	0,178262742	0,805557956	0,420497777	0,75436738
Sp2	132,9297841	-0,101010732	0,156899205	-0,643793777	0,51970917	0,813876985
Lrriq3	40,26315155	0,528028885	0,27629104	1,911132862	0,055987509	0,285904128

Supplementary Table S1: *Serpina1* KO vs. wildtype all DEGs

Mospd3	621,0271145	-0,244464246	0,105346264	-2,320578227	0,020309618	0,15924885
Nek6	1651,574563	-0,24692241	0,136602143	-1,807602758	0,070668349	0,325220363
Angptl3	23038,1111	0,455763049	0,098720241	4,616713277	3,90E-06	0,000388963
Agrn	810,3697636	-0,239470404	0,094187013	-2,542499191	0,011006286	0,111810815
Tmem8	549,5713102	0,02925925	0,131438665	0,222607633	0,823840895	0,947472731
Nap1l5	1,325563986	-0,249925933	1,612347675	-0,155007469	0,876815429	NA
Cdc20	18,57891994	-1,125476247	0,503626022	-2,234746017	0,025434028	0,182614133
Gsto2	7,773457319	-0,046197146	0,566145681	-0,081599396	0,934965282	0,981974299
Dip2b	133,7581432	-0,094299145	0,136709769	-0,689776203	0,490334936	0,797285888
Prpsap1	1349,505737	0,174699399	0,134524311	1,298645557	0,194065596	0,542888251
Plgrkt	82,90520166	0,326290235	0,218056077	1,496359284	0,134560054	0,456327747
Ebpl	2042,426694	0,324387623	0,118129074	2,7460439	0,006031869	0,077315493
Ddx47	751,8414587	0,155760698	0,087065824	1,788999304	0,073614928	0,334179276
Klhdc7a	612,7543992	-0,077216373	0,193265774	-0,399534649	0,689499298	0,894436573
Ncbp2	619,5680627	-0,227071532	0,098960403	-2,294569591	0,021757803	0,164711248
Ribc2	0,174042788	-1,166820872	3,345560292	-0,348766954	0,727264274	NA
Bard1	3,016989438	-0,880184888	0,898368324	-0,979759486	0,327204855	NA
Snord110	0,093953095	-0,517475177	3,352475198	-0,154356154	0,877328924	NA
Cib3	377,1569555	-0,70286267	0,295675575	-2,377141468	0,017447395	0,1461101
Arf4	2696,14286	-0,228946293	0,10049464	-2,278194072	0,022715017	0,169462649
Arf5	2025,380915	0,068001769	0,082318561	0,82608063	0,408758355	0,746898413
Mir874	0,580715143	-0,289488846	2,834524722	-0,102129589	0,918653815	NA
Klhl9	919,4247091	0,037026228	0,121899908	0,303742869	0,761323795	0,92534041
Oscp1	4,655320188	1,324910793	0,714682637	1,853844945	0,063761346	NA
Cyb5r3	15500,84436	0,038879172	0,112928792	0,344280417	0,73063541	0,911941257
Por	5266,817869	0,400446703	0,131263951	3,050698224	0,002283099	0,041527555
Mir6907	0,122496332	0,780932884	3,352475198	0,232942181	0,815806304	NA
Abcb7	891,7206713	0,022707446	0,149527553	0,151861283	0,879296346	0,965347075
Fzd8	262,9969107	-0,369794582	0,220669038	-1,675788256	0,093779675	0,380194927
Gm12657	0,740626589	-2,313172533	1,870975654	-1,236345609	0,216330125	NA
Ms4a6c	62,12986145	0,545028391	0,31671832	1,720861585	0,085275944	0,362012428
Sema4f	1,483423373	-0,104760041	1,294444018	-0,08093053	0,9354972	NA
Sptlc2	537,56678	0,075714875	0,100676976	0,752057498	0,452016482	0,774656859
Sh2d1a	0,635089825	0,289223264	2,387899885	0,121120347	0,903595713	NA
Nectin4	9,579619575	0,504616274	0,445545177	1,132581611	0,257389995	0,618226166
Hsd3b7	14685,05507	0,132658572	0,099782545	1,32947673	0,183690738	0,529829877
Dusp4	19,9533063	-0,17785215	0,343013218	-0,518499407	0,604109874	0,855575199
Ankef1	0,207615244	0,059593471	3,352475198	0,017775962	0,985817581	NA
4732471J01Rik	13,96259531	-0,271615147	0,470830435	-0,576885281	0,564016955	0,837355044
Tmem67	70,86617975	0,094627691	0,184299302	0,513445738	0,607639558	0,857689206
H2-Q5	67,11385486	0,573173561	0,211242567	2,713343092	0,00666081	0,081996661
Fuk	265,1082897	-0,198633216	0,132510767	-1,498996798	0,133874463	0,455875622
4933425B07Rik	0,560998474	0,622166712	2,387143997	0,260632251	0,794376117	NA
Ildr2	745,1389397	0,955487772	0,240207962	3,977752292	6,96E-05	0,003432024
Erlin1	1715,23933	-0,259904003	0,16172854	-1,607038579	0,108045905	0,408238928
Dpm1	24,10733918	0,104163742	0,351539857	0,296307062	0,766995593	0,927464619
Tap2	626,9347417	0,102650963	0,118411354	0,86690135	0,385996064	0,730226156
Mageh1	65,96654343	-0,231162302	0,181588113	-1,273003498	0,203016801	0,555491529
Fbl	491,5068505	-0,139749355	0,13117534	-1,065363009	0,286711689	0,648131311
Gcgr	5502,807187	-0,129329215	0,114681612	-1,127724076	0,259436466	0,620981526
Emilin2	43,85283884	0,425404282	0,327993683	1,296989253	0,194634885	0,543798072
0610043K17Rik	33,01739709	0,562842902	0,307154383	1,832443013	0,066885445	0,317011065
Mapk10	0,285269197	-1,521344816	3,321239936	-0,458065315	0,646905517	NA
Kcnj10	47,76834422	-0,162393961	0,417647055	-0,388830614	0,697401453	0,898223509
Pinlyp	0,338296039	0,667319034	3,192071415	0,20905517	0,83440517	NA
Rps6ka3	259,4221249	0,130610769	0,150666945	0,866884032	0,386005553	0,730226156
Usp9y	0,6331987	2,745096301	3,286371339	0,835297055	0,403550475	NA
Sema3g	3,907962034	-0,962326395	1,032101281	-0,932395311	0,351132267	NA
Gm14393	2,360663218	-0,425939039	0,943431612	-0,451478447	0,651644756	NA
Dsg4	0,227324296	1,389394708	3,349408814	0,414817893	0,67827522	NA
Pik3c3	425,3450165	0,074746721	0,098783776	0,756670009	0,449247587	0,773349269
Sh2d1b1	1,530425296	0,803138947	1,227237052	0,654428536	0,512835755	NA
Tpra1	440,7776668	0,002022694	0,086938698	0,023265748	0,981438294	0,994983586
Ntan1	296,6251454	0,109575323	0,10047216	1,090603836	0,275447241	0,636882421
Sirt1	216,8588338	0,139959245	0,126055168	1,11030152	0,266869118	0,627135392
Cox7a2	3722,360018	-0,026386771	0,143818617	-0,183472569	0,85442724	0,957135493

Supplementary Table S1: *Serpina1* KO vs. wildtype all DEGs

Nsmaf	465,1832502	0,217190614	0,083043636	2,615379385	0,008912839	0,097989926
Ccdc103	2,450703003	-0,967008054	0,927216042	-1,042915578	0,296987394	NA
1700040L02Rik	40,44503747	0,867563193	0,303802074	2,855685554	0,004294402	0,061604884
BC147527	4,23379755	2,06407896	0,855609126	2,41240877	0,015847503	NA
Msantd1	3,806192751	-0,034757432	0,778117157	-0,044668636	0,964371433	NA
BC052040	114,0913863	0,07179855	0,156885997	0,457647919	0,647205411	0,876230372
Mtg1	405,8127599	-0,076412926	0,104698996	-0,729834373	0,465491431	0,781663619
Gm5148	2,918659789	-0,104703661	0,835226005	-0,125359675	0,900238811	NA
Iars2	1708,219309	-0,017849982	0,084292531	-0,211762324	0,832292463	0,949906691
Hjurp	233,2300982	-0,30413305	0,181005529	-1,680241772	0,092910285	0,378809881
Ap2s1	1231,549858	-0,023297402	0,120860046	-0,192763475	0,84714421	0,953824301
Rubcnl	10,63677919	0,487810856	0,525854467	0,927653727	0,353587219	0,704458903
Slc22a20	0,381658031	-1,103600119	2,548180767	-0,43309334	0,664946961	NA
Tbc1d22b	144,2243341	-0,235789639	0,152258685	-1,548612077	0,121475001	0,43364467
Zfat	55,72767594	0,198312746	0,202384213	0,979882491	0,327144127	0,684930268
Igsf21	2,436148452	-0,561319861	1,191352711	-0,471161778	0,637525211	NA
1700016K19Rik	0,086476712	0,780932884	3,352475198	0,232942181	0,815806304	NA
Gm5134	0,349600122	-0,716166738	2,64656705	-0,270602152	0,786697041	NA
Tigd3	3,320142838	0,321374479	0,779666659	0,412194719	0,680196708	NA
Trim67	0,086476712	0,780932884	3,352475198	0,232942181	0,815806304	NA
Zc3h4	717,6531726	-0,055105192	0,082207403	-0,670319092	0,502654399	0,80422495
Xxylt1	46,68703584	-0,074471393	0,225448782	-0,330325109	0,741154323	0,916519484
Dnah11	5,093050202	0,551475026	0,639862787	0,861864508	0,388762082	NA
Ttbk2	163,9767018	-0,331014619	0,172369104	-1,920382546	0,054809597	0,283546844
Hlx	79,51733034	-0,172867458	0,261769294	-0,660380961	0,509009387	0,807845752
Hrh2	1,373719977	-0,86648366	1,185977017	-0,730607464	0,465018952	NA
Mapk8	489,8012114	0,023179166	0,103858816	0,223179572	0,82339575	0,947466482
Hras	933,126874	-0,155881839	0,121736259	-1,280488163	0,200373505	0,551270699
Lynx1	29,57308208	0,569499282	0,33931208	1,678393771	0,093270253	0,380045721
Sncg	4,301529586	-0,021248395	0,702662815	-0,030239817	0,975875794	NA
LOC105246506	10,94843411	0,796183359	1,484343557	0,53638752	0,591690755	0,850578037
Siglec1	222,0221998	0,0908688	0,283817019	0,320166848	0,748841853	0,919838327
Lix1l	89,03123232	0,330038922	0,203836385	1,619136461	0,105417905	0,403011718
Tspyl1	560,7147969	-0,052303428	0,100916275	-0,518285363	0,604259184	0,855575199
Tgfb1i1	55,55791553	-0,216970364	0,307863832	-0,704760812	0,4809591	0,792841065
Ttf2	120,328838	0,090244232	0,173617278	0,519788311	0,603211127	0,855178108
Syt1	198,8750601	0,625737497	0,178723907	3,50114044	0,000463272	0,013886723
Prkar1b	9,164403902	-0,136935564	0,487647905	-0,280808269	0,778857461	0,931104837
Eny2	990,8979898	0,169638639	0,083217848	2,038488647	0,041501089	0,240706316
Mmgt2	37,75705676	0,40385446	0,300711424	1,342996733	0,179273032	0,522135977
Mrpl22	613,7691693	0,00524639	0,096350095	0,054451323	0,95657559	0,987707257
Hbq1a	0,093953095	-0,517475177	3,352475198	-0,154356154	0,877328924	NA
Impg2	82,48407432	0,53105507	0,302776255	1,753952169	0,079438703	0,349223642
Zdhhc1	46,07030828	-0,134829037	0,266123791	-0,506640301	0,612407226	0,858888222
Slc31a1	5879,707783	0,005725068	0,092997019	0,061561844	0,950911764	0,985972877
Olfr16	0,093953095	-0,517475177	3,352475198	-0,154356154	0,877328924	NA
Gramd1c	725,7122677	0,260848022	0,158559857	1,645107576	0,099947629	0,392993389
Dazap2	3353,191752	0,039325745	0,078914276	0,498334997	0,618247945	0,861339607
4921513I03Rik	0,213661134	0,059593471	3,352475198	0,017775962	0,985817581	NA
Ankrd11	1136,918343	-0,259130314	0,128399877	-2,01815079	0,043575559	0,245910365
Tmem41a	179,7654586	-0,098890737	0,1327324	-0,745038417	0,456248496	0,778008437
Snora61	3,611376767	-0,632740631	0,867967606	-0,728991066	0,466007127	NA
E330020D12Rik	1,845958768	0,442287303	1,103583051	0,400773918	0,688586583	NA
Miat	0,322635192	1,828478932	2,662988853	0,686626581	0,492318074	NA
Crnde	0,536856592	-1,692630402	2,029121104	-0,834169237	0,404185624	NA
2700046A07Rik	0,488023847	1,327074308	2,47482969	0,536228539	0,591800612	NA
4933407L21Rik	0,087021394	-0,517475177	3,352475198	-0,154356154	0,877328924	NA
Gm1968	0,147722973	0,780932884	3,352475198	0,232942181	0,815806304	NA
Gm16861	6,009562621	-0,079270872	0,571454104	-0,138717828	0,88967313	0,968877905
St5	1868,043033	0,049513114	0,152214773	0,325284549	0,744965723	0,918521233
Pafah1b1	1787,433047	-0,02798468	0,08145962	-0,343540514	0,731191867	0,912061199
Ezh1	438,5610315	0,189173615	0,127056699	1,488891303	0,136515994	0,459349518
Rdh18-ps	37,71632514	0,050991128	0,266593745	0,191269035	0,84831482	0,954095425
Wls	96,74903378	-0,0747066	0,258192354	-0,289344741	0,772317578	0,928273961
Rbm7	835,2227164	0,122073507	0,108593709	1,124130565	0,26095764	0,621964694
Magix	424,1764817	0,029198705	0,112200122	0,260237729	0,794680403	0,937363888

Supplementary Table S1: *Serpina1* KO vs. wildtype all DEGs

Trim10	0,747673759	-1,383504555	1,746289386	-0,792253888	0,428212653	NA
Rnf4	1891,361955	-0,003136373	0,099604112	-0,031488387	0,974880054	0,993185764
Rnf2	363,3811406	0,121104704	0,17573917	0,689116172	0,490750164	0,797476473
Stk11	1857,769081	0,086627003	0,096074227	0,901667446	0,367233551	0,715572853
Uri1	573,6842338	-0,131759125	0,148318899	-0,888350207	0,374352401	0,720933206
Hnrnpa2b1	7391,303613	-0,137946715	0,074337197	-1,855688962	0,063497905	0,306618538
Vegfd	23,76086909	-0,170988351	0,31729233	-0,538898468	0,589956915	0,84953676
S100a4	13,76352274	0,066538077	0,474920364	0,14010365	0,888578097	0,96836734
Sox9	220,993726	-0,12050023	0,230680283	-0,522369004	0,601413433	0,854422974
Siah1b	21,41896646	-0,126322302	0,32910438	-0,383836585	0,70109957	0,899833253
F7	2385,903576	0,196364532	0,072347799	2,714174225	0,006644121	0,081884267
F3	84,92095413	0,148152468	0,218462339	0,678160224	0,497670109	0,801319004
F2	46247,5594	0,06694754	0,059000448	1,134695455	0,256502942	0,616948518
Cyr61	206,6476968	-0,071474291	0,254113662	-0,281268983	0,778504099	0,930864224
Gfra3	0,113662148	0,780932884	3,352475198	0,232942181	0,815806304	NA
Gbp2	163,0605592	1,128558788	0,331234345	3,407130947	0,000656496	0,017730703
Gbp2b	80,74123351	0,864367237	0,376807455	2,293922866	0,02179493	0,164898934
Vill	38,30033569	0,08724782	0,244870946	0,356301232	0,721614987	0,909525085
Ahrr	2,778093903	0,538087238	0,846473679	0,635681003	0,524984361	NA
Tpp2	971,5953638	-0,205548055	0,103616454	-1,983739518	0,047284886	0,259242489
Col3a1	882,5005631	-0,439346278	0,470715906	-0,933357621	0,350635354	0,702050628
Hc	26864,22881	-0,248054612	0,131676778	-1,88381441	0,059590084	0,295164687
Thy1	30,34304738	0,524374869	0,33195029	1,579678901	0,11418042	0,41832777
Gzmc	1,316479986	0,480701578	1,590872538	0,30216222	0,762528403	NA
Apoh	51127,44989	0,340206572	0,076939732	4,421728086	9,79E-06	0,000808037
Cdk2ap1	165,1136404	0,008382321	0,162793054	0,051490657	0,958934547	0,987896001
Dbr1	214,5348026	0,158316872	0,143374521	1,104219014	0,269498144	0,629991376
Lyve1	173,5459629	-0,429185321	0,614290701	-0,698668107	0,48475947	0,79420977
Wdr6	352,9520746	0,144467385	0,11966526	1,20726253	0,227331056	0,582853433
Gtf2a1	381,8626638	0,157427096	0,146045147	1,077934452	0,281063009	0,641764538
Tex14	4,068167465	-0,830419485	0,785078425	-1,057753543	0,290167818	NA
Vps16	563,891238	-0,019901416	0,104079018	-0,191214489	0,848357553	0,954095425
Prex2	80,4575065	-0,284495839	0,236201057	-1,204464716	0,228410012	0,584236803
Fstl3	12,34853657	0,514554527	0,44970173	1,144212915	0,252535348	0,612043137
Tktl1	0,087021394	-0,517475177	3,352475198	-0,154356154	0,877328924	NA
Krt7	37,17027689	1,265373072	0,287688664	4,398411302	1,09E-05	0,000883538
Pik3ap1	676,4527913	-0,178523905	0,12673943	-1,408590088	0,158956412	0,495312554
Cenpj	97,48952435	0,148702019	0,22464816	0,661932951	0,508014193	0,807274415
Fam63a	2592,878932	0,106889885	0,088311168	1,210377883	0,226133925	0,581158101
Adprhl2	260,1341948	-0,080035246	0,183985167	-0,435009233	0,663555726	0,883893982
Top3a	164,230908	-0,00463774	0,145188755	-0,031942832	0,974517641	0,993185764
Snord66	0,259974817	0,586045239	3,35071904	0,174901337	0,86115716	NA
Snord55	1,655824723	-0,173590531	1,38529262	-0,125309648	0,900278414	NA
Plekhg1	80,99895257	-0,159631122	0,280453172	-0,569189933	0,569227251	0,840790976
Kctd10	286,502691	0,078021191	0,158767762	0,49141709	0,623131479	0,863474234
2310007B03Rik	11,16839935	-0,206944475	0,421126414	-0,491407017	0,623138603	0,863474234
Sod3	958,0021084	0,057085198	0,164847808	0,346290306	0,729124553	0,911594858
Sod1	25374,85156	0,025420096	0,100882538	0,251977165	0,801058714	0,939741275
Arxes2	0,407754103	1,083769393	2,547329865	0,425453102	0,670506403	NA
Vipr1	673,6336543	0,11353888	0,191117849	0,594077842	0,55246005	0,830818463
Bhlhe40	1877,638797	-0,175623156	0,229345927	-0,765756594	0,443821146	0,770142004
Nckap5l	25,24738195	0,301318239	0,381999025	0,788793215	0,430232876	0,761019227
Timp2	319,4640081	0,1375597	0,21039762	0,653808254	0,513235348	0,811045316
Adam22	3,729362495	0,732609361	0,824527848	0,888519851	0,374261183	NA
Tbx6	11,48594671	-0,22881613	0,417290891	-0,548337228	0,58346037	0,846950064
Zfr	1220,232275	-0,278556487	0,109205054	-2,550765524	0,010748661	0,109945564
Ufsp1	69,53271621	-0,010730927	0,207753416	-0,051652228	0,958805803	0,987896001
Ten1	43,4203805	-0,234943743	0,269686965	-0,871172038	0,383660225	0,728262608
Avpi1	916,9729538	0,195957317	0,147286724	1,330447931	0,183370731	0,529363703
Rpain	434,6992159	0,015129403	0,090219467	0,16769555	0,866822797	0,961775826
Cryzl1	273,2810568	0,063286273	0,12484177	0,506931882	0,612202615	0,858888222
Mpc2	3408,020282	0,222115497	0,076810235	2,891743489	0,003831106	0,057613111
1700030J22Rik	9,748928528	0,218554755	0,507833659	0,430366817	0,666928829	0,885419218
Ndufaf5	150,5971365	0,02537062	0,12865907	0,197192624	0,843676803	0,953068489
Esam	162,974304	-0,199658921	0,28367818	-0,70382192	0,481543681	0,793315769
Dnajc24	102,8487772	0,144981866	0,158962937	0,912048234	0,361743327	0,71109038

Supplementary Table S1: *Serpina1* KO vs. wildtype all DEGs

Sat2	311,0364062	0,401292483	0,137661796	2,915060646	0,003556194	0,055475801
Nup35	66,7864619	0,151338476	0,252475156	0,599419277	0,548893325	0,829076361
2300009A05Rik	230,7249742	0,247611403	0,148493707	1,667487515	0,095417505	0,384691382
Ptms	18280,36866	-0,064424059	0,087637302	-0,735121431	0,462265544	0,781516914
Ttc9c	404,7843285	-0,128274684	0,131727446	-0,973788588	0,330161521	0,686792356
Ube2cbp	50,93675367	0,218222979	0,21618501	1,009426967	0,312769911	0,672219008
Dhx16	984,8241525	-0,205995004	0,099987691	-2,060203622	0,039379079	0,233056618
Scg2	0,757669934	0,381333215	1,758187625	0,216889944	0,828294115	NA
Emp3	53,83732516	-0,099504062	0,291556472	-0,341285727	0,732888492	0,912884212
Calml3	0,142634598	-0,517475177	3,352475198	-0,154356154	0,877328924	NA
Dnase2a	307,8011368	0,040311319	0,147136111	0,27397298	0,784105387	0,933246486
Pa2g4	2323,737415	-0,152331747	0,097526662	-1,561949767	0,118299823	0,427187007
Zfp61	11,04864099	0,183905062	0,43016548	0,427521665	0,668999409	0,886730269
Atp10a	10,59898793	0,175228552	0,439902545	0,398334935	0,690383312	0,894644908
Casq1	4,003666278	0,073091194	0,76544273	0,095488783	0,9239266	NA
Proc	13661,16546	-0,084487481	0,094237126	-0,896541364	0,369963699	0,717858445
Zfp46	104,3465738	-0,086883024	0,193023129	-0,450117169	0,652625958	0,877662367
Casp6	411,208488	0,662385482	0,179240639	3,695509492	0,000219446	0,008039573
Zfp40	58,04879797	0,07818766	0,202858473	0,385429599	0,699919157	0,899228386
Zfp37	8,012822448	-0,508641253	0,520715452	-0,97681229	0,328662091	0,686006046
Cdh1	2516,543307	-0,590408412	0,179647702	-3,286479073	0,001014483	0,024047207
Zscan2	83,01480959	-0,117984053	0,203825487	-0,578848381	0,562691483	0,83722511
Plaur	9,807900031	0,614310666	0,494031476	1,243464629	0,213696667	0,567064616
Nfs1	1437,48981	0,013861469	0,068824628	0,201402744	0,84038367	0,951657825
Flna	556,8374378	-0,057345328	0,180905796	-0,316989997	0,751251193	0,920659748
Glcci1	124,1810896	0,035443837	0,162959437	0,217500976	0,827817947	0,947580607
Bmpr1b	0,259974817	0,586045239	3,35071904	0,174901337	0,86115716	NA
Parp10	455,8376873	0,413817446	0,116256634	3,559516835	0,000371538	0,011882985
Tjp1	946,9228351	-0,023574003	0,144228812	-0,163448639	0,87016521	0,961775826
Magi2	3,041896145	0,349168872	0,786022088	0,444222723	0,656881566	NA
Ints9	306,3830421	0,02462781	0,120351836	0,204631773	0,83785983	0,951141594
Zmym5	605,8380873	0,038552047	0,109424418	0,352316672	0,724600786	0,910252319
Chuk	2314,118275	0,011587142	0,096503241	0,120069974	0,904427718	0,973666426
Cdh2	1935,514864	-0,009703387	0,182491408	-0,053171745	0,957595068	0,98781716
Slc26a5	0,219943115	0,059593471	3,352475198	0,017775962	0,985817581	NA
Dvl2	137,7833122	-0,23051152	0,154754324	-1,489532014	0,13634733	0,459349518
Sidt2	3014,809285	-0,016940329	0,086566276	-0,19569202	0,844851231	0,953149039
Ppm1h	49,97237109	0,000337169	0,27040067	0,001246922	0,9990051	0,999803624
Fcgbp	4,721157693	-0,192041627	0,692758982	-0,277212756	0,781616745	NA
Lpgat1	3579,582239	-0,052380117	0,159764147	-0,327859021	0,743018259	0,917092327
Apol7e	6,730084125	2,167784707	0,716740563	3,024504011	0,002490412	0,043465162
Hectd2	48,19645406	-0,227762071	0,309422372	-0,736087923	0,461677192	0,781516914
Tmem50b	130,9895391	-0,09728	0,140403145	-0,69286197	0,488396178	0,796400974
Ttc25	8,003142753	-0,009988146	0,513252898	-0,019460477	0,984473766	0,995554769
Cd200r3	0,654882655	0,879130198	2,068830481	0,424940664	0,67087993	NA
Mrpl47	271,6745226	0,095100901	0,130703049	0,727610425	0,466852097	0,782586618
Trmt13	77,17526489	0,156737615	0,221317315	0,70820313	0,478819123	0,79118575
Ndnl2	430,1663702	0,10784663	0,117148727	0,920595829	0,35726148	0,707149983
Slc16a6	174,8102854	-0,394754639	0,224101235	-1,7615014	0,078153576	0,346430756
Zfp784	83,01168451	0,151309905	0,184750717	0,818994956	0,412789292	0,74954611
Pik3r6	17,87343115	-0,0788426	0,432959444	-0,182101582	0,855503007	0,957570303
Leo1	615,1857387	-0,125817205	0,119716664	-1,050958159	0,293277807	0,654344293
Rrn3	399,0869772	-0,067023349	0,199494164	-0,335966465	0,736896155	0,914722813
Nfe2	65,81516252	0,950708455	0,381344369	2,493044426	0,0126653	0,121248489
2010300C02Rik	3,423073029	1,352619793	0,849065559	1,593068732	0,111144773	NA
Plscr2	518,3013122	0,770283098	0,229488409	3,356522887	0,000789292	0,020253442
Zfp808	6,509253761	1,041868604	0,626599779	1,662733755	0,096365742	0,385838157
Slc12a7	2536,752472	-0,213239525	0,130313651	-1,636356006	0,101765101	0,397109642
Oxct1	106,5865706	0,160869445	0,286220818	0,562046627	0,574084251	0,84275089
Oxsr1	630,4647527	-0,231134819	0,108050674	-2,139133537	0,032424855	0,20931332
Hemk1	283,3210079	0,268315258	0,179805326	1,492254225	0,135632512	0,458243885
Ppif	978,2023954	0,320494835	0,102264073	3,133992473	0,001724453	0,034651586
Ubald2	0,086476712	0,780932884	3,352475198	0,232942181	0,815806304	NA
Ubash3b	18,76153762	-0,244624221	0,440181539	-0,555734849	0,57839213	0,84530168
Arglu1	523,3049785	-0,006633198	0,096190754	-0,068958791	0,945022421	0,984340066
Bptf	665,4112463	0,023351115	0,141948762	0,16450383	0,869334532	0,961775826

Supplementary Table S1: *Serpina1* KO vs. wildtype all DEGs

Fbxo2	7,125138575	0,361335628	0,584564557	0,618127841	0,536491071	0,823555991
Ints5	304,6828799	-0,362444124	0,154326709	-2,348550856	0,018846625	0,152703354
Ccdc88a	25,08804616	-0,459077458	0,397490846	-1,15493844	0,248115636	0,60717432
Osbpl11	468,8957835	-0,105584772	0,152834346	-0,690844535	0,489663245	0,796768631
Esrp2	2181,848359	-0,051911184	0,089179285	-0,582099128	0,560499912	0,835469208
Aqp11	429,2651343	0,243833803	0,098161067	2,484017439	0,012990945	0,122739179
Fam76b	159,2177947	0,123998932	0,132420122	0,936405508	0,349064443	0,700954068
Dnajc22	2376,897931	0,029975059	0,084737541	0,353740019	0,723533729	0,910252319
Ppm1f	661,3589746	-0,096122317	0,098836975	-0,972533989	0,330784964	0,687536562
Spire1	13,33875228	-0,121671622	0,401004385	-0,303417186	0,76157195	0,92534041
Ppcdc	348,8775	0,080233311	0,139677092	0,574419968	0,565683643	0,837781736
P3h4	22,02817366	-0,167382487	0,347458849	-0,48173327	0,629995438	0,866235628
1810011O10Rik	593,5173027	1,175838929	0,475511194	2,472789164	0,013406321	0,124997488
Pou6f1	169,4086366	-0,16637855	0,211697899	-0,785924426	0,431911764	0,7622164
Gps2	431,0898835	0,143856521	0,117400935	1,225343914	0,220445669	0,574490867
Lrig1	670,925525	-0,135925562	0,128070529	-1,061333657	0,288538295	0,649625922
Pdpk1	589,2405197	-0,210594284	0,128346003	-1,640832427	0,100832205	0,394274861
Npdc1	84,76025019	0,05348374	0,325973827	0,164073725	0,869673106	0,961775826
Npas2	287,1140405	0,124553119	0,220539405	0,564765826	0,572233055	0,841794408
Npas1	0,147722973	0,780932884	3,352475198	0,232942181	0,815806304	NA
Fgr	60,67896143	0,110410833	0,273332066	0,403943945	0,68625394	0,893161227
Hsdl2	2137,114324	-0,100040469	0,149728333	-0,668146548	0,504040052	0,804997854
Akirin1	677,8192404	-0,129929559	0,154351129	-0,841779128	0,399911597	0,740788928
Katnbl1	117,3319385	-0,05103371	0,256553544	-0,198920309	0,842325083	0,952227637
Slc9a3r2	444,0399402	-0,350265326	0,119120379	-2,940431599	0,003277554	0,052350793
Trp53rkb	17,68192902	0,255578624	0,411710062	0,620773324	0,534748778	0,822042413
Gstp2	32,10319044	-0,343551387	0,401709382	-0,855223708	0,392427314	0,734887345
Gpr141	2,398492193	1,028500409	1,113390704	0,923755161	0,355613805	NA
R3hdm1	534,1857261	-0,271978835	0,105867361	-2,569052741	0,010197694	0,106013192
Aida	188,1169669	0,297321999	0,139928714	2,124810488	0,033602429	0,214233402
Polr1d	841,281242	0,043692272	0,114842049	0,38045535	0,703607435	0,900719679
A230107N01Rik	5,50492672	-0,807071989	0,638401105	-1,264208321	0,206155265	0,558880735
Ric8a	494,5615777	-0,178107116	0,123373952	-1,443636302	0,148841308	0,479794094
2610028H24Rik	0,093953095	-0,517475177	3,352475198	-0,154356154	0,877328924	NA
Nt5c2	359,9564801	-0,008580598	0,147775325	-0,058065158	0,953696728	0,986479135
Flywch2	22,27796684	0,447121229	0,303274501	1,474311975	0,140397612	0,465404335
Lrg1	10702,44023	0,363608935	0,135658307	2,680329308	0,007354976	0,087093605
Cela1	402,5314995	-0,56703625	0,294004933	-1,928662366	0,053772791	0,280050037
Nek8	142,6283024	0,358447564	0,191742908	1,869417579	0,061564741	0,300715754
Pcdhb18	0,294636637	-0,629863441	2,754870789	-0,228636292	0,819151615	NA
Tsacc	72,13612371	-0,257527182	0,200034693	-1,287412593	0,197950518	0,547795584
Mettl15	65,63641077	0,528440747	0,238642095	2,214365188	0,026803668	0,18930315
Pi4ka	994,3139836	0,056175638	0,097941958	0,573560501	0,566265247	0,838041311
Rnft1	870,5025767	-0,021827884	0,133842133	-0,163086793	0,870450098	0,961775826
Naa30	447,8129566	-0,05257733	0,182334606	-0,288356287	0,773074025	0,928591792
Fgf2	0,31434569	0,667660234	3,262174889	0,20466721	0,837832141	NA
Gclm	3817,020881	-0,082922657	0,112956402	-0,734112058	0,462880445	0,781516914
Ear2	11,05602891	0,562155666	0,475928928	1,181175661	0,237532948	0,59478675
Fgf18	1,875944653	2,694044176	1,317531539	2,044766365	0,040877897	NA
Nicn1	499,7328094	0,004799634	0,091744331	0,052315318	0,958277448	0,987896001
Rps27a	3620,939469	0,020911344	0,140952132	0,148357774	0,882060423	0,96610403
Fars2	637,987754	0,135204249	0,098240168	1,376262399	0,16874041	0,507969047
Arfgap3	275,5289736	-1,125522897	0,154240028	-7,297216626	2,94E-13	3,09E-10
Arl2bp	139,3394409	0,415148687	0,176543343	2,351539747	0,018695892	0,152034903
Spp2	1969,55316	0,098861188	0,146276471	0,675851604	0,499134867	0,802710699
Pxdc1	2659,16122	0,057508282	0,134379548	0,427954125	0,66868452	0,886564944
Myo19	218,5363138	-0,237998026	0,151145547	-1,574628102	0,115342312	0,420395685
Rps25	4723,930934	0,114358753	0,130196348	0,878356073	0,379750515	0,725269233
Atp6v1c1	809,8346465	-0,020468294	0,086706881	-0,236063085	0,813383718	0,943900659
Smg8	269,1885739	-0,141910762	0,114615098	-1,238150681	0,215660195	0,569610871
Gadd45g	607,1302891	-2,09360713	0,672115736	-3,114950324	0,001839759	0,036276898
Chchd2	6106,134014	-0,019592299	0,088900976	-0,220383396	0,825572576	0,947537943
BC100451	0,773199728	0,45813135	1,944429464	0,235612224	0,813733587	NA
Usp16	651,7774943	0,011211819	0,114085534	0,098275556	0,921713488	0,977341679
Hdhd3	700,2402446	-0,166000873	0,161183883	-1,029885061	0,303063964	0,662824716
1700020L24Rik	0,093303375	-0,517475177	3,352475198	-0,154356154	0,877328924	NA

Supplementary Table S1: *Serpina1* KO vs. wildtype all DEGs

2810004N23Rik	601,8182459	-0,018671576	0,09224373	-0,202415662	0,839591786		0,951377482
Nkg7	13,9038598	0,955493668	0,445582786	2,144368449	0,032003376		0,207796569
Phf10	460,507394	-0,238182615	0,097398352	-2,445448089	0,014467227		0,13080163
Asrgl1	263,3939987	0,375266254	0,140261693	2,675472161	0,007462407		0,087821232
Bccip	1558,214766	0,042693731	0,091727852	0,46543913	0,641617018		0,872059191
Anapc11	284,0720675	-0,010122544	0,138089313	-0,073304329	0,941563947		0,983128178
Ap3b2	0,093953095	-0,517475177	3,352475198	-0,154356154	0,877328924	NA	
Pias4	298,6243292	-0,12280042	0,13432959	-0,914172527	0,360626197		0,710569143
Ctse	6,127660994	-0,227015837	0,610520959	-0,371839547	0,710012317		0,90384257
Maea	793,5133073	-0,064965377	0,078131314	-0,831489632	0,405697083		0,745391028
Kidins220	1532,902509	-0,102519477	0,102603721	-0,999178935	0,317708019		0,675759967
Tmem200b	35,57037689	0,265685665	0,389328417	0,682420429	0,494973141		0,80006558
Arnt	899,2571241	-0,070449742	0,115586693	-0,609497	0,54219506		0,826611597
Arhgef10	58,63232472	0,113859593	0,305699754	0,372455627	0,709553644		0,90343073
Arnt2	0,351607642	0,61776838	2,632591545	0,23466169	0,814471325	NA	
Ccr1	2,147960801	0,355856424	1,044621451	0,340655865	0,733362668	NA	
Snora16a	2,910090574	-0,153221084	0,811422489	-0,188830217	0,850225888	NA	
Snora81	11,82439495	0,449034618	0,469201876	0,957017953	0,338558194		0,694071514
Snord12	2,134674244	-1,870435112	1,112419362	-1,681411862	0,092682943	NA	
Nucks1	1204,336275	-0,04833542	0,101246933	-0,477401322	0,633076373		0,867527474
Ptprk	1767,506672	0,04527115	0,095897442	0,472078809	0,636870537		0,86976752
Strbp	746,2955932	-0,111337981	0,116092819	-0,959042783	0,337537193		0,692850411
Ctsg	0,293442235	0,617258985	2,768781978	0,222935208	0,823585934	NA	
Dcx	2,946076435	-0,700151157	1,109505895	-0,631047713	0,528009328	NA	
Sema5a	19,76146763	0,878982409	0,412937067	2,128611064	0,033286452		0,213023735
Dpp4	3079,841227	0,18535144	0,113153025	1,63805996	0,101409187		0,395951932
Gabra4	0,443168919	2,196464572	3,313880474	0,662807421	0,5074539	NA	
Ptpn20	1,578382011	1,803715091	1,360471741	1,325801217	0,18490555	NA	
Hk1	82,56968419	0,048668787	0,222458412	0,218777012	0,826823755		0,947537943
Mir7022	0,295445946	1,389394708	3,349408814	0,414817893	0,67827522	NA	
Zfp938	111,3557651	-0,123569879	0,164998294	-0,748916101	0,453907775		0,776393685
Tnfsf13	5,388494548	-1,033772182	0,630475184	-1,639671487	0,101073491		0,394871857
Fth1	43928,11279	0,222564121	0,091254011	2,43895166	0,014729939		0,132226596
Ftl1	86615,03262	0,191998173	0,125117243	1,534546064	0,12489537		0,439748802
5730507C01Rik	5,32894686	0,430953844	0,687850317	0,62652271	0,530972146	NA	
Krt83	0,12663974	0,780932884	3,352475198	0,232942181	0,815806304	NA	
Epn1	3776,228148	-0,071263105	0,089651735	-0,794888193	0,426678543		0,758848669
Rsl1	58,52590736	-0,158237367	0,212122829	-0,745970472	0,455685252		0,77774239
Lipo3	37,27027285	0,132800843	0,333431922	0,398284729	0,690420316		0,894644908
Igfbp3	565,6441985	0,402603209	0,208985829	1,926461758	0,054046742		0,280929588
Mir7024	0,087021394	-0,517475177	3,352475198	-0,154356154	0,877328924	NA	
Cfap126	64,57212568	0,36157771	0,263735738	1,370984883	0,170379668		0,510489867
Rabl3	286,6162411	0,144599464	0,125769663	1,149716551	0,250260635		0,609210778
Trim71	0,093953095	-0,517475177	3,352475198	-0,154356154	0,877328924	NA	
Sqrdl	4201,90888	0,252526916	0,102495089	2,463795271	0,013747461		0,127190178
Taco1	495,0875841	-0,075718144	0,146599479	-0,516496677	0,605507559		0,856421285
Cd209g	4,493398201	-1,812727913	0,722939349	-2,507441205	0,012160881	NA	
Fam162a	1217,988086	0,008545052	0,123433456	0,069228008	0,944808129		0,984210313
Vps36	425,852295	-0,040413641	0,132634522	-0,304699265	0,760595209		0,925072539
2210016F16Rik	753,6713014	0,073041067	0,112806208	0,647491558	0,517313857		0,813424627
Ankrd40	1515,952944	-0,152142024	0,089114334	-1,707267704	0,087772306		0,367306865
Zfp958	105,39897	0,120958166	0,180087024	0,671665086	0,501796933		0,803893049
Srrd	180,5042633	0,029427365	0,15256111	0,192889036	0,847045872		0,953824301
Klhdc3	1209,622596	-0,116098	0,09553312	-1,215264402	0,224265265		0,579586762
Amdhd1	6905,060336	0,154770281	0,139936153	1,106006402	0,268723748		0,629281048
Cfap45	1,679731575	-0,259673173	1,129663654	-0,229867688	0,818194585	NA	
Cotl1	323,3606819	0,040287057	0,211159834	0,190789397	0,848690596		0,954095425
Cdhr5	2330,903002	-0,401753859	0,149759625	-2,682658021	0,007303964		0,086643026
Mir6908	0,086476712	0,780932884	3,352475198	0,232942181	0,815806304	NA	
Dtnb	648,8476272	-0,100375342	0,082286931	-1,219821193	0,222532665		0,577161019
Ephb2	4,985997212	-0,480799788	0,718573951	-0,669102725	0,503429952	NA	
Alox5	1,236536737	-0,313187049	1,247624181	-0,251026754	0,801793424	NA	
Alox15	0,635661175	-1,130066254	2,029805463	-0,556736236	0,577707655	NA	
Mbd4	26,68115582	0,059363258	0,301585075	0,196837518	0,84395469		0,953068489
Aldoc	654,2016094	-0,007702861	0,151108857	-0,050975574	0,959344985		0,987937108
Hivep3	7,012471808	0,275953207	0,552001732	0,499913661	0,617135873		0,860830256

Supplementary Table S1: *Serpina1* KO vs. wildtype all DEGs

Csf2ra	127,3659156	0,059503464	0,244292597	0,243574571	0,807560325	0,942032857
Osbpl8	880,834441	-0,558623799	0,164782982	-3,390057591	0,000698779	0,018572529
B2m	55281,6897	0,149367056	0,071974142	2,07528775	0,037959874	0,228686669
Klrc2	0,592225239	1,669948828	2,344317007	0,712339169	0,476254775	NA
Slc22a2	0,269274339	0,059593471	3,352475198	0,017775962	0,985817581	NA
Cdh5	528,9744554	-0,188720794	0,262712322	-0,718355319	0,472538229	0,787019872
Matr3	1286,06486	0,000954196	0,118935453	0,008022807	0,993598795	0,99822248
Ndn	15,61319839	0,435521443	0,430760684	1,011051981	0,311991551	0,671577331
Klra3	0,216449427	0,059593471	3,352475198	0,017775962	0,985817581	NA
Akap1	2541,158258	-0,110358816	0,07954519	-1,387372597	0,165328176	0,503593618
Selenbp2	17104,50308	-2,150410208	0,727878065	-2,954355012	0,003133233	0,050712104
Scand1	254,4997422	0,344110672	0,198063607	1,737374556	0,082321079	0,354518456
Nrxn2	29,89184171	-0,187770677	0,307593159	-0,610451408	0,541562823	0,826407188
Tyro3	5,877440526	-0,347828987	0,664892603	-0,523135595	0,600879897	0,854422974
Scube2	0,229655992	-1,252384348	3,339190293	-0,375056298	0,707618598	NA
Tmem9b	500,5268366	0,060348117	0,097730141	0,617497492	0,536906635	0,823949426
Slc6a14	0,093303375	-0,517475177	3,352475198	-0,154356154	0,877328924	NA
Med20	310,922806	-0,006387534	0,107696644	-0,059310428	0,952704856	0,986424351
Dhodh	242,5162656	-0,10972886	0,128042688	-0,856970914	0,391460959	0,734518115
Tle2	383,9817695	-0,209440007	0,163959941	-1,277385226	0,201466278	0,552833061
Mir7231	0,213116452	1,337107854	3,350237755	0,399108348	0,689813372	NA
Atp5sl	795,2976352	0,126266798	0,115914732	1,08930759	0,276018267	0,637060499
Armc8	433,1648344	-0,163279988	0,120317045	-1,357081108	0,174755441	0,517225036
Znrf3	146,9302459	-0,069368825	0,15308941	-0,453126217	0,650457858	0,876831587
Rmi2	2,592448268	-3,666697908	1,401498813	-2,616269007	0,008889649	NA
Gria3	29,42002711	-1,023405389	0,484966966	-2,110257937	0,034836145	0,217933747
4933406I18Rik	6,83266065	-0,284512614	0,55189512	-0,515519351	0,606190151	0,856585575
1700028J19Rik	6,291251378	0,213213053	0,572315165	0,372544825	0,709487244	0,90343073
1700003G18Rik	0,378572572	-2,086637537	3,060427456	-0,681812448	0,495357552	NA
Tmem161b	179,8551964	0,007735816	0,163002612	0,047458236	0,962148015	0,988526411
Tgoln1	2091,846163	-0,358776908	0,088581007	-4,050269027	5,12E-05	0,002724868
Mir6905	0,879375383	-1,050630917	1,637753487	-0,641507361	0,5211931	NA
Slc25a34	105,3439523	-0,112633581	0,327934047	-0,343464127	0,731249324	0,912061199
Gypc	319,2856627	0,354695215	0,241770603	1,467073376	0,142356096	0,469336287
Fzd5	211,1392508	-0,428699232	0,175703579	-2,439900392	0,014691312	0,131994726
Smad7	146,2588693	0,416555431	0,244162331	1,706059368	0,087997027	0,367864993
Tead4	4,602634031	0,47037597	0,770393619	0,610565766	0,541487092	NA
Mterf1b	17,52237124	0,248841211	0,339817404	0,732279184	0,463998182	0,781663619
Tubb1	3,631796292	0,760391459	0,909655392	0,835911561	0,403204658	NA
Lclat1	900,6219379	0,116666086	0,116784655	0,998984722	0,317802092	0,675759967
Smek1	1363,168743	0,229217413	0,139025613	1,648742334	0,099200433	0,392602307
Prrt2	8,816206551	0,962543829	0,561407323	1,714519545	0,086433351	0,364537725
Cd59b	87,73919572	0,069003902	0,219443453	0,314449583	0,753179602	0,921533083
Tmc8	47,54665499	0,079745656	0,2707038	0,294586394	0,768309857	0,927598042
Optn	1796,291358	0,033085745	0,126744414	0,261043019	0,794059332	0,936961978
Hcfc1r1	1216,685735	0,064901639	0,077855179	0,833620066	0,404495115	0,744376512
Tmc4	74,10719256	-0,262812169	0,254752098	-1,031638877	0,302241321	0,6622779
Wfikkn2	0,521416251	1,495067211	2,420351971	0,617706527	0,536768808	NA
Ccdc67	4,45429696	-0,619031679	0,716745315	-0,863670353	0,387769004	NA
Cep128	21,4846398	-0,117260246	0,436595315	-0,2685788	0,788253828	0,934897572
Slx1b	221,2411689	-0,079378509	0,134664072	-0,58945573	0,555555601	0,833021852
9130401M01Rik	287,5782012	0,07732392	0,127072577	0,608502021	0,542854566	0,826611597
Slc10a6	2,550644656	0,899926651	1,111802638	0,809430217	0,418267727	NA
Dqx1	219,1000877	0,553796186	0,25424002	2,178241595	0,029388055	0,197530872
Oser1	203,3203768	0,174573722	0,117288819	1,488408896	0,136643092	0,459349518
Mt2	1031,455038	5,496835666	1,054408129	5,213195456	1,86E-07	3,50E-05
Map3k5	600,3567989	-0,113484501	0,112455512	-1,009150184	0,312902614	0,672219008
Htr7	0,620765517	0,845634618	1,852901594	0,456383988	0,648113878	NA
P2rx1	3,695712415	0,398804013	0,92111415	0,432958295	0,665045068	NA
Hsf2	456,4237877	-0,024565635	0,137755709	-0,178327526	0,858465763	0,958795917
Ndst1	2996,35547	-0,170287428	0,164094071	-1,037742722	0,29938985	0,659904651
Nbl1	19,67391815	0,838620525	0,379975773	2,20703683	0,027311486	0,190766594
Rabac1	862,0943485	-0,177206092	0,140720546	-1,259276611	0,207930438	0,560182483
Prkar2a	1977,058275	-0,317584116	0,126024683	-2,520015203	0,011734977	0,115867727
Plekho1	119,3940486	-0,04921178	0,255027066	-0,192966893	0,846984896	0,953824301
Ccdc94	105,2970889	0,141797846	0,145369733	0,975428947	0,329347531	0,686456976

Supplementary Table S1: *Serpina1* KO vs. wildtype all DEGs

Dmgdh	14651,70335	0,575919475	0,192181252	2,996751603	0,00272873	0,046341149
Ccdc97	555,7211657	-0,16978687	0,087114098	-1,949017143	0,051293378	0,27322396
Syvn1	4279,105029	-1,350964575	0,161540968	-8,362984289	6,12E-17	1,64E-13
Ddit4	284,4594337	1,301743643	0,526062368	2,474504399	0,013342118	0,12464764
Gga2	515,6361979	-0,054118581	0,120919062	-0,44756038	0,654470503	0,878857794
Nebl	1,934695619	1,260771421	1,172869258	1,074946259	0,28239879	NA
0610037L13Rik	432,3623243	0,187278356	0,130154154	1,438896493	0,150179851	0,482372211
Tspan17	52,84525888	0,245367894	0,236600233	1,037056858	0,29970936	0,659904651
Lrrc2	0,420731695	1,124606493	2,207195302	0,509518343	0,610388944	NA
Mb21d1	8,715404544	-0,272025877	0,611681978	-0,444717822	0,656523688	0,879906285
Dnd1	10,21004428	-0,286060175	0,592054162	-0,483165551	0,628978193	0,866097574
Adamts2	127,5972429	-0,264432548	0,243110677	-1,087704379	0,276725634	0,637663478
Gpr4	30,88987035	0,057422739	0,367132216	0,156408882	0,875710737	0,964125574
Wdr43	470,7549848	0,282685238	0,174951134	1,615795401	0,106138556	0,404074749
B330016D10Rik	36,49580844	-0,067005681	0,272585132	-0,24581561	0,805824973	0,941208637
Izumo1	1,158837772	-0,332225076	1,486037359	-0,223564417	0,823096254	NA
Adar	596,244537	0,08483924	0,109887286	0,772056927	0,440080711	0,767402932
Fbln5	129,5927218	-0,273677255	0,221561452	-1,235220531	0,21674844	0,57030307
Pacsin2	1613,771032	-0,164105211	0,10935535	-1,500660109	0,133443496	0,454871518
Uqcrq	6296,088373	0,101117081	0,11940089	0,846870407	0,397067359	0,738461552
Xdh	3599,654206	0,092770241	0,107935618	0,859496087	0,390066878	0,733963887
Aif1	134,1947792	0,542090477	0,255149048	2,124603176	0,033619739	0,214233402
Pla2g7	92,42561448	1,059296568	0,247813788	4,274566718	1,92E-05	0,001334746
Trem3	0,543228027	1,556904296	1,881582489	0,827444082	0,407985404	NA
Neurl1a	7,498482234	-0,063324602	0,650700464	-0,097317592	0,922474186	0,977360689
Pgam1	1171,540984	0,000898589	0,106318935	0,00845182	0,993256504	0,998184334
Wfdc1	105,737787	-0,029234235	0,191924958	-0,152321172	0,878933627	0,965347075
Prps1	683,3879265	0,087330181	0,087097782	1,002668245	0,316020956	0,67419376
Ttf1	286,9319706	-0,346235014	0,113966235	-3,038049055	0,002381152	0,042558315
Mir7578	0,273083462	-0,658048199	3,340448562	-0,196993963	0,843832262	NA
Treml2	9,434527632	0,156666919	0,469796383	0,333478342	0,738773235	0,915522746
Serinc4	2,696094288	0,101188313	1,188436701	0,085144049	0,932146871	NA
Cort	0,086476712	0,780932884	3,352475198	0,232942181	0,815806304	NA
Gja1	62,5959673	0,42128813	0,263941631	1,596141266	0,110457257	0,411062987
Stc1	1,528138287	-0,816171013	1,165393602	-0,700339363	0,483715395	NA
Ptk6	32,21614831	0,877737309	0,382028018	2,29757313	0,021586096	0,163772191
Naip6	11,11800639	-0,089023134	0,46231739	-0,19255848	0,847304764	0,953824301
Itih3	30123,07557	0,457234389	0,151089701	3,026244581	0,002476119	0,043424918
Gjb5	0,728690366	0,453866294	2,289811061	0,198211242	0,842879792	NA
2410015M20Rik	1561,497129	0,080214495	0,101805113	0,787922066	0,430742293	0,761618002
Alg3	415,4801308	-0,216741823	0,103417945	-2,095785442	0,036101228	0,222618691
Mydgf	1642,876165	-0,179129525	0,094250652	-1,900565374	0,057358964	0,289356792
Myh7b	1,677742674	1,005119524	1,202691203	0,835725347	0,403309432	NA
Zfp780b	98,37242894	-0,147942762	0,199663221	-0,74096151	0,458716779	0,779631911
Unc79	1,811177338	2,704256457	1,857000948	1,456249368	0,145323697	NA
Tns3	862,1000589	-0,309920249	0,122378695	-2,532468968	0,011326239	0,113783449
Mrgprb2	0,12663974	0,780932884	3,352475198	0,232942181	0,815806304	NA
Fbxo46	125,1403934	0,011333385	0,15997125	0,070846389	0,943520012	0,983618141
E330009J07Rik	30,92472092	-0,552821074	0,360800887	-1,532205418	0,125471752	0,441081213
Clec1a	20,89347808	0,256361237	0,528671075	0,484916329	0,627735699	0,865852703
Zfp74	113,3247539	0,029838129	0,197749615	0,150888431	0,880063727	0,96546599
Gm13034	0,46326227	-0,11021128	2,506875216	-0,043963608	0,964933412	NA
Amica1	18,46666243	0,52745149	0,348728461	1,512499122	0,130406936	0,448869806
Tubb6	225,1773701	-0,598503649	0,224340297	-2,667838357	0,007634098	0,089154096
Nifk	702,360776	0,03937882	0,175104334	0,224887749	0,8220666	0,946779723
Spata6	25,20716508	0,109925619	0,317583717	0,346131155	0,72924415	0,911594858
Atp5g2	913,9649435	0,027183751	0,093861326	0,289616101	0,772109948	0,928273961
Spryd7	779,9685301	-0,111271817	0,11686772	-0,952117634	0,341037327	0,694913583
Rps19-ps3	13,90462148	0,100540338	0,393447266	0,25553701	0,798308362	0,938579236
Lrrc8b	6,14427733	-0,735533177	0,637632436	-1,153537895	0,248689673	0,60747894
Skil	297,4151263	-0,231966901	0,16716283	-1,387670335	0,165237453	0,503431976
Irs3	4,098496101	1,145776978	0,75827446	1,511032006	0,130780297	NA
Irs1	579,8573503	0,105740774	0,186215048	0,567842259	0,570142082	0,841130547
Mir5119	1,803456746	-0,355769007	1,531608762	-0,23228452	0,816317035	NA
Adgrl1	103,7603608	0,135718561	0,17595037	0,771345695	0,440502054	0,768006255
Ctxn1	12,89826194	-0,223819895	0,453122222	-0,493950382	0,621341222	0,862654966

Tmem145	0,707917963	-0,433259464	2,285352986	-0,189580982	0,849637491	NA
Mir5121	0,393546259	1,035669797	2,561579599	0,404309043	0,685985476	NA
Fam19a3	3,299957395	-0,250697929	0,922882916	-0,271646516	0,785893829	NA
Fcrl5	0,234744367	0,059593471	3,352475198	0,017775962	0,985817581	NA
Supt3	195,6136516	0,311107706	0,15249475	2,040120764	0,041338299	0,240072963
Accs	61,05229784	0,665639183	0,249995441	2,662605284	0,007753831	0,089827526
Fam19a1	0,307069547	0,617382779	2,733615373	0,225848444	0,821319297	NA
Spred3	12,2218087	0,239777303	0,442287806	0,542129582	0,587729241	0,848706754
A430033K04Rik	107,8537673	-0,060022434	0,203889383	-0,294387247	0,768462011	0,927598042
Rims4	2,12568262	1,322429967	1,554626594	0,850641545	0,394968504	NA
Thada	194,2297726	-0,17162002	0,139439218	-1,230787312	0,218402422	0,572626394
Atxn2l	1754,3375	-0,14692977	0,125328731	-1,172355047	0,241054539	0,598562061
Arhgef4	2,307660725	0,159656073	0,940071148	0,169834031	0,865140663	NA
Ttll12	346,9885482	-0,195356714	0,096219202	-2,030329808	0,042323026	0,243048341
Cdc42bpb	1970,808628	-0,27920742	0,083828735	-3,33068869	0,000866314	0,021408051
Pcmtd1	2324,337289	0,031140297	0,115118246	0,270507054	0,786770191	0,934196858
Zfp184	4,138017844	-0,939262123	0,824766893	-1,13882132	0,254777687	NA
Asic3	4,564440648	-0,489972569	0,717694688	-0,682703352	0,494794311	NA
Tmem150c	2,302334689	-0,251764217	0,9029184	-0,278833853	0,780372333	NA
Tppp	382,6024147	-1,266865093	0,477514756	-2,653038626	0,007977074	0,09161985
Piezo2	22,9074261	-0,018208857	0,399858551	-0,045538246	0,963678291	0,988989125
Kif21b	67,09909945	0,262081511	0,278889682	0,93973183	0,347355134	0,699260771
Hmbs	1499,482392	-0,022732845	0,104113911	-0,218345893	0,827159619	0,947537943
Cyth2	451,119478	-0,148633959	0,125384516	-1,185425146	0,235849394	0,593239991
Adat3	15,4466957	-0,041731603	0,425131467	-0,098161642	0,921803941	0,977341679
Nxf1	1394,328172	-0,309821416	0,12160017	-2,547869923	0,010838288	0,110439837
Dlx4	2,757290972	2,119357656	1,109506984	1,910179644	0,056110087	NA
Cyp7b1	4180,916527	-1,268516886	0,545004241	-2,327535808	0,019936765	0,157806168
Cyp7a1	3642,422605	1,422720764	0,175519915	8,105751192	5,24E-16	1,17E-12
Ube2d2b	0,093953095	-0,517475177	3,352475198	-0,154356154	0,877328924	NA
Zswim8	915,4694559	-0,206233698	0,110191702	-1,871590085	0,061263336	0,300010821
D430019H16Rik	24,60587376	-0,341853423	0,349750696	-0,977420279	0,328361127	0,685487182
Nampt	1638,030582	-0,157960612	0,126409897	-1,249590545	0,211449159	0,564073259
Ndfip1	2861,295069	0,034066418	0,093030816	0,366184231	0,714227608	0,906371815
Pkmyt1	47,54995223	-1,025484801	0,41201184	-2,488969251	0,012811405	0,121731107
Utp3	826,885388	0,000796326	0,10126735	0,007863598	0,993725821	0,99822248
Tor3a	226,0334648	-0,202188023	0,22611865	-0,89416783	0,371232102	0,719112906
Sult1e1	21,69215515	5,392531201	1,43876818	3,748019505	0,000178236	0,006967375
Rtn4r	0,113662148	0,780932884	3,352475198	0,232942181	0,815806304	NA
Sigirr	454,5369687	0,151334415	0,135972372	1,11297915	0,265717384	0,626006345
Med12	652,5960136	-0,190349609	0,148077307	-1,285474549	0,198626505	0,548812381
Plekhf2	283,9188835	-0,034627279	0,107915895	-0,320872835	0,748306761	0,919838327
Klhl5	570,8518861	0,100261934	0,204630718	0,489965214	0,624158514	0,863896384
Wdr27	1,06262917	1,825653118	1,549839107	1,177962996	0,238811359	NA
Rmdn2	1489,741849	0,228357085	0,197801072	1,1544785	0,248304048	0,607313725
Lyrm4	212,6296264	0,141950604	0,11888613	1,194004752	0,232476109	0,589440547
Tssc1	293,4175925	-0,282415039	0,119087909	-2,371483741	0,017716826	0,147390323
Nat14	9,367441848	-0,311595378	0,581763836	-0,535604585	0,592231859	0,85107672
Ctif	668,3194368	-0,408613605	0,134802223	-3,031208209	0,002435772	0,043130912
Slc6a7	3,013928207	1,476031238	0,894119345	1,650821276	0,098775074	NA
Gdpd5	20,72294017	0,090842384	0,361177619	0,251517201	0,801414265	0,939799232
Cbarp	39,96732568	0,1581731	0,258251665	0,612476592	0,54022248	0,826407188
Ipcef1	6,190999239	0,026064178	0,741259139	0,035162033	0,971950537	0,992423486
Gm44504	2,283802588	-0,966616192	1,010994725	-0,956104091	0,339019648	NA
Mpdz	570,1184253	0,305019633	0,102335603	2,980581781	0,002877014	0,047898881
Asb15	0,234199685	1,337107854	3,350237755	0,399108348	0,689813372	NA
Grin3b	4,700980864	0,024719212	0,867098094	0,028507977	0,977257006	NA
Sh3bp4	43,2547135	0,03745262	0,286639742	0,130660948	0,896043527	0,97084116
Lbr	241,5934077	-0,003173288	0,161046374	-0,019704185	0,984279352	0,995554769
Errfi1	19612,72565	0,281110437	0,216607535	1,297786971	0,194360548	0,543485916
Opa1	2337,900728	-0,107815068	0,100410989	-1,073737739	0,282940242	0,644404577
Fam118a	59,91125672	0,693274088	0,253201426	2,738033901	0,00618077	0,078322957
Lancl2	162,9648519	0,238325272	0,162686605	1,464934815	0,142938703	0,470144068
Ptcd1	328,1586528	-0,051098637	0,128963866	-0,396224449	0,691939457	0,895338108
Cab39	1492,739828	-0,114135674	0,110131296	-1,036360035	0,300034208	0,660164141
Clmp	31,7938379	1,399943564	0,284282557	4,924479296	8,46E-07	0,000113178

Ano10	325,7534213	0,188131369	0,121419192	1,549436834	0,121276745	0,433515723
Cdk16	805,9788704	-0,137854866	0,090396448	-1,525003134	0,127258316	0,444207944
Asnsd1	865,5604142	0,058886914	0,086973573	0,677066743	0,498363609	0,801990802
Kptn	350,4711769	0,190621557	0,108693965	1,753745543	0,079474118	0,349223642
Chrm3	12,57448899	0,649203636	0,765412635	0,848174705	0,396340682	0,737761616
Dusp16	1001,764384	-0,159702958	0,106397161	-1,501007701	0,13335357	0,454680917
Tlr3	150,5694458	0,031245025	0,187480006	0,166657904	0,86763923	0,961775826
Klk10	0,12663974	0,780932884	3,352475198	0,232942181	0,815806304	NA
Ctdspl	168,9369044	0,381340841	0,196717254	1,938522589	0,052559498	0,276515733
Dnttip1	300,2656139	0,077608431	0,09722852	0,798206444	0,424750698	0,757334234
Atp6v1c2	0,18725647	-1,238536202	3,340198538	-0,370797181	0,710788601	NA
Snapc2	275,6447122	0,070362185	0,118969477	0,591430566	0,554231965	0,832018326
AW209491	338,1829046	-0,114599648	0,13500194	-0,848874082	0,395951362	0,737426273
Smim14	1403,928885	-0,214935345	0,115259505	-1,864794973	0,062210148	0,302651918
Tmem97	2022,911847	0,400784647	0,097673507	4,103309669	4,07E-05	0,002287793
Pycr2	151,7801969	-0,393446418	0,173991837	-2,261292391	0,023741157	0,174393148
Fbxl15	121,9290641	0,07905406	0,207439442	0,381094641	0,703133024	0,900719679
5930403L14Rik	2,127106983	0,834021037	1,187233185	0,702491345	0,482372797	NA
Mapt	44,7544279	0,197572008	0,30865432	0,640107702	0,522102582	0,815322684
Msx1	4,114337819	0,634184794	0,746591696	0,849439925	0,395636545	NA
G6pc	10178,67573	0,092186733	0,39164707	0,23538216	0,813912132	0,944076707
Msn	548,6249371	0,074260965	0,21342542	0,347948079	0,727879177	0,910785759
Ccne2	21,52662532	-0,953714374	0,395751213	-2,409883642	0,015957609	0,138620711
Cplx2	22,49327234	-0,111210496	0,29746294	-0,373863365	0,708505974	0,903128944
Cox7a1	35,92504029	0,020037855	0,3026221	0,066214118	0,947207357	0,985157551
Galnt4	359,4826584	-0,102875218	0,143179049	-0,71850748	0,472444438	0,787019872
Cd84	95,85309403	0,381160237	0,237582465	1,604328153	0,108641739	0,409049888
Synpo2	5,06403649	-0,344480209	0,892281601	-0,386066695	0,699447275	NA
Fblim1	43,18320718	0,325560159	0,29242539	1,113310167	0,265575242	0,626006345
Btbd10	52,26076048	-0,221027341	0,265559329	-0,832308703	0,405234719	0,745094617
Cxxc5	1032,418317	0,075851401	0,11204716	0,676959603	0,498431586	0,801990802
Qprt	3851,148996	-0,181162423	0,134342118	-1,348515467	0,177492649	0,520144503
Pcdhb21	0,621310199	0,02014555	2,03728955	0,009888408	0,99211032	NA
Tmem19	2741,440972	-0,101306095	0,159761758	-0,634107289	0,526010806	0,817607077
Sac3d1	107,6478521	-0,100914564	0,214984237	-0,469404481	0,638780546	0,870969619
Osgep	465,9887482	0,137801779	0,106038694	1,29954241	0,193757849	0,542367814
Ncln	1432,330613	-0,138354057	0,094145372	-1,469578958	0,141675823	0,467670141
Sult1a1	10374,12328	0,845344578	0,187613603	4,505774438	6,61E-06	0,000585503
Pdk2	3019,753973	0,248615523	0,123412488	2,014508634	0,043956159	0,247168435
Pcdhb16	3,594908491	-0,996582209	0,837282579	-1,190257906	0,23394504	NA
Pex12	335,3040093	0,156131952	0,123982294	1,259308468	0,207918936	0,560182483
6330403K07Rik	0,501707199	0,362018723	2,454262335	0,147506123	0,882732547	NA
Emilin1	472,7483303	-0,299042889	0,178361904	-1,676607403	0,093619278	0,380194927
Itgb4	14,23556772	0,761501158	0,386255349	1,97149673	0,048667087	0,264148764
Ier3	16,7896758	0,016172588	0,355250043	0,04552452	0,963689231	0,988989125
Slc6a12	1976,639663	0,434990718	0,103138783	4,217528145	2,47E-05	0,001618665
Erg	86,60117684	-0,022542829	0,226479136	-0,099536009	0,920712699	0,977341679
Snapin	449,2031397	0,265003675	0,108838151	2,434841759	0,014898303	0,13331688
Ercc3	587,2157078	-0,127928187	0,115190326	-1,110580997	0,266748746	0,627072972
Cyp2a12	16933,49213	0,007502793	0,120196505	0,062421058	0,950227526	0,985972877
Crk	1545,475383	-0,143034086	0,113955295	-1,255177187	0,209414445	0,561873081
Cd81	5410,695296	-0,073915063	0,126637218	-0,583675668	0,55943854	0,835284102
Ifi44	82,72964552	0,731303441	0,304888545	2,398592705	0,01645821	0,141954714
Mat1a	64431,99511	0,985599835	0,152665517	6,455942741	1,08E-10	5,99E-08
Rbm42	934,2678242	-0,322973831	0,117825635	-2,741116827	0,006123073	0,077961301
Dpysl4	4,517336243	1,306332098	0,809074983	1,614599544	0,106397444	NA
Serpina11	2006,80046	-0,302041983	0,189675881	-1,592411129	0,111292357	0,412992267
Actr10	805,1374533	0,002835096	0,101371124	0,027967488	0,977688082	0,994348571
Stat3	2647,711434	0,335502612	0,127171205	2,638196372	0,00833483	0,094132604
Eml2	294,9432023	0,23301008	0,138933009	1,677139812	0,093515145	0,380194927
Mir1b	0,086476712	0,780932884	3,352475198	0,232942181	0,815806304	NA
AW011738	77,3412702	-0,470492361	0,230429926	-2,041802332	0,041171143	0,239415837
Ltf	1,941718411	-1,608340662	1,462658362	-1,099601044	0,271505987	NA
Mir7666	1,147542088	0,775416261	1,603015213	0,483723582	0,628582055	NA
Gli2	1,541651298	0,003835843	1,174193944	0,003266788	0,997393485	NA
Ppp1r12b	37,93266752	-0,212942939	0,24098289	-0,883643392	0,376888748	0,722798116

Zfp429	35,54356575	0,006693513	0,286858912	0,023333815	0,981383998	0,994983586
Tma7	759,7144858	-0,064912367	0,101507442	-0,639483816	0,522508239	0,815322684
Hist1h2ae	1,971509084	-0,034091794	1,055695345	-0,032293212	0,974238222	NA
Slfn4	5,651982808	-0,083520318	0,679144841	-0,122978654	0,902124001	0,973248278
Serf1	127,5798021	-0,174547284	0,184926384	-0,94387442	0,345233825	0,69817409
Prf1	3,464032244	-0,243992012	0,903181587	-0,270147239	0,787046982	NA
Pfdn2	700,7428123	-0,034161169	0,097605806	-0,349991161	0,726345331	0,910393262
Rps18	6067,560535	0,058635372	0,117320047	0,499789876	0,61722304	0,860830256
Csf2	0,369958895	0,666831354	3,099090077	0,215170046	0,829634744	NA
Cryz	2041,490415	0,026544189	0,083464814	0,318028495	0,750463322	0,920556312
Pex11a	631,2043935	-0,529629585	0,234686589	-2,256752662	0,024023534	0,175887529
Crygs	1,550530868	-0,522875098	1,07301313	-0,487296085	0,626048522	NA
Per2	393,5405893	-1,424720515	0,258656359	-5,508159629	3,63E-08	1,08E-05
Sdc4	5845,223639	0,139236361	0,144760028	0,961842601	0,336128675	0,69270916
Ppard	1080,890491	-0,15174584	0,26235117	-0,578407333	0,562989144	0,83722511
Syngr3	2,449133375	0,494279583	1,046254012	0,472427897	0,636621396	NA
Pou4f1	0,598920576	0,78479382	2,050019807	0,382822555	0,701851337	NA
Pou2f3	0,240301888	1,389394708	3,349408814	0,414817893	0,67827522	NA
Pstpip2	823,721335	0,473148386	0,183834784	2,573769641	0,010059723	0,105069087
Pon1	23587,48807	0,355519829	0,128243112	2,772233319	0,005567312	0,073257273
Spink4	0,093303375	-0,517475177	3,352475198	-0,154356154	0,877328924	NA
Pole	56,13594296	-0,608443536	0,334491762	-1,81900903	0,068910052	0,321204084
Pold1	107,4603563	-0,485912642	0,175180739	-2,773778933	0,005540932	0,073122991
Junb	496,0185709	-0,584272487	0,241822934	-2,416116939	0,015687022	0,137521182
Trex1	223,1775978	0,126258227	0,11773493	1,072393949	0,283543125	0,644784493
Adam30	0,285269197	-1,521344816	3,321239936	-0,458065315	0,646905517	NA
Serpinf1	22195,0763	0,018134955	0,050049067	0,362343512	0,717095346	0,907650451
Ccl3	3,45110462	1,517954543	0,847536961	1,791018697	0,073290295	NA
Rdh14	524,7756112	0,080839915	0,10219505	0,791035524	0,428923262	0,760782936
Tmem86b	2121,054668	0,317462344	0,109877669	2,889234439	0,00386181	0,057879533
Tox4	741,5958535	-0,056348537	0,087039084	-0,647393497	0,517377304	0,813424627
Arl6ip4	592,7681187	-0,066833972	0,11453855	-0,583506356	0,55955248	0,835327361
Osr1	0,681972889	2,865389576	3,259642699	0,879050203	0,379374056	NA
Tenm1	0,113662148	0,780932884	3,352475198	0,232942181	0,815806304	NA
Oasl2	151,5722751	0,467044634	0,229814154	2,032270973	0,042126231	0,242468387
Papd4	587,2034386	0,005882923	0,100401535	0,05859395	0,95327553	0,986479135
Slc15a4	402,0711823	0,207320708	0,157932898	1,312713883	0,189279378	0,536686321
Enpp3	1953,287007	-0,107563964	0,170473967	-0,630970032	0,528060119	0,81852028
Ugt2b38	548,7250024	-2,95971703	0,842827158	-3,511653607	0,000445328	0,013530887
Acacb	4237,891865	0,55085324	0,276335521	1,993421757	0,046215283	0,25552197
Lurap1	3,435526321	-0,67834957	0,75907224	-0,893656143	0,371505897	NA
Fam173b	266,8301903	0,043006418	0,108075759	0,39792844	0,690682935	0,894655572
Chmp2b	629,948631	0,152767335	0,126093995	1,211535367	0,225690289	0,581069718
Cacna1b	0,68594197	0,298061366	1,958494856	0,152188996	0,879037873	NA
Sh2d5	3,362079632	-0,734168811	0,917415388	-0,80025779	0,423561453	NA
Atad2b	364,7371161	-0,095230279	0,151706929	-0,627725311	0,530183902	0,819425454
Ext2	804,3300608	0,04312362	0,094502969	0,456320265	0,648159693	0,876446263
Phpt1	403,0936844	0,037282901	0,106755386	0,349236727	0,726911596	0,910653449
Nup62	339,132523	-0,057244483	0,143581453	-0,398689959	0,690121667	0,894644908
Rnf19b	699,102052	-0,015402386	0,112292758	-0,137162774	0,890902135	0,968882343
Igsf6	104,6177304	0,318249811	0,237093653	1,342295788	0,179500109	0,522135977
Misp	11,21532319	1,101257372	0,456276985	2,413572037	0,015797005	0,137992723
Fabp12	1,827528491	-0,471698346	1,010296996	-0,466890774	0,640578027	NA
Akap13	1194,894636	-0,503503012	0,115478475	-4,36014599	1,30E-05	0,001040506
Fpgt	386,4520671	-0,035714791	0,149924074	-0,238219188	0,811711095	0,942944131
Lyrm7	71,84431736	-0,039490034	0,221773496	-0,178064714	0,858672154	0,958796604
Tex29	0,220592835	0,059593471	3,352475198	0,017775962	0,985817581	NA
Kbtbd13	1,142298232	0,890518345	1,47414215	0,604092587	0,545782088	NA
Ttc32	258,9622677	0,177192545	0,119070321	1,488133595	0,136715665	0,459349518
Slc19a3	0,91678776	-1,693272241	1,523062902	-1,111754635	0,266243662	NA
Clybl	1151,342238	0,08949346	0,079026354	1,132450828	0,257444946	0,618247078
Pdk3	11,09640177	-0,348855032	0,441824283	-0,789578676	0,429773866	0,761012822
Krt79	1,68324764	-0,85431226	1,232460008	-0,693176456	0,488198822	NA
Mcmbp	651,5543775	-0,01657613	0,093858625	-0,176607428	0,859816759	0,959201442
Dmrt2	10,89327536	0,268857827	0,598574104	0,449163813	0,65331349	0,877997875
Zfp65	153,1723881	0,106277312	0,136506934	0,778548818	0,436245539	0,76575019

Supplementary Table S1: *Serpina1* KO vs. wildtype all DEGs

Camkv	0,086476712	0,780932884	3,352475198	0,232942181	0,815806304	NA
C130050O18Rik	12,21673217	0,126116502	0,422737413	0,298332956	0,765449053	0,926709042
Snhg11	135,5789054	0,354931697	0,401181072	0,884716957	0,376309308	0,722097522
Plxna4	33,29629363	0,043270858	0,426287886	0,101506188	0,919148646	0,976952181
Mcfd2	6196,430584	-0,049904393	0,110101094	-0,453259739	0,65036172	0,876831587
Gpr82	0,270219305	1,389394708	3,349408814	0,414817893	0,67827522	NA
Klhl3	43,57003769	-0,091901957	0,253231348	-0,362916981	0,716666901	0,907405448
Ccdc149	97,34646708	-1,043915361	0,230698021	-4,525029549	6,04E-06	0,000541822
Rslcan18	90,99738689	-0,388448437	0,209565097	-1,853593192	0,063797382	0,30724323
Mamld1	12,10468581	0,27847728	0,398218004	0,699308611	0,484359187	0,79420977
Matk	8,146223287	0,424286597	0,658919573	0,643912572	0,519632129	0,813876985
Adora1	590,5787534	0,275599395	0,270503408	1,0188389	0,308279455	0,667160859
Crkl	714,4480135	0,058214896	0,108974648	0,534205864	0,593199112	0,851502416
Efna2	4,843107109	-0,010397573	0,770316932	-0,013497786	0,989230652	NA
Gm15421	4,457931982	-0,622457803	0,734651886	-0,847282659	0,39683759	NA
Zfp612	175,5518061	0,223408023	0,179911167	1,241768518	0,214321981	0,567643514
Lrfn3	422,0471286	-0,180505111	0,176961159	-1,020026724	0,307715787	0,667059844
Pde6h	0,172953423	1,307385949	3,350719078	0,39018071	0,696402925	NA
Snord23	0,342773458	0,617694053	2,65113499	0,232992305	0,815767381	NA
Gpr182	406,9281392	-0,384763025	0,306566278	-1,25507289	0,209452301	0,561873081
Igfbp2	10338,85053	-0,245193178	0,205033009	-1,195871724	0,231746621	0,58867957
Flt4	336,4580832	-0,177888911	0,294033193	-0,604996017	0,545181643	0,827491596
Efna4	3,84736473	0,629741797	0,741199523	0,849625206	0,395533494	NA
Anxa2	318,5651912	0,070888348	0,221133219	0,320568516	0,7485374	0,919838327
Zfhx3	159,3460365	-0,454752887	0,163166117	-2,787054661	0,005318951	0,070973656
F2rl1	10,99487516	1,298465021	0,546107161	2,377674407	0,017422202	0,146029728
Dock2	75,98149898	0,406451888	0,210186032	1,933772116	0,053141153	0,278511661
P2ry13	84,05876018	0,284181884	0,254353658	1,117270678	0,263878609	0,624609263
Pltp	1287,438257	0,713411493	0,267173088	2,670222133	0,007580108	0,088737711
Lpar5	4,133551065	0,528465285	0,770143218	0,686190923	0,492592721	NA
6430571L13Rik	34,84237142	0,330284724	0,351453256	0,939768569	0,347336284	0,699260771
Prtg	2,749974006	-0,012008511	0,904526436	-0,01327602	0,98940758	NA
Coro2b	59,97290817	-0,20404077	0,256656946	-0,794994146	0,426616908	0,758848669
Usp28	208,5894503	0,22020555	0,155085836	1,419894651	0,155638354	0,490622294
Slc35g1	506,5057249	-0,045129486	0,159102132	-0,283651046	0,776677814	0,930009104
2900005J15Rik	47,54818351	0,205523801	0,36187268	0,567945061	0,570072273	0,841130547
Rnf39	135,8525825	-0,005476837	0,202116797	-0,027097386	0,978382059	0,994429459
S100a14	0,693710118	-0,489079397	1,788087058	-0,273521021	0,784452736	NA
Proser2	507,1696482	0,485266872	0,158051872	3,070301324	0,002138429	0,039817073
Lax1	8,287012741	1,10754678	0,534549495	2,071925593	0,038272381	0,229748297
4831440E17Rik	45,97698971	0,199118066	0,305656648	0,651443596	0,514760176	0,812039132
Cct8l1	0,093953095	-0,517475177	3,352475198	-0,154356154	0,877328924	NA
Pappa2	0,646023773	-2,06535522	1,743462315	-1,184628542	0,236164347	NA
Col17a1	1,450976132	-0,870598251	1,281461444	-0,679379201	0,496897625	NA
Prkaca	1722,898637	-0,006565737	0,088529058	-0,074164769	0,940879278	0,983128178
Inhba	441,9063253	-0,843264186	0,297693082	-2,832663027	0,004616202	0,065195409
Ckmt1	1,889083479	2,806242259	1,525056713	1,840090428	0,065754962	NA
Nf1	411,3364019	-0,140371605	0,125034285	-1,122664919	0,261579832	0,622343615
Nr0b2	963,4881957	-0,366015791	0,254185735	-1,439954099	0,149880386	0,482018493
Psmc2	2789,807803	0,016601951	0,086997572	0,190832348	0,848656944	0,954095425
Psmb5	1464,506072	0,003376279	0,120406918	0,028040572	0,977629792	0,994348571
Tie1	198,0554677	-0,37218715	0,193799818	-1,920472137	0,054798289	0,283546844
Tgtp1	165,8569903	1,08066679	0,267902241	4,033810185	5,49E-05	0,002888528
Nt5e	467,4674167	1,46056554	0,669296748	2,182239109	0,02909189	0,196651683
Mid2	140,0357586	-0,002296083	0,175956206	-0,013049173	0,989588562	0,996712773
2610528A11Rik	0,828974167	0,664652951	1,568845792	0,423657287	0,67181577	NA
Tmem200c	0,093953095	-0,517475177	3,352475198	-0,154356154	0,877328924	NA
Gm5129	1,593096024	-0,566809588	1,215895239	-0,466166467	0,64109635	NA
Ric1	454,8105657	-0,100059737	0,155978648	-0,641496377	0,521200234	0,814845193
Sema4c	93,38177209	-0,046937777	0,214703214	-0,218617023	0,826948391	0,947537943
Prpf4	315,9765759	-0,212018534	0,143946947	-1,47289358	0,140779733	0,466258507
Celf4	6,645977456	-0,208928099	0,583622929	-0,357984733	0,720354738	0,908788588
Tlk2	572,1378519	-0,002368828	0,10526699	-0,022503046	0,982046682	0,995338444
Nr5a2	1008,664348	-0,07988129	0,164238547	-0,486373579	0,62670232	0,865356673
Adck5	925,9954778	-0,092924835	0,185938239	-0,499761833	0,617242788	0,860830256
Tfg	2008,917768	-0,311352351	0,095112239	-3,273525622	0,001062148	0,024868391

Supplementary Table S1: *Serpina1* KO vs. wildtype all DEGs

D16Ertd472e	246,094416	-0,208690469	0,204410803	-1,020936598	0,307284479	0,66646434
Dlg1	1159,253677	0,011105276	0,089191615	0,124510315	0,900911234	0,972526743
Tgfb3	28,72969371	0,461008587	0,275205944	1,675140373	0,093906693	0,380551253
Sprr1a	2,646960493	0,146113212	1,254683632	0,116454226	0,907292561	NA
Kitl	42,39976543	-0,051725947	0,3240568	-0,159620002	0,873180422	0,963097638
Ift88	127,8999521	-0,016659079	0,161665236	-0,10304676	0,917925859	0,976907366
Tg	0,086476712	0,780932884	3,352475198	0,232942181	0,815806304	NA
Parp2	267,4409451	0,227790694	0,161788544	1,407953169	0,159144937	0,495312554
Slc3a1	1714,644405	0,191477825	0,38034667	0,50342974	0,614662167	0,859808165
Tgfbi	975,4343515	-0,105029078	0,147716307	-0,711018846	0,477072557	0,789944745
Gzma	12,96791361	0,864692422	0,538366052	1,606142176	0,108242675	0,40832289
Krt18	6027,089401	-0,128656162	0,134449134	-0,956913285	0,338611025	0,694071514
Tll1	0,791750948	-0,835897787	1,755974491	-0,476030712	0,634052498	NA
Tmem179b	315,6930112	0,097655356	0,112686759	0,866608966	0,386156299	0,730304649
Wfdc2	80,57792761	0,699595502	0,271280283	2,578866012	0,009912523	0,104019243
Msra	4160,268591	0,078305142	0,079266228	0,987875214	0,323213765	0,680801139
Fam174a	188,8787249	0,167991098	0,172515176	0,97377577	0,330167886	0,686792356
Aldh3b1	78,23421951	-0,414448915	0,203499288	-2,036611129	0,041689027	0,241064272
1700011L22Rik	0,213661134	0,059593471	3,352475198	0,017775962	0,985817581	NA
Nrarp	36,71694587	0,496728921	0,455301607	1,090988726	0,275277843	0,636882421
Mir7035	0,295449755	1,732896538	3,280168762	0,528294933	0,597294648	NA
Ube2v2	130,0923013	-0,093542429	0,172514837	-0,542228316	0,587661231	0,848702927
Cobl	532,9901506	0,190827605	0,15988517	1,193529115	0,232662216	0,589477023
Pcnt	264,2120025	0,131377517	0,154721242	0,849123982	0,395812307	0,737426273
Pmm1	65,9299818	0,17729267	0,214708391	0,825737035	0,408953279	0,746898413
Ripk2	113,7929549	-0,061988997	0,160385966	-0,386498883	0,699127229	0,898942204
Arf3	1061,298169	-0,070056481	0,087405354	-0,801512469	0,422835029	0,755522295
Arf2	225,2435178	-0,128297228	0,11809166	-1,08642073	0,277292894	0,638192109
Arf1	5481,623478	-0,061418175	0,083969359	-0,731435557	0,464513153	0,781663619
Rplp0	10511,10217	-0,054927247	0,11655096	-0,471272371	0,637446243	0,870117708
Nfkbie	78,65946886	0,147408267	0,181922989	0,810278392	0,417780191	0,752229411
Kcne1l	0,093953095	-0,517475177	3,352475198	-0,154356154	0,877328924	NA
C4bp	12064,27414	0,043864029	0,105033044	0,417621229	0,676224073	0,889369368
Hpgd	3632,475407	0,353009063	0,135554987	2,60417613	0,009209542	0,100023317
Calm3	1254,573692	-0,029650061	0,10626528	-0,279019275	0,780230033	0,931606698
Calm2	2903,362722	-0,003318829	0,104581962	-0,031734243	0,974683987	0,993185764
Rack1	8049,777984	-0,050150902	0,119506538	-0,419649858	0,67474126	0,888730631
Cacng1	1,224596111	3,689779593	1,753888717	2,103770643	0,035398459	NA
Ptk2	529,3108678	-0,140507621	0,112043222	-1,254048381	0,209824421	0,562378244
B4galnt2	3,019852937	5,000287746	3,159057103	1,582841836	0,113457518	NA
Kcnj9	3,749216365	-0,628775518	0,981300422	-0,640757411	0,521680307	NA
Galc	134,7192064	-0,037369451	0,144790627	-0,258093023	0,796335116	0,937785232
Eddm3b	0,215799707	0,059593471	3,352475198	0,017775962	0,985817581	NA
Rwdd4a	478,6275467	-0,146338842	0,10172626	-1,438555219	0,150276582	0,482480216
Eif4ebp1	1072,602103	0,098761488	0,135315672	0,729859943	0,4654758	0,781663619
Ccng2	475,9867236	-0,280108135	0,22186105	-1,262538583	0,206755052	0,55930965
Arhgap36	5,505309421	-1,214976192	0,919520412	-1,321315086	0,186396326	0,532805747
Tigit	0,447267411	1,200692607	2,506185436	0,479091687	0,631873404	NA
Ndufaf2	300,2973641	0,011706239	0,117861515	0,099321982	0,920882626	0,977341679
Sidt1	2,330615025	0,481696958	0,931302446	0,517229349	0,604996067	NA
Cfap73	0,147722973	0,780932884	3,352475198	0,232942181	0,815806304	NA
Dstyk	269,1799489	-0,093219964	0,121276381	-0,768657211	0,442096833	0,769003232
Tmem71	38,70351036	0,404134045	0,313971293	1,28716878	0,198035467	0,54780388
Fam126b	301,8660379	-0,283434064	0,193389046	-1,465615915	0,142752953	0,470144068
Dolk	300,1952831	0,10357239	0,106676199	0,970904396	0,331595889	0,688274004
Stat4	10,51735447	0,40356379	0,591604137	0,682151737	0,495143009	0,80006558
Cdkn3	3,706157017	-1,496476688	0,771523532	-1,939638425	0,052423648	NA
Fam192a	633,3711462	-0,056295413	0,088042986	-0,639408268	0,522557371	0,815322684
Rbm12b1	117,7224629	0,179373218	0,19006569	0,94374328	0,345300852	0,69817409
Stpg1	1,124334742	-0,082992591	1,363871764	-0,060850729	0,951478089	NA
Ak7	8,114203961	1,144163986	0,685381243	1,669383276	0,095041446	0,38363801
Sacm1l	1669,353031	-0,027791964	0,124295679	-0,223595576	0,823072006	0,947365446
Rasd2	0,960520584	2,450717108	1,561674207	1,56928833	0,11658077	NA
Usp54	146,8556	0,013612965	0,171608937	0,079325501	0,936773724	0,982210769
Iqck	3,987531401	1,525409562	0,95263899	1,601246198	0,1093224	NA
Ncoa2	931,915231	-0,102753002	0,161557865	-0,636013618	0,524767546	0,816659969

Npas3	1,213870103	1,420270529	1,805595108	0,786594139	0,431519491	NA
Zmym1	97,09827431	-0,03248075	0,173637229	-0,18706098	0,851612822	0,95593718
Fam114a1	717,8933524	-0,308309405	0,113595334	-2,714102733	0,006645555	0,081884267
Cpn2	8746,768042	0,041306004	0,075873213	0,544408264	0,58616056	0,848001356
Pcgf6	177,3934732	-0,330624251	0,204244153	-1,618769725	0,105496818	0,403046295
Cab39l	1606,558795	-0,02378467	0,099174559	-0,239826328	0,810464895	0,942435559
Vps53	856,557242	-0,060934593	0,110111061	-0,553392115	0,579994937	0,84530168
Mfsd10	83,53414506	0,152675846	0,250801887	0,608750786	0,542689639	0,826611597
Ebf1	5,553898486	0,366276633	0,635786606	0,576099952	0,564547624	0,837355044
Gdpd1	27,26660103	-0,84099989	0,311875539	-2,696588169	0,007005385	0,084455842
Plxnd1	463,8665239	-0,230328428	0,185061716	-1,244603333	0,213277595	0,56686047
Mir6918	1,293306608	-0,769180855	1,795318673	-0,428436949	0,668333029	NA
St14	20,69407188	0,411968062	0,50644463	0,813451338	0,415959335	0,751551608
Lgmn	1394,241842	-0,033151487	0,152115066	-0,217936906	0,827478272	0,947580409
Pter	2783,80705	0,103066701	0,140267307	0,734787767	0,462468758	0,781516914
Prodh	5057,697611	0,791046777	0,189721605	4,169513407	3,05E-05	0,001890049
Procr	17,81984027	0,186222468	0,354779666	0,524896114	0,599655411	0,85430922
Supt4b	1,284779392	0,72290835	1,247312993	0,579572532	0,562202921	NA
Zeb1	314,2062792	-0,504660054	0,165941907	-3,041185091	0,002356489	0,042400948
Tal1	20,07360125	0,15358463	0,39173786	0,392059705	0,6950141	0,896876786
Wbp5	321,2653987	-0,149005652	0,214253766	-0,695463397	0,486764942	0,795157193
Prkdc	202,1609557	-0,085831449	0,140125386	-0,612533183	0,54018505	0,826407188
Ptger3	4,429971207	0,961897811	0,875613432	1,098541635	0,271968048	NA
Tsn	897,4856966	0,038737426	0,088210403	0,439148045	0,660554269	0,882607568
Ptpn13	35,75959234	0,406920071	0,267204459	1,522879046	0,127788969	0,445014516
Ptpn12	515,9212856	0,105840381	0,15054026	0,703070269	0,482011959	0,793477208
Pstpip1	11,03425853	0,978555908	0,590888144	1,656076396	0,097706349	0,389224129
Psme3	1657,417506	-0,210894427	0,101170761	-2,084539294	0,03711114	0,225928429
Ccdc136	4,368285869	2,109002594	0,918444514	2,296276544	0,021660074	NA
Izumo1r	1,521328649	0,276162445	1,233477722	0,223889285	0,822843454	NA
1600023N17Rik	5,407323888	0,108748179	0,618772855	0,175748141	0,860491813	0,959539164
Smyd4	81,62547968	0,43297784	0,182060159	2,378213015	0,017396773	0,145962115
Gm3414	41,2403885	-0,668942749	0,265118683	-2,523182232	0,011629812	0,115306358
Fam134b	1360,508784	0,674738607	0,200004449	3,373617998	0,000741872	0,019409183
Rere	586,6473114	-0,146087946	0,124593525	-1,172516354	0,24098981	0,598512498
Rps6kb1	763,692852	-0,170103255	0,122486435	-1,388751784	0,164908242	0,50334664
Mir6921	0,782801372	2,158153148	1,97346328	1,093586676	0,274136296	NA
Rasal3	22,91615194	0,497544747	0,38405973	1,295487938	0,19515196	0,544259529
Timd4	188,6054696	0,185383944	0,232805356	0,796304463	0,425855092	0,758357009
Zfp532	71,49347488	0,296199611	0,184963044	1,601398876	0,109288602	0,409171591
Rasgrp3	131,3191854	-0,33521673	0,29671464	-1,129761343	0,258576801	0,619629549
Zfp870	165,0679029	0,003531231	0,15912792	0,022191146	0,982295481	0,995338444
Tmem252	0,624183341	2,709031228	1,982595319	1,366406549	0,171811394	NA
Cyp2ab1	0,595583791	1,671936108	2,062354547	0,810692861	0,417542074	NA
Zfyve1	1946,529964	-0,299082061	0,114274203	-2,617231649	0,008864616	0,097781395
BC003965	628,2776592	0,061265051	0,107189043	0,571560759	0,567619586	0,839343684
Zfp598	1325,758124	-0,161944273	0,127447201	-1,270677356	0,203843454	0,556841672
Sprn	29,64784169	0,619761004	0,356471464	1,738599205	0,082105286	0,354079608
Ccin	0,087021394	-0,517475177	3,352475198	-0,154356154	0,877328924	NA
Tmem158	9,525727485	-0,162944434	0,457405913	-0,35623596	0,721663864	0,909525085
Ceacam19	0,12663974	0,780932884	3,352475198	0,232942181	0,815806304	NA
Slamf9	14,67145739	0,876128947	0,434949597	2,014322931	0,04397564	0,247168435
Akr1c6	29647,27455	-0,011208653	0,112684082	-0,099469711	0,920765336	0,977341679
Klrb1b	1,622168889	1,004286973	1,426373969	0,704083919	0,481380514	NA
Lhpp	1335,063087	0,7476623	0,219112248	3,412234169	0,000644327	0,017472642
Fam209	2,320338691	-1,396976452	1,06304283	-1,314129979	0,188802467	NA
1700019B03Rik	1,331536803	0,412772243	1,410507208	0,292641002	0,76979657	NA
1700018B08Rik	0,093303375	-0,517475177	3,352475198	-0,154356154	0,877328924	NA
Gm14207	9,573340933	-0,531677453	0,545912611	-0,973924109	0,330094222	0,686792356
Yipf1	791,4978741	0,011130436	0,091617503	0,121488095	0,903304444	0,973489035
Nfya	244,270781	0,086476752	0,150619974	0,574138669	0,565873968	0,837781736
Trap1a	0,087021394	-0,517475177	3,352475198	-0,154356154	0,877328924	NA
Cpq	1679,584379	0,133311476	0,087699345	1,520096598	0,1284867	0,445818502
Lamc1	435,5206425	-0,241397232	0,106690658	-2,262590149	0,023660966	0,174091059
Jtb	1028,626154	0,036618036	0,102777293	0,356285278	0,721626934	0,909525085
Popdc2	4,073362668	0,01541284	0,720870015	0,021380887	0,98294182	NA

Supplementary Table S1: *Serpina1* KO vs. wildtype all DEGs

Cyp2c54	6994,66163	0,995120885	0,189079617	5,262972829	1,42E-07	2,87E-05
AB124611	79,55536063	0,294586035	0,332501029	0,885970297	0,375633533	0,721655743
Churc1	421,0419686	0,250351176	0,125178869	1,999947577	0,045505925	0,2528931
Tlr13	37,97123483	0,52229562	0,370882416	1,408251236	0,15905669	0,495312554
Tlr11	7,029289122	-0,302668153	0,568531892	-0,532367941	0,594471185	0,852263143
Ccdc122	111,8648196	0,600195608	0,153751116	3,903682939	9,47E-05	0,0043525
Akap9	694,7463853	-0,161272344	0,131046738	-1,23064753	0,21845472	0,572651207
Atg2a	1099,248534	-0,091807958	0,108647648	-0,845006403	0,398107264	0,739515911
Rnf31	373,9530863	-0,028014778	0,103316539	-0,271154827	0,786271955	0,934050588
Fam160b2	241,1562009	0,199142292	0,11089881	1,795711715	0,072540378	0,330761361
Sh3tc1	111,5302372	-0,147243145	0,263146694	-0,559547767	0,575787941	0,843861981
Sun2	1317,191988	0,53316539	0,128696871	4,142799942	3,43E-05	0,002028064
Igsf3	10,36693282	-0,223444852	0,442913128	-0,504489116	0,613917709	0,859682163
Astn2	28,81441872	-0,423382573	0,276114059	-1,533361154	0,125186895	0,440427261
Bms1	719,4246942	-0,200211409	0,080701513	-2,480887912	0,013105558	0,123354086
Prr14l	355,6245155	-0,278662766	0,167382175	-1,664829402	0,095946796	0,385586553
Gbp6	301,3195057	0,202542817	0,213157408	0,950203038	0,342009095	0,695250579
Naif1	61,3905748	-0,41132285	0,265736326	-1,547860829	0,121655807	0,433672149
Tbc1d2b	665,2005354	-0,341178676	0,149709122	-2,278943804	0,022670406	0,169224265
Slc23a3	2,419717362	2,000901598	1,036357125	1,930706654	0,05351934	NA
Morn2	126,9439361	0,01215723	0,1407975	0,086345493	0,931191775	0,980584977
Onecut2	1248,971292	0,002279933	0,157286637	0,014495402	0,988434747	0,996575327
Arid4b	398,5703387	-0,077895329	0,098673323	-0,789426431	0,429862813	0,761019227
Ifi27	1435,145072	0,333060181	0,161043725	2,068135105	0,03862732	0,230668679
Ntf5	1,59481191	0,985644307	1,408726232	0,699670585	0,48413305	NA
D630045J12Rik	77,6125326	-0,313019094	0,21148402	-1,480107545	0,138844548	0,463011416
Nanos3	0,295445946	1,389394708	3,349408814	0,414817893	0,67827522	NA
Nlrp9b	0,43629734	2,237934017	2,448793276	0,913892585	0,36077329	NA
Snord68	0,773608479	-0,551967082	1,519310902	-0,363300942	0,716380089	NA
0610039K10Rik	2,749039543	0,122117015	0,944362093	0,129311644	0,897111058	NA
Hykk	1448,252629	0,142064185	0,194155337	0,731703732	0,464349418	0,781663619
Lrrc39	11,97892154	-0,333354987	0,443525597	-0,751602587	0,452290087	0,774915567
Ust	17,44357984	0,556934885	0,378577154	1,471126504	0,141256909	0,466979132
Taf7	120,0760631	0,071700902	0,206110837	0,347875461	0,727933715	0,910785759
Mex3b	8,379085642	0,049178537	0,691188236	0,071150715	0,943277806	0,983519029
Arl11	5,237221629	0,39804063	0,629906462	0,631904344	0,527449385	NA
Exoc3	806,3103915	-0,085003956	0,097389254	-0,872826853	0,38275746	0,727168465
D330045A20Rik	1,53648861	1,783243125	1,415323154	1,259954746	0,207685688	NA
Myo3b	0,086476712	0,780932884	3,352475198	0,232942181	0,815806304	NA
Rab26	0,180324769	-1,201310802	3,342952034	-0,359356278	0,719328578	NA
Cndp1	0,874493276	-0,876234674	1,573641727	-0,556819674	0,57765064	NA
Srrm1	1306,961618	-0,124573323	0,086005774	-1,448429762	0,147496896	0,47753781
Tox2	14,32868211	0,787843735	0,981658766	0,802563744	NA	NA
Pot1b	82,18805084	0,209281249	0,226314965	0,924734469	0,355104045	0,705239841
Dfnb59	0,633354772	-2,860932266	2,602710545	-1,099212616	0,271675337	NA
Ash2l	502,7603185	0,198848304	0,097899289	2,031151664	0,042239612	0,24277789
Mir7028	0,367686277	-1,151145211	2,581576892	-0,445907776	0,655663865	NA
Bahd1	334,5729243	0,172726133	0,121235673	1,424713773	0,154239956	0,489157561
Shank2	533,1426535	-0,015245435	0,18283992	-0,083381328	0,933548335	0,981500765
Snai2	92,23463583	0,459493163	0,270290436	1,699997858	0,089131328	0,369946206
Slpi	52,92348907	1,953905142	0,465713869	4,195505595	2,72E-05	0,001725067
Smim17	0,37991922	2,003317719	3,326247913	0,602275528	0,546990748	NA
Trove2	94,96543572	-0,047802964	0,264546408	-0,180697836	0,856604757	0,9578959
C4a	614,7434321	-0,696822849	0,491547291	-1,417611005	0,156304365	0,491562705
Arl9	1,165272521	1,994150001	1,525058534	1,307589156	0,191012697	NA
Acss1	8,937798946	0,515796709	0,514050223	1,003397501	0,315669109	0,67419376
Tomm6	1496,248263	-0,006229174	0,097273175	-0,064037938	0,948940019	0,985650844
Cebpd	87,19284747	0,440106495	0,191522563	2,297935485	0,021565461	0,163772191
Mir5100	1,386276493	-0,975894676	1,409665545	-0,692288096	0,488756424	NA
Sbno1	1005,513901	-0,060345155	0,096173115	-0,627463869	0,530355213	0,81950056
Pgap3	45,5739419	0,093405731	0,234421027	0,39845287	0,690296392	0,894644908
Layn	12,15408815	0,254855653	0,620297131	0,410860602	0,681174753	0,89166825
Ddx17	3755,114546	-0,089699006	0,109182422	-0,821551713	0,411332081	0,748889908
Cd37	44,49686886	0,690040831	0,249148586	2,76959562	0,005612593	0,073470343
Ms4a1	13,71228598	0,689823443	0,466958647	1,477268807	0,139603596	0,464512269
Ankrd50	118,3963249	-0,072862336	0,168578418	-0,432216275	0,665584232	0,884945574

Gm20219	1,467653405	-0,501118724	1,19306857	-0,420025082	0,674467131	NA
Mirc35hg	0,12663974	0,780932884	3,352475198	0,232942181	0,815806304	NA
4930447M23Rik	0,087021394	-0,517475177	3,352475198	-0,154356154	0,877328924	NA
E230013L22Rik	18,65900926	0,191075604	0,330329729	0,578439017	0,562967758	0,83722511
2500002B13Rik	27,79326606	-0,261993602	0,298619155	-0,877350289	0,380296405	0,725866642
Gm12992	67,46678237	-0,022732276	0,371490425	-0,06119209	0,951206229	0,985972877
1700066J03Rik	0,093953095	-0,517475177	3,352475198	-0,154356154	0,877328924	NA
Snrnp70	2016,345952	-0,091675918	0,124058969	-0,738970496	0,459924913	0,780953195
Bbs1	28,8235745	0,324656141	0,279897848	1,159909385	0,246085701	0,605518092
Hint3	359,9508061	0,261010588	0,124754587	2,092192304	0,036421317	0,223663557
2810428I15Rik	543,2060379	0,066851867	0,146165503	0,457371028	0,647404385	0,876230372
1700020D05Rik	2,026010147	-0,643912826	0,989919375	-0,65046997	0,515388693	NA
Qsox1	11401,39489	0,071576712	0,086764097	0,824957727	0,409395591	0,746907036
Gm9958	27,25675496	0,366489908	0,347759282	1,053860895	0,291946609	0,653884104
Tarbp1	68,42185989	-0,458349521	0,261756281	-1,751054524	0,079936518	0,34992512
Nr3c1	1626,913708	-0,038317796	0,131422666	-0,291561548	0,770621879	0,927934035
Bckdha	6309,714329	0,048108761	0,09460221	0,508537388	0,611076526	0,858645358
Gm19619	39,27023479	0,744943271	0,266565509	2,794597368	0,005196439	0,07004174
Mafa	0,113662148	0,780932884	3,352475198	0,232942181	0,815806304	NA
Ghdc	432,028664	-0,024526937	0,101005183	-0,242828498	0,808138258	0,942196162
Pard3	1818,449435	0,02609949	0,092080024	0,283443564	0,776836838	0,930019851
Supt16	795,3157429	-0,063065225	0,098797579	-0,638327636	0,523260426	0,815322684
Atp6v0b	1365,300089	-0,164312296	0,102607046	-1,601374397	0,10929402	0,409171591
Pip4k2b	595,7796081	-0,091336334	0,122075831	-0,74819343	0,454343493	0,776572096
Adam11	393,6568241	0,051189286	0,221261673	0,2313518	0,817041508	0,944998904
Rnf111	500,5751164	-0,039473861	0,110742672	-0,356446714	0,721506051	0,909525085
Pabpc5	0,18725647	-1,238536202	3,340198538	-0,370797181	0,710788601	NA
Rnase2a	0,249136072	1,389394708	3,349408814	0,414817893	0,67827522	NA
Setbp1	9,483791103	0,616676085	0,614964034	1,002783986	0,315965097	0,67419376
Clec2d	2948,850662	-0,078895572	0,093647946	-0,842469855	0,399525006	0,740605908
Gpnmb	7,561100214	-0,439608495	0,834295438	-0,526921849	0,598247861	0,853846018
Lmod2	1,288166174	-1,223104252	1,414440529	-0,864726531	0,387188904	NA
Hnmt	617,7236665	0,270277863	0,098370014	2,747563532	0,006003988	0,077031967
Nat8f2	27624,58839	0,146382296	0,124213607	1,178472307	0,238608366	0,595935251
Lmod1	5,920652294	0,287673359	0,746485879	0,385370128	0,699963211	0,899228386
Spock2	8,404374687	0,953064482	0,631197581	1,509930503	0,131061158	0,450194405
C1galt1	408,9677427	-0,300131748	0,203863827	-1,472216787	0,140962346	0,466511942
Sfxn4	4,030273149	-0,54660396	0,844373075	-0,647348876	0,517406176	NA
Mir6919	0,086476712	0,780932884	3,352475198	0,232942181	0,815806304	NA
Nelfe	728,4860172	-0,090370317	0,144681203	-0,624616846	0,532222561	0,820489323
Cdc26	318,8824301	-0,080404299	0,124256997	-0,64708066	0,517579741	0,813424627
Plac8	148,0488344	0,604241838	0,216596944	2,789706203	0,005275589	0,070741574
Slc39a6	65,4429247	-0,007541074	0,188708142	-0,039961574	0,968123761	0,991231429
Oxa1l	1215,704592	0,023442506	0,087550286	0,267760477	0,788883695	0,935202374
1700001L19Rik	1,589997266	-0,455601874	1,293041334	-0,352349041	0,724576514	NA
Sult1c2	108,1122253	0,568367566	0,260554898	2,181373563	0,029155797	0,196960008
Myb	1,247176808	0,869574372	1,541240488	0,56420421	0,572615163	NA
Me1	5929,489834	0,667533816	0,351752176	1,89773898	0,057730475	0,290395259
Zfp664	568,8142342	-0,17628225	0,124186506	-1,41949601	0,155754459	0,490700453
Gm7609	12,10936732	-0,711556167	0,573207555	-1,241358667	0,214473281	0,56766844
Klhl14	0,720872226	2,045567525	2,226874497	0,918582313	0,358314087	NA
Gm1966	57,37730309	0,147162177	0,287003231	0,512754426	0,608123113	0,857753956
Actr3b	5,921951988	0,31630357	0,567046706	0,557808673	0,57697504	0,844928702
Ext1	1061,211593	0,069503662	0,128591449	0,540499873	0,588852348	0,849047352
Phf1	462,7296184	-0,08826265	0,109594903	-0,805353601	0,420615659	0,75436738
Vcp	7011,35236	-0,031708807	0,09766482	-0,324669687	0,745431079	0,918664094
Evi2a	26,23719055	0,159660614	0,325777045	0,490091663	0,624069037	0,863867171
Tial1	1078,088629	-0,06740416	0,070485093	-0,956289584	0,338925951	0,694071514
Tgm2	4343,830171	-0,275030254	0,11209665	-2,453510011	0,01414696	0,129462863
Fkbp5	557,1208823	0,230988482	0,211670401	1,091264918	0,27515633	0,636861537
Hck	129,9245995	0,039554215	0,232940655	0,169803829	0,865164416	0,961775826
Cdyl2	41,11315627	0,287403078	0,279359178	1,028794115	0,303576433	0,66325429
Mir135a-1	1,912499743	0,882419908	1,699670984	0,519171014	0,603641491	NA
Mat2b	678,2349733	0,154940555	0,075806768	2,043888151	0,040964598	0,238807265
2410004P03Rik	0,486199913	0,320550957	2,464466492	0,130069107	0,896511751	NA
Prkca	255,5626867	0,708708144	0,195101748	3,632505348	0,000280683	0,009597053

Coq7	566,7382834	0,035718205	0,19411228	0,184007961	0,85400721	0,957017803
Nisch	2683,950773	-0,00726359	0,124221645	-0,058472821	0,953372011	0,986479135
Pidd1	61,23821798	-0,174602086	0,219412924	-0,79576938	0,426166092	0,758368511
Thop1	270,7499621	-0,183593439	0,114388091	-1,605004838	0,10849274	0,40879949
Lime1	182,4143967	-0,049175133	0,179560309	-0,273864162	0,784189014	0,933246486
Timm8a1	115,6904336	0,22540353	0,164102189	1,373555902	0,169579595	0,509233964
B4galt1	1198,855053	-0,08250576	0,19655604	-0,419756933	0,674663029	0,888715148
Perp	1977,826252	0,070993515	0,136253886	0,521038461	0,602339979	0,854608409
Sv2a	1,085025561	2,708036243	1,597560801	1,695106841	0,090055155	NA
Glrx2	513,6991797	0,069443988	0,094399852	0,735636619	0,461951871	0,781516914
Ndc80	7,662709755	-0,722987845	0,584344355	-1,237263334	0,215989335	0,569610871
Ube4b	1244,769019	0,159922665	0,121338934	1,317983104	0,187509304	0,533818545
Mxra8	135,6234635	-0,209311058	0,186005651	-1,125294084	0,260464436	0,621964694
Rbp7	0,281209566	-1,718135424	3,30990564	-0,519088944	0,603698718	NA
Dusp10	132,6273256	-0,16889994	0,141235233	-1,195876809	0,231744636	0,58867957
Fam129a	58,70398375	0,188433989	0,281018675	0,670539027	0,502514236	0,80422495
Thoc7	728,6152246	0,002439902	0,096219392	0,025357691	0,979769658	0,994664228
Hacd2	1576,451532	-0,120179285	0,084345783	-1,42484047	0,154203321	0,489157561
Rmnd5b	339,760298	0,046697254	0,107135265	0,435871929	0,662929653	0,883542125
Rmnd1	4,634139015	-0,130025545	0,740187149	-0,175665769	0,860556529	NA
Epha1	995,7511627	-0,06731789	0,08291889	-0,81185229	0,416876394	0,752195715
Muc2	1,008161121	1,01711864	1,707140626	0,595802492	0,551307178	NA
Anapc4	449,5275792	0,093639119	0,097580861	0,95960537	0,337253866	0,69270916
Ripk4	128,647277	-0,392296279	0,160241204	-2,448161075	0,014358745	0,130320475
Pcm1	495,2691653	-0,10563469	0,132090878	-0,799712227	0,423877547	0,756584637
Rps23	3442,684656	-0,013090918	0,136645414	-0,095802102	0,923677748	0,977937985
Wnt2	45,36566196	-0,518026656	0,403098341	-1,285112349	0,198753027	0,548812381
Pex13	1471,506434	-0,074916829	0,091209361	-0,821372148	0,411434323	0,748947472
Sep-10	326,155365	-0,010647022	0,104646411	-0,101742827	0,918960807	0,976907366
Idnk	548,6773313	0,050353852	0,126397267	0,398377693	0,690351798	0,894644908
Rpl38	1466,001873	0,061693301	0,132014358	0,46732266	0,640269046	0,871049783
Islr	82,94114425	-0,029290678	0,274468742	-0,106717719	0,915012926	0,976451566
Gipc2	5,558350247	0,932905287	0,824519743	1,13145294	0,2578645	0,618915636
Lypla2	1464,460301	0,057423575	0,097667777	0,587948005	0,556567193	0,833822555
Specc1l	450,2423902	0,013972125	0,102759101	0,135969705	0,89184523	0,969104575
AI987944	346,1214178	0,085256453	0,109659972	0,777461931	0,436886286	0,76575019
Gm14440	0,295449755	1,732896538	3,280168762	0,528294933	0,597294648	NA
Ctla2b	78,72563874	0,494512205	0,282037777	1,753354496	0,079541177	0,349223642
Tinagl1	454,2297775	-0,233222815	0,275520009	-0,846482314	0,397283736	0,738461552
Kras	806,4129149	-0,113933743	0,135914209	-0,838276904	0,401875204	0,742183948
Birc3	354,3035303	0,196219597	0,147116816	1,333767292	0,182280133	0,527224185
Gpr143	0,37991922	2,003317719	3,326247913	0,602275528	0,546990748	NA
Cyp1b1	14,83416849	0,529487388	0,543262589	0,974643569	0,329737094	0,6866113
Prkce	275,5301655	0,020176174	0,173592117	0,116227481	0,907472258	0,9747592
Prkcg	4,809030325	0,101406833	0,839119097	0,120849154	0,903810516	NA
Plk4	20,63385708	-0,690382801	0,41484971	-1,664175685	0,096077326	0,385586553
Sema7a	3,498248469	0,222876995	0,964417629	0,231100084	0,81723705	NA
Sema6c	30,79275066	0,142686603	0,284703355	0,501176402	0,616246981	0,860428813
Pds5b	388,2898762	-0,316275932	0,125743319	-2,51525039	0,011894787	0,116510842
Sema3f	94,25651445	0,306396098	0,296201219	1,034418762	0,300940437	0,661404508
Sema3e	0,855798509	1,471009319	1,769653645	0,831241369	0,405837289	NA
Selp	23,48701024	0,603218773	0,346229517	1,742251146	0,081464508	0,353259489
Sele	1,033803007	0,89511433	1,634414061	0,547666807	0,583920708	NA
Sel1l	4467,392394	-0,566754759	0,142851689	-3,967434768	7,27E-05	0,003544756
Vnn3	1791,524427	0,221400367	0,182784016	1,211267657	0,22579284	0,581069718
Tgfbr3	161,0580418	0,340889224	0,178742809	1,907149312	0,056501255	0,287320381
Lrrc41	734,8079736	-0,010338725	0,073259476	-0,141124747	0,887771394	0,968360648
Cdk5rap3	1755,268193	-0,282127067	0,108864318	-2,591547643	0,009554531	0,101780499
Tmem101	131,5146768	-0,493961768	0,150991357	-3,271457251	0,001069948	0,025000318
Fam204a	99,10218359	0,151274859	0,175445067	0,862234894	0,388558272	0,732257617
Ifitm2	4586,238558	0,096243333	0,099913654	0,963265067	0,335414519	0,691785978
Gatc	609,4954058	-0,046706065	0,090975377	-0,513392377	0,607676877	0,857689206
Gpbp1l1	1010,274909	0,030575516	0,08360485	0,365714621	0,714578035	0,906371815
Plet1	115,1327464	-0,03227759	0,191708129	-0,168368394	0,866293471	0,961775826
Commd9	401,7459234	-0,041171415	0,114849182	-0,35848244	0,719982307	0,908788588
Ip6k2	664,5426889	0,681584546	0,487675155	1,397619992	0,162227212	0,498749809

Supplementary Table S1: *Serpina1* KO vs. wildtype all DEGs

Arfip2	315,7249945	-0,179422018	0,129131779	-1,389448979	0,164696265	0,50334664
Actrt3	0,12663974	0,780932884	3,352475198	0,232942181	0,815806304	NA
Pkd1l2	0,122496332	0,780932884	3,352475198	0,232942181	0,815806304	NA
Ppp1r11	724,4302696	-0,033323345	0,104910375	-0,317636319	0,750760821	0,920556312
Fahd2a	416,1190412	0,223843082	0,108494708	2,063170499	0,039096432	0,232302311
Ppp1r3g	11,69668604	0,329523037	0,653767223	0,504037255	0,614235197	0,859778545
Ly6k	0,716207464	0,30497162	1,985703471	0,153583667	0,877938016	NA
Lmf1	1067,899153	-0,19255919	0,123751075	-1,556020342	0,119703255	0,430423029
Myh7	0,086476712	0,780932884	3,352475198	0,232942181	0,815806304	NA
Mgam	2101,094013	0,214595324	0,092756011	2,313546277	0,020692624	0,160630236
Slc3a2	1157,717994	0,041870033	0,084349668	0,496386453	0,619621781	0,861989968
Abcc1	40,20113345	0,068871555	0,248353255	0,277312873	0,781539876	0,932196631
Gm10416	4,766219342	0,352638199	0,674391209	0,52289857	0,60104484	NA
Ffar4	4,518501736	-1,868386772	0,742256877	-2,51717004	0,011830172	NA
Gprc5a	5,383365047	0,824073186	0,712729278	1,156221881	0,247590412	0,607123298
Clptm1	2406,11281	-0,172413501	0,078160677	-2,205885458	0,027392021	0,190830602
Gosr2	1709,509904	-0,328422529	0,092463596	-3,551911708	0,000382443	0,012115836
Metap2	1485,094503	-0,042105989	0,072929575	-0,577351353	0,56370213	0,837355044
Rpl21	6743,82046	0,008635187	0,093545894	0,092309633	0,926452035	0,978800163
Ctsh	6607,62156	-0,021134077	0,086479548	-0,244382373	0,806934693	0,941758461
Fam60a	8,264809158	0,005638415	0,539951334	0,01044245	0,991668282	0,997337942
Sh3bgrl	1739,491364	-0,084343783	0,221712325	-0,38041991	0,703633738	0,900719679
Ubc	2316,170174	0,648840695	0,381561003	1,700490067	0,089038783	0,369687145
Bloc1s6	331,0347065	0,030596595	0,098385763	0,310986	0,755811265	0,922554343
Styx	29,9155092	0,213688526	0,295616627	0,722856925	0,46976779	0,784373852
Stk3	233,8248502	-0,331470795	0,165337768	-2,004809908	0,044983373	0,251414178
A630023P12Rik	0,616270549	-0,270434398	2,033618422	-0,132981879	0,894207712	NA
Tbc1d2	52,02948854	-0,03603752	0,262404508	-0,137335749	0,890765414	0,968877905
Adgrl3	2,861369608	-0,951749027	0,869870172	-1,094127672	0,273898988	NA
Stpg2	0,709381075	1,976712339	2,004604985	0,986085715	0,324091053	NA
Nlgn2	49,05464203	0,027678961	0,25349424	0,109189704	0,913052027	0,976136949
Zfp174	61,66291294	-0,225553019	0,197091216	-1,144409292	0,252453937	0,612043137
Wfdc17	316,3103222	0,18374909	0,232581286	0,790042451	0,429502977	0,760857915
Il33	41,21811744	0,660811696	0,259943947	2,542131498	0,011017871	0,111843525
Msl3l2	23,82453736	0,303529947	0,294914945	1,029211822	0,303380148	0,663017899
Fance	226,6388855	-0,239690765	0,134179557	-1,786343393	0,074043675	0,335476973
Cnot10	470,0194983	-0,139349376	0,098397154	-1,416193156	0,156718959	0,492287539
Sh2d4a	266,5403606	1,368962429	0,235852061	5,804326772	6,46E-09	2,40E-06
Oraov1	274,1247104	-0,217007008	0,142680273	-1,520932098	0,128276878	0,445402266
Slc35b2	684,1235709	0,198421186	0,140980147	1,407440623	0,159296773	0,495312554
Faap100	129,8705081	0,130706186	0,204514077	0,639106061	0,522753938	0,815322684
Gm6583	0,241026348	0,059593471	3,352475198	0,017775962	0,985817581	NA
Fbxo39	0,113662148	0,780932884	3,352475198	0,232942181	0,815806304	NA
Ehmt1	519,8537692	-0,328211349	0,105090728	-3,12312376	0,001789425	0,035546539
Dhdds	850,5283619	-0,446761647	0,115914738	-3,854226443	0,000116096	0,005055664
Ppil4	378,17383	-0,031243571	0,127182435	-0,245659485	0,805945837	0,941267572
Soga3	0,259430135	1,627812584	3,345388605	0,486584005	0,626553161	NA
Snrnp48	328,3626865	-0,151305691	0,128168751	-1,180519352	0,237793718	0,594996111
Nkap	200,1717686	-0,033139695	0,121397857	-0,272984183	0,784865374	0,933362852
Srpr	5308,610577	-0,339546434	0,121424079	-2,796368204	0,005168048	0,070015871
Erp29	1595,596549	-0,504524763	0,121033247	-4,168480772	3,07E-05	0,001890049
4833420G17Rik	520,6490521	0,051814676	0,119287891	0,434366605	0,664022244	0,884106502
Fundc2	126,6378298	0,164458633	0,156640965	1,049908194	0,293760324	0,654983618
Fam132a	23,1060583	0,294634516	0,317206051	0,928842671	0,352970627	0,703992885
Ppid	1197,755969	-0,321272295	0,130694788	-2,458187505	0,013964025	0,128659575
Unc50	510,414321	-0,060233106	0,085062599	-0,708103281	0,478881122	0,79118575
Bag4	642,9951765	0,064990774	0,118463022	0,548616546	0,583268628	0,846950064
Med4	331,8679873	0,10727511	0,117882074	0,91002055	0,362811672	0,711937362
Carnmt1	121,4439491	0,024306268	0,165315726	0,147029374	0,883108835	0,966395158
Bbs2	58,07747839	0,165772849	0,206782938	0,801675663	0,422740597	0,755522295
Mir7026	1,117078865	1,88945608	1,447714887	1,305129966	0,191848597	NA
Arl4c	47,70784882	0,132135417	0,255186336	0,517799732	0,604598005	0,855783031
Wscd2	0,860512613	-0,488716663	1,872047923	-0,26105991	0,794046307	NA
Rassf4	129,9412865	0,390168946	0,276259011	1,412330208	0,157852756	0,493680148
Ltk	3,754051274	0,541816308	0,720404787	0,752099816	0,451991035	NA
Mrc2	40,36264135	0,086292709	0,392489973	0,219859652	0,82598046	0,947537943

Supplementary Table S1: *Serpina1* KO vs. wildtype all DEGs

Mrc1	689,3281052	-0,07351915	0,243799875	-0,301555322	0,762991071	0,925557106
Nsd1	1487,559855	-0,241085365	0,098309585	-2,452307838	0,014194317	0,129557379
Nrtn	11,24096705	-0,081771515	0,475805616	-0,171859079	0,863548318	0,961272625
Nrp1	1619,203376	-0,235193908	0,227490939	-1,033860551	0,30120136	0,661643276
Lypla1	3237,415088	0,064967212	0,169733474	0,382760163	0,701897602	0,900259931
Rasd1	2,914239234	2,280293609	1,051039682	2,169559959	0,030040198	NA
Pkia	20,11534235	0,551988154	0,387420531	1,424777755	0,154221455	0,489157561
Mir7030	0,561654019	0,53267019	2,413966842	0,220661767	0,825355803	NA
Mir7240	0,483145869	-1,518874621	2,364656598	-0,64232355	0,520663126	NA
Hspa2	53,97503751	-0,117630958	0,292405966	-0,40228645	0,687473225	0,893386271
Endod1	91,65699639	0,16040709	0,247662971	0,647682975	0,517190018	0,813424627
4930515G01Rik	6,106247369	-0,077687851	0,656979572	-0,118250026	0,905869553	0,974066601
Zp3r	0,086476712	0,780932884	3,352475198	0,232942181	0,815806304	NA
Slc30a1	822,2899363	-0,147606051	0,156103694	-0,945564112	0,344370956	0,697519845
Bcl2a1a	2,261148876	1,465306027	1,038855426	1,410500432	0,158391969	NA
Itgb1	4105,563602	-0,032029001	0,063128655	-0,507360743	0,611901725	0,858888222
Zik1	13,88134374	-0,255947354	0,401931493	-0,636793479	0,524259374	0,816308359
Cdc25c	1,163183831	-1,6331404	1,806549204	-0,904011026	0,365989555	NA
Aldh1a7	7699,472483	0,408891759	0,10633352	3,845370293	0,000120371	0,005191076
Zfp148	745,8725678	-0,34649231	0,126262143	-2,744229597	0,00606531	0,077595339
Eif2s3y	1119,544718	-0,352321624	0,877175122	-0,401654829	0,687938072	0,893697803
Eif2s3x	463,1756212	-0,003064168	0,166217194	-0,018434725	0,985292051	0,995899882
Usp18	243,8968873	0,559107811	0,182278899	3,06732053	0,002159871	0,040104608
Srp54a	34,41818966	0,223260716	0,264843589	0,842990826	0,399233574	0,740270965
Spry4	125,3132907	-0,698153818	0,322104782	-2,167474239	0,030198712	0,20087196
Ubl3	1231,032942	0,054634694	0,086005637	0,635245494	0,525268316	0,816792829
1110006O24Rik	13,10543273	0,661428626	0,525837254	1,257858055	0,208443096	0,560812185
Thnsl2	1456,566796	0,191518244	0,09924683	1,929716481	0,053641977	0,279695627
Mtcl1	8,616276712	1,332267615	0,628984366	2,118125165	0,034164469	0,216025826
Hsd17b1	5,735295717	0,305720236	0,613271983	0,498506771	0,618126898	0,861339607
H2-DMb2	14,65219532	0,781136752	0,416571079	1,875158384	0,060770937	0,298693626
H2-DMa	121,2008117	0,245046957	0,217512228	1,126589338	0,259916149	0,621613417
Prdx1	23970,39196	0,083091733	0,076640788	1,084171175	0,27828891	0,638593277
Kcna7	0,267995883	-1,667347305	3,312696416	-0,503320285	0,614739107	NA
Kcna1	0,227324296	1,389394708	3,349408814	0,414817893	0,67827522	NA
Adra2b	132,7060893	-0,086476326	0,201640898	-0,428863025	0,66802291	0,886343617
Adora2a	32,65312848	0,096111629	0,298520029	0,321960403	0,747482693	0,919581864
Tert	125,9182126	0,220563748	0,214283569	1,029307793	0,303335062	0,663017899
Atrx	917,6096721	-0,192058697	0,162811095	-1,179641329	0,238142898	0,595289658
Tfam	929,3709115	0,023163452	0,086642618	0,267344781	0,789203711	0,935277405
Tex9	19,41304223	0,249348704	0,393081914	0,634342855	0,525857095	0,817558262
Alkbh4	97,49867547	0,017372668	0,225777416	0,076945996	0,938666507	0,982918677
Hoxa2	10,00732843	-0,135408248	0,515491535	-0,262677927	0,792798831	0,936714766
Tff2	2,526602532	-0,072292223	1,179588737	-0,061285955	0,951131476	NA
Kdr	560,6312718	-0,291823107	0,309607495	-0,942558275	0,34590689	0,698434374
Kif12	18,93683444	0,559425135	0,348856087	1,603598606	0,108802558	0,409049888
Gcc1	353,670019	0,070245425	0,162407315	0,43252624	0,665358985	0,884828835
Tusc2	829,2981376	-0,031680327	0,10148731	-0,312160479	0,754918565	0,922462845
Fam217a	0,963844574	-1,291779834	1,497675258	-0,862523319	0,388399607	NA
Ncf2	79,16870368	0,318230707	0,218778924	1,454576616	0,145786509	0,474907367
Gpr160	5,429263136	0,974908761	0,654968899	1,488480999	0,136624089	0,459349518
Slc39a4	2240,406209	-0,628180706	0,194875592	-3,223496079	0,00126636	0,028033175
Trmu	193,3981485	0,022375719	0,127513278	0,175477561	0,860704401	0,959574131
2310057J18Rik	0,087021394	-0,517475177	3,352475198	-0,154356154	0,877328924	NA
Wfdc3	4,516764184	0,420640714	0,97281492	0,432395418	0,665454048	NA
Tmco6	193,8636395	0,260148957	0,155062954	1,677698962	0,093405882	0,380085036
Dpep3	0,113662148	0,780932884	3,352475198	0,232942181	0,815806304	NA
Naa50	1370,805392	-0,164081252	0,185479501	-0,884632811	0,376354704	0,722097522
Rdh10	587,2856327	0,040068893	0,119560785	0,335134071	0,737523953	0,91507318
Gltscr2	1713,515558	0,11025762	0,084354703	1,307071397	0,191188465	0,540266029
Mfsd6	316,172256	-0,329174807	0,123860277	-2,657630153	0,00786922	0,090927917
D1Ertd622e	229,1340477	0,166010037	0,246442574	0,673625642	0,500549347	0,803513525
Mmaa	569,9028839	-0,155704707	0,120763196	-1,289339071	0,197280233	0,546971183
Phlpp1	516,5450364	-0,210317459	0,123406333	-1,704267961	0,088331038	0,368454802
Ppp1r15b	2651,027792	-0,112875401	0,129841926	-0,869329375	0,384667	0,729241793
AI597479	277,0943372	0,086216076	0,115141904	0,748781054	0,453989181	0,776433588

Wdr26	1996,624745	0,032521186	0,092606806	0,351174898	0,725457141	0,910252319
Tiprl	412,9268795	0,003119815	0,0929904	0,033549861	0,973236105	0,992710425
Sft2d2	1335,95272	-0,074720212	0,100470176	-0,74370539	0,457054728	0,77843077
Cyp2c70	14527,51814	-0,220977437	0,131810493	-1,676478335	0,093644536	0,380194927
Rabif	373,551801	-0,108748407	0,113257698	-0,96018557	0,336961828	0,69270916
Ube2d2a	828,0978482	-0,091797794	0,097139118	-0,945013665	0,344651899	0,697587612
Dyrk3	19,38173031	1,038063408	0,407106611	2,549856429	0,010776729	0,110148386
Nmrk1	546,3160618	0,007846869	0,135216546	0,058031872	0,953723241	0,986479135
Tmem184c	541,8195596	0,050881688	0,111786209	0,455169632	0,648987206	0,876446263
Ttc13	706,7164827	-0,164422003	0,110539119	-1,48745535	0,136894588	0,459604154
Med13l	268,6845375	-0,298911031	0,171244644	-1,745520469	0,080894313	0,351670169
Ostm1	336,4344843	0,061763014	0,101913468	0,606033869	0,544492263	0,827477215
Rin1	6,173087241	0,284242735	0,647323592	0,43910455	0,660585782	0,882607568
Fam160b1	907,6262423	-0,053048432	0,109626464	-0,4839017	0,628455634	0,865922214
Me2	56,73968688	0,080765086	0,347624302	0,232334406	0,816278291	0,944915099
Zfp521	11,04016838	0,20838295	0,483092583	0,431351997	0,666212448	0,885111436
Ces2c	528,442108	-0,654512766	0,531693784	-1,230995707	0,21832447	0,572534296
Ppp1r42	16,89791065	-0,578321722	0,881950233	-0,655730562	0,511997501	0,810227859
Abhd17b	275,6149331	-0,123944869	0,181644761	-0,682347614	0,495019172	0,80006558
AI661453	1017,847761	-0,411257408	0,11411106	-3,604010062	0,000313345	0,01036908
Ubtd1	188,7602325	0,038746879	0,156123211	0,24818141	0,803994049	0,940361036
Zfp959	131,8477529	0,017849395	0,168358669	0,106020052	0,915566443	0,976559041
Pex6	1900,449982	-0,296394158	0,114867587	-2,580311526	0,009871122	0,103666169
Bhlha9	0,093953095	-0,517475177	3,352475198	-0,154356154	0,877328924	NA
Adam1a	15,19105359	0,31642273	0,362324399	0,873313336	0,382492313	0,72687132
Zfand4	794,538546	-0,099068589	0,138174955	-0,716979348	0,47338684	0,787601178
Gm3776	28,35053963	1,049100874	0,700748313	1,497115091	0,134363314	0,456327747
9230102K24Rik	0,093953095	-0,517475177	3,352475198	-0,154356154	0,877328924	NA
BC052688	0,64505015	1,679015498	2,331199928	0,720236595	0,471379336	NA
Thap6	203,3899827	-0,01832711	0,124733991	-0,146929554	0,883187624	0,966395158
2610005L07Rik	48,49408002	0,031316106	0,304563439	0,102822932	0,918103505	0,976907366
Mirt1	3,257541896	2,239560935	1,025482229	2,183910039	0,028968859	NA
C130080G10Rik	3,092379152	0,307102306	1,417230662	0,21669183	0,828448515	NA
D430020J02Rik	3,270817568	-0,361611856	0,798950341	-0,452608676	0,65083055	NA
Gm7854	8,637841367	0,399467872	0,508988316	0,784827194	0,432554893	0,762604031
Taf1d	184,1628009	0,04916796	0,170969492	0,287583241	0,773665775	0,928621142
B230319C09Rik	1,829565657	-0,010553106	1,115617987	-0,009459427	0,992452582	NA
Gm11696	11,62411123	0,433996042	0,43784462	0,991210174	0,321582964	0,678756338
Col5a3	1250,759626	0,022630934	0,382902045	0,059103717	0,952869498	0,986479135
4930404I05Rik	0,086476712	0,780932884	3,352475198	0,232942181	0,815806304	NA
Snord96a	19,87145566	0,419927746	0,327618287	1,281759179	0,199927138	0,550190595
AA465934	7,936524324	0,115609887	0,547249566	0,211256242	0,832687326	0,950100347
5830417I10Rik	184,6442069	-0,371896663	0,126350221	-2,9433796	0,003246501	0,052103804
Appbp2os	12,34906886	0,003904564	0,416246673	0,00938041	0,992515626	0,997964907
Mdp1	408,7415286	-0,079650152	0,092430921	-0,861726261	0,388838171	0,732681819
4833422C13Rik	23,61896672	0,498751133	0,323355784	1,542422178	0,122971036	0,435526873
Tmem181c-ps	0,172953423	1,307385949	3,350719078	0,39018071	0,696402925	NA
C4bp-ps1	11,7404659	0,358549879	0,443788569	0,807929507	0,419131164	0,753719022
Dubr	5,964420721	0,529521702	0,580011254	0,912950738	0,361268451	0,710682449
Pram1	36,91904362	0,325076423	0,270706478	1,20084464	0,229811472	0,585962127
Gm8801	0,093953095	-0,517475177	3,352475198	-0,154356154	0,877328924	NA
BC037032	1,183196804	1,334018881	1,458621813	0,914574888	0,360414847	NA
Mirg	0,623598726	1,728396338	2,362097325	0,731721052	0,464338845	NA
Dleu2	267,6499242	0,521628865	0,222699378	2,3423005	0,019165277	0,154256827
Gm25500	0,38739533	-0,100597892	2,267273461	-0,044369545	0,964609837	NA
B3galt5	0,174042788	-1,166820872	3,345560292	-0,348766954	0,727264274	NA
Max	418,2747464	-0,008005768	0,110377587	-0,072530743	0,94217954	0,983291729
Slc11a2	959,2718176	-0,417790208	0,117947189	-3,542180298	0,000396834	0,012512442
Dchs1	51,92146887	-0,306814158	0,242563308	-1,264882807	0,205913341	0,558736902
Lrig2	158,6246943	0,075955997	0,146647977	0,517947803	0,604494688	0,855727392
Wars2	110,1998984	0,101946462	0,161983127	0,629364698	0,529110324	0,818910651
Tmem242	720,6183111	0,159925934	0,124485971	1,284690416	0,198900489	0,54883398
Cers4	51,37156907	0,083389654	0,209505745	0,398030393	0,690607783	0,894644908
Cand2	13,03426089	-0,040437562	0,380445429	-0,106290046	0,915352229	0,976559041
Zfp422	473,511484	0,12556439	0,095852367	1,309976925	0,190203642	0,538507515
Cap2	1,076847368	-0,899593538	1,387652204	-0,648284588	0,5168009	NA

Vash1	62,16696464	0,005537531	0,307580753	0,018003504	0,985636058	0,995899882
Mroh3	0,093303375	-0,517475177	3,352475198	-0,154356154	0,877328924	NA
Lrrn4	10,82262422	0,931915203	0,54965591	1,695451984	0,089989712	0,372122627
Mtap7d3	2,24138206	0,610054401	1,025750522	0,594739547	0,552017583	NA
Hnrnpl	1411,33341	0,220305825	0,091498746	2,407746946	0,016051303	0,139253643
Itgb8	2,883825016	1,240616907	1,037638221	1,195616045	0,231846427	NA
Mical2	787,6164421	-0,722724315	0,157156106	-4,598767008	4,25E-06	0,00041778
Spata33	3,228856246	0,040209004	0,751937775	0,053473844	0,95735437	NA
L3mbtl4	0,18660675	-1,235117516	3,340448777	-0,369745983	0,711571767	NA
Ankrd16	193,9352276	0,083241343	0,129334767	0,643611497	0,519827393	0,813876985
Tnpo3	1287,107803	-0,073198843	0,104948414	-0,697474505	0,485505889	0,79430833
Dhrs11	772,1725972	0,001210284	0,105186247	0,011506107	0,990819658	0,99719344
Tnfsf15	1,44162296	0,032586536	1,341538623	0,02429042	0,980620955	NA
Zhx3	535,209943	-0,207344738	0,152855517	-1,356475334	0,174947978	0,517287416
Agtr1a	1803,07082	0,351423267	0,128892999	2,726472878	0,006401521	0,079971795
Lingo4	10,92340826	-0,425443776	0,671541574	-0,633533042	0,52638561	0,817627711
Fastkd1	495,889179	-0,084397543	0,117494128	-0,718312862	0,472564401	0,787019872
Pptc7	272,3478888	0,266698293	0,132467528	2,013310706	0,044081953	0,247514333
Upk3b	35,47150837	1,248977693	0,569111116	2,194611311	0,028191491	0,193277974
Sowahb	175,2960437	0,064117751	0,161302399	0,397500293	0,69099857	0,894804328
Trappc11	957,9507126	-0,217932985	0,103656972	-2,102444066	0,035514394	0,220116801
Amigo3	9,142379189	-0,296912131	0,55092341	-0,538935404	0,589931427	0,84953676
Ccdc158	6,601524166	0,14464643	0,649925832	0,222558365	0,823879244	0,947472731
Zfp629	332,8635316	0,351109957	0,119228717	2,94484388	0,003231177	0,051982671
Samd12	0,447231288	-0,601697645	2,171043183	-0,277146788	0,781667396	NA
Notum	941,9191862	-0,057854871	0,172097012	-0,336175915	0,736738214	0,914722813
Heg1	146,2264026	0,608792376	0,252099353	2,414890676	0,015739933	0,137714114
Faxc	2,268141222	1,270209104	1,210613978	1,049227192	0,294073567	NA
Arhgap26	350,3464997	-0,124068327	0,215667101	-0,575277022	0,565103959	0,83747956
Casc1	0,93279159	2,470141418	1,855727095	1,331090884	0,183159108	NA
Zc3h15	1254,494528	-0,171135662	0,102965029	-1,662075607	0,096497616	0,386250487
Pacrg	1,88557004	2,134762753	1,187995185	1,796945627	0,072344252	NA
Ebna1bp2	621,9304317	-0,038164532	0,136820349	-0,278939001	0,780291638	0,931606698
Efcab9	0,929265557	0,223241936	1,67872072	0,132983368	0,894206534	NA
Dcps	456,4369177	0,173873764	0,102010902	1,704462568	0,088294704	0,368418193
Chac1	50,80083233	-1,010855577	0,419009459	-2,41248868	0,015844029	0,137992723
Lrrc46	31,28118644	0,571908411	0,285197492	2,005306596	0,04493028	0,251272586
Pnlip	0,087021394	-0,517475177	3,352475198	-0,154356154	0,877328924	NA
Atp2c2	0,179780087	0,059593471	3,352475198	0,017775962	0,985817581	NA
Odf3	0,086476712	0,780932884	3,352475198	0,232942181	0,815806304	NA
Zdhhc3	396,445835	-0,07948301	0,10568341	-0,752085976	0,451999357	0,774656859
Bcl2a1d	3,440355761	1,665630723	0,869368901	1,915907875	0,055376823	NA
Ccnd3	758,9773698	-0,271645306	0,144086327	-1,885295514	0,059389951	0,294723185
Brd8	375,8298295	0,200563245	0,139367057	1,439100811	0,150121962	0,482331294
Gm5535	0,35338323	0,909671359	3,184606596	0,285646384	0,775148974	NA
Pcsk4	207,0351938	0,171484399	0,182780767	0,938197172	0,348143091	0,70022243
Bco1	184,7393071	0,089104562	0,170898948	0,521387421	0,602096913	0,854563604
Rps6kb2	445,8975285	-0,180304926	0,12128169	-1,486662383	0,137104002	0,45990048
Ctsz	6239,475631	-0,01461885	0,074586651	-0,195998213	0,844611564	0,953132002
Acap3	83,38918976	-0,19158423	0,170681616	-1,122465527	0,261664557	0,622343615
Mycbp2	585,0291055	-0,232393333	0,106285681	-2,186497101	0,028779257	0,195701872
Exoc5	377,8573249	-0,064079336	0,157745264	-0,406220347	0,684580705	0,892545294
Snx25	288,2346409	-0,106553255	0,100636653	-1,058791721	0,289694645	0,651021636
Wtip	30,51736256	0,14207738	0,267927663	0,530282606	0,595916005	0,853075785
Mrgprg	0,173498105	0,059593471	3,352475198	0,017775962	0,985817581	NA
Chrm2	0,113662148	0,780932884	3,352475198	0,232942181	0,815806304	NA
Foxo6	0,696596606	-0,062174368	2,320555585	-0,02679288	0,978624932	NA
Ffar1	0,26680148	-0,62623849	2,8346967	-0,220919046	0,825155467	NA
Dhx37	336,4602425	-0,145456109	0,110185425	-1,320102986	0,186800636	0,533163472
Btbd6	294,7956803	-0,023976578	0,11693945	-0,205034129	0,83754546	0,951141594
Ugt1a9	1764,103477	0,060162965	0,178900549	0,336292793	0,736650084	0,914722813
Ugt1a7c	2,320770791	0,482428993	1,020997389	0,472507568	0,636564541	NA
Ugt1a10	18,01687843	0,216935131	0,408227632	0,531407268	0,595136586	0,852682566
Mettl14	196,0817548	0,180815532	0,17933305	1,008266643	0,313326472	0,672227864
Chd8	756,9794084	-0,087518097	0,094166649	-0,929395891	0,352683956	0,703631072
Snph	0,942820176	1,626737568	1,706124284	0,953469559	0,340352213	NA

Supplementary Table S1: *Serpina1* KO vs. wildtype all DEGs

Ube2e1	606,0286362	0,088361933	0,111680361	0,791203856	0,428825042	0,760745421
Ndufs2	5572,41051	-0,01375704	0,073881874	-0,186203181	0,85228543	0,955969788
Zfp652	205,6351494	-0,440292664	0,247231566	-1,78089178	0,074930132	0,338311696
Neil2	9,431898152	0,708928045	0,473574056	1,496973993	0,134400025	0,456327747
Erich6	0,683390041	0,978902746	2,063099331	0,474481636	0,63515649	NA
3110062M04Rik	117,0819563	-0,11937392	0,23539625	-0,50711904	0,612071296	0,858888222
Smim13	528,4919884	0,375218331	0,119375445	3,143178502	0,001671239	0,034044289
Sos2	304,3207544	-0,133825564	0,153936401	-0,869356195	0,384652335	0,729241793
Faf1	759,4618798	-0,059045037	0,078090628	-0,756109126	0,449583771	0,773450705
2900060B14Rik	0,653175981	-0,238490974	1,828906568	-0,130400852	0,896249293	NA
Akt3	50,71275002	0,09944632	0,257219765	0,386620059	0,699037506	0,898942204
Adamts5	35,19448263	0,028935627	0,356480785	0,081170229	0,935306575	0,982050665
Ikzf4	32,18204203	-0,923302884	0,53044394	-1,740622929	0,081749696	0,353542587
Csgalnact1	9,470823034	-0,112182762	0,648093026	-0,173096697	0,862575421	0,960942085
Wnk3	0,353927913	-0,100118367	2,670731403	-0,037487247	0,970096509	NA
Cars	844,5687391	0,123474864	0,11488327	1,074785419	0,28247081	0,64409897
Mapk9	1512,194938	-0,003394305	0,096090732	-0,035323958	0,971821419	0,992423486
Fam174b	65,5765195	-0,198283105	0,271770588	-0,729597363	0,465636334	0,781663619
Dguok	306,5685234	0,128024548	0,113893521	1,12407227	0,260982368	0,621964694
Mir6947	0,093303375	-0,517475177	3,352475198	-0,154356154	0,877328924	NA
Fbxl16	9,606431093	-0,466725714	0,48699822	-0,958372524	0,337874945	0,693194655
Col4a3bp	764,2089158	0,094526704	0,109425918	0,863842003	0,387674691	0,731859161
Slco2b1	4212,80228	0,170250312	0,123416419	1,379478628	0,167747231	0,506611452
Dlg2	0,3267786	1,84016758	2,655545515	0,692952755	0,488339201	NA
Mir6948	0,783309409	0,219136952	1,617953915	0,135440787	0,892263378	NA
Glb1	461,2149589	0,234160324	0,133954296	1,748061326	0,080453409	0,350731846
Foxa3	1136,881349	0,352772244	0,383700248	0,919395403	0,357888793	0,707364765
Hn1	258,7752357	-0,060725477	0,13099348	-0,463576332	0,642951315	0,873360712
Bod1l	492,639371	-0,148906309	0,122034539	-1,22019807	0,222389798	0,577161019
Sin3b	829,366758	0,00955	0,113381625	0,084228813	0,93287451	0,981257162
Kifc3	889,2921358	-0,283011922	0,128532782	-2,20186568	0,027674798	0,19164263
Mir8116	0,375162661	-2,131909841	3,036791336	-0,702027109	0,482662258	NA
Zbtb17	237,5360305	0,046484511	0,140306656	0,331306525	0,740412963	0,916128178
Apoa1	233462,7985	-0,341557425	0,127169709	-2,685839477	0,007234784	0,086237888
Plagl1	31,78534907	-0,057060868	0,487080194	-0,117148816	0,906742126	0,974457847
Yy1	697,2612244	0,073811119	0,15786392	0,467561674	0,640098078	0,871049783
Ywhae	4660,336589	-0,035403164	0,067653511	-0,523301211	0,600764659	0,854422974
Tnfaip8	52,79464609	0,458649127	0,27860704	1,646222318	0,099717996	0,392993389
Pwwp2a	196,9998282	0,241215158	0,140862358	1,71241744	0,086819767	0,365572746
Mir6922	1,046998146	1,175787034	1,830238554	0,642422831	0,520598679	NA
Yeats2	249,4602333	-0,162537566	0,155501053	-1,045250578	0,295907173	0,656921786
Thap3	187,8778436	0,139640955	0,114610643	1,218394315	0,223074164	0,57807298
Tnks2	1024,736485	-0,068943299	0,109017715	-0,632404553	0,527122559	0,818299511
Sdc1	9446,851455	0,005579774	0,179961214	0,031005425	0,975265213	0,993185764
Acaa1a	2790,174304	-0,018070715	0,136512396	-0,132374173	0,894688342	0,970433882
Adrbk1	768,6048442	-0,058315126	0,110977939	-0,525465933	0,59925933	0,85430922
Slc35b3	196,6751499	-0,03650063	0,136885282	-0,266651236	0,789737703	0,935361858
Scp2	65979,48059	-0,517178419	0,171897642	-3,008641732	0,002624184	0,045035573
Dna2	222,3706327	-0,252806946	0,128921536	-1,960936503	0,049886431	0,268094
Far2	4,373030722	0,189887503	1,270266077	0,1494864	0,88116984	NA
Zbed5	32,43639052	0,873007141	0,319616064	2,731424476	0,006306119	0,079349252
Zbed4	64,8707457	0,02381582	0,191748983	0,124203112	0,901154458	0,972526743
Bbs10	175,9510794	-0,135141129	0,15745305	-0,858294764	0,39072972	0,734518115
Vwce	729,4222211	-0,013071796	0,114519939	-0,114144275	0,909123425	0,9747592
Supt7l	269,7837733	0,006481057	0,115506154	0,056110054	0,955254135	0,987017436
Setd3	2241,757773	0,045406032	0,093762583	0,484266006	0,628197098	0,865852703
Immp1l	430,8789218	0,113972299	0,110082444	1,035335834	0,300512101	0,66099807
Mef2d	909,5630264	-0,160000599	0,075655033	-2,114870502	0,034440986	0,216957051
Tmem87b	457,9690554	0,090566224	0,096844218	0,9351743	0,349698481	0,701600082
Tram1	3996,29475	-0,292128607	0,126580181	-2,307854247	0,021007245	0,161964159
Rrp1b	139,2275661	-0,003250762	0,150690885	-0,021572385	0,982789062	0,995413661
Ppfia2	4,849584225	-0,418449526	0,84658936	-0,494276854	0,621110669	NA
Cldn23	2,471256437	-0,719957477	1,198864915	-0,60053261	0,548151335	NA
Epn3	5,74666582	1,154376902	0,686584401	1,681332842	0,092698282	0,378291616
Smug1	213,273076	0,117238815	0,144469747	0,811511184	0,417072173	0,752195715
Cul2	1216,763083	-0,396424251	0,160103799	-2,476045254	0,013284674	0,124284678

Cnn3	3742,336032	-0,034393934	0,070649992	-0,486821486	0,626384843	0,865266164
Ercc8	70,08982245	0,031191141	0,1841313	0,169396194	0,865485017	0,961775826
Tmem68	154,1462711	-0,074338738	0,133317948	-0,55760488	0,577114223	0,84496439
Serpina9	50,28405889	-1,366920121	0,967958168	-1,412168589	0,157900327	0,493680148
Tmem55a	296,4394117	-0,084531785	0,146721311	-0,576138423	0,564521622	0,837355044
D830046C22Rik	0,354367556	-2,058435915	3,081765084	-0,667940566	0,504171531	NA
Abca5	126,8242086	0,117722513	0,165881842	0,70967691	0,477904514	0,790241861
Abca6	3209,598549	0,08133725	0,196861899	0,413169081	0,679482739	0,891375208
Zfp2	123,0412416	0,078623469	0,142016898	0,553620521	0,579838579	0,84530168
Gtpbp6	229,4395527	0,018304018	0,17977035	0,101818895	0,918900428	0,976907366
Mfsd7c	73,80744387	0,518782409	0,480877633	1,078824161	0,280666122	0,641688878
Mir7052	11,09670175	0,36051916	0,50645417	0,711849525	0,476557961	0,789577814
Apc	681,3289469	-0,121706692	0,137039488	-0,88811403	0,374479416	0,720969948
Antxr1	40,44678682	-0,048977388	0,286374165	-0,171025859	0,864203432	0,961272625
Foxp3	3,34490803	1,118598723	0,926627351	1,20717214	0,227365857	NA
Rims2	2,976311528	-1,730943112	0,877098022	-1,973488787	0,048439901	NA
Zrsr1	547,4770626	0,122170857	0,118820126	1,028200033	0,303855743	0,663332368
Dlk1	0,31389621	-0,661096352	2,708970037	-0,244039743	0,80720004	NA
Pipox	9755,948127	0,206891467	0,077991821	2,652732877	0,007984303	0,09162416
Adam2	0,608228564	-2,767319959	2,24446818	-1,2329513	0,217593934	NA
Psmc3ip	5,555369954	-0,499647505	0,747173831	-0,668716548	0,503676309	0,804997854
Stk10	103,0102821	0,532418278	0,201571478	2,641337368	0,008257944	0,093480489
Dhfr	986,5179393	0,214864001	0,135736178	1,582953076	0,11343216	0,417072207
Ret	0,20013886	1,337107854	3,350237755	0,399108348	0,689813372	NA
4930427A07Rik	3,805791833	-1,239597069	0,901089775	-1,375664338	0,168925578	NA
Ddx1	1946,926339	-0,099650929	0,108216959	-0,920843924	0,357131919	0,707149983
Srcin1	10,16013335	-0,30773541	0,461403315	-0,666955351	0,504800654	0,805075709
Rrad	4,515321552	0,132844674	0,676100603	0,196486549	0,84422936	NA
Dysf	92,05482224	-0,196231542	0,237784291	-0,825250235	0,409229539	0,746907036
Rgs18	2,812820631	1,11139207	0,970449054	1,145234843	0,252111888	NA
Myl7	5,785936778	2,179088913	0,990776758	2,199374275	0,02785132	0,192227311
Svep1	14,83733709	0,375208411	0,685148672	0,547630648	0,583945541	0,847068435
Scamp2	494,8322227	0,08890384	0,102430463	0,867943352	0,385425345	0,729677128
Polr1e	133,5317649	-0,064871185	0,200292722	-0,323881886	0,746027458	0,919003408
Suv39h2	19,05893934	-0,24592958	0,423791171	-0,580308409	0,561706658	0,836615007
Kcnj14	0,584748855	2,638328875	2,271879867	1,161297705	0,245520853	NA
Pank3	1152,634834	0,026716175	0,161307514	0,16562263	0,868453937	0,961775826
Prr5	123,7386429	0,350972846	0,213348859	1,645065498	0,099956305	0,392993389
Fam91a1	1599,983291	-0,04073295	0,116843578	-0,348610947	0,727381408	0,910691332
Mtrf1	234,0091594	0,005969516	0,121610027	0,049087364	0,960849673	0,988470616
Kbtbd2	369,870504	-0,068103334	0,09508545	-0,716232972	0,473847507	0,787824815
Mir6954	0,18725647	-1,238536202	3,340198538	-0,370797181	0,710788601	NA
Ogfod1	312,5018547	-0,150526602	0,116521163	-1,291839161	0,19641285	0,545402687
Srsf11	899,9051248	0,107561302	0,082616105	1,301941101	0,192936521	0,541594346
Ifi47	694,1157925	0,199441438	0,165549424	1,204724446	0,228309695	0,584236803
Cdkn1b	311,7571453	-0,206241106	0,133651906	-1,543121321	0,122801342	0,435126199
Tcof1	248,3127737	-0,109655198	0,134592636	-0,814719151	0,415233089	0,751551608
Cpt1b	5,084920222	-0,577086179	0,728659709	-0,791983105	0,428370528	NA
Plod3	670,8789416	-0,371743529	0,091614494	-4,057693399	4,96E-05	0,002650258
Ccl9	2155,604433	-0,116033697	0,146654984	-0,791201867	0,428826202	0,760745421
Plod2	16,57208378	0,024737077	0,406687066	0,060825826	0,951497922	0,985972877
Usp12	516,7832605	-0,073707662	0,122838758	-0,60003588	0,548482324	0,828830133
Creb3l1	46,58867567	-0,676276813	0,324647512	-2,08311103	0,037241104	0,226307419
Ube2b	1908,291788	0,14365733	0,067007839	2,14388841	0,032041829	0,207945249
Katna1	392,6427189	0,079598911	0,09909959	0,803221394	0,421846782	0,755380342
Ccl17	0,287160254	0,617200912	2,785784868	0,221553688	0,824661338	NA
Sptb	16,07386175	-0,896095639	0,441836087	-2,028117814	0,042548225	0,243203202
Atp2a3	33,93690672	0,453068269	0,297420784	1,523324174	0,127677622	0,444858517
Kl	0,360859614	-0,137604283	3,170662709	-0,043399218	0,965383301	NA
Stra6l	5284,139015	0,140140687	0,092327242	1,517869318	0,129047346	0,446489123
Utp23	118,7674116	0,095890198	0,182973634	0,524065658	0,600232874	0,854422974
Gale	1017,989237	-0,328184032	0,224298755	-1,463155836	0,143424742	0,470500538
Fam53a	515,4449408	-0,123842012	0,118825217	-1,042219952	0,297309715	0,658144463
Tmem202	0,307614229	-0,630030301	2,72139296	-0,231510227	0,816918442	NA
Cd80	10,16944997	0,327934513	0,584717395	0,560842753	0,574904739	0,843492258
Dock6	725,355265	-0,028050162	0,180314167	-0,155562721	0,876377712	0,964383375

Supplementary Table S1: *Serpina1* KO vs. wildtype all DEGs

Gm8615	0,707987151	-0,467475694	1,752934541	-0,266681774	0,789714188	NA	
Ccdc61	122,9781407	0,047933701	0,136262226	0,351775414	0,725006698		0,910252319
Adap2	610,4863701	-0,180904945	0,187307439	-0,965818263	0,334135128		0,690638919
Lgsn	0,093303375	-0,517475177	3,352475198	-0,154356154	0,877328924	NA	
Zfp825	296,5997836	0,164768403	0,154648068	1,06544107	0,28667638		0,648131311
Klre1	3,066839858	-0,048074032	0,82511038	-0,058263758	0,953538535	NA	
Pcnx4	198,9498204	0,005737634	0,142601237	0,040235513	0,967905365		0,991231429
Chd1l	268,3611979	0,097125009	0,14815554	0,655561104	0,512106559		0,810227859
Alpk3	0,685433411	2,877292447	1,899186559	1,515013063	0,129769096	NA	
Calcb	0,331469375	1,851207654	3,180323419	0,582081571	0,560511738	NA	
Klra17	1,16327708	1,12544504	1,505400587	0,747605023	0,454698434	NA	
Bbs4	88,32902671	0,404145983	0,209599236	1,928184427	0,053832191		0,280141127
Wtap	645,1335284	0,164321054	0,086627988	1,896858716	0,057846587		0,290515036
Mtrf1l	126,6043039	0,003392741	0,15368503	0,022075935	0,982387383		0,995338444
Obfc1	80,87415165	0,260379708	0,186679373	1,394796351	0,16307726		0,49993802
Spty2d1	377,4789583	-0,113052389	0,118612326	-0,953125129	0,340526675		0,694720147
Adamts9	36,1124436	-0,369131613	0,24880078	-1,483643315	0,137903572		0,461254153
Eogt	103,1731262	-0,4924779	0,166142213	-2,964194891	0,00303476		0,049598667
Snora33	2,406338481	-1,087150243	1,020397295	-1,065418585	0,28668655	NA	
Timm9	522,5318152	-0,058240785	0,183575078	-0,317258671	0,751047335		0,920556312
Mir30d	0,267451201	-0,629501011	2,83229786	-0,222258054	0,824113003	NA	
Cnnm2	184,8287606	-0,190345083	0,150493522	-1,264805822	0,205940944		0,558736902
Atp5g1	3746,051213	0,127665278	0,072714934	1,755695455	0,079140423		0,348953931
Cdh6	8,980605617	1,353398701	0,60470639	2,238108813	0,02521396		0,181772069
Sh2d3c	242,1884738	-0,008557318	0,164508118	-0,052017603	0,958514667		0,987896001
Atf6b	799,8592472	-0,447924943	0,097990891	-4,571087574	4,85E-06		0,000450461
Lamb1	230,7583274	-0,13818389	0,138692322	-0,996334107	0,319087837		0,677017187
Ccl7	2,193342586	-0,234155695	1,240145557	-0,188813074	0,850239325	NA	
Ccl5	23,58777766	0,809348213	0,363110304	2,228932103	0,025818423		0,184210699
Trip6	386,0187102	-0,190626747	0,127204724	-1,498582294	0,133982029		0,455962937
Trp53	576,9837734	0,258877618	0,092380187	2,802306699	0,005073861		0,069714749
Tfrc	932,5766202	-0,096112298	0,19918728	-0,48252227	0,629434982		0,866228449
Xpo4	80,10209772	-0,263728537	0,254277779	-1,037167062	0,299658006		0,659904651
Arid3b	82,66590692	-0,150248144	0,182175391	-0,824744458	0,409516686		0,746907036
Pus1	336,172558	0,265741033	0,113143566	2,348706525	0,018838749		0,152703354
Pafah1b2	600,4544159	-0,038914882	0,110290116	-0,352841064	0,724207597		0,910252319
Olfr110	0,086476712	0,780932884	3,352475198	0,232942181	0,815806304	NA	
Fam199x	123,1364039	-0,186518131	0,215497281	-0,86552429	0,38675109		0,73103259
Zfp668	60,71441811	-0,042317478	0,220577594	-0,19184849	0,847860887		0,953880403
Tmie	764,7773587	0,421091073	0,237901994	1,770019098	0,076723959		0,342478335
Slc29a4	0,7455142	-0,281185221	2,00158407	-0,140481344	0,888279691	NA	
Slc1a7	1,105515386	2,725581878	1,568282631	1,737940486	0,082221301	NA	
Taf1	478,0834614	-0,134548014	0,140937745	-0,954662738	0,339748279		0,694297577
Zbtb6	134,6425636	-0,081832818	0,215125317	-0,380396036	0,703651457		0,900719679
Rab4b	426,0985037	0,137013533	0,104655113	1,309191004	0,190469659		0,538804246
Fam35a	668,7729112	-0,15654021	0,170604215	-0,917563555	0,358847405		0,708107891
Tmem238	257,797253	0,163062286	0,194123277	0,839993473	0,400912046		0,741644961
Ppm1k	1313,965755	-0,101024226	0,165984468	-0,608636623	0,542765324		0,826611597
Elfn1	1,138097439	-0,642993358	1,358897611	-0,473172778	0,636089917	NA	
Pus7l	51,49248169	0,462749325	0,246679427	1,875913736	0,060667126		0,298512628
Tmem245	154,1842943	-0,366282627	0,236139903	-1,551125511	0,120871609		0,432530122
Exoc6	434,3683724	0,125439233	0,134223681	0,934553664	0,350018368		0,701844955
Catsperd	27,34079848	-0,267762065	0,37590014	-0,712322335	0,476265196		0,789577814
Srl	37,16692237	-0,229200765	0,404475454	-0,566661742	0,570944018		0,8413217
Dhrs3	5738,938609	0,054643782	0,120551795	0,453280533	0,650346749		0,876831587
D630033O11Rik	10,34281937	0,6863744	0,578051547	1,187393069	0,235072613		0,592624129
Dthd1	0,236587694	-1,308576888	3,335186821	-0,392354899	0,694796005	NA	
Recql5	371,8214896	-0,142774081	0,123590707	-1,155216964	0,24800159		0,607129663
Bbox1	2685,065477	0,253274195	0,143310718	1,767308115	0,077176642		0,34369571
Colec12	1531,687476	0,142806926	0,205766637	0,694023716	0,487667336		0,796312031
Trio	410,0128328	-0,171995155	0,136897828	-1,256376069	0,208979654		0,561671273
Ankrd22	0,227324296	1,389394708	3,349408814	0,414817893	0,67827522	NA	
Nop56	485,1400545	-0,048537534	0,234515423	-0,206969477	0,836033696		0,951141594
Mrps16	677,3367297	0,080307671	0,112016953	0,716924264	0,473420829		0,787601178
Eif1ax	677,6306745	-0,038924018	0,105355739	-0,369453233	0,711789926		0,905415749
Ccdc127	600,2557131	-0,147798828	0,09409578	-1,570727488	0,116245961		0,422672193

Lrrc63	1,234663587	1,383654936	1,654377655	0,836359783	0,402952531	NA
Mocs2	3042,006203	0,118952123	0,079362497	1,498845527	0,133913711	0,45589315
Nhsl2	26,3228161	-0,039134143	0,299629453	-0,130608464	0,896085047	0,97084116
Tmem175	248,9001858	0,024332202	0,111230924	0,21875393	0,826841736	0,947537943
Ddx49	422,933309	-0,178114884	0,147864167	-1,204584502	0,228363743	0,584236803
Zbtb34	97,42825117	-0,065098234	0,170538268	-0,381722148	0,70266747	0,900494767
Dtx4	636,2482113	-0,207822879	0,114615867	-1,813212121	0,069799111	0,323505282
Rcc2	656,5886021	-0,281954271	0,139744803	-2,017636902	0,043629091	0,246108571
Gpt2	9432,378084	0,248323904	0,120903507	2,053901582	0,039985218	0,235109182
Slc41a1	111,021393	-0,051229374	0,170074448	-0,301217349	0,763248761	0,925598865
C1s2	0,589111923	-1,811185789	2,294975533	-0,789196121	0,429997389	NA
Znrf2	781,1066156	-0,332710035	0,101967823	-3,262892397	0,001102814	0,025552025
Apobr	53,04051758	0,271759166	0,252703737	1,075406203	0,282192906	0,643821185
Dgkz	1028,109716	0,004166464	0,094787894	0,043955658	0,96493975	0,989816582
Mtbp	50,04554264	0,669569666	0,249345712	2,685306524	0,007246332	0,086265544
Tnnt1	25,19735545	-0,70037284	0,464590694	-1,5075051	0,131681238	0,451628134
Prex1	146,0206285	0,333823173	0,222236217	1,502109681	0,133068785	0,453941462
Gm9992	39,64025366	0,583423808	0,253394869	2,302429449	0,021310966	0,163264046
Eya4	8,834850197	1,342481213	0,610291737	2,199736833	0,027825572	0,192152372
Dpf1	6,835850405	1,086533059	0,764116231	1,421947362	0,155041532	0,489743509
Rnf11	2066,980825	-0,212042971	0,175054242	-1,211298676	0,225780956	0,581069718
Fbxl17	626,1152795	0,033517186	0,088722371	0,377776042	0,705596973	0,902021937
Bok	195,1426844	0,128008755	0,225095091	0,568687455	0,569568263	0,840923037
Fbxo18	621,449767	-0,102054034	0,105027332	-0,971690248	0,33120467	0,687985586
Fbxo8	1299,543015	0,204521361	0,095941887	2,131721255	0,033029768	0,211989906
Lsr	7013,268638	0,211037685	0,083806168	2,518164108	0,011796834	0,116136542
Srpx	6,263953619	0,077102754	0,762930006	0,101061373	0,919501741	0,977134804
Syt5	1,503797731	-0,59926694	1,142256676	-0,52463422	0,599837493	NA
Zfp748	106,1101277	-0,029379228	0,205486823	-0,142973782	0,886310888	0,967825717
Slc4a5	4,894500597	-0,546397954	0,767635006	-0,711793952	0,476592379	NA
Hoxa4	10,37439119	-0,340172193	0,48693202	-0,698603047	0,484800139	0,79420977
Foxq1	859,6198226	-1,679812367	0,606684669	-2,768839319	0,005625637	0,073493174
Flt1	138,484579	-0,366118431	0,281639947	-1,299952069	0,193617397	0,542139655
Cldn11	0,113662148	0,780932884	3,352475198	0,232942181	0,815806304	NA
Slc22a18	2186,249212	0,145070372	0,090549673	1,602108179	0,10913169	0,40910239
Mfsd4b1	1006,32343	-0,00631593	0,143236707	-0,044094355	0,964829192	0,989779119
Ccdc92	5,078740351	0,331252611	0,701545008	0,472175851	0,636801276	NA
Aif1l	3,519858211	0,417680931	0,756842486	0,551872998	0,58103537	NA
Smyd5	403,9592835	-0,193437885	0,094826045	-2,039923586	0,041357937	0,240072963
Camk1g	0,093303375	-0,517475177	3,352475198	-0,154356154	0,877328924	NA
Rhbdl1	69,11850108	-0,122383853	0,295444755	-0,414235999	0,67870128	0,890709117
Cecr5	505,637138	-0,110828473	0,089478782	-1,2386006	0,215493446	0,569354126
Ccdc28a	323,5185229	0,355577141	0,169430635	2,098659076	0,035846965	0,221459367
Rcor3	147,5595257	0,059012968	0,16145694	0,365502828	0,714736096	0,906371815
Apoa1bp	1335,774143	0,108966357	0,073438385	1,483779328	0,137867473	0,46124881
Lmcd1	9,009493478	0,382313978	0,678858133	0,563172125	0,573317682	0,842365544
F11r	2077,060599	0,047854489	0,094866223	0,504441803	0,613950948	0,859682163
Engase	135,698708	0,013027332	0,203370124	0,064057254	0,948924638	0,985650844
Fbf1	178,3046554	-0,373088157	0,176983639	-2,108037546	0,035027742	0,218499774
4930480K23Rik	43,76255902	0,241330386	0,261012957	0,924591596	0,355178386	0,705239841
Twsg1	1092,262461	-0,153739986	0,096800763	-1,588210475	0,112238753	0,414622794
Pde8b	24,48033993	-0,231397358	0,322733837	-0,716991313	0,473379457	0,787601178
Rufy4	4,195428193	-0,609090537	0,728403613	-0,836199226	0,403042834	NA
Taf1c	62,25377714	-0,172827505	0,230385528	-0,7501665	0,453154432	0,775628467
Rab38	10,92673101	-1,20657313	0,634433873	-1,901810702	0,057195907	0,289000033
Arpc5l	956,9894395	-0,154869561	0,115337249	-1,342754072	0,179351619	0,522135977
Lrpprc	2750,448317	-0,27219057	0,117913365	-2,308394556	0,020977202	0,161926817
Mpdu1	1216,764064	-0,005163881	0,078326338	-0,065927779	0,947435324	0,985164724
Hsd17b2	5358,204607	-0,158453976	0,151752012	-1,044163919	0,296409557	0,657709439
Rel	25,55727974	0,188282239	0,456373316	0,412561892	0,679927626	0,891462871
Psma2	2001,859966	0,126217193	0,103744948	1,216610497	0,223752447	0,5790477
Adm2	0,087021394	-0,517475177	3,352475198	-0,154356154	0,877328924	NA
Zkscan2	0,940745259	-0,209924635	1,449424218	-0,144833122	0,88484263	NA
Chsy3	83,11382645	-0,296034138	0,228315671	-1,296600171	0,194768794	0,543831245
Car1	144,3839128	0,066232731	0,204948078	0,323168343	0,746567754	0,919391514
Agl	1228,114345	0,237713176	0,063919042	3,718972761	0,000200035	0,007483408

Vwa5a	592,7950743	0,130148996	0,095826981	1,358166505	0,174410856	0,517225036
Pla2g4f	0,53897958	0,519359315	2,133903132	0,243384673	0,807707416	NA
Slc25a47	5616,864336	0,39212677	0,268678928	1,459462313	0,144437906	0,472355765
Slc6a19	1,321866926	3,007472618	1,75692383	1,711783155	0,086936637	NA
Palm3	0,12663974	0,780932884	3,352475198	0,232942181	0,815806304	NA
Bud31	1205,627964	-0,01973368	0,096186443	-0,205160721	0,837446557	0,951141594
Snx19	839,3887923	0,002928512	0,114848609	0,025498888	0,979657035	0,994664228
Dnajc14	956,5086376	-0,012436965	0,079514143	-0,156411984	0,875708292	0,964125574
5730559C18Rik	4,307619962	-0,023897487	1,151262426	-0,020757637	0,983438991	NA
2010310C07Rik	0,569622581	-0,971166401	2,020602756	-0,480632028	0,630778048	NA
Rtn4rl2	2,974278512	0,124263941	0,876457143	0,141779826	0,88725392	NA
2010109A12Rik	4,1956377	0,4903366	0,700466491	0,700014356	0,483918339	NA
Chmp4b	1504,283333	0,035678244	0,062722567	0,568826269	0,569474046	0,840876797
Ipo7	1508,562427	-0,207301621	0,125333059	-1,654005924	0,098126309	0,390315569
Zdhhc15	3,385528236	-0,610617257	1,035553169	-0,589653216	0,555423166	NA
Peg13	113,0977235	0,079074123	0,185599512	0,426047042	0,670073568	0,887127503
Rnu11	0,320676396	1,743349119	3,194451911	0,545742797	0,585242749	NA
Lect2	2617,994488	0,579127141	0,182323438	3,176372421	0,001491294	0,031298438
Tmed1	273,9762227	0,12348446	0,116141099	1,063227929	0,2876786	0,649109738
Laptm5	503,5665234	0,197841065	0,194335525	1,018038597	0,308659614	0,667523388
Cd276	197,5610634	-0,45279378	0,210679258	-2,149209106	0,031617828	0,206478957
Slc5a9	0,294091955	0,617264955	2,767052958	0,223076668	0,823475837	NA
Efcab7	6,683696635	0,33406018	0,725422974	0,460503998	0,645154503	0,874664897
Gtf2b	513,5419853	-0,0441394	0,126239335	-0,349648547	0,726602473	0,910398169
Clcc1	1251,498184	0,06001191	0,075986082	0,789775021	0,429659169	0,760910509
Rap1a	1120,54382	0,052367393	0,131557193	0,398058003	0,69058743	0,894644908
Tm4sf4	1576,732605	0,331761317	0,15901659	2,086331473	0,036948608	0,225266506
Tmem189	302,3598775	0,068380506	0,171968896	0,397632989	0,69090074	0,894804328
Edem2	2031,072681	-0,43310008	0,118079945	-3,667854697	0,000244594	0,008743258
Ccm2l	94,41380619	-0,473728183	0,164622132	-2,877670083	0,004006239	0,059165556
Sdcbp2	1,930255175	-0,338045711	1,183484593	-0,28563592	0,775156989	NA
Btbd3	124,3210169	-0,016750094	0,175495461	-0,095444598	0,923961694	0,977951927
Irf2bpl	596,9713697	-0,254142883	0,168402065	-1,509143514	0,131262112	0,45054454
Spg11	447,8746505	-0,128160518	0,113633655	-1,127839441	0,259387733	0,620981526
Rhot2	1211,452095	-0,254969476	0,08356642	-3,051099681	0,002280049	0,041527555
Foxk1	510,9328633	-0,193285748	0,16179082	-1,194664495	0,23221814	0,589093797
Lexm	0,215799707	0,059593471	3,352475198	0,017775962	0,985817581	NA
Hcrtr2	8,528168246	0,422395396	0,678381156	0,622652018	0,53351322	0,821247926
Rbm12b2	72,62813861	-0,105467222	0,232388306	-0,453840488	0,64994364	0,87680086
Chst9	0,20013886	1,337107854	3,350237755	0,399108348	0,689813372	NA
Thap1	122,3137897	-0,242190091	0,173580189	-1,395263438	0,162936413	0,49993802
Casc4	112,5352685	-0,68874167	0,240796182	-2,860268237	0,004232828	0,061069276
Hn1l	932,8661102	-0,526148428	0,120984688	-4,348884436	1,37E-05	0,001082429
Scx	2,265484775	-0,36500285	1,083601134	-0,33684244	0,736235678	NA
Alg8	214,8077075	-0,280766086	0,159460225	-1,760728014	0,078284449	0,34686144
Mirlet7d	1,850584139	-0,897223033	1,282779196	-0,699436844	0,484279069	NA
Cwf19l1	340,5369573	-0,049453001	0,115530982	-0,428049688	0,668614945	0,886564944
Amy1	5685,171155	0,3270069	0,106900056	3,058996527	0,002220797	0,040895094
Depdc1b	1,451578579	0,129394769	1,466133772	0,088255772	0,92967339	NA
Fcho2	532,3671822	-0,161520722	0,145143714	-1,112833046	0,26578014	0,626006345
Mir1198	0,12663974	0,780932884	3,352475198	0,232942181	0,815806304	NA
Samm50	2218,533813	0,219863533	0,119138787	1,84544041	0,064973499	0,310757431
Slitrk5	0,22911131	0,059593471	3,352475198	0,017775962	0,985817581	NA
Cox11	279,7916797	0,063418713	0,117705806	0,538790016	0,590031753	0,84953676
Lrrc75a	17,28681061	0,443295733	0,361133417	1,227512358	0,21963008	0,574490867
Lrrc75b	2,331177528	0,871443765	1,114021053	0,782250715	0,434067245	NA
Sec23a	1575,368217	-0,073904284	0,112455713	-0,657185676	0,511061535	0,809427989
Gcnt4	222,3524316	-0,652591111	0,215413254	-3,02948449	0,002449715	0,043266247
Nudt3	475,076591	-0,027391763	0,114900329	-0,238395862	0,811574077	0,942944131
Vwf	78,66408141	0,034343527	0,312617752	0,109857892	0,912522078	0,975960612
Vtn	68855,68152	0,008813165	0,102214395	0,086222343	0,931289669	0,980584977
Vegfb	421,0837548	-0,219020915	0,152584748	-1,435405029	0,151171714	0,483843582
Esrrb	18,67610042	-0,163121247	0,420746947	-0,387694429	0,698242179	0,898700268
Vdac2	2127,289975	0,10420198	0,07820115	1,332486543	0,182700361	0,527998513
Map3k2	421,8780183	-0,226905699	0,13037435	-1,74041672	0,081785872	0,353542587
Gstp1	23254,4377	-1,370307409	0,550787718	-2,487904804	0,012849812	0,121814305

Map3k1	359,6131378	-0,023447632	0,18071648	-0,129748167	0,896765673	0,971044462
Mras	103,2888088	-0,07729137	0,280304638	-0,275740601	0,782747309	0,93249269
Ptdss1	1088,348069	-0,168610594	0,10924345	-1,543438933	0,122724314	0,43508389
Tcam1	0,700546891	1,964020902	2,268651099	0,865721883	0,386642697	NA
Arl5b	188,8075884	-0,384306973	0,198979127	-1,931393401	0,053434421	0,278939777
Slamf6	12,30991185	0,527838742	0,50578314	1,043606835	0,296667329	0,657874966
Tmprss5	0,417017501	-1,327209753	2,517155588	-0,52726568	0,598009104	NA
Pcdhb14	4,784003902	0,153638304	0,730049494	0,210449161	0,833317126	NA
Zfp423	5,125045647	0,026307336	0,673618956	0,039053734	0,968847548	NA
Eldr	2,061517845	-1,587294786	1,04168512	-1,523776002	0,127564676	NA
Pdha1	3108,364727	0,007299997	0,097204984	0,075098995	0,940135947	0,983128178
Bhlha15	27,68344455	-2,452042804	0,406049784	-6,038773815	1,55E-09	7,16E-07
Meis1	92,42564758	-0,176942872	0,160269701	-1,104031959	0,269579275	0,630070861
Cldn4	5,367931078	1,500558084	0,801219277	1,872843212	0,061090039	NA
Celsr1	795,974151	-0,26087107	0,188817375	-1,381605217	0,167092951	0,506067344
Calb1	0,187906191	-1,241948083	3,339949325	-0,371846385	0,710007226	NA
Pdia4	7616,486704	-0,661786016	0,161976839	-4,085682981	4,39E-05	0,00239809
Ercc4	191,6529622	0,113794355	0,136780311	0,83194982	0,405437269	0,745264401
Nelfcd	729,6662486	-0,005456464	0,136842838	-0,039873946	0,968193623	0,991231429
Sema6a	105,3722479	-0,218709162	0,256692468	-0,852027968	0,394198569	0,736368577
Nphp1	92,77224195	0,327491959	0,208831898	1,568208504	0,11683248	0,424156926
Bet1l	413,5399949	0,010130381	0,110936361	0,091317047	0,927240673	0,978888597
Rp9	838,4727422	0,116465753	0,109735462	1,061331969	0,288539062	0,649625922
Htr1b	0,087021394	-0,517475177	3,352475198	-0,154356154	0,877328924	NA
Pja2	910,2655588	-0,3972509	0,136829275	-2,903259562	0,003693004	0,05655415
Kdm7a	330,4009843	-0,173655899	0,180085839	-0,964295135	0,334897979	0,691360575
Fbxl6	370,0722843	0,163695889	0,156924268	1,043152158	0,296877827	0,657874966
Fbxw5	1018,8412	-0,221208435	0,076590059	-2,888213398	0,003874369	0,058002735
Fbxw4	253,2762578	-0,139744088	0,108741532	-1,285103176	0,198756232	0,548812381
Cntn6	0,113662148	0,780932884	3,352475198	0,232942181	0,815806304	NA
Timm13	1265,999123	-0,034791304	0,107925175	-0,322365044	0,747176163	0,91954512
Heyl	83,46986079	-0,259375243	0,220728816	-1,175085554	0,239960489	0,59751011
Hey2	4,428712141	-0,493611476	0,688081907	-0,717373137	0,47314389	NA
Eml5	90,72768852	-0,052459433	0,243401049	-0,215526732	0,829356672	0,948231365
Fxr1	1117,087615	0,100752569	0,07674376	1,312843788	0,189235592	0,536686321
Gm32511	36,75774973	-0,160390264	0,293051016	-0,547311748	0,584164574	0,847068435
Mir8112	14,68734411	0,441595484	0,432129056	1,021906482	0,306825164	0,666116534
Cbx4	421,6864854	0,242772101	0,221298181	1,097036134	0,272625597	0,63463897
Commd6	590,5818825	-0,029654952	0,092178333	-0,321712831	0,747670257	0,919586841
Ces2h	6,602720034	0,161780714	0,630591893	0,256553749	0,797523283	0,938325224
4921531C22Rik	35,11271677	-0,044658024	0,284440204	-0,157003207	0,875242321	0,964021167
2810001G20Rik	41,03015363	0,224791034	0,271545715	0,827820221	0,407772321	0,746679655
Minos1	962,4635681	0,071784512	0,12919144	0,555644493	0,578453909	0,84530168
Ttc7b	346,8417291	-0,284329831	0,119025874	-2,388806918	0,016903183	0,14387057
A630072M18Rik	81,37060429	-0,324097281	0,181043911	-1,790158416	0,073428448	0,333672645
Fbxl12os	5,527963264	-0,0384311	0,631092424	-0,060896151	0,951441914	0,985972877
Ces2d-ps	7,794923992	-0,900110135	0,579890688	-1,55220657	0,120612805	0,432066609
Elmo2	294,3528847	-0,093069527	0,13160927	-0,707165434	0,479463677	0,791837124
Particl	27,51802685	0,561882096	0,350755813	1,601918131	0,109173715	0,40910239
Dapp1	27,43758224	0,550986996	0,298613107	1,845153419	0,065015224	0,310757431
Hmgb1	1899,844074	0,029839676	0,083792634	0,356113356	0,721755675	0,909525085
Fam133b	191,0979197	-0,167074506	0,185484302	-0,900747417	0,367722631	0,716004058
Timm23	941,5193583	0,083529925	0,110420954	0,756468065	0,449368613	0,773349269
Map3k14	98,17436071	0,184748218	0,226875407	0,814315753	0,415464087	0,751551608
Stam2	373,784594	0,153815104	0,110738793	1,388990255	0,164835713	0,50334664
Pias1	320,0249035	0,041884664	0,107095064	0,391097987	0,695724809	0,897068317
Ywhag	2402,343054	-0,272553248	0,12999192	-2,096693758	0,036020693	0,222224573
Grk5	276,7439235	-0,230080719	0,265864896	-0,865404658	0,386816726	0,731036585
Asna1	813,7016511	-0,18962074	0,094830107	-1,999583739	0,045545231	0,252968092
Ptpn9	573,4636282	0,334560458	0,104691435	3,195681279	0,001395011	0,0296973
Akap5	11,06002528	0,100419873	0,434001393	0,231381454	0,817018472	0,944998904
Ptpn23	514,0271544	-0,040892214	0,123074616	-0,332255465	0,739696365	0,916067243
Zfp956	89,14880032	-0,032731343	0,168389755	-0,194378472	0,845879545	0,953584926
Thoc2	544,7408458	-0,203897632	0,136105245	-1,498087991	0,134110391	0,456110978
Mir703	17,76618868	0,0290049	0,60939364	0,047596329	0,962037958	0,988526411
Ttc30b	122,0867366	0,077193818	0,209287178	0,368841603	0,712245793	0,905478699

Supplementary Table S1: *Serpina1* KO vs. wildtype all DEGs

Cyp2s1	12,76129135	1,679541866	0,639175541	2,627669174	0,008597208	0,096020113
Tfip11	609,7324426	0,004413611	0,085662819	0,051523065	0,958908723	0,987896001
Glb1l	84,14863293	0,057942192	0,196253795	0,295241129	0,767809685	0,927586634
Tex38	2,263451642	2,419172713	1,113735748	2,172124508	0,029846273	NA
Cygb	206,6697671	0,376238136	0,230418811	1,632844709	0,102501661	0,398751316
Strip2	1,440195501	0,531274096	1,343141701	0,395545828	0,692440108	NA
Mir7054	0,172953423	1,307385949	3,350719078	0,39018071	0,696402925	NA
Prr16	830,9589221	-0,107668863	0,162620413	-0,662087013	0,507915459	0,807274415
Abcg2	2396,1991	-0,67332884	0,241697334	-2,785834778	0,005339008	0,070973656
Pcdhgb1	2,976174108	-0,318077598	0,958545193	-0,3318337	0,740014836	NA
2410124H12Rik	0,122496332	0,780932884	3,352475198	0,232942181	0,815806304	NA
Fam227a	9,129280753	-0,337884218	0,524498246	-0,644204666	0,519442726	0,813866972
Zfp346	103,2884664	0,078180542	0,178009053	0,439194189	0,660520835	0,882607568
Xrn2	965,0233482	0,048081985	0,122203259	0,393459108	0,693980421	0,896061836
Lrfn5	0,086476712	0,780932884	3,352475198	0,232942181	0,815806304	NA
Tmem260	542,8004133	-0,120524045	0,100118047	-1,203819373	0,2286594	0,584390655
Wif1	1,971474828	0,460040361	1,179206784	0,390126963	0,696442666	NA
Nelfa	358,6678817	0,007656984	0,127513119	0,060048599	0,952116928	0,986187919
Copa	5340,807921	-0,296287926	0,118389454	-2,502654725	0,012326573	0,119329441
Slc30a3	13,04064046	-1,778188777	0,448445397	-3,965229185	7,33E-05	0,003564684
Acot8	285,0516197	0,0065684	0,164106042	0,040025337	0,968072926	0,991231429
Cd209a	5,195712321	0,270227307	0,790172479	0,341985218	0,732362017	NA
Apcdd1	1,291902426	0,454016201	1,190701862	0,381301327	0,702979669	NA
Tm9sf3	3650,735471	-0,230765827	0,125760231	-1,834966632	0,066510634	0,316036222
Fubp3	420,3957262	-0,030146962	0,103374399	-0,291628896	0,77057038	0,927934035
Fermt1	17,18558542	1,182952399	0,442859752	2,671167096	0,007558801	0,088565827
Acer2	191,3230665	-0,152107717	0,200736172	-0,757749416	0,44860101	0,77297141
Pdzd4	3,586991592	-0,23330842	0,729889607	-0,319648914	0,74923449	NA
Tssk2	0,174042788	-1,166820872	3,345560292	-0,348766954	0,727264274	NA
Sez6l2	9,28682577	0,283168497	0,533694848	0,530581283	0,595708969	0,853045544
Gm38426	171,3477996	-0,398867058	0,175729018	-2,269784827	0,023220642	0,172167884
Cttn	1185,529036	-0,098471062	0,09953438	-0,989317083	0,322508029	0,67984555
Stx7	482,5145366	0,028635325	0,100455251	0,28505553	0,775601599	0,929623254
Fbxo17	39,1222007	-0,14713949	0,331657236	-0,443649269	0,657296178	0,880359254
Pdxp	9,700139748	-0,21745853	0,517771805	-0,41998913	0,674493395	0,888627551
Wisp2	4,316715308	0,340320099	0,783303082	0,434467969	0,66394865	NA
Fbxo16	0,813127813	2,074109005	2,214498732	0,936604287	0,348962146	NA
2610306M01Rik	30,55279791	0,125015677	0,2831153	0,441571605	0,658799236	0,881654058
Cpsf6	573,1822031	-0,219873926	0,155458104	-1,414361301	0,157255847	0,493047236
Ankrd66	0,093953095	-0,517475177	3,352475198	-0,154356154	0,877328924	NA
Zfp850	6,362227575	0,377128022	0,564648824	0,667898356	0,504198477	0,804997854
BC035044	11,52933746	-0,203028602	0,444973905	-0,456270805	0,648195255	0,876446263
BC017643	233,2872464	0,010927722	0,116344119	0,093925869	0,925168044	0,978216671
Tnfsf18	0,113662148	0,780932884	3,352475198	0,232942181	0,815806304	NA
Fat4	35,68709181	-0,360082925	0,351447883	-1,024569906	0,30556617	0,664636701
Zbtb26	93,83835583	-0,226718543	0,164899731	-1,374887283	0,169166392	0,508707843
Shc4	3,96009443	-0,200286757	0,756478467	-0,264762006	0,791192807	NA
C1qtnf9	9,425444963	0,223626827	0,544754393	0,410509451	0,681432272	0,89166825
Ccp110	107,5533267	-0,374887687	0,161993382	-2,314216063	0,020655873	0,160578725
Stard8	190,3201436	0,220858584	0,188357781	1,172548239	0,240977017	0,598512498
9230110C19Rik	5,725593075	1,460515969	0,687385357	2,124741171	0,033608216	0,214233402
Enpp4	278,3099198	-0,202539477	0,131119319	-1,544695918	0,122419833	0,434465289
Phf11d	60,66404854	0,019574247	0,202100292	0,096854127	0,922842239	0,977458239
Xkrx	2,579311872	-0,888084244	0,926460212	-0,958577856	0,337771452	NA
Alk	0,81485876	2,21511801	2,20264068	1,005664714	0,314576882	NA
Crmp1	0,597562776	-0,090069237	1,846780378	-0,048770952	0,961101832	NA
Entpd8	2297,578826	0,02659764	0,154501701	0,172151116	0,863318728	0,961272625
Mblac1	29,88542908	0,572712361	0,316468886	1,809695631	0,070343	0,324561524
Kremen2	0,093303375	-0,517475177	3,352475198	-0,154356154	0,877328924	NA
Tmem138	43,61288881	0,451027129	0,247229565	1,824325213	0,068102921	0,319375503
Thap12	802,5339353	0,235652172	0,148085492	1,591325177	0,111536414	0,413513675
Nsun7	5,35759733	-0,169720316	0,645370393	-0,262981255	0,792565027	NA
Elovl7	12,82929292	0,960626475	0,500591292	1,91898359	0,054986411	0,28404688
Top1mt	606,0874276	0,167728667	0,119509808	1,403471986	0,16047614	0,497178481
Hspb11	109,9731469	0,23442477	0,173811779	1,348727752	0,177424428	0,520144503
Fcho1	12,6970519	1,232554885	0,535602448	2,301249536	0,02137753	0,163367723

Supplementary Table S1: *Serpina1* KO vs. wildtype all DEGs

Ydjc	1,897764346	-0,19159882	1,411646501	-0,135727195	0,892036948	NA
Rftn2	16,84942143	0,204648933	0,422021106	0,484925824	0,627728963	0,865852703
Slc25a27	25,42776928	0,531984493	0,327881477	1,622490231	0,104698423	0,402447734
Abcd2	542,1095605	0,91188234	0,644910347	1,413967608	0,157371414	0,493178253
Ube2d1	204,520426	0,203759082	0,136174003	1,49631411	0,13457182	0,456327747
Cd248	23,23782532	0,140929273	0,310418686	0,453997388	0,649830707	0,87680086
AA414768	2,364734404	-0,450296298	0,926275834	-0,48613629	0,626870538	NA
Prob1	210,0731047	0,461296332	0,179348613	2,572065242	0,010109384	0,105423052
Pibf1	61,93608648	0,336272688	0,19202491	1,75119305	0,079912662	0,34992512
Spon2	447,0556601	-0,760334329	0,151055589	-5,033473652	4,82E-07	7,21E-05
Zc2hc1c	87,82637564	-0,567933622	0,250359762	-2,26847005	0,023300572	0,172483584
Rap2c	766,7168602	0,090021926	0,109821982	0,819707716	0,41238275	0,749317374
Gpc2	4,171827634	-0,2295833	0,745974794	-0,307762811	0,758262821	NA
Bnip1	437,3150182	0,202670959	0,105869933	1,914339174	0,055576832	0,285223289
1700095A21Rik	0,322959367	-1,877436289	2,642076408	-0,710591216	0,477337588	NA
3110009F21Rik	0,147722973	0,780932884	3,352475198	0,232942181	0,815806304	NA
4930548J01Rik	0,501718624	-1,484104967	2,418581897	-0,613626096	0,539462435	NA
1700110C19Rik	0,947189566	-0,802204449	1,828540351	-0,438713014	0,660869496	NA
4933433H22Rik	0,215799707	0,059593471	3,352475198	0,017775962	0,985817581	NA
3010001F23Rik	0,236587694	-1,308576888	3,335186821	-0,392354899	0,694796005	NA
4933438K21Rik	0,12663974	0,780932884	3,352475198	0,232942181	0,815806304	NA
2700054A10Rik	3,193552993	1,041079566	0,974565473	1,068249999	0,285407757	NA
2010106C02Rik	0,360209894	-0,130834948	2,660913913	-0,049169177	0,960784475	NA
Prickle1	58,97019479	-0,032505169	0,310263674	-0,104766273	0,916561273	0,976559041
Rraga	907,3113284	-0,050756817	0,097360665	-0,52132776	0,602138466	0,854563604
Ptprh	1,117407943	2,602897547	1,920808664	1,355105064	0,175384086	NA
Ccdc87	0,559699033	0,62796718	2,388360228	0,262928169	0,792605945	NA
Wdfy2	92,58115253	-0,27461486	0,191042844	-1,437451701	0,150589687	0,483083894
Pcyt1b	8,370722415	1,84277162	0,76046282	2,42322382	0,015383445	0,135929466
Mettl7a2	0,353822874	-1,099898186	2,602809238	-0,422581175	0,672600864	NA
Rrm2b	66,3752245	-0,006814403	0,205446049	-0,033168822	0,973539961	0,992775209
Nexn	20,40284361	0,192162743	0,356660363	0,538783569	0,590036202	0,84953676
Cdh24	10,99669554	-0,493334737	0,504730617	-0,977421857	0,328360347	0,685487182
Atp5e	1996,65295	0,11893651	0,136682596	0,870165719	0,384209847	0,728788514
Eloc	771,3215922	0,01654395	0,088026317	0,187943223	0,85092116	0,95564222
Ccdc38	13,7345325	-0,207279147	0,446746804	-0,46397455	0,64266598	0,873061831
Tubb2a-ps2	0,093303375	-0,517475177	3,352475198	-0,154356154	0,877328924	NA
Nipal2	8,212909846	0,940144866	0,581395832	1,617047825	0,105867958	0,403578195
Hps4	308,1097147	-0,004874378	0,172980632	-0,02817875	0,977519585	0,994348571
Itgbl1	6,452000909	0,006680576	0,735517153	0,009082829	0,992753051	0,99797846
Ggact	1885,425399	0,005146161	0,080704513	0,063765463	0,949156979	0,985650844
Gys2	3002,55171	-0,094123354	0,153507342	-0,613152128	0,539775755	0,826407188
Shisa2	8,914993716	0,718997832	0,57047055	1,260359246	0,207539796	0,559959544
Haus4	256,4372952	-0,089508874	0,152788628	-0,585834662	0,557986628	0,834302421
Zfp503	39,97513252	-0,140501523	0,491260232	-0,286002233	0,774876411	0,929351416
Oxnad1	892,9589868	-0,13067601	0,105355966	-1,240328524	0,214853906	0,56833832
Pxk	696,15164	-0,1742403	0,09433026	-1,847130488	0,064728231	0,309828757
Zswim6	74,66779193	-0,007174458	0,272689354	-0,026310002	0,979010077	0,994593468
Zcchc9	215,860912	-0,063096993	0,156580286	-0,402968948	0,686971068	0,89330962
Rasa1	527,9692953	-0,057009837	0,119698063	-0,476280367	0,633874651	0,867989994
Gpx6	21,74484738	0,564381573	0,426641218	1,322848213	0,18588586	0,532805747
Ifi27l2b	148,8953644	1,251816749	0,3097539	4,041326837	5,31E-05	0,002819672
9030617O03Rik	1161,319241	0,099830882	0,101072243	0,987718089	0,323290733	0,680855987
Grrp1	0,830243309	-0,694993932	1,704396266	-0,407765463	0,683445872	NA
P2ry4	149,2540318	-0,236826104	0,313540099	-0,755329556	0,450051269	0,773657634
Mogs	1485,184886	-0,405174456	0,101536882	-3,990416559	6,60E-05	0,003282365
Smarce1	801,9850162	-0,042039421	0,081671965	-0,514735026	0,606738194	0,856921485
Akip1	452,9327525	0,096189702	0,166272215	0,57850737	0,562921623	0,83722511
Nat8f3	0,633838037	-1,335181351	1,979740168	-0,674422519	0,50004273	NA
Exosc7	561,8956563	0,143638532	0,122577993	1,171813382	0,241271989	0,598879543
Htra4	25,10370638	0,65702263	0,518771499	1,266497159	0,205335146	0,558251976
Stx1a	9,738023676	-0,199165292	0,489315458	-0,407028409	0,683987123	0,892293506
Mpp2	6,483426238	0,115027554	0,64674949	0,177854882	0,858836945	0,958796604
C6	6563,882238	-1,205658614	0,567822235	-2,123302929	0,033728475	0,214383309
Pcdhga5	3,030176387	0,369722399	0,863503604	0,428165438	0,668530677	NA
Zbtb33	493,3102792	-0,276318441	0,197137954	-1,401650143	0,161019745	0,497842964

Supplementary Table S1: *Serpina1* KO vs. wildtype all DEGs

Nasp	363,4011545	-0,05732437	0,142167502	-0,403217114	0,686788511	0,893245997
Mybbp1a	1505,920385	-0,220275491	0,112968058	-1,949891808	0,051189016	0,272865212
Atp5b	31664,73184	-0,000454025	0,055804257	-0,00813603	0,993508459	0,99822248
Ech1	6784,364193	-0,067031258	0,103785481	-0,645863541	0,518367733	0,813725618
Mir7669	1,07810643	1,129088386	1,666160482	0,677658844	0,497988027	NA
Mir7668	0,093953095	-0,517475177	3,352475198	-0,154356154	0,877328924	NA
Mir7667	0,219943115	0,059593471	3,352475198	0,017775962	0,985817581	NA
Cct6b	15,92976713	0,207216336	0,413757355	0,50081608	0,616500568	0,860513269
Oxct2b	0,483352137	-0,058222963	2,923182374	-0,019917663	0,984109055	NA
Trmt6	316,9333043	-0,195416751	0,140760357	-1,388293944	0,165047555	0,503431976
Vwa2	0,12663974	0,780932884	3,352475198	0,232942181	0,815806304	NA
Slc16a12	1054,217042	-0,241936914	0,176182683	-1,373216198	0,169685146	0,509436495
9930021J03Rik	563,9469893	-0,245233988	0,150885854	-1,625294758	0,104099768	0,401498609
Loxhd1	0,086476712	0,780932884	3,352475198	0,232942181	0,815806304	NA
Malt1	181,8334885	0,102760075	0,115785883	0,887500897	0,374809284	0,720996253
Pcyox1l	32,18699323	-0,369468371	0,336902394	-1,096662944	0,272788761	0,63465219
Slc38a2	1751,092488	-0,100814512	0,148232313	-0,680111578	0,496433814	0,800292254
Dact2	430,7246344	0,002951865	0,105288982	0,028035842	0,977633565	0,994348571
Pnpla7	3932,625938	-0,133269015	0,120344904	-1,107392251	0,268124375	0,628492966
Ccdc14	22,28172375	0,334450162	0,359077705	0,931414446	0,351639221	0,702880198
Lmln	30,27383242	-0,709100955	0,356241047	-1,990508847	0,04653491	0,256440732
Pigz	3,159845629	0,2404966	0,816803849	0,294436174	0,768424629	NA
Palm2	0,467816316	-2,450022923	2,80824434	-0,87243937	0,38296873	NA
Ccnj	75,58379829	0,150111462	0,22228527	0,675309982	0,499478843	0,802860694
Ttll8	6,374672554	-1,779145544	0,775174575	-2,295154668	0,021724262	0,164550515
Zfp647	16,69622219	0,06459166	0,416908564	0,154930039	0,876876472	0,96453526
Slc30a8	1,65885389	2,561637693	1,346539257	1,902386194	0,057120685	NA
Map3k13	379,2910669	-0,545055752	0,291995055	-1,866660901	0,061948957	0,301977792
Lrp12	89,89182005	-0,203775419	0,195771715	-1,040882839	0,297929927	0,658568981
Enox1	0,551742692	1,342664827	2,402066313	0,558962431	0,57618736	NA
Ttll6	0,087021394	-0,517475177	3,352475198	-0,154356154	0,877328924	NA
Ppwd1	178,4964351	0,14784071	0,138906048	1,064321623	0,287183026	0,648651236
Slfn9	49,00630635	-0,286856435	0,320177723	-0,895928773	0,370290806	0,718388882
Btbd7	183,5332119	-0,248854917	0,255599416	-0,97361301	0,330248724	0,686853639
Zfp454	47,08838796	-0,256060053	0,261378391	-0,979652724	0,32725757	0,684930268
Btnl9	26,99175167	-0,276036015	0,441561289	-0,625136355	0,531881569	0,820247398
Ivns1abp	2121,646827	-0,246001653	0,104065228	-2,363917881	0,01808282	0,149135857
Gnb1l	36,05967112	0,024012998	0,234527234	0,102388955	0,91844795	0,976907366
Mpzl3	53,87895121	0,070670371	0,255395532	0,276709504	0,782003174	0,932281116
Ahsg	273217,1107	-0,007088711	0,094443546	-0,07505765	0,940168843	0,983128178
Alad	3621,335989	-0,064135727	0,067107873	-0,955710921	0,339218304	0,694071514
Cass4	1,384355763	-1,47405156	1,236140553	-1,192462747	0,23307985	NA
Slc2a4	10,61660812	1,74481089	0,57732027	3,022258145	0,002508965	0,043600932
Fcnb	0,086476712	0,780932884	3,352475198	0,232942181	0,815806304	NA
Pdcd2	319,2497895	0,106644736	0,108960212	0,97874934	0,327703847	0,685077831
Selenbp1	8504,419664	0,652540689	0,086827347	7,51538213	5,67E-14	7,59E-11
Snord16a	0,860850379	0,716816515	1,568867008	0,456900752	0,647742384	NA
Fam171a1	79,47925378	-0,251605762	0,310707888	-0,809782345	0,418065281	0,752438711
Mdga1	2,851512333	-0,08068464	0,870905025	-0,092644592	0,926185917	NA
Capn3	42,51657551	-1,056443062	0,255396185	-4,136487248	3,53E-05	0,002067873
Tet1	1,465888743	-0,150683302	1,187257491	-0,126917121	0,899005996	NA
Asb13	1426,936016	-0,344943751	0,092633784	-3,723735933	0,000196296	0,007455354
Ankrd34a	0,300137846	0,617320176	2,751219827	0,224380535	0,822461216	NA
Rimklb	0,434883226	1,149382856	2,19554927	0,523505836	0,600622291	NA
Dcaf7	884,5751076	0,210424311	0,111397061	1,888957486	0,058897526	0,293376491
Hexim2	47,41371273	0,002647395	0,206334407	0,012830604	0,98976294	0,996771431
4933405L10Rik	0,710105535	0,994439334	2,022997399	0,491567282	0,623025277	NA
Ttc9	0,88732694	-0,155942612	1,706509564	-0,091381036	0,92718983	NA
Lin54	165,4874426	0,022308635	0,195593966	0,114055844	0,909193525	0,9747592
Exosc5	503,2051404	-0,041440968	0,131878838	-0,314235162	0,753342439	0,921533083
Slc25a36	25,10146419	0,512905908	0,331335608	1,54799513	0,121623469	0,433672149
Tango2	874,925229	0,135957928	0,143850963	0,945130469	0,344592272	0,697587612
Vwa7	9,847719348	-0,319662048	0,527710589	-0,605752573	0,544679067	0,827491596
Tcf7l1	252,7258096	-0,538502495	0,174870664	-3,079433002	0,00207395	0,038941909
Ralgapa1	186,128243	-0,145042464	0,141519205	-1,024895978	0,305412273	0,664560005
Rsad2	281,8543624	0,435343244	0,238837252	1,822761067	0,068339587	0,319740014

Supplementary Table S1: *Serpina1* KO vs. wildtype all DEGs

Dctn5	697,5080033	0,01292452	0,090207546	0,143275378	0,8860727	0,96772371
Suclg1	5066,020005	-0,010090893	0,091131825	-0,110728525	0,911831625	0,975612405
Copz2	472,4586783	0,251828917	0,117521918	2,14282511	0,032127144	0,208308055
Fetub	15439,8908	0,219375921	0,127751792	1,717204255	0,085941858	0,363485758
Clstn2	5,400252848	0,15054947	0,780950863	0,192777135	0,847133511	0,953824301
Aatf	364,3524145	0,061275299	0,121731849	0,503362921	0,614709136	0,859808165
Ppp4c	750,0922463	0,061330863	0,090056438	0,681026968	0,495854426	0,80006558
Rpl35a	3216,592023	-0,039745592	0,135179398	-0,294021073	0,768741801	0,927598042
Spib	13,30618651	0,286000011	0,447881091	0,638562371	0,523107667	0,815322684
Rpl36a	2165,646814	0,045586873	0,14215417	0,320686144	0,748448249	0,919838327
Atr	298,191121	0,121010378	0,146924306	0,823623957	0,410153259	0,747558135
Ctsf	666,3928927	-0,31662695	0,087618775	-3,613688407	0,000301872	0,010095144
Noa1	312,6665337	-0,143010307	0,144707038	-0,988274719	0,323018123	0,680496263
B4galt5	599,4802715	-0,614024415	0,227085835	-2,703930942	0,006852453	0,083591067
Git2	329,3854825	0,094444304	0,124071651	0,761207761	0,446532983	0,771877354
Fam69b	2,566149278	1,786162698	0,97164375	1,8382897	0,066019728	NA
Gkap1	252,807451	0,045229969	0,114850947	0,393814509	0,693717992	0,895984052
Sult1b1	590,9482773	0,511200664	0,164924246	3,099608925	0,001937763	0,037599354
Cxcl10	79,8127432	0,111379445	0,27746949	0,401411503	0,688117182	0,893843626
Dnajb5	147,1348331	-0,304346448	0,206198536	-1,475987435	0,139947265	0,465064626
Pdcd5	609,1978259	-0,024152818	0,11268705	-0,214335348	0,830285555	0,948720627
Ngef	568,4127521	0,027354937	0,102528019	0,266804503	0,789619687	0,935361858
1700061G19Rik	0,087021394	-0,517475177	3,352475198	-0,154356154	0,877328924	NA
Rflnb	20,59420455	-0,08497088	0,376090358	-0,225932088	0,82125424	0,946779723
Zfp449	16,38965768	-0,031350622	0,404505708	-0,077503535	0,938222979	0,982847752
Rps9	6294,210894	0,024592461	0,109313931	0,224970968	0,822001859	0,946779723
Srbd1	419,3392691	0,082334741	0,156946163	0,524604995	0,599857814	0,85441073
Zfp395	920,0584985	-0,082297077	0,233597091	-0,352303517	0,724610651	0,910252319
Bend3	115,4001321	-0,092953712	0,167876637	-0,553702494	0,579782468	0,84530168
3110021A11Rik	1,521595195	1,254114681	1,292640054	0,970196364	0,331948623	NA
Gm128	0,440535951	-0,057726528	2,529251586	-0,022823561	0,981791014	NA
Gm4951	3496,521379	-0,201023806	0,104834424	-1,917536238	0,055169842	0,284116184
Gm20939	59,83507126	0,138218516	0,261983473	0,527584869	0,597787497	0,853597141
Arfgef2	1294,41685	0,013779757	0,121538651	0,113377569	0,909731222	0,9747592
Zfp362	184,0180558	-0,211180555	0,166399401	-1,269118479	0,20439881	0,557220166
Fam186b	0,302276418	0,61733957	2,745727361	0,224836442	0,822106515	NA
Fbxo43	0,371745909	-0,928546775	3,151455697	-0,294640593	0,768268449	NA
Abcd3	15941,47564	-0,031249765	0,177404615	-0,176149671	0,860176359	0,959267413
L1cam	3,067368987	1,151820045	0,851717033	1,352350605	0,176263183	NA
Scd2	269,2202411	0,511847867	0,267084313	1,916428045	0,055310634	0,284475895
Scd1	287886,706	0,517949568	0,351299577	1,474381415	0,140378926	0,465404335
Ubiad1	258,2698501	-0,012717947	0,145269974	-0,087546975	0,930236748	0,980359737
Spcs1	1441,394999	-0,046874567	0,093180832	-0,503049456	0,614929501	0,859936455
Thap7	365,4590435	0,173637289	0,117718583	1,475020209	0,14020711	0,465234266
Rnaseh2a	410,7848865	-0,351497949	0,161049554	-2,182545312	0,029069311	0,196651683
Mzb1	5,061007497	0,298053086	0,738492664	0,403596542	0,686509429	NA
Wipf3	154,2168875	0,883474261	0,278789968	3,168960013	0,001529854	0,031758732
Map1a	6,62123641	0,172130999	0,617146365	0,278914386	0,780310528	0,931606698
Bex3	31,97824442	0,443739431	0,312481898	1,420048438	0,15559358	0,490622294
Rev1	262,641768	-0,016238511	0,121515517	-0,133633228	0,893692608	0,970023258
Was	41,62490185	0,195815937	0,322122335	0,607893077	0,543258392	0,82684051
Vpreb3	5,690512172	-0,453708498	0,6811379	-0,666103733	0,505344799	0,805643837
Igf1r	39,85203447	0,343712445	0,25814394	1,331475937	0,183032457	0,528729672
Dhcr24	17772,30175	0,448824352	0,188032022	2,386957004	0,016988478	0,144212847
Loxl2	81,92640413	-0,416265117	0,283317052	-1,46925543	0,141763521	0,467844115
Dgcr8	257,1589196	-0,135033621	0,141915203	-0,951509196	0,341345952	0,695224564
Hes2	0,113662148	0,780932884	3,352475198	0,232942181	0,815806304	NA
P2rx5	2,284859778	0,470307103	1,367880322	0,343821821	0,730980289	NA
Glce	653,5123386	-0,105383231	0,134868633	-0,78137688	0,434580865	0,764361477
Slco2a1	3173,059149	-0,662215553	0,139007424	-4,763886238	1,90E-06	0,000220444
E2f6	328,3608684	-0,083606839	0,119714928	-0,69838274	0,484937868	0,79420977
Actn2	0,52691746	2,517419776	2,305576007	1,091883229	0,27488443	NA
Mir7027	0,647602079	0,753391228	2,027077528	0,371663746	0,710143221	NA
Pitx3	5,046111731	-0,593172236	0,664948732	-0,892057097	0,372362329	NA
Tnfrsf18	3,299085977	0,557052633	0,776180378	0,717684507	0,472951838	NA
Sat1	1124,833335	0,438283631	0,13672006	3,205700993	0,001347339	0,028866309

Sap18	360,1706957	0,105712582	0,094488982	1,118782099	0,26323311	0,623393822
Ehd1	1083,396423	-0,021696041	0,085403759	-0,254040817	0,799464025	0,93892144
Nfatc3	810,797476	-0,148991069	0,119174835	-1,250189005	0,211230512	0,564073259
Pfdn6	641,1339276	-0,131280653	0,147669728	-0,889015338	0,373994837	0,720823634
Kdsr	381,2567445	0,057281939	0,131253666	0,436421629	0,662530849	0,883417459
Bcl2a1b	19,86539495	0,922759083	0,38372083	2,404766723	0,016182792	0,140121599
Phb2	4679,525683	0,139532681	0,069413239	2,010173904	0,044412786	0,249002293
Bcl6b	52,48296975	0,136435664	0,331308067	0,41180906	0,68047938	0,891658301
Bax	489,7099583	-0,257409991	0,131272605	-1,960881251	0,049892877	0,268094
Dsg2	3947,857266	-0,106006292	0,124574802	-0,850944897	0,394799963	0,736750517
Gm6614	2,601657699	-2,020678433	1,101036469	-1,835251139	0,066468487	NA
Camsap2	361,7343548	-0,306667271	0,12330585	-2,487045601	0,012880889	0,121957933
Ldha	14870,44658	-0,177873263	0,107974963	-1,647356562	0,099484778	0,392688228
Vma21	574,7080503	-0,00374065	0,167755818	-0,022298185	0,982210096	0,995338444
Ttc37	354,0586958	-0,161997227	0,161576491	-1,002603941	0,316051993	0,67419376
Zfp800	144,1526808	0,015306948	0,160289994	0,095495342	0,92392139	0,977951927
Socs1	10,70394282	-0,113050948	0,448219114	-0,252222506	0,800869083	0,939606737
Hist1h1t	0,361299258	-2,093941636	2,509883782	-0,834278324	0,404124163	NA
Mir7060	0,35273351	0,909632601	3,18640607	0,285472906	0,775281859	NA
Etv1	8,499519611	-0,552515513	0,529907223	-1,04266462	0,297103649	0,658144463
Prdx3	2590,969079	0,223092536	0,091331184	2,442676487	0,014578799	0,131336907
Snord52	6,960241691	0,192629799	0,705081188	0,273202296	0,784697715	0,933246486
Rab42	1,750035592	-0,935570883	1,115648065	-0,838589617	0,401699639	NA
Ctsc	2525,196663	-0,021135139	0,163562018	-0,129217891	0,89718524	0,971044462
Smarcb1	528,036591	-0,048567046	0,127120155	-0,382056226	0,70241966	0,900494767
Gdi1	1533,706981	-0,205643658	0,100968361	-2,036713844	0,041678726	0,241064272
Srp14	874,5313895	-0,097154953	0,094027797	-1,033257788	0,301483276	0,661958526
Srm	962,2778703	-0,682550087	0,184593289	-3,697588842	0,000217657	0,008029789
Gpd1	11147,06817	-0,259451806	0,13290052	-1,952225659	0,050911419	0,271818994
Sptlc1	536,6580131	-0,096423972	0,10169066	-0,948208736	0,3430232	0,695663554
Traf4	949,5497189	-0,185097987	0,103585687	-1,786906986	0,073952523	0,335256454
Sqle	3491,177038	0,659758656	0,161284806	4,090643573	4,30E-05	0,002366686
Mucl1	0,113662148	0,780932884	3,352475198	0,232942181	0,815806304	NA
Gda	640,5800117	0,204895282	0,160313648	1,278090072	0,201217669	0,552377621
Ddx5	8280,765227	0,116389664	0,105037556	1,10807666	0,26782871	0,628175794
Car5a	1788,942963	0,24586302	0,148450343	1,656197054	0,09768192	0,389224129
Car3	101261,1129	-0,61349803	0,189883496	-3,230918136	0,001233933	0,027678602
Caml	395,0352499	-0,008481361	0,15636973	-0,054239146	0,956744632	0,987715172
Ip6k1	1118,406107	0,08118933	0,10603672	0,765671834	0,44387159	0,770142004
Mkln1	1562,422517	-0,037377266	0,09861129	-0,37903638	0,704660849	0,901666433
Ube2d3	4501,956876	0,052585867	0,155098823	0,339047491	0,734573954	0,913706661
Vps37a	1053,68058	-0,228930354	0,12195317	-1,877198876	0,060490842	0,29803066
Tkfc	8517,924751	0,189276152	0,216340381	0,874899782	0,381628438	0,72637014
Sgip1	6,515939086	0,055917293	0,781517266	0,071549658	0,942960305	0,983437952
Gatb	863,5125621	-0,032809848	0,089719766	-0,365692533	0,714594519	0,906371815
Tmem268	235,5900515	0,105103278	0,162075482	0,648483516	0,516672269	0,813424627
Pomt1	539,3942096	0,311192204	0,165132591	1,884499008	0,059497509	0,294928511
Csdc2	12,40445377	0,306900945	0,43759188	0,701340586	0,483090491	0,79399946
Stk24	464,6474414	0,03679773	0,102133321	0,360291134	0,718629429	0,90815359
Hoxb5os	1,022331717	0,375819578	1,492154857	0,251863656	0,801146452	NA
Ncoa5	430,0307465	-0,367807148	0,159021475	-2,312940117	0,020725932	0,160722147
Slc35a3	763,8321257	-0,280706301	0,162104377	-1,731639247	0,083337814	0,357211681
Atp1a1	6520,278013	0,162683411	0,098264163	1,655572139	0,097808496	0,389398983
Trpv3	0,31324649	-0,658042201	3,258021693	-0,201976004	0,839935484	NA
Zdhhc5	1930,219975	-0,108745097	0,103024284	-1,055528784	0,291183552	0,653114648
Klf7	25,57190644	-0,345945446	0,358165382	-0,965881861	0,3341033	0,690638919
Rpl30	16,22747106	0,20150562	0,370017272	0,544584361	0,586039413	0,847917848
Itga4	61,58421696	0,597135202	0,283982405	2,102719013	0,035490339	0,220116801
Lrrc8c	23,38018743	-0,035649524	0,434884616	-0,081974672	0,934666854	0,981892281
Sptssa	733,9637258	0,286794256	0,211527774	1,355823161	0,17515544	0,517378056
Dapk1	2904,882731	0,041795934	0,13710357	0,304849347	0,760480896	0,925072539
Macrod1	933,6517303	0,420359859	0,145594626	2,887193509	0,003886951	0,058126003
Med1	488,1160638	-0,135002322	0,098789043	-1,366571813	0,171759557	0,512405749
Hpcal1	235,3160386	0,178576347	0,171755618	1,039711824	0,298473806	0,65922622
Psmb8	781,3650377	0,294199211	0,147739337	1,991339725	0,046443551	0,256042817
Smc6	504,7381575	0,072896196	0,131721517	0,553411451	0,579981699	0,84530168

Rpl35	2440,508192	0,069236548	0,137478113	0,5036187	0,614529349	0,859778545
Mrps36	187,8336567	-0,185305944	0,163319987	-1,134618902	0,25653503	0,616948518
Fam213b	376,690998	0,085366652	0,19755175	0,432122988	0,665652028	0,884945574
Chchd1	679,1410548	0,070809551	0,107381221	0,659422104	0,509624752	0,808332547
Etfdh	7881,584535	0,102227485	0,125354969	0,815504049	0,414783848	0,751551608
Tbcb	538,1995063	-0,263472573	0,104096351	-2,53104524	0,011372318	0,113885033
Fmc1	414,8025171	0,300408354	0,137605465	2,183113536	0,02902745	0,196651683
Tmed3	579,058565	-0,728729724	0,116578272	-6,250990983	4,08E-10	2,10E-07
Ndufa9	2833,787479	0,052780912	0,095272805	0,553997674	0,579580438	0,84530168
Lsm7	159,3735867	0,103668282	0,170046152	0,609647916	0,542095063	0,826611597
Ndufa3	1916,130802	0,033807367	0,098952856	0,341651254	0,732613361	0,912764338
Zfp408	143,3709924	-0,215247301	0,191899966	-1,121664094	0,262005289	0,622562722
Mks1	56,65448385	0,086551328	0,210936183	0,41031997	0,681571244	0,89166825
Siglecf	2,176955135	0,365451016	1,009884848	0,361873947	0,71744623	NA
Ptrh1	28,01612918	0,034742611	0,294291509	0,11805509	0,906024008	0,974075992
Nop16	799,7983427	-0,310703398	0,158633981	-1,958618165	0,050157521	0,269025436
Bcorl1	228,0468242	-0,129214299	0,125996914	-1,02553543	0,305110617	0,66433613
Zfp867	105,4012507	-0,012870829	0,171914509	-0,074867615	0,940320044	0,983128178
Lin7b	0,113662148	0,780932884	3,352475198	0,232942181	0,815806304	NA
Urah	6777,131134	0,053024162	0,08527052	0,621834632	0,534050611	0,821602143
Cd200r2	0,093303375	-0,517475177	3,352475198	-0,154356154	0,877328924	NA
Lrp2	29,94603242	-1,483953142	0,411709107	-3,604372889	0,000312908	0,01036908
Angptl7	7,275194001	0,023103058	0,714502923	0,032334449	0,974205337	0,993072906
Ap2a1	1031,844983	-0,267659626	0,076018913	-3,520960995	0,000429986	0,01312798
Adgrg1	91,87512323	0,634573561	0,278067896	2,282081358	0,022484537	0,168400998
Ulbp1	1,651721871	0,545145465	1,16741773	0,466966923	0,640523544	NA
Gtf2ird2	119,4256512	-0,447060297	0,166966682	-2,677541968	0,007416456	0,087576343
Vapa	1568,755056	0,097404732	0,069998046	1,391535012	0,16406325	0,502143222
Fnbp4	495,4008845	0,012681029	0,144840483	0,087551689	0,930233001	0,980359737
Ttr	198582,8205	0,105126854	0,136879742	0,768023466	0,442473244	0,769135977
Ddc	3864,078421	-0,237232612	0,172157215	-1,377999826	0,168203342	0,507127649
Il27ra	22,7020912	-0,013702097	0,371633144	-0,036869954	0,970588697	0,992007633
Crym	15,23794182	1,015506052	0,476867946	2,129533053	0,033210183	0,21273931
Pknox1	334,5700496	-0,102990293	0,109949715	-0,936703592	0,348911048	0,700915372
Bhmt	68087,55314	1,202636139	0,186760811	6,439445888	1,20E-10	6,41E-08
Sntb1	1083,881246	-0,099132328	0,193933082	-0,511167699	0,609233634	0,857753956
Rnf10	3597,534346	-0,031765855	0,093556318	-0,339537247	0,734205043	0,913675851
Syt3	142,4985842	-0,073775363	0,177995185	-0,414479545	0,678522944	0,890709117
Ahcy	19394,0195	0,345500761	0,065985127	5,236039981	1,64E-07	3,23E-05
Mpc1	2641,236411	0,382429839	0,105177577	3,636039653	0,000276862	0,009515076
Wbp2	3636,970095	0,011142806	0,067505022	0,165066323	0,86889178	0,961775826
Akap8l	703,0586859	0,146326971	0,152710966	0,958195565	0,337964153	0,693194655
Adamts1	236,7399837	-0,001314187	0,167567281	-0,00784274	0,993742463	0,99822248
Actg1	1248,714919	0,288052808	0,393289629	0,732419029	0,463912848	0,781663619
Mup5	2,042881777	-1,963300402	1,109381356	-1,769725435	0,076772891	NA
Mut	4616,927762	0,121793766	0,105630188	1,153020439	0,248901995	0,607886513
Mup4	2,48107094	-0,992382334	1,349698875	-0,735262029	0,462179928	NA
Rbmxl1	184,1297596	0,17550669	0,124637775	1,408134019	0,159091389	0,495312554
Ascl3	0,791235272	0,259903517	1,780812415	0,1459466	0,883963544	NA
Tacstd2	0,142634598	-0,517475177	3,352475198	-0,154356154	0,877328924	NA
Vps8	232,4476989	-0,00930667	0,156867878	-0,059328077	0,952690798	0,986424351
Kif23	16,92637812	-0,038971574	0,427673703	-0,09112455	0,927393626	0,978888597
Ufc1	1290,327695	-0,041433547	0,141960956	-0,291865792	0,770389239	0,927934035
Riok1	151,4155693	-0,009743774	0,15952779	-0,06107885	0,951296413	0,985972877
Kif24	19,00084758	0,436747882	0,350894245	1,244670975	0,213252719	0,56686047
Eef1e1	165,295508	-0,106224686	0,227285519	-0,467362314	0,64024068	0,871049783
Gins4	319,4552276	-0,190517273	0,111360395	-1,710817146	0,087114873	0,366110999
Ifitm3	8476,555174	0,074809688	0,142745443	0,524077592	0,600224573	0,854422974
Tipin	122,2942954	-0,039868899	0,145821715	-0,273408515	0,784539208	0,933246486
Galnt9	1,164883663	-0,295312754	1,457931315	-0,202556013	0,839482074	NA
Gm10400	0,093953095	-0,517475177	3,352475198	-0,154356154	0,877328924	NA
Kazn	20,09300275	0,311617698	0,422385627	0,737756396	0,460662495	0,780953195
Plekhg4	1,618488325	-1,384007969	1,260281967	-1,098173271	0,272128836	NA
Jakmip2	0,086476712	0,780932884	3,352475198	0,232942181	0,815806304	NA
Slc22a8	0,236587694	-1,308576888	3,335186821	-0,392354899	0,694796005	NA
Slc45a4	182,4939817	-0,17477334	0,134782692	-1,296704623	0,194732838	0,543831245

Supplementary Table S1: *Serpina1* KO vs. wildtype all DEGs

Sorcs1	0,086476712	0,780932884	3,352475198	0,232942181	0,815806304	NA
BC030499	4,214327621	-0,463294318	0,739261239	-0,626699053	0,530856525	NA
Npnt	10,69540661	0,642125862	0,639150879	1,004654586	0,315063203	0,673856248
Kdm6a	357,282749	0,061232272	0,168135028	0,364185101	0,715719792	0,906719207
Eef2	35063,0367	-0,053055019	0,062983531	-0,842363357	0,399584597	0,74061368
Micalcl	51,10101469	-0,976515243	0,234669995	-4,161227533	3,17E-05	0,001932349
Xrcc4	187,7988644	0,163844743	0,134169566	1,221176661	0,222019139	0,576792435
4930503L19Rik	39,83116076	0,032047181	0,278033026	0,115263937	0,908235922	0,9747592
Slc24a5	4,57480893	0,156787979	0,706932269	0,221786423	0,824480149	NA
Lpar4	1,030306634	0,347168711	1,370197836	0,25337123	0,799981358	NA
Sppl2b	345,2358194	-0,13697485	0,117274242	-1,167987509	0,242811803	0,601014405
Gimap5	15,65567143	-0,008248206	0,452181798	-0,018240907	0,985446669	0,995899882
Cramp1l	257,9959859	0,084669544	0,113583215	0,745440638	0,456005385	0,77799081
Hist1h3g	0,142634598	-0,517475177	3,352475198	-0,154356154	0,877328924	NA
Sssca1	230,494425	-0,018527789	0,169462558	-0,109332644	0,912938656	0,976126761
Ltb4r2	17,83773696	-0,382350386	0,416424802	-0,918173904	0,358527828	0,707901565
Apip	370,6434161	0,18512347	0,139262006	1,329317843	0,18374313	0,529866675
Zc3h8	78,76485119	-0,11660278	0,219696918	-0,530743812	0,595596321	0,853006203
Wdr46	554,017943	-0,103942196	0,133562596	-0,778228329	0,436434419	0,76575019
Tbl1x	434,6095777	-0,195394427	0,154316948	-1,266189033	0,205445413	0,558251976
Atad2	80,71457816	-1,209907702	0,181026405	-6,683597918	2,33E-11	1,56E-08
Eif2b4	764,2643878	-0,06789181	0,139914926	-0,485236363	0,627508691	0,865852703
Tmem262	2,145657906	-0,641077182	0,937058184	-0,684138075	0,493887981	NA
Cldn14	299,395571	-0,060671755	0,128981136	-0,470392466	0,638074646	0,870273408
Serpinb10	0,827963352	1,390700208	2,252840923	0,617309546	0,537030571	NA
Gm4788	7327,240511	-0,17187735	0,137307105	-1,251773167	0,21065253	0,563130109
Ston1	122,453533	-0,311217941	0,214270931	-1,452450595	0,146376357	0,476248605
Bcl7a	209,3464431	-0,056947111	0,161484984	-0,352646479	0,724353488	0,910252319
Raf1	2613,192797	0,039523115	0,111753315	0,353663913	0,72359077	0,910252319
AI464131	891,2912335	0,02153545	0,153270976	0,140505727	0,888260428	0,96836734
1700049E15Rik	0,299593163	1,742274127	3,264336744	0,533729901	0,593528419	NA
H2-Q1	257,0577163	1,959347238	0,539529828	3,631582787	0,000281688	0,009606863
H2-Ob	21,20187041	0,62067413	0,391545804	1,58518907	0,112923383	0,416117064
Prdm1	7,700027881	-0,137124949	0,65146695	-0,210486425	0,833288046	0,950519804
Aoc3	3,525712344	0,550542063	1,217943727	0,452025862	0,651250351	NA
Anxa5	2558,158574	0,317662613	0,129736113	2,44852884	0,014344095	0,130275952
D7Ertd143e	0,422085686	2,126130089	3,001494427	0,708357167	0,478723485	NA
Mirlet7f-1	0,494775497	0,400254628	2,155675782	0,185674781	0,852699807	NA
Usp13	0,901975082	-0,406661193	1,4842832	-0,273978169	0,784101399	NA
Mocs3	112,0492423	0,302438795	0,219571181	1,377406609	0,168386571	0,507353427
Nipal1	221,8612766	2,030825949	0,981879646	2,068304355	0,038611412	0,230668679
Mir3473d	0,294091955	0,617264955	2,767052958	0,223076668	0,823475837	NA
Asb4	4,633664499	0,592345765	0,709198841	0,83523228	0,403586938	NA
Dpysl5	1,277038382	-0,10415491	1,2609868	-0,082597939	0,93417124	NA
Car6	0,295994437	0,617282395	2,762021179	0,223489378	0,823154649	NA
Fads6	870,7760916	-0,004101531	0,147304584	-0,027843882	0,977786667	0,994354646
Setd1a	729,9015648	-0,281704657	0,127602219	-2,207678361	0,027266702	0,190567596
5730455P16Rik	382,0004521	-0,073793671	0,138200832	-0,533959668	0,593369439	0,851610953
Pak4	356,3565508	-0,142525077	0,106253297	-1,341370871	0,17980007	0,522553726
Gfod2	234,4966886	-0,003269323	0,193930844	-0,016858188	0,986549749	0,99608667
Cpm	70,88829926	0,255945375	0,237196398	1,079044106	0,280568066	0,641688878
Kansl1l	271,3208827	-0,151692114	0,154634549	-0,980971682	0,326606706	0,684457358
Dnase1l3	1117,532547	-0,204066104	0,382918268	-0,532923396	0,594086609	0,851817233
Dpp3	1803,314622	-0,114543599	0,087944144	-1,302458506	0,192759694	0,541552905
Actn1	629,9524168	-0,094103285	0,11403035	-0,825247704	0,409230975	0,746907036
Brms1	450,2902051	0,000898466	0,112144693	0,008011665	0,993607685	0,99822248
Lpxn	36,43239876	0,414617953	0,321800701	1,288430857	0,197596023	0,54738008
Yars	891,7156274	-0,107037687	0,102718433	-1,042049457	0,29738875	0,658144463
Carns1	48,06269818	0,193714658	0,267201677	0,724975458	0,468467087	0,78348591
Nrbp1	1261,515709	-0,031031693	0,076573812	-0,405252036	0,685292259	0,893124606
Rtp3	2502,773719	-0,382040852	0,123855697	-3,084564222	0,002038506	0,038665614
Zcchc17	382,9646349	-0,098042537	0,107189795	-0,914662977	0,360368586	0,710270917
Olfr920	2,328227015	1,13076204	0,95542444	1,183518019	0,236603909	NA
Olfr1030	0,45822262	-0,386912745	3,012574128	-0,128432606	0,897806629	NA
Cecr6	0,093953095	-0,517475177	3,352475198	-0,154356154	0,877328924	NA
Utrn	673,0316173	-0,213727101	0,185078386	-1,154792336	0,248175476	0,60717432

Gpat2	66,38461221	0,325469079	0,229504136	1,418140366	0,156149788	0,49142338
Lrp3	524,3587773	-0,556881886	0,150797494	-3,692912065	0,000221701	0,008076805
Trim30a	133,258471	0,160228542	0,21977947	0,729042355	0,465975754	0,781831056
Qdpr	12083,91049	0,329873109	0,110553917	2,983821104	0,002846732	0,047572443
Brd7	995,0930713	0,06495785	0,080947871	0,802465208	0,422283906	0,755522295
Synj2bp-cox16	2,380456207	0,324819772	1,14209004	0,284408199	0,776097576	NA
Borcs8	539,288471	-0,141414217	0,098889091	-1,430028489	0,152708843	0,486898287
Lin7c	767,3696757	-0,176349435	0,174992739	-1,00775287	0,313573117	0,672348265
Sp9	0,086476712	0,780932884	3,352475198	0,232942181	0,815806304	NA
Stum	4,753877352	-0,619971256	0,920569144	-0,673465171	0,5006514	NA
2310009B15Rik	75,72334294	-0,071244066	0,185771549	-0,383503645	0,701346368	0,90000956
Fam178a	345,340182	-0,078101147	0,131152454	-0,595498938	0,551510009	0,830638744
Fmn1	273,3588432	0,218824278	0,268593719	0,814703631	0,415241974	0,751551608
Adamtsl5	24,57569848	0,448108185	0,333510033	1,343612304	0,179073789	0,522031722
Sult2a7	46,53727766	2,373953574	0,457647396	5,187298328	2,13E-07	3,86E-05
Mreg	1422,903228	0,164011012	0,10483702	1,564437936	0,117714758	0,425792369
Ccdc102a	21,8450301	0,230827431	0,334764849	0,689521114	0,490495391	0,797288719
Snord8	0,861111196	3,163276619	2,579302514	1,22640776	0,220045267	NA
Phtf1	114,3021256	0,18515947	0,146539018	1,263550637	0,206391361	0,558880735
Trafd1	1286,755	0,287489187	0,101978355	2,819119666	0,004815556	0,066991853
Zfp236	407,8389387	-0,388809588	0,121474928	-3,200739413	0,001370754	0,029274146
Prom2	0,219943115	0,059593471	3,352475198	0,017775962	0,985817581	NA
Arhgef37	226,7484941	0,233929188	0,298064522	0,784827347	0,432554804	0,762604031
BC020402	33,57650163	0,133301063	0,241624221	0,551687504	0,581162473	0,845803031
Mir186	0,581242373	-1,859076064	1,934603536	-0,960959716	0,336572423	NA
Pet100	432,931216	-0,108918059	0,154123733	-0,706692323	0,479757701	0,791837124
Nmbr	0,696440053	-1,28325094	2,261019115	-0,56755422	0,5703377	NA
Sftpd	12,98827792	1,176982865	0,562244438	2,093365066	0,036316578	0,223328582
Gdnf	0,122496332	0,780932884	3,352475198	0,232942181	0,815806304	NA
Ccdc96	3,510608303	-1,165049663	1,144725489	-1,017754627	0,30879458	NA
Ifit1	294,837529	0,612244583	0,244799172	2,501007574	0,012384053	0,119799135
Ifi204	186,9153081	-0,070600199	0,234130675	-0,301541858	0,763001337	0,925557106
Nog	0,093953095	-0,517475177	3,352475198	-0,154356154	0,877328924	NA
Prkch	66,18419828	0,086337186	0,348225422	0,24793476	0,804184885	0,940361036
Pnmal2	2,491529416	0,133145538	0,996782102	0,13357537	0,893738361	NA
Ddias	18,63998656	-0,079943156	0,354441935	-0,225546551	0,821554116	0,946779723
Ar	51,50094671	0,443263873	0,400818024	1,105898054	0,268770647	0,629281048
Chst8	2,045787689	1,239012847	1,075200444	1,152355223	0,249175133	NA
Dnaic1	44,3187366	-1,051993719	1,017212133	-1,03419305	NA	NA
Vars2	606,3953433	-0,12910966	0,10522154	-1,227026896	0,219812482	0,574490867
Rnf122	17,05538509	0,614641982	0,435437506	1,41155039	0,158082389	0,493849511
Phlda1	1660,012518	-1,671837673	0,688016246	-2,429939241	0,015101354	0,13432477
Metrn	79,4523268	-0,522569245	0,265008262	-1,971897937	0,048621259	0,264127432
Tmem30a	4363,601974	-0,130246407	0,163460195	-0,796808097	0,425562492	0,758328303
Rab43	3217,082434	-0,136683376	0,143279438	-0,953963655	0,34010204	0,694383655
Smap2	1136,998726	0,174713483	0,119308332	1,464386262	0,14308844	0,470269006
Zfp873	72,91160533	-0,079446451	0,262001384	-0,303229127	0,761715253	0,92534041
Smyd1	120,3256128	-0,056625106	0,190570447	-0,29713477	0,76636362	0,926944289
Tbc1d8b	568,6636275	-0,408146836	0,187467531	-2,177160141	0,029468621	0,19767486
Ppfia1	843,4810116	-0,180821246	0,109269221	-1,654823243	0,097960358	0,389790319
Hpd	62109,45203	0,580728898	0,112402417	5,166516091	2,38E-07	4,14E-05
Kiss1	2,997944182	-0,247124226	1,063264322	-0,232420312	0,816211574	NA
Tmem203	156,6061443	0,185086877	0,147694006	1,253177989	0,210140939	0,562549902
Vtcn1	24,31936739	1,014391706	0,39053165	2,597463495	0,00939151	0,100691997
Mrpl50	1295,254415	-0,001141083	0,102422121	-0,011140985	0,991110964	0,99719344
Spx	2,170735542	-0,36945082	1,310860016	-0,2818385	0,778067349	NA
Cnnm4	122,7861036	-0,225113868	0,200028482	-1,125409075	0,260415727	0,621964694
Dpp7	600,6776619	-0,376118398	0,183596501	-2,048614195	0,040499856	0,236747953
Gigyf1	563,4331178	0,093689494	0,127330658	0,735796825	0,461854354	0,781516914
Ifitm5	3,150853367	-0,079520099	0,795668969	-0,099941184	0,92039102	NA
Slc15a1	0,286615571	1,706362568	3,314438116	0,514827101	0,606673845	NA
Gimap3	53,21433643	0,581755503	0,328658528	1,770091001	0,076711983	0,342478335
Arsk	210,6548985	0,304712285	0,137253323	2,220072185	0,026413868	0,187387171
Atg16l1	706,1299397	0,07973247	0,091133738	0,874895207	0,381630927	0,72637014
Peli2	505,4876148	0,028918808	0,15084182	0,191716117	0,84796458	0,953880403
1700109H08Rik	7,234824312	-0,18726176	0,523047977	-0,358020236	0,72032817	0,908788588

Kdm8	61,60968513	-0,120244266	0,206692415	-0,581754614	0,560731979	0,835721943
2510039O18Rik	590,8408303	-0,000878031	0,103330859	-0,008497275	0,993220237	0,998184334
Tstd3	700,8773075	0,553998408	0,200757488	2,759540449	0,005788272	0,075056653
Ticrr	3,400247021	-0,215404326	0,821917779	-0,262075273	0,79326341	NA
Ppp1r12c	613,4101825	-0,126908379	0,107202425	-1,183820038	0,236484308	0,593719947
Ddrgk1	3256,517792	0,00335787	0,084353179	0,039807267	0,968246783	0,991231429
Asah1	1170,802628	0,259337855	0,116689279	2,222465143	0,026251887	0,186582392
Naf1	92,56131249	0,166095875	0,184782151	0,898874018	0,368719769	0,716901482
Tmem136	16,23742323	-0,417811972	0,389557176	-1,07253055	0,2834818	0,644784493
Smco3	0,23615848	1,389394708	3,349408814	0,414817893	0,67827522	NA
Pigl	120,79789	0,159007726	0,163143285	0,974650756	0,329733528	0,6866113
Ccdc82	112,642468	-0,052467636	0,185267061	-0,283200024	0,77702351	0,930160038
Cep164	309,4787161	-0,316924098	0,114905914	-2,758118252	0,005813516	0,075238044
Ces1b	2976,085076	0,556023133	0,11393005	4,880390516	1,06E-06	0,000134805
Dmxl1	456,5025519	-0,247355089	0,154631198	-1,599645424	0,109677265	0,40991763
Cdc14b	897,9370909	0,248432724	0,155188179	1,600848245	0,109410535	0,409493125
Zfp239	4,172919265	0,365253283	0,7644278	0,477812663	0,632783548	NA
Mns1	6,466097623	0,538651617	0,632288023	0,85190862	0,394264812	0,736368577
Eif3a	4419,213285	-0,075378491	0,077189153	-0,976542538	0,328795679	0,686006046
Pkd2	336,2254041	-0,03201523	0,141300793	-0,226575018	0,82075422	0,94673539
Prkcq	29,62253052	-0,006749646	0,349934954	-0,019288288	0,984611127	0,995554769
Tulp2	13,80725602	1,51544161	0,607593357	2,494170801	0,012625178	0,121248489
Hspa4l	679,1342005	-0,509090517	0,176034123	-2,89199906	0,003827991	0,057613111
Cnih1	1396,109954	-0,052851514	0,110399738	-0,478728621	0,632131702	0,867306747
Tspyl4	131,4685267	-0,103712215	0,611430852	-0,169622148	0,865307303	0,961775826
Msl1	1016,258017	-0,111260311	0,080936186	-1,374667087	0,16923468	0,508724295
Cnih2	1,301652422	-0,300317418	1,506715892	-0,199319208	0,842013055	NA
Nkx2-6	1,227484136	-0,554733352	1,660085964	-0,334159413	0,738259269	NA
Enpp1	471,2598991	0,481225081	0,153355175	3,137977451	0,00170118	0,034511488
Tpp1	2302,533456	-0,222832641	0,0608552	-3,661686111	0,000250561	0,008861763
Mir6963	0,367488995	1,963806258	3,328796297	0,589944858	0,555227619	NA
Sox4	28,49346627	0,421795094	0,359461715	1,17340756	0,240632407	0,59816966
Dnaaf5	189,3208351	-0,112628185	0,143283264	-0,786052619	0,431836661	0,7622164
Filip1	12,91260631	-0,52193244	0,434346303	-1,201650472	0,229498978	0,585417255
Tln2	198,6796158	-0,050526669	0,185699804	-0,272087897	0,785554435	0,933683966
Prrg1	10,08246806	0,490497014	0,844865142	0,580562494	0,561535357	0,836472635
Nat10	630,1392572	-0,13255444	0,094679085	-1,400039306	0,161501548	0,497950692
Sec16a	2851,263284	-0,27841918	0,11354109	-2,452144668	0,014200756	0,129557379
Oplah	2625,563062	-0,201486619	0,108894786	-1,850287101	0,064272181	0,308676071
Lysmd1	45,74610161	-0,137393778	0,257083825	-0,534431827	0,593042804	0,851477265
Edc4	443,1797624	0,078713448	0,118674903	0,663269535	0,507157944	0,806685849
Itpkc	159,855047	-0,097165226	0,144072725	-0,674417911	0,500045659	0,803112736
Ckap2l	12,80699194	-1,251779587	0,569856147	-2,196658919	0,028044815	0,192787383
Cmbl	3936,351867	0,474828676	0,14066595	3,375576495	0,000736612	0,019347284
Pik3r3	52,21310061	-0,449679157	0,283458001	-1,586404882	0,112647494	0,415902884
Gab3	5,185942806	0,339880676	0,680779787	0,499252009	0,61760186	NA
Pof1b	2,667762569	0,327912666	0,906731598	0,361642483	0,717619213	NA
Bend5	1,806570836	1,181278901	1,121318054	1,053473541	0,292124015	NA
Med31	73,37890155	-0,186903884	0,175335613	-1,065977873	0,286433644	0,648131311
Sowahc	140,6360757	-0,261788498	0,170973458	-1,531164548	0,12572873	0,441404253
Amdhd2	388,3996684	-0,184595053	0,13308671	-1,387028446	0,165433089	0,503683664
Cpb1	0,087021394	-0,517475177	3,352475198	-0,154356154	0,877328924	NA
Acy3	2097,254149	-0,091437837	0,154395685	-0,592230522	0,553696233	0,831920164
Ifi203	215,1306471	-0,152587377	0,199835673	-0,763564258	0,445126955	0,770855289
Pld2	191,947739	0,139224123	0,131168085	1,061417663	0,288500133	0,649625922
Mir3471-1	0,086476712	0,780932884	3,352475198	0,232942181	0,815806304	NA
Zfp777	205,3407957	0,255530614	0,124076425	2,05946144	0,039450055	0,233292042
Cap1	1981,101807	0,027139327	0,076472765	0,354888791	0,722672906	0,909652018
Hadh	7915,979278	0,275335333	0,119287848	2,308159101	0,020990289	0,161926817
Gna14	77,5788277	0,822276736	0,784826676	1,047717618	0,294768728	0,655777576
Ogn	35,28938047	0,36473287	0,349550061	1,043435294	0,296746734	0,657874966
Ocln	500,7368818	0,225718894	0,206055686	1,095426668	0,273329756	0,634872238
Oaz1	5,502650066	0,655125937	0,628097103	1,043032891	0,29693306	0,657888312
Lgr5	144,2500448	-0,068254007	0,229349845	-0,297597788	0,766010163	0,926936085
Nxn	44,2304004	-0,234427891	0,267474624	-0,876449088	0,380785942	0,725866642
Slfn3	1,525979642	-0,037250914	1,366635598	-0,027257386	0,978254446	NA

Suclg2	6113,755289	0,230348718	0,089053354	2,586637207	0,009691758	0,102588366
Slfn2	92,29600689	0,403714902	0,217695427	1,854494174	0,063668493	0,306733002
Slc23a1	1685,90774	0,440090573	0,135816819	3,240324549	0,001193937	0,027008032
Slfn1	13,55596105	0,504110031	0,477925565	1,054787749	0,291522409	0,653216736
Slc22a5	308,8332914	-0,126296568	0,195362792	-0,646471964	0,517973748	0,813532782
Slc7a7	93,44919309	-0,380014619	0,215339605	-1,764722377	0,077610436	0,345167637
Vrk1	146,8707054	-0,011262661	0,158083737	-0,071244904	0,943202844	0,983517575
Slc7a5	79,97067376	0,067905873	0,245494208	0,27660886	0,782080461	0,932290118
Igip	115,291701	0,062287496	0,293915026	0,211923484	0,832166729	0,9499007
Zfp3	46,20560288	0,144034621	0,215734987	0,66764609	0,504359529	0,804997854
Usp46	266,535282	-0,414199642	0,160897748	-2,574303544	0,010044211	0,104989098
Vangl1	199,8703447	-0,162003668	0,130319149	-1,243130189	0,213819863	0,567174156
Zfp280b	198,7191779	-0,367933639	0,117069678	-3,142860262	0,001673057	0,034044289
Ccdc190	0,852232097	2,26565899	1,91936482	1,180421234	0,237832721	NA
Gpr55	1,85928376	1,124142825	1,075654807	1,045077676	0,295987071	NA
Mir7063	0,394090941	-0,073152886	2,256596235	-0,032417357	0,974139221	NA
Cd79a	36,25709078	0,534844916	0,368627225	1,450909971	0,146804931	0,476487281
E4f1	202,1051036	0,020618484	0,152568661	0,13514233	0,892499343	0,969104575
Cd53	133,0651302	0,183542976	0,207254509	0,885592199	0,375837317	0,721655743
Cd48	60,19418036	0,196629469	0,276896706	0,710118484	0,477630665	0,790092068
Cd5	5,930601056	0,438661592	0,759913359	0,577252113	0,563769158	0,837355044
Cd3e	11,12833229	0,551173991	0,53684606	1,026689088	0,304566889	0,664033955
Irf6	1324,140893	-0,223055042	0,10727321	-2,079317298	0,037588196	0,227177484
Irf3	1595,43865	0,025710763	0,089005645	0,288866655	0,772683422	0,928372847
Avpr1a	1207,586295	-0,041797057	0,158974227	-0,262917192	0,792614405	0,936597818
Spag5	14,215691	-1,340321384	0,555770926	-2,411643578	0,015880798	0,138222906
Tnfsf14	6,018230583	-0,612020527	0,696762077	-0,878378068	0,379738583	0,725269233
Rps28	2428,152025	0,095253834	0,088822147	1,072410847	0,283535538	0,644784493
Syt11	97,18155029	-0,303460535	0,193364084	-1,569373837	0,116560857	0,423336618
Dgka	203,50854	0,008034195	0,171782351	0,046769616	0,962696845	0,988622522
Gosr1	903,1777937	0,029779683	0,104239581	0,285684982	0,775119408	0,929461958
Rbm38	71,68004122	-0,096262879	0,189444154	-0,508133278	0,611359879	0,858645358
Pdlim3	4,712548504	0,478251491	0,860068718	0,556061953	0,578168504	NA
Becn1	1299,2986	-0,193961799	0,093750212	-2,068921175	0,038553484	0,23061366
Il17rb	310,7306626	-0,089557079	0,23455912	-0,38181026	0,702602108	0,900494767
2010107E04Rik	1801,700712	0,106261668	0,134464156	0,79026018	0,429375837	0,760857915
Faim	184,4224292	0,097503052	0,137906076	0,707025063	0,479550903	0,791837124
Mir7673	1,025181711	2,558898054	2,142708369	1,194235338	0,232385923	NA
Sec23ip	566,909235	-0,229492825	0,146089919	-1,570901172	0,116205606	0,422672193
Vkorc1l1	1004,183466	-0,22354876	0,117076291	-1,909428104	0,056206888	0,286695875
Tm4sf1	239,2566209	0,436331523	0,18667139	2,337431155	0,019416777	0,155304678
Lyl1	19,5377483	-0,425478603	0,392955388	-1,082765666	0,278912449	0,63936566
Cd180	53,99194505	0,24737475	0,273870546	0,903254307	0,366390941	0,714711689
Epcam	46,72229128	0,710764793	0,360851914	1,96968553	0,048874422	0,264749107
Ly6f	2,108924211	1,808427888	1,183376716	1,528192893	0,126464653	NA
Blnk	65,10135924	0,032957196	0,304243986	0,10832489	0,913737979	0,976245748
Ccdc89	3,799527135	1,02088469	0,838547129	1,217444619	0,223435095	NA
Ppp2r5e	972,7076939	-0,37942744	0,130990203	-2,896609295	0,003772192	0,057372511
Prdx5	5899,39006	-0,042513064	0,12932498	-0,328730488	0,742359408	0,916955577
Adamts17	1,154291818	0,54640728	1,553684038	0,351684942	0,725074555	NA
Aplp2	8833,360181	-0,057658886	0,070316469	-0,819991203	0,412221122	0,749265881
Lats1	679,022824	-0,018425726	0,123590751	-0,149086609	0,881485292	0,965734294
Birc5	28,46854514	-1,030704303	0,342471417	-3,009606795	0,002615861	0,044950444
Myo5a	14,66317874	0,257133702	0,412106015	0,623950374	0,532660177	0,820654481
Cfp	553,7491436	0,125815508	0,207386562	0,606671459	0,544068967	0,827477215
Hivep2	185,7336694	-0,170375228	0,173288369	-0,983189056	0,3255144	0,682862556
Dlx1	0,086476712	0,780932884	3,352475198	0,232942181	0,815806304	NA
Slc35f6	441,494567	-0,12897664	0,109329515	-1,17970559	0,23811733	0,595289658
Ap4e1	157,5815052	0,001383339	0,150704195	0,009179169	0,992676186	0,997976232
Ttc22	0,929878434	2,469415189	1,856846189	1,329897545	0,18355203	NA
4933430I17Rik	2,227651997	-0,382202096	1,192827028	-0,320417032	0,748652216	NA
Bloc1s3	140,1312013	-0,129081327	0,147480637	-0,875242536	0,381441953	0,72637014
D030056L22Rik	138,6388359	-0,061081605	0,145081406	-0,421016078	0,673743342	0,888626355
Crb3	383,403568	0,139269724	0,130708145	1,065501498	0,286649048	0,648131311
Birc6	1429,165391	-0,133158142	0,115810086	-1,149797461	0,250227301	0,609210778
Bmp4	103,3332754	-0,997612177	0,268185565	-3,719857836	0,000199335	0,007483408

Ap3d1	1520,880516	-0,207442221	0,072997906	-2,841755788	0,004486584	0,063741921
Ap2a2	2514,493476	-0,259216667	0,087889971	-2,949331581	0,003184621	0,051419321
C1qc	1436,316958	0,133882334	0,198074602	0,675918735	0,499092242	0,802710699
Dock1	409,0248021	-0,219645889	0,13537569	-1,622491371	0,104698179	0,402447734
Hmgn1	1324,189326	-0,061267328	0,150933745	-0,405922002	0,684799911	0,892717658
S1pr1	2363,295994	-0,182698308	0,155264513	-1,176690696	0,239318983	0,59669069
Alx4	0,113662148	0,780932884	3,352475198	0,232942181	0,815806304	NA
Ctnnb1	4895,503867	-0,074551963	0,08213044	-0,907726326	0,364022831	0,712849163
Casp7	573,7175347	0,251863952	0,106070296	2,374500322	0,017572721	0,146719059
L1td1	0,209517726	0,059593471	3,352475198	0,017775962	0,985817581	NA
Fam159a	0,704682875	-0,484853545	2,298313603	-0,210960569	0,83291804	NA
Gm5640	0,800409617	2,152837832	2,286712035	0,941455592	0,346471437	NA
Slc2a6	6,577303678	-0,152867089	0,667974383	-0,228851724	0,818984163	0,945934875
4930579G24Rik	22,8732505	-0,716453647	0,357877653	-2,001951337	0,045289966	0,251969018
Vmp1	2791,147215	-0,347831104	0,139279106	-2,497367443	0,012511923	0,120599786
Entpd7	66,5238274	-0,973573822	0,23155243	-4,204550233	2,62E-05	0,001682953
Cox4i2	2,959005037	-0,241496626	0,907464161	-0,266122494	0,790144872	NA
Trim8	678,2709769	0,089567717	0,17157281	0,522039111	0,601643099	0,854462419
Adam8	8,448468079	0,084003558	0,598864138	0,140271479	0,888445498	0,96836734
Ldhb	42,83944572	0,302845349	0,246978065	1,226203426	0,220122132	0,574490867
Ctsa	4126,677942	0,121386603	0,093718523	1,295225307	0,195242517	0,544259529
Sebox	25,33293804	-1,049005485	0,418516091	-2,506487821	0,012193726	0,118300378
Cyp4v3	11587,45122	-0,745171439	0,147119635	-5,065071279	4,08E-07	6,50E-05
Cytip	18,16349533	0,623937134	0,391542724	1,593535254	0,111040166	0,412474571
Zdhhc7	390,684515	0,008681728	0,106106786	0,081820664	0,934789323	0,981943778
Cd300c2	97,13412907	0,43426477	0,226414895	1,918004421	0,055110451	0,284116184
Plekha1	283,3527182	-0,229449753	0,16549292	-1,386462653	0,165605677	0,503882503
Dohh	449,6506184	-0,080472342	0,12337605	-0,652252539	0,514238272	0,811764185
Fam208b	282,1771497	0,049138262	0,158525378	0,309970952	0,756583046	0,923140802
Gpr37l1	0,383881453	2,001586489	2,57081031	0,778581944	0,436226019	NA
Il17rd	10,89929279	0,376495675	0,537757407	0,700121783	0,483851253	0,794055997
Arhgef18	668,4719098	-0,019791017	0,094501738	-0,2094249	0,834116556	0,95084595
Dtymk	361,1017411	-0,162397931	0,132632271	-1,224422452	0,220792904	0,575059484
Gm11545	7,475066907	-0,259395132	0,572630154	-0,452988949	0,650556699	0,876831587
Snord99	2,71577835	0,439227993	0,928314463	0,473145696	0,636109237	NA
Gnat2	50,07962419	-0,64822399	0,314642651	-2,060191102	0,039380275	0,233056618
Gnaq	385,4594361	-0,107074696	0,114389328	-0,936054946	0,349244898	0,700954068
Hsd3b5	4313,884765	-0,987173352	0,929328668	-1,062243516	0,288125149	0,649500693
Sdc2	4748,45384	0,111889195	0,105113698	1,064458745	0,287120934	0,648651236
Hspe1	3305,860746	-0,131162726	0,132944217	-0,986599707	0,323838914	0,680937786
Mir6966	0,273083462	-0,658048199	3,340448562	-0,196993963	0,843832262	NA
Pdia3	17286,92423	-0,457561396	0,153129587	-2,988066556	0,002807484	0,047271102
Rab15	0,502251881	-0,528787427	2,421170007	-0,21840161	0,827116211	NA
Mycl	166,7953164	0,676087779	0,345200113	1,958538699	0,050166835	0,269025436
Mir7067	0,122496332	0,780932884	3,352475198	0,232942181	0,815806304	NA
Marf1	1555,269659	-0,309669672	0,122133758	-2,535496148	0,011228816	0,11338221
A1cf	2681,684142	-0,173958591	0,183383447	-0,94860574	0,342821171	0,695589013
Cep76	106,4522265	-0,294453836	0,191213851	-1,539918969	0,123580106	0,436497342
Slc27a6	8,626958253	-0,224138027	0,519652431	-0,431322965	0,666233554	0,885111436
Arfgap2	3470,579363	-0,02351361	0,097585534	-0,240953851	0,809590884	0,94241441
Sdc3	1268,877082	0,057693464	0,222817394	0,25892711	0,795691481	0,937598879
Six5	104,5290663	-0,230641344	0,1759581	-1,310774235	0,18993405	0,5379721
Six4	0,087021394	-0,517475177	3,352475198	-0,154356154	0,877328924	NA
Tm4sf19	0,313801008	1,795331527	3,226882632	0,55636716	0,577959884	NA
Dusp23	331,9854891	0,138720891	0,120629424	1,149975576	0,250153931	0,609210778
Pir	512,1073039	0,269743369	0,212442314	1,269725246	0,204182517	0,557198625
Cr2	8,897607419	-0,734514066	0,492140062	-1,492489889	0,135570766	0,458151054
H60b	0,99491542	2,433215313	1,675159696	1,452527373	0,146355024	NA
Snord35b	0,646825739	1,849373671	2,28718529	0,808580608	0,418756423	NA
Arrdc3	1395,389878	0,700109647	0,199949236	3,501436972	0,000462756	0,013886723
Snord35a	1,31532196	-0,234545038	1,373468698	-0,17076839	0,864405885	NA
Snord34	2,045191627	1,331144512	1,147238541	1,160303166	0,245925396	NA
Snord33	0,542912318	1,42428031	2,343424905	0,607777235	0,54333523	NA
Snord32a	1,292499642	0,560498717	1,358951225	0,412449473	0,680010008	NA
Thnsl1	104,006451	0,020703602	0,190314719	0,108786133	0,913372123	0,976232707
Arhgef15	139,9606698	-0,116882967	0,225511157	-0,518302368	0,604247321	0,855575199

Supplementary Table S1: *Serpina1* KO vs. wildtype all DEGs

Scarna6	0,944679884	-0,55654895	1,864365898	-0,298519164	0,765306952	NA
Ccdc189	29,77207085	-0,22162105	0,301945099	-0,733977967	0,462962167	0,781516914
Tmem191c	14,4842927	-0,257707823	0,429410694	-0,600143001	0,548410937	0,828815941
Fras1	97,50700267	0,100837436	0,223744528	0,450681127	0,652219386	0,877662367
Fdx1l	57,28238083	-0,112688261	0,257692303	-0,437297738	0,661895436	0,882945528
Cct2	2933,915246	-0,115728037	0,08943119	-1,29404559	0,195649674	0,544698145
Thtpa	848,8863527	0,044635555	0,111619606	0,399889914	0,689237601	0,894327388
Lrrc25	69,80229323	0,205668047	0,242440255	0,848324659	0,396257188	0,737761616
Fmr1	399,1649104	0,097112326	0,191300353	0,507643214	0,611703579	0,858821257
Itgam	26,37474597	0,180986702	0,410962378	0,440397252	0,659649415	0,882238198
Mtag2	0,147722973	0,780932884	3,352475198	0,232942181	0,815806304	NA
Pprc1	300,5221016	0,434017525	0,305070813	1,422677972	0,154829529	0,489743509
Ermp1	725,6823642	-0,432336638	0,09959239	-4,341060971	1,42E-05	0,001108582
Irs2	266,8453556	-0,647683957	0,468699931	-1,38187338	0,167010583	0,505951617
Kcna3	1,05980519	-0,918849508	1,499905567	-0,612604906	0,540137614	NA
Fbp2	1,977264555	-0,936347211	1,103212361	-0,84874612	0,396022577	NA
Ltb	21,77101878	0,73540274	0,347501688	2,116256599	0,03432299	0,216695243
Pip4k2a	107,2954995	-0,121719564	0,20887689	-0,582733514	0,560072708	0,835463907
Fpr2	28,90063252	0,33249732	0,40659328	0,817763932	0,413491997	0,75016617
Cd28	5,673170324	1,444279156	0,65711435	2,197911454	0,027955416	0,192647404
Il2	0,093953095	-0,517475177	3,352475198	-0,154356154	0,877328924	NA
Gch1	3289,25857	0,434724744	0,134823484	3,224399284	0,001262373	0,028033175
Prpf4b	865,4826233	-0,286626463	0,097619727	-2,936153104	0,003323103	0,052888761
Scly	2713,357044	0,065991111	0,098867294	0,667471604	0,504470942	0,804997854
Mmp13	5,83725166	-0,001104839	0,666708772	-0,001657153	0,998677784	0,999803624
Sp100	240,9816593	0,066137561	0,245700269	0,26917985	0,787791286	0,934597719
Nup50	747,8191164	0,044129744	0,09535585	0,462790104	0,643514825	0,873795284
Eif3i	1931,395817	-0,084585449	0,09159232	-0,923499362	0,355747033	0,705740033
Cd244	4,020377717	1,515261878	0,832853946	1,819360869	0,068856393	NA
Auh	711,4260072	0,093850127	0,113090065	0,829870658	0,406611916	0,745871812
Hmgn5	1347,058664	-0,111021798	0,080268071	-1,38313773	0,166622639	0,505463594
Bmpr1a	760,1155968	0,035493419	0,101223487	0,350644103	0,725855366	0,910393262
Hoxa7	0,12663974	0,780932884	3,352475198	0,232942181	0,815806304	NA
Ngp	2,807588621	-3,253345269	1,408006723	-2,310603504	0,020854766	NA
Dpy19l4	99,59673112	-0,082300764	0,197811346	-0,416056843	0,677368409	0,890086324
Skap1	6,519738041	0,173072138	0,785769899	0,22025804	0,825670197	0,947537943
Dennd1b	246,5047093	-0,294648753	0,210390707	-1,400483686	0,161368525	0,497950692
Trpm4	17,74598403	0,286409889	0,351382561	0,815094205	0,415018389	0,751551608
Clic5	63,3682919	-0,537429255	0,387878087	-1,385562303	0,165880596	0,504089882
Vps52	595,4549352	-0,180653751	0,094086342	-1,920084759	0,054847194	0,283546844
Zfp523	507,5426474	-0,2622348	0,096072473	-2,729551882	0,006342047	0,079462816
Twistnb	258,2025959	0,172842533	0,138299341	1,249771197	0,211383141	0,564073259
Mcm10	999,4928717	-0,79051366	0,405784992	-1,948109655	0,051401844	0,273238289
Tmem44	41,2459511	0,016585317	0,350352282	0,047338973	0,962243067	0,988526411
Rnd1	355,0229856	-0,007102739	0,208406704	-0,034081143	0,972812445	0,992650147
Gramd4	280,7940097	0,165729088	0,130876971	1,266296785	0,205406847	0,558251976
Kctd3	307,4777991	-0,054144409	0,153624387	-0,352446703	0,724503282	0,910252319
Atg14	325,4901297	-0,05500498	0,17259153	-0,318700343	0,749953755	0,920166276
Mpped1	0,652141962	2,760640272	2,275437626	1,21323487	0,225040036	NA
Plekhh2	29,52606431	0,145453719	0,437889202	0,332170144	0,739760787	0,916067243
Mkx	17,57434035	0,113070606	0,575773445	0,196380377	0,844312454	0,953068489
Rgs12	85,14634416	0,483116413	0,276334773	1,748301188	0,080411888	0,350731846
Il34	40,75264827	0,000711373	0,263613412	0,002698545	0,997846875	0,999641429
Gm8787	0,381922657	1,99626423	3,069452389	0,650364944	0,515456516	NA
Gm7173	0,087021394	-0,517475177	3,352475198	-0,154356154	0,877328924	NA
Armcx4	33,64626762	0,163297059	0,367404756	0,44446093	0,65670937	0,879978708
Slc12a4	366,3711483	-0,435830648	0,173622972	-2,510213038	0,012065834	0,117400394
Ddit4l	52,86760349	0,367315501	0,348710968	1,053352304	0,292179556	0,653967601
Usp47	2379,287014	-0,126437306	0,086292832	-1,465212153	0,142863045	0,470144068
Cd74	2230,642826	0,501797125	0,213387052	2,351581882	0,018693775	0,152034903
Zfp105	15,11283943	-0,745384861	0,366736299	-2,032481819	0,042104903	0,242468387
Lrp11	62,81342328	-0,278353121	0,31880831	-0,873104973	0,382605863	0,726983768
Ints6l	66,89263282	0,118760364	0,236069959	0,503072752	0,614913123	0,859936455
Cntnap5b	0,086476712	0,780932884	3,352475198	0,232942181	0,815806304	NA
Elmo3	603,8666917	-0,081167324	0,107674898	-0,753818442	0,450958248	0,774175112
Nfrkb	266,711632	-0,08081653	0,143335429	-0,563828012	0,572871186	0,842079702

Supplementary Table S1: *Serpina1* KO vs. wildtype all DEGs

Heatr3	567,6588186	-0,042376836	0,130126394	-0,325659034	0,744682342	0,918333939
Zfp646	377,2745784	0,041270565	0,129489436	0,318717618	0,749940654	0,920166276
Adprhl1	0,209517726	0,059593471	3,352475198	0,017775962	0,985817581	NA
Fbxl19	113,2417732	-0,354823167	0,172112506	-2,061576904	0,039248035	0,232686021
Kctd13	70,43240059	0,214139883	0,181719866	1,178406562	0,238634563	0,595935251
Ttll4	190,0571844	-0,457447302	0,174776682	-2,617324552	0,008862203	0,097781395
BC024139	142,9062612	0,422392182	0,256343829	1,647756392	0,099402671	0,392688228
Bcl2l15	1,38994109	-3,273177402	1,425743603	-2,295768605	0,021689115	NA
Mir3060	2,014071134	0,465974309	1,043978029	0,446344939	0,6553481	NA
Gfra1	3026,33191	-0,011987698	0,078317877	-0,153064648	0,878347294	0,965046146
Gfi1	2,076700433	-0,449405475	1,33778028	-0,335933697	0,736920866	NA
Lamp1	17190,77277	-0,081562931	0,059561185	-1,369397401	0,170875086	0,510601034
Mir3101	4,056980999	0,569297835	0,728838058	0,781103332	0,434741723	NA
Peg12	2,818340659	-0,488952477	0,847262985	-0,577096469	0,563874289	NA
E030018B13Rik	11,05589404	0,722060007	0,642166764	1,124411987	0,260838289	0,621964694
Zcwpw1	117,5444327	-0,270464439	0,183030809	-1,477698974	0,139488369	0,464463264
Pan3	295,8829135	0,090265401	0,121177971	0,744899428	0,456332521	0,778052477
Mon1a	415,0522025	-0,081668961	0,133653607	-0,611049435	0,541166854	0,826407188
1700112E06Rik	12,52665096	0,912555611	0,527605435	1,729617533	0,083698631	0,358069441
Acap2	390,1609172	-0,041154503	0,119016323	-0,345788729	0,729501497	0,911594858
Bloc1s2	228,4731306	0,195383395	0,134824081	1,449172835	0,147289322	0,47747598
Adamts15	19,99057104	-0,792048007	0,330344471	-2,397642696	0,016500952	0,142154471
Rexo1	677,3863721	-0,052447554	0,11509205	-0,455700925	0,648605057	0,876446263
Slmo2	839,7131443	-0,163694864	0,120560297	-1,357784185	0,174532175	0,517225036
Nudt8	388,0491012	-0,134414394	0,136785046	-0,982668778	0,325770482	0,68327982
Ndufc1	627,1710336	0,161327471	0,153423906	1,05151456	0,293022326	0,654211001
4931406P16Rik	269,9190254	0,049847392	0,128287833	0,388559001	0,697602401	0,898309237
Mir6972	0,749841991	0,169129588	1,752504209	0,096507379	0,923117614	NA
Gng3	0,240301888	1,389394708	3,349408814	0,414817893	0,67827522	NA
Mapkbp1	163,630761	0,003915922	0,201499255	0,019433928	0,984494945	0,995554769
Clpx	6597,152383	0,259699836	0,134951455	1,92439449	0,054305155	0,282163083
Cfdp1	1229,350865	-0,084432376	0,083070481	-1,016394443	0,309441591	0,668431835
Rhoa	3735,164334	-0,023708164	0,069632791	-0,340474127	0,733499504	0,913475069
Psmd13	1478,681332	0,037915669	0,083680919	0,453098141	0,650478074	0,876831587
Plcl2	406,0786111	0,112385385	0,107498135	1,045463582	0,295808764	0,656921489
Wbp2nl	0,187906191	-1,241948083	3,339949325	-0,371846385	0,710007226	NA
Zfp820	71,53850874	0,523242975	0,290262558	1,802654046	0,071442563	0,32776789
Btbd18	0,142634598	-0,517475177	3,352475198	-0,154356154	0,877328924	NA
Nop14	507,5001352	-0,076186513	0,126142847	-0,60397014	0,545863495	0,827961092
Iqce	150,16245	-0,001963135	0,173609118	-0,011307789	0,990977882	0,99719344
Ppp2r2b	5,517701304	0,831870568	0,731090037	1,137849685	0,255183254	0,615600706
Zfp493	41,57808643	-0,134183894	0,259677045	-0,516733751	0,605342032	0,856421285
Rpap1	284,8985255	-0,034547173	0,133751733	-0,258293271	0,796180579	0,937785232
Apba1	5,621296474	-0,892331079	0,677154341	-1,317766165	0,187581938	0,53391163
6030419C18Rik	2,145349145	0,643949643	1,148482927	0,560695878	0,575004878	NA
Lrtm1	313,4031115	2,725192746	0,762067596	3,576051208	0,000348823	0,011264298
Mettl4	120,1933229	-0,030340683	0,20972924	-0,144665966	0,884974611	0,967327774
Slc17a4	1096,676938	0,656074445	0,227661607	2,881796601	0,003954149	0,058659427
G6pc3	144,5746823	0,301485099	0,136827916	2,203388816	0,027567356	0,191254789
Rapgef5	33,02639416	-0,196418872	0,385853101	-0,509050909	0,610716541	0,858645358
Iqgap3	7,978558207	-2,378881878	0,623574495	-3,814912087	0,000136231	0,005691493
Zcchc24	2454,226477	-0,233394082	0,122877866	-1,899398887	0,05751205	0,289924055
Mkrn2os	591,4339759	0,332019274	0,095952818	3,460234748	0,000539705	0,015417333
Dip2c	364,6371531	-0,217588832	0,098532931	-2,20828539	0,027224385	0,190567596
Galns	220,1935549	0,022036279	0,133273617	0,165346146	0,86867154	0,961775826
Iqgap1	266,6572792	0,154465191	0,179005227	0,86290883	0,388187595	0,731971786
Pcnx	707,7495635	0,028814349	0,140863958	0,204554445	0,837920251	0,951141594
Cpsf3	1024,537275	-0,016732992	0,082671564	-0,202403231	0,839601503	0,951377482
Fmo2	239,3143957	1,112446515	0,313002717	3,554111367	0,000379259	0,012087778
Nprl2	397,3221505	-0,109749534	0,12823498	-0,855847087	0,392082366	0,734686211
Scamp5	369,3888347	-0,286247601	0,129973615	-2,20235162	0,027640481	0,191563289
Trpm5	1,776118192	-1,234748403	1,257226062	-0,982121227	0,326040129	NA
Hhip	46,79800471	0,376686073	0,300337916	1,25420752	0,209766587	0,562335974
Hebp2	17,65961725	0,540194904	0,399015454	1,353819503	0,17579397	0,518576695
Sap30bp	410,6546169	0,006256243	0,100304011	0,062372815	0,950265944	0,985972877
Bag5	319,251591	0,253290119	0,151275066	1,674367929	0,094058312	0,380819373

Supplementary Table S1: *Serpina1* KO vs. wildtype all DEGs

Hfe2	2780,774091	0,281798866	0,091859788	3,067706469	0,002157084	0,040104608
Gon4l	337,4982214	-0,267229109	0,11038628	-2,420854378	0,015484079	0,136457909
Ppp1r1a	0,227324296	1,389394708	3,349408814	0,414817893	0,67827522	NA
Hgfac	5927,949519	0,097720242	0,085980619	1,136538012	0,255731466	0,616235395
Cactin	361,9002423	0,010847432	0,122438297	0,088595089	0,929403711	0,979997334
Wdr5b	48,98887758	-0,059419915	0,25361555	-0,234291292	0,814758846	0,944076707
Mettl7b	20303,3817	-0,064627357	0,105839228	-0,610618184	0,541452382	0,826407188
4921507P07Rik	6,116059553	0,254763889	0,598947398	0,425352694	0,670579586	0,887361539
Cfap43	0,515937552	0,455134974	1,938901514	0,234738573	0,814411647	NA
Pgrmc2	1834,148485	0,213266085	0,135308611	1,576145699	0,114992229	0,419921091
Cep192	85,27578355	0,192940181	0,200960532	0,960089918	0,337009962	0,69270916
Hps6	88,86084728	-0,104407238	0,212176922	-0,492076315	0,622665395	0,863349271
Naa40	259,2656994	-0,091086008	0,117748655	-0,773563043	0,439189234	0,766717272
Psmd1	3297,41209	0,05076025	0,089988604	0,564074204	0,572703633	0,841925981
Rnf168	331,5879774	-0,096348976	0,148278792	-0,649782578	0,515832674	0,813164632
Poc1a	53,22847634	-0,108838003	0,283176548	-0,38434681	0,700721418	0,899663133
6330409D20Rik	0,213661134	0,059593471	3,352475198	0,017775962	0,985817581	NA
Ptgs2	0,373092283	2,037329247	3,059752286	0,665847774	0,505508405	NA
Gfpt2	9,329287774	0,215873784	0,513225436	0,420621756	0,674031304	0,888627551
Hmha1	227,6624645	0,068175821	0,223714464	0,304744806	0,760560522	0,925072539
Medag	5,387729365	0,71202803	0,779498374	0,913443895	0,361009129	0,710581841
Gpr137c	2,488352816	-0,324293178	1,015694306	-0,319282264	0,74951248	NA
Pvr	133,5605936	0,014702459	0,154748206	0,095008914	0,924307747	0,977951927
Nup205	315,499435	-0,169285814	0,136783541	-1,237618298	0,215857626	0,569610871
Os9	7468,632711	-0,15953857	0,055648065	-2,866920332	0,004144873	0,060626709
Gm21948	0,710223645	-0,646017223	1,753506854	-0,368414427	0,712564241	NA
Raet1a	8,70747763	1,145997133	0,664172521	1,725450988	0,084446229	0,359771077
Plekho2	133,6188179	0,110097872	0,255740969	0,430505417	0,666828027	0,885394917
Cpne5	0,180974489	-1,204797453	3,342691436	-0,360427361	0,718527568	NA
C1ql3	2,799924302	0,455301073	1,236212466	0,368303254	0,712647127	NA
Svil	1172,998936	-0,162827774	0,125165083	-1,300904138	0,19329127	0,541742344
Acat3	5772,38535	0,129860577	0,082541183	1,573282237	0,11565348	0,421300104
Slc25a1	6121,797127	0,096774808	0,079425094	1,218441216	0,223056351	0,57807298
Abca8a	2752,648538	-0,431045238	0,120484502	-3,577599046	0,000346765	0,011252182
Slc36a1	959,6715232	-0,219971999	0,102766154	-2,140510182	0,032313559	0,208695637
Uros	437,5973464	0,07705695	0,133958836	0,575228572	0,565136721	0,83747956
Sync	1,851153847	1,491967611	1,113239418	1,340203722	0,180179122	NA
Wdr81	1152,646344	0,008645196	0,122776715	0,070413971	0,943864172	0,983823492
Zfp286	1,95960513	0,410110948	1,185347701	0,34598367	0,729354987	NA
Fancf	59,08560066	0,201776879	0,197519821	1,021552558	0,306992721	0,666222391
Gm10778	2,273567955	-0,050814676	1,062563207	-0,047822732	0,96185752	NA
Ankrd45	0,655607114	0,016546817	2,025326545	0,00816995	0,993481395	NA
Nudt17	2,421948179	0,741330605	0,920732195	0,805153344	0,420731196	NA
Rpn2	5517,906729	-0,270937754	0,090337703	-2,999165851	0,002707199	0,04610515
Micu2	1057,345783	-0,010678372	0,080955142	-0,131904804	0,895059588	0,970433882
Ttc7	1081,331459	-0,233083207	0,09817349	-2,374197013	0,017587164	0,146719059
Hspb9	0,113662148	0,780932884	3,352475198	0,232942181	0,815806304	NA
Psors1c2	0,274362713	1,389394708	3,349408814	0,414817893	0,67827522	NA
Pcdhga9	0,848222657	-0,63056122	1,919328666	-0,328532175	0,742509321	NA
Pcdhga8	0,339756192	1,876228608	3,160061454	0,593731684	0,552691588	NA
Naa15	819,3375662	-0,029634984	0,131593492	-0,225200983	0,821822925	0,946779723
4931440F15Rik	0,093953095	-0,517475177	3,352475198	-0,154356154	0,877328924	NA
Ldhal6b	0,793919181	0,548482204	1,908829498	0,287339548	0,773852344	NA
Ssrp1	1589,548336	-0,044314612	0,093278997	-0,475075994	0,634732808	0,868410828
Ap1ar	480,3036343	0,110316148	0,109383604	1,008525443	0,31320228	0,672219008
Mir145a	0,993355958	0,527796645	1,806886964	0,292102747	0,770208067	NA
Phf11c	4,815413431	-0,380917963	0,7126514	-0,534508124	0,592990031	NA
Fes	149,3373474	0,227961034	0,214013434	1,06517161	0,286798279	0,648217445
Chic1	139,782609	0,1916106	0,269050414	0,712173591	0,476357288	0,789577814
Saa1	10549,94391	2,139737086	0,673018023	3,179316175	0,00147623	0,031079866
Prrx2	0,968000777	1,638259587	1,565128075	1,046725577	0,295226164	NA
Cd5l	2065,157489	0,3378393	0,237757983	1,420937779	0,155334849	0,49024353
S100b	0,3267786	1,84016758	2,655545515	0,692952755	0,488339201	NA
Fxyd3	13,59381619	1,302114772	0,538759635	2,416875149	0,015654386	0,137325118
Masp1	1743,6935	-0,169907101	0,177148154	-0,95912431	0,337496125	0,692850411
Polr1b	366,43014	-0,109860192	0,18469526	-0,594818685	0,551964676	0,830638744

Supplementary Table S1: *Serpina1* KO vs. wildtype all DEGs

Ptrf	176,9843704	0,070739712	0,286668695	0,24676469	0,805090339	0,940843771
Tcf7	114,6457758	0,569923122	0,235491636	2,42014167	0,015514461	0,136494096
Tgfb2	17,67891757	0,283483619	0,364601245	0,777516872	0,436853884	0,76575019
Slc5a2	1,719766337	0,661694404	1,634430917	0,40484697	0,685590001	NA
Naip1	8,054792339	0,241114057	0,596193165	0,404422712	0,685901901	0,893161227
Pgk1	3499,101809	-0,020763598	0,090732896	-0,228843104	0,818990863	0,945934875
Nab2	469,273538	0,699495826	0,221934118	3,151817448	0,001622577	0,033401397
Nab1	1612,412759	0,123298397	0,127256873	0,968893816	0,332598171	0,689808401
Spcs3	1996,459108	-0,309095304	0,124474513	-2,483201553	0,013020739	0,122933801
Apoc4-apoc2	5,150931056	-0,615647877	0,704755037	-0,873562932	0,382356321	NA
Stambpl1	21,57699882	1,044382845	0,452072805	2,310209403	0,020876564	0,161328775
Slc52a2	194,7192091	-0,068803827	0,193701645	-0,355205175	0,722435888	0,909644412
Kctd6	227,1064081	-0,022773724	0,139835772	-0,162860501	0,870628271	0,961775826
Mro	0,581150129	1,661207107	2,388777339	0,695421494	0,486791194	NA
4930538K18Rik	2,410088801	0,301043831	1,011595384	0,297593125	0,766013722	NA
Gins3	25,91871839	0,097362637	0,283723384	0,343160426	0,731477774	0,912146849
Trmt44	64,25486514	-0,287684983	0,205283619	-1,401402534	0,161093735	0,497950692
Meiob	143,3568548	0,549484086	0,126428894	4,346190716	1,39E-05	0,001089354
BC005561	145,6103345	-0,251702931	0,168594781	-1,492946164	0,13545128	0,458151054
8430408G22Rik	1832,313827	0,389833428	0,54418063	0,716367703	0,473764332	0,787824815
Gc	191727,6863	0,024364732	0,058608165	0,415722492	0,677613081	0,890320322
Birc2	505,6765564	-0,06149545	0,112026803	-0,548935153	0,583049953	0,846950064
Nmb	4,395636192	-0,449993917	0,667543745	-0,674104012	0,500245189	NA
Apba2	5,20572024	-0,381568473	0,879412359	-0,433890278	0,66436812	NA
Myg1	430,036949	-0,061522012	0,112840471	-0,545212293	0,585607518	0,847482024
Snord15b	2,727393208	0,376670776	1,006804688	0,374124972	0,708311341	NA
Snord15a	0,526295545	-1,479881071	2,387762898	-0,619777228	0,535404463	NA
Tmco1	1869,47054	-0,056075649	0,090816852	-0,617458628	0,536932261	0,823949426
9130409I23Rik	466,7210821	0,340464038	0,271909153	1,252124226	0,210524601	0,562962817
Gm5150	22,67035551	0,516021182	0,330391597	1,561847177	0,118323995	0,427187007
C330027C09Rik	26,0176033	-0,076006014	0,440964575	-0,172363083	0,863152093	0,961272625
Arhgap29	1754,30216	0,176425157	0,160618424	1,098411706	0,272024753	0,633801597
Nipal4	0,147722973	0,780932884	3,352475198	0,232942181	0,815806304	NA
Slc18a2	2,084332279	-0,486107895	1,104804644	-0,439994435	0,659941138	NA
Gpx2-ps1	0,180429807	0,059593471	3,352475198	0,017775962	0,985817581	NA
Stox2	15,66925256	-0,412861803	0,481578263	-0,857309879	0,39127365	0,734518115
Krt17	0,51448455	1,494857447	2,428757534	0,615482372	0,538236207	NA
H2-T23	5107,9508	0,065116921	0,095620183	0,680995574	0,495874291	0,80006558
Slc2a13	8,717881013	0,89559417	0,576676325	1,553027463	0,120416574	0,4317798
Akr1b7	21,6111028	1,72014719	0,518444103	3,317902896	0,00090696	0,021941826
Efs	3,913084431	0,874630817	0,831548178	1,051810153	0,29288666	NA
Tff1	0,31389621	-0,661096352	2,708970037	-0,244039743	0,80720004	NA
Mir3100	1,166180842	1,974802304	1,429043853	1,381904621	0,167000989	NA
Cdx4	2,89301822	0,899299632	1,368638155	0,657076254	0,511131887	NA
Myo10	832,277097	-0,541726189	0,163361081	-3,316127601	0,000912741	0,022026065
Mgmt	297,9524555	0,469984831	0,14985801	3,136200936	0,001711519	0,034605997
Hars2	652,7249274	-0,20224506	0,085689455	-2,360209423	0,018264621	0,14991666
Ube2n	667,6520025	-0,07795529	0,098251901	-0,793422712	0,427531583	0,759759368
Mmp12	9,332664793	1,157048241	0,880752205	1,31370462	0,188945626	0,53642261
Mesp1	0,866732076	-0,815655019	1,459185032	-0,558979843	0,576175477	NA
Mertk	298,9407382	-0,084517537	0,209932132	-0,402594575	0,687246502	0,893386271
Mep1b	2,330822387	-1,666826513	1,30219917	-1,280008888	0,20054201	NA
Nqo1	179,8052572	1,325202615	0,205158451	6,459410301	1,05E-10	5,99E-08
Nid2	414,1106222	-0,483951766	0,154933114	-3,123617365	0,001786426	0,035539776
Pten	2115,702252	-0,106345272	0,110794514	-0,9598424	0,337134539	0,69270916
Srpk2	422,343895	0,161329431	0,094933793	1,699388862	0,089245939	0,370192045
Psen1	923,8309466	-0,035388159	0,084403219	-0,419274992	0,675015171	0,889003825
Prss12	4,586633968	2,665205847	0,950818199	2,803065665	0,005061936	NA
Ifnar1	866,2523688	-0,077630583	0,080458747	-0,964849512	0,334620192	0,691222347
Usp9x	1655,991959	-0,346053285	0,16930213	-2,043998414	0,040953704	0,238807265
Rsf1	328,8523594	-0,140898723	0,139646006	-1,008970662	0,312988704	0,672219008
Cyp26c1	0,241026348	0,059593471	3,352475198	0,017775962	0,985817581	NA
Cyp2c69	1913,73007	3,927611165	1,247325163	3,148827012	0,001639272	0,033664251
Tgtp2	56,73404628	0,492301524	0,296017127	1,663084595	0,096295503	0,385838157
Morc2a	432,028243	0,051276621	0,128431903	0,39925143	0,689707951	0,894436573
Ece1	5483,987247	-0,108362273	0,092955029	-1,165749433	0,243715768	0,602261755

Supplementary Table S1: *Serpina1* KO vs. wildtype all DEGs

Ccdc32	161,4420722	0,268874699	0,131151513	2,050107487	0,040353943	0,236409184
2310043O21Rik	0,093953095	-0,517475177	3,352475198	-0,154356154	0,877328924	NA
Ssxb5	0,142634598	-0,517475177	3,352475198	-0,154356154	0,877328924	NA
Cep162	82,3861876	0,138835564	0,192070108	0,722837952	0,469779447	0,784373852
Clec4a1	61,24164451	0,419280438	0,328685537	1,27562789	0,20208709	0,553627523
Jade2	242,698677	-0,260524374	0,214980106	-1,211853403	0,225568502	0,580946341
Cdk10	516,8970609	-0,14933308	0,106526981	-1,401833398	0,160965003	0,497812792
Unc93a	1,517590978	-0,172292295	1,284303479	-0,134152323	0,893282126	NA
Kcnk12	0,086476712	0,780932884	3,352475198	0,232942181	0,815806304	NA
Cd300c	1,089909809	0,554192337	1,435215134	0,386138861	0,699393831	NA
Ccdc160	4,902414086	-0,617360752	0,72197793	-0,855096432	0,392497765	NA
Cubn	0,812797611	2,198924817	1,614475149	1,362005986	0,173195996	NA
Idua	124,0629479	0,137424352	0,193222713	0,711222558	0,476946332	0,789927591
Cyb5d2	319,5248506	-0,087500744	0,115225715	-0,759385557	0,447621951	0,772775895
Trim59	9,295869739	0,08123771	0,513290361	0,158268529	0,874245206	0,963600184
Arl1	1392,490296	-0,329682984	0,134217594	-2,456332095	0,014036338	0,128967987
Rmnd5a	2983,163903	0,342821373	0,130444803	2,628095291	0,008586446	0,095980096
Zkscan14	124,9148563	-0,128336642	0,202724734	-0,63305863	0,526695356	0,817815356
Snw1	811,2250057	0,044154116	0,075120137	0,587780016	0,556679959	0,833822555
Tmem79	58,08415508	-0,411672692	0,216115225	-1,904875939	0,0567962	0,288162578
Ndufa13	4080,991032	0,101639339	0,1105792	0,919154233	0,358014906	0,707401735
Yipf5	1001,810357	-0,323859818	0,125705173	-2,576344391	0,009985114	0,104553371
Cblb	151,8414777	-0,089300019	0,234725841	-0,380443918	0,703615919	0,900719679
Clec4d	1,56989389	1,837098839	1,492651749	1,230761858	0,218411945	NA
Pim1	396,4913974	-0,724742466	0,212824396	-3,405354273	0,000660783	0,017773611
Mir692-1	0,12663974	0,780932884	3,352475198	0,232942181	0,815806304	NA
Scimp	23,14421867	0,269468012	0,436726966	0,617017115	0,537223437	0,824035032
Cyb5d1	113,2636095	-0,550126998	0,248447969	-2,214254361	0,026811286	0,18930315
Cuzd1	0,18660675	-1,235117516	3,340448777	-0,369745983	0,711571767	NA
Smcp	0,180974489	-1,204797453	3,342691436	-0,360427361	0,718527568	NA
Crls1	1171,97547	0,242208832	0,11048895	2,192154335	0,028368364	0,193993179
Smyd2	545,0562279	-0,125544809	0,111900498	-1,121932537	0,261891125	0,622562722
Gnl3	523,4228324	0,177322359	0,233948615	0,757954301	0,448478341	0,77297141
Washc5	695,4025893	0,083943912	0,12479428	0,672658327	0,501164685	0,803641654
Lgals1	452,474872	0,122461699	0,215551386	0,568132273	0,569945155	0,841119289
Taf6	478,7218313	-0,234394054	0,110668012	-2,117992808	0,034175677	0,216025826
Bgn	2514,159175	0,013490073	0,163674661	0,082420046	0,934312696	0,98189048
Psme4	3129,90162	-0,171468131	0,096502409	-1,776827466	0,075596631	0,34005766
Ascl2	0,093303375	-0,517475177	3,352475198	-0,154356154	0,877328924	NA
Gm8979	0,147722973	0,780932884	3,352475198	0,232942181	0,815806304	NA
5830411N06Rik	0,588672279	-0,122173094	2,038732723	-0,059925999	0,952214573	NA
Tbck	369,9846129	0,09031006	0,126755466	0,712474674	0,476170889	0,789577814
Mir2861	0,897595583	-0,371539481	1,420826211	-0,261495374	0,793710517	NA
Ano6	251,011822	0,119887858	0,151987064	0,78880304	0,430227133	0,761019227
Fam47e	583,2038658	-0,481665038	0,165821434	-2,904721219	0,003675803	0,056484846
Lrrc24	3,680813732	0,228594072	0,963304137	0,237302077	0,812422451	NA
Olig1	84,50583773	0,043832275	0,282508572	0,155153787	0,876700079	0,964420584
Adnp	8,236003217	-0,203813374	0,543759318	-0,374822771	0,707792278	0,902731692
Ubl5	1595,178227	0,034485555	0,102095345	0,337777934	0,73553054	0,914229638
AI450353	5,6111869	-0,227207208	0,781395256	-0,290771163	0,771226341	0,928273961
Snord83b	1,970116886	0,467006015	1,041930258	0,448212356	0,653999946	NA
Snord43	0,518620807	-0,524944802	2,404460565	-0,218321236	0,82717883	NA
AF357399	4,800245183	0,043537422	0,621747127	0,070024323	0,9441743	NA
Kcna6	0,898422316	2,384444885	2,239656674	1,064647503	0,287035475	NA
Rabgap1l	595,1826826	0,150159078	0,149558985	1,004012415	0,315372629	0,674163205
Uroc1	11198,43417	0,19817499	0,111698623	1,774193679	0,076031119	0,341208471
Celsr2	15,4256877	-0,325035445	0,42420102	-0,766229758	0,443539605	0,770142004
Cbx8	151,9217678	0,001998297	0,135246269	0,014775249	0,988211486	0,996575327
Adat1	126,432818	-0,335078796	0,168934122	-1,983487953	0,047312951	0,259242489
Kpna6	1183,352832	-0,061721137	0,112011137	-0,551026787	0,581615312	0,84591113
Mecr	738,3608929	-0,105682709	0,079931456	-1,322166691	0,18611265	0,532805747
Pam16	291,9259842	-0,040072811	0,130959063	-0,305994948	0,759608493	0,924882144
Nt5dc1	391,9413561	0,015385775	0,134653769	0,114261749	0,909030304	0,9747592
Tceal8	550,2340661	0,661629627	0,183130225	3,61289147	0,000302801	0,010095144
Srp72	2874,582449	-0,181027787	0,105305942	-1,71906527	0,085602488	0,36284707
Swsap1	146,0151576	-0,130548971	0,149270431	-0,874580256	0,381802335	0,726456076

Tctn3	28,87673006	-0,03294083	0,347344879	-0,094836089	0,924445021	0,977951927
Ccdc51	221,9766453	-0,186760073	0,134139383	-1,392283679	0,163836512	0,501678957
Tex12	19,13977782	0,630031594	0,365368265	1,724374159	0,08464032	0,360024318
Brf2	130,3580279	0,148233546	0,149474837	0,991695655	0,321346011	0,678746017
Col27a1	1486,014343	-0,935903175	0,414911238	-2,255670825	0,024091254	0,176190358
Pspc1	118,6521989	0,36747939	0,150870242	2,435731425	0,014861715	0,133167736
Lix1	3,024210262	-1,714100044	0,954267084	-1,796247689	0,072455133	NA
Ctnnbl1	381,6429004	-0,015995671	0,133489576	-0,119827119	0,904620099	0,973666426
Rcc1	148,4350951	0,107895445	0,17789768	0,606502824	0,544180907	0,827477215
Kdm1a	360,3470396	0,09490948	0,098069017	0,967782515	0,333152998	0,68995097
Scaf8	560,1967569	0,04846472	0,129221416	0,375051769	0,707621966	0,902731692
Hook1	1210,599729	-0,435756449	0,144181475	-3,02227765	0,002508803	0,043600932
Zfp595	131,6738461	-0,530896442	0,185421026	-2,863194389	0,004193932	0,060944211
Wscd1	4,703275641	-0,104168019	0,638787949	-0,163071359	0,870462251	NA
Ppm1n	0,930587233	-0,238030826	1,656051083	-0,143733987	0,885710532	NA
Tmem69	342,6857391	-0,030059761	0,119335787	-0,251892262	0,801124341	0,939741275
Qrsl1	119,6891765	-0,252324693	0,162010354	-1,55746029	0,119361242	0,429754718
Nfe2l3	10,32867565	0,375740042	0,559964452	0,671006956	0,502216098	0,804113015
Nek2	66,47230814	-0,782958957	0,356831237	-2,194199595	0,028221064	0,19328248
Strap	1955,846197	-0,010430481	0,090805138	-0,114866638	0,908550829	0,9747592
Mdfic	430,392115	0,046745856	0,182471171	0,256182145	0,797810195	0,938325224
Mzt1	273,7585738	0,03774586	0,192182322	0,196406514	0,844291998	0,953068489
Plcg2	50,30732393	0,166842022	0,238229666	0,700341084	0,48371432	0,794055997
Sash1	805,9768136	0,333453912	0,138471544	2,408104241	0,016035602	0,139207766
Gimap9	350,8093633	-0,208767877	0,15391912	-1,356347908	0,174988499	0,517287416
Rae1	527,5667227	-0,080623684	0,110730544	-0,728107	0,466548089	0,782300439
Trib3	89,24222185	0,871921674	0,32913328	2,64914467	0,008069578	0,092364887
Rhof	8,291948762	-0,019758397	0,579504269	-0,034095344	0,972801121	0,992650147
Leprot	359,0801486	0,195848659	0,120031037	1,631650151	0,102753207	0,398985659
Mylk3	8,242746954	0,683163103	0,710942374	0,960926127	0,336589313	0,69270916
Tbccd1	701,9699916	-0,411788177	0,126499688	-3,255250541	0,001132924	0,025979516
Stk36	20,21509366	-0,939418889	0,332729179	-2,823373923	0,004752111	0,066454995
Tctex1d4	1,470958326	1,206041153	1,456328486	0,828138133	0,407592276	NA
Hectd3	1462,166452	-0,031487217	0,116841033	-0,269487663	0,787554434	0,934424153
Adnp2	169,2840019	-0,338013424	0,147920633	-2,285099897	0,022306971	0,167540389
Gnptg	634,3958037	0,089314728	0,11144517	0,801423046	0,422886778	0,755522295
Nrn1l	1,30704889	1,493014325	1,508825435	0,989520915	0,322408342	NA
Samd4b	513,0112309	-0,090934956	0,097141648	-0,936106785	0,34921821	0,700954068
Wdr35	85,93849926	-0,089053996	0,185648264	-0,479692052	0,63144638	0,86707135
Pgm5	23,31319198	-0,159478924	0,481868522	-0,330959416	0,740675141	0,916181159
Slc5a3	77,69655279	-1,013283516	0,362625417	-2,794298107	0,005201251	0,07004174
Klhl20	218,6185826	0,009693691	0,154024203	0,062936152	0,949817347	0,985972877
Safb	1264,430859	0,034986793	0,133734667	0,261613489	0,793619444	0,936746676
Rb1	189,923486	0,188915343	0,160720296	1,175429291	0,239823011	0,597390316
Ddx3x	3855,373435	-0,069467395	0,175730196	-0,395307107	0,692616255	0,895338108
Syngr1	11,51075354	0,047589435	0,588934855	0,080805941	0,935596283	0,982081594
Ddt	9723,433963	0,24471922	0,088539034	2,763969849	0,005710282	0,074261434
Prkg2	0,746643549	1,179261581	1,774548511	0,664541754	0,506343634	NA
Gcat	2560,833042	0,131001784	0,100285603	1,306287038	0,191454963	0,540562071
Terf1	241,3710001	0,215526714	0,145436089	1,481934198	0,138357804	0,461964408
Dcp1a	177,6911882	-0,125687728	0,143287007	-0,877174638	0,380391789	0,725866642
Ppp1r32	2,401133621	-1,586776604	1,115165413	-1,422906939	0,154763134	NA
Acaca	2412,759208	0,365986474	0,180930427	2,022802244	0,04309354	0,244843833
Zfp617	234,1899631	0,078433135	0,141482589	0,554365987	0,579328399	0,84530168
Pcdhb6	0,147722973	0,780932884	3,352475198	0,232942181	0,815806304	NA
Pcdhb5	1,040764434	-1,480190182	1,570809947	-0,942310166	0,346033865	NA
1110038F14Rik	184,4757045	0,016853932	0,119942624	0,140516618	0,888251824	0,96836734
Trim16	4,605772156	0,996650349	0,819753132	1,21579328	0,22406368	NA
Sumo2	229,8460719	-0,019847778	0,144973183	-0,136906546	0,891104665	0,968944958
Unc119b	899,579759	-0,260527975	0,116248348	-2,24113271	0,02501748	0,180984139
Mob3c	361,5779791	-0,000431776	0,117913055	-0,003661817	0,997078299	0,999481197
Fam46b	2,787865244	3,306232082	1,226446707	2,695781287	0,007022376	NA
Phactr4	898,0067066	-0,123832281	0,137727355	-0,899111736	0,368593147	0,716892516
4930413G21Rik	1,805488016	0,128207268	1,091669143	0,117441506	0,906510195	NA
Map4k4	309,5952759	0,132328625	0,165626599	0,798957568	0,424315016	0,756961229
Adamtsl3	4,681051215	0,370916754	0,807882595	0,4591221	0,646146487	NA

Supplementary Table S1: *Serpina1* KO vs. wildtype all DEGs

Kcng2	4,852253303	0,917114746	0,707354564	1,29654178	0,194788895	NA
Ankrd29	7,335220028	-0,267538226	0,568347605	-0,470729927	0,63783361	0,870273408
Gimap6	229,9998436	-0,042289042	0,233694429	-0,180958709	0,856399987	0,9578959
Sqstm1	10853,30718	0,151577607	0,073345572	2,066622469	0,038769742	0,230874245
Kcnn2	623,8793583	0,197377865	0,174396923	1,131773782	0,257729553	0,618708277
Serpina3c	5,306119878	-0,797685439	0,841563677	-0,94786106	0,343200191	NA
Cyp17a1	1077,896746	2,90965395	0,945864031	3,076186274	0,002096668	0,039203296
Bap1	804,6806879	-0,009144378	0,084183678	-0,108624121	0,913500628	0,976232707
Rhebl1	9,826714821	0,084761354	0,587938016	0,14416716	0,885368472	0,967477365
Ss18l2	504,5574378	0,038116873	0,120378376	0,316642195	0,751515115	0,920691412
Myct1	60,72367808	-0,527268055	0,360182693	-1,463890591	0,143223845	0,470341336
Agpat5	283,8418811	-0,142490691	0,143315916	-0,994241914	0,320105107	0,677494232
Fbxw9	633,4377506	-0,279192994	0,115727189	-2,412509948	0,015843105	0,137992723
Igsf5	649,290574	0,016084534	0,106819511	0,150576747	0,880309605	0,96546599
Gsdmd	925,0821518	0,343777532	0,161005324	2,135193573	0,032745202	0,210669202
Mthfd2l	15,4501744	0,925104632	0,3795634	2,437286189	0,014797963	0,13268542
1110012L19Rik	316,6954837	0,088996766	0,105523591	0,843382658	0,399014468	0,739993697
Stxbp2	883,6581851	0,050248526	0,10204241	0,492427864	0,622416905	0,863183776
Zfp758	120,9798066	0,181393846	0,23133375	0,784121844	0,432968619	0,763032854
Zfp160	148,1976947	0,03929704	0,172917548	0,227258833	0,820222482	0,946381744
Setd4	103,8343985	0,088914521	0,243646612	0,364932308	0,715161937	0,906371815
Rfc4	66,99746598	0,166617228	0,219510624	0,759039471	0,447828946	0,772918303
Zfp772	53,99962774	0,064200069	0,200811761	0,319702737	0,749193685	0,919838327
Mir6973a	0,207615244	0,059593471	3,352475198	0,017775962	0,985817581	NA
Insr	2367,705624	0,111256114	0,086590335	1,284856036	0,198842596	0,548812381
Sars	1487,732401	-0,127572631	0,103515845	-1,232397135	0,217800771	0,571609443
Apcs	2699,532546	0,908964445	0,17192896	5,286860613	1,24E-07	2,64E-05
Scap	3648,985856	0,181486777	0,085147537	2,131438961	0,033052996	0,21203719
Saa3	190,611023	0,489877629	0,370021833	1,323915468	0,18553112	0,532435718
Saa2	3906,528907	2,327373894	0,754355783	3,085246969	0,002033832	0,038665614
S100a6	37,86734666	0,514870027	0,440897171	1,167778024	0,242896315	0,601014405
Ppp1r13b	480,2326178	-0,259551039	0,128591322	-2,018417996	0,043547747	0,245880557
Clec3b	4,373813316	1,954512333	0,991265488	1,97173447	0,048639926	NA
Hcar2	21,07585275	0,026647456	0,653928549	0,040749797	0,967495362	0,991221204
Tnfsf11	0,368335998	-1,145474084	3,124856885	-0,366568494	0,713940912	NA
Kdm5d	324,1079141	-0,101404969	0,879302025	-0,11532439	0,908188007	0,9747592
Sgcb	50,29645582	-0,02503719	0,255039583	-0,098169818	0,921797448	0,977341679
Dctn6	343,893412	0,085828206	0,10437741	0,822287181	0,410913471	0,748353623
Zfp106	1792,262604	-0,375218384	0,112351447	-3,339684467	0,000838736	0,020998249
Wnt9b	13,82821145	0,304272164	0,388955931	0,782279274	0,434050465	0,763946284
Wnt10b	0,213661134	0,059593471	3,352475198	0,017775962	0,985817581	NA
Wfs1	428,6400887	-0,692038134	0,124803826	-5,545007362	2,94E-08	9,36E-06
Wdr1	1085,988388	0,031116956	0,081901391	0,379931954	0,703995927	0,900988086
Baz1b	1160,254566	-0,375569784	0,10127615	-3,708373447	0,000208595	0,007724944
Lrrc17	1,719886959	0,560897159	1,600976259	0,350346956	0,72607833	NA
Malsu1	310,7298481	0,061904668	0,118426364	0,522727087	0,601164186	0,854422974
Dusp9	0,927837749	1,579074437	1,704561999	0,926381345	0,354247835	NA
Gm4532	7,245537031	-0,35784121	0,523678133	-0,683322803	0,494402888	0,799621608
Usp31	112,4332365	-0,099234653	0,157390943	-0,630497865	0,5283689	0,81852028
Slc6a21	1,081319833	-0,998981757	1,605412262	-0,622258706	0,533771769	NA
Cln6	64,73815136	0,15319468	0,256766619	0,596630049	0,550754405	0,830245606
Osbp	1987,104291	-0,513280307	0,112129626	-4,577561933	4,70E-06	0,000442897
Angptl8	2132,993414	0,591390234	0,624133098	0,947538652	0,34336437	0,695942733
Fmnl1	43,07465189	0,62072374	0,306412803	2,025776125	0,042787732	0,243209687
Mir142	0,404019719	0,662784949	2,545415247	0,260383821	0,794567721	NA
5033428I22Rik	1,911297167	-0,232007812	1,069961273	-0,216837579	0,828334925	NA
Gm4832	0,093953095	-0,517475177	3,352475198	-0,154356154	0,877328924	NA
Rps6ka5	49,64107865	-0,018882914	0,251643199	-0,075038444	0,940184124	0,983128178
1700120K04Rik	1,251202331	-0,048146637	1,463415611	-0,03290018	0,973754189	NA
Xrcc6	508,2446898	0,077429828	0,096229456	0,804637488	0,421028902	0,754607148
Cyp2c37	6282,276917	1,104081749	0,380718853	2,899992315	0,003731718	0,0569513
Ppp1r14b	351,7792408	-0,152167588	0,160232103	-0,949669795	0,342280062	0,695326265
Ighmbp2	131,0240287	-0,054414899	0,173220717	-0,314136208	0,753417591	0,921540692
Cyp2b9	2253,141552	8,012976157	2,113540328	3,791257755	0,000149886	0,006072212
Rab24	808,2206122	0,063442072	0,100768132	0,629584677	0,528966351	0,818868823
Pms2	44,6612956	0,03971881	0,282586314	0,140554614	0,888221805	0,96836734

Pmp22	91,17984381	0,065452611	0,178283313	0,367126964	0,713524315	0,905984098
Clec11a	43,43353305	0,312440853	0,21438105	1,45740891	0,145003539	0,473279373
Atxn2	1670,666558	-0,117257136	0,12432324	-0,94316345	0,345597306	0,698427993
Eif4ebp2	1627,323658	-0,139544122	0,164135313	-0,850177329	0,395226504	0,737237775
Ghrh	0,12663974	0,780932884	3,352475198	0,232942181	0,815806304	NA
Tnfaip1	879,7355188	-0,061548561	0,082548253	-0,745607072	0,45590481	0,77799081
Grb7	3197,668768	-0,028298237	0,129056999	-0,219269297	0,826440278	0,947537943
Plg	71434,21703	0,082393762	0,073601687	1,119454808	0,26294616	0,622953609
Morf4l1-ps1	9,191195782	0,33889337	0,549686189	0,616521529	0,537550367	0,82433022
4933413J09Rik	0,588567241	-0,952112744	2,035034035	-0,467860845	0,639884105	NA
Ly6g6f	0,229655992	-1,252384348	3,339190293	-0,375056298	0,707618598	NA
Atp5k	1943,105657	0,08089731	0,13552586	0,596914198	0,550564668	0,830100264
Atf1	594,8344847	0,07923719	0,130109657	0,609003144	0,542522355	0,826611597
Itgav	413,3284036	-0,128709733	0,128938832	-0,998223195	0,318171141	0,675759967
Cspg5	54,97021734	-2,748589316	1,248346713	-2,201783597	0,027680598	0,19164263
Txnrd2	840,0804507	0,116395916	0,116095087	1,002591233	0,316058128	0,67419376
Atp2a1	0,584030666	-1,875581266	1,935812793	-0,968885665	0,332602238	NA
Parp9	1468,137641	0,063726914	0,12434583	0,512497397	0,608302942	0,857753956
Smim12	397,3866454	0,005471315	0,094758596	0,057739516	0,953956117	0,986508391
Abtb1	512,4405488	0,075665224	0,104213815	0,726057518	0,467803511	0,782859415
Etnk1	2100,874457	-0,340490661	0,140393333	-2,425262317	0,015297329	0,135526833
Amn	2,851663632	1,186292525	0,960902475	1,234560796	0,216994007	NA
4930550C14Rik	0,673275482	0,063380619	2,021291426	0,031356497	0,974985234	NA
Tada1	289,1555662	0,039565582	0,104830374	0,377424794	0,705857944	0,902122257
Tm4sf5	1,495094929	-0,677709446	1,449929991	-0,467408392	0,64020772	NA
Gm1141	0,647914829	-0,566052001	1,773958251	-0,31908981	0,749658411	NA
Brpf3	641,1332515	-0,244218971	0,104670043	-2,333227009	0,019636234	0,156064296
C2cd4b	2,080857963	-0,352876268	1,005000173	-0,351120604	0,725497871	NA
Pik3r4	522,356589	-0,123859305	0,118092963	-1,048828839	0,294256902	0,655325757
Cnot9	539,5679416	-0,03136657	0,105555305	-0,297157683	0,766346128	0,926944289
Vmn2r1	0,696272157	2,027338104	1,942227092	1,043821349	0,296568052	NA
Ralb	296,4140681	0,055264967	0,156696542	0,352687853	0,724322467	0,910252319
Dcpp1	0,093953095	-0,517475177	3,352475198	-0,154356154	0,877328924	NA
Uba52	7464,848693	0,089143625	0,117023919	0,761755593	0,446205888	0,771638844
Sav1	294,5326865	0,0507688	0,119289451	0,425593371	0,670404171	0,887361539
Nsa2	970,1564212	0,048768417	0,08808496	0,55365203	0,579817011	0,84530168
Ulk4	1,856225961	-0,207355722	1,029032836	-0,201505448	0,84030337	NA
Psmc1	2771,2799	0,112289601	0,081343325	1,380440252	0,167451134	0,506597468
Dgat1	422,5693678	0,132249055	0,116968467	1,130638531	0,258207263	0,619410174
Rela	1131,286441	-0,199301224	0,08386537	-2,376442429	0,017480489	0,146243215
Ackr1	23,71958979	-0,07063504	0,343572431	-0,205589954	0,837111225	0,951141594
Psmb4	2574,890124	0,13108521	0,100804305	1,300392965	0,19346632	0,542005706
Ccr1l1	1,226627057	-0,614263032	1,374951865	-0,44675239	0,65505385	NA
Cc2d1a	441,3667256	-0,067039972	0,1074557	-0,623884748	0,532703278	0,820654481
Spidr	123,9623433	0,00538815	0,174258508	0,030920441	0,975332988	0,993185764
Phyhip	2,105993014	1,161848654	1,251188834	0,928595766	0,353098617	NA
Cpped1	1966,754923	0,137050983	0,088608971	1,546694211	0,121937	0,433849653
Abhd14b	6672,090274	0,326374827	0,081763089	3,991713501	6,56E-05	0,003282365
Tsen54	140,4133248	0,009722065	0,144158022	0,06744033	0,946231163	0,984653942
H2afx	224,1762901	-0,568462742	0,249984483	-2,27399211	0,022966461	0,17076675
Hira	271,9880791	0,09298995	0,144248968	0,644648979	0,519154687	0,813725618
Zxdb	20,54222983	0,047497886	0,423947605	0,11203716	0,910793943	0,974815504
Tbc1d31	244,5057687	-0,288538811	0,184689726	-1,562289456	0,118219815	0,427187007
Fam65c	25,54735778	-0,044980566	0,396254254	-0,113514405	0,909622743	0,9747592
Gm13238	0,514267002	1,246424445	2,425527231	0,513877737	0,607337473	NA
Fcna	1129,270234	-0,041059322	0,225073244	-0,182426488	0,85524804	0,957445239
Dscr3	555,7843208	0,025795471	0,084964279	0,303603715	0,761429821	0,92534041
Slc25a4	121,5043257	0,104078951	0,219614984	0,473915527	0,635560146	0,869060407
Pde7b	118,1223121	0,13681901	0,237871807	0,575179598	0,565169839	0,83747956
Bmp2	217,5786992	0,121823309	0,265378552	0,459054841	0,646194785	0,875808808
Alx3	0,12663974	0,780932884	3,352475198	0,232942181	0,815806304	NA
Klkb1	7088,474841	-0,159838547	0,106895985	-1,495271753	0,134843534	0,45707992
Fam49a	93,86178959	0,307220443	0,23602059	1,301667972	0,193029913	0,541594346
Smim19	385,291061	0,479902921	0,11572821	4,146810208	3,37E-05	0,002003207
Ascc3	776,2856154	-0,237481028	0,124558041	-1,906589305	0,056573791	0,287361327
Coa5	2139,653594	-0,115796295	0,08751021	-1,323231816	0,185758299	0,532804698

Gm11837	44,37648631	0,011778099	0,281617518	0,041823032	0,966639774	0,990593599
Trappc2l	261,993373	0,108509223	0,144135068	0,752830142	0,451551992	0,774656859
Ccdc88b	57,9033783	0,119316039	0,254669972	0,468512396	0,639418208	0,871049783
Ak6	207,889023	-0,34898499	0,132856594	-2,626779592	0,008619715	0,096170324
Lpcat2b	0,087021394	-0,517475177	3,352475198	-0,154356154	0,877328924	NA
Mär-03	22,53171436	0,338118697	0,338629851	0,998490522	0,318041558	0,675759967
Arhgef25	33,05092407	0,136690378	0,277283053	0,492963334	0,622038495	0,863017086
Wdr73	255,611158	0,116503345	0,116683742	0,998453965	0,318059277	0,675759967
Tha1	136,1057346	0,018958375	0,183082663	0,1035509	0,917525754	0,976907366
Pdgfd	15,97776289	0,70168813	0,422456295	1,660972125	0,096719045	0,386789385
Nkiras2	472,932177	0,142319663	0,12051247	1,180953828	0,237621066	0,59478675
Cdca4	61,66578002	0,165177223	0,244651108	0,675154201	0,499577801	0,802860694
Gatsl3	34,14244382	-0,308072184	0,266602411	-1,15554913	0,247865626	0,607129663
B3gat2	1,468701293	-0,411643505	1,754692072	-0,234595865	0,814522419	NA
Rnf135	309,7521743	0,294414188	0,135607609	2,171074241	0,029925561	0,199737805
Mär-08	2247,368187	-0,301060758	0,113772232	-2,646170786	0,00814087	0,092812169
Ist1	1006,080291	0,042020971	0,075584985	0,555943372	0,578249567	0,84530168
Aldh1b1	2919,835592	0,78756883	0,376465839	2,092006094	0,036437971	0,223663557
Rbm33	904,9724444	-0,283246314	0,14878876	-1,903680859	0,056951761	0,288295377
Shroom1	961,6091839	0,284668703	0,118367597	2,404954655	0,016174473	0,140121599
Napg	369,6554563	0,010477388	0,110553668	0,094771957	0,924495961	0,977951927
Cers5	44,47077181	0,050430165	0,229697115	0,219550714	0,82622108	0,947537943
Tmem94	955,2011581	0,105047581	0,12647908	0,830553008	0,406226191	0,745871812
Pawr	167,0288649	-0,15099415	0,206819327	-0,730077559	0,465342778	0,781663619
Slc13a3	496,5235917	0,666415978	0,21365064	3,119185507	0,001813517	0,035971684
Brdt	38,35950067	0,025473849	0,257279164	0,099012486	0,921128357	0,977341679
Tgs1	287,756225	-0,024038543	0,114675796	-0,209621771	0,833962887	0,950814415
Tgif2	11,37855003	0,230031882	0,44970195	0,511520758	0,608986457	0,857753956
Snhg17	163,3849359	0,397120319	0,22965304	1,729218643	0,083769971	0,358161894
Plpp5	448,9338854	-0,074559502	0,167411876	-0,445365666	0,65605552	0,879592859
Snora52	3,49585747	-0,416814155	0,78289809	-0,532398994	0,594449682	NA
Tmem8b	49,90284469	0,643324796	0,304551541	2,112367563	0,034654935	0,217104886
Phlda3	15,75089434	-0,185225593	0,442913478	-0,418198141	0,675802255	0,889164486
Plek2	13,86377019	1,22363665	0,482165057	2,537796202	0,011155292	0,112895613
Ppp3cb	485,4773792	0,121827215	0,141975883	0,858083866	0,390846156	0,734518115
Shank3	88,93076692	-0,143534431	0,300521992	-0,477617062	0,632922785	0,867527474
Stk25	1025,319012	-0,029325756	0,09150781	-0,32047271	0,748610015	0,919838327
Egfl6	1,515781759	1,253339928	1,193619969	1,05003264	0,293703106	NA
Eno2	1,845957317	-0,636113701	1,397875	-0,455057642	0,649067771	NA
Nr4a3	2,651995812	-0,881621507	0,950266441	-0,927762435	0,353530814	NA
Ak2	6639,189823	-0,153255139	0,084464184	-1,814439354	0,069610112	0,322876701
Syn1	12,71729797	-0,319552104	0,500400222	-0,63859305	0,523087704	0,815322684
Aqp7	1,734183214	0,388544088	1,321084577	0,294109926	0,768673906	NA
Clstn3	346,8496662	-0,38467257	0,309558847	-1,242647638	0,213997707	0,567220469
Olfm3	70,07286101	0,128174412	0,268791073	0,476855169	0,633465258	0,867882458
Phykpl	324,3881304	0,236028002	0,123683155	1,908327793	0,056348863	0,287091444
Defb1	49,69747846	1,382160952	0,322892912	4,280555259	1,86E-05	0,001332808
Rps12	5136,966178	0,044735183	0,16500939	0,271106895	0,786308819	0,934050588
Sub1	1152,336109	0,164057915	0,084789514	1,934884485	0,053004472	0,278107062
Arih2	834,3665089	0,009274349	0,091367855	0,101505595	0,919149116	0,976952181
Acly	15610,34508	0,606035894	0,23279776	2,60327202	0,009233866	0,100023317
Brk1	1168,122546	0,071442916	0,065501236	1,090710947	0,275400092	0,636882421
Tesk2	355,3649252	-0,045069286	0,123991374	-0,36348727	0,716240921	0,907279218
Dnm3os	8,54039422	-0,012711136	0,700027606	-0,01815805	0,985512769	0,995899882
Maml1	442,0116336	-0,043901917	0,098745864	-0,444594994	0,656612466	0,879937055
Sobp	3,152324872	1,24830191	0,943347726	1,323268055	0,185746251	NA
Chchd10	13077,94724	-0,039518434	0,128001003	-0,308735344	0,757522856	0,923804952
Psat1	55,05249313	1,802157483	1,026180547	1,756179737	0,079057723	0,348953931
Abca4	4,716088758	1,5231709	0,866094028	1,7586669	0,078634103	NA
Mir6909	0,32815278	0,617580015	2,680419673	0,230404224	0,817777679	NA
Mir3470b	0,172953423	1,307385949	3,350719078	0,39018071	0,696402925	NA
Dnajb14	125,9452188	-0,184413331	0,148883231	-1,238644069	0,21547734	0,569354126
1700029J07Rik	24,41749462	0,568218595	0,340517054	1,6686935	0,095178138	0,384073808
Akna	74,39302962	0,180716507	0,257802828	0,700987295	0,483310944	0,794038919
Gm16432	1,124738836	1,24720457	1,577837085	0,790452057	0,429263811	NA
Ankrd54	203,7729861	-0,195607884	0,125826752	-1,554581053	0,120045879	0,430959549

Supplementary Table S1: *Serpina1* KO vs. wildtype all DEGs

BC005624	678,7823664	-0,20394591	0,080412077	-2,536259676	0,011204362	0,113220795
Tor1a	1222,535864	-0,121168408	0,102275502	-1,184725626	0,236125947	0,593377403
Spats2l	26,17207292	0,213152892	0,40080714	0,531809118	0,594858207	0,852405191
Hhat	21,63070574	0,609631398	0,378997152	1,608538204	0,107717356	0,407655486
Ppp2r5a	4492,129734	0,068522952	0,080921607	0,846781894	0,397116702	0,738461552
Frmd4b	1351,094943	0,066070803	0,231270946	0,285685702	0,775118857	0,929461958
Mettl13	166,2930781	0,088046099	0,185938811	0,473521898	0,635840882	0,869346129
Rab29	278,3638991	0,092112115	0,112712369	0,817231652	0,413796058	0,750615942
Cox15	826,0544934	-0,17467187	0,116607888	-1,497942152	0,134148281	0,456110978
Usf2	1181,153284	0,019861439	0,090628891	0,219151298	0,826532192	0,947537943
P2rx6	0,964657692	-0,171501925	1,836485869	-0,093385921	0,925596975	NA
Cd36	884,4440802	1,640497173	0,542179618	3,025744825	0,002480215	0,043424918
Frk	129,2385952	0,033716296	0,217990844	0,154668406	0,877082739	0,964682775
Cox7a2l	2223,260869	-0,011526876	0,083938064	-0,137325964	0,890773149	0,968877905
Pex19	2443,41653	0,118233517	0,097125704	1,217324691	0,223480704	0,578565749
Pfas	172,3508828	-0,145814541	0,163497004	-0,891848395	0,372474198	0,719447489
Wipf2	260,1136676	0,123731313	0,119595581	1,034580975	0,300864643	0,661404508
Cachd1	23,14167164	-0,053501335	0,436578854	-0,122546785	0,902465996	0,973338566
Zbtb39	223,5271285	0,20378204	0,142016146	1,434921627	0,151309432	0,484052596
Ifnz	0,12663974	0,780932884	3,352475198	0,232942181	0,815806304	NA
Tubgcp3	354,1967452	0,176891458	0,099045489	1,785961791	0,074105445	0,335608297
Caprin2	37,4456051	0,647211803	0,332310805	1,947609868	0,051461662	0,273331789
Alkbh6	248,0431618	-0,243646506	0,16316836	-1,493221511	0,135379213	0,458151054
Iqcc	167,6580965	-0,097489997	0,128574565	-0,758237036	0,448309093	0,77297141
Ranbp3l	9,043353809	1,136697112	0,664875687	1,709638561	0,08733273	0,366463048
Rcor1	258,9110296	-0,399835981	0,182056981	-2,196213403	0,028076672	0,192787383
Mir7662	0,086476712	0,780932884	3,352475198	0,232942181	0,815806304	NA
Srebf2	1496,055424	0,382827379	0,162950018	2,349354628	0,018805986	0,152616689
Ngfr	149,2445756	-0,011520893	0,234423305	-0,049145681	0,960803199	0,988470616
Aipl1	0,113662148	0,780932884	3,352475198	0,232942181	0,815806304	NA
Lcn8	8,485388917	0,518602143	0,903228911	0,574164685	0,565856365	0,837781736
Yipf3	1327,218555	0,00897932	0,087790268	0,102281498	0,91853324	0,976907366
Nsun5	190,7131961	-0,023824756	0,148256481	-0,160699594	0,872330009	0,962623186
Lpcat1	20,80147462	-0,233025284	0,41372524	-0,563236809	0,573273641	0,842365544
Alms1	57,12487961	-0,148316025	0,22924739	-0,64696931	0,517651806	0,813424627
B3gnt7	1,782853073	2,099988129	1,410612076	1,488707041	0,13656453	NA
Appl1	478,5950045	-0,05269251	0,121035621	-0,435347126	0,663310483	0,88377495
Appl2	488,0947951	0,359369941	0,1319754	2,723007017	0,006469068	0,080389854
Wbscr17	0,296539119	-0,589942906	3,316285543	-0,177892675	0,858807264	NA
Oas1a	72,8329292	0,356514319	0,240027696	1,485304926	0,137463067	0,460587406
Mir7036b	1,051829511	-0,684816511	1,448516058	-0,472771087	0,636376504	NA
Ephx3	1,356836517	-0,183247595	1,433843833	-0,127801641	0,898305951	NA
Fam83e	0,450248828	-1,290755526	2,138800154	-0,603495153	0,546179339	NA
1700066B19Rik	2,020676323	-0,081062543	1,301145272	-0,062300916	0,950323199	NA
Mrpl23	253,4357317	0,260576305	0,131100003	1,987614792	0,046854309	0,25756384
Rlim	660,1139169	0,067118066	0,095875571	0,700053891	0,48389365	0,794055997
Cd14	54,55312175	0,277991112	0,278648519	0,997640733	0,318453602	0,676100715
Timm17a	516,2990119	0,14424219	0,11361758	1,269541117	0,204248135	0,557220166
Agxt	7684,959026	0,064334335	0,182205411	0,353086852	0,72402333	0,910252319
Trim28	1429,143025	0,029096887	0,075957409	0,383068457	0,701669006	0,900078003
Rpusd4	204,7012648	-0,117382675	0,124698277	-0,941333573	0,346533943	0,698497639
Zcchc12	0,299593163	1,742274127	3,264336744	0,533729901	0,593528419	NA
Kif1bp	342,4762763	-0,03631438	0,133616292	-0,271781077	0,785790356	0,933798335
2700097O09Rik	80,28361764	-0,148746471	0,164165598	-0,906075774	0,364895747	0,713569421
Cyth4	130,4293688	0,21046609	0,189999821	1,107717305	0,267983923	0,628319374
Tmem50a	1541,60709	0,066939438	0,07660001	0,873882891	0,382182033	0,726783025
Dhh	3,482636371	-0,48222329	0,798942773	-0,60357676	0,546125068	NA
Amigo1	175,0364828	-0,097907268	0,123925397	-0,790050071	0,429498527	0,760857915
Cdh3	20,33429229	0,867475317	0,435298348	1,992829337	0,046280138	0,255563473
Prkx	83,77882625	-0,266404971	0,198366551	-1,342993409	0,179274108	0,522135977
Tpst1	768,5233827	-0,670406981	0,144434444	-4,641600451	3,46E-06	0,000361089
Ell2	1273,773271	0,120143611	0,128589443	0,934319404	0,350139158	0,701844955
1110002L01Rik	152,2441921	-0,324375952	0,169630677	-1,91224817	0,05584437	0,285438885
4930412C18Rik	106,5503247	-0,035748437	0,164707733	-0,217041641	0,828175893	0,94762353
A930011G23Rik	3,21878028	0,788902667	0,886955277	0,889450334	0,373761104	NA
4930539J05Rik	4,572329055	0,662524509	0,811272945	0,8166481	0,414129559	NA

Supplementary Table S1: *Serpina1* KO vs. wildtype all DEGs

2810410L24Rik	41,36404288	0,56958736	0,247677448	2,299714268	0,021464413	0,163367723
Gm6548	252,3837482	0,010836192	0,159777006	0,067820721	0,945928349	0,984653942
Fam64a	4,080313097	-0,800481817	0,808563068	-0,990005417	0,322171471	NA
Gata2	17,45892426	0,180224723	0,445186559	0,404829659	0,685602726	0,893161227
Eefsec	1064,255467	0,063725178	0,092620802	0,688022315	0,491438729	0,797760449
Nup160	190,9137676	0,155369669	0,14310955	1,085669471	0,277625252	0,638252527
Hnrnph1	1405,86973	-0,242268686	0,094845782	-2,554343284	0,010638831	0,109274782
Wdr8	257,1742163	-0,169487164	0,164159853	-1,032451971	0,301860436	0,662128551
Moxd1	2,508331989	-0,346412458	1,260770425	-0,274762519	0,783498696	NA
Rsc1a1	1,199116923	1,212815502	1,37038814	0,885016053	0,376147974	NA
Ddx56	656,1345373	-0,043352185	0,13422803	-0,322974156	0,746714814	0,919391514
Serpina12	1492,992675	-0,768614028	0,852770357	-0,901314195	0,367421288	0,715730031
Ubxn2b	83,07642896	0,140773096	0,184660409	0,762335017	0,44586008	0,771412373
Hypk	1376,124001	-0,136496148	0,107038287	-1,275208638	0,202235405	0,553920329
A930013F10Rik	12,13183673	0,053166225	0,453519081	0,117230404	0,906677474	0,974457847
Efna1	1379,898009	-0,333695079	0,15686563	-2,127266998	0,033397905	0,213595723
Pak6	12,76760006	0,873663635	0,432244033	2,021227753	0,043256194	0,245350894
Enoph1	91,95130381	-0,013098431	0,164586958	-0,079583651	0,936568399	0,982191945
Zufsp	106,1417857	0,443694535	0,190770035	2,325808324	0,020028778	0,158066552
Bbs5	72,40412916	-0,024024085	0,191943165	-0,125162491	0,900394911	0,972376014
Uaca	216,3765385	0,272177753	0,14449433	1,883656983	0,059611389	0,295164687
Pcbd2	406,3600233	0,01868082	0,110690919	0,168765603	0,865981015	0,961775826
Lig4	133,7469338	-0,06761181	0,190665883	-0,354608854	0,722882642	0,909744709
Kif6	0,086476712	0,780932884	3,352475198	0,232942181	0,815806304	NA
Fbxl7	17,55718454	-0,282224703	0,487153508	-0,579334232	0,562363672	0,837220482
Rnf222	2,318126425	0,216706121	1,011821883	0,214174179	0,830411231	NA
Hif1an	627,6043338	0,039746038	0,099410925	0,399815588	0,689292349	0,894327388
Mir1968	2,352868887	-0,204834653	1,022671462	-0,200293702	0,841250888	NA
Aim1l	33,05652729	0,889310306	0,491563162	1,809147582	0,070428079	0,324561524
Megf6	21,2345574	0,176071571	0,342413796	0,514207001	0,607107274	0,857252713
Nps	0,285269197	-1,521344816	3,321239936	-0,458065315	0,646905517	NA
Psma8	16,82968076	0,035977681	0,494001625	0,072829075	0,941942134	0,983128178
Nhsl1	369,3066629	-0,428339256	0,145017003	-2,953717469	0,003139713	0,050755531
Adssl1	515,3701223	0,336703074	0,127723434	2,636188708	0,008384309	0,094532042
Prkd1	2,796699579	0,706169587	0,945821406	0,746620432	0,455292712	NA
Cacng8	0,387627262	0,666608971	3,05922977	0,217900916	0,827506313	NA
Cacng6	0,122496332	0,780932884	3,352475198	0,232942181	0,815806304	NA
Il6	1,176353826	-0,348837431	1,394952989	-0,250071102	0,802532363	NA
Sart3	363,0461459	-0,201400995	0,092084289	-2,187137436	0,028732492	0,195483304
Sec61a1	5181,294009	-0,420303798	0,108879571	-3,860263185	0,000113265	0,004964719
Agpat1	232,6893363	-0,181233972	0,155830683	-1,163018533	0,244821983	0,603656417
Pdzrn3	73,11974087	0,394778346	0,448228919	0,880751619	0,378452271	0,72423825
Snora70	7,976895016	0,140605541	0,602892106	0,233218414	0,815591807	0,944267443
Snora65	16,25502325	-0,107288487	0,44763901	-0,239676357	0,810581165	0,942482136
Snora64	3,208136848	-0,347895319	0,898454891	-0,387215121	0,698596957	NA
Acadvl	10274,38687	-0,12634152	0,10356176	-1,219963044	0,222478885	0,577161019
Emcn	64,12303334	-0,485078808	0,311792319	-1,55577536	0,119761519	0,430516739
Ggnbp2	1888,933	0,173413964	0,07751513	2,237162784	0,025275703	0,181959388
Leap2	1622,530461	0,483443022	0,204613919	2,362708386	0,018141939	0,14951702
Csrnp1	163,4313515	-1,130481727	0,337370748	-3,350858758	0,000805614	0,020514764
Ddx27	641,845193	-0,264064787	0,130766386	-2,019362887	0,043449516	0,24557177
Ago4	113,9022671	0,00402021	0,181195276	0,022187169	0,982298652	0,995338444
Slc37a1	51,12410236	1,214894828	0,420067101	2,892144673	0,003826217	0,057613111
Tmem5	413,4489358	0,005511005	0,1099214	0,050135869	0,960014116	0,988252
Nomo1	1205,114609	-0,33487883	0,096977518	-3,453159436	0,000554061	0,015726639
Sirt7	1024,276458	-0,047507475	0,097961963	-0,484958378	0,62770587	0,865852703
Sec63	5148,49454	-0,140381356	0,090738834	-1,54709235	0,121840978	0,43379282
BC048644	6,615976058	0,811622435	1,786106724	0,4544087	0,649534691	0,87669042
Serpina7	1066,918688	0,348149514	0,262797627	1,324781804	0,185243532	0,531898792
Ndufb6	1385,720059	0,070789754	0,099069485	0,714546505	0,47488931	0,788782114
Hyal3	9,199609134	-0,014892732	0,544572889	-0,027347546	0,978182535	0,994429459
Hmgb1-rs17	0,086476712	0,780932884	3,352475198	0,232942181	0,815806304	NA
Asl	12610,75509	0,268561969	0,115673408	2,321726093	0,020247689	0,159043099
Rpl23	8138,634477	0,039154986	0,137751416	0,284243804	0,776223549	0,930009104
Asz1	1,804428819	0,804126383	1,349887114	0,595698984	0,551376337	NA
Xk	145,6616567	0,063864305	0,180425835	0,353964303	0,723365635	0,910181193

Supplementary Table S1: *Serpina1* KO vs. wildtype all DEGs

Elac2	679,3689301	-0,043936646	0,11520024	-0,381393702	0,702911134	0,900661526
Svbp	136,8796603	0,40023609	0,159359985	2,511521889	0,012021183	0,117051126
Hsbp1	1788,462158	-0,009020796	0,086793243	-0,103934308	0,917221481	0,976907366
Pno1	735,2834665	-0,248078324	0,105691979	-2,347182116	0,018916007	0,15301751
Sdccag8	179,6577034	0,041673106	0,16627361	0,250629707	0,802100412	0,939799232
Bri3bp	797,9612325	-0,057542044	0,095787871	-0,6007237	0,548024031	0,828793356
Rpl18a	6506,33387	0,010477914	0,120113031	0,087233783	0,930485687	0,980427423
Sgpp1	1579,265716	-0,191690741	0,196013168	-0,97794828	0,328099905	0,685487182
Sil1	1340,163018	-0,029087582	0,110955688	-0,262154941	0,79320199	0,936746676
Usp42	187,3465189	-0,04227397	0,170289294	-0,248247962	0,803942559	0,940361036
Mir1901	1,007972751	-2,825992115	1,507511795	-1,874606968	0,060846814	NA
Zcchc6	2337,980778	-0,166437762	0,129766889	-1,28259037	0,199635626	0,549954396
Cntrob	184,1588275	-0,147904703	0,151533678	-0,976051699	0,329038845	0,686259021
Cntf	4,235061924	-0,510699446	0,68997012	-0,740176178	0,459193101	NA
Igsf11	590,699936	0,439856158	0,180228426	2,440548186	0,014664989	0,13193556
Mylip	135,6734559	0,390394215	0,303914741	1,284551755	0,198948967	0,548854465
Acap1	60,2444582	-0,075826649	0,228857611	-0,331326752	0,740397686	0,916128178
Dcaf8	3403,843424	0,044825468	0,072457503	0,618644952	0,536150282	0,823221904
BC028528	24,95336079	0,190845093	0,311869259	0,611939419	0,54057784	0,826407188
Nrn1	7583,805247	0,267175303	0,103373771	2,584556019	0,009750446	0,103046417
Tram1l1	0,577305232	-0,544105498	2,801612879	-0,194211521	0,846010262	NA
Insig1	10577,26946	0,102378859	0,148443622	0,689681767	0,490394334	0,797285888
Tmem41b	425,1656753	-0,198574685	0,148389643	-1,338197735	0,180831987	0,524412763
4930402H24Rik	1019,573102	-0,618230203	0,126397377	-4,891163249	1,00E-06	0,000131386
Ccdc65	0,567484008	-0,951208918	2,054225886	-0,463049816	0,64332866	NA
Bcl2l13	905,9610838	0,048373875	0,096359857	0,50201273	0,615658567	0,860018481
Rhobtb2	215,6432546	-0,01596931	0,122656168	-0,130195735	0,896411568	0,970995624
Parvg	28,71385409	0,402880074	0,34966463	1,152189953	0,249243025	0,608497079
Arrdc1	77,89199683	0,386617101	0,217865987	1,77456383	0,075969934	0,34104837
Trpm6	6,065354567	0,184522601	0,80220183	0,230020169	0,818076097	0,945522551
Aaas	263,3914246	-0,334932692	0,112227335	-2,984412779	0,002841232	0,047572443
Hif1a	789,7439618	-0,079384347	0,127363986	-0,623287236	0,533095784	0,820918853
Nars2	544,141711	-0,052762533	0,126077189	-0,418493885	0,675586057	0,889164486
Fkbp14	182,8424931	-0,103130769	0,137059997	-0,752449813	0,4517806	0,774656859
Qsox2	166,1554345	0,078934674	0,134270119	0,587879679	0,556613057	0,833822555
Thoc1	429,9793516	0,034573411	0,105854735	0,326611855	0,743961476	0,917783609
Fermt2	4085,665275	-0,086552156	0,128201478	-0,67512604	0,499595691	0,802860694
Bhlhe22	0,220592835	0,059593471	3,352475198	0,017775962	0,985817581	NA
Cxcl9	370,7924506	0,451101064	0,273483404	1,649464124	0,099052587	0,392481932
Fdx1	1665,74841	0,098593905	0,131100839	0,752046327	0,4520232	0,774656859
Cnr1	0,803018461	1,136246044	1,763247359	0,644405357	0,519312611	NA
Itga7	182,9201864	0,376944994	0,238834386	1,578269361	0,114503741	0,418961344
Krt15	0,147722973	0,780932884	3,352475198	0,232942181	0,815806304	NA
Ackr3	19,23500911	-0,084330929	0,501694396	-0,168092228	0,866510724	0,961775826
Ccr7	7,196942522	0,008970786	0,5631025	0,015931	0,987289439	0,996231603
H1f0	3551,420876	0,02317124	0,111984366	0,206914957	0,836076276	0,951141594
Zfand5	1061,097343	0,15317461	0,096593201	1,585770092	0,112791472	0,41594735
Il16	93,43410203	0,225567719	0,240290503	0,938729228	0,347869783	0,699980604
Cfap77	1,649795285	-1,684489554	1,191917307	-1,41326042	0,157579167	NA
Orc2	117,1029836	0,037639445	0,163577227	0,230101985	0,818012523	0,945522551
Slc25a28	429,1270294	0,115398569	0,104108445	1,108445809	0,26766933	0,628022337
Aco1	7709,596863	-0,038442816	0,08986114	-0,427802453	0,668794951	0,886576073
Mfn1	1139,626866	-0,094401866	0,07589879	-1,243786178	0,213578269	0,566983296
Cdc42ep5	104,5163208	-0,00368373	0,730457296	-0,005043046	0,995976248	0,998991922
Bbip1	254,1762441	0,19945476	0,124494653	1,602115074	0,109130165	0,40910239
Zfp53	144,3488416	-0,107607903	0,147942497	-0,72736303	0,467003596	0,782586618
Zfp385a	508,1208931	0,08244024	0,204365258	0,403396548	0,686656526	0,893161227
Fyn	148,4389823	-0,243256741	0,285299431	-0,852636616	0,393860851	0,736368577
Zbtb18	513,2803063	-0,309458274	0,166081172	-1,863295345	0,062420725	0,303234981
Fam84b	220,0429103	1,19282477	0,290645151	4,104058729	4,06E-05	0,002287793
Zfp108	41,35266432	0,098830416	0,242435321	0,407656837	0,68352563	0,892293506
Copg2	679,2585776	0,099692978	0,118901166	0,838452481	0,401776625	0,742183948
Mir7078	0,336157466	0,663936047	2,673207616	0,24836681	0,803850611	NA
Gmeb2	359,8030301	0,031044874	0,121248944	0,256042425	0,797918078	0,938325224
Ppp2r5b	295,2433525	-0,43186961	0,116050246	-3,721401937	0,00019812	0,007482101
Tmem63b	2070,831443	-0,305473282	0,088963488	-3,433692733	0,000595419	0,016618274

Fgb	140566,6982	0,153158719	0,128606368	1,190910854	0,233688583	0,590806102
Rab35	464,6045316	-0,125799966	0,095162392	-1,321950447	0,186184652	0,532805747
Setx	511,9080999	-0,163719878	0,125481335	-1,304734913	0,191983129	0,541481529
Dagla	28,02956019	0,092755765	0,343653249	0,269910922	0,787228785	0,934401226
Ssh3	328,9272321	0,112511669	0,123607989	0,91022975	0,362701358	0,711918625
Akap6	0,951201268	-1,081541511	1,486799031	-0,727429524	0,466962874	NA
Zfyve9	499,5760325	-0,073243857	0,108639354	-0,674192682	0,500188821	0,803121392
Ces2g	1280,903934	0,168506302	0,176538102	0,95450387	0,339828651	0,694330921
Gnl3l	316,2692209	-0,216186179	0,172281091	-1,254845657	0,209534795	0,561873081
Zgrf1	17,69927117	-0,265740912	0,352533097	-0,753804152	0,45096683	0,774175112
Tspan15	88,87828868	0,300834093	0,26177618	1,149203464	0,250472093	0,609605208
Slc9c1	0,093303375	-0,517475177	3,352475198	-0,154356154	0,877328924	NA
Bst2	3987,851335	-0,219691949	0,094427617	-2,32656458	0,019988452	0,157934757
Fam120c	157,9003216	-0,384405062	0,204231449	-1,882203078	0,059808448	0,2959149
2010320M18Rik	130,5183109	0,115988891	0,190407694	0,609160739	0,542417901	0,826611597
Phb	1835,836685	0,174746026	0,079482019	2,198560485	0,027909189	0,192481874
9330020H09Rik	29,5927092	0,038745387	0,25935195	0,149393083	0,88124347	0,965734294
Mir7655	0,180429807	0,059593471	3,352475198	0,017775962	0,985817581	NA
Kcnh6	0,12663974	0,780932884	3,352475198	0,232942181	0,815806304	NA
Washc2	1441,832523	-0,000636712	0,112319429	-0,00566876	0,995477008	0,998970884
D10Wsu102e	423,8599142	0,013860567	0,108991549	0,127171025	0,898805038	0,971783074
Wdr83	438,2302786	0,009454779	0,126151934	0,074947556	0,940256439	0,983128178
Mphosph6	84,17462395	-0,012694753	0,177714823	-0,071433279	0,943052924	0,983437952
Iah1	2629,89471	0,139811893	0,113849464	1,228041729	0,219431304	0,574443934
Dph2	101,9746343	0,15121157	0,194780626	0,776317303	0,437561659	0,766076718
Gcsh	2525,484337	0,14181922	0,085170804	1,665115445	0,095889726	0,385586553
Yeats4	489,6916286	0,197361513	0,091059916	2,167380798	0,03020583	0,20087196
Atg101	809,321427	0,108195967	0,120833998	0,895409971	0,370567973	0,718718007
Txndc17	1311,027491	0,038350554	0,101388848	0,378252193	0,705243256	0,901980015
Cdca8	23,11035228	-0,744476587	0,327858813	-2,270723123	0,023163744	0,171946751
Yif1a	1149,586399	0,029772435	0,087041681	0,34204803	0,732314747	0,912649428
Mybphl	0,087021394	-0,517475177	3,352475198	-0,154356154	0,877328924	NA
Dcakd	643,8437318	-0,33620527	0,111934316	-3,003594278	0,002668109	0,045497379
Pdcd2l	189,6317447	0,126506594	0,137551357	0,919704444	0,35772723	0,707149983
Mbtd1	275,441681	0,177641351	0,155527842	1,142183604	0,25337771	0,612785071
Mmp15	1192,194827	-0,63560333	0,172812616	-3,677991488	0,000235078	0,008493933
Mir6981	0,113662148	0,780932884	3,352475198	0,232942181	0,815806304	NA
Sult2a1	521,611762	12,15643237	3,225999095	3,768268996	NA	NA
Mpz	4,537640561	-0,02624055	0,83201258	-0,031538645	0,974839973	NA
Mpp1	823,5012226	-0,150462637	0,099225361	-1,516372796	0,129425114	0,446986398
Ccl2	17,33298753	0,951963838	0,493241898	1,930014139	0,053605086	0,279612327
Adgrg3	103,7609303	-0,112529224	0,236530225	-0,47574987	0,634252587	0,868071544
Endog	756,6255765	-0,090787925	0,127885039	-0,70991826	0,477754826	0,790092068
Enc1	112,5062296	0,677991244	0,240106898	2,823705809	0,004747194	0,066454995
Emp2	284,8257658	0,708971415	0,227909007	3,110765235	0,001866032	0,036579158
Atpif1	440,5527208	0,263513836	0,155705591	1,692385186	0,090572555	0,373377888
Pdcd1	0,507208407	1,467019232	2,09428414	0,700487199	0,483623097	NA
Cfb	34071,78527	-0,224829803	0,091659904	-2,452869713	0,014172166	0,129557379
H2-T24	59,91631954	-0,248872831	0,309155243	-0,805009251	0,420814341	0,754542239
Gzmk	0,180429807	0,059593471	3,352475198	0,017775962	0,985817581	NA
Mir5107	1,221096321	-1,182253577	1,423284567	-0,830651582	0,406170486	NA
Gm10649	6,730614573	-0,573113906	0,711376202	-0,8056411	0,42044982	0,75436738
Zfp975	98,28315561	-0,041384534	0,18532862	-0,223303526	0,823299283	0,947466482
Mthfsl	301,189011	0,297843066	0,140499859	2,119881599	0,034016032	0,215686483
0610038B21Rik	0,502844492	-0,777699405	2,133680668	-0,364487253	0,715494191	NA
Mphosph10	296,7766541	0,175059801	0,230972105	0,757926164	0,448495186	0,77297141
Atp2b1	790,1797451	-0,268185843	0,146438595	-1,831387706	0,067042695	0,317311846
Tppp3	9,204610043	-0,562262347	0,583075523	-0,964304493	0,334893289	0,691360575
Zcchc10	68,37268845	-0,155851388	0,215724267	-0,722456449	0,470013892	0,784465484
Ergic2	1146,049722	-0,155717688	0,103442075	-1,505361216	0,132231235	0,452933481
Abhd16a	876,7125115	0,049147806	0,108326781	0,453699493	0,650045132	0,876831587
3110035E14Rik	4,214529259	0,189444126	0,813483403	0,232880136	0,815854483	NA
Wipi2	1537,010758	-0,044226433	0,083465562	-0,52987642	0,596197616	0,853075785
Gpr153	24,50943919	0,439177198	0,40603881	1,081613844	0,27942415	0,640209334
Atp1a2	11,96481014	0,292707491	0,592566337	0,493965777	0,621330349	0,862654966
Zc3h6	53,4851385	0,571709017	0,489537951	1,167854332	0,242865528	0,601014405

Supplementary Table S1: *Serpina1* KO vs. wildtype all DEGs

Tspan3	78,24278966	0,106068032	0,187015173	0,567162709	0,570603641	0,8413217
Ano9	3,327691609	2,439297833	1,084135379	2,249993757	0,024449342	NA
Lhfpl1	0,234199685	1,337107854	3,350237755	0,399108348	0,689813372	NA
Klf11	184,3215577	-0,372760859	0,250659417	-1,487120906	0,136982881	0,459785121
Cdc23	323,9740875	0,210123492	0,107839361	1,948486066	0,05135683	0,27322396
Tbce	514,9111414	-0,091754075	0,127436795	-0,719996728	0,47152701	0,786015536
Slc25a31	1,187172382	0,171476124	1,441517501	0,118955284	0,905310782	NA
Mysm1	249,7334351	-0,059390849	0,148328906	-0,400399697	0,688862147	0,894264943
Hmgxb3	344,8785299	-0,13572662	0,120171032	-1,129445403	0,258709989	0,619837606
Acsl1	30969,95724	-0,327590705	0,159565599	-2,053015854	0,04007104	0,235476806
Dffb	60,79129235	-0,071461627	0,215597526	-0,331458474	0,740298203	0,916128178
Dhcr7	3958,144847	0,620956796	0,145274942	4,274355833	1,92E-05	0,001334746
Ddx46	903,3278461	0,151460649	0,105985467	1,429069987	0,152984119	0,487543429
Mir425	2,697746886	-1,14093626	1,085829427	-1,050750911	0,293373007	NA
Rimkla	0,113662148	0,780932884	3,352475198	0,232942181	0,815806304	NA
Mir697	0,518076125	0,416568915	2,441692841	0,1706066	0,864533108	NA
Apol11a	0,086476712	0,780932884	3,352475198	0,232942181	0,815806304	NA
Gml	0,086476712	0,780932884	3,352475198	0,232942181	0,815806304	NA
Plekhd1	1,865321758	0,713670028	1,093789255	0,652474894	0,514094864	NA
Wisp3	8,965671208	-1,281496874	0,830852998	-1,542387014	0,122979575	0,435526873
Ltb4r1	11,63219259	-0,423018291	0,45598887	-0,927694335	0,353566148	0,704458903
Rps8	6172,982564	0,064732568	0,129326875	0,500534545	0,616698738	0,86061017
Rhd	0,260519499	-0,589751774	3,345560051	-0,176278938	0,860074808	NA
Ndufs4	1522,941074	0,063109589	0,103235782	0,611315072	0,540991015	0,826407188
Ube2l3	2003,774856	0,056567564	0,090189585	0,627207271	0,530523378	0,819570954
Slc20a2	1909,410498	0,174213599	0,084144434	2,07041144	0,038413831	0,229984108
Skp1a	3979,747695	-0,033907212	0,069975666	-0,484557196	0,627990483	0,865852703
Srebf1	7566,836496	-0,114607183	0,166602867	-0,687906428	0,491511708	0,797760449
Ndufa4	6666,966153	0,063892642	0,108064159	0,591247298	0,554354736	0,832018326
Ndufa2	1436,810783	0,155075196	0,140215285	1,105979254	0,268735499	0,629281048
Ctsb	16246,73017	0,145439481	0,09649373	1,507242806	0,131748432	0,451742699
Cebpg	1396,038827	-0,086328407	0,091712321	-0,941295632	0,346553381	0,698497639
Aknad1	0,261385121	1,389394708	3,349408814	0,414817893	0,67827522	NA
Dennd2c	13,68008254	0,102660707	0,427716655	0,240020363	0,810314471	0,942417935
E130311K13Rik	146,1748548	0,435632732	0,161079916	2,704450952	0,006841737	0,083591067
Gcfc2	87,55971827	0,0921878	0,180491478	0,510759845	0,60951923	0,857753956
Mir7081	0,086476712	0,780932884	3,352475198	0,232942181	0,815806304	NA
Kmt5c	405,9883085	-0,277697504	0,116164595	-2,390551997	0,016823067	0,143473504
Itga11	5,340714292	-0,303466598	0,653018977	-0,464713291	0,642136789	NA
Slc22a30	3355,781043	-0,682211847	0,130562123	-5,225189587	1,74E-07	3,33E-05
Pik3c2b	164,1760569	0,016635833	0,180941747	0,09194027	0,926745496	0,978888597
Pla2g2c	0,12663974	0,780932884	3,352475198	0,232942181	0,815806304	NA
Pla2r1	59,50017236	0,049669736	0,212326573	0,233930852	0,815038661	0,944076707
A630020A06	1,792708155	0,17011038	1,179983505	0,144163355	0,885371477	NA
4930509E16Rik	6,140089365	0,763186446	0,577584659	1,321341268	0,1863876	0,532805747
1700060C16Rik	0,087021394	-0,517475177	3,352475198	-0,154356154	0,877328924	NA
Gm10638	2,049579309	-1,407445583	1,24253304	-1,132722864	0,257330653	NA
1600010M07Rik	5,388163685	0,861891194	0,6952051	1,239765349	0,215062198	0,568551815
Pkm	374,1610638	0,15879781	0,195402175	0,812671662	0,416406333	0,751673636
Cyp2b10	185,2136873	1,652124109	0,95738731	1,725659084	0,084408763	0,359726093
Pnpla1	0,443773723	1,189879927	2,509339132	0,474180597	0,635371129	NA
Zfp455	27,70163762	-0,222813041	0,379989636	-0,5863661	0,55762952	0,834166841
Tbc1d14	426,7838569	-0,274244954	0,126410942	-2,169471648	0,030046895	0,200147949
Phip	244,3582744	-0,014408429	0,131140392	-0,109870259	0,91251227	0,975960612
Sh3d21	27,25994635	0,465051774	0,275725617	1,686646962	0,091671256	0,375827372
Mcur1	879,3593992	-0,136799931	0,107943294	-1,267331452	0,205036799	0,558049056
Pparg	317,9102666	0,130323537	0,228578612	0,570147559	0,568577621	0,840388525
Ryr3	0,355338216	0,663614712	2,635698453	0,251779452	0,801211541	NA
Cdc25b	22,63399453	0,138321902	0,353693595	0,391078334	0,695739335	0,897068317
Abi1	821,4236602	0,021827699	0,100566424	0,217047581	0,828171264	0,94762353
Clint1	1680,749601	-0,16215551	0,125818679	-1,288803149	0,197466531	0,547135762
Tmod2	0,34331814	-0,671915911	2,654828049	-0,253092064	0,800197071	NA
Lrch2	1,526787681	1,257814044	1,270375217	0,990112234	0,322119264	NA
Itga2b	59,01432878	0,006190744	0,225515595	0,027451511	0,978099614	0,994429459
Mir6986	0,093953095	-0,517475177	3,352475198	-0,154356154	0,877328924	NA
Scara5	104,1392434	1,67117945	0,854937106	1,95473964	0,050613809	0,270337998

Zfp960	46,45571357	0,02692907	0,225259073	0,119547105	0,904841923	0,973666426
BC051142	1,533213589	0,788247367	1,227200657	0,642313352	0,520669747	NA
Prss36	159,779188	0,10073053	0,167174039	0,602548881	0,546808836	0,828173482
Mup16	0,560893709	0,578830709	2,449820287	0,236274763	0,813219468	NA
Magoh	454,3806365	-0,018182595	0,142305537	-0,127771519	0,89832979	0,971699969
H2-T-ps	7,863615159	0,601541278	0,535802452	1,122692284	0,261568206	0,622343615
Eif1a	1592,393652	0,060969066	0,157583649	0,386899698	0,698830465	0,898938178
Sag	1,346627029	0,55200034	1,404002291	0,393161993	0,694199839	NA
Cyp2d9	29940,40175	-0,778929956	0,663043353	-1,174779827	0,240082813	0,597592092
Gm10069	87,39049046	-0,052656687	1,346123272	-0,039117284	0,968796881	0,991555135
Gm14005	13,4798818	0,21867296	0,430020536	0,508517481	0,611090483	0,858645358
Gm10825	0,142634598	-0,517475177	3,352475198	-0,154356154	0,877328924	NA
Gm10451	6,228123008	0,447162556	0,648541608	0,689489387	0,49051535	0,797288719
Hdac10	244,575033	0,135043229	0,124688218	1,083047225	0,278787462	0,639188747
Gnpat	1199,699907	0,118205084	0,099678813	1,185859666	0,235677724	0,593010215
Alox12e	0,20013886	1,337107854	3,350237755	0,399108348	0,689813372	NA
Cox19	551,0844541	-0,126440026	0,125343205	-1,008750545	0,313094285	0,672219008
Kif18b	4,995182577	-1,48665151	1,08360153	-1,371954052	0,170077742	NA
Cyp2j6	1976,870549	0,17785411	0,100564284	1,768561399	0,076967097	0,342991041
Tgfbr1	494,839945	-0,109286619	0,145283171	-0,752231786	0,451911681	0,774656859
Hapln1	19,76489524	-0,459118956	0,534161707	-0,859513046	0,390057526	0,733963887
Ercc5	358,1012376	0,078062886	0,097488681	0,800737946	0,42328337	0,756028774
Xpa	289,0893506	0,305357618	0,122483773	2,493045497	0,012665262	0,121248489
Psmb9	360,3261241	0,402090256	0,20128078	1,997658474	0,045753699	0,253809628
Grcc10	1632,857451	-0,110193331	0,109891766	-1,002744198	0,315984299	0,67419376
Vars	836,6925308	-0,182682633	0,094042647	-1,942550953	0,052070438	0,275125315
Xirp1	13,72966099	0,575686741	0,532337872	1,081431119	0,279505385	0,640243137
Zfp39	147,0187175	0,016310497	0,15299372	0,106608929	0,915099235	0,976451566
Itgb1bp2	1,679049785	-0,407190832	1,149007711	-0,354384769	0,723050548	NA
Ccl4	2,554615906	0,963550983	1,064670017	0,905023123	0,365453138	NA
Gla	146,5124456	0,30288896	0,137602566	2,201186865	0,027722798	0,191736205
Adrb3	140,6145489	0,068165714	0,287801988	0,23684935	0,812773665	0,943554283
Adra1d	0,274277864	-1,699264605	2,746395376	-0,61872541	0,536097268	NA
Nt5dc2	127,0834947	-0,549042437	0,261056889	-2,103152457	0,035452445	0,220038873
Manba	548,1519469	-0,272910826	0,128760412	-2,119524331	0,03404618	0,215686483
Sergef	74,02586147	0,08015397	0,195773632	0,409421683	0,68223023	0,891829074
Jak1	2628,441878	-0,142526876	0,076059477	-1,873887137	0,060945983	0,298973723
Rasal1	5,814517175	0,690003045	0,667807711	1,033236117	0,301493415	0,661958526
Pou3f1	0,113662148	0,780932884	3,352475198	0,232942181	0,815806304	NA
Aff2	0,22911131	0,059593471	3,352475198	0,017775962	0,985817581	NA
Ccnb2	16,59586916	-0,746540663	0,399156605	-1,870295151	0,061442843	0,300339803
Rai1	226,822603	-0,066907969	0,156624432	-0,427187306	0,669242906	0,886730269
Grin2d	2,547801188	-0,597595679	1,064760289	-0,56124903	0,574627783	NA
Grin2b	0,122496332	0,780932884	3,352475198	0,232942181	0,815806304	NA
Inpp5b	929,9951786	-0,080913652	0,098918475	-0,817983216	0,413366771	0,750040766
Mir7085	1,037041053	2,549598434	1,614876269	1,57881968	0,114377423	NA
Kcnt1	0,460245003	1,234802867	2,493894437	0,495130367	0,6205081	NA
Fabp1	113443,3299	-0,63993978	0,201817376	-3,170885441	0,001519751	0,031647268
Stk39	47,67270097	0,128931395	0,27434775	0,469956089	0,63838639	0,870609778
Fkbp1b	3,139306922	-0,019585798	0,828910451	-0,023628364	0,981149047	NA
Vti1a	287,1035437	0,217586832	0,1310475	1,660366145	0,096840816	0,387044806
Pdlim1	772,0736649	-0,037290599	0,136487416	-0,273216387	0,784686884	0,933246486
Bysl	452,0979666	-0,143761252	0,17871987	-0,804394344	0,421169267	0,754607148
Actr1a	1332,64935	-0,119630583	0,092565413	-1,292389657	0,196222237	0,545184577
Sdhaf4	246,2687694	0,085205712	0,138063396	0,617149181	0,537136332	0,824035032
Cspp1	329,3609362	-0,260032261	0,130743864	-1,988867797	0,046715797	0,257129914
Rab14	3425,631881	0,021570839	0,086365417	0,249762457	0,802771054	0,940105661
0610030E20Rik	988,0319174	-0,049551796	0,115307339	-0,429736707	0,667387179	0,885763843
Etfb	6357,785286	0,044951636	0,091954771	0,488845069	0,624951387	0,864383696
Ndufa8	2598,582829	0,081525314	0,129576402	0,629167913	0,529239133	0,818910651
Pbld1	4453,460717	0,155061637	0,115785127	1,33921896	0,180499402	0,523789126
Mul1	935,9662343	0,143878797	0,092052234	1,563012554	0,118049642	0,42677276
Phospho1	460,0752125	-0,057820313	0,24279377	-0,238145787	0,811768024	0,942944131
Kif1c	5018,825641	-0,088444816	0,09560845	-0,92507321	0,354927827	0,705239841
Mrgpra2b	0,12663974	0,780932884	3,352475198	0,232942181	0,815806304	NA
Ctdsp1	3497,007582	-0,039468762	0,107098688	-0,36852704	0,712480287	0,905518486

Supplementary Table S1: *Serpina1* KO vs. wildtype all DEGs

Gtf3c5	209,9634163	-0,043132195	0,150438141	-0,286710505	0,774333994	0,929186892
Gm1943	12,21509519	-0,853172968	0,813132204	-1,049242625	0,294066465	0,65521643
Pgd	1655,437563	0,479030479	0,160848134	2,978153779	0,002899904	0,048005373
Insig2	6650,532174	0,134269842	0,158351579	0,847922344	0,39648122	0,73782815
Adam12	7,506983631	-0,023889768	0,634433289	-0,037655288	0,969962526	0,991981655
2010111I01Rik	392,6108319	0,166582032	0,130351255	1,27794728	0,201268016	0,552402402
Arhgap9	58,26255337	0,187028171	0,288721807	0,647779859	0,517127344	0,813424627
Pars2	81,64699767	0,141190642	0,186664057	0,756389015	0,449415992	0,773349269
Gls2	12151,12116	0,313845038	0,214612062	1,46238303	0,143636277	0,470584709
Hist1h1e	7,359431088	0,591306453	0,548457478	1,078126339	0,280977379	0,641764538
Gart	900,7888059	-0,094296053	0,112997395	-0,834497583	0,404000649	0,744258602
Fv1	37,88433401	0,339348509	0,260365358	1,303355071	0,192453568	0,541552905
Mir16-1	0,562352465	1,583400458	2,357035635	0,671776207	0,501726178	NA
Zfp970	1165,969955	-0,395015371	0,286002916	-1,381158543	0,167230218	0,506158205
Hmgcs1	15813,42173	1,230111587	0,222484063	5,528987416	3,22E-08	9,79E-06
Snord45b	5,381513943	-0,660288068	0,839833785	-0,786212796	0,431742831	0,7622164
Rpusd2	84,70807758	0,271187079	0,198657493	1,365098667	0,172222041	0,513592788
Pp2d1	0,296539119	-0,589942906	3,316285543	-0,177892675	0,858807264	NA
Ephb1	1,017624561	1,717371091	1,471067827	1,167431616	0,243036109	NA
Rccd1	68,42630616	0,189014296	0,254557811	0,742520119	0,457772267	0,778822589
Nbeal1	206,382265	-0,494724036	0,233448519	-2,119199719	0,034073592	0,215686483
Hmgcll1	0,686807592	1,925880628	1,794941089	1,072949212	0,283293904	NA
Arsj	0,093953095	-0,517475177	3,352475198	-0,154356154	0,877328924	NA
Coq8a	16994,57821	0,476876807	0,144094625	3,309469781	0,000934729	0,022354895
Susd1	163,7882896	0,125938156	0,136760539	0,92086619	0,357120292	0,707149983
Nptx1	2,849635717	0,251100852	0,973445686	0,257950552	0,796445069	NA
Rpl5	4191,600146	0,013222468	0,112870814	0,117146916	0,906743632	0,974457847
Mtor	951,8492016	0,006034185	0,117339423	0,051425046	0,958986827	0,987896001
Kat2a	581,3980675	-0,14589665	0,088252241	-1,653177845	0,098294674	0,390573569
Glmp	1362,544758	-0,042940867	0,090412052	-0,474946272	0,634825269	0,868410828
Rec8	46,51150536	-0,540416447	0,45502238	-1,187670037	0,234963433	0,59257237
Pigb	152,6085709	-0,262812733	0,156737663	-1,676768218	0,093587815	0,380194927
Epb41l4b	2244,364846	-0,249470975	0,107395007	-2,322928988	0,020182968	0,158753836
Taf2	400,3268726	-0,125634456	0,130939052	-0,959488054	0,337312935	0,69270916
Sh3d19	1893,999819	-0,057330801	0,104949836	-0,546268605	0,584881314	0,84725087
Vmn2r96	0,086476712	0,780932884	3,352475198	0,232942181	0,815806304	NA
Cyp2d11	12,12314922	-0,32132617	0,509644912	-0,630490293	0,528373851	0,81852028
2200002D01Rik	218,5722115	0,092828438	0,230003991	0,4035949	0,686510636	0,893161227
Ndufab1	1165,266426	0,068604545	0,093749231	0,731787815	0,464298087	0,781663619
Cda	271,8510438	0,531032057	0,209679465	2,532589723	0,011322339	0,113783449
Ndufs7	1774,827699	-0,035262418	0,133907115	-0,26333491	0,792292455	0,936597818
Mrpl32	533,1838895	0,018528178	0,091569942	0,20233908	0,839651651	0,951377482
Mcm9	87,75740088	-0,011308379	0,213056319	-0,053076949	0,957670597	0,98781716
Emc7	1245,756108	-0,048287004	0,097026232	-0,497669576	0,618716955	0,861717572
Calhm2	31,37228895	0,281169787	0,337695031	0,832614522	0,405062166	0,744879793
Ccdc71	494,4572302	0,045699415	0,084622334	0,540039641	0,589169695	0,849321722
Snrk	942,9226875	-0,354131304	0,139142216	-2,545103228	0,010924544	0,111159545
Prmt3	198,9196257	-0,254004939	0,174539534	-1,455285988	0,145590104	0,474401806
Mir6990	1,092930885	-0,006692503	1,347500657	-0,004966605	0,996037239	NA
Hilpda	58,19859917	0,073913098	0,225945217	0,327128401	0,743570772	0,917386273
Mcl1	2510,799256	0,178000969	0,075969994	2,343043067	0,019127175	0,154084887
Mcm4	133,3533014	-0,343018153	0,261378163	-1,312344343	0,189403977	0,536698128
Mcm2	115,3280498	-0,395230489	0,261625291	-1,510673863	0,130871565	0,449709746
Fosl2	193,112593	-0,008895654	0,17789969	-0,050003764	0,960119389	0,988284271
Grhl2	6,14228269	0,570011059	0,573027946	0,994735183	0,319865078	0,677483559
Coq5	711,8604377	-0,161933996	0,115808752	-1,398288066	0,16202658	0,498749809
Ppcs	523,0993636	-0,116026574	0,126006677	-0,92079703	0,357156406	0,707149983
Pmpcb	3468,721278	-0,031836413	0,06651535	-0,478632572	0,632200042	0,867306747
Mcee	1282,723774	0,105569418	0,091104783	1,158769214	0,246550269	0,605683673
Mvb12a	1112,609979	0,011691124	0,091530073	0,12772987	0,898362751	0,971699969
Ndufaf7	433,9714009	0,130289939	0,130510432	0,998310532	0,318128802	0,675759967
Slc18a1	92,33117177	0,082966081	0,215101476	0,385706701	0,699713899	0,899209374
Lce6a	4,59838967	0,740226103	0,724080712	1,022297779	0,306639984	NA
Ccdc74a	0,215799707	0,059593471	3,352475198	0,017775962	0,985817581	NA
Osbpl5	49,36394393	-0,098214764	0,29600223	-0,331804136	0,740037161	0,916128178
9330133O14Rik	25,77332504	0,259692985	0,315557864	0,822964705	0,410528065	0,748139272

Supplementary Table S1: *Serpina1* KO vs. wildtype all DEGs

Otud1	113,6653076	-0,12293277	0,203029135	-0,605493247	0,54485131	0,827491596
Fpgs	5128,107179	0,096458183	0,078211764	1,233295067	0,217465698	0,571290807
Zfp94	36,52408252	0,133600888	0,294068663	0,454318686	0,649599469	0,87669042
Klf5	2,160416117	0,856276836	1,069652235	0,800518906	0,423410214	NA
Bmp10	13,1335576	-0,335726451	0,44737887	-0,750429834	0,452995868	0,775627787
Bet1	1502,048411	-0,30179747	0,139908572	-2,157104925	0,03099749	0,204100361
Gm5796	0,093303375	-0,517475177	3,352475198	-0,154356154	0,877328924	NA
Zswim5	91,47179497	-0,186088024	0,222242159	-0,837320989	0,402412164	0,742560142
Mir6991	0,286615571	1,706362568	3,314438116	0,514827101	0,606673845	NA
Tmem236	0,174042788	-1,166820872	3,345560292	-0,348766954	0,727264274	NA
Arg1	34474,92424	0,202023578	0,140243897	1,440516002	0,149721466	0,481899934
Arf6	1077,16813	0,076719258	0,145736905	0,526422997	0,598594341	0,854045336
Arl4a	902,3501081	-0,246104656	0,101814553	-2,41718545	0,015641046	0,13729819
Fscn1	77,92263346	-0,274474086	0,28642739	-0,958267596	0,337927839	0,693194655
Irak3	29,24748529	0,954629451	0,375990416	2,538972833	0,011117846	0,112687246
Pdzd11	504,842038	-0,138499651	0,108168473	-1,280406827	0,200402094	0,551270699
Ppme1	577,1369598	-0,328974883	0,096620304	-3,40482143	0,000662073	0,017773611
Fbxo30	242,1591686	0,089039805	0,205760974	0,432734174	0,6652079	0,884715918
1600002H07Rik	1377,907419	-0,248745244	0,114865709	-2,165530908	0,030347051	0,201544822
Crocc	49,10242335	-0,179972714	0,272337743	-0,660843817	0,50871248	0,80781294
Bst1	8,209389279	1,28805797	0,626613274	2,055586795	0,039822362	0,234944905
Sox13	215,4072792	0,006890703	0,134291022	0,051311715	0,959077133	0,987896001
Ccndbp1	343,5302154	-0,092162787	0,11412917	-0,80753051	0,419360904	0,753719022
Bnip3l	1393,579431	-0,023663857	0,123080367	-0,192263458	0,84753584	0,953880403
Scel	0,113662148	0,780932884	3,352475198	0,232942181	0,815806304	NA
Slc30a5	1004,067629	-0,184786222	0,106740128	-1,73117858	0,083419919	0,357449003
Gpx7	43,78474508	0,460821825	0,276821092	1,664691886	0,095974243	0,385586553
Fam120b	507,8186031	-0,179105542	0,098866282	-1,811593776	0,070048987	0,32382454
Arpc1b	786,5018121	-0,041228599	0,142582408	-0,289156282	0,772461785	0,928273961
Ubr4	3157,529005	-0,253520913	0,101402404	-2,50014697	0,012414179	0,119917026
Rhox5	3,837549276	-0,263115496	0,763560903	-0,344590058	0,730402581	NA
Il2rb	33,67066635	0,339409406	0,354827564	0,956547461	0,338795718	0,694071514
Il2ra	2,339828264	0,966545465	1,011230099	0,955811606	0,339167424	NA
Myo1f	74,96336303	0,238565135	0,247330814	0,964558887	0,3347658	0,691301202
Tldc2	0,213661134	0,059593471	3,352475198	0,017775962	0,985817581	NA
Tbxa2r	26,21704802	-0,636600428	0,349470023	-1,821616696	0,068513166	0,320263116
Fhdc1	5,472756422	0,067909998	0,616018171	0,110240252	0,91221884	0,975948598
Micall1	338,1950557	-0,176747245	0,115377053	-1,53190986	0,12554468	0,441147786
Parp16	636,1625944	0,047855302	0,090230193	0,530369052	0,595856079	0,853075785
Lrrc29	87,9699991	0,277979478	0,232871189	1,193704896	0,232593425	0,589477023
Slfnl1	0,227324296	1,389394708	3,349408814	0,414817893	0,67827522	NA
BC049762	7,547949882	-0,394620188	0,573843571	-0,687679027	0,49165493	0,797760449
Acaa2	18476,96702	-0,131875447	0,101919133	-1,293922379	0,195692234	0,5447032
Prkaa1	259,7521021	-0,021027148	0,150797149	-0,139439954	0,889102502	0,968705223
Ofd1	97,80358137	0,118726828	0,159000663	0,7467065	0,455240745	0,777281421
Tmcc1	959,5718684	-0,310239311	0,140261914	-2,211857099	0,02697654	0,189715606
Rab5b	1857,141637	0,022417908	0,105460755	0,212571095	0,831661519	0,949486153
Esco2	14,84835111	-1,86208481	0,561257236	-3,317702992	0,000907609	0,021941826
2310022A10Rik	142,1787135	-0,097254626	0,131930137	-0,737167631	0,461020415	0,781332773
Uqcc3	598,9554303	0,225935353	0,120599828	1,873430144	0,061009011	0,299094046
Sult6b2	0,087021394	-0,517475177	3,352475198	-0,154356154	0,877328924	NA
Rnf144a	202,8794249	0,707684054	0,313252306	2,259150341	0,023874035	0,174984632
Bmp7	64,35142907	-0,005572832	0,28815985	-0,019339377	0,984570372	0,995554769
Mdh2	5779,288437	0,079653878	0,088986329	0,895124895	0,370720327	0,71880494
Mir1930	0,093303375	-0,517475177	3,352475198	-0,154356154	0,877328924	NA
Cdh19	0,487843795	0,427018354	2,473856998	0,172612384	0,862956117	NA
Dot1l	431,1116384	-0,179049528	0,097001052	-1,845851407	0,064913784	0,310605719
Espl1	10,8968788	-0,716417909	0,462870603	-1,547771459	0,12167733	0,433672149
H2-D1	5863,111082	0,364163436	0,121723617	2,991723747	0,002774072	0,046885669
Rest	148,9185355	0,102549076	0,217468604	0,471558075	0,637242258	0,869936868
Dusp13	0,716505626	-0,647743941	1,622623846	-0,399195379	0,689749248	NA
Stard4	2784,977471	0,004888853	0,114661029	0,042637439	0,965990551	0,990271183
Ugt1a2	1,119267629	-0,241864961	1,866129675	-0,129607799	0,896876732	NA
Thbs3	14,52072138	-0,415953094	0,411846585	-1,009970969	0,312509199	0,672017932
Pnp	2466,845953	0,235369692	0,130028075	1,810145171	0,070273278	0,324561524
Ninj1	3358,313007	0,040859798	0,072082204	0,56685001	0,57081609	0,8413217

Supplementary Table S1: *Serpina1* KO vs. wildtype all DEGs

Axin2	223,1406002	0,900973194	0,214580992	4,198755844	2,68E-05	0,001708593
Pkd1	472,5092489	-0,291464942	0,119797368	-2,43298284	0,014975011	0,133824142
Pam	440,7597807	-0,147619058	0,17828216	-0,828008019	0,407665959	0,746679655
Otog	2,972884218	4,985226514	3,238837837	1,539202259	0,123754926	NA
Snord1c	0,241676068	0,059593471	3,352475198	0,017775962	0,985817581	NA
Hibch	2027,819182	0,270650797	0,12228607	2,213259428	0,026879765	0,189333814
Dyrk4	0,220592835	0,059593471	3,352475198	0,017775962	0,985817581	NA
Dnase2b	825,9321162	-0,039737719	0,152294227	-0,260927284	0,794148584	0,936984593
Mbd3l2	0,172953423	1,307385949	3,350719078	0,39018071	0,696402925	NA
Mmel1	0,093303375	-0,517475177	3,352475198	-0,154356154	0,877328924	NA
Zfp146	478,5582831	0,153310098	0,159740024	0,959747555	0,337182283	0,69270916
Slc27a2	24777,40091	-0,168925335	0,119009151	-1,419431478	0,155773261	0,490700453
Slc27a1	134,7251939	0,234508387	0,205337909	1,142060849	0,253428728	0,612785071
Sema4g	11291,27232	-0,156960262	0,102688084	-1,528514858	0,126384757	0,442197807
Rpl27a	4389,006331	0,051285829	0,135765173	0,377753943	0,705613391	0,902021937
Galnt3	8,839232898	0,550801211	0,530736122	1,03780615	0,299360313	0,659904651
Psmb3	1638,141661	0,096882979	0,11221604	0,863361234	0,387938887	0,731971786
Psmb2	2208,281478	0,065824541	0,079275729	0,830324011	0,406355616	0,745871812
Psma5	1474,739353	0,099868754	0,104373826	0,956837146	0,33864946	0,694071514
Psma4	3398,465955	0,14248162	0,0755182	1,886718957	0,059198137	0,294098806
Psma1	2625,986148	0,151069413	0,090729825	1,665046881	0,095903403	0,385586553
Pde6g	10,30297721	0,222684481	0,494772026	0,450074922	0,652656419	0,877662367
Coil	96,49328647	0,142377031	0,17634663	0,807370292	0,419453177	0,753719022
Tbp	219,4729467	-0,003481036	0,132527808	-0,026266458	0,979044808	0,994593468
Mir6993	1,130457933	0,090319483	1,616575575	0,05587087	0,955444677	NA
Gm13889	57,01287633	0,099006711	0,259611339	0,381365128	0,702932333	0,900661526
Cyp2j8	4,295356675	0,006112275	0,774163482	0,007895329	0,993700505	NA
Rps26	4289,227884	-0,014746699	0,090107789	-0,16365621	0,870001792	0,961775826
Tbl2	426,2371881	-0,471265611	0,128110088	-3,678598769	0,000234519	0,008493933
Rpl3	5781,687059	-0,057320501	0,116847213	-0,490559418	0,623738095	0,863666879
Mir7090	0,12663974	0,780932884	3,352475198	0,232942181	0,815806304	NA
Galnt16	0,808474425	2,214320598	1,927114792	1,149034094	0,250541923	NA
Chil1	12,77570289	3,442654598	0,77605971	4,436069227	9,16E-06	0,000765512
Neurl1b	15,06582079	-0,887670086	0,494235546	-1,796046627	0,072487102	0,330736787
Chrna7	1,496472292	-3,40316323	1,509348896	-2,254722708	0,024150739	NA
Ifi207	140,0996145	0,175132859	0,228921211	0,765035524	0,444250392	0,770322112
Trmt2b	717,0854664	0,01974852	0,083980333	0,235156488	0,814087277	0,944076707
St6galnac3	19,48019055	0,550325606	0,48909845	1,125183704	0,260511197	0,621964694
1300017J02Rik	13587,54927	-0,098830038	0,081877496	-1,207047629	0,227413802	0,582859866
Rassf8	322,9116928	-0,715785791	0,213399354	-3,354207864	0,000795925	0,020384536
Fsip1	2,183751744	1,860457721	1,318982723	1,410524709	0,158384806	NA
Snrnp200	1683,813131	-0,094490029	0,113839931	-0,83002535	0,406524451	0,745871812
Arntl	1021,48324	0,065196341	0,093330221	0,698555525	0,484829846	0,79420977
Chaf1a	41,72859011	-0,625776835	0,397192953	-1,575498331	0,115141463	0,420121786
Tctn1	77,62061081	0,103657215	0,226930947	0,456778664	0,647830143	0,876425944
Tpgs2	211,9519842	0,019645394	0,127908155	0,153589844	0,877933145	0,965046146
Chid1	1101,097326	-0,380253918	0,138650492	-2,742535664	0,006096683	0,077728349
Rsph4a	13,56021756	5,211222667	1,622109338	3,212621089	0,001315297	0,028669412
Inhbb	19,18184526	0,861408071	0,459334038	1,875341254	0,060745791	0,29867984
Nodal	6,278250001	-0,043333387	0,583770367	-0,074230193	0,940827222	0,983128178
Ovca2	179,9105897	0,077709151	0,157627458	0,492992477	0,622017903	0,863017086
Sec24d	2222,255335	-0,56019672	0,170058121	-3,294148589	0,000987203	0,023507922
Mtfmt	228,586836	0,126102339	0,131363301	0,959951051	0,337079851	0,69270916
Gpr39	214,2199976	0,347925045	0,178793161	1,94596394	0,051659071	0,273663551
Prss41	0,420081975	1,124593069	2,207967604	0,509334044	0,610518099	NA
Ly6g6e	2,71550394	2,182561801	1,071648193	2,03664021	0,04168611	NA
Chp2	53,96823405	0,075395286	0,254725995	0,295985836	0,767240898	0,927501905
Icos	23,52055465	-0,288511647	0,392461583	-0,735133475	0,462258209	0,781516914
Dnpep	1254,588916	-0,198808774	0,081287704	-2,445742256	0,01445543	0,13080163
Slc26a6	81,11423419	-0,18252567	0,253903036	-0,71887943	0,472215212	0,786770335
Mylpf	25,28197846	0,051253457	0,335261157	0,152876215	0,878495893	0,965046146
Lxn	19,44174417	-0,176711191	0,451664923	-0,391244	0,695616888	0,897068317
Slc22a14	0,407104383	1,08374679	2,548423831	0,425261598	0,670645985	NA
Cyp4a12a	6488,958988	-1,005411441	0,943544464	-1,065568693	0,286618658	0,648131311
Cited4	10,75179732	1,369345255	0,572617977	2,391376641	0,016785324	0,143473504
Scarb2	3913,391944	-0,321200404	0,083330099	-3,854554453	0,000115941	0,005055664

Gap43	0,086476712	0,780932884	3,352475198	0,232942181	0,815806304	NA
Ipp	246,190794	-0,135768124	0,119184804	-1,139139544	0,254644956	0,61494733
Acvr1b	224,6096849	-0,071571736	0,192966738	-0,370901932	0,710710576	0,904386988
Adcy6	483,9273916	-0,114741011	0,108769003	-1,054905426	0,29146858	0,653216736
Tmem29	181,8872152	0,242693713	0,207320546	1,170620651	0,241751293	0,599735207
Dap	6391,433453	0,292045939	0,068210859	4,281516795	1,86E-05	0,001332808
Zdhhc14	122,1604873	-0,336478532	0,150056735	-2,242342081	0,024939272	0,180613829
Sf3b4	342,6511049	-0,185084966	0,143303332	-1,291560798	0,196509287	0,545500968
Lins1	104,4114384	-0,312779804	0,158932873	-1,967999438	0,049068102	0,265261408
Rbm17	1062,333461	0,322468565	0,091901254	3,508859231	0,000450033	0,013642838
Lsm4	503,3909323	0,064242075	0,12394456	0,518312984	0,604239915	0,855575199
Dkk3	27,00320834	0,193583382	0,34234996	0,565454665	0,571764551	0,841407954
Pcdhga4	2,815719126	-0,2657523	1,064764803	-0,249587796	0,802906137	NA
Pcdhga2	1,320015281	0,387946195	1,268118409	0,30592269	0,759663509	NA
Pcdhga1	2,48865187	0,061875046	0,886715562	0,069780038	0,944368735	NA
Rgs1	19,3990928	0,713684788	0,547665329	1,30314035	0,19252685	0,541552905
Fbxo15	0,482601187	-0,575616405	2,13257714	-0,269915866	0,787224981	NA
Sec24b	557,4017251	0,052861338	0,086562304	0,610673879	0,541415502	0,826407188
Slc38a9	185,8344643	-0,057590741	0,145476749	-0,395875914	0,69219657	0,895338108
Mrps26	464,308862	0,047645152	0,119101393	0,400038578	0,689128103	0,894327388
Cebpe	130,3282396	-0,121487507	0,197116244	-0,616324178	0,537680584	0,82443534
Ninl	21,26057067	0,394888132	0,335209476	1,178033917	0,238783085	0,596132785
Prr36	33,01112687	0,129114077	0,279553179	0,461858734	0,644182628	0,873967684
Ccdc120	39,0408762	0,460655476	0,2874466	1,602577577	0,109027948	0,40910239
Tes	36,35623737	-0,544577176	0,263505893	-2,066660329	0,038766172	0,230874245
Tmem132e	39,63795541	-0,326748616	0,37124909	-0,88013311	0,378787204	0,724671742
Dnph1	162,9662224	-0,00433366	0,16610293	-0,026090209	0,979185387	0,994660697
Rnf216	463,7885225	-0,012215693	0,105586073	-0,115694165	0,907894931	0,9747592
H2-Ab1	697,3132079	0,522056738	0,223331459	2,337587101	0,019408678	0,155304678
Gpr152	0,093953095	-0,517475177	3,352475198	-0,154356154	0,877328924	NA
Rapgef2	315,0883454	-0,182113403	0,107464793	-1,694633177	0,090145029	0,372419313
Sox7	34,89359905	-0,008140188	0,377590409	-0,021558249	0,982800339	0,995413661
Col9a3	6,525939095	0,199118748	0,713173313	0,279201064	0,780090527	0,931606698
Ndufaf3	273,8698061	0,023127889	0,14841329	0,155834354	0,876163591	0,9643065
Palmd	1134,619933	0,154590216	0,135173164	1,14364576	0,252770576	0,612079303
Diablo	531,6563873	-0,088577228	0,084135885	-1,052787728	0,292438291	0,654074594
Stoml2	1278,681982	-0,055421309	0,081829535	-0,677277581	0,498229852	0,801990802
Ube2v1	380,7877875	-0,06595503	0,108694818	-0,606790936	0,543989664	0,827477215
0610009B22Rik	448,9184514	-0,08801123	0,113685568	-0,774163611	0,438834045	0,766497562
Ciao1	1083,189865	0,017051261	0,075149476	0,226897932	0,820503111	0,946609086
Lcmt1	267,9985925	-0,020119295	0,123422793	-0,163011181	0,870509631	0,961775826
Tril	9,298213567	-0,111528875	0,509375377	-0,218952231	0,826687259	0,947537943
Tnfaip8l1	584,8111579	-0,2896136	0,153971952	-1,880950373	0,05997867	0,296324774
Mtmr2	650,486191	-0,027737166	0,091468536	-0,303242703	0,761704908	0,92534041
Pgpep1	1521,217471	0,029097307	0,099895646	0,291277028	0,770839455	0,927992136
Slc30a7	560,5593493	-0,172779678	0,090752512	-1,903855603	0,056928993	0,288295377
Usmg5	2369,947179	0,129450516	0,101129428	1,280047938	0,200528277	0,551390896
Dctpp1	506,5331607	-0,011096821	0,131531922	-0,084365988	0,932765449	0,981257162
Bpifb2	0,142634598	-0,517475177	3,352475198	-0,154356154	0,877328924	NA
Jazf1	29,72420613	-0,362817232	0,540791895	-0,670899907	0,502284296	0,804113015
Ercc2	351,020601	-0,010970804	0,115335973	-0,095120405	0,92421919	0,977951927
Kri1	273,5304344	-0,044464398	0,120800577	-0,368081011	0,712812829	0,905682828
Rtcb	1610,378345	0,018078644	0,082196906	0,219943116	0,825915457	0,947537943
Phf11a	6,518704963	0,250196988	0,584150964	0,428308782	0,668426326	0,886438999
BC029214	899,9823784	-0,017112409	0,19499584	-0,087757814	0,930069168	0,980359737
Abhd3	1526,994196	0,012102565	0,116737223	0,10367357	0,917428402	0,976907366
Rab2b	61,05021524	-0,039574996	0,234479292	-0,168778212	0,865971097	0,961775826
Lhfpl2	161,7012971	-0,327539246	0,247199165	-1,325001422	0,18517068	0,531803827
Noc4l	294,7282768	-0,195767922	0,159717216	-1,225715839	0,220305628	0,574490867
Serinc5	829,5760193	-0,069561699	0,104570182	-0,665215437	0,505912708	0,80595177
Mirlet7c-1	0,172953423	1,307385949	3,350719078	0,39018071	0,696402925	NA
Npc2	2153,709808	0,250001012	0,111746371	2,237218174	0,025272084	0,181959388
Aaed1	689,3305393	0,119009739	0,124718654	0,954225656	0,339969431	0,694369844
Mesdc2	800,9301758	-0,291075996	0,128998086	-2,256436545	0,024043305	0,175935931
Kdelc1	276,9624167	-0,240233253	0,128346197	-1,871759805	0,061239841	0,300005658
Aadac	13761,00995	-0,103496168	0,126158593	-0,820365587	0,412007727	0,749099865

Hdac11	1547,463837	-0,099387627	0,109352848	-0,90887095	0,363418249	0,712361223
Klhl22	372,4938768	0,047817057	0,133240932	0,35887663	0,719687383	0,908631658
Tmem150a	4142,422333	-0,15458102	0,132515715	-1,166510853	0,243407963	0,601946182
Daglb	610,7501893	-0,004928156	0,158523784	-0,031087805	0,975199515	0,993185764
Mepce	557,0759924	0,030722165	0,100104513	0,306900904	0,758918801	0,924631864
Rhov	0,932627068	1,50728271	1,592653668	0,946397036	0,343946117	NA
Lrrc3	2624,876877	-0,43246068	0,10840048	-3,989471995	6,62E-05	0,003282365
Eif3m	1367,949693	0,001811073	0,086300646	0,020985621	0,983257126	0,995413661
Gckr	3777,991371	0,289402664	0,138696605	2,086587941	0,036925398	0,225266506
Adamtsl4	69,60836642	0,050261357	0,211116613	0,238073909	0,811823771	0,942944131
Rhoq	197,5407666	-0,01586788	0,217015904	-0,073118512	0,941711811	0,983128178
Galnt11	304,4592846	0,047765229	0,124099349	0,384895085	0,700315147	0,899463273
Rnf26	296,8440207	-0,257862947	0,108808885	-2,369870305	0,017794326	0,147933805
Stard5	2340,62305	0,074025484	0,137236324	0,539401533	0,589609824	0,84953676
Sdhb	5933,60899	0,243193844	0,09542569	2,548515444	0,01081825	0,110412601
Pin1	401,2126437	-0,241344859	0,103394638	-2,334210595	0,019584697	0,156018012
Zfp788	58,89149925	0,258682094	0,262276111	0,986296821	0,323987479	0,681035945
Snap29	776,6016031	-0,085087516	0,091410003	-0,930833757	0,351939565	0,702880198
Ilkap	749,6851472	-0,106554953	0,103684433	-1,027685156	0,304097952	0,663427795
Il4ra	957,9458934	-0,047915094	0,088137041	-0,5436431	0,586687095	0,848304107
Crip1	76,48443432	0,697717292	0,229547677	3,039531052	0,002369468	0,042463022
Rsu1	642,5307825	0,057366543	0,114960416	0,499011265	0,617771448	0,861267197
Klf2	161,628482	0,349009447	0,208840932	1,671173572	0,094687399	0,38267105
Napsa	12,43151607	0,93226095	0,510949509	1,824565704	0,068066593	0,319375503
Dvl1	1877,001679	-0,117767684	0,085376033	-1,379399809	0,167771518	0,506611452
Gjb2	6757,451928	0,065025475	0,076338297	0,85180673	0,39432137	0,73637054
Kcnj8	96,31745947	0,115452065	0,211055614	0,547022003	0,584363616	0,847068435
Pik3ca	819,8115753	-0,085985084	0,095125247	-0,90391443	0,366040777	0,714472885
Map4k1	19,71251255	0,062899118	0,319855882	0,196648307	0,844102765	0,953068489
Atn1	651,1826255	-0,278751131	0,110356423	-2,525916686	0,011539684	0,115044027
Drd4	0,25327948	1,389394708	3,349408814	0,414817893	0,67827522	NA
Lilr4b	39,21888495	1,106557222	0,374322995	2,956156145	0,003114993	0,050477997
Dpagt1	865,7098302	-0,273850676	0,076066369	-3,600154425	0,000318028	0,010498073
Lpar3	0,811504917	0,508168744	2,264727989	0,224384009	0,822458513	NA
Ipo5	1201,114347	-0,097746702	0,090299336	-1,082474212	0,279041868	0,639552672
Zfp110	765,4380063	0,007339282	0,075692504	0,096961805	0,922756727	0,977445106
Igf2bp3	8,141341363	-1,388913653	0,93117894	-1,491564719	0,135813293	0,458391294
Sdha	13110,24254	-0,064117842	0,090869173	-0,705606088	0,480433138	0,792267252
Prrg2	586,4909929	-0,140432364	0,109719016	-1,279927298	0,200570706	0,551394153
Vps35	2061,403876	0,009454312	0,111825685	0,084545082	0,93262306	0,981243003
Gpn3	277,4435738	0,036209898	0,118024258	0,306800471	0,75899525	0,924640741
Rsrp1	5050,753068	0,400146254	0,194585591	2,056402285	0,039743756	0,234688285
Rgl3	780,3461682	-0,278920289	0,095909792	-2,908152375	0,003635711	0,056318411
Arl6ip5	871,8688271	0,103618546	0,107394371	0,964841497	0,334624208	0,691222347
Arl6ip6	170,4844703	-0,001547435	0,152114117	-0,010172854	0,991883377	0,997479229
Mir7115	3,641045467	0,491738434	0,74210706	0,662624655	0,507570976	NA
Nucb2	175,7883255	-0,949295403	0,180329246	-5,264234302	1,41E-07	2,87E-05
Kctd1	21,68697955	-0,150816526	0,381279506	-0,395553717	0,692434286	0,895338108
Aplp1	18,39661713	0,246845557	0,371237891	0,664925546	0,506098116	0,805958988
Ddx28	310,9851573	-0,061516566	0,105154393	-0,58501185	0,558539749	0,83440808
Rnf180	6,493450684	-0,320343621	0,681008899	-0,470395646	0,638072375	0,870273408
Gm10677	1,732739846	0,330168007	1,722711741	0,19165598	0,84801169	NA
Thbs1	21,55625018	-0,058263642	0,556710755	-0,104656936	0,916648034	0,976559041
Ets2	507,4464094	0,075785754	0,146804447	0,516236094	0,605689524	0,856421285
Cdc6	8,46646315	-0,895892478	0,614209793	-1,458609889	0,14467251	0,472725517
Rpl11	5686,682697	0,081944375	0,115044629	0,712283362	0,476289325	0,789577814
Hacd4	19,93879304	0,038791834	0,384028432	0,101012922	0,919540202	0,977134804
Rras2	363,4989658	0,129939688	0,108075822	1,202301177	0,229246861	0,585109066
Rgcc	1,414629024	0,681708938	1,516963737	0,449390398	0,653150057	NA
Pacrgl	85,15292942	0,045976393	0,171126709	0,268668713	0,788184631	0,934897572
Adat2	65,98781851	0,082748293	0,215989854	0,383111945	0,701636763	0,900078003
Uqcc2	409,9596263	0,101259337	0,12181119	0,831281075	0,405814863	0,745391028
Tmem88	60,52533843	-0,467883462	0,344892288	-1,356607494	0,174905959	0,517287416
Actr6	214,8471832	-0,051492169	0,154626138	-0,333010768	0,739126155	0,915635905
Erich2	0,249136072	1,389394708	3,349408814	0,414817893	0,67827522	NA
Erp27	1,266547417	1,506937275	1,606320693	0,938129778	0,34817772	NA

Supplementary Table S1: *Serpina1* KO vs. wildtype all DEGs

Fhit	105,6871969	0,160887032	0,210929942	0,762751036	0,445611888	0,771326444
Eef1g	3609,422296	-0,183660345	0,122736581	-1,49637821	0,134555125	0,456327747
Lrrk2	36,16053764	-0,220988104	0,282991407	-0,780900404	0,434861074	0,764552565
Mfsd3	239,6227509	-0,022710704	0,135553594	-0,167540405	0,866944859	0,961775826
Klhdc2	718,046129	0,178428483	0,091642576	1,947004226	0,051534228	0,273331789
Ccdc115	274,1094098	0,049552242	0,141674154	0,349762044	0,726517287	0,910393262
Tsr1	453,3983073	0,165951974	0,205594979	0,807179121	0,419563293	0,753773001
Arhgef26	462,8044722	0,6764713	0,140165964	4,826216575	1,39E-06	0,000171184
Dmap1	253,8344983	0,161753621	0,119854755	1,349580342	0,177150633	0,520079413
Nuf2	10,42433412	-1,977905485	0,713848526	-2,770763564	0,005592502	0,073372088
Atf7	534,957639	-0,053909151	0,131148006	-0,411055821	0,681031604	0,89166825
Mfsd1	1682,294435	-0,067475722	0,111002325	-0,607876657	0,543269283	0,82684051
Hmg20a	312,3911221	-0,008086098	0,132036781	-0,061241252	0,951167077	0,985972877
Nhlrc2	285,9847961	0,077519711	0,172157053	0,450284837	0,652505071	0,877662367
Phlpp2	83,26166828	-0,347064637	0,18885187	-1,837761188	0,066097603	0,314805435
4930405A21Rik	3,6051569	0,303888736	0,769064127	0,395140958	0,692738863	NA
4930443O20Rik	0,200683542	0,059593471	3,352475198	0,017775962	0,985817581	NA
Trim34b	0,287160254	0,617200912	2,785784868	0,221553688	0,824661338	NA
Afp	25,43292534	0,568802372	0,500845108	1,135685191	0,256088341	0,616650761
Bin2	92,67194945	-0,277962062	0,247817334	-1,121640915	0,262015148	0,622562722
Lrr1	0,673209209	-2,064412925	1,729726673	-1,193490831	0,232677201	NA
AY074887	1,580068419	0,848625197	1,555749012	0,545476931	0,585425542	NA
Cdh15	6,162922734	-1,373480415	0,753547432	-1,822686081	0,06835095	0,319740014
Tcf24	175,2872611	-0,687091362	0,991571002	-0,692932086	0,488352173	0,796400974
Kif16b	261,0594284	-0,100490297	0,164475397	-0,610974645	0,541216366	0,826407188
Klra5	0,340986444	1,884176617	3,333184127	0,565278288	0,571884494	NA
Rab33b	191,9257191	0,084150811	0,13711424	0,613727727	0,539395263	0,826211649
1700037C18Rik	5,989512917	0,288845951	0,552865113	0,522452845	0,601355071	0,854422974
Ccdc18	3,950283603	-1,003938399	0,881955096	-1,138310106	0,254991016	NA
Adgra2	111,178725	-0,17155383	0,234967696	-0,730116664	0,465318876	0,781663619
Ciart	8,700042371	-0,028212981	0,494108698	-0,057098733	0,954466545	0,986643721
Cpsf2	670,2873689	-0,176698492	0,126604781	-1,395669978	0,162813899	0,499921685
Abhd10	130,3374758	0,032934693	0,179561039	0,183417813	0,8544702	0,957135493
Fcgr1	103,2109739	0,223159452	0,285119427	0,782687641	0,433810562	0,763709955
Fcer1g	359,760896	0,365621562	0,245373323	1,490062398	0,13620783	0,459143338
Stil	6,27959526	-0,514140392	0,632253482	-0,813187127	0,416110779	0,751551608
Klrd1	5,77234372	0,005409566	0,823801644	0,006566588	0,994760659	0,998569999
Fcer1a	0,093953095	-0,517475177	3,352475198	-0,154356154	0,877328924	NA
St8sia3	17,20352762	-0,118438799	0,654739032	-0,180894667	0,856450255	0,9578959
St8sia2	1,281436307	-0,582076369	1,255813503	-0,463505423	0,643002129	NA
Il5	0,23615848	1,389394708	3,349408814	0,414817893	0,67827522	NA
Il1a	67,61078727	0,168972882	0,285213737	0,592442999	0,55355398	0,831886596
St3gal2	51,06798389	-0,233126042	0,249714567	-0,933570054	0,350525718	0,702050628
St6galnac2	20,45204029	0,223177651	0,382098948	0,584083397	0,559164204	0,83506102
St3gal4	1344,631574	0,178008278	0,118739677	1,499147391	0,133835399	0,455858715
Aes	12152,48792	-0,04921319	0,090685576	-0,542679356	0,58735059	0,848572815
Fah	18181,09077	0,304213291	0,083166292	3,657891734	0,000254298	0,00894662
Fadd	242,7867284	0,208930714	0,122755799	1,702002805	0,088754842	0,369300805
Fabp3	0,086476712	0,780932884	3,352475198	0,232942181	0,815806304	NA
Siah2	443,469568	0,135331137	0,166910492	0,810800659	0,417480156	0,752195715
C8b	13457,79935	-0,787572995	0,215781882	-3,649856923	0,000262386	0,009196845
Faah	3972,355828	-0,02247218	0,066598002	-0,337430235	0,735792595	0,914312906
Tcea3	1953,475501	0,056643362	0,100944491	0,561133758	0,574706357	0,84338631
Mdm2	553,1379174	0,004039125	0,113109152	0,035709973	0,971513618	0,99222044
Nat8f1	4804,040248	0,175806898	0,145450904	1,208702686	0,22677709	0,581916106
Cxcl16	206,7124314	0,285054089	0,200058671	1,424852459	0,154199854	0,489157561
Ethe1	3547,60759	0,582931321	0,141899299	4,108063421	3,99E-05	0,002269832
Cwc15	1414,45938	-0,073969328	0,07818659	-0,94606156	0,344117189	0,697257153
Mina	437,7590767	0,165834424	0,111689301	1,484783432	0,137601202	0,460640235
Mum1	196,7252931	0,097130731	0,145950546	0,665504406	0,505727926	0,805849422
Tollip	1139,824966	-0,074108602	0,087211268	-0,84975949	0,395458816	0,737426273
Capn9	0,997191049	1,699689447	1,568798457	1,08343391	0,278615871	NA
Gm20199	0,77750626	1,259350194	2,22053538	0,56713809	0,570620366	NA
H2-M3	59,30278156	0,048299608	0,220269451	0,219275108	0,826435751	0,947537943
Insl6	3,550850897	0,23486805	0,869120395	0,270236496	0,786978317	NA
Pald1	278,3709626	-0,090360234	0,12619493	-0,716036961	0,473968527	0,787826089

Supplementary Table S1: *Serpina1* KO vs. wildtype all DEGs

Nbn	426,9237545	0,01193196	0,137218572	0,086955865	0,930706594	0,980582903
Hrasls	0,443773723	1,189879927	2,509339132	0,474180597	0,635371129	NA
Mos	0,093953095	-0,517475177	3,352475198	-0,154356154	0,877328924	NA
Ccl28	18,38441669	-3,778173808	1,535496294	-2,460555472	NA	NA
Tnfrsf10b	14,14382429	-0,179886328	0,473904631	-0,379583393	0,70425469	0,901232981
Rab3b	9,265047624	-0,488736919	0,616640094	-0,792580508	0,428022269	0,75995385
Ddit3	149,6584243	-0,533642738	0,302762537	-1,762578502	0,077971606	0,345738771
Hook3	1251,947139	-0,129115074	0,112619991	-1,146466748	0,251602078	0,611020561
A530032D15Rik	0,086476712	0,780932884	3,352475198	0,232942181	0,815806304	NA
Prdm6	1,962992857	1,319292918	1,041832964	1,266319039	0,205398883	NA
Csta1	0,093953095	-0,517475177	3,352475198	-0,154356154	0,877328924	NA
Ugt3a1	1034,173363	-0,123701444	0,180209302	-0,686432068	0,492440688	0,798476599
Snord49a	2,544241456	0,538959406	1,020352138	0,528209219	0,597354131	NA
Exo1	4,344898466	-1,267287021	0,806647722	-1,571053865	0,116170138	NA
Fam219b	208,0935229	0,131057866	0,141290198	0,927579322	0,353625828	0,704458903
Ccr9	3,828833928	0,724163733	0,875368722	0,827267087	0,408085694	NA
Fmr1nb	6,644292612	-4,528799701	1,06688242	-4,244891112	2,19E-05	0,001491732
Themis	2,753133876	0,090419241	0,903325939	0,10009592	0,920268174	NA
Zfp972	7,417099462	-0,2739283	0,537937709	-0,509219367	0,610598469	0,858645358
Cast	1014,661276	-0,094560994	0,107260479	-0,881601447	0,377992372	0,723903192
Syn3	48,03811993	-0,226922326	0,280145158	-0,81001695	0,417930434	0,752384753
Mnt	286,9062671	-0,347033611	0,143986069	-2,410188806	0,015944267	0,138594866
Ndst2	573,2031859	-0,049838146	0,107201155	-0,464903073	0,64200087	0,872335566
Mmp9	15,89586558	0,031473619	0,505836334	0,062220953	0,950386877	0,985972877
Ptcra	0,579905834	-1,793734642	2,011159983	-0,891890579	0,372451585	NA
Minpp1	698,7682449	0,20891462	0,11741592	1,779269973	0,075195511	0,338937556
Zfpm2	15,72638214	-0,202816786	0,406150527	-0,499363592	0,617523263	0,86111508
Nfkbib	632,8187719	0,006106448	0,08324493	0,073355199	0,941523467	0,983128178
Mif	3075,496524	0,045156201	0,146214115	0,308836128	0,757446186	0,923804952
Nfkbia	703,14507	0,435820528	0,198659334	2,193808465	0,028249182	0,193375993
Ptpn1	597,5610596	-0,134606032	0,086175894	-1,561991708	0,118289942	0,427187007
Marcksl1	295,5851859	-0,096116103	0,193881405	-0,495746887	0,620073002	0,862258786
Rad52	132,1218061	0,027863456	0,157785878	0,176590305	0,85983021	0,959201442
Cish	653,320539	-1,283440301	0,920728761	-1,393939622	0,163335838	0,500604498
Zfp59	56,23064163	0,040337904	0,195433315	0,206402393	0,836476601	0,951141594
Omd	40,73941749	0,135378479	0,464385647	0,291521669	0,770652374	0,927934035
Tbc1d5	391,210998	0,113673837	0,126985131	0,895174387	0,370693874	0,71880494
Rassf7	519,4764395	0,014554805	0,112532742	0,129338405	0,897089884	0,971044462
Med19	406,4894655	-0,145587842	0,122446571	-1,188990763	0,234443302	0,591595413
Hmbox1	444,192749	-0,225034901	0,117936011	-1,908110159	0,05637698	0,287125274
Kdm4d	0,734627906	0,330695678	1,759745203	0,187922477	0,850937422	NA
Hist1h4f	0,18660675	-1,235117516	3,340448777	-0,369745983	0,711571767	NA
Mir7647	0,142634598	-0,517475177	3,352475198	-0,154356154	0,877328924	NA
Rgag1	0,180429807	0,059593471	3,352475198	0,017775962	0,985817581	NA
Tas2r137	0,093953095	-0,517475177	3,352475198	-0,154356154	0,877328924	NA
9530091C08Rik	0,610340127	0,859653519	2,054924452	0,41833826	0,67569982	NA
Cenpf	8,588862267	-1,712552955	0,550259619	-3,112263548	0,001856587	0,036447442
Pla2g12b	1314,54548	0,16758397	0,0774764	2,1630325	0,03053868	0,202340409
Zfp827	5,392119486	0,222738186	0,63229018	0,352272095	0,724634214	0,910252319
Map3k10	252,979311	-0,177941698	0,133616384	-1,331735625	0,182947078	0,528597253
Adam4	5,806039228	0,105431334	0,613376294	0,171886875	0,863526465	0,961272625
Mlph	30,70370298	0,477878764	0,338778591	1,410593161	0,158364609	0,494205524
Agpat3	4047,103784	0,080575738	0,113000903	0,713053932	0,475812385	0,789452019
Intu	108,1321737	-0,216769721	0,235534884	-0,92032958	0,357400556	0,707149983
Cdk20	217,6658168	0,038744097	0,167456389	0,231368279	0,817028707	0,944998904
Zfp91	1306,578969	-0,137058401	0,109132907	-1,25588519	0,209157599	0,561671273
Mir103-2	0,086476712	0,780932884	3,352475198	0,232942181	0,815806304	NA
Lman1l	0,147722973	0,780932884	3,352475198	0,232942181	0,815806304	NA
Ppp1r37	1687,959478	-0,061404074	0,10878218	-0,564468131	0,572435583	0,841794408
Tmem199	235,0100437	-0,250109078	0,201834874	-1,239176723	0,215280059	0,569015245
Rbfa	835,4893721	0,01148287	0,111127783	0,10333033	0,917700805	0,976907366
Serpina3g	102,0057919	0,601884447	0,291430965	2,065272807	0,038897195	0,231427058
Trim47	96,76529575	-0,471305789	0,297967846	-1,581733716	0,113710372	0,417177269
Spint1	20,7668015	0,712254697	0,455172365	1,564802155	0,117629306	0,425713642
Fgfr1	135,6409469	0,278417344	0,182327593	1,52701706	0,126756769	0,443035621
Rfng	379,7361836	-0,109715509	0,09792781	-1,120371309	0,262555566	0,622562722

Mad2l1bp	183,0104436	-0,267549423	0,233508423	-1,145780607	0,251885941	0,61148777
Uqcrh	5686,187588	0,041955942	0,1163902	0,360476582	0,718490766	0,90815359
Lage3	353,4982256	0,121162875	0,115511385	1,04892583	0,294212256	0,655325757
Acer3	287,6606345	0,029728134	0,166756119	0,17827312	0,858508487	0,958795917
Med11	246,3078819	0,039207792	0,147597722	0,265639551	0,790516823	0,935843529
Tab1	132,9320492	0,169536307	0,175080809	0,968331756	0,33287871	0,68995097
Pgls	1201,001957	4,35E-07	0,11271419	3,86E-06	0,999996918	0,999996918
Chchd5	331,5293989	0,030825046	0,118795039	0,259480917	0,795264204	0,937556185
Arl4d	894,2455336	0,642167345	0,519149611	1,236960081	0,216101903	0,569610871
Tmed10	2968,696386	-0,054766111	0,062136311	-0,881386575	0,378108622	0,723903192
Ogfod3	195,0834186	0,023465417	0,114796048	0,204409628	0,838033407	0,951141594
Mcoln2	6,600264189	0,038219914	0,588553272	0,06493875	0,948222768	0,985571619
Tmed5	982,1169161	-0,226600005	0,220437962	-1,027953636	0,303971638	0,663347176
Gjc3	22,78891705	-0,316014925	0,346535102	-0,911927602	0,36180683	0,71109038
Gpr137b	20,37430937	0,282159169	0,383606923	0,735542432	0,462009208	0,781516914
Cep41	74,56528623	-0,174652702	0,182627513	-0,956332919	0,338904064	0,694071514
Tmem2	208,226041	-0,224773614	0,234009232	-0,960533102	0,336786979	0,69270916
Csmd3	0,294091955	0,617264955	2,767052958	0,223076668	0,823475837	NA
Naip7	0,541220507	0,478915702	2,131177343	0,224718841	0,822198007	NA
Tnfaip3	61,80911551	0,18296572	0,307473955	0,595060872	0,551802783	0,830638744
Dok2	63,98718953	-0,009393502	0,296307643	-0,031701857	0,974709814	0,993185764
Tmem131	921,2839727	-0,096205308	0,09871196	-0,974606403	0,329755536	0,6866113
Smad3	420,2495894	-0,551597112	0,173696399	-3,175639317	0,001495067	0,031328452
Nupr1	40,82418673	-0,395563959	0,355107043	-1,113928791	0,265309737	0,626006345
Trim44	1347,293735	0,025958535	0,108791229	0,238608709	0,811409012	0,942886004
Diaph3	4,948011049	-1,422121307	0,80279617	-1,771460006	0,076484238	NA
Tubb2b	5,118360145	0,443355044	0,687484758	0,644894361	0,518995646	NA
Spns1	589,0987271	-0,212442619	0,104681435	-2,029420194	0,04241551	0,243203202
Slc31a2	198,5057896	-0,052419406	0,124321193	-0,421644971	0,673284176	0,888626355
Fuca1	1846,782923	0,138724738	0,072288655	1,919038861	0,054979416	0,28404688
Stambp	376,1845673	0,113853615	0,099275659	1,146843212	0,251446426	0,610832604
Txn2	5381,848177	0,070006083	0,079663778	0,878769316	0,379526366	0,725074694
Cxcl11	41,30327538	0,128734388	0,331688102	0,388118801	0,697928119	0,898426898
Srf	331,5040061	0,020982621	0,109417245	0,191767034	0,847924694	0,953880403
Efhd2	781,7737228	0,162683715	0,084290723	1,930031068	0,053602988	0,279612327
Cdca7	47,87988329	-0,109056835	0,34991257	-0,311668811	0,755292233	0,922462845
2310030G06Rik	375,8085581	-0,110830172	0,131685982	-0,841624676	0,399998072	0,740788928
Tmem206	71,1702786	0,010400943	0,289525806	0,035924064	0,971342908	0,992121884
3110040N11Rik	236,3740408	0,048702112	0,118870606	0,409706941	0,682020938	0,89172987
Acad8	1582,296637	0,07784049	0,070236535	1,108262096	0,26774864	0,628098188
Ddx18	373,8047947	-0,068790546	0,121308672	-0,567070307	0,570666416	0,8413217
Ptgr1	130,6753334	-0,217851873	0,183917479	-1,184508803	0,236211714	0,593481376
Spc24	314,9380013	0,103303349	0,14955104	0,690756477	0,489718591	0,796768631
Tm7sf3	1824,60673	0,15051206	0,103688313	1,451581722	0,146617944	0,476487281
Mxra7	83,25955184	-0,37107389	0,238835064	-1,553682628	0,120260139	0,431381217
Lrp2bp	31,04134199	0,043784455	0,320069477	0,13679672	0,891191476	0,968960544
Mto1	452,4120137	-0,040483226	0,103511947	-0,391097139	0,695725436	0,897068317
Aasdhppt	795,9994984	-0,104324968	0,122907962	-0,848805608	0,395989469	0,737426273
Rspry1	472,3142733	-0,004878034	0,111535518	-0,043735256	0,965115436	0,989920843
4930453N24Rik	461,0629802	0,112905716	0,10532882	1,071935644	0,283748939	0,645033084
Narf	757,1405813	-0,348040886	0,099249161	-3,506738822	0,000453634	0,013720894
Ift22	64,24717349	0,18651156	0,208474959	0,894647305	0,370975656	0,719091423
Rps6ka6	1,443535401	1,17831984	1,340386606	0,879089536	0,379352731	NA
Dusp6	773,8144553	-0,076754828	0,17917843	-0,428370918	0,668381095	0,886438999
Rcn3	149,7488818	-0,043579088	0,225601271	-0,193168626	0,846826909	0,953824301
Vps13c	730,1975643	-0,12393985	0,188254065	-0,658364799	0,510303749	0,808610978
Lmbrd2	388,8118199	-0,672699622	0,288890143	-2,328565503	0,019882095	0,157530566
Rab39b	0,32815278	0,617580015	2,680419673	0,230404224	0,817777679	NA
Pik3r5	20,84065783	0,3776144	0,430738934	0,876666513	0,380667801	0,725866642
Dyrk2	340,1536792	-0,276087551	0,24498974	-1,126935159	0,259769897	0,621485997
Wdr36	579,7257513	0,079469171	0,111396788	0,713388349	0,475605481	0,789452019
Mfge8	351,6687649	0,487014952	0,180624702	2,696281001	0,007011849	0,084455842
Met	1829,183092	-0,01893285	0,129645482	-0,146035555	0,883893321	0,966686011
Arhgef38	0,12663974	0,780932884	3,352475198	0,232942181	0,815806304	NA
Mtmr3	907,5878137	-0,103542749	0,09087047	-1,139454319	0,254513711	0,614741428
Pdilt	311,2392194	-0,827231021	0,274270384	-3,016115008	0,002560361	0,044338692

Col24a1	6,421718981	0,129172323	0,603345292	0,214093529	0,830474122	0,948778716
Brf1	345,018271	0,001479773	0,13572157	0,010903006	0,991300832	0,99719344
Wdr89	44,0506758	-0,515687534	0,282678522	-1,824289762	0,068108277	0,319375503
Tdrkh	126,9731468	-0,119317748	0,225144635	-0,529960431	0,596139366	0,853075785
Cyp2c65	0,747605498	0,364585378	1,980397946	0,184097029	0,853937337	NA
B3gnt3	34,96169202	-0,364818577	0,310882326	-1,173494105	0,24059772	0,59816966
Dgkd	377,1318893	-0,064556458	0,130562858	-0,494447344	0,620990285	0,862626101
Rasal2	219,7907603	-0,181948178	0,15750193	-1,155212369	0,248003471	0,607129663
3000002C10Rik	39,22775844	-0,263507838	0,258450855	-1,019566519	0,307934091	0,667059844
Ccdc42	0,18725647	-1,238536202	3,340198538	-0,370797181	0,710788601	NA
Armc7	69,89525899	0,000654195	0,196703932	0,003325786	0,997346412	0,999481197
D11Wsu47e	103,5599963	0,071636674	0,14915419	0,48028603	0,631024021	0,866751905
Smtnl2	16,51926169	0,598829943	0,580633561	1,031338839	0,302381951	0,6622779
Esyt3	1,590111253	1,672827291	1,598398084	1,046564875	0,295300309	NA
Srsf12	0,857021884	0,678857898	1,95138243	0,347885626	0,727926081	NA
4933408B17Rik	3,439058615	-0,415632481	0,880868721	-0,471843842	0,637038255	NA
Klhl18	223,3793529	0,049569594	0,131109099	0,378078976	0,705371926	0,902021937
Slc35e1	1252,51926	-0,370322497	0,093721365	-3,951313526	7,77E-05	0,003751207
Rpl29	3218,503771	-0,011269468	0,086834589	-0,129780865	0,896739802	0,971044462
Slc26a10	65,7814715	0,003400746	0,673293794	0,005050909	0,995969975	0,998991922
B3gat3	1015,75797	-0,113239844	0,093216946	-1,214798905	0,224442799	0,579668007
Sftpa1	14,43005206	-0,839997255	0,438339491	-1,916316628	0,055324806	0,284475895
Myl12b	2272,878534	0,081945466	0,067024329	1,222622704	0,221472234	0,57615534
Cmpk1	1793,286473	-0,112216327	0,107069147	-1,04807342	0,294604781	0,655663613
Ppp1r7	608,4891387	0,082682402	0,093093339	0,888166681	0,374451098	0,720969948
Mir6516	3,947160609	-0,036688745	0,767810957	-0,047783565	0,961888735	NA
B430319G15Rik	2,136512935	0,909957694	1,109520207	0,820136207	0,412138464	NA
Scn4b	1,033360024	0,595526347	1,923344137	0,309630677	0,756841825	NA
Gm7904	0,712038042	2,041902249	1,936262072	1,054558821	0,291627145	NA
Smarca5-ps	0,208973043	1,337107854	3,350237755	0,399108348	0,689813372	NA
Hist1h2bc	2839,398402	-0,050653757	0,100065306	-0,50620699	0,612711349	0,859036852
Cttnbp2	3,482070026	0,864464235	0,84903695	1,018170333	0,308597015	NA
Mrps21	806,2492769	-0,0072506	0,102499234	-0,070738089	0,943606206	0,983631296
Ftcd	10726,85045	-0,06626947	0,133973462	-0,494646247	0,620849851	0,862608071
Fubp1	775,7835962	-0,041595351	0,118169856	-0,35199629	0,724841044	0,910252319
Elovl6	3518,523489	0,725446637	0,409788414	1,770295628	0,076677906	0,342478335
Acsm1	11098,26803	0,0195167	0,098473706	0,198191996	0,84289485	0,952476217
Eps8l2	2998,578343	-0,247884446	0,143410365	-1,728497423	0,083899083	0,358468148
B3galt6	52,02810495	-0,155165939	0,297379031	-0,521778348	0,601824667	0,854462419
Celsr3	0,093953095	-0,517475177	3,352475198	-0,154356154	0,877328924	NA
4930579K19Rik	2,498017874	-0,2172513	1,110591406	-0,195617667	0,844909431	NA
Mettl7a3	0,087021394	-0,517475177	3,352475198	-0,154356154	0,877328924	NA
Rpl12	5228,376786	-0,021844575	0,153820797	-0,142013144	0,887069624	0,968354576
Rpia	141,0631752	0,056304722	0,213952541	0,263164542	0,792423759	0,936597818
Scn8a	11,82236168	1,414760545	0,558919208	2,531243381	0,011365895	0,113885033
Ncr1	2,881017027	1,514007581	0,916134094	1,652604778	0,098411325	NA
Cd3g	14,82447469	0,598210147	0,513704645	1,164502118	0,244220585	0,602840659
Stxbp3-ps	0,274277864	-1,699264605	2,746395376	-0,61872541	0,536097268	NA
3110039I08Rik	8,874193548	-0,255301356	0,637440459	-0,400510121	0,68878083	0,894264943
Rbp1	777,1544624	0,039364625	0,516105323	0,076272464	0,939202333	0,982918677
Tln1	2097,631602	-0,096541537	0,096409545	-1,00136908	0,316648407	0,674637563
Scgb1a1	0,093303375	-0,517475177	3,352475198	-0,154356154	0,877328924	NA
Uchl1	1,391278903	-0,314978315	1,531113673	-0,205718439	0,837010854	NA
Tyrobp	352,2735287	0,292405461	0,184527046	1,584621154	0,113052436	0,41624842
Zfp330	327,4811546	-0,104030468	0,106175944	-0,979793196	0,327188211	0,684930268
Prr14	1036,774308	-0,046676334	0,068409343	-0,682309337	0,49504337	0,80006558
BC017158	588,5778878	-0,071984441	0,121111765	-0,59436374	0,552268855	0,830773388
B3galt1	635,5050732	0,451596737	0,401805687	1,123918232	0,261047716	0,621983054
Gabarap	5313,188118	-0,053080499	0,066335622	-0,800180918	0,423605984	0,756445602
Zfp593	129,2470636	0,080845769	0,174722279	0,462710135	0,643572153	0,873795284
Samsn1	16,96806843	0,682818119	0,472143465	1,446208982	0,148118595	0,478308575
Pecr	5294,640516	0,208377198	0,12606206	1,652973129	0,098336332	0,390573569
Sbds	1461,129951	0,050720527	0,095786833	0,529514602	0,596448518	0,853075785
Mrps9	552,7810616	-0,011623865	0,095268933	-0,122011073	0,902890249	0,973489035
Ier3ip1	575,7531551	-0,024629476	0,09418825	-0,26149202	0,793713103	0,936746676
Uqcrc1	6393,527724	-0,002178384	0,091190971	-0,023888154	0,980941823	0,994882668

Ing2	242,2667835	0,129435463	0,119227152	1,085620692	0,277646841	0,638252527
Nat8f5	845,0983868	-1,352176298	0,822104985	-1,644773262	0,100016578	0,392993389
Hist1h4h	22,52323595	0,857713819	0,422157136	2,031740663	0,042179917	0,242539058
Shisa4	5,511005327	0,80257325	0,62042394	1,293588461	0,19580761	0,544880737
Kif14	2,283870209	-1,574810726	0,945302638	-1,665932859	0,095726788	NA
Mphosph9	58,27691629	-0,344878228	0,22267656	-1,548785499	0,121433293	0,433611562
Mir568	22,5855354	0,409952404	0,340946983	1,202393404	0,229211144	0,585109066
Dpm2	405,8195342	-0,084933885	0,103094338	-0,823846269	0,410026914	0,747429753
Ptn	0,388024861	2,013582874	2,565692911	0,784810553	0,432564652	NA
Rom1	54,0419257	-0,245967468	0,217625994	-1,130230185	0,258379244	0,619489259
Gm11266	68,18028546	0,798887356	0,270180973	2,956860162	0,00310789	0,050424012
Dpm1-adnp	0,087021394	-0,517475177	3,352475198	-0,154356154	0,877328924	NA
Rock2	519,163363	-0,106283576	0,122344831	-0,868721426	0,384999519	0,729665236
Ptma	3367,060464	-0,302837741	0,13924381	-2,17487399	0,029639561	0,198245973
Rock1	858,0514323	-0,023989324	0,133424021	-0,179797637	0,857311439	0,958260148
Unc5c	0,180429807	0,059593471	3,352475198	0,017775962	0,985817581	NA
Stra8	0,086476712	0,780932884	3,352475198	0,232942181	0,815806304	NA
Scg5	0,20013886	1,337107854	3,350237755	0,399108348	0,689813372	NA
Uggt1	2243,466346	-0,571442857	0,13715942	-4,166267675	3,10E-05	0,00189881
Zbtb12	79,79133393	-0,421447055	0,220953173	-1,907404399	0,05646824	0,287261759
Apod	0,147722973	0,780932884	3,352475198	0,232942181	0,815806304	NA
Plekhm2	306,5013788	0,210365995	0,122509233	1,717143997	0,085952865	0,363485758
Hcfc2	168,4604924	-0,313134044	0,17004585	-1,841468315	0,06555296	0,312991971
Specc1	71,73825177	0,01067538	0,211775606	0,050408923	0,959796526	0,988106124
Baiap3	6,138148154	0,480970104	0,658967847	0,729884025	0,465461078	0,781663619
Robo3	6,457717117	0,572516375	0,576859883	0,992470428	0,320968098	0,678403192
Zfp961	248,1145335	0,263377077	0,113025987	2,3302347	0,01979375	0,157046079
BC030336	277,4577089	-0,244016463	0,162159284	-1,5047949	0,132376815	0,452967915
Myo1h	0,774790934	0,428198454	1,976817124	0,216610049	0,828512254	NA
Acnat1	3949,534897	-0,416333846	0,147943589	-2,814139145	0,004890805	0,067826947
Mir192	1,928333615	-0,175238693	1,206043999	-0,145300414	0,884473688	NA
Elovl2	12710,6008	-0,410874323	0,174124642	-2,359656382	0,018291869	0,149935011
Lyz2	1679,656965	0,185530793	0,198402265	0,93512437	0,349724209	0,701600082
Hpx	140171,1501	0,234590452	0,219200553	1,070209219	0,284525146	0,645809283
Hp	80782,35268	0,385153211	0,167486348	2,299609587	0,021470348	0,163367723
Syt7	24,84305376	0,340016551	0,441728275	0,769741421	0,441453294	0,768930234
Socs5	127,4906638	-0,183807102	0,240420268	-0,764524154	0,444554949	0,770550383
Pebp1	1782,755362	0,101110334	0,124680309	0,810956716	0,417390528	0,752195715
Paxip1	465,8540471	-0,1160215	0,097328061	-1,192066279	0,233235258	0,590144372
Atp8a2	1,840465176	-0,517125492	1,108565579	-0,466481642	0,640870785	NA
Cmpk2	201,3664342	0,006775828	0,204421725	0,03314632	0,973557905	0,992775209
Ddx21	879,2045	0,048368695	0,150859343	0,320621143	0,748497513	0,919838327
Cib2	120,9886304	0,249449043	0,220674158	1,130395355	0,25830967	0,61943354
Col5a2	91,86189142	-0,161250508	0,337749987	-0,477425652	0,633059051	0,867527474
Chd1	700,8284515	0,187211636	0,105727426	1,770700778	0,076610473	0,342478335
Meig1	7,577050521	-1,381866343	0,618905059	-2,232759813	0,025564788	0,183454453
Nedd1	351,8028595	0,03899686	0,12725815	0,306438996	0,759270421	0,924882144
Ndrg1	704,3400341	0,208964604	0,240767002	0,867912141	0,385442432	0,729677128
Dock5	429,2445798	-0,259132116	0,123353638	-2,100725359	0,035665082	0,220786205
Mir7117	0,147722973	0,780932884	3,352475198	0,232942181	0,815806304	NA
1810041L15Rik	1,393419439	1,562402127	1,353332737	1,154484839	0,248301451	NA
Tacc3	35,36744565	-0,005224914	0,313848019	-0,016647911	0,986717502	0,996105587
Cd69	4,893451276	0,96079948	0,798191247	1,203720893	0,228697474	NA
Rnf17	1,091160525	0,04102846	1,62702736	0,025216823	0,979882019	NA
Scrib	632,2715368	-0,372207211	0,124202559	-2,996775712	0,002728514	0,046341149
Adi1	4233,978712	0,001284838	0,106651822	0,012047034	0,99038809	0,997175657
Ciapin1	567,8265508	0,14153235	0,15854326	0,892704932	0,372015209	0,719340958
Ptov1	942,8431523	-0,150163676	0,090418772	-1,660757736	0,096762113	0,386845898
Cxcl5	0,219943115	0,059593471	3,352475198	0,017775962	0,985817581	NA
Sar1a	1832,163294	-0,166010208	0,081005625	-2,049366428	0,040426297	0,236524801
Cd6	8,600050105	0,251601439	0,56704201	0,44370864	0,657253248	0,880359254
Ppox	491,366263	0,018011599	0,158225913	0,113834696	0,909368834	0,9747592
Cyp46a1	32,37452857	0,592695823	0,489087408	1,211840283	0,225573525	0,580946341
Gdap2	776,4974152	0,132597707	0,109544976	1,21044079	0,226109798	0,581158101
Eno1	12,38292756	-0,086352071	0,395327858	-0,218431535	0,827092897	0,947537943
Rassf10	2,60781839	0,738548856	0,977985031	0,755173988	0,450144594	NA

Mir24-2	0,179780087	0,059593471	3,352475198	0,017775962	0,985817581	NA
Serhl	556,4238227	0,269610521	0,093907138	2,871033312	0,004091324	0,059974684
Mphosph8	324,1570902	0,066085259	0,129756965	0,509300287	0,610541757	0,858645358
Tfcp2l1	156,0522346	-0,295129926	0,192186798	-1,535640994	0,124626454	0,439132771
Zfp84	199,775494	-0,099286801	0,153696784	-0,645991404	0,518284923	0,813725618
Rps15a	2829,649575	0,087523148	0,147419347	0,593701913	0,552711503	0,83087907
Creg2	0,333605263	0,663964464	2,676598865	0,248062746	0,804085859	NA
Disp2	74,72221855	-0,156934945	0,288835074	-0,543337563	0,586897406	0,848346794
Ntmt1	166,2668001	0,170577312	0,123630726	1,379732354	0,167669067	0,506611452
Nupl1	207,9877413	-0,197140629	0,157813597	-1,249199261	0,211592202	0,564073259
Cpne1	620,4346044	0,062533781	0,111711338	0,559780073	0,575629458	0,843861602
Arhgap15	15,88857571	0,559147523	0,378712762	1,476442251	0,139825207	0,464879971
Polr2b	1379,355302	-0,343436407	0,116072301	-2,958814496	0,003088249	0,050227259
Acsf2	5168,363996	-0,222554799	0,15337566	-1,451043786	0,146767668	0,476487281
Dusp12	396,3038782	0,168873337	0,098611788	1,712506595	0,08680335	0,365572746
Ndufb9	5237,434115	0,073534819	0,106245832	0,692119559	0,48886225	0,796400974
Grina	4050,158026	-0,0225881	0,107571388	-0,209982413	0,833681399	0,950655632
Mrpl4	975,3623626	0,010595643	0,10595063	0,100005476	0,920339978	0,977341679
Znrd1	239,3217916	0,092310197	0,131833048	0,700205283	0,483799113	0,794055997
Palld	1879,684705	-0,860466635	0,15657791	-5,495453567	3,90E-08	1,10E-05
Cgn	1329,869992	-0,244671873	0,103941469	-2,353938953	0,018575661	0,151643025
Stmn1-rs1	0,207615244	0,059593471	3,352475198	0,017775962	0,985817581	NA
4930428G15Rik	0,219943115	0,059593471	3,352475198	0,017775962	0,985817581	NA
Jmjd7	7,388413181	0,411446842	0,679960523	0,605104015	0,545109887	0,827491596
Spred1	240,1206194	0,172127525	0,206725294	0,83263892	0,405048402	0,744879793
Mcam	117,9225202	-0,147493378	0,294569318	-0,500708558	0,616576248	0,86052906
Fchsd2	530,1057968	-0,312880268	0,141965285	-2,203920973	0,027529902	0,191150756
Zbtb45	44,72859429	0,081169284	0,301863751	0,268893776	0,788011427	0,934775933
Lemd3	218,1003463	-0,069905036	0,153406016	-0,455686404	0,6486155	0,876446263
Tpbgl	122,6254535	-0,199136908	0,378535415	-0,526072066	0,598838137	0,854232506
4930430F08Rik	116,901294	-0,302477636	0,196721951	-1,537589647	0,124148981	0,437955618
Fbxo28	220,1257268	-0,073688631	0,114180181	-0,645371471	0,518686487	0,813725618
1110051M20Rik	40,98145438	0,487572381	0,341645825	1,427128169	0,153542954	0,488738507
Dusp28	172,4944309	0,065899347	0,155655494	0,423366661	0,672027765	0,887904019
Ikzf5	119,6279779	-0,22751363	0,177477556	-1,281929027	0,199867544	0,550139839
Trak1	1103,400893	-0,057945514	0,124344624	-0,466007388	0,641210212	0,871881542
Zfp689	44,72309865	-0,058963969	0,288992814	-0,204032648	0,838327986	0,951141594
Hspbap1	96,18569495	0,122542229	0,210443154	0,582305611	0,560360847	0,835469208
Rps19bp1	295,1378665	-0,114207962	0,130145519	-0,877540487	0,380193138	0,725803522
Tmem177	256,7243576	0,106109316	0,11958681	0,887299494	0,374917678	0,720996253
Commd2	306,4615358	0,242176941	0,10449603	2,317570732	0,020472662	0,159698791
Fam53c	400,0763578	-0,130113876	0,110366075	-1,178929997	0,238426052	0,595686393
Pdhx	766,0864556	-0,265623985	0,116992826	-2,270429683	0,023181525	0,171983247
Bola2	386,4652656	0,055980106	0,153380613	0,364975109	0,715129987	0,906371815
Smagp	849,9110991	-0,201622828	0,134184721	-1,502576644	0,13294825	0,453761846
Adgrb1	0,12663974	0,780932884	3,352475198	0,232942181	0,815806304	NA
Nhlrc1	32,08216567	0,20746944	0,319652876	0,649046062	0,516308604	0,813339545
Ddx58	816,6001961	0,183062649	0,143621045	1,27462273	0,202442808	0,554261295
Rell1	301,0662555	0,180853879	0,143194204	1,262997204	0,206590185	0,558976761
Srek1	405,0760661	-0,033947692	0,121877992	-0,278538328	0,780599147	0,931770536
Mast4	108,6053256	-0,288243243	0,203596536	-1,415757105	0,156846632	0,492560538
Zfp28	47,35595246	-0,027253415	0,243283337	-0,112023351	0,910804892	0,974815504
Celf6	0,113662148	0,780932884	3,352475198	0,232942181	0,815806304	NA
Wdr41	340,2551625	0,237643581	0,113807357	2,088121436	0,036786882	0,224876005
5830454E08Rik	16,09015242	0,378358157	0,385247484	0,982117139	0,326042143	0,683635102
Hsd17b12	4529,27348	-0,108107705	0,116699524	-0,926376578	0,354250312	0,704551502
2210010C04Rik	0,173498105	0,059593471	3,352475198	0,017775962	0,985817581	NA
Noct	185,2129472	0,191405206	0,617041474	0,310198283	0,756410176	0,923140802
Fam19a5	17,10541189	0,104261032	0,463011517	0,225180213	0,821839082	0,946779723
Klf15	2752,519284	-0,011140692	0,115335482	-0,096593797	0,923048983	0,977522327
Ctrl	5,110276582	0,252365432	0,671070382	0,376064029	0,706869295	NA
Atp6v1g2	49,9797963	-0,045348207	0,264248245	-0,171612142	0,863742461	0,961272625
Farsa	487,3350437	-0,201523995	0,12782058	-1,576616185	0,114883866	0,41975469
Nit2	4523,006959	0,130096727	0,0758273	1,715697746	0,086217378	0,364068263
E530011L22Rik	1,647381834	2,580385925	1,433524431	1,800029263	0,071856018	NA
Recql4	65,17182192	0,453383962	0,23389859	1,938378341	0,052577081	0,276515733

Ccdc117	544,5744654	-0,325798875	0,146327558	-2,226503872	0,02598045	0,185046689
Rpn1	4719,096453	-0,555380377	0,129858156	-4,276823238	1,90E-05	0,001333939
Tada3	305,4087822	0,003582919	0,099285557	0,036087008	0,971212982	0,992121884
Hoxb2	10,39696669	0,268472417	0,567067216	0,473440201	0,635899155	0,869346129
Pot1a	119,9188098	0,391881589	0,193313425	2,027182485	0,042643754	0,243203202
Psmg2	262,9595373	-0,079975762	0,140207267	-0,570410958	0,568398999	0,840292266
Tubg2	3,231413285	0,840708729	0,854378396	0,984000454	0,32511529	NA
Chchd4	308,2303902	-0,144035029	0,15796407	-0,911821458	0,361862712	0,71109038
Itfg2	212,7244706	-0,121201267	0,126034235	-0,961653528	0,336223673	0,69270916
Ube3b	1384,679807	0,028402179	0,081208367	0,349744493	0,72653046	0,910393262
Usp35	34,96099834	0,118159998	0,271070209	0,435901822	0,662907963	0,883542125
Zfp971	130,0249222	-0,337588753	0,155567687	-2,170044176	0,0300035	0,200058247
Mfsd7a	80,32931252	0,281812175	0,231996258	1,214727245	0,224470138	0,579668007
Styk1	1,269510118	0,436470793	1,267929202	0,344239089	0,730666488	NA
Siglecg	10,72485947	0,783895407	0,471766571	1,66161711	0,09658957	0,386394815
Slc24a4	0,093303375	-0,517475177	3,352475198	-0,154356154	0,877328924	NA
Elof1	774,6339217	-0,123480072	0,0946826	-1,304147455	0,192183312	0,541552905
Edc3	338,3066301	0,145721572	0,13004281	1,120566158	0,262472577	0,622562722
Fermt3	117,6911804	0,374835657	0,245393305	1,527489336	0,126639377	0,442946503
Zfp882	49,60000869	-0,016371019	0,254828556	-0,064243268	0,948776525	0,985650844
Fam196a	0,280559845	-1,715696706	3,310037588	-0,518331487	0,604227008	NA
4930526I15Rik	48,74941432	0,05570828	0,290639186	0,191675048	0,847996753	0,953880403
Psd4	89,75576987	0,856604861	0,280463906	3,054242782	0,002256294	0,041377771
Terc	0,636531402	0,841466826	1,715857678	0,490405957	0,623846662	NA
Pxmp2	3650,84229	0,452741865	0,076527521	5,916066001	3,30E-09	1,34E-06
Dennd4a	812,8468669	0,122149571	0,134504136	0,908147324	0,363800391	0,712726762
Trpv6	0,122496332	0,780932884	3,352475198	0,232942181	0,815806304	NA
Meox1	9,173088998	-1,159404434	0,659570654	-1,757816888	0,078778671	0,348162662
Nov	1,83299188	0,835558426	1,071444202	0,779843154	0,435483202	NA
Ube2ql1	2,050889307	0,134651591	1,262985609	0,106613718	0,915095435	NA
Dhx9	1586,910488	-0,20085168	0,115457273	-1,739619132	0,081925918	0,353997283
S100g	0,855213513	0,366269878	1,613879686	0,226949927	0,820462678	NA
Mup9	138,2332838	-0,591998822	1,35004493	-0,438503052	0,661021658	0,882666274
Decr2	4034,621776	0,014407065	0,164983831	0,087324103	0,930413896	0,980427423
Tinag	4,61830073	0,397016267	1,296467628	0,306229217	0,759430128	NA
Ddb1	4665,371037	-0,165471687	0,099474851	-1,663452474	0,096221897	0,385838157
Snap47	1981,880529	-0,099771328	0,093328517	-1,069033681	0,285054493	0,646352361
Cspg4	2,358512376	1,368845408	1,332002955	1,027659438	0,304110054	NA
Tmlhe	361,6926614	0,152996697	0,188658012	0,810973755	0,417380743	0,752195715
Asun	482,3857575	-0,032101563	0,096402708	-0,332994406	0,739138506	0,915635905
Satb2	11,52617258	-0,031580841	0,512344925	-0,061639804	0,950849679	0,985972877
Ncaph	22,23612664	-0,730162275	0,407507759	-1,791775147	0,073168991	0,333173109
Tmem47	68,72043588	0,070906929	0,268387861	0,264195737	0,791629096	0,936325699
Rab40c	282,5522262	-0,028622618	0,138112673	-0,207241068	0,835821596	0,951141594
Ptpa	996,0520698	-0,097281999	0,096798835	-1,004991422	0,31490098	0,673776132
Nop2	424,3480292	-0,07662338	0,218097394	-0,351326435	0,725343465	0,910252319
Fam50b	12,36359701	-0,085454526	0,538278744	-0,158755156	0,873861781	0,963438739
Lgi4	8,329846569	1,73506565	0,61683729	2,812841696	0,004910582	0,067955708
Nmnat3	179,9026308	0,39893704	0,142540647	2,798759846	0,005129928	0,069981634
Smim11	297,279067	-0,001927771	0,134046268	-0,014381387	0,988525709	0,996575327
Wt1	14,0276828	0,643285895	0,544441314	1,181552315	0,237383384	0,594575536
Wfdc12	1,355975924	-1,110728554	1,428441582	-0,777580665	0,436816263	NA
Ecscr	64,10299127	0,083002661	0,323164623	0,256843279	0,797299759	0,938325224
Ash1l	784,5401503	0,047192017	0,106095663	0,44480628	0,656459756	0,879906285
Btnl10	0,093953095	-0,517475177	3,352475198	-0,154356154	0,877328924	NA
Edem1	4482,502851	-0,1719278	0,108727823	-1,58126775	0,11381683	0,417338783
Cbx7	217,5537432	0,509108873	0,177011224	2,876138935	0,004025725	0,059273033
Med9	188,3415992	0,136685029	0,138890981	0,984117387	0,325057799	0,682335948
B630019K06Rik	14,52463096	0,056777171	0,412787942	0,137545614	0,890599541	0,968877905
H2al1m	2,982213421	0,50481878	0,89614794	0,563320806	0,573216453	NA
Rin2	387,054511	-0,024276284	0,117731135	-0,206201053	0,836633866	0,951141594
Fam189a2	13,87104014	0,07767817	0,468012952	0,165974402	0,868177095	0,961775826
Dicer1	864,1100092	-0,112456109	0,104307459	-1,078121448	0,280979561	0,641764538
Plk2	316,2915805	-0,242632777	0,240629987	-1,008323108	0,313299373	0,672227864
Hpse	81,71646331	0,255395392	0,202365741	1,262048561	0,206931313	0,559462075
Hamp2	2424,855316	2,462663398	0,956270919	2,575277937	NA	NA

Supplementary Table S1: *Serpina1* KO vs. wildtype all DEGs

Arhgef6	70,48249564	0,284945345	0,22550396	1,263593533	0,206375956	0,558880735
Plcd3	14,08650073	0,49101454	0,489496594	1,003101035	0,315812116	0,67419376
Fgl1	14071,67414	0,951203476	0,243776965	3,901941585	9,54E-05	0,004368925
Asb16	0,882777901	-3,368897236	2,395176422	-1,406534068	0,159565596 NA	
E2f4	466,514377	-0,151735329	0,116715927	-1,300039612	0,193587393	0,542139655
Gipc3	1,920661924	0,266873103	1,035224229	0,257792559	0,796567008 NA	
Ly75	54,67779878	0,416300051	0,264348558	1,574814911	0,115299174	0,420353054
Chac2	577,1298542	0,046625053	0,117878203	0,39553583	0,692447484	0,895338108
Eef1akmt1	220,2005096	0,190916611	0,13158444	1,450905677	0,146806127	0,476487281
Igsf23	11,98163206	0,528971302	0,735406529	0,719291005	0,471961637	0,786445858
Zfp706	2436,499357	-0,189050132	0,120576975	-1,567879211	0,116909324	0,424256449
Emc4	802,5724632	-0,099300228	0,10697335	-0,92827071	0,353267163	0,704269118
Tmem178	10,44997288	0,129622625	0,473789986	0,273586671	0,784402278	0,933246486
Cdc42ep3	36,06293849	0,456100438	0,384575503	1,185984115	0,235628573	0,593010215
Pdf	657,566871	0,177512763	0,112662536	1,575614839	0,115114594	0,420121786
Ggt7	7,380634312	0,400845298	0,616991794	0,64967687	0,515900968	0,813164632
G6b	1,208157694	0,277003822	1,293647384	0,214126218	0,830448631 NA	
Rfxank	779,1816591	0,116725344	0,188592168	0,618929963	0,535962499	0,823122647
Cct6a	3017,909421	-0,178132306	0,085888468	-2,073995607	0,038079719	0,229112404
Pcdhga7	1,520548398	0,483238387	1,121091078	0,431042933	0,666437153 NA	
Nyx	0,586753417	1,748687435	2,036683205	0,858595696	0,390563613 NA	
Lancl3	0,340300874	0,667300037	3,188296412	0,209296737	0,834216598 NA	
Rab8b	77,12860203	0,032314275	0,25556409	0,126442939	0,899381315	0,972093847
Dleu7	0,732172499	0,879951715	1,825374886	0,482066299	0,62975885 NA	
Aspg	3880,509031	-0,071053462	0,146864775	-0,483801929	0,628526446	0,865922214
Gm7694	142,8660919	0,71370762	0,221385721	3,223819567	0,001264931	0,028033175
BC049352	3,631514252	0,322180261	0,874089749	0,368589451	0,71243376 NA	
Ucp2	812,4578197	0,349604285	0,219893563	1,589879579	0,111861952	0,413860252
Gpr1	0,12663974	0,780932884	3,352475198	0,232942181	0,815806304 NA	
Erlec1	857,2744592	0,03555512	0,098427355	0,361232104	0,717925945	0,9078653
Riiad1	0,48002297	1,376729191	2,449311449	0,562088252	0,574055892 NA	
Cdrt4	0,12663974	0,780932884	3,352475198	0,232942181	0,815806304 NA	
Cenpp	7,775524929	-0,28686351	0,582046683	-0,492853096	0,622116391	0,863035595
Emc2	907,3185979	0,000526407	0,094038321	0,005597795	0,995533629	0,998970884
Dnase1l2	20,41938802	-0,193329328	0,374345189	-0,516446674	0,605542474	0,856421285
Mrpl37	963,2420823	-0,056504573	0,101056879	-0,559136332	0,576068681	0,844088359
1700001K19Rik	0,093953095	-0,517475177	3,352475198	-0,154356154	0,877328924 NA	
Tmem208	599,9278681	-0,017657195	0,125247811	-0,140978075	0,887887263	0,968360648
Mrps22	636,8407007	-0,16745754	0,097130551	-1,724046016	0,084699536	0,36016161
Cfap97	294,6231983	0,015528673	0,135728915	0,114409465	0,908913212	0,9747592
Pyurf	1069,29472	-0,021546922	0,122820558	-0,175434163	0,860738498	0,959574131
Mplkip	244,7941699	0,417649592	0,158585582	2,633591186	0,008448715	0,094996523
Isoc1	1623,056188	-0,015843894	0,114966539	-0,137813091	0,890388138	0,968877905
Pcp4l1	215,6199358	-0,943399833	0,511258187	-1,845251297	0,065000992	0,310757431
Cox20	581,0509589	0,270297989	0,139350537	1,939698218	0,052416376	0,276322766
Mrps14	596,9317407	-0,012002042	0,099097103	-0,121113954	0,903600777	0,973489035
Ube3a	652,7793318	-0,112607958	0,147126337	-0,765382737	0,44404367	0,770157058
5330417C22Rik	40,60157217	0,892163996	0,413111691	2,159619339	0,03080215	0,203556077
Tmem126a	833,08462	0,041585329	0,111334796	0,37351601	0,708764432	0,903200046
Tmed6	0,625780468	-1,960208775	1,921389226	-1,020203896	0,30763177 NA	
Slc47a1	2704,766948	0,383900956	0,1294998	2,964490712	0,003031844	0,049598667
Tmem128	300,3166966	0,026146499	0,119526949	0,218749821	0,826844937	0,947537943
Ing5	190,5608098	-0,006075924	0,129714748	-0,046840655	0,962640227	0,988622522
Camk2n1	1221,012164	0,019228675	0,116889371	0,164503197	0,86933503	0,961775826
Mrps17	821,3780896	0,110714533	0,097905114	1,130835037	0,25812453	0,619322836
Sike1	173,6848162	-0,03909541	0,133622441	-0,292581169	0,769842309	0,927934035
Tssk1	3,482769482	-0,623772028	0,813202692	-0,76705603	0,443048203 NA	
Ak1	6,724441798	0,380578046	0,544361306	0,69912766	0,484472254	0,79420977
Ccpg1os	175,4562128	-0,004971958	0,134241041	-0,037037543	0,970455071	0,992007633
Clca1	0,468403178	2,359796225	2,888369456	0,816999439	0,413928749 NA	
Usf3	1054,541189	-0,395381805	0,146514548	-2,698583929	0,006963518	0,084198069
Setd1b	508,1274143	-0,184564546	0,133612569	-1,381341192	0,167174078	0,506102865
Ctcfl	15,14366048	0,80119383	0,631258339	1,269201182	0,20436932	0,557220166
Sp3	905,0371403	0,060320989	0,136701228	0,441261502	0,659023693	0,881654058
Maip1	379,2127431	0,102864568	0,100249946	1,026081033	0,30485339	0,664100532
Tmem185a	425,8531986	0,184054477	0,096695271	1,9034486	0,056982035	0,288339448

Supplementary Table S1: *Serpina1* KO vs. wildtype all DEGs

Dhrsx	426,7565518	-0,013250207	0,145917068	-0,090806423	0,927646407	0,978977289
Riok3	1539,327675	-0,163799244	0,081109151	-2,019491546	0,043436155	0,24557177
Paqr9	6556,195548	-0,024428399	0,212630372	-0,114886686	0,908534938	0,9747592
Csnk1g3	649,6519892	0,060520799	0,112064624	0,540052666	0,589160712	0,849321722
Vwc2	0,957820219	-0,205696173	1,543488424	-0,133267065	0,893982173	NA
Gsap	828,5180651	0,022923705	0,112448272	0,203859998	0,838462905	0,951141594
Lrrn4cl	2,333136463	0,986224805	1,017972168	0,968813132	0,332638433	NA
Ndufb3	1507,195095	0,030554119	0,140517975	0,217439219	0,82786607	0,947580607
Acta2	56,08714809	-0,217854547	0,539117526	-0,404094722	0,686143066	0,893161227
Slc9a6	308,4297435	-0,2615716	0,119462816	-2,189565003	0,028555799	0,194708634
Tmem120b	102,0650419	-0,219968729	0,257294447	-0,85492995	0,392589929	0,734985963
Cd163l1	0,541565944	-1,663506702	2,854024186	-0,582863562	0,559985152	NA
Ssc5d	10,27164224	-0,494096303	0,565125134	-0,874313092	0,381947772	0,726456076
Olfml1	453,3762468	0,062941821	0,217119317	0,289895077	0,771896508	0,928273961
Pon3	1892,220095	-0,032796269	0,117641652	-0,278781101	0,780412819	0,931631304
Fam71e2	0,429460841	-0,031227966	2,21995908	-0,014066911	0,988776599	NA
Ppp6r1	2501,691845	-0,069401793	0,064291779	-1,079481608	0,280373088	0,6416138
Sft2d3	86,86705654	-0,057101732	0,214912344	-0,265697777	0,790471975	0,935843529
Krt19	51,1246638	0,678106346	0,25237809	2,686866937	0,007212568	0,086093589
Fa2h	0,259430135	1,627812584	3,345388605	0,486584005	0,626553161	NA
Mir10a	0,454707671	-1,551721951	2,086851925	-0,743570702	0,457136233	NA
Smg5	889,2908091	-0,112905344	0,126476577	-0,892697662	0,372019103	0,719340958
Pm20d1	3735,434662	0,200191211	0,145649713	1,374470337	0,169295713	0,508724295
Ncapd3	219,4284366	-0,062584504	0,125494858	-0,498701741	0,617989518	0,861339607
Arap2	398,1850957	0,098526779	0,149377725	0,659581465	0,509522452	0,808331038
Yars2	387,6777	-0,012939643	0,120138799	-0,107705776	0,914229088	0,976392941
Raet1e	9,721566156	1,408697426	0,560141236	2,514896844	0,011906722	0,116510842
Ces2b	29,92597012	-3,949435856	0,580217033	-6,806825083	9,98E-12	7,41E-09
Gm3636	0,227324296	1,389394708	3,349408814	0,414817893	0,67827522	NA
Eaf1	953,8762251	-0,199735488	0,123545455	-1,616696364	0,105943839	0,403646669
Dnal1	16,12591222	-0,08263003	0,384284028	-0,215023328	0,829749131	0,948328541
1700017B05Rik	456,2623392	-0,380749615	0,159677625	-2,384489472	0,017102837	0,144899552
Spag17	1,569583875	0,260809664	1,424702608	0,18306253	0,854748955	NA
2810403A07Rik	843,1000017	0,026054914	0,096553572	0,269849302	0,787276192	0,934401226
Gtf2e1	349,2493214	-0,110444884	0,199070172	-0,554803783	0,57902888	0,84530168
Rnd3	423,4578942	-0,306777867	0,181336891	-1,691756521	0,090692407	0,373642945
Sgms2	1175,914681	-0,085275207	0,147866829	-0,57670275	0,564140275	0,837355044
Ccdc3	46,7484955	0,117970686	0,322550913	0,365742836	0,714556979	0,906371815
Gbe1	2703,26292	0,264477793	0,196917582	1,343088765	0,179243233	0,522135977
Josd1	873,8558045	0,224967843	0,103226388	2,179363698	0,029304661	0,19731337
Pdia6	9045,017206	-0,580422801	0,164289486	-3,532927238	0,000410986	0,012865677
Dcbld2	64,55149939	-0,048243796	0,206040993	-0,23414659	0,814871177	0,944076707
Tpt1	20162,64997	-0,020168317	0,119023381	-0,169448365	0,865443984	0,961775826
Aspm	8,123917475	-1,187192428	0,524969666	-2,261449574	0,023731432	0,174393148
Dennd1c	47,36490984	0,445349381	0,272372995	1,635071721	0,102034013	0,397810652
Mir505	0,113662148	0,780932884	3,352475198	0,232942181	0,815806304	NA
Mir26b	0,142634598	-0,517475177	3,352475198	-0,154356154	0,877328924	NA
Il6ra	446,8280804	0,594337953	0,274983513	2,1613585	0,030667657	0,202767512
Creg1	11603,85196	0,154179338	0,119101496	1,294520585	0,195485664	0,544454182
Cxx1a	90,12119451	-0,041130451	0,175843502	-0,233903731	0,815059716	0,944076707
Cxx1b	79,6386111	-0,029867314	0,219237926	-0,136232425	0,891637542	0,969104575
Fam167a	31,74332171	0,013948076	0,357289819	0,039038548	0,968859655	0,991555135
Scarna3a	0,147722973	0,780932884	3,352475198	0,232942181	0,815806304	NA
Tram2	47,02087571	-0,285166478	0,32425258	-0,879457854	0,379153075	0,725074694
Snora31	14,22359695	-0,44913094	0,450584658	-0,996773706	0,318874364	0,676671647
Endov	155,0515199	0,18014162	0,174635877	1,031526988	0,302293759	0,6622779
Nalcn	0,797136764	3,09285347	1,657937609	1,865482424	0,062113814	NA
Ubxn7	465,6633575	0,16986682	0,10404682	1,632599822	0,102553189	0,398788127
D6Ertd527e	0,831922049	2,225929917	1,91929999	1,159761334	0,246145991	NA
1810041H14Rik	9,957534682	-0,314894365	0,455034031	-0,692023769	0,488922402	0,796400974
Rgsl1	0,209517726	0,059593471	3,352475198	0,017775962	0,985817581	NA
Cxcr2	3,232050225	-0,506095479	0,936962662	-0,540144767	0,5890972	NA
Exosc4	409,7372875	0,056961838	0,14160697	0,402253068	0,68749779	0,893386271
6330408A02Rik	71,739458	-0,104510753	0,198556434	-0,526352888	0,598643043	0,854045336
Xpnpep3	485,5021754	-0,516111057	0,104218474	-4,952203178	7,34E-07	0,000101133
Cyp4f39	20,08650509	0,410148469	0,45739472	0,896705735	0,369875959	0,717858445

Supplementary Table S1: *Serpina1* KO vs. wildtype all DEGs

Gm715	1,981432832	-1,44781908	1,310772775	-1,104553823	0,269352969	NA
Slc48a1	1253,052737	0,122440664	0,114253013	1,071662445	0,283871674	0,645202383
Chmp2a	2329,580932	0,104520746	0,067114741	1,557344095	0,119388812	0,429754718
Kiss1r	12,48606305	0,145473212	0,436611478	0,33318687	0,738993229	0,915625623
Mir30c-2	0,9626378	1,192607475	2,261212812	0,527419387	0,597902383	NA
Slc4a1	1,97247319	-1,166129104	1,482292017	-0,786706729	0,431453564	NA
Slc34a2	168,8132033	0,785970297	0,759687342	1,034597069	0,300857124	0,661404508
Slc34a1	0,113662148	0,780932884	3,352475198	0,232942181	0,815806304	NA
Slc16a7	961,0584302	0,658256768	0,194339051	3,387156431	0,000706211	0,018658767
Slc2a3	5,932046968	-0,302383877	0,611134085	-0,49479138	0,620747389	0,862608071
Nr2c1	442,7062416	0,133882442	0,126259836	1,06037238	0,288975223	0,650101875
Akirin2	461,181267	-0,092135308	0,119933144	-0,768222234	0,442355166	0,769135977
Foxk2	398,5838625	-0,328401691	0,169509981	-1,937359019	0,052701472	0,276952038
Snora23	1,451743395	1,181897826	1,340731655	0,881531977	0,378029954	NA
Cisd3	1031,866009	0,398035325	0,0920666	4,323341228	1,54E-05	0,00116079
Ddr2	92,74276129	-0,027402491	0,209480084	-0,130811913	0,895924099	0,97084116
Sp5	25,95962658	0,568472781	0,401766868	1,414931959	0,157088447	0,492826562
Clns1a	657,3296015	0,006144344	0,088249748	0,069624497	0,944492539	0,984094829
Lrat	430,336436	-0,14741723	0,202963916	-0,726322355	0,467641178	0,782859415
Sfpq	1414,628102	-0,232628647	0,156214361	-1,489163003	0,136444451	0,459349518
Gpr88	0,629055292	2,732753141	2,727361299	1,001976944	0,316354728	NA
Gng13	0,180324769	-1,201310802	3,342952034	-0,359356278	0,719328578	NA
Tmub1	204,9566716	-0,078483062	0,129659058	-0,605303351	0,544977455	0,827491596
Itm2c	813,4648226	-0,07508981	0,091556544	-0,820146839	0,412132403	0,749224653
Ptges	12,37304259	-0,586135061	0,702764137	-0,834042363	0,404257112	0,744376512
Ngb	2,422769027	0,722937325	0,999395428	0,723374657	0,469449737	NA
Slc13a2	172,1857968	-0,400873745	0,243073258	-1,649189002	0,09910892	0,392588787
Myh9	3093,302595	-0,212019544	0,110848947	-1,912688832	0,0557879	0,285429939
Zfp296	16,95283701	-0,30090323	0,377685643	-0,796702853	0,425623627	0,758328303
Smurf2	867,0059515	-0,001542701	0,081143171	-0,019012088	0,984831463	0,995554769
Mrpl38	1875,138369	-0,214199678	0,097781293	-2,190599783	0,028480767	0,194503289
Rpf2	233,1878203	0,101780149	0,139442115	0,729909678	0,465445396	0,781663619
Sdf2l1	1333,850109	-1,266068756	0,255926224	-4,947006743	7,54E-07	0,000102809
Atp6v0e	668,1849886	0,078567427	0,118152094	0,664968553	0,506070607	0,805958988
Rab12	549,4846623	0,043966735	0,099648788	0,44121696	0,659055936	0,881654058
Slc28a3	0,778850291	-0,062899308	1,935300166	-0,032501061	0,97407247	NA
Fbln7	14,51411522	0,029185275	0,496452072	0,058787699	0,953121206	0,986479135
Zdhhc16	352,4304689	-0,152937694	0,147446571	-1,037241442	0,299623349	0,659904651
Ercc6l2	312,4571522	-0,014931362	0,125001622	-0,119449345	0,904919369	0,973671392
Tbc1d20	388,5033962	-0,084748314	0,147089603	-0,576167941	0,564501672	0,837355044
Tcte2	10,25331638	-0,123547198	0,529291228	-0,233420075	0,815435226	0,944248697
Itpk1	708,4566337	0,076714875	0,146856233	0,522380788	0,601405229	0,854422974
S100a13	513,6386187	-0,034935464	0,128940787	-0,270941915	0,786435706	0,934050588
Lpl	961,4364834	0,241243911	0,51075655	0,472326612	0,636693678	0,86976752
Mir5133	3,347583194	-0,381243172	0,781593917	-0,487776534	0,625708135	NA
Ddx23	916,8623337	-0,107897554	0,071738757	-1,504034334	0,132572527	0,453057798
Ogfrl1	319,5223654	0,105881122	0,183098927	0,578272763	0,56307998	0,83722511
Zfp984	53,66782526	0,051508378	0,236110893	0,218153334	0,827309644	0,947569588
Ly6c2	8,797333469	0,753214875	0,504641988	1,49257274	0,135549063	0,458151054
Mir7001	1,331565688	1,491274116	1,509971949	0,987617099	0,323340209	NA
P2ry12	42,4328474	0,31559382	0,33130405	0,952580628	0,340802596	0,694866457
Rpap3	236,8770272	0,07395879	0,137678233	0,537185789	0,591139287	0,850384506
Klhdc1	4,832359949	0,574599736	0,72886921	0,788344093	0,430495463	NA
Cytl1	0,093953095	-0,517475177	3,352475198	-0,154356154	0,877328924	NA
Klhl29	3,683889365	1,112536136	0,799847712	1,390934948	0,164245154	NA
Cideb	7168,10717	0,097117326	0,094531978	1,027348929	0,30425619	0,66366471
Nktr	1064,317609	-0,639927532	0,116653161	-5,485728181	4,12E-08	1,10E-05
Notch2	906,4625556	-0,154541754	0,110556732	-1,397850229	0,162158047	0,498749809
Spag4	22,13204506	-0,123961007	0,325609087	-0,380704999	0,703422159	0,900719679
Pabpc1l	0,842516631	-1,114422578	1,850080802	-0,602364273	0,546931686	NA
Mup17	0,63985726	-1,039371769	2,021301378	-0,514209202	0,607105735	NA
Mup15	275,2373067	-2,171499113	1,187719941	-1,828292208	0,067505709	0,318406623
Tmppe	143,1768946	0,089412622	0,171805175	0,520430319	0,602763683	0,85472504
Ambp	73747,25891	0,05849066	0,118227976	0,494727747	0,620792313	0,862608071
LOC100038947	2,750950723	1,605035596	0,975660638	1,645075688	0,099954203	NA
Dnajc3	6708,187509	-0,528266078	0,14498897	-3,643491481	0,000268965	0,009293414

Mir1931	0,122496332	0,780932884	3,352475198	0,232942181	0,815806304	NA
Rasgrf2	5,478580192	0,646578783	0,714373774	0,905098713	0,365413094	0,713689942
Tanc1	249,6740849	-0,240229216	0,136994824	-1,753564177	0,079505214	0,349223642
Cep126	2,710404152	0,223714492	0,891422189	0,250963566	0,801842278	NA
Zxda	22,42867545	0,119093981	0,362021812	0,328969077	0,742179061	0,916899135
Gp1ba	1,304889853	2,172806207	1,381737387	1,57251749	0,115830586	NA
Gimd1	59,98361335	0,213407678	0,330871887	0,644985829	0,518936369	0,813725618
Chek1	16,12692191	-1,579288213	0,530359475	-2,977769394	0,002903544	0,048005373
Fzd4	306,2909543	-0,331022261	0,239994035	-1,379293703	0,167804217	0,506611452
Vps26b	797,8726844	-0,07286937	0,103489625	-0,704122463	0,481356512	0,793276609
D030047H15Rik	1,622427281	-1,488814011	1,277709925	-1,165220667	0,243929682	NA
Col28a1	3,606562715	-3,051353751	1,30897459	-2,331102355	0,019747963	NA
Filip1l	186,1217384	0,101113267	0,188972354	0,535069095	0,592602079	0,851332066
Csf1r	1346,844191	0,200664334	0,212551796	0,944072631	0,345132534	0,698150529
Erich4	0,442772444	-0,108006636	2,2108556	-0,048852867	0,96103655	NA
Kif26a	20,39689101	-0,656307307	0,359382846	-1,826206551	0,067819155	0,319033919
Ppp1r36	1,100207301	0,457610359	1,348925685	0,3392406	0,734428486	NA
Gpr15	0,808612629	3,110352523	2,592430842	1,199782256	0,230223917	NA
Cfap58	0,12663974	0,780932884	3,352475198	0,232942181	0,815806304	NA
Usp45	289,3722795	-0,076906913	0,256220254	-0,300159383	0,764055585	0,925867457
Zfp865	225,5691749	-0,204063833	0,146704087	-1,390989425	0,164228634	0,502534356
Zscan22	132,4740512	-0,036579101	0,203967811	-0,179337618	0,857672611	0,958260148
Ttbk1	9,173446679	-0,596816775	0,481002289	-1,240777411	0,214687988	0,568124251
Ccr5	138,3225341	0,232935696	0,213251746	1,092303819	0,274699582	0,636474647
Nid1	402,6269861	0,000760179	0,249468274	0,003047196	0,997568693	0,999545959
Ccr4	0,353927913	-0,100118367	2,670731403	-0,037487247	0,970096509	NA
Plod1	1373,55181	-0,185088007	0,115725571	-1,599370013	0,109738411	0,410031531
Ccr2	29,9764387	0,544545823	0,348923301	1,560646197	0,118607252	0,427632243
Ccr3	9,970576944	-1,507853335	0,68273852	-2,208537078	0,027206856	0,190567596
Cyp21a1	9,781556272	-0,943236198	1,086551092	-0,8681011	NA	NA
4931403E22Rik	14,58219017	-0,177746307	1,786947186	-0,099469256	0,920765697	0,977341679
2810029C07Rik	4,404186483	0,389566946	0,707594555	0,55055108	0,581941452	NA
Gm4285	79,39908582	0,388157531	0,184932894	2,098910167	0,03582482	0,221424884
1700102H20Rik	2,523952442	1,798697248	1,086251949	1,655874818	0,097747172	NA
D630045M09Rik	2,661993057	-0,130646336	0,827679868	-0,157846458	0,874577789	NA
Cd101	1,191484484	-0,789200723	1,454146595	-0,54272432	0,587319627	NA
Pmepa1	83,08423934	-0,240287276	0,238383764	-1,007985073	0,313461628	0,672227864
Zfp235	99,05143967	0,185962849	0,160992061	1,15510571	0,24804714	0,607129663
2810454H06Rik	4,871879692	1,108065561	0,748415232	1,480549184	0,138726745	NA
Gm9159	0,816212477	2,208063419	1,922149888	1,148746741	0,250660426	NA
Rnf187	1664,512113	-0,150115253	0,082866317	-1,811535231	0,070058041	0,32382454
Spink5	7,917459688	0,559433666	0,662557838	0,844354461	0,398471361	0,739635153
Chrnb1	17,59517866	1,238436498	0,373169051	3,318700983	0,000904372	0,021941826
F5	12764,34998	-0,13561162	0,152297598	-0,890438337	0,373230565	0,720641164
Gnai3	2229,003986	-0,155879995	0,115664634	-1,347689349	0,177758319	0,52021398
Supt4a	558,0633626	0,039592676	0,114365129	0,346195349	0,72919591	0,911594858
Gli1	1,530192285	-1,625043581	1,159556315	-1,40143567	0,161083832	NA
Tnfsf8	0,754014099	-0,611612705	1,966647343	-0,310992567	0,755806273	NA
Syp	15,02604819	0,297173775	0,398810436	0,745150447	0,456180775	0,778008437
Phlda2	7,210625247	-1,141553636	0,603902073	-1,890295939	0,058718392	0,293022089
Gnai1	10,90184404	0,039805419	0,462997326	0,085973324	0,931487623	0,980584977
Ier2	537,401358	-0,121877689	0,215942611	-0,564398515	0,572482949	0,841794408
Gna15	8,106787223	0,173115163	0,527486525	0,328188788	0,742768925	0,917092327
Cct8	3659,376958	-0,078189596	0,094429008	-0,828025176	0,407656242	0,746679655
Apob	95296,10448	-0,210617488	0,119653528	-1,760227984	0,078369159	0,347041168
Zfpm1	1238,939073	-0,644609213	0,14982674	-4,302364281	1,69E-05	0,001248156
Zfp93	91,75064286	0,042661061	0,167280532	0,255027054	0,798702204	0,938733973
Slc25a18	0,339756192	1,876228608	3,160061454	0,593731684	0,552691588	NA
Ppp1r3c	3516,378082	0,044774373	0,212047255	0,211152806	0,832768036	0,950100347
Zfp367	336,5287988	-0,309771026	0,124504851	-2,488023752	0,012845515	0,121814305
Opn3	15,8398223	0,218200139	0,40510511	0,538625985	0,590144954	0,84953676
Fbn2	0,379918946	1,000946436	2,589354372	0,386562167	0,699080371	NA
Plau	22,49788821	0,31835945	0,512396162	0,621315057	0,534392349	0,821767588
Serpine1	9,2416822	0,013353409	0,778319229	0,017156725	0,986311585	0,996004319
Bora	16,61581893	-0,453984687	0,360649651	-1,258796968	0,208103676	0,56023257
Nt5dc3	382,7745699	-0,22802089	0,159009088	-1,434011687	0,151568928	0,48474643

Supplementary Table S1: *Serpina1* KO vs. wildtype all DEGs

Adprh	245,6791658	-0,11774159	0,123804845	-0,951025701	0,341591329	0,695250579
Adora2b	0,433709287	1,155703805	2,51809517	0,458959542	0,64626322	NA
Axdnd1	0,88878841	-3,328366891	1,71841265	-1,936884537	0,052759459	NA
Gm14325	263,7421049	0,052187282	0,164840325	0,316592931	0,751552501	0,920691412
Adh1	81478,50529	0,078584161	0,122239334	0,642871312	0,520307605	0,814232983
Bik	112,8966353	-0,578983771	0,375850198	-1,540464191	0,123447245	0,436341444
Calr	30982,84449	-0,346881645	0,141824378	-2,44585346	0,014450972	0,13080163
Onecut1	402,0156164	-0,476946884	0,706622911	-0,674966629	0,499696967	0,802860694
Hells	24,06132914	-0,311320967	0,322059432	-0,966656885	0,333715587	0,690305383
Ubn1	672,2222171	-0,055250356	0,095943847	-0,575861375	0,564708885	0,837355044
Ahctf1	1738,08593	-0,107243787	0,096384142	-1,112670448	0,265849992	0,626006345
Med30	118,9280338	0,2357089	0,167371767	1,408295466	0,159043598	0,495312554
Babam1	1568,845753	-0,181533992	0,089515688	-2,027957299	0,042564606	0,243203202
Zfp607a	32,40643112	0,188794029	0,310887997	0,607273458	0,54366945	0,827311545
Cyp2c67	5078,356114	-0,289037582	0,209528482	-1,379466792	0,167750878	0,506611452
Abt1	231,4458271	0,005070664	0,130878887	0,038743178	0,969095148	0,991650667
Galt	1653,869552	0,299631469	0,127215364	2,355308822	0,018507318	0,151329864
Fbxl14	209,5388238	0,095642654	0,137009011	0,698075644	0,485129889	0,79430833
Gde1	1066,442029	-0,258663285	0,133576545	-1,9364424	0,052813541	0,277322948
Znhit1	864,2188794	-0,113911213	0,090085347	-1,26448104	0,206057422	0,558880735
Dkk2	0,648724138	0,888216614	2,034829722	0,436506605	0,662469208	NA
Mybph	0,281209566	-1,718135424	3,30990564	-0,519088944	0,603698718	NA
Rab11a	1114,047822	0,02478433	0,086182066	0,287581056	0,773667448	0,928621142
Alyref2	104,5943993	-0,006111633	0,157779019	-0,038735398	0,969101351	0,991650667
C3	223143,9316	0,395056497	0,132317631	2,985667845	0,002829598	0,047523739
Vprbp	635,7251858	-0,14660268	0,098269918	-1,491836797	0,135741935	0,458266143
4930524B15Rik	0,331469375	1,851207654	3,180323419	0,582081571	0,560511738	NA
Urm1	274,1372134	0,079288304	0,112962254	0,701900864	0,48274099	0,793999136
Ccdc71l	107,002542	0,118357819	0,204727651	0,578123271	0,563180897	0,83722511
Vim	442,489465	-0,099180985	0,287861768	-0,344543791	0,730437369	0,911941257
Tmsb15l	2,17360961	1,470009844	1,123033256	1,308963769	0,190546624	NA
Tubgcp6	249,6741415	-0,325151217	0,120660888	-2,694752406	0,007044095	0,084687508
Gm11744	1,536961045	-0,159462594	1,262423025	-0,126314706	0,899482815	NA
Tifab	69,12928523	-0,258961809	0,213875657	-1,210805437	0,225969981	0,581158101
9130011E15Rik	209,3900274	0,210445161	0,163868558	1,284231478	0,199060974	0,54896375
Tmx3	352,7271607	0,082099354	0,159108703	0,51599537	0,605857643	0,856477829
Klhl17	197,6543155	0,320452579	0,130381565	2,45780589	0,013978871	0,128707661
Nup188	220,0496104	0,070239367	0,141642997	0,495890149	0,619971917	0,862258786
Eif5b	1903,137517	-0,160849762	0,090447199	-1,778383014	0,075340971	0,339364365
Map6d1	0,086476712	0,780932884	3,352475198	0,232942181	0,815806304	NA
Rai2	50,77196521	0,161443821	0,265215542	0,608726848	0,542705508	0,826611597
Tex2	4478,898741	-0,042378474	0,120490196	-0,351717197	0,725050362	0,910252319
Eloa	1118,243184	-0,056879189	0,078601007	-0,723644525	0,469283999	0,784101987
Elavl1	716,8470801	-0,03944337	0,121437245	-0,324804552	0,745328999	0,918622973
Pacs1	77,40937923	0,627074852	0,179272878	3,497879084	0,000468974	0,013963718
Prps1l3	697,4903643	0,039694031	0,120320707	0,329901913	0,741474078	0,916735109
Cybb	277,9707543	0,303988258	0,256721854	1,184115233	0,236367451	0,593649531
Cfap20	612,0862139	-0,12069288	0,119998068	-1,005790192	0,314516506	0,673320811
Itgb2l	0,122496332	0,780932884	3,352475198	0,232942181	0,815806304	NA
Gstt1	7200,866849	0,376250371	0,14576019	2,581297199	0,00984298	0,103533285
Cyb561	124,7379307	0,306710029	0,227029905	1,350967524	0,176705836	0,519981998
Gstm6	3269,243724	0,375006175	0,130429294	2,875168327	0,004038122	0,059390151
Cux2	10,03069298	1,723056775	0,964967351	1,785611474	0,074162188	0,335751537
Csrp2	705,9957902	0,036837562	0,131559073	0,280007764	0,779471548	0,931533405
Ctsk	12,18003655	-0,553298976	0,460706353	-1,200979695	0,229759078	0,585962127
Zc3h18	1139,980578	-0,087624004	0,080147229	-1,093288001	0,27426737	0,635693562
Ncan	0,122496332	0,780932884	3,352475198	0,232942181	0,815806304	NA
Aqp3	0,993569046	0,127763197	2,229835166	0,057297149	0,954308492	NA
Snord19	1,823450137	-1,425127731	1,115440524	-1,277636683	0,20137756	NA
Hhipl1	3,226035608	-0,496295802	0,773990367	-0,641217028	0,521381688	NA
Cacna1i	2,195638234	0,073778298	0,95323139	0,0773981	0,938306853	NA
Ndufaf8	221,5522891	0,104364285	0,125967405	0,828502299	0,407386094	0,746679655
Slc39a12	0,899074802	-0,397694434	1,638263367	-0,242753664	0,808196233	NA
Atp8b2	53,87522454	0,364836027	0,334758253	1,089849239	0,27577956	0,637060499
Gm15104	0,093953095	-0,517475177	3,352475198	-0,154356154	0,877328924	NA
Mir7682	0,4541428	1,189143672	2,18195475	0,54499007	0,585760347	NA

Gm3435	9,012915187	0,173380576	0,532135206	0,325820532	0,744560143	0,918321563
9030025P20Rik	18,52103186	0,270679442	0,354098653	0,764418165	0,444618087	0,770559918
Vps9d1	390,4150486	0,052386646	0,107241616	0,488491759	0,625201561	0,864534513
Il7r	6,206979951	0,694740686	0,810524873	0,857149126	0,391362474	0,734518115
Hmgcl	4439,088661	0,010011199	0,087011077	0,115056601	0,908400258	0,9747592
Ruvbl2	334,2814018	-0,186122771	0,098621439	-1,887244517	0,059127445	0,293856809
Polr2j	648,2183975	0,028289875	0,086314702	0,327752683	0,743098666	0,917092327
Zfp13	35,77465093	0,679721505	0,372206104	1,826196559	0,06782066	0,319033919
Rpl9	5421,503306	0,031987598	0,092009789	0,347654287	0,728099831	0,910908351
Rs1	0,147722973	0,780932884	3,352475198	0,232942181	0,815806304	NA
Rpl7	8591,728496	0,026577711	0,097758677	0,271870613	0,785721508	0,933798335
Rps7	4374,314461	0,068405154	0,133894973	0,510886651	0,609430429	0,857753956
Rpl27	1517,392797	0,118612258	0,143178395	0,828422875	0,407431056	0,746679655
Rpl6	5063,654794	-0,014170225	0,114023077	-0,12427506	0,901097493	0,972526743
Rpl10a	3031,141805	0,008971084	0,114013562	0,078684355	0,93728369	0,982325623
Sema3c	18,42191982	1,592364734	0,524036208	3,038654029	0,002376376	0,042529812
Timp1	11,65609813	3,258505224	0,697994872	4,668379893	3,04E-06	0,000322112
Rpa2	143,5763441	-0,233708539	0,194463133	-1,201814122	0,229435553	0,585386105
Mxi1	587,5545186	-0,077484076	0,085173194	-0,909723731	0,362968226	0,712106207
Dusp5	7,88677781	0,101817146	0,510253276	0,199542365	0,841838507	0,952074658
Fam229a	0,97575105	0,673744339	1,584566286	0,425191641	0,670696978	NA
Kat8	421,2344068	-0,333889677	0,110250596	-3,028461422	0,002458025	0,043295561
Ascl1	0,093953095	-0,517475177	3,352475198	-0,154356154	0,877328924	NA
Marcks	316,3523768	0,137783596	0,280586976	0,491054853	0,623387652	0,863525452
Foxd4	0,266906519	0,586045239	3,35071904	0,174901337	0,86115716	NA
Map1s	147,7514662	-0,041951684	0,146408215	-0,286539139	0,774465221	0,929237326
Usp27x	23,81995185	-0,176247845	0,407036119	-0,433002963	0,665012618	0,884653503
G3bp1	1215,548045	0,020448884	0,101844575	0,200785209	0,840866529	0,951786015
Samd10	46,4852361	-0,160645143	0,265595888	-0,604848004	0,545279994	0,827491596
Phf20	197,5865504	-0,318899367	0,126652842	-2,517901386	0,011805637	0,116136542
Frmd5	4,502619073	-0,318007307	3,093799774	-0,102788587	NA	NA
Ganc	458,8821566	-0,111984991	0,109315498	-1,024420082	0,305636901	0,664636701
Dnajc25	520,4471286	-0,069397113	0,102786984	-0,675154677	0,499577499	0,802860694
2510002D24Rik	317,3334495	0,103746802	0,162450241	0,638637417	0,523058834	0,815322684
Neto2	6,182860962	0,367350942	0,553193325	0,664055269	0,506654938	0,806233823
Tnks1bp1	1037,461784	-0,191133015	0,115866159	-1,649601711	0,099024425	0,392481932
Mapk15	474,2809151	-0,564237504	0,438656511	-1,286285487	0,198343446	0,548428857
Ism1	2,110515513	0,16864316	0,95252011	0,177049449	0,859469549	NA
Herc2	930,4261909	-0,361256824	0,116250813	-3,10756384	0,001886362	0,036692532
Col9a2	0,414629492	1,038268337	2,526623974	0,410931087	0,681123067	NA
Rgag4	20,69979537	0,352674897	0,387014974	0,911269385	0,362153453	0,711376655
Has1	0,626371763	2,765939832	2,215259683	1,248584919	0,211816931	NA
Hars	968,8287335	-0,206626486	0,107601322	-1,92029692	0,054820405	0,283546844
Capn6	28,52246453	0,191942485	0,469573409	0,408759272	0,682716331	0,891903322
Drg1	1164,264661	-0,074667904	0,096908544	-0,770498665	0,441004147	0,768480766
Sec14l2	21109,04099	-0,016554713	0,13197655	-0,125436777	0,900177774	0,972376014
Tbc1d19	61,82092088	0,696723545	0,233877064	2,979016129	0,002891756	0,048005373
Zfp52	16,92917455	-0,502277862	0,401352771	-1,251462299	0,21076586	0,563320429
Slc6a13	7105,826078	-0,035675005	0,085250645	-0,418471962	0,675602082	0,889164486
Mir3091	1,134659425	-0,627580614	1,387117262	-0,452435156	0,650955526	NA
A1bg	390,6194613	7,192099761	2,102706494	3,420401175	NA	NA
Pwwp2b	56,33786537	0,1141458	0,257362413	0,443521643	0,657388468	0,880359254
9930111J21Rik1	3,268653768	-0,673358908	0,801647282	-0,839969053	0,400925738	NA
Fmo3	532,6218982	8,216353981	1,785358492	4,602075168	NA	NA
Smc2	61,94070117	-0,473601317	0,277937081	-1,703987519	0,088383419	0,368519746
Col8a1	11,61676594	-0,738297102	0,593442899	-1,244091224	0,21346599	0,566976917
Ogt	1349,234851	0,128413427	0,125968532	1,019408778	0,308008941	0,667059844
Igsf9	14,15066469	0,478667681	0,397971345	1,202769212	0,229065642	0,584981579
Pdcd6	1452,824305	0,117392658	0,073522964	1,596680149	0,110337023	0,411004641
Vamp2	487,5378954	0,064083452	0,128901551	0,497150353	0,619083029	0,861782322
Vhl	223,5907649	-0,103687717	0,151584418	-0,684026227	0,493958605	0,799386586
Vegfc	65,68021011	0,294157362	0,221080801	1,33054232	0,183339653	0,529363703
Haus5	146,0699609	-0,127074471	0,140864141	-0,9021066	0,367000243	0,715431066
Grin2c	0,612163645	1,800827837	2,792944104	0,644777614	0,519071311	NA
Inmt	20842,30054	-0,240648418	0,155785242	-1,544744642	0,122408042	0,434465289
C330021F23Rik	136,8511579	0,777851794	0,285865736	2,721038924	0,00650771	0,080706468

Fbxl18	52,89001546	-0,46595033	0,27531723	-1,692412531	0,090567345	0,373377888
Vsig10	43,30232112	0,538493537	0,281515097	1,912840704	0,055768449	0,285429939
Rgs11	4,416965805	0,447195298	0,679988008	0,657651741	0,510761939	NA
Ltn1	644,6353773	-0,044216926	0,133442567	-0,331355482	0,740375987	0,916128178
Ifit1bl1	8,724849992	0,536987297	0,588829801	0,911956725	0,361791499	0,71109038
Ctsl	17627,51511	0,557903814	0,146280871	3,813921877	0,000136779	0,005696557
Klhl41	2,293951701	-1,93377633	1,285581736	-1,504203332	0,13252902	NA
Ppig	868,3703286	0,093882939	0,079038805	1,187808172	0,234908994	0,592546857
Ccar2	577,9203369	-0,018806062	0,12279317	-0,153152349	0,878278134	0,965046146
Heatr6	343,7507071	-0,176352933	0,127068244	-1,387859996	0,165179681	0,503431976
Nle1	92,13982825	0,348257056	0,193220059	1,802385626	0,071484755	0,327836675
Fam222b	554,7149845	-0,138832658	0,113758188	-1,220419026	0,222306069	0,577161019
Gm13212	5,102713529	-0,447607363	0,706434978	-0,633614383	0,526332512	NA
BB218582	1,828972979	2,735428558	1,378671113	1,984105224	0,047244111	NA
Abcf1	1780,384497	-0,230723474	0,088745079	-2,599845269	0,00932658	0,100311385
Limd1	717,1217014	0,018210819	0,087549235	0,208006605	0,83522381	0,951141594
Rac3	5,6281302	-0,695477242	0,588559011	-1,181661021	0,23734023	0,594575536
Ttc36	10882,40421	0,375973687	0,097144653	3,870245814	0,000108726	0,004829081
Top3b	628,5624461	-0,106912417	0,099894483	-1,070253476	0,284505229	0,645809283
Bag3	1022,531103	-0,401107365	0,179029327	-2,240456197	0,025061321	0,181203249
Polg	828,8843807	0,094684234	0,089906875	1,053136747	0,292278323	0,654063202
Cldn2	2914,630429	0,624171875	0,147233682	4,239328024	2,24E-05	0,001513735
Samd1	285,3918464	-0,355678694	0,210906127	-1,686431303	0,091712756	0,375876101
Psg22	0,227324296	1,389394708	3,349408814	0,414817893	0,67827522	NA
Aga	354,8816747	-0,088427587	0,129749369	-0,681526139	0,495538633	0,80006558
Ttll7	14,51515624	0,405961574	0,389516827	1,042218322	0,29731047	0,658144463
Srcap	1137,578549	-0,050931344	0,1524874	-0,334003622	0,738376826	0,915438384
Rps27l	2391,167539	0,03766378	0,129110749	0,291716841	0,770503131	0,927934035
Ube2r2	2940,462994	0,032944814	0,079597226	0,413893992	0,678951744	0,890853456
Ankrd39	73,81316174	-0,261978508	0,216547511	-1,209796901	0,226356836	0,581402791
Gm21188	1,86848084	1,958287998	1,371410926	1,427936705	0,153310078	NA
S100a1	3368,102227	0,086090021	0,104171699	0,826424282	0,408563454	0,746898413
N4bp2l1	529,0373837	-0,082389934	0,269237393	-0,306012228	0,759595336	0,924882144
Tpr	1703,053732	-0,024959946	0,088738753	-0,281274468	0,778499892	0,930864224
Rbm26	305,1896069	-0,196400644	0,126948029	-1,547094863	0,121840372	0,43379282
Rpl10	9005,055621	0,011222246	0,112732048	0,099547965	0,920703206	0,977341679
Stx12	425,7627329	-0,099772735	0,116655029	-0,855280182	0,392396056	0,734887345
Ssbp4	90,73039101	0,286404856	0,200016607	1,431905386	0,152170901	0,485646401
Lpar6	560,6585091	-0,477608076	0,156779593	-3,046366347	0,002316254	0,041959355
Sf3b5	377,8112742	-0,064065644	0,142093412	-0,450869908	0,652083313	0,877662367
Ccny	1261,121744	-0,079508243	0,098508287	-0,807122377	0,419595981	0,753773001
Ssr3	7063,403936	-0,28805296	0,117737998	-2,446559002	0,014422719	0,130723613
Pgpep1l	5,233747101	-0,521190591	0,636104046	-0,81934802	0,412587882	NA
Rrp7a	562,1430249	0,021139646	0,083740665	0,252441825	0,800699575	0,939490312
Borcs6	213,7024683	-0,107644609	0,145819318	-0,738205408	0,460389637	0,780953195
Selenon	58,58897238	0,108891711	0,218456158	0,498460248	0,618159681	0,861339607
Gzf1	1033,899992	0,021696815	0,092751449	0,233924268	0,815043773	0,944076707
Gm6904	0,891519074	0,52413511	1,556476069	0,336744728	0,736309343	NA
Nudt19	1041,565085	0,135943723	0,174829369	0,777579442	0,436816984	0,76575019
Apex2	131,296404	-0,442040841	0,142260519	-3,107262965	0,001888283	0,036692532
Kdm5b	541,9537969	0,007181079	0,218101954	0,032925329	0,973734134	0,992775209
Atp5g3	8251,23861	0,049796928	0,103437917	0,481418505	0,630219087	0,866281784
E2f5	148,3026856	0,08547425	0,162641906	0,525536448	0,599210323	0,85430922
Fgd1	41,23789807	0,249265377	0,289082136	0,862264891	0,388541768	0,732257617
Serpina10	5032,97838	0,00518598	0,10199077	0,050847546	0,959447004	0,987937108
Ssh1	179,261706	-0,153322914	0,122175391	-1,254941057	0,209500158	0,561873081
Pnpla8	2639,215952	0,11995521	0,140544476	0,853503553	0,393380118	0,73584704
Agap2	43,79318317	-0,823530082	0,294689654	-2,79456734	0,005196922	0,07004174
Wapl	1676,11561	-0,195884687	0,120140426	-1,630464392	0,103003385	0,399261309
Agtrap	126,6510865	0,19052935	0,281742945	0,676252425	0,498880393	0,802566549
Ccdc180	1,658837658	-0,529856001	1,244139527	-0,425881494	0,6701942	NA
Inpp4b	10,2733693	-0,304862153	0,593337131	-0,513809329	0,607385305	0,857549281
Psmg1	488,0643423	-0,096384056	0,11051436	-0,872140562	0,383131701	0,727569276
Dnah10	0,180974489	-1,204797453	3,342691436	-0,360427361	0,718527568	NA
Gas6	1365,026534	0,601021334	0,231067255	2,601066666	0,009293439	0,100196765
Slc25a20	2307,450636	-0,054715475	0,168212071	-0,325276743	0,744971631	0,918521233

Rabggta	753,7798425	-0,090007516	0,107930732	-0,833937786	0,404316043	0,744376512
Grasp	20,29770369	-0,190742557	0,472470541	-0,403713122	0,686423689	0,893161227
Vsig2	12,95985398	-0,140107483	0,483136354	-0,289995737	0,771819499	0,928273961
Atrn	1690,660401	-0,200565185	0,126229767	-1,588889761	0,112085283	0,414284808
Lenep	64,26994574	-0,250666172	0,243315061	-1,030212312	0,302910352	0,662675256
Lgals12	0,113662148	0,780932884	3,352475198	0,232942181	0,815806304	NA
Sh3rf1	59,8605248	-0,364552041	0,263123353	-1,385479611	0,165905863	0,504089882
Guca1a	44,82048966	-0,322455052	0,289817142	-1,112615525	0,26587359	0,626006345
Ncoa1	442,6307023	-0,203193025	0,151550461	-1,340761511	0,179997896	0,52301497
Mgat3	5,704980177	0,598963689	0,605408772	0,989354163	0,322489893	0,67984555
Cat	99435,69328	-0,041358645	0,117784623	-0,351137897	0,725484898	0,910252319
Capn2	1568,629046	-0,33925113	0,129234772	-2,625076254	0,008662956	0,096351967
Tspan33	377,6729953	-0,880829127	0,178873496	-4,924313252	8,47E-07	0,000113178
Mpp3	1,387121355	-0,961030622	1,277381061	-0,752344504	0,451843911	NA
Doc2b	2,332545441	-0,125438334	0,939940066	-0,133453545	0,893834701	NA
Itgb3bp	63,17978908	0,375190699	0,202612163	1,851767899	0,064059159	0,308060036
Umad1	314,7989189	0,033144707	0,129438216	0,256065849	0,797899991	0,938325224
P2ry2	232,0438146	0,031366247	0,186855873	0,167863319	0,866690808	0,961775826
Atg10	98,31043589	0,093032677	0,183547476	0,506858928	0,612253806	0,858888222
2310011J03Rik	320,7928223	0,032339896	0,105740278	0,305842733	0,75972439	0,924925298
Bcs1l	448,942398	-0,15013043	0,098480333	-1,524471178	0,127391051	0,444323237
Azin1	1804,965478	-0,086958511	0,116077056	-0,749144698	0,453769997	0,776283856
Rngtt	245,9542915	-0,226032912	0,120478868	-1,876120817	0,060638692	0,298482574
Ifi211	28,12017704	-0,119335811	0,298399858	-0,399919126	0,689216084	0,894327388
Kcnk3	6,753384121	0,2349984	0,551038624	0,42646448	0,669769425	0,887039558
Aqp4	99,22398023	-0,488118039	0,240802429	-2,027047825	0,042657522	0,243203202
Usp10	1949,847171	-0,253184212	0,119670923	-2,115670255	0,034372862	0,216862101
Ednra	99,78350367	0,152170146	0,192049991	0,792346544	0,42815864	0,760002028
Micu3	46,1014061	-0,074036663	0,234043524	-0,316337159	0,751746609	0,920844825
Mrpl58	640,0764003	-0,023669157	0,100253439	-0,236093217	0,813360338	0,943900659
Ndufs3	2234,969837	-0,002524215	0,090885766	-0,02777349	0,97784281	0,994354646
Uxs1	218,3929848	-0,401835003	0,167308704	-2,401757916	0,016316502	0,141031297
Dcxr	2432,803251	0,556478606	0,106510352	5,224643359	1,74E-07	3,33E-05
Nupr1l	3,870301632	0,843895317	0,781739481	1,079509654	0,280360593	NA
Mrrf	598,029509	-0,057349333	0,08753847	-0,655132909	0,512382185	0,810227859
Paip2	1795,323814	0,011517103	0,074905254	0,153755615	0,877802432	0,965046146
Eef1b2	4364,325197	-0,020020762	0,132626187	-0,150956322	0,880010171	0,96546599
Angptl2	384,6244892	0,010398002	0,180126019	0,057726262	0,953966675	0,986508391
Slit3	2,24656098	1,524375612	1,05293051	1,447745694	0,147688185	NA
Pmel	8,681547116	-0,588390173	0,481012609	-1,223232329	0,221241958	0,575780366
Best1	0,800959233	0,668824129	1,911385999	0,349915784	0,726401901	NA
Tekt2	5,397936109	0,453567601	0,805097037	0,563370104	0,57318289	0,84235265
Nfatc2ip	87,73374171	-0,100116237	0,197629291	-0,506586026	0,612445315	0,858888222
Tstd1	308,7090198	0,122721475	0,195918735	0,626389685	0,531059373	0,819884984
Mup11	119,0188684	-1,512261133	0,930898905	-1,624517039	0,104265507	0,401673966
Oas1c	34,85167447	0,21545476	0,36038994	0,597837885	0,549948109	0,829638487
Spred2	135,5437671	-0,348885354	0,188868551	-1,847239005	0,064712509	0,309828757
Laptm4b	488,3878005	-0,137803731	0,143572843	-0,959817531	0,337147057	0,69270916
Ly6g6d	2,53580307	0,165999302	0,932946514	0,177930138	0,858777841	NA
D17H6S53E	190,2725487	0,119734327	0,134598132	0,889569004	0,373697356	0,720823634
Clic1	475,7606654	0,042350491	0,135411409	0,312754233	0,754467389	0,922298112
Jmjd6	401,835445	0,012986196	0,133915894	0,096972775	0,922748015	0,977445106
Atg4a	77,29154137	-0,414597664	0,399955569	-1,036609305	0,299917975	0,660125684
Fgd5	100,8177515	-0,237228703	0,242358247	-0,978834871	0,327661577	0,685077831
4930442L01Rik	0,368315808	0,66341466	2,61309566	0,25388074	0,799587694	NA
4632427E13Rik	4,805888527	0,250203323	0,69213153	0,361496785	0,717728108	NA
Phactr1	13,41126878	-0,385316758	0,444423513	-0,867003538	0,385940071	0,730226156
Zbed3	101,3146536	0,138981421	0,15849301	0,876893061	0,380544726	0,725866642
Cpeb3	205,1344028	-0,099325781	0,204958634	-0,484613794	0,627950327	0,865852703
Ptar1	24,86993601	-0,038277931	0,30812786	-0,124227426	0,901135208	0,972526743
Gabpa	954,7461913	0,127316673	0,147838427	0,861187957	0,389134532	0,73293034
Galr2	0,374512941	-2,130054738	3,003457951	-0,709200786	0,478199886	NA
Serpina3m	7044,213826	0,7652928	0,168183387	4,550347168	5,36E-06	0,000487082
Kcnk7	0,244992664	1,389394708	3,349408814	0,414817893	0,67827522	NA
Serpina3n	9750,601541	0,761867915	0,097035978	7,851396268	4,11E-15	7,86E-12
1700003F12Rik	1,318047497	2,962947992	1,607335271	1,843391386	0,065271887	NA

Supplementary Table S1: *Serpina1* KO vs. wildtype all DEGs

Ascc2	620,6190761	-0,258721373	0,096242297	-2,688229409	0,007183203	0,085973355
Twf2	78,64326362	0,29241988	0,1964373	1,488616879	0,136588284	0,459349518
Tmem43	307,5895752	-0,131541113	0,114186743	-1,151982353	0,249328325	0,608508653
Acoxl	0,173498105	0,059593471	3,352475198	0,017775962	0,985817581	NA
Cbx6	94,84745621	0,26767361	0,196934173	1,359203466	0,174082123	0,517013028
Crtc2	809,892287	0,012270541	0,109703653	0,111851705	0,91094099	0,974815504
Parn	488,6594021	-0,103080569	0,120321879	-0,856706778	0,391606957	0,734532481
Rnf138rt1	0,413979771	1,038228131	2,527692918	0,410741401	0,681262165	NA
Bcl2l12	131,4341761	0,082024743	0,156850646	0,522948075	0,601010388	0,854422974
Frat1	157,3998647	0,214752687	0,273084471	0,786396554	0,431635202	0,7622164
Dusp8	28,22830808	-0,234286607	0,483928988	-0,484134269	0,628290582	0,865852703
Rad54l2	571,8843742	-0,264268005	0,136746395	-1,932540926	0,053292776	0,278635557
Cemip	0,213116452	1,337107854	3,350237755	0,399108348	0,689813372	NA
Trank1	2,357985543	2,469011604	1,133293563	2,178616102	0,029360199	NA
Clk4	521,1094349	0,051051215	0,093568959	0,5455999	0,585340993	0,847482024
Cfap46	0,885431383	1,526954037	1,915892381	0,796993637	0,425454727	NA
Mnat1	186,2569644	0,235972976	0,129168976	1,826854895	0,06772159	0,318792229
Ppm1g	1132,038794	-0,214407657	0,103299305	-2,07559631	0,037931303	0,228686669
Ehf	28,34899053	0,626510763	0,437320416	1,432612655	0,151968563	0,485352203
Itga10	37,46273763	-0,791488637	0,294703535	-2,68571138	0,007237558	0,086237888
Agpat9	95,74909507	0,374067402	0,380022803	0,98432883	0,324953858	0,682335948
Pacs2	471,7694228	0,077213493	0,099598867	0,77524469	0,438195085	0,766215379
Il12b	1,156315993	0,475757356	1,350412601	0,352305181	0,724609403	NA
Osbp2	28,21410025	1,864459306	0,443460194	4,204344223	2,62E-05	0,001682953
Gm4956	640,6060928	-0,685976719	0,90984124	-0,753952106	0,450877981	0,774175112
0610040B10Rik	29,75071612	0,407052273	0,283278084	1,436935281	0,150736384	0,483144262
Nespas	0,234744367	0,059593471	3,352475198	0,017775962	0,985817581	NA
Tsix	3,081440544	-0,551546292	1,002874327	-0,549965512	0,582343029	NA
Gas5	1765,086353	-0,027843929	0,170515446	-0,163292707	0,870287976	0,961775826
Meg3	34,07726172	0,504291686	0,354364772	1,423086397	0,154711111	0,489743509
Xist	953,4621576	11,00110224	2,886332051	3,811447211	NA	NA
Dph5	179,2677968	0,097340038	0,176767492	0,550667077	0,581861918	0,846177741
Cnr2	44,8831001	-0,09022191	0,402378597	-0,224221443	0,822584999	0,946967955
Gm12338	22,42681845	-0,225892746	0,369144971	-0,611935049	0,540580731	0,826407188
Tmem265	59,36688078	0,469596471	0,210514329	2,230710249	0,025700328	0,183933452
4921529L05Rik	0,415069136	-1,102913832	2,488874704	-0,443137547	0,657666248	NA
Zc3h7a	804,1115772	-0,307567213	0,121910849	-2,522886309	0,011639603	0,115306358
Psmd2	4497,04909	-0,034019588	0,092221639	-0,368889433	0,71221014	0,905478699
AA543186	11,87013461	-0,729559966	0,451916765	-1,614368002	0,106447628	0,40497035
A230072C01Rik	81,73116561	-0,030518011	0,195318142	-0,156247701	0,875837779	0,96416635
Gm5468	0,20013886	1,337107854	3,350237755	0,399108348	0,689813372	NA
LOC101056073	0,087021394	-0,517475177	3,352475198	-0,154356154	0,877328924	NA
Pdxk-ps	0,087021394	-0,517475177	3,352475198	-0,154356154	0,877328924	NA
Uckl1os	0,930651893	3,303025961	1,761470605	1,875152473	0,06077175	NA
AI854703	0,616172629	-0,081543763	1,840040784	-0,04431628	0,964652295	NA
Tmem51os1	3,61687596	1,211742801	0,881099821	1,375261658	0,169050339	NA
Gm5577	0,903133361	-1,993073185	1,794366155	-1,1107394	0,266680538	NA
Pigg	297,563253	-0,09760089	0,156566907	-0,623381353	0,533033948	0,820918853
Gm10941	2,96294254	0,960678527	0,90954533	1,056218415	0,290868439	NA
A930012L18Rik	1,756340426	-0,31786311	1,182618684	-0,268779036	0,788099727	NA
Bach2os	29,03881098	-0,377353455	0,340095678	-1,109550867	0,267192614	0,627508918
Zfp389	1,264097568	1,469673943	1,404210468	1,046619418	0,295275142	NA
Gm14379	2,034608834	1,348620157	1,14537459	1,177448992	0,239016345	NA
2700099C18Rik	3,885637155	-0,070613567	0,812106439	-0,086951123	0,930710363	NA
2610001J05Rik	349,8762509	0,2034297	0,111962613	1,816943123	0,069225823	0,321794167
Gm11346	1,208014762	-0,268143184	1,367833664	-0,196034935	0,844582822	NA
9230116N13Rik	4,275686937	0,193863724	0,700357981	0,276806618	0,781928599	NA
4930412O13Rik	0,093953095	-0,517475177	3,352475198	-0,154356154	0,877328924	NA
BC006965	0,713388223	2,889268111	2,668281978	1,082819632	0,27888849	NA
Btbd19	29,85319376	-0,865419116	0,472765265	-1,830547165	0,067168161	0,317560326
Snhg7	32,52924851	-0,020804182	0,309538607	-0,067210299	0,946414286	0,984653942
Snhg6	181,1462335	0,269079218	0,191471811	1,405320276	0,159926062	0,495885425
Gm6981	8,627782732	-0,525079854	0,544858271	-0,963699887	0,335196411	0,691456742
A930015D03Rik	5,236195439	0,635121928	0,629400571	1,009090167	0,312931393	NA
4732416N19Rik	5,531815549	0,571597602	0,661191317	0,864496533	0,387315184	0,731668319
9530059O14Rik	0,093953095	-0,517475177	3,352475198	-0,154356154	0,877328924	NA

2810047C21Rik1	1,475181527	-0,548910472	1,317840448	-0,416522708	0,677027555	NA
1810058I24Rik	1184,952101	0,076014562	0,133537362	0,569238156	0,56919453	0,840790976
2210417A02Rik	55,27759091	0,638397988	0,229034129	2,787348722	0,005314126	0,070973656
E130112N10Rik	4,204504527	0,878498952	0,896327411	0,980109435	0,327032102	NA
4930483J18Rik	0,113662148	0,780932884	3,352475198	0,232942181	0,815806304	NA
F730043M19Rik	0,92436811	-3,438262079	1,910683299	-1,799493448	0,071940659	NA
Fam120aos	246,6955322	0,418293859	0,140186236	2,983843997	0,002846519	0,047572443
5430405H02Rik	33,9887266	0,045606067	0,251188343	0,181561241	0,855927068	0,957884562
A230072E10Rik	2,139840819	0,061655902	1,261162426	0,048888154	0,96100843	NA
9330111N05Rik	0,511895373	1,37054747	2,45342665	0,558625818	0,576417116	NA
A330035P11Rik	37,28493814	0,081875223	0,249466798	0,32820088	0,742759782	0,917092327
BC024386	6954,132873	0,158006434	0,156432453	1,010061729	0,312465716	0,672017932
5430402O13Rik	15,51514724	0,303235915	0,375796493	0,806915233	0,419715322	0,753867914
2810032G03Rik	0,733810355	-1,353423227	1,747377699	-0,774545325	0,438608376	NA
Ube2d-ps	193,0395033	-0,055302011	0,153998183	-0,359108208	0,719514142	0,908631658
4930513N10Rik	1,689529099	-0,733316396	1,311512483	-0,559137946	0,576067579	NA
1700012D14Rik	10,97328571	0,275753485	0,443834992	0,621297308	0,534404025	0,821767588
1810014B01Rik	55,95937805	0,63164808	0,224157196	2,817880002	0,004834187	0,067181133
A930007I19Rik	4,380458857	0,372706233	0,721406477	0,516638324	0,605408658	NA
A330023F24Rik	120,0138191	-0,09074011	0,235828094	-0,384772265	0,700406149	0,89948941
2610028E06Rik	0,3267786	1,84016758	2,655545515	0,692952755	0,488339201	NA
2610035D17Rik	32,35732501	0,01346193	0,267067625	0,050406448	0,959798498	0,988106124
4933404O12Rik	118,7259499	0,23395537	0,181620156	1,288157523	0,197691135	0,547387188
Trmt61b	86,6322407	-0,168488639	0,184418165	-0,913622797	0,360915083	0,710581841
5830432E09Rik	1,744359813	0,714111337	1,223944072	0,583450954	0,559589765	NA
1700009J07Rik	0,300918618	-0,661123873	2,741506622	-0,241153484	0,809436161	NA
4930481A15Rik	193,7770936	0,382010958	0,148349889	2,575067365	0,010022057	0,104839494
Mir99ahg	20,77499353	0,450415034	0,344572966	1,30716881	0,191155386	0,540266029
9230114K14Rik	19,73085796	-0,167056353	0,34886594	-0,47885544	0,632041473	0,867306747
1110038B12Rik	228,2263558	0,090853244	0,199184692	0,456125634	0,648299638	0,876446263
Dancr	51,20868994	-0,067485248	0,339843607	-0,198577364	0,842593362	0,952289345
R74862	10,50448848	-0,297173952	0,431648934	-0,688462148	0,491161799	0,797760449
9530026P05Rik	2,205736954	-0,58099954	1,058873499	-0,548695893	0,583214165	NA
BC029722	443,2418925	0,179256522	0,087866471	2,040101528	0,041340215	0,240072963
9430060I03Rik	0,861576237	0,12603575	2,234523271	0,056403866	0,955020078	NA
Cep83os	53,98198205	0,465189046	0,258325117	1,800789063	0,071736133	0,328550999
2810429I04Rik	0,236587694	-1,308576888	3,335186821	-0,392354899	0,694796005	NA
1700030C10Rik	19,21381939	0,678071279	0,446996352	1,516950364	0,129279216	0,446717571
AI662270	53,1729864	-0,098267212	0,354536122	-0,277171228	0,781648631	0,932196631
6430562O15Rik	2,437424917	0,596185578	1,059731286	0,562581841	0,57371966	NA
9330175E14Rik	9,170598821	-0,26599113	0,604736228	-0,439846527	0,660048267	0,882506779
E030011O05Rik	0,509110889	1,486646382	2,096419285	0,709135998	0,478240086	NA
C030034L19Rik	0,478247382	1,147019579	2,148340809	0,533909505	0,593404145	NA
1500004A13Rik	0,423844164	-2,27808312	2,428184812	-0,93818358	0,348150075	NA
C130036L24Rik	1,531126897	0,717358621	1,126232488	0,636954295	0,524154615	NA
4833418N02Rik	46,73976166	0,458681798	0,281308951	1,630526849	0,102990195	0,399261309
A230056P14Rik	23,65470722	-0,004801758	0,354119782	-0,013559701	0,989181255	0,996575327
4930511M06Rik	1,755626596	-0,751982763	1,244671629	-0,604161568	0,54573623	NA
2810403D21Rik	7,614045156	1,167898496	0,609669803	1,915624638	0,055412891	0,284600439
A330040F15Rik	13,92201194	-0,352104681	0,512072991	-0,68760643	0,491700658	0,797760449
E030003E18Rik	6,802280081	0,042290908	0,580552515	0,072845965	0,941928694	0,983128178
C230035I16Rik	3,982264312	0,528479277	0,723803712	0,730141706	0,465303571	NA
A630089N07Rik	12,12881705	-0,496449493	0,510109184	-0,973222024	0,330442967	0,687043861
2900041M22Rik	0,481151143	2,354177818	2,89108379	0,814289031	0,415479392	NA
A530013C23Rik	6,15123786	-0,678803411	0,684266141	-0,992016659	0,321189399	0,678675688
D130017N08Rik	13,54783482	-0,296163591	0,428519893	-0,691131489	0,489482914	0,796616325
1700108F19Rik	1,325802538	0,065575695	1,3791218	0,047548879	0,962075775	NA
C230091D08Rik	0,31389621	-0,661096352	2,708970037	-0,244039743	0,80720004	NA
5730422E09Rik	1,455183849	-1,466197626	1,196061722	-1,225854485	0,220253439	NA
1500011K16Rik	202,2944872	0,484900508	0,194610674	2,491643948	0,012715343	0,121459059
1700086O06Rik	37,82582762	0,111606683	0,243546852	0,458255494	0,646768895	0,876230372
Il1bos	1,025280723	0,974034239	1,427148116	0,682503959	0,49492034	NA
9530082P21Rik	68,99137415	0,713064995	0,231622795	3,078561389	0,002080027	0,03900123
3830408C21Rik	9,517695587	0,403553494	0,517783282	0,779386875	0,435751853	0,765414075
2610203C22Rik	5,904493553	0,385682097	0,719045302	0,536380804	0,591695396	0,850578037
5530601H04Rik	57,23585193	0,192778629	0,226256677	0,852035093	0,394194615	0,736368577

Supplementary Table S1: *Serpina1* KO vs. wildtype all DEGs

C920009B18Rik	2,595378562	-0,344494796	1,105431696	-0,311638247	0,755315464	NA
A330069E16Rik	9,363496935	0,379313569	0,478272031	0,793091683	0,427724408	0,759900015
4933431E20Rik	122,2183636	-0,028873987	0,182345808	-0,158347416	0,874183047	0,963600184
1700047M11Rik	7,600381736	-0,026268531	0,649744879	-0,040428993	0,967751116	0,991231429
B430010I23Rik	2,432898609	1,576774591	1,328943671	1,186487151	0,235429973	NA
Snhg14	18,2966209	0,294228413	0,361452373	0,814017102	0,415635154	0,751551608
Ccdc8	11,4350223	-0,523535327	0,675432674	-0,775111047	0,438274045	0,766215379
Daam2	59,50981509	-0,095368043	0,240611397	-0,396357134	0,691841584	0,895338108
Themis2	103,3229512	0,194207143	0,280989849	0,69115359	0,489469026	0,796616325
Brms1l	102,4700177	0,034515642	0,184123631	0,187459057	0,851300722	0,955747343
Shb	779,4553202	-0,183123985	0,161305573	-1,135261367	0,256265824	0,616816726
Uap1l1	111,510291	0,014743105	0,273737448	0,053858559	0,957047854	0,98781716
Tspyl5	5,204084032	-0,359789203	0,709847583	-0,506854164	0,612257149	NA
D8Ertd738e	1440,032198	-0,037528689	0,127618407	-0,29406956	0,768704751	0,927598042
Zfp575	0,359560173	-0,130902947	3,173808443	-0,041244754	0,967100776	NA
Myo6	1252,066922	-0,132780271	0,120696269	-1,100119101	0,271280233	0,633246849
Aunip	2,162855655	-1,952895893	1,407351236	-1,387639307	0,165246906	NA
1700057G04Rik	0,394221993	-1,149575818	2,531438941	-0,454119513	0,64974281	NA
Tmem181a	76,82500631	0,176133264	0,226642088	0,77714279	0,437074531	0,76575019
Nynrin	36,10103976	-0,18315654	0,303078652	-0,604320162	0,545630804	0,827795985
A4gnt	0,12663974	0,780932884	3,352475198	0,232942181	0,815806304	NA
Trim63	0,295445946	1,389394708	3,349408814	0,414817893	0,67827522	NA
Mon1b	329,8907544	-0,066667726	0,123094609	-0,541597448	0,58809585	0,848706754
A430105I19Rik	48,97492225	-0,187364586	0,276783766	-0,676934881	0,498447272	0,801990802
Ripply1	12,0754773	1,612019083	0,512125948	3,14770046	0,001645602	0,033742419
Zfp69	26,95127889	0,277320372	0,323756228	0,856571543	0,391681718	0,734532481
Slc25a42	2122,323939	-0,204001713	0,109946494	-1,855463559	0,063530058	0,306618538
Parl	587,3077752	0,117344528	0,098878613	1,186753369	0,235324917	0,592813043
Lif	2,067332697	-0,787970561	1,062890413	-0,741346945	0,458483104	NA
Gm5544	0,901822716	2,345391775	1,918458251	1,222539909	0,221503522	NA
Zbtb9	238,7687001	-0,058410231	0,132405226	-0,441147473	0,659106237	0,881654058
Clec4a4	0,086476712	0,780932884	3,352475198	0,232942181	0,815806304	NA
Ifit3b	92,5899507	0,344994069	0,272964381	1,263879438	0,206273303	0,558880735
Gm609	8,013807672	1,410211017	0,723587929	1,948914513	0,051305635	0,27322396
D5Ertd579e	1048,683814	-0,263105441	0,121978199	-2,156987418	0,031006645	0,204100361
Ces1a	7,110313942	-0,08061973	0,732220626	-0,110103057	0,912327644	0,975956816
Lingo3	0,093953095	-0,517475177	3,352475198	-0,154356154	0,877328924	NA
Grhl3	4,009359059	0,149412642	0,681978058	0,219087168	0,826582146	NA
Pcdh17	17,66494729	-0,145445244	0,519657369	-0,279886812	0,779564345	0,931533405
Tmem151b	0,310402523	-0,664825715	3,267927442	-0,203439558	0,838791483	NA
Ddn	0,227324296	1,389394708	3,349408814	0,414817893	0,67827522	NA
Vmo1	156,9622726	-0,194601857	0,160080788	-1,215647797	0,224119118	0,579427376
Srsf1	2267,17943	-0,095594456	0,113006694	-0,845918522	0,397598199	0,738775583
Sdhaf3	123,3797705	0,09163628	0,15278763	0,59976243	0,548664575	0,828986591
Mir682	51,69053633	0,145505176	0,326386807	0,445805935	0,655737434	0,879592859
Lipt1	195,0852953	-0,087500831	0,157311767	-0,556225594	0,578056645	0,845288649
Gm6377	10,61491769	0,293785516	0,595726749	0,493154818	0,6219032	0,863008499
Ccdc17	96,42582352	0,372538301	0,194251529	1,917813997	0,0551346	0,284116184
Gnpda2	63,15945086	0,372912836	0,180903481	2,061391163	0,039265737	0,232687784
Ccdc173	9,388524094	0,274805464	0,566363206	0,485210658	0,627526923	0,865852703
Ccdc107	1273,748989	0,194205932	0,124681154	1,55762058	0,119323217	0,429750025
Nell1	0,147722973	0,780932884	3,352475198	0,232942181	0,815806304	NA
Mup3	173839,1499	-0,642893451	0,350879331	-1,832235174	0,066916391	0,317011065
Pbxip1	397,5480312	0,044728756	0,137661211	0,324919093	0,745242306	0,918622973
Haus3	109,1847855	-0,129006421	0,182077192	-0,70852598	0,478618685	0,791129228
Tor4a	131,4376609	0,500106819	0,180653939	2,768313939	0,005634715	0,073493174
Guca1b	5,507068052	-1,133181122	0,667108515	-1,698645867	0,089385929	0,370643577
Kdm5a	488,7722888	-0,051278046	0,168843227	-0,30370212	0,761354843	0,92534041
Paip2b	584,3707504	-0,118586055	0,129183568	-0,91796547	0,358636944	0,707901565
Tfb1m	270,8414442	0,11738851	0,104540896	1,12289558	0,261481845	0,622343615
Ell3	68,74346785	1,320957875	0,395474017	3,340188779	0,000837215	0,020998249
Gm4925	2,653380993	-0,079264732	0,872284488	-0,090870275	0,927595669	NA
2610507B11Rik	3678,612544	-0,155587888	0,070676172	-2,201419294	0,027706354	0,191721662
F11	2196,242535	-0,501668909	0,127443966	-3,936388071	8,27E-05	0,003935395
H2-K1	36922,56503	0,27724007	0,103733684	2,672613752	0,007526285	0,0882622
Tmem151a	8,114262182	1,800509729	0,625709773	2,877547719	0,004007793	0,059165556

Prr7	2,426286742	0,095820153	0,864607924	0,110824977	0,911755139	NA
Gm4981	0,086476712	0,780932884	3,352475198	0,232942181	0,815806304	NA
Otud7b	679,9832195	-0,13156738	0,094435035	-1,393205182	0,163557753	0,501054904
Snx22	75,07116494	0,27627782	1,421044065	0,194418897	0,845847895	0,953584926
Trim43a	0,087021394	-0,517475177	3,352475198	-0,154356154	0,877328924	NA
Tmem171	13,00368752	0,55676666	0,52204492	1,066511019	0,2861927	0,64794415
Gm527	19,94063453	-0,032608848	0,363816557	-0,089629918	0,928581309	0,979424341
Nr2c2ap	273,8775042	-0,336792571	0,140785304	-2,392242386	0,016745779	0,143473504
Kcnc2	0,259430135	1,627812584	3,345388605	0,486584005	0,626553161	NA
Ccdc141	269,318108	0,305492474	0,24955034	1,224171744	0,220887446	0,575078806
Ankrd12	98,33521312	-0,341484306	0,25155935	-1,35747014	0,174631875	0,517225036
AW146154	114,1503878	-0,091127141	0,149207088	-0,610742712	0,541369925	0,826407188
Usp36	336,2383175	-0,196931924	0,150993879	-1,3042378	0,192152515	0,541552905
Kcnk6	74,89129611	-0,081756401	0,267553884	-0,305569855	0,759932176	0,924925298
Gramd2	0,387627262	0,666608971	3,05922977	0,217900916	0,827506313	NA
Zfp213	139,5656072	0,251520299	0,187377681	1,342317282	0,179493142	0,522135977
Wdr72	0,756925011	2,120694874	1,97869724	1,071763194	0,283826408	NA
Pusl1	151,3747255	-0,146801266	0,194640109	-0,754218987	0,450717738	0,774175112
Rnf207	0,179780087	0,059593471	3,352475198	0,017775962	0,985817581	NA
Fbxo11	690,7235917	0,159614474	0,089561664	1,782174059	0,074720852	0,338051799
Slc16a5	345,4401307	1,177339953	0,52302106	2,251037373	0,02438317	0,177536802
Selenoo	1655,85899	0,220970095	0,094387852	2,34108617	0,019227729	0,154666369
Gm8909	1,140321134	1,201061681	1,487503067	0,80743476	0,419416047	NA
Zfp541	0,229655992	-1,252384348	3,339190293	-0,375056298	0,707618598	NA
Ccdc154	0,509183582	-0,542260133	2,886713761	-0,187846866	0,850996696	NA
Tmem243	1624,041222	0,134276323	0,101614537	1,321428286	0,1863586	0,532805747
Kcnt2	18,41151403	-0,204377864	0,362969885	-0,563071132	0,573386448	0,842372044
Lrp8	0,658264355	0,871532425	1,842636888	0,472981102	0,636226662	NA
Maats1	1,014141228	-1,193487108	1,564740481	-0,762738053	0,445619631	NA
Rapgefl1	10,42326261	0,235665508	0,461911288	0,510196468	0,609913826	0,858128599
Sco1	338,1332388	-0,099577453	0,100321888	-0,992579537	0,320914901	0,678403192
Cntnap3	0,339756192	1,876228608	3,160061454	0,593731684	0,552691588	NA
Qrich2	0,946697834	0,303085004	1,614630745	0,187711652	0,851102696	NA
Gm525	0,12663974	0,780932884	3,352475198	0,232942181	0,815806304	NA
Mär-09	11,39764587	-0,283927157	0,516596883	-0,549610666	0,582586442	0,846891345
Zfc3h1	931,8618819	-0,10418549	0,137199773	-0,759370713	0,447630828	0,772775895
Stox1	1,984073939	-1,307863951	1,203811068	-1,086436224	0,277286042	NA
Arfgef3	0,367036557	-1,148477416	2,582636864	-0,444691792	0,656542501	NA
Spata5l1	68,28257933	-0,031224157	0,185764601	-0,168084538	0,866516773	0,961775826
Zfp583	5,450152922	0,225413165	0,647787978	0,347973678	0,727859952	0,910785759
Cfap44	1,842661262	0,956034436	1,207921286	0,791470808	0,428669305	NA
Fancd2	6,989709983	-0,200425447	0,689517043	-0,290675117	0,771299804	0,928273961
Ccdc114	5,888790642	-0,058905968	0,646618995	-0,091098419	0,927414389	0,978888597
Cln5	867,2566298	0,076914237	0,093954327	0,818634316	0,412995082	0,749760726
Iqsec1	1023,053564	-0,200389664	0,109804921	-1,824960679	0,068006963	0,319375503
Nol4l	40,38216902	-0,287460225	0,284981688	-1,008697182	0,313119884	0,672219008
Arhgef33	0,187906191	-1,241948083	3,339949325	-0,371846385	0,710007226	NA
Zkscan17	340,0878854	0,27724806	0,144173434	1,923017661	0,054477832	0,28262497
Slc26a8	0,812073152	3,116242041	1,80973613	1,721931717	0,085081889	NA
Dnm3	12,36027112	0,609149787	0,423115569	1,43967708	0,149958781	0,482038697
Papolg	119,8661061	-0,082644159	0,164355198	-0,502838733	0,615077659	0,859955845
Wdhd1	46,6869777	-0,399110228	0,276155051	-1,445239646	0,148390584	0,478955508
Polrmt	593,4506068	-0,119756472	0,113007138	-1,059724847	0,289269796	0,650613711
Cabin1	445,2781523	-0,048782746	0,112384129	-0,434071491	0,664236527	0,884226961
Ybey	101,1315824	0,168068567	0,155422574	1,081365225	0,279534684	0,640243137
Fam117a	81,86177326	-0,186364424	0,224189701	-0,831280042	0,405815447	0,745391028
Ino80c	285,7163611	0,067349546	0,117678834	0,57231656	0,567107533	0,838772055
Zfp609	456,6695788	0,105780026	0,178738882	0,591813181	0,553975696	0,832018326
Iqub	0,447267411	1,200692607	2,506185436	0,479091687	0,631873404	NA
Aldh5a1	1800,068582	0,015822736	0,085458428	0,185151265	0,853110393	0,95633346
She	66,40110582	-0,690426566	0,350332146	-1,970777085	0,048749379	0,26439369
Rbm27	378,8428072	-0,077696586	0,144197604	-0,538820231	0,590010903	0,84953676
Mtmr12	189,4706313	0,044031797	0,158218677	0,278297089	0,78078431	0,93182516
Ankrd13b	80,56622389	-0,075132014	0,178589518	-0,420696658	0,673976602	0,888627551
Alkbh5	1304,141454	-0,131170331	0,097481881	-1,345586781	0,178435817	0,520853371
Tbcel	1787,61945	-0,214197613	0,124085931	-1,726203851	0,084310746	0,359537594

Scml4	2,827569824	0,994124685	0,900947942	1,103420785	0,269844475	NA
Tango6	184,3688353	-0,258797827	0,132820906	-1,948472085	0,051358502	0,27322396
Rln3	0,142634598	-0,517475177	3,352475198	-0,154356154	0,877328924	NA
Sap130	610,9928939	0,077657435	0,128110333	0,606176198	0,544397757	0,827477215
Rbbp8nl	0,592454212	0,490227921	1,908274537	0,256895909	0,797259129	NA
Adcy10	0,180324769	-1,201310802	3,342952034	-0,359356278	0,719328578	NA
Vps13a	327,1624433	-0,167520558	0,119841207	-1,397854388	0,162156798	0,498749809
Ip6k3	1,124704929	1,864285693	2,107365462	0,884652295	0,376344192	NA
Kdm1b	689,6589526	-0,131788061	0,158169812	-0,833206153	0,404728473	0,744675881
Serpina5	7,150769454	0,550586318	0,690684441	0,797160447	0,425357854	0,758214553
Zbtb11	234,5475682	0,14891996	0,153154378	0,972351962	0,330875482	0,687536562
Klhl31	0,093953095	-0,517475177	3,352475198	-0,154356154	0,877328924	NA
Peak1	288,6832754	-0,254198848	0,144197597	-1,762850789	0,077925659	0,345649679
Serpina3b	0,087021394	-0,517475177	3,352475198	-0,154356154	0,877328924	NA
Tmem266	34,58323521	-0,006069848	0,366970994	-0,016540403	0,98680327	0,996116952
BC048403	115,9051831	0,187188535	0,22283694	0,840024704	0,400894535	0,741644961
Nxpe4	13,7746976	0,783078706	0,43821703	1,786965482	0,073943068	0,335256454
Pfkfb4	34,44598517	-0,019970368	0,312569786	-0,063890909	0,949057091	0,985650844
Dpy19l1	1278,879787	-0,254171801	0,149431255	-1,700927971	0,088956514	0,369687145
Zfp846	90,31356975	-0,119923552	0,208644548	-0,574774434	0,56544386	0,837608749
Snx14	582,7589757	0,082231172	0,089365146	0,920170506	0,357483664	0,707149983
Zfp317	281,1715257	0,160320227	0,132394256	1,210930379	0,225922088	0,581158101
Vat1l	0,173498105	0,059593471	3,352475198	0,017775962	0,985817581	NA
Ccdc113	0,086476712	0,780932884	3,352475198	0,232942181	0,815806304	NA
Bmt2	154,3404242	-0,19958292	0,189615228	-1,052567997	0,292539031	0,654074594
Tox3	2,702332146	1,033426927	1,081163615	0,955846934	0,339149572	NA
Letm2	60,63257372	0,064517153	0,215168343	0,299845006	0,764295384	0,925950611
Abcc12	7,983882605	-0,728954842	0,673005055	-1,083134274	0,278748828	0,639188747
Prag1	34,17822333	-0,015569654	0,294964766	-0,052784794	0,957903378	0,987896001
Idh2	4049,118537	0,285079037	0,072771656	3,917446056	8,95E-05	0,004154234
Iyd	2466,996209	0,12964539	0,082166576	1,577836106	0,114603267	0,41907305
Whamm	1270,42919	-0,399237767	0,211166559	-1,8906297	0,058673793	0,29301828
Slc28a1	5,452717051	1,12918218	0,772007596	1,462656826	0,143561305	0,470584709
AA415398	133,4325195	0,225753637	0,144588731	1,561350151	0,118441157	0,427494553
Zfp36l3	0,407765529	-0,919879351	3,064390271	-0,300183485	0,764037202	NA
Cdkl5	42,88277288	0,138118302	0,261006208	0,529176311	0,596683148	0,853161177
Nova2	14,38275013	-0,323388541	0,426786476	-0,757729121	0,448613162	0,77297141
Urb2	190,2898918	-0,0624472	0,147491122	-0,42339633	0,672006122	0,887904019
Unc13a	7,214089254	-0,573763884	0,545361699	-1,052079539	0,292763059	0,654074594
Cep44	191,1480852	-0,20566464	0,130313076	-1,578234866	0,114511663	0,418961344
Rmdn3	1813,601983	-0,022954308	0,135880189	-0,168930495	0,865851312	0,961775826
Ccdc64	20,41389885	0,007197185	0,327339249	0,021986929	0,982458382	0,995338444
2810408A11Rik	3,329835453	-0,778108378	0,957776879	-0,812410902	0,416555892	NA
Rap1gap2	14,35943376	0,663862969	0,402863211	1,647861981	0,099380997	0,392688228
Nrbf2	362,0596639	0,108500208	0,134338348	0,807663702	0,419284204	0,753719022
Slc22a28	303,0773742	-1,770944424	0,768336832	-2,304906326	0,02117182	0,16249252
Sp140	65,21221221	0,7285858	0,241846237	3,012599278	0,002590207	0,044679824
Gm5622	0,439754904	0,241146527	2,563116173	0,094083339	0,925042955	NA
Amt	1289,747074	0,074219293	0,112170525	0,661664846	0,508186039	0,807274415
Zyg11b	989,8059874	-0,094714851	0,124936667	-0,758102908	0,448389379	0,77297141
Ankrd13c	376,5890226	-0,017013215	0,116422688	-0,146133159	0,88381627	0,966680824
Akr1c19	1927,820298	0,172356165	0,131745512	1,30825075	0,190788274	0,539591376
Ccdc92b	3,36244362	0,47071711	0,999415201	0,470992545	0,637646058	NA
Fam71b	0,093303375	-0,517475177	3,352475198	-0,154356154	0,877328924	NA
Aim2	32,91845369	1,068082038	0,320734303	3,330114765	0,000868102	0,021412648
Tvp23a	6,190254845	0,438867061	0,596680129	0,735514792	0,462026035	0,781516914
Gal3st4	0,602515493	1,671696616	2,057167531	0,812620553	0,416435644	NA
Zfp985	0,818889705	-0,721007265	1,937692984	-0,372095719	0,709821583	NA
Dscaml1	0,180429807	0,059593471	3,352475198	0,017775962	0,985817581	NA
Klri1	0,462252524	2,30185817	2,424586724	0,94938166	0,342426534	NA
H19	12,62867936	-0,021135562	0,886241574	-0,023848534	0,980973426	0,994882668
Ralgapa2	664,1083599	-0,07956304	0,13326715	-0,597019141	0,550494601	0,830088239
D430041D05Rik	0,235937973	-1,303449117	3,335546383	-0,390775294	0,695963339	NA
Lrrc55	0,259974817	0,586045239	3,35071904	0,174901337	0,86115716	NA
Sec31b	171,6224471	0,150737371	0,184194717	0,818358819	0,413152329	0,749855212
Nom1	508,8637214	0,03093604	0,11041545	0,280178543	0,779340528	0,931515737

Slc4a8	10,84813411	0,654530626	0,455983317	1,435426696	0,151165543	0,483843582
Gm5127	0,087021394	-0,517475177	3,352475198	-0,154356154	0,877328924	NA
Cracr2a	0,735257163	1,128850767	1,773894088	0,636368752	0,524536103	NA
Stxbp5	358,1712558	-0,237022582	0,13288626	-1,783650038	0,074480549	0,337078695
Fbxo40	2,837866244	0,126598178	1,277345509	0,099110364	0,921050644	NA
Alpk2	0,295994437	0,617282395	2,762021179	0,223489378	0,823154649	NA
Helq	98,78992172	-0,274468025	0,164011214	-1,673471089	0,094234593	0,381417584
Tomm40l	354,319756	-0,483704418	0,219012035	-2,208574601	0,027204244	0,190567596
Slc25a43	1,262814507	0,411189813	1,243228777	0,330743481	0,740838256	NA
Gm7457	1,888487593	-0,220137586	1,427507979	-0,154211107	0,877443285	NA
Gm4961	0,686207721	1,990976729	2,255293994	0,882801415	0,377343576	NA
AW549542	121,9135498	0,088782588	0,29556913	0,300378419	0,763888523	0,925867457
C330013E15Rik	40,59075759	0,111505744	0,251350258	0,443626932	0,657312331	0,880359254
1700028E10Rik	40,06234587	0,188500918	0,272756861	0,691095058	0,489505806	0,796616325
Gm9895	17,10492786	0,267647607	0,422735287	0,633132873	0,526646877	0,817815356
Gm16062	15,29260722	0,356539671	0,418223296	0,852510307	0,393930921	0,736368577
Tk2	495,0715191	-0,017503653	0,102126435	-0,171391995	0,863915549	0,961272625
A230056J06Rik	0,639894037	1,849293552	2,293471156	0,806329544	0,42005286	NA
D630010B17Rik	0,400277719	2,124816161	2,974820356	0,71426705	0,475062063	NA
1700120C14Rik	1,977109506	0,600826517	1,093977136	0,549213048	0,582859251	NA
Zfp572	0,427903795	-2,257267924	3,274223637	-0,689405543	0,490568096	NA
Gm15706	18,87692516	0,702730611	0,384388904	1,828176113	0,067523125	0,318406623
5031425E22Rik	151,5157015	0,1389986	0,170844282	0,8135982	0,41587517	0,751551608
1700016L21Rik	0,093953095	-0,517475177	3,352475198	-0,154356154	0,877328924	NA
A230001M10Rik	11,19604207	1,067709476	0,556548151	1,918449419	0,05505405	0,284066998
Platr4	24,0603056	-0,3100613	0,358112912	-0,865819939	0,386588913	0,731019403
AI646519	0,174042788	-1,166820872	3,345560292	-0,348766954	0,727264274	NA
Kantr	40,10684556	-0,02565068	0,32466394	-0,079006865	0,937027163	0,982210769
Rnf8-cmtr1	7,168613457	0,106584243	0,580768335	0,183522821	0,854387814	0,957135493
Trim72	6,702161066	0,441477697	0,694534072	0,635645844	0,525007282	0,816743321
Tmem109	569,9413386	-0,212946289	0,092489074	-2,302393999	0,021312963	0,163264046
Wdr74	489,417567	-0,257130094	0,148825482	-1,727728944	0,084036832	0,358827341
Lars	726,2107082	-0,010050675	0,107340853	-0,09363327	0,92540048	0,978307688
Fbxo38	638,1879153	-0,085882863	0,091255789	-0,941122349	0,346642164	0,698497639
Slc39a3	213,1358084	0,19415676	0,118911293	1,632786555	0,102513896	0,398751316
Smim3	18,13939037	1,002286051	0,38088489	2,631467086	0,00850171	0,095431875
Gstt3	2368,10658	1,933115913	0,278299926	6,946160351	3,75E-12	3,14E-09
Trip12	2619,509781	-0,050379606	0,095833689	-0,525698281	0,599097859	0,85430922
Ift140	256,4035134	-0,307628458	0,140165956	-2,194744478	0,028181932	0,193277974
Galnt10	176,1821859	-0,031339293	0,154524909	-0,202810623	0,839283055	0,951362994
Syt12	17,02621028	0,824328125	0,378258369	2,179272668	0,029311418	0,19731337
Nrm	23,63256738	-0,32622739	0,321840819	-1,013629627	0,310759515	0,669979674
Pcdh18	30,95284924	-0,317759392	0,334461211	-0,950063509	0,342079983	0,695250579
Sdpr	221,3529766	-0,047891332	0,318231164	-0,150492274	0,880376246	0,96546599
Stab2	764,2484125	-0,012808179	0,244736959	-0,052334469	0,958262188	0,987896001
Stk38	1430,323407	-0,123744886	0,076962174	-1,607866305	0,107864463	0,407932109
Sft2d1	281,9603696	0,040669792	0,105822228	0,384321822	0,700739936	0,899663133
Fbxo4	287,744396	0,077573269	0,136971953	0,566344186	0,571159829	0,8413217
Fam207a	716,2341218	-0,078467326	0,10907311	-0,719401197	0,471893759	0,786445858
Topors	644,3637874	-0,099849409	0,118769216	-0,840701086	0,400515412	0,741413811
Apof	17436,72877	0,094171491	0,086209342	1,092358306	0,274675642	0,636474647
Apon	8230,57772	0,355118102	0,09425099	3,767791761	0,000164698	0,006550662
Upb1	8873,626909	0,149155217	0,070861184	2,104893097	0,035300615	0,219460476
Desi1	1240,373433	-0,085104042	0,092706646	-0,917992894	0,358622586	0,707901565
Pwp1	411,4936027	0,16442352	0,124413454	1,321589539	0,186304869	0,532805747
Letmd1	721,885709	0,003869112	0,102547665	0,037729888	0,969903046	0,991981655
Pcnx3	901,0742461	-0,208465978	0,10014324	-2,081677984	0,037371893	0,226546639
Utp6	955,564046	-0,194779848	0,09670658	-2,01413231	0,043995644	0,247168435
Pla1a	1486,145973	-0,008308352	0,07381149	-0,112561774	0,910377992	0,9747592
Slc23a4	0,469492543	0,36867987	2,490308645	0,148045854	0,882306581	NA
Pdzd2	19,14320299	0,004444872	0,382927904	0,011607595	0,990738687	0,99719344
Kdm6b	500,1701952	-0,065851447	0,139931028	-0,470599321	0,637926892	0,870273408
Pstk	195,4349214	0,037484193	0,111722876	0,335510453	0,737240061	0,914979797
Rabl6	469,7760535	-0,342201604	0,132514736	-2,58236641	0,009812534	0,103375706
1700007G11Rik	0,087021394	-0,517475177	3,352475198	-0,154356154	0,877328924	NA
Pdp2	418,1106406	-0,361300454	0,161439558	-2,237992084	0,025221571	0,181772069

Supplementary Table S1: *Serpina1* KO vs. wildtype all DEGs

Ankrd28	596,9459782	0,038757636	0,162035362	0,239192456	0,810956352	0,942672417
Cep170b	940,0485071	-0,240604173	0,097146147	-2,476723777	0,013259448	0,124135542
Cdhr3	0,093303375	-0,517475177	3,352475198	-0,154356154	0,877328924	NA
Diras2	0,335612783	1,865434111	2,640376653	0,706503032	0,479875368	NA
Ak8	0,944325409	0,233626367	1,488381246	0,15696675	0,875271053	NA
Gm5431	16,37263031	0,524959018	0,412514849	1,272582113	0,203166369	0,55567332
Mki67	61,22945648	-1,405082298	0,454921088	-3,088628633	0,002010826	0,038349124
Erich1	62,43454398	-0,200223828	0,208162322	-0,961863924	0,336117962	0,69270916
Hs3st6	35,22523819	-0,314472139	0,339374462	-0,926622874	0,354122374	0,704551502
Hspb6	534,6088666	-0,423404378	0,185066865	-2,287845415	0,022146525	0,166616147
AU022252	677,1924205	0,069966622	0,110001892	0,63604926	0,524744316	0,816659969
Paqr6	1,291213816	1,510971031	1,517974773	0,995386127	0,319548503	NA
Smg6	722,5749896	0,073092067	0,097043314	0,753190134	0,451335667	0,774518953
Rasl10b	9,477348617	0,706841182	0,493710303	1,431692184	0,152231934	0,485725233
Podnl1	4,575269835	0,067523774	0,716408775	0,094253137	0,924908075	NA
Rsad1	141,8691701	-0,059438906	0,155627102	-0,381931585	0,702512112	0,900494767
Dync1li2	567,7930916	0,002955169	0,088103521	0,033542013	0,973242363	0,992710425
Kctd14	1,000054082	0,948350606	1,426841113	0,664650463	0,506274085	NA
Arhgef39	10,02308868	-1,033749055	0,55328979	-1,868368213	0,061710765	0,301208913
Rpp38	79,83999247	0,354140487	0,301773501	1,173530763	0,240583028	0,59816966
Sipa1l3	200,9172805	-0,511863425	0,165577895	-3,091375352	0,001992316	0,038104827
Dnah1	8,499431169	2,534850717	0,619286889	4,093176782	4,26E-05	0,002350713
Hipk4	0,273083462	-0,658048199	3,340448562	-0,196993963	0,843832262	NA
Lgr6	1,119037387	1,271773523	1,565767161	0,812236681	0,416655835	NA
Myrf	962,9313674	-0,327633553	0,110077838	-2,976380706	0,002916725	0,048081012
Tmed8	57,49036694	-0,438010932	0,211872841	-2,067329301	0,038703135	0,230786
A830080D01Rik	55,21126285	0,322406086	0,250890051	1,285049305	0,198775055	0,548812381
Zbtb2	142,1416511	-0,124548925	0,209478806	-0,594565759	0,552133774	0,830686071
6430531B16Rik	0,411908937	-0,918926577	3,055071653	-0,300787242	0,763576737	NA
Insc	739,8581435	-0,559693774	0,233774505	-2,394160881	0,01665844	0,14321671
Aasdh	211,9398483	0,237852608	0,156395596	1,52083955	0,128300107	0,445402266
Tapt1	1128,237541	0,184917444	0,07244473	2,552531332	0,010694329	0,10955746
Cenpe	10,01910146	-0,568713644	0,509353635	-1,116539875	0,264191112	0,625127605
Ythdf1	789,5918729	-0,054127775	0,125917788	-0,429865991	0,667293126	0,885726946
Coa6	448,8928283	0,364081791	0,139948295	2,601545029	0,009280488	0,10013789
Lin52	134,6467914	-0,509325826	0,204433292	-2,491403539	0,012723951	0,121459059
Sdk2	4,848333731	0,205754076	0,699224653	0,294260329	0,768558984	NA
Taf8	244,7620469	0,050107954	0,12022204	0,416795072	0,676828308	0,889551479
Ppp1r9b	203,731853	0,129508134	0,213643562	0,606187861	0,544390013	0,827477215
Slc36a3	0,093953095	-0,517475177	3,352475198	-0,154356154	0,877328924	NA
Lrrc66	0,548601688	1,563544501	2,867966945	0,545175217	0,585633015	NA
Yrdc	292,4225425	-0,255133668	0,182385415	-1,398870999	0,161851671	0,498455421
Mill2	6,903649383	0,230593552	0,764496822	0,301627875	0,762935756	0,925557106
Snx17	320,8388136	0,13892099	0,113666863	1,222176688	0,221640817	0,576257504
Rrnad1	178,6677563	0,106376463	0,158478889	0,671234281	0,502071293	0,80404781
Jrkl	69,66158781	0,086073943	0,230887014	0,372796815	0,709299673	0,90343073
Dok7	0,436842022	1,189885456	2,519829803	0,47220866	0,636777859	NA
Cfap61	0,667740346	-0,445851722	2,021474807	-0,220557644	0,825436884	NA
4930554G24Rik	0,113662148	0,780932884	3,352475198	0,232942181	0,815806304	NA
Zfp950	496,9229749	0,032663902	0,144950394	0,225345381	0,821710598	0,946779723
C730036E19Rik	810,6499173	-0,332797307	0,207021066	-1,60755286	0,107933143	0,407961039
Zfp141	218,3493948	-0,40010604	0,219516477	-1,822669736	0,068353427	0,319740014
9530062K07Rik	14,22289464	0,293188003	0,39718878	0,738157818	0,460418553	0,780953195
Kdm2a	1514,680276	-0,066521362	0,090598442	-0,734243997	0,462800043	0,781516914
Pid1	2728,410468	-0,084876142	0,133893757	-0,633906644	0,526141749	0,817627711
Tmem259	2601,892551	-0,070973604	0,10542206	-0,673232941	0,500799108	0,803541005
Itgad	0,12663974	0,780932884	3,352475198	0,232942181	0,815806304	NA
Zfp787	312,5840637	0,174650673	0,126654784	1,378950462	0,167910028	0,506724417
Adcy5	28,69801916	-0,217512033	0,317700826	-0,684644216	0,493568458	0,799142148
Ccdc27	0,468350644	1,251550749	2,486830678	0,503271397	0,614773473	NA
Acd	314,9662739	-0,076935355	0,102561311	-0,750140124	0,453170316	0,775628467
Gm2897	0,475611127	-1,522655558	2,375800649	-0,640902072	0,52158631	NA
Eea1	814,4268693	-0,149108932	0,122788789	-1,21435298	0,224612963	0,579757618
Syne2	552,9863119	-0,283079331	0,123218242	-2,29738168	0,021597006	0,163772191
Eif2a	1047,231328	0,106410529	0,09373807	1,135190098	0,256295677	0,616816726
Arhgap30	113,3973507	0,12495516	0,248135637	0,503576034	0,614559337	0,859778545

Lmod3	6,485640597	0,416305754	0,632256796	0,658444095	0,510252809	0,808610978
Gm826	22,37970834	-0,202676017	0,356987954	-0,567739091	0,570212143	0,841130547
Cep63	239,9194233	0,109725221	0,137320476	0,799044859	0,424264401	0,756961229
Gfod1	123,2190078	-0,618990132	0,288227371	-2,147575813	0,031747469	0,206737412
Grik3	0,266906519	0,586045239	3,35071904	0,174901337	0,86115716	NA
Tmem88b	44,2888003	-0,217655188	0,250625344	-0,868448435	0,385148889	0,729677128
Tmem104	292,3628575	-0,387305937	0,116493195	-3,32470868	0,00088511	0,021672218
Gucy2g	0,460658359	0,345791082	2,504501221	0,138067843	0,8901868	NA
Cacna2d4	1,326786204	3,836930081	1,47103032	2,608328346	0,009098563	NA
Dcp1b	62,76760202	0,443026691	0,231687266	1,912175398	0,055853701	0,285438885
A430078G23Rik	1,709846549	-0,300756878	1,117243665	-0,269195421	0,787779304	NA
Tmem237	50,92138084	0,243793514	0,266921979	0,913351216	0,361057854	0,710581841
Cdk15	0,31434569	0,667660234	3,262174889	0,20466721	0,837832141	NA
Nlrc4	8,434837281	-0,48328287	0,525757375	-0,919212726	0,357984316	0,707401735
Cdhr2	4,754626141	0,310139455	0,769627998	0,402973198	0,686967942	NA
Klhl21	540,6877317	0,072952133	0,105386438	0,692234552	0,488790043	0,796400974
Ankub1	7,759134772	0,216413404	0,509285176	0,424935605	0,670883618	0,887361539
Rdh12	1,379279016	-1,705543628	1,733954672	-0,983614887	0,325304903	NA
Cyp20a1	300,8756801	0,089090125	0,13755048	0,647690397	0,517185217	0,813424627
Trp53rka	118,5772857	0,338556004	0,170449441	1,986254703	0,04700505	0,258180159
Fndc3c1	0,534252681	-0,996320774	2,051562273	-0,485640035	0,627222406	NA
Safb2	901,3893533	0,144635124	0,114253482	1,265914363	0,205543744	0,558300827
Rilp	670,9863635	-0,099584453	0,118929133	-0,837342801	0,402399907	0,742560142
Trim38	0,086476712	0,780932884	3,352475198	0,232942181	0,815806304	NA
Usp32	717,0410616	-0,160773293	0,147018142	-1,093560907	0,274147603	0,635693562
Zfp114	1,269226024	-1,329605705	1,368895636	-0,971298082	0,331399864	NA
Chd4	2312,725966	-0,112219876	0,102423158	-1,095649451	0,273232212	0,634872238
Sumf1	634,0617234	-0,151183062	0,101942375	-1,483024715	0,138067844	0,461457253
N4bp3	111,3069513	-0,274243336	0,200544462	-1,367493936	0,171470537	0,511694109
Ankmy2	241,3560364	0,26281041	0,131463492	1,999113254	0,045596101	0,253145465
Srp68	1576,159893	-0,31675533	0,084083697	-3,767143244	0,000165126	0,006550662
Plekhh3	199,1881399	-0,026780321	0,171105196	-0,156513783	0,875628056	0,964125574
AW549877	755,9128304	0,110652459	0,124696566	0,887373746	0,374877713	0,720996253
Gga1	820,3129747	-0,057608227	0,083263966	-0,691874649	0,489016052	0,796400974
Stac2	1,104711061	0,610951908	1,58666291	0,385054635	0,700196937	NA
Tmem55b	495,2457662	-0,062191528	0,137846123	-0,451166321	0,651869681	0,877662367
Fntb	89,44087766	-0,348022154	0,164993423	-2,109309247	0,034917897	0,218240938
Mgat4b	719,4857034	0,120956413	0,081875264	1,477325475	0,139588412	0,464512269
Prr15l	15,41059267	1,114050146	0,49461173	2,25237308	0,024298705	0,177029642
Pttg1ip	5324,777524	0,131728312	0,089129552	1,477942049	0,13942329	0,464404798
Samd14	13,21228277	0,822738597	0,415667242	1,97932027	0,047779959	0,261149793
Nadsyn1	552,7865002	0,01856454	0,124738367	0,148827827	0,881689492	0,965856016
Olfr558	0,447336326	0,189178528	3,012656534	0,062794589	0,949930075	NA
Kcnc4	0,259974817	0,586045239	3,35071904	0,174901337	0,86115716	NA
Olah	0,086476712	0,780932884	3,352475198	0,232942181	0,815806304	NA
Evc2	47,1844796	0,97825955	0,414348086	2,360960709	0,018227662	0,14991666
Chd3	2778,377538	-0,319415712	0,129975725	-2,457502831	0,013990671	0,128727652
Gltpd2	1401,88534	-0,099930443	0,098840405	-1,011028264	0,312002902	0,671577331
Hpdl	3,723799774	-0,50365455	0,837829308	-0,601142196	0,547745281	NA
Tagap	6,937924224	-0,091897017	0,583310936	-0,157543793	0,874816297	0,963775779
Gmds	77,00527709	-0,452555927	0,193664006	-2,336809691	0,019449082	0,155418275
Efemp1	248,2774873	0,241522169	0,180022671	1,341620848	0,179718963	0,522431575
Sec14l4	4411,046491	-0,469696453	0,109370089	-4,294560403	1,75E-05	0,001278747
Cep290	62,16691411	-0,28767673	0,227456013	-1,264757639	0,205958221	0,558736902
Rpl39	1484,839345	0,17627577	0,189357315	0,930916081	0,351896976	0,702880198
Pmf1	502,4300868	0,255768944	0,119816776	2,134667222	0,032788202	0,210743017
Tcp11l2	541,8868627	0,526873279	0,240542546	2,1903538	0,028498588	0,194503289
Zc3h13	363,1665163	0,033453187	0,11367645	0,294284237	0,768540717	0,927598042
Col6a2	164,0800784	-0,257010299	0,293729767	-0,874988947	0,38157992	0,72637014
Zfp979	5,833752308	-0,320206616	0,848940234	-0,377183933	0,70603692	0,902122257
Neto1	0,681277209	-1,245718372	2,216824238	-0,561938268	0,57415808	NA
Slc16a4	0,916215272	1,603153643	1,566524714	1,023382286	0,306127131	NA
Pcif1	389,7795275	0,105598154	0,136554184	0,77330588	0,439341377	0,766839495
Cutal	1773,389826	0,254327944	0,108545443	2,343055002	0,019126563	0,154084887
D130043K22Rik	147,7208113	0,248577727	0,188046196	1,321897129	0,186202409	0,532805747
Gpr108	610,3330638	-0,312169023	0,164565157	-1,896932674	0,057836824	0,290515036

Pigu	438,1563438	-0,292835392	0,095847785	-3,055212914	0,002249008	0,041300808
Itpripl2	119,7682212	-0,006135883	0,182100057	-0,033695121	0,97312027	0,992710425
Cxxc4	3,831996562	-0,661088367	0,958329548	-0,689834064	0,490298544	NA
Cracr2b	54,9031018	0,226060425	0,250043444	0,904084594	0,365950547	0,714426527
2610008E11Rik	238,90848	0,213756193	0,137754498	1,55171843	0,120729611	0,432369185
Scaf1	968,9554922	-0,153185389	0,094481293	-1,621330365	0,104946806	0,402692427
Nol10	153,802076	-0,1207849	0,218471909	-0,552862381	0,580357649	0,845461633
Parp14	946,8072279	0,268417758	0,122405484	2,192857286	0,028317663	0,193745567
Aox2	0,481657017	-1,49944657	2,400877415	-0,624541078	0,532272302	NA
Plekhn1	14,96888955	0,442798453	0,407530566	1,08654047	0,277239946	0,638192109
Ccdc177	0,174042788	-1,166820872	3,345560292	-0,348766954	0,727264274	NA
Vcpkmt	53,6539139	0,814218072	0,304153354	2,676998502	0,007428497	0,087576343
Mtmr7	94,99573809	-0,29903089	0,282363975	-1,05902635	0,289587779	0,650994097
Slc5a10	0,366941628	1,960817963	3,100252574	0,632470393	0,527079549	NA
Pnrc1	2525,644922	-0,013594179	0,120215435	-0,113081808	0,909965697	0,9747592
Pdzd8	794,8341359	-0,186897357	0,119005194	-1,570497476	0,116299421	0,422731637
AU021092	28,25813047	0,664080046	0,318004237	2,088274209	0,036773107	0,224876005
Uprt	17,21943416	-0,046467137	0,507598493	-0,091543095	0,927061065	0,978888597
Stbd1	1560,12507	-0,361695715	0,176028197	-2,054760095	0,039902182	0,235069225
Gpr171	2,864784932	0,246343541	1,173763303	0,209874973	0,833765256	NA
Fitm2	411,8073257	-0,413777735	0,131292735	-3,151566108	0,001623974	0,033401397
Amz1	15,87407475	0,2934491	0,443066496	0,662313903	0,507770069	0,807274415
Ticam2	9,277271466	0,66801775	0,476010603	1,403367372	0,160507317	0,497178481
Xkr6	3,879582121	-0,179112275	0,790318737	-0,22663296	0,820709161	NA
Zfyve16	135,3465435	-0,13776483	0,156754694	-0,878856172	0,379479265	0,725074694
Haus6	68,14154338	0,130632656	0,211048013	0,618971266	0,535935289	0,823122647
Zfp879	15,18294211	-0,44647436	0,356496671	-1,25239419	0,210426262	0,562962817
Cilp	4,482768472	0,855406784	0,806908539	1,06010377	0,289097393	NA
Sox30	1,137751704	-1,485920241	1,461661609	-1,016596614	0,309345366	NA
Cc2d2a	101,6240277	0,882739143	0,262716124	3,36004935	0,000779286	0,020138259
Proser1	221,3323994	0,114954174	0,121580221	0,945500612	0,344403358	0,697519845
P3h2	17,04123344	-0,068534813	0,398868357	-0,17182314	0,863576573	0,961272625
Trp53bp2	529,3185441	-0,1653121	0,139526474	-1,184808126	0,236093319	0,593377403
Fam180a	18,0083327	0,476858416	0,480120938	0,993204792	0,320610164	0,67804688
Ube2o	255,0939684	-0,196473767	0,150774167	-1,303099671	0,192540736	0,541552905
H6pd	11562,13119	0,036941342	0,108762443	0,339651637	0,734118886	0,913675851
Cds1	9,939853592	-0,224260861	0,467780312	-0,479414921	0,631643481	0,867163863
Fnip1	562,8339282	-0,200657311	0,174310935	-1,151145857	0,249672235	0,60896459
Tdrp	249,0186258	-0,060092763	0,157759465	-0,380913836	0,703267186	0,900719679
Nrip1	175,1929628	-0,276997575	0,252878052	-1,09538006	0,273350166	0,634872238
Fkrp	284,4723231	-0,088804579	0,117550392	-0,755459658	0,449973229	0,773622955
Zfp445	1666,261198	-0,12280233	0,171335627	-0,71673552	0,473537304	0,787696929
Fam63b	416,7978815	0,06746129	0,167150201	0,40359682	0,686509225	0,893161227
Prune	430,4733642	-0,103384592	0,108248747	-0,955065023	0,339544816	0,694134533
Mcph1	80,27326552	-0,368491884	0,172021618	-2,142125438	0,032183389	0,208308055
Csnk1g1	118,3133617	-0,184986086	0,212413016	-0,870879243	0,383820092	0,72829995
Fndc3b	716,9971103	-0,790724327	0,166698279	-4,743446262	2,10E-06	0,000238051
Zc2hc1a	24,10818036	0,338745576	0,413538652	0,819138849	0,412707199	0,74954611
Pmpca	1784,257466	-0,090192012	0,088592374	-1,01805616	0,308651268	0,667523388
Gpkow	519,8534733	-0,207143772	0,126103757	-1,642645532	0,100456294	0,393294268
Ppp2r2c	0,12663974	0,780932884	3,352475198	0,232942181	0,815806304	NA
Zfp512	232,6223111	0,016105431	0,128038363	0,125785983	0,899901338	0,972184322
Gm16938	9,181494208	0,713000201	0,484099628	1,472837739	0,140794793	0,466258507
Gm2848	0,413799994	1,124462558	2,215523392	0,507538111	0,611777302	NA
Gm12409	0,113662148	0,780932884	3,352475198	0,232942181	0,815806304	NA
Gm1976	18,49722379	-0,109871543	0,33505795	-0,327918029	0,742973641	0,917092327
Gm10373	0,353927913	-0,100118367	2,670731403	-0,037487247	0,970096509	NA
Gm10640	0,77513806	1,247450586	1,986551766	0,627947687	0,53003821	NA
1810013L24Rik	587,2054492	-0,018080472	0,101438819	-0,178240169	0,858534364	0,958795917
Elp6	94,67310851	-0,279170556	0,175999301	-1,586202644	0,112693348	0,41594735
Rbm24	0,093303375	-0,517475177	3,352475198	-0,154356154	0,877328924	NA
Washc4	537,2050596	-0,302166389	0,138438872	-2,18267012	0,029060112	0,196651683
Ecm2	16,88188181	-0,181741627	0,360723181	-0,503825748	0,614383832	0,859778545
Mup20	118130,3689	-1,12040429	0,529448666	-2,116171713	0,034330206	0,216695243
Sctr	1,540993144	1,895228289	1,666434154	1,137295635	0,255414719	NA
Gm10767	19,80861854	0,142527733	0,345835746	0,412125509	0,680247433	0,891590973

Gm16973	55,09580423	0,235413155	0,286717871	0,821062022	0,411610941	0,748989611
Bcas3os2	6,051812667	0,285091709	0,626002374	0,455416339	0,648809743	0,876446263
4930447K03Rik	0,093303375	-0,517475177	3,352475198	-0,154356154	0,877328924	NA
BC051537	0,122496332	0,780932884	3,352475198	0,232942181	0,815806304	NA
A630019I02Rik	0,274362713	1,389394708	3,349408814	0,414817893	0,67827522	NA
6430584L05Rik	0,3267786	1,84016758	2,655545515	0,692952755	0,488339201	NA
Gm5086	3,096780807	0,304166067	0,886040756	0,343286768	0,731382734	NA
1700113A16Rik	89,44825402	0,210315847	0,248485553	0,846390642	0,397334857	0,738461552
1110019D14Rik	69,04712653	-0,179313129	0,201586409	-0,88951001	0,373729046	0,720823634
A930001C03Rik	35,9401459	0,172367536	0,269523793	0,639526233	0,522480654	0,815322684
9430018G01Rik	0,093303375	-0,517475177	3,352475198	-0,154356154	0,877328924	NA
4930471M09Rik	0,562382562	0,539849959	2,41422825	0,223611814	0,82305937	NA
Gm4265	1,744878918	-0,022285881	1,405941182	-0,015851218	0,987353087	NA
Atxn7l3b	2147,219272	0,115679519	0,07991733	1,447489783	0,147759796	0,477899684
Ppp3cc	59,88555484	0,6953157	0,225502322	3,08340816	0,002046443	0,038697159
Ddx11	138,1753008	-0,303010384	0,202985531	-1,492768387	0,135497825	0,458151054
Gm38416	85,79702402	-1,286657457	0,658161519	-1,954926593	0,050591735	0,270337998
Usp7	1680,303928	-0,09703661	0,073129453	-1,326915572	0,184536613	0,530941722
4632404H12Rik	49,75017583	0,048517905	0,287313581	0,168867425	0,865900922	0,961775826
B230307C23Rik	53,32502988	0,098863952	0,305673995	0,323429384	0,746370079	0,919312842
Nudt10	0,300137846	0,617320176	2,751219827	0,224380535	0,822461216	NA
Man1b1	841,3687971	-0,247967457	0,101067552	-2,453482377	0,014148047	0,129462863
2010001A14Rik	73,13987195	0,333426828	0,210248429	1,585870724	0,112768638	0,41594735
4930455G09Rik	4,656927436	0,215901673	0,801916169	0,269232223	0,787750985	NA
Piezo1	129,6866578	0,170000591	0,190942324	0,890324302	0,373291776	0,720655271
Bex6	0,086476712	0,780932884	3,352475198	0,232942181	0,815806304	NA
Tnfaip8l3	28,41315441	0,883536035	0,415808778	2,124861429	0,033598177	0,214233402
AW554918	63,55925594	-0,120126948	0,214467017	-0,560118522	0,575398598	0,843846408
Med13	626,924635	-0,133225117	0,142211754	-0,936808052	0,348857302	0,700915372
Smg1	899,614341	-0,117302068	0,113183816	-1,036385519	0,300022324	0,660164141
Lin28b	0,187906191	-1,241948083	3,339949325	-0,371846385	0,710007226	NA
Abca17	0,200683542	0,059593471	3,352475198	0,017775962	0,985817581	NA
Nr3c2	145,9216528	-0,143129301	0,244617249	-0,585115327	0,558470174	0,83440808
4933404K13Rik	0,113662148	0,780932884	3,352475198	0,232942181	0,815806304	NA
Gm5144	0,087021394	-0,517475177	3,352475198	-0,154356154	0,877328924	NA
Hecw2	34,36638283	-0,358264462	0,389317463	-0,920237327	0,357448752	0,707149983
Slc39a10	113,6168215	-0,508862239	0,180926847	-2,812530304	0,004915339	0,067955708
Kansl3	1156,888791	0,0074743	0,079799059	0,093664012	0,925376059	0,978307688
Zmpste24	1605,154	-0,062234865	0,119415621	-0,521161842	0,602254033	0,854608409
Ifi205	15,91806147	0,36224722	0,539399621	0,671574851	0,501854393	0,803893049
Acad9	808,676595	0,083841352	0,151404397	0,553757707	0,579744676	0,84530168
Scarf2	81,64592736	-0,101884845	0,232559778	-0,438101748	0,661312527	0,882666274
Phf24	3,305394247	0,34434909	0,822355419	0,418735113	0,675409731	NA
Epb42	0,093953095	-0,517475177	3,352475198	-0,154356154	0,877328924	NA
Zbtb41	284,3906283	0,022548134	0,184146834	0,122446494	0,902545419	0,973338566
Dpcd	161,2137101	-0,178089079	0,138485028	-1,285980744	0,198449782	0,548609416
Hace1	67,88640246	0,286285747	0,190379366	1,503764582	0,132641994	0,453114769
Fam234b	807,1079221	0,28344655	0,158374881	1,789719107	0,073499079	0,333880118
Gpat4	3454,23382	-0,134636509	0,119751426	-1,12429984	0,260885846	0,621964694
Ldlrad4	106,037084	0,17850014	0,268666569	0,664392821	0,506438926	0,806213623
Epc2	313,2022869	0,19619193	0,155074387	1,265147222	0,205818558	0,558736902
Sh3tc2	12,12812986	-0,234612784	0,430075673	-0,545515123	0,585399282	0,847482024
Pggt1b	150,8685041	-0,06346641	0,211154006	-0,300569292	0,76374295	0,925784704
A330009N23Rik	8,037875489	0,150736764	0,581561929	0,259192971	0,795486354	0,937598879
Gm19705	24,98174311	-0,11696353	0,297636281	-0,392974706	0,694338163	0,896264063
Gm14827	1,647338563	0,511966773	1,189082443	0,430556162	0,666791122	NA
Gm19557	0,997935972	0,384098955	1,905184574	0,201607215	0,840223805	NA
4930449E18Rik	1,032893836	2,495270905	1,82616551	1,366399098	0,171813732	NA
4930552P12Rik	1,765875347	1,027529867	1,107503147	0,927789568	0,353516736	NA
4930524O05Rik	0,371632404	1,974446396	3,090981855	0,638776443	0,522968375	NA
Rptoros	1,751028212	-1,113088342	1,376490604	-0,808642165	0,418721004	NA
6030407O03Rik	0,889026026	1,479168886	1,59404778	0,92793259	0,353442538	NA
A430088P11Rik	0,956479529	2,450493397	1,661453339	1,474909551	0,140236862	NA
2900076A07Rik	33,64914968	0,04723545	0,258181042	0,182954756	0,854833519	0,957431562
2900009J06Rik	18,04474273	-0,129149961	0,597718874	-0,216071412	0,828932083	0,947989138
Ube2k	1150,815312	0,034737811	0,084679734	0,410225788	0,681640326	0,89166825

Supplementary Table S1: *Serpina1* KO vs. wildtype all DEGs

Pgrmc1	28015,40465	0,018589135	0,086125519	0,215837718	0,829114247	0,948035269
Pcdhac2	0,142634598	-0,517475177	3,352475198	-0,154356154	0,877328924	NA
Zfp457	1,298930128	0,583178919	1,385322603	0,420969757	0,673777166	NA
Nup85	443,642706	-0,073117792	0,102087157	-0,716229093	0,473849902	0,787824815
Penk	0,353927913	-0,100118367	2,670731403	-0,037487247	0,970096509	NA
Sirpb1a	1,96813416	-0,194367913	1,058037845	-0,183706012	0,854244092	NA
Atg9b	7,927118401	-0,88574746	0,555243169	-1,595242428	0,110658034	0,411311191
Bfsp2	0,554876081	2,564126462	2,741308839	0,935365773	0,349599829	NA
Smim9	3,064649701	0,604207165	0,998451777	0,605144063	0,545083279	NA
Sbspon	2,125526588	-0,569326661	1,236722677	-0,460351113	0,645264219	NA
BC026585	541,9066505	0,11080524	0,103061955	1,075132327	0,282315488	0,64396447
Cdr2l	46,50721593	0,01367432	0,28909745	0,047300038	0,962274098	0,988526411
Fat3	0,23615848	1,389394708	3,349408814	0,414817893	0,67827522	NA
Spryd3	535,0439798	-0,135526763	0,104034912	-1,302704644	0,192675616	0,541552905
Kmt2d	676,7593696	-0,194133884	0,113760576	-1,706512846	0,087912637	0,36774219
Gxylt1	331,8809494	-0,265594909	0,122684334	-2,164864092	0,030398095	0,20178358
Brd1	490,542815	-0,032549278	0,118560125	-0,274538156	0,783671085	0,933234199
5031439G07Rik	608,6210933	0,077880502	0,100485132	0,775045029	0,438313052	0,766215379
Trappc6b	629,4178789	-0,06125667	0,080177876	-0,764009641	0,444861497	0,770752398
Slc4a7	143,6791547	-0,140551316	0,152548805	-0,921353106	0,356866103	0,707120636
Fam120a	3425,614227	-0,105919292	0,092015776	-1,151099262	0,249691401	0,60896459
C7	8,318225829	0,774415629	0,768609592	1,007553949	0,313668646	0,672348265
Gm14137	0,562251236	0,697322864	2,097791227	0,332408132	0,739581099	NA
AI429214	15,95184672	0,535348375	0,41010605	1,305390094	0,19176005	0,541080647
Mdc1	322,8644561	-0,020120424	0,131419421	-0,153100842	0,878318752	0,965046146
Ficd	228,3697911	-0,503566061	0,203688	-2,472242163	0,013426853	0,125089615
A3galt2	0,686486652	-0,437836407	1,981835956	-0,220924646	0,825151107	NA
Mettl17	291,2326326	-0,065057264	0,129606515	-0,501959827	0,61569578	0,860018481
Cerkl	28,10331097	0,873266525	0,377978618	2,310359589	0,020868254	0,161328775
Nlrc5	439,1980241	0,651010369	0,209105463	3,113311141	0,001850009	0,036371715
Chil5	0,187906191	-1,241948083	3,339949325	-0,371846385	0,710007226	NA
Hist1h2ab	0,274927585	-1,694186121	3,311210374	-0,511651611	0,608894858	NA
Arpc1a	1144,023027	-0,112420392	0,063901505	-1,759276117	0,078530621	0,347468495
Coq8b	460,3400562	0,00865473	0,139968397	0,061833456	0,95069546	0,985972877
Hist1h3d	0,778316759	-2,428980601	1,623520578	-1,49611938	0,134622548	NA
Lrrc8e	6,534508218	1,131706729	0,601616242	1,881110665	0,059956867	0,296324774
Fbxl20	763,0036479	0,19825421	0,150975273	1,313156825	0,189130109	0,536686321
Fam71e1	19,38429376	0,891760111	0,323533941	2,756310849	0,005845741	0,075581929
1700030K09Rik	81,4102313	0,059906051	0,247729543	0,241820373	0,808919352	0,94241441
Lsm11	84,53726166	0,059928682	0,226670509	0,264386764	0,791481909	0,936283441
1810062G17Rik	22,78022745	0,475549906	0,314611335	1,511547277	0,130649073	0,449471811
Adgrf1	325,8728194	-0,77141428	0,479831437	-1,607677655	0,107905795	0,407961039
Adgra3	1053,483856	-0,464108898	0,147572328	-3,144958847	0,001661102	0,033991735
Coq2	737,2264361	-0,028340978	0,105158319	-0,269507713	0,787539007	0,934424153
Elac1	246,9880514	0,373569711	0,148607307	2,513804458	0,011943664	0,116550977
Fbxo31	714,2709157	0,075842212	0,116260514	0,652347127	0,514177265	0,811764185
Atp6v0e2	21,9294091	0,377512903	0,341420954	1,105710996	0,268851629	0,629360433
Serpina4-ps1	3653,809734	-1,506453116	0,922075339	-1,633763589	0,1023085	0,398531
Smim10l2a	1,17431658	1,311813429	1,568294773	0,836458459	0,402897037	NA
Mug-ps1	3659,011853	0,013564734	0,240212501	0,056469724	0,954967614	0,986932748
Kctd12	210,383618	0,038648751	0,252655428	0,1529702	0,878421775	0,965046146
Memo1	707,2997459	-0,044020176	0,111932852	-0,393273064	0,69411781	0,89615268
Fgg	119773,4591	0,200323327	0,132238608	1,51486264	0,129807193	0,447902843
Slc25a3	5466,049217	-0,005281519	0,073624748	-0,071735646	0,942812288	0,983437952
Sdsl	305,1169887	-0,111976318	0,327512899	-0,341898954	0,732426937	0,912649428
Traf3ip2	141,7728668	0,012311714	0,161839275	0,076073707	0,939360458	0,982961018
Bsdc1	1577,097223	-0,048309105	0,07229615	-0,668211315	0,503998714	0,804997854
Psph	152,2878164	-0,363603667	0,196058054	-1,854571435	0,06365745	0,306733002
Smpdl3b	13,42993085	0,455864324	0,383381855	1,18906077	0,234415754	0,591595413
Trnp1	2,146237246	0,782951424	0,990690099	0,790309124	0,42934726	NA
Dhdh	2977,774397	0,114994786	0,105794295	1,086965856	0,2770519	0,638054582
Dus4l	112,6874921	0,01940866	0,143510388	0,135242199	0,892420383	0,969104575
Pgm2	5599,321242	0,152192233	0,097897715	1,554604547	0,12004028	0,430959549
Chst14	46,71623129	-0,07884298	0,225150216	-0,350179454	0,726204025	0,910393262
Phf7	164,5600691	0,031571271	0,152796865	0,206622505	0,836304683	0,951141594
Jsrp1	0,359084026	-0,720209661	2,627628721	-0,274091106	0,784014609	NA

Fbxo22	1347,016924	-0,084076682	0,093420232	-0,89998366	0,368128947	0,716690824
Sox10	0,093303375	-0,517475177	3,352475198	-0,154356154	0,877328924	NA
Sorl1	32,68720796	0,626935096	0,397549256	1,576999799	0,114795571	0,419546745
Ms4a4b	14,19751457	0,473099337	0,390232542	1,212352343	0,225377535	0,580946341
Katnal2	0,179780087	0,059593471	3,352475198	0,017775962	0,985817581	NA
Lrsam1	384,040763	-0,031118896	0,114831824	-0,270995398	0,786394571	0,934050588
6030468B19Rik	0,093303375	-0,517475177	3,352475198	-0,154356154	0,877328924	NA
Mrps5	997,2777157	-0,026606394	0,109142295	-0,24377712	0,807403441	0,942032857
Spaca6	31,10778217	-0,4936468	0,39061907	-1,263754993	0,20631798	0,558880735
Dpep1	14,54673915	0,769392395	0,511163613	1,505178333	0,132278234	0,452967915
Mterf1a	34,05184296	-0,229864449	0,261582999	-0,878743839	0,379540183	0,725074694
Pah	26171,89258	0,175790872	0,13391014	1,312752504	0,189266359	0,536686321
Pafah1b3	69,84099152	-0,056616145	0,256894916	-0,220386399	0,825570237	0,947537943
Fut4	3,351834075	0,07236807	0,831562861	0,087026578	0,930650386	NA
Sox11	0,266906519	0,586045239	3,35071904	0,174901337	0,86115716	NA
Slc12a2	170,2216883	-0,342844841	0,177308065	-1,93361109	0,053160963	0,278511661
Arhgap40	0,086476712	0,780932884	3,352475198	0,232942181	0,815806304	NA
Pcdhga12	3,346984705	1,405890202	0,817970131	1,718754938	0,085659004	NA
Gm2a	3834,826423	0,009681793	0,083516339	0,11592693	0,907710453	0,9747592
Zscan29	335,58545	0,027347371	0,109832885	0,248990733	0,80336795	0,940228147
Aff3	8,35231626	0,457907006	0,54211842	0,844662326	0,3982994	0,739635153
Sco2	344,7004353	0,246871953	0,172843717	1,428295784	0,153206741	0,488020233
Tigd2	309,063877	0,318628198	0,121820633	2,615551978	0,008908336	0,097989926
Gnat3	0,285269197	-1,521344816	3,321239936	-0,458065315	0,646905517	NA
Kcnq4	7,55005405	0,461999304	0,608827897	0,758833993	0,447951868	0,77297141
Stab1	694,393071	-0,144265777	0,227682984	-0,633625643	0,526325162	0,817627711
Spire2	2,942945653	-0,202496993	1,077885157	-0,187865091	0,850982409	NA
Kti12	438,8307857	-0,029156138	0,165457677	-0,176215079	0,860124975	0,959267413
Tmco3	170,808911	-0,140679538	0,166649466	-0,844164349	0,398577574	0,739635153
Rnf40	747,0586615	0,06075188	0,08733474	0,695621005	0,486666208	0,795157193
Prune2	8,756985281	-0,122855581	0,50834581	-0,241677177	0,809030315	0,94241441
Iqcg	34,14021809	0,689254709	0,295306712	2,334029948	0,019594154	0,156018012
Emsy	206,5891485	-0,187572675	0,176060433	-1,065388012	0,28670038	0,648131311
Sfswap	398,9247254	0,088775953	0,135463411	0,655350053	0,512242401	0,810227859
Traf3ip3	15,05935886	0,328226504	0,48318921	0,679291873	0,496952945	0,80083943
Nudt15	16,31352151	-0,027764889	0,391387123	-0,070939709	0,94344574	0,983617413
Mfsd9	74,13609252	0,121704985	0,230579242	0,527822816	0,597622319	0,85359111
Vps18	405,0204347	-0,021236726	0,11214381	-0,189370473	0,849802465	0,954750514
Nup214	330,7247847	-0,08362096	0,120778254	-0,692351122	0,488716853	0,796400974
Eif2b5	996,2810157	-0,058984691	0,115589898	-0,510292784	0,609846357	0,858123982
Homer1	182,0991285	-0,395635044	0,162770698	-2,430628168	0,015072674	0,134248221
Inpp5j	4,131335037	0,712710881	0,713249623	0,999244665	0,317676184	NA
Dse	155,4447181	-0,056082903	0,272094303	-0,206115682	0,836700549	0,951141594
Myl6b	23,08596465	-0,312999617	0,317011208	-0,987345586	0,32347325	0,680937786
Dync2li1	30,73544727	0,896325737	0,313267284	2,86121719	0,004220178	0,060994124
Wdr11	804,1053142	-0,027298557	0,119582864	-0,228281514	0,819427395	0,946091978
2810025M15Rik	97,16730148	0,004765556	0,189716802	0,025119313	0,979959795	0,994664228
1810064F22Rik	106,7593322	-0,64931419	0,57392952	-1,131348305	0,25790852	0,618915636
5730522E02Rik	2,770797417	-0,938282517	0,837919765	-1,119776089	0,262809191	NA
Atad3aos	17,59069205	0,643028645	0,48200315	1,334075608	0,182179079	0,527061698
Zfp85os	15,89358415	0,067376075	0,404718383	0,166476438	0,867782024	0,961775826
Zfp783	6,944632338	0,736738228	0,647015376	1,1386719	0,254840027	0,615196158
9130230L23Rik	0,886949688	-1,843208608	1,539587276	-1,197209562	0,231224884	NA
D930048N14Rik	284,736192	-0,338404101	0,268121868	-1,262127942	0,206902752	0,559462075
Ppp4r1l-ps	93,84791614	0,199512523	0,245287267	0,813383119	0,415998435	0,751551608
4931440P22Rik	3,284245135	-0,452576941	0,8107285	-0,558234897	0,576683994	NA
1110020A21Rik	75,74654654	0,286317153	0,212413853	1,347921281	0,177683702	0,52020802
2010204K13Rik	5,644579822	-0,156104747	0,607347672	-0,257026994	0,797157935	0,938299386
Ppifos	1,63748731	0,551894232	1,729860797	0,319039678	0,749696425	NA
BB123696	0,590891892	2,621142212	3,291727258	0,796281711	0,425868314	NA
Snhg9	1,188707391	0,348310054	1,633402977	0,213241961	0,831138241	NA
4930583K01Rik	0,65628918	2,82109352	1,938929521	1,454974763	0,145676248	NA
Nlrp1c-ps	5,401886737	0,503782042	0,734874234	0,685535047	0,493006353	0,798745371
2410006H16Rik	214,3011698	0,056587459	0,206650708	0,273831429	0,784214169	0,933246486
Foxd2os	3,4852528	-0,228414331	0,740599822	-0,308418021	0,757764271	NA
F630028O10Rik	11,88839683	0,286674554	0,479850974	0,597424138	0,550224242	0,829906108

Mir22hg	682,1824183	0,126433615	0,167173012	0,75630398	0,449466962	0,773349269
Gm16386	22,20533563	-0,536633774	0,331396803	-1,619308842	0,105380829	0,403011718
6820431F20Rik	47,5046873	-0,205716857	0,265700917	-0,77424218	0,438787589	0,766497562
9930014A18Rik	6,765406621	1,329721595	0,668885987	1,987964497	0,046815617	0,2575448
Afm	6354,2679	-0,029273635	0,071550612	-0,409131854	0,682442901	0,891903322
Cabp4	2,327979364	-0,79339267	1,003811716	-0,790379967	0,429305898	NA
Usp53	136,6222035	-0,232850432	0,188263559	-1,236832204	0,216149384	0,569623716
Spats2	114,4505735	-0,034220973	0,196575885	-0,174085308	0,86179842	0,960515471
Gm10494	0,113662148	0,780932884	3,352475198	0,232942181	0,815806304	NA
Zfp133-ps	5,830773246	-0,046776977	0,650722167	-0,071884714	0,942693655	0,983437952
Gm6225	2,113375628	-0,062623977	1,118542013	-0,055987148	0,955352046	NA
Gm4013	74,62997647	0,242243735	0,23047852	1,051046907	0,293237047	0,654344293
Serpina3h	4,572384365	-0,939893961	0,703785935	-1,335482729	0,181718404	NA
5430416O09Rik	7,679496162	1,084697736	0,603018011	1,798781656	0,072053226	0,329488907
Gm12216	11,84104523	-0,686311369	0,481826573	-1,424395017	0,154332156	0,489157561
BC065397	26,35136183	-0,26667969	0,297296906	-0,897014683	0,369711082	0,717858445
Slc22a13b-ps	3,522951212	-0,213850869	0,802661183	-0,266427322	0,789910126	NA
Gm5627	0,113662148	0,780932884	3,352475198	0,232942181	0,815806304	NA
Prr33	0,093953095	-0,517475177	3,352475198	-0,154356154	0,877328924	NA
Gm13375	66,78523357	0,925369618	0,207000651	4,470370564	7,81E-06	0,000664909
9330151L19Rik	104,1693673	0,073889609	0,155736755	0,474451959	0,635177648	0,868715087
BC039771	15,71819015	-0,608389997	0,381108273	-1,59637048	0,110406103	0,411062987
1700069L16Rik	0,443168919	2,196464572	3,313880474	0,662807421	0,5074539	NA
2700046G09Rik	24,42719474	0,162467173	0,343353866	0,47317706	0,636086863	0,869424933
2010016I18Rik	2,622620466	0,659036619	1,071104801	0,615286775	0,53836535	NA
Gm839	5,013067875	0,168488575	0,77911295	0,21625693	0,828787479	NA
Gm20605	16,86699263	0,540270198	0,369390501	1,462599056	0,143577122	0,470584709
AI427809	1,966397381	0,472265977	1,044237373	0,452259218	0,651082252	NA
Ubac1	364,7567824	-0,017006772	0,108632109	-0,156553821	0,875596499	0,964125574
Pgs1	301,9411217	-0,076056043	0,169999839	-0,447388913	0,65459428	0,87890639
Hlcs	421,0588473	0,16787227	0,118353354	1,418398927	0,156074329	0,491301554
Car9	3,281778057	0,612523726	0,878481365	0,697252953	0,485644505	NA
Braf	377,3531123	-0,108260136	0,109007139	-0,993147208	0,320638221	0,67804688
Kif18a	13,16063849	-0,480237371	0,480532487	-0,999385856	0,317607809	0,675759967
Mtmr6	602,4968098	-0,061232572	0,107068363	-0,571901632	0,567388619	0,83909496
Spice1	71,32324847	-0,06934768	0,221275019	-0,313400403	0,753976478	0,921880511
Uhrf2	719,42462	-0,178575928	0,110243369	-1,619833733	0,105267998	0,403011718
BC022687	14,29043534	-0,073832064	0,395789154	-0,18654393	0,852018232	0,95595696
Eno4	0,865958421	0,679890191	1,690885899	0,402091112	0,687616974	NA
Tigd5	37,59936679	0,574936577	0,273187989	2,104545585	0,035330883	0,219488183
Mrm1	68,42425306	0,035248714	0,258121721	0,136558497	0,891379784	0,969086471
Abhd17a	1009,469221	0,051640852	0,108817491	0,47456389	0,635097849	0,868694817
Slmo1	0,26513093	0,059593471	3,352475198	0,017775962	0,985817581	NA
Etf1	1896,902391	-0,008826248	0,092662459	-0,095251604	0,924114983	0,977951927
Fam49b	185,8207556	-0,158854359	0,15018384	-1,057732708	0,290177319	0,651777651
Creb3l3	6564,593727	-0,444704357	0,108077443	-4,114682436	3,88E-05	0,002224606
Otub1	873,3921008	0,008962801	0,087481541	0,10245362	0,918396625	0,976907366
Pnpo	5166,222067	0,009970589	0,063169955	0,157837519	0,874584833	0,963600184
Efr3a	1173,547764	0,031549951	0,131351821	0,240194241	0,810179678	0,942417935
Mark1	26,08180693	-0,382311866	0,287836303	-1,328226711	0,184103223	0,530561756
Sgtb	48,52509777	-0,486799389	0,363926467	-1,337631179	0,181016695	0,52483457
Ice1	428,7373114	-0,077518067	0,126759411	-0,61153698	0,540844144	0,826407188
Slc17a2	4583,213781	-0,472843072	0,133924144	-3,530678328	0,000414495	0,012865677
Heatr1	406,2263393	0,012942535	0,132902965	0,09738334	0,922421974	0,977360689
Rasl10a	1,678389435	0,333574949	1,228520974	0,271525644	0,785986779	NA
Dhx8	471,7185261	-0,121100461	0,107492661	-1,126592823	0,259914675	0,621613417
Tmem106a	199,7386928	0,125531037	0,221367905	0,567069722	0,570666813	0,8413217
Aarsd1	541,6513677	-0,042859825	0,142544487	-0,300676836	0,763660934	0,925769227
Lims2	1456,538788	-0,295428862	0,162980862	-1,812659831	0,069884304	0,323505282
Rprd1a	193,5072133	-0,211262412	0,148275792	-1,424793691	0,154216847	0,489157561
9130019P16Rik	0,314545931	-0,664815037	3,254120508	-0,204299452	0,838119498	NA
4632428C04Rik	20,78607002	-0,713302931	0,356098882	-2,003103542	0,045166176	0,251804256
BC021767	35,43417916	-0,715515635	0,522493183	-1,369425781	0,17086622	0,510601034
Mfsd4b4	15,09449409	0,082846971	0,414912647	0,199673285	0,841736109	0,952074658
1810010D01Rik	1,332591464	-0,271139523	1,435048172	-0,18894106	0,850139012	NA
D5Ertd605e	2,034538115	1,140478328	1,289543324	0,884404817	0,376477724	NA

Supplementary Table S1: *Serpina1* KO vs. wildtype all DEGs

1500015A07Rik	24,45465188	0,036408362	0,299368034	0,121617401	0,903202032	0,973489035
Cyp2c53-ps	4,581499051	-1,86449344	0,945531829	-1,971899181	0,048621117	NA
AY512931	1,527884809	0,340729167	1,550350502	0,219775571	0,826045946	NA
Cyp4f41-ps	4,16736585	0,691694185	0,74422383	0,929416874	0,352673086	NA
Adh6-ps1	1086,902672	-0,714879186	0,271199752	-2,635987609	0,008389279	0,094532042
Cyp4b1-ps2	0,087021394	-0,517475177	3,352475198	-0,154356154	0,877328924	NA
1810062O18Rik	4,703814454	0,526050276	0,893692018	0,588625908	0,55611225	NA
AI507597	3,145365886	1,291053088	1,342336202	0,961795626	0,336152276	NA
Gm16897	1,033694955	0,475556643	1,680012509	0,283067323	0,77712523	NA
Rbm3os	2,203592548	0,15145914	0,978764036	0,154745305	0,877022112	NA
F830002L21Rik	0,552872643	2,5663597	2,74466897	0,935034326	0,34977061	NA
D330041H03Rik	11,77207135	0,318120049	0,659982687	0,482012719	0,629796911	0,866228449
1700048O20Rik	19,88906945	0,341237289	0,436487343	0,78178049	0,434343589	0,764145208
Gm10125	1,197424838	0,346855729	1,263174008	0,274590616	0,783630777	NA
Gm12359	4,876849936	-0,017240423	0,681087786	-0,02531307	0,979805249	NA
Gm15348	11,47895503	0,170777329	0,533778337	0,319940539	0,749013406	0,919838327
Gm10790	1,024167131	-1,29480761	1,519722287	-0,852002778	0,39421255	NA
Gm16287	0,227324296	1,389394708	3,349408814	0,414817893	0,67827522	NA
Gm10389	0,18660675	-1,235117516	3,340448777	-0,369745983	0,711571767	NA
F630042J09Rik	0,234744367	0,059593471	3,352475198	0,017775962	0,985817581	NA
Gm10516	45,98609309	-0,091481059	0,313774471	-0,291550357	0,770630437	0,927934035
Snord95	2,04122	1,826181396	1,138044253	1,604666419	0,108567236	NA
B230206H07Rik	16,07291631	0,385580059	1,755315438	0,219664256	0,826132645	0,947537943
Gm11517	2,085394381	-0,172381514	1,01785293	-0,169357978	0,865515075	NA
Ftx	47,90689159	-0,053389932	0,23098758	-0,231137676	0,817207846	0,945090977
Cyp2d37-ps	775,4100617	-0,03242842	0,198191189	-0,163621908	0,870028797	0,961775826
2310015A10Rik	134,4758376	0,420921858	0,245250357	1,716294576	0,086108141	0,363775092
1500017E21Rik	852,5810781	0,721830975	0,291234216	2,478523932	0,013192726	0,123857833
Gm9961	0,227324296	1,389394708	3,349408814	0,414817893	0,67827522	NA
AI504432	3,889914801	1,052903188	0,776348072	1,356225675	0,175027375	NA
Gm11110	0,56762517	1,628972311	2,360890399	0,689982183	0,490205392	NA
Tnk2os	1,395679915	0,628211473	1,354670791	0,463737373	0,642835918	NA
Lztfl1	209,2918915	-0,274110136	0,129845748	-2,11104437	0,034768498	0,217612385
Vdac1	4411,858967	0,128474707	0,083075139	1,546488029	0,121986749	0,433849653
Rab40b	10,6688525	-0,13172551	0,492471918	-0,267478215	0,789100985	0,93523857
L2hgdh	1163,015917	0,051874289	0,088550622	0,585815076	0,557999791	0,834302421
Ubxn2a	640,9758026	-0,325975699	0,126967246	-2,567399928	0,010246436	0,106437143
Ldlrap1	671,6682108	-0,049175633	0,087349879	-0,562973113	0,573453192	0,842372044
Cerk	110,9489573	0,15737255	0,227295959	0,692368445	0,488705977	0,796400974
Cyp2d34	0,18725647	-1,238536202	3,340198538	-0,370797181	0,710788601	NA
Lrrc14	195,0490851	0,000439176	0,13414841	0,003273805	0,997387886	0,999481197
5430416N02Rik	72,80746552	-0,268569406	0,213864716	-1,255791098	0,20919172	0,561671273
6030408B16Rik	0,093953095	-0,517475177	3,352475198	-0,154356154	0,877328924	NA
Il27	3,350975628	0,254724492	1,02497658	0,248517378	0,803734126	NA
Adig	1,030654384	1,028538474	1,877972007	0,547685732	0,583907711	NA
Polr2h	187,8571449	-0,101485938	0,179485976	-0,565425447	0,57178442	0,841407954
Herc1	861,0180512	-0,143063862	0,12141092	-1,178344269	0,238659386	0,595935251
1810026B05Rik	148,3521049	0,201006659	0,147853503	1,359498792	0,173988585	0,516900754
Gm8579	0,113662148	0,780932884	3,352475198	0,232942181	0,815806304	NA
Arhgap33os	1,105230719	-3,65247127	1,844483273	-1,980213821	0,047679507	NA
Efcab8	0,087021394	-0,517475177	3,352475198	-0,154356154	0,877328924	NA
Gm3086	0,087021394	-0,517475177	3,352475198	-0,154356154	0,877328924	NA
4933421O10Rik	83,18939124	-0,071391556	0,172756345	-0,41324998	0,679423473	0,891375208
A730020M07Rik	1,92632687	-0,452567927	0,984949518	-0,459483374	0,645887089	NA
E230016K23Rik	0,122496332	0,780932884	3,352475198	0,232942181	0,815806304	NA
Gm14403	685,89965	-0,465405953	0,284949647	-1,633291913	0,102407617	0,398685914
Olfr1372-ps1	12,95919983	0,514355948	0,446172447	1,152818716	0,248984801	0,60797768
Foxh1	1,052652008	-1,252966296	1,636339532	-0,765712905	0,443847147	NA
Dph1	253,0312676	0,052992852	0,134923321	0,392762731	0,694494734	0,896379619
Gatad2b	136,5827485	-0,045610114	0,214549948	-0,212585062	0,831650624	0,949486153
Pim3	686,5866078	-0,520672942	0,230008487	-2,263711871	0,023591842	0,173678048
Nxnl1	0,375812382	-2,131890464	3,281425708	-0,649684209	0,515896226	NA
Kank2	1250,929943	-0,107188428	0,132693162	-0,807791651	0,419210532	0,753719022
Msantd4	545,2704063	-0,12991628	0,133200702	-0,975342304	0,329390493	0,686456976
Cables2	805,0578948	-0,015624298	0,089694913	-0,174193809	0,861713151	0,96050051
Ppan	360,7109218	0,437922037	0,28917485	1,514384938	0,129928237	0,448145151

Supplementary Table S1: *Serpina1* KO vs. wildtype all DEGs

Map3k21	182,7309598	-0,051307629	0,181435294	-0,282787477	0,777339754	0,930455294
Rpp40	167,9613687	-0,154274663	0,198841987	-0,775865629	0,437828329	0,766215379
Ahsa1	2815,081701	-0,355756285	0,116341411	-3,057864613	0,002229203	0,040993412
Gm2011	5,265753681	0,713315249	0,665903868	1,071198538	0,284080169	NA
2610507I01Rik	626,4558703	0,265566305	0,114327699	2,32285183	0,020187114	0,158753836
2410004I01Rik	0,086476712	0,780932884	3,352475198	0,232942181	0,815806304	NA
1600020E01Rik	113,8750302	0,156752868	0,169013471	0,927457831	0,353688877	0,704479528
5830428M24Rik	0,697692815	-1,292180713	1,721641714	-0,750551466	0,452922639	NA
1300002E11Rik	307,822887	-0,101598992	0,118705028	-0,855894594	0,392056085	0,734686211
C030037D09Rik	19,3988447	0,223012305	0,344923953	0,646554996	0,517919993	0,813532782
4930579G18Rik	1,697983097	1,940662368	1,783686142	1,088006641	0,276592176	NA
D130020L05Rik	281,0018766	0,305946507	0,136674479	2,238505024	0,02518814	0,181772069
Gm10440	0,473872042	0,298297561	2,173760948	0,137226479	0,890851782	NA
1700028I16Rik	0,787945517	-0,601180465	2,228865612	-0,26972486	0,787371934	NA
C730027H18Rik	398,8443794	-0,219175444	0,230231709	-0,951977664	0,34110831	0,6949523
Hk1os	0,552872643	2,5663597	2,74466897	0,935034326	0,34977061	NA
E130307A14Rik	15,66160705	-0,372595832	0,432976181	-0,860545795	0,389488251	0,73337178
Gm15663	3,552904315	0,569700253	0,824476599	0,69098414	0,489575508	NA
4930562F07Rik	0,25327948	1,389394708	3,349408814	0,414817893	0,67827522	NA
1700061I17Rik	12,32679599	0,884478131	0,532302651	1,661607602	0,096591478	0,386394815
B230208H11Rik	12,55625496	0,270562441	0,418331066	0,646766312	0,517783198	0,813424627
4933412E12Rik	16,04980229	0,30208578	0,380486107	0,79394694	0,427226321	0,759435698
Gm15910	3,232148673	2,34008141	1,196648108	1,955530113	0,050520533	NA
Gm20125	0,294091955	0,617264955	2,767052958	0,223076668	0,823475837	NA
Gm20110	0,396859045	2,039561942	2,555229094	0,798191421	0,424759415	NA
Gm15915	0,093303375	-0,517475177	3,352475198	-0,154356154	0,877328924	NA
Gm16998	7,96847843	0,555747052	0,585862152	0,948596952	0,342825642	0,695589013
Gm17745	1,076105404	1,144711758	1,403134734	0,815824546	0,414600492	NA
Gm17751	0,093303375	-0,517475177	3,352475198	-0,154356154	0,877328924	NA
D17H6S56E-5	90,91985285	-0,667912153	0,410635884	-1,626531382	0,103836663	0,400979881
2810408I11Rik	56,09057641	-1,046939984	0,390510839	-2,680949872	0,007341351	0,08700933
Gm16548	242,3756285	0,239874432	0,126194742	1,900827468	0,057324615	0,289352857
Platr22	12,51829959	0,38438272	0,430337001	0,89321327	0,371742973	0,719235192
Gm19461	7,201751774	-0,849655079	0,639958825	-1,327671477	0,18428666	0,530663359
9330159M07Rik	21,41522623	0,322105521	0,345233754	0,933007034	0,350816336	0,70210533
Gm16894	0,113662148	0,780932884	3,352475198	0,232942181	0,815806304	NA
Gm2447	0,113662148	0,780932884	3,352475198	0,232942181	0,815806304	NA
1700001G11Rik	0,086476712	0,780932884	3,352475198	0,232942181	0,815806304	NA
Gm20597	0,261385121	1,389394708	3,349408814	0,414817893	0,67827522	NA
Snhg4	28,24708532	-0,027055255	0,316466095	-0,085491795	0,931870418	0,980804253
Snhg18	34,9774426	0,141995785	0,346062797	0,410317973	0,68157271	0,89166825
C430049B03Rik	2,726678584	1,316564534	0,986688817	1,334325992	0,182097043	NA
1700025B11Rik	0,353964036	1,923250882	3,12828742	0,614793535	0,538691079	NA
4931408D14Rik	268,2112986	0,744008765	0,270565196	2,749831744	0,005962587	0,076721684
2310069G16Rik	1,517572932	0,42996668	1,458206704	0,294859898	0,768100907	NA
Apol7d	3,128908892	0,338475612	0,836714538	0,404529378	0,685823479	NA
Gm2061	53,80596164	0,02650509	0,213421849	0,124191082	0,901163983	0,972526743
2310040G24Rik	93,67482356	-0,381181421	0,203694767	-1,871336351	0,061298475	0,300072982
Gm20743	0,12663974	0,780932884	3,352475198	0,232942181	0,815806304	NA
Mkln1os	15,96947914	0,522870591	0,446979187	1,169787333	0,242086563	0,600344139
4933424G06Rik	0,764545322	0,371681899	1,624654486	0,228775966	0,819043048	NA
Dlx4os	0,306419827	0,617376943	2,735241395	0,22571205	0,821425385	NA
E230016M11Rik	17,52600847	0,382401181	0,334807881	1,142151074	0,253391229	0,612785071
Plxna4os1	7,41074494	0,609601036	0,637904234	0,955630962	0,339258713	0,694071514
4732490B19Rik	1,202223791	0,297238572	1,602373324	0,185498951	0,852837703	NA
9330179D12Rik	0,216449427	0,059593471	3,352475198	0,017775962	0,985817581	NA
9530051G07Rik	0,965667712	-1,07380287	1,553319806	-0,691295421	0,489379909	NA
A730046J19Rik	0,62556133	1,816069204	1,987661553	0,913671244	0,360889619	NA
Hccs	232,5348135	-0,211043959	0,16046758	-1,315181289	0,188448982	0,535621781
2310001H17Rik	241,0881434	-0,10564192	0,20518709	-0,514856567	0,606653254	0,856921485
Gm15417	141,2737556	0,000596097	0,170171551	0,003502916	0,997205083	0,999481197
2210408F21Rik	214,2103877	0,257648114	0,133025841	1,936827556	0,052766426	0,277184422
Gm14718	0,32750306	0,617574361	2,68189888	0,230275036	0,817878058	NA
AV039307	4,525839004	-0,061715337	0,767076606	-0,080455246	0,935875189	NA
2610206C17Rik	1,368361179	-0,594023597	1,733810275	-0,342611649	0,731890637	NA
4931403G20Rik	0,147722973	0,780932884	3,352475198	0,232942181	0,815806304	NA

Supplementary Table S1: *Serpina1* KO vs. wildtype all DEGs

0610005C13Rik	4037,091856	0,212985601	0,085319861	2,496319123	0,012548965	0,120782658
Stamos	21,84834114	0,299391475	0,33428047	0,895629575	0,370450635	0,71859468
5730405O15Rik	12,93493094	-0,069868788	0,415502027	-0,168155107	0,866461257	0,961775826
2610020C07Rik	3,247586993	-0,380421673	0,96981489	-0,392262149	0,694864528	NA
1810044D09Rik	52,25305687	0,322783101	0,232568374	1,38790626	0,165165591	0,503431976
Smkr-ps	1,036052345	-0,058816678	1,702693286	-0,03454332	0,972443899	NA
Rps15a-ps4	1,313886978	0,110397175	1,564679527	0,070555774	0,94375131	NA
Trp53cor1	3,350976642	0,502995216	0,850767539	0,591225209	0,554369534	NA
0610009L18Rik	7,077734205	-0,371529172	0,536445525	-0,692575768	0,488575822	0,796400974
4930558J18Rik	1,710564282	-0,745667825	1,562780852	-0,477141644	0,633261262	NA
D030040B21Rik	0,142634598	-0,517475177	3,352475198	-0,154356154	0,877328924	NA
Fchsd1	42,75989306	0,545522678	0,284320943	1,918686228	0,055024056	0,284066998
Pakap	0,093303375	-0,517475177	3,352475198	-0,154356154	0,877328924	NA
Diaph1	2020,514809	-0,343178476	0,138144565	-2,484198172	0,012984353	0,122739179
Adipor1	2407,370404	0,021438765	0,062522137	0,342898789	0,731674603	0,912222117
Dtl	12,82394857	-1,687077301	0,486611434	-3,466990671	0,00052632	0,015164599
2500004C02Rik	36,25681458	0,284638736	0,266591455	1,067696399	0,285657483	0,647170801
B230217O12Rik	22,44101391	0,622182587	0,343872452	1,809341176	0,070398016	0,324561524
A730056A06Rik	0,113662148	0,780932884	3,352475198	0,232942181	0,815806304	NA
Platr14	0,295445946	1,389394708	3,349408814	0,414817893	0,67827522	NA
Ctcflos	84,82618565	-0,01639081	0,377437845	-0,043426515	0,965361542	0,989924013
1810024B03Rik	40,00069959	-0,464202991	0,276663849	-1,677859222	0,093374585	0,380085036
Stkld1	3,29913761	0,792370948	0,804829111	0,984520735	0,324859542	NA
Ercc6l	6,03449929	-1,425473068	0,786709597	-1,811943155	0,06999498	0,32382454
Prss50	0,25327948	1,389394708	3,349408814	0,414817893	0,67827522	NA
E130215H24Rik	0,303470821	-0,629977435	2,731860529	-0,230603806	0,817622611	NA
4833419F23Rik	5,357626001	1,191334861	0,700670522	1,700278266	0,089078597	NA
E130018N17Rik	2,218180572	2,458824376	1,3804622	1,781160234	0,074886278	NA
Gm16982	1,682447276	0,6209076	1,575865028	0,394010647	0,693573178	NA
D330050G23Rik	0,991345624	-1,784172245	1,824035238	-0,978145711	0,328002262	NA
D330023K18Rik	25,73922066	0,700066641	0,414198961	1,690169959	0,090995444	0,374323747
1700063D05Rik	1,853213597	1,477225659	1,139917045	1,295906281	0,195007775	NA
Gm19522	95,66273113	-0,569801194	0,270717024	-2,104785235	0,035310007	0,219460476
C920006O11Rik	67,85870259	0,069630233	0,21154965	0,329143693	0,74204708	0,916899135
Gm10785	8,579479006	0,528245906	0,486659725	1,085452276	0,27772139	0,638271605
A730090N16Rik	0,656643975	-1,27972748	1,957336596	-0,653810634	0,513233815	NA
AI115009	0,086476712	0,780932884	3,352475198	0,232942181	0,815806304	NA
4930520O04Rik	0,50160216	-0,540014904	2,115618792	-0,255251516	0,798528844	NA
G730013B05Rik	1,370843599	0,463738037	1,322269391	0,350713735	0,72580312	NA
5730403I07Rik	0,086476712	0,780932884	3,352475198	0,232942181	0,815806304	NA
Gm16793	34,62508972	-0,958527485	0,302369267	-3,170055923	0,001524096	0,031688398
B130034C11Rik	15,13205366	0,491147389	0,440917613	1,113921002	0,265313079	0,626006345
A230028O05Rik	0,507753089	0,43378515	1,941821507	0,223390847	0,823231328	NA
Gm14169	0,23615848	1,389394708	3,349408814	0,414817893	0,67827522	NA
Gm14023	21,19005906	0,392172007	0,335322502	1,169536803	0,242187422	0,600476091
Gm13293	0,407649065	-0,024181934	2,572872293	-0,009398808	0,992500946	NA
Gm16796	0,43988623	-0,051302854	2,993416216	-0,017138563	0,986326074	NA
Gm15446	28,6337768	-0,199048613	0,334463679	-0,595127738	0,551758089	0,830638744
9430091E24Rik	8,855901399	0,735392667	0,577396098	1,273636363	0,202792319	0,555104527
Znf41-ps	8,17734932	0,456911819	0,509621291	0,896571291	0,369947723	0,717858445
Pcsk2os1	0,122496332	0,780932884	3,352475198	0,232942181	0,815806304	NA
E030013I19Rik	5,044832282	-0,687010575	0,726590746	-0,945526183	0,34439031	NA
4930473A02Rik	3,844332528	0,526881441	1,35709687	0,388241586	0,697837261	NA
1810019D21Rik	1208,246441	0,2535735	0,265272188	0,955899303	0,339123111	0,694071514
Gm15880	2,257342635	0,95296556	1,18480475	0,804322872	0,421210532	NA
Gm16157	337,8268415	0,53449244	0,137488997	3,887528842	0,00010127	0,004577077
Gm19897	1,149277861	0,192757893	2,107266146	0,09147297	0,927116783	NA
Redrum	0,122496332	0,780932884	3,352475198	0,232942181	0,815806304	NA
Pnldc1	14,83731753	0,06436977	0,379905123	0,169436435	0,865453367	0,961775826
Ap5b1	58,33007709	-0,035607586	0,276730143	-0,128672597	0,89761672	0,971242863
Dhps	228,6221678	-0,16554988	0,161005837	-1,028222847	0,303845014	0,663332368
Flnc	3,71661078	0,456168583	0,80757898	0,564859406	0,572169397	NA
Armc5	234,0698366	-0,042243355	0,134547155	-0,313966912	0,75354617	0,921613644
Btbd1	841,6421888	-0,034927119	0,101152831	-0,345290572	0,729875934	0,911594858
Neil3	5,542954033	-1,346607407	0,703191137	-1,91499485	0,05549316	0,284903249
Slc5a11	3,201660156	-0,08636541	0,890931794	-0,096938296	0,922775396	NA

Supplementary Table S1: *Serpina1* KO vs. wildtype all DEGs

Rbm5	830,6839318	0,146019625	0,110739963	1,318581121	0,187309189	0,533703441
Tagap1	114,6234039	-1,422514276	0,153818038	-9,2480329	2,29E-20	1,02E-16
Trhde	23,35483224	-1,023751628	0,641089547	-1,596893339	0,110289485	0,410942063
Zfp263	243,1261894	0,210224712	0,151654669	1,386206661	0,165683808	0,503987902
Zfand3	1608,791066	0,022956462	0,091675322	0,250410489	0,802269919	0,939799232
Wdr19	58,74361302	0,290990472	0,231735554	1,255700591	0,209224545	0,561671273
Gm15708	3,519946595	0,808348595	0,745982887	1,083602062	0,278541276	NA
Gm15787	8,607436203	-0,138261048	0,473251297	-0,29215144	0,770170838	0,927934035
Gm15408	52,44077941	0,558207448	0,356899542	1,56404641	0,11780667	0,426009566
Gm9899	0,355338216	0,663614712	2,635698453	0,251779452	0,801211541	NA
Gm15401	26,69491484	-0,417866245	0,375242599	-1,113589573	0,265455302	0,626006345
Tor2a	785,6743657	0,125391362	0,10792573	1,161830095	0,245304487	0,604623098
Gm16023	6,985867222	0,25331253	0,582469772	0,434893864	0,663639468	0,883893982
1810006J02Rik	0,086476712	0,780932884	3,352475198	0,232942181	0,815806304	NA
3110045C21Rik	58,00019291	0,668626166	0,207814344	3,217420671	0,001293488	0,028455407
Kank4os	0,093953095	-0,517475177	3,352475198	-0,154356154	0,877328924	NA
E130218I03Rik	0,661128786	0,052806816	2,337526179	0,022590898	0,981976604	NA
1810021B22Rik	71,24243498	0,099122043	0,200881107	0,493436364	0,621704293	0,862965544
Tbx3os2	0,241026348	0,059593471	3,352475198	0,017775962	0,985817581	NA
5033403H07Rik	536,3687961	0,17561361	0,135606403	1,295024464	0,19531179	0,544324229
Gm15471	0,519920248	-0,529423058	2,403495657	-0,220272109	0,82565924	NA
Gm11549	0,227324296	1,389394708	3,349408814	0,414817893	0,67827522	NA
Gm15441	4,288929321	-0,411902669	0,848380797	-0,485516257	0,627310184	NA
Gm16845	11,86275694	0,877167699	0,554449367	1,582051946	0,113637712	0,417177269
Gm10548	1,298479529	1,314948283	1,68076842	0,78234947	0,434009221	NA
Gm10804	11,36949078	1,380249796	0,93006121	1,48404189	0,137797808	0,461131137
Gm13807	0,25327948	1,389394708	3,349408814	0,414817893	0,67827522	NA
1700025N23Rik	0,122496332	0,780932884	3,352475198	0,232942181	0,815806304	NA
5330411J11Rik	1,850013466	0,760479805	1,366424119	0,55654741	0,577836693	NA
Ccdc137	224,8557808	-0,015110549	0,160827082	-0,09395525	0,925144704	0,978216671
Cdc5l	506,1454586	0,086165765	0,093513089	0,921430002	0,356825971	0,707120636
Otud6b	835,3895353	-0,318801057	0,127997932	-2,490673496	0,012750123	0,121580879
Ugt2b1	14331,23572	-0,60453413	0,230272413	-2,625299848	0,008657269	0,096351967
2210414B05Rik	0,086476712	0,780932884	3,352475198	0,232942181	0,815806304	NA
2310005A03Rik	0,830459211	0,574066827	1,970698187	0,291301241	0,770820939	NA
Gm20753	1,413836006	0,561243598	1,798827097	0,312005306	0,755036491	NA
Gm17066	124,7499959	0,119736379	0,201617167	0,593879883	0,552592455	0,83087907
Chn1os3	9,038283678	0,064172945	0,484969798	0,132323591	0,894728349	0,970433882
Arhgap15os	0,543657241	-0,753153359	2,096115672	-0,359309064	0,719363895	NA
D930015M05Rik	0,460989652	-1,562110858	2,403620225	-0,649899199	0,515757336	NA
4930573O16Rik	0,086476712	0,780932884	3,352475198	0,232942181	0,815806304	NA
A130077B15Rik	5,691788562	0,11933763	0,755887734	0,157877453	0,874553365	0,963600184
8030442B05Rik	0,142634598	-0,517475177	3,352475198	-0,154356154	0,877328924	NA
4930455H04Rik	3,292885879	1,075740401	1,131383754	0,950818321	0,341696611	NA
Lockd	3,479558469	-0,036434279	0,802374421	-0,045408076	0,963782044	NA
1700094J05Rik	0,829934992	0,531043496	1,93661252	0,27421257	0,783921269	NA
1700123M08Rik	0,811108442	-0,968652437	1,710053251	-0,566445774	0,571090786	NA
Gm10560	23,08530765	0,183124648	0,375744136	0,487365284	0,625999491	0,865266164
2810442N19Rik	1,283336744	-1,045827059	1,294488785	-0,807907392	0,419143895	NA
Gm12522	0,559767568	-0,685359321	1,880251039	-0,364504158	0,71548157	NA
4933439K11Rik	0,172953423	1,307385949	3,350719078	0,39018071	0,696402925	NA
1700125G22Rik	0,086476712	0,780932884	3,352475198	0,232942181	0,815806304	NA
B230354K17Rik	149,8629237	-0,242900249	0,144872395	-1,676649636	0,093611014	0,380194927
BC031181	1333,128435	0,001054424	0,094628458	0,011142778	0,991109534	0,99719344
Zfp941	17,18950379	-0,092642718	0,50320981	-0,184103561	0,853932213	0,957013979
BC048546	921,0934551	0,048259578	0,261353786	0,1846523	0,853501762	0,956611759
Ccdc148	22,29026709	0,306627077	0,337329446	0,908984022	0,36335856	0,712361223
Mansc4	7,169241315	0,923486993	0,780945286	1,182524577	0,23699762	0,594227528
Zfp458	16,58784164	-0,225910073	0,408071376	-0,553604312	0,579849674	0,84530168
Zfp85	60,05779953	0,04493198	0,220853769	0,203446744	0,838785867	0,951141594
Colec11	796,9203337	-0,079163431	0,159324615	-0,496868806	0,61928157	0,861846105
Trim68	86,5912103	-0,046642336	0,227591128	-0,204939166	0,837619655	0,951141594
Arhgdib	247,0560167	0,023421565	0,195196382	0,119989749	0,904491269	0,973666426
Angpt2	24,77666597	0,05377669	0,495145087	0,108607944	0,91351346	0,976232707
Rcor2	2,862898298	-0,714605012	0,952305951	-0,750394357	0,453017228	NA
Adrb1	11,73064639	0,482993952	0,457280404	1,056231467	0,290862478	0,65276825

Adrb2	24,35433273	0,043248159	0,420371363	0,102880841	0,918057544	0,976907366
Ssna1	311,9802383	0,133196682	0,123696519	1,076802181	0,281568651	0,642699555
Pkn3	25,25138539	-0,457629924	0,373418924	-1,225513476	0,220381816	0,574490867
Plekhg3	687,8761621	-0,002269145	0,162561511	-0,013958687	0,988862941	0,996575327
Fam210a	2254,673893	-0,054856533	0,105663137	-0,519164347	0,603646139	0,855462946
Glb1l2	0,473767003	-0,57532997	2,141592808	-0,268645827	0,788202244	NA
Rell2	8,203574544	-0,659454838	0,496712303	-1,327639427	0,184297253	0,530663359
Zfp128	46,33190038	-0,0231606	0,241933716	-0,09573118	0,923734077	0,977937985
Flywch1	296,9984136	-0,221546897	0,106055709	-2,088967191	0,036710677	0,224821363
Arhgap22	6,360465137	-0,041588259	0,608181841	-0,06838129	0,945482115	0,984440501
Rnase12	0,093953095	-0,517475177	3,352475198	-0,154356154	0,877328924	NA
Ncbp1	709,9242113	-0,228226607	0,116140639	-1,96508827	0,049404022	0,266538487
Zfp36l2	632,8193382	-0,386384163	0,143185612	-2,698484561	0,006965597	0,084198069
Ephx4	3,115846406	1,124855915	0,903210202	1,245397708	0,212985595	NA
Rab31	101,9574534	0,158284683	0,226369814	0,699230521	0,48440798	0,79420977
Nlrp1a	2,919791408	-0,120730855	1,058462843	-0,11406244	0,909188296	NA
Tnip3	0,259430135	1,627812584	3,345388605	0,486584005	0,626553161	NA
Lca5l	0,879069784	1,388241416	1,587198842	0,874648708	0,381765077	NA
Fam8a1	388,7715402	-0,037146999	0,19373437	-0,191741917	0,84794437	0,953880403
Carmil3	4,458336508	-0,368776031	0,707641553	-0,52113394	0,602273469	NA
Mir7651	0,722844716	-0,417791213	1,974034591	-0,211643309	0,832385319	NA
Gm29683	1,818487665	0,674043371	1,152375214	0,584916581	0,558603809	NA
Pla2g3	0,346451529	0,909256324	3,204051153	0,283783336	0,776576426	NA
Ankrd52	541,3066635	-0,396688204	0,144912454	-2,737433494	0,006192064	0,078391763
Ppip5k2	964,2081609	-0,051407294	0,16072575	-0,319844792	0,749085991	0,919838327
Rgp1	955,8290363	-0,199709242	0,11521224	-1,733402994	0,083024066	0,356552758
Sh3rf3	0,614683776	-0,045739082	2,038076145	-0,022442283	0,982095152	NA
Zmynd11	1311,406963	-0,03628169	0,080779335	-0,449145685	0,653326566	0,877997875
L3mbtl3	126,9998406	-0,345251675	0,194957759	-1,770905028	0,076576496	0,342478335
Il20ra	0,413255311	2,173474447	2,471778342	0,879316082	0,379229918	NA
Gm996	2,325869502	1,475605657	1,161197355	1,270762158	0,203813275	NA
Mir7116	0,172953423	1,307385949	3,350719078	0,39018071	0,696402925	NA
Fmr1os	0,086476712	0,780932884	3,352475198	0,232942181	0,815806304	NA
Clcn6	214,4439389	-0,030499735	0,125790634	-0,242464275	0,808420435	0,942326905
Zcchc4	110,2667968	0,19867187	0,147120063	1,350406363	0,176885671	0,520079413
Hhipl2	17,59556172	1,889979186	0,591043551	3,197698688	0,001385289	0,029537375
Pm20d2	221,3835234	-0,051970486	0,165796511	-0,313459466	0,753931611	0,921880511
Zfp366	7,461749331	-0,361912343	0,573828739	-0,630697486	0,528238343	0,81852028
Gmps	1253,98042	-0,182545198	0,104873279	-1,740626392	0,081749088	0,353542587
Slc13a5	26,86521781	0,382794181	0,401592623	0,953190271	0,340493675	0,694720147
Git1	306,2848267	-0,012502034	0,105723039	-0,118252695	0,905867438	0,974066601
Usp22	592,6792469	0,106257382	0,09464169	1,122733353	0,261550758	0,622343615
Zfp619	28,11640596	-0,03983963	0,266432592	-0,149529868	0,881135543	0,965734294
1700001J03Rik	0,087021394	-0,517475177	3,352475198	-0,154356154	0,877328924	NA
Gbp11	338,4583587	-1,375061244	0,543625264	-2,529428515	0,011424844	0,114223875
Wdr34	124,4466353	-0,241720831	0,159984173	-1,510904655	0,130812745	0,449687731
Zfp760	142,893067	0,047301416	0,228934308	0,206615671	0,836310021	0,951141594
Klhdc10	1315,684523	-0,343937913	0,103412417	-3,325886029	0,00088138	0,021620484
Iws1	501,378581	0,084585634	0,101574992	0,832740737	0,404990964	0,744879793
Maf	315,3353028	0,338770974	0,28062855	1,207186418	0,22736036	0,582853433
Kbtbd7	204,2779523	-0,084638698	0,192865629	-0,438848012	0,660771669	0,882666274
Iqch	2,70449988	2,729643723	1,14862235	2,376450121	0,017480125	NA
5033403F01Rik	7,158867349	0,027548693	0,596131956	0,046212407	0,963140953	0,988725638
Api5	1440,551184	-0,096761074	0,103296369	-0,936732577	0,348896135	0,700915372
2510003B16Rik	1,34779245	1,544708442	1,580606112	0,977288668	0,328426261	NA
1700112J16Rik	3,25149618	0,576008841	1,034337702	0,556886634	0,577604888	NA
Zc3h12b	0,757297912	0,195175665	1,619786143	0,120494712	0,904091268	NA
1700007F19Rik	3,062409296	1,675231734	1,01372324	1,652553348	0,098421799	NA
1700119H24Rik	15,57570976	1,004179194	0,538868747	1,863494959	0,062392661	0,303208828
Slc35e4	11,47491578	-0,114748958	0,440221494	-0,26066187	0,794353273	0,937091195
Mmrn2	166,2978282	-0,444894906	0,270009668	-1,64769991	0,099414267	0,392688228
Crygn	29,80980642	0,017461065	0,504392236	0,034618029	0,972384325	0,992650147
Catsper2	59,68152871	0,122519364	0,231318728	0,529656051	0,596350425	0,853075785
Dnajc27	18,6124346	0,211818269	0,421541054	0,502485503	0,615326047	0,859955845
Slc16a11	234,3482284	0,524484577	0,290804256	1,803565686	0,071299419	0,327375771
Tinf2	267,2716307	-0,076665092	0,118846491	-0,645076617	0,518877536	0,813725618

Supplementary Table S1: *Serpina1* KO vs. wildtype all DEGs

Ackr4	24,11432792	-0,208261957	0,350257657	-0,594596444	0,552113258	0,830686071
Nudt18	378,6429705	-0,095228767	0,127259147	-0,748305869	0,454275685	0,776572096
Il1f9	2,835462178	1,37264276	1,104245896	1,243058965	0,213846106	NA
Irgq	569,1981918	-0,378195631	0,124839158	-3,029463173	0,002449888	0,043266247
Rdh9	769,3853991	0,879484233	0,452243004	1,944716063	0,051809161	0,274159572
Kctd7	101,4554144	-0,009717514	0,18066229	-0,053788282	0,957103845	0,98781716
Cnksr3	213,4201275	0,085725551	0,187521222	0,457151198	0,647562373	0,876329726
Stradb	863,7438675	-0,052283196	0,087835497	-0,595239935	0,551683099	0,830638744
Tomm22	1127,467083	0,091500596	0,103326544	0,885547818	0,375861241	0,721655743
Efhb	0,458109115	2,291960889	2,428288334	0,943858625	0,345241897	NA
Boc	6,534049849	0,133010351	0,590193378	0,225367406	0,821693465	0,946779723
Tpk1	393,2972288	0,084555092	0,124660142	0,6782849	0,497591071	0,801288247
9130015A21Rik	0,339756192	1,876228608	3,160061454	0,593731684	0,552691588	NA
1700012D01Rik	47,4078401	0,208998444	0,234944238	0,889566159	0,373698884	0,720823634
4930593A02Rik	1,025665817	-0,59268113	1,449791727	-0,408804326	0,682683265	NA
4933431G14Rik	0,12663974	0,780932884	3,352475198	0,232942181	0,815806304	NA
Slc2a4rg-ps	117,3126638	-0,069581497	0,266178413	-0,261409242	0,793776931	0,936746676
Gm7008	2,007882511	1,766890718	1,210291555	1,459888496	0,144320721	NA
E130008D07Rik	0,087021394	-0,517475177	3,352475198	-0,154356154	0,877328924	NA
BC051226	67,2936807	-0,072391805	0,203895411	-0,355043819	0,722556764	0,909644412
1110028F11Rik	30,48919817	0,490769482	0,293644539	1,671304642	0,09466152	0,38267105
Nlrp5-ps	1,596579648	1,700978837	1,36276202	1,248184798	0,21196339	NA
Gm2115	0,087021394	-0,517475177	3,352475198	-0,154356154	0,877328924	NA
Gm9920	10,36581619	0,346735552	0,459745965	0,75418944	0,450735478	0,774175112
Gm12159	0,113662148	0,780932884	3,352475198	0,232942181	0,815806304	NA
AW495222	8,73041888	1,44871192	1,919112075	0,754886564	0,450317049	0,773849236
4933406C10Rik	1,893513408	0,924387842	1,062001754	0,870420259	0,384070779	NA
2210039B01Rik	49,36463472	0,263485349	0,261873409	1,006155417	0,314340815	0,673320811
AI197445	3,579360081	-0,481483267	0,81007618	-0,594367887	0,552266082	NA
3300005D01Rik	1,669701014	0,956901252	1,40559293	0,680781207	0,496009943	NA
Gm3716	0,306419827	0,617376943	2,735241395	0,22571205	0,821425385	NA
Gm16576	9,176338006	-0,024471245	0,697151918	-0,035101739	0,971998614	0,992423486
4930592A05Rik	1,682286744	1,404960726	1,869731511	0,751423783	0,452397654	NA
Gm20324	22,03263572	0,411807104	0,34371259	1,198114693	0,230872369	0,587687111
4930556M19Rik	143,3587677	0,030317389	0,163646474	0,185261485	0,853023946	0,956316741
Gm11351	0,113662148	0,780932884	3,352475198	0,232942181	0,815806304	NA
4930431P03Rik	0,908285599	1,484642295	1,870151883	0,793861883	0,427275842	NA
Gm20337	30,38700799	0,027515628	0,36799766	0,074771205	0,940396753	0,983128178
3110056K07Rik	57,18628594	0,18430854	0,204450922	0,901480598	0,367332845	0,715662023
2610307P16Rik	2,411672692	0,792856073	1,004536138	0,789275809	0,429950822	NA
5730420D15Rik	3,754578069	1,013357059	0,923437042	1,097375363	0,272477339	NA
A330032B11Rik	0,093953095	-0,517475177	3,352475198	-0,154356154	0,877328924	NA
C130060C02Rik	0,142634598	-0,517475177	3,352475198	-0,154356154	0,877328924	NA
4930483K19Rik	1,689619898	-0,657697977	1,145066942	-0,574375133	0,565713977	NA
4930467K11Rik	1,038018141	0,473957458	1,869584563	0,253509506	0,799874517	NA
4930455C13Rik	0,942338352	-0,939431018	2,138561668	-0,439281706	0,660457428	NA
2610016A17Rik	0,576459354	1,645357159	2,070773199	0,794561741	0,426868481	NA
Arhgap27os3	2,493548571	1,41241159	1,044758611	1,351902321	0,176406565	NA
4933440J02Rik	1,666840148	-1,267643017	1,114973286	-1,136926806	0,255568885	NA
E030044B06Rik	0,086476712	0,780932884	3,352475198	0,232942181	0,815806304	NA
2010308F09Rik	3,594735042	-0,968586411	0,751907291	-1,288172654	0,197685869	NA
Gm6277	3,919713761	0,140878589	0,710449653	0,198294964	0,842814291	NA
Bvht	11,53070852	0,425131623	0,41706878	1,019332164	0,308045299	0,667059844
Gm10549	0,796507233	1,321013766	1,740420848	0,759019732	0,447840753	NA
Gm15328	1,241827275	0,531928774	1,32285809	0,402105697	0,68760624	NA
A830052D11Rik	26,28669151	0,296227274	0,366148323	0,809036271	0,418494283	0,753109446
5830416I19Rik	0,285269197	-1,521344816	3,321239936	-0,458065315	0,646905517	NA
4930455D15Rik	0,122496332	0,780932884	3,352475198	0,232942181	0,815806304	NA
4930563E18Rik	1,210155255	-0,448366183	1,287365626	-0,348281929	0,727628464	NA
4930549G23Rik	0,735981896	1,039571333	2,018274496	0,515079259	0,606497635	NA
4933424G05Rik	0,266906519	0,586045239	3,35071904	0,174901337	0,86115716	NA
Pitpnm2os1	1,112066026	0,638959238	1,467292179	0,435468305	0,66322254	NA
A630012P03Rik	0,316677386	-1,85847818	3,205847439	-0,579715103	0,562106756	NA
1700024F13Rik	0,113662148	0,780932884	3,352475198	0,232942181	0,815806304	NA
1110028F18Rik	0,18725647	-1,238536202	3,340198538	-0,370797181	0,710788601	NA
1810012K16Rik	4,258276546	0,187561136	0,695067564	0,269845905	0,787278805	NA

1810020O05Rik	0,12663974	0,780932884	3,352475198	0,232942181	0,815806304	NA
1700007J10Rik	1,272229421	-1,601805167	1,753169846	-0,913662285	0,360894328	NA
5730480H06Rik	15,99954096	-0,303345675	0,366640133	-0,827366259	0,408029499	0,746892025
2410003L11Rik	2,781315575	0,846674335	1,359710737	0,6226871	0,533490162	NA
Fam219aos	0,220592835	0,059593471	3,352475198	0,017775962	0,985817581	NA
E130310I04Rik	4,861147227	0,214025714	0,680197062	0,314652512	0,753025504	NA
AU022793	0,547135071	0,685902857	2,404393878	0,285270589	0,775436844	NA
C330022C24Rik	15,15597948	0,714574042	3,050469876	0,234250483	0,814790525	0,944076707
F420014N23Rik	2,064930569	-0,720546954	0,987199906	-0,729889609	0,465457664	NA
1700007L15Rik	32,87401091	0,189581221	0,302563715	0,626582805	0,530932743	0,819884984
Gm9833	6,654517943	-0,821535976	0,583562039	-1,407795438	0,159191652	0,495312554
Gm11944	2,049707126	0,053947755	1,021603123	0,05280696	0,957885716	NA
E330011O21Rik	96,24109874	0,555414077	0,161588008	3,437223362	0,000587711	0,016471918
Snhg15	27,15382699	0,235940259	0,44340757	0,532106971	0,594651909	0,852263143
Gm11747	0,703295252	0,129157014	2,014419816	0,064116235	0,948877675	NA
Gm10658	180,7982557	-0,111595836	0,141939934	-0,78621874	0,431739349	0,7622164
Gm10787	0,261064182	-1,640042134	3,314234586	-0,494847933	0,620707466	NA
Gm10556	4,077578249	-0,925463087	0,795149358	-1,163885851	0,244470274	NA
Gm15612	0,909817822	0,682055901	1,569570602	0,43454936	0,663889559	NA
Gm10532	2,51367715	-0,50659833	1,302296577	-0,38900381	0,697273328	NA
9030404E10Rik	0,093953095	-0,517475177	3,352475198	-0,154356154	0,877328924	NA
Gata5os	0,219943115	0,059593471	3,352475198	0,017775962	0,985817581	NA
Gm9054	0,309752802	-0,661105099	2,719147868	-0,243129514	0,807905068	NA
4930428O21Rik	0,295445946	1,389394708	3,349408814	0,414817893	0,67827522	NA
Gm14634	1,914820857	-0,733711853	1,153162923	-0,636260357	0,524606739	NA
4933433G19Rik	3,193147205	-0,263133824	1,010482654	-0,260404098	0,794552082	NA
4933417D19Rik	3,596211331	0,251084506	0,809833624	0,310044556	0,756527074	NA
B130024G19Rik	15,28840898	-0,408051507	0,386491649	-1,055783503	0,291067137	0,653114648
4933416M06Rik	3,561782478	0,812303931	0,843911945	0,962545838	0,335775489	NA
Gm5547	1,747621568	0,512527272	1,104116196	0,464196861	0,64250671	NA
Gm17746	14,26985197	1,117650365	0,706354379	1,582279941	0,113585678	0,417177269
4933416M07Rik	0,727151796	2,019660894	2,724619109	0,741263572	0,458533644	NA
A730036I17Rik	1,721618729	0,573060299	1,30709473	0,438422928	0,661079729	NA
A330041J22Rik	5,776157813	0,062727044	0,671719884	0,093382742	0,925599501	0,978363356
E130006D01Rik	0,12663974	0,780932884	3,352475198	0,232942181	0,815806304	NA
1700016L04Rik	0,23615848	1,389394708	3,349408814	0,414817893	0,67827522	NA
Gm4890	27,16362251	0,481402329	0,29996301	1,604872312	0,108521908	0,40879949
6330418K02Rik	23,33001478	0,261435821	0,311582098	0,839059185	0,401436099	0,742090598
A930024E05Rik	9,849381181	0,151936327	0,480879102	0,315955354	0,752036395	0,920946649
E130102H24Rik	49,70594339	0,249160154	0,321607036	0,774734773	0,438496399	0,766308282
4930527F14Rik	0,379918946	1,000946436	2,589354372	0,386562167	0,699080371	NA
Gm29684	0,285269197	-1,521344816	3,321239936	-0,458065315	0,646905517	NA
4930511A08Rik	3,856735255	-0,263307019	0,807009668	-0,326274925	0,744216358	NA
8430430B14Rik	0,261064182	-1,640042134	3,314234586	-0,494847933	0,620707466	NA
1110058D11Rik	0,172953423	1,307385949	3,350719078	0,39018071	0,696402925	NA
Coq6	301,2740421	0,110549014	0,129046933	0,856657431	0,391634236	0,734532481
Acot6	12,51428999	0,434190096	0,494059862	0,878820826	0,379498432	0,725074694
Mis18bp1	4,67930544	-1,313421883	0,738377082	-1,778795572	0,075273283	NA
Gzmb	16,01749927	0,555098518	0,403276334	1,376471844	0,168675598	0,507888305
Cpne7	0,694464877	0,24770325	1,765069622	0,140336249	0,888394325	NA
Dppa2	0,427903795	-2,257267924	3,274223637	-0,689405543	0,490568096	NA
Ccdc186	124,278795	-0,39043167	0,328534564	-1,188403634	0,234674425	0,592066879
Zfp628	144,3749468	-0,284568153	0,198636397	-1,432608312	0,151969805	0,485352203
U2af1l4	344,312247	0,024583265	0,129345223	0,190059317	0,849262649	0,9545815
Cd300a	98,21961939	0,349268629	0,251650452	1,387911788	0,165163908	0,503431976
Pgbd5	0,12663974	0,780932884	3,352475198	0,232942181	0,815806304	NA
Wwc1	1588,646322	-0,515137075	0,123068537	-4,185773951	2,84E-05	0,001783764
Dio3	0,804613148	2,187864166	1,614482936	1,355148523	0,175370242	NA
Vps41	1263,087838	-0,088293534	0,07993322	-1,104591237	0,26933675	0,629944543
Myl9	80,47945397	-0,148886442	0,299410808	-0,497264754	0,619002363	0,861782322
Nup93	172,8017576	0,009278502	0,125527776	0,073915931	0,941077279	0,983128178
Pddc1	377,1198517	-0,047012991	0,119378978	-0,39381298	0,693719121	0,895984052
Ppat	409,4848706	-0,253173508	0,166423479	-1,521260762	0,128194413	0,445315232
Eva1b	108,7022936	0,236026933	0,225708426	1,045716093	0,295692131	0,656880707
Inafm2	233,9686215	0,088324725	0,135593347	0,651394233	0,514792033	0,812039132
Frs3os	0,093303375	-0,517475177	3,352475198	-0,154356154	0,877328924	NA

Gm13031	0,12663974	0,780932884	3,352475198	0,232942181	0,815806304	NA
2310010J17Rik	97,40214395	0,137450849	0,179534191	0,765597057	0,443916096	0,770142004
2700069I18Rik	2,737952561	2,183173196	1,05520593	2,068954632	0,038550344	NA
Gm16325	0,113662148	0,780932884	3,352475198	0,232942181	0,815806304	NA
Gm13710	3,608928381	1,320054282	1,08847832	1,21275202	0,225224645	NA
Tomm6os	17,17950694	-0,086531138	0,443144914	-0,195266008	0,845184707	0,953247119
AI463170	49,30771711	1,291287488	0,321688226	4,014096205	5,97E-05	0,003092177
4930404H11Rik	31,41654096	0,793159318	0,331794137	2,390516379	0,016824699	0,143473504
4933405D12Rik	0,727046484	0,354357752	1,762435806	0,201061367	0,84065059	NA
Gm4827	0,113662148	0,780932884	3,352475198	0,232942181	0,815806304	NA
Slfn5os	0,569808659	2,561392329	2,794860266	0,916465256	0,359422923	NA
1700092C10Rik	17,93733713	-0,838485204	0,545765126	-1,53634808	0,124453033	0,438769146
2700070H01Rik	0,113662148	0,780932884	3,352475198	0,232942181	0,815806304	NA
1700054M17Rik	0,82570019	2,279694449	1,85098312	1,231612771	0,218093768	NA
9530052E02Rik	0,984213457	1,685213427	1,56846264	1,074436448	0,282627114	NA
9530027J09Rik	2,389219835	1,037726202	1,491029137	0,695979828	0,486441463	NA
Ino80dos	9,790895829	-0,13361887	0,461164321	-0,289742427	0,772013296	0,928273961
Gm1720	0,087021394	-0,517475177	3,352475198	-0,154356154	0,877328924	NA
4930432J09Rik	0,372290591	-2,063763532	3,073186103	-0,671538743	0,501877387	NA
4930401O12Rik	0,285269197	-1,521344816	3,321239936	-0,458065315	0,646905517	NA
Scpep1os	1,122747969	0,088847534	1,83666839	0,048374293	0,961417947	NA
Gm5039	0,18660675	-1,235117516	3,340448777	-0,369745983	0,711571767	NA
Lars2	25582,3406	0,115465526	0,18905267	0,610758507	0,541359467	0,826407188
Arhgef19	2056,329049	0,07798364	0,08770363	0,889172316	0,373910479	0,720823634
Fbxo42	231,9327938	0,022494103	0,133718013	0,168220443	0,866409859	0,961775826
Mettl2	272,478003	-0,070718744	0,118895261	-0,594798675	0,551978054	0,830638744
4930506C21Rik	14,85085682	0,290832906	0,918017385	0,316805444	0,751391233	0,920662578
9130221H12Rik	66,18012048	-0,09938457	0,20004969	-0,496799422	0,619330503	0,861846105
D930028M14Rik	0,180974489	-1,204797453	3,342691436	-0,360427361	0,718527568	NA
4930599N23Rik	5,283124336	0,961825366	0,681693923	1,410934341	0,158263975	NA
Gm10364	0,941441062	3,291214192	2,055799712	1,60094107	0,109389972	NA
Slfn10-ps	0,782299389	1,291453848	1,784708378	0,723621777	0,469297968	NA
G630064G18Rik	0,087021394	-0,517475177	3,352475198	-0,154356154	0,877328924	NA
Gm21284	3,393982438	-0,239743384	0,764004944	-0,31379821	0,753674304	NA
8430426J06Rik	1,798139048	0,154575697	1,058602024	0,146018705	0,883906622	NA
Gm6297	22,88139953	-0,156967144	0,373392482	-0,420381105	0,674207069	0,888627551
9330102E08Rik	15,05960459	0,050423708	0,396768531	0,127085955	0,898872367	0,971783074
Gm15008	0,122496332	0,780932884	3,352475198	0,232942181	0,815806304	NA
A930006K02Rik	5,556195764	1,693808241	0,691688567	2,448801849	0,014333228	0,130265751
2610027K06Rik	1,463776838	-0,343744789	1,310261972	-0,262348138	0,793053052	NA
AI839979	0,413979771	1,038228131	2,527692918	0,410741401	0,681262165	NA
D830026I12Rik	1,546539432	0,892659982	1,184139003	0,753847293	0,450940922	NA
A330033J07Rik	0,213116452	1,337107854	3,350237755	0,399108348	0,689813372	NA
5830444B04Rik	13,15434255	0,403526552	0,70752391	0,570336276	0,568449642	0,840292266
4833411C07Rik	366,8744354	0,221112642	0,236278722	0,935812759	0,349369601	0,701099099
AI314278	0,468350644	1,251550749	2,486830678	0,503271397	0,614773473	NA
Arhgap35	2451,624439	-0,268627679	0,119487276	-2,248169749	0,024565368	0,178292292
C130046K22Rik	56,60720168	-0,026208716	0,218986327	-0,119681976	0,904735079	0,973666426
C230037L18Rik	6,195289086	0,111418191	0,666747484	0,167107029	0,867285835	0,961775826
Gramd1b	15,55095876	0,368661445	0,381041564	0,967509793	0,333289248	0,689998647
Teddm1b	1,171629515	1,923119298	1,49453707	1,286765874	0,198175905	NA
Slco5a1	0,273733182	-0,661182784	2,816590698	-0,234745781	0,814406052	NA
Gm28042	0,427322268	0,014665161	3,020716141	0,004854862	0,996126395	NA
Klra14-ps	0,582186543	-0,999440238	2,389718777	-0,418225043	0,675782588	NA
Dock4	595,7079512	0,013683763	0,216583616	0,063180049	0,949623132	0,985972877
Grhpr	4994,470041	-0,149276369	0,151200845	-0,987272056	0,323509286	0,680937786
Gm3704	218,3085049	0,549971719	0,25574217	2,150492894	0,031516248	0,206236277
Prkar1a	3063,090647	0,06604117	0,073453836	0,899084017	0,368607911	0,716892516
Setdb2	5,69754791	0,696249591	0,649519155	1,071946203	0,283744196	0,645033084
Prrxl1	0,093953095	-0,517475177	3,352475198	-0,154356154	0,877328924	NA
Zfp738	82,15888194	-0,25695898	0,23609934	-1,088351115	0,276440133	0,637501364
Zfp456	22,40904567	0,120522495	0,308758725	0,390345227	0,696281284	0,897385953
Primpol	181,8971009	0,189059573	0,19065488	0,991632488	0,321376835	0,678746017
Tmem204	203,8372262	-0,102334409	0,24541787	-0,416980266	0,676692842	0,889548339
Baz2b	291,7634958	-0,235808286	0,1809992	-1,30281397	0,19263828	0,541552905
Lsm12	375,8790892	-0,172266701	0,104702503	-1,645296864	0,099908606	0,392993389

Cbr3	11,62878973	0,665080154	0,483981013	1,374186458	0,169383804	0,508874623
Gm21283	0,113662148	0,780932884	3,352475198	0,232942181	0,815806304	NA
Dreh	27,39293962	0,502555485	0,387781022	1,295977513	0,194983232	0,544089091
Adam10	692,6463137	-0,068206376	0,134752498	-0,50616038	0,612744066	0,859036852
Dgkeos	0,463855677	-0,107586204	2,186279157	-0,049209729	0,960752158	NA
4930596I21Rik	1,049798139	0,664684699	1,619962942	0,410308583	0,681579597	NA
9030619P08Rik	344,0556348	2,147893944	0,847852843	2,533333422	0,011298343	0,113783449
Gm16740	36,62330375	0,046084348	0,248064933	0,185775343	0,852620942	0,95618567
Gm16712	3,280062567	0,166366855	0,845981743	0,196655373	0,844097235	NA
Gm20319	1406,326645	0,215055336	0,138732236	1,550146837	0,121106276	0,433132274
Ttc12	12,08437099	0,971591877	0,422341606	2,300488189	0,021420578	0,163367723
2210406O10Rik	9,967933515	0,457916863	0,527423627	0,868214542	0,385276895	0,729677128
E230029C05Rik	8,629761698	1,134597966	0,547544027	2,072158421	0,03825067	0,229727404
Lypd1	1,863954969	2,064078278	1,401370867	1,472899378	0,14077817	NA
Cnga4	0,453963022	1,234906816	2,502925914	0,493385285	0,621740377	NA
Tmevpg1	0,209517726	0,059593471	3,352475198	0,017775962	0,985817581	NA
Gm15694	6,216204566	-0,351834571	0,672367303	-0,523277335	0,600781271	0,854422974
Mir5620	0,375775812	1,987694774	3,082915622	0,644745111	0,519092378	NA
Arih1	1086,715757	-0,203315581	0,095182831	-2,136053098	0,032675086	0,210319319
Tmsb4x	2042,583286	0,242663794	0,201341835	1,205232849	0,228113425	0,584111928
Rps6ka4	207,5212095	0,108589279	0,140369037	0,773598514	0,439168251	0,766717272
Cnbd2	13,99125857	-0,360691602	0,517437074	-0,697073364	0,485756882	0,79430833
1500026H17Rik	42,21858811	-0,188112086	0,259247882	-0,725607032	0,468079709	0,783100692
Tmem209	293,0561759	0,274777026	0,115018533	2,388980436	0,016895202	0,14387057
4930459C07Rik	9,257562913	2,013287814	0,618062962	3,257415408	0,001124318	0,025826465
9030625G05Rik	1,321380669	0,385809307	1,350693568	0,285637924	0,775155454	NA
Gm1647	0,093953095	-0,517475177	3,352475198	-0,154356154	0,877328924	NA
Uph	31,12113275	0,55602679	0,265377279	2,095231332	0,036150433	0,222803109
9230116L04Rik	0,220592835	0,059593471	3,352475198	0,017775962	0,985817581	NA
Gm16159	0,093953095	-0,517475177	3,352475198	-0,154356154	0,877328924	NA
Gm30505	205,7785372	0,432503086	1,186565856	0,364499858	0,71548478	0,90658298
A330074K22Rik	1,056230545	0,353301742	1,889244476	0,187006895	0,851655227	NA
LOC102631757	187,9383605	0,580216687	0,137544627	4,218388602	2,46E-05	0,001618665
AW822252	0,173498105	0,059593471	3,352475198	0,017775962	0,985817581	NA
A230103J11Rik	10,68845572	-0,314603429	0,485080243	-0,64855956	0,516623102	0,813424627
Zeb2os	15,63160395	0,312185998	0,397903127	0,784577896	0,432701094	0,762761198
1700047A11Rik	0,241026348	0,059593471	3,352475198	0,017775962	0,985817581	NA
Lin9	59,0445355	0,286527842	0,213139757	1,34431908	0,178845228	0,5215929
Mir762	0,824417403	1,274370652	1,647771745	0,77339028	0,43929144	NA
Mir674	0,087021394	-0,517475177	3,352475198	-0,154356154	0,877328924	NA
Mir670	0,413255311	2,173474447	2,471778342	0,879316082	0,379229918	NA
Tcf15	0,379918946	1,000946436	2,589354372	0,386562167	0,699080371	NA
Hnf1a	1025,8834	0,292826193	0,119285241	2,454840108	0,014094726	0,12927605
Emc3	1314,274299	-0,075066333	0,075905593	-0,988943369	0,32269085	0,680021118
Ifnlr1	4,75137555	0,938302591	0,809702793	1,158823458	0,246528154	NA
Zfp641	5,914767406	0,439291857	0,668580671	0,657051387	0,511147875	0,80946164
Mir802	0,093953095	-0,517475177	3,352475198	-0,154356154	0,877328924	NA
Mir677	1,629196135	-1,107217261	1,543614138	-0,717288883	0,473195865	NA
Mir744	0,086476712	0,780932884	3,352475198	0,232942181	0,815806304	NA
Nck1	512,5868091	-0,182235787	0,149022842	-1,222871502	0,221378234	0,57602289
Mamdc2	54,54724599	-0,316292694	0,261486321	-1,209595564	0,226434121	0,581402791
Disc1	1,839189286	0,214586788	1,29771691	0,165357164	0,868662867	NA
Phf12	613,9941399	0,073099538	0,11789243	0,620052857	0,535222988	0,822553871
Micall2	59,1174131	-0,138900757	0,231066309	-0,601129423	0,547753787	0,828572118
Gm38670	7,463675732	-0,469395955	0,60733528	-0,7728778	0,439594704	0,766945637
Prr12	286,7934791	-0,287349992	0,133642106	-2,15014564	0,031543696	0,206314911
Arl16	163,4494786	-0,149075737	0,146252279	-1,019305391	0,308058006	0,667059844
Morn4	6,938277443	-0,287506222	0,623285172	-0,461275569	0,644600911	0,874269004
Pcgf1	124,346957	-0,140113142	0,177221834	-0,790608805	0,429172307	0,760857915
Gm1965	0,273733182	-0,661182784	2,816590698	-0,234745781	0,814406052	NA
4933427E11Rik	1,707530459	3,301482118	1,566238573	2,107905	0,035039208	NA
Tmem181b-ps	17,51478874	0,400165412	0,382723677	1,045572657	0,295758379	0,656918718
Cahm	5,342300692	-0,552421872	0,755198658	-0,731492126	0,464478612	NA
Mir421	0,285269197	-1,521344816	3,321239936	-0,458065315	0,646905517	NA
Mir652	0,122496332	0,780932884	3,352475198	0,232942181	0,815806304	NA
Mrpl10	1248,225713	-0,03195726	0,069823509	-0,457686248	0,647177869	0,876230372

Supplementary Table S1: *Serpina1* KO vs. wildtype all DEGs

Hoga1	3861,250235	0,274086728	0,108212101	2,532865788	0,011313426	0,113783449
Celf5	0,179780087	0,059593471	3,352475198	0,017775962	0,985817581	NA
Tmem198	2,25478854	0,897391541	1,017026793	0,88236765	0,377578024	NA
A630001G21Rik	13,7771415	0,713979649	0,412211779	1,732069983	0,083261103	0,357192041
Mir669n	0,093303375	-0,517475177	3,352475198	-0,154356154	0,877328924	NA
Mir3113	2,842002109	-1,072010911	1,168474509	-0,917444842	0,358909584	NA
Vmn1r4	0,12663974	0,780932884	3,352475198	0,232942181	0,815806304	NA
A930002C04Rik	1,204268953	0,39406647	1,350105028	0,291878381	0,770379613	NA
A330015K06Rik	0,187906191	-1,241948083	3,339949325	-0,371846385	0,710007226	NA
Gm3363	0,179780087	0,059593471	3,352475198	0,017775962	0,985817581	NA
Gm11681	0,677368603	2,819293777	2,691240386	1,047581551	0,294831441	NA
Gm11423	42,8378696	0,195510338	0,292283777	0,668905884	0,503555517	0,804978322
Gm15972	0,894604011	1,465870496	1,952692871	0,750691784	0,452838168	NA
Gm1600	27,63874756	0,915200698	0,441790071	2,071573713	0,038305214	0,229748297
4930506A18Rik	0,481243387	-1,55094357	2,058283273	-0,753513178	0,451141595	NA
Ankrd46	1418,280636	-0,226868365	0,122506885	-1,851882561	0,064042689	0,308060036
2310022B05Rik	347,6735382	0,400897154	0,16795509	2,386930665	0,016989695	0,144212847
Ptk7	22,51926428	0,061811835	0,309995345	0,199396009	0,841952982	0,952074658
Tatdn1	191,6007632	0,188914049	0,158103595	1,194875101	0,232135832	0,58906385
1700022H01Rik	0,294091955	0,617264955	2,767052958	0,223076668	0,823475837	NA
6330415G19Rik	10,95283883	0,634598345	0,487927809	1,300598844	0,193395803	0,541921713
3830432H09Rik	0,093303375	-0,517475177	3,352475198	-0,154356154	0,877328924	NA
Hsdl1	215,8837236	-0,076990423	0,13745106	-0,560129713	0,575390964	0,843846408
Mvb12b	46,30723264	0,359198247	0,233636432	1,537423955	0,124189524	0,437955618
Eif4h	3118,655451	-0,07231568	0,069406339	-1,041917507	0,297449927	0,658152471
Amer1	128,3506517	-0,263778979	0,210038565	-1,255859746	0,209166825	0,561671273
Hist3h2ba	0,473767003	-0,57532997	2,141592808	-0,268645827	0,788202244	NA
Trim14	1249,401346	-0,213145408	0,148004613	-1,440126789	0,149831531	0,481977319
Zbtb4	389,3625107	-0,131273391	0,141921253	-0,924973455	0,354979716	0,705239841
Bloodlinc	4,567769425	-0,424820061	0,790670754	-0,537290723	0,591066812	NA
Ppp1r1b	255,478595	-0,149015726	0,24683558	-0,603704399	0,546040189	0,828069865
Syna	0,187906191	-1,241948083	3,339949325	-0,371846385	0,710007226	NA
Fam222a	290,5736931	-0,3597491	0,239175728	-1,504120435	0,132550359	0,453057798
Gm15326	1,024567689	0,538430532	1,682008531	0,320111653	0,748883692	NA
Bola3	1191,805504	0,090969071	0,076987705	1,181605171	0,237362401	0,594575536
Tmem62	401,7796622	0,364204657	0,11685447	3,116737047	0,001828646	0,036111032
Nlrp4f	0,113662148	0,780932884	3,352475198	0,232942181	0,815806304	NA
Tprn	92,16894867	0,038316151	0,184606567	0,207555732	0,835575872	0,951141594
Slc25a16	1336,535204	-0,087183625	0,124309731	-0,701341917	0,48308966	0,79399946
Dzip1l	13,60589764	0,309952054	0,464049731	0,667928529	0,504179215	0,804997854
Ube2e3	565,6427386	0,171143932	0,116827004	1,464934698	0,142938735	0,470144068
Lemd2	493,406396	-0,058710303	0,108863765	-0,539300685	0,589679396	0,84953676
Adgrv1	42,25171311	-0,712278966	0,498818294	-1,427932724	0,153311224	0,488120445
Fam126a	244,4864526	0,529551268	0,267909511	1,976604955	0,048086302	0,262394191
Mcoln1	715,744758	-0,217675002	0,107846488	-2,018378224	0,043551886	0,245880557
Snx4	872,9480894	0,080750604	0,085071339	0,949210446	0,34251359	0,695589013
Epdr1	12,12181725	1,049281594	0,53554619	1,959273755	0,050080736	0,268956645
Kdelr1	2143,631558	-0,018707731	0,070079146	-0,266951472	0,789506525	0,935361858
Nt5m	212,6556879	-0,058800812	0,139276736	-0,422186893	0,672888607	0,888566554
Itga5	1069,351406	0,001268022	0,16716017	0,007585668	0,993947571	0,998278497
Hspb8	4204,95648	0,184665409	0,149363611	1,236348044	0,21632922	0,56973247
H2afv	651,74266	0,053052999	0,097808576	0,542416636	0,587531522	0,848648685
Zfp142	132,4925857	-0,124055502	0,176546616	-0,702678447	0,482256163	0,793477208
Atg4b	741,1800192	-0,070613572	0,078576528	-0,898659856	0,368833866	0,717019042
Pank4	294,5320103	-0,123886838	0,116823161	-1,060464696	0,288933243	0,650101875
Ankle1	5,649784902	-1,159035001	0,684326359	-1,6936875	0,090324681	0,372867926
Ttc26	15,04140794	-0,157981986	0,405056074	-0,390024977	0,696518077	0,897560279
Dars2	462,3545282	0,040096615	0,106081493	0,37797936	0,705445927	0,902021937
Txndc16	101,0050479	0,502224217	0,240631088	2,087112772	0,036877942	0,225226681
Sgpl1	2373,640378	-0,230050882	0,103934675	-2,213418009	0,02686884	0,189333814
Ncapg2	32,53246972	-0,419399505	0,414339099	-1,012213201	0,311436126	0,670897449
Ccdc126	36,26675464	-0,17760971	0,240638789	-0,738075979	0,46046828	0,780953195
Mgst2	1,49182426	0,391840785	1,194709057	0,327980091	0,742926716	NA
Ube2s	211,5373008	-0,480944276	0,182240892	-2,639057958	0,008313677	0,094031762
Gm1673	1,542213561	0,546121044	1,440934619	0,379004736	0,704684347	NA
1810030O07Rik	199,2540378	0,431679858	0,142712414	3,024823454	0,002487783	0,043465162

Supplementary Table S1: *Serpina1* KO vs. wildtype all DEGs

Synpo2l	3,361627067	-0,010233264	0,859502978	-0,011906025	0,990500591	NA
Mbnl3	25,81545099	0,265753396	0,504273354	0,527002654	0,598191745	0,853846018
Trib2	37,03169912	0,167706122	0,381850798	0,439192802	0,66052184	0,882607568
Trib1	989,213922	-0,186979575	0,153996355	-1,214181818	0,224678302	0,579757618
Mllt6	373,2373114	0,140664255	0,13770032	1,021524531	0,307005993	0,666222391
Il13ra1	971,2421459	0,317387966	0,165427226	1,918595715	0,055035518	0,284066998
Als2cr12	5,951020141	3,98104012	1,304417501	3,05196773	0,002273465	0,041521802
Zfp526	60,34555609	-0,183571525	0,224728101	-0,81686057	0,414008113	0,75089872
Lincred1	0,086476712	0,780932884	3,352475198	0,232942181	0,815806304	NA
Ube4bos3	0,220592835	0,059593471	3,352475198	0,017775962	0,985817581	NA
Gm10371	0,187906191	-1,241948083	3,339949325	-0,371846385	0,710007226	NA
Ugt1a1	5032,367479	0,088476074	0,179336004	0,493353661	0,621762719	0,862965544
Etv4	2,515591954	0,894365734	0,982908917	0,909917205	0,362866176	NA
Glis3	17,90970425	0,908808588	0,438389399	2,07306242	0,038166471	0,22938054
Pum3	782,6491121	0,096027527	0,097516698	0,984729065	0,324757172	0,682117617
Rint1	414,3734896	-0,32751793	0,123828201	-2,644938118	0,008170586	0,092884831
Zfp653	127,3474416	-0,081093552	0,170327651	-0,476103271	0,634000807	0,867989994
Mdrl	1,179739239	2,794141089	1,488572673	1,877060582	0,060509791	NA
LOC100049077	2,333507635	1,611516546	1,024978024	1,572244973	0,115893749	NA
Mir155hg	0,8166455	0,387337281	1,718787132	0,225355004	0,821703113	NA
Cenpi	3,863133034	-0,954056811	0,776874637	-1,228070483	0,21942051	NA
Nepro	126,2043126	-0,019542879	0,204933624	-0,095361996	0,924027301	0,977951927
B4galt7	284,0173811	-0,40552536	0,126339749	-3,209800259	0,001328272	0,028782722
Tpcn2	72,30117675	0,297843277	0,229926935	1,295382277	0,195188389	0,544259529
Pcdhga6	1,94184666	0,796145889	1,068609163	0,745030004	0,456253582	NA
Mir7-1	5,026022363	0,040691211	0,657367727	0,061900226	0,950642287	NA
Ptafr	23,88185032	0,036317428	0,356077724	0,101992979	0,918762248	0,976907366
B3gnt6	0,427322268	0,014665161	3,020716141	0,004854862	0,996126395	NA
Clk2-scamp3	0,267451201	-0,629501011	2,83229786	-0,222258054	0,824113003	NA
Hapln3	0,241026348	0,059593471	3,352475198	0,017775962	0,985817581	NA
Cpa3	0,726475513	2,092190592	2,284040359	0,916004213	0,359664685	NA
Pomt2	499,0155863	-0,341035952	0,096442768	-3,536148537	0,000406006	0,012741546
Ankrd23	64,43236935	-0,535647583	0,272918478	-1,962665137	0,049685097	0,267192299
Pms1	165,3955484	0,481313532	0,215220876	2,236370095	0,025327538	0,181982407
Pif1	0,846954489	-0,518220805	1,675668014	-0,309262217	0,75712207	NA
Ube2z	1184,463107	-0,001291245	0,0809612	-0,015948934	0,987275131	0,996231603
Larp4b	1436,344422	-0,055879084	0,111778	-0,499911292	0,617137541	0,860830256
Scara3	122,2819926	0,995718212	0,328069005	3,035087731	0,002404657	0,042750903
Arl15	268,4904396	-0,066690502	0,169076369	-0,394440114	0,693256133	0,895850714
Tns4	9,428645221	0,567123642	0,608344727	0,932240582	0,351212207	0,702687219
Cpne2	228,2477365	-0,655441741	0,155351492	-4,219088812	2,45E-05	0,001618665
Neurl3	171,3575394	0,09105634	0,20429306	0,445714311	0,655803625	0,879592859
Dsg1b	1,937939419	3,55511196	1,438293455	2,471757031	0,013445086	NA
Lrrtm3	0,293986917	-0,62664923	2,757017991	-0,227292398	0,820196384	NA
Pde4c	380,6546767	0,173051332	0,169821432	1,01901939	0,308193761	0,667160859
Flrt2	10,0096867	0,787535637	0,46312634	1,700476888	0,08904126	0,369687145
Ccbe1	83,27314988	-0,147287931	0,28166623	-0,52291654	0,601032334	0,854422974
H1fx	4,993791976	-0,650404927	0,687628331	-0,945866973	0,344216441	NA
Sep-05	48,56960827	-0,680281301	0,350248431	-1,942282222	0,052102944	0,275125315
Mtss1l	66,84992949	-0,23387748	0,230389045	-1,015141501	0,310038381	0,669180355
Zfp626	98,98895717	-0,005032743	0,199717373	-0,025199325	0,979895975	0,994664228
9430078K24Rik	0,122496332	0,780932884	3,352475198	0,232942181	0,815806304	NA
Gm31763	0,307069547	0,617382779	2,733615373	0,225848444	0,821319297	NA
Rpl14-ps1	24,99815263	0,040801091	0,289219622	0,14107304	0,887812242	0,968360648
Gm35978	1,709881951	1,50075577	1,525408766	0,983838433	0,325194959	NA
Lncpint	1,377598706	-0,502535052	1,451883568	-0,346126276	0,729247817	NA
Jpx	44,07473024	0,159212671	0,28120713	0,566175799	0,57127428	0,841381253
Pvt1	47,48158704	0,035123478	0,265785857	0,132149536	0,894866015	0,970433882
Zfp512b	620,6941476	0,169590392	0,118854152	1,426878148	0,15361502	0,488738507
Gpr61	0,295445946	1,389394708	3,349408814	0,414817893	0,67827522	NA
Aldh2	55840,84599	0,08488962	0,103238646	0,822265917	0,41092557	0,748353623
Ttc6	40,12129032	0,082044574	0,268006308	0,306129265	0,759506227	0,924882144
Gm10037	9,824027224	-0,575876561	0,428866327	-1,342788009	0,179340627	0,522135977
2310009A05Rik	165,6153929	0,069924045	0,130846061	0,534399313	0,593065294	0,851477265
Lrrc31	0,122496332	0,780932884	3,352475198	0,232942181	0,815806304	NA
Cnpy1	0,949551637	2,51123264	1,628864109	1,541707885	0,123144595	NA

Supplementary Table S1: *Serpina1* KO vs. wildtype all DEGs

Crybb1	0,113662148	0,780932884	3,352475198	0,232942181	0,815806304	NA
BC051628	3,049539361	0,420536384	1,23032495	0,341809198	0,732494487	NA
Tmtc1	16,60582752	0,517307638	0,374289894	1,38210421	0,166939706	0,505901813
Crocc2	0,086476712	0,780932884	3,352475198	0,232942181	0,815806304	NA
Cdk14	37,58284998	0,347125583	0,272675869	1,273033743	0,203006068	0,555491529
Sep-11	481,4876204	-0,583796288	0,218857244	-2,667475274	0,007642351	0,089154096
Mmp27	11,88138873	0,137310059	0,500499861	0,274345847	0,783818854	0,933246486
Trim5	30,90787955	0,402401593	0,38680151	1,040330978	0,298186157	0,658808582
Ociad2	805,1033812	-0,336009727	0,129083261	-2,60304647	0,009239943	0,100023317
D3Ertd254e	137,6704173	0,115899219	0,173049503	0,669746042	0,503019696	0,804506557
Morc3	295,6732244	-0,055988406	0,12443334	-0,449946988	0,652748666	0,877662367
Adgrd1	26,57136022	0,819560201	0,38019855	2,155611062	0,031114048	0,204593286
Zfp951	54,22559634	-0,530094366	0,357368641	-1,483326472	0,137987692	0,461420075
Wdr83os	784,5983865	0,128537263	0,099079822	1,297310197	0,194524477	0,543617834
Larp1b	2342,114343	-0,097075471	0,090892355	-1,068026789	0,285508428	0,646942741
Zfp442	48,09852723	-0,181362112	0,269261938	-0,673552725	0,500595718	0,803513525
Rpe	653,5462548	-0,151333165	0,171723655	-0,881259865	0,378177184	0,723919068
Nmnat1	278,2993026	-0,187565657	0,148897119	-1,259699708	0,207777711	0,560178344
Gm15867	0,207615244	0,059593471	3,352475198	0,017775962	0,985817581	NA
Gm13648	1,200126669	0,004434506	1,314403874	0,003373778	0,99730812	NA
Panct2	0,754348384	-0,260766877	2,000526151	-0,130349147	0,896290198	NA
Mmp3	0,556786151	1,588729592	2,399291923	0,662166023	0,507864826	NA
Rnf113a1	33,98050396	-0,116553068	0,2844314	-0,409775671	0,681970514	0,89172987
Stard3nl	181,3902251	0,082282285	0,123555344	0,665954889	0,505439935	0,805643837
Cyp27a1	15264,65447	0,067634779	0,127936709	0,528658114	0,597042638	0,853484526
Esm1	35,27288984	-0,354817661	1,302932518	-0,272322362	NA	NA
Abcb6	1749,908651	-0,091854028	0,075430789	-1,217725936	0,223328138	0,578394783
Cfap36	329,2631763	0,088652535	0,116583944	0,760418048	0,447004738	0,772195705
Ube2q1	885,9599402	-0,07771826	0,104779499	-0,741731551	0,45825	0,779234832
Dnaaf2	88,55771378	0,311372215	0,197307607	1,578105477	0,11454138	0,418961344
Pold4	71,65214001	0,13129576	0,209032724	0,628111031	0,529931207	0,819306989
Asb11	0,449704146	-0,148438176	2,207290246	-0,067249052	0,946383436	NA
Ube2c	15,79318612	-1,082238088	0,610445037	-1,77286737	0,076250687	0,341964251
Plk1	11,3492641	-0,941399356	0,578596454	-1,627039624	0,103728683	0,400910309
Magee1	87,20239774	-0,342006864	0,167256971	-2,044798864	0,040874692	0,238521936
Gm35612	3,701809281	-0,148772845	0,723325294	-0,205679031	0,837041639	NA
Ggnbp2os	49,70479118	0,107997133	0,237180261	0,455337776	0,648866253	0,876446263
Ttc39aos1	25,38487971	0,381452039	0,461475284	0,826592565	0,408468033	0,746898413
Hoxaas2	1,413715175	-1,857506053	1,400002424	-1,32678774	0,184578907	NA
Hotairm1	4,990228883	-0,082092977	0,678426876	-0,121004901	0,903687153	NA
2210404E10Rik	0,113662148	0,780932884	3,352475198	0,232942181	0,815806304	NA
Gm8013	0,433709287	1,155703805	2,51809517	0,458959542	0,64626322	NA
A430046D13Rik	97,11296376	-0,000937616	0,175759597	-0,005334651	0,995743585	0,998991922
Gm29685	0,499732023	2,454007834	2,362230605	1,038851935	0,298873603	NA
Lgals3bp	2465,678242	-0,071567928	0,138735264	-0,515859674	0,605952421	0,856521242
Trim25	1715,220064	0,10701588	0,144220351	0,742030368	0,458068937	0,779143636
Gemin2	250,7998798	-0,27286653	0,126594368	-2,155439733	0,03112744	0,204593286
Ush1g	8,24129913	0,083106001	0,511004157	0,162632729	0,870807616	0,961784159
Ube2g2	977,5804185	-0,351373103	0,117334007	-2,994639928	0,00274769	0,046557499
Gm5532	4,004197442	1,366739879	0,786030644	1,738787016	0,082072233	NA
LOC100041034	0,381008311	-1,101268028	2,549068975	-0,432027551	0,665721389	NA
LOC106557447	35,70366033	0,191558553	0,299491447	0,639612767	0,52242438	0,815322684
Clcn7	786,2833181	-0,078757555	0,097661554	-0,80643357	0,419992897	0,75408072
Nnmt	2312,754597	0,955716316	0,562725651	1,698369913	0,089437967	0,370643577
Gm12348	0,485294552	2,361941835	2,406379013	0,981533592	0,326329678	NA
LOC102640359	11,49692785	0,238357285	0,477393472	0,49928895	0,617575839	0,86111508
Rab1a	3797,369762	-0,183755007	0,09174919	-2,002797045	0,045199077	0,251865618
Camp	1,210107706	-0,430674631	1,70948105	-0,251932966	0,801092878	NA
Cxcr3	9,708718814	1,091065325	0,509362887	2,142019672	0,032191899	0,208308055
Usp5	1590,058163	-0,066165975	0,081335062	-0,813498801	0,415932133	0,751551608
Uty	245,8577639	-0,265746168	1,102374307	-0,241067092	0,809503118	0,94241441
Map2k4	913,6452209	0,081592004	0,087694621	0,93041058	0,352158543	0,703107461
Aurkb	11,26172498	-1,428806685	0,557794517	-2,561528736	0,010421262	0,107584441
Rnf167	1062,888629	0,209530547	0,12683262	1,652024116	0,098529635	0,390989221
Pctp	2818,578357	0,237691391	0,168313283	1,412196267	0,157892179	0,493680148
Traf1	10,3205292	0,594508488	0,471642382	1,260506925	0,207486551	0,559928887

Kcnk10	17,94787649	-1,241273189	0,435136029	-2,852609542	0,004336186	0,061982086
Nudt14	230,5908915	0,254720601	0,105444097	2,415693321	0,015705283	0,13759104
Nkiras1	503,3964232	-0,193148066	0,138304093	-1,396546282	0,162550054	0,499455681
Il17ra	367,1320906	0,036890057	0,122247809	0,301764563	0,762831547	0,925557106
Gabrr2	2,313891868	-1,358722876	1,015741034	-1,337666621	0,181005136	NA
Purb	632,7556031	-0,04092146	0,119298328	-0,343017881	0,731585008	0,912195484
Nudt16l1	391,5812377	0,10040488	0,119151536	0,842665423	0,399415591	0,740505759
Chd7	299,7629347	-0,330155208	0,149763628	-2,204508612	0,027488595	0,191005731
4930405O22Rik	0,180429807	0,059593471	3,352475198	0,017775962	0,985817581	NA
Mrpl2	913,0318636	-0,018612477	0,11659072	-0,159639439	0,873165109	0,963097638
Qpct	190,6785214	-0,272820406	0,220646899	-1,236457013	0,216288735	0,56973247
Gabrd	2,098311694	-0,345396167	1,05255741	-0,328149481	0,742798643	NA
Slc27a3	17,00920331	-0,184426867	0,382289987	-0,482426621	0,629502914	0,866228449
Bmp8b	3,273750456	-0,413120957	1,243132069	-0,332322661	0,73964563	NA
Selenok	1599,806722	-0,10902802	0,105130264	-1,037075493	0,299700676	0,659904651
Rnf223	3,055078277	-1,23679655	0,852290696	-1,451144024	0,14673976	NA
Brip1os	602,4615215	0,079640482	0,1137293	0,700263542	0,483762736	0,794055997
Gm11532	1,008842912	2,621757909	1,658385605	1,580909712	0,113898683	NA
Zfp369	65,18417767	-0,388543946	0,269953629	-1,439298843	0,150065871	0,482266977
Il22ra1	280,4490405	0,127004903	0,290400982	0,437343229	0,661862449	0,882945528
Hist1h4k	4,547047516	0,393647682	0,896173364	0,439253941	0,660477544	NA
Hist1h2bf	0,093953095	-0,517475177	3,352475198	-0,154356154	0,877328924	NA
Hist1h4b	2,885113473	-1,178168889	1,057207364	-1,114416083	0,265100727	NA
Hist1h2bm	0,113662148	0,780932884	3,352475198	0,232942181	0,815806304	NA
Lman2l	355,0591881	-0,493771481	0,134464883	-3,67212219	0,000240545	0,008621558
Clnk	0,506806999	-0,108289439	2,160441672	-0,050123751	0,960023773	NA
Lss	3350,484219	0,730422818	0,216505759	3,373687702	0,000741685	0,019409183
Tbcc	206,244636	0,218680452	0,14854332	1,472166181	0,140976008	0,466511942
1700001L05Rik	87,44719657	0,52562929	0,211066284	2,490351756	0,012761672	0,121604273
Hnf4aos	133,8366389	0,66130418	0,277673012	2,381593282	0,017237924	0,145577599
Zbtb11os1	60,61458102	0,488167287	0,340445596	1,433906892	0,151598835	0,48474643
Gm8883	457,4070313	0,742081117	0,17704913	4,191385286	2,77E-05	0,001748415
1700096K18Rik	49,4087694	0,596648691	0,223659802	2,667661712	0,007638112	0,089154096
Snhg20	78,3601166	0,117565963	0,263850785	0,445577461	0,655902494	0,879592859
Abhd11os	10,28810832	0,496692116	0,585091565	0,848913478	0,395929439	0,737426273
Gm14057	27,51551509	-0,697166368	0,340186254	-2,049366661	0,040426275	0,236524801
Rab10os	113,0620174	0,285631641	0,147417645	1,937567523	0,052676008	0,276927075
Firre	721,5313677	0,174440321	0,196962945	0,88565045	0,375805917	0,721655743
Wt1os	0,173498105	0,059593471	3,352475198	0,017775962	0,985817581	NA
Zfhx2os	0,274362713	1,389394708	3,349408814	0,414817893	0,67827522	NA
Snhg3	97,22721545	0,232073201	0,207705126	1,117320528	0,263857302	0,624609263
Snora62	1,131968045	-0,203927708	1,340449807	-0,152133789	0,879081415	NA
Npm3-ps1	0,420731695	1,124606493	2,207195302	0,509518343	0,610388944	NA
Kcnq1ot1	29,71606857	0,100095199	0,361152632	0,277154837	0,781661216	0,932196631
Magef1	15,74593427	0,160949803	0,391147471	0,411481128	0,680719776	0,89166825
Gm13830	0,330111575	0,664020704	2,683347155	0,247459857	0,804552355	NA
Kif16bos	1,071958515	0,371833	1,460054004	0,254670717	0,798977435	NA
Etaa1os	19,24790025	-0,183091857	0,363536051	-0,503641541	0,614513296	0,859778545
Hist3h2bb-ps	0,980420892	-0,695337471	1,616866615	-0,430052463	0,667157478	NA
Heca	476,7973831	0,028262437	0,166924518	0,169312675	0,865550706	0,961775826
Shisa3	3,67897367	-0,084686203	0,831854793	-0,10180407	0,918912195	NA
Arid4a	495,3554351	-0,164425616	0,126949021	-1,295209801	0,195247865	0,544259529
Gucy1a2	29,28036025	-0,220257021	0,359867091	-0,612051023	0,540504	0,826407188
Mcu	569,1711484	0,331559458	0,18734738	1,769757649	0,076767522	0,342558411
Gm266	0,213116452	1,337107854	3,350237755	0,399108348	0,689813372	NA
Pls1	40,32351297	1,754996433	0,513386007	3,418473446	0,000629735	0,017215486
Myzap	20,01140622	0,089244659	0,436615298	0,204401126	0,83804005	0,951141594
Sdhaf1	342,6553447	-0,176975398	0,141344187	-1,252088267	0,210537703	0,562962817
Cfhr2	10317,94426	-0,036965042	0,117214073	-0,31536351	0,752485669	0,92117599
Fam196b	0,122496332	0,780932884	3,352475198	0,232942181	0,815806304	NA
Otulin	823,9413582	0,204510301	0,132145574	1,547613708	0,121715329	0,433673318
Ccl26	0,281209566	-1,718135424	3,30990564	-0,519088944	0,603698718	NA
Dtx3l	1163,102361	-0,106761362	0,138905025	-0,768592512	0,442135252	0,769003232
Adam6b	0,093953095	-0,517475177	3,352475198	-0,154356154	0,877328924	NA
Rragb	2,719289461	0,87214245	1,01963938	0,855344024	0,392360723	NA
Erbb2	21,25303499	0,009536354	0,398127785	0,023952998	0,9808901	0,994882668

AI413582	51,73714357	0,498705173	0,297797241	1,674646722	0,094003566	0,380783818
Zfp948	107,7430309	0,161497373	0,248708703	0,649343471	0,516116394	0,813290909
Mex3c	352,6956846	0,012637022	0,165025875	0,076576003	0,93896085	0,982918677
Sirpb1b	4,434544495	1,34140971	0,972618412	1,379173675	0,167841213	NA
Kcnk1	7,898422656	0,541740498	0,607277108	0,892081211	0,372349405	0,719447489
Mug2	17201,61028	0,091096422	0,17441528	0,522296108	0,601464179	0,854422974
Ctso	1378,69699	0,412456488	0,128398563	3,212313896	0,001316704	0,028669412
Gpr18	4,426308749	0,473348836	0,826557272	0,572675182	0,566864644	NA
Sdr16c5	15,27607429	-2,314010618	0,709862921	-3,259799249	0,001114911	0,025698698
Mmp2	84,11777302	-0,203244102	0,218211725	-0,931407799	0,351642658	0,702880198
Rad9a	146,6126768	0,060780514	0,176157955	0,345034172	0,730068681	0,911670524
Mmp16	0,465585499	1,247378868	2,489242062	0,501107902	0,616295186	NA
Mmp17	17,72463461	0,409719776	0,53639142	0,763844762	0,444959758	0,770752398
Mex3a	4,050947423	0,648697343	0,962745286	0,673799552	0,500438759	NA
Zbtb21	355,7536539	-0,216826848	0,163599155	-1,325354331	0,185053657	0,531690351
Klk1b4	122,3854512	-0,569857232	0,212384706	-2,683136859	0,007293514	0,0865959
Klk1b5	0,76065135	-1,383534024	1,742663403	-0,793919251	0,427242442	NA
Spi1	130,1708206	0,165873046	0,222907141	0,744135182	0,456794697	0,77843077
Eif1	3633,580584	0,361348242	0,14492673	2,493316738	0,01265559	0,121248489
Abcd1	734,6942328	0,444044497	0,206260288	2,152835631	0,031331599	0,205567535
4933439C10Rik	75,8438722	0,248623401	0,189432011	1,312467724	0,189362369	0,536693982
Srek1ip1	252,6324751	0,205089393	0,125034208	1,640266257	0,100949818	0,394503982
Ints12	187,8197472	0,185113539	0,14358687	1,289209377	0,197325306	0,546971183
Ppbp	6,326884705	1,091039464	0,659835552	1,65350209	0,098228721	0,390573569
Srrm4os	14,07448455	0,130882932	0,455376493	0,287416971	0,773793068	0,928621142
Fam72a	34,60095652	0,002708312	0,371660771	0,007287053	0,994185824	0,99844278
5031425F14Rik	4,62110509	0,4449896	0,773338119	0,575414026	0,56501132	NA
BC064078	3,250340493	0,64140045	1,096890539	0,584744264	0,558719686	NA
3930402G23Rik	15,76679553	-0,279583187	0,508453855	-0,549869341	0,582408994	0,846789107
Setdb2-phf11c	1,493042191	-2,609201842	1,379117016	-1,891936516	0,05849944	NA
Gm29669	8,225902909	0,741543173	0,527241575	1,406458079	0,159588144	0,495312554
Gm19582	0,180429807	0,059593471	3,352475198	0,017775962	0,985817581	NA
Adgb	53,15055155	0,584633549	0,335070549	1,744807326	0,081018413	0,351895765
Gm29571	31,33464557	0,367348856	0,297512788	1,234732995	0,216929892	0,570555917
Gm8624	0,087021394	-0,517475177	3,352475198	-0,154356154	0,877328924	NA
Sycp2l	0,540987101	1,560820077	2,411217914	0,647316059	0,51742741	NA
Gm5767	1,574403519	-0,752335326	1,111762495	-0,676705078	0,498593094	NA
Gm14486	0,495320179	-0,504313129	2,426647566	-0,20782298	0,835367189	NA
Gm32921	0,505862032	-1,483769114	2,413301995	-0,61482944	0,538667365	NA
Gm20619	0,328697462	-0,630293653	2,671069774	-0,235970493	0,813455567	NA
Gm7443	1,484195108	-0,84899308	1,240174788	-0,684575342	0,493611931	NA
Slc6a16	14,88989627	-0,278954113	0,497638019	-0,560556273	0,575100068	0,84359368
Adam24	0,086476712	0,780932884	3,352475198	0,232942181	0,815806304	NA
Akt2	2168,781202	-0,096445978	0,064138836	-1,50370639	0,132656983	0,453114769
Gm12339	0,147722973	0,780932884	3,352475198	0,232942181	0,815806304	NA
LOC102634812	1,142822648	-1,487986604	1,602265045	-0,928676944	0,353056533	NA
Adam21	0,733186329	-0,313114738	2,003571037	-0,156278331	0,875813636	NA
Gm6712	72,82283553	0,009201795	0,187930629	0,048963784	0,960948158	0,988470616
Morf4l1b	16,68676876	-0,223549507	0,37785516	-0,591627509	0,554100048	0,832018326
Adam33	24,65209017	0,4571875	0,423578811	1,079344595	0,28043414	0,641643679
Gm12925	0,800678447	-1,51413496	1,707636552	-0,886684557	0,375248755	NA
Gm31520	0,806691598	2,152622482	2,282571487	0,943069032	0,345645595	NA
Gm29443	0,273733182	-0,661182784	2,816590698	-0,234745781	0,814406052	NA
Add1	1101,821616	0,173380849	0,107573792	1,611738757	0,1070188	0,405981674
Adamts8	0,208973043	1,337107854	3,350237755	0,399108348	0,689813372	NA
Alcam	1059,613824	-0,066313464	0,164291228	-0,403633624	0,686482156	0,893161227
Gm15511	0,12663974	0,780932884	3,352475198	0,232942181	0,815806304	NA
Stac	0,783834424	0,571802002	2,240154647	0,255251129	0,798529144	NA
Adamts6	29,84445068	1,587241859	0,335818275	4,726490416	2,28E-06	0,000254494
Serpind1	15404,8504	-0,007183184	0,085601313	-0,083914413	0,93312448	0,981308229
Poll	197,538831	0,102673232	0,131865961	0,778618159	0,436204679	0,76575019
Fastkd3	130,8005005	0,115773121	0,156831032	0,73820289	0,460391167	0,780953195
Fmo1	6629,029753	0,475890432	0,164314156	2,896222967	0,003776839	0,057377917
Gpr85	0,219943115	0,059593471	3,352475198	0,017775962	0,985817581	NA
C1d	625,3882582	0,190775521	0,136732436	1,395246999	0,162941369	0,49993802
Gas2	1367,680215	0,141138188	0,17010292	0,829722311	0,406695804	0,745871812

Supplementary Table S1: *Serpina1* KO vs. wildtype all DEGs

Cst3	3031,205317	0,110436473	0,098560418	1,12049517	0,26250281	0,622562722
Fuca2	236,2535196	0,481719492	0,14938618	3,224659012	0,001261228	0,028033175
Cradd	322,7837744	0,182832027	0,11179092	1,635481903	0,101948065	0,397591507
Gm5092	0,216449427	0,059593471	3,352475198	0,017775962	0,985817581	NA
5033417F24Rik	16,41026501	-0,131424937	0,356985218	-0,368152322	0,712759659	0,905682828
Itga8	115,181418	0,163456391	0,37906073	0,431214257	0,666312589	0,885128477
Ap1m1	787,6899847	0,009346176	0,076051641	0,122892493	0,902192231	0,973248278
Atp2b2	143,9505767	-0,063049188	0,285192055	-0,221076243	0,825033068	0,947537943
Enpp6	30,34143267	-0,078637096	0,290313992	-0,270869122	0,786491695	0,934050588
Fndc5	8,119852795	0,052888718	0,662020763	0,079889818	0,936324888	0,982191945
Adamts12	8,859924176	-0,170309207	0,507104453	-0,335846404	0,736986695	0,914750267
LOC215458	0,093303375	-0,517475177	3,352475198	-0,154356154	0,877328924	NA
Gm4651	0,433709287	1,155703805	2,51809517	0,458959542	0,64626322	NA
Hexa	1487,592364	0,028336172	0,100639688	0,281560606	0,77828045	0,930864224
Habp2	5422,888753	-0,011946752	0,071578597	-0,166903964	0,867445614	0,961775826
Gdf5	0,093953095	-0,517475177	3,352475198	-0,154356154	0,877328924	NA
Rap1gap	126,3303204	0,48744277	0,234641009	2,077398033	0,037764838	0,228038895
Defb36	0,113662148	0,780932884	3,352475198	0,232942181	0,815806304	NA
Pbx4	8,868476529	-0,103570141	0,650965627	-0,15910232	0,873588261	0,963216615
Slc30a10	1488,27493	-0,233884017	0,110876865	-2,109403239	0,03490979	0,218240938
Cebpz	1009,453787	-0,118177107	0,11441925	-1,032842872	0,301677437	0,661990308
Carmil2	12,59443738	-0,522435054	0,420341924	-1,242881152	0,213911632	0,567220469
Pcnp	914,0266068	-0,10286249	0,123746867	-0,831233082	0,40584197	0,745391028
Snx13	744,7298325	-0,008646951	0,109214478	-0,079174039	0,936894193	0,982210769
Capn11	8,1311124	-3,392171079	1,001857339	-3,385882347	0,000709498	0,018708635
Itga1	402,3368775	-0,278454749	0,119835784	-2,323636059	0,020145008	0,158702778
Tcaim	525,4492266	-0,3128996	0,167975456	-1,86276976	0,062494666	0,303271436
Utp18	314,664526	-0,194755882	0,144901838	-1,344053907	0,178930956	0,521729105
Ntrk1	6,674613031	-0,930051681	0,678244817	-1,371262498	0,170293142	0,510344993
Slc25a51	1051,41111	0,014374233	0,154836283	0,092835041	0,926034613	0,978534437
Crebbp	366,8076907	-0,056117812	0,155838535	-0,360102285	0,718770644	0,908246195
Defb30	0,086476712	0,780932884	3,352475198	0,232942181	0,815806304	NA
Ppp1r26	5,762895505	0,028885364	0,595722315	0,048487967	0,961327354	0,988526411
Ckap2	8,721614932	-1,61351856	0,622133829	-2,593523268	0,009499811	0,101415126
Crtc1	225,7793425	-0,093773341	0,13105074	-0,715549881	0,47426933	0,788130102
Panx2	0,408298785	-0,027276467	2,571361419	-0,010607792	0,991536365	NA
Itprip	18,35216908	0,552002935	0,361476379	1,527078855	0,126741404	0,443035621
Tbc1d32	165,7020076	-6,09E-05	0,139375709	-0,000436943	0,99965137	0,999875741
Slc22a4	65,84233096	0,580470781	0,247091502	2,349213862	0,018813098	0,152616689
Psmc5	2096,149428	-0,02850385	0,093520508	-0,30478716	0,760528262	0,925072539
Tra2b	813,2342938	0,066507219	0,163073817	0,407835054	0,683394777	0,892293506
Trp53i11	199,5004549	-0,13226578	0,161063823	-0,821201043	0,411531762	0,748947472
Trp53i13	50,58184985	0,28360731	0,213990517	1,325326532	0,185062873	0,531690351
Snx32	122,5811773	-0,011781525	0,164905143	-0,071444256	0,943044188	0,983437952
Shc2	6,53192252	0,815259374	0,802542648	1,015845545	0,309702944	0,66878027
Klrg2	0,584748855	2,638328875	2,271879867	1,161297705	0,245520853	NA
Cox17	527,0981714	0,033620209	0,133917972	0,251050759	0,801774866	0,939799232
Cdc42bpa	622,3948334	-0,094999323	0,127032605	-0,747834173	0,454560187	0,776615353
Gsk3a	1256,155116	-0,147217709	0,077998702	-1,887437924	0,059101448	0,293856809
Naaladl1	0,093953095	-0,517475177	3,352475198	-0,154356154	0,877328924	NA
Slc38a6	111,521014	0,075460058	0,224582632	0,336001309	0,73686988	0,914722813
Scube3	4,435170138	1,227537487	0,811646082	1,51240487	0,130430897	NA
Gpr17	1,217384872	0,782401704	2,114926051	0,369942818	0,711425097	NA
Miip	320,6307525	0,087795758	0,136841691	0,641586329	0,521141812	0,814845193
Rtp1	0,147722973	0,780932884	3,352475198	0,232942181	0,815806304	NA
Ifi214	1,89468782	0,425943437	1,124655557	0,378732346	0,704886631	NA
Adgrg6	24,5482004	0,09067652	0,340099111	0,266617926	0,789763352	0,935361858
Nat8l	1,315880114	0,547427515	1,347854624	0,406147299	0,684634374	NA
Tmprss13	0,295445946	1,389394708	3,349408814	0,414817893	0,67827522	NA
Slc22a23	2145,093758	-0,140906077	0,126904129	-1,110334855	0,266854759	0,627135392
Bmp15	0,086476712	0,780932884	3,352475198	0,232942181	0,815806304	NA
Gdf11	3,358849632	1,090434728	0,839070528	1,29957458	0,193746817	NA
Gdf3	0,280559845	-1,715696706	3,310037588	-0,518331487	0,604227008	NA
Gdf15	89,92544554	-0,342815664	0,3962219	-0,865211297	0,386922826	0,731133747
Inip	124,8185867	0,100007022	0,143799264	0,695462686	0,486765388	0,795157193
Slc47a2	1,704296495	2,483453272	1,312435753	1,8922475	0,058458012	NA

Supplementary Table S1: *Serpina1* KO vs. wildtype all DEGs

Asap3	375,6672078	-0,568452109	0,193121482	-2,943494966	0,003245291	0,052103804
Nuak1	81,09659981	-0,610550248	0,167575363	-3,643436831	0,000269022	0,009293414
Garem1	577,8727535	0,029742509	0,154841123	0,192084042	0,847676375	0,953880403
Scarf1	274,2953751	-0,08007307	0,184478861	-0,434050113	0,664252051	0,884226961
Rab3ip	675,6878128	0,002941344	0,085764251	0,034295693	0,972641359	0,992650147
Rbm44	0,683389767	0,264508181	1,636681378	0,161612507	0,871611008	NA
Inhbe	868,2113451	0,139088731	0,300822759	0,462361065	0,643822416	0,873795284
B9d1	6,874003194	-0,590415666	0,62744724	-0,940980577	0,346714813	0,698497639
Trpv1	5,575397361	-0,996618843	0,620772958	-1,605448224	0,108395199	0,408552414
Ghrhr	0,827050372	3,098200764	2,19834974	1,409330239	0,158737541	NA
Tfb2m	416,1872016	0,017751076	0,088020308	0,201670228	0,840174539	0,951657825
Med25	987,9920567	-0,002855302	0,108176739	-0,026394789	0,978942451	0,994593468
Tcf7l2	311,600713	-0,358476082	0,162182325	-2,210327676	0,027082429	0,190360145
Surf6	251,7539156	0,036281976	0,136634449	0,26554047	0,790593138	0,935845552
Zfas1	181,7819822	0,232966522	0,210478169	1,106844113	0,268361332	0,628652996
Zfand1	305,4675526	0,117884115	0,117600151	1,002414654	0,316143367	0,67419376
Saa4	7154,409997	-0,139111142	0,180387787	-0,771178269	0,440601273	0,768079074
Upf1	1569,529677	-0,181123674	0,078275249	-2,313932902	0,020671403	0,160578725
Pik3r2	571,6597291	0,014765067	0,110851295	0,133197062	0,894037534	0,970273198
Hsf1	404,1830166	-0,230899358	0,103457839	-2,231820823	0,025626808	0,183556901
Trappc6a	309,478624	0,119724217	0,11984621	0,998982084	0,31780337	0,675759967
Rap1gapos	3,3106789	-0,033001059	0,834096589	-0,039565032	0,968439906	NA
Mettl21c	0,086476712	0,780932884	3,352475198	0,232942181	0,815806304	NA
Trim6	6,384443917	0,795420026	0,679217774	1,171082468	0,24156563	0,599385841
Nsrp1	330,1785046	-0,050374434	0,118826436	-0,423932888	0,671614758	0,887849076
Il19	0,093303375	-0,517475177	3,352475198	-0,154356154	0,877328924	NA
Ppp1r13l	88,91429767	-0,371435708	0,173995833	-2,1347391	0,032782327	0,210743017
Pabpn1l	0,307614229	-0,630030301	2,72139296	-0,231510227	0,816918442	NA
Rab44	2,224569181	0,992508541	1,123864611	0,883121091	0,37717085	NA
Rpl17	7917,628005	0,11169934	0,109924691	1,016144225	0,309560712	0,668581124
Nxpe5	0,905471455	0,184361547	1,646555825	0,111967991	0,910848787	NA
Tpm4	548,3587101	-0,09367492	0,177622074	-0,527383324	0,597927421	0,853662078
Slc35d2	1010,585333	-0,055036031	0,118263131	-0,465369307	0,641667011	0,872059191
Cnnm3	445,2637182	0,098018485	0,10412014	0,941397936	0,346500971	0,698497639
A930033H14Rik	15,9392937	0,692238534	0,581940186	1,189535541	0,234228994	0,591501213
Eapp	611,7697205	0,032523025	0,088280797	0,368404296	0,712571794	0,9055487
A430057M04Rik	1,001534698	0,991063988	2,203818124	0,449703166	0,652924488	NA
Arl13b	211,1572325	0,291101747	0,264789159	1,09937185	0,271605904	0,633536415
Ccdc64b	5,404014613	0,268728181	0,829596417	0,3239264	0,745993756	0,919003408
Nudt12os	1,335890608	-0,218498487	1,355643641	-0,161176935	0,871954047	NA
Tut1	360,5265342	0,10434729	0,173051134	0,602985302	0,546518467	0,828173482
Tbc1d13	716,9111735	-0,173500099	0,098018196	-1,770080514	0,076713729	0,342478335
Exosc2	338,9650449	-0,175241098	0,15312935	-1,144399146	0,252458142	0,612043137
Bmf	210,21742	-0,083611315	0,283768419	-0,294646302	0,768264087	0,927598042
Anp32b	1266,725407	-0,124709092	0,09641199	-1,293501892	0,19583753	0,544880737
Maneal	0,187906191	-1,241948083	3,339949325	-0,371846385	0,710007226	NA
Fbxo21	1707,3157	0,066004848	0,218004605	0,302768135	0,762066569	0,92545349
Snx8	345,9837225	-0,031860948	0,116959978	-0,272408976	0,785307572	0,933556547
Luc7l2	2254,365274	-0,016753338	0,094201348	-0,177846051	0,85884388	0,958796604
3010003L21Rik	5,438093531	-0,151618673	0,611137453	-0,248092589	0,804062769	0,940361036
Cyb5rl	273,8198765	-0,101270674	0,106604187	-0,949969016	0,342127996	0,695250579
A530021J07Rik	0,093953095	-0,517475177	3,352475198	-0,154356154	0,877328924	NA
Hyal1	1247,921315	-0,469391815	0,15760724	-2,978237647	0,002899111	0,048005373
Nat6	1139,203707	-0,239560766	0,13264871	-1,80597886	0,070921642	0,326161482
Dock11	26,13148689	0,155220765	0,410761129	0,377885719	0,705515492	0,902021937
Unc13d	26,17220729	0,205657974	0,310121695	0,663152491	0,507232895	0,806709086
Znhit3	89,26743474	0,042667941	0,177523875	0,240350438	0,810058597	0,942417935
Nif3l1	416,2993332	-0,081247833	0,117492328	-0,691516072	0,489241284	0,796573709
Cenpc1	133,1648076	0,003635205	0,141417505	0,025705484	0,97949225	0,994664228
Acsf3	786,9101172	-0,033208423	0,101282517	-0,327879123	0,743003059	0,917092327
Cd300e	52,31984796	0,852720801	0,356617886	2,391133017	0,016796466	0,143473504
Mgp	29,14834976	0,649422631	0,506222563	1,282879664	0,199534239	0,549901719
Txnl4b	323,7564182	-0,031673529	0,131469801	-0,240918663	0,809618158	0,94241441
F2rl3	0,093953095	-0,517475177	3,352475198	-0,154356154	0,877328924	NA
Mag	5,245620029	0,728664833	0,733648747	0,993206675	0,320609246	NA
Star	18,90525528	-0,262755244	0,363907747	-0,722038063	0,470271076	0,784704695

Rlf	395,9532488	-0,313893347	0,224187065	-1,40014031	0,161471306	0,497950692
Zfp637	368,0591409	0,054553058	0,101570513	0,537095427	0,591201699	0,850384506
Tet2	214,9260538	-0,003537126	0,159372028	-0,022194146	0,982293088	0,995338444
Msrb1	6555,864205	-0,109352738	0,087554994	-1,248960606	0,211679483	0,564073259
Zfp33b	181,9643605	0,064454967	0,154640067	0,416806383	0,676820033	0,889551479
Scaf4	530,4687752	0,088722655	0,111639992	0,794721084	0,426775765	0,758920617
Ly6l	0,572315946	1,636842493	2,072243984	0,789888887	0,429592662	NA
Gm15506	12,34456498	0,498890388	0,399232626	1,249623292	0,21143719	0,564073259
LOC108167416	0,43629734	2,237934017	2,448793276	0,913892585	0,36077329	NA
Tmem168	447,6221521	0,157249079	0,107740219	1,459520697	0,144421848	0,472355765
Gm32856	67,10768024	0,043088408	0,22006068	0,195802394	0,844764836	0,953132002
Cyp11a1	0,113662148	0,780932884	3,352475198	0,232942181	0,815806304	NA
Rasef	1,093082476	1,874216826	1,555091113	1,205213514	0,228120887	NA
Rsrc2	784,6645058	0,065495649	0,100209825	0,653585112	0,513379139	0,811058031
9430070O13Rik	0,087021394	-0,517475177	3,352475198	-0,154356154	0,877328924	NA
Klhl2	657,8284202	-0,117851053	0,101699935	-1,158811487	0,246533034	0,605683673
Nipsnap3b	1161,242316	-0,002745502	0,08189347	-0,03352528	0,973255706	0,992710425
Cfap54	7,780361924	0,123614935	0,630758729	0,19597816	0,844627261	0,953132002
Map2k2	2422,658559	-0,159365168	0,09643702	-1,652531032	0,098426344	0,390695308
Plk5	46,17531163	0,004523377	0,309734816	0,014604031	0,988348083	0,996575327
Gamt	3930,708583	0,362384216	0,070041815	5,173826717	2,29E-07	4,09E-05
Gm21320	47,53417584	0,003624178	0,245471131	0,014764171	0,988220324	0,996575327
Cers6	217,9952842	-0,237744769	0,287386955	-0,827263607	0,408087666	0,746892025
Mbd6	809,1295566	-0,083814616	0,130761547	-0,640972962	0,52154025	0,814920768
Rarres2	12214,55691	0,22687588	0,099406067	2,282314219	0,022470796	0,168400998
Gucd1	5046,356695	-0,094032831	0,117530526	-0,800071554	0,423669341	0,756445602
Ipmk	1647,574645	-0,010493294	0,096009988	-0,109293776	0,912969483	0,976126761
Bicc1	120,4985552	0,895971595	0,242666245	3,692197058	0,000222325	0,008076805
1810022K09Rik	386,3330139	0,2684392	0,111258935	2,412742846	0,015832986	0,137992723
Tsc22d2	416,3065726	-0,35862934	0,159467151	-2,248922971	0,024517397	0,178140896
0610010F05Rik	326,4153158	-0,13252441	0,139458125	-0,950281026	0,341969478	0,695250579
Tatdn2	622,9558206	-0,235230848	0,117309089	-2,005222703	0,044939244	0,251272586
Ylpm1	527,3895415	-0,003839523	0,115424034	-0,033264499	0,973463664	0,992775209
Wdr20	380,4652201	0,098196543	0,108395583	0,905909076	0,36498398	0,713569421
Papola	2329,010624	-0,225354089	0,086751254	-2,597704102	0,009384933	0,100691997
Zscan26	1083,569807	0,022778145	0,120194124	0,189511301	0,849692098	0,954743122
Hfe	1457,883455	0,077546933	0,076239985	1,017142554	0,309085619	0,667986686
Malat1	4964,490635	0,017626795	0,360844037	0,048848791	0,961039799	0,988470616
Nkd2	5,133632106	-0,512428505	0,776828146	-0,659642043	0,509483567	NA
Nr2f1	10,85102061	-0,452210131	0,501503039	-0,901709653	0,367211124	0,715572853
Pip5kl1	0,093303375	-0,517475177	3,352475198	-0,154356154	0,877328924	NA
Sh3bp5	1555,938165	-0,067389375	0,102025636	-0,660514136	0,50892395	0,807845752
Ccnh	472,8141729	0,139241631	0,1166972	1,193187423	0,232795979	0,589632107
Slc8a2	0,220592835	0,059593471	3,352475198	0,017775962	0,985817581	NA
Polk	108,0793018	-0,045454955	0,217094693	-0,20937847	0,834152799	0,95084595
Msl2	605,2450802	0,021864578	0,106659453	0,204994278	0,837576596	0,951141594
Raph1	1592,326861	-0,484286214	0,105457625	-4,592235177	4,39E-06	0,000424828
Zfp982	52,85441162	-0,308613485	0,327209325	-0,943168368	0,34559479	0,698427993
Drc1	31,53180978	-0,245446557	0,407941283	-0,601671287	0,547392966	0,828528375
Fam169b	698,5447896	0,081049931	0,1048832	0,772763711	0,439662234	0,766945637
Zfp930	143,7508285	-0,039923125	0,160155059	-0,249277951	0,803145785	0,940189734
Pdgfra	107,2456171	0,487197424	0,20240575	2,407033518	0,016082694	0,139435494
