# Supplementary material for: Loss of Serpina1 in Mice Leads to Altered Gene Expression in Inflammatory and Metabolic Pathways
Source: Int J Mol Sci. 2022 Sep 9;23(18):10425. doi: 10.3390/ijms231810425 (PMC9499171; doi:10.3390/ijms231810425)
Supplement: Supplementary file 1 [file ijms-23-10425-s001.zip › ijms-1882105-supplementary material.pdf]

Supplementary material

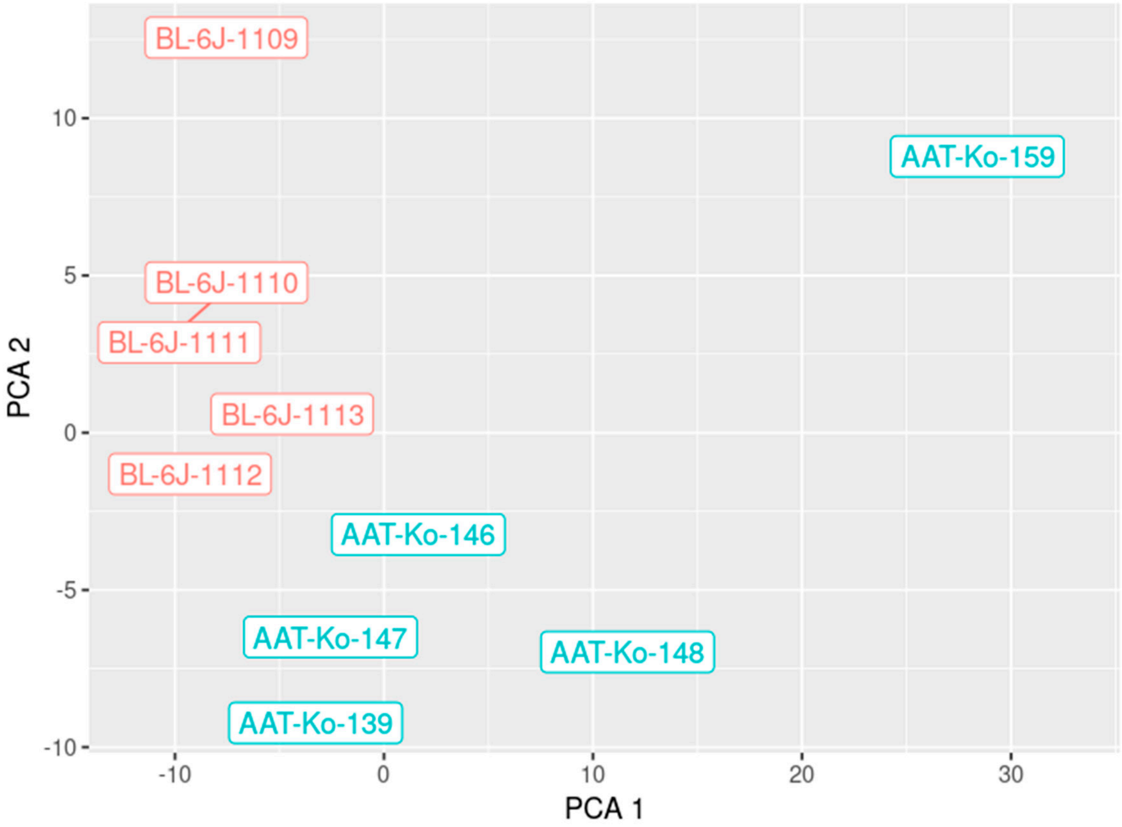

Supplementary figure S1: Principal component analysis of all the samples. PCA plot is shown here using the top 500 most variable genes across the samples. BL-6J-1109 was later dropped as an outlier for the analysis. Thus four WT and five KO were used to perform further downstream analysis.

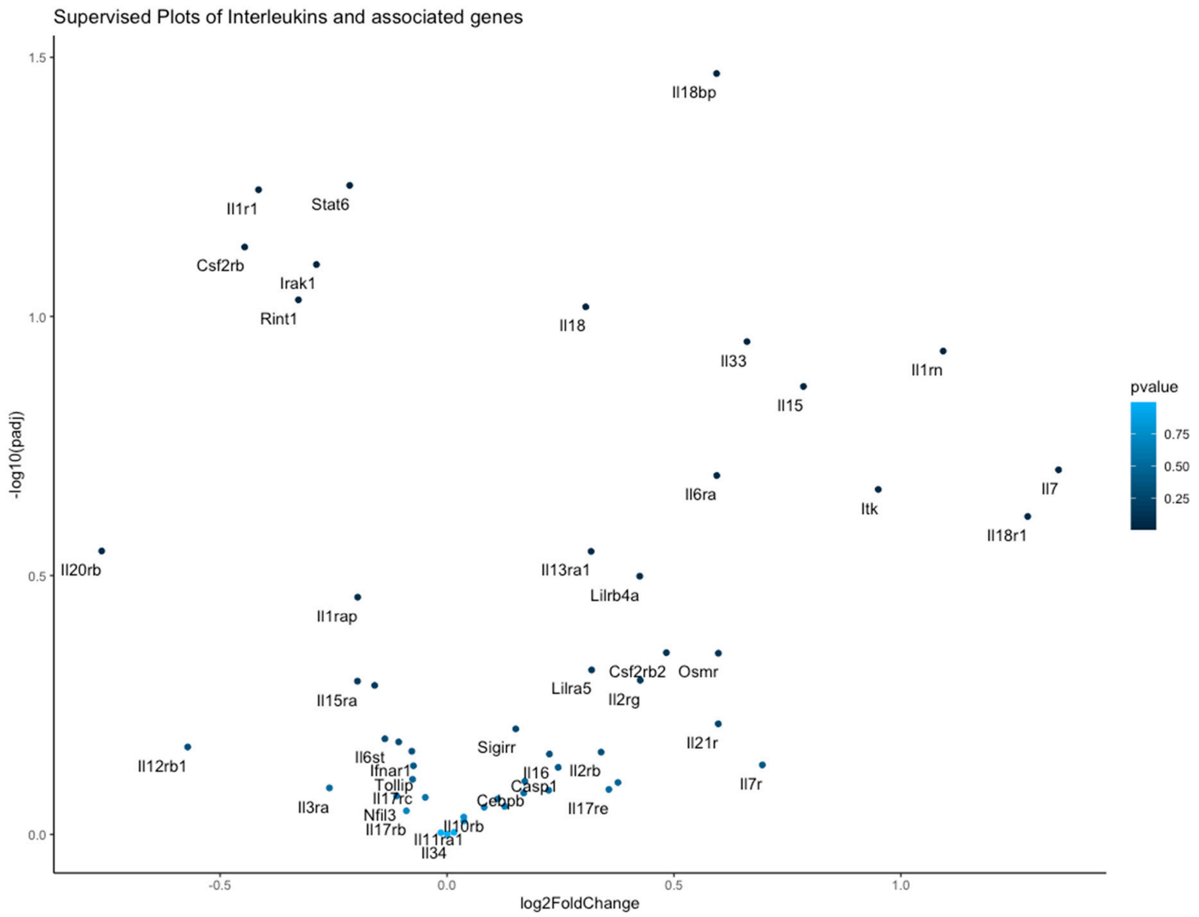

Supplementary figure S2: Changes in expression of Interleukins. The foldchange values were plotted as a volcano plot for Interleukins and associated genes to interpret any significant changes in transcription of interleukins.

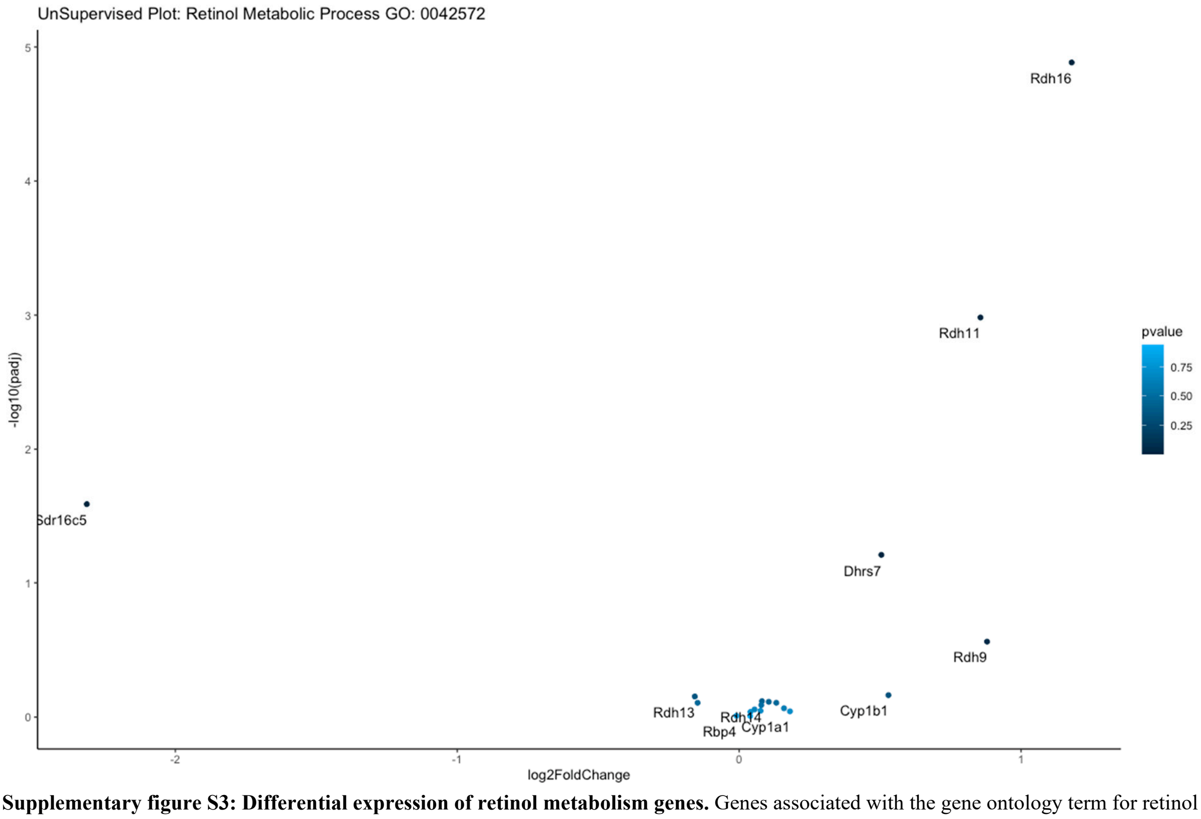

Supplementary figure S3: Differential expression of retinol metabolism genes. Genes associated with the gene ontology term for retinol metabolic processes (n = 29) were plotted as a volcano plot to see the changes in expression of these genes in the dataset

Supplementary table S1: *Serpina1a-e* KO vs wildtype mice: the most highly significant DEGs sorted by adjusted *p*-value

**Gene Name**	**baseMean**	**log$_2$FoldChange**	**lfcSE**	**stat**	***p*-value**	**adjusted *p*-value**
Serpina1d	36726,78572	-2,771899239	0,090336145	-30,68427632	9,23E-207	1,23E-202
Serpina1b	43560,11467	-5,433134138	0,216039364	-25,14881566	1,46E-139	9,73E-136
Tagap1	114,6234039	-1,422514276	0,153818038	-9,2480329	2,29E-20	1,02E-16
6430548M08Rik	215,5051748	-1,359184784	0,149457882	-9,094099073	9,54E-20	3,19E-16
Syvn1	4279,105029	-1,350964575	0,161540968	-8,362984289	6,12E-17	1,64E-13
Cyp7a1	3642,422605	1,422720764	0,175519915	8,105751192	5,24E-16	1,17E-12
Serpina3n	9750,601541	0,761867915	0,097035978	7,851396268	4,11E-15	7,86E-12
Serpina1c	36363,74467	-7,515214679	0,972315849	-7,72919076	1,08E-14	1,81E-11
1810053B23Rik	37,01654086	5,038046699	0,66001341	7,633248994	2,29E-14	3,40E-11
Selenbp1	8504,419664	0,652540689	0,086827347	7,51538213	5,67E-14	7,59E-11
Serpina1e	170596,6025	-6,001166507	0,810128453	-7,4076728	1,29E-13	1,56E-10
Cacnb1	48,14485669	2,807211446	0,384862254	7,294067982	3,01E-13	3,09E-10
Arfgap3	275,5289736	-1,125522897	0,154240028	-7,297216626	2,94E-13	3,09E-10
Derl3	124,7132768	-2,509200309	0,357222318	-7,024198056	2,15E-12	2,06E-09
Serpina1a	24564,22893	-7,677876464	1,101299547	-6,971651343	3,13E-12	2,79E-09
Gstt3	2368,10658	1,933115913	0,278299926	6,946160351	3,75E-12	3,14E-09
Hsph1	1907,501388	-1,350996724	0,195030526	-6,927103941	4,30E-12	3,38E-09
Ces2b	29,92597012	-3,949435856	0,580217033	-6,806825083	9,98E-12	7,41E-09
Gstm2	2714,065957	1,115125145	0,166037882	6,716088715	1,87E-11	1,31E-08
Atad2	80,71457816	-1,209907702	0,181026405	-6,683597918	2,33E-11	1,56E-08
Ampd2	913,2648352	-0,576719695	0,088175199	-6,540611194	6,13E-11	3,90E-08
Gmppb	462,5951536	-1,092263521	0,167551893	-6,518956601	7,08E-11	4,30E-08
Mat1a	64431,99511	0,985599835	0,152665517	6,455942741	1,08E-10	5,99E-08
Nqo1	179,8052572	1,325202615	0,205158451	6,459410301	1,05E-10	5,99E-08
Bhmt	68087,55314	1,202636139	0,186760811	6,439445888	1,20E-10	6,41E-08
Tmed3	579,058565	-0,728729724	0,116578272	-6,250990983	4,08E-10	2,10E-07
Zbtb40	197,425417	-0,795145769	0,12846733	-6,189478422	6,04E-10	2,99E-07
Bckdhb	1737,719653	0,497745841	0,081237737	6,127027406	8,95E-10	4,28E-07
Bhlha15	27,68344455	-2,452042804	0,406049784	-6,038773815	1,55E-09	7,16E-07
Lcn2	784,4901943	3,758258103	0,625663937	6,006831912	1,89E-09	8,43E-07
Gfpt1	653,9101306	-0,895698699	0,149599921	-5,987293945	2,13E-09	8,95E-07
Med12l	97,29102232	-1,425906663	0,238182777	-5,986606917	2,14E-09	8,95E-07
Pxmp2	3650,84229	0,452741865	0,076527521	5,916066001	3,30E-09	1,34E-06
Atp2a2	5377,203759	-0,689766857	0,11748842	-5,870934839	4,33E-09	1,70E-06
Orm2	1820,464979	3,15121766	0,539103341	5,845294251	5,06E-09	1,93E-06
Sh2d4a	266,5403606	1,368962429	0,235852061	5,804326772	6,46E-09	2,40E-06
Msmo1	6645,370361	0,92539382	0,160723837	5,757663815	8,53E-09	3,07E-06
Sult1d1	2143,196842	1,109730237	0,192877263	5,753556537	8,74E-09	3,07E-06
Lncbate1	28,20509705	2,627539247	0,458891798	5,725836148	1,03E-08	3,53E-06

Mlec	3490,349468	-0,734638579	0,129501076	-5,672837651	1,40E-08	4,69E-06
P3h1	446,5957182	-0,604058419	0,106761897	-5,657996315	1,53E-08	4,99E-06
Wfs1	428,6400887	-0,692038134	0,124803826	-5,545007362	2,94E-08	9,36E-06
Acss2	14034,2408	1,328671576	0,240288018	5,529495765	3,21E-08	9,79E-06
Hmgcs1	15813,42173	1,230111587	0,222484063	5,528987416	3,22E-08	9,79E-06
Per2	393,5405893	-1,424720515	0,258656359	-5,508159629	3,63E-08	1,08E-05
Nsdhl	3474,716847	0,824448245	0,149783313	5,504272999	3,71E-08	1,08E-05
Cd4	68,03792704	-1,450247246	0,264360962	-5,485860063	4,11E-08	1,10E-05
Creld2	2161,780487	-1,49858684	0,272945795	-5,490419219	4,01E-08	1,10E-05
Palld	1879,684705	-0,860466635	0,15657791	-5,495453567	3,90E-08	1,10E-05
Nktr	1064,317609	-0,639927532	0,116653161	-5,485728181	4,12E-08	1,10E-05
Camk1d	2925,227806	0,914266075	0,167209302	5,467794342	4,56E-08	1,19E-05
Sc5d	19032,80951	0,623365682	0,114283276	5,454566075	4,91E-08	1,26E-05
Rdh16	2045,939804	1,179679786	0,216646474	5,445183422	5,18E-08	1,31E-05
Hyou1	7002,411624	-1,146946816	0,21167488	-5,41843613	6,01E-08	1,49E-05
Cyp2c38	1413,913705	1,485989759	0,277907337	5,347069184	8,94E-08	2,10E-05
Eif2ak3	473,6167123	-0,695614385	0,130103435	-5,346625802	8,96E-08	2,10E-05
Tbx3	589,5843844	1,015173032	0,189791609	5,348882599	8,85E-08	2,10E-05
Cyp8b1	13306,18112	0,613741888	0,115004361	5,33668361	9,47E-08	2,18E-05
Prg4	1172,051329	0,595700965	0,111948043	5,321227152	1,03E-07	2,34E-05
Ces4a	98,8750855	-2,287800671	0,431162687	-5,306119335	1,12E-07	2,50E-05
Serpinb1a	119,222666	2,038521121	0,385369902	5,28977772	1,22E-07	2,64E-05
Hrg	25395,50388	0,487322531	0,092091617	5,291714361	1,21E-07	2,64E-05
Apcs	2699,532546	0,908964445	0,17192896	5,286860613	1,24E-07	2,64E-05
Ang	4333,497118	0,620370319	0,117616705	5,274508558	1,33E-07	2,78E-05
Cyp2c54	6994,66163	0,995120885	0,189079617	5,262972829	1,42E-07	2,87E-05
Nucb2	175,7883255	-0,949295403	0,180329246	-5,264234302	1,41E-07	2,87E-05
Odc1	2023,202556	-0,836841361	0,159366199	-5,251059294	1,51E-07	3,02E-05
Ahcy	19394,0195	0,345500761	0,065985127	5,236039981	1,64E-07	3,23E-05
Slc22a30	3355,781043	-0,682211847	0,130562123	-5,225189587	1,74E-07	3,33E-05
Dcxr	2432,803251	0,556478606	0,106510352	5,224643359	1,74E-07	3,33E-05
Mt2	1031,455038	5,496835666	1,054408129	5,213195456	1,86E-07	3,50E-05
Cad	492,8336619	-1,020917929	0,196561036	-5,193897779	2,06E-07	3,82E-05
Tnfrsf19	126,3537893	1,149312023	0,221576045	5,18698681	2,14E-07	3,86E-05
Sult2a7	46,53727766	2,373953574	0,457647396	5,187298328	2,13E-07	3,86E-05
Gamt	3930,708583	0,362384216	0,070041815	5,173826717	2,29E-07	4,09E-05
Atp6v0a2	754,170336	-0,451913666	0,08740269	-5,170477782	2,33E-07	4,11E-05
Hpd	62109,45203	0,580728898	0,112402417	5,166516091	2,38E-07	4,14E-05
Ubqln4	1207,696805	-0,394980264	0,076865411	-5,138595622	2,77E-07	4,74E-05
Dusp1	578,0251049	-0,965413607	0,18838331	-5,124730039	2,98E-07	4,98E-05
Orm3	191,2022565	4,057770062	0,791769674	5,124937458	2,98E-07	4,98E-05
Txndc5	5832,163659	-0,51457291	0,100674712	-5,111242929	3,20E-07	5,28E-05

Wfdc21	9871,164858	0,685045528	0,134155914	5,106338666	3,28E-07	5,36E-05
Manf	3906,809074	-0,871978359	0,171403685	-5,087278952	3,63E-07	5,85E-05
Cyp4v3	11587,45122	-0,745171439	0,147119635	-5,065071279	4,08E-07	6,50E-05
Fst	186,6220123	-1,708307877	0,337885445	-5,055878858	4,28E-07	6,66E-05
Aldh1l1	42667,93295	0,597676848	0,118200515	5,056465697	4,27E-07	6,66E-05
Cyp2g1	43,19088159	2,712535388	0,536909798	5,052124953	4,37E-07	6,71E-05
Spc25	71,85538832	-1,33397146	0,264508186	-5,043214271	4,58E-07	6,95E-05
Srprb	704,4479448	-0,701698855	0,139449908	-5,031906185	4,86E-07	7,21E-05
Spon2	447,0556601	-0,760334329	0,151055589	-5,033473652	4,82E-07	7,21E-05
Slc25a13	3827,02022	-0,946747008	0,189428693	-4,997907099	5,80E-07	8,51E-05
Rnase4	20080,89714	0,707119728	0,141663045	4,991560976	5,99E-07	8,66E-05
Oat	16877,54203	1,318855301	0,264271055	4,990540108	6,02E-07	8,66E-05
Spata2l	564,7022256	0,878040141	0,176678187	4,969714471	6,71E-07	9,54E-05
Abhd8	159,6795934	-0,811669813	0,163523435	-4,963629912	6,92E-07	9,74E-05
C2cd2l	897,9009416	-0,53717752	0,108450351	-4,95321145	7,30E-07	0,000101133
Xpnpep3	485,5021754	-0,516111057	0,104218474	-4,952203178	7,34E-07	0,000101133
Sdf2l1	1333,850109	-1,266068756	0,255926224	-4,947006743	7,54E-07	0,000102809
Clmp	31,7938379	1,399943564	0,284282557	4,924479296	8,46E-07	0,000113178
Tspan33	377,6729953	-0,880829127	0,178873496	-4,924313252	8,47E-07	0,000113178
Stx5a	1373,355257	-0,473701092	0,096807584	-4,893222964	9,92E-07	0,000131305
4930402H24Rik	1019,573102	-0,618230203	0,126397377	-4,891163249	1,00E-06	0,000131386
Snx10	417,8203936	0,612242613	0,125302493	4,886116777	1,03E-06	0,000132204
Slc27a5	23963,53558	0,350161817	0,071656876	4,886646387	1,03E-06	0,000132204
Ces1b	2976,085076	0,556023133	0,11393005	4,880390516	1,06E-06	0,000134805
Ropn1l	295,6910692	0,698869917	0,144356183	4,841288436	1,29E-06	0,000162698
Cry1	688,2855664	-1,103091287	0,228415063	-4,829328129	1,37E-06	0,000171166
Tubb5	2100,865008	-0,553354047	0,114670073	-4,825618694	1,40E-06	0,000171184
Arhgef26	462,8044722	0,6764713	0,140165964	4,826216575	1,39E-06	0,000171184
Mfhas1	701,5419922	-0,638054689	0,132621489	-4,811095785	1,50E-06	0,000182432
Fdps	11450,27418	0,799464025	0,166372936	4,805252851	1,55E-06	0,000185241
Trabd	2195,028828	-0,424500632	0,088355958	-4,804437003	1,55E-06	0,000185241
Rap2a	701,2436411	-0,72021956	0,150030779	-4,800478712	1,58E-06	0,000187269
Aadat	3068,875173	-0,462729368	0,096866167	-4,776996782	1,78E-06	0,000208665
Igfbp5	321,8063866	1,282023616	0,269195237	4,76243054	1,91E-06	0,000220444
Slco2a1	3173,059149	-0,662215553	0,139007424	-4,763886238	1,90E-06	0,000220444
Nfxl1	262,6999949	-0,636022744	0,134024807	-4,745559865	2,08E-06	0,000237592
Fndc3b	716,9971103	-0,790724327	0,166698279	-4,743446262	2,10E-06	0,000238051
Akr1d1	5715,882961	0,684340989	0,144714157	4,72891528	2,26E-06	0,000253587
Adamts6	29,84445068	1,587241859	0,335818275	4,726490416	2,28E-06	0,000254494
Hspa8	25268,78087	-0,786025033	0,166508696	-4,720624512	2,35E-06	0,00025978
Fam129b	232,386058	1,104359449	0,2351419	4,696565981	2,65E-06	0,000289923
Cyp2a5	9177,056308	0,904309265	0,193312727	4,67796032	2,90E-06	0,000314924

0610007P14Rik	2210,513483	0,562053816	0,12039012	4,668604177	3,03E-06	0,000322112
Sdr42e1	3777,363533	0,567257721	0,121468724	4,669989965	3,01E-06	0,000322112
Timp1	11,65609813	3,258505224	0,697994872	4,668379893	3,04E-06	0,000322112
Tubgcp4	714,2988997	-0,436540931	0,093582744	-4,664758839	3,09E-06	0,000325255
Tpst1	768,5233827	-0,670406981	0,144434444	-4,641600451	3,46E-06	0,000361089
Txlna	766,830198	-0,614500043	0,132438776	-4,639880094	3,49E-06	0,000361286
Kng1	68781,05526	0,432503932	0,093278868	4,636676483	3,54E-06	0,000361326
Marveld1	780,0208334	-0,504025204	0,108689749	-4,637283731	3,53E-06	0,000361326
Trp53inp2	4502,853325	-0,629961591	0,136085321	-4,629166376	3,67E-06	0,000371841
Fmo4	235,4100287	0,99567523	0,215663542	4,616799013	3,90E-06	0,000388963
Angptl3	23038,1111	0,455763049	0,098720241	4,616713277	3,90E-06	0,000388963
Fdft1	5901,133389	0,687151271	0,148912326	4,614468719	3,94E-06	0,000390278
Mical2	787,6164421	-0,722724315	0,157156106	-4,598767008	4,25E-06	0,00041778
Atp6v0d2	235,5747221	3,018565795	0,657001511	4,594457919	4,34E-06	0,000423393
Raph1	1592,326861	-0,484286214	0,105457625	-4,592235177	4,39E-06	0,000424828
Sec23b	1661,972329	-0,617433946	0,13454578	-4,589024994	4,45E-06	0,000428309
Slc17a9	254,8400768	-1,054081046	0,229903006	-4,584894581	4,54E-06	0,000433743
Fus	1450,220434	0,472597819	0,103184781	4,580111656	4,65E-06	0,000440634
Osbp	1987,104291	-0,513280307	0,112129626	-4,577561933	4,70E-06	0,000442897
Fkbp11	369,8169723	-0,915404337	0,200177936	-4,572953215	4,81E-06	0,000449589
Atf6b	799,8592472	-0,447924943	0,097990891	-4,571087574	4,85E-06	0,000450461
Adk	13643,53003	0,390877336	0,085656975	4,5632867	5,04E-06	0,00046431
Hspa13	932,4671779	-0,75676492	0,166136779	-4,555071576	5,24E-06	0,000479524
Serpina3m	7044,213826	0,7652928	0,168183387	4,550347168	5,36E-06	0,000487082
Nans	437,1651986	-0,614783264	0,13518689	-4,547654458	5,42E-06	0,00049002
Ccdc149	97,34646708	-1,043915361	0,230698021	-4,525029549	6,04E-06	0,000541822
Pla2g6	1126,039979	-0,616403181	0,136545667	-4,514263942	6,35E-06	0,000566285
Sult1a1	10374,12328	0,845344578	0,187613603	4,505774438	6,61E-06	0,000585503
Pdzk1ip1	199,6752053	1,081954187	0,240368615	4,501229032	6,76E-06	0,000590348
Mt1	1918,593263	4,275998625	0,949921581	4,50142276	6,75E-06	0,000590348
Acpp	254,1426806	2,096576785	0,467414986	4,485471899	7,28E-06	0,000631579
Slc25a37	1008,593532	-0,516677993	0,115334558	-4,47981944	7,47E-06	0,000644353
Papss1	504,0679255	-0,533414686	0,119276634	-4,472080312	7,75E-06	0,000663842
Gm13375	66,78523357	0,925369618	0,207000651	4,470370564	7,81E-06	0,000664909
Uap1	881,6262474	-0,45440057	0,102003235	-4,454766242	8,40E-06	0,000710627
Alpl	513,4433642	-0,704577788	0,158803871	-4,436779675	9,13E-06	0,000765512
Chil1	12,77570289	3,442654598	0,77605971	4,436069227	9,16E-06	0,000765512
Tspan8	30,04978669	1,425258756	0,322294582	4,422223758	9,77E-06	0,000808037
Apoh	51127,44989	0,340206572	0,076939732	4,421728086	9,79E-06	0,000808037
Tiam2	279,4927884	-1,057862636	0,239774612	-4,41190427	1,02E-05	0,000840405
Golga4	1629,836484	-0,491285128	0,111688854	-4,398694324	1,09E-05	0,000883538
Krt7	37,17027689	1,265373072	0,287688664	4,398411302	1,09E-05	0,000883538

Mitf	121,2344742	-0,75526764	0,173177515	-4,36123384	1,29E-05	0,001040506
Akap13	1194,894636	-0,503503012	0,115478475	-4,36014599	1,30E-05	0,001040506
Rdh11	2515,848031	0,856155023	0,196426538	4,358652512	1,31E-05	0,001041395
Hn1l	932,8661102	-0,526148428	0,120984688	-4,348884436	1,37E-05	0,001082429
Meiob	143,3568548	0,549484086	0,126428894	4,346190716	1,39E-05	0,001089354
Ermp1	725,6823642	-0,432336638	0,09959239	-4,341060971	1,42E-05	0,001108582
Cflar	1663,591181	-0,504049195	0,116165783	-4,339050486	1,43E-05	0,001112267
Alg12	295,4802764	-0,614717023	0,14182447	-4,334350925	1,46E-05	0,001129725
Hacl1	13407,94474	0,604500007	0,139520616	4,332693076	1,47E-05	0,001131727
Uba5	895,0301699	-0,477146199	0,110162134	-4,3313086	1,48E-05	0,00113236
Fabp2	1322,536224	-0,606497335	0,140071559	-4,329910658	1,49E-05	0,001133098
Cisd3	1031,866009	0,398035325	0,0920666	4,323341228	1,54E-05	0,00116079
Pnpla6	575,5524685	-0,489669594	0,113342173	-4,320277097	1,56E-05	0,001170414
Ssh2	474,7789231	-0,488515429	0,11313172	-4,318111944	1,57E-05	0,001175349
Orm1	28300,89604	0,612168399	0,141877785	4,31475864	1,60E-05	0,001186702
Zfpm1	1238,939073	-0,644609213	0,14982674	-4,302364281	1,69E-05	0,001248156
Hsd17b10	3641,027845	0,383524948	0,089274479	4,296020018	1,74E-05	0,001277341
Sec14l4	4411,046491	-0,469696453	0,109370089	-4,294560403	1,75E-05	0,001278747
Golga3	619,9581966	-0,481284151	0,112149035	-4,291469393	1,77E-05	0,001289634
Ostc	1735,790951	-0,40364923	0,094202846	-4,284894215	1,83E-05	0,001321191
Defb1	49,69747846	1,382160952	0,322892912	4,280555259	1,86E-05	0,001332808
Dap	6391,433453	0,292045939	0,068210859	4,281516795	1,86E-05	0,001332808
Papss2	6685,829878	0,637530136	0,148988486	4,279056404	1,88E-05	0,001333939
Foxa1	545,1313027	-1,026172997	0,239914835	-4,277238619	1,89E-05	0,001333939
Rpn1	4719,096453	-0,555380377	0,129858156	-4,276823238	1,90E-05	0,001333939
Pla2g7	92,42561448	1,059296568	0,247813788	4,274566718	1,92E-05	0,001334746
Dhcr7	3958,144847	0,620956796	0,145274942	4,274355833	1,92E-05	0,001334746
Usp2	111,5395461	-1,114497026	0,261077104	-4,268842457	1,96E-05	0,001361075
Hao1	6373,916459	-0,666112033	0,156322512	-4,261139517	2,03E-05	0,001401589
Hspa5	31486,9589	-0,809349355	0,190027022	-4,259127713	2,05E-05	0,00140701
Fmr1nb	6,644292612	-4,528799701	1,06688242	-4,244891112	2,19E-05	0,001491732
H2-Q4	1039,88224	0,637449289	0,15026676	4,242117742	2,21E-05	0,001502624
Cldn2	2914,630429	0,624171875	0,147233682	4,239328024	2,24E-05	0,001513735
1810010H24Rik	65,46229462	0,9427587	0,222627461	4,234691878	2,29E-05	0,001532624

A table of all the genes that were part of the dataset is provided in the file "Full Supplementary Table S1".
